# The structure of Photosystem I acclimated to far-red light illuminates an ecologically important acclimation process in photosynthesis

**DOI:** 10.1126/sciadv.aay6415

**Published:** 2020-02-05

**Authors:** Christopher Gisriel, Gaozhong Shen, Vasily Kurashov, Ming-Yang Ho, Shangji Zhang, Dewight Williams, John H. Golbeck, Petra Fromme, Donald A. Bryant

**Affiliations:** 1 Biodesign Center for Applied Structural Discovery, Arizona State University, Tempe, AZ 85287-5001, USA.; 2 School of Molecular Sciences, Arizona State University, Tempe, AZ 85287-1604, USA.; 3 Department of Biochemistry and Molecular Biology, The Pennsylvania State University, University Park, PA 16802 USA.; 4 Intercollege Graduate Program in Plant Biology, The Pennsylvania State University, University Park, PA 16802 USA.; 5 Department of Chemistry, The Pennsylvania State University, University Park, PA 16802 USA.; 6 Department of Chemistry and Biochemistry, Montana State University, Bozeman, MT 59717 USA.

## Abstract

Phototrophic organisms are superbly adapted to different light environments but often must acclimate to challenging competition for visible light wavelengths in their niches. Some cyanobacteria overcome this challenge by expressing paralogous photosynthetic proteins and by synthesizing and incorporating ~8% chlorophyll f into their Photosystem I (PSI) complexes, enabling them to grow under far-red light (FRL). We solved the structure of FRL-acclimated PSI from the cyanobacterium *Fischerella thermalis* PCC 7521 by single-particle, cryo–electron microscopy to understand its structural and functional differences. Four binding sites occupied by chlorophyll f are proposed. Subtle structural changes enable FRL-adapted PSI to extend light utilization for oxygenic photosynthesis to nearly 800 nm. This structure provides a platform for understanding FRL-driven photosynthesis and illustrates the robustness of adaptive and acclimation mechanisms in nature.

## Teaser

The Photosystem I structure from far-red light cells illuminates an ecologically important acclimation process in photosynthesis.

## INTRODUCTION

**Fig. 1:**
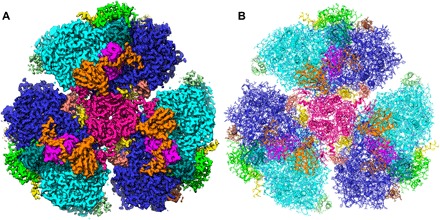
Identification of FRL-PSI subunits within the cryo-EM–derived density map. (**A**) View down on the stromal (cytoplasm-facing) side of the density map after three-dimensional (3D) reconstruction and postprocessing at 4*σ, where the map was carved around the individual subunit model to which it corresponds with a 2-Å radius. (**B**) Model that was built with colors corresponding to their densities in (A).

The ability of some cyanobacteria to use far-red light (FRL) for oxygenic photosynthesis has recently been realized to be an important and ecologically widespread function that is likely to be an underappreciated contributor to global primary production (*1*–*3*). FRL photoacclimation, or FaRLiP, results in the ability of these cyanobacteria to make use of lower-energy FRL (λ = 700 to 800 nm) (*3*–*6*). This process allows cells to perform photosynthesis under conditions where visible light is severely attenuated by competing photosynthetic organisms or physical conditions that absorb or scatter most of the visible light (*3*). Compared to the Photosystem I (PSI) complexes produced in cells grown in white light (WL), a major change during FaRLiP is the incorporation of chlorophyll f (Chl f) molecules into PSI, which replace ~8% of the Chl a molecules (*5*–*10*). To accommodate the insertion of Chl f molecules and their subsequent roles in FRL absorption, energy transfer, and successful photochemistry, cells express a unique PSI form in which 6 of the 12 PSI subunits (PsaA1, PsaB1, PsaL1, PsaI1, PsaF1, and PsaJ1) are replaced by paralogs (PsaA2, PsaB2, PsaL2, PsaI2, PsaF2, and PsaJ2) encoded within the FaRLiP gene cluster (*3*–*5*). The biochemical and biophysical aspects of energy transfer and photochemistry have only recently begun to be characterized (*6*–*10*), and structure-function relationships have, so far, only been speculative because no structural information was available. To understand this essential aspect of FaRLiP, we solved the structure of FRL-acclimated PSI (FRL-PSI) from *Fischerella thermalis* PCC 7521 (hereafter referred to as *F. thermalis*) by single-particle cryo–electron microscopy (cryo-EM; Fig. 1).

## RESULTS

### Structure determination and structural overview

Trimeric FRL-PSI complexes were isolated by two rounds of sucrose gradient centrifugation from *F. thermalis* cells that had been grown in FRL (fig. S1, A and C, and see Materials and Methods for details on growth and FRL-PSI complex isolation). FRL-PSI had an absorption maximum at 680 nm and a 77-K fluorescence emission maximum at 746 nm (fig. S1D). Unlike FRL-PSII, which has obvious absorption maxima at 675 and 723 nm and a shoulder at ~730 nm (fig. S1B), absorbance of the FRL-PSI complexes gradually decreases in a featureless manner from 700 to 800 nm. The cryo-EM structure of FRL-PSI was obtained by single-particle reconstruction. Particle picking and filtering resulted in a dataset containing 178,666 particles that were processed using C_3_ symmetry; this resulted in a density map exhibiting a global resolution of 3.19 Å, with local resolution of important elements of the density as high as ~3.0 Å (fig. S2). Cryo-EM data collection, refinement, and validation statistics are shown in table S1. Additional important information, data collection, and data processing are also included in Materials and Methods.

The *F. thermalis* FRL-PSI trimer is a large membrane protein complex with a molecular mass of ~1 MDa, ~26% of which is attributed to its 372 cofactors that are coordinated by 36 individual subunits. The overall structure of FRL-PSI is similar to other known cyanobacterial PSI trimer structures, which is remarkable considering the low sequence identity of some subunits (see sequence identity matrices in table S2 and structural superposition comparisons in table S3) (*11*–*17*). The 10 central transmembrane helices (TMHs), which coordinate the electron transfer chain (ETC) cofactors and which comprise ~20% of the amino acids of the core subunits PsaA and PsaB, exhibit high sequence identity between FRL- and WL-adapted PSI.

**Fig. 2:**
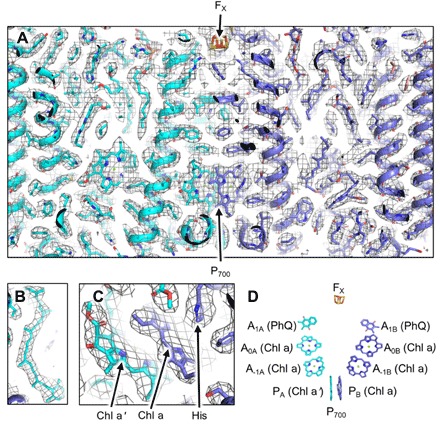
Structure of *F. thermalis* FRL-PSI within the density map. (**A**) Cross section of a monomer from a membrane plane view including the P_700_ special pair and the intersubunit [4Fe-4S] cluster, F_X_. (**B**) Carotenoid molecule. (**C**) Closer view of P_700_ where the axial coordination from the side chain of a His can be observed. The map contour is 5*σ for (A) to (C). (**D**) Nomenclature and identities of core-associated FRL-PSI electron transport chain cofactors. Each cofactor is listed adjacent to its spectroscopic site name and biochemical identity. “PhQ” stands for phylloquinone, and “F_X_” is the intersubunit [4Fe-4S] cluster coordinated by PsaA and PsaB. In all panels, oxygen atoms are colored red, nitrogen atoms are colored blue, iron atoms are colored orange, and sulfur atoms are colored yellow.

In each PSI monomer, 89 Chls, 21 carotenoids, 3 [4Fe-4S] clusters, 2 phylloquinones, 2 phosphatidylglycerols, 4 monogalactosyl diglycerides, and 2 *n*-dodecyl-β-d-maltoside (DM) detergent molecules were identified in the density map. Selected views of the model within the density map and the nomenclature used here for ETC cofactors are shown in Fig. 2.

### Biochemical pigment analyses

Figure S1 (E and F) shows high-performance liquid chromatography (HPLC) elution profiles for extracted pigments of the FRL-PSI complexes monitored at 665 nm (Chl a) and 707 nm (Chl f). Figure S4A shows elution profiles for extracted pigments from both WL-PSI and FRL-PSI complexes monitored at 270 nm. The deconvoluted peaks for phylloquinone, all-*trans*-echinenone, and *cis* isomers of echinenone are indicated. Figure S4B shows HPLC elution profiles for extracted pigments from WL-PSI and FRL-PSI complexes monitored at 453 nm. Major Chls and carotenoid peaks are identified on the figure. The slightly later eluting and smaller peaks associated with echinenone and β-carotene are due to *cis* isomers of these two carotenoids.

Because PSI complexes contain two phylloquinone molecules, we used the phylloquinone content as a means to calculate the number of pigments bound to the purified WL-PSI and FRL-PSI trimers. Direct quantitation of the Chls in FRL-PSI by reversed-phase HPLC analyses indicates that FRL-PSI from *F. thermalis* contains 7.1 ± 0.3 Chl f_P_ and 89.1 ± 14.1 Chl a_P_ per monomer (Chl a_P_/Chl f_P_ ratio, 12. 7 ± 0.7; note: the subscript “P” indicates that the esterifying alcohol is phytol) (figs. S1E and S4A). However, if there are actually only 90 total Chls in FRL-PSI, then one expects 83.5 Chl a_P_ and 6.5 ± 0.3 Chl f_P_ per monomer. In consideration of these values, we conclude that there are 7 Chl f molecules and 83 other tetrapyrrole pigments (Chl a_P_ + Chl a_P_′) per FRL-PSI monomer. Thus, 82 Chls are modeled as Chl a_P_, 4 are modeled as Chl f_P_, 1 is modeled as Chl a_P_′, and 1 Chl a_P_ bound to PsaK was left unmodeled because of the poor resolution in the PsaK region of the density map.

Similarly, reversed-phase HPLC analyses additionally indicate that FRL-PSI contains 15.8 ± 0.6 carotenoids and WL-PSI contains 19.1 ± 1.1 carotenoid molecules per monomer and that both complexes contain a mixture of β-carotene and echinenone (4-keto-β-carotene; fig. S4B). Possibly, because of incomplete extraction or the hydrophobicity of these compounds, the measured carotenoid content for FRL-PSI complexes is lower than the 21 total carotenoids that could be identified in the density map. The ratio of β-carotene to echinenone was 1.44 in WL-PSI but was higher, 1.82, in FRL-PSI. These data suggest that FRL-PSI may contain fewer echinenone molecules than WL-PSI in *F. thermalis*. Because echinenone differs from β-carotene only by a 4-keto group (i.e., a single oxygen atom), it is not possible to distinguish β-carotene from echinenone at the resolution achieved. Therefore, all carotenoids are modeled as β-carotene (BCR); one (BCR 7) is modeled as 15-*cis*-β-carotene.

### FRL-PSI sequence and pigment site variation

Although there is currently no structure of *F. thermalis* PSI from cells grown in WL for direct comparison, the known structure of PSI from *Thermosynechococcus elongatus* [Protein Data Bank (PDB) ID, 1JB0] is likely to be a good representation of WL-PSI from *F. thermalis* due to very high sequence similarity (table S2) and especially because all residues coordinating cofactors are conserved (see sequence alignments in fig. S3).

**Fig. 3:**
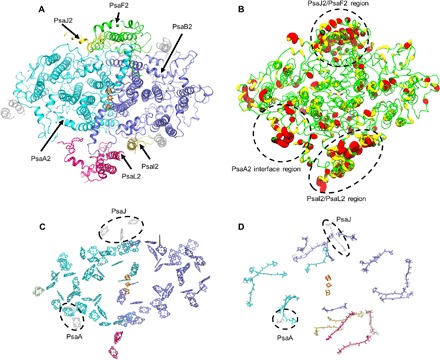
FRL-PSI variation compared to WL-PSI as viewed down on the stromal surface. (**A**) Subunits of a FRL-PSI monomer where the unchanged subunits are colored in transparent gray and the FRL-specific subunits are colored. The stromal subunits PsaC, PsaD, and PsaE are not shown for clarity. (**B**) FRL-specific subunits as ribbons with color and radius shown according to sequence identity compared to their WL isoform (red, yellow, and green: low, intermediate, and high sequence identity; small to large radius: high to low sequence identity). Therefore, large red regions are of lowest sequence identity, and narrow green regions are highly similar in sequence identity. (**C**) Tetrapyrrole rings in FRL-PSI superimposed with those of *T. elongatus* PSI. (**D**) Superposition instead showing only carotenoids. In both (C) and (D), FRL-PSI pigments are colored by the subunit to which they are assigned, and *T. elongatus* pigments are shown in transparent gray. Black dashed circles show two main locations of pigment variation and list the subunits to which the variable pigment was assigned in the *T. elongatus* PSI structure. The Chl originally assigned in the *T. elongatus* structure to PsaM and the possible PsaK-coordinated Chl that was not resolved in the FRL-PSI structure is not shown as discussed in the main text.

FRL-specific subunits of FRL-PSI are shown in Fig. 3A, and a visual depiction of sequence identity compared with WL-PSI subunits is shown in Fig. 3B. Notably, the sequence differs most substantially in three areas: near the PsaA2 monomer-monomer interface, near the periphery of PsaB2 and the peripheral subunits PsaF2 and PsaJ2, and near the trimer connection region composed of PsaI2 and PsaL2 (Fig. 3B). Five Chls that are present in the *T. elongatus* PSI structure and in other cyanobacterial PSI structures are obviously missing in FRL-PSI. Three are coordinated by PsaJ, and two are coordinated by PsaA. Twenty-one of the 22 carotenoids in the *T. elongatus* PSI structure were identified in FRL-PSI, and the missing one is also located near the interface of PsaB, PsaJ, and PsaF (Fig. 3D). Substantial structural differences for pigments between FRL-PSI and *T. elongatus* PSI specifically correspond to three regions with low sequence identity: (i) near the monomer-monomer interface region of PsaA2, (ii) near PsaF2 and PsaJ2 toward the periphery of the trimer, and (iii) in the trimerization domain including PsaI2 and PsaL2 (Fig. 3, A and B).

## DISCUSSION

### Structural observations and differences in pigment binding sites

The density map allowed 11 of the 12 subunits to be modeled confidently. The exception was the PsaK subunit and its associated Chls. Of the 96 Chls in the monomeric *T. elongatus* x-ray structure (*11*, *12*), 89 could be identified in the FRL-PSI structure (see below). However, PsaK and its associated Chls are poorly resolved in both the *T. elongatus* PSI structure and the FRL-PSI structure presented here and are incompletely modeled in both (*11*, *12*). In the *T. elongatus* PSI structure, PsaK is modeled as a poly-Ala helix coordinating two Chls. In the FRL-PSI structure presented here, PsaK is partially modeled as a poly-Ala helix and partially modeled with side chains, and a single Chl molecule and its coordinating His side chain are apparent in the density map and have been included in the complete model. Notably, PsaK does not exhibit a specific FRL isoform encoded within the FaRLiP gene cluster in *F. thermalis* (*4*, *5*). There are three genes for PsaK paralogs that are expressed in WL, which may lead to structural variation that results in poor local resolution for PsaK in the density map. It is likely that an additional PsaK-associated Chl is present in FRL-PSI but unresolved in the density because in the *Synechocystis* sp. PCC 6803 PSI structure, the backbone carbonyl of PsaK Gly^79^ residue has been shown to H bond to a water molecule providing the axial coordination of this Chl (*15*) and this Gly is conserved among the *T. elongatus* PSI PsaK sequence and the three *F. thermalis* PsaK sequences. The Chl associated with PsaM in the *T. elongatus* PSI structure is probably a modeling error and is unlikely to be present because it lacks evident coordinating interactions with the protein. Similarly, this Chl is absent in the PSI structure from *Synechocystis* sp. PCC 6083 (*15*) and is thus unlikely to be present in any of the PSI structures. If PsaK coordinates two Chls in FRL-PSI, although one is not resolved in the density map, and the Chl associated with PsaM is not included, then *T. elongatus* PSI coordinates 95 Chls, such as *Synechocystis* sp. PCC 6803 (*15*, *16*), but FRL-PSI only binds 90 total Chls.

In the discussion that follows below, pigments and pigment binding sites are referred to first by the polypeptide chain to which the pigment is assigned together with the last two numbers according to the pigment identifier as first assigned in the structure of PSI from *T. elongatus* (*11*, *12*). For example, the Chl associated with PsaA that is numbered 1120 in the *T. elongatus* PSI structure is referred to here as A20. Of the three Chls assigned to PsaA in the *T. elongatus* PSI structure that could not be identified in the FRL-PSI density, one is likely to be coordinated by PsaK (Chl PsaA 1402/A02) because no coordinating interactions from residues in PsaA can be observed in the structure. The TMH of PsaK closest to the nearby PsaA2 subunit in the monomer coordinates the Chl that was identified. In addition, 7 residues before and 10 residues after the coordinating His (His^76^) could be identified. Although the density map in this region is less well defined compared to the remainder of the structure, we believe that PsaK2 corresponds better to the density map than the PsaK1 and PsaK3 paralogs (a sequence alignment appears in fig. S3).

**Fig. 4:**
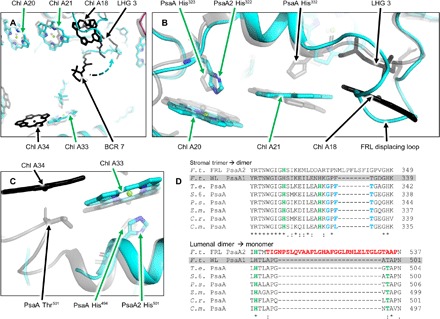
PsaA2 monomer-monomer interface region pigment variation. (**A**) View of this region from the perspective of the adjacent PSI monomer. A dashed arrow colored from cyan to gray in a gradient signifies a difference in the orientation of BCR 7 between the two structures. (**B** and **C**) FRL-PSI Chl dimer and monomer, respectively, where these are trimer and dimer in *T. elongatus* PSI. For (A) to (C), the FRL-PSI and *T. elongatus* structures are superimposed. FRL-PSI is colored, and *T. elongatus* PSI is shown in transparent gray except where the pigment site is not conserved with FRL-PSI, in which case, it is colored solid black. Pigment sites and residues are labeled, and their corresponding arrows are colored either green, signifying that the residue or pigment site is conserved, or black, signifying that the residue or pigment site is not conserved. All Chls are shown as tetrapyrrole rings, and only selected residues are shown for clarity. (**D**) Sequence alignments corresponding to the FRL-PSI dimer (top) and monomer (bottom) for all currently known PSI structures and the WL-acclimated *F. thermalis* PsaA sequence (gray highlight, no structure) are shown. Blue letters correspond to residues that coordinate LHG 3, green letters correspond to residues that coordinate a Chl, and red letters correspond to residues that could not be modeled because of poor density as described in the main text. *F.t.*, *F. thermalis*; *T.e.*, *T. elongatus*; *S.*6., *Synechocystis* sp. PCC 6803; *P.s.*, *Pisum sativum*; *Z.m.*, *Zea mays*; *C.r.*, *Chlamydomonas reinhardtii*; *C.m.*, *Cyanidioschyzon merolae*.

Changes in pigment binding near the monomer-monomer interface region (corresponds to “PsaA2 interface region” in Fig. 3B) are shown in detail in Fig. 4. A trimer of Chls toward the stromal face and a dimer of Chls toward the lumenal face in *T. elongatus* PSI each contain one fewer Chl in FRL-PSI, converting the trimer into a dimer and the dimer into a monomer, respectively. The stromal trimer in *T. elongatus* PSI is composed of Chl A20, Chl A21, and Chl A18 (Fig. 4, A and B). While the axial His residue coordinating Chl A20 is conserved in all other PSI sequences and structures, the axial His coordinating Chl A21 is not conserved in FRL-PSI (Fig. 4D). Nevertheless, this pigment is still present in the density map of FRL-PSI. Chl A18 in *T. elongatus* PSI (and all other known PSI structures) is unique because it is the only Chl coordinated by the headgroup of a phosphatidylglycerol, LHG 3. However, both Chl A18 and LHG 3 are missing in the FRL-PSI structure. Instead, the LHG 3/Chl A18 binding site is devoid of these cofactors and is replaced by a long protein loop region that is inserted in the PsaA2 sequence (Fig. 4B). This inserted loop, which is unique to FRL-PSI, is well resolved in the density map. None of the three residues that coordinate the headgroup of LHG 3 in *T. elongatus* PSI are conserved in FRL-PSI (Fig. 4D). The absence of Chl A18 and its coordinating LHG 3 is unexpected because these two cofactors are conserved in all other known PSI structures. Moreover, a phosphatidylglycerol is even found in a similar location in the heliobacterial photosystem (fig. S5) (*18*), implying that conservation of this lipid binding site is a fundamental property of most type 1 reaction centers. As a result, the planes of Chl A20 and Chl A21 appear to be less parallel than in the *T. elongatus* PSI structure (fig. S6A), which is a consequence of the missing LHG 3, Chl A18, and the axial ligand to Chl A20. This implies that these two Chls are more weakly coupled energetically in FRL-PSI compared to WL-PSI.

A Chl dimer composed of Chl A33 and Chl A34 is located near the Chl trimer in *T. elongatus* PSI; the axial coordination for these Chls is provided by the backbone carbonyl of PsaA Thr^501^ and the side chain of PsaA His^494^, respectively (Fig. 4, A and C). A sequence alignment suggests that *F. thermalis* WL-PSI retains these ligands and the environment for this Chl dimer but FRL-PSI has additional inserted residues between the two corresponding residues (Fig. 4D). The density for the α helix including PsaA2 His^501^ (analogous to the *T. elongatus* PsaA His^494^) and the density for Chl A33 are resolved at 5*σ, but the map sharply terminates where the nonconserved region begins and no density is observed for the Chl A34 site (fig. S6B). The JPred4 server (*19*) was used to make secondary structure predictions of the nonconserved region (data S1). The JPred4 algorithm predicted the region to be largely solvent accessible and disordered except for a small, ~5-residue helix, suggesting that the nonconserved region comprises a large, flexible surface loop. It is possible that Chl A34 is simply unresolved in the density map presented here; however, even if this were the case, it seems unlikely that the stability required to maintain an energetically coupled Chl dimer would be maintained in such a flexible loop.

The conversion of a Chl trimer (Chl A20, Chl A21, and Chl A18) in WL-PSI to a dimer (Chl A20 and Chl A21) in FRL-PSI, the conversion of a dimer (Chl A34 and Chl A33) in WL-PSI to a monomer (Chl A33) in FRL-PSI, and the additional reorganization of a carotenoid molecule (Fig. 4A) are major differences that would result in profound changes to the energetic landscape of the collective antenna system. “Stacked” Chls such as these have been shown to be low-energy sites due to the overlapping conjugated π-systems of the tetrapyrroles; they exhibit energetic coupling that contribute low-energy frequencies to the overall absorption profile of PSI (“far-red Chls”) (*20*).

**Fig. 5:**
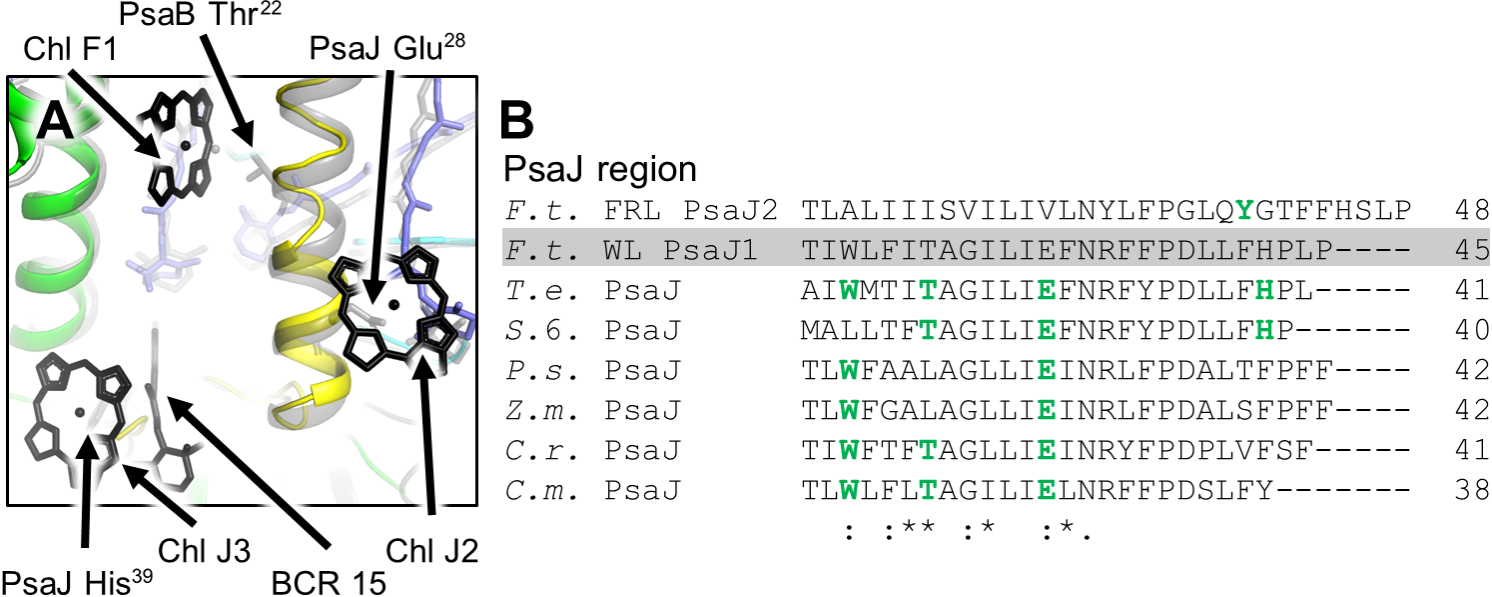
PsaJ-region pigment variation. (**A**) View of this region from outside the monomer. The FRL-PSI and *T. elongatus* structures are superimposed. FRL-PSI is colored, and *T. elongatus* PSI is shown in transparent gray except where the pigment site is not conserved with FRL-PSI, in which case, it is colored solid black. Pigment sites and residues are labeled with a black arrow, signifying that the residue or pigment site is not conserved. All Chls are shown as tetrapyrrole rings, and only selected residues are shown for clarity. (**B**) Sequence alignment corresponding to this region of PsaJ from all currently known PSI structures and the WL-acclimated *F. thermalis* PsaJ1 sequence (gray highlight, no structure) is shown. Green letters correspond to residues that coordinate a Chl or participate in a H-bonding network with one or more water molecules that coordinate a Chl.

Another notable characteristic of FRL-PSI is that, compared to all other known PSI structures, all three Chls coordinated by PsaJ are not observed in the density map and an associated carotenoid conserved in the cyanobacterial PSI structures is not present in the density map (a lipid fills this site in all noncyanobacterial PSI structures). The three PsaJ residues that have coordinating interactions with the three corresponding Chls in the *T. elongatus* are Thr^22^, Glu^28^, and His^39^ (Fig. 5). The former two residues are not conserved in the *F. thermalis* PsaJ2 sequence (Fig. 5B). His^39^ is near the C terminus in both sequences, but *F. thermalis* PsaJ2 contains an extension before the analogous His residue (His^45^ in PsaJ2), causing it to be relocated into a flexible region disconnected from the main complex, an unlikely Chl binding location. A Tyr near the C terminus of PsaJ2, which does not occur in WL-type PsaJ sequences but is fully conserved in PsaJ2 sequences for other FRL-PSI complexes, is a potential H-bonding partner to a Chl f molecule and is further discussed below. The missing carotenoid in FRL-PSI is associated with residues in PsaJ, PsaF, and PsaB in *T. elongatus* PSI. Because PsaJ is located on the periphery of the PSI trimer, it could be that in WL-PSI, these pigments provide bridges to transfer energy to/from another protein in a supercomplex array (*21*). Arrays of PSI trimers, dimers, and monomers associated with PSII dimers have been observed in thylakoid membranes by atomic force microscopy imaging (*22*). PsaJ2 does not coordinate pigments, implying that these interactions might be altered in FRL-PSI.

It is unexpected that FRL-PSI has fewer Chls than WL-PSI because one might expect that FRL-adapted cyanobacteria are light limited in the ecological niches in soil crusts and lower-level microbial mats where FaRLiP is known to occur (*3*–*5*, *23*, *24*). Because Chl f molecules are incorporated into the complex, however, it may be expected that changes are observed in low-energy, Chl multimer sites because uphill energy transfer must occur from Chl f to Chl a. If an energy well is too deep, then excitation energy transfer to P_700_ would become less likely to occur, and loss of the excited state energy could compete with productive photochemistry. By incorporating Chl f molecules into sites where far-red–absorbing Chl a molecules might already have occurred, nature possibly avoided this problem.

### Insights into the binding locations for Chl f

HPLC analyses have shown that ~8% of the total Chls within FRL-PSI are Chl f and the remainder Chls are Chl a (see fig. S1F) (*5*–*10*). If there are ~90 total Chls in FRL-PSI, then ~7 Chls should be Chl f rather than Chl a as discussed above. The authors of several spectroscopic studies of FRL-PSI complexes have recently suggested that one or both of the A_−1_ Chls of the ETC of FRL-PSI might be Chl f molecules (*10*, *25*–*27*). Results from femtosecond transient visible (*25*) and infrared (*26*) absorption spectroscopy, together with an electrochromic band shift observed when P_700_ is photo-oxidized (*10*), have been interpreted to mean that one (or both) of the A_−1_ Chls of the ETC chain in FRL-PSI from *Chroococcidiopsis thermalis* PCC 7203 is Chl f. On the basis of difference Fourier transform infrared (FTIR) spectroscopic analyses of FRL-PSI from *F. thermalis* and density functional theory calculations, another study also concluded that one or both Chls occupying the A_−1_ position in the ETC are Chl f (*27*). These FTIR studies further showed that the H bond between the 13^1^-keto group of P_A_ of the special pair, which is a Chl a_P_′ molecule, and the hydroxyl group of Thr^776^ of PsaA2 is weakened in FRL-PSI. The FRL-PSI structure supports this latter conclusion (fig. S7A). The distance between the H-bonding, hydroxyl group of PsaA2 Thr^776^ and the 13^1^ keto-oxygen of P_A_ in FRL-PSI is 3.47 Å, whereas the corresponding distance is 2.98 Å in *T. elongatus* PSI and only 2.82 Å in *Synechocystis* sp. PCC 6803 PSI. As a result, the charge distribution of excited-state special pair, P*, should be more evenly distributed over P_A_ and P_B_ in FRL-PSI (*27*), which would probably alter the partitioning of electrons into the A and B branches of the ETC chain. Consistent with these small differences in the arrangement of the P_A_ and P_B_ Chls, a high-resolution photobleaching difference spectrum for P_700_ shows a small but reproducible red shift of about 3 to 4 nm compared to WL-PSI (*6*). Regardless of whether the A_−1_ positions are occupied by Chl a or Chl f, the increase in symmetry between the two ETC branches could imply that both A_−1_ sites are occupied by the same type of Chl molecule. However, no features in the cryo-EM structural model support the assignment of any of the six Chls of the ETC as Chl f. There are no residues that could contribute H bonding to a formyl group to confer site selectivity for specific binding of Chl f rather than Chl a. Furthermore, amino acid residues near the pigments of the ETC are very highly conserved and nearly identical to the same regions of WL-PSI (fig. S3), making it difficult to rationalize structurally why Chl f would replace Chl a. These observations obviously disagree with the interpretation of the published spectroscopic observations. Spectroscopic evidence already strongly supports the assignment of the P_700_ special pair in FRL-PSI as a Chl a_P_/Chl a_P_′ heterodimer (*6*, *10*), and we have modeled the two corresponding Chls accordingly. Considering the current discrepancy between the interpretation of the spectroscopic and structural data concerning the assignment of the four A_−1_ and A_0_ pigments as Chl a or Chl f, we have modeled all of them as Chl a until their identities are established by additional experimentation.

The structural difference between Chl f and Chl a is very small: Chl f has a formyl substituent at the C_2_ position on the chlorin ring rather than the methyl group for Chl a. Thus, two hydrogen atoms are replaced by an oxygen atom, and a carbon-hydrogen bond is replaced by a carbon-oxygen double bond. As was shown for the similar problem in distinguishing Chl b from Chl a molecules in the light-harvesting complex II (LHCII) from algae and plants (*28*–*30*), resolution of at least ~2.7 Å or better would likely be required for direct detection of such a small difference, and this resolution is difficult to achieve using current structural biology techniques on FRL-PSI, especially cryo-EM. Of the six PSI structures solved by cryo-EM in the PDB, the highest resolution achieved thus far is 2.9 Å (*31*, *32*), and of the 12 x-ray structures of PSI that have been solved to date, the highest resolution achieved has been 2.5 Å (*11*, *12*). Future improvements in crystallization and cryo-EM methods may achieve the resolution required to observe these structural details. Despite the current limits on resolution, the protein environment and sequence comparisons may be used to infer the locations of these small but important structural differences indirectly. To this end, we have performed a thorough analysis of the environment of each Chl molecule.

**Fig. 6:**
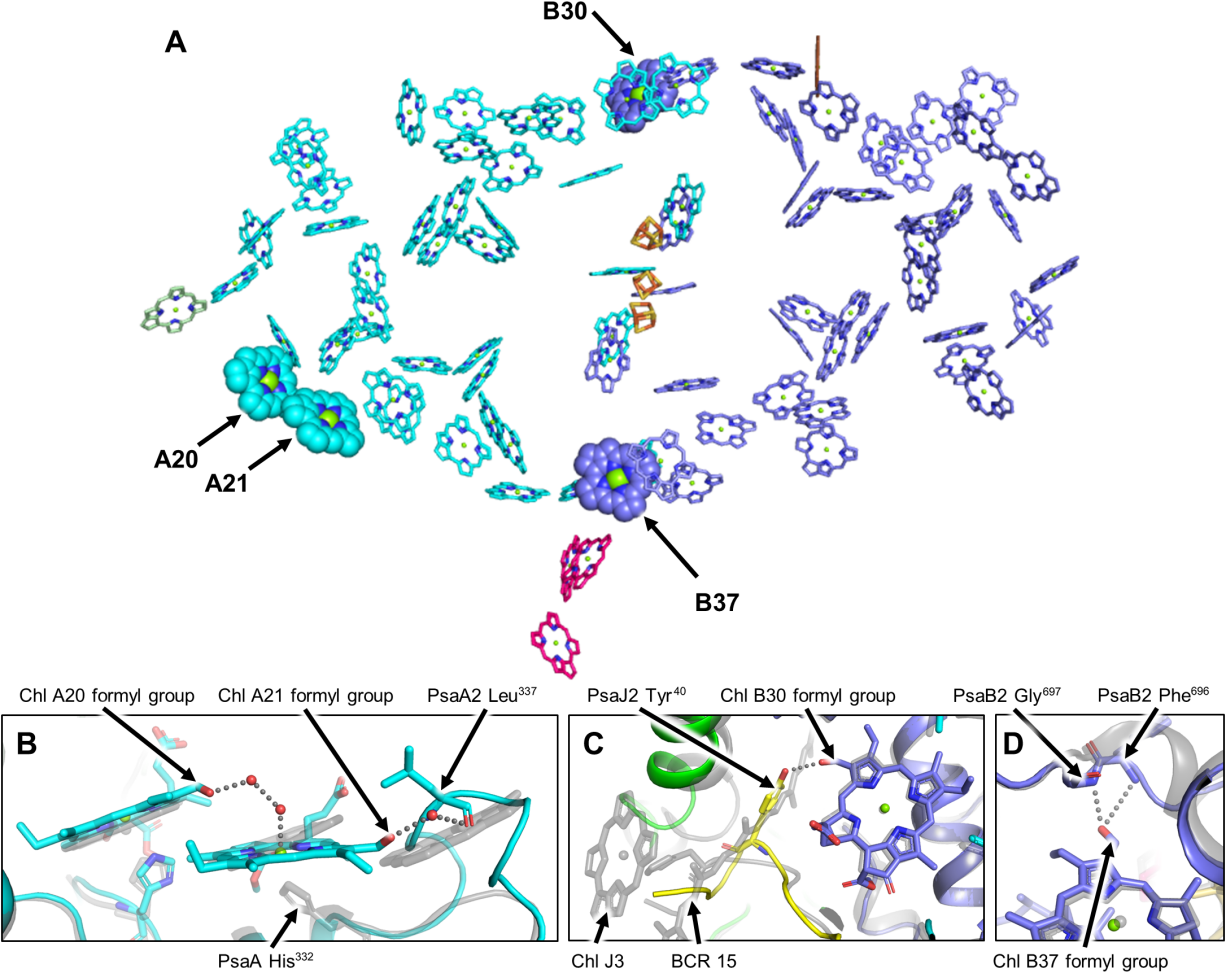
Locations of Chl f molecules. (**A**) Chl sites in a FRL-PSI monomer as tetrapyrrole rings only. Arrows with labels designate sites that are assigned as Chl f molecules in the structure presented here. These Chls are shown as spheres. The view shown is down onto the stromal face. [4Fe-4S] clusters are shown for orientation. (**B**) Possible interactions to provide coordination if Chl A21/A20 are a Chl f dimer. Red spheres are hypothetical water molecules. (**C**) PsaJ Tyr^40^ that likely donates a H bond to the formyl group of Chl B30 if it is a Chl f. (**D**) FRL-specific region of PsaB2 that may H-bond to a formyl substituent on Chl B37. FRL-PSI is colored, and *T. elongatus* PSI is shown in transparent grey in (B) to (D). In (B) to (D), all Chls except Chl f are shown as tetrapyrrole rings, and only selected residues are shown for clarity.

Because Chl f has a formyl group rather than a methyl group at the C_2_ position of the tetrapyrrole ring, this substituent may participate in H bonding by accepting a H bond from nearby water or amino acid donors. This feature was crucial for the identification of Chl b in early structural studies of LHCII, in which site specificity for Chl b is conferred by H bonding to the formyl substituent at C_7_ in the tetrapyrrole ring of that pigment (*30*). H bonding to the C_7_ formyl group is believed to occur for all six Chl b molecules in higher plant LHCII, and this observation was used to identify Chl b tentatively in LHCII initial, lower-resolution structures determined by electron diffraction (*30*). Adopting this same approach, the C_2_ position of each Chl in the FRL-PSI structure was inspected for nearby residues that could participate in direct H bonding or in a H-bonding network, especially focusing on regions that exhibit substantial sequence differences in FRL-PSI relative to *F. thermalis* WL-PSI and *T. elongatus* PSI (Fig. 3). We identified four potential Chl f sites (Fig. 6A) and discuss each of them here. Note that additional binding sites will likely be identified once water molecules can be modeled into the FRL-PSI structure because water can participate in forming a H bond to the formyl moiety of Chl b in LHCII and by analogy to the formyl side chain of Chl f in FRL-PSI.

#### **Chl A20/A21 dimer**

As depicted in Fig. 4, the trimer of Chls found in the *T. elongatus* PSI structure, which is likely present in the WL-PSI structure, does not occur in FRL-PSI. In addition, the orientation of Chl A20 with respect to the nearby Chl A21 is not parallel, as in the *T. elongatus* PSI structure (fig. S6A), modifying the coupling between the two pigments. The axial ligand to Chl A21 is not conserved in FRL-PSI, leaving the possibility that axial coordination occurs via a water molecule in a H-bonding network involving the C_2_ formyl substituent of Chl A20 if that Chl was Chl f as shown in Fig. 6B. It is conceivable that the oxygen atom of a formyl group at the C_2_ position of Chl A20 H-bonds with one to two water molecules to form the axial coordination of Chl A21. Furthermore, the loop insertion that displaces the Chl A18/LHG 3 binding site could provide a H bond from the backbone carbonyl oxygen of PsaA2 Leu^337^ to a formyl substituent on Chl A21, converting the Chl A20/A21 into a Chl f dimer as shown in Fig. 6B. Spectroscopic studies on the FRL-PSI of *C. thermalis* PCC 7203 strongly suggest the presence of an energetically coupled Chl f dimer (*10*). Although other dimeric Chl pairs exist within the FRL-PSI structure, the marked change in structure (implementing a loop and removing a highly conserved lipid/Chl binding site) relative to the WL-PSI is good evidence that the Chl A20/A21 dimer represents a pair of Chl f molecules, and the corresponding molecules have been modeled as such in the structure presented here. Because this site was already a low-energy site in WL-PSI and presumably optimized for efficient uphill energy transfer to other Chl a molecules, evolution may have simply modified this site further. Replacement of these two Chl a molecules by Chl f could extend light harvesting further into the far-red/infrared region while avoiding the formation of an energy transfer well that might reduce the overall efficiency of charge separation in FRL-PSI (*6*).

#### *Chl B30*

As shown in Fig. 5, the three Chls coordinated by PsaJ and an associated carotenoid in the same region are not observed in FRL-PSI. The C-terminal extension of PsaJ2 in FRL-PSI results in a looping region that eliminates the Chl J3/β-carotene (BCR) 15 binding site (Fig. 6C). Within this additional loop is Tyr^40^ of PsaJ2 whose side-chain hydroxyl substituent is in close proximity to the C_2_ position of Chl B30. When Chl f is modeled into this site, the distance between the hydroxyl oxygen atom of Tyr^40^ of PsaJ2 and the C_2_ formyl oxygen Chl B30 is ~2.5 Å, suggesting the presence of a H bond. PsaJ2 exhibits only ~40% sequence homology to non-FaRLiP paralogs (table S2) and, in FRL-PSI PsaJ2, lacks all the pigments likely associated with PsaJ1 in WL-PSI. Thus, it seems probable that these large and conserved differences from WL-PSI accommodate a Chl f molecule and thus enhance FRL absorption. With this in mind and because PsaJ2 Tyr^40^ is an ideal distance to H bond with a formyl group on Chl B30, we have modeled a Chl f molecule into this site in the FRL-PSI structure.

#### *Chl B37*

Near the center of the PSI trimer is the Chl B37/38 dimer that was proposed to be a Chl f dimer (*10*). There are two atoms that could donate a H bond to the C_2_ formyl substituent if Chl B37 is Chl f: the backbone amide nitrogen of Phe^696^ and the backbone amide nitrogen of Gly^697^ (Fig. 6D). This small helical region is conserved between *T. elongatus* PSI and *F. thermalis* WL-PSI but is not conserved in FRL-PSI, instead losing its helical character, perhaps to accommodate a Chl f molecule in this site. The dimer partner of Chl B37, Chl B38, has no apparent H-bonding partners nearby the C_2_ substituent, and the protein environment here is well conserved between *T. elongatus* PSI and WL-PSI. While we cannot rule out Chl B38 being a Chl f molecule, we see no structural evidence that both of these Chls are Chl f molecules. Nonetheless, because of the low sequence identity in the vicinity of Chl B37 and the associated structural changes and potential H-bond donors near the C_2_ position of the chlorin ring, we have modeled Chl B37 as a Chl f molecule.

#### *Chl A38*

Chls A38 and A39 form a dimer that was suggested to be a pair of energetically coupled Chl f molecules (*10*), which lie toward the outside of the PSI trimer near PsaF, PsaJ, and Chl B30 but closer to the stromal face. Four atoms from residues in PsaF and a carbonyl group from Chl A39 are located near the C_2_ substituent on Chl A38 and could participate in H bonding: the carbonyl oxygen from Ile^95^, the carbonyl oxygen from Gly^96^, the carbonyl oxygen from Gly^99^, and the amide nitrogen from Gly^99^ (fig. S7B). All of these residues, however, are conserved in FRL-PSI, WL-PSI, and *T. elongatus* PSI. The carbonyl groups in the TMH of PsaF2 are pointed outward, away from the TMH and more so than in PsaF in the *T. elongatus* structure. This may partly be due to a lack of steric stability on the opposite side of the TMH; in the *T. elongatus* PsaF structure (and conserved in WL-PsaF1), Trp^76^ lies on the opposite side of the TMH from Chl A38, where it is pinched between the surface helix from PsaF and Chl F1. However, in FRL-PSI, this residue aligns with the slightly smaller Phe^94^, which is not sterically hindered by a Chl molecule (fig. S7B). Recall that Chl F1 is not present in the FRL-PSI structure and the amino acid that provides its axial coordination, PsaJ2 Thr^22^ is not conserved (Fig. 3B and fig. S3). Furthermore, PsaF2 and PsaJ2 in FRL-PSI have only ~50 and 39% sequence identity with the analogous subunits from *T. elongatus*, respectively. If Chl A38 were a Chl f molecule, then these differences might be important for establishing the specificity of this site.

The other Chl in the dimer pair with Chl A38 is Chl A39. If this Chl dimer is composed of two Chl f molecules, then there is nothing in the FRL-PSI structure to suggest that H bonding exists nearby the C_2_ substituent of Chl A39. Although multiple atoms nearby could participate in H bonding, every atom is conserved in sequence and/or position with the structure from *T. elongatus* PSI. Possible H-bonding partners in this region are the hydroxyl group of PsaA2 Thr^44^, the carbonyl group of PsaA2 Thr^44^, and the carbonyl groups on ring 5 and the phytyl tail of Chl A11.

#### *Chl A33*

While we described the environment of Chl A33 in *T. elongatus* PSI in the context of its missing Chl dimer partner in detail previously, the question arises whether Chl A33 is replaced by a Chl f to make use of this potentially adapted low-energy site. The C_2_ methyl substituent of Chl A33 in *T. elongatus* points directly toward the ring of Chl A25 whose site and amino acid environment are well conserved between FRL-PSI and *T. elongatus* PSI. If Chl A33 is a Chl f, then its formyl group would likely participate in or clash with the axial coordination with Chl A25, both of which seem unlikely and argue against Chl A33 being a Chl f molecule.

#### *“Linker” Chl A40 and Chl B39*

Nürnberg *et al.* (*10*) also suggested that one of the linker Chl A40 and Chl B39, which are antenna pigments that likely link the bulk antenna to the Chls of the ETC, could be Chl f molecules. The C_2_ positions for both of these pigments lie in highly hydrophobic regions of the structure, and the only nearby residues containing atoms that could be involved in a H-bonding network with a possible Chl f are well conserved among the non-FaRLiP sequences analyzed here. Thus, there is no structural evidence to assign either of these two Chls as Chl f molecules.

### Concluding remarks

The cryo-EM structure of FRL-PSI reveals important structural differences that cause changes in the energy landscape of the antenna pigments, which should allow it to make efficient use of FRL for energy transfer and photochemistry. While some of the pigments discussed here show clear structural evidence supporting their assignment, as Chl f and other possible sites are discussed above (also see fig. S7B), note that, consistent with the results from FTIR difference spectroscopy, the formyl groups of Chl f do not necessarily need to be H-bonded (*27*). Therefore, although the search for nearby H-bonding partners to the formyl groups of Chl f may provide clues as to which pigments could be Chl f (e.g., Chl B30), the absence of H-bonding partners does not exclude protein environments that cannot provide these interactions, which could be provided by water molecules not visible at this resolution and possibly explaining why we could only identify four of the expected seven Chl f molecules.

We hypothesize that the strongest structural evidence for the locations of Chl f molecules at present is the sites of Chl A20, Chl A21, Chl B30, and Chl B37, accounting for four of the anticipated seven Chl f molecules indicated by pigment analyses. The structural changes near Chl A20 and Chl A21, especially the absence of a highly conserved and structurally important lipid molecule, imply substantial changes in this site to accommodate Chl binding and energy transfer. The PsaJ2 Tyr^40^ interaction with Chl B30 appears to exhibit obvious H bonding with a nearby substituent, presumably a formyl moiety, at the C_2_ position. The nearby PsaF2 and PsaJ2 subunits, which exhibit low sequence identity relative to their WL counterparts, also suggest important changes in this region—probably to incorporate Chl f molecules as proposed here. Similarly, the nonconserved region surrounding Chl B37, which comprises a PsaB surface loop and nearby subunits PsaI2 and PsaL2, implies structural variations to accommodate the presence of a Chl f molecule. The placement of Chl f molecules on the periphery of the complex may have served two purposes. First, it was probably easier to accommodate the considerable structural remodeling changes that were required by making those changes at the periphery of the highly conserved PSI complex. Second, there might have been an advantage in placing Chl f away from the ETC to avoid any interference that could affect electron transfer efficiency.

This structure provides a platform to direct biochemical and biophysical experimentation [such as the characterization of mutants and two-dimensional (2D) electronic spectroscopy] and serves as the basis for site-targeted mutagenesis studies to verify binding sites for Chl f. It will also inform attempts to introduce the capacity for FRL utilization into plants, which could substantially improve crop yields by increasing the wavelengths of light supporting oxygenic photosynthesis (*6*, *33*, *34*).

## MATERIALS AND METHODS

### Cyanobacterial strain and growth conditions

*F. thermalis* PCC 7521(*F. thermalis*) was obtained from the Pasteur Culture Collection (*35*). Cultures of *F. thermalis* were grown in B-Hepes medium (*36*), a modified BG-11 medium that is buffered at pH 8 with 4.6 mM Hepes as described previously (*9*). Liquid cultures in culture tubes or bottles were sparged with 1% (v/v) CO_2_ in air at 38°C. Standard WL growth conditions were produced using cool white fluorescent lights (250 μmol photons m^−2^ s^−1^). For growth of liquid cultures under FRL conditions, FRL (28 μmol photons m^−2^ s^−1^) was provided with 720-nm light-emitting diode light panels (L720-06 AU) (Marubeni, Santa Clara, CA, USA) or with a combination of green and red light transmitting filters, as described previously (*9*). For full acclimation under FRL, liquid cultures in culture bottles were grown with stirring and sparging in FRL for 6 to 8 weeks, with dilution and medium exchange every 2 weeks.

### Isolation of trimeric PSI complexes

PSI complexes were isolated from cells grown in WL or FRL as previously described (*4*, *6*) with minor modifications. For purification of photosystem complexes, all glassware and chemical reagents were sterilized either by autoclaving or by filtration, as appropriate. *F. thermalis* cells were harvested by centrifugation from liquid cultures grown under WL or FRL, washed once with the photosystem isolation buffer (PIB), which is composed of 50 mM MES (pH 6.5), 10 mM CaCl_2_, and 10 mM MgCl_2_, through resuspension and centrifugation. Cells were resuspended in PIB and lysed first by sonication and then by three passages through a chilled French pressure cell operated at 138 MPa. After centrifugation at 6900*g* to remove unbroken cells and large cell debris, total membranes were pelleted by ultracentrifugation (126,000*g*) for 1 hour. The isolated membranes were washed once by resuspension in PIB and were pelleted by ultracentrifugation. The membranes were then resuspended in PIB with a glass Teflon Dounce homogenizer. The Chl concentration was measured and adjusted to 0.4 mg Chl ml^−1^ by dilution with PIB. Membranes were then solubilized by addition of DM to a final concentration of 1% (w/v) and incubated at 4°C for 1 hour. Solubilized membranes were separated from insoluble debris by centrifugation (24,000*g* for 20 min). The solubilized membranes were loaded onto 5 to 20% (w/v) sucrose gradients containing 0.1% DM in PIB, and the gradients were centrifuged for about 18 hours at 108,000*g*. Green-colored fractions, containing mostly trimeric FRL-PSI complexes and dimeric FRL-PSII complexes, were collected from the sucrose gradients (see fig. S1A), dialyzed against the PIB, and concentrated using Millipore Centriprep 100 K Centrifugal Filtration Devices (EMD Millipore, Darmstadt, Germany). For further purification, green fractions containing trimeric FRL-PSI complexes and the dimer fraction containing FRL-PSII were loaded onto 5 to 20% (w/v) sucrose gradients containing 0.1% DM and subjected to a second ultracentrifugation at 108,000*g* for 18 hours (fig. S1C). Fractions containing trimeric FRL-PSI and dimeric FRL-PSII were collected and dialyzed against PIB. After concentration with centrifugal devices, the purified trimeric FRL-PSI and dimeric FRL-PSII complexes were resuspended in PIB containing 0.05% (w/v) DM and 5% (w/v) glycerol. The absorption and 77-K fluorescence emission spectra of a typical preparation of trimeric FRL-PSI complexes, dimeric FR-PSII complexes, and trimeric WL-PSI complexes are shown in fig. S1 (B and D). The purity of the isolated complexes was assessed by tryptic peptide fingerprinting by mass spectrometry. After purification, samples were immediately shipped to Arizona State University at 4°C. An Amicon Stirred Cell (EMD Millipore, Darmstadt, Germany) was used to exchange the buffer into 300 mM tricine (pH 8.0 with NaOH) and 50 mM NaCl, and the solution was subsequently concentrated to protein (~2 mg/ml). These were the conditions in which the protein was plunge-frozen for cryo-EM data collection.

### Pigment analyses

The contents of Chl a and Chl f in trimeric WL-PSI and FRL-PSI complexes from *F. thermalis* PCC 7521 were determined by reversed-phase HPLC as previously described (*9*), as shown in fig. S1 (E and F). The Chl pigment content of FRL-PSII (FRL-PSII) complexes is shown for comparison. Pigments were generally quantified by absorption spectroscopy as previously described. Equations based on published molar extinction coefficients (*37*) for calculating Chl a and Chl f concentrations can be found in (*38*). Chl a and Chl f concentrations were also sometimes determined from absorption spectra of extracts from the Q_y_ absorption band of Chl a at 665 nm and of Chl f at 707 nm using the molar extinction coefficients in methanol for Chl a (70.54 mM^−1^ cm^−1^) (*39*) and Chl f (71.11 mM^−1^ cm^−1^). The trimeric FRL-PSI complexes from *F. thermalis* PCC 7521 contained 92% Chl a and ~8% Chl f.

Quantitative analyses were also performed using the content of phylloquinone as an internal standard to determine the concentration of PSI complexes in solutions (PSI binds two phylloquinone molecules, for which standards can be purchased). All sample preparation procedures were performed under very dim light. Pigments were extracted from isolated PSI complexes by dilution in acetone/methanol (7:2, v/v) to a final Chl concentration of 100 μg ml^−1^. After 20 min, the extracts were centrifuged to pellet-precipitated proteins, and the resulting supernatant was filtered through a 0.2-μm polytetrafluoroethylene membrane syringe filter. An aliquot (100 μl) of the eluate was analyzed by reversed-phase HPLC on an Agilent 1100 HPLC system (Agilent Technologies, Santa Clara, CA) with an analytical Discovery C18 column (4.6 mm by 25 cm) (Supelco, Sigma-Aldrich, St. Louis, MO) and a diode array detector. The gradient elution program was slightly modified from a previous method (*40*). The elution gradient [B, minutes] using 100% methanol as solvent A and 100% isopropanol as solvent B was set as solvent A as [0%, 0 min], [97%, 75 min], [97%, 85 min], and [0%, 90 min] at a flow rate of 0.5 ml min^−1^. The column eluate was monitored at 270, 453, 458, 665, and 705 nm for the absorbance maxima of phylloquinone, β-carotene, echinenone, Chl a, and Chl f, respectively. The area under the corresponding peaks were used to determine the amount of each pigment in the sample according to Eq. 1 below, where FR is the flow rate, ε is the corresponding extinction coefficient, and *l* is optical path (0.98 cm). If the absorbance of any pigment exceeded one optical density unit, then a 1:3 or 1:5 dilution was performed, and the analysis was repeated.

$$M[\text{μmol}]=\frac{\text{Area}\;[m\text{OD}\cdot \text{min}]\cdot \text{FR}[\text{ml}\;{\text{min}}^{-1}]}{\varepsilon \;[M^{-1}\;{\text{cm}}^{-1}]\cdot l\;[\text{cm}]}$$ (1)

The area under the peak was calculated using Igor Pro (WaveMetrics Inc., Lake Oswego, OR) software. If peaks were merged, then a deconvolution procedure was used to fit the peaks with Gaussian curves. The extinction coefficients for Chl a and Chl f were 70,540 and 71,110 M^−1^ cm^−1^, respectively (*37*). The extinction coefficients used for β-carotene and echinenone (141,000 and 120,000 M^−1^ cm^−1^, respectively) were estimated on the basis of values for similar solvent conditions (*41*, *42*). The inline extinction coefficient for phylloquinone was experimentally determined to be 17,900 ± 500 M^−1^ cm^−1^ by running a standard (Vitamin K_1_ from Sigma-Aldrich, CAS number 84-80-0). The data reported are averages for 12 determinations, which resulted from two independent preparations of each type of PSI complex (WL-PSI and FRL-PSI), two independent solvent extractions for each PSI sample, and three independent injections of the resulting extracts on the HPLC for each extract.

### Grid preparation

A 3-μl drop of FRL-PSI complex (1.5 mg/ml) was applied on to a holey carbon grid (C-flat 1.2/1.3 Cu 400-mesh grids, Protochips, Raleigh, NC). Excess liquid was blotted away for 6 s to form a vicinal film on the grid, which was then immediately vitrified by plunging the grid into liquid ethane cooled by liquid nitrogen. The grids were stored in liquid nitrogen until data collection.

### Data collection

Imaging of FRL-PSI was performed on a Titan Krios G2 transmission electron microscope (Thermo Fisher Scientific/FEI, Hillsboro, OR). Images were recorded on a K2 Summit direct electron detect camera (Gatan, Pleasanton, CA) in super-resolution mode. The defocus varied from −1 to −3 μm, and the nominal magnification was 47,600, corresponding to a super-resolution pixel size of 0.53 Å at the specimen level. The counting rate was adjusted to 7.166 *e*^−^ pixel^−1^ s^−1^. The total exposure time was 8 s, accumulating to a dose of 57.328 *e*^−^ Å^−2^. In total, 6593 micrograph images were collected. SerialEM automated data collection using the K2 Summit and Titan Krios GS resulted in 3692 LZW-compressed tiff movie files (*43*).

### Data processing

Dose-fractionated movie stacks were gain-corrected, aligned, and dose-weighted using IMOD (*44*), and motion-corrected using MotionCor2 (*45*) within RELION 3.0 (*46*). Gctf (*47*) version 1.06 was used for estimation of defocus. About two thousands particles were selected by manual picking, and 2D classes were constructed from these in RELION 3.0; these classes were then used as templates for autopicking of all micrographs, yielding 213,528 picked particles. Two rounds of 2D classification of these particles were performed, and well-resolved classes were chosen yielding 178,666 particles. An initial ab initio model was created using RELION 3.0’s “InitialModel” function. The “Refine3D” function was used to create a higher-resolution model of the density map using the initial model as a template. Masking and postprocessing in RELION 3.0 resulted in a global resolution of 3.5 Å without applying symmetry. The contrast transfer function values were refined, and Bayesian polishing was performed in RELION 3.0. The final particle dataset was refined using C_3_ symmetry. After masking and postprocessing, the global resolution was determined using the gold-standard Fourier shell correlation of 0.143 cutoff criterion to be 3.19 Å (fig. S2) (*46*, *48*).

### Model building

An initial model was constructed from the *T. elongatus* PSI x-ray structure (PDB ID, 1JB0) (*11*). *T. elongatus* PSI subunits were individually isolated into separate files to be used as templates, and a structure-based homology model was constructed using the corresponding FRL-PSI subunit sequence using the SWISS-MODEL server (*49*). The new subunit models were reassembled by performing superpositions in PyMOL (*50*) and were combined into a single file. For this initial model, all cofactors were maintained from the *T. elongatus* PSI structure. The model was fit into the density using University of California, San Francisco (UCSF) Chimera (*51*) and was manually edited using Coot (*52*). The model was further refined into the density using the real_space_refine function in Phenix (*53*, *54*). Iterations of manual correction and automated refinement in Phenix led to the final molecular model.

## Supplementary Material

http://advances.sciencemag.org/cgi/content/full/6/6/eaay6415/DC1

Download PDF

Data S1

Data S2

The structure of Photosystem I acclimated to far-red light illuminates an ecologically important acclimation proces in photosynthesis

## SUPPLEMENTARY MATERIALS

Supplementary material for this article is available at http://advances.sciencemag.org/cgi/content/full/6/6/eaay6415/DC1 Fig. S1. Isolation and characterization of trimeric WL-PSI and FRL-PSI and dimeric FRL-PSII complexes from *F. thermalis* PCC 7521. Fig. S2. Resolution of the FRL-PSI density map. Fig. S3. Sequence alignments of PSI subunit polypeptides. Fig. S4. Reversed-phase HPLC elution profiles identifying various pigments. Fig. S5. LHG 3 and Chl A18 site conservation in all type 1 reaction center structures excluding the FRL-PSI structure presented here. Fig. S6. Details of A20/A21 and A33 Chls. Fig. S7. P_A_ H bonding and possible Chl f A38. Table S1. Cryo-EM data collection, refinement, and validation statistics for FRL-PSI. Table S2. Sequence identity matrices of homologous PSI subunit polypeptides from *F. thermalis* and *T. elongatus*. Table S3. Superposition RMSD comparing *T. elongatus* PSI (PDB ID, 1JB0) subunits to *F. thermalis* FRL-PSI subunits (PDB ID, 6PNJ). Data S1. (JPred4 output). Data S2. (caption, data separate). Final PDB verification report for 6PNJ. References (*55*, *56*)

## Appendix

View/request a protocol for this paper from *Bio-protocol*.
