# Supplementary material for: The structure of Photosystem I acclimated to far-red light illuminates an ecologically important acclimation process in photosynthesis
Source: Sci Adv. 2020 Feb 5;6(6):eaay6415. doi: 10.1126/sciadv.aay6415 (PMC7002129; doi:10.1126/sciadv.aay6415)
Supplement: http://advances.sciencemag.org/cgi/content/full/6/6/eaay6415/DC1 [file supp_6_6_eaay6415__index.html]

Science Advances | Science AdvancesAAASSearchScience AdvancesMenu

## Supplementary Materials

**The PDFset includes:**

- Fig. S1. Isolation and characterization of trimeric WL-PSI and FRL-PSI and dimeric FRL-PSII complexes from *F. thermalis* PCC 7521.
- Fig. S2. Resolution of the FRL-PSI density map.
- Fig. S3. Sequence alignments of PSI subunit polypeptides.
- Fig. S4. Reversed-phase HPLC elution profiles identifying various pigments.
- Fig. S5. LHG 3 and Chl A18 site conservation in all type 1 reaction center structures excluding the FRL-PSI structure presented here.
- Fig. S6. Details of A20/A21 and A33 Chls.
- Fig. S7. PA H bonding and possible Chl f A38.
- Table S1. Cryo-EM data collection, refinement, and validation statistics for FRL-PSI.
- Table S2. Sequence identity matrices of homologous PSI subunit polypeptides from *F. thermalis* and *T. elongatus*.
- Table S3. Superposition RMSD comparing *T. elongatus* PSI (PDB ID, 1JB0) subunits to *F. thermalis* FRL-PSI subunits (PDB ID, 6PNJ).
- Legends for data S1 and S2
- References (*55*, *56*)

Download PDF

**Other Supplementary Material for this manuscript includes the following:**

- Data S1 (.pdf format). (JPred4 output).
- Data S2 (.pdf format). (caption, data separate). Final PDB verification report for 6PNJ.

**Files in this Data Supplement:**

- Adobe PDF - aay6415\_SM.pdf
- Adobe PDF - aay6415\_Data\_S1.pdf
- Adobe PDF - aay6415\_Data\_S2.pdf
