# Supplementary material for: The structure of Photosystem I acclimated to far-red light illuminates an ecologically important acclimation process in photosynthesis
Source: Sci Adv. 2020 Feb 5;6(6):eaay6415. doi: 10.1126/sciadv.aay6415 (PMC7002129; doi:10.1126/sciadv.aay6415)
Supplement: Download PDF [file aay6415_SM.pdf]

Science Advances
AAAS

advances.sciencemag.org/cgi/content/full/6/6/eaay6415/DC1

Supplementary Materials for

The structure of Photosystem I acclimated to far-red light illuminates an ecologically important acclimation process in photosynthesis

Christopher Gisriel, Gaozhong Shen, Vasily Kurashov, Ming-Yang Ho, Shangji Zhang, Dewight Williams, John H. Golbeck, Petra Fromme, Donald A. Bryant*

*Corresponding author. Email: dab14@psu.edu

Published 19 February 2020, *Sci. Adv.* **6**, eaay6415 (2020)
DOI: 10.1126/sciadv.aay6415

The PDF file includes:

Fig. S1. Isolation and characterization of trimeric WL-PSI and FRL-PSI and dimeric FRL-PSII complexes from *F. thermalis* PCC 7521.
Fig. S2. Resolution of the FRL-PSI density map.
Fig. S3. Sequence alignments of PSI subunit polypeptides.
Fig. S4. Reversed-phase HPLC elution profiles identifying various pigments.
Fig. S5. LHG 3 and Chl A18 site conservation in all type 1 reaction center structures excluding the FRL-PSI structure presented here.
Fig. S6. Details of A20/A21 and A33 Chls.
Fig. S7. P_A H bonding and possible Chl f A38.
Table S1. Cryo-EM data collection, refinement, and validation statistics for FRL-PSI.
Table S2. Sequence identity matrices of homologous PSI subunit polypeptides from *F. thermalis* and *T. elongatus*.
Table S3. Superposition RMSD comparing *T. elongatus* PSI (PDB ID, 1JB0) subunits to *F. thermalis* FRL-PSI subunits (PDB ID, 6PNJ).
Legends for data S1 and S2
References (*55*, *56*)

Other Supplementary Material for this manuscript includes the following:

(available at advances.sciencemag.org/cgi/content/full/6/6/eaay6415/DC1)

Data S1 (.pdf format). (JPred4 output).
Data S2 (.pdf format). (caption, data separate). Final PDB verification report for 6PNJ.

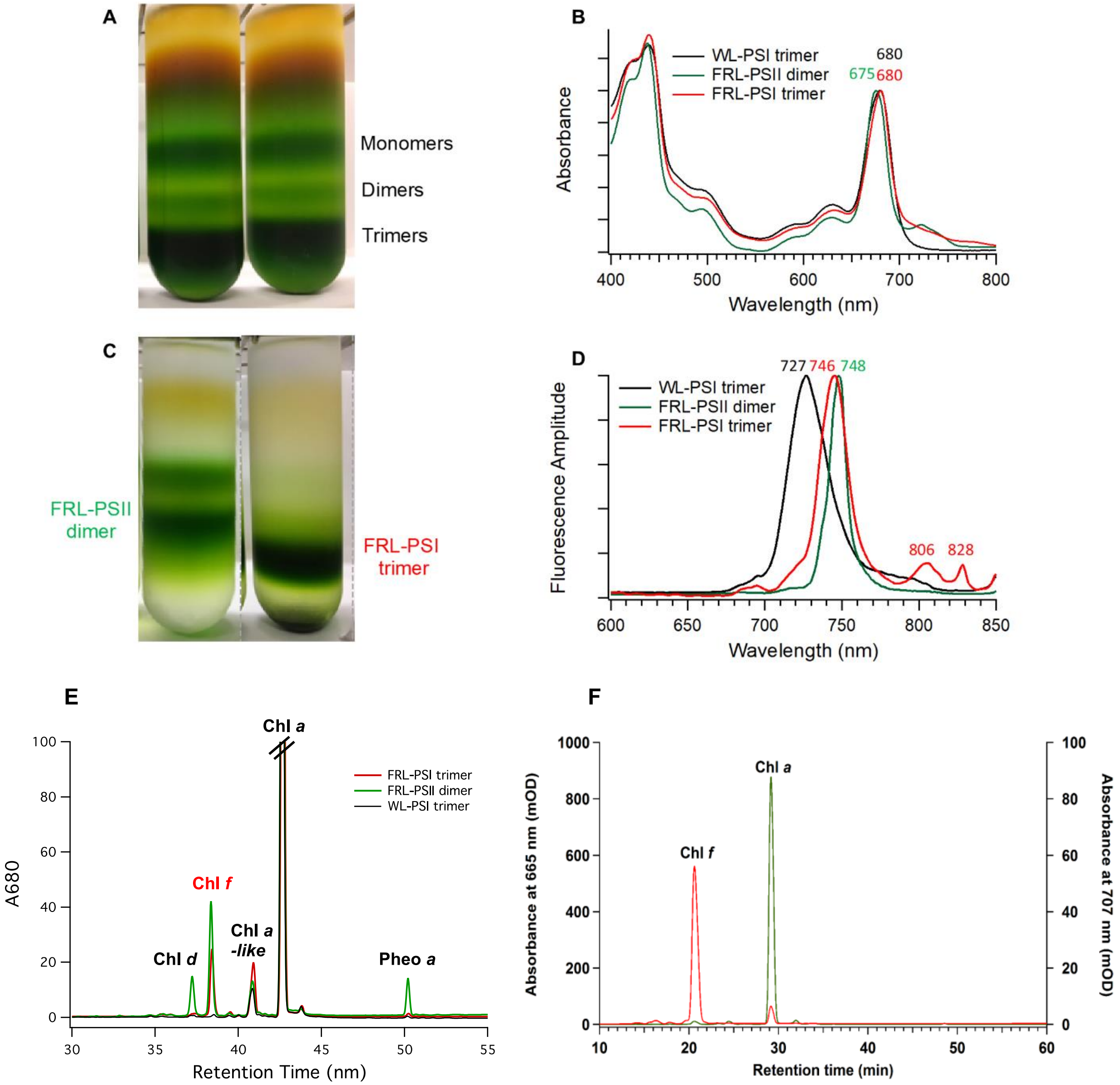

A
Monomers
Dimers
Trimers
B
Absorbance
WL-PSI trimer
FRL-PSII dimer
FRL-PSI trimer
680
675
680
400
500
600
700
800
Wavelength (nm)
C
FRL-PSII dimer
FRL-PSI trimer
D
Fluorescence Amplitude
727
746
748
WL-PSI trimer
FRL-PSII dimer
FRL-PSI trimer
806
828
600
650
700
750
800
850
Wavelength (nm)
E
A680
Chl a
Chl f
Chl a -like
Chl d
Pheo a
FRL-PSI trimer
FRL-PSII dimer
WL-PSI trimer
100
80
60
40
20
0
30
35
40
45
50
55
Retention Time (nm)
F
Absorbance at 665 nm (mOD)
Absorbance at 707 nm (mOD)
Chl a
Chl f
1000
800
600
400
200
0
100
80
60
40
20
0
10
20
30
40
50
60
Retention time (min)

Fig. S1. Isolation and characterization of trimeric WL-PSI and FRL-PSI and dimeric FRL-PSII complexes from *F. thermalis* PCC 7521. A. Separation of photosynthetic complexes from crude thylakoid membranes on initial sucrose gradients by ultracentrifugation. Two faster migrating green bands, containing principally trimeric FRL-PSI and dimeric FRL-PSII complexes, were collected from the gradients and concentrated by ultrafiltration. Photo credit: Dr. Gaozhong Shen, The Pennsylvania State University. **B**. Appearance of gradients after second sucrose gradient ultracentrifugation to purify FRL-PSI trimers and FRL-PSII dimers further. **C**. Absorption spectra of purified FRL-PSI trimers (red line), FRL-PSII dimers (green line) and WL-PSI trimers (black line) from *F. thermalis*. Photo credit: Dr. Gaozhong Shen, The Pennsylvania State University.**D**. 77-K fluorescence emission spectra for the purified FRL-PSI trimers, FRL-PSII dimers, and WL-PSI trimer complexes. Excitation wavelength was set at 440 nm to predominantly excite chlorophylls. **E**. Reversed-phase HPLC analysis of pigments extracted from FRL-PSI trimers, FRL-PSII dimers, and WL-PSI trimer complexes purified from *F. thermalis* PCC 7521. Reversed-phase HPLC elution profiles monitored at 680 nm of the pigments from trimeric FRL-PSI complexes and dimeric FRL-PSII complexes. The traces were normalized to equal amounts of Chl *a* for this comparison. Note that the Chl *a* absorbance is purposefully shown off-scale to visualize the less abundant compounds better. **F**. Elution profiles from reversed-phase HPLC analysis of a of a pigment extract with a final Chl *a* concentration of 33.3 μg mL^{-1} from isolated FRL-PSI complexes of *Fischerella thermalis* PCC 7521 grown under far-red light conditions. The elution of Chl *a* (green trace) was monitored at 665 nm, the elution of Chl *f* (red trace) was monitored at 707 nm. The calculated ratio of Chl *a* to Chl *f* was 12.7 ± 0.7.

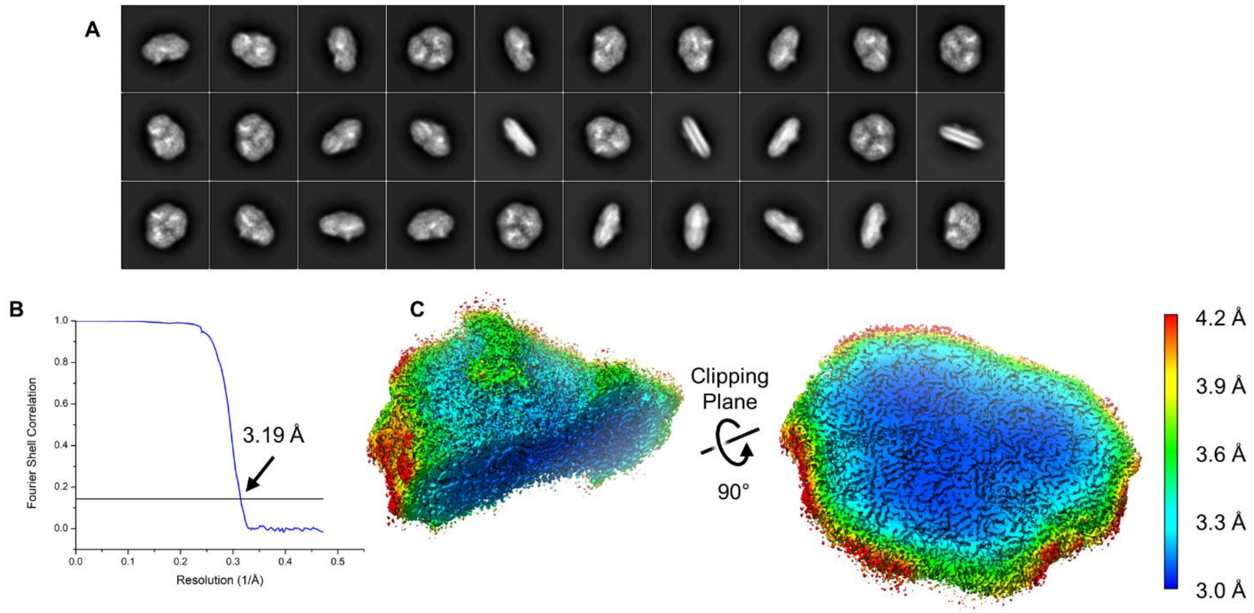

Fig. S2. Resolution of the FRL-PSI density map. A. The 2D classes selected after autopicking (213,528 particles) that comprise 178,666 particles. **B.** FSC curves from postprocessing showing the global resolution to be 3.19 Å using the Gold-standard FSC cutoff (*42*). **C.** The sharpened density map colored by the output the implementation of Local Resolution in RELION 3.0 (*46*). On the left, the clipping plane is aligned perpendicular to the membrane plane. On the right, the clipping plane is rotated 90° so that it is parallel to the membrane plane. A scale bar is shown from 3.0 to 4.2 Å. Note that a disordered detergent micelle composes most of the low-resolution regions.

Helps to coordinate a non-Chl ligand | Helps to coordinate a Chl | Could implicate Chl *f*

Non-terminal residues removed from FRL PSI structure because of poor density | Unmodelled terminal residue(s) | Modelled as poly-Ala helix

PsaA

```
F.t. FRL PsaA2 MTLTP-E-REQEVRVVVDNDPVPTSFQKWSQPGHFDRTLAKGAKTTTWIWNLHANAHDFD 58
F.t. WL PsaA1  MTISPPEREEKKARVIVDNDPVPTSFERWAKPGHFDRTLAKGPKTTTWIWNLHALAHDFD 60
T.e. PsaA      MTISPPEREP-KVRVVVDNDPVPTSFEKWAKPGHFDRTLARGPQTTTWIWNLHALAHDFD 59
               **::* * .  :.**:**********::*::*********:* :********** *****

F.t. FRL PsaA2 THTSDLEDISRKIFAAHFGHLAVVFIWLSGMYFHGARFSNFEAWMANPTGIKPSAQVVWP 118
F.t. WL PsaA1  THTSDLEDISRKIFAAHFGHLAVVTLWLSGMIFHGARFSNYEAWLSDPLNVRPSAQVVWP 120
T.e. PsaA      THTSDLEDISRKIFSAHFGHLAVVFIWLSGMYFHGAKFSNYEAWLADPTGIKPSAQVVWP 119
               **************:********* :***** ****:***:***:::* .::********

F.t. FRL PsaA2 IFGQEILNGDMGGGFHGIQITSGLFQMWRAAGFTNTFQLYCTAIGGLVMAALMLFAGWFH 178
F.t. WL PsaA1  IVGQDILNGDVGGGFHGIQITSGLFQVWRGWGITNSFQLYCTAIGGLVLAGLLLFAGWFH 180
T.e. PsaA      IVGQGILNGDVGGGFHGIQITSGLFQLWRASGITNEFQLYCTAIGGLVMAGLMLFAGWFH 179
               *.** *****:***************:**. *:** ************:*.*:*******

F.t. FRL PsaA2 YHKRAPKLEWFQNTQSMLNHHLAGLLGLGSLGWTGHLIHVSLPTNKLLDTGVALKDIPLP 238
F.t. WL PsaA1  YHKRAPKLEWFQNVESMLNHHLQVLLGCGSLGWAGHIIHVSAPTNKLLDAGVAVKDIPLP 240
T.e. PsaA      YHKRAPKLEWFQNVESMLNHHLAGLLGLGSLAWAGHQIHVSLPINKLLDAGVAAKDIPLP 239
               *************.:*******  *** ***.*:** **** * *****:*** ******

F.t. FRL PsaA2 HEFILNPSLMNKLYPHADWGFVKGVVPFFTLQWGHFTDFLTFKGGLNPVTGGLWLTDVAH 298
F.t. WL PsaA1  HEFILNSANLINLYPS----FAKGLAPFFTLNWGVYSDFLTFKGGLNPVTGGLWMTDIAH 296
T.e. PsaA      HEFILNPSLMAELYPKVDWGFFSGVIPFFTFNWAAYSDFLTFNGGLNPVTGGLWLSDTAH 299
               ****** : : :***     * .*: ****::*. ::*****:***********::* **

F.t. FRL PsaA2 HHLAIAVMFIIAGHMYRTNWGIGHSIKEMLDDARTPNMLPFLSFIGPVGHKGLFEVLTTS 358
F.t. WL PsaA1  HHLAIAVLFIIAGHQYRTNWGIGHSIKEILENHKGPF--------TGDGHKGLYENMTTS 348
T.e. PsaA      HHLAIAVLFIIAGHMYRTNWGIGHSLKEILEAHKGPF--------TGAGHKGLYEVLTTS 351
               *******:****** **********:**:*:  : *            *****:* :***

F.t. FRL PsaA2 WHAQLSINLAMLGSLSIIIAHHMYAMPPYPYLATDYGTVVSLFTHHVWIGGFLIVGGAAH 418
F.t. WL PsaA1  WHAQLGTNLAMLGSLTIIVAHHMYAMPPYPYLATDYATQLCIFTHHMWIGGFLIVGGAAH 408
T.e. PsaA      WHAQLAINLAMMGSLSIIVAQHMYAMPPYPYLATDYPTQLSLFTHHMWIGGFLVVGGAAH 411
               *****. ****:***:**:*:*************** * :.:****:******:******

F.t. FRL PsaA2 AAIYMVRDYDPEQNFNNVLDRVLRHRDAIISHLAWVCQFLGFHSFAMYCHNDTMRAFGRP 478
F.t. WL PsaA1  AAIFMVRDYDPVVNQNNVLDRVIRHRDAIISHLNWVCIFLGFHSFGLYVHNDTMRALGRP 468
T.e. PsaA      GAIFMVRDYDPAMNQNNVLDRVLRHRDAIISHLNWVCIFLGFHSFGLYVHNDTMRAFGRP 471
               .**:*******  * *******:********** *** *******.:* *******:***

F.t. FRL PsaA2 QDMFSDTGIQLQPVFAQWLQHIHTMTIGNPSLQVAAPLGHAFGGLRNLELTGLGTAAPNL 538
F.t. WL PsaA1  QDMFSDTAIQLQPVFAQWVQNLHTLAPG--------------------------ATAPNA 502
T.e. PsaA      QDMFSDTGIQLQPVFAQWVQNLHTLAPG--------------------------GTAPNA 505
               *******.**********:*::**:: *                           :***

F.t. FRL PsaA2 HDPVSYAFGGGVVAVGGKVAMMPITLGTADFLIHHIHAFTIHVTVLVLLKGVLFARSSRL 598
F.t. WL PsaA1  LEPVSYAFGGGILAVGGKVAMMPIALGTADFMIHHIHAFQIHVTTLILLKGFLFARSSRL 562
T.e. PsaA      AATASVAFGGDVVAVGGKVAMMPIVLGTADFMVHHIHAFTIHVTVLILLKGVLFARSSRL 565
                  .* ****.::***********.******::****** ****.*:****.********

F.t. FRL PsaA2 IPDKANLGFRFPCDGPGRGGTCQVSAWDHVFLGLFWMYNSLSMVIFHFFWKMQSDVWGTV 658
F.t. WL PsaA1  IPDKANLGFRFPCDGPGRGGTCQVSGWDHVFLGLFWMYNTISIAIFHFSWKMQSDVWGTV 622
T.e. PsaA      IPDKANLGFRFPCDGPGRGGTCQVSGWDHVFLGLFWMYNCISVVIFHFSWKMQSDVWGTV 625
               *************************.************* :*:.**** ***********

F.t. FRL PsaA2 GADGVVTHITGGNFATSSITNNGWLRDFLWAQSTQVITSYNTSLSAYGLMFLGGHFIFGF 718
F.t. WL PsaA1  DAAGNVSHVTGGNFAQSAITINGWLRDFLWAQATQVINSYGSALSAYGLMFLGAHFIWAF 682
T.e. PsaA      APDGTVSHITGGNFAQSAITINGWLRDFLWAQASQVIGSYGSALSAYGLLFLGAHFIWAF 685
                  * *:*:****** *:** ***********::*** **.::******:***.***:.*

F.t. FRL PsaA2 SLMFLFSGRGYWQELIESIVWAHNKLKVAPAIQPRALSIIHGRAVGVAHYLLGGIVTTWA 778
```

```
F.t. WL PsaA1  SLMFLFSGRGYWQELIESIVWAHNKLKVAPAIQPRALSIIQGRAVGVAHYLLGGIATTWA  742
T.e. PsaA      SLMFLFSGRGYWQELIESIVWAHNKLKVAPAIQPRALSIIQGRAVGVAHYLLGGIATTWA  745
               ****************************************:**************.****

F.t. FRL PsaA2 FFLARMTAFG 788
F.t. WL PsaA1  FFHAHILSIG 752
T.e. PsaA      FFLARIISVG 755
               ** *:: :.*
```

PsaB

```
F.t. FRL PsaB2 MATKFPKFSQDLANDPTTRRIFYAIATAHDFESHDGMTEENLYQRIFASHFGHLAIIFLW  60
F.t. WL PsaB1  MATKFPKFSQDLAQDPTTRRIWYAMAMGNDFESHDGMTEENLYQKIFATHFGHLAIIFLW  60
T.e. PsaB      MATKFPKFSQDLAQDPTTRRIWYAIAMAHDFESHDGMTEENLYQKIFASHFGHLAIIFLW  60
               *************:*******:**:* .:***************:***:***********

F.t. FRL PsaB2 ASGILFHVAWQGNFEVWIKDPVHVRPIAHAIWDAQFGPGAIKAFTQAGARNPVDICYSGV  120
F.t. WL PsaB1  ASSLLFHVAWQGNFEQWIKDPLHIRPIAHAIWDPHFGKPAIEAFTQGGASNPVNIAYSGV  120
T.e. PsaB      VSGSLFHVAWQGNFEQWVQDPVNTRPIAHAIWDPQFGKAAVDAFTQAGASNPVDIAYSGV  120
               .*. *********** *::**:: ********* :**  *:.****.** ***:*.****

F.t. FRL PsaB2 YHWWYTIGLRTNTELYVGALFLILLAAVFLFAGWLHLQPRYRPNLGWFKNSEARLNHHLA  180
F.t. WL PsaB1  YHWWYTIGMRTNGDLYMGSIFLLVLSSLFLFAGWLHLQPKFRPSLAWFKMAESRLNHHLA  180
T.e. PsaB      YHWWYTIGMRTNGDLYQGAIFLLILASLALFAGWLHLQPKFRPSLSWFKNAESRLNHHLA  180
               ********:*** :** *::**::*:*:: **********::**.*.*** :*:*******

F.t. FRL PsaB2 GLFGVSSLAWAGHLVHVAIPESRGQHVGWDNFLSTPPHPAGLWAFFTGNWGAYAQNPDTA  240
F.t. WL PsaB1  GLFGVSSLAWTGHLVHVAIPESRGQHVGWDNFLSTLPHPAGLQPFFTGNWGVYAENPDTA  240
T.e. PsaB      GLFGVSSLAWAGHLIHVAIPESRGQHVGWDNFLSTMPHPAGLAPFFTGNWGVYAQNPDTA  240
               **********:***:******************** ******  *******.**:*****

F.t. FRL PsaB2 EHVFSTSQGAGTAILTFLGGFHPQTQSLWLTDMAHHHLAIAVVLIIAGHMYRTNWRIGHS  300
F.t. WL PsaB1  SHVFGTSQGAGTAILTFLGGFHPQTESLWLTDMAHHHLAIAVLFIVAGHMYRTNFGIGHS  300
T.e. PsaB      SHVFGTAQGAGTAILTFLGGFHPQTESLWLTDMAHHHLAIAVLFIVAGHMYRTQFGIGHS  300
               .***.*:******************:****************::*:********:: ****

F.t. FRL PsaB2 IKEMMDSKTFFGRKVEGPFNLPHQGLYETVNNSLHFQLSLALACLGVASSLTAQHMYSMP  360
F.t. WL PsaB1  IKEMMDAKTFFGKPVEGPFNMPHQGIYETYNNSLHFQLGWHLACLGVITSLVAQHMYSLP  360
T.e. PsaB      IKEMMDAKDFFGTKVEGPFNMPHQGIYETYNNSLHFQLGWHLACLGVITSLVAQHMYSLP  360
               ******:* ***  ******:****:*** ********.  ****** :**.******:*

F.t. FRL PsaB2 PYAFIAKDFTTMAALYTHHQYIAGFLMVGAFSHAAIFWIKDYDPEQNKGNVLERVLKHKE  420
F.t. WL PsaB1  PYAFIAKDYTTQAALYTHHQYIAIFLMLGAFAHGAIFWVRDYDPEQNKGNVLERVLKHKE  420
T.e. PsaB      PYAFIAQDHTTMAALYTHHQYIAGFLMVGAFAHGAIFLVRDYDPAQNKGNVLDRVLQHKE  420
               ******:*.** *********** ***:***:*.*** ::**** *******:***:***

F.t. FRL PsaB2 AIIAHLSWVSLFLGFHTLGLYVHNDVEVAFGAADKQILIEPVFAQFIQSANGKILYGFHT  480
F.t. WL PsaB1  AIISHLSWVSLFLGFHTLGLYVHNDVVVAFGTPEKQILIEPVFAQFIQASHGKVLYGLNV  480
T.e. PsaB      AIISHLSWVSLFLGFHTLGLYVHNDVVVAFGTPEKQILIEPVFAQFIQAAHGKLLYGFDT  480
               ***:********************** ****: :**************:::**:***:..

F.t. FRL PsaB2 LLSNPDSIAFTAWPNHANVWLPGWLDAINNGTNSLFLTIGPGDFYVHHAIALGLHVTTLI  540
F.t. WL PsaB1  LLSNPDSVAYTAYPNYGNVWLSGWLDAINSGTNSLFLTIGPGDFLVHHAFALAIHTTTLV  540
T.e. PsaB      LLSNPDSIASTAWPNYGNVWLPGWLDAINSGTNSLFLTIGPGDFLVHHAIALGLHTTTLI  540
               *******:* **:**:.****.*******.************** ****:**.:*.***:

F.t. FRL PsaB2 LVKGALDARGSKLMPDKKDFGYAFPCDGPGRGGTCDISAWDASYLAVFWMLNTLGWVTFY  600
F.t. WL PsaB1  LVKGALDARGSKLMPDKKDFGYAFPCDGPGRGGTCDISAWDAFYLATFWALNTVGWVTFY  600
T.e. PsaB      LVKGALDARGSKLMPDKKDFGYAFPCDGPGRGGTCDISAWDAFYLAMFWMLNTIGWVTFY  600
               ****************************************** *** ** ***:******

F.t. FRL PsaB2 WHWKHLSIWQGNVAQFNESSTYLMGWFRDYLWANSAQLINGYNPYGTNNLAVWAWMFLFG  660
F.t. WL PsaB1  WHWKHLGIWQGNVAQFNESSTYLMGWFRDYLWANSAQLINGYNPYGMNNLSVWAWMFLFG  660
T.e. PsaB      WHWKHLGVWEGNVAQFNESSTYLMGWLRDYLWLNSSQLINGYNPFGTNNLSVWAWMFLFG  660
               ******.:*:****************:***** **:********:* ***:*********

F.t. FRL PsaB2 HLAWAVSFMFLITWRGYWQELIETLAWAHEQTPLS-FGYWRDKPVALSIVQARLVGLTHF  719
F.t. WL PsaB1  HLVWATGFMFLISWRGYWQELIETLVWAHERTPIANLVRWKDKPVALSIVQARLVGLVHF  720
T.e. PsaB      HLVWATGFMFLISWRGYWQELIETLVWAHERTPLANLVRWKDKPVALSIVQARLVGLAHF  720
               **.**..*****:************.****:**:: :  *:****************.**
```

```
F.t. FRL PsaB2 TVGYIATYGAFLIASTASKFGQ 741
F.t. WL PsaB1  SVGYVLTYAAFLIASTAGKFG- 741
T.e. PsaB      SVGYILTYAAFLIASTAAKFG- 741
               :***: **.********.***
```

PsaC

```
F.t. PsaC      MSHTVKIYDTCIGCTQCVRACPTDVLEMVPWDGCRAGQIASSPRTEDCVGCKRCETACPT  60
T.e. PsaC      MAHTVKIYDTCIGCTQCVRACPTDVLEMVPWDGCKAGQIASSPRTEDCVGCKRCETACPT  60
               *:********************************:*************************

F.t. PsaC      DFLSIRVYLGAETTRSMGLAY 81
T.e. PsaC      DFLSIRVYLGAETTRSMGLAY 81
               *********************
```

PsaD

```
F.t. PsaD      MSPSAVLGCRKNVERRFMAETLSGQTPIFGGSTGGLLKKAEVEEKYAITWTSPKEQVFEM  60
T.e. PsaD      ------------------MTTLTGQPPLYGGSTGGLLSAADTEEKYAITWTSPKEQVFEM  42
                                   **:** *::********. *:.******************

F.t. PsaD      PTGGAAIMRQGQNLLYLARKEQCIALGG-QLRKFKITDYKIYRIYPNGETVYIHPADGVF  119
T.e. PsaD      PTAGAAVMREGENLVYFARKEQCLALAAQQLRPRKINDYKIYRIFPDGETVLIHPKDGVF  102
               **.***:**:*:**:*:******:**.. ***  **.*******:*:**** *** ****

F.t. PsaD      PEKVNQGREKVRYNDRRIGQNPSPSKVKFSGIATYDAPNS 159
T.e. PsaD      PEKVNKGREAVNSVPRSIGQNPNPSQLKFTGKKPYDP--- 139
               *****:*** *.   * *****.**::**:*   **
```

PsaE

```
F.t. PsaE      MVQRGSKVRILRPESYWFQDVGTVASIEQGGTSRYPVIVRFDKVNYAG-------VNTNN  53
T.e. PsaE      MVQRGSKVKILRPESYWYNEVGTVASVDQTPGVKYPVIVRFDKVNYTGYSGSASGVNTNN  60
               ********:********:::******::*    :************:*       *****

F.t. PsaE      FAQDELVEVEAPKAKAKKA 72
T.e. PsaE      FALHEVQEVAPPKKGK--- 76
               ** .*: **  **
```

PsaF

```
F.t. FRL PsaF2 MKRIFALILAIFIWFSAVSTALAENTTLVPCYKSPAFVERMKNAP----DSYYTTKPLKA  56
F.t. WL PsaF1  MRRLFALILAICLWFNFAPAANALGADLVPCSESSAFAQRAQVARNTTADPQSGQKRFER  60
T.e. PsaF      MRRFLALLLVLTLWLGFTPLASADVAGLVPCKDSPAFQKRAAAAVNTTADPASGQKRFER  60
               *:*::**:*.: :*:. .  * *  : **** .* ** :*   *     *     * ::

F.t. FRL PsaF2 YSQLLCGEDGLPRIALD-RLSLAVDVAIPIAIFLYTAGFIGWSGRSYLQAIKKQ-DKAEE 114
F.t. WL PsaF1  YSQAYCGPEGLPHLIVDGRLDRAGDFLIPSILFLYIAGWIGWVGRAYLQTIKKQGGDVEQ 120
T.e. PsaF      YSQALCGEDGLPHLVVDGRLSRAGDFLIPSVLFLYIAGWIGWVGRAYLIAVRNS-GEANE 119
               ***  ** :***:: :* **. * *. **  :*** **:*** **:** ::::. ...::

F.t. FRL PsaF2 KEVFIDVPLFISCMVMALFWPMAVIKELLAGELVAKDEEIPISVR 159
F.t. WL PsaF1  KEIQIDVPLALPIMLSGFAWPAAAIKELLSGELTAKDEEIPISPR 165
T.e. PsaF      KEIIIDVPLAIKCMLTGFAWPLAALKELASGELTAKDNEITVSPR 164
               **: ***** :  *: .: ** *.:*** :***.***:** :* *
```

PsaI

```
F.t. FRL PsaI2 MMVDMTQLTGDYAASWLPWIMIPLVFYILPFPVFAILFLWIQKEASEEIKETDNNLAEIG  60
F.t. WL PsaI1  ----MASLLASYPASFLSPILVYSIGWIVPIVVFSFMLIYIEREDIA-------------  43
T.e. PsaI      -------MMGSYAASFLPWIFIPVVCWLMPTVVMGLLFLYIEGEA---------------  38
                      : ..* **:*  *::  : :::*  *:.:::::*: *

F.t. FRL PsaI2 ELEVPNS   67
F.t. WL PsaI1  -------   43
T.e. PsaI      -------   38
```

PsaJ

```
F.t. FRL PsaJ2 -MEARYLFRYLSSAPVVATLALIIISVILIVLNYLFPGLQYGTFFHSLP  48
F.t. WL PsaJ1  MDNQSPFFKFLSTAPVITTIWLFITAGILIEFNRFFPDLLF----HPLP  45
T.e. PsaJ      ---MKHFLTYLSTAPVLAAIWMTITAGILIEFNRFYPDLLF----HPL-  41
                     :: :**:***:::: : * : *** :* ::*.* :    * *
```

PsaK[#]

```
F.t. PsaK1     VISSILFAVQSTVPNTYTSWGLSEFLYITGACLLCLLIIPRVVWYPHVGPKMPLPFPSVF 60
F.t. PsaK2     -MSSILLAAAATVPPT-AEWSPKVALIISISCLVAVLLSFR-IEKPKVGPKMPGL----- 52
F.t. PsaK3     LLTSTLLA--A--ATTPLQWSPTVGVTMILCNILAIFFGKFTIKYPNAEPALPS--NQFF 54
T.e. PsaK      -----MVL--A--TLPDTTWTPSVGLVVILCNLFAIALGRYAIQSRGKGPGLPIALPALF 51
                    :.   :        *  .  : :  . :..: :    :      * :*

F.t. PsaK1     NNPSVATFLAAMAAGHILGVGAVLGLTNLGIL 92
F.t. PsaK2     -PLSIPTFVAAMAFGHVIGVAIVLGLTNIGKI 83
F.t. PsaK3     GGFGVPALLATTAFGHILGAGAILGLHNLGRF 86
T.e. PsaK      EGFGLPELLATTSFGHLLAAGVVSGLQYAGAL 83
                  .:  ::*: : **::... : **   * :
```

PsaL

```
F.t. FRL PsaL2 --------MSNTVDTVDNDIIKPFKGDPCLGNLSTPINDSPLAKAFINNLPAYRKGLTPF 52
F.t. WL PsaL1  MAQAVDASKNLPSDPRNREVVFPATRDPQIGNLETPINSSALTKWFINNLPAYRPGITPF 60
T.e.  PsaL     ---------------MAEELVKPYNGDPFVGHLSTPISDSGLVKTFIGNLPAYRQGLSPI 45
                                .::: * . ** :*:*.***..* *.* **.****** *::*:

F.t. FRL PsaL2 MRGLEIGMAHGYFLVGPEVVIGPLRESAHGANLSGLITAIYIAVSACLGISIFAITTFQG 112
F.t. WL PsaL1  RRGLEVGMAHGYWIFGPFAKLGPLRNTVN-ADLAGLLSTIGLLVILTIALSLYANSN--- 116
T.e. PsaL      LRGLEVGMAHGYFLIGPWVKLGPLRDSDV-ANLGGLISGIALILVATACLAAYGLVSFQK 104
                ****:******::.** . :****::   *:*.**:: * : :     :: :.  .

F.t. FRL PsaL2 NPKGSYSSYSKDSLRPLRTREEWSQLNGGIFLGAMGGAIFAYLLLENFDALDAILRGAVN 172
F.t. WL PsaL1  PPEPVASVTAPHPSDAFHTKEGWSNFGSAFLIGGIGGAVTAYFLTANFGLIQGFFG---- 172
T.e. PsaL      G---------GSSSDPLKTSEGWSQFTAGFFVGAMGSAFVAFFLLENFSVVDGIMTGLFN 155
                               ::* * **:: ..:::*.:*.*. *::*  **. ::.::

F.t. FRL PsaL2 AS 174
F.t. WL PsaL1  -- 172
T.e. PsaL      -- 155
```

PsaM

```
F.t. PsaM      MSISDTQVYIALVVALVPGFLAWRLATELYK 31
T.e. PsaM      MALTDTQVYVALVIALLPAVLAFRLSTELYK 31
               *:::*****:***:**:*..**:**:*****
```

PsaX

```
F.t. PsaX      MAKADTTADLPVAKSTTAKPPYTFRTAWALLLLAINFIVAAYYFHIIE 48
T.e. PsaX      ---------MSTMATKSAKPTYAFRTFWAVLLLAINFLVAAYYFGILK 39
                        : .  :.:*** *:*** **:*******:****** *::
```

Fig. S3. Sequence alignments of PSI subunit polypeptides. Polypeptides for FRL-PSI from *F. thermalis* ("*F.t.* FRL") that are only produced in FRL (PsaA2, PsaB2, PsaF2, PsaI2, PsaJ2, PsaL2), the polypeptides of PSI produced in WL-adapted *F. thermalis* ("*F.t.* WL") (PsaA1, PsaB1, PsaF1, PsaI1, PsaJ1, and PsaL1), subunits produced from genes whose expression is not modulated by light conditions (PsaC, PsaD, PsaE, PsaK, PsaM and PsaX), and PSI subunit sequences from *T. elongatus* PSI ("*T.e.*"). The alignment was performed using the Clustal Omega server (*55*). As shown in the legend, residues that ligate any Chl molecule are highlighted in green, residues that interact with a non-Chl ligand are highlighted in blue, non-terminal residues that could not be modeled into the FRL-PSI structure are highlighted in red, residues that may be implicated in the coordination of Chl *f* are boxed, and terminal residues that are not present in the model are highlighted in grey.

[#]Note that only part of PsaK could be modeled into the FRL-PSI density map. Additionally, the structure of PSI from *T. elongatus* does not have sidechains modeled into the structure.

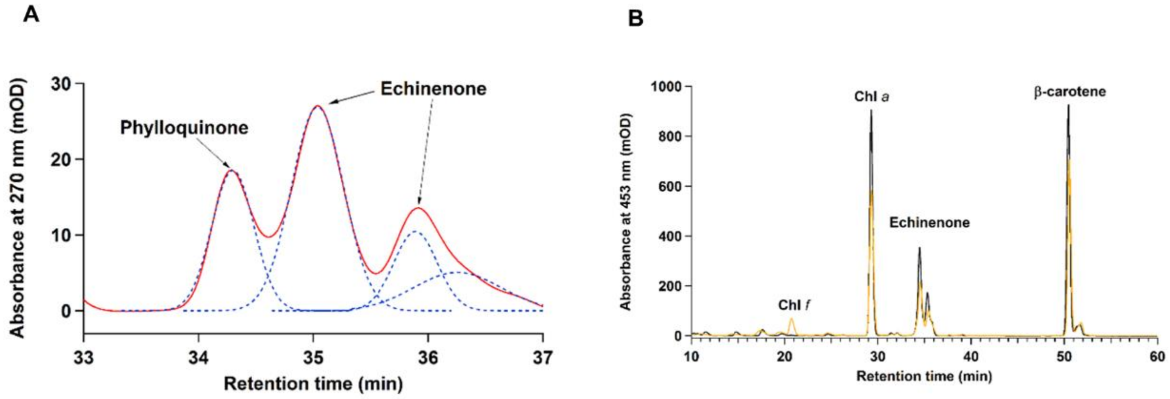

Fig. S4. Reversed-phase HPLC elution profiles identifying various pigments. A. Elution profile from reversed-phase HPLC analysis of pigment extract with a final Chl *a* concentration of 100 μg mL^{-1} from isolated FRL-PSI of *Fischerella thermalis* PCC 7521 grown under FRL conditions (red trace) and its deconvolution. By comparison to a standard curve, the area under the phylloquinone peak allows one to determine the concentration of phylloquinone in the sample. Because there are two phylloquinones per PSI monomer, the molar concentration of the PSI in the sample can be calculated. Echinenone elutes as two peaks, which were interpreted to be all-*trans*-echinenone (35 min) and a mixture of two or three *cis*-isomers of echinone (36 to 37 min). **B**. Elution profiles monitored at 453 nm from reversed-phase HPLC analysis of pigment extracts final Chl *a* concentration of 100 μg mL^{-1} from isolated PSI complexes of *Fischerella thermalis* PCC 7521 grown under WL conditions (black trace) and under FRL conditions (orange trace). The FRL-PSI complexes contained 15.8 ± 0.6 carotenoid molecules per monomer, and the WL-PSI complexes contained 19.1 ± 1.1 carotenoid molecules per monomer. FRL-PSI complexes contained 10.2 ± 0.9 total β-carotene molecules (8.9 ± 0.9 all-*trans* and 1.3 ± 0.1 *cis*) and 5.6 ± 0.9 total echinenone molecules (3.5 ± 0.9 all-*trans* and 2.1 ± 0.3 *cis*). WL-PSI complexes contained 11.3 ± 0.7 total β-carotene molecules (10.1 ± 0.6 all-*trans* and 1.1 ± 0.2 *cis*) and 7.8 ± 0.7 total echinenone molecules (4.7 ± 0.4 all-*trans* and 3.0 ± 0.8 *cis*). The ratio of β-carotene to echinenone was higher in the FRL-PSI (1.82) than in WL-PSI (1.44) complexes.

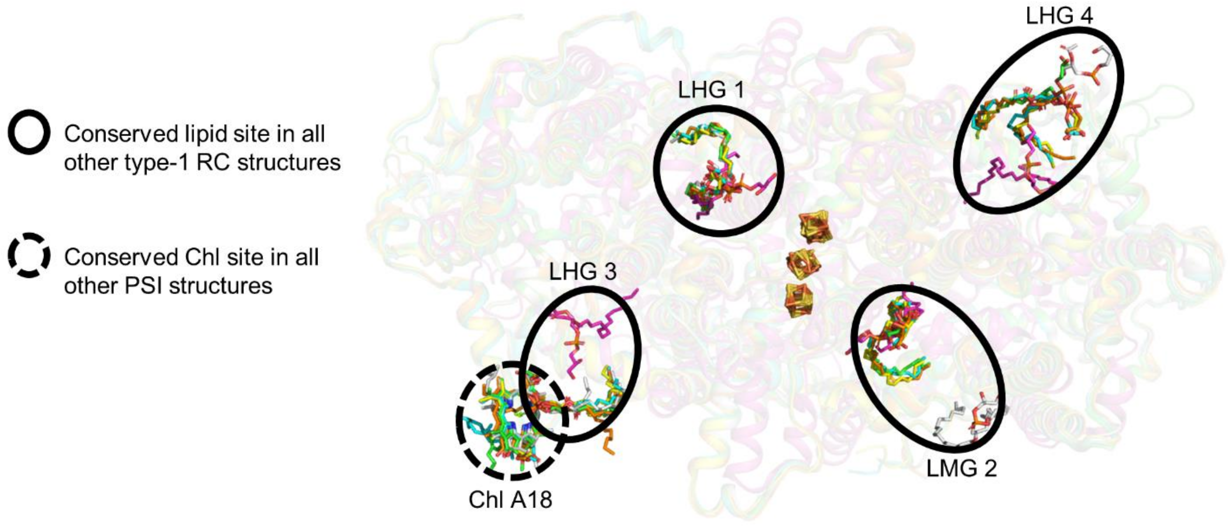

Fig. S5. LHG 3 and Chl A18 site conservation in all type 1 reaction center structures excluding the FRL-PSI structure presented here. The core subunits from all six type-1 photosystem structures (five unique PSI structures and one homodimeric photosystem structure from heliobacteria, PDB ID=5V8K (*18*)) were superimposed, colored individually, and the 4 conserved lipid sites are shown (solid circles). Additionally, the Chl A18 site is shown which is present in all five PSI structures (*T. elongatus* (*11*,*12*), PDB ID=1JB0, *Synechocystis* sp. PCC 6803 trimer (*15*), PDB ID=5OY0, *Synechocystis* sp. PCC 6803 monomer (*16*), PDB ID=6HQB, *Pisum sativum* (*56*), PDB ID=5L8R). [4Fe-4S] clusters and transparent protein cartoons are shown for orientation.

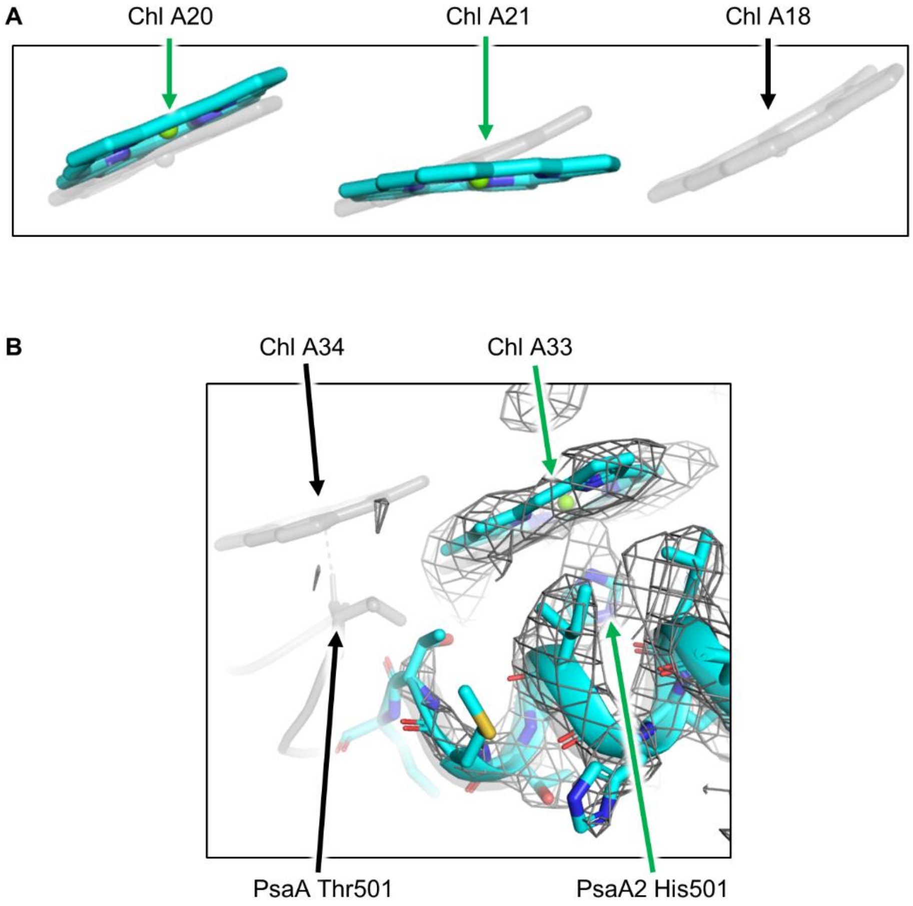

Fig. S6. Details of A20/A21 and A33 Chls. A. Orientations of Chl planes in the FRL-PSI A20/A21 dimer. For clarity, only the Chl rings are shown. FRL-PSI pigments are colored and the superimposed *T. elongatus* pigments are shown in transparent grey. Note that whereas in *T. elongatus* PSI, the three pigments of the trimer have the planes of their Chls approximately parallel to one-another. In the dimer in FRL-PSI the Chl planes are not oriented parallel to one another. Green arrows denote Chl positions that are conserved between the two structures and the black arrow denotes a non-conserved position. **B**. Density map of Chl A33 region plotted at 6*σ. Elements of FRL-PSI are shown in colors and elements of *T. elongatus* PSI are shown in transparent grey. Chl substituents and tails have been omitted for clarity.

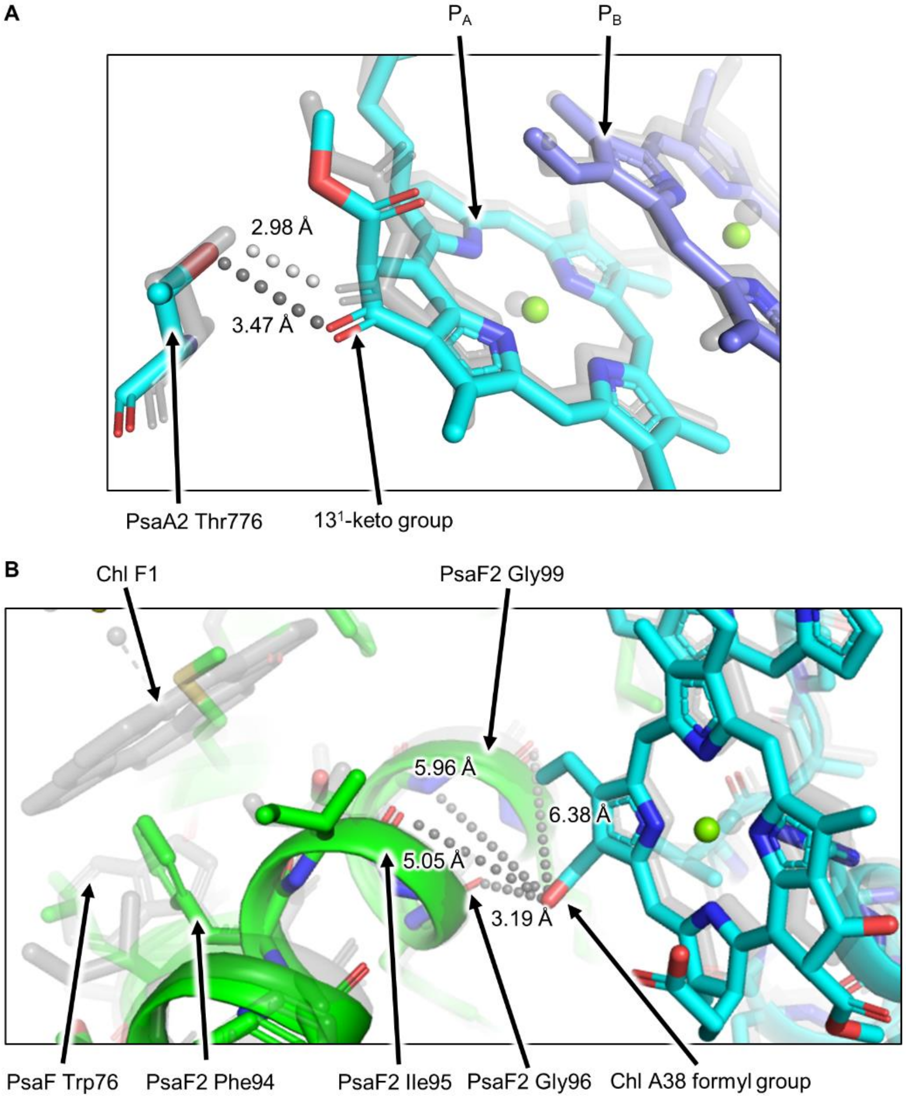

Fig. S7. P_A H bonding and possible Chl f A38. A. Weakened H-bond between 13^1-keto-oxygen of P_A and the hydroxyl group of Thr776. P_A and P_B of P_{700} and Thr776 are shown with the superposition of the corresponding elements from *T. elongatus* PSI in transparent grey. **B**. Possible H-bonding interactions near Chl A38 if it is Chl *f*. Various measurements near atoms that could be involved in a H-bonding network near the formyl substituent of Chl A38 if it is a Chl *f* are shown. On the other side of this PsaF TMH, the corresponding residue to PsaF Trp76 in *T. elongatus* PSI is PsaF2 Phe94 which is not sterically hindered by the presence of Chl *f* in FRL-PSI.

Table S1. Cryo-EM data collection, refinement, and validation statistics for FRL-PSI. (PDB ID = 6PNJ, EMDB ID = EMD-20397).

**Data collection and processing**	
Magnification	x47,600
Voltage (kV)	300
Electron exposure	57.3
Defocus range (μm)	-1 to -3
Pixel size (Å)	0.53
Symmetry imposed	C3
Initial particle images (no.)	213,528
Final particle images (no.)	178,666
Map resolution (Å)	3.19
FSC threshold	0.143
Map resolution range (Å)	3.0-5
**Refinement**	
Initial model used (PDB code)	1JB0
Model resolution (Å)	3.19
FSC threshold	0.143
Map resolution range (Å)	3.0-5
Map-sharpening *B* factor ($Å^2$)	-100
Model composition	
Non-hydrogen atoms	71,520
Protein residues	6,708
Ligands	372
B factors ($Å^2$)	
Protein	59
Ligands	88
R.m.s. deviations	
Bond lengths (Å)	0.010
Bond angles (°)	1.920
**Validation**	
MolProbity	1.76
Clashscore	6.27
Rotamer outliers (%)	0.94
Ramachandran plot	
Favored (%)	94
Allowed (%)	6
Disallowed (%)	0

Table S2. Sequence identity matrices of homologous PSI subunit polypeptides from *F. thermalis* and *T. elongatus*.

	F.t. FRL PsaA2	*F.t.* WL PsaA1	*T.e.* PsaA
F.t. FRL PsaA2	100.00	80.40	83.00
F.t. WL PsaA1	80.40	100.00	86.82
T.e. PsaA	83.00	86.82	100.00

	F.t. FRL PsaA2	*F.t.* WL PsaB1	*T.e.* PsaB
F.t. FRL PsaA2	100.00	83.11	84.59
F.t. WL PsaB1	83.11	100.00	90.42
T.e. PsaB	84.59	90.42	100.00

	F.t. PsaC	*T.e.* PsaC
F.t. PsaC	100.00	97.53
T.e. PsaC	97.53	100.00

	F.t. PsaD	*T.e.* PsaD
F.t. PsaD	100.00	70.29
T.e. PsaD	70.29	100.00

	F.t. PsaE	*T.e.* PsaE
F.t. PsaE	100.00	69.57
T.e. PsaE	69.57	100.00

	F.t. FRL PsaF2	*F.t.* WL PsaF1	*T.e.* PsaF
F.t. FRL PsaF2	100.00	54.09	50.31
F.t. WL PsaF1	54.09	100.00	69.51
T.e. PsaF	50.31	69.51	100.00

	F.t. FRL PsaI2	*F.t.* WL PsaI1	*T.e.* PsaI
F.t. FRL PsaI2	100.00	30.23	39.47
F.t. WL PsaI1	30.23	100.00	52.63
T.e. PsaI	39.47	52.63	100.00

	F.t. FRL PsaJ2	*F.t.* WL PsaJ1	*T.e.* PsaJ
F.t. FRL PsaJ2	100.00	43.18	39.02
F.t. WL PsaJ1	43.18	100.00	70.73
T.e. PsaJ	39.02	70.73	100.00

	F.t. PsaK1	*F.t.* PsaK2	*F.t.* PsaK3	*T.e.* PsaK
F.t. PsaK1	100.00	49.40	32.56	25.30
F.t. PsaK2	49.40	100.00	34.62	28.00
F.t. PsaK3	32.56	34.62	100.00	43.21
T.e. PsaK	25.30	28.00	43.21	100.00

	F.t. FRL PsaL	*F.t.* WL PsaL	*T.e.* PsaL
F.t. FRL PsaL	100.00	40.24	47.30
F.t. WL PsaL	40.24	100.00	52.26
T.e. PsaL	47.30	52.26	100.00

	F.t. PsaM	*T.e.* PsaM
F.t. PsaM	100.00	67.74
T.e. PsaM	67.74	100.00

	F.t. PsaX	*T.e.* PsaX
F.t. PsaX	100.00	58.97
T.e. PsaX	58.97	100.00

Table S3. Superposition RMSD comparing *T. elongatus* PSI (PDB ID, 1JB0) subunits to *F. thermalis* FRL-PSI subunits (PDB ID, 6PNJ).

**Chain[1]**	**RMSD (Å)**
A	0.503
B	0.509
C	0.449
D	0.566
E	0.626
F	0.791
I	0.925
J	0.878
K	ND[2]
L	0.875
M	0.379
X	0.651

[1]Chains corresponding to a comparison of a *T. elongatus* PSI subunit to a *F. thermalis* FRL-PSI subunit that is only expressed in cells grown in far-red light are highlighted in pink.

[2]ND, not determinable. The K chain could only partially be modeled into the FRL-PSI structure and was therefore excluded from this superposition comparison.

Data S1. (JPred4 output).

Data S2. (caption, data separate). Final PDB verification report for 6PNJ.
