# Supplementary material for: The structure of Photosystem I acclimated to far-red light illuminates an ecologically important acclimation process in photosynthesis
Source: Sci Adv. 2020 Feb 5;6(6):eaay6415. doi: 10.1126/sciadv.aay6415 (PMC7002129; doi:10.1126/sciadv.aay6415)
Supplement: Data S2 [file aay6415_Data_S2.pdf]

Full wwPDB/EMDataBank EM Map/Model Validation Report (i)

Jul 2, 2019 – 05:46 PM EDT

Deposition ID : D_1000241325
PDB ID : *(not yet assigned)*

This is a Full wwPDB/EMDataBank EM Map/Model Validation Report.

This report is produced by the wwPDB Deposition System during initial deposition but before annotation of the structure.

We welcome your comments at *validation@mail.wwpdb.org*
A user guide is available at
https://www.wwpdb.org/validation/2017/EMValidationReportHelp
with specific help available everywhere you see the (i) symbol.

MolProbity	:	4.02b-467
Mogul	:	1.8.0 (224370), CSD as540be (2019)
Percentile statistics	:	20171227.v01 (using entries in the PDB archive December 27th 2017)
Ideal geometry (proteins)	:	Engh & Huber (2001)
Ideal geometry (DNA, RNA)	:	Parkinson et. al. (1996)
Validation Pipeline (wwPDB-VP)	:	2.4

PRELIMINARY VALIDATION REPORT

1 Overall quality at a glance (i)

The following experimental techniques were used to determine the structure:
ELECTRON MICROSCOPY

The reported resolution of this entry is 3.20 Å.

Percentile scores (ranging between 0-100) for global validation metrics of the entry are shown in the following graphic. The table shows the number of entries on which the scores are based.

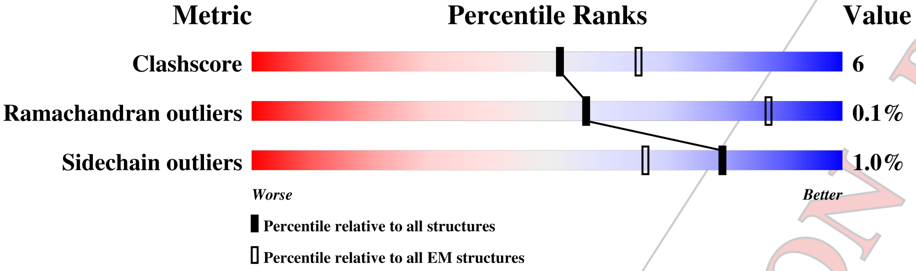

Metric	Whole archive (#Entries)	EM structures (#Entries)
Clashscore	136327	1886
Ramachandran outliers	132723	1663
Sidechain outliers	132532	1531

The table below summarises the geometric issues observed across the polymeric chains. The red, orange, yellow and green segments on the bar indicate the fraction of residues that contain outliers for >=3, 2, 1 and 0 types of geometric quality criteria. A grey segment represents the fraction of residues that are not modelled. The numeric value for each fraction is indicated below the corresponding segment, with a dot representing fractions <=5%

Mol	Chain	Length	Quality of chain
1	A	788	78% 17% 5%
1	G	788	78% 16% 5%
1	a	788	94% • 5%
2	B	741	81% 18%
2	H	741	82% 17%
2	b	741	99% •
3	C	81	77% 21% ••
3	N	81	74% 23% ••
3	c	81	98% ••

Continued on next page...

PRELIMINARY VALIDATION REPORT

Continued from previous page...

Mol	Chain	Length	Quality of chain
4	D	159	72% 16% 13%
4	O	159	70% 17% 13%
4	d	159	86% • 13%
5	E	72	76% 10% 14%
5	P	72	75% 11% 14%
5	e	72	86% 14%
6	F	159	71% 11% • 15%
6	Q	159	67% 16% • 15%
6	f	159	81% • • 15%
7	I	67	48% 15% 37%
7	R	67	51% 12% 37%
7	i	67	63% 37%
8	J	48	65% 23% • 10%
8	S	48	67% 21% • 10%
8	j	48	83% 6% 10%
9	K	82	37% 5% 59%
9	T	82	39% • 59%
9	k	82	41% 59%
10	L	174	74% 16% 10%
10	U	174	75% 14% 10%
10	l	174	88% • 10%
11	M	31	81% 19%
11	V	31	84% 16%
11	m	31	100%
12	W	101	28% • 71%

Continued on next page...

PRELIMINARY VALIDATION REPORT

Continued from previous page...

Mol	Chain	Length	Quality of chain
12	X	101	28% • 71%
12	x	101	29% 71%

The following table lists non-polymeric compounds, carbohydrate monomers and non-standard residues in protein, DNA, RNA chains that are outliers for geometric or electron-density-fit criteria:

Mol	Type	Chain	Res	Chirality	Geometry	Clashes	Electron density
13	CL0	AA	1011	X	-	-	-
13	CL0	GA	1011	X	-	-	-
13	CL0	aA	1011	X	-	-	-
14	CLA	0	1012	X	-	-	-
14	CLA	AA	1012	X	-	-	-
14	CLA	AA	1013	X	-	-	-
14	CLA	AA	1101	X	-	-	-
14	CLA	AA	1102	X	-	-	-
14	CLA	AA	1103	X	-	-	-
14	CLA	AA	1104	X	-	-	-
14	CLA	AA	1105	X	-	-	-
14	CLA	AA	1106	X	-	-	-
14	CLA	AA	1107	X	-	-	-
14	CLA	AA	1108	X	-	-	-
14	CLA	AA	1109	X	-	-	-
14	CLA	AA	1110	X	-	-	-
14	CLA	AA	1111	X	-	-	-
14	CLA	AA	1112	X	-	-	-
14	CLA	AA	1113	X	-	-	-
14	CLA	AA	1114	X	-	-	-
14	CLA	AA	1115	X	-	-	-
14	CLA	AA	1116	X	-	-	-
14	CLA	AA	1117	X	-	-	-
14	CLA	AA	1118	X	-	-	-
14	CLA	AA	1119	X	-	-	-
14	CLA	AA	1122	X	-	-	-
14	CLA	AA	1123	X	-	-	-
14	CLA	AA	1124	X	-	-	-
14	CLA	AA	1125	X	-	-	-
14	CLA	AA	1126	X	-	-	-
14	CLA	AA	1127	X	-	-	-
14	CLA	AA	1128	X	-	-	-
14	CLA	AA	1129	X	-	-	-

Continued on next page...

PRELIMINARY VALIDATION REPORT

Continued from previous page...

Mol	Type	Chain	Res	Chirality	Geometry	Clashes	Electron density
14	CLA	AA	1130	X	-	-	-
14	CLA	AA	1131	X	-	-	-
14	CLA	AA	1132	X	-	-	-
14	CLA	AA	1133	X	-	-	-
14	CLA	AA	1135	X	-	-	-
14	CLA	AA	1136	X	-	-	-
14	CLA	AA	1137	X	-	-	-
14	CLA	AA	1138	X	-	-	-
14	CLA	AA	1139	X	-	-	-
14	CLA	AA	1140	X	-	-	-
14	CLA	BA	1021	X	-	-	-
14	CLA	BA	1022	X	-	-	-
14	CLA	BA	1023	X	-	-	-
14	CLA	BA	1201	X	-	-	-
14	CLA	BA	1202	X	-	-	-
14	CLA	BA	1203	X	-	-	-
14	CLA	BA	1204	X	-	-	-
14	CLA	BA	1205	X	-	-	-
14	CLA	BA	1206	X	-	-	-
14	CLA	BA	1207	X	-	-	-
14	CLA	BA	1208	X	-	-	-
14	CLA	BA	1209	X	-	-	-
14	CLA	BA	1210	X	-	-	-
14	CLA	BA	1211	X	-	-	-
14	CLA	BA	1212	X	-	-	-
14	CLA	BA	1213	X	-	-	-
14	CLA	BA	1214	X	-	-	-
14	CLA	BA	1215	X	-	-	-
14	CLA	BA	1216	X	-	-	-
14	CLA	BA	1217	X	-	-	-
14	CLA	BA	1218	X	-	-	-
14	CLA	BA	1219	X	-	-	-
14	CLA	BA	1220	X	-	-	-
14	CLA	BA	1221	X	-	-	-
14	CLA	BA	1222	X	-	-	-
14	CLA	BA	1223	X	-	-	-
14	CLA	BA	1224	X	-	-	-
14	CLA	BA	1225	X	-	-	-
14	CLA	BA	1226	X	-	-	-
14	CLA	BA	1227	X	-	-	-
14	CLA	BA	1228	X	-	-	-
14	CLA	BA	1229	X	-	-	-

Continued on next page...

PRELIMINARY VALIDATION REPORT

Continued from previous page...

Mol	Type	Chain	Res	Chirality	Geometry	Clashes	Electron density
14	CLA	BA	1231	X	-	-	-
14	CLA	BA	1232	X	-	-	-
14	CLA	BA	1233	X	-	-	-
14	CLA	BA	1234	X	-	-	-
14	CLA	BA	1235	X	-	-	-
14	CLA	BA	1236	X	-	-	-
14	CLA	BA	1238	X	-	-	-
14	CLA	BA	1239	X	-	-	-
14	CLA	GA	1012	X	-	-	-
14	CLA	GA	1013	X	-	-	-
14	CLA	GA	1101	X	-	-	-
14	CLA	GA	1102	X	-	-	-
14	CLA	GA	1103	X	-	-	-
14	CLA	GA	1104	X	-	-	-
14	CLA	GA	1105	X	-	-	-
14	CLA	GA	1106	X	-	-	-
14	CLA	GA	1107	X	-	-	-
14	CLA	GA	1108	X	-	-	-
14	CLA	GA	1109	X	-	-	-
14	CLA	GA	1110	X	-	-	-
14	CLA	GA	1111	X	-	-	-
14	CLA	GA	1112	X	-	-	-
14	CLA	GA	1113	X	-	-	-
14	CLA	GA	1114	X	-	-	-
14	CLA	GA	1115	X	-	-	-
14	CLA	GA	1116	X	-	-	-
14	CLA	GA	1117	X	-	-	-
14	CLA	GA	1118	X	-	-	-
14	CLA	GA	1119	X	-	-	-
14	CLA	GA	1122	X	-	-	-
14	CLA	GA	1123	X	-	-	-
14	CLA	GA	1124	X	-	-	-
14	CLA	GA	1125	X	-	-	-
14	CLA	GA	1126	X	-	-	-
14	CLA	GA	1127	X	-	-	-
14	CLA	GA	1128	X	-	-	-
14	CLA	GA	1129	X	-	-	-
14	CLA	GA	1130	X	-	-	-
14	CLA	GA	1131	X	-	-	-
14	CLA	GA	1132	X	-	-	-
14	CLA	GA	1133	X	-	-	-
14	CLA	GA	1135	X	-	-	-

Continued on next page...

PRELIMINARY VALIDATION REPORT

Continued from previous page...

Mol	Type	Chain	Res	Chirality	Geometry	Clashes	Electron density
14	CLA	GA	1136	X	-	-	-
14	CLA	GA	1137	X	-	-	-
14	CLA	GA	1138	X	-	-	-
14	CLA	GA	1139	X	-	-	-
14	CLA	GA	1140	X	-	-	-
14	CLA	HA	1021	X	-	-	-
14	CLA	HA	1023	X	-	-	-
14	CLA	HA	1201	X	-	-	-
14	CLA	HA	1202	X	-	-	-
14	CLA	HA	1203	X	-	-	-
14	CLA	HA	1204	X	-	-	-
14	CLA	HA	1205	X	-	-	-
14	CLA	HA	1206	X	-	-	-
14	CLA	HA	1207	X	-	-	-
14	CLA	HA	1208	X	-	-	-
14	CLA	HA	1209	X	-	-	-
14	CLA	HA	1210	X	-	-	-
14	CLA	HA	1211	X	-	-	-
14	CLA	HA	1212	X	-	-	-
14	CLA	HA	1213	X	-	-	-
14	CLA	HA	1214	X	-	-	-
14	CLA	HA	1215	X	-	-	-
14	CLA	HA	1216	X	-	-	-
14	CLA	HA	1217	X	-	-	-
14	CLA	HA	1218	X	-	-	-
14	CLA	HA	1219	X	-	-	-
14	CLA	HA	1220	X	-	-	-
14	CLA	HA	1221	X	-	-	-
14	CLA	HA	1222	X	-	-	-
14	CLA	HA	1223	X	-	-	-
14	CLA	HA	1224	X	-	-	-
14	CLA	HA	1225	X	-	-	-
14	CLA	HA	1226	X	-	-	-
14	CLA	HA	1227	X	-	-	-
14	CLA	HA	1228	X	-	-	-
14	CLA	HA	1229	X	-	-	-
14	CLA	HA	1231	X	-	-	-
14	CLA	HA	1232	X	-	-	-
14	CLA	HA	1233	X	-	-	-
14	CLA	HA	1234	X	-	-	-
14	CLA	HA	1235	X	-	-	-
14	CLA	HA	1236	X	-	-	-

Continued on next page...

PRELIMINARY VALIDATION REPORT

Continued from previous page...

Mol	Type	Chain	Res	Chirality	Geometry	Clashes	Electron density
14	CLA	HA	1238	X	-	-	-
14	CLA	HA	1239	X	-	-	-
14	CLA	KA	1401	X	-	-	-
14	CLA	LA	1501	X	-	-	-
14	CLA	LA	1502	X	-	-	-
14	CLA	LA	1503	X	-	-	-
14	CLA	TA	1401	X	-	-	-
14	CLA	UA	1501	X	-	-	-
14	CLA	UA	1502	X	-	-	-
14	CLA	UA	1503	X	-	-	-
14	CLA	WA	1701	X	-	-	-
14	CLA	XA	1701	X	-	-	-
14	CLA	aA	1012	X	-	-	-
14	CLA	aA	1013	X	-	-	-
14	CLA	aA	1101	X	-	-	-
14	CLA	aA	1102	X	-	-	-
14	CLA	aA	1103	X	-	-	-
14	CLA	aA	1104	X	-	-	-
14	CLA	aA	1105	X	-	-	-
14	CLA	aA	1106	X	-	-	-
14	CLA	aA	1107	X	-	-	-
14	CLA	aA	1108	X	-	-	-
14	CLA	aA	1109	X	-	-	-
14	CLA	aA	1110	X	-	-	-
14	CLA	aA	1111	X	-	-	-
14	CLA	aA	1112	X	-	-	-
14	CLA	aA	1113	X	-	-	-
14	CLA	aA	1114	X	-	-	-
14	CLA	aA	1115	X	-	-	-
14	CLA	aA	1116	X	-	-	-
14	CLA	aA	1117	X	-	-	-
14	CLA	aA	1118	X	-	-	-
14	CLA	aA	1119	X	-	-	-
14	CLA	aA	1122	X	-	-	-
14	CLA	aA	1123	X	-	-	-
14	CLA	aA	1124	X	-	-	-
14	CLA	aA	1125	X	-	-	-
14	CLA	aA	1126	X	-	-	-
14	CLA	aA	1127	X	-	-	-
14	CLA	aA	1128	X	-	-	-
14	CLA	aA	1129	X	-	-	-
14	CLA	aA	1130	X	-	-	-

Continued on next page...

PRELIMINARY VALIDATION REPORT

Continued from previous page...

Mol	Type	Chain	Res	Chirality	Geometry	Clashes	Electron density
14	CLA	aA	1131	X	-	-	-
14	CLA	aA	1132	X	-	-	-
14	CLA	aA	1133	X	-	-	-
14	CLA	aA	1135	X	-	-	-
14	CLA	aA	1136	X	-	-	-
14	CLA	aA	1137	X	-	-	-
14	CLA	aA	1138	X	-	-	-
14	CLA	aA	1139	X	-	-	-
14	CLA	aA	1140	X	-	-	-
14	CLA	bA	1021	X	-	-	-
14	CLA	bA	1022	X	-	-	-
14	CLA	bA	1023	X	-	-	-
14	CLA	bA	1201	X	-	-	-
14	CLA	bA	1202	X	-	-	-
14	CLA	bA	1203	X	-	-	-
14	CLA	bA	1204	X	-	-	-
14	CLA	bA	1205	X	-	-	-
14	CLA	bA	1206	X	-	-	-
14	CLA	bA	1207	X	-	-	-
14	CLA	bA	1208	X	-	-	-
14	CLA	bA	1209	X	-	-	-
14	CLA	bA	1210	X	-	-	-
14	CLA	bA	1211	X	-	-	-
14	CLA	bA	1212	X	-	-	-
14	CLA	bA	1213	X	-	-	-
14	CLA	bA	1214	X	-	-	-
14	CLA	bA	1215	X	-	-	-
14	CLA	bA	1216	X	-	-	-
14	CLA	bA	1217	X	-	-	-
14	CLA	bA	1218	X	-	-	-
14	CLA	bA	1219	X	-	-	-
14	CLA	bA	1220	X	-	-	-
14	CLA	bA	1221	X	-	-	-
14	CLA	bA	1222	X	-	-	-
14	CLA	bA	1223	X	-	-	-
14	CLA	bA	1224	X	-	-	-
14	CLA	bA	1225	X	-	-	-
14	CLA	bA	1226	X	-	-	-
14	CLA	bA	1227	X	-	-	-
14	CLA	bA	1228	X	-	-	-
14	CLA	bA	1229	X	-	-	-
14	CLA	bA	1231	X	-	-	-

Continued on next page...

PRELIMINARY VALIDATION REPORT

Continued from previous page...

Mol	Type	Chain	Res	Chirality	Geometry	Clashes	Electron density
14	CLA	bA	1232	X	-	-	-
14	CLA	bA	1233	X	-	-	-
14	CLA	bA	1234	X	-	-	-
14	CLA	bA	1235	X	-	-	-
14	CLA	bA	1236	X	-	-	-
14	CLA	bA	1238	X	-	-	-
14	CLA	bA	1239	X	-	-	-
14	CLA	kA	1401	X	-	-	-
14	CLA	lA	1501	X	-	-	-
14	CLA	lA	1502	X	-	-	-
14	CLA	lA	1503	X	-	-	-
14	CLA	xA	1701	X	-	-	-

PRELIMINARY VALIDATION REPORT

2 Entry composition (i)

There are 22 unique types of molecules in this entry. The entry contains 71520 atoms, of which 0 are hydrogens and 0 are deuteriums.

In the tables below, the AltConf column contains the number of residues with at least one atom in alternate conformation and the Trace column contains the number of residues modelled with at most 2 atoms.

- Molecule 1 is a protein called photosystem I core protein PsaA.

Mol	Chain	Residues	Atoms					AltConf	Trace
1	A	747	Total 5906	C 3877	N 1008	O 988	S 33	0	0
1	G	747	Total 5906	C 3877	N 1008	O 988	S 33	0	0
1	a	747	Total 5906	C 3877	N 1008	O 988	S 33	0	0

- Molecule 2 is a protein called photosystem I core protein PsaB.

Mol	Chain	Residues	Atoms					AltConf	Trace
2	B	738	Total 5910	C 3894	N 996	O 1002	S 18	0	0
2	H	738	Total 5910	C 3894	N 996	O 1002	S 18	0	0
2	b	738	Total 5910	C 3894	N 996	O 1002	S 18	0	0

- Molecule 3 is a protein called photosystem I iron-sulfur center protein PsaC.

Mol	Chain	Residues	Atoms					AltConf	Trace
3	C	80	Total 601	C 367	N 105	O 118	S 11	0	0
3	N	80	Total 601	C 367	N 105	O 118	S 11	0	0
3	c	80	Total 601	C 367	N 105	O 118	S 11	0	0

- Molecule 4 is a protein called photosystem I protein PsaD.

Mol	Chain	Residues	Atoms					AltConf	Trace
4	D	139	Total 1092	C 694	N 192	O 203	S 3	0	0

Continued on next page...

PRELIMINARY VALIDATION REPORT

Continued from previous page...

Mol	Chain	Residues	Atoms					AltConf	Trace
4	O	139	Total 1092	C 694	N 192	O 203	S 3	0	0
4	d	139	Total 1092	C 694	N 192	O 203	S 3	0	0

- Molecule 5 is a protein called photosystem I reaction center subunit IV.

Mol	Chain	Residues	Atoms				AltConf	Trace
5	E	62	Total 498	C 313	N 88	O 97	0	0
5	P	62	Total 498	C 313	N 88	O 97	0	0
5	e	62	Total 498	C 313	N 88	O 97	0	0

- Molecule 6 is a protein called photosystem I reaction center protein PsaF subunit III.

Mol	Chain	Residues	Atoms					AltConf	Trace
6	F	135	Total 1060	C 694	N 167	O 192	S 7	0	0
6	Q	135	Total 1060	C 694	N 167	O 192	S 7	0	0
6	f	135	Total 1060	C 694	N 167	O 192	S 7	0	0

- Molecule 7 is a protein called photosystem I subunit VIII.

Mol	Chain	Residues	Atoms					AltConf	Trace
7	I	42	Total 350	C 246	N 48	O 54	S 2	0	0
7	R	42	Total 350	C 246	N 48	O 54	S 2	0	0
7	i	42	Total 350	C 246	N 48	O 54	S 2	0	0

- Molecule 8 is a protein called photosystem I reaction center subunit IX.

Mol	Chain	Residues	Atoms				AltConf	Trace
8	J	43	Total 344	C 238	N 51	O 55	0	0
8	S	43	Total 344	C 238	N 51	O 55	0	0

Continued on next page...

PRELIMINARY VALIDATION REPORT

Continued from previous page...

Mol	Chain	Residues	Atoms				AltConf	Trace
8	j	43	Total 344	C 238	N 51	O 55	0	0

- Molecule 9 is a protein called photosystem I reaction center subunit PsaK.

Mol	Chain	Residues	Atoms					AltConf	Trace
9	K	34	Total 201	C 130	N 36	O 34	S 1	0	0
9	T	34	Total 201	C 130	N 36	O 34	S 1	0	0
9	k	34	Total 201	C 130	N 36	O 34	S 1	0	0

- Molecule 10 is a protein called photosystem I reaction center subunit XI.

Mol	Chain	Residues	Atoms					AltConf	Trace
10	L	156	Total 1173	C 760	N 198	O 210	S 5	0	0
10	U	156	Total 1173	C 760	N 198	O 210	S 5	0	0
10	l	156	Total 1173	C 760	N 198	O 210	S 5	0	0

- Molecule 11 is a protein called photosystem I reaction center subunit XII.

Mol	Chain	Residues	Atoms					AltConf	Trace
11	M	31	Total 245	C 164	N 37	O 43	S 1	0	0
11	V	31	Total 245	C 164	N 37	O 43	S 1	0	0
11	m	31	Total 245	C 164	N 37	O 43	S 1	0	0

- Molecule 12 is a protein called photosystem one PsaX.

Mol	Chain	Residues	Atoms				AltConf	Trace
12	W	29	Total 246	C 172	N 36	O 38	0	0
12	X	29	Total 246	C 172	N 36	O 38	0	0
12	x	29	Total 246	C 172	N 36	O 38	0	0

PRELIMINARY VALIDATION REPORT

- Molecule 13 is CHLOROPHYLL A ISOMER (three-letter code: CL0) (formula: $C_{55}H_{72}MgN_4O_5$).

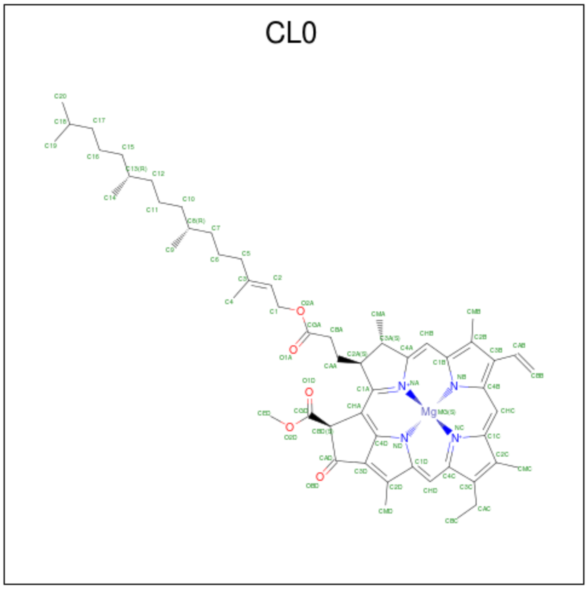

Mol	Chain	Residues	Atoms	AltConf
13	AA	1	Total C Mg N O<br>65 55 1 4 5	0
13	GA	1	Total C Mg N O<br>65 55 1 4 5	0
13	aA	1	Total C Mg N O<br>65 55 1 4 5	0

- Molecule 14 is CHLOROPHYLL A (three-letter code: CLA) (formula: $C_{55}H_{72}MgN_4O_5$).

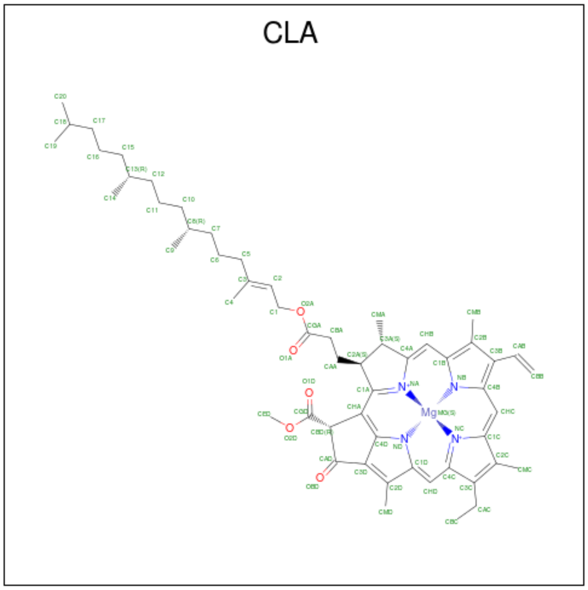

VALIDATION REPORT

Mol	Chain	Residues	Atoms	AltConf
14	AA	1	Total C Mg N O<br>2140 1750 39 156 195	0
14	AA	1	Total C Mg N O<br>2140 1750 39 156 195	0
14	AA	1	Total C Mg N O<br>2140 1750 39 156 195	0
14	AA	1	Total C Mg N O<br>2140 1750 39 156 195	0
14	AA	1	Total C Mg N O<br>2140 1750 39 156 195	0
14	AA	1	Total C Mg N O<br>2140 1750 39 156 195	0
14	AA	1	Total C Mg N O<br>2140 1750 39 156 195	0
14	AA	1	Total C Mg N O<br>2140 1750 39 156 195	0
14	AA	1	Total C Mg N O<br>2140 1750 39 156 195	0
14	AA	1	Total C Mg N O<br>2140 1750 39 156 195	0
14	AA	1	Total C Mg N O<br>2140 1750 39 156 195	0
14	AA	1	Total C Mg N O<br>2140 1750 39 156 195	0
14	AA	1	Total C Mg N O<br>2140 1750 39 156 195	0
14	AA	1	Total C Mg N O<br>2140 1750 39 156 195	0
14	AA	1	Total C Mg N O<br>2140 1750 39 156 195	0
14	AA	1	Total C Mg N O<br>2140 1750 39 156 195	0
14	AA	1	Total C Mg N O<br>2140 1750 39 156 195	0
14	AA	1	Total C Mg N O<br>2140 1750 39 156 195	0
14	AA	1	Total C Mg N O<br>2140 1750 39 156 195	0
14	AA	1	Total C Mg N O<br>2140 1750 39 156 195	0
14	AA	1	Total C Mg N O<br>2140 1750 39 156 195	0
14	AA	1	Total C Mg N O<br>2140 1750 39 156 195	0

Continued on next page...

PRELIMINARY VALIDATION REPORT

Continued from previous page...

Mol	Chain	Residues	Atoms					AltConf
14	AA	1	Total 2140	C 1750	Mg 39	N 156	O 195	0
14	AA	1	Total 2140	C 1750	Mg 39	N 156	O 195	0
14	AA	1	Total 2140	C 1750	Mg 39	N 156	O 195	0
14	AA	1	Total 2140	C 1750	Mg 39	N 156	O 195	0
14	AA	1	Total 2140	C 1750	Mg 39	N 156	O 195	0
14	AA	1	Total 2140	C 1750	Mg 39	N 156	O 195	0
14	AA	1	Total 2140	C 1750	Mg 39	N 156	O 195	0
14	AA	1	Total 2140	C 1750	Mg 39	N 156	O 195	0
14	AA	1	Total 2140	C 1750	Mg 39	N 156	O 195	0
14	AA	1	Total 2140	C 1750	Mg 39	N 156	O 195	0
14	AA	1	Total 2140	C 1750	Mg 39	N 156	O 195	0
14	AA	1	Total 2140	C 1750	Mg 39	N 156	O 195	0
14	AA	1	Total 2140	C 1750	Mg 39	N 156	O 195	0
14	AA	1	Total 2140	C 1750	Mg 39	N 156	O 195	0
14	AA	1	Total 2140	C 1750	Mg 39	N 156	O 195	0
14	AA	1	Total 2140	C 1750	Mg 39	N 156	O 195	0
14	AA	1	Total 2140	C 1750	Mg 39	N 156	O 195	0
14	BA	1	Total 2280	C 1880	Mg 40	N 160	O 200	0
14	BA	1	Total 2280	C 1880	Mg 40	N 160	O 200	0
14	BA	1	Total 2280	C 1880	Mg 40	N 160	O 200	0
14	BA	1	Total 2280	C 1880	Mg 40	N 160	O 200	0

Continued on next page...

PRELIMINARY VALIDATION REPORT

Continued from previous page...

Mol	Chain	Residues	Atoms					AltConf
14	BA	1	Total 2280	C 1880	Mg 40	N 160	O 200	0
14	BA	1	Total 2280	C 1880	Mg 40	N 160	O 200	0
14	BA	1	Total 2280	C 1880	Mg 40	N 160	O 200	0
14	BA	1	Total 2280	C 1880	Mg 40	N 160	O 200	0
14	BA	1	Total 2280	C 1880	Mg 40	N 160	O 200	0
14	BA	1	Total 2280	C 1880	Mg 40	N 160	O 200	0
14	BA	1	Total 2280	C 1880	Mg 40	N 160	O 200	0
14	BA	1	Total 2280	C 1880	Mg 40	N 160	O 200	0
14	BA	1	Total 2280	C 1880	Mg 40	N 160	O 200	0
14	BA	1	Total 2280	C 1880	Mg 40	N 160	O 200	0
14	BA	1	Total 2280	C 1880	Mg 40	N 160	O 200	0
14	BA	1	Total 2280	C 1880	Mg 40	N 160	O 200	0
14	BA	1	Total 2280	C 1880	Mg 40	N 160	O 200	0
14	BA	1	Total 2280	C 1880	Mg 40	N 160	O 200	0
14	BA	1	Total 2280	C 1880	Mg 40	N 160	O 200	0
14	BA	1	Total 2280	C 1880	Mg 40	N 160	O 200	0
14	BA	1	Total 2280	C 1880	Mg 40	N 160	O 200	0
14	BA	1	Total 2280	C 1880	Mg 40	N 160	O 200	0
14	BA	1	Total 2280	C 1880	Mg 40	N 160	O 200	0
14	BA	1	Total 2280	C 1880	Mg 40	N 160	O 200	0
14	BA	1	Total 2280	C 1880	Mg 40	N 160	O 200	0

Continued on next page...

PRELIMINARY VALIDATION REPORT

Continued from previous page...

Mol	Chain	Residues	Atoms					AltConf
14	BA	1	Total 2280	C 1880	Mg 40	N 160	O 200	0
14	BA	1	Total 2280	C 1880	Mg 40	N 160	O 200	0
14	BA	1	Total 2280	C 1880	Mg 40	N 160	O 200	0
14	BA	1	Total 2280	C 1880	Mg 40	N 160	O 200	0
14	BA	1	Total 2280	C 1880	Mg 40	N 160	O 200	0
14	BA	1	Total 2280	C 1880	Mg 40	N 160	O 200	0
14	BA	1	Total 2280	C 1880	Mg 40	N 160	O 200	0
14	BA	1	Total 2280	C 1880	Mg 40	N 160	O 200	0
14	BA	1	Total 2280	C 1880	Mg 40	N 160	O 200	0
14	BA	1	Total 2280	C 1880	Mg 40	N 160	O 200	0
14	BA	1	Total 2280	C 1880	Mg 40	N 160	O 200	0
14	BA	1	Total 2280	C 1880	Mg 40	N 160	O 200	0
14	BA	1	Total 2280	C 1880	Mg 40	N 160	O 200	0
14	BA	1	Total 2280	C 1880	Mg 40	N 160	O 200	0
14	BA	1	Total 2280	C 1880	Mg 40	N 160	O 200	0
14	GA	1	Total 2140	C 1750	Mg 39	N 156	O 195	0
14	GA	1	Total 2140	C 1750	Mg 39	N 156	O 195	0
14	GA	1	Total 2140	C 1750	Mg 39	N 156	O 195	0
14	GA	1	Total 2140	C 1750	Mg 39	N 156	O 195	0
14	GA	1	Total 2140	C 1750	Mg 39	N 156	O 195	0
14	GA	1	Total 2140	C 1750	Mg 39	N 156	O 195	0

Continued on next page...

PRELIMINARY VALIDATION REPORT

Continued from previous page...

Mol	Chain	Residues	Atoms					AltConf
14	GA	1	Total 2140	C 1750	Mg 39	N 156	O 195	0
14	GA	1	Total 2140	C 1750	Mg 39	N 156	O 195	0
14	GA	1	Total 2140	C 1750	Mg 39	N 156	O 195	0
14	GA	1	Total 2140	C 1750	Mg 39	N 156	O 195	0
14	GA	1	Total 2140	C 1750	Mg 39	N 156	O 195	0
14	GA	1	Total 2140	C 1750	Mg 39	N 156	O 195	0
14	GA	1	Total 2140	C 1750	Mg 39	N 156	O 195	0
14	GA	1	Total 2140	C 1750	Mg 39	N 156	O 195	0
14	GA	1	Total 2140	C 1750	Mg 39	N 156	O 195	0
14	GA	1	Total 2140	C 1750	Mg 39	N 156	O 195	0
14	GA	1	Total 2140	C 1750	Mg 39	N 156	O 195	0
14	GA	1	Total 2140	C 1750	Mg 39	N 156	O 195	0
14	GA	1	Total 2140	C 1750	Mg 39	N 156	O 195	0
14	GA	1	Total 2140	C 1750	Mg 39	N 156	O 195	0
14	GA	1	Total 2140	C 1750	Mg 39	N 156	O 195	0
14	GA	1	Total 2140	C 1750	Mg 39	N 156	O 195	0
14	GA	1	Total 2140	C 1750	Mg 39	N 156	O 195	0
14	GA	1	Total 2140	C 1750	Mg 39	N 156	O 195	0
14	GA	1	Total 2140	C 1750	Mg 39	N 156	O 195	0
14	GA	1	Total 2140	C 1750	Mg 39	N 156	O 195	0
14	GA	1	Total 2140	C 1750	Mg 39	N 156	O 195	0

Continued on next page...

PRELIMINARY VALIDATION REPORT

Continued from previous page...

Mol	Chain	Residues	Atoms	AltConf
14	GA	1	Total 2140, C 1750, Mg 39, N 156, O 195	0
14	GA	1	Total 2140, C 1750, Mg 39, N 156, O 195	0
14	GA	1	Total 2140, C 1750, Mg 39, N 156, O 195	0
14	GA	1	Total 2140, C 1750, Mg 39, N 156, O 195	0
14	GA	1	Total 2140, C 1750, Mg 39, N 156, O 195	0
14	GA	1	Total 2140, C 1750, Mg 39, N 156, O 195	0
14	GA	1	Total 2140, C 1750, Mg 39, N 156, O 195	0
14	GA	1	Total 2140, C 1750, Mg 39, N 156, O 195	0
14	GA	1	Total 2140, C 1750, Mg 39, N 156, O 195	0
14	GA	1	Total 2140, C 1750, Mg 39, N 156, O 195	0
14	GA	1	Total 2140, C 1750, Mg 39, N 156, O 195	0
14	GA	1	Total 2140, C 1750, Mg 39, N 156, O 195	0
14	0	1	Total 65, C 55, Mg 1, N 4, O 5	0
14	HA	1	Total 2215, C 1825, Mg 39, N 156, O 195	0
14	HA	1	Total 2215, C 1825, Mg 39, N 156, O 195	0
14	HA	1	Total 2215, C 1825, Mg 39, N 156, O 195	0
14	HA	1	Total 2215, C 1825, Mg 39, N 156, O 195	0
14	HA	1	Total 2215, C 1825, Mg 39, N 156, O 195	0
14	HA	1	Total 2215, C 1825, Mg 39, N 156, O 195	0
14	HA	1	Total 2215, C 1825, Mg 39, N 156, O 195	0
14	HA	1	Total 2215, C 1825, Mg 39, N 156, O 195	0

Continued on next page...

PRELIMINARY VALIDATION REPORT

Continued from previous page...

Mol	Chain	Residues	Atoms	AltConf
14	HA	1	Total C Mg N O<br>2215 1825 39 156 195	0
14	HA	1	Total C Mg N O<br>2215 1825 39 156 195	0
14	HA	1	Total C Mg N O<br>2215 1825 39 156 195	0
14	HA	1	Total C Mg N O<br>2215 1825 39 156 195	0
14	HA	1	Total C Mg N O<br>2215 1825 39 156 195	0
14	HA	1	Total C Mg N O<br>2215 1825 39 156 195	0
14	HA	1	Total C Mg N O<br>2215 1825 39 156 195	0
14	HA	1	Total C Mg N O<br>2215 1825 39 156 195	0
14	HA	1	Total C Mg N O<br>2215 1825 39 156 195	0
14	HA	1	Total C Mg N O<br>2215 1825 39 156 195	0
14	HA	1	Total C Mg N O<br>2215 1825 39 156 195	0
14	HA	1	Total C Mg N O<br>2215 1825 39 156 195	0
14	HA	1	Total C Mg N O<br>2215 1825 39 156 195	0
14	HA	1	Total C Mg N O<br>2215 1825 39 156 195	0
14	HA	1	Total C Mg N O<br>2215 1825 39 156 195	0
14	HA	1	Total C Mg N O<br>2215 1825 39 156 195	0
14	HA	1	Total C Mg N O<br>2215 1825 39 156 195	0
14	HA	1	Total C Mg N O<br>2215 1825 39 156 195	0
14	HA	1	Total C Mg N O<br>2215 1825 39 156 195	0
14	HA	1	Total C Mg N O<br>2215 1825 39 156 195	0
14	HA	1	Total C Mg N O<br>2215 1825 39 156 195	0

Continued on next page...

PRELIMINARY VALIDATION REPORT

Continued from previous page...

Mol	Chain	Residues	Atoms	AltConf
14	HA	1	Total C Mg N O<br>2215 1825 39 156 195	0
14	HA	1	Total C Mg N O<br>2215 1825 39 156 195	0
14	HA	1	Total C Mg N O<br>2215 1825 39 156 195	0
14	HA	1	Total C Mg N O<br>2215 1825 39 156 195	0
14	HA	1	Total C Mg N O<br>2215 1825 39 156 195	0
14	HA	1	Total C Mg N O<br>2215 1825 39 156 195	0
14	HA	1	Total C Mg N O<br>2215 1825 39 156 195	0
14	HA	1	Total C Mg N O<br>2215 1825 39 156 195	0
14	HA	1	Total C Mg N O<br>2215 1825 39 156 195	0
14	HA	1	Total C Mg N O<br>2215 1825 39 156 195	0
14	KA	1	Total C Mg N O<br>45 35 1 4 5	0
14	LA	1	Total C Mg N O<br>185 155 3 12 15	0
14	LA	1	Total C Mg N O<br>185 155 3 12 15	0
14	LA	1	Total C Mg N O<br>185 155 3 12 15	0
14	TA	1	Total C Mg N O<br>45 35 1 4 5	0
14	UA	1	Total C Mg N O<br>185 155 3 12 15	0
14	UA	1	Total C Mg N O<br>185 155 3 12 15	0
14	UA	1	Total C Mg N O<br>185 155 3 12 15	0
14	WA	1	Total C Mg N O<br>45 35 1 4 5	0
14	XA	1	Total C Mg N O<br>45 35 1 4 5	0
14	aA	1	Total C Mg N O<br>2140 1750 39 156 195	0

Continued on next page...

PRELIMINARY VALIDATION REPORT

Continued from previous page...

Mol	Chain	Residues	Atoms					AltConf
14	aA	1	Total 2140	C 1750	Mg 39	N 156	O 195	0
14	aA	1	Total 2140	C 1750	Mg 39	N 156	O 195	0
14	aA	1	Total 2140	C 1750	Mg 39	N 156	O 195	0
14	aA	1	Total 2140	C 1750	Mg 39	N 156	O 195	0
14	aA	1	Total 2140	C 1750	Mg 39	N 156	O 195	0
14	aA	1	Total 2140	C 1750	Mg 39	N 156	O 195	0
14	aA	1	Total 2140	C 1750	Mg 39	N 156	O 195	0
14	aA	1	Total 2140	C 1750	Mg 39	N 156	O 195	0
14	aA	1	Total 2140	C 1750	Mg 39	N 156	O 195	0
14	aA	1	Total 2140	C 1750	Mg 39	N 156	O 195	0
14	aA	1	Total 2140	C 1750	Mg 39	N 156	O 195	0
14	aA	1	Total 2140	C 1750	Mg 39	N 156	O 195	0
14	aA	1	Total 2140	C 1750	Mg 39	N 156	O 195	0
14	aA	1	Total 2140	C 1750	Mg 39	N 156	O 195	0
14	aA	1	Total 2140	C 1750	Mg 39	N 156	O 195	0
14	aA	1	Total 2140	C 1750	Mg 39	N 156	O 195	0
14	aA	1	Total 2140	C 1750	Mg 39	N 156	O 195	0
14	aA	1	Total 2140	C 1750	Mg 39	N 156	O 195	0
14	aA	1	Total 2140	C 1750	Mg 39	N 156	O 195	0
14	aA	1	Total 2140	C 1750	Mg 39	N 156	O 195	0
14	aA	1	Total 2140	C 1750	Mg 39	N 156	O 195	0

Continued on next page...

PRELIMINARY VALIDATION REPORT

Continued from previous page...

Mol	Chain	Residues	Atoms	AltConf
14	aA	1	Total C Mg N O<br>2140 1750 39 156 195	0
14	aA	1	Total C Mg N O<br>2140 1750 39 156 195	0
14	aA	1	Total C Mg N O<br>2140 1750 39 156 195	0
14	aA	1	Total C Mg N O<br>2140 1750 39 156 195	0
14	aA	1	Total C Mg N O<br>2140 1750 39 156 195	0
14	aA	1	Total C Mg N O<br>2140 1750 39 156 195	0
14	aA	1	Total C Mg N O<br>2140 1750 39 156 195	0
14	aA	1	Total C Mg N O<br>2140 1750 39 156 195	0
14	aA	1	Total C Mg N O<br>2140 1750 39 156 195	0
14	aA	1	Total C Mg N O<br>2140 1750 39 156 195	0
14	aA	1	Total C Mg N O<br>2140 1750 39 156 195	0
14	aA	1	Total C Mg N O<br>2140 1750 39 156 195	0
14	aA	1	Total C Mg N O<br>2140 1750 39 156 195	0
14	aA	1	Total C Mg N O<br>2140 1750 39 156 195	0
14	aA	1	Total C Mg N O<br>2140 1750 39 156 195	0
14	aA	1	Total C Mg N O<br>2140 1750 39 156 195	0
14	aA	1	Total C Mg N O<br>2140 1750 39 156 195	0
14	bA	1	Total C Mg N O<br>2280 1880 40 160 200	0
14	bA	1	Total C Mg N O<br>2280 1880 40 160 200	0
14	bA	1	Total C Mg N O<br>2280 1880 40 160 200	0
14	bA	1	Total C Mg N O<br>2280 1880 40 160 200	0

Continued on next page...

PRELIMINARY VALIDATION REPORT

Continued from previous page...

Mol	Chain	Residues	Atoms					AltConf
14	bA	1	Total<br>2280	C<br>1880	Mg<br>40	N<br>160	O<br>200	0
14	bA	1	Total<br>2280	C<br>1880	Mg<br>40	N<br>160	O<br>200	0
14	bA	1	Total<br>2280	C<br>1880	Mg<br>40	N<br>160	O<br>200	0
14	bA	1	Total<br>2280	C<br>1880	Mg<br>40	N<br>160	O<br>200	0
14	bA	1	Total<br>2280	C<br>1880	Mg<br>40	N<br>160	O<br>200	0
14	bA	1	Total<br>2280	C<br>1880	Mg<br>40	N<br>160	O<br>200	0
14	bA	1	Total<br>2280	C<br>1880	Mg<br>40	N<br>160	O<br>200	0
14	bA	1	Total<br>2280	C<br>1880	Mg<br>40	N<br>160	O<br>200	0
14	bA	1	Total<br>2280	C<br>1880	Mg<br>40	N<br>160	O<br>200	0
14	bA	1	Total<br>2280	C<br>1880	Mg<br>40	N<br>160	O<br>200	0
14	bA	1	Total<br>2280	C<br>1880	Mg<br>40	N<br>160	O<br>200	0
14	bA	1	Total<br>2280	C<br>1880	Mg<br>40	N<br>160	O<br>200	0
14	bA	1	Total<br>2280	C<br>1880	Mg<br>40	N<br>160	O<br>200	0
14	bA	1	Total<br>2280	C<br>1880	Mg<br>40	N<br>160	O<br>200	0
14	bA	1	Total<br>2280	C<br>1880	Mg<br>40	N<br>160	O<br>200	0
14	bA	1	Total<br>2280	C<br>1880	Mg<br>40	N<br>160	O<br>200	0
14	bA	1	Total<br>2280	C<br>1880	Mg<br>40	N<br>160	O<br>200	0
14	bA	1	Total<br>2280	C<br>1880	Mg<br>40	N<br>160	O<br>200	0
14	bA	1	Total<br>2280	C<br>1880	Mg<br>40	N<br>160	O<br>200	0
14	bA	1	Total<br>2280	C<br>1880	Mg<br>40	N<br>160	O<br>200	0
14	bA	1	Total<br>2280	C<br>1880	Mg<br>40	N<br>160	O<br>200	0

Continued on next page...

PRELIMINARY VALIDATION REPORT

Continued from previous page...

Mol	Chain	Residues	Atoms	AltConf
14	bA	1	Total C Mg N O<br>2280 1880 40 160 200	0
14	bA	1	Total C Mg N O<br>2280 1880 40 160 200	0
14	bA	1	Total C Mg N O<br>2280 1880 40 160 200	0
14	bA	1	Total C Mg N O<br>2280 1880 40 160 200	0
14	bA	1	Total C Mg N O<br>2280 1880 40 160 200	0
14	bA	1	Total C Mg N O<br>2280 1880 40 160 200	0
14	bA	1	Total C Mg N O<br>2280 1880 40 160 200	0
14	bA	1	Total C Mg N O<br>2280 1880 40 160 200	0
14	bA	1	Total C Mg N O<br>2280 1880 40 160 200	0
14	bA	1	Total C Mg N O<br>2280 1880 40 160 200	0
14	bA	1	Total C Mg N O<br>2280 1880 40 160 200	0
14	bA	1	Total C Mg N O<br>2280 1880 40 160 200	0
14	bA	1	Total C Mg N O<br>2280 1880 40 160 200	0
14	bA	1	Total C Mg N O<br>2280 1880 40 160 200	0
14	bA	1	Total C Mg N O<br>2280 1880 40 160 200	0
14	kA	1	Total C Mg N O<br>45 35 1 4 5	0
14	lA	1	Total C Mg N O<br>185 155 3 12 15	0
14	lA	1	Total C Mg N O<br>185 155 3 12 15	0
14	lA	1	Total C Mg N O<br>185 155 3 12 15	0
14	xA	1	Total C Mg N O<br>45 35 1 4 5	0

- Molecule 15 is a ligand with the chemical component id F6C but there is no existing wwPDB Chemical Component Dictionary definition for F6C. Consequently no firm identification of ligand chemistry can be made. Once the structure is annotated then an identification and

PRELIMINARY VALIDATION REPORT

diagram will be given here.

Mol	Chain	Residues	Atoms	AltConf
15	AA	1	Total C Mg N O<br>92 70 2 8 12	0
15	AA	1	Total C Mg N O<br>92 70 2 8 12	0
15	BA	1	Total C Mg N O<br>107 85 2 8 12	0
15	BA	1	Total C Mg N O<br>107 85 2 8 12	0
15	GA	1	Total C Mg N O<br>92 70 2 8 12	0
15	GA	1	Total C Mg N O<br>92 70 2 8 12	0
15	HA	1	Total C Mg N O<br>107 85 2 8 12	0
15	HA	1	Total C Mg N O<br>107 85 2 8 12	0
15	aA	1	Total C Mg N O<br>92 70 2 8 12	0
15	aA	1	Total C Mg N O<br>92 70 2 8 12	0
15	bA	1	Total C Mg N O<br>107 85 2 8 12	0
15	bA	1	Total C Mg N O<br>107 85 2 8 12	0

- Molecule 16 is PHYLLOQUINONE (three-letter code: PQN) (formula: $C_{31}H_{46}O_2$).

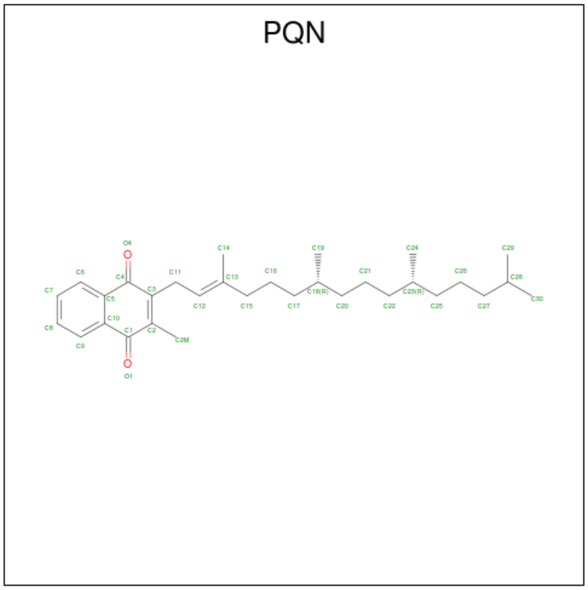

PRELIMINARY VALIDATION REPORT

Mol	Chain	Residues	Atoms			AltConf
16	AA	1	Total 33	C 31	O 2	0
16	BA	1	Total 33	C 31	O 2	0
16	GA	1	Total 33	C 31	O 2	0
16	HA	1	Total 33	C 31	O 2	0
16	aA	1	Total 33	C 31	O 2	0
16	bA	1	Total 33	C 31	O 2	0

- Molecule 17 is IRON/SULFUR CLUSTER (three-letter code: SF4) (formula: Fe_4S_4).

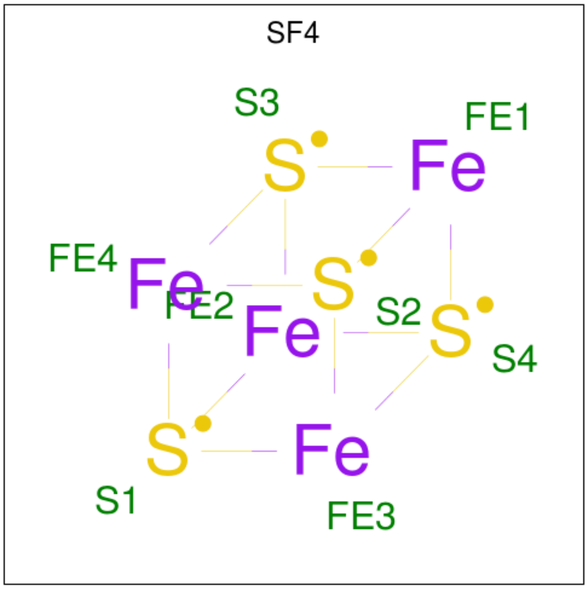

Mol	Chain	Residues	Atoms			AltConf
17	AA	1	Total 8	Fe 4	S 4	0
17	CA	1	Total 16	Fe 8	S 8	0
17	CA	1	Total 16	Fe 8	S 8	0
17	GA	1	Total 8	Fe 4	S 4	0
17	NA	1	Total 16	Fe 8	S 8	0
17	NA	1	Total 16	Fe 8	S 8	0

Continued on next page...

PRELIMINARY VALIDATION REPORT

Continued from previous page...

Mol	Chain	Residues	Atoms	AltConf
17	aA	1	Total Fe S<br>8 4 4	0
17	cA	1	Total Fe S<br>16 8 8	0
17	cA	1	Total Fe S<br>16 8 8	0

- Molecule 18 is BETA-CAROTENE (three-letter code: BCR) (formula: $C_{40}H_{56}$).

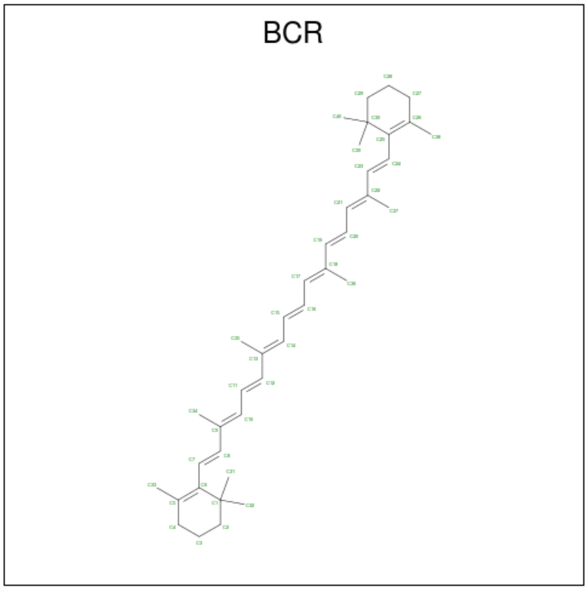

Mol	Chain	Residues	Atoms	AltConf
18	AA	1	Total C<br>240 240	0
18	AA	1	Total C<br>240 240	0
18	AA	1	Total C<br>240 240	0
18	AA	1	Total C<br>240 240	0
18	AA	1	Total C<br>240 240	0
18	AA	1	Total C<br>240 240	0
18	BA	1	Total C<br>400 400	0
18	BA	1	Total C<br>400 400	0

Continued on next page...

PRELIMINARY VALIDATION REPORT

Continued from previous page...

Mol	Chain	Residues	Atoms	AltConf
18	BA	1	Total C<br>400 400	0
18	BA	1	Total C<br>400 400	0
18	BA	1	Total C<br>400 400	0
18	BA	1	Total C<br>400 400	0
18	BA	1	Total C<br>400 400	0
18	BA	1	Total C<br>400 400	0
18	BA	1	Total C<br>400 400	0
18	BA	1	Total C<br>400 400	0
18	GA	1	Total C<br>240 240	0
18	GA	1	Total C<br>240 240	0
18	GA	1	Total C<br>240 240	0
18	GA	1	Total C<br>240 240	0
18	GA	1	Total C<br>240 240	0
18	GA	1	Total C<br>240 240	0
18	HA	1	Total C<br>400 400	0
18	HA	1	Total C<br>400 400	0
18	HA	1	Total C<br>400 400	0
18	HA	1	Total C<br>400 400	0
18	HA	1	Total C<br>400 400	0
18	HA	1	Total C<br>400 400	0
18	HA	1	Total C<br>400 400	0

Continued on next page...

PRELIMINARY VALIDATION REPORT

Continued from previous page...

Mol	Chain	Residues	Atoms	AltConf
18	HA	1	Total C<br>400 400	0
18	HA	1	Total C<br>400 400	0
18	HA	1	Total C<br>400 400	0
18	IA	1	Total C<br>80 80	0
18	IA	1	Total C<br>80 80	0
18	LA	1	Total C<br>80 80	0
18	LA	1	Total C<br>80 80	0
18	MA	1	Total C<br>40 40	0
18	RA	1	Total C<br>80 80	0
18	RA	1	Total C<br>80 80	0
18	UA	1	Total C<br>80 80	0
18	UA	1	Total C<br>80 80	0
18	VA	1	Total C<br>40 40	0
18	aA	1	Total C<br>240 240	0
18	aA	1	Total C<br>240 240	0
18	aA	1	Total C<br>240 240	0
18	aA	1	Total C<br>240 240	0
18	aA	1	Total C<br>240 240	0
18	aA	1	Total C<br>240 240	0
18	bA	1	Total C<br>400 400	0
18	bA	1	Total C<br>400 400	0

Continued on next page...

PRELIMINARY VALIDATION REPORT

Continued from previous page...

Mol	Chain	Residues	Atoms	AltConf
18	bA	1	Total C<br>400 400	0
18	bA	1	Total C<br>400 400	0
18	bA	1	Total C<br>400 400	0
18	bA	1	Total C<br>400 400	0
18	bA	1	Total C<br>400 400	0
18	bA	1	Total C<br>400 400	0
18	bA	1	Total C<br>400 400	0
18	bA	1	Total C<br>400 400	0
18	iA	1	Total C<br>80 80	0
18	iA	1	Total C<br>80 80	0
18	lA	1	Total C<br>80 80	0
18	lA	1	Total C<br>80 80	0
18	mA	1	Total C<br>40 40	0

- Molecule 19 is 1,2-DIPALMITOYL-PHOSPHATIDYL-GLYCEROLE (three-letter code: LHG) (formula: $C_{38}H_{75}O_{10}P$).

PRELIMINARY VALIDATION REPORT

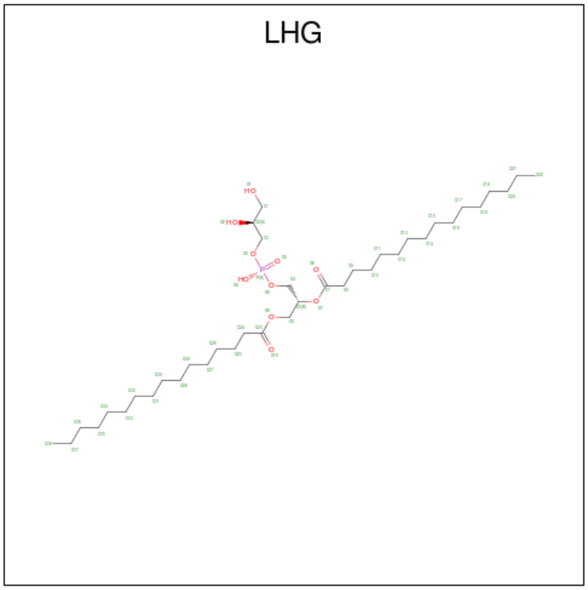

Mol	Chain	Residues	Atoms				AltConf
19	AA	1	Total 42	C 31	O 10	P 1	0
19	BA	1	Total 44	C 33	O 10	P 1	0
19	GA	1	Total 42	C 31	O 10	P 1	0
19	HA	1	Total 44	C 33	O 10	P 1	0
19	aA	1	Total 42	C 31	O 10	P 1	0
19	bA	1	Total 44	C 33	O 10	P 1	0

- Molecule 20 is 1,2-DISTEAROYL-MONOGALACTOSYL-DIGLYCERIDE (three-letter code: LMG) (formula: $C_{45}H_{86}O_{10}$).

PRELIMINARY VALIDATION REPORT

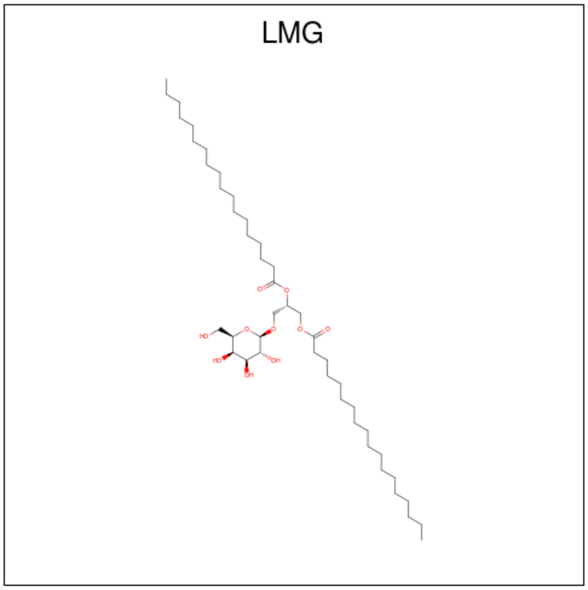

Mol	Chain	Residues	Atoms	AltConf
20	AA	1	Total C O<br>46 36 10	0
20	BA	1	Total C O<br>46 36 10	0
20	GA	1	Total C O<br>46 36 10	0
20	HA	1	Total C O<br>46 36 10	0
20	IA	1	Total C O<br>37 27 10	0
20	LA	1	Total C O<br>50 40 10	0
20	RA	1	Total C O<br>37 27 10	0
20	UA	1	Total C O<br>50 40 10	0
20	aA	1	Total C O<br>46 36 10	0
20	bA	1	Total C O<br>46 36 10	0
20	iA	1	Total C O<br>37 27 10	0
20	lA	1	Total C O<br>50 40 10	0

- Molecule 21 is DODECYL-BETA-D-MALTOSIDE (three-letter code: LMT) (formula: $C_{24}H_{46}O_{11}$).

PRELIMINARY VALIDATION REPORT

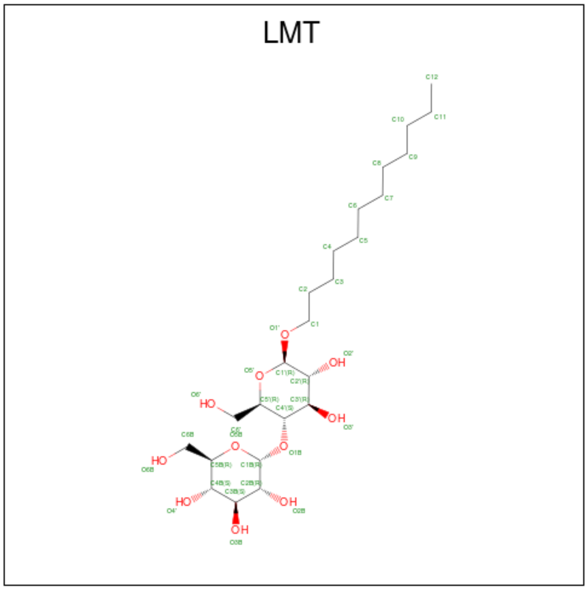

Mol	Chain	Residues	Atoms	AltConf
21	AA	1	Total C O<br>59 37 22	0
21	AA	1	Total C O<br>59 37 22	0
21	GA	1	Total C O<br>59 37 22	0
21	GA	1	Total C O<br>59 37 22	0
21	aA	1	Total C O<br>59 37 22	0
21	aA	1	Total C O<br>59 37 22	0

- Molecule 22 is CALCIUM ION (three-letter code: CA) (formula: Ca).

Mol	Chain	Residues	Atoms	AltConf
22	LA	1	Total Ca<br>1 1	0
22	UA	1	Total Ca<br>1 1	0
22	lA	1	Total Ca<br>1 1	0

PRELIMINARY VALIDATION REPORT

3 Residue-property plots (i)

These plots are drawn for all protein, RNA and DNA chains in the entry. The first graphic for a chain summarises the proportions of the various outlier classes displayed in the second graphic. The second graphic shows the sequence view annotated by issues in geometry. Residues are color-coded according to the number of geometric quality criteria for which they contain at least one outlier: green = 0, yellow = 1, orange = 2 and red = 3 or more. Stretches of 2 or more consecutive residues without any outlier are shown as a green connector. Residues present in the sample, but not in the model, are shown in grey.

- Molecule 1: photosystem I core protein PsaA

Chain A:

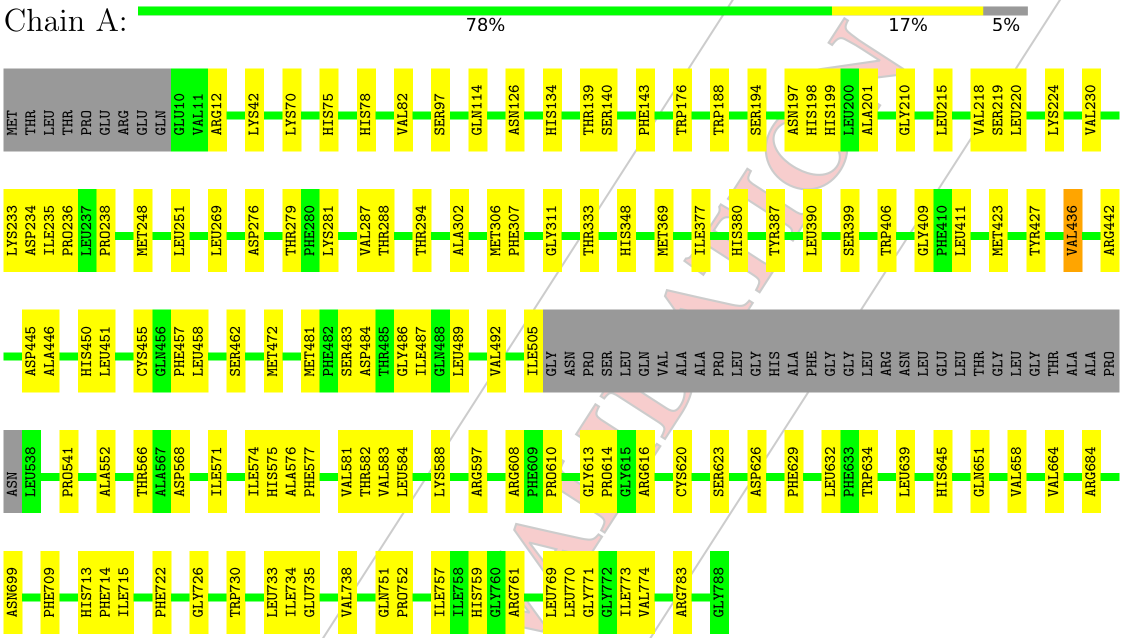

- Molecule 1: photosystem I core protein PsaA

Chain G:

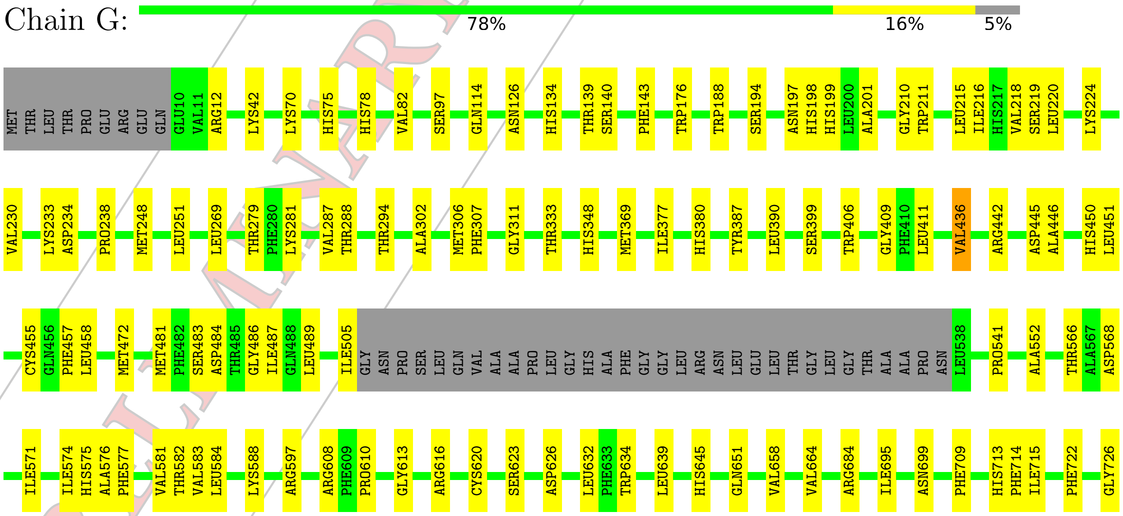

PRELIMINARY VALIDATION REPORT

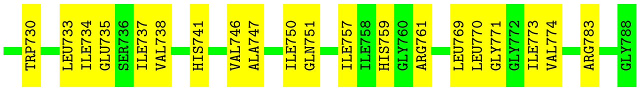

• Molecule 1: photosystem I core protein PsaA

Chain a:

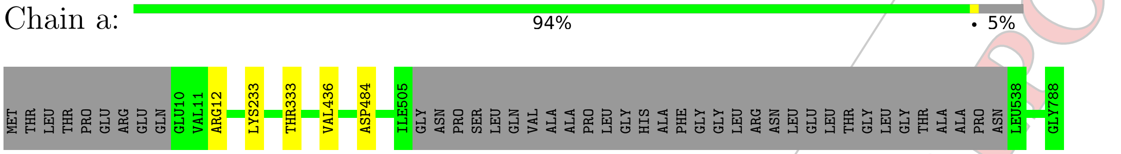

• Molecule 2: photosystem I core protein PsaB

Chain B:

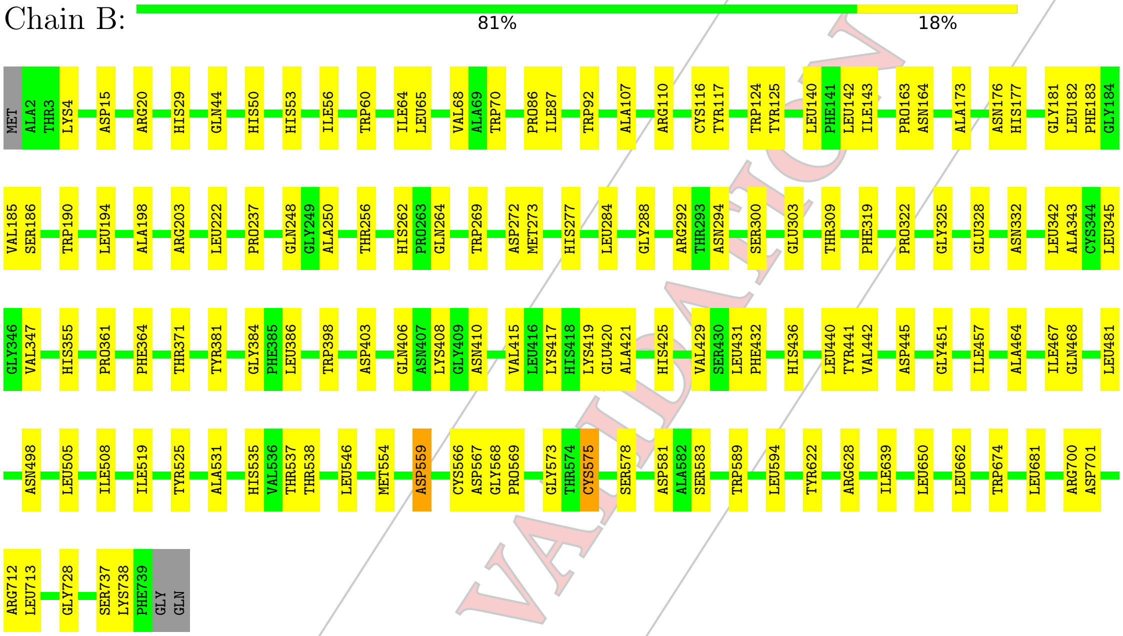

• Molecule 2: photosystem I core protein PsaB

Chain H:

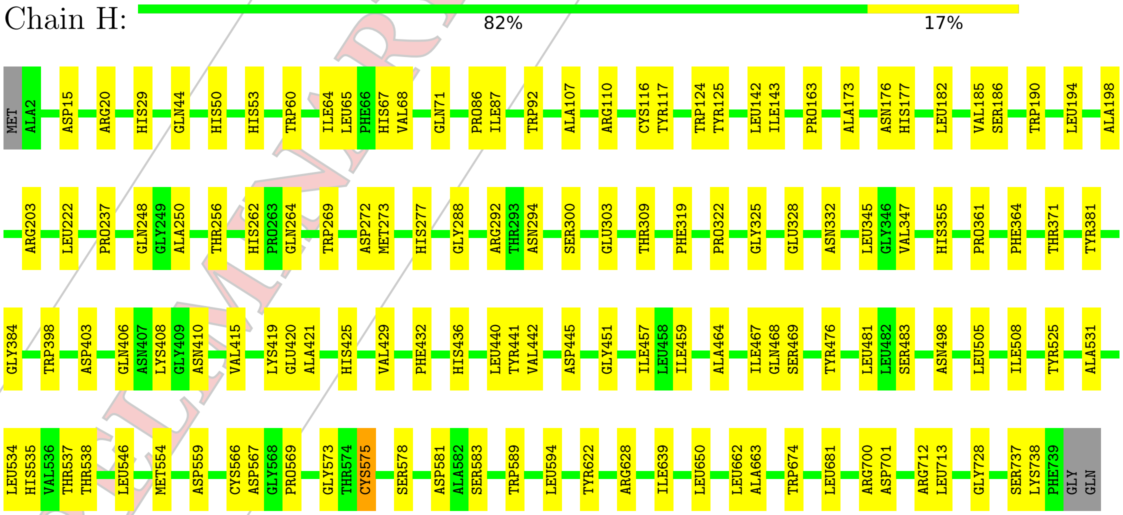

• Molecule 2: photosystem I core protein PsaB

PRELIMINARY VALIDATION REPORT

Chain b:

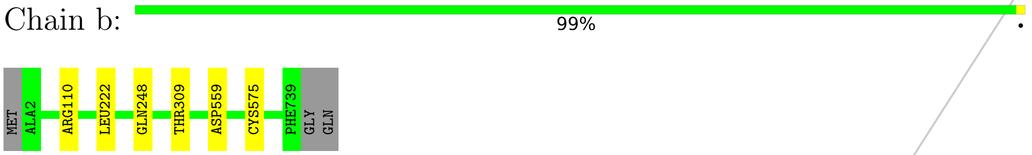

• Molecule 3: photosystem I iron-sulfur center protein PsaC

Chain C:

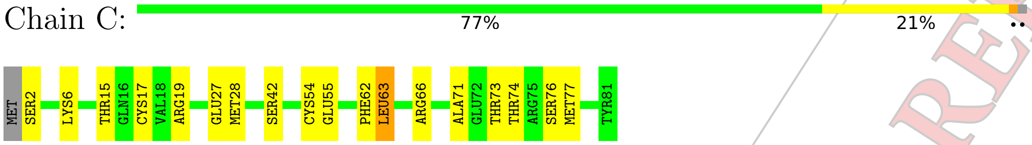

• Molecule 3: photosystem I iron-sulfur center protein PsaC

Chain N:

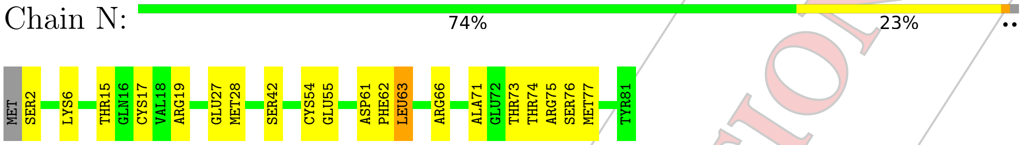

• Molecule 3: photosystem I iron-sulfur center protein PsaC

Chain c:

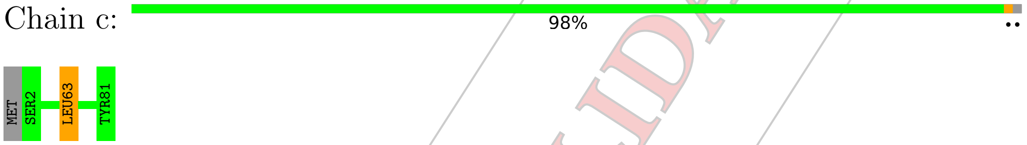

• Molecule 4: photosystem I protein PsaD

Chain D:

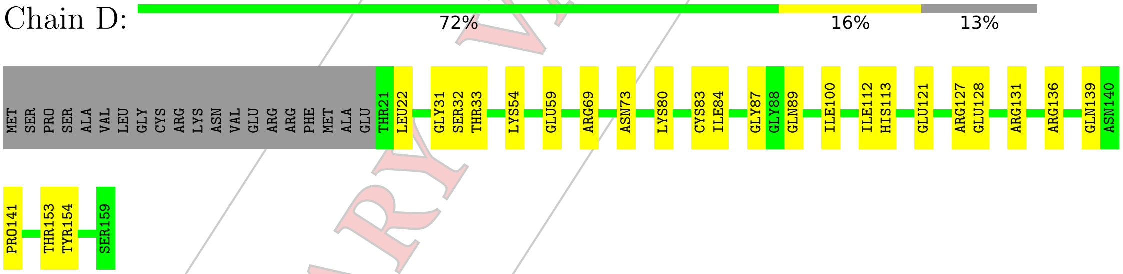

• Molecule 4: photosystem I protein PsaD

Chain O:

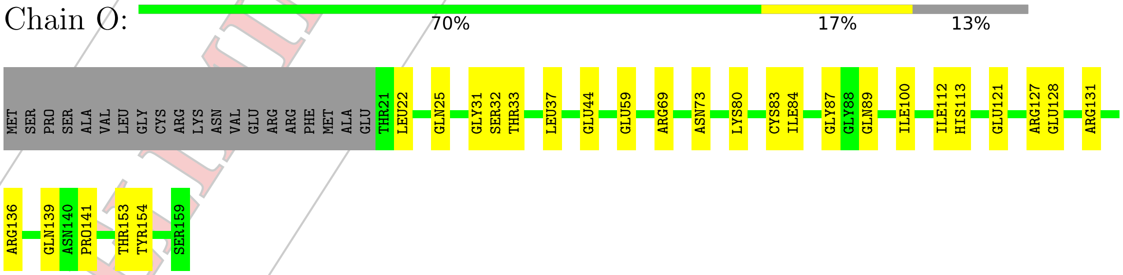

• Molecule 4: photosystem I protein PsaD

PRELIMINARY VALIDATION REPORT

Chain d:

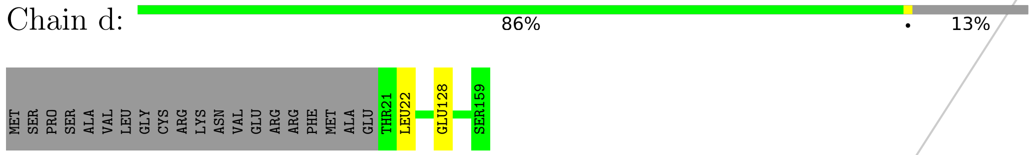

• Molecule 5: photosystem I reaction center subunit IV

Chain E:

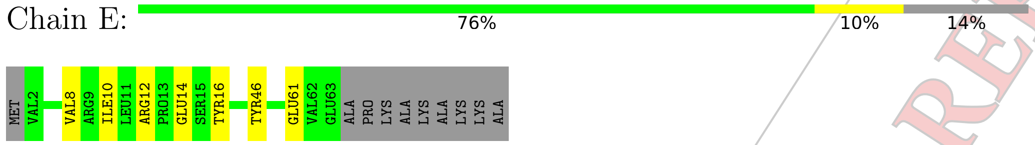

• Molecule 5: photosystem I reaction center subunit IV

Chain P:

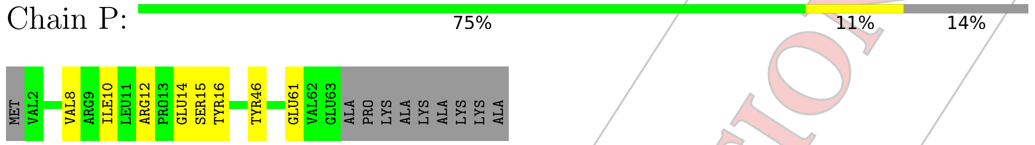

• Molecule 5: photosystem I reaction center subunit IV

Chain e:

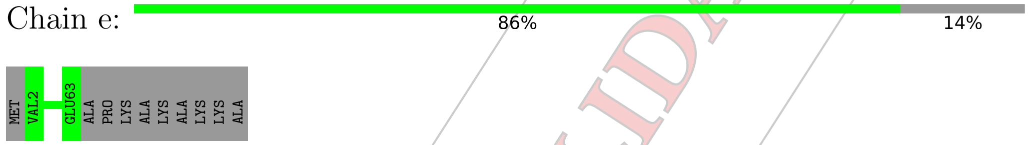

• Molecule 6: photosystem I reaction center protein PsaF subunit III

Chain F:

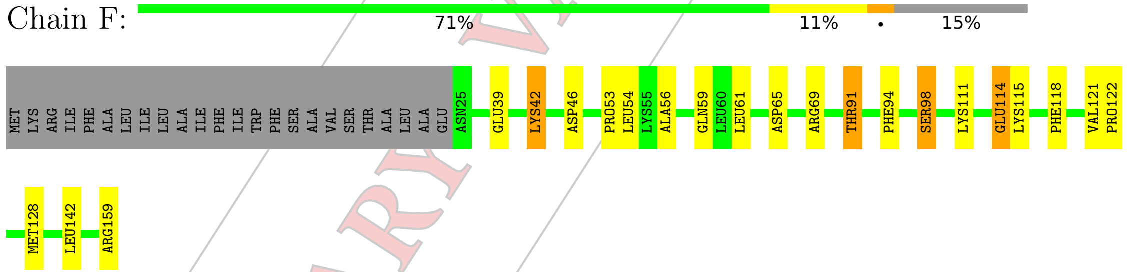

• Molecule 6: photosystem I reaction center protein PsaF subunit III

Chain Q:

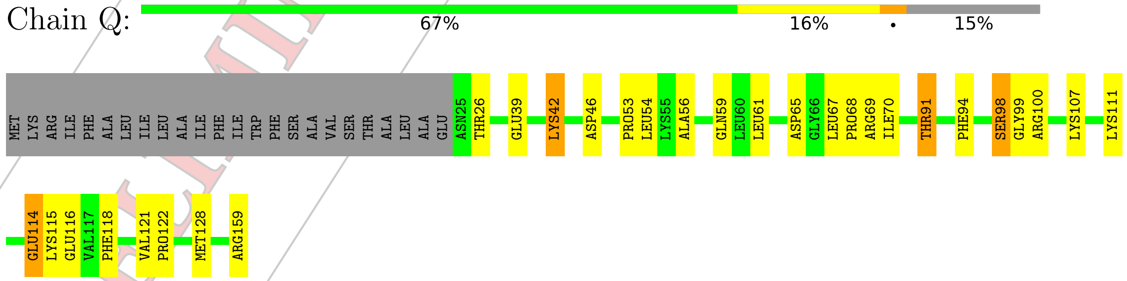

• Molecule 6: photosystem I reaction center protein PsaF subunit III

PRELIMINARY VALIDATION REPORT

Chain f:

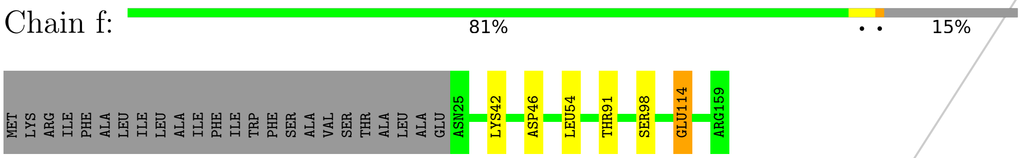

• Molecule 7: photosystem I subunit VIII

Chain I:

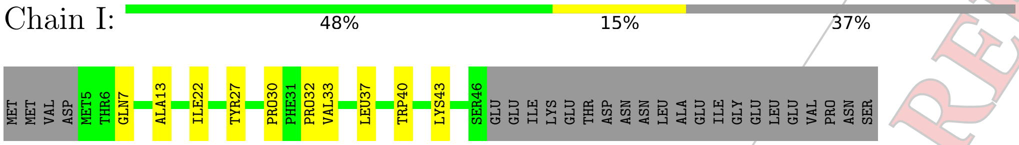

• Molecule 7: photosystem I subunit VIII

Chain R:

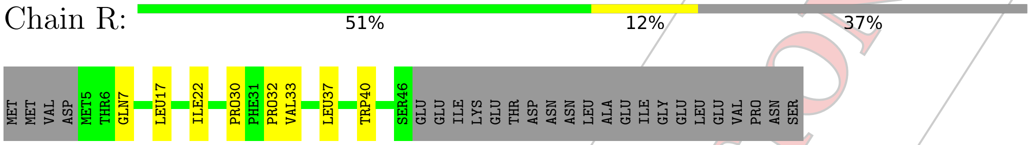

• Molecule 7: photosystem I subunit VIII

Chain i:

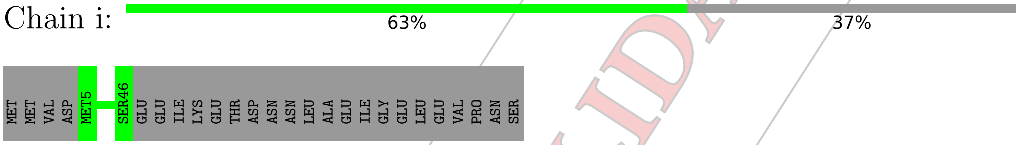

• Molecule 8: photosystem I reaction center subunit IX

Chain J:

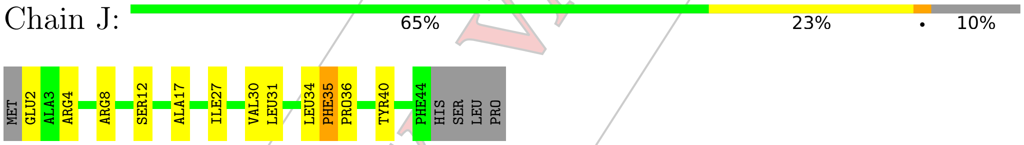

• Molecule 8: photosystem I reaction center subunit IX

Chain S:

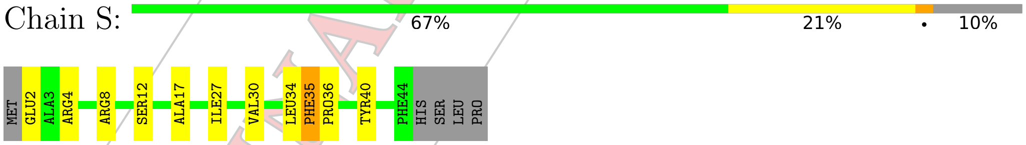

• Molecule 8: photosystem I reaction center subunit IX

Chain j:

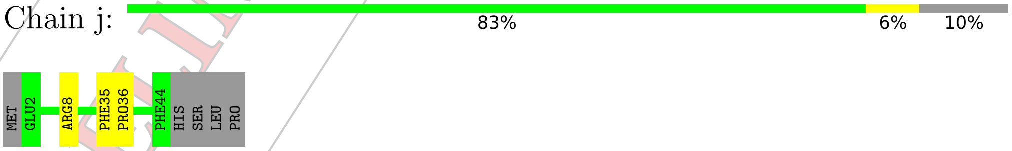

• Molecule 9: photosystem I reaction center subunit PsaK

PRELIMINARY VALIDATION REPORT

Chain K:

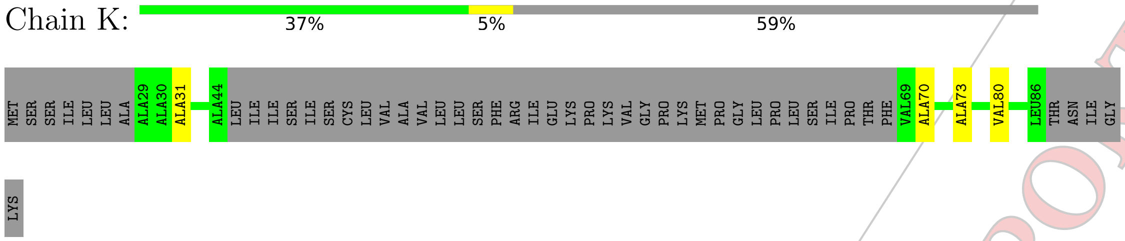

• Molecule 9: photosystem I reaction center subunit PsaK

Chain T:

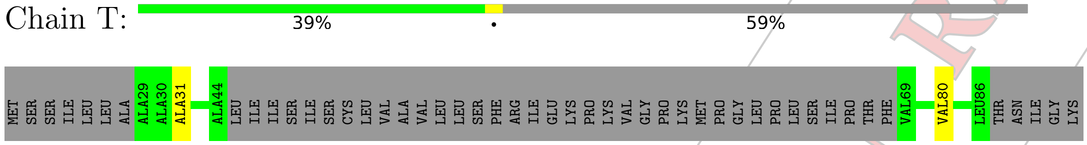

• Molecule 9: photosystem I reaction center subunit PsaK

Chain k:

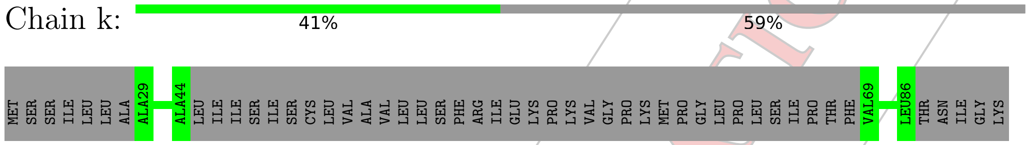

• Molecule 10: photosystem I reaction center subunit XI

Chain L:

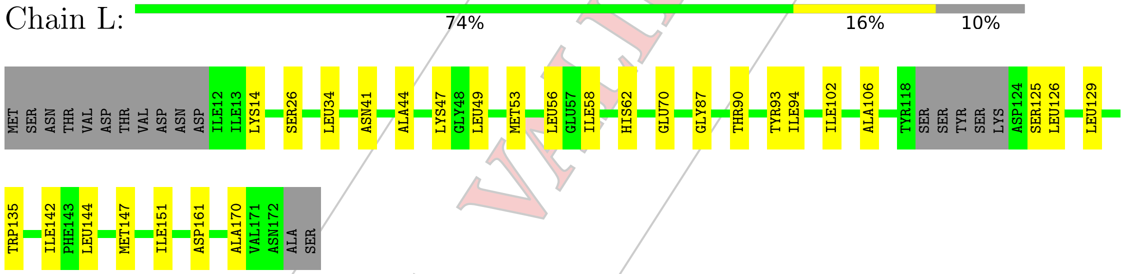

• Molecule 10: photosystem I reaction center subunit XI

Chain U:

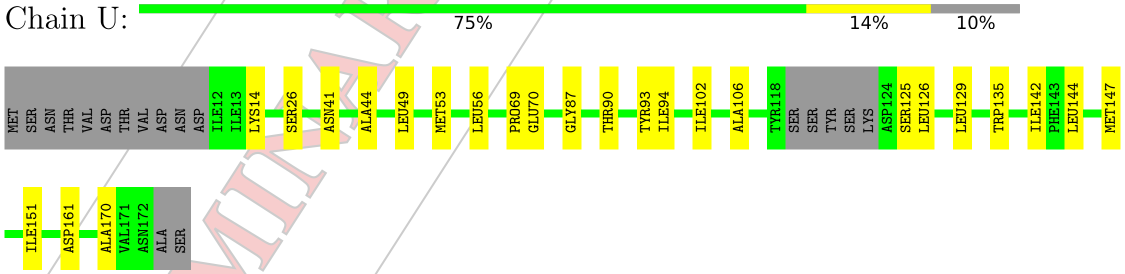

• Molecule 10: photosystem I reaction center subunit XI

Chain l:

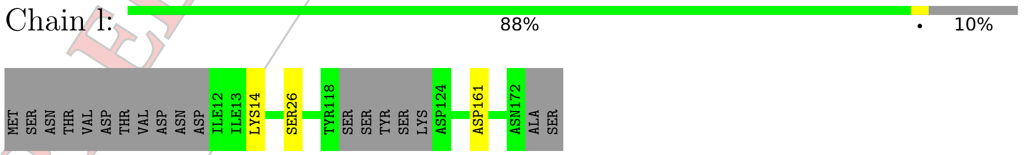

PRELIMINARY VALIDATION REPORT

• Molecule 11: photosystem I reaction center subunit XII

Chain M: 81% 19%

MET1 SER4 ASP5 ILE10 ALA11 LEU12 ALA26 LYS31

• Molecule 11: photosystem I reaction center subunit XII

Chain V: 84% 16%

MET1 SER4 ASP5 LEU12 TRP23 ALA26 LYS31

• Molecule 11: photosystem I reaction center subunit XII

Chain m: 100%

There are no outlier residues recorded for this chain.

• Molecule 12: photosystem one PsaX

Chain W: 28% • 71%

MET PRO GLY LYS GLU SER THR ALA LEU PRO ARG LEU HIS THR LEU PRO THR SER LEU PRO LEU ALA THR GLN ASN HIS GLU ASN ILE PHE ASN GLU SER GLY GLU LEU GLY HIS LEU PHE GLY ILE SER GLU GLU VAL LEU LYS LEU GLY VAL HIS PHE MET ALA LYS ALA ASP THR THR

ALA ASP LEU PRO VAL ALA LYS SER THR THR ALA LYS PRO73 TYR96 GLU101

• Molecule 12: photosystem one PsaX

Chain X: 28% • 71%

MET PRO GLY LYS GLU SER THR ALA LEU PRO ARG LEU HIS THR LEU PRO THR SER LEU PRO LEU ALA THR GLN ASN HIS GLU ASN ILE PHE ASN GLU SER GLY GLU LEU GLY HIS LEU PHE GLY ILE SER GLU GLU VAL LEU LYS LEU GLY VAL HIS PHE MET ALA LYS ALA ASP THR THR

ALA ASP LEU PRO VAL ALA LYS SER THR THR ALA LYS PRO73 TYR96 GLU101

• Molecule 12: photosystem one PsaX

Chain x: 29% 71%

MET PRO GLY LYS GLU SER THR ALA LEU PRO ARG LEU HIS THR LEU PRO THR SER LEU PRO LEU ALA THR GLN ASN HIS GLU ASN ILE PHE ASN GLU SER GLY GLU LEU GLY HIS LEU PHE GLY ILE SER GLU GLU VAL LEU LYS LEU GLY VAL HIS PHE MET ALA LYS ALA ASP THR THR

ALA ASP LEU PRO VAL ALA LYS SER THR THR ALA LYS PRO73 GLU101

PRELIMINARY VALIDATION REPORT

4 Experimental information (i)

Property	Value	Source
Reconstruction method	Not provided	Depositor
Imposed symmetry	POINT, Not provided	Depositor
Number of images used	Not provided	Depositor
Resolution determination method	Not provided	Depositor
CTF correction method	Not provided	Depositor
Microscope	Not provided	Depositor
Voltage (kV)	Not provided	Depositor
Electron dose ($e^-/\text{Å}^2$)	Not provided	Depositor
Minimum defocus (nm)	Not provided	Depositor
Maximum defocus (nm)	Not provided	Depositor
Magnification	Not provided	Depositor
Image detector	Not provided	Depositor

PRELIMINARY VALIDATION REPORT

5 Model quality (i)

5.1 Standard geometry (i)

Bond lengths and bond angles in the following residue types are not validated in this section: LHG, LMT, SF4, F6C, CLA, PQN, CL0, CA, BCR, LMG

The Z score for a bond length (or angle) is the number of standard deviations the observed value is removed from the expected value. A bond length (or angle) with $|Z| > 5$ is considered an outlier worth inspection. RMSZ is the root-mean-square of all Z scores of the bond lengths (or angles).

Mol	Chain	Bond lengths		Bond angles	
		RMSZ	#|Z| >2	RMSZ	#|Z| >2
1	A	0.47	0/6112	0.54	0/8325
1	G	0.47	0/6112	0.54	0/8325
1	a	0.47	0/6112	0.54	0/8325
10	L	0.47	0/1199	0.62	1/1624 (0.1%)
10	U	0.47	0/1199	0.62	1/1624 (0.1%)
10	l	0.47	0/1199	0.62	1/1624 (0.1%)
11	M	0.36	0/250	0.56	0/340
11	V	0.36	0/250	0.56	0/340
11	m	0.37	0/250	0.56	0/340
12	W	0.31	0/256	0.57	0/350
12	X	0.31	0/256	0.57	0/350
12	x	0.32	0/256	0.57	0/350
2	B	0.50	0/6132	0.57	1/8380 (0.0%)
2	H	0.50	0/6132	0.57	1/8380 (0.0%)
2	b	0.50	0/6132	0.57	1/8380 (0.0%)
3	C	0.48	0/611	0.68	1/828 (0.1%)
3	N	0.48	0/611	0.68	1/828 (0.1%)
3	c	0.48	0/611	0.68	1/828 (0.1%)
4	D	0.38	0/1116	0.55	0/1505
4	O	0.38	0/1116	0.55	0/1505
4	d	0.38	0/1116	0.55	0/1505
5	E	0.38	0/507	0.52	0/686
5	P	0.38	0/507	0.52	0/686
5	e	0.38	0/507	0.52	0/686
6	F	0.34	0/1084	0.57	1/1470 (0.1%)
6	Q	0.33	0/1084	0.57	1/1470 (0.1%)
6	f	0.34	0/1084	0.57	1/1470 (0.1%)
7	I	0.48	0/365	0.69	0/502
7	R	0.48	0/365	0.69	0/502
7	i	0.48	0/365	0.69	0/502
8	J	0.32	0/353	0.68	0/482
8	S	0.32	0/353	0.68	0/482

PRELIMINARY VALIDATION REPORT

Mol	Chain	Bond lengths		Bond angles	
		RMSZ	#|Z| >2	RMSZ	#|Z| >2
8	j	0.32	0/353	0.68	0/482
9	K	0.27	0/201	0.47	0/274
9	T	0.27	0/201	0.47	0/274
9	k	0.27	0/201	0.47	0/274
All	All	0.46	0/54558	0.57	12/74298 (0.0%)

Chiral center outliers are detected by calculating the chiral volume of a chiral center and verifying if the center is modelled as a planar moiety or with the opposite hand.A planarity outlier is detected by checking planarity of atoms in a peptide group, atoms in a mainchain group or atoms of a sidechain that are expected to be planar.

Mol	Chain	#Chirality outliers	#Planarity outliers
1	A	0	1
1	G	0	1
1	a	0	1
6	F	0	1
6	Q	0	1
6	f	0	1
8	J	0	1
8	S	0	1
8	j	0	1
All	All	0	9

There are no bond length outliers.

All (12) bond angle outliers are listed below:

Mol	Chain	Res	Type	Atoms	Z	Observed(^{o})	Ideal(^{o})
10	U	26	SER	C-N-CA	6.98	139.15	121.70
10	l	26	SER	C-N-CA	6.96	139.10	121.70
10	L	26	SER	C-N-CA	6.96	139.09	121.70
2	B	222	LEU	CA-CB-CG	6.25	129.67	115.30
2	b	222	LEU	CA-CB-CG	6.24	129.66	115.30
2	H	222	LEU	CA-CB-CG	6.23	129.63	115.30
6	f	114	GLU	CA-CB-CG	5.96	126.51	113.40
6	F	114	GLU	CA-CB-CG	5.95	126.48	113.40
6	Q	114	GLU	CA-CB-CG	5.93	126.45	113.40
3	N	63	LEU	CB-CG-CD1	-5.17	102.22	111.00
3	c	63	LEU	CB-CG-CD1	-5.16	102.23	111.00
3	C	63	LEU	CB-CG-CD1	-5.15	102.25	111.00

There are no chirality outliers.

PRELIMINARY VALIDATION REPORT

All (9) planarity outliers are listed below:

Mol	Chain	Res	Type	Group
1	A	333	THR	Peptide
6	F	46	ASP	Peptide
1	G	333	THR	Peptide
8	J	35	PHE	Peptide
6	Q	46	ASP	Peptide
8	S	35	PHE	Peptide
1	a	333	THR	Peptide
6	f	46	ASP	Peptide
8	j	35	PHE	Peptide

5.2 Too-close contacts (i)

In the following table, the Non-H and H(model) columns list the number of non-hydrogen atoms and hydrogen atoms in the chain respectively. The H(added) column lists the number of hydrogen atoms added and optimized by MolProbity. The Clashes column lists the number of clashes within the asymmetric unit, whereas Symm-Clashes lists symmetry related clashes.

Mol	Chain	Non-H	H(model)	H(added)	Clashes	Symm-Clashes
1	A	5906	0	5753	102	0
1	G	5906	0	5753	97	0
1	a	5906	0	5753	0	0
2	B	5910	0	5668	106	0
2	H	5910	0	5668	96	0
2	b	5910	0	5668	0	0
3	C	601	0	585	15	0
3	N	601	0	585	17	0
3	c	601	0	585	0	0
4	D	1092	0	1098	15	0
4	O	1092	0	1098	17	0
4	d	1092	0	1098	0	0
5	E	498	0	482	5	0
5	P	498	0	482	6	0
5	e	498	0	482	0	0
6	F	1060	0	1091	15	0
6	Q	1060	0	1091	21	0
6	f	1060	0	1091	0	0
7	I	350	0	354	10	0
7	R	350	0	354	6	0
7	i	350	0	354	0	0
8	J	344	0	360	10	0
8	S	344	0	360	7	0

Continued on next page...

PRELIMINARY VALIDATION REPORT

Continued from previous page...

Mol	Chain	Non-H	H(model)	H(added)	Clashes	Symm-Clashes
8	j	344	0	360	0	0
9	K	201	0	212	4	0
9	T	201	0	212	2	0
9	k	201	0	212	0	0
10	L	1173	0	1190	23	0
10	U	1173	0	1190	20	0
10	l	1173	0	1190	0	0
11	M	245	0	259	5	0
11	V	245	0	259	4	0
11	m	245	0	259	0	0
12	W	246	0	246	1	0
12	X	246	0	246	1	0
12	x	246	0	246	0	0
13	AA	65	0	72	1	0
13	GA	65	0	72	2	0
13	aA	65	0	72	0	0
14	0	65	0	72	6	0
14	AA	2140	0	1975	92	0
14	BA	2280	0	2206	115	0
14	GA	2140	0	1975	79	0
14	HA	2215	0	2134	99	0
14	KA	45	0	33	1	0
14	LA	185	0	189	14	0
14	TA	45	0	33	1	0
14	UA	185	0	189	12	0
14	WA	45	0	32	2	0
14	XA	45	0	32	1	0
14	aA	2140	0	1975	0	0
14	bA	2280	0	2206	0	0
14	kA	45	0	33	0	0
14	lA	185	0	189	0	0
14	xA	45	0	32	0	0
15	AA	92	0	0	0	0
15	BA	107	0	0	6	0
15	GA	92	0	0	0	0
15	HA	107	0	0	3	0
15	aA	92	0	0	0	0
15	bA	107	0	0	0	0
16	AA	33	0	46	7	0
16	BA	33	0	46	4	0
16	GA	33	0	46	6	0
16	HA	33	0	46	4	0

Continued on next page...

PRELIMINARY VALIDATION REPORT

Continued from previous page...

Mol	Chain	Non-H	H(model)	H(added)	Clashes	Symm-Clashes
16	aA	33	0	46	0	0
16	bA	33	0	46	0	0
17	AA	8	0	0	0	0
17	CA	16	0	0	0	0
17	GA	8	0	0	0	0
17	NA	16	0	0	0	0
17	aA	8	0	0	0	0
17	cA	16	0	0	0	0
18	AA	240	0	293	22	0
18	BA	400	0	487	30	0
18	GA	240	0	293	17	0
18	HA	400	0	487	26	0
18	IA	80	0	97	11	0
18	LA	80	0	98	8	0
18	MA	40	0	48	3	0
18	RA	80	0	97	8	0
18	UA	80	0	98	6	0
18	VA	40	0	48	4	0
18	aA	240	0	292	0	0
18	bA	400	0	487	0	0
18	iA	80	0	97	0	0
18	lA	80	0	98	0	0
18	mA	40	0	48	0	0
19	AA	42	0	54	1	0
19	BA	44	0	61	0	0
19	GA	42	0	54	1	0
19	HA	44	0	61	1	0
19	aA	42	0	54	0	0
19	bA	44	0	61	0	0
20	AA	46	0	65	1	0
20	BA	46	0	62	3	0
20	GA	46	0	65	1	0
20	HA	46	0	62	3	0
20	IA	37	0	44	4	0
20	LA	50	0	70	4	0
20	RA	37	0	44	5	0
20	UA	50	0	70	3	0
20	aA	46	0	65	0	0
20	bA	46	0	62	0	0
20	iA	37	0	44	0	0
20	lA	50	0	70	0	0
21	AA	59	0	58	0	0

Continued on next page...

PRELIMINARY VALIDATION REPORT

Continued from previous page...

Mol	Chain	Non-H	H(model)	H(added)	Clashes	Symm-Clashes
21	GA	59	0	57	1	0
21	aA	59	0	57	0	0
22	LA	1	0	0	0	0
22	UA	1	0	0	0	0
22	lA	1	0	0	0	0
All	All	71520	0	69999	878	0

The all-atom clashscore is defined as the number of clashes found per 1000 atoms (including hydrogen atoms). The all-atom clashscore for this structure is 6.

All (878) close contacts within the same asymmetric unit are listed below, sorted by their clash magnitude.

Atom-1	Atom-2	Interatomic distance (Å)	Clash overlap (Å)
3:N:2:SER:N	3:N:76:SER:HG	1.63	0.95
3:C:2:SER:N	3:C:76:SER:HG	1.66	0.94
8:J:40:TYR:OH	15:BA:1230:F6C:OMB	2.27	0.92
14:BA:1223:CLA:H2	18:BA:4010:BCR:H14C	1.66	0.78
14:HA:1223:CLA:H2	18:HA:4010:BCR:H14C	1.66	0.77
1:A:406:TRP:CD1	14:AA:1126:CLA:HAB	2.20	0.76
1:G:406:TRP:CD1	14:GA:1126:CLA:HAB	2.20	0.76
14:HA:1204:CLA:HHB	14:HA:1205:CLA:HMB3	1.70	0.74
14:BA:1204:CLA:HHB	14:BA:1205:CLA:HMB3	1.70	0.73
8:J:31:LEU:HD11	15:BA:1230:F6C:O1A	2.50	0.71
2:H:29:HIS:HD2	14:HA:1202:CLA:HAB	1.56	0.71
16:BA:2002:PQN:H301	20:BA:5002:LMG:H191	1.73	0.70
2:B:29:HIS:HD2	14:BA:1202:CLA:HAB	1.56	0.70
16:HA:2002:PQN:H301	20:HA:5002:LMG:H191	1.73	0.70
2:H:442:VAL:HG23	15:HA:1230:F6C:CMC	2.21	0.70
14:HA:1209:CLA:HBB2	14:HA:1217:CLA:H92	1.74	0.69
14:HA:1214:CLA:HBA2	14:HA:1223:CLA:HBB2	1.75	0.69
14:BA:1214:CLA:HBA2	14:BA:1223:CLA:HBB2	1.75	0.69
2:B:442:VAL:HG23	15:BA:1230:F6C:CMC	2.23	0.69
4:D:69:ARG:H	4:D:73:ASN:HD21	1.41	0.68
4:O:69:ARG:H	4:O:73:ASN:HD21	1.41	0.68
14:BA:1209:CLA:HBB2	14:BA:1217:CLA:H92	1.73	0.68
1:A:409:GLY:HA3	1:A:639:LEU:HD11	1.76	0.67
4:O:136:ARG:H	4:O:139:GLN:HE21	1.44	0.66
4:D:136:ARG:H	4:D:139:GLN:HE21	1.44	0.66
1:G:409:GLY:HA3	1:G:639:LEU:HD11	1.76	0.65
7:I:7:GLN:HG3	10:U:170:ALA:HB2	1.78	0.65
1:A:114:GLN:NE2	14:AA:1107:CLA:OBD	2.31	0.63

Continued on next page...

Continued from previous page...

Atom-1	Atom-2	Interatomic distance (Å)	Clash overlap (Å)
1:G:114:GLN:NE2	14:GA:1107:CLA:OBD	2.31	0.63
1:A:70:LYS:HZ1	14:AA:1109:CLA:HED2	1.64	0.62
1:G:651:GLN:HE22	1:G:783:ARG:HE	1.48	0.62
1:A:651:GLN:HE22	1:A:783:ARG:HE	1.48	0.62
2:B:322:PRO:HB2	2:B:410:ASN:HA	1.82	0.62
8:S:27:ILE:HA	8:S:30:VAL:HG12	1.82	0.62
2:H:468:GLN:NE2	14:HA:1234:CLA:OBD	2.33	0.62
14:BA:1021:CLA:H202	14:BA:1022:CLA:HMA1	1.83	0.61
2:H:322:PRO:HB2	2:H:410:ASN:HA	1.82	0.61
8:J:27:ILE:HA	8:J:30:VAL:HG12	1.82	0.61
2:H:442:VAL:HA	2:H:445:ASP:HB2	1.82	0.61
2:B:442:VAL:HA	2:B:445:ASP:HB2	1.82	0.61
14:AA:1127:CLA:H61	18:AA:4003:BCR:H23C	1.82	0.61
2:B:468:GLN:NE2	14:BA:1234:CLA:OBD	2.33	0.61
2:B:70:TRP:NE1	7:I:13:ALA:O	2.40	0.61
14:GA:1124:CLA:H52	14:GA:1133:CLA:HAB	1.83	0.60
1:A:626:ASP:OD2	1:A:761:ARG:NH1	2.34	0.60
14:0:1012:CLA:HMA1	14:HA:1021:CLA:H202	1.83	0.60
14:AA:1124:CLA:H52	14:AA:1133:CLA:HAB	1.84	0.60
1:G:626:ASP:OD2	1:G:761:ARG:NH1	2.34	0.60
14:GA:1127:CLA:H61	18:GA:4003:BCR:H23C	1.82	0.60
1:A:576:ALA:HB1	14:AA:1136:CLA:HMB3	1.84	0.60
6:F:53:PRO:HD3	8:J:35:PHE:HB2	1.83	0.60
1:G:269:LEU:HD21	9:T:80:VAL:HG22	1.83	0.60
1:G:70:LYS:HZ1	14:GA:1109:CLA:HED2	1.66	0.59
1:G:78:HIS:HB2	14:GA:1103:CLA:HMB2	1.84	0.59
14:BA:1216:CLA:HMB2	14:BA:1221:CLA:HMA3	1.85	0.59
7:I:7:GLN:HG3	10:L:170:ALA:HB2	37.18	0.59
20:IA:5006:LMG:HC61	20:LA:5007:LMG:H322	40.29	0.59
2:B:537:THR:HG21	14:BA:1222:CLA:HBC3	1.85	0.59
1:G:576:ALA:HB1	14:GA:1136:CLA:HMB3	1.84	0.59
8:J:17:ALA:HB1	16:AA:2001:PQN:H292	1.92	0.59
14:HA:1023:CLA:H142	18:RA:4018:BCR:H271	1.84	0.59
3:C:15:THR:HG22	3:C:28:MET:HG3	1.83	0.59
14:BA:1023:CLA:H142	18:IA:4018:BCR:H271	1.84	0.59
3:N:15:THR:HG22	3:N:28:MET:HG3	1.83	0.59
1:A:632:LEU:HB3	1:A:769:LEU:HD11	1.85	0.58
1:A:307:PHE:HE1	14:AA:1119:CLA:HAB	1.68	0.58
1:A:78:HIS:HB2	14:AA:1103:CLA:HMB2	1.84	0.58
14:HA:1216:CLA:HMB2	14:HA:1221:CLA:HMA3	1.85	0.58
10:L:170:ALA:HB2	7:R:7:GLN:HG3	36.10	0.58

Continued on next page...

PRELIMINARY VALIDATION REPORT

Continued from previous page...

Atom-1	Atom-2	Interatomic distance (Å)	Clash overlap (Å)
1:G:197:ASN:ND2	1:G:311:GLY:O	2.35	0.58
14:GA:1119:CLA:H2	14:GA:1123:CLA:HBB1	1.86	0.57
14:AA:1119:CLA:H2	14:AA:1123:CLA:HBB1	1.86	0.57
14:BA:1221:CLA:HMA2	14:BA:1221:CLA:H12	1.85	0.57
1:G:632:LEU:HB3	1:G:769:LEU:HD11	1.85	0.57
1:G:380:HIS:ND1	14:GA:1116:CLA:OBD	2.38	0.57
2:H:537:THR:HG21	14:HA:1222:CLA:HBC3	1.85	0.57
8:S:40:TYR:OH	15:HA:1230:F6C:OMB	2.11	0.57
2:B:15:ASP:HB3	2:B:20:ARG:HB2	1.87	0.57
1:A:446:ALA:O	1:A:450:HIS:ND1	2.32	0.57
1:G:751:GLN:NE2	5:P:16:TYR:OH	2.38	0.57
1:A:632:LEU:HD21	14:AA:1128:CLA:HBC1	1.87	0.57
1:G:307:PHE:HE1	14:GA:1119:CLA:HAB	1.68	0.57
1:A:445:ASP:OD2	1:A:597:ARG:NH1	2.37	0.57
14:HA:1221:CLA:H12	14:HA:1221:CLA:HMA2	1.85	0.57
1:A:608:ARG:NH1	19:AA:5001:LHG:O10	2.38	0.57
2:B:578:SER:OG	2:B:581:ASP:OD2	2.23	0.57
1:G:632:LEU:HD21	14:GA:1128:CLA:HBC1	1.87	0.56
1:A:380:HIS:ND1	14:AA:1116:CLA:OBD	2.38	0.56
1:G:126:ASN:HB3	1:G:134:HIS:HB3	1.87	0.56
10:L:41:ASN:HB3	14:LA:1501:CLA:HAC1	1.87	0.56
1:A:126:ASN:HB3	1:A:134:HIS:HB3	1.87	0.56
1:A:751:GLN:NE2	5:E:16:TYR:OH	2.39	0.56
2:B:182:LEU:O	2:B:186:SER:OG	2.22	0.56
2:H:15:ASP:HB3	2:H:20:ARG:HB2	1.87	0.56
20:IA:5006:LMG:HC61	20:UA:5007:LMG:H322	1.88	0.56
2:B:269:TRP:HB2	2:B:272:ASP:HB2	1.88	0.56
1:G:608:ARG:NH1	19:GA:5001:LHG:O10	2.38	0.56
2:H:429:VAL:HG13	14:HA:1235:CLA:HBB1	1.88	0.56
2:H:355:HIS:ND1	14:HA:1214:CLA:OBD	2.38	0.56
3:C:42:SER:O	4:D:131:ARG:NH2	2.39	0.56
2:B:429:VAL:HG13	14:BA:1235:CLA:HBB1	1.88	0.55
3:N:42:SER:O	4:O:131:ARG:NH2	2.39	0.55
4:O:100:ILE:HB	4:O:113:HIS:HB3	1.88	0.55
14:HA:1238:CLA:HAB	16:HA:2002:PQN:H141	1.88	0.55
2:B:415:VAL:HG11	18:BA:4009:BCR:H381	1.88	0.55
10:L:147:MET:HB3	18:LA:4022:BCR:H393	1.89	0.55
10:U:147:MET:HB3	18:UA:4022:BCR:H393	1.89	0.55
10:U:41:ASN:HB3	14:UA:1501:CLA:HAC1	1.87	0.55
1:A:411:LEU:HD21	14:AA:1104:CLA:H142	1.89	0.55
14:AA:1132:CLA:H172	14:LA:1502:CLA:HMB2	1.89	0.55

Continued on next page...

PRELIMINARY VALIDATION REPORT

Continued from previous page...

Atom-1	Atom-2	Interatomic distance (Å)	Clash overlap (Å)
2:H:182:LEU:O	2:H:186:SER:OG	2.22	0.55
2:H:578:SER:OG	2:H:581:ASP:OD2	2.23	0.55
1:A:279:THR:HG1	1:A:294:THR:HG1	1.51	0.55
1:A:197:ASN:ND2	1:A:311:GLY:O	2.35	0.55
1:A:583:VAL:HG11	14:AA:1137:CLA:HMB3	1.89	0.55
2:B:662:LEU:HD22	14:AA:1012:CLA:H2	1.89	0.55
2:B:68:VAL:HG21	2:B:124:TRP:HZ3	1.72	0.55
14:BA:1238:CLA:HAB	16:BA:2002:PQN:H141	1.88	0.55
3:C:55:GLU:OE2	3:C:66:ARG:NH1	2.40	0.55
2:H:68:VAL:HG21	2:H:124:TRP:HZ3	1.72	0.55
2:H:269:TRP:HB2	2:H:272:ASP:HB2	1.88	0.55
14:AA:1126:CLA:H142	14:BA:1022:CLA:H112	1.89	0.55
4:D:100:ILE:HB	4:D:113:HIS:HB3	1.89	0.55
2:H:415:VAL:HG11	18:HA:4009:BCR:H381	1.88	0.55
7:I:32:PRO:HG3	14:UA:1501:CLA:H12	1.88	0.55
1:G:583:VAL:HG11	14:GA:1137:CLA:HMB3	1.88	0.54
6:Q:94:PHE:O	6:Q:98:SER:OG	2.24	0.54
1:G:445:ASP:OD2	1:G:597:ARG:NH1	2.37	0.54
1:A:406:TRP:HD1	14:AA:1126:CLA:HAB	1.71	0.54
2:H:662:LEU:HD22	14:GA:1012:CLA:H2	1.89	0.54
14:HA:1212:CLA:HHB	18:HA:4006:BCR:HC8	1.90	0.54
2:B:498:ASN:ND2	14:BA:1232:CLA:O1D	2.41	0.54
2:B:50:HIS:HE1	14:BA:1202:CLA:H122	1.73	0.54
14:HA:1215:CLA:H102	14:HA:1225:CLA:HMD2	1.90	0.54
2:B:355:HIS:ND1	14:BA:1214:CLA:OBD	2.38	0.54
2:B:319:PHE:CD1	14:BA:1219:CLA:HAB	2.43	0.54
2:H:50:HIS:HE1	14:HA:1202:CLA:H122	1.73	0.54
6:F:94:PHE:O	6:F:98:SER:OG	2.24	0.54
2:H:319:PHE:CD1	14:HA:1219:CLA:HAB	2.43	0.54
1:A:623:SER:OG	1:A:626:ASP:OD1	2.26	0.54
2:B:86:PRO:HB2	2:B:116:CYS:HB3	1.90	0.54
1:G:411:LEU:HD21	14:GA:1104:CLA:H142	1.89	0.54
2:B:662:LEU:HG	14:BA:1239:CLA:HAB	1.91	0.53
2:H:262:HIS:HD2	2:H:264:GLN:H	1.56	0.53
2:H:701:ASP:OD1	2:H:701:ASP:N	2.41	0.53
3:N:55:GLU:OE2	3:N:66:ARG:NH1	2.40	0.53
14:BA:1212:CLA:HHB	18:BA:4006:BCR:HC8	1.90	0.53
2:H:662:LEU:HG	14:HA:1239:CLA:HAB	1.91	0.53
2:H:498:ASN:ND2	14:HA:1232:CLA:O1D	2.41	0.53
10:L:102:ILE:HB	10:L:135:TRP:HE1	1.73	0.53
14:GA:1126:CLA:H142	14:0:1012:CLA:H112	1.89	0.53

Continued on next page...

PRELIMINARY VALIDATION REPORT

Continued from previous page...

Atom-1	Atom-2	Interatomic distance (Å)	Clash overlap (Å)
14:GA:1132:CLA:H172	14:UA:1502:CLA:HMB2	1.89	0.53
2:H:420:GLU:HG3	6:Q:159:ARG:HH11	1.74	0.53
2:B:173:ALA:O	2:B:177:HIS:ND1	2.36	0.53
2:B:701:ASP:N	2:B:701:ASP:OD1	2.41	0.53
14:BA:1215:CLA:H102	14:BA:1225:CLA:HMD2	1.90	0.53
1:G:582:THR:HG21	14:GA:1124:CLA:HAC2	1.91	0.53
3:C:17:CYS:HB2	3:C:54:CYS:HB2	1.91	0.53
1:G:759:HIS:HE1	14:GA:1140:CLA:HMD1	1.74	0.53
6:F:91:THR:HA	6:F:94:PHE:HB3	1.91	0.53
1:G:623:SER:OG	1:G:626:ASP:OD1	2.26	0.53
1:A:759:HIS:HE1	14:AA:1140:CLA:HMD1	1.74	0.53
2:B:262:HIS:HD2	2:B:264:GLN:H	1.56	0.53
2:B:292:ARG:HD3	2:B:300:SER:HB2	1.91	0.53
6:Q:91:THR:HA	6:Q:94:PHE:HB3	1.91	0.53
14:BA:1225:CLA:H202	18:BA:4005:BCR:H352	1.91	0.53
8:J:31:LEU:HD11	15:BA:1230:F6C:CGA	2.66	0.52
1:G:406:TRP:HD1	14:GA:1126:CLA:HAB	1.71	0.52
2:H:86:PRO:HB2	2:H:116:CYS:HB3	1.90	0.52
7:R:32:PRO:HG3	14:LA:1501:CLA:H12	34.74	0.52
1:A:455:CYS:HB3	1:A:581:VAL:HG22	1.92	0.52
10:L:151:ILE:HG13	18:LA:4022:BCR:H291	1.92	0.52
10:U:102:ILE:HB	10:U:135:TRP:HE1	1.73	0.52
1:A:377:ILE:HD13	14:AA:1127:CLA:HHD	1.92	0.52
14:AA:1124:CLA:HAB	18:AA:4008:BCR:H311	1.91	0.52
2:B:650:LEU:HD11	2:B:737:SER:HB3	1.92	0.52
14:BA:1204:CLA:H112	14:BA:1204:CLA:HBB1	1.91	0.52
1:A:582:THR:HG21	14:AA:1124:CLA:HAC2	1.91	0.52
2:B:381:TYR:CD2	14:BA:1224:CLA:HAB	2.44	0.52
2:H:292:ARG:HD3	2:H:300:SER:HB2	1.91	0.52
3:N:17:CYS:HB2	3:N:54:CYS:HB2	1.91	0.52
18:BA:4009:BCR:H402	18:BA:4010:BCR:H383	1.92	0.52
18:BA:4009:BCR:H383	18:BA:4010:BCR:H272	1.92	0.52
1:G:446:ALA:O	1:G:450:HIS:ND1	2.32	0.52
18:HA:4009:BCR:H383	18:HA:4010:BCR:H272	1.92	0.52
2:B:56:ILE:HD11	18:MA:4021:BCR:HC7	1.90	0.52
2:B:92:TRP:HB3	7:I:22:ILE:HG13	1.99	0.52
1:G:634:TRP:HH2	14:GA:1012:CLA:HAB	1.75	0.52
14:BA:1021:CLA:H71	14:BA:1022:CLA:HED2	1.93	0.51
14:BA:1229:CLA:CAB	15:BA:1230:F6C:CMB	2.95	0.51
14:BA:1023:CLA:H201	14:BA:1239:CLA:H61	1.93	0.51
14:GA:1124:CLA:HAB	18:GA:4008:BCR:H311	1.91	0.51

Continued on next page...

PRELIMINARY VALIDATION REPORT

Continued from previous page...

Atom-1	Atom-2	Interatomic distance (Å)	Clash overlap (Å)
2:H:273:MET:O	2:H:277:HIS:ND1	2.43	0.51
2:H:650:LEU:HD11	2:H:737:SER:HB3	1.92	0.51
1:A:634:TRP:CH2	14:AA:1012:CLA:HAB	2.45	0.51
2:B:176:ASN:ND2	2:B:288:GLY:O	2.43	0.51
14:0:1012:CLA:HED2	14:HA:1021:CLA:H71	1.92	0.51
14:HA:1204:CLA:HBB1	14:HA:1204:CLA:H112	1.91	0.51
14:HA:1225:CLA:H202	18:HA:4005:BCR:H352	1.91	0.51
1:A:634:TRP:HH2	14:AA:1012:CLA:HAB	1.75	0.51
2:B:347:VAL:HG13	14:BA:1222:CLA:HED1	1.93	0.51
1:G:377:ILE:HD13	14:GA:1127:CLA:HHD	1.91	0.51
2:H:176:ASN:ND2	2:H:288:GLY:O	2.44	0.51
14:HA:1023:CLA:H201	14:HA:1239:CLA:H61	1.92	0.51
14:BA:1239:CLA:H193	18:IA:4018:BCR:H372	1.93	0.51
6:F:111:LYS:HD2	6:F:115:LYS:HE3	1.93	0.51
1:G:455:CYS:HB3	1:G:581:VAL:HG22	1.92	0.51
14:GA:1125:CLA:H172	14:GA:1133:CLA:H93	1.92	0.51
2:H:381:TYR:CD2	14:HA:1224:CLA:HAB	2.44	0.51
2:B:273:MET:O	2:B:277:HIS:ND1	2.43	0.51
1:A:699:ASN:HD22	2:B:451:GLY:HA2	1.75	0.51
2:H:347:VAL:HG13	14:HA:1222:CLA:HED1	1.93	0.51
1:G:699:ASN:HD22	2:H:451:GLY:HA2	1.74	0.51
2:H:713:LEU:HD13	20:HA:5002:LMG:H322	1.93	0.51
1:A:230:VAL:HB	1:A:234:ASP:HB3	1.93	0.51
14:AA:1125:CLA:H172	14:AA:1133:CLA:H93	1.92	0.51
14:BA:1210:CLA:H141	14:BA:1216:CLA:HBB2	1.93	0.51
1:G:634:TRP:CH2	14:GA:1012:CLA:HAB	2.45	0.51
1:G:75:HIS:ND1	14:GA:1111:CLA:OBD	2.42	0.51
14:HA:1203:CLA:H151	14:HA:1225:CLA:HBB2	1.92	0.51
10:U:151:ILE:HG13	18:UA:4022:BCR:H291	1.92	0.51
2:B:117:TYR:HA	2:B:371:THR:HG22	1.93	0.51
2:B:481:LEU:HD21	14:BA:1232:CLA:HMC3	1.94	0.50
1:G:287:VAL:HG13	1:G:288:THR:HG23	1.94	0.50
2:H:481:LEU:HD21	14:HA:1232:CLA:HMC3	1.93	0.50
6:Q:111:LYS:HD2	6:Q:115:LYS:HE3	1.93	0.50
1:A:287:VAL:HG13	1:A:288:THR:HG23	1.94	0.50
1:A:733:LEU:HD21	14:AA:1013:CLA:HED2	1.94	0.50
14:BA:1224:CLA:H202	18:IA:4018:BCR:H311	2.09	0.50
18:HA:4009:BCR:H402	18:HA:4010:BCR:H383	1.92	0.50
1:G:224:LYS:HG2	1:G:251:LEU:HD22	1.93	0.50
2:B:713:LEU:HD13	20:BA:5002:LMG:H322	1.93	0.50
14:HA:1222:CLA:H18	14:HA:1232:CLA:HMC2	1.93	0.50

Continued on next page...

PRELIMINARY VALIDATION REPORT

Continued from previous page...

Atom-1	Atom-2	Interatomic distance (Å)	Clash overlap (Å)
9:K:73:ALA:HB1	18:AA:4001:BCR:H353	2.03	0.50
14:HA:1239:CLA:H193	18:RA:4018:BCR:H372	1.93	0.50
1:A:75:HIS:ND1	14:AA:1111:CLA:OBD	2.42	0.50
14:BA:1203:CLA:H151	14:BA:1225:CLA:HBB2	1.92	0.50
1:G:230:VAL:HB	1:G:234:ASP:HB3	1.93	0.50
2:H:117:TYR:HA	2:H:371:THR:HG22	1.93	0.50
14:HA:1204:CLA:H61	18:RA:4018:BCR:HC32	1.93	0.50
14:BA:1222:CLA:H18	14:BA:1232:CLA:HMC2	1.93	0.50
14:HA:1210:CLA:H141	14:HA:1216:CLA:HBB2	1.93	0.50
4:D:80:LYS:HE3	4:D:112:ILE:HD12	1.94	0.50
4:O:80:LYS:HE3	4:O:112:ILE:HD12	1.94	0.50
1:A:224:LYS:HG2	1:A:251:LEU:HD22	1.93	0.50
2:B:566:CYS:SG	2:B:567:ASP:N	2.85	0.50
2:H:476:TYR:HB3	6:Q:26:THR:HG23	1.93	0.50
10:L:106:ALA:HB2	10:L:135:TRP:CD1	2.47	0.50
6:Q:118:PHE:HA	8:S:12:SER:HA	1.94	0.50
1:A:276:ASP:OD1	1:A:276:ASP:N	2.42	0.50
14:GA:1125:CLA:HMB3	14:GA:1133:CLA:H2	1.94	0.50
8:S:17:ALA:HB1	16:GA:2001:PQN:H292	1.93	0.50
14:AA:1125:CLA:HMB3	14:AA:1133:CLA:H2	1.94	0.49
14:BA:1022:CLA:H191	18:BA:4012:BCR:H363	1.94	0.49
20:LA:5007:LMG:H322	20:RA:5006:LMG:HC61	39.18	0.49
14:GA:1128:CLA:H122	14:GA:1140:CLA:HMA2	1.94	0.49
14:HA:1229:CLA:CAB	15:HA:1230:F6C:CMB	2.89	0.49
2:B:432:PHE:O	2:B:436:HIS:ND1	2.39	0.49
1:G:733:LEU:HD21	14:GA:1013:CLA:HED2	1.94	0.49
14:0:1012:CLA:H191	18:HA:4012:BCR:H363	1.94	0.49
1:A:201:ALA:HB2	1:A:311:GLY:HA3	1.94	0.49
18:AA:4011:BCR:H393	18:BA:4012:BCR:H321	1.94	0.49
1:A:726:GLY:HA3	2:B:575:CYS:HB2	1.94	0.49
2:H:505:LEU:HA	2:H:508:ILE:HG22	1.95	0.49
2:H:566:CYS:SG	2:H:567:ASP:N	2.85	0.49
14:HA:1210:CLA:H203	14:HA:1215:CLA:HBB2	1.95	0.49
14:BA:1204:CLA:H61	18:IA:4018:BCR:HC32	1.93	0.49
1:A:269:LEU:HD21	9:K:80:VAL:HG22	1.93	0.49
10:L:70:GLU:HB3	10:L:87:GLY:HA2	1.94	0.49
10:U:70:GLU:HB3	10:U:87:GLY:HA2	1.94	0.49
14:AA:1128:CLA:H122	14:AA:1140:CLA:HMA2	1.94	0.49
1:G:726:GLY:HA3	2:H:575:CYS:HB2	1.93	0.49
14:BA:1210:CLA:H203	14:BA:1215:CLA:HBB2	1.95	0.49
14:BA:1223:CLA:HMA3	18:BA:4010:BCR:HC41	1.95	0.49

Continued on next page...

PRELIMINARY VALIDATION REPORT

Continued from previous page...

Atom-1	Atom-2	Interatomic distance (Å)	Clash overlap (Å)
2:B:140:LEU:HD21	11:M:11:ALA:HA	1.95	0.49
3:N:2:SER:OG	3:N:71:ALA:O	2.30	0.49
2:B:403:ASP:N	2:B:403:ASP:OD2	2.46	0.49
1:A:574:ILE:HD12	13:AA:1011:CL0:H61	1.94	0.49
14:AA:1116:CLA:H3A	14:AA:1116:CLA:HBA2	1.67	0.49
2:B:505:LEU:HA	2:B:508:ILE:HG22	1.95	0.49
3:C:2:SER:OG	3:C:71:ALA:O	2.30	0.49
10:U:106:ALA:HB2	10:U:135:TRP:CD1	2.47	0.49
1:A:505:ILE:HG21	1:A:541:PRO:HA	1.95	0.49
14:BA:1205:CLA:H43	14:BA:1224:CLA:H11	1.95	0.49
3:C:6:LYS:NZ	4:D:154:TYR:O	2.46	0.49
1:G:201:ALA:HB2	1:G:311:GLY:HA3	1.94	0.49
2:B:421:ALA:O	2:B:425:HIS:ND1	2.46	0.49
2:B:53:HIS:HB2	14:BA:1202:CLA:HMB2	1.95	0.49
2:H:421:ALA:O	2:H:425:HIS:ND1	2.46	0.49
14:HA:1210:CLA:H102	14:HA:1215:CLA:H92	1.95	0.49
16:HA:2002:PQN:H141	16:HA:2002:PQN:H161	1.61	0.49
14:HA:1223:CLA:HMA3	18:HA:4010:BCR:HC41	1.95	0.48
14:BA:1022:CLA:H61	14:BA:1022:CLA:H41	1.58	0.48
14:HA:1205:CLA:H43	14:HA:1224:CLA:H11	1.95	0.48
3:N:15:THR:OG1	3:N:19:ARG:NH1	2.47	0.48
14:BA:1210:CLA:H102	14:BA:1215:CLA:H92	1.95	0.48
20:RA:5006:LMG:H361	18:VA:4021:BCR:H392	1.95	0.48
14:AA:1111:CLA:H102	18:AA:4003:BCR:H353	1.95	0.48
2:H:432:PHE:O	2:H:436:HIS:ND1	2.39	0.48
18:GA:4011:BCR:H393	18:HA:4012:BCR:H321	1.94	0.48
14:AA:1140:CLA:HAC1	16:AA:2001:PQN:H161	1.96	0.48
14:BA:1238:CLA:HAB	16:BA:2002:PQN:H161	1.95	0.48
7:I:32:PRO:HG3	14:LA:1501:CLA:H12	34.72	0.48
6:Q:91:THR:OG1	6:Q:91:THR:O	2.29	0.48
1:G:574:ILE:HD12	13:GA:1011:CL0:H61	1.94	0.48
3:N:62:PHE:CD2	5:P:14:GLU:HB2	2.49	0.48
6:F:91:THR:OG1	6:F:91:THR:O	2.29	0.48
2:H:441:TYR:O	2:H:445:ASP:N	2.46	0.48
14:AA:1103:CLA:H42	18:AA:4003:BCR:H272	1.96	0.48
18:AA:4007:BCR:H15C	18:AA:4007:BCR:H351	1.63	0.48
14:BA:1201:CLA:H62	14:BA:1201:CLA:H41	1.64	0.48
14:BA:1206:CLA:H3A	14:BA:1206:CLA:HBA2	1.53	0.48
18:BA:4009:BCR:H15C	18:BA:4009:BCR:H351	1.58	0.48
14:GA:1111:CLA:H102	18:GA:4003:BCR:H353	1.95	0.48
2:H:53:HIS:HB2	14:HA:1202:CLA:HMB2	1.95	0.48

Continued on next page...

PRELIMINARY VALIDATION REPORT

Continued from previous page...

Atom-1	Atom-2	Interatomic distance (Å)	Clash overlap (Å)
8:J:2:GLU:HG2	8:J:4:ARG:H	1.79	0.48
5:P:10:ILE:HG22	5:P:12:ARG:H	1.79	0.48
14:BA:1223:CLA:H111	14:BA:1223:CLA:H71	1.75	0.48
8:S:2:GLU:HG2	8:S:4:ARG:H	1.79	0.48
2:B:325:GLY:O	2:B:406:GLN:NE2	2.47	0.47
3:C:15:THR:OG1	3:C:19:ARG:NH1	2.47	0.47
5:E:10:ILE:HG22	5:E:12:ARG:H	1.79	0.47
14:GA:1130:CLA:H61	14:GA:1130:CLA:H41	1.70	0.47
1:A:97:SER:HB2	1:A:143:PHE:HZ	1.79	0.47
14:BA:1227:CLA:HMC1	18:BA:4009:BCR:H393	1.96	0.47
2:H:325:GLY:O	2:H:406:GLN:NE2	2.47	0.47
1:A:709:PHE:O	1:A:713:HIS:ND1	2.43	0.47
9:K:70:ALA:HB1	18:AA:4001:BCR:H343	2.05	0.47
1:G:505:ILE:HG21	1:G:541:PRO:HA	1.95	0.47
18:LA:4022:BCR:H14C	14:UA:1503:CLA:H193	1.97	0.47
2:H:403:ASP:N	2:H:403:ASP:OD2	2.46	0.47
14:HA:1202:CLA:H3A	14:HA:1202:CLA:HBA1	1.78	0.47
14:HA:1227:CLA:HMC1	18:HA:4009:BCR:H393	1.96	0.47
3:N:6:LYS:NZ	4:O:154:TYR:O	2.47	0.47
1:A:458:LEU:O	1:A:462:SER:OG	2.26	0.47
14:BA:1217:CLA:HBA2	14:BA:1217:CLA:H3A	1.66	0.47
2:H:361:PRO:HG3	14:HA:1215:CLA:HBA1	1.97	0.47
2:H:29:HIS:CD2	14:HA:1202:CLA:HAB	2.45	0.47
2:H:345:LEU:HD22	14:HA:1202:CLA:HED1	1.97	0.47
14:HA:1203:CLA:H41	14:HA:1203:CLA:H61	1.54	0.47
2:B:441:TYR:O	2:B:445:ASP:N	2.46	0.47
10:L:34:LEU:HB2	11:V:23:TRP:CH2	59.57	0.47
6:Q:53:PRO:HD3	8:S:35:PHE:HB2	1.96	0.47
14:AA:1013:CLA:HBB1	14:AA:1013:CLA:HMB1	1.96	0.47
1:G:97:SER:HB2	1:G:143:PHE:HZ	1.79	0.47
7:I:30:PRO:HA	7:I:33:VAL:HB	1.96	0.47
14:UA:1501:CLA:H143	14:UA:1503:CLA:H192	1.97	0.47
2:B:345:LEU:HD22	14:BA:1202:CLA:HED1	1.97	0.47
18:BA:4010:BCR:H371	18:BA:4010:BCR:H24C	1.67	0.47
14:HA:1229:CLA:H62	14:HA:1229:CLA:H41	1.72	0.47
18:LA:4022:BCR:H351	18:LA:4022:BCR:H15C	1.64	0.47
18:GA:4011:BCR:H14C	14:0:1012:CLA:H161	1.97	0.47
18:AA:4001:BCR:H351	18:AA:4001:BCR:H15C	1.73	0.47
2:B:44:GLN:NE2	2:B:163:PRO:O	2.47	0.47
2:B:420:GLU:HG3	6:F:159:ARG:HH11	1.80	0.47
2:B:581:ASP:OD1	2:B:712:ARG:NH1	2.48	0.47

Continued on next page...

PRELIMINARY VALIDATION REPORT

Continued from previous page...

Atom-1	Atom-2	Interatomic distance (Å)	Clash overlap (Å)
2:H:173:ALA:O	2:H:177:HIS:ND1	2.36	0.47
14:HA:1206:CLA:H3A	14:HA:1206:CLA:HBA2	1.53	0.47
7:R:30:PRO:HA	7:R:33:VAL:HB	1.96	0.47
1:A:194:SER:O	1:A:198:HIS:ND1	2.40	0.46
6:F:121:VAL:HG23	6:F:122:PRO:HD3	1.97	0.46
14:GA:1103:CLA:H42	18:GA:4003:BCR:H272	1.95	0.46
14:LA:1501:CLA:H143	14:LA:1503:CLA:H192	1.97	0.46
14:AA:1130:CLA:HMD3	18:AA:4007:BCR:H292	1.98	0.46
2:B:419:LYS:HB2	2:B:546:LEU:HD13	1.97	0.46
14:BA:1234:CLA:H52	14:BA:1234:CLA:H11	1.70	0.46
14:BA:1238:CLA:HBD	14:BA:1238:CLA:HBA1	1.98	0.46
18:BA:4005:BCR:H15C	18:BA:4005:BCR:H351	1.70	0.46
14:LA:1502:CLA:HMA1	14:LA:1503:CLA:HBC1	1.97	0.46
14:GA:1013:CLA:HMB1	14:GA:1013:CLA:HBB1	1.97	0.46
14:GA:1140:CLA:HAC1	16:GA:2001:PQN:H161	1.96	0.46
2:H:457:ILE:O	2:H:525:TYR:OH	2.25	0.46
14:HA:1211:CLA:H112	14:HA:1211:CLA:H142	1.80	0.46
14:BA:1205:CLA:H2	14:BA:1205:CLA:H61	1.72	0.46
1:G:552:ALA:HB1	1:G:658:VAL:HG21	1.98	0.46
18:GA:4002:BCR:H351	18:GA:4002:BCR:H15C	1.68	0.46
2:H:384:GLY:HA3	2:H:594:LEU:HD11	1.97	0.46
1:A:210:GLY:C	14:AA:1112:CLA:HAB	2.36	0.46
14:BA:1211:CLA:H51	14:BA:1211:CLA:H12	1.74	0.46
14:HA:1238:CLA:HAB	16:HA:2002:PQN:H161	1.95	0.46
1:A:552:ALA:HB1	1:A:658:VAL:HG21	1.98	0.46
2:B:384:GLY:HA3	2:B:594:LEU:HD11	1.97	0.46
18:AA:4011:BCR:H14C	14:BA:1022:CLA:H161	1.97	0.46
14:GA:1106:CLA:HBB1	18:HA:4012:BCR:H19C	1.98	0.46
14:UA:1502:CLA:HMA1	14:UA:1503:CLA:HBC1	1.97	0.46
18:UA:4022:BCR:H24C	18:UA:4022:BCR:H371	1.70	0.46
19:HA:5004:LHG:H361	14:WA:1701:CLA:HBC1	1.98	0.46
14:AA:1133:CLA:H62	14:AA:1133:CLA:H92	1.80	0.46
2:B:143:ILE:HD11	14:BA:1204:CLA:HMC1	1.98	0.46
18:GA:4003:BCR:H15C	18:GA:4003:BCR:H351	1.58	0.46
2:H:203:ARG:HD3	2:H:250:ALA:HB1	1.98	0.46
10:U:125:SER:OG	10:U:126:LEU:N	2.49	0.46
14:AA:1127:CLA:H12	18:AA:4003:BCR:H403	1.98	0.46
14:AA:1140:CLA:H41	14:AA:1140:CLA:H62	1.74	0.46
2:B:361:PRO:HG3	14:BA:1215:CLA:HBA1	1.97	0.46
2:B:381:TYR:HB3	14:BA:1224:CLA:HMC3	1.98	0.46
2:B:441:TYR:HD2	14:BA:1021:CLA:H203	1.81	0.46

Continued on next page...

PRELIMINARY VALIDATION REPORT

Continued from previous page...

Atom-1	Atom-2	Interatomic distance (Å)	Clash overlap (Å)
14:BA:1211:CLA:H142	14:BA:1211:CLA:H112	1.80	0.46
2:H:441:TYR:HD2	14:HA:1021:CLA:H203	1.81	0.46
2:H:583:SER:OG	2:H:583:SER:O	2.33	0.46
2:H:143:ILE:HD11	14:HA:1204:CLA:HMC1	1.98	0.46
18:RA:4018:BCR:H371	18:RA:4018:BCR:H24C	1.74	0.46
18:AA:4003:BCR:H351	18:AA:4003:BCR:H15C	1.58	0.46
1:G:210:GLY:C	14:GA:1112:CLA:HAB	2.36	0.46
1:A:176:TRP:HB2	14:AA:1109:CLA:HMC3	1.98	0.46
1:A:774:VAL:HG11	18:AA:4011:BCR:H352	1.98	0.46
2:B:203:ARG:HD3	2:B:250:ALA:HB1	1.98	0.46
14:BA:1203:CLA:H152	14:BA:1203:CLA:H112	1.66	0.46
10:L:125:SER:OG	10:L:126:LEU:N	2.49	0.46
2:B:457:ILE:O	2:B:525:TYR:OH	2.25	0.45
2:B:583:SER:O	2:B:583:SER:OG	2.33	0.45
2:B:622:TYR:OH	2:B:628:ARG:NH2	2.49	0.45
1:G:139:THR:O	1:G:399:SER:OG	2.35	0.45
14:HA:1203:CLA:H112	14:HA:1203:CLA:H152	1.66	0.45
14:HA:1225:CLA:HBA2	14:HA:1225:CLA:H3A	1.55	0.45
10:L:142:ILE:HD13	18:LA:4019:BCR:H292	1.98	0.45
6:Q:121:VAL:HG23	6:Q:122:PRO:HD3	1.97	0.45
1:A:215:LEU:HA	1:A:219:SER:HB2	1.99	0.45
14:AA:1136:CLA:H62	14:AA:1136:CLA:H41	1.61	0.45
14:BA:1203:CLA:H61	14:BA:1203:CLA:H41	1.54	0.45
6:F:61:LEU:O	6:F:69:ARG:N	2.49	0.45
1:G:451:LEU:HG	1:G:584:LEU:HB2	1.98	0.45
2:H:381:TYR:HB3	14:HA:1224:CLA:HMC3	1.98	0.45
1:A:139:THR:O	1:A:399:SER:OG	2.35	0.45
1:A:451:LEU:HG	1:A:584:LEU:HB2	1.98	0.45
14:AA:1133:CLA:HBA2	14:AA:1133:CLA:H3A	1.64	0.45
14:HA:1206:CLA:HMA1	14:HA:1207:CLA:CAB	2.46	0.45
2:H:65:LEU:HD11	18:HA:4006:BCR:H281	1.99	0.45
1:A:620:CYS:SG	2:B:674:TRP:HB3	2.57	0.45
14:BA:1206:CLA:HMA1	14:BA:1207:CLA:CAB	2.46	0.45
14:BA:1208:CLA:H12	14:BA:1208:CLA:H52	1.73	0.45
14:BA:1220:CLA:H61	14:BA:1220:CLA:H2	1.68	0.45
2:B:65:LEU:HD11	18:BA:4006:BCR:H281	1.99	0.45
6:F:56:ALA:O	6:F:59:GLN:NE2	2.50	0.45
14:GA:1127:CLA:H12	18:GA:4003:BCR:H403	1.98	0.45
14:HA:1023:CLA:H111	14:HA:1023:CLA:H143	1.85	0.45
14:HA:1220:CLA:H2	14:HA:1220:CLA:H61	1.68	0.45
2:B:589:TRP:HE1	14:AA:1013:CLA:C1D	2.30	0.45

Continued on next page...

PRELIMINARY VALIDATION REPORT

Continued from previous page...

Atom-1	Atom-2	Interatomic distance (Å)	Clash overlap (Å)
3:C:19:ARG:HG2	4:D:121:GLU:HG2	1.98	0.45
1:G:215:LEU:HA	1:G:219:SER:HB2	1.99	0.45
14:GA:1125:CLA:H112	14:GA:1125:CLA:H152	1.50	0.45
14:GA:1133:CLA:HBA2	14:GA:1133:CLA:H3A	1.64	0.45
2:H:419:LYS:HB2	2:H:546:LEU:HD13	1.97	0.45
2:H:589:TRP:HE1	14:GA:1013:CLA:C1D	2.30	0.45
2:H:581:ASP:OD1	2:H:712:ARG:NH1	2.48	0.45
14:HA:1231:CLA:H52	14:HA:1231:CLA:H12	1.72	0.45
14:HA:1238:CLA:H61	14:HA:1238:CLA:H41	1.71	0.45
10:L:56:LEU:HD11	10:U:144:LEU:HD21	8.24	0.45
6:Q:61:LEU:O	6:Q:69:ARG:N	2.50	0.45
14:BA:1208:CLA:H201	14:BA:1211:CLA:H191	1.98	0.45
18:BA:4017:BCR:H15C	18:BA:4017:BCR:H351	1.60	0.45
6:F:42:LYS:HD2	6:F:42:LYS:HA	1.58	0.45
1:G:737:ILE:O	1:G:741:HIS:ND1	2.41	0.45
14:GA:1130:CLA:HMD3	18:GA:4007:BCR:H292	1.98	0.45
1:G:684:ARG:HA	2:H:639:ILE:HD12	1.99	0.45
14:AA:1130:CLA:H61	14:AA:1130:CLA:H41	1.70	0.45
18:BA:4006:BCR:H15C	18:BA:4006:BCR:H351	1.64	0.45
1:G:176:TRP:HB2	14:GA:1109:CLA:HMC3	1.98	0.45
18:LA:4022:BCR:H371	18:LA:4022:BCR:H24C	1.70	0.45
6:Q:39:GLU:HA	6:Q:42:LYS:HB2	1.99	0.45
1:A:735:GLU:OE2	5:E:46:TYR:OH	2.27	0.45
16:AA:2001:PQN:H192	16:AA:2001:PQN:H162	1.80	0.45
2:B:554:MET:SD	3:C:66:ARG:NH2	2.89	0.45
14:HA:1208:CLA:H201	14:HA:1211:CLA:H191	1.98	0.45
3:N:61:ASP:OD2	5:P:15:SER:OG	2.28	0.45
6:Q:42:LYS:HA	6:Q:42:LYS:HD2	1.58	0.45
14:AA:1104:CLA:H71	14:AA:1104:CLA:H112	1.87	0.45
16:AA:2001:PQN:H211	16:AA:2001:PQN:H191	1.80	0.45
1:A:684:ARG:HA	2:B:639:ILE:HD12	1.99	0.45
14:BA:1238:CLA:H92	14:BA:1238:CLA:H62	1.82	0.45
14:HA:1234:CLA:H52	14:HA:1234:CLA:H11	1.70	0.45
14:HA:1238:CLA:HBD	14:HA:1238:CLA:HBA1	1.98	0.45
6:Q:56:ALA:O	6:Q:59:GLN:NE2	2.50	0.45
14:AA:1106:CLA:HBB1	18:BA:4012:BCR:H19C	1.99	0.45
14:AA:1125:CLA:H152	14:AA:1125:CLA:H112	1.50	0.45
14:LA:1503:CLA:H142	14:LA:1503:CLA:H111	1.86	0.45
14:BA:1226:CLA:H112	14:BA:1226:CLA:H142	1.82	0.44
14:BA:1229:CLA:H41	14:BA:1229:CLA:H62	1.72	0.44
1:G:774:VAL:HG11	18:GA:4011:BCR:H352	1.98	0.44

Continued on next page...

PRELIMINARY VALIDATION REPORT

Continued from previous page...

Atom-1	Atom-2	Interatomic distance (Å)	Clash overlap (Å)
14:GA:1124:CLA:H3A	14:GA:1124:CLA:HBA2	1.63	0.44
2:H:319:PHE:HD1	14:HA:1219:CLA:HAB	1.82	0.44
1:A:306:MET:HG2	14:AA:1119:CLA:HMC1	1.99	0.44
1:A:489:LEU:HB2	1:A:566:THR:HG23	1.99	0.44
1:A:722:PHE:HA	16:AA:2001:PQN:H9	2.00	0.44
14:AA:1126:CLA:H41	14:AA:1126:CLA:H62	1.81	0.44
2:H:44:GLN:NE2	2:H:163:PRO:O	2.47	0.44
11:V:4:SER:OG	11:V:5:ASP:N	2.50	0.44
14:AA:1117:CLA:HBA2	14:AA:1117:CLA:H3A	1.74	0.44
1:A:307:PHE:CE1	14:AA:1119:CLA:HAB	2.51	0.44
14:BA:1023:CLA:H143	14:BA:1023:CLA:H111	1.85	0.44
6:F:118:PHE:HA	8:J:12:SER:HA	2.02	0.44
1:G:620:CYS:SG	2:H:674:TRP:HB3	2.57	0.44
18:HA:4009:BCR:H24C	18:HA:4009:BCR:H371	1.73	0.44
10:U:142:ILE:HD13	18:UA:4019:BCR:H292	1.98	0.44
7:R:17:LEU:HD21	11:V:12:LEU:HD11	1.98	0.44
2:B:190:TRP:NE1	14:BA:1215:CLA:O1D	2.48	0.44
1:G:722:PHE:HA	16:GA:2001:PQN:H9	2.00	0.44
2:H:622:TYR:OH	2:H:628:ARG:NH2	2.49	0.44
2:H:92:TRP:HB3	7:R:22:ILE:HG13	1.99	0.44
18:IA:4020:BCR:H351	18:IA:4020:BCR:H15C	1.80	0.44
1:A:369:MET:HG3	14:AA:1123:CLA:HHB	1.99	0.44
4:D:83:CYS:O	4:D:87:GLY:N	2.47	0.44
1:G:489:LEU:HB2	1:G:566:THR:HG23	1.99	0.44
14:GA:1116:CLA:HBA2	14:GA:1116:CLA:H3A	1.67	0.44
14:GA:1136:CLA:H62	14:GA:1136:CLA:H41	1.61	0.44
2:H:328:GLU:O	2:H:332:ASN:ND2	2.51	0.44
18:LA:4019:BCR:H15C	18:LA:4019:BCR:H351	1.83	0.44
2:B:29:HIS:CD2	14:BA:1202:CLA:HAB	2.45	0.44
14:BA:1202:CLA:H3A	14:BA:1202:CLA:HBA1	1.78	0.44
14:BA:1222:CLA:HBA2	14:BA:1222:CLA:H3A	1.83	0.44
2:H:294:ASN:N	2:H:294:ASN:OD1	2.50	0.44
10:L:44:ALA:HB2	14:LA:1502:CLA:HMD1	2.00	0.44
18:AA:4011:BCR:H281	18:BA:4012:BCR:H321	1.99	0.44
16:BA:2002:PQN:H161	16:BA:2002:PQN:H141	1.61	0.44
18:BA:4014:BCR:H15C	18:BA:4014:BCR:H351	1.77	0.44
18:HA:4017:BCR:H15C	18:HA:4017:BCR:H351	1.60	0.44
11:M:4:SER:OG	11:M:5:ASP:N	2.50	0.44
1:A:610:PRO:HB3	1:A:757:ILE:HB	2.00	0.44
2:B:294:ASN:OD1	2:B:294:ASN:N	2.50	0.44
2:B:441:TYR:CD2	14:BA:1021:CLA:H203	2.53	0.44

Continued on next page...

PRELIMINARY VALIDATION REPORT

Continued from previous page...

Atom-1	Atom-2	Interatomic distance (Å)	Clash overlap (Å)
4:D:54:LYS:HB2	4:D:54:LYS:HE3	1.83	0.44
14:HA:1224:CLA:H92	14:HA:1224:CLA:H62	1.81	0.44
10:L:90:THR:HA	18:IA:4020:BCR:H332	2.00	0.44
14:BA:1210:CLA:H112	14:BA:1210:CLA:H142	1.78	0.44
18:BA:4004:BCR:H351	18:BA:4004:BCR:H15C	1.72	0.44
18:BA:4009:BCR:H24C	18:BA:4009:BCR:H371	1.73	0.44
4:D:32:SER:OG	4:D:33:THR:N	2.51	0.44
1:G:42:LYS:HD3	1:G:42:LYS:HA	1.87	0.44
1:G:451:LEU:HD21	1:G:583:VAL:HG12	1.99	0.44
18:GA:4007:BCR:H15C	18:GA:4007:BCR:H351	1.62	0.44
2:H:738:LYS:HA	2:H:738:LYS:HD2	1.86	0.44
14:LA:1503:CLA:H193	18:UA:4022:BCR:H14C	31.57	0.44
1:G:616:ARG:HA	3:N:77:MET:HA	2.00	0.44
10:U:44:ALA:HB2	14:UA:1502:CLA:HMD1	2.00	0.44
14:BA:1226:CLA:HBC2	20:BA:5002:LMG:H331	2.00	0.43
14:BA:1229:CLA:HMB1	14:BA:1229:CLA:HBB1	2.00	0.43
18:BA:4005:BCR:H371	18:BA:4005:BCR:H24C	1.77	0.43
6:F:39:GLU:HA	6:F:42:LYS:HB2	1.99	0.43
2:H:464:ALA:HA	2:H:467:ILE:HG12	2.00	0.43
4:O:83:CYS:O	4:O:87:GLY:N	2.47	0.43
14:0:1012:CLA:H41	14:0:1012:CLA:H61	1.57	0.43
1:A:451:LEU:HD21	1:A:583:VAL:HG12	1.99	0.43
14:BA:1231:CLA:H52	14:BA:1231:CLA:H12	1.72	0.43
14:BA:1238:CLA:H61	14:BA:1238:CLA:H41	1.70	0.43
14:GA:1140:CLA:H62	14:GA:1140:CLA:H41	1.74	0.43
2:H:728:GLY:HA3	14:HA:1224:CLA:H43	2.00	0.43
10:L:56:LEU:HD11	10:L:144:LEU:HD21	7.26	0.43
3:C:62:PHE:CD2	5:E:14:GLU:HB2	2.54	0.43
4:D:141:PRO:O	4:D:153:THR:OG1	2.36	0.43
1:A:770:LEU:HD22	14:AA:1140:CLA:HMA1	2.00	0.43
2:B:328:GLU:O	2:B:332:ASN:ND2	2.51	0.43
2:B:700:ARG:HB2	14:BA:1238:CLA:HED3	2.01	0.43
2:B:728:GLY:HA3	14:BA:1224:CLA:H43	2.00	0.43
2:B:319:PHE:HD1	14:BA:1219:CLA:HAB	1.82	0.43
14:BA:1219:CLA:HBC3	18:BA:4009:BCR:H323	2.00	0.43
1:G:306:MET:HG2	14:GA:1119:CLA:HMC1	1.99	0.43
1:G:140:SER:HA	14:GA:1126:CLA:HMA2	2.00	0.43
2:H:441:TYR:CD2	14:HA:1021:CLA:H203	2.53	0.43
14:HA:1208:CLA:H12	14:HA:1208:CLA:H52	1.73	0.43
14:HA:1238:CLA:H92	14:HA:1238:CLA:H62	1.82	0.43
18:GA:4011:BCR:H281	18:HA:4012:BCR:H321	2.00	0.43

Continued on next page...

PRELIMINARY VALIDATION REPORT

Continued from previous page...

Atom-1	Atom-2	Interatomic distance (Å)	Clash overlap (Å)
10:L:129:LEU:HA	10:L:129:LEU:HD23	1.92	0.43
4:O:80:LYS:HE2	4:O:84:ILE:HD11	2.01	0.43
8:S:34:LEU:HA	8:S:34:LEU:HD23	1.87	0.43
1:A:442:ARG:NH1	4:D:31:GLY:O	2.50	0.43
1:G:387:TYR:HB2	1:G:390:LEU:HB2	2.01	0.43
14:AA:1136:CLA:H3A	14:AA:1136:CLA:HBA2	1.80	0.43
3:C:66:ARG:HA	3:C:66:ARG:HD2	1.85	0.43
14:HA:1205:CLA:H2	14:HA:1205:CLA:H61	1.72	0.43
7:I:37:LEU:HD12	7:I:40:TRP:HE1	1.83	0.43
3:N:19:ARG:HG2	4:O:121:GLU:HG2	1.99	0.43
1:A:715:ILE:HB	1:A:771:GLY:HA3	2.01	0.43
20:AA:5005:LMG:H322	20:AA:5005:LMG:H291	1.78	0.43
2:B:64:ILE:HG23	14:BA:1205:CLA:HED2	2.01	0.43
14:BA:1221:CLA:H92	14:BA:1221:CLA:H61	1.83	0.43
14:BA:1222:CLA:H91	14:BA:1222:CLA:H111	1.80	0.43
2:B:538:THR:HG21	14:BA:1236:CLA:HMB3	2.01	0.43
6:F:65:ASP:OD2	6:F:69:ARG:NH1	2.52	0.43
14:GA:1106:CLA:HBA2	14:GA:1106:CLA:H3A	1.53	0.43
2:H:64:ILE:HG23	14:HA:1205:CLA:HED2	2.01	0.43
14:HA:1223:CLA:H62	14:HA:1223:CLA:H92	1.85	0.43
1:A:70:LYS:NZ	14:AA:1109:CLA:OBD	2.44	0.43
14:AA:1126:CLA:H92	14:AA:1126:CLA:H62	1.87	0.43
2:B:125:TYR:HE2	2:B:364:PHE:HE2	1.66	0.43
2:B:531:ALA:O	2:B:535:HIS:ND1	2.33	0.43
14:BA:1213:CLA:H92	14:BA:1213:CLA:H61	1.88	0.43
2:H:408:LYS:HD2	2:H:408:LYS:HA	1.85	0.43
10:L:144:LEU:HD21	10:U:56:LEU:HD11	2.01	0.43
18:RA:4020:BCR:H351	18:RA:4020:BCR:H15C	1.80	0.43
14:AA:1013:CLA:H8	14:AA:1140:CLA:HMC2	2.01	0.43
2:B:738:LYS:HD2	2:B:738:LYS:HA	1.86	0.43
14:BA:1202:CLA:HED3	14:BA:1226:CLA:HBB2	2.01	0.43
3:C:27:GLU:OE1	4:D:127:ARG:NH1	2.52	0.43
1:G:715:ILE:HB	1:G:771:GLY:HA3	2.01	0.43
20:GA:5005:LMG:H322	20:GA:5005:LMG:H291	1.79	0.43
14:HA:1229:CLA:HMB1	14:HA:1229:CLA:HBB1	2.00	0.43
18:HA:4005:BCR:H351	18:HA:4005:BCR:H15C	1.70	0.43
18:IA:4018:BCR:H24C	18:IA:4018:BCR:H371	1.74	0.43
4:O:37:LEU:HA	4:O:37:LEU:HD23	1.91	0.43
14:AA:1110:CLA:HAB	14:AA:1118:CLA:C4C	2.49	0.43
14:AA:1131:CLA:H111	14:AA:1131:CLA:H143	1.86	0.43
14:BA:1224:CLA:H92	14:BA:1224:CLA:H62	1.81	0.43

Continued on next page...

PRELIMINARY VALIDATION REPORT

Continued from previous page...

Atom-1	Atom-2	Interatomic distance (Å)	Clash overlap (Å)
14:BA:1235:CLA:H92	14:BA:1236:CLA:HAC2	2.01	0.43
1:G:734:ILE:HG22	1:G:738:VAL:HG23	2.01	0.43
14:GA:1013:CLA:H8	14:GA:1140:CLA:HMC2	2.01	0.43
14:GA:1117:CLA:H3A	14:GA:1117:CLA:HBA2	1.74	0.43
18:HA:4010:BCR:H24C	18:HA:4010:BCR:H371	1.67	0.43
11:M:26:ALA:HB2	18:MA:4021:BCR:HC41	2.01	0.43
1:G:750:ILE:HG12	6:Q:116:GLU:OE1	2.19	0.43
7:R:37:LEU:HD12	7:R:40:TRP:HE1	1.83	0.43
14:AA:1132:CLA:H61	14:AA:1132:CLA:H92	1.79	0.42
2:B:164:ASN:N	2:B:164:ASN:OD1	2.49	0.42
1:G:610:PRO:HB3	1:G:757:ILE:HB	2.00	0.42
14:GA:1012:CLA:H62	14:GA:1012:CLA:H92	1.86	0.42
2:H:125:TYR:HE2	2:H:364:PHE:HE2	1.66	0.42
14:HA:1202:CLA:H91	14:HA:1202:CLA:H112	1.87	0.42
1:G:442:ARG:NH1	4:O:31:GLY:O	2.50	0.42
14:AA:1130:CLA:H52	14:LA:1502:CLA:H12	2.01	0.42
18:AA:4008:BCR:H371	18:AA:4008:BCR:H24C	1.84	0.42
14:BA:1203:CLA:H161	14:BA:1203:CLA:H193	1.82	0.42
14:HA:1226:CLA:H203	14:HA:1226:CLA:H161	1.88	0.42
14:LA:1502:CLA:HMB3	14:LA:1503:CLA:HBC2	2.01	0.42
3:N:75:ARG:NH2	4:O:44:GLU:OE2	2.41	0.42
10:U:49:LEU:HD22	10:U:53:MET:HB3	2.01	0.42
10:U:90:THR:HA	18:RA:4020:BCR:H332	2.00	0.42
1:A:387:TYR:HB2	1:A:390:LEU:HB2	2.01	0.42
1:A:472:MET:HB2	1:A:481:MET:HB3	2.02	0.42
2:B:87:ILE:HD11	2:B:107:ALA:HB3	2.01	0.42
1:G:369:MET:HG3	14:GA:1123:CLA:HHB	1.99	0.42
14:HA:1226:CLA:HBC2	20:HA:5002:LMG:H331	2.00	0.42
2:H:700:ARG:HB2	14:HA:1238:CLA:HED3	2.01	0.42
3:N:27:GLU:OE1	4:O:127:ARG:NH1	2.52	0.42
3:N:66:ARG:HA	3:N:66:ARG:HD2	1.85	0.42
14:UA:1501:CLA:H112	14:UA:1501:CLA:H91	1.88	0.42
14:BA:1225:CLA:H3A	14:BA:1225:CLA:HBA2	1.55	0.42
1:G:770:LEU:HD22	14:GA:1140:CLA:HMA1	2.01	0.42
14:GA:1103:CLA:H112	18:GA:4003:BCR:H372	2.01	0.42
2:H:87:ILE:HD11	2:H:107:ALA:HB3	2.01	0.42
18:MA:4021:BCR:H371	18:MA:4021:BCR:H24C	1.79	0.42
12:W:96:TYR:CG	14:WA:1701:CLA:HED1	2.55	0.42
1:A:140:SER:HA	14:AA:1126:CLA:HMA2	2.00	0.42
14:AA:1131:CLA:HAB	14:AA:1132:CLA:HHB	2.02	0.42
2:B:398:TRP:HE1	18:BA:4010:BCR:H271	1.84	0.42

Continued on next page...

PRELIMINARY VALIDATION REPORT

Continued from previous page...

Atom-1	Atom-2	Interatomic distance (Å)	Clash overlap (Å)
15:BA:1230:F6C:C4C	18:BA:4012:BCR:H332	2.49	0.42
4:D:80:LYS:HE2	4:D:84:ILE:HD11	2.01	0.42
1:G:709:PHE:O	1:G:713:HIS:ND1	2.43	0.42
16:GA:2001:PQN:H261	16:GA:2001:PQN:H243	1.86	0.42
2:H:185:VAL:HG13	18:HA:4005:BCR:HC31	2.02	0.42
2:H:534:LEU:O	2:H:538:THR:OG1	2.31	0.42
14:HA:1202:CLA:HED3	14:HA:1226:CLA:HBB2	2.00	0.42
14:HA:1219:CLA:HBC3	18:HA:4009:BCR:H323	2.00	0.42
14:AA:1124:CLA:HBA2	14:AA:1124:CLA:H3A	1.63	0.42
1:A:616:ARG:HA	3:C:77:MET:HA	2.01	0.42
1:G:458:LEU:HB3	1:G:577:PHE:HB2	2.02	0.42
14:HA:1204:CLA:H141	14:HA:1204:CLA:H161	1.87	0.42
2:H:60:TRP:HB2	14:HA:1205:CLA:H192	2.01	0.42
2:H:190:TRP:NE1	14:HA:1215:CLA:O1D	2.48	0.42
14:HA:1217:CLA:HBA2	14:HA:1217:CLA:H3A	1.66	0.42
14:LA:1503:CLA:H141	14:LA:1503:CLA:H162	1.85	0.42
4:O:32:SER:OG	4:O:33:THR:N	2.51	0.42
18:UA:4022:BCR:H351	18:UA:4022:BCR:H15C	1.64	0.42
1:A:613:GLY:HA2	2:B:569:PRO:HD3	2.02	0.42
1:A:734:ILE:HG22	1:A:738:VAL:HG23	2.01	0.42
14:AA:1103:CLA:H61	14:AA:1103:CLA:H41	1.94	0.42
14:AA:1117:CLA:HBB1	14:AA:1117:CLA:HMB1	2.02	0.42
1:A:457:PHE:HZ	14:AA:1136:CLA:HMC3	1.85	0.42
2:B:185:VAL:HG13	18:BA:4005:BCR:HC31	2.02	0.42
5:E:8:VAL:HA	5:E:61:GLU:HA	2.02	0.42
1:G:279:THR:OG1	1:G:294:THR:OG1	2.29	0.42
14:GA:1110:CLA:HAB	14:GA:1118:CLA:C4C	2.49	0.42
2:H:554:MET:SD	3:N:66:ARG:NH2	2.89	0.42
12:X:96:TYR:CG	14:XA:1701:CLA:HED1	2.55	0.42
18:AA:4002:BCR:H15C	18:AA:4002:BCR:H351	1.68	0.42
14:BA:1214:CLA:H41	14:BA:1214:CLA:H62	1.87	0.42
1:G:472:MET:HB2	1:G:481:MET:HB3	2.02	0.42
1:G:82:VAL:HG11	14:GA:1103:CLA:H41	2.02	0.42
14:GA:1131:CLA:HAB	14:GA:1132:CLA:HHB	2.02	0.42
1:G:487:ILE:HD11	14:GA:1132:CLA:HED1	2.02	0.42
2:H:237:PRO:HB3	2:H:256:THR:HG21	2.02	0.42
1:G:746:VAL:HG23	6:Q:100:ARG:HA	2.02	0.42
18:VA:4021:BCR:H371	18:VA:4021:BCR:H24C	1.79	0.42
1:A:82:VAL:HG11	14:AA:1103:CLA:H41	2.02	0.42
1:A:773:ILE:HG21	14:AA:1126:CLA:HMC2	2.02	0.42
14:AA:1131:CLA:HMB2	18:IA:4020:BCR:H352	2.12	0.42

Continued on next page...

PRELIMINARY VALIDATION REPORT

Continued from previous page...

Atom-1	Atom-2	Interatomic distance (Å)	Clash overlap (Å)
14:BA:1203:CLA:H93	14:BA:1203:CLA:H112	1.85	0.42
14:GA:1117:CLA:HBB1	14:GA:1117:CLA:HMB1	2.02	0.42
1:G:199:HIS:ND1	14:GA:1123:CLA:OBD	2.36	0.42
2:H:538:THR:HG21	14:HA:1236:CLA:HMB3	2.01	0.42
14:GA:1012:CLA:CAD	14:HA:1021:CLA:HMB3	2.50	0.42
14:HA:1205:CLA:H91	14:HA:1224:CLA:H203	2.01	0.42
18:HA:4009:BCR:H15C	18:HA:4009:BCR:H351	1.58	0.42
2:H:398:TRP:HE1	18:HA:4010:BCR:H271	1.84	0.42
20:UA:5007:LMG:H342	20:UA:5007:LMG:H311	1.80	0.42
1:A:730:TRP:O	1:A:734:ILE:HG12	2.20	0.42
1:A:751:GLN:HA	1:A:752:PRO:HD3	1.94	0.42
14:AA:1103:CLA:H112	18:AA:4003:BCR:H372	2.02	0.42
2:B:464:ALA:HA	2:B:467:ILE:HG12	2.00	0.42
2:B:60:TRP:HB2	14:BA:1205:CLA:H192	2.01	0.42
14:BA:1205:CLA:H91	14:BA:1224:CLA:H203	2.01	0.42
14:GA:1103:CLA:H61	14:GA:1103:CLA:H92	1.90	0.42
14:HA:1201:CLA:H62	14:HA:1201:CLA:H41	1.64	0.42
14:HA:1215:CLA:H112	14:HA:1215:CLA:H91	1.77	0.42
10:L:94:ILE:HD11	18:IA:4020:BCR:C10	2.50	0.42
14:LA:1503:CLA:H193	18:LA:4022:BCR:H14C	32.90	0.42
6:Q:65:ASP:OD2	6:Q:69:ARG:NH1	2.52	0.42
1:A:220:LEU:HA	1:A:220:LEU:HD23	1.91	0.41
2:B:237:PRO:HB3	2:B:256:THR:HG21	2.02	0.41
14:BA:1215:CLA:H3A	14:BA:1215:CLA:HBA2	1.70	0.41
1:G:575:HIS:HE1	1:G:645:HIS:ND1	2.18	0.41
1:G:613:GLY:HA2	2:H:569:PRO:HD3	2.02	0.41
14:HA:1211:CLA:H12	14:HA:1211:CLA:H51	1.74	0.41
14:HA:1235:CLA:H91	14:HA:1235:CLA:H112	1.84	0.41
10:L:49:LEU:HD22	10:L:53:MET:HB3	2.01	0.41
4:O:141:PRO:O	4:O:153:THR:OG1	2.36	0.41
11:V:26:ALA:HB2	18:VA:4021:BCR:HC41	2.01	0.41
1:A:483:SER:HB2	1:A:486:GLY:H	1.86	0.41
1:G:483:SER:HB2	1:G:486:GLY:H	1.85	0.41
1:A:458:LEU:HB3	1:A:577:PHE:HB2	2.02	0.41
14:AA:1122:CLA:HAC1	18:AA:4007:BCR:HC8	2.03	0.41
2:B:417:LYS:HE2	2:B:417:LYS:HB2	1.88	0.41
2:B:440:LEU:HA	2:B:440:LEU:HD23	1.87	0.41
2:B:319:PHE:HA	14:BA:1219:CLA:CAB	2.50	0.41
14:BA:1239:CLA:H161	14:BA:1239:CLA:H203	1.91	0.41
1:G:248:MET:HB2	1:G:248:MET:HE2	2.00	0.41
14:GA:1130:CLA:H52	14:UA:1502:CLA:H12	2.01	0.41

Continued on next page...

PRELIMINARY VALIDATION REPORT

Continued from previous page...

Atom-1	Atom-2	Interatomic distance (Å)	Clash overlap (Å)
2:H:194:LEU:HA	2:H:198:ALA:HB3	2.02	0.41
8:J:34:LEU:HD23	8:J:34:LEU:HA	1.87	0.41
5:P:8:VAL:HA	5:P:61:GLU:HA	2.02	0.41
14:AA:1115:CLA:H62	14:AA:1115:CLA:H41	1.79	0.41
14:AA:1131:CLA:HMA1	18:IA:4018:BCR:H292	2.09	0.41
6:F:98:SER:HB3	6:F:128:MET:HA	2.03	0.41
2:H:67:HIS:ND1	2:H:71:GLN:OE1	2.49	0.41
14:HA:1235:CLA:H92	14:HA:1236:CLA:HAC2	2.01	0.41
2:H:142:LEU:HD21	18:HA:4006:BCR:H23C	2.02	0.41
10:U:94:ILE:HD11	18:RA:4020:BCR:C10	2.50	0.41
14:AA:1012:CLA:CAD	14:BA:1021:CLA:HMB3	2.50	0.41
1:A:487:ILE:HD11	14:AA:1132:CLA:HED1	2.02	0.41
1:A:757:ILE:HD11	2:B:573:GLY:HA3	2.02	0.41
14:BA:1204:CLA:H161	14:BA:1204:CLA:H141	1.87	0.41
14:BA:1215:CLA:H61	14:BA:1215:CLA:H2	1.70	0.41
10:L:94:ILE:HG23	14:BA:1238:CLA:H141	2.03	0.41
2:B:142:LEU:HD21	18:BA:4006:BCR:H23C	2.03	0.41
16:GA:2001:PQN:H192	16:GA:2001:PQN:H162	1.80	0.41
2:H:531:ALA:O	2:H:535:HIS:ND1	2.33	0.41
20:LA:5007:LMG:H342	20:LA:5007:LMG:H311	1.79	0.41
20:RA:5006:LMG:H141	20:RA:5006:LMG:H112	1.88	0.41
14:UA:1502:CLA:HMB3	14:UA:1503:CLA:HBC2	2.01	0.41
1:A:218:VAL:HG13	1:A:238:PRO:HB3	2.02	0.41
1:A:348:HIS:HB3	1:A:436:VAL:HB	2.03	0.41
1:A:575:HIS:HE1	1:A:645:HIS:ND1	2.18	0.41
1:A:302:ALA:CB	14:AA:1116:CLA:HAB	2.51	0.41
1:A:406:TRP:HB3	14:AA:1126:CLA:HMC3	2.02	0.41
18:AA:4011:BCR:H351	18:AA:4011:BCR:H15C	1.83	0.41
4:D:59:GLU:H	4:D:89:GLN:NE2	2.19	0.41
1:G:773:ILE:HG21	14:GA:1126:CLA:HMC2	2.02	0.41
14:GA:1133:CLA:H62	14:GA:1133:CLA:H92	1.80	0.41
14:HA:1208:CLA:H18	14:HA:1208:CLA:HBB1	2.03	0.41
2:H:319:PHE:HA	14:HA:1219:CLA:CAB	2.50	0.41
1:A:188:TRP:CZ2	14:AA:1108:CLA:HMA1	2.56	0.41
1:A:714:PHE:CD2	18:AA:4011:BCR:H363	2.55	0.41
2:B:4:LYS:HD3	7:I:43:LYS:HG2	2.08	0.41
2:B:343:ALA:HB2	18:BA:4010:BCR:H372	2.03	0.41
1:G:216:ILE:HA	1:G:220:LEU:HB2	2.03	0.41
1:G:730:TRP:O	1:G:734:ILE:HG12	2.20	0.41
1:G:757:ILE:HD11	2:H:573:GLY:HA3	2.02	0.41
4:O:59:GLU:H	4:O:89:GLN:NE2	2.19	0.41

Continued on next page...

PRELIMINARY VALIDATION REPORT

Continued from previous page...

Atom-1	Atom-2	Interatomic distance (Å)	Clash overlap (Å)
6:Q:98:SER:HB3	6:Q:128:MET:HA	2.03	0.41
1:A:492:VAL:H	14:AA:1136:CLA:HMD1	1.86	0.41
14:AA:1119:CLA:H161	14:AA:1119:CLA:H122	1.88	0.41
2:B:342:LEU:HD22	2:B:386:LEU:HD22	2.03	0.41
2:B:408:LYS:HD2	2:B:408:LYS:HA	1.85	0.41
6:F:142:LEU:HA	6:F:142:LEU:HD23	1.91	0.41
1:G:194:SER:O	1:G:198:HIS:ND1	2.40	0.41
1:G:588:LYS:HE3	2:H:681:LEU:HB2	2.03	0.41
1:G:695:ILE:HD13	1:G:695:ILE:HA	1.95	0.41
1:G:457:PHE:HZ	14:GA:1136:CLA:HMC3	1.85	0.41
2:H:440:LEU:HA	2:H:440:LEU:HD23	1.87	0.41
14:HA:1223:CLA:H111	14:HA:1223:CLA:H71	1.75	0.41
9:K:31:ALA:HB2	14:KA:1401:CLA:HED3	2.03	0.41
10:L:58:ILE:O	10:L:62:HIS:ND1	2.54	0.41
6:Q:67:LEU:HA	6:Q:68:PRO:HD3	1.95	0.41
1:A:568:ASP:HA	1:A:571:ILE:HG22	2.03	0.41
2:B:431:LEU:HD22	14:AA:1013:CLA:HBA2	2.03	0.41
14:AA:1106:CLA:H3A	14:AA:1106:CLA:HBA2	1.53	0.41
14:AA:1124:CLA:H12	14:AA:1136:CLA:HBA1	2.03	0.41
2:B:183:PHE:HB3	2:B:284:LEU:HD13	2.03	0.41
7:I:27:TYR:HE1	14:BA:1207:CLA:HMB3	1.95	0.41
14:BA:1238:CLA:H8	14:BA:1239:CLA:H152	2.03	0.41
3:C:73:THR:OG1	3:C:74:THR:N	2.54	0.41
1:G:568:ASP:HA	1:G:571:ILE:HG22	2.03	0.41
14:GA:1103:CLA:H41	14:GA:1103:CLA:H61	1.94	0.41
18:HA:4004:BCR:H351	18:HA:4004:BCR:H15C	1.72	0.41
10:L:47:LYS:HA	10:L:47:LYS:HD3	1.92	0.41
6:Q:99:GLY:HA3	14:GA:1138:CLA:HBB2	2.03	0.41
9:T:31:ALA:HB2	14:TA:1401:CLA:HED3	2.03	0.41
10:U:129:LEU:HA	10:U:129:LEU:HD23	1.92	0.41
1:A:235:ILE:HA	1:A:236:PRO:HD3	1.92	0.41
14:AA:1140:CLA:H93	14:AA:1140:CLA:H61	1.91	0.41
1:A:614:PRO:HD3	2:B:568:GLY:HA2	2.03	0.41
14:BA:1210:CLA:H202	14:BA:1210:CLA:H162	1.89	0.41
2:B:50:HIS:ND1	14:BA:1210:CLA:OBD	2.49	0.41
14:BA:1215:CLA:HMB1	14:BA:1215:CLA:HBB1	2.03	0.41
1:G:188:TRP:CZ2	14:GA:1108:CLA:HMA1	2.56	0.41
1:G:279:THR:HG21	1:G:281:LYS:HE3	2.03	0.41
1:G:302:ALA:CB	14:GA:1116:CLA:HAB	2.51	0.41
14:GA:1111:CLA:H11	14:GA:1111:CLA:H51	1.88	0.41
18:GA:4011:BCR:H351	18:GA:4011:BCR:H15C	1.83	0.41

Continued on next page...

PRELIMINARY VALIDATION REPORT

Continued from previous page...

Atom-1	Atom-2	Interatomic distance (Å)	Clash overlap (Å)
18:HA:4016:BCR:H371	18:HA:4016:BCR:H24C	1.84	0.41
11:M:12:LEU:HA	14:BA:1204:CLA:HBC1	2.09	0.41
2:H:459:ILE:HD13	6:Q:70:ILE:HD12	2.03	0.41
20:IA:5006:LMG:H111	20:UA:5007:LMG:H162	2.03	0.41
2:B:559:ASP:OD2	2:B:559:ASP:N	2.55	0.41
18:BA:4014:BCR:H371	18:BA:4014:BCR:H24C	1.75	0.41
1:G:218:VAL:HG13	1:G:238:PRO:HB3	2.03	0.41
1:G:348:HIS:HB3	1:G:436:VAL:HB	2.03	0.41
1:G:747:ALA:O	6:Q:107:LYS:NZ	2.38	0.41
14:HA:1226:CLA:H142	14:HA:1226:CLA:H112	1.82	0.41
20:IA:5006:LMG:H112	20:IA:5006:LMG:H141	1.88	0.41
11:M:10:ILE:HD11	21:GA:6001:LMT:H72	2.03	0.41
1:A:42:LYS:HD3	1:A:42:LYS:HA	1.87	0.40
2:B:194:LEU:HA	2:B:198:ALA:HB3	2.02	0.40
13:GA:1011:CL0:H50	13:GA:1011:CL0:H59	1.67	0.40
1:G:211:TRP:NE1	14:GA:1117:CLA:O1D	2.53	0.40
14:GA:1140:CLA:H102	16:GA:2001:PQN:H202	2.03	0.40
14:HA:1224:CLA:H11	14:HA:1224:CLA:H52	1.87	0.40
2:H:663:ALA:HB2	14:HA:1239:CLA:HMB3	2.03	0.40
18:HA:4010:BCR:H281	18:HA:4010:BCR:H393	1.92	0.40
8:J:30:VAL:HG22	8:J:34:LEU:HD12	2.03	0.40
14:LA:1501:CLA:H91	14:LA:1501:CLA:H112	1.88	0.40
3:N:73:THR:OG1	3:N:74:THR:N	2.54	0.40
4:O:25:GLN:HG2	4:O:25:GLN:H	1.74	0.40
10:U:94:ILE:HG23	14:HA:1238:CLA:H141	2.02	0.40
1:A:658:VAL:HG12	1:A:664:VAL:HG22	2.03	0.40
2:B:181:GLY:HA3	14:BA:1210:CLA:HBB1	2.04	0.40
1:G:714:PHE:CD2	18:GA:4011:BCR:H363	2.55	0.40
14:GA:1104:CLA:H71	14:GA:1104:CLA:H112	1.87	0.40
18:HA:4005:BCR:H24C	18:HA:4005:BCR:H371	1.77	0.40
10:L:93:TYR:CG	18:IA:4020:BCR:HC41	2.56	0.40
20:RA:5006:LMG:H341	18:VA:4021:BCR:H391	2.02	0.40
10:U:93:TYR:CG	18:RA:4020:BCR:HC41	2.56	0.40
1:A:248:MET:HE2	1:A:248:MET:HB2	1.98	0.40
1:A:279:THR:OG1	1:A:294:THR:OG1	2.29	0.40
14:AA:1117:CLA:H193	14:AA:1117:CLA:H102	2.03	0.40
14:AA:1139:CLA:HMC2	18:BA:4014:BCR:H381	2.03	0.40
14:AA:1140:CLA:H102	16:AA:2001:PQN:H202	2.03	0.40
14:BA:1021:CLA:H202	14:BA:1021:CLA:H161	1.88	0.40
14:BA:1214:CLA:H12	14:BA:1214:CLA:H52	1.88	0.40
1:G:735:GLU:OE2	5:P:46:TYR:OH	2.31	0.40

Continued on next page...

PRELIMINARY VALIDATION REPORT

Continued from previous page...

Atom-1	Atom-2	Interatomic distance (Å)	Clash overlap (Å)
1:G:307:PHE:CE1	14:GA:1119:CLA:HAB	2.51	0.40
14:GA:1122:CLA:HAC1	18:GA:4007:BCR:HC8	2.02	0.40
14:HA:1214:CLA:H12	14:HA:1214:CLA:HBA1	1.87	0.40
20:LA:5007:LMG:H162	20:RA:5006:LMG:H111	42.14	0.40
1:A:588:LYS:HE3	2:B:681:LEU:HB2	2.03	0.40
1:A:626:ASP:HA	1:A:629:PHE:HB3	2.04	0.40
14:AA:1102:CLA:H12	14:AA:1102:CLA:H52	1.83	0.40
1:A:199:HIS:ND1	14:AA:1123:CLA:OBD	2.36	0.40
16:AA:2001:PQN:H243	16:AA:2001:PQN:H261	1.86	0.40
2:B:300:SER:HB3	2:B:303:GLU:HG3	2.03	0.40
14:BA:1208:CLA:H18	14:BA:1208:CLA:HBB1	2.03	0.40
14:BA:1231:CLA:HBB1	14:BA:1231:CLA:HMB1	2.04	0.40
14:GA:1117:CLA:H193	14:GA:1117:CLA:H102	2.03	0.40
14:UA:1501:CLA:HBA1	14:UA:1501:CLA:HBD	2.04	0.40
1:A:279:THR:HG21	1:A:281:LYS:HE3	2.03	0.40
1:A:423:MET:HA	1:A:427:TYR:HB2	2.04	0.40
14:AA:1102:CLA:HMA2	14:AA:1109:CLA:HMD2	2.04	0.40
14:AA:1104:CLA:H161	14:AA:1104:CLA:H193	1.92	0.40
14:AA:1119:CLA:H193	18:AA:4007:BCR:HC41	2.04	0.40
2:B:140:LEU:HA	2:B:140:LEU:HD23	1.90	0.40
2:B:519:ILE:HA	2:B:519:ILE:HD13	1.94	0.40
1:G:658:VAL:HG12	1:G:664:VAL:HG22	2.04	0.40
14:GA:1102:CLA:H52	14:GA:1102:CLA:H12	1.83	0.40
14:GA:1124:CLA:H12	14:GA:1136:CLA:HBA1	2.03	0.40
2:H:300:SER:HB3	2:H:303:GLU:HG3	2.03	0.40
2:H:469:SER:O	2:H:483:SER:OG	2.30	0.40
14:HA:1215:CLA:HMB1	14:HA:1215:CLA:HBB1	2.03	0.40
14:HA:1222:CLA:HBA2	14:HA:1222:CLA:H3A	1.83	0.40
10:U:41:ASN:HA	10:U:41:ASN:HD22	1.70	0.40
10:U:69:PRO:HB3	14:UA:1503:CLA:HBB1	2.04	0.40

There are no symmetry-related clashes.

5.3 Torsion angles (i)

5.3.1 Protein backbone (i)

In the following table, the Percentiles column shows the percent Ramachandran outliers of the chain as a percentile score with respect to all PDB entries followed by that with respect to all EM entries.

PRELIMINARY VALIDATION REPORT

The Analysed column shows the number of residues for which the backbone conformation was analysed, and the total number of residues.

Mol	Chain	Analysed	Favoured	Allowed	Outliers	Percentiles	
1	A	743/788 (94%)	699 (94%)	44 (6%)	0	100	100
1	G	743/788 (94%)	700 (94%)	43 (6%)	0	100	100
1	a	743/788 (94%)	699 (94%)	44 (6%)	0	100	100
2	B	736/741 (99%)	706 (96%)	30 (4%)	0	100	100
2	H	736/741 (99%)	706 (96%)	30 (4%)	0	100	100
2	b	736/741 (99%)	706 (96%)	30 (4%)	0	100	100
3	C	78/81 (96%)	72 (92%)	5 (6%)	1 (1%)	13	52
3	N	78/81 (96%)	72 (92%)	5 (6%)	1 (1%)	13	52
3	c	78/81 (96%)	72 (92%)	5 (6%)	1 (1%)	13	52
4	D	137/159 (86%)	125 (91%)	12 (9%)	0	100	100
4	O	137/159 (86%)	125 (91%)	12 (9%)	0	100	100
4	d	137/159 (86%)	125 (91%)	12 (9%)	0	100	100
5	E	60/72 (83%)	54 (90%)	6 (10%)	0	100	100
5	P	60/72 (83%)	54 (90%)	6 (10%)	0	100	100
5	e	60/72 (83%)	54 (90%)	6 (10%)	0	100	100
6	F	133/159 (84%)	122 (92%)	11 (8%)	0	100	100
6	Q	133/159 (84%)	122 (92%)	11 (8%)	0	100	100
6	f	133/159 (84%)	122 (92%)	11 (8%)	0	100	100
7	I	40/67 (60%)	33 (82%)	7 (18%)	0	100	100
7	R	40/67 (60%)	33 (82%)	7 (18%)	0	100	100
7	i	40/67 (60%)	33 (82%)	7 (18%)	0	100	100
8	J	41/48 (85%)	36 (88%)	4 (10%)	1 (2%)	6	37
8	S	41/48 (85%)	36 (88%)	4 (10%)	1 (2%)	6	37
8	j	41/48 (85%)	36 (88%)	4 (10%)	1 (2%)	6	37
9	K	30/82 (37%)	29 (97%)	1 (3%)	0	100	100
9	T	30/82 (37%)	29 (97%)	1 (3%)	0	100	100
9	k	30/82 (37%)	29 (97%)	1 (3%)	0	100	100
10	L	152/174 (87%)	140 (92%)	12 (8%)	0	100	100
10	U	152/174 (87%)	140 (92%)	12 (8%)	0	100	100
10	l	152/174 (87%)	140 (92%)	12 (8%)	0	100	100

Continued on next page...

PRELIMINARY VALIDATION REPORT

Continued from previous page...

Mol	Chain	Analysed	Favoured	Allowed	Outliers	Percentiles	
11	M	29/31 (94%)	27 (93%)	2 (7%)	0	100	100
11	V	29/31 (94%)	27 (93%)	2 (7%)	0	100	100
11	m	29/31 (94%)	27 (93%)	2 (7%)	0	100	100
12	W	27/101 (27%)	25 (93%)	2 (7%)	0	100	100
12	X	27/101 (27%)	25 (93%)	2 (7%)	0	100	100
12	x	27/101 (27%)	25 (93%)	2 (7%)	0	100	100
All	All	6618/7509 (88%)	6205 (94%)	407 (6%)	6 (0%)	56	87

All (6) Ramachandran outliers are listed below:

Mol	Chain	Res	Type
3	C	63	LEU
3	N	63	LEU
3	c	63	LEU
8	J	36	PRO
8	S	36	PRO
8	j	36	PRO

5.3.2 Protein sidechains (i)

In the following table, the Percentiles column shows the percent sidechain outliers of the chain as a percentile score with respect to all PDB entries followed by that with respect to all EM entries.

The Analysed column shows the number of residues for which the sidechain conformation was analysed, and the total number of residues.

Mol	Chain	Analysed	Rotameric	Outliers	Percentiles	
1	A	611/641 (95%)	607 (99%)	4 (1%)	85	95
1	G	611/641 (95%)	607 (99%)	4 (1%)	85	95
1	a	611/641 (95%)	607 (99%)	4 (1%)	85	95
2	B	595/597 (100%)	590 (99%)	5 (1%)	83	94
2	H	595/597 (100%)	590 (99%)	5 (1%)	83	94
2	b	595/597 (100%)	590 (99%)	5 (1%)	83	94
3	C	68/69 (99%)	68 (100%)	0	100	100
3	N	68/69 (99%)	68 (100%)	0	100	100
3	c	68/69 (99%)	68 (100%)	0	100	100

Continued on next page...

PRELIMINARY VALIDATION REPORT

Continued from previous page...

Mol	Chain	Analysed	Rotameric	Outliers	Percentiles	
4	D	115/132 (87%)	113 (98%)	2 (2%)	63	86
4	O	115/132 (87%)	113 (98%)	2 (2%)	63	86
4	d	115/132 (87%)	113 (98%)	2 (2%)	63	86
5	E	54/60 (90%)	54 (100%)	0	100	100
5	P	54/60 (90%)	54 (100%)	0	100	100
5	e	54/60 (90%)	54 (100%)	0	100	100
6	F	115/134 (86%)	110 (96%)	5 (4%)	32	68
6	Q	115/134 (86%)	110 (96%)	5 (4%)	32	68
6	f	115/134 (86%)	110 (96%)	5 (4%)	32	68
7	I	37/60 (62%)	37 (100%)	0	100	100
7	R	37/60 (62%)	37 (100%)	0	100	100
7	i	37/60 (62%)	37 (100%)	0	100	100
8	J	36/42 (86%)	35 (97%)	1 (3%)	47	77
8	S	36/42 (86%)	35 (97%)	1 (3%)	47	77
8	j	36/42 (86%)	35 (97%)	1 (3%)	47	77
9	K	11/65 (17%)	11 (100%)	0	100	100
9	T	11/65 (17%)	11 (100%)	0	100	100
9	k	11/65 (17%)	11 (100%)	0	100	100
10	L	121/138 (88%)	119 (98%)	2 (2%)	63	86
10	U	121/138 (88%)	119 (98%)	2 (2%)	63	86
10	l	121/138 (88%)	119 (98%)	2 (2%)	63	86
11	M	26/26 (100%)	26 (100%)	0	100	100
11	V	26/26 (100%)	26 (100%)	0	100	100
11	m	26/26 (100%)	26 (100%)	0	100	100
12	W	24/84 (29%)	24 (100%)	0	100	100
12	X	24/84 (29%)	24 (100%)	0	100	100
12	x	24/84 (29%)	24 (100%)	0	100	100
All	All	5439/6144 (88%)	5382 (99%)	57 (1%)	80	92

All (57) residues with a non-rotameric sidechain are listed below:

Mol	Chain	Res	Type
1	A	12	ARG

Continued on next page...

PRELIMINARY VALIDATION REPORT

Continued from previous page...

Mol	Chain	Res	Type
1	A	233	LYS
1	A	436	VAL
1	A	484	ASP
2	B	110	ARG
2	B	248	GLN
2	B	309	THR
2	B	559	ASP
2	B	575	CYS
4	D	22	LEU
4	D	128	GLU
6	F	42	LYS
6	F	54	LEU
6	F	91	THR
6	F	98	SER
6	F	114	GLU
1	G	12	ARG
1	G	233	LYS
1	G	436	VAL
1	G	484	ASP
2	H	110	ARG
2	H	248	GLN
2	H	309	THR
2	H	559	ASP
2	H	575	CYS
8	J	8	ARG
10	L	14	LYS
10	L	161	ASP
4	O	22	LEU
4	O	128	GLU
6	Q	42	LYS
6	Q	54	LEU
6	Q	91	THR
6	Q	98	SER
6	Q	114	GLU
8	S	8	ARG
10	U	14	LYS
10	U	161	ASP
1	a	12	ARG
1	a	233	LYS
1	a	436	VAL
1	a	484	ASP
2	b	110	ARG

Continued on next page...

PRELIMINARY VALIDATION REPORT

Continued from previous page...

Mol	Chain	Res	Type
2	b	248	GLN
2	b	309	THR
2	b	559	ASP
2	b	575	CYS
4	d	22	LEU
4	d	128	GLU
6	f	42	LYS
6	f	54	LEU
6	f	91	THR
6	f	98	SER
6	f	114	GLU
8	j	8	ARG
10	l	14	LYS
10	l	161	ASP

Some sidechains can be flipped to improve hydrogen bonding and reduce clashes. All (87) such sidechains are listed below:

Mol	Chain	Res	Type
1	A	49	ASN
1	A	53	ASN
1	A	217	HIS
1	A	223	ASN
1	A	360	HIS
1	A	362	GLN
1	A	379	HIS
1	A	432	ASN
1	A	575	HIS
1	A	651	GLN
1	A	680	ASN
1	A	699	ASN
1	A	751	GLN
2	B	176	ASN
2	B	262	HIS
2	B	332	ASN
2	B	354	GLN
2	B	380	GLN
2	B	510	ASN
2	B	661	HIS
2	B	679	GLN
2	B	691	GLN
4	D	73	ASN
4	D	89	GLN

Continued on next page...

PRELIMINARY VALIDATION REPORT

Continued from previous page...

Mol	Chain	Res	Type
4	D	139	GLN
6	F	109	GLN
1	G	49	ASN
1	G	53	ASN
1	G	217	HIS
1	G	223	ASN
1	G	360	HIS
1	G	362	GLN
1	G	379	HIS
1	G	432	ASN
1	G	575	HIS
1	G	651	GLN
1	G	680	ASN
1	G	699	ASN
1	G	751	GLN
2	H	176	ASN
2	H	262	HIS
2	H	332	ASN
2	H	354	GLN
2	H	380	GLN
2	H	510	ASN
2	H	661	HIS
2	H	679	GLN
2	H	691	GLN
10	L	41	ASN
11	M	7	GLN
4	O	73	ASN
4	O	89	GLN
4	O	139	GLN
6	Q	104	GLN
6	Q	109	GLN
10	U	41	ASN
11	V	7	GLN
12	W	89	ASN
12	X	89	ASN
1	a	49	ASN
1	a	53	ASN
1	a	217	HIS
1	a	223	ASN
1	a	360	HIS
1	a	362	GLN
1	a	379	HIS

Continued on next page...

PRELIMINARY VALIDATION REPORT

Continued from previous page...

Mol	Chain	Res	Type
1	a	432	ASN
1	a	575	HIS
1	a	651	GLN
1	a	680	ASN
1	a	699	ASN
1	a	751	GLN
2	b	176	ASN
2	b	262	HIS
2	b	332	ASN
2	b	354	GLN
2	b	380	GLN
2	b	510	ASN
2	b	661	HIS
2	b	679	GLN
2	b	691	GLN
4	d	73	ASN
4	d	89	GLN
4	d	139	GLN
6	f	109	GLN
10	l	41	ASN
11	m	7	GLN

5.3.3 RNA (i)

There are no RNA molecules in this entry.

5.4 Non-standard residues in protein, DNA, RNA chains (i)

There are no non-standard protein/DNA/RNA residues in this entry.

5.5 Carbohydrates (i)

There are no carbohydrates in this entry.

5.6 Ligand geometry (i)

Of 372 ligands modelled in this entry, 12 could not be matched to an existing wwPDB Chemical Component Dictionary definition at this stage and 3 are monoatomic - leaving 357 for Mogul analysis.

PRELIMINARY VALIDATION REPORT

In the following table, the Counts columns list the number of bonds (or angles) for which Mogul statistics could be retrieved, the number of bonds (or angles) that are observed in the model and the number of bonds (or angles) that are defined in the Chemical Component Dictionary. The Link column lists molecule types, if any, to which the group is linked. The Z score for a bond length (or angle) is the number of standard deviations the observed value is removed from the expected value. A bond length (or angle) with $|Z| > 2$ is considered an outlier worth inspection. RMSZ is the root-mean-square of all Z scores of the bond lengths (or angles).

Mol	Type	Chain	Res	Link	Bond lengths			Bond angles		
					Counts	RMSZ	$\#\|Z\| > 2$	Counts	RMSZ	$\#\|Z\| > 2$
14	CLA	0	1012	-	57,73,73	2.03	13 (22%)	66,113,113	2.29	21 (31%)
13	CL0	AA	1011	-	57,73,73	2.08	15 (26%)	66,113,113	2.54	20 (30%)
14	CLA	AA	1012	-	57,73,73	2.01	14 (24%)	66,113,113	2.23	19 (28%)
14	CLA	AA	1013	-	52,68,73	2.16	12 (23%)	60,107,113	2.48	24 (40%)
14	CLA	AA	1101	-	34,53,73	2.53	13 (38%)	37,89,113	2.43	13 (35%)
14	CLA	AA	1102	-	52,68,73	2.22	14 (26%)	60,107,113	2.40	16 (26%)
14	CLA	AA	1103	-	52,68,73	2.10	14 (26%)	60,107,113	2.33	17 (28%)
14	CLA	AA	1104	-	57,73,73	2.03	14 (24%)	66,113,113	2.19	17 (25%)
14	CLA	AA	1105	-	34,53,73	2.61	14 (41%)	37,89,113	2.28	10 (27%)
14	CLA	AA	1106	-	34,53,73	2.54	14 (41%)	37,89,113	2.53	12 (32%)
14	CLA	AA	1107	-	34,53,73	2.47	13 (38%)	37,89,113	2.36	10 (27%)
14	CLA	AA	1108	-	34,53,73	2.59	13 (38%)	37,89,113	2.31	11 (29%)
14	CLA	AA	1109	-	34,53,73	2.53	14 (41%)	37,89,113	2.43	12 (32%)
14	CLA	AA	1110	-	34,53,73	2.55	14 (41%)	37,89,113	2.56	11 (29%)
14	CLA	AA	1111	-	52,68,73	2.17	16 (30%)	60,107,113	2.32	18 (30%)
14	CLA	AA	1112	-	34,53,73	2.58	15 (44%)	37,89,113	2.34	9 (24%)
14	CLA	AA	1113	-	34,53,73	2.55	13 (38%)	37,89,113	2.57	11 (29%)
14	CLA	AA	1114	-	34,53,73	2.63	15 (44%)	37,89,113	2.49	13 (35%)
14	CLA	AA	1115	-	47,63,73	2.33	14 (29%)	54,101,113	2.43	16 (29%)
14	CLA	AA	1116	-	47,63,73	2.28	14 (29%)	54,101,113	2.39	18 (33%)
14	CLA	AA	1117	-	57,73,73	2.06	15 (26%)	66,113,113	2.21	19 (28%)
14	CLA	AA	1118	-	47,63,73	2.26	14 (29%)	54,101,113	2.52	19 (35%)
14	CLA	AA	1119	-	57,73,73	2.10	14 (24%)	66,113,113	2.10	18 (27%)
14	CLA	AA	1122	-	47,63,73	2.27	15 (31%)	54,101,113	2.27	18 (33%)
14	CLA	AA	1123	-	52,68,73	2.18	14 (26%)	60,107,113	2.38	20 (33%)
14	CLA	AA	1124	-	47,63,73	2.26	14 (29%)	54,101,113	2.34	17 (31%)
14	CLA	AA	1125	-	57,73,73	2.04	13 (22%)	66,113,113	2.39	19 (28%)
14	CLA	AA	1126	-	52,68,73	2.18	14 (26%)	60,107,113	2.32	16 (26%)
14	CLA	AA	1127	-	57,73,73	2.09	14 (24%)	66,113,113	2.26	19 (28%)

PRELIMINARY VALIDATION REPORT

Mol	Type	Chain	Res	Link	Bond lengths			Bond angles		
					Counts	RMSZ	#|Z| > 2	Counts	RMSZ	#|Z| > 2
14	CLA	AA	1128	-	52,68,73	2.16	15 (28%)	60,107,113	2.46	18 (30%)
14	CLA	AA	1129	1	34,53,73	2.49	13 (38%)	37,89,113	2.55	13 (35%)
14	CLA	AA	1130	-	47,63,73	2.24	14 (29%)	54,101,113	2.30	16 (29%)
14	CLA	AA	1131	1	52,68,73	2.14	15 (28%)	60,107,113	2.32	16 (26%)
14	CLA	AA	1132	-	57,73,73	2.04	14 (24%)	66,113,113	2.26	17 (25%)
14	CLA	AA	1133	-	52,68,73	2.18	15 (28%)	60,107,113	2.32	16 (26%)
14	CLA	AA	1135	-	42,58,73	2.46	15 (35%)	48,95,113	2.61	18 (37%)
14	CLA	AA	1136	-	47,63,73	2.23	15 (31%)	54,101,113	2.52	14 (25%)
14	CLA	AA	1137	-	34,53,73	2.50	13 (38%)	37,89,113	2.70	13 (35%)
14	CLA	AA	1138	-	52,68,73	2.18	15 (28%)	60,107,113	2.30	19 (31%)
14	CLA	AA	1139	-	34,53,73	2.54	13 (38%)	37,89,113	2.32	11 (29%)
14	CLA	AA	1140	-	57,73,73	2.08	14 (24%)	66,113,113	2.13	15 (22%)
16	PQN	AA	2001	-	34,34,34	1.28	2 (5%)	42,45,45	1.08	1 (2%)
17	SF4	AA	3001	-	0,12,12	0.00	-	-		
18	BCR	AA	4001	-	41,41,41	3.00	6 (14%)	56,56,56	6.49	25 (44%)
18	BCR	AA	4002	-	41,41,41	2.98	6 (14%)	56,56,56	6.18	24 (42%)
18	BCR	AA	4003	-	41,41,41	2.94	6 (14%)	56,56,56	6.50	24 (42%)
18	BCR	AA	4007	-	41,41,41	3.03	7 (17%)	56,56,56	6.66	24 (42%)
18	BCR	AA	4008	-	41,41,41	3.20	10 (24%)	56,56,56	6.34	22 (39%)
18	BCR	AA	4011	-	41,41,41	3.00	7 (17%)	56,56,56	6.53	27 (48%)
19	LHG	AA	5001	-	41,41,48	1.00	2 (4%)	44,47,54	1.09	2 (4%)
20	LMG	AA	5005	-	46,46,55	1.26	5 (10%)	54,54,63	1.14	2 (3%)
21	LMT	AA	6001	-	32,32,36	1.29	6 (18%)	43,43,47	1.03	3 (6%)
21	LMT	AA	6002	-	29,29,36	1.39	5 (17%)	40,40,47	1.39	7 (17%)
14	CLA	BA	1021	-	57,73,73	2.04	13 (22%)	66,113,113	2.40	21 (31%)
14	CLA	BA	1022	-	57,73,73	2.04	13 (22%)	66,113,113	2.29	21 (31%)
14	CLA	BA	1023	-	57,73,73	2.03	15 (26%)	66,113,113	2.60	25 (37%)
14	CLA	BA	1201	-	47,63,73	2.27	15 (31%)	54,101,113	2.57	17 (31%)
14	CLA	BA	1202	-	52,68,73	2.11	14 (26%)	60,107,113	2.29	18 (30%)
14	CLA	BA	1203	-	57,73,73	1.99	13 (22%)	66,113,113	2.14	17 (25%)
14	CLA	BA	1204	2	57,73,73	2.02	14 (24%)	66,113,113	2.18	18 (27%)
14	CLA	BA	1205	-	57,73,73	2.02	15 (26%)	66,113,113	2.49	20 (30%)
14	CLA	BA	1206	-	34,53,73	2.48	13 (38%)	37,89,113	2.46	13 (35%)
14	CLA	BA	1207	-	34,53,73	2.53	13 (38%)	37,89,113	2.55	12 (32%)
14	CLA	BA	1208	-	57,73,73	2.09	14 (24%)	66,113,113	2.15	14 (21%)

PRELIMINARY VALIDATION REPORT

Mol	Type	Chain	Res	Link	Bond lengths			Bond angles		
					Counts	RMSZ	#|Z| > 2	Counts	RMSZ	#|Z| > 2
14	CLA	BA	1209	-	34,53,73	2.55	14 (41%)	37,89,113	2.38	10 (27%)
14	CLA	BA	1210	-	57,73,73	2.07	15 (26%)	66,113,113	2.27	20 (30%)
14	CLA	BA	1211	-	57,73,73	2.09	14 (24%)	66,113,113	2.09	15 (22%)
14	CLA	BA	1212	-	34,53,73	2.60	14 (41%)	37,89,113	2.52	13 (35%)
14	CLA	BA	1213	-	47,63,73	2.34	14 (29%)	54,101,113	2.42	16 (29%)
14	CLA	BA	1214	-	52,68,73	2.17	15 (28%)	60,107,113	2.33	22 (36%)
14	CLA	BA	1215	-	52,68,73	2.19	14 (26%)	60,107,113	2.31	18 (30%)
14	CLA	BA	1216	-	47,63,73	2.29	15 (31%)	54,101,113	2.31	20 (37%)
14	CLA	BA	1217	-	47,63,73	2.29	15 (31%)	54,101,113	2.36	16 (29%)
14	CLA	BA	1218	-	34,53,73	2.61	13 (38%)	37,89,113	2.49	11 (29%)
14	CLA	BA	1219	-	34,53,73	2.59	14 (41%)	37,89,113	2.39	12 (32%)
14	CLA	BA	1220	-	47,63,73	2.32	14 (29%)	54,101,113	2.43	17 (31%)
14	CLA	BA	1221	-	52,68,73	2.15	14 (26%)	60,107,113	2.25	20 (33%)
14	CLA	BA	1222	-	57,73,73	2.10	14 (24%)	66,113,113	2.30	21 (31%)
14	CLA	BA	1223	-	57,73,73	2.08	14 (24%)	66,113,113	2.26	19 (28%)
14	CLA	BA	1224	-	57,73,73	2.06	13 (22%)	66,113,113	2.24	19 (28%)
14	CLA	BA	1225	-	57,73,73	2.07	13 (22%)	66,113,113	2.12	13 (19%)
14	CLA	BA	1226	-	57,73,73	2.06	15 (26%)	66,113,113	2.33	19 (28%)
14	CLA	BA	1227	-	34,53,73	2.57	13 (38%)	37,89,113	2.38	13 (35%)
14	CLA	BA	1228	-	34,53,73	2.58	14 (41%)	37,89,113	2.39	13 (35%)
14	CLA	BA	1229	-	52,68,73	2.21	14 (26%)	60,107,113	2.32	15 (25%)
14	CLA	BA	1231	-	47,63,73	2.35	14 (29%)	54,101,113	2.34	16 (29%)
14	CLA	BA	1232	-	34,53,73	2.58	14 (41%)	37,89,113	2.38	13 (35%)
14	CLA	BA	1233	-	34,53,73	2.61	14 (41%)	37,89,113	2.40	12 (32%)
14	CLA	BA	1234	-	47,63,73	2.21	13 (27%)	54,101,113	2.17	17 (31%)
14	CLA	BA	1235	-	52,68,73	2.19	14 (26%)	60,107,113	2.41	19 (31%)
14	CLA	BA	1236	-	34,53,73	2.52	13 (38%)	37,89,113	2.38	10 (27%)
14	CLA	BA	1238	-	57,73,73	2.08	13 (22%)	66,113,113	2.41	21 (31%)
14	CLA	BA	1239	-	57,73,73	2.08	15 (26%)	66,113,113	2.44	21 (31%)
16	PQN	BA	2002	-	34,34,34	1.32	2 (5%)	42,45,45	1.17	1 (2%)
18	BCR	BA	4004	-	41,41,41	2.89	7 (17%)	56,56,56	6.47	25 (44%)
18	BCR	BA	4005	-	41,41,41	2.89	7 (17%)	56,56,56	6.62	27 (48%)
18	BCR	BA	4006	-	41,41,41	2.88	6 (14%)	56,56,56	6.54	22 (39%)
18	BCR	BA	4009	-	41,41,41	2.95	6 (14%)	56,56,56	6.57	23 (41%)
18	BCR	BA	4010	-	41,41,41	3.02	7 (17%)	56,56,56	6.59	22 (39%)

PRELIMINARY VALIDATION REPORT

Mol	Type	Chain	Res	Link	Bond lengths			Bond angles		
					Counts	RMSZ	#|Z| > 2	Counts	RMSZ	#|Z| > 2
18	BCR	BA	4012	-	41,41,41	2.79	6 (14%)	56,56,56	6.63	27 (48%)
18	BCR	BA	4013	-	41,41,41	2.85	6 (14%)	56,56,56	6.57	25 (44%)
18	BCR	BA	4014	-	41,41,41	3.01	6 (14%)	56,56,56	6.44	23 (41%)
18	BCR	BA	4016	-	41,41,41	2.88	6 (14%)	56,56,56	6.50	22 (39%)
18	BCR	BA	4017	-	41,41,41	3.02	6 (14%)	56,56,56	6.60	19 (33%)
20	LMG	BA	5002	-	46,46,55	1.26	5 (10%)	54,54,63	1.15	3 (5%)
19	LHG	BA	5004	-	43,43,48	0.96	2 (4%)	46,49,54	1.18	3 (6%)
17	SF4	CA	3002	-	0,12,12	0.00	-	-		
17	SF4	CA	3003	-	0,12,12	0.00	-	-		
13	CL0	GA	1011	-	57,73,73	2.08	15 (26%)	66,113,113	2.54	21 (31%)
14	CLA	GA	1012	-	57,73,73	2.01	14 (24%)	66,113,113	2.23	19 (28%)
14	CLA	GA	1013	-	52,68,73	2.17	12 (23%)	60,107,113	2.48	24 (40%)
14	CLA	GA	1101	-	34,53,73	2.53	13 (38%)	37,89,113	2.42	13 (35%)
14	CLA	GA	1102	-	52,68,73	2.22	14 (26%)	60,107,113	2.39	15 (25%)
14	CLA	GA	1103	-	52,68,73	2.09	14 (26%)	60,107,113	2.32	17 (28%)
14	CLA	GA	1104	-	57,73,73	2.03	14 (24%)	66,113,113	2.18	17 (25%)
14	CLA	GA	1105	-	34,53,73	2.62	13 (38%)	37,89,113	2.28	10 (27%)
14	CLA	GA	1106	-	34,53,73	2.54	14 (41%)	37,89,113	2.53	12 (32%)
14	CLA	GA	1107	-	34,53,73	2.46	13 (38%)	37,89,113	2.35	10 (27%)
14	CLA	GA	1108	-	34,53,73	2.60	13 (38%)	37,89,113	2.31	11 (29%)
14	CLA	GA	1109	-	34,53,73	2.54	14 (41%)	37,89,113	2.44	12 (32%)
14	CLA	GA	1110	-	34,53,73	2.54	14 (41%)	37,89,113	2.55	11 (29%)
14	CLA	GA	1111	-	52,68,73	2.17	16 (30%)	60,107,113	2.31	18 (30%)
14	CLA	GA	1112	-	34,53,73	2.58	15 (44%)	37,89,113	2.34	10 (27%)
14	CLA	GA	1113	-	34,53,73	2.55	13 (38%)	37,89,113	2.56	11 (29%)
14	CLA	GA	1114	-	34,53,73	2.63	15 (44%)	37,89,113	2.48	13 (35%)
14	CLA	GA	1115	-	47,63,73	2.33	14 (29%)	54,101,113	2.42	16 (29%)
14	CLA	GA	1116	-	47,63,73	2.28	14 (29%)	54,101,113	2.40	18 (33%)
14	CLA	GA	1117	-	57,73,73	2.06	15 (26%)	66,113,113	2.22	19 (28%)
14	CLA	GA	1118	-	47,63,73	2.27	14 (29%)	54,101,113	2.53	19 (35%)
14	CLA	GA	1119	-	57,73,73	2.10	14 (24%)	66,113,113	2.09	18 (27%)
14	CLA	GA	1122	-	47,63,73	2.27	15 (31%)	54,101,113	2.28	18 (33%)
14	CLA	GA	1123	-	52,68,73	2.17	14 (26%)	60,107,113	2.37	19 (31%)
14	CLA	GA	1124	-	47,63,73	2.26	14 (29%)	54,101,113	2.33	17 (31%)
14	CLA	GA	1125	-	57,73,73	2.04	13 (22%)	66,113,113	2.40	20 (30%)

PRELIMINARY VALIDATION REPORT

Mol	Type	Chain	Res	Link	Bond lengths			Bond angles		
					Counts	RMSZ	#|Z| > 2	Counts	RMSZ	#|Z| > 2
14	CLA	GA	1126	-	52,68,73	2.18	14 (26%)	60,107,113	2.32	16 (26%)
14	CLA	GA	1127	-	57,73,73	2.08	14 (24%)	66,113,113	2.26	19 (28%)
14	CLA	GA	1128	-	52,68,73	2.16	15 (28%)	60,107,113	2.45	18 (30%)
14	CLA	GA	1129	-	34,53,73	2.50	13 (38%)	37,89,113	2.55	13 (35%)
14	CLA	GA	1130	-	47,63,73	2.24	14 (29%)	54,101,113	2.29	16 (29%)
14	CLA	GA	1131	1	52,68,73	2.13	15 (28%)	60,107,113	2.32	17 (28%)
14	CLA	GA	1132	-	57,73,73	2.04	14 (24%)	66,113,113	2.26	17 (25%)
14	CLA	GA	1133	-	52,68,73	2.18	15 (28%)	60,107,113	2.33	17 (28%)
14	CLA	GA	1135	-	42,58,73	2.46	15 (35%)	48,95,113	2.61	18 (37%)
14	CLA	GA	1136	-	47,63,73	2.22	15 (31%)	54,101,113	2.51	14 (25%)
14	CLA	GA	1137	-	34,53,73	2.51	13 (38%)	37,89,113	2.70	13 (35%)
14	CLA	GA	1138	-	52,68,73	2.18	15 (28%)	60,107,113	2.31	20 (33%)
14	CLA	GA	1139	-	34,53,73	2.55	13 (38%)	37,89,113	2.31	11 (29%)
14	CLA	GA	1140	-	57,73,73	2.08	14 (24%)	66,113,113	2.13	15 (22%)
16	PQN	GA	2001	-	34,34,34	1.29	2 (5%)	42,45,45	1.08	1 (2%)
17	SF4	GA	3001	-	0,12,12	0.00	-	-		
18	BCR	GA	4001	-	41,41,41	2.99	6 (14%)	56,56,56	6.49	25 (44%)
18	BCR	GA	4002	-	41,41,41	2.98	6 (14%)	56,56,56	6.18	24 (42%)
18	BCR	GA	4003	-	41,41,41	2.94	6 (14%)	56,56,56	6.51	24 (42%)
18	BCR	GA	4007	-	41,41,41	3.02	7 (17%)	56,56,56	6.65	24 (42%)
18	BCR	GA	4008	-	41,41,41	3.20	10 (24%)	56,56,56	6.34	22 (39%)
18	BCR	GA	4011	-	41,41,41	3.00	7 (17%)	56,56,56	6.53	27 (48%)
19	LHG	GA	5001	-	41,41,48	1.00	2 (4%)	44,47,54	1.09	2 (4%)
20	LMG	GA	5005	-	46,46,55	1.27	5 (10%)	54,54,63	1.14	2 (3%)
21	LMT	GA	6001	-	32,32,36	1.28	6 (18%)	43,43,47	1.02	3 (6%)
21	LMT	GA	6002	-	29,29,36	1.39	5 (17%)	40,40,47	1.39	7 (17%)
14	CLA	HA	1021	-	57,73,73	2.04	13 (22%)	66,113,113	2.41	21 (31%)
14	CLA	HA	1023	-	57,73,73	2.04	15 (26%)	66,113,113	2.60	25 (37%)
14	CLA	HA	1201	-	47,63,73	2.28	15 (31%)	54,101,113	2.57	17 (31%)
14	CLA	HA	1202	-	52,68,73	2.10	14 (26%)	60,107,113	2.29	16 (26%)
14	CLA	HA	1203	-	57,73,73	1.99	13 (22%)	66,113,113	2.14	17 (25%)
14	CLA	HA	1204	2	57,73,73	2.03	14 (24%)	66,113,113	2.18	18 (27%)
14	CLA	HA	1205	-	57,73,73	2.01	14 (24%)	66,113,113	2.49	20 (30%)
14	CLA	HA	1206	-	34,53,73	2.48	14 (41%)	37,89,113	2.46	13 (35%)
14	CLA	HA	1207	-	34,53,73	2.53	13 (38%)	37,89,113	2.56	12 (32%)

PRELIMINARY VALIDATION REPORT

Mol	Type	Chain	Res	Link	Bond lengths			Bond angles		
					Counts	RMSZ	#|Z| > 2	Counts	RMSZ	#|Z| > 2
14	CLA	HA	1208	-	57,73,73	2.09	14 (24%)	66,113,113	2.15	14 (21%)
14	CLA	HA	1209	-	34,53,73	2.56	14 (41%)	37,89,113	2.38	10 (27%)
14	CLA	HA	1210	-	57,73,73	2.08	15 (26%)	66,113,113	2.27	21 (31%)
14	CLA	HA	1211	-	57,73,73	2.09	14 (24%)	66,113,113	2.09	15 (22%)
14	CLA	HA	1212	-	34,53,73	2.59	14 (41%)	37,89,113	2.51	13 (35%)
14	CLA	HA	1213	-	47,63,73	2.34	14 (29%)	54,101,113	2.43	16 (29%)
14	CLA	HA	1214	-	52,68,73	2.17	15 (28%)	60,107,113	2.33	21 (35%)
14	CLA	HA	1215	-	52,68,73	2.19	14 (26%)	60,107,113	2.30	18 (30%)
14	CLA	HA	1216	-	47,63,73	2.30	15 (31%)	54,101,113	2.31	20 (37%)
14	CLA	HA	1217	-	47,63,73	2.29	15 (31%)	54,101,113	2.35	16 (29%)
14	CLA	HA	1218	-	34,53,73	2.61	13 (38%)	37,89,113	2.49	11 (29%)
14	CLA	HA	1219	-	34,53,73	2.58	14 (41%)	37,89,113	2.40	12 (32%)
14	CLA	HA	1220	-	47,63,73	2.32	14 (29%)	54,101,113	2.43	17 (31%)
14	CLA	HA	1221	-	52,68,73	2.15	14 (26%)	60,107,113	2.25	20 (33%)
14	CLA	HA	1222	-	57,73,73	2.10	14 (24%)	66,113,113	2.29	21 (31%)
14	CLA	HA	1223	-	57,73,73	2.07	14 (24%)	66,113,113	2.26	19 (28%)
14	CLA	HA	1224	-	57,73,73	2.06	13 (22%)	66,113,113	2.23	19 (28%)
14	CLA	HA	1225	-	57,73,73	2.06	13 (22%)	66,113,113	2.12	13 (19%)
14	CLA	HA	1226	-	57,73,73	2.06	15 (26%)	66,113,113	2.33	19 (28%)
14	CLA	HA	1227	-	34,53,73	2.58	13 (38%)	37,89,113	2.39	13 (35%)
14	CLA	HA	1228	-	34,53,73	2.58	14 (41%)	37,89,113	2.39	13 (35%)
14	CLA	HA	1229	-	52,68,73	2.22	14 (26%)	60,107,113	2.33	15 (25%)
14	CLA	HA	1231	-	47,63,73	2.35	14 (29%)	54,101,113	2.35	16 (29%)
14	CLA	HA	1232	-	34,53,73	2.57	14 (41%)	37,89,113	2.37	13 (35%)
14	CLA	HA	1233	-	34,53,73	2.61	14 (41%)	37,89,113	2.40	12 (32%)
14	CLA	HA	1234	-	47,63,73	2.20	13 (27%)	54,101,113	2.18	17 (31%)
14	CLA	HA	1235	-	52,68,73	2.19	14 (26%)	60,107,113	2.42	19 (31%)
14	CLA	HA	1236	-	34,53,73	2.52	13 (38%)	37,89,113	2.39	10 (27%)
14	CLA	HA	1238	-	57,73,73	2.08	14 (24%)	66,113,113	2.41	21 (31%)
14	CLA	HA	1239	-	57,73,73	2.08	16 (28%)	66,113,113	2.44	21 (31%)
16	PQN	HA	2002	-	34,34,34	1.32	2 (5%)	42,45,45	1.17	1 (2%)
18	BCR	HA	4004	-	41,41,41	2.90	6 (14%)	56,56,56	6.48	25 (44%)
18	BCR	HA	4005	-	41,41,41	2.89	7 (17%)	56,56,56	6.62	28 (50%)
18	BCR	HA	4006	-	41,41,41	2.88	6 (14%)	56,56,56	6.55	22 (39%)
18	BCR	HA	4009	-	41,41,41	2.95	6 (14%)	56,56,56	6.57	25 (44%)

PRELIMINARY VALIDATION REPORT

Mol	Type	Chain	Res	Link	Bond lengths			Bond angles		
					Counts	RMSZ	#|Z| > 2	Counts	RMSZ	#|Z| > 2
18	BCR	HA	4010	-	41,41,41	3.02	7 (17%)	56,56,56	6.59	22 (39%)
18	BCR	HA	4012	-	41,41,41	2.79	6 (14%)	56,56,56	6.63	27 (48%)
18	BCR	HA	4013	-	41,41,41	2.85	6 (14%)	56,56,56	6.57	24 (42%)
18	BCR	HA	4014	-	41,41,41	3.01	6 (14%)	56,56,56	6.45	24 (42%)
18	BCR	HA	4016	-	41,41,41	2.89	6 (14%)	56,56,56	6.50	22 (39%)
18	BCR	HA	4017	-	41,41,41	3.03	7 (17%)	56,56,56	6.60	19 (33%)
20	LMG	HA	5002	-	46,46,55	1.26	5 (10%)	54,54,63	1.15	3 (5%)
19	LHG	HA	5004	-	43,43,48	0.96	2 (4%)	46,49,54	1.18	3 (6%)
18	BCR	IA	4018	-	41,41,41	3.15	9 (21%)	56,56,56	6.69	23 (41%)
18	BCR	IA	4020	-	41,41,41	3.19	10 (24%)	56,56,56	6.31	25 (44%)
20	LMG	IA	5006	-	37,37,55	1.12	2 (5%)	45,45,63	1.19	4 (8%)
14	CLA	KA	1401	-	34,53,73	2.68	15 (44%)	37,89,113	2.33	9 (24%)
14	CLA	LA	1501	-	52,68,73	2.17	15 (28%)	60,107,113	2.60	19 (31%)
14	CLA	LA	1502	-	52,68,73	2.10	14 (26%)	60,107,113	2.32	17 (28%)
14	CLA	LA	1503	-	57,73,73	2.05	14 (24%)	66,113,113	2.19	17 (25%)
18	BCR	LA	4019	-	41,41,41	2.92	6 (14%)	56,56,56	6.55	23 (41%)
18	BCR	LA	4022	-	41,41,41	3.10	9 (21%)	56,56,56	6.48	24 (42%)
20	LMG	LA	5007	-	50,50,55	1.32	7 (14%)	58,58,63	1.08	2 (3%)
18	BCR	MA	4021	-	41,41,41	2.97	6 (14%)	56,56,56	6.56	23 (41%)
17	SF4	NA	3002	-	0,12,12	0.00	-	-		
17	SF4	NA	3003	-	0,12,12	0.00	-	-		
18	BCR	RA	4018	-	41,41,41	3.14	8 (19%)	56,56,56	6.70	23 (41%)
18	BCR	RA	4020	-	41,41,41	3.19	10 (24%)	56,56,56	6.31	25 (44%)
20	LMG	RA	5006	-	37,37,55	1.12	2 (5%)	45,45,63	1.19	4 (8%)
14	CLA	TA	1401	-	34,53,73	2.69	15 (44%)	37,89,113	2.32	9 (24%)
14	CLA	UA	1501	-	52,68,73	2.17	15 (28%)	60,107,113	2.60	19 (31%)
14	CLA	UA	1502	-	52,68,73	2.11	14 (26%)	60,107,113	2.31	17 (28%)
14	CLA	UA	1503	-	57,73,73	2.06	14 (24%)	66,113,113	2.19	17 (25%)
18	BCR	UA	4019	-	41,41,41	2.92	6 (14%)	56,56,56	6.55	23 (41%)
18	BCR	UA	4022	-	41,41,41	3.10	9 (21%)	56,56,56	6.47	24 (42%)
20	LMG	UA	5007	-	50,50,55	1.32	7 (14%)	58,58,63	1.07	2 (3%)
18	BCR	VA	4021	-	41,41,41	2.98	6 (14%)	56,56,56	6.55	23 (41%)
14	CLA	WA	1701	-	34,53,73	2.56	13 (38%)	37,89,113	2.34	11 (29%)
14	CLA	XA	1701	-	34,53,73	2.57	13 (38%)	37,89,113	2.33	13 (35%)
13	CL0	aA	1011	-	57,73,73	2.09	15 (26%)	66,113,113	2.54	21 (31%)

PRELIMINARY VALIDATION REPORT

Mol	Type	Chain	Res	Link	Bond lengths			Bond angles		
					Counts	RMSZ	#|Z| > 2	Counts	RMSZ	#|Z| > 2
14	CLA	aA	1012	-	57,73,73	2.01	14 (24%)	66,113,113	2.24	19 (28%)
14	CLA	aA	1013	-	52,68,73	2.16	12 (23%)	60,107,113	2.49	24 (40%)
14	CLA	aA	1101	-	34,53,73	2.53	13 (38%)	37,89,113	2.42	13 (35%)
14	CLA	aA	1102	-	52,68,73	2.22	14 (26%)	60,107,113	2.40	15 (25%)
14	CLA	aA	1103	-	52,68,73	2.09	14 (26%)	60,107,113	2.33	17 (28%)
14	CLA	aA	1104	-	57,73,73	2.03	14 (24%)	66,113,113	2.19	17 (25%)
14	CLA	aA	1105	-	34,53,73	2.61	13 (38%)	37,89,113	2.29	10 (27%)
14	CLA	aA	1106	-	34,53,73	2.54	14 (41%)	37,89,113	2.53	12 (32%)
14	CLA	aA	1107	-	34,53,73	2.46	13 (38%)	37,89,113	2.35	10 (27%)
14	CLA	aA	1108	-	34,53,73	2.59	13 (38%)	37,89,113	2.30	11 (29%)
14	CLA	aA	1109	-	34,53,73	2.52	14 (41%)	37,89,113	2.42	11 (29%)
14	CLA	aA	1110	-	34,53,73	2.55	14 (41%)	37,89,113	2.55	11 (29%)
14	CLA	aA	1111	-	52,68,73	2.17	16 (30%)	60,107,113	2.31	18 (30%)
14	CLA	aA	1112	-	34,53,73	2.58	15 (44%)	37,89,113	2.36	10 (27%)
14	CLA	aA	1113	-	34,53,73	2.55	13 (38%)	37,89,113	2.56	11 (29%)
14	CLA	aA	1114	-	34,53,73	2.64	15 (44%)	37,89,113	2.49	13 (35%)
14	CLA	aA	1115	-	47,63,73	2.33	14 (29%)	54,101,113	2.42	16 (29%)
14	CLA	aA	1116	-	47,63,73	2.28	14 (29%)	54,101,113	2.39	18 (33%)
14	CLA	aA	1117	-	57,73,73	2.06	15 (26%)	66,113,113	2.22	19 (28%)
14	CLA	aA	1118	-	47,63,73	2.27	14 (29%)	54,101,113	2.52	19 (35%)
14	CLA	aA	1119	-	57,73,73	2.10	14 (24%)	66,113,113	2.09	18 (27%)
14	CLA	aA	1122	-	47,63,73	2.27	15 (31%)	54,101,113	2.29	18 (33%)
14	CLA	aA	1123	-	52,68,73	2.18	14 (26%)	60,107,113	2.37	19 (31%)
14	CLA	aA	1124	-	47,63,73	2.26	14 (29%)	54,101,113	2.33	17 (31%)
14	CLA	aA	1125	-	57,73,73	2.04	13 (22%)	66,113,113	2.39	20 (30%)
14	CLA	aA	1126	-	52,68,73	2.18	14 (26%)	60,107,113	2.32	16 (26%)
14	CLA	aA	1127	-	57,73,73	2.08	14 (24%)	66,113,113	2.26	19 (28%)
14	CLA	aA	1128	-	52,68,73	2.16	15 (28%)	60,107,113	2.45	18 (30%)
14	CLA	aA	1129	1	34,53,73	2.50	13 (38%)	37,89,113	2.54	13 (35%)
14	CLA	aA	1130	-	47,63,73	2.24	14 (29%)	54,101,113	2.30	16 (29%)
14	CLA	aA	1131	1	52,68,73	2.13	15 (28%)	60,107,113	2.32	16 (26%)
14	CLA	aA	1132	-	57,73,73	2.04	14 (24%)	66,113,113	2.26	17 (25%)
14	CLA	aA	1133	-	52,68,73	2.18	15 (28%)	60,107,113	2.31	17 (28%)
14	CLA	aA	1135	-	42,58,73	2.45	15 (35%)	48,95,113	2.60	18 (37%)
14	CLA	aA	1136	-	47,63,73	2.23	15 (31%)	54,101,113	2.52	14 (25%)

PRELIMINARY VALIDATION REPORT

PRELIMINARY VALIDATION REPORT

Mol	Type	Chain	Res	Link	Bond lengths			Bond angles		
					Counts	RMSZ	#|Z| > 2	Counts	RMSZ	#|Z| > 2
14	CLA	aA	1137	-	34,53,73	2.51	13 (38%)	37,89,113	2.70	13 (35%)
14	CLA	aA	1138	-	52,68,73	2.18	15 (28%)	60,107,113	2.30	19 (31%)
14	CLA	aA	1139	-	34,53,73	2.54	13 (38%)	37,89,113	2.31	11 (29%)
14	CLA	aA	1140	-	57,73,73	2.08	14 (24%)	66,113,113	2.12	15 (22%)
16	PQN	aA	2001	-	34,34,34	1.28	2 (5%)	42,45,45	1.08	1 (2%)
17	SF4	aA	3001	-	0,12,12	0.00	-	-		
18	BCR	aA	4001	-	41,41,41	2.99	6 (14%)	56,56,56	6.48	25 (44%)
18	BCR	aA	4002	-	41,41,41	2.98	6 (14%)	56,56,56	6.17	24 (42%)
18	BCR	aA	4003	-	41,41,41	2.94	6 (14%)	56,56,56	6.50	24 (42%)
18	BCR	aA	4007	-	41,41,41	3.02	7 (17%)	56,56,56	6.66	24 (42%)
18	BCR	aA	4008	-	41,41,41	3.19	9 (21%)	56,56,56	6.33	22 (39%)
18	BCR	aA	4011	-	41,41,41	3.00	7 (17%)	56,56,56	6.53	27 (48%)
19	LHG	aA	5001	-	41,41,48	1.00	2 (4%)	44,47,54	1.09	2 (4%)
20	LMG	aA	5005	-	46,46,55	1.27	5 (10%)	54,54,63	1.14	2 (3%)
21	LMT	aA	6001	-	32,32,36	1.29	6 (18%)	43,43,47	1.03	4 (9%)
21	LMT	aA	6002	-	29,29,36	1.39	5 (17%)	40,40,47	1.39	8 (20%)
14	CLA	bA	1021	-	57,73,73	2.04	13 (22%)	66,113,113	2.41	21 (31%)
14	CLA	bA	1022	-	57,73,73	2.03	13 (22%)	66,113,113	2.29	21 (31%)
14	CLA	bA	1023	-	57,73,73	2.03	15 (26%)	66,113,113	2.60	25 (37%)
14	CLA	bA	1201	-	47,63,73	2.27	15 (31%)	54,101,113	2.57	17 (31%)
14	CLA	bA	1202	-	52,68,73	2.11	14 (26%)	60,107,113	2.29	18 (30%)
14	CLA	bA	1203	-	57,73,73	1.99	13 (22%)	66,113,113	2.14	17 (25%)
14	CLA	bA	1204	2	57,73,73	2.03	14 (24%)	66,113,113	2.19	18 (27%)
14	CLA	bA	1205	-	57,73,73	2.01	14 (24%)	66,113,113	2.49	20 (30%)
14	CLA	bA	1206	-	34,53,73	2.49	13 (38%)	37,89,113	2.46	13 (35%)
14	CLA	bA	1207	-	34,53,73	2.52	13 (38%)	37,89,113	2.56	12 (32%)
14	CLA	bA	1208	-	57,73,73	2.09	14 (24%)	66,113,113	2.15	14 (21%)
14	CLA	bA	1209	-	34,53,73	2.56	14 (41%)	37,89,113	2.39	11 (29%)
14	CLA	bA	1210	-	57,73,73	2.07	15 (26%)	66,113,113	2.26	20 (30%)
14	CLA	bA	1211	-	57,73,73	2.09	14 (24%)	66,113,113	2.09	15 (22%)
14	CLA	bA	1212	-	34,53,73	2.59	14 (41%)	37,89,113	2.50	13 (35%)
14	CLA	bA	1213	-	47,63,73	2.34	14 (29%)	54,101,113	2.43	16 (29%)
14	CLA	bA	1214	-	52,68,73	2.18	15 (28%)	60,107,113	2.33	22 (36%)
14	CLA	bA	1215	-	52,68,73	2.19	14 (26%)	60,107,113	2.30	18 (30%)
14	CLA	bA	1216	-	47,63,73	2.30	15 (31%)	54,101,113	2.31	19 (35%)

Mol	Type	Chain	Res	Link	Bond lengths			Bond angles		
					Counts	RMSZ	#|Z| > 2	Counts	RMSZ	#|Z| > 2
14	CLA	bA	1217	-	47,63,73	2.29	15 (31%)	54,101,113	2.35	16 (29%)
14	CLA	bA	1218	-	34,53,73	2.60	13 (38%)	37,89,113	2.48	11 (29%)
14	CLA	bA	1219	-	34,53,73	2.59	14 (41%)	37,89,113	2.40	12 (32%)
14	CLA	bA	1220	-	47,63,73	2.32	14 (29%)	54,101,113	2.43	17 (31%)
14	CLA	bA	1221	-	52,68,73	2.15	14 (26%)	60,107,113	2.25	20 (33%)
14	CLA	bA	1222	-	57,73,73	2.10	14 (24%)	66,113,113	2.30	21 (31%)
14	CLA	bA	1223	-	57,73,73	2.08	14 (24%)	66,113,113	2.25	18 (27%)
14	CLA	bA	1224	-	57,73,73	2.06	13 (22%)	66,113,113	2.24	19 (28%)
14	CLA	bA	1225	-	57,73,73	2.06	13 (22%)	66,113,113	2.12	13 (19%)
14	CLA	bA	1226	-	57,73,73	2.06	15 (26%)	66,113,113	2.33	19 (28%)
14	CLA	bA	1227	-	34,53,73	2.57	13 (38%)	37,89,113	2.38	13 (35%)
14	CLA	bA	1228	-	34,53,73	2.58	14 (41%)	37,89,113	2.38	13 (35%)
14	CLA	bA	1229	-	52,68,73	2.21	14 (26%)	60,107,113	2.32	15 (25%)
14	CLA	bA	1231	-	47,63,73	2.35	14 (29%)	54,101,113	2.35	16 (29%)
14	CLA	bA	1232	-	34,53,73	2.57	14 (41%)	37,89,113	2.37	13 (35%)
14	CLA	bA	1233	-	34,53,73	2.61	14 (41%)	37,89,113	2.41	12 (32%)
14	CLA	bA	1234	-	47,63,73	2.21	13 (27%)	54,101,113	2.17	17 (31%)
14	CLA	bA	1235	-	52,68,73	2.18	14 (26%)	60,107,113	2.42	19 (31%)
14	CLA	bA	1236	-	34,53,73	2.51	13 (38%)	37,89,113	2.38	10 (27%)
14	CLA	bA	1238	-	57,73,73	2.08	14 (24%)	66,113,113	2.41	21 (31%)
14	CLA	bA	1239	-	57,73,73	2.09	16 (28%)	66,113,113	2.44	21 (31%)
16	PQN	bA	2002	-	34,34,34	1.32	2 (5%)	42,45,45	1.17	1 (2%)
18	BCR	bA	4004	-	41,41,41	2.90	6 (14%)	56,56,56	6.47	25 (44%)
18	BCR	bA	4005	-	41,41,41	2.89	7 (17%)	56,56,56	6.63	28 (50%)
18	BCR	bA	4006	-	41,41,41	2.88	6 (14%)	56,56,56	6.55	22 (39%)
18	BCR	bA	4009	-	41,41,41	2.95	6 (14%)	56,56,56	6.57	23 (41%)
18	BCR	bA	4010	-	41,41,41	3.01	7 (17%)	56,56,56	6.58	22 (39%)
18	BCR	bA	4012	-	41,41,41	2.79	6 (14%)	56,56,56	6.62	27 (48%)
18	BCR	bA	4013	-	41,41,41	2.85	6 (14%)	56,56,56	6.57	24 (42%)
18	BCR	bA	4014	-	41,41,41	3.01	6 (14%)	56,56,56	6.45	24 (42%)
18	BCR	bA	4016	-	41,41,41	2.88	6 (14%)	56,56,56	6.50	22 (39%)
18	BCR	bA	4017	-	41,41,41	3.02	6 (14%)	56,56,56	6.60	19 (33%)
20	LMG	bA	5002	-	46,46,55	1.25	5 (10%)	54,54,63	1.16	3 (5%)
19	LHG	bA	5004	-	43,43,48	0.96	2 (4%)	46,49,54	1.18	3 (6%)
17	SF4	cA	3002	-	0,12,12	0.00	-	-		

PRELIMINARY VALIDATION REPORT

PRELIMINARY VALIDATION REPORT

Mol	Type	Chain	Res	Link	Bond lengths			Bond angles		
					Counts	RMSZ	#|Z| > 2	Counts	RMSZ	#|Z| > 2
17	SF4	cA	3003	-	0,12,12	0.00	-	-		
18	BCR	iA	4018	-	41,41,41	3.15	8 (19%)	56,56,56	6.70	23 (41%)
18	BCR	iA	4020	-	41,41,41	3.19	10 (24%)	56,56,56	6.31	25 (44%)
20	LMG	iA	5006	-	37,37,55	1.12	2 (5%)	45,45,63	1.19	4 (8%)
14	CLA	kA	1401	-	34,53,73	2.68	15 (44%)	37,89,113	2.32	9 (24%)
14	CLA	lA	1501	-	52,68,73	2.17	15 (28%)	60,107,113	2.60	19 (31%)
14	CLA	lA	1502	-	52,68,73	2.10	14 (26%)	60,107,113	2.31	17 (28%)
14	CLA	lA	1503	-	57,73,73	2.06	14 (24%)	66,113,113	2.19	17 (25%)
18	BCR	lA	4019	-	41,41,41	2.93	6 (14%)	56,56,56	6.55	23 (41%)
18	BCR	lA	4022	-	41,41,41	3.10	9 (21%)	56,56,56	6.48	24 (42%)
20	LMG	lA	5007	-	50,50,55	1.32	7 (14%)	58,58,63	1.08	2 (3%)
18	BCR	mA	4021	-	41,41,41	2.97	6 (14%)	56,56,56	6.55	23 (41%)
14	CLA	xA	1701	-	34,53,73	2.57	13 (38%)	37,89,113	2.34	12 (32%)

In the following table, the Chirals column lists the number of chiral outliers, the number of chiral centers analysed, the number of these observed in the model and the number defined in the Chemical Component Dictionary. Similar counts are reported in the Torsion and Rings columns. '-' means no outliers of that kind were identified.

Mol	Type	Chain	Res	Link	Chirals	Torsions	Rings
14	CLA	0	1012	-	3/3/20/25	16/37/135/135	-
13	CL0	AA	1011	-	3/3/20/25	9/37/135/135	-
14	CLA	AA	1012	-	3/3/20/25	15/37/135/135	-
14	CLA	AA	1013	-	3/3/19/25	13/31/129/135	-
14	CLA	AA	1101	-	3/3/16/25	4/11/111/135	-
14	CLA	AA	1102	-	3/3/19/25	13/31/129/135	-
14	CLA	AA	1103	-	2/2/19/25	17/31/129/135	-
14	CLA	AA	1104	-	3/3/20/25	12/37/135/135	-
14	CLA	AA	1105	-	3/3/16/25	3/11/111/135	-
14	CLA	AA	1106	-	3/3/16/25	7/11/111/135	-
14	CLA	AA	1107	-	3/3/16/25	3/11/111/135	-
14	CLA	AA	1108	-	3/3/16/25	4/11/111/135	-
14	CLA	AA	1109	-	3/3/16/25	3/11/111/135	-
14	CLA	AA	1110	-	3/3/16/25	4/11/111/135	-
14	CLA	AA	1111	-	3/3/19/25	13/31/129/135	-
14	CLA	AA	1112	-	3/3/16/25	4/11/111/135	-

Continued on next page...

Continued from previous page...

Mol	Type	Chain	Res	Link	Chirals	Torsions	Rings
14	CLA	AA	1113	-	3/3/16/25	7/11/111/135	-
14	CLA	AA	1114	-	3/3/16/25	5/11/111/135	-
14	CLA	AA	1115	-	3/3/18/25	9/25/123/135	-
14	CLA	AA	1116	-	3/3/18/25	10/25/123/135	-
14	CLA	AA	1117	-	3/3/20/25	17/37/135/135	-
14	CLA	AA	1118	-	3/3/18/25	12/25/123/135	-
14	CLA	AA	1119	-	3/3/20/25	17/37/135/135	-
14	CLA	AA	1122	-	3/3/18/25	10/25/123/135	-
14	CLA	AA	1123	-	3/3/19/25	8/31/129/135	-
14	CLA	AA	1124	-	3/3/18/25	7/25/123/135	-
14	CLA	AA	1125	-	3/3/20/25	22/37/135/135	-
14	CLA	AA	1126	-	3/3/19/25	24/31/129/135	-
14	CLA	AA	1127	-	3/3/20/25	18/37/135/135	-
14	CLA	AA	1128	-	3/3/19/25	11/31/129/135	-
14	CLA	AA	1129	1	3/3/16/25	4/11/111/135	-
14	CLA	AA	1130	-	3/3/18/25	8/25/123/135	-
14	CLA	AA	1131	1	3/3/19/25	15/31/129/135	-
14	CLA	AA	1132	-	3/3/20/25	18/37/135/135	-
14	CLA	AA	1133	-	3/3/19/25	17/31/129/135	-
14	CLA	AA	1135	-	3/3/17/25	4/19/117/135	-
14	CLA	AA	1136	-	3/3/18/25	14/25/123/135	-
14	CLA	AA	1137	-	3/3/16/25	5/11/111/135	-
14	CLA	AA	1138	-	3/3/19/25	15/31/129/135	-
14	CLA	AA	1139	-	3/3/16/25	4/11/111/135	-
14	CLA	AA	1140	-	3/3/20/25	20/37/135/135	-
16	PQN	AA	2001	-	-	9/23/43/43	0/2/2/2
17	SF4	AA	3001	-	-	-	0/6/5/5
18	BCR	AA	4001	-	-	12/29/63/63	0/2/2/2
18	BCR	AA	4002	-	-	9/29/63/63	0/2/2/2
18	BCR	AA	4003	-	-	14/29/63/63	0/2/2/2
18	BCR	AA	4007	-	-	14/29/63/63	0/2/2/2
18	BCR	AA	4008	-	-	10/29/63/63	0/2/2/2
18	BCR	AA	4011	-	-	15/29/63/63	0/2/2/2
19	LHG	AA	5001	-	-	21/46/46/53	-

Continued on next page...

PRELIMINARY VALIDATION REPORT

Continued from previous page...

Mol	Type	Chain	Res	Link	Chirals	Torsions	Rings
20	LMG	AA	5005	-	-	17/41/61/70	0/1/1/1
21	LMT	AA	6001	-	-	9/17/57/61	0/2/2/2
21	LMT	AA	6002	-	-	3/14/54/61	0/2/2/2
14	CLA	BA	1021	-	3/3/20/25	16/37/135/135	-
14	CLA	BA	1022	-	3/3/20/25	16/37/135/135	-
14	CLA	BA	1023	-	3/3/20/25	15/37/135/135	-
14	CLA	BA	1201	-	3/3/18/25	12/25/123/135	-
14	CLA	BA	1202	-	3/3/19/25	11/31/129/135	-
14	CLA	BA	1203	-	3/3/20/25	17/37/135/135	-
14	CLA	BA	1204	2	3/3/20/25	20/37/135/135	-
14	CLA	BA	1205	-	3/3/20/25	15/37/135/135	-
14	CLA	BA	1206	-	3/3/16/25	6/11/111/135	-
14	CLA	BA	1207	-	3/3/16/25	7/11/111/135	-
14	CLA	BA	1208	-	3/3/20/25	10/37/135/135	-
14	CLA	BA	1209	-	3/3/16/25	2/11/111/135	-
14	CLA	BA	1210	-	3/3/20/25	11/37/135/135	-
14	CLA	BA	1211	-	3/3/20/25	17/37/135/135	-
14	CLA	BA	1212	-	3/3/16/25	7/11/111/135	-
14	CLA	BA	1213	-	3/3/18/25	14/25/123/135	-
14	CLA	BA	1214	-	3/3/19/25	16/31/129/135	-
14	CLA	BA	1215	-	3/3/19/25	18/31/129/135	-
14	CLA	BA	1216	-	3/3/18/25	10/25/123/135	-
14	CLA	BA	1217	-	3/3/18/25	10/25/123/135	-
14	CLA	BA	1218	-	3/3/16/25	4/11/111/135	-
14	CLA	BA	1219	-	3/3/16/25	5/11/111/135	-
14	CLA	BA	1220	-	3/3/18/25	12/25/123/135	-
14	CLA	BA	1221	-	3/3/19/25	12/31/129/135	-
14	CLA	BA	1222	-	3/3/20/25	16/37/135/135	-
14	CLA	BA	1223	-	3/3/20/25	11/37/135/135	-
14	CLA	BA	1224	-	2/2/20/25	11/37/135/135	-
14	CLA	BA	1225	-	3/3/20/25	16/37/135/135	-
14	CLA	BA	1226	-	3/3/20/25	18/37/135/135	-
14	CLA	BA	1227	-	3/3/16/25	6/11/111/135	-
14	CLA	BA	1228	-	3/3/16/25	5/11/111/135	-

Continued on next page...

PRELIMINARY VALIDATION REPORT

Continued from previous page...

Mol	Type	Chain	Res	Link	Chirals	Torsions	Rings
14	CLA	BA	1229	-	3/3/19/25	15/31/129/135	-
14	CLA	BA	1231	-	3/3/18/25	5/25/123/135	-
14	CLA	BA	1232	-	3/3/16/25	3/11/111/135	-
14	CLA	BA	1233	-	3/3/16/25	5/11/111/135	-
14	CLA	BA	1234	-	3/3/18/25	9/25/123/135	-
14	CLA	BA	1235	-	3/3/19/25	9/31/129/135	-
14	CLA	BA	1236	-	3/3/16/25	5/11/111/135	-
14	CLA	BA	1238	-	3/3/20/25	16/37/135/135	-
14	CLA	BA	1239	-	3/3/20/25	18/37/135/135	-
16	PQN	BA	2002	-	-	12/23/43/43	0/2/2/2
18	BCR	BA	4004	-	-	7/29/63/63	0/2/2/2
18	BCR	BA	4005	-	-	4/29/63/63	0/2/2/2
18	BCR	BA	4006	-	-	11/29/63/63	0/2/2/2
18	BCR	BA	4009	-	-	5/29/63/63	0/2/2/2
18	BCR	BA	4010	-	-	8/29/63/63	0/2/2/2
18	BCR	BA	4012	-	-	20/29/63/63	0/2/2/2
18	BCR	BA	4013	-	-	18/29/63/63	0/2/2/2
18	BCR	BA	4014	-	-	11/29/63/63	0/2/2/2
18	BCR	BA	4016	-	-	15/29/63/63	0/2/2/2
18	BCR	BA	4017	-	-	12/29/63/63	0/2/2/2
20	LMG	BA	5002	-	-	17/41/61/70	0/1/1/1
19	LHG	BA	5004	-	-	21/48/48/53	-
17	SF4	CA	3002	-	-	-	0/6/5/5
17	SF4	CA	3003	-	-	-	0/6/5/5
13	CL0	GA	1011	-	3/3/20/25	9/37/135/135	-
14	CLA	GA	1012	-	3/3/20/25	15/37/135/135	-
14	CLA	GA	1013	-	3/3/19/25	13/31/129/135	-
14	CLA	GA	1101	-	3/3/16/25	4/11/111/135	-
14	CLA	GA	1102	-	3/3/19/25	13/31/129/135	-
14	CLA	GA	1103	-	2/2/19/25	17/31/129/135	-
14	CLA	GA	1104	-	3/3/20/25	12/37/135/135	-
14	CLA	GA	1105	-	3/3/16/25	3/11/111/135	-
14	CLA	GA	1106	-	3/3/16/25	7/11/111/135	-
14	CLA	GA	1107	-	3/3/16/25	3/11/111/135	-

Continued on next page...

PRELIMINARY VALIDATION REPORT

Continued from previous page...

Mol	Type	Chain	Res	Link	Chirals	Torsions	Rings
14	CLA	GA	1108	-	3/3/16/25	4/11/111/135	-
14	CLA	GA	1109	-	3/3/16/25	3/11/111/135	-
14	CLA	GA	1110	-	3/3/16/25	4/11/111/135	-
14	CLA	GA	1111	-	3/3/19/25	13/31/129/135	-
14	CLA	GA	1112	-	3/3/16/25	4/11/111/135	-
14	CLA	GA	1113	-	3/3/16/25	7/11/111/135	-
14	CLA	GA	1114	-	3/3/16/25	5/11/111/135	-
14	CLA	GA	1115	-	3/3/18/25	9/25/123/135	-
14	CLA	GA	1116	-	3/3/18/25	10/25/123/135	-
14	CLA	GA	1117	-	3/3/20/25	17/37/135/135	-
14	CLA	GA	1118	-	3/3/18/25	12/25/123/135	-
14	CLA	GA	1119	-	3/3/20/25	17/37/135/135	-
14	CLA	GA	1122	-	3/3/18/25	10/25/123/135	-
14	CLA	GA	1123	-	3/3/19/25	8/31/129/135	-
14	CLA	GA	1124	-	3/3/18/25	7/25/123/135	-
14	CLA	GA	1125	-	3/3/20/25	22/37/135/135	-
14	CLA	GA	1126	-	3/3/19/25	24/31/129/135	-
14	CLA	GA	1127	-	3/3/20/25	18/37/135/135	-
14	CLA	GA	1128	-	3/3/19/25	11/31/129/135	-
14	CLA	GA	1129	-	3/3/16/25	4/11/111/135	-
14	CLA	GA	1130	-	3/3/18/25	8/25/123/135	-
14	CLA	GA	1131	1	3/3/19/25	15/31/129/135	-
14	CLA	GA	1132	-	3/3/20/25	18/37/135/135	-
14	CLA	GA	1133	-	3/3/19/25	17/31/129/135	-
14	CLA	GA	1135	-	3/3/17/25	4/19/117/135	-
14	CLA	GA	1136	-	3/3/18/25	14/25/123/135	-
14	CLA	GA	1137	-	3/3/16/25	5/11/111/135	-
14	CLA	GA	1138	-	3/3/19/25	15/31/129/135	-
14	CLA	GA	1139	-	3/3/16/25	4/11/111/135	-
14	CLA	GA	1140	-	3/3/20/25	20/37/135/135	-
16	PQN	GA	2001	-	-	9/23/43/43	0/2/2/2
17	SF4	GA	3001	-	-	-	0/6/5/5
18	BCR	GA	4001	-	-	12/29/63/63	0/2/2/2
18	BCR	GA	4002	-	-	9/29/63/63	0/2/2/2

Continued on next page...

PRELIMINARY VALIDATION REPORT

Continued from previous page...

Mol	Type	Chain	Res	Link	Chirals	Torsions	Rings
18	BCR	GA	4003	-	-	14/29/63/63	0/2/2/2
18	BCR	GA	4007	-	-	14/29/63/63	0/2/2/2
18	BCR	GA	4008	-	-	10/29/63/63	0/2/2/2
18	BCR	GA	4011	-	-	15/29/63/63	0/2/2/2
19	LHG	GA	5001	-	-	21/46/46/53	-
20	LMG	GA	5005	-	-	17/41/61/70	0/1/1/1
21	LMT	GA	6001	-	-	9/17/57/61	0/2/2/2
21	LMT	GA	6002	-	-	3/14/54/61	0/2/2/2
14	CLA	HA	1021	-	3/3/20/25	16/37/135/135	-
14	CLA	HA	1023	-	3/3/20/25	15/37/135/135	-
14	CLA	HA	1201	-	3/3/18/25	12/25/123/135	-
14	CLA	HA	1202	-	3/3/19/25	11/31/129/135	-
14	CLA	HA	1203	-	3/3/20/25	17/37/135/135	-
14	CLA	HA	1204	2	3/3/20/25	20/37/135/135	-
14	CLA	HA	1205	-	3/3/20/25	15/37/135/135	-
14	CLA	HA	1206	-	3/3/16/25	6/11/111/135	-
14	CLA	HA	1207	-	3/3/16/25	7/11/111/135	-
14	CLA	HA	1208	-	3/3/20/25	10/37/135/135	-
14	CLA	HA	1209	-	3/3/16/25	2/11/111/135	-
14	CLA	HA	1210	-	3/3/20/25	11/37/135/135	-
14	CLA	HA	1211	-	3/3/20/25	17/37/135/135	-
14	CLA	HA	1212	-	3/3/16/25	7/11/111/135	-
14	CLA	HA	1213	-	3/3/18/25	14/25/123/135	-
14	CLA	HA	1214	-	3/3/19/25	16/31/129/135	-
14	CLA	HA	1215	-	3/3/19/25	18/31/129/135	-
14	CLA	HA	1216	-	3/3/18/25	10/25/123/135	-
14	CLA	HA	1217	-	3/3/18/25	10/25/123/135	-
14	CLA	HA	1218	-	3/3/16/25	4/11/111/135	-
14	CLA	HA	1219	-	3/3/16/25	5/11/111/135	-
14	CLA	HA	1220	-	3/3/18/25	12/25/123/135	-
14	CLA	HA	1221	-	3/3/19/25	12/31/129/135	-
14	CLA	HA	1222	-	3/3/20/25	16/37/135/135	-
14	CLA	HA	1223	-	3/3/20/25	11/37/135/135	-
14	CLA	HA	1224	-	2/2/20/25	11/37/135/135	-

Continued on next page...

PRELIMINARY VALIDATION REPORT

Continued from previous page...

Mol	Type	Chain	Res	Link	Chirals	Torsions	Rings
14	CLA	HA	1225	-	3/3/20/25	16/37/135/135	-
14	CLA	HA	1226	-	3/3/20/25	18/37/135/135	-
14	CLA	HA	1227	-	3/3/16/25	6/11/111/135	-
14	CLA	HA	1228	-	3/3/16/25	5/11/111/135	-
14	CLA	HA	1229	-	3/3/19/25	15/31/129/135	-
14	CLA	HA	1231	-	3/3/18/25	5/25/123/135	-
14	CLA	HA	1232	-	3/3/16/25	3/11/111/135	-
14	CLA	HA	1233	-	3/3/16/25	5/11/111/135	-
14	CLA	HA	1234	-	3/3/18/25	9/25/123/135	-
14	CLA	HA	1235	-	3/3/19/25	9/31/129/135	-
14	CLA	HA	1236	-	3/3/16/25	5/11/111/135	-
14	CLA	HA	1238	-	3/3/20/25	15/37/135/135	-
14	CLA	HA	1239	-	3/3/20/25	18/37/135/135	-
16	PQN	HA	2002	-	-	12/23/43/43	0/2/2/2
18	BCR	HA	4004	-	-	8/29/63/63	0/2/2/2
18	BCR	HA	4005	-	-	4/29/63/63	0/2/2/2
18	BCR	HA	4006	-	-	11/29/63/63	0/2/2/2
18	BCR	HA	4009	-	-	5/29/63/63	0/2/2/2
18	BCR	HA	4010	-	-	8/29/63/63	0/2/2/2
18	BCR	HA	4012	-	-	20/29/63/63	0/2/2/2
18	BCR	HA	4013	-	-	18/29/63/63	0/2/2/2
18	BCR	HA	4014	-	-	11/29/63/63	0/2/2/2
18	BCR	HA	4016	-	-	15/29/63/63	0/2/2/2
18	BCR	HA	4017	-	-	12/29/63/63	0/2/2/2
20	LMG	HA	5002	-	-	17/41/61/70	0/1/1/1
19	LHG	HA	5004	-	-	21/48/48/53	-
18	BCR	IA	4018	-	-	10/29/63/63	0/2/2/2
18	BCR	IA	4020	-	-	11/29/63/63	0/2/2/2
20	LMG	IA	5006	-	-	13/32/52/70	0/1/1/1
14	CLA	KA	1401	-	3/3/16/25	7/11/111/135	-
14	CLA	LA	1501	-	3/3/19/25	12/31/129/135	-
14	CLA	LA	1502	-	3/3/19/25	12/31/129/135	-
14	CLA	LA	1503	-	3/3/20/25	12/37/135/135	-
18	BCR	LA	4019	-	-	7/29/63/63	0/2/2/2

Continued on next page...

PRELIMINARY VALIDATION REPORT

Continued from previous page...

Mol	Type	Chain	Res	Link	Chirals	Torsions	Rings
18	BCR	LA	4022	-	-	7/29/63/63	0/2/2/2
20	LMG	LA	5007	-	-	13/45/65/70	0/1/1/1
18	BCR	MA	4021	-	-	12/29/63/63	0/2/2/2
17	SF4	NA	3002	-	-	-	0/6/5/5
17	SF4	NA	3003	-	-	-	0/6/5/5
18	BCR	RA	4018	-	-	11/29/63/63	0/2/2/2
18	BCR	RA	4020	-	-	11/29/63/63	0/2/2/2
20	LMG	RA	5006	-	-	13/32/52/70	0/1/1/1
14	CLA	TA	1401	-	3/3/16/25	7/11/111/135	-
14	CLA	UA	1501	-	3/3/19/25	12/31/129/135	-
14	CLA	UA	1502	-	3/3/19/25	12/31/129/135	-
14	CLA	UA	1503	-	3/3/20/25	12/37/135/135	-
18	BCR	UA	4019	-	-	7/29/63/63	0/2/2/2
18	BCR	UA	4022	-	-	7/29/63/63	0/2/2/2
20	LMG	UA	5007	-	-	13/45/65/70	0/1/1/1
18	BCR	VA	4021	-	-	12/29/63/63	0/2/2/2
14	CLA	WA	1701	-	3/3/16/25	4/11/111/135	-
14	CLA	XA	1701	-	3/3/16/25	4/11/111/135	-
13	CL0	aA	1011	-	3/3/20/25	9/37/135/135	-
14	CLA	aA	1012	-	3/3/20/25	15/37/135/135	-
14	CLA	aA	1013	-	3/3/19/25	13/31/129/135	-
14	CLA	aA	1101	-	3/3/16/25	4/11/111/135	-
14	CLA	aA	1102	-	3/3/19/25	13/31/129/135	-
14	CLA	aA	1103	-	2/2/19/25	17/31/129/135	-
14	CLA	aA	1104	-	3/3/20/25	12/37/135/135	-
14	CLA	aA	1105	-	3/3/16/25	3/11/111/135	-
14	CLA	aA	1106	-	3/3/16/25	7/11/111/135	-
14	CLA	aA	1107	-	3/3/16/25	3/11/111/135	-
14	CLA	aA	1108	-	3/3/16/25	4/11/111/135	-
14	CLA	aA	1109	-	3/3/16/25	3/11/111/135	-
14	CLA	aA	1110	-	3/3/16/25	4/11/111/135	-
14	CLA	aA	1111	-	3/3/19/25	13/31/129/135	-
14	CLA	aA	1112	-	3/3/16/25	4/11/111/135	-
14	CLA	aA	1113	-	3/3/16/25	7/11/111/135	-

Continued on next page...

PRELIMINARY VALIDATION REPORT

Continued from previous page...

Mol	Type	Chain	Res	Link	Chirals	Torsions	Rings
14	CLA	aA	1114	-	3/3/16/25	5/11/111/135	-
14	CLA	aA	1115	-	3/3/18/25	9/25/123/135	-
14	CLA	aA	1116	-	3/3/18/25	10/25/123/135	-
14	CLA	aA	1117	-	3/3/20/25	17/37/135/135	-
14	CLA	aA	1118	-	3/3/18/25	12/25/123/135	-
14	CLA	aA	1119	-	3/3/20/25	17/37/135/135	-
14	CLA	aA	1122	-	3/3/18/25	10/25/123/135	-
14	CLA	aA	1123	-	3/3/19/25	8/31/129/135	-
14	CLA	aA	1124	-	3/3/18/25	7/25/123/135	-
14	CLA	aA	1125	-	3/3/20/25	22/37/135/135	-
14	CLA	aA	1126	-	3/3/19/25	24/31/129/135	-
14	CLA	aA	1127	-	3/3/20/25	18/37/135/135	-
14	CLA	aA	1128	-	3/3/19/25	11/31/129/135	-
14	CLA	aA	1129	1	3/3/16/25	4/11/111/135	-
14	CLA	aA	1130	-	3/3/18/25	8/25/123/135	-
14	CLA	aA	1131	1	3/3/19/25	15/31/129/135	-
14	CLA	aA	1132	-	3/3/20/25	18/37/135/135	-
14	CLA	aA	1133	-	3/3/19/25	17/31/129/135	-
14	CLA	aA	1135	-	3/3/17/25	4/19/117/135	-
14	CLA	aA	1136	-	3/3/18/25	14/25/123/135	-
14	CLA	aA	1137	-	3/3/16/25	5/11/111/135	-
14	CLA	aA	1138	-	3/3/19/25	15/31/129/135	-
14	CLA	aA	1139	-	3/3/16/25	4/11/111/135	-
14	CLA	aA	1140	-	3/3/20/25	20/37/135/135	-
16	PQN	aA	2001	-	-	9/23/43/43	0/2/2/2
17	SF4	aA	3001	-	-	-	0/6/5/5
18	BCR	aA	4001	-	-	12/29/63/63	0/2/2/2
18	BCR	aA	4002	-	-	9/29/63/63	0/2/2/2
18	BCR	aA	4003	-	-	14/29/63/63	0/2/2/2
18	BCR	aA	4007	-	-	14/29/63/63	0/2/2/2
18	BCR	aA	4008	-	-	10/29/63/63	0/2/2/2
18	BCR	aA	4011	-	-	15/29/63/63	0/2/2/2
19	LHG	aA	5001	-	-	21/46/46/53	-
20	LMG	aA	5005	-	-	17/41/61/70	0/1/1/1

Continued on next page...

PRELIMINARY VALIDATION REPORT

Continued from previous page...

Mol	Type	Chain	Res	Link	Chirals	Torsions	Rings
21	LMT	aA	6001	-	-	9/17/57/61	0/2/2/2
21	LMT	aA	6002	-	-	3/14/54/61	0/2/2/2
14	CLA	bA	1021	-	3/3/20/25	16/37/135/135	-
14	CLA	bA	1022	-	3/3/20/25	16/37/135/135	-
14	CLA	bA	1023	-	3/3/20/25	15/37/135/135	-
14	CLA	bA	1201	-	3/3/18/25	12/25/123/135	-
14	CLA	bA	1202	-	3/3/19/25	11/31/129/135	-
14	CLA	bA	1203	-	3/3/20/25	17/37/135/135	-
14	CLA	bA	1204	2	3/3/20/25	20/37/135/135	-
14	CLA	bA	1205	-	3/3/20/25	15/37/135/135	-
14	CLA	bA	1206	-	3/3/16/25	6/11/111/135	-
14	CLA	bA	1207	-	3/3/16/25	7/11/111/135	-
14	CLA	bA	1208	-	3/3/20/25	10/37/135/135	-
14	CLA	bA	1209	-	3/3/16/25	2/11/111/135	-
14	CLA	bA	1210	-	3/3/20/25	11/37/135/135	-
14	CLA	bA	1211	-	3/3/20/25	17/37/135/135	-
14	CLA	bA	1212	-	3/3/16/25	7/11/111/135	-
14	CLA	bA	1213	-	3/3/18/25	14/25/123/135	-
14	CLA	bA	1214	-	3/3/19/25	16/31/129/135	-
14	CLA	bA	1215	-	3/3/19/25	18/31/129/135	-
14	CLA	bA	1216	-	3/3/18/25	10/25/123/135	-
14	CLA	bA	1217	-	3/3/18/25	10/25/123/135	-
14	CLA	bA	1218	-	3/3/16/25	4/11/111/135	-
14	CLA	bA	1219	-	3/3/16/25	5/11/111/135	-
14	CLA	bA	1220	-	3/3/18/25	12/25/123/135	-
14	CLA	bA	1221	-	3/3/19/25	12/31/129/135	-
14	CLA	bA	1222	-	3/3/20/25	16/37/135/135	-
14	CLA	bA	1223	-	3/3/20/25	11/37/135/135	-
14	CLA	bA	1224	-	2/2/20/25	11/37/135/135	-
14	CLA	bA	1225	-	3/3/20/25	16/37/135/135	-
14	CLA	bA	1226	-	3/3/20/25	18/37/135/135	-
14	CLA	bA	1227	-	3/3/16/25	6/11/111/135	-
14	CLA	bA	1228	-	3/3/16/25	5/11/111/135	-
14	CLA	bA	1229	-	3/3/19/25	15/31/129/135	-

Continued on next page...

PRELIMINARY VALIDATION REPORT

Continued from previous page...

Mol	Type	Chain	Res	Link	Chirals	Torsions	Rings
14	CLA	bA	1231	-	3/3/18/25	5/25/123/135	-
14	CLA	bA	1232	-	3/3/16/25	3/11/111/135	-
14	CLA	bA	1233	-	3/3/16/25	5/11/111/135	-
14	CLA	bA	1234	-	3/3/18/25	9/25/123/135	-
14	CLA	bA	1235	-	3/3/19/25	9/31/129/135	-
14	CLA	bA	1236	-	3/3/16/25	5/11/111/135	-
14	CLA	bA	1238	-	3/3/20/25	16/37/135/135	-
14	CLA	bA	1239	-	3/3/20/25	18/37/135/135	-
16	PQN	bA	2002	-	-	12/23/43/43	0/2/2/2
18	BCR	bA	4004	-	-	7/29/63/63	0/2/2/2
18	BCR	bA	4005	-	-	4/29/63/63	0/2/2/2
18	BCR	bA	4006	-	-	11/29/63/63	0/2/2/2
18	BCR	bA	4009	-	-	5/29/63/63	0/2/2/2
18	BCR	bA	4010	-	-	8/29/63/63	0/2/2/2
18	BCR	bA	4012	-	-	19/29/63/63	0/2/2/2
18	BCR	bA	4013	-	-	19/29/63/63	0/2/2/2
18	BCR	bA	4014	-	-	11/29/63/63	0/2/2/2
18	BCR	bA	4016	-	-	15/29/63/63	0/2/2/2
18	BCR	bA	4017	-	-	12/29/63/63	0/2/2/2
20	LMG	bA	5002	-	-	17/41/61/70	0/1/1/1
19	LHG	bA	5004	-	-	21/48/48/53	-
17	SF4	cA	3002	-	-	-	0/6/5/5
17	SF4	cA	3003	-	-	-	0/6/5/5
18	BCR	iA	4018	-	-	11/29/63/63	0/2/2/2
18	BCR	iA	4020	-	-	11/29/63/63	0/2/2/2
20	LMG	iA	5006	-	-	13/32/52/70	0/1/1/1
14	CLA	kA	1401	-	3/3/16/25	7/11/111/135	-
14	CLA	lA	1501	-	3/3/19/25	12/31/129/135	-
14	CLA	lA	1502	-	3/3/19/25	12/31/129/135	-
14	CLA	lA	1503	-	3/3/20/25	12/37/135/135	-
18	BCR	lA	4019	-	-	7/29/63/63	0/2/2/2
18	BCR	lA	4022	-	-	7/29/63/63	0/2/2/2
20	LMG	lA	5007	-	-	13/45/65/70	0/1/1/1
18	BCR	mA	4021	-	-	12/29/63/63	0/2/2/2

Continued on next page...

PRELIMINARY VALIDATION REPORT

Continued from previous page...

Mol	Type	Chain	Res	Link	Chirals	Torsions	Rings
14	CLA	xA	1701	-	3/3/16/25	4/11/111/135	-

All (4119) bond length outliers are listed below:

Mol	Chain	Res	Type	Atoms	Z	Observed(Å)	Ideal(Å)
18	RA	4020	BCR	C10-C9	-9.32	1.23	1.35
18	iA	4020	BCR	C10-C9	-9.31	1.23	1.35
18	IA	4020	BCR	C10-C9	-9.30	1.23	1.35
18	AA	4008	BCR	C10-C9	-9.22	1.23	1.35
18	GA	4008	BCR	C10-C9	-9.20	1.23	1.35
18	aA	4008	BCR	C10-C9	-9.15	1.23	1.35
18	iA	4018	BCR	C10-C9	-8.99	1.23	1.35
18	IA	4018	BCR	C10-C9	-8.97	1.23	1.35
18	RA	4018	BCR	C10-C9	-8.96	1.23	1.35
18	GA	4008	BCR	C11-C10	-8.88	1.16	1.43
18	AA	4008	BCR	C11-C10	-8.87	1.16	1.43
18	aA	4008	BCR	C11-C10	-8.86	1.16	1.43
18	GA	4002	BCR	C10-C9	-8.85	1.24	1.35
18	aA	4002	BCR	C10-C9	-8.83	1.24	1.35
18	AA	4002	BCR	C10-C9	-8.82	1.24	1.35
18	LA	4022	BCR	C11-C10	-8.77	1.16	1.43
18	UA	4022	BCR	C11-C10	-8.76	1.16	1.43
18	iA	4018	BCR	C11-C10	-8.75	1.16	1.43
18	lA	4022	BCR	C11-C10	-8.73	1.16	1.43
18	aA	4008	BCR	C8-C9	-8.73	1.27	1.45
18	IA	4018	BCR	C11-C10	-8.72	1.17	1.43
18	AA	4008	BCR	C8-C9	-8.72	1.27	1.45
18	GA	4008	BCR	C8-C9	-8.71	1.27	1.45
18	RA	4018	BCR	C11-C10	-8.71	1.17	1.43
18	RA	4020	BCR	C11-C10	-8.62	1.17	1.43
18	iA	4020	BCR	C11-C10	-8.59	1.17	1.43
18	IA	4020	BCR	C11-C10	-8.58	1.17	1.43
18	lA	4022	BCR	C8-C9	-8.58	1.27	1.45
18	aA	4007	BCR	C11-C10	-8.57	1.17	1.43
18	LA	4022	BCR	C8-C9	-8.57	1.27	1.45
18	MA	4021	BCR	C11-C10	-8.57	1.17	1.43
18	GA	4007	BCR	C11-C10	-8.56	1.17	1.43
18	HA	4017	BCR	C11-C10	-8.56	1.17	1.43
18	BA	4017	BCR	C11-C10	-8.56	1.17	1.43
18	VA	4021	BCR	C11-C10	-8.55	1.17	1.43
18	UA	4022	BCR	C8-C9	-8.55	1.27	1.45
18	bA	4017	BCR	C11-C10	-8.55	1.17	1.43

Continued on next page...

PRELIMINARY VALIDATION REPORT

Continued from previous page...

Mol	Chain	Res	Type	Atoms	Z	Observed(Å)	Ideal(Å)
18	IA	4018	BCR	C8-C9	-8.53	1.27	1.45
18	mA	4021	BCR	C11-C10	-8.53	1.17	1.43
18	GA	4001	BCR	C11-C10	-8.53	1.17	1.43
18	AA	4007	BCR	C11-C10	-8.53	1.17	1.43
18	bA	4014	BCR	C11-C10	-8.53	1.17	1.43
18	BA	4014	BCR	C11-C10	-8.53	1.17	1.43
18	aA	4001	BCR	C11-C10	-8.52	1.17	1.43
18	lA	4022	BCR	C10-C9	-8.52	1.24	1.35
18	aA	4007	BCR	C8-C9	-8.52	1.27	1.45
18	RA	4018	BCR	C8-C9	-8.52	1.27	1.45
18	AA	4001	BCR	C11-C10	-8.51	1.17	1.43
18	HA	4014	BCR	C11-C10	-8.51	1.17	1.43
18	bA	4016	BCR	C11-C10	-8.51	1.17	1.43
18	iA	4018	BCR	C8-C9	-8.50	1.27	1.45
18	UA	4022	BCR	C10-C9	-8.50	1.24	1.35
18	VA	4021	BCR	C8-C9	-8.50	1.27	1.45
18	GA	4007	BCR	C8-C9	-8.50	1.27	1.45
18	GA	4011	BCR	C8-C9	-8.49	1.27	1.45
18	AA	4007	BCR	C8-C9	-8.49	1.27	1.45
18	AA	4011	BCR	C11-C10	-8.49	1.17	1.43
18	AA	4011	BCR	C8-C9	-8.49	1.27	1.45
18	mA	4021	BCR	C8-C9	-8.48	1.27	1.45
18	HA	4016	BCR	C11-C10	-8.48	1.17	1.43
18	LA	4022	BCR	C10-C9	-8.47	1.24	1.35
18	GA	4011	BCR	C11-C10	-8.47	1.17	1.43
18	BA	4016	BCR	C11-C10	-8.47	1.17	1.43
18	mA	4021	BCR	C10-C9	-8.47	1.24	1.35
18	aA	4011	BCR	C11-C10	-8.46	1.17	1.43
18	aA	4002	BCR	C8-C9	-8.45	1.27	1.45
18	MA	4021	BCR	C8-C9	-8.45	1.27	1.45
18	aA	4011	BCR	C8-C9	-8.45	1.27	1.45
18	MA	4021	BCR	C10-C9	-8.44	1.24	1.35
18	AA	4001	BCR	C8-C9	-8.44	1.27	1.45
18	BA	4010	BCR	C11-C10	-8.44	1.17	1.43
18	VA	4021	BCR	C10-C9	-8.44	1.24	1.35
18	GA	4001	BCR	C8-C9	-8.44	1.27	1.45
18	BA	4009	BCR	C11-C10	-8.43	1.17	1.43
18	RA	4020	BCR	C8-C9	-8.43	1.27	1.45
18	bA	4009	BCR	C11-C10	-8.43	1.17	1.43
18	bA	4010	BCR	C11-C10	-8.42	1.17	1.43
18	aA	4001	BCR	C8-C9	-8.42	1.27	1.45
18	GA	4002	BCR	C8-C9	-8.42	1.27	1.45

Continued on next page...

PRELIMINARY VALIDATION REPORT

Continued from previous page...

Mol	Chain	Res	Type	Atoms	Z	Observed(Å)	Ideal(Å)
18	IA	4020	BCR	C8-C9	-8.42	1.27	1.45
18	HA	4009	BCR	C11-C10	-8.42	1.17	1.43
18	HA	4010	BCR	C11-C10	-8.42	1.17	1.43
18	AA	4002	BCR	C8-C9	-8.40	1.27	1.45
18	GA	4007	BCR	C10-C9	-8.40	1.24	1.35
18	AA	4007	BCR	C10-C9	-8.39	1.24	1.35
18	bA	4017	BCR	C8-C9	-8.37	1.27	1.45
18	BA	4010	BCR	C8-C9	-8.37	1.27	1.45
18	iA	4020	BCR	C8-C9	-8.37	1.27	1.45
18	BA	4017	BCR	C8-C9	-8.37	1.27	1.45
18	HA	4010	BCR	C8-C9	-8.37	1.27	1.45
18	AA	4003	BCR	C11-C10	-8.36	1.18	1.43
18	HA	4017	BCR	C10-C9	-8.36	1.24	1.35
18	bA	4010	BCR	C8-C9	-8.36	1.27	1.45
18	aA	4003	BCR	C11-C10	-8.35	1.18	1.43
18	GA	4003	BCR	C11-C10	-8.35	1.18	1.43
18	aA	4007	BCR	C10-C9	-8.35	1.24	1.35
18	HA	4010	BCR	C10-C9	-8.34	1.24	1.35
18	bA	4004	BCR	C11-C10	-8.34	1.18	1.43
18	bA	4010	BCR	C10-C9	-8.34	1.24	1.35
18	UA	4019	BCR	C11-C10	-8.34	1.18	1.43
18	BA	4004	BCR	C11-C10	-8.34	1.18	1.43
18	BA	4010	BCR	C10-C9	-8.33	1.24	1.35
18	HA	4004	BCR	C11-C10	-8.32	1.18	1.43
18	HA	4017	BCR	C8-C9	-8.32	1.27	1.45
18	LA	4019	BCR	C11-C10	-8.31	1.18	1.43
18	HA	4014	BCR	C10-C9	-8.31	1.24	1.35
18	bA	4017	BCR	C10-C9	-8.31	1.24	1.35
18	lA	4019	BCR	C11-C10	-8.31	1.18	1.43
18	HA	4005	BCR	C11-C10	-8.31	1.18	1.43
18	bA	4005	BCR	C11-C10	-8.30	1.18	1.43
18	BA	4014	BCR	C10-C9	-8.30	1.24	1.35
18	HA	4013	BCR	C11-C10	-8.30	1.18	1.43
18	BA	4017	BCR	C10-C9	-8.30	1.24	1.35
18	BA	4005	BCR	C11-C10	-8.29	1.18	1.43
18	BA	4014	BCR	C8-C9	-8.28	1.28	1.45
18	HA	4014	BCR	C8-C9	-8.28	1.28	1.45
18	bA	4014	BCR	C8-C9	-8.28	1.28	1.45
18	GA	4003	BCR	C8-C9	-8.28	1.28	1.45
18	BA	4013	BCR	C11-C10	-8.28	1.18	1.43
18	aA	4003	BCR	C8-C9	-8.28	1.28	1.45
18	bA	4013	BCR	C11-C10	-8.28	1.18	1.43

Continued on next page...

PRELIMINARY VALIDATION REPORT

Continued from previous page...

Mol	Chain	Res	Type	Atoms	Z	Observed(Å)	Ideal(Å)
18	bA	4012	BCR	C8-C9	-8.28	1.28	1.45
18	HA	4006	BCR	C8-C9	-8.27	1.28	1.45
18	AA	4003	BCR	C8-C9	-8.27	1.28	1.45
18	bA	4014	BCR	C10-C9	-8.25	1.24	1.35
18	bA	4006	BCR	C8-C9	-8.24	1.28	1.45
18	AA	4001	BCR	C10-C9	-8.23	1.24	1.35
18	bA	4004	BCR	C8-C9	-8.22	1.28	1.45
18	BA	4012	BCR	C8-C9	-8.22	1.28	1.45
18	HA	4016	BCR	C8-C9	-8.22	1.28	1.45
18	HA	4012	BCR	C8-C9	-8.22	1.28	1.45
18	BA	4016	BCR	C8-C9	-8.22	1.28	1.45
18	aA	4001	BCR	C10-C9	-8.22	1.24	1.35
18	HA	4006	BCR	C11-C10	-8.21	1.18	1.43
18	BA	4006	BCR	C11-C10	-8.21	1.18	1.43
18	HA	4004	BCR	C8-C9	-8.21	1.28	1.45
18	bA	4006	BCR	C11-C10	-8.21	1.18	1.43
18	BA	4004	BCR	C8-C9	-8.21	1.28	1.45
18	bA	4016	BCR	C8-C9	-8.20	1.28	1.45
18	BA	4006	BCR	C8-C9	-8.20	1.28	1.45
18	lA	4019	BCR	C8-C9	-8.18	1.28	1.45
18	aA	4011	BCR	C10-C9	-8.16	1.25	1.35
18	GA	4001	BCR	C10-C9	-8.16	1.25	1.35
18	GA	4011	BCR	C10-C9	-8.15	1.25	1.35
18	AA	4008	BCR	C20-C21	-8.14	1.18	1.43
18	bA	4013	BCR	C8-C9	-8.14	1.28	1.45
18	LA	4019	BCR	C8-C9	-8.13	1.28	1.45
18	AA	4002	BCR	C11-C10	-8.13	1.18	1.43
18	HA	4013	BCR	C8-C9	-8.13	1.28	1.45
18	aA	4002	BCR	C11-C10	-8.12	1.18	1.43
18	UA	4019	BCR	C8-C9	-8.12	1.28	1.45
18	BA	4013	BCR	C8-C9	-8.12	1.28	1.45
18	GA	4002	BCR	C11-C10	-8.11	1.18	1.43
18	GA	4008	BCR	C20-C21	-8.08	1.18	1.43
18	aA	4008	BCR	C20-C21	-8.08	1.18	1.43
18	AA	4011	BCR	C10-C9	-8.07	1.25	1.35
18	HA	4009	BCR	C8-C9	-8.06	1.28	1.45
18	IA	4020	BCR	C16-C17	-8.06	1.19	1.43
18	RA	4020	BCR	C16-C17	-8.06	1.19	1.43
18	BA	4009	BCR	C8-C9	-8.05	1.28	1.45
18	iA	4020	BCR	C16-C17	-8.05	1.19	1.43
18	UA	4022	BCR	C20-C21	-8.04	1.19	1.43
18	bA	4009	BCR	C8-C9	-8.03	1.28	1.45

Continued on next page...

PRELIMINARY VALIDATION REPORT

Continued from previous page...

Mol	Chain	Res	Type	Atoms	Z	Observed(Å)	Ideal(Å)
18	bA	4012	BCR	C11-C10	-8.03	1.19	1.43
18	LA	4022	BCR	C20-C21	-8.03	1.19	1.43
18	HA	4012	BCR	C11-C10	-8.03	1.19	1.43
18	RA	4018	BCR	C20-C21	-8.03	1.19	1.43
18	lA	4022	BCR	C20-C21	-8.02	1.19	1.43
18	BA	4012	BCR	C11-C10	-8.02	1.19	1.43
18	AA	4008	BCR	C16-C17	-8.01	1.19	1.43
18	GA	4011	BCR	C20-C21	-8.01	1.19	1.43
18	GA	4008	BCR	C16-C17	-8.01	1.19	1.43
18	IA	4018	BCR	C20-C21	-8.00	1.19	1.43
18	aA	4008	BCR	C16-C17	-8.00	1.19	1.43
18	iA	4018	BCR	C20-C21	-7.98	1.19	1.43
18	AA	4011	BCR	C20-C21	-7.98	1.19	1.43
18	aA	4011	BCR	C20-C21	-7.97	1.19	1.43
18	HA	4009	BCR	C20-C21	-7.97	1.19	1.43
18	BA	4009	BCR	C20-C21	-7.96	1.19	1.43
18	bA	4009	BCR	C20-C21	-7.95	1.19	1.43
18	LA	4022	BCR	C16-C17	-7.93	1.19	1.43
18	lA	4019	BCR	C10-C9	-7.93	1.25	1.35
18	UA	4022	BCR	C16-C17	-7.93	1.19	1.43
18	IA	4020	BCR	C20-C21	-7.91	1.19	1.43
18	iA	4020	BCR	C20-C21	-7.91	1.19	1.43
18	RA	4020	BCR	C20-C21	-7.90	1.19	1.43
18	HA	4005	BCR	C8-C9	-7.90	1.28	1.45
18	lA	4022	BCR	C16-C17	-7.90	1.19	1.43
18	iA	4018	BCR	C16-C17	-7.89	1.19	1.43
18	LA	4019	BCR	C10-C9	-7.89	1.25	1.35
18	HA	4010	BCR	C16-C17	-7.89	1.19	1.43
18	BA	4017	BCR	C20-C21	-7.88	1.19	1.43
18	BA	4005	BCR	C8-C9	-7.88	1.28	1.45
18	UA	4019	BCR	C10-C9	-7.88	1.25	1.35
18	IA	4018	BCR	C16-C17	-7.88	1.19	1.43
18	RA	4018	BCR	C16-C17	-7.87	1.19	1.43
18	HA	4017	BCR	C20-C21	-7.87	1.19	1.43
18	BA	4010	BCR	C20-C21	-7.87	1.19	1.43
18	HA	4014	BCR	C16-C17	-7.86	1.19	1.43
18	bA	4014	BCR	C16-C17	-7.86	1.19	1.43
18	bA	4005	BCR	C8-C9	-7.86	1.28	1.45
18	bA	4009	BCR	C10-C9	-7.86	1.25	1.35
18	bA	4017	BCR	C20-C21	-7.86	1.19	1.43
18	BA	4010	BCR	C16-C17	-7.86	1.19	1.43
18	HA	4010	BCR	C20-C21	-7.86	1.19	1.43

Continued on next page...

PRELIMINARY VALIDATION REPORT

Continued from previous page...

Mol	Chain	Res	Type	Atoms	Z	Observed(Å)	Ideal(Å)
18	bA	4010	BCR	C16-C17	-7.85	1.19	1.43
18	BA	4014	BCR	C20-C21	-7.85	1.19	1.43
18	BA	4014	BCR	C16-C17	-7.85	1.19	1.43
18	BA	4009	BCR	C10-C9	-7.85	1.25	1.35
18	HA	4009	BCR	C10-C9	-7.84	1.25	1.35
18	bA	4010	BCR	C20-C21	-7.84	1.19	1.43
18	HA	4014	BCR	C20-C21	-7.83	1.19	1.43
18	bA	4014	BCR	C20-C21	-7.82	1.19	1.43
18	HA	4017	BCR	C16-C17	-7.81	1.19	1.43
18	bA	4017	BCR	C16-C17	-7.80	1.19	1.43
18	aA	4003	BCR	C20-C21	-7.80	1.19	1.43
18	BA	4017	BCR	C16-C17	-7.79	1.19	1.43
18	GA	4003	BCR	C20-C21	-7.79	1.19	1.43
18	AA	4003	BCR	C20-C21	-7.77	1.19	1.43
18	bA	4009	BCR	C16-C17	-7.77	1.19	1.43
18	BA	4009	BCR	C16-C17	-7.76	1.19	1.43
18	aA	4003	BCR	C10-C9	-7.76	1.25	1.35
18	bA	4006	BCR	C10-C9	-7.75	1.25	1.35
18	HA	4006	BCR	C10-C9	-7.75	1.25	1.35
18	AA	4003	BCR	C10-C9	-7.74	1.25	1.35
18	AA	4002	BCR	C20-C21	-7.74	1.20	1.43
18	HA	4009	BCR	C16-C17	-7.74	1.20	1.43
18	bA	4005	BCR	C20-C21	-7.74	1.20	1.43
18	GA	4003	BCR	C10-C9	-7.73	1.25	1.35
18	HA	4016	BCR	C20-C21	-7.73	1.20	1.43
18	AA	4001	BCR	C20-C21	-7.73	1.20	1.43
18	aA	4001	BCR	C16-C17	-7.72	1.20	1.43
18	GA	4003	BCR	C16-C17	-7.72	1.20	1.43
18	HA	4004	BCR	C20-C21	-7.72	1.20	1.43
18	aA	4001	BCR	C20-C21	-7.72	1.20	1.43
18	AA	4007	BCR	C16-C17	-7.72	1.20	1.43
18	GA	4002	BCR	C20-C21	-7.71	1.20	1.43
18	AA	4003	BCR	C16-C17	-7.71	1.20	1.43
18	bA	4004	BCR	C20-C21	-7.71	1.20	1.43
18	GA	4001	BCR	C20-C21	-7.71	1.20	1.43
18	GA	4001	BCR	C16-C17	-7.71	1.20	1.43
18	aA	4002	BCR	C20-C21	-7.71	1.20	1.43
18	BA	4005	BCR	C20-C21	-7.71	1.20	1.43
18	AA	4001	BCR	C16-C17	-7.70	1.20	1.43
18	UA	4019	BCR	C20-C21	-7.70	1.20	1.43
18	BA	4006	BCR	C10-C9	-7.70	1.25	1.35
18	GA	4007	BCR	C16-C17	-7.70	1.20	1.43

Continued on next page...

PRELIMINARY VALIDATION REPORT

Continued from previous page...

Mol	Chain	Res	Type	Atoms	Z	Observed(Å)	Ideal(Å)
18	BA	4016	BCR	C20-C21	-7.70	1.20	1.43
18	bA	4016	BCR	C20-C21	-7.70	1.20	1.43
18	HA	4005	BCR	C20-C21	-7.70	1.20	1.43
18	aA	4002	BCR	C16-C17	-7.70	1.20	1.43
18	LA	4019	BCR	C20-C21	-7.70	1.20	1.43
18	BA	4004	BCR	C20-C21	-7.70	1.20	1.43
18	lA	4019	BCR	C20-C21	-7.70	1.20	1.43
18	aA	4003	BCR	C16-C17	-7.69	1.20	1.43
18	aA	4007	BCR	C16-C17	-7.68	1.20	1.43
18	AA	4007	BCR	C20-C21	-7.68	1.20	1.43
18	AA	4002	BCR	C16-C17	-7.67	1.20	1.43
18	HA	4005	BCR	C10-C9	-7.67	1.25	1.35
18	aA	4007	BCR	C20-C21	-7.67	1.20	1.43
18	bA	4005	BCR	C10-C9	-7.66	1.25	1.35
18	GA	4002	BCR	C16-C17	-7.66	1.20	1.43
18	bA	4013	BCR	C10-C9	-7.66	1.25	1.35
18	BA	4005	BCR	C10-C9	-7.66	1.25	1.35
18	GA	4007	BCR	C20-C21	-7.65	1.20	1.43
18	AA	4011	BCR	C16-C17	-7.65	1.20	1.43
18	lA	4019	BCR	C16-C17	-7.64	1.20	1.43
18	LA	4019	BCR	C16-C17	-7.64	1.20	1.43
18	UA	4019	BCR	C16-C17	-7.64	1.20	1.43
18	GA	4011	BCR	C16-C17	-7.63	1.20	1.43
18	bA	4005	BCR	C16-C17	-7.63	1.20	1.43
18	aA	4011	BCR	C16-C17	-7.63	1.20	1.43
18	BA	4006	BCR	C20-C21	-7.62	1.20	1.43
18	VA	4021	BCR	C16-C17	-7.62	1.20	1.43
18	BA	4013	BCR	C10-C9	-7.62	1.25	1.35
18	bA	4013	BCR	C20-C21	-7.62	1.20	1.43
18	BA	4005	BCR	C16-C17	-7.61	1.20	1.43
18	bA	4004	BCR	C10-C9	-7.61	1.25	1.35
18	HA	4013	BCR	C10-C9	-7.61	1.25	1.35
18	MA	4021	BCR	C16-C17	-7.61	1.20	1.43
18	HA	4004	BCR	C10-C9	-7.61	1.25	1.35
18	bA	4006	BCR	C20-C21	-7.61	1.20	1.43
18	HA	4006	BCR	C20-C21	-7.60	1.20	1.43
18	BA	4004	BCR	C10-C9	-7.60	1.25	1.35
18	mA	4021	BCR	C16-C17	-7.60	1.20	1.43
18	HA	4005	BCR	C16-C17	-7.59	1.20	1.43
18	HA	4013	BCR	C20-C21	-7.58	1.20	1.43
18	BA	4013	BCR	C20-C21	-7.58	1.20	1.43
18	HA	4004	BCR	C16-C17	-7.58	1.20	1.43

Continued on next page...

PRELIMINARY VALIDATION REPORT

Continued from previous page...

Mol	Chain	Res	Type	Atoms	Z	Observed(Å)	Ideal(Å)
14	BA	1223	CLA	MG-NA	7.57	2.24	2.06
14	KA	1401	CLA	MG-NA	7.57	2.24	2.06
14	TA	1401	CLA	MG-NA	7.57	2.24	2.06
18	BA	4004	BCR	C16-C17	-7.56	1.20	1.43
18	bA	4004	BCR	C16-C17	-7.56	1.20	1.43
18	BA	4006	BCR	C16-C17	-7.55	1.20	1.43
18	HA	4006	BCR	C16-C17	-7.55	1.20	1.43
14	kA	1401	CLA	MG-NA	7.55	2.24	2.06
14	bA	1233	CLA	MG-NA	7.54	2.24	2.06
14	HA	1223	CLA	MG-NA	7.54	2.24	2.06
18	bA	4006	BCR	C16-C17	-7.54	1.20	1.43
18	BA	4016	BCR	C16-C17	-7.53	1.20	1.43
18	HA	4016	BCR	C16-C17	-7.53	1.20	1.43
18	bA	4016	BCR	C16-C17	-7.53	1.20	1.43
14	bA	1223	CLA	MG-NA	7.52	2.24	2.06
14	BA	1232	CLA	MG-NA	7.52	2.24	2.06
14	HA	1233	CLA	MG-NA	7.51	2.24	2.06
18	HA	4012	BCR	C20-C21	-7.51	1.20	1.43
14	BA	1233	CLA	MG-NA	7.51	2.24	2.06
18	BA	4012	BCR	C20-C21	-7.50	1.20	1.43
18	BA	4013	BCR	C16-C17	-7.50	1.20	1.43
18	bA	4013	BCR	C16-C17	-7.49	1.20	1.43
14	AA	1110	CLA	MG-NA	7.49	2.24	2.06
18	bA	4012	BCR	C20-C21	-7.48	1.20	1.43
14	BA	1212	CLA	MG-NA	7.48	2.24	2.06
14	AA	1123	CLA	MG-NA	7.48	2.24	2.06
14	bA	1232	CLA	MG-NA	7.47	2.24	2.06
14	GA	1111	CLA	MG-NA	7.47	2.24	2.06
14	HA	1232	CLA	MG-NA	7.47	2.24	2.06
18	HA	4013	BCR	C16-C17	-7.47	1.20	1.43
14	GA	1123	CLA	MG-NA	7.47	2.24	2.06
18	VA	4021	BCR	C20-C21	-7.47	1.20	1.43
14	aA	1110	CLA	MG-NA	7.46	2.24	2.06
18	MA	4021	BCR	C20-C21	-7.46	1.20	1.43
14	AA	1111	CLA	MG-NA	7.46	2.24	2.06
14	GA	1110	CLA	MG-NA	7.46	2.24	2.06
14	HA	1212	CLA	MG-NA	7.46	2.24	2.06
18	mA	4021	BCR	C20-C21	-7.45	1.20	1.43
14	bA	1212	CLA	MG-NA	7.45	2.24	2.06
14	BA	1214	CLA	MG-NA	7.45	2.24	2.06
14	aA	1111	CLA	MG-NA	7.44	2.23	2.06
14	aA	1123	CLA	MG-NA	7.43	2.23	2.06

Continued on next page...

PRELIMINARY VALIDATION REPORT

Continued from previous page...

Mol	Chain	Res	Type	Atoms	Z	Observed(Å)	Ideal(Å)
14	HA	1214	CLA	MG-NA	7.42	2.23	2.06
14	bA	1214	CLA	MG-NA	7.42	2.23	2.06
14	WA	1701	CLA	MG-NA	7.41	2.23	2.06
14	GA	1135	CLA	MG-NA	7.41	2.23	2.06
14	GA	1133	CLA	MG-NA	7.40	2.23	2.06
14	aA	1135	CLA	MG-NA	7.40	2.23	2.06
18	HA	4016	BCR	C10-C9	-7.39	1.26	1.35
14	AA	1135	CLA	MG-NA	7.39	2.23	2.06
14	aA	1114	CLA	MG-NA	7.39	2.23	2.06
14	GA	1140	CLA	MG-NA	7.39	2.23	2.06
14	AA	1140	CLA	MG-NA	7.39	2.23	2.06
18	BA	4016	BCR	C10-C9	-7.39	1.26	1.35
14	aA	1140	CLA	MG-NA	7.39	2.23	2.06
14	HA	1217	CLA	MG-NA	7.38	2.23	2.06
14	aA	1133	CLA	MG-NA	7.38	2.23	2.06
14	aA	1116	CLA	MG-NA	7.38	2.23	2.06
14	AA	1114	CLA	MG-NA	7.38	2.23	2.06
14	GA	1012	CLA	MG-NA	7.38	2.23	2.06
14	GA	1116	CLA	MG-NA	7.38	2.23	2.06
14	GA	1114	CLA	MG-NA	7.38	2.23	2.06
14	AA	1133	CLA	MG-NA	7.38	2.23	2.06
14	BA	1217	CLA	MG-NA	7.37	2.23	2.06
14	AA	1116	CLA	MG-NA	7.37	2.23	2.06
14	BA	1239	CLA	MG-NA	7.37	2.23	2.06
14	bA	1218	CLA	MG-NA	7.37	2.23	2.06
18	bA	4016	BCR	C10-C9	-7.37	1.26	1.35
14	bA	1217	CLA	MG-NA	7.37	2.23	2.06
14	XA	1701	CLA	MG-NA	7.37	2.23	2.06
14	bA	1239	CLA	MG-NA	7.37	2.23	2.06
14	bA	1213	CLA	MG-NA	7.36	2.23	2.06
14	HA	1239	CLA	MG-NA	7.36	2.23	2.06
14	xA	1701	CLA	MG-NA	7.36	2.23	2.06
14	HA	1227	CLA	MG-NA	7.35	2.23	2.06
14	BA	1218	CLA	MG-NA	7.34	2.23	2.06
14	bA	1209	CLA	MG-NA	7.34	2.23	2.06
14	HA	1218	CLA	MG-NA	7.34	2.23	2.06
14	HA	1213	CLA	MG-NA	7.34	2.23	2.06
14	AA	1012	CLA	MG-NA	7.33	2.23	2.06
14	BA	1224	CLA	MG-NA	7.33	2.23	2.06
14	HA	1224	CLA	MG-NA	7.32	2.23	2.06
14	AA	1136	CLA	MG-NA	7.32	2.23	2.06
18	HA	4012	BCR	C16-C17	-7.32	1.21	1.43

Continued on next page...

PRELIMINARY VALIDATION REPORT

Continued from previous page...

Mol	Chain	Res	Type	Atoms	Z	Observed(Å)	Ideal(Å)
14	aA	1130	CLA	MG-NA	7.32	2.23	2.06
14	HA	1209	CLA	MG-NA	7.32	2.23	2.06
18	BA	4012	BCR	C16-C17	-7.32	1.21	1.43
14	aA	1012	CLA	MG-NA	7.32	2.23	2.06
14	aA	1112	CLA	MG-NA	7.32	2.23	2.06
14	AA	1127	CLA	MG-NA	7.32	2.23	2.06
14	bA	1227	CLA	MG-NA	7.32	2.23	2.06
14	BA	1227	CLA	MG-NA	7.31	2.23	2.06
14	AA	1139	CLA	MG-NA	7.31	2.23	2.06
18	bA	4012	BCR	C16-C17	-7.31	1.21	1.43
14	AA	1105	CLA	MG-NA	7.31	2.23	2.06
14	BA	1213	CLA	MG-NA	7.31	2.23	2.06
14	GA	1139	CLA	MG-NA	7.31	2.23	2.06
14	BA	1210	CLA	MG-NA	7.31	2.23	2.06
14	GA	1130	CLA	MG-NA	7.31	2.23	2.06
14	aA	1136	CLA	MG-NA	7.30	2.23	2.06
14	AA	1115	CLA	MG-NA	7.30	2.23	2.06
14	BA	1209	CLA	MG-NA	7.30	2.23	2.06
14	HA	1210	CLA	MG-NA	7.30	2.23	2.06
14	AA	1130	CLA	MG-NA	7.30	2.23	2.06
14	HA	1231	CLA	MG-NA	7.30	2.23	2.06
14	HA	1238	CLA	MG-NA	7.30	2.23	2.06
14	BA	1022	CLA	MG-NA	7.30	2.23	2.06
14	AA	1107	CLA	MG-NA	7.30	2.23	2.06
14	GA	1105	CLA	MG-NA	7.29	2.23	2.06
14	bA	1224	CLA	MG-NA	7.29	2.23	2.06
14	AA	1112	CLA	MG-NA	7.29	2.23	2.06
14	bA	1238	CLA	MG-NA	7.29	2.23	2.06
14	BA	1238	CLA	MG-NA	7.29	2.23	2.06
14	GA	1136	CLA	MG-NA	7.29	2.23	2.06
14	aA	1115	CLA	MG-NA	7.29	2.23	2.06
14	aA	1105	CLA	MG-NA	7.29	2.23	2.06
14	AA	1108	CLA	MG-NA	7.28	2.23	2.06
14	0	1012	CLA	MG-NA	7.28	2.23	2.06
14	UA	1501	CLA	MG-NA	7.28	2.23	2.06
14	HA	1234	CLA	MG-NA	7.28	2.23	2.06
14	BA	1231	CLA	MG-NA	7.28	2.23	2.06
14	bA	1225	CLA	MG-NA	7.28	2.23	2.06
14	AA	1101	CLA	MG-NA	7.28	2.23	2.06
14	aA	1129	CLA	MG-NA	7.27	2.23	2.06
14	GA	1127	CLA	MG-NA	7.27	2.23	2.06
14	GA	1112	CLA	MG-NA	7.27	2.23	2.06

Continued on next page...

PRELIMINARY VALIDATION REPORT

Continued from previous page...

Mol	Chain	Res	Type	Atoms	Z	Observed(Å)	Ideal(Å)
14	bA	1022	CLA	MG-NA	7.27	2.23	2.06
14	bA	1231	CLA	MG-NA	7.27	2.23	2.06
14	HA	1225	CLA	MG-NA	7.27	2.23	2.06
14	bA	1210	CLA	MG-NA	7.27	2.23	2.06
14	aA	1139	CLA	MG-NA	7.27	2.23	2.06
14	GA	1115	CLA	MG-NA	7.27	2.23	2.06
14	BA	1225	CLA	MG-NA	7.27	2.23	2.06
14	aA	1127	CLA	MG-NA	7.27	2.23	2.06
14	GA	1129	CLA	MG-NA	7.27	2.23	2.06
14	LA	1501	CLA	MG-NA	7.26	2.23	2.06
14	AA	1129	CLA	MG-NA	7.26	2.23	2.06
14	BA	1234	CLA	MG-NA	7.26	2.23	2.06
14	bA	1234	CLA	MG-NA	7.25	2.23	2.06
14	aA	1107	CLA	MG-NA	7.25	2.23	2.06
14	HA	1229	CLA	MG-NA	7.25	2.23	2.06
14	GA	1107	CLA	MG-NA	7.24	2.23	2.06
14	aA	1101	CLA	MG-NA	7.24	2.23	2.06
14	aA	1137	CLA	MG-NA	7.24	2.23	2.06
14	GA	1137	CLA	MG-NA	7.24	2.23	2.06
14	AA	1138	CLA	MG-NA	7.24	2.23	2.06
14	BA	1229	CLA	MG-NA	7.23	2.23	2.06
14	aA	1102	CLA	MG-NA	7.23	2.23	2.06
14	GA	1108	CLA	MG-NA	7.23	2.23	2.06
14	aA	1108	CLA	MG-NA	7.23	2.23	2.06
14	lA	1501	CLA	MG-NA	7.23	2.23	2.06
14	bA	1229	CLA	MG-NA	7.23	2.23	2.06
14	GA	1101	CLA	MG-NA	7.23	2.23	2.06
14	AA	1102	CLA	MG-NA	7.22	2.23	2.06
14	bA	1220	CLA	MG-NA	7.22	2.23	2.06
14	HA	1236	CLA	MG-NA	7.22	2.23	2.06
14	GA	1102	CLA	MG-NA	7.22	2.23	2.06
14	aA	1138	CLA	MG-NA	7.22	2.23	2.06
14	AA	1137	CLA	MG-NA	7.21	2.23	2.06
14	BA	1220	CLA	MG-NA	7.21	2.23	2.06
14	aA	1106	CLA	MG-NA	7.21	2.23	2.06
14	GA	1138	CLA	MG-NA	7.19	2.23	2.06
14	HA	1220	CLA	MG-NA	7.19	2.23	2.06
14	BA	1219	CLA	MG-NA	7.19	2.23	2.06
14	AA	1106	CLA	MG-NA	7.19	2.23	2.06
14	HA	1207	CLA	MG-NA	7.18	2.23	2.06
14	HA	1219	CLA	MG-NA	7.18	2.23	2.06
14	bA	1207	CLA	MG-NA	7.18	2.23	2.06

Continued on next page...

PRELIMINARY VALIDATION REPORT

Continued from previous page...

Mol	Chain	Res	Type	Atoms	Z	Observed(Å)	Ideal(Å)
14	BA	1207	CLA	MG-NA	7.18	2.23	2.06
14	BA	1236	CLA	MG-NA	7.17	2.23	2.06
14	BA	1208	CLA	MG-NA	7.17	2.23	2.06
14	GA	1106	CLA	MG-NA	7.17	2.23	2.06
14	HA	1208	CLA	MG-NA	7.16	2.23	2.06
14	bA	1236	CLA	MG-NA	7.16	2.23	2.06
14	aA	1122	CLA	MG-NA	7.16	2.23	2.06
14	bA	1219	CLA	MG-NA	7.16	2.23	2.06
14	AA	1119	CLA	MG-NA	7.15	2.23	2.06
14	bA	1208	CLA	MG-NA	7.15	2.23	2.06
14	GA	1122	CLA	MG-NA	7.15	2.23	2.06
14	HA	1204	CLA	MG-NA	7.14	2.23	2.06
18	HA	4012	BCR	C10-C9	-7.14	1.26	1.35
14	bA	1204	CLA	MG-NA	7.14	2.23	2.06
14	AA	1122	CLA	MG-NA	7.14	2.23	2.06
14	GA	1131	CLA	MG-NA	7.14	2.23	2.06
14	GA	1119	CLA	MG-NA	7.14	2.23	2.06
18	BA	4012	BCR	C10-C9	-7.13	1.26	1.35
14	bA	1228	CLA	MG-NA	7.13	2.23	2.06
14	BA	1204	CLA	MG-NA	7.13	2.23	2.06
14	HA	1228	CLA	MG-NA	7.12	2.23	2.06
14	GA	1128	CLA	MG-NA	7.12	2.23	2.06
14	AA	1113	CLA	MG-NA	7.12	2.23	2.06
14	aA	1132	CLA	MG-NA	7.12	2.23	2.06
14	AA	1131	CLA	MG-NA	7.12	2.23	2.06
14	aA	1119	CLA	MG-NA	7.11	2.23	2.06
14	aA	1131	CLA	MG-NA	7.11	2.23	2.06
14	aA	1113	CLA	MG-NA	7.11	2.23	2.06
14	GA	1113	CLA	MG-NA	7.11	2.23	2.06
14	AA	1132	CLA	MG-NA	7.10	2.23	2.06
14	aA	1118	CLA	MG-NA	7.10	2.23	2.06
14	bA	1211	CLA	MG-NA	7.10	2.23	2.06
14	aA	1126	CLA	MG-NA	7.10	2.23	2.06
14	AA	1126	CLA	MG-NA	7.10	2.23	2.06
14	aA	1128	CLA	MG-NA	7.10	2.23	2.06
14	bA	1215	CLA	MG-NA	7.09	2.23	2.06
14	HA	1215	CLA	MG-NA	7.09	2.23	2.06
14	BA	1226	CLA	MG-NA	7.09	2.23	2.06
18	bA	4012	BCR	C10-C9	-7.09	1.26	1.35
14	BA	1203	CLA	MG-NA	7.08	2.23	2.06
14	BA	1211	CLA	MG-NA	7.08	2.23	2.06
14	GA	1126	CLA	MG-NA	7.08	2.23	2.06

Continued on next page...

PRELIMINARY VALIDATION REPORT

Continued from previous page...

Mol	Chain	Res	Type	Atoms	Z	Observed(Å)	Ideal(Å)
14	BA	1228	CLA	MG-NA	7.08	2.23	2.06
14	AA	1128	CLA	MG-NA	7.08	2.23	2.06
14	HA	1203	CLA	MG-NA	7.08	2.23	2.06
14	HA	1202	CLA	MG-NA	7.08	2.23	2.06
14	BA	1222	CLA	MG-NA	7.07	2.23	2.06
14	HA	1211	CLA	MG-NA	7.07	2.23	2.06
14	BA	1215	CLA	MG-NA	7.07	2.23	2.06
14	bA	1203	CLA	MG-NA	7.07	2.23	2.06
14	GA	1118	CLA	MG-NA	7.07	2.23	2.06
14	HA	1206	CLA	MG-NA	7.06	2.23	2.06
14	AA	1118	CLA	MG-NA	7.06	2.23	2.06
14	GA	1132	CLA	MG-NA	7.06	2.23	2.06
14	aA	1117	CLA	MG-NA	7.06	2.23	2.06
14	bA	1222	CLA	MG-NA	7.05	2.23	2.06
14	bA	1226	CLA	MG-NA	7.05	2.23	2.06
14	HA	1222	CLA	MG-NA	7.05	2.23	2.06
14	GA	1117	CLA	MG-NA	7.05	2.23	2.06
14	bA	1202	CLA	MG-NA	7.05	2.23	2.06
14	AA	1117	CLA	MG-NA	7.04	2.23	2.06
14	BA	1221	CLA	MG-NA	7.04	2.23	2.06
14	BA	1206	CLA	MG-NA	7.04	2.23	2.06
14	bA	1206	CLA	MG-NA	7.04	2.23	2.06
14	HA	1226	CLA	MG-NA	7.04	2.23	2.06
14	BA	1202	CLA	MG-NA	7.04	2.23	2.06
14	aA	1125	CLA	MG-NA	7.03	2.23	2.06
14	bA	1235	CLA	MG-NA	7.02	2.22	2.06
14	AA	1125	CLA	MG-NA	7.01	2.22	2.06
14	LA	1503	CLA	MG-NA	7.01	2.22	2.06
14	GA	1125	CLA	MG-NA	7.00	2.22	2.06
14	AA	1124	CLA	MG-NA	7.00	2.22	2.06
14	lA	1503	CLA	MG-NA	7.00	2.22	2.06
14	BA	1201	CLA	MG-NA	7.00	2.22	2.06
14	bA	1201	CLA	MG-NA	7.00	2.22	2.06
14	GA	1124	CLA	MG-NA	6.99	2.22	2.06
14	bA	1221	CLA	MG-NA	6.99	2.22	2.06
14	HA	1221	CLA	MG-NA	6.99	2.22	2.06
14	HA	1201	CLA	MG-NA	6.99	2.22	2.06
14	aA	1124	CLA	MG-NA	6.99	2.22	2.06
14	HA	1235	CLA	MG-NA	6.98	2.22	2.06
14	UA	1503	CLA	MG-NA	6.98	2.22	2.06
14	BA	1235	CLA	MG-NA	6.97	2.22	2.06
14	HA	1216	CLA	MG-NA	6.97	2.22	2.06

Continued on next page...

PRELIMINARY VALIDATION REPORT

Continued from previous page...

Mol	Chain	Res	Type	Atoms	Z	Observed(Å)	Ideal(Å)
14	BA	1216	CLA	MG-NA	6.97	2.22	2.06
14	GA	1109	CLA	MG-NA	6.96	2.22	2.06
14	aA	1109	CLA	MG-NA	6.95	2.22	2.06
14	bA	1216	CLA	MG-NA	6.95	2.22	2.06
14	AA	1109	CLA	MG-NA	6.93	2.22	2.06
14	UA	1502	CLA	MG-NA	6.92	2.22	2.06
14	lA	1502	CLA	MG-NA	6.91	2.22	2.06
14	LA	1502	CLA	MG-NA	6.91	2.22	2.06
14	HA	1021	CLA	MG-NA	6.90	2.22	2.06
14	bA	1021	CLA	MG-NA	6.89	2.22	2.06
14	BA	1021	CLA	MG-NA	6.89	2.22	2.06
14	GA	1104	CLA	MG-NA	6.84	2.22	2.06
13	GA	1011	CL0	MG-NA	6.83	2.22	2.06
14	aA	1104	CLA	MG-NA	6.83	2.22	2.06
13	aA	1011	CL0	MG-NA	6.83	2.22	2.06
14	HA	1205	CLA	MG-NA	6.83	2.22	2.06
14	AA	1104	CLA	MG-NA	6.82	2.22	2.06
14	BA	1205	CLA	MG-NA	6.82	2.22	2.06
13	AA	1011	CL0	MG-NA	6.82	2.22	2.06
14	bA	1205	CLA	MG-NA	6.80	2.22	2.06
14	GA	1103	CLA	MG-NA	6.65	2.22	2.06
14	aA	1103	CLA	MG-NA	6.64	2.22	2.06
14	AA	1103	CLA	MG-NA	6.64	2.22	2.06
14	aA	1013	CLA	MG-NA	6.62	2.22	2.06
14	GA	1013	CLA	MG-NA	6.61	2.22	2.06
14	AA	1013	CLA	MG-NA	6.59	2.21	2.06
14	bA	1023	CLA	MG-NA	6.57	2.21	2.06
14	HA	1023	CLA	MG-NA	6.57	2.21	2.06
14	BA	1023	CLA	MG-NA	6.54	2.21	2.06
16	bA	2002	PQN	C3-C2	6.26	1.48	1.35
16	BA	2002	PQN	C3-C2	6.26	1.48	1.35
16	HA	2002	PQN	C3-C2	6.24	1.48	1.35
16	GA	2001	PQN	C3-C2	6.03	1.48	1.35
16	AA	2001	PQN	C3-C2	6.01	1.48	1.35
16	aA	2001	PQN	C3-C2	6.01	1.48	1.35
14	HA	1215	CLA	O2A-C1	5.25	1.61	1.46
14	bA	1215	CLA	O2A-C1	5.24	1.61	1.46
14	BA	1215	CLA	O2A-C1	5.22	1.61	1.46
14	AA	1102	CLA	O2A-C1	5.21	1.61	1.46
14	aA	1102	CLA	O2A-C1	5.21	1.61	1.46
14	GA	1102	CLA	O2A-C1	5.18	1.61	1.46
14	GA	1118	CLA	O2A-C1	5.16	1.61	1.46

Continued on next page...

PRELIMINARY VALIDATION REPORT

Continued from previous page...

Mol	Chain	Res	Type	Atoms	Z	Observed(Å)	Ideal(Å)
14	AA	1116	CLA	O2A-C1	5.16	1.61	1.46
14	bA	1216	CLA	O2A-C1	5.16	1.61	1.46
14	aA	1133	CLA	O2A-C1	5.15	1.61	1.46
14	HA	1216	CLA	O2A-C1	5.15	1.61	1.46
14	BA	1216	CLA	O2A-C1	5.15	1.61	1.46
14	aA	1118	CLA	O2A-C1	5.14	1.61	1.46
14	AA	1118	CLA	O2A-C1	5.14	1.61	1.46
14	AA	1013	CLA	O2A-C1	5.13	1.61	1.46
14	AA	1133	CLA	O2A-C1	5.13	1.61	1.46
14	GA	1133	CLA	O2A-C1	5.13	1.61	1.46
14	aA	1116	CLA	O2A-C1	5.13	1.60	1.46
13	AA	1011	CL0	O2A-C1	5.13	1.60	1.46
14	GA	1116	CLA	O2A-C1	5.12	1.60	1.46
13	GA	1011	CL0	O2A-C1	5.12	1.60	1.46
14	GA	1013	CLA	O2A-C1	5.12	1.60	1.46
13	aA	1011	CL0	O2A-C1	5.11	1.60	1.46
14	aA	1013	CLA	O2A-C1	5.10	1.60	1.46
14	GA	1113	CLA	C3B-C2B	5.10	1.47	1.40
14	AA	1122	CLA	O2D-CGD	5.10	1.45	1.33
14	AA	1111	CLA	O2A-C1	5.09	1.60	1.46
14	GA	1122	CLA	O2D-CGD	5.08	1.45	1.33
14	aA	1113	CLA	C3B-C2B	5.08	1.47	1.40
14	GA	1111	CLA	O2A-C1	5.07	1.60	1.46
14	aA	1111	CLA	O2A-C1	5.07	1.60	1.46
14	aA	1122	CLA	O2D-CGD	5.06	1.45	1.33
14	kA	1401	CLA	O2D-CGD	5.06	1.45	1.33
14	GA	1140	CLA	O2A-C1	5.06	1.60	1.46
14	AA	1113	CLA	C3B-C2B	5.05	1.47	1.40
14	TA	1401	CLA	O2D-CGD	5.05	1.45	1.33
14	AA	1125	CLA	O2A-C1	5.05	1.60	1.46
14	BA	1213	CLA	O2A-C1	5.05	1.60	1.46
14	AA	1140	CLA	O2A-C1	5.05	1.60	1.46
14	BA	1225	CLA	O2A-C1	5.05	1.60	1.46
14	HA	1225	CLA	O2A-C1	5.05	1.60	1.46
14	GA	1136	CLA	O2A-C1	5.05	1.60	1.46
14	bA	1213	CLA	O2A-C1	5.05	1.60	1.46
14	bA	1221	CLA	O2A-C1	5.05	1.60	1.46
14	aA	1125	CLA	O2A-C1	5.05	1.60	1.46
14	BA	1221	CLA	O2A-C1	5.05	1.60	1.46
14	HA	1213	CLA	O2A-C1	5.04	1.60	1.46
14	aA	1130	CLA	O2A-C1	5.04	1.60	1.46
14	BA	1022	CLA	O2A-C1	5.04	1.60	1.46

Continued on next page...

PRELIMINARY VALIDATION REPORT

Continued from previous page...

Mol	Chain	Res	Type	Atoms	Z	Observed(Å)	Ideal(Å)
14	HA	1205	CLA	O2A-C1	5.04	1.60	1.46
14	GA	1125	CLA	O2A-C1	5.04	1.60	1.46
14	aA	1136	CLA	O2A-C1	5.04	1.60	1.46
14	bA	1225	CLA	O2A-C1	5.04	1.60	1.46
14	bA	1022	CLA	O2A-C1	5.04	1.60	1.46
14	HA	1221	CLA	O2A-C1	5.04	1.60	1.46
14	BA	1239	CLA	O2A-C1	5.03	1.60	1.46
14	0	1012	CLA	O2A-C1	5.03	1.60	1.46
14	KA	1401	CLA	O2D-CGD	5.03	1.45	1.33
14	AA	1136	CLA	O2A-C1	5.03	1.60	1.46
14	BA	1220	CLA	O2D-CGD	5.03	1.45	1.33
14	aA	1119	CLA	O2A-C1	5.03	1.60	1.46
14	aA	1140	CLA	O2A-C1	5.03	1.60	1.46
14	GA	1119	CLA	O2A-C1	5.02	1.60	1.46
14	GA	1130	CLA	O2A-C1	5.02	1.60	1.46
14	AA	1130	CLA	O2A-C1	5.02	1.60	1.46
14	BA	1217	CLA	O2A-C1	5.02	1.60	1.46
14	GA	1013	CLA	CHC-C1C	5.01	1.48	1.35
14	bA	1239	CLA	O2A-C1	5.01	1.60	1.46
14	UA	1502	CLA	O2A-C1	5.01	1.60	1.46
14	bA	1205	CLA	O2A-C1	5.01	1.60	1.46
14	HA	1217	CLA	O2A-C1	5.00	1.60	1.46
14	bA	1217	CLA	O2A-C1	5.00	1.60	1.46
14	bA	1220	CLA	O2D-CGD	5.00	1.45	1.33
14	HA	1220	CLA	O2D-CGD	5.00	1.45	1.33
14	AA	1119	CLA	O2A-C1	5.00	1.60	1.46
14	lA	1502	CLA	O2A-C1	5.00	1.60	1.46
14	BA	1227	CLA	O2D-CGD	5.00	1.45	1.33
14	BA	1220	CLA	O2A-C1	5.00	1.60	1.46
14	AA	1013	CLA	CHC-C1C	4.99	1.48	1.35
14	BA	1201	CLA	O2A-C1	4.99	1.60	1.46
14	bA	1238	CLA	O2D-CGD	4.99	1.45	1.33
14	GA	1138	CLA	O2D-CGD	4.99	1.45	1.33
14	aA	1013	CLA	CHC-C1C	4.99	1.48	1.35
14	AA	1138	CLA	O2D-CGD	4.99	1.45	1.33
14	bA	1201	CLA	O2A-C1	4.99	1.60	1.46
14	BA	1205	CLA	O2A-C1	4.99	1.60	1.46
14	BA	1238	CLA	O2D-CGD	4.99	1.45	1.33
14	HA	1239	CLA	O2A-C1	4.99	1.60	1.46
14	aA	1127	CLA	O2A-C1	4.98	1.60	1.46
14	LA	1502	CLA	O2A-C1	4.98	1.60	1.46
14	HA	1220	CLA	O2A-C1	4.98	1.60	1.46

Continued on next page...

PRELIMINARY VALIDATION REPORT

Continued from previous page...

Mol	Chain	Res	Type	Atoms	Z	Observed(Å)	Ideal(Å)
14	AA	1127	CLA	O2A-C1	4.98	1.60	1.46
14	bA	1220	CLA	O2A-C1	4.98	1.60	1.46
14	bA	1227	CLA	O2D-CGD	4.98	1.45	1.33
14	HA	1201	CLA	O2A-C1	4.98	1.60	1.46
14	AA	1114	CLA	O2D-CGD	4.97	1.45	1.33
14	HA	1227	CLA	O2D-CGD	4.97	1.45	1.33
14	HA	1238	CLA	O2D-CGD	4.97	1.45	1.33
14	GA	1127	CLA	O2A-C1	4.96	1.60	1.46
14	AA	1012	CLA	O2A-C1	4.96	1.60	1.46
14	aA	1138	CLA	O2D-CGD	4.96	1.45	1.33
14	aA	1012	CLA	O2A-C1	4.96	1.60	1.46
14	bA	1219	CLA	O2D-CGD	4.96	1.45	1.33
14	bA	1218	CLA	O2D-CGD	4.96	1.45	1.33
14	HA	1231	CLA	C3B-C2B	4.95	1.47	1.40
14	AA	1135	CLA	O2A-C1	4.95	1.60	1.46
14	BA	1231	CLA	C3B-C2B	4.94	1.47	1.40
14	aA	1135	CLA	O2A-C1	4.94	1.60	1.46
14	BA	1219	CLA	O2D-CGD	4.94	1.45	1.33
14	HA	1219	CLA	O2D-CGD	4.94	1.45	1.33
14	GA	1135	CLA	O2A-C1	4.94	1.60	1.46
14	GA	1012	CLA	O2A-C1	4.94	1.60	1.46
14	AA	1128	CLA	O2A-C1	4.94	1.60	1.46
14	bA	1233	CLA	O2D-CGD	4.94	1.45	1.33
14	HA	1208	CLA	O2A-C1	4.94	1.60	1.46
14	AA	1115	CLA	O2A-C1	4.94	1.60	1.46
14	GA	1114	CLA	O2D-CGD	4.94	1.45	1.33
14	BA	1218	CLA	O2D-CGD	4.94	1.45	1.33
14	HA	1218	CLA	O2D-CGD	4.94	1.45	1.33
14	GA	1115	CLA	O2A-C1	4.94	1.60	1.46
14	HA	1233	CLA	O2D-CGD	4.93	1.45	1.33
14	HA	1211	CLA	O2A-C1	4.93	1.60	1.46
14	GA	1105	CLA	O2D-CGD	4.93	1.45	1.33
14	bA	1228	CLA	O2D-CGD	4.93	1.45	1.33
14	aA	1114	CLA	O2D-CGD	4.93	1.45	1.33
14	AA	1117	CLA	O2A-C1	4.93	1.60	1.46
14	GA	1108	CLA	O2D-CGD	4.93	1.45	1.33
14	aA	1108	CLA	O2D-CGD	4.93	1.45	1.33
14	bA	1231	CLA	C3B-C2B	4.92	1.47	1.40
14	aA	1123	CLA	O2A-C1	4.92	1.60	1.46
14	AA	1132	CLA	O2A-C1	4.92	1.60	1.46
14	aA	1128	CLA	O2A-C1	4.92	1.60	1.46
14	bA	1208	CLA	O2A-C1	4.92	1.60	1.46

Continued on next page...

PRELIMINARY VALIDATION REPORT

Continued from previous page...

Mol	Chain	Res	Type	Atoms	Z	Observed(Å)	Ideal(Å)
14	bA	1229	CLA	C3B-C2B	4.92	1.47	1.40
14	BA	1233	CLA	O2D-CGD	4.92	1.45	1.33
14	aA	1132	CLA	O2A-C1	4.91	1.60	1.46
14	GA	1117	CLA	O2A-C1	4.91	1.60	1.46
14	HA	1238	CLA	O2A-C1	4.91	1.60	1.46
14	bA	1216	CLA	O2D-CGD	4.91	1.45	1.33
14	AA	1108	CLA	O2D-CGD	4.91	1.45	1.33
14	HA	1229	CLA	O2A-C1	4.91	1.60	1.46
14	BA	1208	CLA	O2A-C1	4.91	1.60	1.46
14	bA	1229	CLA	O2A-C1	4.91	1.60	1.46
14	HA	1216	CLA	O2D-CGD	4.91	1.45	1.33
14	GA	1119	CLA	O2D-CGD	4.91	1.45	1.33
14	aA	1105	CLA	O2D-CGD	4.91	1.45	1.33
14	GA	1128	CLA	O2A-C1	4.91	1.60	1.46
14	GA	1109	CLA	O2D-CGD	4.90	1.45	1.33
14	aA	1115	CLA	O2A-C1	4.90	1.60	1.46
14	AA	1105	CLA	O2D-CGD	4.90	1.45	1.33
14	aA	1117	CLA	O2A-C1	4.90	1.60	1.46
14	HA	1229	CLA	C3B-C2B	4.90	1.47	1.40
14	bA	1211	CLA	O2A-C1	4.90	1.60	1.46
14	BA	1216	CLA	O2D-CGD	4.90	1.45	1.33
14	bA	1204	CLA	O2A-C1	4.90	1.60	1.46
14	HA	1228	CLA	O2D-CGD	4.89	1.45	1.33
14	BA	1211	CLA	O2A-C1	4.89	1.60	1.46
14	AA	1109	CLA	O2D-CGD	4.89	1.45	1.33
14	GA	1115	CLA	O2D-CGD	4.89	1.45	1.33
14	BA	1229	CLA	O2A-C1	4.89	1.60	1.46
14	BA	1235	CLA	O2A-C1	4.89	1.60	1.46
14	BA	1238	CLA	O2A-C1	4.89	1.60	1.46
14	AA	1123	CLA	O2A-C1	4.88	1.60	1.46
14	HA	1235	CLA	O2A-C1	4.88	1.60	1.46
14	bA	1235	CLA	O2A-C1	4.88	1.60	1.46
14	aA	1109	CLA	O2D-CGD	4.88	1.45	1.33
14	bA	1223	CLA	O2A-C1	4.88	1.60	1.46
14	HA	1204	CLA	O2A-C1	4.88	1.60	1.46
14	aA	1119	CLA	O2D-CGD	4.87	1.45	1.33
14	bA	1238	CLA	O2A-C1	4.87	1.60	1.46
14	GA	1123	CLA	O2A-C1	4.87	1.60	1.46
14	AA	1119	CLA	O2D-CGD	4.87	1.45	1.33
14	BA	1229	CLA	C3B-C2B	4.87	1.47	1.40
14	BA	1223	CLA	O2A-C1	4.87	1.60	1.46
14	BA	1207	CLA	O2D-CGD	4.87	1.45	1.33

Continued on next page...

PRELIMINARY VALIDATION REPORT

Continued from previous page...

Mol	Chain	Res	Type	Atoms	Z	Observed(Å)	Ideal(Å)
14	BA	1228	CLA	O2D-CGD	4.87	1.45	1.33
14	BA	1232	CLA	O2D-CGD	4.87	1.45	1.33
14	BA	1204	CLA	O2A-C1	4.86	1.60	1.46
14	GA	1132	CLA	O2A-C1	4.86	1.60	1.46
14	bA	1204	CLA	O2D-CGD	4.85	1.45	1.33
14	AA	1115	CLA	O2D-CGD	4.85	1.45	1.33
14	bA	1232	CLA	O2D-CGD	4.85	1.45	1.33
14	HA	1223	CLA	O2A-C1	4.85	1.60	1.46
14	HA	1232	CLA	O2D-CGD	4.85	1.45	1.33
14	lA	1503	CLA	O2A-C1	4.85	1.60	1.46
14	HA	1214	CLA	O2D-CGD	4.85	1.45	1.33
14	GA	1138	CLA	O2A-C1	4.85	1.60	1.46
14	HA	1231	CLA	O2A-C1	4.85	1.60	1.46
14	HA	1207	CLA	O2D-CGD	4.85	1.45	1.33
14	GA	1103	CLA	O2A-C1	4.85	1.60	1.46
14	aA	1116	CLA	O2D-CGD	4.84	1.45	1.33
14	aA	1115	CLA	O2D-CGD	4.84	1.45	1.33
14	bA	1207	CLA	O2D-CGD	4.84	1.45	1.33
14	bA	1202	CLA	O2A-C1	4.84	1.60	1.46
14	LA	1503	CLA	O2A-C1	4.84	1.60	1.46
14	bA	1231	CLA	O2A-C1	4.84	1.60	1.46
14	aA	1138	CLA	O2A-C1	4.84	1.60	1.46
14	UA	1503	CLA	O2A-C1	4.84	1.60	1.46
14	HA	1204	CLA	O2D-CGD	4.84	1.45	1.33
14	AA	1104	CLA	O2D-CGD	4.83	1.45	1.33
14	AA	1103	CLA	O2A-C1	4.83	1.60	1.46
14	bA	1239	CLA	O2D-CGD	4.83	1.45	1.33
14	bA	1214	CLA	O2A-C1	4.83	1.60	1.46
14	BA	1202	CLA	O2A-C1	4.83	1.60	1.46
14	BA	1213	CLA	O2D-CGD	4.83	1.45	1.33
14	BA	1204	CLA	O2D-CGD	4.83	1.45	1.33
14	AA	1138	CLA	O2A-C1	4.83	1.60	1.46
14	BA	1214	CLA	O2A-C1	4.82	1.60	1.46
14	bA	1213	CLA	O2D-CGD	4.82	1.45	1.33
14	HA	1213	CLA	O2D-CGD	4.82	1.45	1.33
14	HA	1202	CLA	O2A-C1	4.82	1.60	1.46
14	BA	1239	CLA	O2D-CGD	4.82	1.45	1.33
14	BA	1231	CLA	O2A-C1	4.81	1.60	1.46
14	GA	1104	CLA	O2D-CGD	4.81	1.45	1.33
14	aA	1104	CLA	O2D-CGD	4.81	1.45	1.33
14	aA	1103	CLA	O2A-C1	4.81	1.60	1.46
14	HA	1214	CLA	O2A-C1	4.81	1.60	1.46

Continued on next page...

PRELIMINARY VALIDATION REPORT

Continued from previous page...

Mol	Chain	Res	Type	Atoms	Z	Observed(Å)	Ideal(Å)
14	HA	1239	CLA	O2D-CGD	4.81	1.45	1.33
14	GA	1104	CLA	O2A-C1	4.81	1.60	1.46
14	BA	1214	CLA	O2D-CGD	4.81	1.45	1.33
14	HA	1222	CLA	C3B-C2B	4.81	1.47	1.40
14	AA	1124	CLA	O2A-C1	4.80	1.60	1.46
14	HA	1203	CLA	O2A-C1	4.80	1.60	1.46
14	aA	1124	CLA	O2A-C1	4.80	1.60	1.46
14	bA	1214	CLA	O2D-CGD	4.80	1.45	1.33
14	LA	1501	CLA	O2A-C1	4.80	1.60	1.46
14	aA	1104	CLA	O2A-C1	4.80	1.60	1.46
14	GA	1124	CLA	O2A-C1	4.80	1.60	1.46
14	BA	1203	CLA	O2A-C1	4.80	1.60	1.46
14	AA	1122	CLA	O2A-C1	4.79	1.60	1.46
14	UA	1501	CLA	O2A-C1	4.79	1.60	1.46
14	AA	1126	CLA	C3C-C2C	4.79	1.46	1.36
14	bA	1206	CLA	O2D-CGD	4.79	1.45	1.33
14	HA	1210	CLA	O2A-C1	4.79	1.60	1.46
14	HA	1226	CLA	O2A-C1	4.79	1.60	1.46
14	AA	1104	CLA	O2A-C1	4.79	1.60	1.46
14	bA	1239	CLA	C3C-C2C	4.79	1.46	1.36
14	AA	1113	CLA	O2D-CGD	4.79	1.45	1.33
14	lA	1501	CLA	O2A-C1	4.79	1.60	1.46
14	bA	1226	CLA	O2A-C1	4.79	1.60	1.46
14	BA	1235	CLA	O2D-CGD	4.78	1.45	1.33
14	bA	1235	CLA	O2D-CGD	4.78	1.45	1.33
14	aA	1126	CLA	C3C-C2C	4.78	1.46	1.36
14	bA	1210	CLA	O2A-C1	4.78	1.60	1.46
14	AA	1116	CLA	O2D-CGD	4.78	1.45	1.33
14	BA	1209	CLA	O2D-CGD	4.78	1.45	1.33
14	GA	1135	CLA	C3B-C2B	4.78	1.47	1.40
14	bA	1209	CLA	O2D-CGD	4.78	1.45	1.33
14	BA	1210	CLA	O2A-C1	4.77	1.59	1.46
14	HA	1208	CLA	O2D-CGD	4.77	1.45	1.33
14	aA	1101	CLA	O2D-CGD	4.77	1.45	1.33
14	GA	1101	CLA	O2D-CGD	4.77	1.45	1.33
14	GA	1126	CLA	C3C-C2C	4.77	1.46	1.36
14	HA	1235	CLA	O2D-CGD	4.77	1.45	1.33
14	bA	1222	CLA	C3B-C2B	4.77	1.47	1.40
14	GA	1113	CLA	O2D-CGD	4.77	1.45	1.33
14	aA	1130	CLA	O2D-CGD	4.77	1.45	1.33
14	HA	1206	CLA	O2D-CGD	4.77	1.45	1.33
14	HA	1209	CLA	O2D-CGD	4.77	1.45	1.33

Continued on next page...

PRELIMINARY VALIDATION REPORT

Continued from previous page...

Mol	Chain	Res	Type	Atoms	Z	Observed(Å)	Ideal(Å)
14	GA	1139	CLA	O2D-CGD	4.77	1.45	1.33
14	aA	1113	CLA	O2D-CGD	4.77	1.45	1.33
14	BA	1226	CLA	O2A-C1	4.77	1.59	1.46
14	bA	1021	CLA	C3B-C2B	4.76	1.47	1.40
14	BA	1021	CLA	O2A-C1	4.76	1.59	1.46
14	bA	1208	CLA	O2D-CGD	4.76	1.45	1.33
14	BA	1222	CLA	C3B-C2B	4.76	1.47	1.40
14	GA	1122	CLA	O2A-C1	4.76	1.59	1.46
14	AA	1135	CLA	C3B-C2B	4.76	1.47	1.40
14	GA	1116	CLA	O2D-CGD	4.76	1.45	1.33
14	BA	1231	CLA	CHC-C1C	4.76	1.47	1.35
14	BA	1208	CLA	O2D-CGD	4.76	1.45	1.33
14	HA	1215	CLA	O2D-CGD	4.76	1.45	1.33
14	aA	1122	CLA	O2A-C1	4.76	1.59	1.46
14	bA	1231	CLA	CHC-C1C	4.76	1.47	1.35
14	bA	1238	CLA	CHC-C1C	4.76	1.47	1.35
14	bA	1211	CLA	O2D-CGD	4.76	1.45	1.33
14	HA	1225	CLA	O2D-CGD	4.75	1.45	1.33
14	TA	1401	CLA	C3C-C2C	4.75	1.46	1.36
14	HA	1214	CLA	C3C-C2C	4.75	1.46	1.36
14	BA	1215	CLA	O2D-CGD	4.75	1.44	1.33
14	aA	1118	CLA	O2D-CGD	4.75	1.44	1.33
14	AA	1126	CLA	O2D-CGD	4.75	1.44	1.33
14	bA	1224	CLA	O2A-C1	4.75	1.59	1.46
14	BA	1235	CLA	C3B-C2B	4.75	1.47	1.40
14	HA	1231	CLA	CHC-C1C	4.75	1.47	1.35
14	HA	1239	CLA	C3C-C2C	4.75	1.46	1.36
14	xA	1701	CLA	O2D-CGD	4.75	1.44	1.33
14	BA	1224	CLA	O2A-C1	4.75	1.59	1.46
14	HA	1224	CLA	O2A-C1	4.75	1.59	1.46
14	bA	1203	CLA	O2A-C1	4.75	1.59	1.46
14	bA	1214	CLA	C3C-C2C	4.74	1.46	1.36
14	KA	1401	CLA	C3C-C2C	4.74	1.46	1.36
14	GA	1130	CLA	O2D-CGD	4.74	1.44	1.33
14	bA	1215	CLA	O2D-CGD	4.74	1.44	1.33
14	bA	1211	CLA	C3B-C2B	4.74	1.47	1.40
14	HA	1211	CLA	O2D-CGD	4.74	1.44	1.33
14	AA	1101	CLA	O2D-CGD	4.74	1.44	1.33
14	HA	1235	CLA	C3B-C2B	4.74	1.47	1.40
14	GA	1127	CLA	O2D-CGD	4.74	1.44	1.33
14	HA	1021	CLA	O2A-C1	4.74	1.59	1.46
14	BA	1211	CLA	O2D-CGD	4.74	1.44	1.33

Continued on next page...

PRELIMINARY VALIDATION REPORT

Continued from previous page...

Mol	Chain	Res	Type	Atoms	Z	Observed(Å)	Ideal(Å)
14	WA	1701	CLA	O2D-CGD	4.74	1.44	1.33
14	BA	1206	CLA	O2D-CGD	4.74	1.44	1.33
14	BA	1211	CLA	C3B-C2B	4.74	1.47	1.40
14	bA	1225	CLA	O2D-CGD	4.74	1.44	1.33
14	BA	1239	CLA	C3C-C2C	4.74	1.46	1.36
14	AA	1125	CLA	O2D-CGD	4.73	1.44	1.33
14	AA	1118	CLA	O2D-CGD	4.73	1.44	1.33
14	HA	1021	CLA	C3B-C2B	4.73	1.46	1.40
14	BA	1214	CLA	C3C-C2C	4.73	1.46	1.36
14	GA	1125	CLA	O2D-CGD	4.73	1.44	1.33
14	bA	1203	CLA	O2D-CGD	4.73	1.44	1.33
14	bA	1226	CLA	O2D-CGD	4.73	1.44	1.33
14	aA	1123	CLA	O2D-CGD	4.73	1.44	1.33
14	AA	1130	CLA	O2D-CGD	4.73	1.44	1.33
14	bA	1021	CLA	O2A-C1	4.73	1.59	1.46
14	aA	1131	CLA	O2A-C1	4.73	1.59	1.46
14	XA	1701	CLA	O2D-CGD	4.73	1.44	1.33
14	GA	1126	CLA	O2D-CGD	4.73	1.44	1.33
14	AA	1131	CLA	O2A-C1	4.73	1.59	1.46
14	kA	1401	CLA	C3C-C2C	4.72	1.46	1.36
14	aA	1139	CLA	O2D-CGD	4.72	1.44	1.33
14	HA	1215	CLA	C3B-C2B	4.72	1.46	1.40
14	GA	1131	CLA	O2A-C1	4.72	1.59	1.46
14	AA	1127	CLA	O2D-CGD	4.72	1.44	1.33
14	bA	1235	CLA	C3B-C2B	4.72	1.46	1.40
13	aA	1011	CL0	O2D-CGD	4.72	1.44	1.33
14	HA	1203	CLA	O2D-CGD	4.72	1.44	1.33
14	aA	1126	CLA	O2D-CGD	4.72	1.44	1.33
14	GA	1118	CLA	O2D-CGD	4.72	1.44	1.33
14	HA	1226	CLA	O2D-CGD	4.72	1.44	1.33
14	HA	1238	CLA	CHC-C1C	4.72	1.47	1.35
14	AA	1139	CLA	O2D-CGD	4.72	1.44	1.33
14	BA	1215	CLA	C3B-C2B	4.72	1.46	1.40
14	BA	1203	CLA	O2D-CGD	4.71	1.44	1.33
14	GA	1106	CLA	O2D-CGD	4.71	1.44	1.33
14	AA	1126	CLA	O2A-C1	4.71	1.59	1.46
14	BA	1021	CLA	C3B-C2B	4.71	1.46	1.40
14	aA	1127	CLA	O2D-CGD	4.71	1.44	1.33
14	bA	1234	CLA	O2A-C1	4.71	1.59	1.46
14	BA	1226	CLA	O2D-CGD	4.71	1.44	1.33
14	aA	1135	CLA	C3B-C2B	4.71	1.46	1.40
14	HA	1234	CLA	O2A-C1	4.70	1.59	1.46

Continued on next page...

PRELIMINARY VALIDATION REPORT

Continued from previous page...

Mol	Chain	Res	Type	Atoms	Z	Observed(Å)	Ideal(Å)
14	aA	1123	CLA	C3B-C2B	4.70	1.46	1.40
14	BA	1238	CLA	CHC-C1C	4.70	1.47	1.35
14	HA	1023	CLA	O2D-CGD	4.70	1.44	1.33
14	AA	1106	CLA	O2D-CGD	4.70	1.44	1.33
14	AA	1133	CLA	O2D-CGD	4.70	1.44	1.33
13	GA	1011	CL0	O2D-CGD	4.70	1.44	1.33
14	bA	1215	CLA	C3B-C2B	4.69	1.46	1.40
14	BA	1225	CLA	O2D-CGD	4.69	1.44	1.33
14	AA	1101	CLA	CHC-C1C	4.69	1.47	1.35
14	BA	1023	CLA	O2A-C1	4.69	1.59	1.46
14	aA	1126	CLA	O2A-C1	4.69	1.59	1.46
14	GA	1126	CLA	O2A-C1	4.69	1.59	1.46
14	BA	1234	CLA	O2A-C1	4.69	1.59	1.46
14	AA	1123	CLA	O2D-CGD	4.69	1.44	1.33
14	GA	1123	CLA	O2D-CGD	4.69	1.44	1.33
14	HA	1023	CLA	O2A-C1	4.69	1.59	1.46
14	aA	1125	CLA	C3B-C2B	4.69	1.46	1.40
14	GA	1133	CLA	O2D-CGD	4.69	1.44	1.33
14	bA	1023	CLA	O2D-CGD	4.69	1.44	1.33
14	aA	1125	CLA	O2D-CGD	4.69	1.44	1.33
14	BA	1218	CLA	C3B-C2B	4.68	1.46	1.40
14	HA	1217	CLA	O2D-CGD	4.68	1.44	1.33
14	aA	1106	CLA	O2D-CGD	4.68	1.44	1.33
14	AA	1112	CLA	O2D-CGD	4.68	1.44	1.33
14	GA	1101	CLA	CHC-C1C	4.68	1.47	1.35
14	bA	1217	CLA	O2D-CGD	4.68	1.44	1.33
14	0	1012	CLA	CHC-C1C	4.68	1.47	1.35
14	HA	1222	CLA	CHC-C1C	4.68	1.47	1.35
14	aA	1105	CLA	CHC-C1C	4.68	1.47	1.35
14	HA	1211	CLA	C3B-C2B	4.68	1.46	1.40
14	aA	1140	CLA	O2D-CGD	4.67	1.44	1.33
14	bA	1023	CLA	O2A-C1	4.67	1.59	1.46
14	AA	1123	CLA	C3B-C2B	4.67	1.46	1.40
13	AA	1011	CL0	O2D-CGD	4.67	1.44	1.33
14	GA	1140	CLA	O2D-CGD	4.67	1.44	1.33
14	aA	1133	CLA	O2D-CGD	4.67	1.44	1.33
14	GA	1123	CLA	C3B-C2B	4.67	1.46	1.40
14	AA	1125	CLA	C3B-C2B	4.67	1.46	1.40
14	aA	1102	CLA	O2D-CGD	4.67	1.44	1.33
14	GA	1112	CLA	O2D-CGD	4.67	1.44	1.33
14	HA	1218	CLA	C3B-C2B	4.67	1.46	1.40
14	AA	1105	CLA	CHC-C1C	4.67	1.47	1.35

Continued on next page...

PRELIMINARY VALIDATION REPORT

Continued from previous page...

Mol	Chain	Res	Type	Atoms	Z	Observed(Å)	Ideal(Å)
14	GA	1131	CLA	O2D-CGD	4.67	1.44	1.33
14	BA	1023	CLA	O2D-CGD	4.66	1.44	1.33
14	bA	1219	CLA	CHC-C1C	4.66	1.47	1.35
14	bA	1218	CLA	C3B-C2B	4.66	1.46	1.40
14	bA	1222	CLA	CHC-C1C	4.66	1.47	1.35
14	GA	1108	CLA	CHC-C1C	4.66	1.47	1.35
14	bA	1224	CLA	O2D-CGD	4.66	1.44	1.33
14	aA	1112	CLA	O2D-CGD	4.66	1.44	1.33
14	BA	1022	CLA	CHC-C1C	4.66	1.47	1.35
14	AA	1108	CLA	CHC-C1C	4.66	1.47	1.35
14	BA	1222	CLA	CHC-C1C	4.65	1.47	1.35
14	aA	1105	CLA	C3B-C2B	4.65	1.46	1.40
14	AA	1117	CLA	O2D-CGD	4.65	1.44	1.33
14	GA	1105	CLA	CHC-C1C	4.65	1.47	1.35
14	BA	1217	CLA	O2D-CGD	4.65	1.44	1.33
14	AA	1140	CLA	O2D-CGD	4.65	1.44	1.33
14	BA	1223	CLA	O2D-CGD	4.65	1.44	1.33
14	bA	1234	CLA	CHC-C1C	4.65	1.47	1.35
14	HA	1224	CLA	O2D-CGD	4.65	1.44	1.33
14	BA	1234	CLA	CHC-C1C	4.65	1.47	1.35
14	aA	1101	CLA	CHC-C1C	4.65	1.47	1.35
14	GA	1102	CLA	O2D-CGD	4.65	1.44	1.33
14	aA	1108	CLA	CHC-C1C	4.65	1.47	1.35
14	aA	1110	CLA	CHC-C1C	4.65	1.47	1.35
14	HA	1234	CLA	CHC-C1C	4.65	1.47	1.35
14	BA	1224	CLA	O2D-CGD	4.65	1.44	1.33
14	GA	1117	CLA	O2D-CGD	4.65	1.44	1.33
14	xA	1701	CLA	CHC-C1C	4.65	1.47	1.35
14	HA	1212	CLA	O2D-CGD	4.65	1.44	1.33
14	bA	1022	CLA	CHC-C1C	4.65	1.47	1.35
14	aA	1115	CLA	C3B-C2B	4.64	1.46	1.40
14	XA	1701	CLA	CHC-C1C	4.64	1.47	1.35
14	bA	1212	CLA	CHC-C1C	4.64	1.47	1.35
14	aA	1117	CLA	O2D-CGD	4.64	1.44	1.33
14	aA	1131	CLA	O2D-CGD	4.64	1.44	1.33
14	GA	1105	CLA	C3B-C2B	4.64	1.46	1.40
14	HA	1219	CLA	CHC-C1C	4.64	1.47	1.35
14	AA	1131	CLA	O2D-CGD	4.64	1.44	1.33
14	BA	1212	CLA	CHC-C1C	4.64	1.47	1.35
14	AA	1110	CLA	CHC-C1C	4.64	1.47	1.35
14	HA	1212	CLA	CHC-C1C	4.64	1.47	1.35
14	HA	1211	CLA	CHC-C1C	4.63	1.47	1.35

Continued on next page...

PRELIMINARY VALIDATION REPORT

Continued from previous page...

Mol	Chain	Res	Type	Atoms	Z	Observed(Å)	Ideal(Å)
14	BA	1205	CLA	O2D-CGD	4.63	1.44	1.33
14	AA	1115	CLA	C3B-C2B	4.63	1.46	1.40
14	aA	1130	CLA	CHC-C1C	4.63	1.47	1.35
14	AA	1102	CLA	O2D-CGD	4.63	1.44	1.33
14	HA	1223	CLA	O2D-CGD	4.63	1.44	1.33
14	BA	1211	CLA	CHC-C1C	4.63	1.47	1.35
14	bA	1223	CLA	O2D-CGD	4.62	1.44	1.33
14	GA	1013	CLA	O2D-CGD	4.62	1.44	1.33
14	HA	1222	CLA	O2A-C1	4.62	1.59	1.46
14	bA	1211	CLA	CHC-C1C	4.62	1.47	1.35
14	bA	1205	CLA	O2D-CGD	4.62	1.44	1.33
14	BA	1219	CLA	CHC-C1C	4.62	1.47	1.35
14	GA	1119	CLA	CHC-C1C	4.62	1.47	1.35
14	GA	1115	CLA	C3B-C2B	4.62	1.46	1.40
14	BA	1212	CLA	O2D-CGD	4.61	1.44	1.33
14	bA	1212	CLA	O2D-CGD	4.61	1.44	1.33
14	AA	1137	CLA	O2D-CGD	4.61	1.44	1.33
14	bA	1222	CLA	O2A-C1	4.61	1.59	1.46
14	aA	1124	CLA	CHC-C1C	4.61	1.47	1.35
14	GA	1125	CLA	C3B-C2B	4.61	1.46	1.40
14	HA	1223	CLA	C3B-C2B	4.61	1.46	1.40
14	AA	1124	CLA	CHC-C1C	4.61	1.47	1.35
14	AA	1127	CLA	C3B-C2B	4.61	1.46	1.40
14	HA	1205	CLA	O2D-CGD	4.61	1.44	1.33
14	BA	1218	CLA	CHC-C1C	4.61	1.47	1.35
14	HA	1201	CLA	O2D-CGD	4.61	1.44	1.33
14	aA	1013	CLA	O2D-CGD	4.61	1.44	1.33
14	aA	1119	CLA	CHC-C1C	4.61	1.47	1.35
13	AA	1011	CL0	C3B-C2B	4.61	1.46	1.40
14	BA	1217	CLA	CHC-C1C	4.60	1.47	1.35
14	aA	1127	CLA	C3B-C2B	4.60	1.46	1.40
14	GA	1124	CLA	CHC-C1C	4.60	1.47	1.35
14	WA	1701	CLA	CHC-C1C	4.60	1.47	1.35
14	bA	1223	CLA	C3B-C2B	4.60	1.46	1.40
14	AA	1102	CLA	CHC-C1C	4.60	1.47	1.35
14	BA	1222	CLA	O2A-C1	4.60	1.59	1.46
14	GA	1130	CLA	CHC-C1C	4.60	1.47	1.35
14	HA	1221	CLA	CHC-C1C	4.60	1.47	1.35
14	GA	1110	CLA	CHC-C1C	4.60	1.47	1.35
14	aA	1102	CLA	CHC-C1C	4.60	1.47	1.35
14	HA	1217	CLA	CHC-C1C	4.60	1.47	1.35
14	bA	1201	CLA	O2D-CGD	4.60	1.44	1.33

Continued on next page...

PRELIMINARY VALIDATION REPORT

Continued from previous page...

Mol	Chain	Res	Type	Atoms	Z	Observed(Å)	Ideal(Å)
14	GA	1102	CLA	CHC-C1C	4.59	1.47	1.35
14	bA	1220	CLA	C3B-C2B	4.59	1.46	1.40
14	bA	1221	CLA	O2D-CGD	4.59	1.44	1.33
14	BA	1213	CLA	CHC-C1C	4.59	1.47	1.35
14	HA	1218	CLA	CHC-C1C	4.59	1.47	1.35
14	aA	1102	CLA	C3B-C2B	4.59	1.46	1.40
14	bA	1221	CLA	CHC-C1C	4.59	1.47	1.35
14	AA	1114	CLA	OBD-CAD	4.59	1.28	1.22
14	BA	1221	CLA	CHC-C1C	4.59	1.47	1.35
13	GA	1011	CL0	C3B-C2B	4.59	1.46	1.40
14	BA	1201	CLA	O2D-CGD	4.59	1.44	1.33
14	GA	1127	CLA	C3B-C2B	4.59	1.46	1.40
14	GA	1137	CLA	O2D-CGD	4.59	1.44	1.33
14	BA	1235	CLA	CHC-C1C	4.59	1.47	1.35
14	AA	1013	CLA	O2D-CGD	4.58	1.44	1.33
14	bA	1213	CLA	CHC-C1C	4.58	1.47	1.35
14	AA	1130	CLA	CHC-C1C	4.58	1.47	1.35
14	lA	1503	CLA	O2D-CGD	4.58	1.44	1.33
14	aA	1114	CLA	C3C-C2C	4.58	1.46	1.36
14	HA	1222	CLA	O2D-CGD	4.58	1.44	1.33
14	HA	1221	CLA	O2D-CGD	4.58	1.44	1.33
14	AA	1119	CLA	CHC-C1C	4.58	1.47	1.35
14	BA	1224	CLA	CHC-C1C	4.58	1.47	1.35
14	bA	1233	CLA	CHC-C1C	4.58	1.47	1.35
14	kA	1401	CLA	OBD-CAD	4.58	1.28	1.22
14	HA	1220	CLA	C3B-C2B	4.58	1.46	1.40
14	BA	1205	CLA	CHC-C1C	4.58	1.47	1.35
14	GA	1114	CLA	C3C-C2C	4.58	1.46	1.36
14	bA	1229	CLA	CHC-C1C	4.58	1.47	1.35
14	UA	1501	CLA	O2D-CGD	4.58	1.44	1.33
14	TA	1401	CLA	OBD-CAD	4.58	1.28	1.22
14	BA	1236	CLA	O2D-CGD	4.57	1.44	1.33
14	HA	1236	CLA	O2D-CGD	4.57	1.44	1.33
14	AA	1112	CLA	CHC-C1C	4.57	1.47	1.35
14	GA	1129	CLA	CHC-C1C	4.57	1.47	1.35
14	HA	1229	CLA	CHC-C1C	4.57	1.47	1.35
14	HA	1235	CLA	CHC-C1C	4.57	1.47	1.35
14	HA	1229	CLA	O2D-CGD	4.57	1.44	1.33
14	aA	1129	CLA	CHC-C1C	4.57	1.47	1.35
14	lA	1501	CLA	O2D-CGD	4.57	1.44	1.33
14	BA	1222	CLA	O2D-CGD	4.57	1.44	1.33
14	BA	1229	CLA	CHC-C1C	4.57	1.47	1.35

Continued on next page...

PRELIMINARY VALIDATION REPORT

Continued from previous page...

Mol	Chain	Res	Type	Atoms	Z	Observed(Å)	Ideal(Å)
14	AA	1129	CLA	CHC-C1C	4.57	1.47	1.35
14	bA	1218	CLA	CHC-C1C	4.57	1.47	1.35
14	bA	1229	CLA	O2D-CGD	4.57	1.44	1.33
14	bA	1224	CLA	CHC-C1C	4.57	1.47	1.35
14	HA	1205	CLA	CHC-C1C	4.57	1.47	1.35
14	TA	1401	CLA	CHC-C1C	4.57	1.47	1.35
14	BA	1213	CLA	OBD-CAD	4.56	1.28	1.22
14	aA	1112	CLA	CHC-C1C	4.56	1.47	1.35
14	LA	1501	CLA	C3B-C2B	4.56	1.46	1.40
14	BA	1233	CLA	OBD-CAD	4.56	1.28	1.22
14	BA	1233	CLA	CHC-C1C	4.56	1.47	1.35
14	AA	1102	CLA	C3B-C2B	4.56	1.46	1.40
14	LA	1501	CLA	O2D-CGD	4.56	1.44	1.33
14	HA	1213	CLA	CHC-C1C	4.56	1.47	1.35
14	AA	1114	CLA	C3C-C2C	4.56	1.46	1.36
14	aA	1107	CLA	O2D-CGD	4.56	1.44	1.33
14	bA	1236	CLA	O2D-CGD	4.56	1.44	1.33
14	BA	1221	CLA	O2D-CGD	4.56	1.44	1.33
14	bA	1222	CLA	O2D-CGD	4.56	1.44	1.33
14	GA	1112	CLA	CHC-C1C	4.56	1.47	1.35
14	BA	1223	CLA	C3B-C2B	4.56	1.46	1.40
14	aA	1137	CLA	O2D-CGD	4.56	1.44	1.33
14	BA	1220	CLA	C3B-C2B	4.55	1.46	1.40
14	aA	1110	CLA	O2D-CGD	4.55	1.44	1.33
14	BA	1222	CLA	C3C-C2C	4.55	1.46	1.36
14	GA	1106	CLA	CHC-C1C	4.55	1.47	1.35
14	bA	1217	CLA	CHC-C1C	4.55	1.47	1.35
14	aA	1129	CLA	O2D-CGD	4.55	1.44	1.33
14	UA	1503	CLA	O2D-CGD	4.55	1.44	1.33
14	AA	1105	CLA	C3B-C2B	4.55	1.46	1.40
14	GA	1124	CLA	C3C-C2C	4.54	1.46	1.36
14	HA	1224	CLA	CHC-C1C	4.54	1.47	1.35
14	bA	1235	CLA	CHC-C1C	4.54	1.47	1.35
14	LA	1503	CLA	O2D-CGD	4.54	1.44	1.33
14	bA	1205	CLA	CHC-C1C	4.54	1.47	1.35
14	AA	1129	CLA	O2D-CGD	4.54	1.44	1.33
14	GA	1110	CLA	O2D-CGD	4.54	1.44	1.33
14	KA	1401	CLA	CHC-C1C	4.54	1.47	1.35
14	GA	1129	CLA	O2D-CGD	4.54	1.44	1.33
14	HA	1233	CLA	OBD-CAD	4.54	1.28	1.22
14	AA	1110	CLA	O2D-CGD	4.54	1.44	1.33
14	AA	1106	CLA	CHC-C1C	4.54	1.47	1.35

Continued on next page...

PRELIMINARY VALIDATION REPORT

Continued from previous page...

Mol	Chain	Res	Type	Atoms	Z	Observed(Å)	Ideal(Å)
14	bA	1209	CLA	CHC-C1C	4.54	1.47	1.35
14	AA	1107	CLA	O2D-CGD	4.54	1.44	1.33
14	HA	1233	CLA	CHC-C1C	4.54	1.47	1.35
14	bA	1222	CLA	C3C-C2C	4.54	1.46	1.36
14	BA	1229	CLA	O2D-CGD	4.54	1.44	1.33
14	GA	1107	CLA	O2D-CGD	4.54	1.44	1.33
14	bA	1233	CLA	OBD-CAD	4.54	1.28	1.22
14	kA	1401	CLA	CHC-C1C	4.53	1.47	1.35
14	aA	1124	CLA	C3C-C2C	4.53	1.46	1.36
14	GA	1114	CLA	OBD-CAD	4.53	1.28	1.22
14	KA	1401	CLA	OBD-CAD	4.53	1.28	1.22
14	AA	1124	CLA	C3C-C2C	4.53	1.46	1.36
14	AA	1128	CLA	O2D-CGD	4.53	1.44	1.33
14	xA	1701	CLA	C3B-C2B	4.53	1.46	1.40
14	aA	1106	CLA	CHC-C1C	4.53	1.47	1.35
14	aA	1128	CLA	O2D-CGD	4.53	1.44	1.33
14	HA	1209	CLA	CHC-C1C	4.53	1.47	1.35
14	GA	1102	CLA	C3B-C2B	4.53	1.46	1.40
14	bA	1203	CLA	CHC-C1C	4.53	1.47	1.35
14	HA	1222	CLA	C3C-C2C	4.53	1.46	1.36
13	aA	1011	CL0	C3B-C2B	4.52	1.46	1.40
14	HA	1229	CLA	C3C-C2C	4.52	1.46	1.36
14	aA	1123	CLA	CHC-C1C	4.52	1.47	1.35
14	aA	1139	CLA	CHC-C1C	4.52	1.47	1.35
14	BA	1227	CLA	CHC-C1C	4.52	1.47	1.35
14	aA	1114	CLA	OBD-CAD	4.51	1.28	1.22
14	lA	1501	CLA	C3B-C2B	4.51	1.46	1.40
14	GA	1123	CLA	CHC-C1C	4.51	1.47	1.35
14	UA	1501	CLA	CHC-C1C	4.51	1.47	1.35
14	lA	1501	CLA	CHC-C1C	4.51	1.47	1.35
14	bA	1227	CLA	CHC-C1C	4.51	1.47	1.35
14	AA	1123	CLA	CHC-C1C	4.51	1.47	1.35
14	UA	1501	CLA	C3B-C2B	4.50	1.46	1.40
14	AA	1112	CLA	OBD-CAD	4.50	1.28	1.22
14	HA	1213	CLA	OBD-CAD	4.50	1.28	1.22
14	GA	1115	CLA	CHC-C1C	4.50	1.47	1.35
14	GA	1139	CLA	CHC-C1C	4.50	1.47	1.35
14	BA	1203	CLA	CHC-C1C	4.50	1.47	1.35
14	aA	1138	CLA	CHC-C1C	4.50	1.47	1.35
14	HA	1227	CLA	CHC-C1C	4.50	1.47	1.35
14	AA	1139	CLA	CHC-C1C	4.50	1.47	1.35
14	HA	1203	CLA	CHC-C1C	4.50	1.47	1.35

Continued on next page...

PRELIMINARY VALIDATION REPORT

Continued from previous page...

Mol	Chain	Res	Type	Atoms	Z	Observed(Å)	Ideal(Å)
14	GA	1112	CLA	OBD-CAD	4.50	1.28	1.22
14	GA	1126	CLA	CHC-C1C	4.50	1.47	1.35
14	AA	1126	CLA	CHC-C1C	4.50	1.47	1.35
14	BA	1229	CLA	C3C-C2C	4.49	1.46	1.36
14	GA	1108	CLA	C3C-C2C	4.49	1.46	1.36
14	LA	1501	CLA	CHC-C1C	4.49	1.47	1.35
14	BA	1218	CLA	OBD-CAD	4.49	1.28	1.22
14	aA	1108	CLA	C3C-C2C	4.49	1.46	1.36
14	GA	1128	CLA	O2D-CGD	4.49	1.44	1.33
14	BA	1231	CLA	O2D-CGD	4.48	1.44	1.33
14	GA	1103	CLA	O2D-CGD	4.48	1.44	1.33
14	aA	1112	CLA	OBD-CAD	4.48	1.28	1.22
14	AA	1132	CLA	CHC-C1C	4.48	1.47	1.35
14	bA	1213	CLA	OBD-CAD	4.48	1.28	1.22
14	aA	1135	CLA	O2D-CGD	4.48	1.44	1.33
14	bA	1229	CLA	C3C-C2C	4.48	1.46	1.36
14	bA	1231	CLA	O2D-CGD	4.48	1.44	1.33
14	AA	1115	CLA	CHC-C1C	4.48	1.47	1.35
14	aA	1115	CLA	CHC-C1C	4.48	1.47	1.35
14	BA	1209	CLA	CHC-C1C	4.48	1.47	1.35
14	HA	1210	CLA	O2D-CGD	4.48	1.44	1.33
14	AA	1138	CLA	CHC-C1C	4.47	1.47	1.35
14	GA	1132	CLA	C3C-C2C	4.47	1.46	1.36
14	aA	1114	CLA	C3B-C2B	4.47	1.46	1.40
14	HA	1228	CLA	C3C-C2C	4.47	1.46	1.36
14	HA	1201	CLA	CHC-C1C	4.47	1.47	1.35
14	aA	1132	CLA	CHC-C1C	4.47	1.47	1.35
14	GA	1135	CLA	O2D-CGD	4.47	1.44	1.33
14	GA	1138	CLA	CHC-C1C	4.47	1.47	1.35
14	bA	1210	CLA	O2D-CGD	4.47	1.44	1.33
14	bA	1231	CLA	OBD-CAD	4.47	1.28	1.22
14	GA	1114	CLA	CHC-C1C	4.47	1.47	1.35
14	BA	1201	CLA	CHC-C1C	4.46	1.47	1.35
14	GA	1128	CLA	C3C-C2C	4.46	1.46	1.36
14	aA	1126	CLA	CHC-C1C	4.46	1.47	1.35
14	BA	1228	CLA	CHC-C1C	4.46	1.47	1.35
14	aA	1114	CLA	CHC-C1C	4.46	1.47	1.35
14	bA	1201	CLA	CHC-C1C	4.46	1.47	1.35
14	AA	1103	CLA	O2D-CGD	4.46	1.44	1.33
14	aA	1103	CLA	O2D-CGD	4.46	1.44	1.33
14	AA	1114	CLA	CHC-C1C	4.46	1.47	1.35
14	UA	1501	CLA	C3C-C2C	4.46	1.46	1.36

Continued on next page...

PRELIMINARY VALIDATION REPORT

Continued from previous page...

Mol	Chain	Res	Type	Atoms	Z	Observed(Å)	Ideal(Å)
14	aA	1127	CLA	CHC-C1C	4.46	1.47	1.35
14	GA	1132	CLA	O2D-CGD	4.45	1.44	1.33
14	AA	1108	CLA	C3C-C2C	4.45	1.46	1.36
14	aA	1128	CLA	C3C-C2C	4.45	1.46	1.36
14	BA	1228	CLA	C3C-C2C	4.45	1.46	1.36
14	GA	1109	CLA	CHC-C1C	4.45	1.47	1.35
14	AA	1128	CLA	C3C-C2C	4.45	1.46	1.36
14	BA	1231	CLA	OBD-CAD	4.45	1.28	1.22
14	bA	1228	CLA	C3C-C2C	4.45	1.46	1.36
14	XA	1701	CLA	C3B-C2B	4.45	1.46	1.40
14	bA	1228	CLA	CHC-C1C	4.45	1.47	1.35
14	HA	1231	CLA	O2D-CGD	4.45	1.44	1.33
14	HA	1218	CLA	OBD-CAD	4.45	1.28	1.22
14	AA	1135	CLA	O2D-CGD	4.45	1.44	1.33
14	HA	1228	CLA	CHC-C1C	4.44	1.47	1.35
14	BA	1221	CLA	C3B-C2B	4.44	1.46	1.40
14	GA	1116	CLA	CHC-C1C	4.44	1.47	1.35
14	aA	1136	CLA	CHC-C1C	4.44	1.47	1.35
14	GA	1132	CLA	CHC-C1C	4.44	1.47	1.35
14	GA	1114	CLA	C3B-C2B	4.44	1.46	1.40
14	lA	1501	CLA	C3C-C2C	4.44	1.46	1.36
14	GA	1012	CLA	CHC-C1C	4.44	1.47	1.35
14	aA	1122	CLA	CHC-C1C	4.44	1.47	1.35
14	bA	1212	CLA	OBD-CAD	4.44	1.28	1.22
14	AA	1136	CLA	CHC-C1C	4.44	1.47	1.35
14	HA	1219	CLA	C3B-C2B	4.44	1.46	1.40
14	BA	1236	CLA	CHC-C1C	4.43	1.47	1.35
14	HA	1236	CLA	CHC-C1C	4.43	1.47	1.35
14	BA	1219	CLA	C3B-C2B	4.43	1.46	1.40
14	AA	1109	CLA	CHC-C1C	4.43	1.47	1.35
14	BA	1210	CLA	O2D-CGD	4.43	1.44	1.33
14	aA	1137	CLA	C3B-C2B	4.43	1.46	1.40
14	HA	1231	CLA	OBD-CAD	4.43	1.28	1.22
14	AA	1116	CLA	CHC-C1C	4.43	1.47	1.35
14	aA	1132	CLA	O2D-CGD	4.43	1.44	1.33
14	AA	1135	CLA	CHC-C1C	4.43	1.47	1.35
14	GA	1135	CLA	CHC-C1C	4.43	1.47	1.35
14	HA	1212	CLA	OBD-CAD	4.43	1.28	1.22
14	aA	1104	CLA	CHC-C1C	4.43	1.47	1.35
14	AA	1012	CLA	CHC-C1C	4.43	1.47	1.35
14	bA	1218	CLA	OBD-CAD	4.43	1.28	1.22
14	GA	1127	CLA	CHC-C1C	4.43	1.47	1.35

Continued on next page...

PRELIMINARY VALIDATION REPORT

Continued from previous page...

Mol	Chain	Res	Type	Atoms	Z	Observed(Å)	Ideal(Å)
14	GA	1122	CLA	CHC-C1C	4.43	1.47	1.35
14	HA	1219	CLA	C3C-C2C	4.43	1.46	1.36
14	GA	1137	CLA	C3B-C2B	4.43	1.46	1.40
14	aA	1135	CLA	CHC-C1C	4.43	1.47	1.35
14	aA	1132	CLA	C3C-C2C	4.42	1.46	1.36
14	aA	1116	CLA	CHC-C1C	4.42	1.47	1.35
14	GA	1118	CLA	C3C-C2C	4.42	1.46	1.36
14	HA	1226	CLA	C3C-C2C	4.42	1.46	1.36
14	HA	1232	CLA	CHC-C1C	4.42	1.47	1.35
14	bA	1236	CLA	CHC-C1C	4.42	1.47	1.35
14	BA	1212	CLA	OBD-CAD	4.42	1.28	1.22
14	lA	1502	CLA	O2D-CGD	4.42	1.44	1.33
14	LA	1501	CLA	C3C-C2C	4.42	1.46	1.36
14	HA	1220	CLA	CHC-C1C	4.42	1.47	1.35
14	AA	1132	CLA	C3C-C2C	4.42	1.46	1.36
14	AA	1132	CLA	O2D-CGD	4.42	1.44	1.33
14	BA	1219	CLA	C3C-C2C	4.41	1.46	1.36
14	BA	1216	CLA	C3C-C2C	4.41	1.46	1.36
20	aA	5005	LMG	O8-C28	4.41	1.46	1.33
14	bA	1221	CLA	C3B-C2B	4.41	1.46	1.40
14	bA	1220	CLA	CHC-C1C	4.41	1.47	1.35
14	GA	1108	CLA	OBD-CAD	4.41	1.28	1.22
14	AA	1127	CLA	CHC-C1C	4.41	1.47	1.35
14	GA	1104	CLA	CHC-C1C	4.41	1.47	1.35
14	HA	1202	CLA	O2D-CGD	4.41	1.44	1.33
14	aA	1012	CLA	CHC-C1C	4.41	1.47	1.35
14	bA	1216	CLA	C3C-C2C	4.41	1.46	1.36
14	aA	1109	CLA	CHC-C1C	4.41	1.47	1.35
14	GA	1136	CLA	CHC-C1C	4.41	1.47	1.35
14	HA	1021	CLA	O2D-CGD	4.41	1.44	1.33
14	HA	1221	CLA	C3B-C2B	4.41	1.46	1.40
14	AA	1122	CLA	CHC-C1C	4.41	1.47	1.35
14	HA	1216	CLA	C3C-C2C	4.41	1.46	1.36
14	aA	1102	CLA	C3C-C2C	4.41	1.46	1.36
14	bA	1219	CLA	C3C-C2C	4.41	1.46	1.36
14	HA	1228	CLA	OBD-CAD	4.41	1.28	1.22
14	AA	1104	CLA	CHC-C1C	4.41	1.47	1.35
14	bA	1219	CLA	C3B-C2B	4.40	1.46	1.40
14	bA	1232	CLA	CHC-C1C	4.40	1.46	1.35
14	bA	1202	CLA	O2D-CGD	4.40	1.44	1.33
14	aA	1108	CLA	OBD-CAD	4.40	1.28	1.22
14	BA	1223	CLA	CHC-C1C	4.40	1.46	1.35

Continued on next page...

PRELIMINARY VALIDATION REPORT

Continued from previous page...

Mol	Chain	Res	Type	Atoms	Z	Observed(Å)	Ideal(Å)
14	AA	1137	CLA	C3B-C2B	4.40	1.46	1.40
14	HA	1234	CLA	C3C-C2C	4.40	1.46	1.36
14	bA	1227	CLA	C3C-C2C	4.40	1.46	1.36
14	HA	1023	CLA	C1C-NC	-4.40	1.31	1.37
14	HA	1204	CLA	CHC-C1C	4.40	1.46	1.35
14	bA	1204	CLA	CHC-C1C	4.40	1.46	1.35
14	HA	1208	CLA	C3C-C2C	4.40	1.46	1.36
14	BA	1225	CLA	CHC-C1C	4.40	1.46	1.35
14	AA	1140	CLA	CHC-C1C	4.40	1.46	1.35
14	HA	1223	CLA	CHC-C1C	4.40	1.46	1.35
14	BA	1226	CLA	C3C-C2C	4.40	1.46	1.36
14	BA	1232	CLA	CHC-C1C	4.40	1.46	1.35
14	GA	1130	CLA	C3C-C2C	4.40	1.46	1.36
14	BA	1228	CLA	OBD-CAD	4.39	1.28	1.22
14	GA	1118	CLA	CHC-C1C	4.39	1.46	1.35
20	GA	5005	LMG	O8-C28	4.39	1.46	1.33
14	bA	1208	CLA	OBD-CAD	4.39	1.28	1.22
14	BA	1204	CLA	CHC-C1C	4.39	1.46	1.35
14	bA	1234	CLA	C3C-C2C	4.39	1.46	1.36
14	aA	1140	CLA	CHC-C1C	4.39	1.46	1.35
14	AA	1113	CLA	CHC-C1C	4.39	1.46	1.35
14	BA	1227	CLA	OBD-CAD	4.39	1.28	1.22
14	WA	1701	CLA	C3B-C2B	4.39	1.46	1.40
14	GA	1115	CLA	OBD-CAD	4.39	1.28	1.22
14	GA	1102	CLA	C3C-C2C	4.39	1.46	1.36
14	UA	1502	CLA	O2D-CGD	4.39	1.44	1.33
14	AA	1130	CLA	C3C-C2C	4.39	1.46	1.36
14	LA	1502	CLA	O2D-CGD	4.39	1.44	1.33
14	bA	1223	CLA	CHC-C1C	4.39	1.46	1.35
14	BA	1214	CLA	CHC-C1C	4.39	1.46	1.35
14	HA	1214	CLA	CHC-C1C	4.39	1.46	1.35
14	aA	1113	CLA	CHC-C1C	4.39	1.46	1.35
14	BA	1234	CLA	C3C-C2C	4.39	1.46	1.36
14	AA	1118	CLA	C3C-C2C	4.38	1.46	1.36
14	GA	1102	CLA	OBD-CAD	4.38	1.28	1.22
14	HA	1227	CLA	OBD-CAD	4.38	1.28	1.22
14	AA	1111	CLA	CHC-C1C	4.38	1.46	1.35
14	bA	1228	CLA	OBD-CAD	4.38	1.28	1.22
14	HA	1209	CLA	C3C-C2C	4.38	1.46	1.36
14	GA	1125	CLA	CHC-C1C	4.38	1.46	1.35
14	bA	1226	CLA	C3C-C2C	4.38	1.46	1.36
14	bA	1235	CLA	C3C-C2C	4.38	1.46	1.36

Continued on next page...

PRELIMINARY VALIDATION REPORT

Continued from previous page...

Mol	Chain	Res	Type	Atoms	Z	Observed(Å)	Ideal(Å)
20	AA	5005	LMG	O8-C28	4.38	1.46	1.33
14	AA	1115	CLA	OBD-CAD	4.38	1.28	1.22
14	BA	1202	CLA	OBD-CAD	4.38	1.28	1.22
14	bA	1021	CLA	O2D-CGD	4.38	1.44	1.33
14	AA	1118	CLA	CHC-C1C	4.38	1.46	1.35
14	BA	1208	CLA	C3C-C2C	4.38	1.46	1.36
14	GA	1110	CLA	C3C-C2C	4.38	1.46	1.36
14	HA	1227	CLA	C3C-C2C	4.38	1.46	1.36
14	GA	1103	CLA	C3B-C2B	4.38	1.46	1.40
14	HA	1225	CLA	CHC-C1C	4.37	1.46	1.35
14	bA	1214	CLA	CHC-C1C	4.37	1.46	1.35
14	AA	1108	CLA	OBD-CAD	4.37	1.28	1.22
14	GA	1133	CLA	CHC-C1C	4.37	1.46	1.35
14	BA	1208	CLA	CHC-C1C	4.37	1.46	1.35
14	GA	1124	CLA	O2D-CGD	4.37	1.44	1.33
14	bA	1208	CLA	C3C-C2C	4.37	1.46	1.36
14	bA	1209	CLA	C3C-C2C	4.37	1.46	1.36
14	BA	1220	CLA	CHC-C1C	4.37	1.46	1.35
14	GA	1111	CLA	CHC-C1C	4.37	1.46	1.35
14	BA	1202	CLA	O2D-CGD	4.37	1.44	1.33
14	bA	1225	CLA	CHC-C1C	4.37	1.46	1.35
14	aA	1118	CLA	C3C-C2C	4.37	1.46	1.36
14	aA	1130	CLA	C3C-C2C	4.37	1.46	1.36
14	BA	1209	CLA	C3C-C2C	4.37	1.46	1.36
14	HA	1235	CLA	C3C-C2C	4.37	1.46	1.36
14	BA	1023	CLA	C1C-NC	-4.37	1.31	1.37
14	bA	1202	CLA	CHC-C1C	4.37	1.46	1.35
14	AA	1102	CLA	OBD-CAD	4.37	1.28	1.22
14	GA	1140	CLA	CHC-C1C	4.37	1.46	1.35
14	aA	1125	CLA	CHC-C1C	4.37	1.46	1.35
14	UA	1503	CLA	CHC-C1C	4.37	1.46	1.35
14	AA	1124	CLA	O2D-CGD	4.37	1.44	1.33
14	kA	1401	CLA	C3B-C2B	4.37	1.46	1.40
14	AA	1102	CLA	C3C-C2C	4.37	1.46	1.36
14	BA	1021	CLA	O2D-CGD	4.37	1.44	1.33
14	BA	1227	CLA	C3C-C2C	4.37	1.46	1.36
14	TA	1401	CLA	C3B-C2B	4.36	1.46	1.40
14	AA	1110	CLA	C3C-C2C	4.36	1.45	1.36
14	AA	1107	CLA	CHC-C1C	4.36	1.46	1.35
14	AA	1114	CLA	C3B-C2B	4.36	1.46	1.40
14	aA	1102	CLA	OBD-CAD	4.36	1.28	1.22
14	GA	1119	CLA	C3C-C2C	4.36	1.45	1.36

Continued on next page...

PRELIMINARY VALIDATION REPORT

Continued from previous page...

Mol	Chain	Res	Type	Atoms	Z	Observed(Å)	Ideal(Å)
14	aA	1115	CLA	OBD-CAD	4.36	1.28	1.22
14	BA	1212	CLA	C3C-C2C	4.36	1.45	1.36
14	AA	1125	CLA	CHC-C1C	4.36	1.46	1.35
14	aA	1133	CLA	OBD-CAD	4.36	1.28	1.22
14	BA	1236	CLA	C3C-C2C	4.36	1.45	1.36
14	bA	1208	CLA	CHC-C1C	4.36	1.46	1.35
14	LA	1503	CLA	CHC-C1C	4.36	1.46	1.35
14	GA	1113	CLA	CHC-C1C	4.36	1.46	1.35
14	HA	1208	CLA	CHC-C1C	4.35	1.46	1.35
14	AA	1119	CLA	C3C-C2C	4.35	1.45	1.36
14	BA	1208	CLA	OBD-CAD	4.35	1.28	1.22
14	HA	1210	CLA	CHC-C1C	4.35	1.46	1.35
14	aA	1124	CLA	O2D-CGD	4.35	1.43	1.33
14	bA	1201	CLA	OBD-CAD	4.35	1.28	1.22
14	HA	1208	CLA	OBD-CAD	4.35	1.28	1.22
14	lA	1503	CLA	CHC-C1C	4.35	1.46	1.35
14	aA	1111	CLA	CHC-C1C	4.35	1.46	1.35
14	bA	1023	CLA	C1C-NC	-4.35	1.31	1.37
14	KA	1401	CLA	C3B-C2B	4.35	1.46	1.40
14	aA	1133	CLA	CHC-C1C	4.34	1.46	1.35
14	aA	1119	CLA	C3C-C2C	4.34	1.45	1.36
14	AA	1131	CLA	CHC-C1C	4.34	1.46	1.35
14	xA	1701	CLA	C3C-C2C	4.34	1.45	1.36
14	AA	1133	CLA	CHC-C1C	4.34	1.46	1.35
14	HA	1201	CLA	C3C-C2C	4.34	1.45	1.36
14	bA	1227	CLA	OBD-CAD	4.34	1.28	1.22
19	GA	5001	LHG	O8-C23	4.34	1.46	1.33
14	HA	1202	CLA	CHC-C1C	4.34	1.46	1.35
14	aA	1110	CLA	C3C-C2C	4.34	1.45	1.36
14	aA	1118	CLA	CHC-C1C	4.34	1.46	1.35
14	HA	1201	CLA	OBD-CAD	4.34	1.28	1.22
14	bA	1202	CLA	OBD-CAD	4.34	1.28	1.22
14	GA	1109	CLA	OBD-CAD	4.34	1.28	1.22
14	BA	1215	CLA	CHC-C1C	4.33	1.46	1.35
14	BA	1202	CLA	CHC-C1C	4.33	1.46	1.35
14	HA	1232	CLA	OBD-CAD	4.33	1.28	1.22
14	GA	1107	CLA	CHC-C1C	4.33	1.46	1.35
14	HA	1236	CLA	C3C-C2C	4.33	1.45	1.36
14	bA	1215	CLA	CHC-C1C	4.33	1.46	1.35
14	BA	1232	CLA	OBD-CAD	4.33	1.28	1.22
14	GA	1105	CLA	C3C-C2C	4.33	1.45	1.36
14	AA	1133	CLA	OBD-CAD	4.33	1.28	1.22

Continued on next page...

PRELIMINARY VALIDATION REPORT

Continued from previous page...

Mol	Chain	Res	Type	Atoms	Z	Observed(Å)	Ideal(Å)
14	AA	1105	CLA	OBD-CAD	4.33	1.28	1.22
14	HA	1223	CLA	C3C-C2C	4.33	1.45	1.36
14	HA	1215	CLA	CHC-C1C	4.33	1.46	1.35
14	bA	1216	CLA	CHC-C1C	4.33	1.46	1.35
14	bA	1236	CLA	C3C-C2C	4.32	1.45	1.36
14	BA	1223	CLA	C3C-C2C	4.32	1.45	1.36
14	bA	1201	CLA	C3C-C2C	4.32	1.45	1.36
14	bA	1216	CLA	OBD-CAD	4.32	1.28	1.22
14	BA	1210	CLA	CHC-C1C	4.32	1.46	1.35
14	BA	1212	CLA	C3B-C2B	4.32	1.46	1.40
14	HA	1216	CLA	OBD-CAD	4.32	1.28	1.22
14	AA	1132	CLA	OBD-CAD	4.32	1.28	1.22
14	bA	1210	CLA	CHC-C1C	4.32	1.46	1.35
20	lA	5007	LMG	O8-C28	4.32	1.46	1.33
14	lA	1503	CLA	C3C-C2C	4.32	1.45	1.36
14	bA	1233	CLA	C3C-C2C	4.32	1.45	1.36
14	BA	1216	CLA	CHC-C1C	4.32	1.46	1.35
14	bA	1213	CLA	C3B-C2B	4.32	1.46	1.40
14	bA	1212	CLA	C3C-C2C	4.32	1.45	1.36
14	GA	1132	CLA	OBD-CAD	4.31	1.28	1.22
14	BA	1213	CLA	C3B-C2B	4.31	1.46	1.40
14	BA	1201	CLA	C3C-C2C	4.31	1.45	1.36
14	aA	1109	CLA	OBD-CAD	4.31	1.28	1.22
14	aA	1107	CLA	CHC-C1C	4.31	1.46	1.35
19	AA	5001	LHG	O8-C23	4.31	1.46	1.33
14	BA	1235	CLA	C3C-C2C	4.31	1.45	1.36
14	HA	1232	CLA	C3C-C2C	4.31	1.45	1.36
14	AA	1103	CLA	CHC-C1C	4.31	1.46	1.35
14	HA	1220	CLA	OBD-CAD	4.31	1.28	1.22
14	GA	1105	CLA	OBD-CAD	4.31	1.28	1.22
20	LA	5007	LMG	O8-C28	4.31	1.46	1.33
14	bA	1232	CLA	OBD-CAD	4.31	1.28	1.22
19	aA	5001	LHG	O8-C23	4.31	1.46	1.33
14	bA	1223	CLA	C3C-C2C	4.31	1.45	1.36
14	aA	1105	CLA	C3C-C2C	4.31	1.45	1.36
14	GA	1133	CLA	OBD-CAD	4.30	1.28	1.22
14	BA	1225	CLA	C3B-C2B	4.30	1.46	1.40
14	GA	1131	CLA	CHC-C1C	4.30	1.46	1.35
14	AA	1133	CLA	C3C-C2C	4.30	1.45	1.36
14	aA	1106	CLA	C3C-C2C	4.30	1.45	1.36
14	GA	1133	CLA	C3C-C2C	4.30	1.45	1.36
14	BA	1201	CLA	OBD-CAD	4.30	1.28	1.22

Continued on next page...

PRELIMINARY VALIDATION REPORT

Continued from previous page...

Mol	Chain	Res	Type	Atoms	Z	Observed(Å)	Ideal(Å)
14	XA	1701	CLA	C3C-C2C	4.30	1.45	1.36
14	GA	1135	CLA	C3C-C2C	4.30	1.45	1.36
14	aA	1131	CLA	CHC-C1C	4.30	1.46	1.35
14	GA	1117	CLA	OBD-CAD	4.30	1.28	1.22
14	BA	1233	CLA	C3C-C2C	4.30	1.45	1.36
14	HA	1212	CLA	C3C-C2C	4.30	1.45	1.36
14	HA	1213	CLA	C3B-C2B	4.30	1.46	1.40
14	HA	1216	CLA	CHC-C1C	4.30	1.46	1.35
14	UA	1502	CLA	C3C-C2C	4.30	1.45	1.36
20	UA	5007	LMG	O8-C28	4.30	1.46	1.33
14	HA	1202	CLA	OBD-CAD	4.30	1.28	1.22
14	HA	1225	CLA	C3B-C2B	4.30	1.46	1.40
14	AA	1109	CLA	OBD-CAD	4.30	1.28	1.22
14	lA	1502	CLA	C3C-C2C	4.30	1.45	1.36
14	aA	1138	CLA	C3C-C2C	4.30	1.45	1.36
14	GA	1138	CLA	C3C-C2C	4.29	1.45	1.36
14	GA	1139	CLA	OBD-CAD	4.29	1.28	1.22
14	AA	1105	CLA	C3C-C2C	4.29	1.45	1.36
14	LA	1502	CLA	CHC-C1C	4.29	1.46	1.35
14	aA	1105	CLA	OBD-CAD	4.29	1.28	1.22
14	WA	1701	CLA	C3C-C2C	4.29	1.45	1.36
14	HA	1233	CLA	C3C-C2C	4.29	1.45	1.36
14	UA	1503	CLA	C3C-C2C	4.29	1.45	1.36
14	AA	1138	CLA	C3C-C2C	4.29	1.45	1.36
14	LA	1502	CLA	C3C-C2C	4.29	1.45	1.36
14	aA	1139	CLA	OBD-CAD	4.29	1.28	1.22
14	AA	1135	CLA	C3C-C2C	4.29	1.45	1.36
14	bA	1232	CLA	C3C-C2C	4.29	1.45	1.36
14	HA	1229	CLA	OBD-CAD	4.29	1.28	1.22
14	BA	1232	CLA	C3C-C2C	4.28	1.45	1.36
14	aA	1133	CLA	C3C-C2C	4.28	1.45	1.36
14	aA	1117	CLA	OBD-CAD	4.28	1.28	1.22
14	AA	1139	CLA	OBD-CAD	4.28	1.28	1.22
14	BA	1216	CLA	OBD-CAD	4.28	1.28	1.22
14	AA	1112	CLA	C3C-C2C	4.28	1.45	1.36
14	aA	1112	CLA	C3C-C2C	4.28	1.45	1.36
14	GA	1106	CLA	C3C-C2C	4.28	1.45	1.36
14	aA	1132	CLA	OBD-CAD	4.28	1.28	1.22
14	AA	1131	CLA	C3C-C2C	4.28	1.45	1.36
14	AA	1103	CLA	C3B-C2B	4.27	1.46	1.40
14	GA	1131	CLA	C3C-C2C	4.27	1.45	1.36
14	bA	1225	CLA	C3B-C2B	4.27	1.46	1.40

Continued on next page...

PRELIMINARY VALIDATION REPORT

Continued from previous page...

Mol	Chain	Res	Type	Atoms	Z	Observed(Å)	Ideal(Å)
14	GA	1103	CLA	CHC-C1C	4.27	1.46	1.35
14	BA	1229	CLA	OBD-CAD	4.27	1.28	1.22
14	aA	1103	CLA	CHC-C1C	4.27	1.46	1.35
14	AA	1117	CLA	OBD-CAD	4.27	1.28	1.22
14	HA	1238	CLA	C3C-C2C	4.27	1.45	1.36
14	aA	1103	CLA	C3B-C2B	4.27	1.46	1.40
14	GA	1137	CLA	C3C-C2C	4.27	1.45	1.36
14	aA	1135	CLA	C3C-C2C	4.27	1.45	1.36
14	aA	1137	CLA	C3C-C2C	4.27	1.45	1.36
14	aA	1138	CLA	C3B-C2B	4.26	1.46	1.40
14	LA	1503	CLA	C3C-C2C	4.26	1.45	1.36
14	bA	1224	CLA	C3B-C2B	4.26	1.46	1.40
14	aA	1111	CLA	C3B-C2B	4.26	1.46	1.40
14	bA	1228	CLA	C3B-C2B	4.26	1.46	1.40
14	bA	1211	CLA	C3C-C2C	4.26	1.45	1.36
14	GA	1111	CLA	C3B-C2B	4.26	1.46	1.40
14	bA	1217	CLA	C3B-C2B	4.26	1.46	1.40
14	UA	1502	CLA	CHC-C1C	4.26	1.46	1.35
14	aA	1110	CLA	OBD-CAD	4.26	1.28	1.22
14	bA	1238	CLA	C3C-C2C	4.26	1.45	1.36
14	AA	1138	CLA	C3B-C2B	4.26	1.46	1.40
14	AA	1104	CLA	C3B-C2B	4.26	1.46	1.40
14	aA	1131	CLA	C3C-C2C	4.25	1.45	1.36
14	BA	1023	CLA	CHC-C1C	4.25	1.46	1.35
14	GA	1140	CLA	C3C-C2C	4.25	1.45	1.36
14	HA	1217	CLA	C3C-C2C	4.25	1.45	1.36
14	GA	1113	CLA	C3C-C2C	4.25	1.45	1.36
14	GA	1104	CLA	C3B-C2B	4.25	1.46	1.40
14	HA	1021	CLA	CHC-C1C	4.25	1.46	1.35
14	AA	1110	CLA	OBD-CAD	4.25	1.28	1.22
14	GA	1109	CLA	C3C-C2C	4.25	1.45	1.36
14	GA	1138	CLA	C3B-C2B	4.25	1.46	1.40
14	BA	1215	CLA	OBD-CAD	4.25	1.28	1.22
14	bA	1212	CLA	C3B-C2B	4.25	1.46	1.40
14	AA	1137	CLA	C3C-C2C	4.25	1.45	1.36
14	HA	1211	CLA	C3C-C2C	4.25	1.45	1.36
14	AA	1137	CLA	CHC-C1C	4.25	1.46	1.35
14	AA	1113	CLA	C3C-C2C	4.25	1.45	1.36
14	BA	1238	CLA	C3C-C2C	4.25	1.45	1.36
14	lA	1502	CLA	CHC-C1C	4.24	1.46	1.35
14	BA	1217	CLA	C3B-C2B	4.24	1.46	1.40
14	BA	1210	CLA	OBD-CAD	4.24	1.28	1.22

Continued on next page...

PRELIMINARY VALIDATION REPORT

Continued from previous page...

Mol	Chain	Res	Type	Atoms	Z	Observed(Å)	Ideal(Å)
14	HA	1218	CLA	C3C-C2C	4.24	1.45	1.36
14	bA	1220	CLA	OBD-CAD	4.24	1.28	1.22
14	AA	1106	CLA	C3C-C2C	4.24	1.45	1.36
14	BA	1022	CLA	C3C-C2C	4.24	1.45	1.36
13	AA	1011	CL0	C3C-C2C	4.24	1.45	1.36
14	HA	1023	CLA	CHC-C1C	4.24	1.46	1.35
14	BA	1021	CLA	CHC-C1C	4.24	1.46	1.35
14	HA	1224	CLA	C3B-C2B	4.24	1.46	1.40
14	GA	1112	CLA	C3C-C2C	4.24	1.45	1.36
14	bA	1023	CLA	CHC-C1C	4.24	1.46	1.35
14	HA	1213	CLA	C3C-C2C	4.24	1.45	1.36
14	BA	1224	CLA	C3B-C2B	4.24	1.46	1.40
14	aA	1137	CLA	CHC-C1C	4.24	1.46	1.35
14	bA	1231	CLA	C3C-C2C	4.24	1.45	1.36
14	AA	1123	CLA	C3C-C2C	4.24	1.45	1.36
14	AA	1115	CLA	C3C-C2C	4.24	1.45	1.36
14	0	1012	CLA	C3C-C2C	4.24	1.45	1.36
14	AA	1109	CLA	C3C-C2C	4.24	1.45	1.36
14	GA	1111	CLA	O2D-CGD	4.23	1.43	1.33
14	HA	1201	CLA	C3B-C2B	4.23	1.46	1.40
14	bA	1224	CLA	OBD-CAD	4.23	1.28	1.22
14	BA	1211	CLA	C3C-C2C	4.23	1.45	1.36
14	AA	1111	CLA	O2D-CGD	4.23	1.43	1.33
14	BA	1217	CLA	C3C-C2C	4.23	1.45	1.36
14	HA	1217	CLA	C3B-C2B	4.23	1.46	1.40
14	aA	1113	CLA	C3C-C2C	4.23	1.45	1.36
14	bA	1217	CLA	C3C-C2C	4.23	1.45	1.36
14	GA	1137	CLA	CHC-C1C	4.23	1.46	1.35
14	BA	1228	CLA	C3B-C2B	4.23	1.46	1.40
14	HA	1224	CLA	OBD-CAD	4.23	1.28	1.22
14	BA	1218	CLA	C3C-C2C	4.23	1.45	1.36
14	BA	1213	CLA	C3C-C2C	4.23	1.45	1.36
14	BA	1231	CLA	C3C-C2C	4.23	1.45	1.36
14	BA	1022	CLA	O2D-CGD	4.23	1.43	1.33
14	BA	1224	CLA	OBD-CAD	4.23	1.28	1.22
14	bA	1021	CLA	CHC-C1C	4.22	1.46	1.35
14	HA	1210	CLA	C3B-C2B	4.22	1.46	1.40
14	HA	1212	CLA	C3B-C2B	4.22	1.46	1.40
14	aA	1111	CLA	O2D-CGD	4.22	1.43	1.33
14	HA	1228	CLA	C3B-C2B	4.22	1.46	1.40
14	AA	1127	CLA	C3C-C2C	4.22	1.45	1.36
14	bA	1213	CLA	C3C-C2C	4.22	1.45	1.36

Continued on next page...

PRELIMINARY VALIDATION REPORT

Continued from previous page...

Mol	Chain	Res	Type	Atoms	Z	Observed(Å)	Ideal(Å)
14	bA	1022	CLA	C3C-C2C	4.22	1.45	1.36
14	bA	1215	CLA	OBD-CAD	4.22	1.28	1.22
14	HA	1231	CLA	C3C-C2C	4.22	1.45	1.36
13	aA	1011	CL0	C3C-C2C	4.22	1.45	1.36
14	aA	1104	CLA	C3B-C2B	4.22	1.46	1.40
14	AA	1111	CLA	C3B-C2B	4.21	1.46	1.40
14	bA	1204	CLA	C3C-C2C	4.21	1.45	1.36
14	BA	1219	CLA	OBD-CAD	4.21	1.28	1.22
14	aA	1109	CLA	C3C-C2C	4.21	1.45	1.36
20	RA	5006	LMG	O8-C28	4.21	1.45	1.33
14	bA	1229	CLA	OBD-CAD	4.21	1.28	1.22
14	HA	1206	CLA	CHC-C1C	4.21	1.46	1.35
14	aA	1140	CLA	C3C-C2C	4.21	1.45	1.36
14	bA	1218	CLA	C3C-C2C	4.21	1.45	1.36
14	HA	1215	CLA	OBD-CAD	4.21	1.28	1.22
14	GA	1123	CLA	C3C-C2C	4.21	1.45	1.36
14	bA	1219	CLA	OBD-CAD	4.21	1.28	1.22
14	0	1012	CLA	O2D-CGD	4.20	1.43	1.33
14	BA	1220	CLA	OBD-CAD	4.20	1.28	1.22
14	aA	1115	CLA	C3C-C2C	4.20	1.45	1.36
14	GA	1115	CLA	C3C-C2C	4.20	1.45	1.36
14	AA	1135	CLA	OBD-CAD	4.20	1.28	1.22
14	bA	1226	CLA	CHC-C1C	4.20	1.46	1.35
18	RA	4018	BCR	C11-C12	-4.20	1.23	1.34
14	AA	1140	CLA	C3C-C2C	4.20	1.45	1.36
14	aA	1127	CLA	OBD-CAD	4.19	1.28	1.22
14	AA	1013	CLA	OBD-CAD	4.19	1.28	1.22
14	GA	1125	CLA	C3C-C2C	4.19	1.45	1.36
14	HA	1226	CLA	OBD-CAD	4.19	1.28	1.22
14	aA	1127	CLA	C3C-C2C	4.19	1.45	1.36
14	aA	1128	CLA	CHC-C1C	4.19	1.46	1.35
13	GA	1011	CL0	C3C-C2C	4.19	1.45	1.36
14	BA	1201	CLA	C3B-C2B	4.19	1.46	1.40
14	GA	1119	CLA	OBD-CAD	4.19	1.28	1.22
14	HA	1204	CLA	C3C-C2C	4.19	1.45	1.36
14	GA	1110	CLA	OBD-CAD	4.19	1.28	1.22
14	GA	1127	CLA	C3C-C2C	4.19	1.45	1.36
14	aA	1124	CLA	C3B-C2B	4.19	1.46	1.40
20	iA	5006	LMG	O8-C28	4.18	1.45	1.33
14	BA	1202	CLA	C3C-C2C	4.18	1.45	1.36
14	bA	1023	CLA	C3C-C2C	4.18	1.45	1.36
14	aA	1126	CLA	OBD-CAD	4.18	1.28	1.22

Continued on next page...

PRELIMINARY VALIDATION REPORT

Continued from previous page...

Mol	Chain	Res	Type	Atoms	Z	Observed(Å)	Ideal(Å)
14	GA	1128	CLA	CHC-C1C	4.18	1.46	1.35
14	GA	1013	CLA	OBD-CAD	4.18	1.28	1.22
14	bA	1206	CLA	CHC-C1C	4.18	1.46	1.35
14	AA	1104	CLA	C3C-C2C	4.18	1.45	1.36
18	IA	4018	BCR	C11-C12	-4.18	1.23	1.34
14	BA	1226	CLA	CHC-C1C	4.18	1.46	1.35
14	bA	1022	CLA	O2D-CGD	4.18	1.43	1.33
19	bA	5004	LHG	O8-C23	4.18	1.45	1.33
14	aA	1012	CLA	C3C-C2C	4.18	1.45	1.36
18	iA	4018	BCR	C11-C12	-4.18	1.23	1.34
14	HA	1209	CLA	OBD-CAD	4.17	1.28	1.22
14	aA	1013	CLA	OBD-CAD	4.17	1.28	1.22
14	AA	1012	CLA	C3C-C2C	4.17	1.45	1.36
14	GA	1136	CLA	O2D-CGD	4.17	1.43	1.33
14	aA	1125	CLA	C3C-C2C	4.17	1.45	1.36
14	bA	1201	CLA	C3B-C2B	4.17	1.46	1.40
14	HA	1219	CLA	OBD-CAD	4.17	1.28	1.22
14	HA	1226	CLA	CHC-C1C	4.17	1.46	1.35
14	GA	1129	CLA	C3C-C2C	4.17	1.45	1.36
14	bA	1215	CLA	C3C-C2C	4.17	1.45	1.36
14	BA	1209	CLA	OBD-CAD	4.17	1.28	1.22
19	BA	5004	LHG	O8-C23	4.17	1.45	1.33
20	IA	5006	LMG	O8-C28	4.17	1.45	1.33
14	GA	1124	CLA	C3B-C2B	4.17	1.46	1.40
14	HA	1210	CLA	OBD-CAD	4.17	1.28	1.22
14	aA	1136	CLA	O2D-CGD	4.17	1.43	1.33
14	AA	1122	CLA	OBD-CAD	4.17	1.28	1.22
14	BA	1226	CLA	OBD-CAD	4.17	1.28	1.22
14	AA	1136	CLA	O2D-CGD	4.17	1.43	1.33
14	AA	1128	CLA	CHC-C1C	4.17	1.46	1.35
14	GA	1012	CLA	C3C-C2C	4.17	1.45	1.36
14	BA	1206	CLA	CHC-C1C	4.16	1.46	1.35
14	bA	1210	CLA	OBD-CAD	4.16	1.28	1.22
14	aA	1122	CLA	OBD-CAD	4.16	1.28	1.22
14	HA	1202	CLA	C3C-C2C	4.16	1.45	1.36
14	bA	1226	CLA	OBD-CAD	4.16	1.28	1.22
14	GA	1135	CLA	OBD-CAD	4.16	1.28	1.22
14	aA	1123	CLA	C3C-C2C	4.16	1.45	1.36
14	bA	1202	CLA	C3C-C2C	4.16	1.45	1.36
14	AA	1127	CLA	OBD-CAD	4.16	1.28	1.22
14	AA	1129	CLA	C3C-C2C	4.16	1.45	1.36
14	HA	1023	CLA	C3C-C2C	4.16	1.45	1.36

Continued on next page...

PRELIMINARY VALIDATION REPORT

Continued from previous page...

Mol	Chain	Res	Type	Atoms	Z	Observed(Å)	Ideal(Å)
19	HA	5004	LHG	O8-C23	4.16	1.45	1.33
20	aA	5005	LMG	O7-C10	4.16	1.46	1.34
14	GA	1104	CLA	OBD-CAD	4.16	1.28	1.22
14	aA	1117	CLA	C3C-C2C	4.16	1.45	1.36
14	BA	1210	CLA	C3B-C2B	4.16	1.46	1.40
14	AA	1109	CLA	C3B-C2B	4.15	1.46	1.40
14	bA	1236	CLA	OBD-CAD	4.15	1.28	1.22
14	BA	1236	CLA	OBD-CAD	4.15	1.28	1.22
14	LA	1503	CLA	C3B-C2B	4.15	1.46	1.40
14	BA	1023	CLA	C3C-C2C	4.15	1.45	1.36
14	GA	1126	CLA	OBD-CAD	4.15	1.28	1.22
14	aA	1104	CLA	C3C-C2C	4.15	1.45	1.36
14	AA	1101	CLA	C3C-C2C	4.15	1.45	1.36
14	AA	1136	CLA	C3C-C2C	4.15	1.45	1.36
14	AA	1119	CLA	OBD-CAD	4.15	1.28	1.22
14	BA	1205	CLA	C3C-C2C	4.15	1.45	1.36
14	HA	1222	CLA	OBD-CAD	4.15	1.28	1.22
14	aA	1135	CLA	OBD-CAD	4.15	1.28	1.22
14	AA	1126	CLA	OBD-CAD	4.15	1.28	1.22
14	aA	1107	CLA	C3C-C2C	4.15	1.45	1.36
14	HA	1215	CLA	C3C-C2C	4.14	1.45	1.36
14	BA	1215	CLA	C3C-C2C	4.14	1.45	1.36
14	bA	1239	CLA	OBD-CAD	4.14	1.28	1.22
14	aA	1129	CLA	C3C-C2C	4.14	1.45	1.36
14	GA	1136	CLA	C3C-C2C	4.14	1.45	1.36
14	bA	1222	CLA	OBD-CAD	4.14	1.28	1.22
14	GA	1127	CLA	OBD-CAD	4.14	1.28	1.22
14	HA	1239	CLA	OBD-CAD	4.14	1.28	1.22
14	lA	1503	CLA	C3B-C2B	4.14	1.46	1.40
14	AA	1124	CLA	C3B-C2B	4.14	1.46	1.40
14	BA	1207	CLA	C3B-C2B	4.14	1.46	1.40
14	bA	1210	CLA	C3B-C2B	4.14	1.46	1.40
14	BA	1204	CLA	C3C-C2C	4.14	1.45	1.36
14	HA	1205	CLA	C3C-C2C	4.14	1.45	1.36
14	HA	1236	CLA	OBD-CAD	4.14	1.28	1.22
20	GA	5005	LMG	O7-C10	4.13	1.46	1.34
14	HA	1225	CLA	C3C-C2C	4.13	1.45	1.36
14	aA	1136	CLA	C3C-C2C	4.13	1.45	1.36
20	HA	5002	LMG	O8-C28	4.13	1.45	1.33
14	GA	1122	CLA	OBD-CAD	4.13	1.28	1.22
14	GA	1109	CLA	C3B-C2B	4.13	1.46	1.40
14	GA	1101	CLA	C3C-C2C	4.13	1.45	1.36

Continued on next page...

PRELIMINARY VALIDATION REPORT

Continued from previous page...

Mol	Chain	Res	Type	Atoms	Z	Observed(Å)	Ideal(Å)
14	HA	1224	CLA	C3C-C2C	4.13	1.45	1.36
14	GA	1104	CLA	C3C-C2C	4.13	1.45	1.36
14	BA	1220	CLA	C3C-C2C	4.13	1.45	1.36
14	AA	1125	CLA	C3C-C2C	4.13	1.45	1.36
20	AA	5005	LMG	O7-C10	4.13	1.46	1.34
14	BA	1222	CLA	OBD-CAD	4.13	1.28	1.22
14	BA	1224	CLA	C3C-C2C	4.13	1.45	1.36
14	GA	1013	CLA	C3C-C2C	4.12	1.45	1.36
14	AA	1107	CLA	C3C-C2C	4.12	1.45	1.36
14	aA	1013	CLA	C3C-C2C	4.12	1.45	1.36
14	bA	1209	CLA	OBD-CAD	4.12	1.28	1.22
14	GA	1117	CLA	C3C-C2C	4.12	1.45	1.36
14	BA	1225	CLA	C3C-C2C	4.12	1.45	1.36
14	UA	1503	CLA	C3B-C2B	4.12	1.46	1.40
14	aA	1119	CLA	OBD-CAD	4.12	1.28	1.22
14	BA	1225	CLA	OBD-CAD	4.12	1.28	1.22
14	aA	1124	CLA	OBD-CAD	4.12	1.28	1.22
14	GA	1107	CLA	C3C-C2C	4.12	1.45	1.36
14	AA	1013	CLA	C3C-C2C	4.12	1.45	1.36
14	bA	1205	CLA	C3C-C2C	4.11	1.45	1.36
14	bA	1224	CLA	C3C-C2C	4.11	1.45	1.36
14	HA	1220	CLA	C3C-C2C	4.11	1.45	1.36
14	aA	1101	CLA	C3C-C2C	4.11	1.45	1.36
20	BA	5002	LMG	O8-C28	4.11	1.45	1.33
14	aA	1119	CLA	C3B-C2B	4.11	1.46	1.40
20	bA	5002	LMG	O8-C28	4.11	1.45	1.33
14	AA	1117	CLA	C3C-C2C	4.11	1.45	1.36
20	LA	5007	LMG	O7-C10	4.10	1.46	1.34
14	aA	1104	CLA	OBD-CAD	4.10	1.28	1.22
14	GA	1138	CLA	OBD-CAD	4.10	1.28	1.22
14	bA	1225	CLA	OBD-CAD	4.10	1.28	1.22
14	GA	1124	CLA	OBD-CAD	4.10	1.28	1.22
14	AA	1116	CLA	C3C-C2C	4.10	1.45	1.36
20	lA	5007	LMG	O7-C10	4.10	1.46	1.34
14	bA	1207	CLA	C3B-C2B	4.10	1.46	1.40
14	bA	1220	CLA	C3C-C2C	4.10	1.45	1.36
20	UA	5007	LMG	O7-C10	4.10	1.46	1.34
13	GA	1011	CL0	CHC-C1C	4.10	1.46	1.35
14	HA	1211	CLA	OBD-CAD	4.10	1.28	1.22
14	aA	1129	CLA	C3B-C2B	4.10	1.46	1.40
14	AA	1124	CLA	OBD-CAD	4.10	1.28	1.22
14	bA	1225	CLA	C3C-C2C	4.09	1.45	1.36

Continued on next page...

PRELIMINARY VALIDATION REPORT

Continued from previous page...

Mol	Chain	Res	Type	Atoms	Z	Observed(Å)	Ideal(Å)
14	aA	1106	CLA	OBD-CAD	4.09	1.28	1.22
14	HA	1207	CLA	C3B-C2B	4.09	1.46	1.40
14	GA	1112	CLA	C3B-C2B	4.09	1.46	1.40
14	AA	1110	CLA	C3B-C2B	4.09	1.46	1.40
14	GA	1116	CLA	C3C-C2C	4.09	1.45	1.36
14	AA	1128	CLA	OBD-CAD	4.09	1.28	1.22
14	GA	1118	CLA	C3B-C2B	4.09	1.46	1.40
14	aA	1117	CLA	C3B-C2B	4.08	1.46	1.40
14	GA	1106	CLA	C3B-C2B	4.08	1.46	1.40
14	AA	1104	CLA	OBD-CAD	4.08	1.28	1.22
13	aA	1011	CL0	CHC-C1C	4.08	1.46	1.35
14	bA	1217	CLA	OBD-CAD	4.08	1.28	1.22
13	AA	1011	CL0	CHC-C1C	4.08	1.46	1.35
14	GA	1106	CLA	OBD-CAD	4.08	1.28	1.22
14	bA	1211	CLA	OBD-CAD	4.08	1.28	1.22
14	aA	1118	CLA	C3B-C2B	4.08	1.46	1.40
14	aA	1116	CLA	C3C-C2C	4.08	1.45	1.36
14	BA	1211	CLA	OBD-CAD	4.08	1.28	1.22
14	aA	1101	CLA	OBD-CAD	4.07	1.28	1.22
14	GA	1117	CLA	C3B-C2B	4.07	1.46	1.40
14	BA	1021	CLA	OBD-CAD	4.07	1.28	1.22
14	AA	1106	CLA	C3B-C2B	4.07	1.46	1.40
14	bA	1023	CLA	OBD-CAD	4.07	1.28	1.22
14	GA	1140	CLA	OBD-CAD	4.07	1.28	1.22
14	GA	1128	CLA	OBD-CAD	4.07	1.28	1.22
14	AA	1140	CLA	OBD-CAD	4.07	1.28	1.22
14	BA	1239	CLA	OBD-CAD	4.07	1.28	1.22
14	aA	1138	CLA	OBD-CAD	4.07	1.28	1.22
14	GA	1119	CLA	C3B-C2B	4.07	1.46	1.40
14	AA	1117	CLA	C3B-C2B	4.07	1.46	1.40
14	GA	1101	CLA	OBD-CAD	4.07	1.28	1.22
14	aA	1140	CLA	OBD-CAD	4.07	1.28	1.22
14	HA	1225	CLA	OBD-CAD	4.06	1.28	1.22
14	HA	1023	CLA	OBD-CAD	4.06	1.28	1.22
14	BA	1206	CLA	C3C-C2C	4.06	1.45	1.36
14	aA	1109	CLA	C3B-C2B	4.06	1.46	1.40
14	XA	1701	CLA	OBD-CAD	4.06	1.28	1.22
14	AA	1112	CLA	C3B-C2B	4.06	1.46	1.40
14	AA	1118	CLA	C3B-C2B	4.06	1.46	1.40
14	aA	1112	CLA	C3B-C2B	4.06	1.46	1.40
14	AA	1138	CLA	OBD-CAD	4.06	1.28	1.22
14	HA	1217	CLA	OBD-CAD	4.05	1.28	1.22

Continued on next page...

PRELIMINARY VALIDATION REPORT

Continued from previous page...

Mol	Chain	Res	Type	Atoms	Z	Observed(Å)	Ideal(Å)
14	GA	1013	CLA	C3D-C2D	4.05	1.47	1.39
14	BA	1217	CLA	OBD-CAD	4.05	1.28	1.22
14	aA	1110	CLA	C3B-C2B	4.05	1.46	1.40
14	GA	1125	CLA	C1C-NC	-4.05	1.31	1.37
14	aA	1106	CLA	C3B-C2B	4.05	1.46	1.40
14	AA	1125	CLA	C1C-NC	-4.05	1.31	1.37
14	GA	1101	CLA	C3B-C2B	4.05	1.46	1.40
14	AA	1013	CLA	C3D-C2D	4.04	1.47	1.39
14	AA	1101	CLA	OBD-CAD	4.04	1.28	1.22
14	GA	1117	CLA	CHC-C1C	4.04	1.46	1.35
14	bA	1206	CLA	C3C-C2C	4.04	1.45	1.36
14	BA	1239	CLA	CHC-C1C	4.04	1.46	1.35
14	GA	1110	CLA	C3B-C2B	4.04	1.46	1.40
14	bA	1239	CLA	CHC-C1C	4.04	1.45	1.35
14	AA	1106	CLA	OBD-CAD	4.03	1.28	1.22
14	AA	1119	CLA	C3B-C2B	4.03	1.46	1.40
14	GA	1129	CLA	C3B-C2B	4.03	1.46	1.40
20	iA	5006	LMG	O7-C10	4.03	1.45	1.34
14	HA	1239	CLA	CHC-C1C	4.03	1.45	1.35
14	aA	1013	CLA	C3D-C2D	4.03	1.47	1.39
14	GA	1122	CLA	C3B-C2B	4.03	1.46	1.40
14	BA	1023	CLA	OBD-CAD	4.03	1.28	1.22
14	AA	1129	CLA	C3B-C2B	4.03	1.46	1.40
14	HA	1021	CLA	OBD-CAD	4.03	1.28	1.22
14	AA	1119	CLA	C3D-C2D	4.03	1.47	1.39
14	LA	1501	CLA	OBD-CAD	4.03	1.28	1.22
14	AA	1117	CLA	CHC-C1C	4.03	1.45	1.35
14	HA	1207	CLA	C3D-C2D	4.03	1.47	1.39
14	GA	1116	CLA	OBD-CAD	4.02	1.28	1.22
14	aA	1128	CLA	OBD-CAD	4.02	1.28	1.22
18	GA	4008	BCR	C11-C12	-4.02	1.24	1.34
14	HA	1232	CLA	C3B-C2B	4.02	1.46	1.40
18	AA	4008	BCR	C11-C12	-4.02	1.24	1.34
14	HA	1206	CLA	C3C-C2C	4.02	1.45	1.36
14	HA	1207	CLA	OBD-CAD	4.02	1.28	1.22
14	bA	1232	CLA	C3B-C2B	4.02	1.46	1.40
14	aA	1125	CLA	C1C-NC	-4.02	1.31	1.37
18	aA	4008	BCR	C11-C12	-4.02	1.24	1.34
20	IA	5006	LMG	O7-C10	4.02	1.45	1.34
14	bA	1221	CLA	C3C-C2C	4.01	1.45	1.36
14	xA	1701	CLA	OBD-CAD	4.01	1.28	1.22
20	RA	5006	LMG	O7-C10	4.01	1.45	1.34

Continued on next page...

PRELIMINARY VALIDATION REPORT

Continued from previous page...

Mol	Chain	Res	Type	Atoms	Z	Observed(Å)	Ideal(Å)
14	HA	1238	CLA	OBD-CAD	4.01	1.28	1.22
14	aA	1130	CLA	C3B-C2B	4.01	1.45	1.40
14	aA	1117	CLA	CHC-C1C	4.01	1.45	1.35
14	AA	1140	CLA	C3B-C2B	4.00	1.45	1.40
14	BA	1232	CLA	C3B-C2B	4.00	1.45	1.40
14	AA	1130	CLA	C3B-C2B	4.00	1.45	1.40
14	BA	1207	CLA	OBD-CAD	4.00	1.28	1.22
14	BA	1221	CLA	C3C-C2C	4.00	1.45	1.36
14	bA	1021	CLA	OBD-CAD	4.00	1.28	1.22
14	WA	1701	CLA	OBD-CAD	4.00	1.28	1.22
14	aA	1116	CLA	OBD-CAD	4.00	1.28	1.22
14	bA	1207	CLA	CHC-C1C	4.00	1.45	1.35
14	lA	1503	CLA	OBD-CAD	4.00	1.28	1.22
14	GA	1012	CLA	OBD-CAD	4.00	1.28	1.22
14	HA	1221	CLA	C3C-C2C	4.00	1.45	1.36
14	AA	1139	CLA	C3C-C2C	4.00	1.45	1.36
14	BA	1022	CLA	OBD-CAD	4.00	1.28	1.22
14	BA	1207	CLA	C3D-C2D	3.99	1.47	1.39
14	bA	1207	CLA	C3D-C2D	3.99	1.47	1.39
14	bA	1207	CLA	OBD-CAD	3.99	1.28	1.22
14	aA	1131	CLA	OBD-CAD	3.99	1.28	1.22
13	AA	1011	CL0	OBD-CAD	3.99	1.28	1.22
14	GA	1130	CLA	C3B-C2B	3.99	1.45	1.40
14	BA	1234	CLA	O2D-CGD	3.99	1.43	1.33
14	aA	1119	CLA	C3D-C2D	3.99	1.47	1.39
14	HA	1234	CLA	O2D-CGD	3.99	1.43	1.33
14	HA	1207	CLA	CHC-C1C	3.99	1.45	1.35
14	GA	1118	CLA	OBD-CAD	3.98	1.28	1.22
14	aA	1136	CLA	OBD-CAD	3.98	1.28	1.22
14	AA	1128	CLA	C3D-C2D	3.98	1.47	1.39
14	bA	1234	CLA	O2D-CGD	3.98	1.43	1.33
14	lA	1501	CLA	OBD-CAD	3.98	1.28	1.22
13	aA	1011	CL0	OBD-CAD	3.98	1.28	1.22
14	GA	1139	CLA	C3C-C2C	3.98	1.45	1.36
14	UA	1503	CLA	OBD-CAD	3.98	1.28	1.22
14	AA	1116	CLA	OBD-CAD	3.98	1.28	1.22
14	GA	1111	CLA	C3C-C2C	3.97	1.45	1.36
14	AA	1131	CLA	OBD-CAD	3.97	1.28	1.22
14	GA	1117	CLA	C3D-C2D	3.97	1.47	1.39
14	GA	1013	CLA	C3B-C2B	3.97	1.45	1.40
14	GA	1119	CLA	C3D-C2D	3.97	1.47	1.39
14	aA	1101	CLA	C3B-C2B	3.97	1.45	1.40

Continued on next page...

PRELIMINARY VALIDATION REPORT

Continued from previous page...

Mol	Chain	Res	Type	Atoms	Z	Observed(Å)	Ideal(Å)
14	BA	1207	CLA	CHC-C1C	3.97	1.45	1.35
14	bA	1022	CLA	OBD-CAD	3.97	1.28	1.22
14	AA	1012	CLA	O2D-CGD	3.97	1.43	1.33
14	BA	1204	CLA	OBD-CAD	3.97	1.28	1.22
14	AA	1111	CLA	C3C-C2C	3.97	1.45	1.36
14	GA	1128	CLA	C3D-C2D	3.96	1.47	1.39
14	bA	1214	CLA	OBD-CAD	3.96	1.27	1.22
14	aA	1139	CLA	C3C-C2C	3.96	1.45	1.36
14	UA	1501	CLA	OBD-CAD	3.96	1.27	1.22
14	aA	1118	CLA	OBD-CAD	3.96	1.27	1.22
14	aA	1128	CLA	C3D-C2D	3.96	1.47	1.39
14	bA	1204	CLA	OBD-CAD	3.96	1.27	1.22
14	BA	1238	CLA	OBD-CAD	3.96	1.27	1.22
14	GA	1131	CLA	OBD-CAD	3.96	1.27	1.22
14	AA	1013	CLA	C3B-C2B	3.96	1.45	1.40
14	HA	1203	CLA	C3B-C2B	3.96	1.45	1.40
14	aA	1013	CLA	C3B-C2B	3.96	1.45	1.40
14	BA	1216	CLA	C3D-C2D	3.96	1.47	1.39
14	aA	1122	CLA	C3B-C2B	3.95	1.45	1.40
14	aA	1117	CLA	C3D-C2D	3.95	1.47	1.39
14	0	1012	CLA	OBD-CAD	3.95	1.27	1.22
14	AA	1012	CLA	OBD-CAD	3.95	1.27	1.22
14	AA	1111	CLA	OBD-CAD	3.95	1.27	1.22
14	aA	1111	CLA	OBD-CAD	3.95	1.27	1.22
14	HA	1220	CLA	C3D-C2D	3.95	1.47	1.39
14	aA	1012	CLA	OBD-CAD	3.95	1.27	1.22
14	HA	1204	CLA	OBD-CAD	3.95	1.27	1.22
14	GA	1136	CLA	OBD-CAD	3.94	1.27	1.22
14	HA	1216	CLA	C3D-C2D	3.94	1.47	1.39
16	GA	2001	PQN	C10-C5	3.94	1.47	1.40
14	BA	1214	CLA	OBD-CAD	3.94	1.27	1.22
14	HA	1209	CLA	C3B-C2B	3.94	1.45	1.40
14	AA	1101	CLA	C3B-C2B	3.94	1.45	1.40
14	HA	1226	CLA	C3D-C2D	3.94	1.47	1.39
14	bA	1221	CLA	OBD-CAD	3.94	1.27	1.22
14	AA	1136	CLA	OBD-CAD	3.94	1.27	1.22
14	bA	1203	CLA	C3B-C2B	3.94	1.45	1.40
14	aA	1122	CLA	C3C-C2C	3.94	1.45	1.36
14	aA	1111	CLA	C3C-C2C	3.94	1.45	1.36
14	bA	1220	CLA	C3D-C2D	3.94	1.47	1.39
14	AA	1118	CLA	OBD-CAD	3.94	1.27	1.22
14	GA	1122	CLA	C3C-C2C	3.94	1.45	1.36

Continued on next page...

PRELIMINARY VALIDATION REPORT

Continued from previous page...

Mol	Chain	Res	Type	Atoms	Z	Observed(Å)	Ideal(Å)
14	bA	1238	CLA	OBD-CAD	3.94	1.27	1.22
14	aA	1012	CLA	O2D-CGD	3.93	1.42	1.33
14	BA	1205	CLA	OBD-CAD	3.93	1.27	1.22
14	bA	1216	CLA	C3D-C2D	3.93	1.47	1.39
14	AA	1122	CLA	C3B-C2B	3.93	1.45	1.40
14	AA	1117	CLA	C3D-C2D	3.93	1.47	1.39
14	BA	1220	CLA	C3D-C2D	3.93	1.47	1.39
14	GA	1126	CLA	C3B-C2B	3.93	1.45	1.40
14	HA	1221	CLA	OBD-CAD	3.93	1.27	1.22
14	HA	1206	CLA	C3D-C2D	3.92	1.47	1.39
14	GA	1114	CLA	C3D-C2D	3.92	1.47	1.39
14	GA	1012	CLA	O2D-CGD	3.92	1.42	1.33
14	AA	1103	CLA	OBD-CAD	3.92	1.27	1.22
14	BA	1235	CLA	OBD-CAD	3.92	1.27	1.22
14	bA	1226	CLA	C3D-C2D	3.92	1.47	1.39
14	HA	1214	CLA	OBD-CAD	3.92	1.27	1.22
14	aA	1140	CLA	C3B-C2B	3.92	1.45	1.40
14	aA	1114	CLA	C3D-C2D	3.92	1.47	1.39
14	HA	1235	CLA	OBD-CAD	3.91	1.27	1.22
14	bA	1203	CLA	C3C-C2C	3.91	1.45	1.36
14	HA	1203	CLA	C3C-C2C	3.91	1.45	1.36
19	aA	5001	LHG	O7-C7	3.91	1.45	1.34
13	GA	1011	CL0	OBD-CAD	3.91	1.27	1.22
14	GA	1111	CLA	OBD-CAD	3.91	1.27	1.22
19	bA	5004	LHG	O7-C7	3.91	1.45	1.34
14	LA	1503	CLA	OBD-CAD	3.91	1.27	1.22
19	HA	5004	LHG	O7-C7	3.91	1.45	1.34
14	bA	1205	CLA	OBD-CAD	3.91	1.27	1.22
14	BA	1203	CLA	C3C-C2C	3.91	1.45	1.36
14	GA	1140	CLA	C3B-C2B	3.91	1.45	1.40
14	HA	1207	CLA	C3C-C2C	3.90	1.45	1.36
14	BA	1209	CLA	C3B-C2B	3.90	1.45	1.40
14	bA	1210	CLA	C3C-C2C	3.90	1.45	1.36
14	aA	1126	CLA	C3B-C2B	3.90	1.45	1.40
14	BA	1226	CLA	C3D-C2D	3.90	1.47	1.39
14	bA	1214	CLA	C3D-C2D	3.90	1.47	1.39
16	AA	2001	PQN	C10-C5	3.90	1.47	1.40
14	bA	1206	CLA	C3D-C2D	3.90	1.47	1.39
14	bA	1235	CLA	OBD-CAD	3.90	1.27	1.22
14	GA	1113	CLA	OBD-CAD	3.90	1.27	1.22
14	bA	1209	CLA	C3B-C2B	3.90	1.45	1.40
14	AA	1114	CLA	C3D-C2D	3.90	1.47	1.39

Continued on next page...

PRELIMINARY VALIDATION REPORT

Continued from previous page...

Mol	Chain	Res	Type	Atoms	Z	Observed(Å)	Ideal(Å)
14	BA	1206	CLA	C3D-C2D	3.90	1.47	1.39
16	aA	2001	PQN	C10-C5	3.89	1.47	1.40
14	HA	1205	CLA	OBD-CAD	3.89	1.27	1.22
14	GA	1129	CLA	OBD-CAD	3.89	1.27	1.22
14	BA	1207	CLA	C3C-C2C	3.89	1.44	1.36
19	AA	5001	LHG	O7-C7	3.89	1.45	1.34
14	BA	1214	CLA	C3D-C2D	3.89	1.47	1.39
14	GA	1103	CLA	OBD-CAD	3.89	1.27	1.22
14	HA	1214	CLA	C3D-C2D	3.89	1.47	1.39
14	AA	1122	CLA	C3C-C2C	3.88	1.44	1.36
14	bA	1207	CLA	C3C-C2C	3.88	1.44	1.36
14	AA	1126	CLA	C3B-C2B	3.88	1.45	1.40
14	BA	1221	CLA	OBD-CAD	3.88	1.27	1.22
14	BA	1203	CLA	C3B-C2B	3.88	1.45	1.40
14	aA	1113	CLA	OBD-CAD	3.88	1.27	1.22
19	GA	5001	LHG	O7-C7	3.88	1.45	1.34
19	BA	5004	LHG	O7-C7	3.87	1.45	1.34
14	BA	1210	CLA	C3C-C2C	3.87	1.44	1.36
14	HA	1227	CLA	C3B-C2B	3.86	1.45	1.40
14	GA	1139	CLA	C3B-C2B	3.86	1.45	1.40
14	BA	1219	CLA	C3D-C2D	3.86	1.47	1.39
18	iA	4020	BCR	C11-C12	-3.86	1.24	1.34
14	aA	1103	CLA	OBD-CAD	3.86	1.27	1.22
14	HA	1219	CLA	C3D-C2D	3.86	1.47	1.39
14	AA	1113	CLA	OBD-CAD	3.85	1.27	1.22
14	GA	1107	CLA	OBD-CAD	3.85	1.27	1.22
14	BA	1233	CLA	C3B-C2B	3.85	1.45	1.40
14	HA	1207	CLA	C1C-NC	-3.85	1.32	1.37
14	BA	1235	CLA	C3D-C2D	3.84	1.47	1.39
14	HA	1218	CLA	C3D-C2D	3.84	1.47	1.39
14	bA	1207	CLA	C1C-NC	-3.84	1.32	1.37
14	HA	1023	CLA	C3B-C2B	3.84	1.45	1.40
14	aA	1129	CLA	OBD-CAD	3.84	1.27	1.22
14	bA	1238	CLA	C3B-C2B	3.84	1.45	1.40
14	bA	1223	CLA	OBD-CAD	3.84	1.27	1.22
14	BA	1021	CLA	C3C-C2C	3.84	1.44	1.36
14	bA	1227	CLA	C3B-C2B	3.84	1.45	1.40
14	HA	1210	CLA	C3C-C2C	3.84	1.44	1.36
14	BA	1238	CLA	C3B-C2B	3.84	1.45	1.40
14	aA	1107	CLA	OBD-CAD	3.84	1.27	1.22
14	AA	1107	CLA	OBD-CAD	3.83	1.27	1.22
14	HA	1021	CLA	C3C-C2C	3.83	1.44	1.36

Continued on next page...

PRELIMINARY VALIDATION REPORT

Continued from previous page...

Mol	Chain	Res	Type	Atoms	Z	Observed(Å)	Ideal(Å)
18	RA	4020	BCR	C11-C12	-3.83	1.24	1.34
14	AA	1131	CLA	C3B-C2B	3.83	1.45	1.40
14	bA	1219	CLA	C3D-C2D	3.83	1.47	1.39
14	HA	1238	CLA	C3B-C2B	3.83	1.45	1.40
18	IA	4020	BCR	C11-C12	-3.83	1.24	1.34
14	bA	1233	CLA	C3B-C2B	3.83	1.45	1.40
14	kA	1401	CLA	C3D-C2D	3.83	1.47	1.39
14	AA	1139	CLA	C3B-C2B	3.83	1.45	1.40
14	aA	1103	CLA	C3C-C2C	3.83	1.44	1.36
14	BA	1236	CLA	C3B-C2B	3.83	1.45	1.40
14	aA	1139	CLA	C3B-C2B	3.82	1.45	1.40
14	GA	1116	CLA	C3B-C2B	3.82	1.45	1.40
14	HA	1235	CLA	C3D-C2D	3.82	1.46	1.39
14	BA	1213	CLA	C3D-C2D	3.82	1.46	1.39
14	AA	1103	CLA	C3C-C2C	3.82	1.44	1.36
14	GA	1115	CLA	C3D-C2D	3.81	1.46	1.39
14	aA	1116	CLA	C3B-C2B	3.81	1.45	1.40
14	bA	1021	CLA	C3C-C2C	3.81	1.44	1.36
14	AA	1116	CLA	C3B-C2B	3.81	1.45	1.40
14	AA	1115	CLA	C3D-C2D	3.81	1.46	1.39
14	GA	1112	CLA	C3D-C2D	3.81	1.46	1.39
14	AA	1129	CLA	OBD-CAD	3.81	1.27	1.22
14	BA	1212	CLA	C3D-C2D	3.81	1.46	1.39
14	HA	1233	CLA	C3B-C2B	3.81	1.45	1.40
14	BA	1023	CLA	C3B-C2B	3.81	1.45	1.40
14	aA	1115	CLA	C3D-C2D	3.81	1.46	1.39
14	BA	1218	CLA	C3D-C2D	3.81	1.46	1.39
14	KA	1401	CLA	C3D-C2D	3.81	1.46	1.39
14	BA	1227	CLA	C3B-C2B	3.81	1.45	1.40
14	bA	1213	CLA	C3D-C2D	3.81	1.46	1.39
14	BA	1228	CLA	C3D-C2D	3.81	1.46	1.39
14	bA	1218	CLA	C3D-C2D	3.81	1.46	1.39
14	BA	1205	CLA	C3B-C2B	3.81	1.45	1.40
14	BA	1207	CLA	C1C-NC	-3.80	1.32	1.37
14	GA	1012	CLA	C3D-C2D	3.80	1.46	1.39
14	HA	1228	CLA	C3D-C2D	3.80	1.46	1.39
14	HA	1213	CLA	C3D-C2D	3.80	1.46	1.39
14	bA	1212	CLA	C3D-C2D	3.80	1.46	1.39
14	UA	1502	CLA	OBD-CAD	3.79	1.27	1.22
14	GA	1103	CLA	C3C-C2C	3.79	1.44	1.36
14	HA	1236	CLA	C3B-C2B	3.79	1.45	1.40
14	HA	1210	CLA	C1C-NC	-3.79	1.32	1.37

Continued on next page...

PRELIMINARY VALIDATION REPORT

Continued from previous page...

Mol	Chain	Res	Type	Atoms	Z	Observed(Å)	Ideal(Å)
14	LA	1502	CLA	OBD-CAD	3.79	1.27	1.22
14	aA	1102	CLA	C3D-C2D	3.79	1.46	1.39
14	AA	1112	CLA	C3D-C2D	3.79	1.46	1.39
14	bA	1023	CLA	C3B-C2B	3.79	1.45	1.40
14	GA	1131	CLA	C3B-C2B	3.79	1.45	1.40
14	bA	1228	CLA	C3D-C2D	3.79	1.46	1.39
14	bA	1205	CLA	C3B-C2B	3.79	1.45	1.40
14	TA	1401	CLA	C3D-C2D	3.78	1.46	1.39
14	HA	1223	CLA	OBD-CAD	3.78	1.27	1.22
18	LA	4022	BCR	C11-C12	-3.78	1.24	1.34
14	HA	1233	CLA	C3D-C2D	3.78	1.46	1.39
14	aA	1012	CLA	C3D-C2D	3.78	1.46	1.39
14	aA	1131	CLA	C3B-C2B	3.78	1.45	1.40
14	aA	1112	CLA	C3D-C2D	3.78	1.46	1.39
14	bA	1233	CLA	C3D-C2D	3.77	1.46	1.39
18	lA	4022	BCR	C11-C12	-3.77	1.24	1.34
14	bA	1235	CLA	C3D-C2D	3.77	1.46	1.39
14	aA	1123	CLA	OBD-CAD	3.77	1.27	1.22
14	bA	1210	CLA	C1C-NC	-3.77	1.32	1.37
14	BA	1223	CLA	OBD-CAD	3.77	1.27	1.22
14	HA	1205	CLA	C3B-C2B	3.77	1.45	1.40
14	AA	1012	CLA	C3D-C2D	3.76	1.46	1.39
14	GA	1108	CLA	C3B-C2B	3.76	1.45	1.40
14	bA	1236	CLA	C3B-C2B	3.76	1.45	1.40
20	BA	5002	LMG	O7-C10	3.76	1.45	1.34
20	HA	5002	LMG	O7-C10	3.76	1.45	1.34
18	AA	4007	BCR	C11-C12	-3.75	1.25	1.34
14	BA	1209	CLA	C3D-C2D	3.75	1.46	1.39
14	AA	1123	CLA	OBD-CAD	3.75	1.27	1.22
14	BA	1210	CLA	C1C-NC	-3.75	1.32	1.37
14	BA	1023	CLA	C3D-C2D	3.75	1.46	1.39
14	HA	1212	CLA	C3D-C2D	3.75	1.46	1.39
14	lA	1502	CLA	OBD-CAD	3.74	1.27	1.22
14	GA	1102	CLA	C3D-C2D	3.74	1.46	1.39
18	aA	4001	BCR	C11-C12	-3.74	1.25	1.34
14	HA	1023	CLA	C3D-C2D	3.74	1.46	1.39
14	HA	1209	CLA	C3D-C2D	3.74	1.46	1.39
14	bA	1023	CLA	C3D-C2D	3.74	1.46	1.39
14	bA	1209	CLA	C3D-C2D	3.74	1.46	1.39
14	aA	1138	CLA	C3D-C2D	3.74	1.46	1.39
18	GA	4007	BCR	C11-C12	-3.73	1.25	1.34
14	HA	1232	CLA	C3D-C2D	3.73	1.46	1.39

Continued on next page...

PRELIMINARY VALIDATION REPORT

Continued from previous page...

Mol	Chain	Res	Type	Atoms	Z	Observed(Å)	Ideal(Å)
14	AA	1102	CLA	C3D-C2D	3.73	1.46	1.39
18	BA	4017	BCR	C11-C12	-3.73	1.25	1.34
14	lA	1502	CLA	C3D-C2D	3.73	1.46	1.39
18	bA	4017	BCR	C11-C12	-3.73	1.25	1.34
18	UA	4022	BCR	C11-C12	-3.73	1.25	1.34
14	aA	1108	CLA	C3B-C2B	3.73	1.45	1.40
14	BA	1233	CLA	C3D-C2D	3.72	1.46	1.39
14	GA	1123	CLA	OBD-CAD	3.72	1.27	1.22
14	HA	1215	CLA	C3D-C2D	3.72	1.46	1.39
14	AA	1128	CLA	C3B-C2B	3.72	1.45	1.40
14	UA	1502	CLA	C3D-C2D	3.72	1.46	1.39
18	GA	4001	BCR	C11-C12	-3.72	1.25	1.34
18	AA	4001	BCR	C11-C12	-3.72	1.25	1.34
18	aA	4007	BCR	C11-C12	-3.71	1.25	1.34
14	HA	1208	CLA	C3D-C2D	3.71	1.46	1.39
20	bA	5002	LMG	O7-C10	3.71	1.44	1.34
14	0	1012	CLA	C3D-C2D	3.71	1.46	1.39
18	HA	4017	BCR	C11-C12	-3.71	1.25	1.34
14	aA	1132	CLA	C3D-C2D	3.71	1.46	1.39
14	BA	1022	CLA	C3D-C2D	3.71	1.46	1.39
14	AA	1137	CLA	OBD-CAD	3.71	1.27	1.22
14	aA	1137	CLA	OBD-CAD	3.71	1.27	1.22
14	AA	1138	CLA	C3D-C2D	3.71	1.46	1.39
14	bA	1215	CLA	C3D-C2D	3.70	1.46	1.39
14	BA	1215	CLA	C3D-C2D	3.70	1.46	1.39
14	BA	1208	CLA	C3D-C2D	3.70	1.46	1.39
14	bA	1208	CLA	C3D-C2D	3.70	1.46	1.39
14	aA	1128	CLA	C3B-C2B	3.70	1.45	1.40
14	bA	1022	CLA	C3D-C2D	3.70	1.46	1.39
14	AA	1108	CLA	C3D-C2D	3.70	1.46	1.39
14	bA	1206	CLA	C3B-C2B	3.70	1.45	1.40
14	AA	1108	CLA	C3B-C2B	3.69	1.45	1.40
14	LA	1502	CLA	C3D-C2D	3.69	1.46	1.39
14	BA	1232	CLA	C3D-C2D	3.69	1.46	1.39
18	aA	4011	BCR	C11-C12	-3.69	1.25	1.34
14	BA	1234	CLA	OBD-CAD	3.68	1.27	1.22
14	aA	1113	CLA	C3D-C2D	3.68	1.46	1.39
14	GA	1128	CLA	C3B-C2B	3.68	1.45	1.40
14	GA	1138	CLA	C3D-C2D	3.68	1.46	1.39
14	GA	1109	CLA	C3D-C2D	3.67	1.46	1.39
14	aA	1108	CLA	C3D-C2D	3.67	1.46	1.39
14	aA	1109	CLA	C3D-C2D	3.67	1.46	1.39

Continued on next page...

PRELIMINARY VALIDATION REPORT

Continued from previous page...

Mol	Chain	Res	Type	Atoms	Z	Observed(Å)	Ideal(Å)
14	GA	1137	CLA	OBD-CAD	3.67	1.27	1.22
14	GA	1140	CLA	C3D-C2D	3.67	1.46	1.39
14	bA	1232	CLA	C3D-C2D	3.67	1.46	1.39
14	AA	1140	CLA	C3D-C2D	3.67	1.46	1.39
14	GA	1132	CLA	C3D-C2D	3.67	1.46	1.39
18	HA	4014	BCR	C11-C12	-3.67	1.25	1.34
14	lA	1501	CLA	C3D-C2D	3.66	1.46	1.39
14	aA	1140	CLA	C3D-C2D	3.66	1.46	1.39
14	AA	1113	CLA	C3D-C2D	3.66	1.46	1.39
14	AA	1109	CLA	C3D-C2D	3.66	1.46	1.39
14	AA	1132	CLA	C3D-C2D	3.65	1.46	1.39
14	aA	1105	CLA	C3D-C2D	3.65	1.46	1.39
18	BA	4014	BCR	C11-C12	-3.65	1.25	1.34
14	GA	1113	CLA	C3D-C2D	3.65	1.46	1.39
14	BA	1206	CLA	C3B-C2B	3.65	1.45	1.40
18	GA	4011	BCR	C11-C12	-3.65	1.25	1.34
18	bA	4014	BCR	C11-C12	-3.65	1.25	1.34
14	LA	1501	CLA	C3D-C2D	3.64	1.46	1.39
14	GA	1108	CLA	C3D-C2D	3.64	1.46	1.39
18	HA	4010	BCR	C11-C12	-3.64	1.25	1.34
14	UA	1501	CLA	C3D-C2D	3.63	1.46	1.39
18	AA	4011	BCR	C11-C12	-3.63	1.25	1.34
18	bA	4010	BCR	C11-C12	-3.63	1.25	1.34
14	AA	1105	CLA	C3D-C2D	3.63	1.46	1.39
14	bA	1221	CLA	C3D-C2D	3.63	1.46	1.39
14	GA	1136	CLA	C3B-C2B	3.62	1.45	1.40
14	BA	1221	CLA	C3D-C2D	3.62	1.46	1.39
14	AA	1136	CLA	C3B-C2B	3.62	1.45	1.40
18	BA	4010	BCR	C11-C12	-3.62	1.25	1.34
14	aA	1131	CLA	C3D-C2D	3.62	1.46	1.39
14	AA	1131	CLA	C3D-C2D	3.62	1.46	1.39
14	aA	1126	CLA	C3D-C2D	3.61	1.46	1.39
14	GA	1126	CLA	C3D-C2D	3.61	1.46	1.39
14	UA	1503	CLA	C3D-C2D	3.61	1.46	1.39
14	HA	1206	CLA	C3B-C2B	3.61	1.45	1.40
14	GA	1131	CLA	C3D-C2D	3.61	1.46	1.39
14	GA	1118	CLA	C3D-C2D	3.61	1.46	1.39
14	AA	1126	CLA	C3D-C2D	3.60	1.46	1.39
14	aA	1118	CLA	C3D-C2D	3.60	1.46	1.39
14	AA	1118	CLA	C3D-C2D	3.60	1.46	1.39
14	aA	1106	CLA	C3D-C2D	3.60	1.46	1.39
14	GA	1139	CLA	C3D-C2D	3.60	1.46	1.39

Continued on next page...

PRELIMINARY VALIDATION REPORT

Continued from previous page...

Mol	Chain	Res	Type	Atoms	Z	Observed(Å)	Ideal(Å)
14	AA	1106	CLA	C3D-C2D	3.60	1.46	1.39
14	GA	1105	CLA	C3D-C2D	3.60	1.46	1.39
16	HA	2002	PQN	C10-C5	3.60	1.46	1.40
16	BA	2002	PQN	C10-C5	3.59	1.46	1.40
14	GA	1106	CLA	C3D-C2D	3.59	1.46	1.39
14	aA	1139	CLA	C3D-C2D	3.59	1.46	1.39
16	bA	2002	PQN	C10-C5	3.59	1.46	1.40
14	aA	1136	CLA	C3B-C2B	3.59	1.45	1.40
14	GA	1122	CLA	C3D-C2D	3.59	1.46	1.39
18	bA	4009	BCR	C11-C12	-3.59	1.25	1.34
14	GA	1101	CLA	C3D-C2D	3.58	1.46	1.39
14	LA	1503	CLA	C3D-C2D	3.58	1.46	1.39
14	lA	1503	CLA	C3D-C2D	3.58	1.46	1.39
14	HA	1221	CLA	C3D-C2D	3.58	1.46	1.39
14	BA	1222	CLA	C3D-C2D	3.58	1.46	1.39
14	bA	1229	CLA	C3D-C2D	3.58	1.46	1.39
14	bA	1239	CLA	C1C-NC	-3.57	1.32	1.37
14	BA	1229	CLA	C3D-C2D	3.57	1.46	1.39
14	bA	1234	CLA	OBD-CAD	3.57	1.27	1.22
18	BA	4016	BCR	C11-C12	-3.57	1.25	1.34
18	HA	4009	BCR	C11-C12	-3.57	1.25	1.34
14	HA	1224	CLA	C3D-C2D	3.56	1.46	1.39
14	HA	1234	CLA	OBD-CAD	3.56	1.27	1.22
14	AA	1122	CLA	C3D-C2D	3.56	1.46	1.39
14	aA	1122	CLA	C3D-C2D	3.56	1.46	1.39
14	bA	1222	CLA	C3D-C2D	3.56	1.46	1.39
18	BA	4009	BCR	C11-C12	-3.56	1.25	1.34
14	HA	1229	CLA	C3D-C2D	3.56	1.46	1.39
14	aA	1101	CLA	C3D-C2D	3.56	1.46	1.39
18	VA	4021	BCR	C11-C12	-3.55	1.25	1.34
14	BA	1239	CLA	C1C-NC	-3.55	1.32	1.37
18	mA	4021	BCR	C11-C12	-3.55	1.25	1.34
14	AA	1103	CLA	C3D-C2D	3.55	1.46	1.39
14	BA	1201	CLA	C3D-C2D	3.55	1.46	1.39
14	BA	1225	CLA	C1C-NC	-3.55	1.32	1.37
14	bA	1201	CLA	C3D-C2D	3.55	1.46	1.39
14	HA	1239	CLA	C1C-NC	-3.54	1.32	1.37
14	HA	1205	CLA	C3D-C2D	3.54	1.46	1.39
14	bA	1205	CLA	C3D-C2D	3.54	1.46	1.39
18	HA	4016	BCR	C11-C12	-3.54	1.25	1.34
14	AA	1101	CLA	C3D-C2D	3.54	1.46	1.39
18	bA	4016	BCR	C11-C12	-3.54	1.25	1.34

Continued on next page...

PRELIMINARY VALIDATION REPORT

Continued from previous page...

Mol	Chain	Res	Type	Atoms	Z	Observed(Å)	Ideal(Å)
14	AA	1139	CLA	C3D-C2D	3.54	1.46	1.39
14	HA	1201	CLA	C3D-C2D	3.54	1.46	1.39
14	HA	1210	CLA	C3D-C2D	3.54	1.46	1.39
14	BA	1227	CLA	C3D-C2D	3.54	1.46	1.39
14	UA	1502	CLA	C3B-C2B	3.53	1.45	1.40
14	bA	1216	CLA	C3B-C2B	3.53	1.45	1.40
14	GA	1122	CLA	C1C-NC	-3.53	1.32	1.37
14	BA	1210	CLA	C3D-C2D	3.53	1.46	1.39
14	BA	1236	CLA	C3D-C2D	3.53	1.46	1.39
18	MA	4021	BCR	C11-C12	-3.53	1.25	1.34
14	BA	1205	CLA	C3D-C2D	3.53	1.46	1.39
14	HA	1222	CLA	C3D-C2D	3.53	1.46	1.39
14	bA	1224	CLA	C3D-C2D	3.52	1.46	1.39
14	BA	1224	CLA	C3D-C2D	3.52	1.46	1.39
14	bA	1225	CLA	C1C-NC	-3.52	1.32	1.37
14	AA	1133	CLA	C3B-C2B	3.52	1.45	1.40
13	aA	1011	CL0	C3D-C2D	3.51	1.46	1.39
14	bA	1236	CLA	C3D-C2D	3.51	1.46	1.39
14	GA	1103	CLA	C3D-C2D	3.51	1.46	1.39
14	bA	1227	CLA	C3D-C2D	3.51	1.46	1.39
14	AA	1107	CLA	C3B-C2B	3.51	1.45	1.40
14	bA	1210	CLA	C3D-C2D	3.51	1.46	1.39
13	AA	1011	CL0	C3D-C2D	3.51	1.46	1.39
14	HA	1227	CLA	C3D-C2D	3.51	1.46	1.39
14	lA	1502	CLA	C3B-C2B	3.50	1.45	1.40
14	HA	1216	CLA	C3B-C2B	3.50	1.45	1.40
14	BA	1206	CLA	OBD-CAD	3.50	1.27	1.22
14	HA	1211	CLA	C3D-C2D	3.50	1.46	1.39
14	HA	1225	CLA	C1C-NC	-3.50	1.32	1.37
14	aA	1133	CLA	C3B-C2B	3.50	1.45	1.40
14	bA	1226	CLA	C3B-C2B	3.50	1.45	1.40
14	HA	1226	CLA	C3B-C2B	3.50	1.45	1.40
14	HA	1236	CLA	C3D-C2D	3.50	1.46	1.39
14	aA	1103	CLA	C3D-C2D	3.50	1.46	1.39
14	XA	1701	CLA	C3D-C2D	3.49	1.46	1.39
13	GA	1011	CL0	C1C-NC	-3.49	1.32	1.37
14	xA	1701	CLA	C3D-C2D	3.49	1.46	1.39
14	bA	1206	CLA	OBD-CAD	3.49	1.27	1.22
14	aA	1133	CLA	C3D-C2D	3.49	1.46	1.39
14	BA	1226	CLA	C3B-C2B	3.49	1.45	1.40
14	bA	1206	CLA	C1C-NC	-3.49	1.32	1.37
14	BA	1211	CLA	C3D-C2D	3.49	1.46	1.39

Continued on next page...

PRELIMINARY VALIDATION REPORT

Continued from previous page...

Mol	Chain	Res	Type	Atoms	Z	Observed(Å)	Ideal(Å)
18	LA	4019	BCR	C11-C12	-3.49	1.25	1.34
13	GA	1011	CL0	C3D-C2D	3.49	1.46	1.39
14	GA	1107	CLA	C3B-C2B	3.48	1.45	1.40
14	GA	1133	CLA	C3B-C2B	3.48	1.45	1.40
14	aA	1122	CLA	C1C-NC	-3.48	1.32	1.37
14	HA	1206	CLA	C1C-NC	-3.48	1.32	1.37
14	GA	1133	CLA	C3D-C2D	3.48	1.46	1.39
14	LA	1502	CLA	C3B-C2B	3.48	1.45	1.40
14	aA	1107	CLA	C3B-C2B	3.48	1.45	1.40
14	WA	1701	CLA	C3D-C2D	3.47	1.46	1.39
14	BA	1204	CLA	C3D-C2D	3.47	1.46	1.39
14	aA	1136	CLA	C3D-C2D	3.47	1.46	1.39
14	BA	1216	CLA	C3B-C2B	3.47	1.45	1.40
14	aA	1125	CLA	C3D-C2D	3.47	1.46	1.39
13	aA	1011	CL0	C1C-NC	-3.47	1.32	1.37
14	AA	1136	CLA	C3D-C2D	3.47	1.46	1.39
14	aA	1128	CLA	C1C-NC	-3.47	1.32	1.37
14	GA	1128	CLA	C1C-NC	-3.47	1.32	1.37
14	aA	1123	CLA	C3D-C2D	3.47	1.46	1.39
18	aA	4003	BCR	C11-C12	-3.47	1.25	1.34
14	BA	1217	CLA	C3D-C2D	3.47	1.46	1.39
14	HA	1204	CLA	C3D-C2D	3.47	1.46	1.39
14	AA	1122	CLA	C1C-NC	-3.47	1.32	1.37
14	bA	1211	CLA	C3D-C2D	3.46	1.46	1.39
14	bA	1226	CLA	C1C-NC	-3.46	1.32	1.37
14	AA	1123	CLA	C3D-C2D	3.46	1.46	1.39
14	HA	1217	CLA	C3D-C2D	3.46	1.46	1.39
18	GA	4003	BCR	C11-C12	-3.46	1.25	1.34
14	aA	1130	CLA	C3D-C2D	3.46	1.46	1.39
14	GA	1124	CLA	C3D-C2D	3.45	1.46	1.39
18	UA	4019	BCR	C11-C12	-3.45	1.25	1.34
18	bA	4005	BCR	C11-C12	-3.45	1.25	1.34
14	HA	1206	CLA	OBD-CAD	3.45	1.27	1.22
14	GA	1130	CLA	C3D-C2D	3.45	1.46	1.39
14	AA	1130	CLA	C3D-C2D	3.45	1.46	1.39
18	bA	4013	BCR	C11-C12	-3.45	1.25	1.34
14	bA	1217	CLA	C3D-C2D	3.45	1.46	1.39
14	GA	1136	CLA	C3D-C2D	3.45	1.46	1.39
18	lA	4019	BCR	C11-C12	-3.45	1.25	1.34
14	AA	1133	CLA	C3D-C2D	3.45	1.46	1.39
13	AA	1011	CL0	C1C-NC	-3.44	1.32	1.37
14	bA	1204	CLA	C3D-C2D	3.44	1.46	1.39

Continued on next page...

PRELIMINARY VALIDATION REPORT

Continued from previous page...

Mol	Chain	Res	Type	Atoms	Z	Observed(Å)	Ideal(Å)
14	GA	1123	CLA	C3D-C2D	3.44	1.46	1.39
14	AA	1124	CLA	C3D-C2D	3.44	1.46	1.39
18	BA	4005	BCR	C11-C12	-3.44	1.25	1.34
14	AA	1125	CLA	C3D-C2D	3.44	1.46	1.39
14	BA	1234	CLA	C3B-C2B	3.43	1.45	1.40
14	HA	1234	CLA	C3B-C2B	3.43	1.45	1.40
14	BA	1202	CLA	C3D-C2D	3.43	1.46	1.39
18	AA	4003	BCR	C11-C12	-3.43	1.25	1.34
18	aA	4002	BCR	C11-C12	-3.43	1.25	1.34
14	BA	1206	CLA	C1C-NC	-3.43	1.32	1.37
14	bA	1202	CLA	C3D-C2D	3.43	1.46	1.39
14	BA	1239	CLA	C3D-C2D	3.43	1.46	1.39
18	bA	4004	BCR	C11-C12	-3.43	1.25	1.34
14	AA	1128	CLA	C1C-NC	-3.43	1.32	1.37
14	aA	1111	CLA	C3D-C2D	3.43	1.46	1.39
18	GA	4002	BCR	C11-C12	-3.43	1.25	1.34
14	GA	1137	CLA	C3D-C2D	3.43	1.46	1.39
14	bA	1205	CLA	C1C-NC	-3.43	1.32	1.37
14	GA	1111	CLA	C3D-C2D	3.43	1.46	1.39
14	HA	1226	CLA	C1C-NC	-3.42	1.32	1.37
18	HA	4013	BCR	C11-C12	-3.42	1.25	1.34
14	AA	1111	CLA	C3D-C2D	3.42	1.46	1.39
18	BA	4013	BCR	C11-C12	-3.42	1.25	1.34
14	BA	1231	CLA	C3D-C2D	3.42	1.46	1.39
14	aA	1137	CLA	C3D-C2D	3.42	1.46	1.39
14	bA	1234	CLA	C1C-NC	-3.42	1.32	1.37
14	HA	1202	CLA	C3D-C2D	3.42	1.46	1.39
18	BA	4004	BCR	C11-C12	-3.41	1.25	1.34
14	BA	1226	CLA	C1C-NC	-3.41	1.32	1.37
14	aA	1124	CLA	C3D-C2D	3.41	1.46	1.39
14	bA	1223	CLA	C3D-C2D	3.41	1.46	1.39
18	HA	4004	BCR	C11-C12	-3.41	1.25	1.34
14	AA	1135	CLA	C3D-C2D	3.41	1.46	1.39
14	HA	1231	CLA	C3D-C2D	3.41	1.46	1.39
14	HA	1208	CLA	C3B-C2B	3.41	1.45	1.40
14	bA	1234	CLA	C3B-C2B	3.40	1.45	1.40
14	BA	1234	CLA	C1C-NC	-3.40	1.32	1.37
14	aA	1135	CLA	C3D-C2D	3.40	1.46	1.39
14	GA	1125	CLA	C3D-C2D	3.40	1.46	1.39
14	AA	1110	CLA	C3D-C2D	3.40	1.46	1.39
14	GA	1135	CLA	C3D-C2D	3.40	1.46	1.39
18	HA	4005	BCR	C11-C12	-3.40	1.25	1.34

Continued on next page...

PRELIMINARY VALIDATION REPORT

Continued from previous page...

Mol	Chain	Res	Type	Atoms	Z	Observed(Å)	Ideal(Å)
14	bA	1239	CLA	C3D-C2D	3.40	1.46	1.39
18	AA	4002	BCR	C11-C12	-3.40	1.25	1.34
14	bA	1231	CLA	C3D-C2D	3.40	1.46	1.39
14	BA	1205	CLA	C1C-NC	-3.39	1.32	1.37
14	bA	1208	CLA	C3B-C2B	3.39	1.45	1.40
14	HA	1239	CLA	C3D-C2D	3.38	1.46	1.39
14	BA	1021	CLA	C3D-C2D	3.38	1.46	1.39
14	aA	1110	CLA	C3D-C2D	3.38	1.46	1.39
14	HA	1205	CLA	C1C-NC	-3.38	1.32	1.37
14	HA	1223	CLA	C3D-C2D	3.38	1.46	1.39
14	AA	1137	CLA	C3D-C2D	3.38	1.46	1.39
14	HA	1021	CLA	C3D-C2D	3.38	1.46	1.39
14	GA	1111	CLA	C1C-NC	-3.38	1.32	1.37
14	lA	1503	CLA	C1C-NC	-3.38	1.32	1.37
14	bA	1021	CLA	C3D-C2D	3.37	1.46	1.39
14	aA	1127	CLA	C3D-C2D	3.37	1.46	1.39
14	bA	1216	CLA	C1C-NC	-3.37	1.32	1.37
14	BA	1223	CLA	C3D-C2D	3.37	1.46	1.39
14	GA	1110	CLA	C3D-C2D	3.37	1.46	1.39
14	AA	1107	CLA	C3D-C2D	3.36	1.46	1.39
14	HA	1234	CLA	C1C-NC	-3.36	1.32	1.37
14	BA	1208	CLA	C3B-C2B	3.35	1.45	1.40
14	GA	1125	CLA	OBD-CAD	3.35	1.27	1.22
14	AA	1125	CLA	OBD-CAD	3.35	1.27	1.22
14	aA	1107	CLA	C3D-C2D	3.35	1.46	1.39
14	HA	1216	CLA	C1C-NC	-3.35	1.32	1.37
14	aA	1125	CLA	OBD-CAD	3.35	1.27	1.22
14	LA	1503	CLA	C1C-NC	-3.35	1.32	1.37
14	GA	1127	CLA	C3D-C2D	3.35	1.46	1.39
14	GA	1107	CLA	C3D-C2D	3.35	1.46	1.39
14	GA	1116	CLA	C3D-C2D	3.34	1.46	1.39
14	GA	1013	CLA	C1C-NC	-3.34	1.32	1.37
14	AA	1127	CLA	C3D-C2D	3.34	1.46	1.39
14	LA	1502	CLA	C1C-NC	-3.33	1.32	1.37
14	aA	1111	CLA	C1C-NC	-3.33	1.32	1.37
14	BA	1202	CLA	C3B-C2B	3.33	1.45	1.40
14	aA	1140	CLA	C1C-NC	-3.33	1.32	1.37
14	BA	1021	CLA	C1C-NC	-3.33	1.32	1.37
14	UA	1503	CLA	C1C-NC	-3.33	1.32	1.37
14	AA	1116	CLA	C3D-C2D	3.33	1.46	1.39
14	aA	1116	CLA	C3D-C2D	3.33	1.46	1.39
14	BA	1216	CLA	C1C-NC	-3.32	1.32	1.37

Continued on next page...

PRELIMINARY VALIDATION REPORT

Continued from previous page...

Mol	Chain	Res	Type	Atoms	Z	Observed(Å)	Ideal(Å)
14	AA	1140	CLA	C1C-NC	-3.32	1.32	1.37
14	AA	1103	CLA	C1C-NC	-3.32	1.32	1.37
14	bA	1202	CLA	C3B-C2B	3.32	1.45	1.40
14	bA	1239	CLA	C3B-C2B	3.32	1.45	1.40
14	HA	1202	CLA	C3B-C2B	3.32	1.45	1.40
20	lA	5007	LMG	C19-C18	-3.31	1.32	1.51
14	AA	1013	CLA	C1C-NC	-3.31	1.32	1.37
20	UA	5007	LMG	C19-C18	-3.31	1.32	1.51
20	LA	5007	LMG	C19-C18	-3.31	1.32	1.51
14	HA	1021	CLA	C1C-NC	-3.30	1.32	1.37
14	aA	1104	CLA	C3D-C2D	3.30	1.45	1.39
14	AA	1104	CLA	C3D-C2D	3.30	1.45	1.39
18	bA	4006	BCR	C11-C12	-3.30	1.26	1.34
14	aA	1103	CLA	C1C-NC	-3.30	1.32	1.37
14	aA	1013	CLA	C1C-NC	-3.30	1.32	1.37
14	AA	1126	CLA	C1C-NC	-3.30	1.32	1.37
18	HA	4006	BCR	C11-C12	-3.29	1.26	1.34
14	TA	1401	CLA	C1D-C2D	3.29	1.50	1.42
14	UA	1502	CLA	C1C-NC	-3.29	1.32	1.37
14	lA	1502	CLA	C1C-NC	-3.29	1.32	1.37
18	BA	4006	BCR	C11-C12	-3.29	1.26	1.34
14	GA	1104	CLA	C3D-C2D	3.29	1.45	1.39
14	KA	1401	CLA	C1D-C2D	3.29	1.50	1.42
14	HA	1239	CLA	C3B-C2B	3.28	1.44	1.40
14	aA	1126	CLA	C1C-NC	-3.28	1.32	1.37
14	GA	1140	CLA	C1C-NC	-3.28	1.32	1.37
14	bA	1021	CLA	C1C-NC	-3.27	1.32	1.37
14	AA	1111	CLA	C1C-NC	-3.27	1.32	1.37
20	UA	5007	LMG	C40-C39	-3.27	1.33	1.51
14	GA	1103	CLA	C1C-NC	-3.27	1.32	1.37
20	LA	5007	LMG	C40-C39	-3.27	1.33	1.51
14	HA	1214	CLA	C3B-C2B	3.26	1.44	1.40
14	BA	1239	CLA	C3B-C2B	3.26	1.44	1.40
20	lA	5007	LMG	C40-C39	-3.26	1.33	1.51
14	BA	1236	CLA	C1C-NC	-3.26	1.32	1.37
14	kA	1401	CLA	C1D-C2D	3.26	1.49	1.42
20	HA	5002	LMG	C22-C21	-3.25	1.33	1.51
14	GA	1126	CLA	C1C-NC	-3.25	1.32	1.37
14	HA	1236	CLA	C1C-NC	-3.25	1.33	1.37
14	bA	1214	CLA	C3B-C2B	3.25	1.44	1.40
14	BA	1228	CLA	C1C-NC	-3.25	1.33	1.37
14	bA	1236	CLA	C1C-NC	-3.25	1.33	1.37

Continued on next page...

PRELIMINARY VALIDATION REPORT

Continued from previous page...

Mol	Chain	Res	Type	Atoms	Z	Observed(Å)	Ideal(Å)
20	HA	5002	LMG	C37-C36	-3.25	1.33	1.51
20	BA	5002	LMG	C22-C21	-3.25	1.33	1.51
14	aA	1131	CLA	C1C-NC	-3.25	1.33	1.37
20	bA	5002	LMG	C22-C21	-3.25	1.33	1.51
14	GA	1132	CLA	C1C-NC	-3.25	1.33	1.37
14	AA	1132	CLA	C1C-NC	-3.24	1.33	1.37
20	BA	5002	LMG	C37-C36	-3.24	1.33	1.51
14	HA	1201	CLA	C1C-NC	-3.24	1.33	1.37
14	BA	1201	CLA	C1C-NC	-3.24	1.33	1.37
14	AA	1131	CLA	C1C-NC	-3.24	1.33	1.37
14	bA	1201	CLA	C1C-NC	-3.24	1.33	1.37
14	aA	1113	CLA	C1C-NC	-3.24	1.33	1.37
20	bA	5002	LMG	C37-C36	-3.24	1.33	1.51
14	AA	1113	CLA	C1C-NC	-3.23	1.33	1.37
14	HA	1203	CLA	C1C-NC	-3.22	1.33	1.37
14	aA	1132	CLA	C1C-NC	-3.22	1.33	1.37
14	aA	1139	CLA	C1C-NC	-3.22	1.33	1.37
14	bA	1204	CLA	C3B-C2B	3.22	1.44	1.40
14	BA	1203	CLA	C1C-NC	-3.22	1.33	1.37
20	aA	5005	LMG	C40-C39	-3.22	1.33	1.51
20	GA	5005	LMG	C40-C39	-3.22	1.33	1.51
14	BA	1214	CLA	C3B-C2B	3.22	1.44	1.40
20	aA	5005	LMG	C43-C42	-3.21	1.33	1.51
14	bA	1202	CLA	C1C-NC	-3.21	1.33	1.37
14	aA	1114	CLA	C1D-C2D	3.21	1.49	1.42
14	BA	1225	CLA	C3D-C2D	3.21	1.45	1.39
14	BA	1223	CLA	C1C-NC	-3.20	1.33	1.37
20	HA	5002	LMG	C19-C18	-3.20	1.33	1.51
14	aA	1130	CLA	C1C-NC	-3.20	1.33	1.37
14	bA	1203	CLA	C1C-NC	-3.20	1.33	1.37
14	HA	1228	CLA	C1C-NC	-3.20	1.33	1.37
20	AA	5005	LMG	C43-C42	-3.20	1.33	1.51
20	AA	5005	LMG	C40-C39	-3.20	1.33	1.51
20	bA	5002	LMG	C19-C18	-3.20	1.33	1.51
14	bA	1203	CLA	OBD-CAD	3.20	1.26	1.22
14	AA	1106	CLA	C1C-NC	-3.19	1.33	1.37
14	GA	1106	CLA	C1C-NC	-3.19	1.33	1.37
14	AA	1114	CLA	C1D-C2D	3.19	1.49	1.42
14	aA	1106	CLA	C1C-NC	-3.19	1.33	1.37
20	BA	5002	LMG	C19-C18	-3.19	1.33	1.51
14	BA	1202	CLA	C1C-NC	-3.19	1.33	1.37
14	BA	1204	CLA	C3B-C2B	3.19	1.44	1.40

Continued on next page...

PRELIMINARY VALIDATION REPORT

Continued from previous page...

Mol	Chain	Res	Type	Atoms	Z	Observed(Å)	Ideal(Å)
14	HA	1225	CLA	C3D-C2D	3.18	1.45	1.39
14	HA	1203	CLA	OBD-CAD	3.18	1.26	1.22
14	bA	1225	CLA	C3D-C2D	3.18	1.45	1.39
20	GA	5005	LMG	C43-C42	-3.18	1.33	1.51
14	GA	1113	CLA	C1C-NC	-3.18	1.33	1.37
14	GA	1139	CLA	C1C-NC	-3.18	1.33	1.37
14	bA	1238	CLA	C3D-C2D	3.18	1.45	1.39
20	lA	5007	LMG	C37-C36	-3.18	1.33	1.51
14	AA	1136	CLA	C1C-NC	-3.18	1.33	1.37
14	GA	1131	CLA	C1C-NC	-3.17	1.33	1.37
14	HA	1238	CLA	C3D-C2D	3.17	1.45	1.39
14	bA	1228	CLA	C1C-NC	-3.17	1.33	1.37
14	BA	1203	CLA	OBD-CAD	3.17	1.26	1.22
14	bA	1223	CLA	C1C-NC	-3.17	1.33	1.37
14	GA	1114	CLA	C1D-C2D	3.16	1.49	1.42
20	LA	5007	LMG	C37-C36	-3.16	1.33	1.51
14	aA	1136	CLA	C1C-NC	-3.16	1.33	1.37
14	GA	1136	CLA	C1C-NC	-3.16	1.33	1.37
20	UA	5007	LMG	C37-C36	-3.16	1.33	1.51
14	BA	1238	CLA	C3D-C2D	3.16	1.45	1.39
14	AA	1130	CLA	C1C-NC	-3.15	1.33	1.37
20	aA	5005	LMG	C37-C36	-3.15	1.33	1.51
14	bA	1220	CLA	C1C-NC	-3.15	1.33	1.37
14	bA	1233	CLA	C1D-C2D	3.15	1.49	1.42
14	HA	1223	CLA	C1C-NC	-3.15	1.33	1.37
14	aA	1124	CLA	C1C-NC	-3.14	1.33	1.37
14	HA	1202	CLA	C1C-NC	-3.14	1.33	1.37
14	GA	1127	CLA	C1C-NC	-3.14	1.33	1.37
20	GA	5005	LMG	C37-C36	-3.14	1.33	1.51
14	aA	1127	CLA	C1C-NC	-3.14	1.33	1.37
14	AA	1124	CLA	C1C-NC	-3.14	1.33	1.37
14	AA	1127	CLA	C1C-NC	-3.14	1.33	1.37
20	AA	5005	LMG	C37-C36	-3.13	1.33	1.51
14	GA	1124	CLA	C1C-NC	-3.13	1.33	1.37
14	bA	1215	CLA	C1C-NC	-3.13	1.33	1.37
14	LA	1501	CLA	C1C-NC	-3.13	1.33	1.37
14	HA	1204	CLA	C3B-C2B	3.13	1.44	1.40
14	HA	1222	CLA	C1C-NC	-3.13	1.33	1.37
14	HA	1233	CLA	C1D-C2D	3.13	1.49	1.42
14	aA	1129	CLA	C3D-C2D	3.13	1.45	1.39
14	BA	1234	CLA	C3D-C2D	3.12	1.45	1.39
14	AA	1129	CLA	C3D-C2D	3.12	1.45	1.39

Continued on next page...

PRELIMINARY VALIDATION REPORT

Continued from previous page...

Mol	Chain	Res	Type	Atoms	Z	Observed(Å)	Ideal(Å)
14	UA	1501	CLA	C1C-NC	-3.12	1.33	1.37
14	AA	1139	CLA	C1C-NC	-3.12	1.33	1.37
14	bA	1208	CLA	C1C-NC	-3.12	1.33	1.37
14	GA	1129	CLA	C3D-C2D	3.12	1.45	1.39
14	bA	1216	CLA	C1D-C2D	3.12	1.49	1.42
18	HA	4012	BCR	C11-C12	-3.12	1.26	1.34
18	bA	4012	BCR	C11-C12	-3.12	1.26	1.34
14	GA	1130	CLA	C1C-NC	-3.12	1.33	1.37
14	BA	1220	CLA	C1C-NC	-3.11	1.33	1.37
14	GA	1117	CLA	C1C-NC	-3.11	1.33	1.37
14	lA	1501	CLA	C1C-NC	-3.11	1.33	1.37
14	bA	1213	CLA	C1D-C2D	3.11	1.49	1.42
14	bA	1208	CLA	C1D-C2D	3.11	1.49	1.42
14	bA	1222	CLA	C1C-NC	-3.11	1.33	1.37
14	BA	1215	CLA	C1C-NC	-3.11	1.33	1.37
14	HA	1220	CLA	C1C-NC	-3.11	1.33	1.37
14	BA	1213	CLA	C1D-C2D	3.10	1.49	1.42
14	BA	1233	CLA	C1D-C2D	3.10	1.49	1.42
14	BA	1222	CLA	C1C-NC	-3.10	1.33	1.37
14	BA	1216	CLA	C1D-C2D	3.10	1.49	1.42
18	BA	4012	BCR	C11-C12	-3.10	1.26	1.34
14	AA	1115	CLA	C1D-C2D	3.10	1.49	1.42
14	AA	1117	CLA	C1C-NC	-3.10	1.33	1.37
14	GA	1138	CLA	C1C-NC	-3.09	1.33	1.37
14	AA	1137	CLA	C1C-NC	-3.09	1.33	1.37
14	GA	1123	CLA	MG-NC	3.09	2.13	2.06
14	AA	1119	CLA	C1C-NC	-3.09	1.33	1.37
14	HA	1208	CLA	C1D-C2D	3.09	1.49	1.42
14	AA	1108	CLA	C1D-C2D	3.09	1.49	1.42
14	HA	1203	CLA	C3D-C2D	3.09	1.45	1.39
14	AA	1138	CLA	C1C-NC	-3.09	1.33	1.37
14	HA	1224	CLA	C1C-NC	-3.09	1.33	1.37
14	aA	1137	CLA	C1C-NC	-3.09	1.33	1.37
14	BA	1208	CLA	C1C-NC	-3.09	1.33	1.37
14	HA	1229	CLA	C1D-C2D	3.09	1.49	1.42
14	KA	1401	CLA	MG-NC	3.09	2.13	2.06
14	kA	1401	CLA	MG-NC	3.09	2.13	2.06
14	GA	1137	CLA	C1C-NC	-3.09	1.33	1.37
14	BA	1208	CLA	C1D-C2D	3.08	1.49	1.42
14	BA	1224	CLA	C1C-NC	-3.08	1.33	1.37
14	AA	1123	CLA	MG-NC	3.08	2.13	2.06
14	BA	1209	CLA	C1D-C2D	3.08	1.49	1.42

Continued on next page...

PRELIMINARY VALIDATION REPORT

Continued from previous page...

Mol	Chain	Res	Type	Atoms	Z	Observed(Å)	Ideal(Å)
14	bA	1204	CLA	C1C-NC	-3.08	1.33	1.37
14	TA	1401	CLA	MG-NC	3.08	2.13	2.06
14	aA	1117	CLA	C1C-NC	-3.08	1.33	1.37
14	aA	1138	CLA	C1C-NC	-3.08	1.33	1.37
14	aA	1117	CLA	C1D-C2D	3.08	1.49	1.42
14	GA	1109	CLA	C1C-NC	-3.08	1.33	1.37
14	AA	1109	CLA	C1C-NC	-3.08	1.33	1.37
14	GA	1108	CLA	C1D-C2D	3.08	1.49	1.42
14	HA	1204	CLA	C1C-NC	-3.08	1.33	1.37
14	BA	1229	CLA	C1D-C2D	3.07	1.49	1.42
14	bA	1229	CLA	C1D-C2D	3.07	1.49	1.42
14	HA	1208	CLA	C1C-NC	-3.07	1.33	1.37
14	BA	1203	CLA	C3D-C2D	3.07	1.45	1.39
14	HA	1216	CLA	C1D-C2D	3.07	1.49	1.42
14	aA	1123	CLA	MG-NC	3.07	2.13	2.06
14	GA	1115	CLA	C1D-C2D	3.07	1.49	1.42
14	HA	1209	CLA	C1D-C2D	3.07	1.49	1.42
14	aA	1108	CLA	C1D-C2D	3.06	1.49	1.42
14	aA	1119	CLA	C1C-NC	-3.06	1.33	1.37
14	bA	1234	CLA	C3D-C2D	3.06	1.45	1.39
14	HA	1213	CLA	C1D-C2D	3.06	1.49	1.42
14	BA	1022	CLA	C3B-C2B	3.06	1.44	1.40
14	GA	1012	CLA	C1C-NC	-3.06	1.33	1.37
14	bA	1238	CLA	C1C-NC	-3.06	1.33	1.37
14	bA	1209	CLA	C1D-C2D	3.05	1.49	1.42
14	HA	1234	CLA	C3D-C2D	3.05	1.45	1.39
14	GA	1119	CLA	C1C-NC	-3.05	1.33	1.37
14	aA	1109	CLA	C1D-C2D	3.05	1.49	1.42
14	bA	1231	CLA	C4B-CHC	3.05	1.48	1.40
14	BA	1204	CLA	C1C-NC	-3.05	1.33	1.37
14	HA	1215	CLA	C1C-NC	-3.05	1.33	1.37
14	aA	1012	CLA	C1C-NC	-3.05	1.33	1.37
14	bA	1224	CLA	C1C-NC	-3.05	1.33	1.37
14	HA	1231	CLA	C4B-CHC	3.05	1.48	1.40
14	BA	1235	CLA	C1C-NC	-3.05	1.33	1.37
14	bA	1203	CLA	C3D-C2D	3.04	1.45	1.39
14	AA	1117	CLA	C1D-C2D	3.04	1.49	1.42
14	aA	1104	CLA	C1C-NC	-3.04	1.33	1.37
14	aA	1115	CLA	C1D-C2D	3.04	1.49	1.42
14	AA	1013	CLA	C4B-CHC	3.04	1.48	1.40
14	AA	1012	CLA	C1C-NC	-3.04	1.33	1.37
14	bA	1223	CLA	MG-NC	3.04	2.13	2.06

Continued on next page...

PRELIMINARY VALIDATION REPORT

Continued from previous page...

Mol	Chain	Res	Type	Atoms	Z	Observed(Å)	Ideal(Å)
14	GA	1109	CLA	C1D-C2D	3.04	1.49	1.42
14	HA	1238	CLA	C1C-NC	-3.03	1.33	1.37
14	bA	1228	CLA	C1D-C2D	3.03	1.49	1.42
14	aA	1013	CLA	C4B-CHC	3.02	1.48	1.40
14	GA	1140	CLA	C4B-CHC	3.02	1.48	1.40
14	0	1012	CLA	C3B-C2B	3.02	1.44	1.40
14	GA	1117	CLA	C1D-C2D	3.02	1.49	1.42
14	BA	1231	CLA	C4B-CHC	3.02	1.48	1.40
14	aA	1107	CLA	C1C-NC	-3.02	1.33	1.37
14	bA	1211	CLA	C1C-NC	-3.01	1.33	1.37
14	GA	1107	CLA	C1C-NC	-3.01	1.33	1.37
14	BA	1223	CLA	MG-NC	3.01	2.13	2.06
14	aA	1105	CLA	C1D-C2D	3.01	1.49	1.42
14	AA	1140	CLA	C4B-CHC	3.01	1.48	1.40
14	BA	1228	CLA	C1D-C2D	3.01	1.49	1.42
14	AA	1105	CLA	C1D-C2D	3.01	1.49	1.42
14	aA	1112	CLA	C1D-C2D	3.01	1.49	1.42
14	AA	1107	CLA	C1C-NC	-3.01	1.33	1.37
14	GA	1013	CLA	C4B-CHC	3.01	1.48	1.40
14	bA	1227	CLA	MG-NC	3.01	2.13	2.06
14	AA	1139	CLA	C4B-CHC	3.01	1.48	1.40
14	aA	1140	CLA	C4B-CHC	3.00	1.48	1.40
14	HA	1221	CLA	C1C-NC	-3.00	1.33	1.37
14	bA	1235	CLA	C1C-NC	-3.00	1.33	1.37
14	HA	1223	CLA	MG-NC	3.00	2.13	2.06
14	aA	1109	CLA	C1C-NC	-3.00	1.33	1.37
14	GA	1104	CLA	C1C-NC	-3.00	1.33	1.37
14	HA	1235	CLA	C1C-NC	-3.00	1.33	1.37
14	AA	1109	CLA	C1D-C2D	3.00	1.49	1.42
14	BA	1227	CLA	MG-NC	3.00	2.13	2.06
14	BA	1217	CLA	MG-NC	3.00	2.13	2.06
14	HA	1233	CLA	MG-NC	3.00	2.13	2.06
14	BA	1232	CLA	C1D-C2D	3.00	1.49	1.42
14	AA	1104	CLA	C1C-NC	-3.00	1.33	1.37
14	AA	1112	CLA	C1D-C2D	3.00	1.49	1.42
14	GA	1112	CLA	C1D-C2D	2.99	1.49	1.42
14	GA	1139	CLA	C4B-CHC	2.99	1.48	1.40
14	HA	1228	CLA	C1D-C2D	2.99	1.49	1.42
14	bA	1217	CLA	MG-NC	2.99	2.13	2.06
14	BA	1233	CLA	MG-NC	2.99	2.13	2.06
14	GA	1105	CLA	C1D-C2D	2.99	1.49	1.42
14	bA	1022	CLA	C1C-NC	-2.99	1.33	1.37

Continued on next page...

PRELIMINARY VALIDATION REPORT

Continued from previous page...

Mol	Chain	Res	Type	Atoms	Z	Observed(Å)	Ideal(Å)
14	bA	1221	CLA	C1C-NC	-2.99	1.33	1.37
14	bA	1232	CLA	C1D-C2D	2.99	1.49	1.42
13	GA	1011	CL0	C1B-CHB	2.99	1.48	1.40
14	BA	1236	CLA	C4B-CHC	2.98	1.48	1.40
14	HA	1217	CLA	MG-NC	2.98	2.13	2.06
14	BA	1238	CLA	C1C-NC	-2.98	1.33	1.37
14	BA	1211	CLA	C1C-NC	-2.98	1.33	1.37
14	HA	1227	CLA	MG-NC	2.98	2.13	2.06
14	XA	1701	CLA	C1C-NC	-2.98	1.33	1.37
14	aA	1138	CLA	C1D-C2D	2.98	1.49	1.42
14	BA	1221	CLA	C1C-NC	-2.98	1.33	1.37
14	GA	1138	CLA	C1D-C2D	2.98	1.49	1.42
14	HA	1232	CLA	MG-NC	2.97	2.13	2.06
14	HA	1236	CLA	C4B-CHC	2.97	1.48	1.40
14	HA	1211	CLA	C1C-NC	-2.97	1.33	1.37
14	bA	1232	CLA	MG-NC	2.97	2.13	2.06
14	BA	1214	CLA	MG-NC	2.97	2.13	2.06
14	BA	1022	CLA	C1C-NC	-2.97	1.33	1.37
14	bA	1236	CLA	C4B-CHC	2.97	1.48	1.40
13	AA	1011	CL0	C1B-CHB	2.97	1.48	1.40
14	bA	1212	CLA	C1C-NC	-2.97	1.33	1.37
14	bA	1022	CLA	C3B-C2B	2.96	1.44	1.40
14	aA	1139	CLA	C4B-CHC	2.96	1.48	1.40
14	HA	1232	CLA	C1D-C2D	2.96	1.49	1.42
14	bA	1233	CLA	MG-NC	2.96	2.13	2.06
14	aA	1112	CLA	C1C-NC	-2.96	1.33	1.37
14	xA	1701	CLA	C1C-NC	-2.96	1.33	1.37
14	bA	1214	CLA	MG-NC	2.96	2.13	2.06
14	AA	1138	CLA	C1D-C2D	2.95	1.49	1.42
14	BA	1212	CLA	C1C-NC	-2.95	1.33	1.37
14	GA	1135	CLA	C1C-NC	-2.95	1.33	1.37
14	bA	1231	CLA	C1C-NC	-2.95	1.33	1.37
14	BA	1022	CLA	C4B-CHC	2.95	1.48	1.40
14	HA	1214	CLA	MG-NC	2.95	2.13	2.06
14	bA	1022	CLA	C4B-CHC	2.95	1.48	1.40
14	WA	1701	CLA	C1C-NC	-2.95	1.33	1.37
14	BA	1232	CLA	MG-NC	2.94	2.13	2.06
14	HA	1212	CLA	C1C-NC	-2.94	1.33	1.37
13	aA	1011	CL0	C1B-CHB	2.94	1.47	1.40
14	AA	1129	CLA	C1C-NC	-2.94	1.33	1.37
14	GA	1112	CLA	C1C-NC	-2.94	1.33	1.37
14	0	1012	CLA	C4B-CHC	2.93	1.47	1.40

Continued on next page...

PRELIMINARY VALIDATION REPORT

Continued from previous page...

Mol	Chain	Res	Type	Atoms	Z	Observed(Å)	Ideal(Å)
14	GA	1133	CLA	C1D-C2D	2.93	1.49	1.42
14	HA	1231	CLA	C1C-NC	-2.93	1.33	1.37
14	GA	1116	CLA	MG-NC	2.93	2.13	2.06
14	AA	1116	CLA	MG-NC	2.93	2.13	2.06
14	BA	1231	CLA	C1C-NC	-2.92	1.33	1.37
14	aA	1113	CLA	C1D-C2D	2.92	1.49	1.42
14	AA	1112	CLA	C1C-NC	-2.92	1.33	1.37
14	AA	1115	CLA	C1C-NC	-2.92	1.33	1.37
14	BA	1238	CLA	C4B-CHC	2.92	1.47	1.40
14	aA	1115	CLA	C1C-NC	-2.92	1.33	1.37
14	GA	1115	CLA	C1C-NC	-2.92	1.33	1.37
14	AA	1133	CLA	C1D-C2D	2.91	1.49	1.42
14	0	1012	CLA	C1C-NC	-2.91	1.33	1.37
14	BA	1229	CLA	C1C-NC	-2.91	1.33	1.37
14	aA	1110	CLA	MG-NC	2.91	2.13	2.06
14	aA	1133	CLA	C1D-C2D	2.91	1.49	1.42
14	BA	1214	CLA	C1D-C2D	2.91	1.49	1.42
14	HA	1229	CLA	C1C-NC	-2.91	1.33	1.37
14	bA	1238	CLA	C4B-CHC	2.91	1.47	1.40
14	HA	1214	CLA	C1D-C2D	2.91	1.49	1.42
21	aA	6002	LMT	O3'-C3'	-2.91	1.36	1.43
14	GA	1113	CLA	C1D-C2D	2.90	1.49	1.42
14	GA	1133	CLA	C1C-NC	-2.90	1.33	1.37
14	bA	1209	CLA	C1C-NC	-2.90	1.33	1.37
14	AA	1135	CLA	C1C-NC	-2.90	1.33	1.37
14	GA	1111	CLA	C1D-C2D	2.90	1.49	1.42
14	GA	1129	CLA	C1C-NC	-2.90	1.33	1.37
21	AA	6002	LMT	O3'-C3'	-2.90	1.36	1.43
14	AA	1133	CLA	MG-NC	2.90	2.13	2.06
14	aA	1132	CLA	C3B-C2B	2.90	1.44	1.40
14	AA	1113	CLA	C1D-C2D	2.90	1.49	1.42
14	AA	1118	CLA	C1C-NC	-2.90	1.33	1.37
14	AA	1133	CLA	C1C-NC	-2.90	1.33	1.37
14	HA	1231	CLA	C1D-C2D	2.90	1.49	1.42
14	GA	1105	CLA	C1C-NC	-2.89	1.33	1.37
14	bA	1233	CLA	C1B-CHB	2.89	1.47	1.40
14	aA	1111	CLA	C1D-C2D	2.89	1.49	1.42
14	AA	1118	CLA	C1D-C2D	2.89	1.49	1.42
14	WA	1701	CLA	C4B-CHC	2.89	1.47	1.40
14	AA	1124	CLA	C4B-CHC	2.89	1.47	1.40
14	aA	1118	CLA	C1C-NC	-2.89	1.33	1.37
14	BA	1234	CLA	C4B-CHC	2.89	1.47	1.40

Continued on next page...

PRELIMINARY VALIDATION REPORT

Continued from previous page...

Mol	Chain	Res	Type	Atoms	Z	Observed(Å)	Ideal(Å)
14	aA	1133	CLA	C1C-NC	-2.89	1.33	1.37
14	BA	1231	CLA	C1D-C2D	2.89	1.49	1.42
14	AA	1111	CLA	C1D-C2D	2.89	1.49	1.42
14	HA	1238	CLA	C4B-CHC	2.89	1.47	1.40
14	GA	1108	CLA	MG-NC	2.89	2.13	2.06
14	BA	1218	CLA	MG-NC	2.89	2.13	2.06
14	bA	1234	CLA	C4B-CHC	2.89	1.47	1.40
14	GA	1108	CLA	C4B-CHC	2.89	1.47	1.40
14	xA	1701	CLA	MG-NC	2.89	2.13	2.06
14	aA	1124	CLA	C4B-CHC	2.88	1.47	1.40
14	GA	1118	CLA	C1C-NC	-2.88	1.33	1.37
14	aA	1102	CLA	MG-NC	2.88	2.13	2.06
14	aA	1118	CLA	C1D-C2D	2.88	1.49	1.42
14	AA	1119	CLA	C1D-C2D	2.88	1.49	1.42
14	XA	1701	CLA	MG-NC	2.88	2.13	2.06
14	AA	1140	CLA	MG-NC	2.88	2.13	2.06
14	bA	1214	CLA	C1D-C2D	2.88	1.49	1.42
14	GA	1132	CLA	C3B-C2B	2.88	1.44	1.40
14	HA	1234	CLA	C4B-CHC	2.88	1.47	1.40
14	AA	1101	CLA	C1C-NC	-2.88	1.33	1.37
14	AA	1135	CLA	C1D-C2D	2.88	1.49	1.42
14	GA	1110	CLA	MG-NC	2.88	2.13	2.06
14	GA	1124	CLA	C4B-CHC	2.88	1.47	1.40
14	aA	1101	CLA	C4B-CHC	2.88	1.47	1.40
14	AA	1108	CLA	MG-NC	2.88	2.13	2.06
14	HA	1218	CLA	C1D-C2D	2.88	1.49	1.42
14	bA	1235	CLA	C1D-C2D	2.87	1.49	1.42
14	bA	1215	CLA	C1D-C2D	2.87	1.49	1.42
14	aA	1135	CLA	C1C-NC	-2.87	1.33	1.37
21	GA	6002	LMT	O3'-C3'	-2.87	1.36	1.43
14	aA	1116	CLA	MG-NC	2.87	2.13	2.06
14	aA	1108	CLA	MG-NC	2.87	2.13	2.06
14	aA	1108	CLA	C4B-CHC	2.87	1.47	1.40
14	AA	1102	CLA	C1D-C2D	2.87	1.49	1.42
14	GA	1119	CLA	C1D-C2D	2.87	1.49	1.42
14	aA	1105	CLA	C1C-NC	-2.87	1.33	1.37
14	aA	1135	CLA	MG-NC	2.87	2.13	2.06
14	aA	1133	CLA	MG-NC	2.87	2.13	2.06
14	GA	1102	CLA	MG-NC	2.87	2.13	2.06
14	HA	1215	CLA	C1D-C2D	2.87	1.49	1.42
14	AA	1110	CLA	MG-NC	2.87	2.13	2.06
14	aA	1119	CLA	C1D-C2D	2.87	1.49	1.42

Continued on next page...

PRELIMINARY VALIDATION REPORT

Continued from previous page...

Mol	Chain	Res	Type	Atoms	Z	Observed(Å)	Ideal(Å)
14	BA	1022	CLA	MG-NC	2.87	2.13	2.06
14	GA	1118	CLA	C1D-C2D	2.87	1.49	1.42
14	GA	1133	CLA	MG-NC	2.87	2.13	2.06
14	BA	1235	CLA	C1D-C2D	2.87	1.49	1.42
14	GA	1102	CLA	C1D-C2D	2.87	1.49	1.42
14	aA	1102	CLA	C1D-C2D	2.87	1.49	1.42
14	aA	1107	CLA	MG-NC	2.87	2.13	2.06
14	BA	1215	CLA	C1D-C2D	2.86	1.49	1.42
14	AA	1105	CLA	C1C-NC	-2.86	1.33	1.37
14	aA	1101	CLA	C1C-NC	-2.86	1.33	1.37
14	GA	1140	CLA	MG-NC	2.86	2.13	2.06
14	AA	1108	CLA	C1C-NC	-2.86	1.33	1.37
14	aA	1129	CLA	C1C-NC	-2.86	1.33	1.37
14	BA	1218	CLA	C1D-C2D	2.86	1.49	1.42
14	AA	1135	CLA	MG-NC	2.86	2.13	2.06
14	GA	1135	CLA	MG-NC	2.86	2.13	2.06
14	bA	1022	CLA	MG-NC	2.86	2.13	2.06
14	HA	1235	CLA	C1D-C2D	2.86	1.49	1.42
14	aA	1130	CLA	OBD-CAD	2.86	1.26	1.22
14	GA	1132	CLA	MG-NC	2.86	2.13	2.06
14	GA	1135	CLA	C1D-C2D	2.86	1.49	1.42
14	AA	1108	CLA	C4B-CHC	2.86	1.47	1.40
14	BA	1233	CLA	C1B-CHB	2.86	1.47	1.40
14	bA	1214	CLA	C1C-NC	-2.85	1.33	1.37
14	AA	1102	CLA	MG-NC	2.85	2.13	2.06
14	UA	1503	CLA	C1D-C2D	2.85	1.49	1.42
14	XA	1701	CLA	C4B-CHC	2.85	1.47	1.40
14	bA	1229	CLA	C1C-NC	-2.85	1.33	1.37
14	HA	1218	CLA	MG-NC	2.85	2.13	2.06
14	aA	1123	CLA	C1C-NC	-2.85	1.33	1.37
14	BA	1222	CLA	C4B-CHC	2.85	1.47	1.40
14	aA	1140	CLA	MG-NC	2.85	2.13	2.06
14	AA	1132	CLA	C3B-C2B	2.85	1.44	1.40
21	aA	6001	LMT	O3'-C3'	-2.85	1.36	1.43
14	GA	1107	CLA	MG-NC	2.85	2.13	2.06
14	GA	1101	CLA	C4B-CHC	2.85	1.47	1.40
14	bA	1223	CLA	C4B-CHC	2.85	1.47	1.40
14	AA	1132	CLA	MG-NC	2.85	2.13	2.06
14	AA	1127	CLA	MG-NC	2.85	2.13	2.06
14	bA	1222	CLA	C4B-CHC	2.85	1.47	1.40
14	bA	1212	CLA	MG-NC	2.85	2.13	2.06
14	BA	1212	CLA	C4B-CHC	2.84	1.47	1.40

Continued on next page...

PRELIMINARY VALIDATION REPORT

Continued from previous page...

Mol	Chain	Res	Type	Atoms	Z	Observed(Å)	Ideal(Å)
14	GA	1108	CLA	C1C-NC	-2.84	1.33	1.37
14	bA	1219	CLA	C1C-NC	-2.84	1.33	1.37
14	HA	1212	CLA	C4B-CHC	2.84	1.47	1.40
21	AA	6001	LMT	O3'-C3'	-2.84	1.36	1.43
21	AA	6001	LMT	O2'-C2'	-2.84	1.36	1.43
14	aA	1135	CLA	C1D-C2D	2.84	1.49	1.42
14	aA	1110	CLA	C1C-NC	-2.84	1.33	1.37
14	AA	1116	CLA	C1D-C2D	2.84	1.49	1.42
14	HA	1210	CLA	C1D-C2D	2.84	1.49	1.42
14	BA	1210	CLA	C1D-C2D	2.84	1.49	1.42
14	GA	1124	CLA	C1D-C2D	2.84	1.49	1.42
14	GA	1101	CLA	C1C-NC	-2.84	1.33	1.37
14	aA	1127	CLA	MG-NC	2.84	2.13	2.06
14	WA	1701	CLA	MG-NC	2.84	2.13	2.06
21	GA	6001	LMT	O2'-C2'	-2.84	1.36	1.43
21	aA	6001	LMT	O2'-C2'	-2.84	1.36	1.43
14	BA	1209	CLA	C1C-NC	-2.84	1.33	1.37
14	BA	1214	CLA	C1C-NC	-2.84	1.33	1.37
14	aA	1124	CLA	C1D-C2D	2.84	1.49	1.42
14	bA	1208	CLA	C4B-CHC	2.84	1.47	1.40
14	HA	1209	CLA	C1C-NC	-2.83	1.33	1.37
14	0	1012	CLA	MG-NC	2.83	2.13	2.06
14	bA	1231	CLA	C1D-C2D	2.83	1.49	1.42
14	BA	1223	CLA	C4B-CHC	2.83	1.47	1.40
14	HA	1214	CLA	C1C-NC	-2.83	1.33	1.37
14	xA	1701	CLA	C4B-CHC	2.83	1.47	1.40
14	bA	1239	CLA	C1D-C2D	2.83	1.49	1.42
14	bA	1218	CLA	C1D-C2D	2.83	1.49	1.42
14	HA	1233	CLA	C1B-CHB	2.83	1.47	1.40
14	bA	1212	CLA	C4B-CHC	2.83	1.47	1.40
14	HA	1021	CLA	C1B-CHB	2.83	1.47	1.40
14	GA	1110	CLA	C4B-CHC	2.83	1.47	1.40
14	GA	1123	CLA	C1C-NC	-2.83	1.33	1.37
14	GA	1127	CLA	MG-NC	2.83	2.13	2.06
14	lA	1503	CLA	C1D-C2D	2.83	1.49	1.42
14	HA	1217	CLA	C1C-NC	-2.83	1.33	1.37
14	HA	1235	CLA	C4B-CHC	2.82	1.47	1.40
14	AA	1101	CLA	C4B-CHC	2.82	1.47	1.40
14	AA	1123	CLA	C1C-NC	-2.82	1.33	1.37
14	HA	1223	CLA	C4B-CHC	2.82	1.47	1.40
21	GA	6001	LMT	O3'-C3'	-2.82	1.36	1.43
14	AA	1110	CLA	C4B-CHC	2.82	1.47	1.40

Continued on next page...

PRELIMINARY VALIDATION REPORT

Continued from previous page...

Mol	Chain	Res	Type	Atoms	Z	Observed(Å)	Ideal(Å)
14	aA	1105	CLA	C4B-CHC	2.82	1.47	1.40
14	HA	1212	CLA	MG-NC	2.82	2.13	2.06
14	aA	1110	CLA	C4B-CHC	2.82	1.47	1.40
14	aA	1116	CLA	C1D-C2D	2.82	1.49	1.42
14	HA	1222	CLA	C4B-CHC	2.82	1.47	1.40
14	GA	1129	CLA	MG-NC	2.82	2.13	2.06
14	bA	1235	CLA	C4B-CHC	2.82	1.47	1.40
14	BA	1212	CLA	MG-NC	2.82	2.13	2.06
14	aA	1132	CLA	MG-NC	2.82	2.13	2.06
14	BA	1211	CLA	C1D-C2D	2.82	1.48	1.42
14	GA	1114	CLA	C1C-NC	-2.82	1.33	1.37
14	AA	1107	CLA	MG-NC	2.81	2.13	2.06
14	bA	1021	CLA	C1B-CHB	2.81	1.47	1.40
14	GA	1130	CLA	OBD-CAD	2.81	1.26	1.22
14	aA	1114	CLA	C1C-NC	-2.81	1.33	1.37
14	HA	1239	CLA	C1D-C2D	2.81	1.48	1.42
14	GA	1116	CLA	C1D-C2D	2.81	1.48	1.42
14	GA	1112	CLA	MG-NC	2.81	2.13	2.06
14	bA	1217	CLA	C1C-NC	-2.81	1.33	1.37
14	AA	1112	CLA	MG-NC	2.81	2.12	2.06
14	lA	1501	CLA	MG-NC	2.81	2.12	2.06
14	BA	1229	CLA	C4B-CHC	2.81	1.47	1.40
14	GA	1105	CLA	C4B-CHC	2.81	1.47	1.40
14	HA	1211	CLA	C1D-C2D	2.81	1.48	1.42
14	BA	1217	CLA	C1C-NC	-2.81	1.33	1.37
14	bA	1213	CLA	C1C-NC	-2.81	1.33	1.37
14	bA	1210	CLA	C1D-C2D	2.81	1.48	1.42
14	GA	1137	CLA	MG-NC	2.81	2.12	2.06
14	BA	1235	CLA	C4B-CHC	2.81	1.47	1.40
14	bA	1218	CLA	MG-NC	2.81	2.12	2.06
14	LA	1503	CLA	C1D-C2D	2.81	1.48	1.42
14	BA	1208	CLA	C4B-CHC	2.81	1.47	1.40
14	aA	1129	CLA	MG-NC	2.80	2.12	2.06
14	aA	1112	CLA	MG-NC	2.80	2.12	2.06
14	BA	1239	CLA	C1D-C2D	2.80	1.48	1.42
14	AA	1131	CLA	C1D-C2D	2.80	1.48	1.42
14	GA	1111	CLA	MG-NC	2.80	2.12	2.06
14	HA	1208	CLA	C4B-CHC	2.80	1.47	1.40
14	aA	1139	CLA	MG-NC	2.80	2.12	2.06
14	HA	1229	CLA	C4B-CHC	2.80	1.47	1.40
14	AA	1137	CLA	MG-NC	2.80	2.12	2.06
14	AA	1105	CLA	C4B-CHC	2.80	1.47	1.40

Continued on next page...

PRELIMINARY VALIDATION REPORT

Continued from previous page...

Mol	Chain	Res	Type	Atoms	Z	Observed(Å)	Ideal(Å)
14	bA	1211	CLA	C1D-C2D	2.80	1.48	1.42
14	GA	1137	CLA	C1D-C2D	2.80	1.48	1.42
14	kA	1401	CLA	C1B-CHB	2.80	1.47	1.40
14	AA	1129	CLA	C4B-CHC	2.80	1.47	1.40
14	AA	1110	CLA	C1D-C2D	2.80	1.48	1.42
14	KA	1401	CLA	C1B-CHB	2.79	1.47	1.40
14	aA	1129	CLA	C4B-CHC	2.79	1.47	1.40
14	AA	1129	CLA	MG-NC	2.79	2.12	2.06
14	aA	1111	CLA	MG-NC	2.79	2.12	2.06
14	AA	1111	CLA	MG-NC	2.79	2.12	2.06
14	bA	1218	CLA	C1B-CHB	2.79	1.47	1.40
14	HA	1218	CLA	C4B-CHC	2.79	1.47	1.40
14	HA	1222	CLA	MG-NC	2.79	2.12	2.06
14	AA	1124	CLA	C1D-C2D	2.79	1.48	1.42
14	HA	1213	CLA	C1C-NC	-2.79	1.33	1.37
14	BA	1021	CLA	C1B-CHB	2.79	1.47	1.40
14	BA	1224	CLA	MG-NC	2.79	2.12	2.06
14	AA	1114	CLA	C1C-NC	-2.79	1.33	1.37
14	HA	1218	CLA	C1C-NC	-2.79	1.33	1.37
14	AA	1133	CLA	C1B-CHB	2.79	1.47	1.40
14	BA	1211	CLA	C4B-CHC	2.79	1.47	1.40
14	bA	1231	CLA	MG-NC	2.79	2.12	2.06
14	aA	1137	CLA	MG-NC	2.79	2.12	2.06
14	HA	1219	CLA	C1D-C2D	2.79	1.48	1.42
14	aA	1133	CLA	C1B-CHB	2.79	1.47	1.40
14	LA	1501	CLA	MG-NC	2.79	2.12	2.06
14	bA	1213	CLA	MG-NC	2.79	2.12	2.06
14	aA	1137	CLA	C1D-C2D	2.78	1.48	1.42
14	bA	1224	CLA	MG-NC	2.78	2.12	2.06
14	HA	1207	CLA	C1D-C2D	2.78	1.48	1.42
14	BA	1219	CLA	C1C-NC	-2.78	1.33	1.37
14	bA	1222	CLA	MG-NC	2.78	2.12	2.06
14	bA	1219	CLA	C1D-C2D	2.78	1.48	1.42
14	HA	1219	CLA	C1C-NC	-2.78	1.33	1.37
14	bA	1219	CLA	MG-NC	2.78	2.12	2.06
14	BA	1218	CLA	C4B-CHC	2.78	1.47	1.40
14	aA	1110	CLA	C1D-C2D	2.78	1.48	1.42
14	GA	1129	CLA	C4B-CHC	2.78	1.47	1.40
14	AA	1139	CLA	MG-NC	2.78	2.12	2.06
14	BA	1213	CLA	MG-NC	2.78	2.12	2.06
14	HA	1213	CLA	MG-NC	2.78	2.12	2.06
14	HA	1213	CLA	C4B-CHC	2.78	1.47	1.40

Continued on next page...

PRELIMINARY VALIDATION REPORT

Continued from previous page...

Mol	Chain	Res	Type	Atoms	Z	Observed(Å)	Ideal(Å)
14	BA	1218	CLA	C1B-CHB	2.78	1.47	1.40
14	aA	1131	CLA	C1D-C2D	2.78	1.48	1.42
14	HA	1224	CLA	MG-NC	2.78	2.12	2.06
14	HA	1218	CLA	C1B-CHB	2.78	1.47	1.40
14	TA	1401	CLA	C1B-CHB	2.78	1.47	1.40
14	WA	1701	CLA	C1B-CHB	2.78	1.47	1.40
14	AA	1137	CLA	C1D-C2D	2.77	1.48	1.42
14	BA	1219	CLA	C1D-C2D	2.77	1.48	1.42
14	BA	1233	CLA	C4B-CHC	2.77	1.47	1.40
14	AA	1102	CLA	C4B-CHC	2.77	1.47	1.40
14	BA	1231	CLA	MG-NC	2.77	2.12	2.06
14	UA	1501	CLA	C4B-CHC	2.77	1.47	1.40
14	BA	1225	CLA	MG-NC	2.77	2.12	2.06
14	BA	1220	CLA	C1D-C2D	2.77	1.48	1.42
14	bA	1229	CLA	C4B-CHC	2.77	1.47	1.40
14	HA	1221	CLA	C4B-CHC	2.77	1.47	1.40
18	IA	4020	BCR	C17-C18	-2.77	1.32	1.35
18	RA	4020	BCR	C17-C18	-2.77	1.32	1.35
14	AA	1130	CLA	OBD-CAD	2.77	1.26	1.22
14	bA	1218	CLA	C4B-CHC	2.77	1.47	1.40
14	kA	1401	CLA	C4B-CHC	2.77	1.47	1.40
14	aA	1108	CLA	C1C-NC	-2.77	1.33	1.37
14	UA	1501	CLA	MG-NC	2.77	2.12	2.06
14	aA	1115	CLA	C4B-CHC	2.77	1.47	1.40
14	BA	1209	CLA	C4B-CHC	2.77	1.47	1.40
14	GA	1133	CLA	C1B-CHB	2.77	1.47	1.40
14	HA	1233	CLA	C4B-CHC	2.77	1.47	1.40
14	HA	1211	CLA	C4B-CHC	2.77	1.47	1.40
14	AA	1110	CLA	C1C-NC	-2.77	1.33	1.37
14	GA	1110	CLA	C1D-C2D	2.76	1.48	1.42
14	BA	1222	CLA	MG-NC	2.76	2.12	2.06
14	xA	1701	CLA	C1B-CHB	2.76	1.47	1.40
14	BA	1207	CLA	C1D-C2D	2.76	1.48	1.42
14	HA	1238	CLA	MG-NC	2.76	2.12	2.06
14	bA	1211	CLA	C4B-CHC	2.76	1.47	1.40
14	BA	1227	CLA	C1C-NC	-2.76	1.33	1.37
14	GA	1132	CLA	C1D-C2D	2.76	1.48	1.42
14	AA	1115	CLA	C4B-CHC	2.76	1.47	1.40
14	AA	1123	CLA	C1B-CHB	2.76	1.47	1.40
14	lA	1501	CLA	C4B-CHC	2.76	1.47	1.40
14	bA	1220	CLA	C1D-C2D	2.76	1.48	1.42
14	GA	1139	CLA	MG-NC	2.76	2.12	2.06

Continued on next page...

PRELIMINARY VALIDATION REPORT

Continued from previous page...

Mol	Chain	Res	Type	Atoms	Z	Observed(Å)	Ideal(Å)
14	HA	1225	CLA	MG-NC	2.76	2.12	2.06
14	GA	1131	CLA	C1D-C2D	2.76	1.48	1.42
14	LA	1501	CLA	C4B-CHC	2.76	1.47	1.40
14	aA	1102	CLA	C4B-CHC	2.76	1.47	1.40
14	bA	1221	CLA	C4B-CHC	2.75	1.47	1.40
14	bA	1233	CLA	C4B-CHC	2.75	1.47	1.40
14	AA	1106	CLA	MG-NC	2.75	2.12	2.06
14	BA	1224	CLA	C4B-CHC	2.75	1.47	1.40
14	bA	1217	CLA	C4B-CHC	2.75	1.47	1.40
14	BA	1221	CLA	C4B-CHC	2.75	1.47	1.40
14	BA	1213	CLA	C1C-NC	-2.75	1.33	1.37
14	AA	1132	CLA	C1D-C2D	2.75	1.48	1.42
14	GA	1123	CLA	C1B-CHB	2.75	1.47	1.40
18	iA	4020	BCR	C17-C18	-2.75	1.32	1.35
14	BA	1213	CLA	C4B-CHC	2.75	1.47	1.40
14	bA	1224	CLA	C4B-CHC	2.75	1.47	1.40
14	HA	1219	CLA	MG-NC	2.75	2.12	2.06
14	bA	1212	CLA	C1B-CHB	2.75	1.47	1.40
14	GA	1102	CLA	C4B-CHC	2.75	1.47	1.40
14	HA	1217	CLA	C1B-CHB	2.75	1.47	1.40
14	HA	1220	CLA	C1D-C2D	2.75	1.48	1.42
14	HA	1227	CLA	C1C-NC	-2.75	1.33	1.37
14	HA	1224	CLA	C4B-CHC	2.75	1.47	1.40
14	GA	1106	CLA	MG-NC	2.75	2.12	2.06
14	AA	1102	CLA	C1C-NC	-2.75	1.33	1.37
14	bA	1219	CLA	C4B-CHC	2.75	1.47	1.40
14	bA	1225	CLA	MG-NC	2.75	2.12	2.06
14	aA	1132	CLA	C1D-C2D	2.75	1.48	1.42
14	bA	1238	CLA	MG-NC	2.75	2.12	2.06
14	BA	1238	CLA	MG-NC	2.75	2.12	2.06
14	BA	1219	CLA	C4B-CHC	2.74	1.47	1.40
14	AA	1138	CLA	C4B-CHC	2.74	1.47	1.40
14	bA	1213	CLA	C4B-CHC	2.74	1.47	1.40
14	aA	1123	CLA	C1B-CHB	2.74	1.47	1.40
14	BA	1217	CLA	C4B-CHC	2.74	1.47	1.40
14	AA	1119	CLA	C4B-CHC	2.74	1.47	1.40
14	BA	1212	CLA	C1B-CHB	2.74	1.47	1.40
14	HA	1232	CLA	C1C-NC	-2.74	1.33	1.37
14	bA	1227	CLA	C1C-NC	-2.74	1.33	1.37
14	bA	1207	CLA	C1D-C2D	2.74	1.48	1.42
14	BA	1219	CLA	MG-NC	2.74	2.12	2.06
14	AA	1103	CLA	C1D-C2D	2.74	1.48	1.42

Continued on next page...

PRELIMINARY VALIDATION REPORT

Continued from previous page...

Mol	Chain	Res	Type	Atoms	Z	Observed(Å)	Ideal(Å)
14	HA	1209	CLA	C4B-CHC	2.74	1.47	1.40
14	XA	1701	CLA	C1B-CHB	2.74	1.47	1.40
14	GA	1110	CLA	C1C-NC	-2.74	1.33	1.37
14	BA	1217	CLA	C1B-CHB	2.74	1.47	1.40
14	KA	1401	CLA	C4B-CHC	2.74	1.47	1.40
14	GA	1138	CLA	C4B-CHC	2.74	1.47	1.40
14	GA	1102	CLA	C1C-NC	-2.74	1.33	1.37
14	HA	1231	CLA	MG-NC	2.74	2.12	2.06
14	HA	1239	CLA	MG-NC	2.74	2.12	2.06
14	GA	1115	CLA	C4B-CHC	2.74	1.47	1.40
14	bA	1217	CLA	C1B-CHB	2.74	1.47	1.40
14	AA	1115	CLA	MG-NC	2.74	2.12	2.06
14	bA	1239	CLA	MG-NC	2.74	2.12	2.06
14	BA	1239	CLA	MG-NC	2.73	2.12	2.06
14	TA	1401	CLA	C4B-CHC	2.73	1.47	1.40
14	AA	1101	CLA	MG-NC	2.73	2.12	2.06
14	bA	1209	CLA	C4B-CHC	2.73	1.47	1.40
14	aA	1101	CLA	C1D-C2D	2.73	1.48	1.42
14	aA	1102	CLA	C1C-NC	-2.73	1.33	1.37
14	HA	1219	CLA	C4B-CHC	2.73	1.47	1.40
14	aA	1116	CLA	C4B-CHC	2.73	1.47	1.40
14	GA	1114	CLA	MG-NC	2.73	2.12	2.06
14	HA	1217	CLA	C4B-CHC	2.73	1.47	1.40
14	BA	1218	CLA	C1C-NC	-2.73	1.33	1.37
14	aA	1138	CLA	C4B-CHC	2.73	1.47	1.40
14	BA	1220	CLA	C1B-CHB	2.73	1.47	1.40
14	AA	1136	CLA	C4B-CHC	2.73	1.47	1.40
14	AA	1130	CLA	C1D-C2D	2.73	1.48	1.42
14	aA	1136	CLA	C4B-CHC	2.73	1.47	1.40
14	GA	1101	CLA	MG-NC	2.72	2.12	2.06
14	aA	1115	CLA	MG-NC	2.72	2.12	2.06
14	HA	1201	CLA	MG-NC	2.72	2.12	2.06
14	GA	1116	CLA	C4B-CHC	2.72	1.47	1.40
14	bA	1201	CLA	MG-NC	2.72	2.12	2.06
14	GA	1105	CLA	MG-NC	2.72	2.12	2.06
14	GA	1137	CLA	C1B-CHB	2.72	1.47	1.40
14	aA	1130	CLA	C1D-C2D	2.72	1.48	1.42
14	bA	1210	CLA	MG-NC	2.72	2.12	2.06
14	AA	1130	CLA	C4B-CHC	2.72	1.47	1.40
14	bA	1208	CLA	MG-NC	2.72	2.12	2.06
14	GA	1119	CLA	C4B-CHC	2.72	1.47	1.40
14	AA	1101	CLA	C1D-C2D	2.72	1.48	1.42

Continued on next page...

PRELIMINARY VALIDATION REPORT

Continued from previous page...

Mol	Chain	Res	Type	Atoms	Z	Observed(Å)	Ideal(Å)
14	AA	1106	CLA	C1B-CHB	2.72	1.47	1.40
14	HA	1239	CLA	C1B-CHB	2.72	1.47	1.40
14	bA	1218	CLA	C1C-NC	-2.72	1.33	1.37
14	AA	1116	CLA	C4B-CHC	2.72	1.47	1.40
14	bA	1239	CLA	C1B-CHB	2.72	1.47	1.40
14	GA	1126	CLA	C1D-C2D	2.71	1.48	1.42
14	AA	1122	CLA	C1D-C2D	2.71	1.48	1.42
14	GA	1130	CLA	C4B-CHC	2.71	1.47	1.40
14	GA	1122	CLA	MG-NC	2.71	2.12	2.06
14	GA	1136	CLA	C4B-CHC	2.71	1.47	1.40
14	aA	1114	CLA	MG-NC	2.71	2.12	2.06
14	aA	1116	CLA	C1B-CHB	2.71	1.47	1.40
14	GA	1101	CLA	C1D-C2D	2.71	1.48	1.42
14	bA	1217	CLA	C1D-C2D	2.71	1.48	1.42
14	GA	1110	CLA	C1B-CHB	2.71	1.47	1.40
14	AA	1137	CLA	C1B-CHB	2.71	1.47	1.40
14	aA	1110	CLA	C1B-CHB	2.71	1.47	1.40
14	AA	1110	CLA	C1B-CHB	2.71	1.47	1.40
14	HA	1212	CLA	C1B-CHB	2.71	1.47	1.40
14	BA	1217	CLA	C1D-C2D	2.71	1.48	1.42
14	GA	1115	CLA	MG-NC	2.71	2.12	2.06
14	GA	1136	CLA	MG-NC	2.71	2.12	2.06
14	GA	1103	CLA	C1D-C2D	2.71	1.48	1.42
14	aA	1101	CLA	MG-NC	2.71	2.12	2.06
14	GA	1104	CLA	C1D-C2D	2.71	1.48	1.42
14	AA	1114	CLA	MG-NC	2.71	2.12	2.06
14	BA	1220	CLA	MG-NC	2.71	2.12	2.06
14	aA	1122	CLA	C1D-C2D	2.71	1.48	1.42
14	bA	1220	CLA	C1B-CHB	2.70	1.47	1.40
14	AA	1104	CLA	C1D-C2D	2.70	1.48	1.42
14	HA	1220	CLA	C1B-CHB	2.70	1.47	1.40
14	AA	1126	CLA	C1D-C2D	2.70	1.48	1.42
14	BA	1208	CLA	C1B-CHB	2.70	1.47	1.40
14	BA	1239	CLA	C1B-CHB	2.70	1.47	1.40
14	GA	1130	CLA	C1D-C2D	2.70	1.48	1.42
14	HA	1220	CLA	MG-NC	2.70	2.12	2.06
14	GA	1108	CLA	C1B-CHB	2.70	1.47	1.40
14	HA	1206	CLA	C1B-CHB	2.70	1.47	1.40
14	aA	1106	CLA	MG-NC	2.70	2.12	2.06
14	aA	1103	CLA	C1D-C2D	2.70	1.48	1.42
14	aA	1136	CLA	C1D-C2D	2.70	1.48	1.42
14	AA	1136	CLA	C1D-C2D	2.70	1.48	1.42

Continued on next page...

PRELIMINARY VALIDATION REPORT

Continued from previous page...

Mol	Chain	Res	Type	Atoms	Z	Observed(Å)	Ideal(Å)
14	aA	1130	CLA	C4B-CHC	2.70	1.47	1.40
14	BA	1232	CLA	C1C-NC	-2.70	1.33	1.37
14	GA	1118	CLA	MG-NC	2.70	2.12	2.06
14	aA	1108	CLA	C1B-CHB	2.70	1.47	1.40
14	GA	1116	CLA	C1B-CHB	2.70	1.47	1.40
14	AA	1105	CLA	MG-NC	2.70	2.12	2.06
14	aA	1106	CLA	C1D-C2D	2.70	1.48	1.42
14	aA	1137	CLA	C1B-CHB	2.70	1.47	1.40
14	bA	1232	CLA	C1C-NC	-2.70	1.33	1.37
14	GA	1106	CLA	C1B-CHB	2.70	1.47	1.40
14	aA	1119	CLA	C4B-CHC	2.69	1.47	1.40
14	aA	1140	CLA	C1B-CHB	2.69	1.47	1.40
14	AA	1106	CLA	C1D-C2D	2.69	1.48	1.42
21	AA	6002	LMT	O3B-C3B	-2.69	1.36	1.43
14	HA	1210	CLA	MG-NC	2.69	2.12	2.06
14	bA	1205	CLA	C4B-CHC	2.69	1.47	1.40
14	bA	1208	CLA	C1B-CHB	2.69	1.47	1.40
14	bA	1227	CLA	C4B-CHC	2.69	1.47	1.40
14	AA	1108	CLA	C1B-CHB	2.69	1.47	1.40
14	aA	1106	CLA	C1B-CHB	2.69	1.47	1.40
14	AA	1118	CLA	MG-NC	2.69	2.12	2.06
14	BA	1208	CLA	MG-NC	2.69	2.12	2.06
14	HA	1227	CLA	C4B-CHC	2.69	1.47	1.40
14	bA	1206	CLA	C1B-CHB	2.69	1.47	1.40
14	aA	1118	CLA	C1B-CHB	2.68	1.47	1.40
14	aA	1131	CLA	C4B-CHC	2.68	1.47	1.40
14	aA	1115	CLA	C1B-CHB	2.68	1.47	1.40
14	AA	1140	CLA	C1B-CHB	2.68	1.47	1.40
14	GA	1131	CLA	C4B-CHC	2.68	1.47	1.40
14	aA	1104	CLA	C1D-C2D	2.68	1.48	1.42
14	GA	1136	CLA	C1D-C2D	2.68	1.48	1.42
14	GA	1122	CLA	C1D-C2D	2.68	1.48	1.42
14	AA	1122	CLA	MG-NC	2.68	2.12	2.06
14	aA	1118	CLA	MG-NC	2.68	2.12	2.06
14	bA	1220	CLA	MG-NC	2.68	2.12	2.06
14	aA	1109	CLA	C1B-CHB	2.68	1.47	1.40
14	HA	1205	CLA	C4B-CHC	2.68	1.47	1.40
21	GA	6002	LMT	O3B-C3B	-2.68	1.36	1.43
14	aA	1122	CLA	MG-NC	2.68	2.12	2.06
14	GA	1126	CLA	MG-NC	2.68	2.12	2.06
14	GA	1106	CLA	C4B-CHC	2.67	1.47	1.40
21	aA	6002	LMT	O3B-C3B	-2.67	1.36	1.43

Continued on next page...

PRELIMINARY VALIDATION REPORT

Continued from previous page...

Mol	Chain	Res	Type	Atoms	Z	Observed(Å)	Ideal(Å)
14	AA	1116	CLA	C1B-CHB	2.67	1.47	1.40
14	HA	1208	CLA	MG-NC	2.67	2.12	2.06
14	GA	1118	CLA	C1B-CHB	2.67	1.47	1.40
14	GA	1140	CLA	C1B-CHB	2.67	1.47	1.40
14	AA	1118	CLA	C1B-CHB	2.67	1.47	1.40
14	HA	1217	CLA	C1D-C2D	2.67	1.48	1.42
14	BA	1201	CLA	MG-NC	2.67	2.12	2.06
14	BA	1205	CLA	C4B-CHC	2.67	1.47	1.40
14	BA	1210	CLA	MG-NC	2.67	2.12	2.06
14	aA	1126	CLA	C1D-C2D	2.67	1.48	1.42
14	XA	1701	CLA	C1D-C2D	2.67	1.48	1.42
14	aA	1112	CLA	C4B-CHC	2.67	1.47	1.40
14	AA	1136	CLA	MG-NC	2.67	2.12	2.06
14	GA	1106	CLA	C1D-C2D	2.67	1.48	1.42
14	aA	1135	CLA	C1B-CHB	2.67	1.47	1.40
14	GA	1109	CLA	C1B-CHB	2.67	1.47	1.40
14	BA	1227	CLA	C4B-CHC	2.67	1.47	1.40
14	aA	1136	CLA	MG-NC	2.67	2.12	2.06
14	bA	1221	CLA	MG-NC	2.67	2.12	2.06
14	HA	1208	CLA	C1B-CHB	2.66	1.47	1.40
14	aA	1126	CLA	MG-NC	2.66	2.12	2.06
14	AA	1135	CLA	C1B-CHB	2.66	1.47	1.40
14	BA	1221	CLA	MG-NC	2.66	2.12	2.06
14	aA	1105	CLA	MG-NC	2.66	2.12	2.06
14	GA	1132	CLA	C4B-CHC	2.66	1.47	1.40
14	HA	1202	CLA	C4B-CHC	2.66	1.47	1.40
14	UA	1502	CLA	C1D-C2D	2.66	1.48	1.42
14	BA	1209	CLA	MG-NC	2.66	2.12	2.06
14	bA	1223	CLA	C1B-CHB	2.66	1.47	1.40
14	aA	1138	CLA	MG-NC	2.66	2.12	2.06
14	bA	1202	CLA	C4B-CHC	2.66	1.47	1.40
14	GA	1126	CLA	C4B-CHC	2.66	1.47	1.40
14	bA	1201	CLA	C4B-CHC	2.66	1.47	1.40
14	GA	1115	CLA	C1B-CHB	2.66	1.47	1.40
14	GA	1135	CLA	C1B-CHB	2.66	1.47	1.40
14	AA	1126	CLA	C4B-CHC	2.66	1.47	1.40
14	bA	1209	CLA	MG-NC	2.66	2.12	2.06
14	AA	1106	CLA	C4B-CHC	2.66	1.47	1.40
14	AA	1109	CLA	C1B-CHB	2.66	1.47	1.40
14	HA	1221	CLA	MG-NC	2.66	2.12	2.06
14	AA	1107	CLA	C1B-CHB	2.66	1.47	1.40
14	AA	1112	CLA	C4B-CHC	2.66	1.47	1.40

Continued on next page...

PRELIMINARY VALIDATION REPORT

Continued from previous page...

Mol	Chain	Res	Type	Atoms	Z	Observed(Å)	Ideal(Å)
14	bA	1201	CLA	C1D-C2D	2.66	1.48	1.42
14	xA	1701	CLA	C1D-C2D	2.65	1.48	1.42
14	BA	1206	CLA	C1B-CHB	2.65	1.47	1.40
14	GA	1138	CLA	MG-NC	2.65	2.12	2.06
14	GA	1107	CLA	C1B-CHB	2.65	1.47	1.40
14	aA	1127	CLA	C1B-CHB	2.65	1.47	1.40
14	AA	1115	CLA	C1B-CHB	2.65	1.47	1.40
14	GA	1123	CLA	C4B-CHC	2.65	1.47	1.40
14	AA	1132	CLA	C4B-CHC	2.65	1.47	1.40
14	aA	1111	CLA	C4B-CHC	2.65	1.47	1.40
14	aA	1126	CLA	C4B-CHC	2.65	1.47	1.40
14	bA	1203	CLA	C4B-CHC	2.65	1.47	1.40
14	WA	1701	CLA	C1D-C2D	2.65	1.48	1.42
14	AA	1126	CLA	MG-NC	2.65	2.12	2.06
14	HA	1201	CLA	C1D-C2D	2.65	1.48	1.42
14	LA	1503	CLA	C1B-CHB	2.65	1.47	1.40
14	aA	1106	CLA	C4B-CHC	2.65	1.47	1.40
14	BA	1227	CLA	C1D-C2D	2.64	1.48	1.42
14	BA	1202	CLA	C4B-CHC	2.64	1.47	1.40
14	aA	1107	CLA	C1B-CHB	2.64	1.47	1.40
14	aA	1122	CLA	C4B-CHC	2.64	1.47	1.40
14	bA	1211	CLA	MG-NC	2.64	2.12	2.06
14	BA	1234	CLA	MG-NC	2.64	2.12	2.06
14	HA	1227	CLA	C1B-CHB	2.64	1.47	1.40
14	BA	1207	CLA	C1B-CHB	2.64	1.47	1.40
14	kA	1401	CLA	C1C-NC	-2.64	1.33	1.37
14	HA	1209	CLA	MG-NC	2.64	2.12	2.06
14	AA	1127	CLA	C1B-CHB	2.64	1.47	1.40
14	GA	1111	CLA	C4B-CHC	2.64	1.47	1.40
14	AA	1138	CLA	MG-NC	2.64	2.12	2.06
14	UA	1503	CLA	C1B-CHB	2.64	1.47	1.40
14	AA	1133	CLA	C4B-CHC	2.64	1.47	1.40
14	aA	1123	CLA	C4B-CHC	2.64	1.47	1.40
14	HA	1203	CLA	C4B-CHC	2.64	1.47	1.40
14	AA	1123	CLA	C4B-CHC	2.64	1.47	1.40
14	BA	1203	CLA	C4B-CHC	2.63	1.47	1.40
14	LA	1502	CLA	C1D-C2D	2.63	1.48	1.42
14	AA	1116	CLA	C1C-NC	-2.63	1.33	1.37
14	BA	1228	CLA	MG-NC	2.63	2.12	2.06
14	aA	1118	CLA	C4B-CHC	2.63	1.47	1.40
14	BA	1227	CLA	C1B-CHB	2.63	1.47	1.40
14	BA	1201	CLA	C1D-C2D	2.63	1.48	1.42

Continued on next page...

PRELIMINARY VALIDATION REPORT

Continued from previous page...

Mol	Chain	Res	Type	Atoms	Z	Observed(Å)	Ideal(Å)
14	HA	1228	CLA	MG-NC	2.63	2.12	2.06
14	lA	1502	CLA	C1D-C2D	2.63	1.48	1.42
14	AA	1122	CLA	C4B-CHC	2.63	1.47	1.40
14	HA	1236	CLA	C1D-C2D	2.63	1.48	1.42
14	aA	1132	CLA	C4B-CHC	2.63	1.47	1.40
14	GA	1112	CLA	C4B-CHC	2.63	1.47	1.40
14	HA	1211	CLA	MG-NC	2.63	2.12	2.06
14	BA	1201	CLA	C4B-CHC	2.63	1.47	1.40
14	TA	1401	CLA	C1C-NC	-2.63	1.33	1.37
14	BA	1223	CLA	C1B-CHB	2.63	1.47	1.40
14	GA	1122	CLA	C4B-CHC	2.63	1.47	1.40
14	HA	1223	CLA	C1B-CHB	2.63	1.47	1.40
14	BA	1236	CLA	C1D-C2D	2.63	1.48	1.42
14	AA	1107	CLA	C4B-CHC	2.63	1.47	1.40
14	AA	1131	CLA	C4B-CHC	2.63	1.47	1.40
14	lA	1503	CLA	C1B-CHB	2.62	1.47	1.40
14	HA	1201	CLA	C4B-CHC	2.62	1.47	1.40
14	AA	1101	CLA	C1B-CHB	2.62	1.47	1.40
14	GA	1133	CLA	C4B-CHC	2.62	1.47	1.40
14	BA	1232	CLA	C4B-CHC	2.62	1.47	1.40
14	BA	1211	CLA	MG-NC	2.62	2.12	2.06
14	bA	1228	CLA	MG-NC	2.62	2.12	2.06
14	aA	1107	CLA	C4B-CHC	2.62	1.47	1.40
14	AA	1012	CLA	MG-NC	2.62	2.12	2.06
14	GA	1107	CLA	C4B-CHC	2.62	1.47	1.40
14	bA	1211	CLA	C1B-CHB	2.62	1.47	1.40
14	bA	1207	CLA	MG-NC	2.62	2.12	2.06
14	bA	1227	CLA	C1B-CHB	2.62	1.47	1.40
14	AA	1118	CLA	C4B-CHC	2.62	1.47	1.40
14	GA	1116	CLA	C1C-NC	-2.62	1.33	1.37
14	HA	1207	CLA	C1B-CHB	2.62	1.47	1.40
14	UA	1502	CLA	C4B-CHC	2.62	1.47	1.40
14	bA	1234	CLA	MG-NC	2.62	2.12	2.06
14	bA	1227	CLA	C1D-C2D	2.62	1.48	1.42
14	AA	1111	CLA	C4B-CHC	2.61	1.47	1.40
14	bA	1203	CLA	C1D-C2D	2.61	1.48	1.42
14	AA	1130	CLA	MG-NC	2.61	2.12	2.06
14	LA	1502	CLA	C1B-CHB	2.61	1.47	1.40
14	GA	1127	CLA	C1B-CHB	2.61	1.47	1.40
14	BA	1207	CLA	MG-NC	2.61	2.12	2.06
14	HA	1227	CLA	C1D-C2D	2.61	1.48	1.42
14	GA	1129	CLA	C1B-CHB	2.61	1.47	1.40

Continued on next page...

PRELIMINARY VALIDATION REPORT

Continued from previous page...

Mol	Chain	Res	Type	Atoms	Z	Observed(Å)	Ideal(Å)
14	bA	1203	CLA	C1B-CHB	2.61	1.47	1.40
14	bA	1207	CLA	C1B-CHB	2.61	1.47	1.40
14	aA	1012	CLA	MG-NC	2.61	2.12	2.06
14	bA	1204	CLA	C1D-C2D	2.61	1.48	1.42
14	BA	1213	CLA	C1B-CHB	2.61	1.47	1.40
14	GA	1139	CLA	C1D-C2D	2.61	1.48	1.42
14	AA	1109	CLA	C4B-CHC	2.61	1.47	1.40
14	GA	1118	CLA	C4B-CHC	2.60	1.47	1.40
14	BA	1232	CLA	C1B-CHB	2.60	1.47	1.40
14	HA	1213	CLA	C1B-CHB	2.60	1.47	1.40
14	BA	1233	CLA	C1C-NC	-2.60	1.33	1.37
14	aA	1119	CLA	MG-NC	2.60	2.12	2.06
14	bA	1232	CLA	C4B-CHC	2.60	1.47	1.40
14	bA	1215	CLA	C4B-CHC	2.60	1.47	1.40
14	HA	1203	CLA	C1B-CHB	2.60	1.47	1.40
14	BA	1204	CLA	C1D-C2D	2.60	1.48	1.42
21	aA	6002	LMT	O2B-C2B	-2.60	1.36	1.43
21	GA	6002	LMT	O2B-C2B	-2.60	1.36	1.43
14	aA	1129	CLA	C1B-CHB	2.60	1.47	1.40
14	GA	1130	CLA	MG-NC	2.60	2.12	2.06
14	BA	1203	CLA	C1B-CHB	2.60	1.47	1.40
14	aA	1109	CLA	C4B-CHC	2.60	1.47	1.40
14	aA	1101	CLA	C1B-CHB	2.60	1.47	1.40
14	HA	1023	CLA	C4C-C3C	2.60	1.49	1.45
14	lA	1502	CLA	C1B-CHB	2.60	1.47	1.40
14	aA	1012	CLA	C1D-C2D	2.60	1.48	1.42
14	GA	1127	CLA	C4B-CHC	2.60	1.47	1.40
14	aA	1116	CLA	C1C-NC	-2.60	1.33	1.37
14	aA	1133	CLA	C4B-CHC	2.60	1.47	1.40
14	HA	1215	CLA	C4B-CHC	2.59	1.47	1.40
14	aA	1139	CLA	C1D-C2D	2.59	1.48	1.42
14	bA	1233	CLA	C1C-NC	-2.59	1.33	1.37
14	HA	1226	CLA	C1D-C2D	2.59	1.48	1.42
14	bA	1232	CLA	C1B-CHB	2.59	1.47	1.40
14	HA	1204	CLA	C1D-C2D	2.59	1.48	1.42
14	GA	1135	CLA	C4B-CHC	2.59	1.47	1.40
14	aA	1135	CLA	C4B-CHC	2.59	1.47	1.40
14	lA	1502	CLA	C4B-CHC	2.59	1.47	1.40
14	HA	1204	CLA	C4B-CHC	2.59	1.47	1.40
14	BA	1023	CLA	C4C-C3C	2.59	1.49	1.45
14	HA	1207	CLA	MG-NC	2.59	2.12	2.06
14	AA	1012	CLA	C1D-C2D	2.59	1.48	1.42

Continued on next page...

PRELIMINARY VALIDATION REPORT

Continued from previous page...

Mol	Chain	Res	Type	Atoms	Z	Observed(Å)	Ideal(Å)
14	AA	1139	CLA	C1D-C2D	2.59	1.48	1.42
14	AA	1129	CLA	C1B-CHB	2.59	1.47	1.40
14	HA	1233	CLA	C1C-NC	-2.59	1.33	1.37
14	AA	1127	CLA	C4B-CHC	2.59	1.47	1.40
14	GA	1109	CLA	C4B-CHC	2.59	1.47	1.40
14	KA	1401	CLA	C1C-NC	-2.59	1.33	1.37
14	AA	1109	CLA	MG-NC	2.59	2.12	2.06
14	UA	1502	CLA	C1B-CHB	2.59	1.47	1.40
14	GA	1101	CLA	C1B-CHB	2.59	1.47	1.40
14	bA	1213	CLA	C1B-CHB	2.58	1.47	1.40
14	bA	1236	CLA	C1D-C2D	2.58	1.48	1.42
14	BA	1211	CLA	C1B-CHB	2.58	1.47	1.40
14	HA	1232	CLA	C4B-CHC	2.58	1.47	1.40
14	bA	1234	CLA	C1B-CHB	2.58	1.47	1.40
14	aA	1139	CLA	C1B-CHB	2.58	1.47	1.40
14	AA	1104	CLA	C4B-CHC	2.58	1.47	1.40
14	HA	1232	CLA	C1B-CHB	2.58	1.47	1.40
21	AA	6002	LMT	O2B-C2B	-2.58	1.36	1.43
14	BA	1215	CLA	C4B-CHC	2.58	1.47	1.40
14	HA	1210	CLA	C4B-CHC	2.58	1.47	1.40
14	AA	1119	CLA	MG-NC	2.58	2.12	2.06
14	bA	1236	CLA	C1B-CHB	2.58	1.47	1.40
14	BA	1235	CLA	MG-NC	2.58	2.12	2.06
14	HA	1211	CLA	C1B-CHB	2.58	1.47	1.40
14	aA	1114	CLA	C4B-CHC	2.58	1.47	1.40
14	AA	1012	CLA	C4B-CHC	2.58	1.47	1.40
14	HA	1234	CLA	MG-NC	2.58	2.12	2.06
14	aA	1012	CLA	C4B-CHC	2.58	1.47	1.40
14	aA	1127	CLA	C4B-CHC	2.58	1.47	1.40
14	GA	1012	CLA	C4B-CHC	2.58	1.47	1.40
14	GA	1012	CLA	MG-NC	2.58	2.12	2.06
14	bA	1023	CLA	C4C-C3C	2.57	1.49	1.45
14	HA	1228	CLA	C4B-CHC	2.57	1.47	1.40
14	HA	1207	CLA	C4B-CHC	2.57	1.47	1.40
14	BA	1210	CLA	C4B-CHC	2.57	1.47	1.40
14	GA	1012	CLA	C1D-C2D	2.57	1.48	1.42
14	GA	1112	CLA	C1B-CHB	2.57	1.47	1.40
14	HA	1222	CLA	C1D-C2D	2.57	1.48	1.42
14	HA	1235	CLA	MG-NC	2.57	2.12	2.06
14	BA	1203	CLA	C1D-C2D	2.57	1.48	1.42
14	bA	1226	CLA	C1D-C2D	2.57	1.48	1.42
14	LA	1502	CLA	C4B-CHC	2.57	1.47	1.40

Continued on next page...

PRELIMINARY VALIDATION REPORT

Continued from previous page...

Mol	Chain	Res	Type	Atoms	Z	Observed(Å)	Ideal(Å)
14	aA	1130	CLA	MG-NC	2.57	2.12	2.06
14	BA	1204	CLA	C4B-CHC	2.57	1.47	1.40
14	bA	1228	CLA	C4B-CHC	2.57	1.47	1.40
14	aA	1113	CLA	MG-NC	2.57	2.12	2.06
14	BA	1207	CLA	C4B-CHC	2.57	1.47	1.40
14	HA	1234	CLA	C1B-CHB	2.56	1.47	1.40
14	BA	1226	CLA	C1D-C2D	2.56	1.48	1.42
14	GA	1104	CLA	C4B-CHC	2.56	1.47	1.40
14	bA	1210	CLA	C4B-CHC	2.56	1.47	1.40
14	bA	1235	CLA	MG-NC	2.56	2.12	2.06
14	bA	1206	CLA	MG-NC	2.56	2.12	2.06
14	aA	1123	CLA	C1D-C2D	2.56	1.48	1.42
14	aA	1131	CLA	MG-NC	2.56	2.12	2.06
14	aA	1104	CLA	C1B-CHB	2.56	1.46	1.40
14	bA	1215	CLA	MG-NC	2.56	2.12	2.06
14	AA	1113	CLA	MG-NC	2.56	2.12	2.06
14	bA	1207	CLA	C4B-CHC	2.56	1.46	1.40
14	AA	1125	CLA	C4B-CHC	2.56	1.46	1.40
14	GA	1125	CLA	C1B-CHB	2.56	1.46	1.40
14	GA	1139	CLA	C1B-CHB	2.56	1.46	1.40
14	AA	1135	CLA	C4B-CHC	2.56	1.46	1.40
14	LA	1502	CLA	MG-NC	2.56	2.12	2.06
14	BA	1236	CLA	C1B-CHB	2.56	1.46	1.40
20	LA	5007	LMG	C43-C42	-2.56	1.33	1.51
14	BA	1228	CLA	C4B-CHC	2.56	1.46	1.40
14	aA	1104	CLA	C4B-CHC	2.56	1.46	1.40
20	lA	5007	LMG	C43-C42	-2.56	1.33	1.51
14	aA	1125	CLA	C1B-CHB	2.56	1.46	1.40
14	bA	1220	CLA	C4B-CHC	2.56	1.46	1.40
14	BA	1234	CLA	C1B-CHB	2.56	1.46	1.40
14	LA	1503	CLA	C4B-CHC	2.55	1.46	1.40
14	GA	1125	CLA	C4B-CHC	2.55	1.46	1.40
14	AA	1128	CLA	MG-NC	2.55	2.12	2.06
14	lA	1503	CLA	C4B-CHC	2.55	1.46	1.40
14	AA	1125	CLA	C1B-CHB	2.55	1.46	1.40
14	BA	1215	CLA	MG-NC	2.55	2.12	2.06
14	aA	1128	CLA	MG-NC	2.55	2.12	2.06
14	BA	1202	CLA	C1D-C2D	2.55	1.48	1.42
14	AA	1139	CLA	C1B-CHB	2.55	1.46	1.40
14	BA	1229	CLA	MG-NC	2.55	2.12	2.06
14	AA	1123	CLA	C1D-C2D	2.55	1.48	1.42
14	BA	1222	CLA	C1D-C2D	2.55	1.48	1.42

Continued on next page...

PRELIMINARY VALIDATION REPORT

Continued from previous page...

Mol	Chain	Res	Type	Atoms	Z	Observed(Å)	Ideal(Å)
14	HA	1226	CLA	MG-NC	2.55	2.12	2.06
14	GA	1119	CLA	MG-NC	2.55	2.12	2.06
14	HA	1203	CLA	C1D-C2D	2.55	1.48	1.42
14	bA	1204	CLA	C4B-CHC	2.55	1.46	1.40
14	UA	1503	CLA	C4B-CHC	2.55	1.46	1.40
21	aA	6002	LMT	O2'-C2'	-2.55	1.36	1.43
14	GA	1114	CLA	C4B-CHC	2.55	1.46	1.40
14	HA	1236	CLA	C1B-CHB	2.55	1.46	1.40
14	AA	1112	CLA	C1B-CHB	2.55	1.46	1.40
14	aA	1112	CLA	C1B-CHB	2.54	1.46	1.40
14	bA	1202	CLA	C1D-C2D	2.54	1.48	1.42
14	bA	1222	CLA	C1D-C2D	2.54	1.48	1.42
14	GA	1105	CLA	C1B-CHB	2.54	1.46	1.40
20	UA	5007	LMG	C43-C42	-2.54	1.33	1.51
14	aA	1125	CLA	C4B-CHC	2.54	1.46	1.40
14	BA	1206	CLA	MG-NC	2.54	2.12	2.06
14	aA	1124	CLA	MG-NC	2.54	2.12	2.06
14	GA	1124	CLA	MG-NC	2.54	2.12	2.06
14	BA	1220	CLA	C4B-CHC	2.54	1.46	1.40
14	AA	1113	CLA	C4B-CHC	2.54	1.46	1.40
14	AA	1104	CLA	C1B-CHB	2.54	1.46	1.40
14	GA	1123	CLA	C1D-C2D	2.54	1.48	1.42
14	aA	1113	CLA	C1B-CHB	2.54	1.46	1.40
14	bA	1229	CLA	MG-NC	2.54	2.12	2.06
21	AA	6002	LMT	O2'-C2'	-2.53	1.37	1.43
14	GA	1113	CLA	C4B-CHC	2.53	1.46	1.40
14	HA	1229	CLA	C1B-CHB	2.53	1.46	1.40
14	AA	1113	CLA	C1B-CHB	2.53	1.46	1.40
14	GA	1109	CLA	MG-NC	2.53	2.12	2.06
14	bA	1229	CLA	C1B-CHB	2.53	1.46	1.40
14	AA	1131	CLA	MG-NC	2.53	2.12	2.06
14	HA	1220	CLA	C4B-CHC	2.53	1.46	1.40
14	GA	1104	CLA	C1B-CHB	2.53	1.46	1.40
14	AA	1114	CLA	C4B-CHC	2.53	1.46	1.40
14	HA	1225	CLA	C1B-CHB	2.53	1.46	1.40
14	GA	1128	CLA	MG-NC	2.53	2.12	2.06
14	LA	1501	CLA	C1B-CHB	2.53	1.46	1.40
14	bA	1226	CLA	MG-NC	2.53	2.12	2.06
14	aA	1113	CLA	C4B-CHC	2.52	1.46	1.40
14	BA	1226	CLA	MG-NC	2.52	2.12	2.06
14	bA	1214	CLA	C4B-CHC	2.52	1.46	1.40
14	AA	1136	CLA	C1B-CHB	2.52	1.46	1.40

Continued on next page...

PRELIMINARY VALIDATION REPORT

Continued from previous page...

Mol	Chain	Res	Type	Atoms	Z	Observed(Å)	Ideal(Å)
14	lA	1502	CLA	MG-NC	2.52	2.12	2.06
14	BA	1229	CLA	C1B-CHB	2.52	1.46	1.40
14	HA	1229	CLA	MG-NC	2.52	2.12	2.06
14	BA	1225	CLA	C4B-CHC	2.52	1.46	1.40
14	BA	1021	CLA	C4B-CHC	2.52	1.46	1.40
14	HA	1215	CLA	MG-NC	2.52	2.12	2.06
14	HA	1206	CLA	MG-NC	2.52	2.12	2.06
14	UA	1502	CLA	MG-NC	2.52	2.12	2.06
14	HA	1021	CLA	C4B-CHC	2.52	1.46	1.40
14	aA	1136	CLA	C1B-CHB	2.52	1.46	1.40
14	AA	1105	CLA	C1B-CHB	2.52	1.46	1.40
14	GA	1113	CLA	MG-NC	2.52	2.12	2.06
14	bA	1225	CLA	C1B-CHB	2.52	1.46	1.40
14	AA	1124	CLA	MG-NC	2.52	2.12	2.06
14	GA	1103	CLA	C1B-CHB	2.52	1.46	1.40
14	aA	1105	CLA	C1B-CHB	2.52	1.46	1.40
14	HA	1202	CLA	C1D-C2D	2.52	1.48	1.42
14	bA	1021	CLA	C4B-CHC	2.52	1.46	1.40
14	aA	1109	CLA	MG-NC	2.51	2.12	2.06
14	aA	1114	CLA	CHD-C4C	2.51	1.48	1.41
14	HA	1225	CLA	C4B-CHC	2.51	1.46	1.40
14	bA	1221	CLA	C1D-C2D	2.51	1.48	1.42
14	HA	1216	CLA	MG-NC	2.51	2.12	2.06
14	BA	1216	CLA	MG-NC	2.51	2.12	2.06
14	BA	1236	CLA	MG-NC	2.51	2.12	2.06
14	AA	1103	CLA	C1B-CHB	2.51	1.46	1.40
14	GA	1136	CLA	C1B-CHB	2.51	1.46	1.40
14	AA	1114	CLA	CHD-C4C	2.51	1.48	1.41
20	UA	5007	LMG	C22-C21	-2.51	1.33	1.51
14	aA	1128	CLA	C1D-C2D	2.51	1.48	1.42
14	aA	1103	CLA	C1B-CHB	2.50	1.46	1.40
14	bA	1225	CLA	C4B-CHC	2.50	1.46	1.40
20	lA	5007	LMG	C22-C21	-2.50	1.33	1.51
20	LA	5007	LMG	C22-C21	-2.50	1.33	1.51
14	BA	1228	CLA	CHD-C4C	2.50	1.48	1.41
14	AA	1117	CLA	CHD-C4C	2.50	1.48	1.41
14	UA	1501	CLA	C1B-CHB	2.50	1.46	1.40
14	BA	1225	CLA	C1B-CHB	2.50	1.46	1.40
18	iA	4020	BCR	C14-C13	-2.50	1.32	1.35
14	GA	1114	CLA	CHD-C4C	2.50	1.48	1.41
21	GA	6002	LMT	O2'-C2'	-2.50	1.37	1.43
14	GA	1131	CLA	MG-NC	2.50	2.12	2.06

Continued on next page...

PRELIMINARY VALIDATION REPORT

Continued from previous page...

Mol	Chain	Res	Type	Atoms	Z	Observed(Å)	Ideal(Å)
14	bA	1228	CLA	CHD-C4C	2.49	1.48	1.41
14	bA	1221	CLA	C1B-CHB	2.49	1.46	1.40
14	BA	1214	CLA	C4B-CHC	2.49	1.46	1.40
21	aA	6001	LMT	O2B-C2B	-2.49	1.37	1.43
14	bA	1216	CLA	C4B-CHC	2.49	1.46	1.40
14	bA	1222	CLA	C1B-CHB	2.49	1.46	1.40
14	bA	1216	CLA	MG-NC	2.49	2.12	2.06
14	GA	1117	CLA	CHD-C4C	2.49	1.48	1.41
14	GA	1122	CLA	C1B-CHB	2.49	1.46	1.40
14	BA	1202	CLA	MG-NC	2.49	2.12	2.06
14	HA	1214	CLA	C4B-CHC	2.49	1.46	1.40
14	bA	1212	CLA	C1D-C2D	2.49	1.48	1.42
14	aA	1117	CLA	CHD-C4C	2.49	1.48	1.41
14	HA	1228	CLA	CHD-C4C	2.49	1.48	1.41
14	BA	1221	CLA	C1B-CHB	2.49	1.46	1.40
14	HA	1221	CLA	C1B-CHB	2.48	1.46	1.40
14	HA	1221	CLA	C1D-C2D	2.48	1.48	1.42
14	GA	1113	CLA	C1B-CHB	2.48	1.46	1.40
14	AA	1137	CLA	C4B-CHC	2.48	1.46	1.40
14	GA	1117	CLA	MG-NC	2.48	2.12	2.06
14	BA	1222	CLA	C1B-CHB	2.48	1.46	1.40
14	bA	1202	CLA	MG-NC	2.48	2.12	2.06
14	BA	1226	CLA	CMB-C2B	-2.48	1.46	1.51
14	AA	1111	CLA	C1B-CHB	2.48	1.46	1.40
14	LA	1503	CLA	MG-NC	2.48	2.12	2.06
14	GA	1137	CLA	C4B-CHC	2.48	1.46	1.40
14	lA	1501	CLA	C1B-CHB	2.48	1.46	1.40
14	HA	1216	CLA	C4B-CHC	2.48	1.46	1.40
14	BA	1204	CLA	MG-NC	2.48	2.12	2.06
14	bA	1236	CLA	MG-NC	2.48	2.12	2.06
14	aA	1137	CLA	C4B-CHC	2.48	1.46	1.40
14	AA	1122	CLA	C1B-CHB	2.48	1.46	1.40
14	BA	1228	CLA	C1B-CHB	2.48	1.46	1.40
14	BA	1221	CLA	C1D-C2D	2.47	1.48	1.42
14	HA	1228	CLA	C1B-CHB	2.47	1.46	1.40
14	bA	1226	CLA	CMB-C2B	-2.47	1.46	1.51
14	HA	1239	CLA	C4B-CHC	2.47	1.46	1.40
14	HA	1212	CLA	C1D-C2D	2.47	1.48	1.42
14	HA	1222	CLA	C1B-CHB	2.47	1.46	1.40
14	HA	1202	CLA	MG-NC	2.47	2.12	2.06
14	bA	1239	CLA	C4B-CHC	2.47	1.46	1.40
14	lA	1503	CLA	MG-NC	2.47	2.12	2.06

Continued on next page...

PRELIMINARY VALIDATION REPORT

Continued from previous page...

Mol	Chain	Res	Type	Atoms	Z	Observed(Å)	Ideal(Å)
14	bA	1228	CLA	C1B-CHB	2.47	1.46	1.40
21	AA	6001	LMT	O2B-C2B	-2.47	1.37	1.43
14	UA	1503	CLA	MG-NC	2.47	2.12	2.06
21	GA	6002	LMT	O4'-C4B	-2.47	1.37	1.43
14	HA	1209	CLA	C1B-CHB	2.47	1.46	1.40
14	HA	1236	CLA	MG-NC	2.46	2.12	2.06
14	BA	1234	CLA	C1D-C2D	2.46	1.48	1.42
14	bA	1204	CLA	CHD-C4C	2.46	1.48	1.41
14	BA	1209	CLA	C1B-CHB	2.46	1.46	1.40
14	bA	1209	CLA	C1B-CHB	2.46	1.46	1.40
21	aA	6002	LMT	O4'-C4B	-2.46	1.37	1.43
14	BA	1219	CLA	C1B-CHB	2.46	1.46	1.40
14	BA	1216	CLA	C4B-CHC	2.46	1.46	1.40
14	AA	1128	CLA	C1D-C2D	2.46	1.48	1.42
14	bA	1219	CLA	C1B-CHB	2.46	1.46	1.40
14	bA	1234	CLA	C1D-C2D	2.46	1.48	1.42
14	BA	1212	CLA	C1D-C2D	2.46	1.48	1.42
14	GA	1140	CLA	C1D-C2D	2.46	1.48	1.42
14	HA	1219	CLA	C1B-CHB	2.46	1.46	1.40
14	aA	1122	CLA	C1B-CHB	2.46	1.46	1.40
14	HA	1204	CLA	CHD-C4C	2.45	1.48	1.41
14	BA	1204	CLA	CHD-C4C	2.45	1.48	1.41
14	AA	1107	CLA	C1D-C2D	2.45	1.48	1.42
14	aA	1111	CLA	C1B-CHB	2.45	1.46	1.40
14	bA	1204	CLA	MG-NC	2.45	2.12	2.06
14	HA	1210	CLA	C1B-CHB	2.45	1.46	1.40
14	BA	1239	CLA	C4B-CHC	2.45	1.46	1.40
14	HA	1204	CLA	MG-NC	2.45	2.12	2.06
14	AA	1117	CLA	MG-NC	2.45	2.12	2.06
14	GA	1107	CLA	C1D-C2D	2.45	1.48	1.42
14	GA	1111	CLA	C1B-CHB	2.45	1.46	1.40
14	aA	1117	CLA	MG-NC	2.45	2.12	2.06
14	HA	1226	CLA	CMB-C2B	-2.44	1.46	1.51
14	BA	1210	CLA	C1B-CHB	2.44	1.46	1.40
14	aA	1107	CLA	C1D-C2D	2.44	1.48	1.42
14	HA	1234	CLA	C1D-C2D	2.44	1.48	1.42
14	aA	1140	CLA	C1D-C2D	2.44	1.48	1.42
14	GA	1128	CLA	C1D-C2D	2.43	1.48	1.42
14	bA	1210	CLA	C1B-CHB	2.43	1.46	1.40
14	GA	1128	CLA	C1B-CHB	2.43	1.46	1.40
21	GA	6001	LMT	O2B-C2B	-2.43	1.37	1.43
14	aA	1128	CLA	C1B-CHB	2.43	1.46	1.40

Continued on next page...

PRELIMINARY VALIDATION REPORT

Continued from previous page...

Mol	Chain	Res	Type	Atoms	Z	Observed(Å)	Ideal(Å)
14	AA	1012	CLA	C3B-C2B	2.43	1.43	1.40
14	bA	1215	CLA	C1B-CHB	2.43	1.46	1.40
14	HA	1231	CLA	C1B-CHB	2.43	1.46	1.40
18	RA	4020	BCR	C21-C22	-2.42	1.32	1.35
14	bA	1022	CLA	C1B-CHB	2.42	1.46	1.40
14	BA	1231	CLA	C1B-CHB	2.42	1.46	1.40
14	bA	1202	CLA	C1B-CHB	2.42	1.46	1.40
14	AA	1140	CLA	C1D-C2D	2.42	1.48	1.42
14	bA	1214	CLA	C1B-CHB	2.42	1.46	1.40
14	aA	1105	CLA	CHD-C4C	2.42	1.48	1.41
14	aA	1103	CLA	C4B-CHC	2.42	1.46	1.40
14	HA	1214	CLA	C1B-CHB	2.42	1.46	1.40
21	AA	6002	LMT	O4'-C4B	-2.42	1.37	1.43
14	HA	1215	CLA	C1B-CHB	2.41	1.46	1.40
14	aA	1117	CLA	C1B-CHB	2.41	1.46	1.40
14	BA	1202	CLA	C1B-CHB	2.41	1.46	1.40
14	AA	1128	CLA	C1B-CHB	2.41	1.46	1.40
14	aA	1129	CLA	C1D-C2D	2.41	1.48	1.42
14	bA	1231	CLA	C1B-CHB	2.41	1.46	1.40
14	HA	1214	CLA	CHD-C4C	2.41	1.48	1.41
14	aA	1012	CLA	C3B-C2B	2.41	1.43	1.40
14	BA	1215	CLA	C1B-CHB	2.41	1.46	1.40
14	AA	1105	CLA	CHD-C4C	2.40	1.48	1.41
14	AA	1103	CLA	C4B-CHC	2.40	1.46	1.40
14	AA	1103	CLA	MG-NC	2.40	2.12	2.06
14	HA	1202	CLA	C1B-CHB	2.40	1.46	1.40
14	HA	1023	CLA	C1B-CHB	2.40	1.46	1.40
14	aA	1103	CLA	MG-NC	2.40	2.12	2.06
14	BA	1214	CLA	CHD-C4C	2.40	1.48	1.41
14	BA	1214	CLA	C1B-CHB	2.40	1.46	1.40
14	BA	1022	CLA	C1B-CHB	2.40	1.46	1.40
14	0	1012	CLA	C1B-CHB	2.40	1.46	1.40
18	RA	4020	BCR	C14-C13	-2.39	1.32	1.35
14	GA	1129	CLA	C1D-C2D	2.39	1.48	1.42
14	AA	1128	CLA	CMB-C2B	-2.39	1.46	1.51
14	GA	1012	CLA	C3B-C2B	2.39	1.43	1.40
14	GA	1103	CLA	C4B-CHC	2.39	1.46	1.40
14	AA	1129	CLA	C1D-C2D	2.39	1.48	1.42
14	AA	1128	CLA	C4B-CHC	2.39	1.46	1.40
14	HA	1216	CLA	CHD-C4C	2.39	1.48	1.41
14	BA	1232	CLA	CHD-C4C	2.38	1.48	1.41
18	IA	4020	BCR	C21-C22	-2.38	1.32	1.35

Continued on next page...

PRELIMINARY VALIDATION REPORT

Continued from previous page...

Mol	Chain	Res	Type	Atoms	Z	Observed(Å)	Ideal(Å)
14	bA	1214	CLA	CHD-C4C	2.38	1.48	1.41
14	BA	1216	CLA	CHD-C4C	2.38	1.48	1.41
18	IA	4020	BCR	C14-C13	-2.38	1.32	1.35
14	GA	1117	CLA	C1B-CHB	2.38	1.46	1.40
14	UA	1501	CLA	C1D-C2D	2.38	1.48	1.42
14	GA	1128	CLA	C4B-CHC	2.38	1.46	1.40
14	GA	1103	CLA	MG-NC	2.38	2.11	2.06
21	AA	6001	LMT	O3B-C3B	-2.38	1.37	1.43
14	lA	1501	CLA	C1D-C2D	2.38	1.47	1.42
14	HA	1201	CLA	C1B-CHB	2.38	1.46	1.40
14	GA	1138	CLA	CHD-C4C	2.37	1.48	1.41
14	GA	1105	CLA	CHD-C4C	2.37	1.48	1.41
13	GA	1011	CL0	MG-NC	2.37	2.11	2.06
14	aA	1102	CLA	CHD-C4C	2.37	1.48	1.41
14	GA	1109	CLA	CHD-C4C	2.37	1.48	1.41
14	HA	1235	CLA	C1B-CHB	2.37	1.46	1.40
14	bA	1201	CLA	C1B-CHB	2.37	1.46	1.40
14	bA	1216	CLA	CHD-C4C	2.37	1.48	1.41
14	BA	1023	CLA	C1B-CHB	2.37	1.46	1.40
14	HA	1226	CLA	CHD-C4C	2.37	1.48	1.41
14	HA	1229	CLA	CHD-C4C	2.36	1.48	1.41
13	AA	1011	CL0	MG-NC	2.36	2.11	2.06
18	iA	4020	BCR	C21-C22	-2.36	1.32	1.35
21	GA	6001	LMT	O3B-C3B	-2.36	1.37	1.43
21	aA	6001	LMT	O3B-C3B	-2.36	1.37	1.43
14	aA	1138	CLA	C1B-CHB	2.36	1.46	1.40
14	GA	1130	CLA	C1B-CHB	2.36	1.46	1.40
14	AA	1117	CLA	C1B-CHB	2.36	1.46	1.40
14	BA	1227	CLA	CHD-C4C	2.36	1.48	1.41
14	bA	1235	CLA	C1B-CHB	2.36	1.46	1.40
14	BA	1226	CLA	CHD-C4C	2.36	1.48	1.41
14	aA	1109	CLA	CHD-C4C	2.36	1.48	1.41
14	LA	1501	CLA	C1D-C2D	2.36	1.47	1.42
14	bA	1023	CLA	C1B-CHB	2.36	1.46	1.40
14	HA	1232	CLA	CHD-C4C	2.36	1.48	1.41
14	BA	1206	CLA	C4B-CHC	2.36	1.46	1.40
14	HA	1021	CLA	MG-NC	2.35	2.11	2.06
14	GA	1102	CLA	C1B-CHB	2.35	1.46	1.40
14	bA	1216	CLA	C1B-CHB	2.35	1.46	1.40
14	AA	1109	CLA	CHD-C4C	2.35	1.47	1.41
14	GA	1128	CLA	CMB-C2B	-2.35	1.46	1.51
14	TA	1401	CLA	CHD-C4C	2.35	1.47	1.41

Continued on next page...

PRELIMINARY VALIDATION REPORT

Continued from previous page...

Mol	Chain	Res	Type	Atoms	Z	Observed(Å)	Ideal(Å)
14	aA	1128	CLA	C4B-CHC	2.35	1.46	1.40
14	bA	1226	CLA	CHD-C4C	2.35	1.47	1.41
14	aA	1128	CLA	CMB-C2B	-2.35	1.46	1.51
14	HA	1203	CLA	MG-NC	2.35	2.11	2.06
14	bA	1229	CLA	CHD-C4C	2.35	1.47	1.41
14	BA	1021	CLA	MG-NC	2.35	2.11	2.06
14	GA	1102	CLA	CHD-C4C	2.35	1.47	1.41
14	kA	1401	CLA	CHD-C4C	2.35	1.47	1.41
14	HA	1205	CLA	MG-NC	2.35	2.11	2.06
14	BA	1235	CLA	C1B-CHB	2.35	1.46	1.40
14	BA	1205	CLA	C1B-CHB	2.35	1.46	1.40
14	HA	1210	CLA	C4C-C3C	2.35	1.49	1.45
14	HA	1227	CLA	CHD-C4C	2.34	1.47	1.41
18	UA	4022	BCR	C14-C13	-2.34	1.32	1.35
14	AA	1138	CLA	CHD-C4C	2.34	1.47	1.41
14	bA	1232	CLA	CHD-C4C	2.34	1.47	1.41
14	bA	1203	CLA	MG-NC	2.34	2.11	2.06
13	aA	1011	CL0	MG-NC	2.34	2.11	2.06
14	AA	1138	CLA	C1B-CHB	2.34	1.46	1.40
14	aA	1102	CLA	C1B-CHB	2.34	1.46	1.40
14	bA	1205	CLA	MG-NC	2.34	2.11	2.06
14	bA	1205	CLA	C1B-CHB	2.34	1.46	1.40
14	AA	1102	CLA	CHD-C4C	2.34	1.47	1.41
14	BA	1216	CLA	C1B-CHB	2.34	1.46	1.40
14	AA	1112	CLA	CHD-C4C	2.34	1.47	1.41
14	bA	1227	CLA	CHD-C4C	2.34	1.47	1.41
14	aA	1112	CLA	CHD-C4C	2.34	1.47	1.41
14	BA	1205	CLA	MG-NC	2.34	2.11	2.06
14	HA	1206	CLA	C4B-CHC	2.34	1.46	1.40
14	KA	1401	CLA	CHD-C4C	2.33	1.47	1.41
18	GA	4008	BCR	C17-C18	-2.33	1.32	1.35
14	HA	1224	CLA	C1B-CHB	2.33	1.46	1.40
14	HA	1213	CLA	CHD-C4C	2.33	1.47	1.41
14	aA	1138	CLA	CHD-C4C	2.33	1.47	1.41
14	bA	1224	CLA	C1B-CHB	2.33	1.46	1.40
14	AA	1102	CLA	C1B-CHB	2.33	1.46	1.40
14	GA	1115	CLA	CHD-C4C	2.33	1.47	1.41
14	bA	1206	CLA	C4B-CHC	2.33	1.46	1.40
14	BA	1229	CLA	CHD-C4C	2.33	1.47	1.41
14	bA	1210	CLA	CHD-C4C	2.33	1.47	1.41
14	AA	1108	CLA	CHD-C4C	2.33	1.47	1.41
14	BA	1201	CLA	C1B-CHB	2.33	1.46	1.40

Continued on next page...

PRELIMINARY VALIDATION REPORT

Continued from previous page...

Mol	Chain	Res	Type	Atoms	Z	Observed(Å)	Ideal(Å)
14	BA	1231	CLA	CHD-C4C	2.33	1.47	1.41
14	BA	1203	CLA	MG-NC	2.33	2.11	2.06
14	HA	1216	CLA	C1B-CHB	2.33	1.46	1.40
18	HA	4005	BCR	C30-C25	-2.33	1.50	1.53
13	AA	1011	CL0	C3A-C2A	-2.33	1.47	1.54
14	GA	1116	CLA	CHD-C4C	2.33	1.47	1.41
14	BA	1224	CLA	C1B-CHB	2.33	1.46	1.40
14	HA	1222	CLA	CHD-C4C	2.32	1.47	1.41
18	lA	4022	BCR	C14-C13	-2.32	1.32	1.35
14	BA	1222	CLA	CHD-C4C	2.32	1.47	1.41
13	GA	1011	CL0	C3A-C2A	-2.32	1.48	1.54
14	bA	1222	CLA	CHD-C4C	2.32	1.47	1.41
14	aA	1130	CLA	C1B-CHB	2.32	1.46	1.40
14	bA	1213	CLA	CHD-C4C	2.32	1.47	1.41
14	GA	1138	CLA	C1B-CHB	2.32	1.46	1.40
14	AA	1130	CLA	C1B-CHB	2.32	1.46	1.40
14	GA	1108	CLA	CHD-C4C	2.32	1.47	1.41
18	LA	4022	BCR	C14-C13	-2.32	1.32	1.35
14	AA	1117	CLA	C4B-CHC	2.32	1.46	1.40
13	GA	1011	CL0	C1D-C2D	2.32	1.47	1.42
14	BA	1213	CLA	CHD-C4C	2.32	1.47	1.41
14	GA	1112	CLA	CHD-C4C	2.32	1.47	1.41
14	HA	1205	CLA	C1B-CHB	2.31	1.46	1.40
14	aA	1135	CLA	CHD-C4C	2.31	1.47	1.41
13	aA	1011	CL0	C3A-C2A	-2.31	1.48	1.54
14	AA	1116	CLA	CHD-C4C	2.31	1.47	1.41
14	bA	1021	CLA	MG-NC	2.31	2.11	2.06
14	BA	1210	CLA	C4C-C3C	2.31	1.49	1.45
14	AA	1115	CLA	CHD-C4C	2.31	1.47	1.41
13	aA	1011	CL0	C1D-C2D	2.31	1.47	1.42
14	GA	1114	CLA	C1B-CHB	2.31	1.46	1.40
18	aA	4008	BCR	C17-C18	-2.31	1.32	1.35
14	GA	1113	CLA	CHD-C4C	2.31	1.47	1.41
14	HA	1233	CLA	CHD-C4C	2.31	1.47	1.41
14	HA	1204	CLA	C1B-CHB	2.31	1.46	1.40
14	AA	1113	CLA	CHD-C4C	2.31	1.47	1.41
14	aA	1104	CLA	MG-NC	2.31	2.11	2.06
14	AA	1104	CLA	MG-NC	2.31	2.11	2.06
14	GA	1104	CLA	MG-NC	2.31	2.11	2.06
14	HA	1231	CLA	CHD-C4C	2.30	1.47	1.41
14	BA	1204	CLA	C1B-CHB	2.30	1.46	1.40
14	BA	1209	CLA	CHD-C4C	2.30	1.47	1.41

Continued on next page...

PRELIMINARY VALIDATION REPORT

Continued from previous page...

Mol	Chain	Res	Type	Atoms	Z	Observed(Å)	Ideal(Å)
14	HA	1210	CLA	CHD-C4C	2.30	1.47	1.41
14	aA	1108	CLA	CHD-C4C	2.30	1.47	1.41
18	AA	4008	BCR	C17-C18	-2.30	1.32	1.35
14	BA	1233	CLA	CHD-C4C	2.30	1.47	1.41
14	AA	1114	CLA	C1B-CHB	2.30	1.46	1.40
14	bA	1231	CLA	CHD-C4C	2.30	1.47	1.41
14	bA	1239	CLA	CHD-C4C	2.30	1.47	1.41
14	HA	1218	CLA	CHD-C4C	2.30	1.47	1.41
14	aA	1113	CLA	CHD-C4C	2.30	1.47	1.41
14	bA	1204	CLA	C1B-CHB	2.30	1.46	1.40
14	aA	1115	CLA	CHD-C4C	2.30	1.47	1.41
14	aA	1118	CLA	CHD-C4C	2.30	1.47	1.41
18	IA	4018	BCR	C21-C22	-2.30	1.32	1.35
14	lA	1503	CLA	CHD-C4C	2.29	1.47	1.41
14	bA	1233	CLA	CHD-C4C	2.29	1.47	1.41
14	aA	1116	CLA	CHD-C4C	2.29	1.47	1.41
14	BA	1210	CLA	CHD-C4C	2.29	1.47	1.41
14	BA	1218	CLA	CHD-C4C	2.29	1.47	1.41
14	aA	1114	CLA	C1B-CHB	2.29	1.46	1.40
14	GA	1119	CLA	CHD-C4C	2.29	1.47	1.41
14	aA	1119	CLA	CHD-C4C	2.29	1.47	1.41
14	BA	1235	CLA	CHD-C4C	2.29	1.47	1.41
14	GA	1135	CLA	CHD-C4C	2.29	1.47	1.41
14	LA	1503	CLA	CHD-C4C	2.29	1.47	1.41
14	bA	1209	CLA	CHD-C4C	2.29	1.47	1.41
14	HA	1228	CLA	C4C-C3C	2.29	1.49	1.45
14	HA	1209	CLA	CHD-C4C	2.29	1.47	1.41
14	bA	1218	CLA	CHD-C4C	2.29	1.47	1.41
14	HA	1235	CLA	CHD-C4C	2.29	1.47	1.41
14	aA	1117	CLA	C4B-CHC	2.28	1.46	1.40
14	AA	1119	CLA	C1B-CHB	2.28	1.46	1.40
14	AA	1132	CLA	C1B-CHB	2.28	1.46	1.40
18	BA	4005	BCR	C30-C25	-2.28	1.50	1.53
14	AA	1135	CLA	CHD-C4C	2.28	1.47	1.41
14	GA	1117	CLA	C4B-CHC	2.28	1.46	1.40
14	aA	1119	CLA	C1B-CHB	2.28	1.46	1.40
14	aA	1132	CLA	C1B-CHB	2.28	1.46	1.40
14	AA	1133	CLA	CHD-C4C	2.28	1.47	1.41
14	bA	1208	CLA	CHD-C4C	2.28	1.47	1.41
14	bA	1210	CLA	C4C-C3C	2.28	1.49	1.45
18	lA	4022	BCR	C17-C18	-2.28	1.32	1.35
14	AA	1118	CLA	CHD-C4C	2.28	1.47	1.41

Continued on next page...

PRELIMINARY VALIDATION REPORT

Continued from previous page...

Mol	Chain	Res	Type	Atoms	Z	Observed(Å)	Ideal(Å)
13	AA	1011	CL0	C1D-C2D	2.27	1.47	1.42
14	AA	1119	CLA	CHD-C4C	2.27	1.47	1.41
14	GA	1119	CLA	C1B-CHB	2.27	1.46	1.40
14	AA	1126	CLA	CHD-C4C	2.27	1.47	1.41
18	bA	4005	BCR	C30-C25	-2.27	1.50	1.53
14	bA	1235	CLA	CHD-C4C	2.27	1.47	1.41
14	GA	1132	CLA	C1B-CHB	2.27	1.46	1.40
14	BA	1224	CLA	C1D-C2D	2.27	1.47	1.42
14	AA	1131	CLA	CHD-C4C	2.27	1.47	1.41
14	BA	1239	CLA	CHD-C4C	2.26	1.47	1.41
14	UA	1503	CLA	CHD-C4C	2.26	1.47	1.41
14	aA	1131	CLA	C1B-CHB	2.26	1.46	1.40
14	HA	1208	CLA	CHD-C4C	2.26	1.47	1.41
14	GA	1118	CLA	CHD-C4C	2.26	1.47	1.41
14	HA	1226	CLA	C4B-CHC	2.26	1.46	1.40
14	HA	1217	CLA	CHD-C4C	2.26	1.47	1.41
18	AA	4008	BCR	C14-C13	-2.26	1.32	1.35
14	GA	1125	CLA	C1D-C2D	2.26	1.47	1.42
14	bA	1206	CLA	C1D-C2D	2.26	1.47	1.42
14	BA	1208	CLA	CHD-C4C	2.26	1.47	1.41
14	HA	1239	CLA	CHD-C4C	2.26	1.47	1.41
14	AA	1131	CLA	C1B-CHB	2.26	1.46	1.40
14	BA	1211	CLA	CHD-C4C	2.26	1.47	1.41
14	BA	1206	CLA	C1D-C2D	2.26	1.47	1.42
18	iA	4018	BCR	C21-C22	-2.25	1.32	1.35
14	AA	1012	CLA	C1B-CHB	2.25	1.46	1.40
14	GA	1133	CLA	CHD-C4C	2.25	1.47	1.41
14	aA	1012	CLA	C1B-CHB	2.25	1.46	1.40
14	HA	1238	CLA	C1B-CHB	2.25	1.46	1.40
14	bA	1211	CLA	CHD-C4C	2.25	1.47	1.41
14	BA	1226	CLA	C4B-CHC	2.25	1.46	1.40
18	aA	4007	BCR	C1-C6	-2.25	1.50	1.53
14	GA	1131	CLA	C1B-CHB	2.25	1.46	1.40
18	aA	4008	BCR	C14-C13	-2.25	1.32	1.35
14	HA	1206	CLA	C1D-C2D	2.24	1.47	1.42
14	aA	1126	CLA	CHD-C4C	2.24	1.47	1.41
14	aA	1126	CLA	C1B-CHB	2.24	1.46	1.40
14	aA	1133	CLA	CHD-C4C	2.24	1.47	1.41
14	aA	1125	CLA	MG-NC	2.24	2.11	2.06
14	bA	1238	CLA	C1B-CHB	2.24	1.46	1.40
14	BA	1228	CLA	C4C-C3C	2.24	1.48	1.45
14	bA	1224	CLA	C1D-C2D	2.24	1.47	1.42

Continued on next page...

PRELIMINARY VALIDATION REPORT

Continued from previous page...

Mol	Chain	Res	Type	Atoms	Z	Observed(Å)	Ideal(Å)
14	bA	1023	CLA	C4B-CHC	2.24	1.46	1.40
14	GA	1012	CLA	C1B-CHB	2.24	1.46	1.40
18	AA	4007	BCR	C1-C6	-2.24	1.50	1.53
14	AA	1126	CLA	C1B-CHB	2.24	1.46	1.40
14	HA	1211	CLA	CHD-C4C	2.24	1.47	1.41
14	GA	1126	CLA	CHD-C4C	2.24	1.47	1.41
14	BA	1023	CLA	C4B-CHC	2.24	1.46	1.40
14	GA	1126	CLA	C1B-CHB	2.23	1.46	1.40
14	BA	1219	CLA	CHD-C4C	2.23	1.47	1.41
14	BA	1023	CLA	MG-NC	2.23	2.11	2.06
14	GA	1111	CLA	CHD-C4C	2.23	1.47	1.41
14	bA	1228	CLA	C4C-C3C	2.23	1.48	1.45
14	HA	1023	CLA	MG-NC	2.23	2.11	2.06
18	aA	4008	BCR	C21-C22	-2.23	1.32	1.35
14	HA	1224	CLA	C1D-C2D	2.23	1.47	1.42
14	bA	1217	CLA	CHD-C4C	2.23	1.47	1.41
14	bA	1219	CLA	CHD-C4C	2.23	1.47	1.41
14	BA	1238	CLA	C1B-CHB	2.23	1.46	1.40
14	aA	1125	CLA	C1D-C2D	2.23	1.47	1.42
14	HA	1226	CLA	C1B-CHB	2.23	1.46	1.40
14	aA	1131	CLA	CHD-C4C	2.23	1.47	1.41
14	AA	1125	CLA	MG-NC	2.23	2.11	2.06
18	GA	4008	BCR	C14-C13	-2.23	1.32	1.35
14	HA	1023	CLA	C4B-CHC	2.23	1.46	1.40
14	bA	1023	CLA	MG-NC	2.23	2.11	2.06
14	GA	1131	CLA	CHD-C4C	2.22	1.47	1.41
14	AA	1132	CLA	CHD-C4C	2.22	1.47	1.41
14	GA	1125	CLA	MG-NC	2.22	2.11	2.06
14	AA	1111	CLA	CHD-C4C	2.22	1.47	1.41
14	BA	1217	CLA	CHD-C4C	2.22	1.47	1.41
14	GA	1124	CLA	CHD-C4C	2.22	1.47	1.41
14	bA	1226	CLA	C1B-CHB	2.22	1.46	1.40
18	RA	4018	BCR	C21-C22	-2.22	1.32	1.35
18	UA	4022	BCR	C17-C18	-2.22	1.32	1.35
14	HA	1201	CLA	CHD-C4C	2.21	1.47	1.41
14	HA	1219	CLA	CHD-C4C	2.21	1.47	1.41
14	BA	1226	CLA	C1B-CHB	2.21	1.46	1.40
14	BA	1238	CLA	CHD-C4C	2.21	1.47	1.41
14	aA	1132	CLA	CHD-C4C	2.21	1.47	1.41
14	GA	1012	CLA	CHD-C4C	2.21	1.47	1.41
14	AA	1124	CLA	CHD-C4C	2.21	1.47	1.41
18	GA	4008	BCR	C21-C22	-2.21	1.32	1.35

Continued on next page...

PRELIMINARY VALIDATION REPORT

Continued from previous page...

Mol	Chain	Res	Type	Atoms	Z	Observed(Å)	Ideal(Å)
18	LA	4022	BCR	C17-C18	-2.21	1.32	1.35
14	GA	1114	CLA	C4C-C3C	2.20	1.48	1.45
14	aA	1111	CLA	CHD-C4C	2.20	1.47	1.41
14	aA	1012	CLA	CHD-C4C	2.20	1.47	1.41
14	bA	1226	CLA	C4B-CHC	2.20	1.46	1.40
18	GA	4007	BCR	C1-C6	-2.20	1.50	1.53
14	AA	1012	CLA	CHD-C4C	2.19	1.47	1.41
14	AA	1114	CLA	C4C-C3C	2.19	1.48	1.45
14	GA	1132	CLA	CHD-C4C	2.19	1.47	1.41
14	aA	1135	CLA	C4C-C3C	2.19	1.48	1.45
18	AA	4008	BCR	C21-C22	-2.19	1.32	1.35
14	GA	1133	CLA	C1A-CHA	2.19	1.52	1.43
14	HA	1238	CLA	CHD-C4C	2.19	1.47	1.41
14	BA	1201	CLA	CHD-C4C	2.19	1.47	1.41
14	bA	1238	CLA	CHD-C4C	2.19	1.47	1.41
14	AA	1125	CLA	C1D-C2D	2.19	1.47	1.42
14	AA	1106	CLA	CHD-C4C	2.19	1.47	1.41
18	RA	4018	BCR	C1-C6	-2.19	1.50	1.53
14	aA	1124	CLA	CHD-C4C	2.19	1.47	1.41
14	bA	1236	CLA	CHD-C4C	2.19	1.47	1.41
18	IA	4018	BCR	C1-C6	-2.19	1.50	1.53
14	AA	1133	CLA	C1A-CHA	2.18	1.52	1.43
14	bA	1201	CLA	CHD-C4C	2.18	1.47	1.41
14	HA	1236	CLA	CHD-C4C	2.18	1.47	1.41
14	HA	1223	CLA	C1D-C2D	2.18	1.47	1.42
14	BA	1236	CLA	CHD-C4C	2.18	1.47	1.41
14	AA	1130	CLA	CHD-C4C	2.18	1.47	1.41
14	BA	1239	CLA	C4C-C3C	2.18	1.48	1.45
14	TA	1401	CLA	C4C-C3C	2.18	1.48	1.45
14	AA	1128	CLA	CHD-C4C	2.18	1.47	1.41
14	aA	1133	CLA	C1A-CHA	2.18	1.52	1.43
14	AA	1135	CLA	C4C-C3C	2.18	1.48	1.45
14	AA	1013	CLA	MG-NC	2.18	2.11	2.06
18	iA	4018	BCR	C1-C6	-2.18	1.50	1.53
14	HA	1239	CLA	C4C-C3C	2.18	1.48	1.45
14	BA	1215	CLA	CHD-C4C	2.18	1.47	1.41
14	LA	1502	CLA	CHD-C4C	2.18	1.47	1.41
14	GA	1130	CLA	CHD-C4C	2.18	1.47	1.41
14	HA	1215	CLA	CHD-C4C	2.18	1.47	1.41
14	BA	1201	CLA	C1A-CHA	2.17	1.52	1.43
14	HA	1021	CLA	C3A-C2A	-2.17	1.48	1.54
14	aA	1114	CLA	C4C-C3C	2.17	1.48	1.45

Continued on next page...

PRELIMINARY VALIDATION REPORT

Continued from previous page...

Mol	Chain	Res	Type	Atoms	Z	Observed(Å)	Ideal(Å)
14	UA	1502	CLA	CHD-C4C	2.17	1.47	1.41
14	aA	1130	CLA	CHD-C4C	2.17	1.47	1.41
21	AA	6001	LMT	O4'-C4B	-2.17	1.37	1.43
14	AA	1127	CLA	C1D-C2D	2.17	1.47	1.42
18	IA	4020	BCR	C1-C6	-2.17	1.50	1.53
14	GA	1106	CLA	CHD-C4C	2.17	1.47	1.41
14	GA	1128	CLA	CHD-C4C	2.17	1.47	1.41
14	lA	1502	CLA	CHD-C4C	2.17	1.47	1.41
14	BA	1223	CLA	C1D-C2D	2.17	1.47	1.42
14	aA	1013	CLA	MG-NC	2.16	2.11	2.06
14	kA	1401	CLA	C4C-C3C	2.16	1.48	1.45
14	KA	1401	CLA	C4C-C3C	2.16	1.48	1.45
14	bA	1215	CLA	CHD-C4C	2.16	1.47	1.41
14	aA	1128	CLA	CHD-C4C	2.16	1.47	1.41
14	AA	1124	CLA	C1B-CHB	2.16	1.45	1.40
21	aA	6001	LMT	O4'-C4B	-2.16	1.37	1.43
14	HA	1201	CLA	C1A-CHA	2.16	1.52	1.43
14	HA	1220	CLA	CHD-C4C	2.16	1.47	1.41
14	bA	1201	CLA	C1A-CHA	2.16	1.52	1.43
14	BA	1021	CLA	C3A-C2A	-2.16	1.48	1.54
14	AA	1104	CLA	CHD-C4C	2.16	1.47	1.41
14	bA	1223	CLA	C1D-C2D	2.16	1.47	1.42
14	bA	1023	CLA	C1D-C2D	2.15	1.47	1.42
14	GA	1104	CLA	CHD-C4C	2.15	1.47	1.41
14	BA	1220	CLA	CHD-C4C	2.15	1.47	1.41
14	AA	1136	CLA	C1A-CHA	2.15	1.52	1.43
14	GA	1127	CLA	C1D-C2D	2.15	1.47	1.42
14	AA	1139	CLA	CHD-C4C	2.15	1.47	1.41
14	aA	1106	CLA	CHD-C4C	2.15	1.47	1.41
21	GA	6001	LMT	O4'-C4B	-2.15	1.37	1.43
14	GA	1124	CLA	C1B-CHB	2.15	1.45	1.40
14	aA	1136	CLA	C1A-CHA	2.15	1.52	1.43
14	aA	1124	CLA	C1B-CHB	2.15	1.45	1.40
14	bA	1207	CLA	CHD-C4C	2.15	1.47	1.41
13	aA	1011	CL0	C4B-CHC	2.14	1.45	1.40
14	GA	1101	CLA	CHD-C4C	2.14	1.47	1.41
14	bA	1223	CLA	C1A-CHA	2.14	1.52	1.43
13	AA	1011	CL0	C4B-CHC	2.14	1.45	1.40
14	GA	1013	CLA	MG-NC	2.14	2.11	2.06
14	AA	1137	CLA	CHD-C4C	2.14	1.47	1.41
14	GA	1135	CLA	C4C-C3C	2.14	1.48	1.45
14	BA	1223	CLA	C1A-CHA	2.14	1.52	1.43

Continued on next page...

PRELIMINARY VALIDATION REPORT

Continued from previous page...

Mol	Chain	Res	Type	Atoms	Z	Observed(Å)	Ideal(Å)
14	BA	1212	CLA	CHD-C4C	2.14	1.47	1.41
14	aA	1137	CLA	CHD-C4C	2.14	1.47	1.41
14	HA	1223	CLA	C1A-CHA	2.14	1.52	1.43
18	lA	4022	BCR	C21-C22	-2.14	1.32	1.35
13	GA	1011	CL0	C4B-CHC	2.14	1.45	1.40
14	bA	1212	CLA	CHD-C4C	2.14	1.47	1.41
14	HA	1207	CLA	CHD-C4C	2.14	1.47	1.41
14	aA	1139	CLA	CHD-C4C	2.14	1.47	1.41
14	aA	1104	CLA	CHD-C4C	2.14	1.47	1.41
14	aA	1101	CLA	CHD-C4C	2.13	1.47	1.41
14	GA	1136	CLA	C1A-CHA	2.13	1.52	1.43
14	bA	1021	CLA	C3A-C2A	-2.13	1.48	1.54
14	GA	1139	CLA	CHD-C4C	2.13	1.47	1.41
14	AA	1103	CLA	CHD-C4C	2.13	1.47	1.41
14	bA	1239	CLA	C4C-C3C	2.13	1.48	1.45
14	BA	1207	CLA	CHD-C4C	2.12	1.47	1.41
14	aA	1127	CLA	C1D-C2D	2.12	1.47	1.42
14	AA	1101	CLA	CHD-C4C	2.12	1.47	1.41
14	aA	1129	CLA	CHD-C4C	2.12	1.47	1.41
14	bA	1220	CLA	CHD-C4C	2.12	1.47	1.41
14	GA	1137	CLA	CHD-C4C	2.12	1.47	1.41
14	LA	1501	CLA	C1A-CHA	2.12	1.51	1.43
14	AA	1131	CLA	C1A-CHA	2.12	1.51	1.43
14	HA	1232	CLA	C1A-CHA	2.12	1.51	1.43
14	GA	1112	CLA	C1A-CHA	2.11	1.51	1.43
14	GA	1103	CLA	CHD-C4C	2.11	1.47	1.41
14	BA	1219	CLA	C1A-CHA	2.11	1.51	1.43
18	RA	4020	BCR	C1-C6	-2.11	1.50	1.53
14	GA	1131	CLA	C1A-CHA	2.11	1.51	1.43
14	HA	1212	CLA	CHD-C4C	2.11	1.47	1.41
14	bA	1232	CLA	C1A-CHA	2.11	1.51	1.43
14	AA	1110	CLA	CHD-C4C	2.11	1.47	1.41
14	BA	1023	CLA	C1D-C2D	2.11	1.47	1.42
14	HA	1219	CLA	C1A-CHA	2.11	1.51	1.43
21	aA	6001	LMT	O1'-C1'	-2.11	1.36	1.40
14	HA	1023	CLA	C1D-C2D	2.11	1.47	1.42
14	HA	1205	CLA	C1D-C2D	2.10	1.47	1.42
14	aA	1103	CLA	CHD-C4C	2.10	1.47	1.41
14	BA	1232	CLA	C1A-CHA	2.10	1.51	1.43
14	aA	1131	CLA	C1A-CHA	2.10	1.51	1.43
14	kA	1401	CLA	C1A-CHA	2.10	1.51	1.43
14	lA	1501	CLA	C1A-CHA	2.10	1.51	1.43

Continued on next page...

PRELIMINARY VALIDATION REPORT

Continued from previous page...

Mol	Chain	Res	Type	Atoms	Z	Observed(Å)	Ideal(Å)
14	KA	1401	CLA	C1A-CHA	2.10	1.51	1.43
14	lA	1501	CLA	CHD-C4C	2.10	1.47	1.41
14	aA	1112	CLA	C1A-CHA	2.10	1.51	1.43
14	GA	1110	CLA	CHD-C4C	2.10	1.47	1.41
14	bA	1219	CLA	C1A-CHA	2.10	1.51	1.43
14	GA	1129	CLA	CHD-C4C	2.10	1.47	1.41
14	BA	1205	CLA	C1D-C2D	2.10	1.47	1.42
14	UA	1501	CLA	C1A-CHA	2.10	1.51	1.43
14	aA	1127	CLA	CHD-C4C	2.10	1.47	1.41
14	GA	1127	CLA	CHD-C4C	2.10	1.47	1.41
14	AA	1127	CLA	CHD-C4C	2.10	1.47	1.41
14	AA	1112	CLA	C1A-CHA	2.10	1.51	1.43
14	bA	1202	CLA	CHD-C4C	2.09	1.47	1.41
14	BA	1216	CLA	C4C-C3C	2.09	1.48	1.45
14	AA	1117	CLA	C1A-CHA	2.09	1.51	1.43
14	HA	1216	CLA	C4C-C3C	2.09	1.48	1.45
14	aA	1122	CLA	CHD-C4C	2.09	1.47	1.41
14	bA	1205	CLA	C1D-C2D	2.09	1.47	1.42
14	AA	1129	CLA	CHD-C4C	2.09	1.47	1.41
14	UA	1501	CLA	CHD-C4C	2.09	1.47	1.41
14	bA	1216	CLA	C4C-C3C	2.09	1.48	1.45
14	BA	1233	CLA	C4C-C3C	2.09	1.48	1.45
14	bA	1205	CLA	C1A-CHA	2.09	1.51	1.43
14	bA	1023	CLA	C1A-CHA	2.09	1.51	1.43
14	HA	1205	CLA	C1A-CHA	2.08	1.51	1.43
14	AA	1122	CLA	CHD-C4C	2.08	1.47	1.41
14	GA	1122	CLA	C1A-CHA	2.08	1.51	1.43
14	TA	1401	CLA	C1A-CHA	2.08	1.51	1.43
18	BA	4010	BCR	C17-C18	-2.08	1.33	1.35
14	HA	1023	CLA	C1A-CHA	2.08	1.51	1.43
14	BA	1202	CLA	CHD-C4C	2.08	1.47	1.41
14	BA	1205	CLA	C1A-CHA	2.08	1.51	1.43
21	AA	6001	LMT	O1'-C1'	-2.08	1.36	1.40
14	AA	1122	CLA	C1A-CHA	2.08	1.51	1.43
18	iA	4020	BCR	C1-C6	-2.08	1.51	1.53
14	XA	1701	CLA	CHD-C4C	2.08	1.47	1.41
14	GA	1117	CLA	C1A-CHA	2.08	1.51	1.43
14	xA	1701	CLA	CHD-C4C	2.07	1.47	1.41
14	aA	1117	CLA	C1A-CHA	2.07	1.51	1.43
14	aA	1111	CLA	C4C-C3C	2.07	1.48	1.45
21	GA	6001	LMT	O1'-C1'	-2.07	1.36	1.40
14	aA	1110	CLA	CHD-C4C	2.07	1.47	1.41

Continued on next page...

PRELIMINARY VALIDATION REPORT

Continued from previous page...

Mol	Chain	Res	Type	Atoms	Z	Observed(Å)	Ideal(Å)
14	BA	1023	CLA	C1A-CHA	2.07	1.51	1.43
14	HA	1202	CLA	CHD-C4C	2.07	1.47	1.41
14	0	1012	CLA	C1D-C2D	2.07	1.47	1.42
14	aA	1122	CLA	C1A-CHA	2.07	1.51	1.43
14	BA	1206	CLA	CHD-C4C	2.07	1.47	1.41
14	aA	1136	CLA	CHD-C4C	2.06	1.47	1.41
13	aA	1011	CL0	CHD-C4C	2.06	1.47	1.41
14	LA	1501	CLA	CHD-C4C	2.06	1.47	1.41
14	AA	1136	CLA	CHD-C4C	2.06	1.47	1.41
14	bA	1022	CLA	C1D-C2D	2.06	1.47	1.42
18	GA	4011	BCR	C1-C6	-2.06	1.51	1.53
14	bA	1212	CLA	C1A-CHA	2.06	1.51	1.43
14	GA	1122	CLA	CHD-C4C	2.06	1.47	1.41
14	HA	1206	CLA	CHD-C4C	2.06	1.47	1.41
18	bA	4010	BCR	C17-C18	-2.06	1.33	1.35
18	HA	4010	BCR	C17-C18	-2.06	1.33	1.35
14	aA	1107	CLA	CHD-C4C	2.06	1.47	1.41
14	HA	1238	CLA	C1D-C2D	2.05	1.47	1.42
14	GA	1107	CLA	CHD-C4C	2.05	1.47	1.41
14	GA	1013	CLA	C3A-C4A	-2.05	1.45	1.51
14	bA	1221	CLA	CHD-C4C	2.05	1.47	1.41
14	WA	1701	CLA	CHD-C4C	2.05	1.47	1.41
18	LA	4022	BCR	C21-C22	-2.05	1.33	1.35
14	bA	1214	CLA	C4C-C3C	2.05	1.48	1.45
13	GA	1011	CL0	CHD-C4C	2.05	1.47	1.41
14	BA	1212	CLA	C1A-CHA	2.05	1.51	1.43
14	aA	1123	CLA	CHD-C4C	2.05	1.47	1.41
14	AA	1013	CLA	C3A-C4A	-2.05	1.45	1.51
14	aA	1013	CLA	C3A-C4A	-2.05	1.45	1.51
14	AA	1110	CLA	C1A-CHA	2.05	1.51	1.43
14	bA	1238	CLA	C1D-C2D	2.04	1.47	1.42
14	AA	1106	CLA	C1A-CHA	2.04	1.51	1.43
14	GA	1114	CLA	C1A-CHA	2.04	1.51	1.43
14	bA	1206	CLA	CHD-C4C	2.04	1.47	1.41
14	GA	1106	CLA	C1A-CHA	2.04	1.51	1.43
14	HA	1225	CLA	C1A-CHA	2.04	1.51	1.43
14	GA	1136	CLA	CHD-C4C	2.04	1.47	1.41
14	HA	1212	CLA	C1A-CHA	2.04	1.51	1.43
14	aA	1112	CLA	C4C-C3C	2.04	1.48	1.45
14	GA	1110	CLA	C1A-CHA	2.04	1.51	1.43
14	aA	1114	CLA	C1A-CHA	2.04	1.51	1.43
13	AA	1011	CL0	CHD-C4C	2.04	1.47	1.41

Continued on next page...

PRELIMINARY VALIDATION REPORT

Continued from previous page...

Mol	Chain	Res	Type	Atoms	Z	Observed(Å)	Ideal(Å)
14	BA	1225	CLA	C1A-CHA	2.03	1.51	1.43
14	GA	1112	CLA	C4C-C3C	2.03	1.48	1.45
14	GA	1111	CLA	C1A-CHA	2.03	1.51	1.43
14	GA	1123	CLA	CHD-C4C	2.03	1.47	1.41
14	AA	1114	CLA	C1A-CHA	2.03	1.51	1.43
14	aA	1110	CLA	C1A-CHA	2.03	1.51	1.43
18	UA	4022	BCR	C21-C22	-2.03	1.33	1.35
14	HA	1217	CLA	C1A-CHA	2.03	1.51	1.43
14	BA	1221	CLA	CHD-C4C	2.03	1.47	1.41
14	AA	1109	CLA	C1A-CHA	2.03	1.51	1.43
14	BA	1022	CLA	C1D-C2D	2.03	1.47	1.42
14	AA	1140	CLA	CHD-C4C	2.03	1.47	1.41
14	GA	1140	CLA	CHD-C4C	2.03	1.47	1.41
14	HA	1233	CLA	C4C-C3C	2.03	1.48	1.45
14	AA	1111	CLA	C1A-CHA	2.03	1.51	1.43
14	aA	1111	CLA	C1A-CHA	2.03	1.51	1.43
14	AA	1123	CLA	CHD-C4C	2.02	1.47	1.41
14	HA	1221	CLA	CHD-C4C	2.02	1.47	1.41
14	aA	1109	CLA	C1A-CHA	2.02	1.51	1.43
14	GA	1111	CLA	C4C-C3C	2.02	1.48	1.45
14	bA	1225	CLA	C1A-CHA	2.02	1.51	1.43
14	BA	1217	CLA	C1A-CHA	2.02	1.51	1.43
14	AA	1107	CLA	CHD-C4C	2.02	1.47	1.41
14	BA	1214	CLA	C4C-C3C	2.02	1.48	1.45
14	bA	1217	CLA	C1A-CHA	2.02	1.51	1.43
14	aA	1106	CLA	C1A-CHA	2.02	1.51	1.43
14	bA	1209	CLA	C1A-CHA	2.02	1.51	1.43
18	IA	4018	BCR	C17-C18	-2.02	1.33	1.35
14	AA	1138	CLA	C1A-CHA	2.01	1.51	1.43
18	aA	4011	BCR	C21-C22	-2.01	1.33	1.35
18	AA	4008	BCR	C30-C25	-2.01	1.51	1.53
14	HA	1209	CLA	C1A-CHA	2.01	1.51	1.43
14	GA	1109	CLA	C1A-CHA	2.01	1.51	1.43
18	BA	4004	BCR	C30-C25	-2.01	1.51	1.53
18	AA	4011	BCR	C21-C22	-2.01	1.33	1.35
14	bA	1233	CLA	C4C-C3C	2.01	1.48	1.45
14	BA	1209	CLA	C1A-CHA	2.01	1.51	1.43
14	AA	1111	CLA	C4C-C3C	2.01	1.48	1.45
18	GA	4008	BCR	C30-C25	-2.01	1.51	1.53
14	HA	1214	CLA	C4C-C3C	2.01	1.48	1.45
14	AA	1105	CLA	C4C-C3C	2.01	1.48	1.45
14	AA	1112	CLA	C4C-C3C	2.01	1.48	1.45

Continued on next page...

PRELIMINARY VALIDATION REPORT

Continued from previous page...

Mol	Chain	Res	Type	Atoms	Z	Observed(Å)	Ideal(Å)
18	HA	4017	BCR	C21-C22	-2.00	1.33	1.35
14	bA	1239	CLA	C1A-CHA	2.00	1.51	1.43
14	aA	1138	CLA	C1A-CHA	2.00	1.51	1.43
14	GA	1138	CLA	C1A-CHA	2.00	1.51	1.43
14	HA	1239	CLA	C1A-CHA	2.00	1.51	1.43
14	HA	1206	CLA	C3A-C2A	-2.00	1.48	1.54
14	BA	1205	CLA	CHD-C4C	2.00	1.46	1.41
14	aA	1140	CLA	CHD-C4C	2.00	1.46	1.41

All (5696) bond angle outliers are listed below:

Mol	Chain	Res	Type	Atoms	Z	Observed(^{o})	Ideal(^{o})
18	HA	4010	BCR	C16-C17-C18	25.08	163.10	127.31
18	BA	4010	BCR	C16-C17-C18	25.06	163.08	127.31
18	bA	4010	BCR	C16-C17-C18	25.04	163.05	127.31
18	BA	4017	BCR	C20-C21-C22	23.50	160.85	127.31
18	bA	4017	BCR	C20-C21-C22	23.48	160.81	127.31
18	HA	4017	BCR	C20-C21-C22	23.46	160.80	127.31
18	RA	4018	BCR	C16-C17-C18	23.34	160.62	127.31
18	IA	4018	BCR	C16-C17-C18	23.33	160.61	127.31
18	iA	4018	BCR	C16-C17-C18	23.32	160.59	127.31
18	HA	4016	BCR	C16-C17-C18	22.95	160.07	127.31
18	bA	4016	BCR	C16-C17-C18	22.94	160.05	127.31
18	BA	4016	BCR	C16-C17-C18	22.93	160.04	127.31
18	AA	4007	BCR	C20-C21-C22	22.89	159.98	127.31
18	aA	4007	BCR	C20-C21-C22	22.88	159.97	127.31
18	GA	4007	BCR	C20-C21-C22	22.86	159.94	127.31
18	HA	4016	BCR	C20-C21-C22	22.26	159.08	127.31
18	BA	4016	BCR	C20-C21-C22	22.26	159.08	127.31
18	bA	4016	BCR	C20-C21-C22	22.23	159.04	127.31
18	bA	4009	BCR	C16-C17-C18	22.18	158.96	127.31
18	BA	4009	BCR	C16-C17-C18	22.16	158.93	127.31
18	HA	4009	BCR	C16-C17-C18	22.16	158.93	127.31
18	HA	4006	BCR	C20-C21-C22	22.09	158.83	127.31
18	BA	4006	BCR	C20-C21-C22	22.06	158.80	127.31
18	bA	4006	BCR	C20-C21-C22	22.06	158.79	127.31
18	AA	4001	BCR	C20-C21-C22	21.78	158.40	127.31
18	GA	4001	BCR	C20-C21-C22	21.75	158.36	127.31
18	aA	4001	BCR	C20-C21-C22	21.73	158.32	127.31
18	BA	4010	BCR	C20-C21-C22	21.55	158.06	127.31
18	HA	4010	BCR	C20-C21-C22	21.54	158.05	127.31
18	bA	4010	BCR	C20-C21-C22	21.49	157.98	127.31

Continued on next page...

PRELIMINARY VALIDATION REPORT

Continued from previous page...

Mol	Chain	Res	Type	Atoms	Z	Observed(^{o})	Ideal(^{o})
18	lA	4019	BCR	C20-C21-C22	21.40	157.85	127.31
18	LA	4019	BCR	C20-C21-C22	21.39	157.84	127.31
18	UA	4019	BCR	C20-C21-C22	21.39	157.84	127.31
18	MA	4021	BCR	C20-C21-C22	21.30	157.71	127.31
18	VA	4021	BCR	C20-C21-C22	21.26	157.66	127.31
18	mA	4021	BCR	C20-C21-C22	21.26	157.65	127.31
18	aA	4003	BCR	C20-C21-C22	21.22	157.59	127.31
18	AA	4003	BCR	C20-C21-C22	21.21	157.59	127.31
18	GA	4003	BCR	C20-C21-C22	21.21	157.58	127.31
18	AA	4007	BCR	C16-C17-C18	21.18	157.54	127.31
18	aA	4007	BCR	C16-C17-C18	21.15	157.49	127.31
18	GA	4007	BCR	C16-C17-C18	21.11	157.43	127.31
18	BA	4012	BCR	C20-C21-C22	21.01	157.29	127.31
18	HA	4014	BCR	C15-C16-C17	21.01	167.65	123.51
18	bA	4014	BCR	C15-C16-C17	21.01	167.65	123.51
18	HA	4012	BCR	C20-C21-C22	20.97	157.24	127.31
18	BA	4014	BCR	C15-C16-C17	20.96	167.56	123.51
18	bA	4012	BCR	C20-C21-C22	20.96	157.22	127.31
18	BA	4005	BCR	C20-C21-C22	20.94	157.19	127.31
18	HA	4005	BCR	C20-C21-C22	20.92	157.16	127.31
18	GA	4001	BCR	C15-C16-C17	20.90	167.41	123.51
18	bA	4005	BCR	C20-C21-C22	20.88	157.11	127.31
18	AA	4001	BCR	C15-C16-C17	20.87	167.36	123.51
18	aA	4001	BCR	C15-C16-C17	20.84	167.31	123.51
18	HA	4013	BCR	C16-C17-C18	20.84	157.06	127.31
18	bA	4013	BCR	C16-C17-C18	20.83	157.03	127.31
18	BA	4013	BCR	C20-C21-C22	20.82	157.02	127.31
18	BA	4013	BCR	C16-C17-C18	20.80	157.00	127.31
18	bA	4013	BCR	C20-C21-C22	20.77	156.96	127.31
18	HA	4013	BCR	C20-C21-C22	20.77	156.96	127.31
18	GA	4011	BCR	C16-C17-C18	20.76	156.93	127.31
18	aA	4011	BCR	C16-C17-C18	20.72	156.88	127.31
18	AA	4011	BCR	C16-C17-C18	20.69	156.84	127.31
18	BA	4012	BCR	C16-C17-C18	20.63	156.75	127.31
18	HA	4012	BCR	C16-C17-C18	20.61	156.72	127.31
18	bA	4012	BCR	C16-C17-C18	20.59	156.69	127.31
18	HA	4012	BCR	C15-C16-C17	20.57	166.72	123.51
18	GA	4003	BCR	C16-C17-C18	20.55	156.64	127.31
18	BA	4012	BCR	C15-C16-C17	20.54	166.67	123.51
18	aA	4003	BCR	C16-C17-C18	20.51	156.58	127.31
18	bA	4012	BCR	C15-C16-C17	20.51	166.61	123.51
18	BA	4017	BCR	C16-C17-C18	20.50	156.56	127.31

Continued on next page...

PRELIMINARY VALIDATION REPORT

Continued from previous page...

Mol	Chain	Res	Type	Atoms	Z	Observed(°)	Ideal(°)
18	HA	4017	BCR	C16-C17-C18	20.49	156.55	127.31
18	RA	4020	BCR	C20-C21-C22	20.49	156.55	127.31
18	IA	4020	BCR	C20-C21-C22	20.48	156.54	127.31
18	iA	4020	BCR	C20-C21-C22	20.47	156.52	127.31
18	bA	4017	BCR	C16-C17-C18	20.47	156.52	127.31
18	bA	4014	BCR	C20-C21-C22	20.46	156.52	127.31
18	AA	4003	BCR	C16-C17-C18	20.45	156.50	127.31
18	BA	4014	BCR	C20-C21-C22	20.42	156.45	127.31
18	HA	4014	BCR	C20-C21-C22	20.40	156.43	127.31
18	aA	4002	BCR	C15-C16-C17	20.40	166.37	123.51
18	AA	4002	BCR	C15-C16-C17	20.40	166.37	123.51
18	GA	4002	BCR	C15-C16-C17	20.39	166.35	123.51
18	bA	4009	BCR	C20-C21-C22	20.25	156.22	127.31
18	HA	4009	BCR	C20-C21-C22	20.25	156.21	127.31
18	BA	4009	BCR	C20-C21-C22	20.23	156.18	127.31
18	lA	4022	BCR	C20-C21-C22	20.22	156.17	127.31
18	LA	4022	BCR	C20-C21-C22	20.19	156.13	127.31
18	GA	4002	BCR	C20-C21-C22	20.19	156.12	127.31
18	UA	4022	BCR	C20-C21-C22	20.19	156.12	127.31
18	AA	4002	BCR	C20-C21-C22	20.18	156.10	127.31
18	aA	4002	BCR	C20-C21-C22	20.15	156.07	127.31
18	LA	4019	BCR	C15-C16-C17	20.14	165.82	123.51
18	UA	4019	BCR	C15-C16-C17	20.14	165.82	123.51
18	lA	4019	BCR	C15-C16-C17	20.13	165.81	123.51
18	MA	4021	BCR	C15-C16-C17	20.12	165.78	123.51
18	bA	4005	BCR	C16-C17-C18	20.12	156.02	127.31
18	mA	4021	BCR	C15-C16-C17	20.11	165.77	123.51
18	HA	4005	BCR	C16-C17-C18	20.09	155.98	127.31
18	VA	4021	BCR	C15-C16-C17	20.08	165.70	123.51
18	BA	4005	BCR	C16-C17-C18	20.07	155.95	127.31
18	bA	4006	BCR	C16-C17-C18	20.00	155.86	127.31
18	BA	4006	BCR	C16-C17-C18	19.97	155.81	127.31
18	HA	4006	BCR	C16-C17-C18	19.96	155.80	127.31
18	bA	4004	BCR	C15-C16-C17	19.96	165.45	123.51
18	BA	4004	BCR	C15-C16-C17	19.96	165.44	123.51
18	HA	4005	BCR	C15-C16-C17	19.93	165.40	123.51
18	HA	4004	BCR	C15-C16-C17	19.93	165.39	123.51
18	BA	4005	BCR	C15-C16-C17	19.92	165.36	123.51
18	bA	4005	BCR	C15-C16-C17	19.92	165.36	123.51
18	GA	4008	BCR	C20-C21-C22	19.80	155.57	127.31
18	AA	4008	BCR	C20-C21-C22	19.79	155.55	127.31
18	aA	4011	BCR	C15-C16-C17	19.77	165.06	123.51

Continued on next page...

PRELIMINARY VALIDATION REPORT

Continued from previous page...

Mol	Chain	Res	Type	Atoms	Z	Observed(°)	Ideal(°)
18	aA	4008	BCR	C20-C21-C22	19.76	155.51	127.31
18	GA	4011	BCR	C15-C16-C17	19.76	165.03	123.51
18	RA	4020	BCR	C16-C17-C18	19.75	155.50	127.31
18	AA	4011	BCR	C15-C16-C17	19.74	164.98	123.51
18	IA	4020	BCR	C16-C17-C18	19.73	155.47	127.31
18	iA	4020	BCR	C16-C17-C18	19.72	155.46	127.31
18	LA	4022	BCR	C15-C16-C17	19.68	164.86	123.51
18	UA	4022	BCR	C15-C16-C17	19.68	164.85	123.51
18	lA	4022	BCR	C15-C16-C17	19.67	164.84	123.51
18	HA	4014	BCR	C16-C17-C18	19.52	155.17	127.31
18	bA	4014	BCR	C16-C17-C18	19.51	155.15	127.31
18	BA	4014	BCR	C16-C17-C18	19.48	155.11	127.31
18	GA	4008	BCR	C15-C16-C17	19.47	164.42	123.51
18	AA	4008	BCR	C15-C16-C17	19.46	164.40	123.51
18	aA	4008	BCR	C15-C16-C17	19.45	164.38	123.51
18	IA	4020	BCR	C15-C16-C17	19.34	164.16	123.51
18	RA	4020	BCR	C15-C16-C17	19.34	164.14	123.51
18	iA	4020	BCR	C15-C16-C17	19.31	164.09	123.51
18	lA	4022	BCR	C16-C17-C18	19.22	154.74	127.31
18	bA	4006	BCR	C15-C16-C17	19.21	163.88	123.51
18	HA	4004	BCR	C20-C21-C22	19.21	154.72	127.31
18	LA	4022	BCR	C16-C17-C18	19.21	154.72	127.31
18	UA	4022	BCR	C16-C17-C18	19.20	154.71	127.31
18	HA	4006	BCR	C15-C16-C17	19.20	163.84	123.51
18	bA	4004	BCR	C20-C21-C22	19.19	154.70	127.31
18	BA	4006	BCR	C15-C16-C17	19.18	163.81	123.51
18	BA	4004	BCR	C20-C21-C22	19.17	154.67	127.31
18	iA	4018	BCR	C20-C21-C22	19.13	154.61	127.31
18	BA	4017	BCR	C15-C16-C17	19.10	163.64	123.51
18	IA	4018	BCR	C20-C21-C22	19.10	154.56	127.31
18	RA	4018	BCR	C20-C21-C22	19.09	154.55	127.31
18	HA	4009	BCR	C15-C16-C17	19.08	163.61	123.51
18	bA	4009	BCR	C15-C16-C17	19.08	163.60	123.51
18	HA	4017	BCR	C15-C16-C17	19.08	163.59	123.51
18	BA	4009	BCR	C15-C16-C17	19.07	163.58	123.51
18	bA	4017	BCR	C15-C16-C17	19.06	163.55	123.51
18	GA	4008	BCR	C16-C17-C18	18.98	154.40	127.31
18	AA	4008	BCR	C16-C17-C18	18.97	154.38	127.31
18	HA	4004	BCR	C16-C17-C18	18.95	154.35	127.31
18	aA	4008	BCR	C16-C17-C18	18.95	154.35	127.31
18	BA	4004	BCR	C16-C17-C18	18.94	154.34	127.31
18	bA	4004	BCR	C16-C17-C18	18.94	154.34	127.31

Continued on next page...

PRELIMINARY VALIDATION REPORT

Continued from previous page...

Mol	Chain	Res	Type	Atoms	Z	Observed(°)	Ideal(°)
18	lA	4019	BCR	C16-C17-C18	18.89	154.28	127.31
18	AA	4011	BCR	C20-C21-C22	18.88	154.26	127.31
18	GA	4011	BCR	C20-C21-C22	18.88	154.26	127.31
18	aA	4011	BCR	C20-C21-C22	18.87	154.24	127.31
18	LA	4019	BCR	C16-C17-C18	18.86	154.23	127.31
18	UA	4019	BCR	C16-C17-C18	18.83	154.19	127.31
18	GA	4003	BCR	C15-C16-C17	18.80	163.00	123.51
18	aA	4003	BCR	C15-C16-C17	18.78	162.97	123.51
18	AA	4003	BCR	C15-C16-C17	18.72	162.85	123.51
18	RA	4018	BCR	C10-C11-C12	18.69	179.53	123.31
18	iA	4018	BCR	C10-C11-C12	18.68	179.49	123.31
18	IA	4018	BCR	C10-C11-C12	18.66	179.43	123.31
18	mA	4021	BCR	C10-C11-C12	18.57	179.14	123.31
18	AA	4007	BCR	C15-C16-C17	18.56	162.51	123.51
18	MA	4021	BCR	C10-C11-C12	18.56	179.13	123.31
18	VA	4021	BCR	C10-C11-C12	18.55	179.09	123.31
18	aA	4007	BCR	C15-C16-C17	18.53	162.45	123.51
18	GA	4007	BCR	C15-C16-C17	18.52	162.42	123.51
18	IA	4018	BCR	C15-C16-C17	18.49	162.36	123.51
18	HA	4006	BCR	C10-C11-C12	18.49	178.91	123.31
18	bA	4006	BCR	C10-C11-C12	18.48	178.88	123.31
18	RA	4018	BCR	C15-C16-C17	18.47	162.31	123.51
18	iA	4018	BCR	C15-C16-C17	18.46	162.31	123.51
18	BA	4006	BCR	C10-C11-C12	18.45	178.79	123.31
18	BA	4017	BCR	C10-C11-C12	18.18	177.97	123.31
18	bA	4017	BCR	C10-C11-C12	18.15	177.90	123.31
18	HA	4017	BCR	C10-C11-C12	18.15	177.90	123.31
18	HA	4004	BCR	C10-C11-C12	18.11	177.78	123.31
18	AA	4003	BCR	C10-C11-C12	18.10	177.76	123.31
18	GA	4003	BCR	C10-C11-C12	18.10	177.75	123.31
18	BA	4004	BCR	C10-C11-C12	18.09	177.71	123.31
18	aA	4003	BCR	C10-C11-C12	18.08	177.69	123.31
18	bA	4004	BCR	C10-C11-C12	18.08	177.69	123.31
18	AA	4002	BCR	C16-C17-C18	18.05	153.07	127.31
18	aA	4002	BCR	C16-C17-C18	18.05	153.07	127.31
18	HA	4013	BCR	C15-C16-C17	18.02	161.38	123.51
18	GA	4002	BCR	C16-C17-C18	18.02	153.03	127.31
18	bA	4013	BCR	C15-C16-C17	18.01	161.36	123.51
18	BA	4013	BCR	C15-C16-C17	18.01	161.36	123.51
18	lA	4022	BCR	C10-C11-C12	17.89	177.12	123.31
18	LA	4022	BCR	C10-C11-C12	17.89	177.12	123.31
18	bA	4013	BCR	C10-C11-C12	17.88	177.07	123.31

Continued on next page...

PRELIMINARY VALIDATION REPORT

Continued from previous page...

Mol	Chain	Res	Type	Atoms	Z	Observed(°)	Ideal(°)
18	UA	4022	BCR	C10-C11-C12	17.88	177.07	123.31
18	HA	4013	BCR	C10-C11-C12	17.87	177.05	123.31
18	bA	4016	BCR	C10-C11-C12	17.86	177.03	123.31
18	BA	4016	BCR	C10-C11-C12	17.85	177.00	123.31
18	BA	4013	BCR	C10-C11-C12	17.85	176.99	123.31
18	HA	4016	BCR	C10-C11-C12	17.85	176.98	123.31
18	MA	4021	BCR	C16-C17-C18	17.81	152.73	127.31
18	BA	4009	BCR	C10-C11-C12	17.79	176.82	123.31
18	bA	4009	BCR	C10-C11-C12	17.79	176.81	123.31
18	mA	4021	BCR	C16-C17-C18	17.78	152.69	127.31
18	HA	4009	BCR	C10-C11-C12	17.78	176.78	123.31
18	AA	4008	BCR	C10-C11-C12	17.76	176.73	123.31
18	GA	4008	BCR	C10-C11-C12	17.75	176.70	123.31
18	GA	4007	BCR	C10-C11-C12	17.75	176.69	123.31
18	aA	4007	BCR	C10-C11-C12	17.74	176.67	123.31
18	aA	4008	BCR	C10-C11-C12	17.74	176.65	123.31
18	AA	4007	BCR	C10-C11-C12	17.73	176.64	123.31
18	VA	4021	BCR	C16-C17-C18	17.73	152.61	127.31
18	aA	4001	BCR	C10-C11-C12	17.73	176.62	123.31
18	GA	4001	BCR	C10-C11-C12	17.72	176.60	123.31
18	AA	4001	BCR	C10-C11-C12	17.71	176.56	123.31
18	HA	4012	BCR	C10-C11-C12	17.70	176.53	123.31
18	bA	4012	BCR	C10-C11-C12	17.69	176.52	123.31
18	LA	4019	BCR	C10-C11-C12	17.69	176.52	123.31
18	BA	4012	BCR	C10-C11-C12	17.69	176.50	123.31
18	lA	4019	BCR	C10-C11-C12	17.66	176.41	123.31
18	UA	4019	BCR	C10-C11-C12	17.65	176.40	123.31
18	HA	4014	BCR	C10-C11-C12	17.56	176.12	123.31
18	bA	4014	BCR	C10-C11-C12	17.54	176.05	123.31
18	BA	4014	BCR	C10-C11-C12	17.54	176.05	123.31
18	bA	4010	BCR	C10-C11-C12	17.50	175.93	123.31
18	BA	4010	BCR	C10-C11-C12	17.49	175.90	123.31
18	HA	4010	BCR	C10-C11-C12	17.47	175.86	123.31
18	BA	4016	BCR	C15-C16-C17	17.30	159.86	123.51
18	HA	4016	BCR	C15-C16-C17	17.29	159.85	123.51
18	bA	4016	BCR	C15-C16-C17	17.29	159.83	123.51
18	IA	4020	BCR	C10-C11-C12	17.28	175.28	123.31
18	RA	4020	BCR	C10-C11-C12	17.27	175.25	123.31
18	iA	4020	BCR	C10-C11-C12	17.26	175.23	123.31
18	bA	4005	BCR	C10-C11-C12	17.15	174.90	123.31
18	GA	4001	BCR	C16-C17-C18	17.15	151.79	127.31
18	BA	4005	BCR	C10-C11-C12	17.15	174.88	123.31

Continued on next page...

PRELIMINARY VALIDATION REPORT

Continued from previous page...

Mol	Chain	Res	Type	Atoms	Z	Observed(°)	Ideal(°)
18	AA	4001	BCR	C16-C17-C18	17.13	151.76	127.31
18	HA	4005	BCR	C10-C11-C12	17.13	174.82	123.31
18	aA	4001	BCR	C16-C17-C18	17.09	151.69	127.31
18	GA	4011	BCR	C10-C11-C12	16.63	173.31	123.31
18	aA	4011	BCR	C10-C11-C12	16.62	173.30	123.31
18	AA	4011	BCR	C10-C11-C12	16.62	173.30	123.31
18	AA	4002	BCR	C10-C11-C12	16.37	172.55	123.31
18	GA	4002	BCR	C10-C11-C12	16.37	172.55	123.31
18	aA	4002	BCR	C10-C11-C12	16.36	172.52	123.31
18	HA	4010	BCR	C15-C16-C17	16.21	157.56	123.51
18	bA	4010	BCR	C15-C16-C17	16.20	157.55	123.51
18	BA	4010	BCR	C15-C16-C17	16.18	157.51	123.51
18	VA	4021	BCR	C16-C15-C14	15.86	156.83	123.51
18	MA	4021	BCR	C16-C15-C14	15.82	156.75	123.51
18	mA	4021	BCR	C16-C15-C14	15.82	156.74	123.51
18	aA	4011	BCR	C11-C10-C9	15.61	149.58	127.31
18	GA	4011	BCR	C11-C10-C9	15.57	149.53	127.31
18	AA	4011	BCR	C11-C10-C9	15.57	149.53	127.31
18	bA	4005	BCR	C11-C10-C9	14.68	148.26	127.31
18	BA	4005	BCR	C11-C10-C9	14.64	148.20	127.31
18	HA	4005	BCR	C11-C10-C9	14.63	148.20	127.31
18	iA	4018	BCR	C16-C15-C14	14.58	154.14	123.51
18	RA	4018	BCR	C16-C15-C14	14.56	154.10	123.51
18	IA	4018	BCR	C16-C15-C14	14.54	154.07	123.51
18	HA	4004	BCR	C21-C20-C19	14.33	166.41	123.31
18	bA	4004	BCR	C21-C20-C19	14.31	166.34	123.31
18	BA	4004	BCR	C21-C20-C19	14.31	166.33	123.31
18	HA	4013	BCR	C16-C15-C14	14.24	153.43	123.51
18	bA	4013	BCR	C16-C15-C14	14.24	153.43	123.51
18	BA	4013	BCR	C16-C15-C14	14.24	153.42	123.51
18	GA	4011	BCR	C21-C20-C19	14.17	165.93	123.31
18	aA	4011	BCR	C21-C20-C19	14.16	165.89	123.31
18	AA	4011	BCR	C21-C20-C19	14.16	165.88	123.31
18	HA	4012	BCR	C11-C10-C9	14.15	147.51	127.31
18	bA	4012	BCR	C11-C10-C9	14.13	147.47	127.31
18	BA	4012	BCR	C11-C10-C9	14.08	147.41	127.31
18	AA	4008	BCR	C21-C20-C19	13.96	165.29	123.31
18	GA	4008	BCR	C21-C20-C19	13.96	165.29	123.31
18	aA	4008	BCR	C21-C20-C19	13.95	165.25	123.31
18	bA	4014	BCR	C21-C20-C19	13.95	165.25	123.31
18	HA	4014	BCR	C21-C20-C19	13.94	165.22	123.31
18	BA	4014	BCR	C21-C20-C19	13.92	165.19	123.31

Continued on next page...

PRELIMINARY VALIDATION REPORT

Continued from previous page...

Mol	Chain	Res	Type	Atoms	Z	Observed(°)	Ideal(°)
18	bA	4013	BCR	C11-C10-C9	13.85	147.08	127.31
18	bA	4016	BCR	C16-C15-C14	13.82	152.55	123.51
18	BA	4016	BCR	C16-C15-C14	13.81	152.53	123.51
18	HA	4013	BCR	C11-C10-C9	13.81	147.01	127.31
18	BA	4013	BCR	C11-C10-C9	13.80	147.01	127.31
18	HA	4016	BCR	C16-C15-C14	13.80	152.50	123.51
18	lA	4022	BCR	C21-C20-C19	13.75	164.67	123.31
18	LA	4022	BCR	C21-C20-C19	13.74	164.64	123.31
18	UA	4022	BCR	C21-C20-C19	13.73	164.61	123.31
18	AA	4002	BCR	C21-C20-C19	13.73	164.60	123.31
18	GA	4002	BCR	C21-C20-C19	13.73	164.59	123.31
18	aA	4002	BCR	C21-C20-C19	13.71	164.55	123.31
18	iA	4018	BCR	C11-C10-C9	13.66	146.81	127.31
18	IA	4018	BCR	C11-C10-C9	13.65	146.79	127.31
18	RA	4018	BCR	C11-C10-C9	13.62	146.75	127.31
18	HA	4010	BCR	C21-C20-C19	13.54	164.04	123.31
18	bA	4010	BCR	C21-C20-C19	13.54	164.03	123.31
18	BA	4010	BCR	C21-C20-C19	13.53	164.01	123.31
18	LA	4022	BCR	C11-C10-C9	13.44	146.49	127.31
18	lA	4022	BCR	C11-C10-C9	13.42	146.47	127.31
18	UA	4022	BCR	C11-C10-C9	13.37	146.40	127.31
18	BA	4010	BCR	C16-C15-C14	13.36	151.58	123.51
18	HA	4010	BCR	C16-C15-C14	13.33	151.52	123.51
18	bA	4010	BCR	C16-C15-C14	13.33	151.51	123.51
18	aA	4001	BCR	C21-C20-C19	13.31	163.34	123.31
18	GA	4001	BCR	C21-C20-C19	13.30	163.32	123.31
18	AA	4001	BCR	C21-C20-C19	13.30	163.32	123.31
18	HA	4009	BCR	C21-C20-C19	13.21	163.02	123.31
18	BA	4009	BCR	C21-C20-C19	13.20	163.01	123.31
18	bA	4009	BCR	C21-C20-C19	13.20	163.00	123.31
18	bA	4009	BCR	C16-C15-C14	13.12	151.08	123.51
18	GA	4003	BCR	C16-C15-C14	13.12	151.08	123.51
18	BA	4009	BCR	C16-C15-C14	13.12	151.07	123.51
18	aA	4003	BCR	C16-C15-C14	13.12	151.07	123.51
18	HA	4009	BCR	C16-C15-C14	13.11	151.06	123.51
18	AA	4003	BCR	C16-C15-C14	13.08	150.99	123.51
18	BA	4014	BCR	C11-C10-C9	13.05	145.93	127.31
18	HA	4014	BCR	C11-C10-C9	13.04	145.92	127.31
18	HA	4006	BCR	C16-C15-C14	13.02	150.86	123.51
18	bA	4006	BCR	C16-C15-C14	13.02	150.86	123.51
18	BA	4006	BCR	C16-C15-C14	13.01	150.84	123.51
18	HA	4004	BCR	C11-C10-C9	13.01	145.87	127.31

Continued on next page...

PRELIMINARY VALIDATION REPORT

Continued from previous page...

Mol	Chain	Res	Type	Atoms	Z	Observed(°)	Ideal(°)
18	bA	4014	BCR	C11-C10-C9	13.00	145.86	127.31
18	bA	4004	BCR	C11-C10-C9	12.98	145.84	127.31
18	GA	4007	BCR	C11-C10-C9	12.97	145.82	127.31
18	BA	4004	BCR	C11-C10-C9	12.96	145.81	127.31
18	aA	4007	BCR	C11-C10-C9	12.95	145.79	127.31
18	AA	4007	BCR	C11-C10-C9	12.92	145.75	127.31
18	AA	4007	BCR	C16-C15-C14	12.87	150.55	123.51
18	HA	4017	BCR	C11-C10-C9	12.87	145.67	127.31
18	aA	4007	BCR	C16-C15-C14	12.85	150.52	123.51
18	GA	4007	BCR	C16-C15-C14	12.84	150.50	123.51
18	bA	4016	BCR	C21-C20-C19	12.84	161.93	123.31
18	HA	4016	BCR	C21-C20-C19	12.84	161.92	123.31
18	BA	4016	BCR	C21-C20-C19	12.84	161.91	123.31
18	BA	4017	BCR	C11-C10-C9	12.84	145.63	127.31
18	bA	4017	BCR	C11-C10-C9	12.81	145.59	127.31
18	LA	4019	BCR	C11-C10-C9	12.74	145.49	127.31
18	lA	4019	BCR	C11-C10-C9	12.72	145.46	127.31
18	aA	4001	BCR	C11-C10-C9	12.71	145.45	127.31
18	AA	4001	BCR	C11-C10-C9	12.69	145.42	127.31
18	UA	4019	BCR	C11-C10-C9	12.69	145.41	127.31
18	GA	4001	BCR	C11-C10-C9	12.69	145.41	127.31
18	lA	4019	BCR	C21-C20-C19	12.66	161.38	123.31
18	UA	4019	BCR	C21-C20-C19	12.65	161.35	123.31
18	LA	4019	BCR	C21-C20-C19	12.65	161.34	123.31
18	HA	4006	BCR	C21-C20-C19	12.62	161.25	123.31
18	bA	4006	BCR	C21-C20-C19	12.61	161.22	123.31
18	BA	4006	BCR	C21-C20-C19	12.60	161.20	123.31
18	HA	4017	BCR	C16-C15-C14	12.58	149.95	123.51
18	HA	4005	BCR	C21-C20-C19	12.58	161.13	123.31
18	BA	4005	BCR	C21-C20-C19	12.57	161.12	123.31
18	bA	4005	BCR	C21-C20-C19	12.56	161.08	123.31
18	VA	4021	BCR	C21-C20-C19	12.56	161.07	123.31
18	mA	4021	BCR	C21-C20-C19	12.56	161.07	123.31
18	BA	4017	BCR	C16-C15-C14	12.55	149.89	123.51
18	MA	4021	BCR	C21-C20-C19	12.55	161.06	123.31
18	AA	4003	BCR	C11-C10-C9	12.53	145.20	127.31
18	bA	4017	BCR	C16-C15-C14	12.53	149.84	123.51
18	aA	4003	BCR	C11-C10-C9	12.52	145.18	127.31
18	AA	4008	BCR	C16-C15-C14	12.52	149.81	123.51
18	GA	4003	BCR	C11-C10-C9	12.52	145.17	127.31
18	GA	4008	BCR	C16-C15-C14	12.50	149.78	123.51
18	HA	4017	BCR	C21-C20-C19	12.50	160.90	123.31

Continued on next page...

PRELIMINARY VALIDATION REPORT

Continued from previous page...

Mol	Chain	Res	Type	Atoms	Z	Observed(°)	Ideal(°)
18	aA	4008	BCR	C16-C15-C14	12.49	149.75	123.51
18	BA	4017	BCR	C21-C20-C19	12.48	160.84	123.31
18	BA	4012	BCR	C21-C20-C19	12.47	160.80	123.31
18	bA	4017	BCR	C21-C20-C19	12.47	160.80	123.31
18	bA	4012	BCR	C21-C20-C19	12.46	160.77	123.31
18	HA	4012	BCR	C21-C20-C19	12.43	160.70	123.31
18	IA	4020	BCR	C11-C12-C13	12.41	161.28	126.42
18	iA	4020	BCR	C11-C12-C13	12.41	161.28	126.42
18	bA	4004	BCR	C16-C15-C14	12.41	149.58	123.51
18	RA	4020	BCR	C11-C12-C13	12.41	161.27	126.42
18	BA	4004	BCR	C16-C15-C14	12.40	149.57	123.51
18	HA	4004	BCR	C16-C15-C14	12.40	149.57	123.51
18	HA	4013	BCR	C21-C20-C19	12.39	160.56	123.31
18	BA	4013	BCR	C21-C20-C19	12.37	160.51	123.31
18	bA	4013	BCR	C21-C20-C19	12.37	160.51	123.31
18	AA	4007	BCR	C21-C20-C19	12.32	160.35	123.31
18	GA	4007	BCR	C21-C20-C19	12.31	160.33	123.31
18	aA	4007	BCR	C21-C20-C19	12.30	160.31	123.31
18	bA	4009	BCR	C11-C10-C9	12.04	144.50	127.31
18	BA	4005	BCR	C16-C15-C14	12.04	148.81	123.51
18	BA	4009	BCR	C11-C10-C9	12.04	144.50	127.31
18	HA	4005	BCR	C16-C15-C14	12.03	148.79	123.51
18	bA	4005	BCR	C16-C15-C14	12.03	148.78	123.51
18	IA	4018	BCR	C21-C20-C19	11.99	159.36	123.31
18	iA	4018	BCR	C21-C20-C19	11.98	159.34	123.31
18	UA	4019	BCR	C16-C15-C14	11.98	148.68	123.51
18	LA	4019	BCR	C16-C15-C14	11.98	148.67	123.51
18	RA	4018	BCR	C21-C20-C19	11.97	159.31	123.31
18	HA	4009	BCR	C11-C10-C9	11.97	144.40	127.31
18	lA	4019	BCR	C16-C15-C14	11.97	148.65	123.51
18	aA	4003	BCR	C21-C20-C19	11.80	158.79	123.31
18	AA	4003	BCR	C21-C20-C19	11.80	158.79	123.31
18	GA	4003	BCR	C21-C20-C19	11.80	158.78	123.31
18	GA	4002	BCR	C16-C15-C14	11.75	148.21	123.51
18	AA	4002	BCR	C16-C15-C14	11.75	148.19	123.51
18	aA	4002	BCR	C16-C15-C14	11.75	148.19	123.51
18	UA	4022	BCR	C16-C15-C14	11.60	147.88	123.51
18	LA	4022	BCR	C16-C15-C14	11.59	147.87	123.51
18	lA	4022	BCR	C16-C15-C14	11.58	147.85	123.51
18	AA	4008	BCR	C11-C10-C9	11.43	143.62	127.31
18	aA	4011	BCR	C16-C15-C14	11.40	147.46	123.51
18	HA	4006	BCR	C11-C10-C9	11.39	143.56	127.31

Continued on next page...

PRELIMINARY VALIDATION REPORT

Continued from previous page...

Mol	Chain	Res	Type	Atoms	Z	Observed(°)	Ideal(°)
18	GA	4008	BCR	C11-C10-C9	11.38	143.56	127.31
18	GA	4011	BCR	C16-C15-C14	11.38	147.42	123.51
18	AA	4011	BCR	C16-C15-C14	11.38	147.42	123.51
18	bA	4006	BCR	C11-C10-C9	11.37	143.54	127.31
18	aA	4008	BCR	C11-C10-C9	11.36	143.52	127.31
18	BA	4005	BCR	C11-C12-C13	11.36	158.32	126.42
18	bA	4005	BCR	C11-C12-C13	11.36	158.31	126.42
18	HA	4005	BCR	C11-C12-C13	11.33	158.24	126.42
18	BA	4006	BCR	C11-C10-C9	11.32	143.47	127.31
18	HA	4012	BCR	C16-C15-C14	11.30	147.25	123.51
18	BA	4012	BCR	C16-C15-C14	11.27	147.19	123.51
18	iA	4020	BCR	C16-C15-C14	11.24	147.14	123.51
18	bA	4012	BCR	C16-C15-C14	11.24	147.14	123.51
18	RA	4020	BCR	C16-C15-C14	11.24	147.13	123.51
18	IA	4020	BCR	C16-C15-C14	11.23	147.11	123.51
18	GA	4001	BCR	C16-C15-C14	11.16	146.96	123.51
18	AA	4001	BCR	C16-C15-C14	11.14	146.92	123.51
18	aA	4001	BCR	C16-C15-C14	11.14	146.91	123.51
18	RA	4020	BCR	C21-C20-C19	11.13	156.78	123.31
18	iA	4020	BCR	C21-C20-C19	11.13	156.78	123.31
18	IA	4020	BCR	C21-C20-C19	11.11	156.72	123.31
18	LA	4019	BCR	C11-C12-C13	11.11	157.62	126.42
18	GA	4001	BCR	C11-C12-C13	11.10	157.59	126.42
18	aA	4001	BCR	C11-C12-C13	11.08	157.54	126.42
18	lA	4019	BCR	C11-C12-C13	11.08	157.53	126.42
18	AA	4001	BCR	C11-C12-C13	11.07	157.50	126.42
18	UA	4019	BCR	C11-C12-C13	11.06	157.49	126.42
18	GA	4008	BCR	C11-C12-C13	11.01	157.35	126.42
18	AA	4008	BCR	C11-C12-C13	11.00	157.33	126.42
18	aA	4008	BCR	C11-C12-C13	10.99	157.30	126.42
18	GA	4011	BCR	C11-C12-C13	10.90	157.03	126.42
18	AA	4011	BCR	C11-C12-C13	10.89	157.01	126.42
18	aA	4011	BCR	C11-C12-C13	10.89	157.01	126.42
18	AA	4007	BCR	C11-C12-C13	10.81	156.78	126.42
18	aA	4007	BCR	C11-C12-C13	10.81	156.77	126.42
18	GA	4007	BCR	C11-C12-C13	10.80	156.75	126.42
18	bA	4014	BCR	C16-C15-C14	10.78	146.17	123.51
18	HA	4014	BCR	C16-C15-C14	10.77	146.14	123.51
18	bA	4016	BCR	C11-C10-C9	10.75	142.65	127.31
18	BA	4014	BCR	C16-C15-C14	10.74	146.08	123.51
18	HA	4016	BCR	C11-C10-C9	10.73	142.62	127.31
18	BA	4016	BCR	C11-C10-C9	10.73	142.62	127.31

Continued on next page...

PRELIMINARY VALIDATION REPORT

Continued from previous page...

Mol	Chain	Res	Type	Atoms	Z	Observed(^{o})	Ideal(^{o})
18	lA	4022	BCR	C11-C12-C13	10.68	156.41	126.42
18	LA	4022	BCR	C11-C12-C13	10.66	156.38	126.42
18	UA	4022	BCR	C11-C12-C13	10.66	156.35	126.42
18	bA	4010	BCR	C11-C10-C9	10.64	142.49	127.31
18	BA	4010	BCR	C11-C10-C9	10.62	142.47	127.31
18	bA	4012	BCR	C11-C12-C13	10.62	156.25	126.42
18	HA	4012	BCR	C11-C12-C13	10.61	156.23	126.42
18	MA	4021	BCR	C11-C10-C9	10.61	142.45	127.31
18	mA	4021	BCR	C11-C10-C9	10.61	142.45	127.31
18	BA	4012	BCR	C11-C12-C13	10.61	156.21	126.42
18	HA	4004	BCR	C11-C12-C13	10.59	156.18	126.42
18	VA	4021	BCR	C11-C10-C9	10.59	142.43	127.31
18	HA	4010	BCR	C11-C10-C9	10.58	142.41	127.31
18	bA	4004	BCR	C11-C12-C13	10.58	156.15	126.42
18	BA	4004	BCR	C11-C12-C13	10.58	156.14	126.42
18	MA	4021	BCR	C11-C12-C13	10.56	156.08	126.42
18	VA	4021	BCR	C11-C12-C13	10.55	156.06	126.42
18	HA	4006	BCR	C11-C12-C13	10.55	156.05	126.42
18	mA	4021	BCR	C11-C12-C13	10.55	156.05	126.42
18	bA	4006	BCR	C11-C12-C13	10.53	156.01	126.42
18	BA	4006	BCR	C11-C12-C13	10.53	155.99	126.42
18	HA	4010	BCR	C11-C12-C13	10.14	154.91	126.42
18	bA	4010	BCR	C11-C12-C13	10.13	154.87	126.42
18	BA	4010	BCR	C11-C12-C13	10.13	154.87	126.42
18	BA	4014	BCR	C11-C12-C13	10.09	154.75	126.42
18	bA	4014	BCR	C11-C12-C13	10.08	154.73	126.42
18	HA	4014	BCR	C11-C12-C13	10.07	154.72	126.42
18	AA	4002	BCR	C11-C12-C13	10.03	154.59	126.42
18	GA	4002	BCR	C11-C12-C13	10.02	154.57	126.42
18	aA	4002	BCR	C11-C12-C13	10.01	154.55	126.42
18	RA	4020	BCR	C11-C10-C9	9.87	141.40	127.31
18	IA	4020	BCR	C11-C10-C9	9.85	141.37	127.31
18	iA	4020	BCR	C11-C10-C9	9.83	141.33	127.31
18	AA	4003	BCR	C11-C12-C13	9.80	153.94	126.42
18	aA	4003	BCR	C11-C12-C13	9.79	153.92	126.42
18	GA	4003	BCR	C11-C12-C13	9.79	153.92	126.42
18	bA	4013	BCR	C11-C12-C13	9.79	153.91	126.42
18	HA	4013	BCR	C11-C12-C13	9.78	153.88	126.42
18	BA	4013	BCR	C11-C12-C13	9.78	153.88	126.42
18	BA	4017	BCR	C11-C12-C13	9.66	153.56	126.42
18	HA	4017	BCR	C11-C12-C13	9.65	153.52	126.42
18	bA	4017	BCR	C11-C12-C13	9.63	153.47	126.42

Continued on next page...

PRELIMINARY VALIDATION REPORT

Continued from previous page...

Mol	Chain	Res	Type	Atoms	Z	Observed(^{o})	Ideal(^{o})
18	bA	4009	BCR	C11-C12-C13	9.49	153.09	126.42
18	HA	4009	BCR	C11-C12-C13	9.49	153.07	126.42
18	BA	4009	BCR	C11-C12-C13	9.47	153.03	126.42
18	RA	4018	BCR	C11-C12-C13	9.37	152.74	126.42
18	iA	4018	BCR	C11-C12-C13	9.36	152.72	126.42
18	IA	4018	BCR	C11-C12-C13	9.35	152.68	126.42
18	bA	4016	BCR	C11-C12-C13	8.95	151.54	126.42
18	BA	4016	BCR	C11-C12-C13	8.94	151.53	126.42
18	HA	4016	BCR	C11-C12-C13	8.93	151.50	126.42
14	HA	1238	CLA	CAA-C2A-C3A	-8.86	88.52	112.78
14	bA	1238	CLA	CAA-C2A-C3A	-8.86	88.52	112.78
14	BA	1238	CLA	CAA-C2A-C3A	-8.86	88.52	112.78
18	LA	4019	BCR	C24-C23-C22	-8.57	113.34	126.21
18	UA	4019	BCR	C24-C23-C22	-8.56	113.35	126.21
18	lA	4019	BCR	C24-C23-C22	-8.55	113.36	126.21
14	HA	1023	CLA	C4A-NA-C1A	8.41	110.49	106.71
14	BA	1023	CLA	C4A-NA-C1A	8.41	110.49	106.71
14	bA	1023	CLA	C4A-NA-C1A	8.39	110.48	106.71
18	HA	4012	BCR	C20-C19-C18	8.36	149.91	126.42
18	bA	4012	BCR	C20-C19-C18	8.36	149.90	126.42
18	BA	4012	BCR	C20-C19-C18	8.35	149.87	126.42
18	GA	4007	BCR	C20-C19-C18	8.34	149.86	126.42
18	AA	4007	BCR	C20-C19-C18	8.34	149.85	126.42
18	BA	4006	BCR	C20-C19-C18	8.34	149.83	126.42
14	bA	1239	CLA	C2C-C1C-NC	8.33	117.78	109.97
18	HA	4006	BCR	C20-C19-C18	8.33	149.82	126.42
18	bA	4006	BCR	C20-C19-C18	8.33	149.81	126.42
18	aA	4007	BCR	C20-C19-C18	8.32	149.78	126.42
14	HA	1239	CLA	C2C-C1C-NC	8.31	117.75	109.97
14	BA	1239	CLA	C2C-C1C-NC	8.30	117.75	109.97
18	HA	4013	BCR	C20-C19-C18	8.27	149.65	126.42
18	bA	4013	BCR	C20-C19-C18	8.27	149.64	126.42
18	BA	4013	BCR	C20-C19-C18	8.26	149.61	126.42
18	iA	4018	BCR	C20-C19-C18	8.24	149.56	126.42
18	IA	4018	BCR	C20-C19-C18	8.24	149.56	126.42
18	RA	4018	BCR	C20-C19-C18	8.23	149.52	126.42
14	BA	1023	CLA	CAC-C3C-C4C	7.95	135.21	124.82
14	bA	1023	CLA	CAC-C3C-C4C	7.94	135.19	124.82
14	HA	1023	CLA	CAC-C3C-C4C	7.91	135.16	124.82
18	HA	4005	BCR	C20-C19-C18	7.80	148.31	126.42
18	bA	4005	BCR	C20-C19-C18	7.79	148.30	126.42
18	BA	4005	BCR	C20-C19-C18	7.78	148.27	126.42

Continued on next page...

PRELIMINARY VALIDATION REPORT

Continued from previous page...

Mol	Chain	Res	Type	Atoms	Z	Observed(°)	Ideal(°)
18	bA	4017	BCR	C20-C19-C18	7.71	148.07	126.42
18	BA	4017	BCR	C20-C19-C18	7.71	148.07	126.42
18	HA	4017	BCR	C20-C19-C18	7.70	148.04	126.42
14	bA	1207	CLA	C2C-C1C-NC	7.65	117.14	109.97
18	aA	4003	BCR	C20-C19-C18	7.63	147.86	126.42
14	HA	1207	CLA	C2C-C1C-NC	7.63	117.12	109.97
18	GA	4003	BCR	C20-C19-C18	7.63	147.85	126.42
14	BA	1207	CLA	C2C-C1C-NC	7.61	117.10	109.97
18	AA	4003	BCR	C20-C19-C18	7.61	147.78	126.42
18	lA	4019	BCR	C20-C19-C18	7.56	147.66	126.42
14	GA	1137	CLA	O2D-CGD-CBD	7.56	124.48	111.25
18	UA	4019	BCR	C20-C19-C18	7.55	147.63	126.42
18	LA	4019	BCR	C20-C19-C18	7.55	147.62	126.42
14	aA	1137	CLA	O2D-CGD-CBD	7.54	124.45	111.25
14	AA	1137	CLA	O2D-CGD-CBD	7.52	124.42	111.25
14	aA	1132	CLA	O2D-CGD-CBD	7.44	124.27	111.25
14	AA	1110	CLA	O2D-CGD-CBD	7.43	124.25	111.25
14	GA	1132	CLA	O2D-CGD-CBD	7.41	124.22	111.25
14	AA	1132	CLA	O2D-CGD-CBD	7.41	124.22	111.25
14	GA	1110	CLA	O2D-CGD-CBD	7.41	124.21	111.25
14	aA	1110	CLA	O2D-CGD-CBD	7.40	124.21	111.25
14	HA	1214	CLA	C2C-C1C-NC	7.36	116.86	109.97
14	BA	1214	CLA	C2C-C1C-NC	7.35	116.86	109.97
14	aA	1114	CLA	C2C-C1C-NC	7.35	116.86	109.97
14	bA	1214	CLA	C2C-C1C-NC	7.35	116.85	109.97
14	AA	1114	CLA	C2C-C1C-NC	7.33	116.84	109.97
14	GA	1114	CLA	C2C-C1C-NC	7.32	116.83	109.97
14	bA	1201	CLA	C4A-NA-C1A	7.32	110.00	106.71
13	aA	1011	CL0	C2C-C1C-NC	7.31	116.82	109.97
13	GA	1011	CL0	C2C-C1C-NC	7.31	116.82	109.97
13	AA	1011	CL0	C2C-C1C-NC	7.29	116.81	109.97
14	HA	1201	CLA	C4A-NA-C1A	7.27	109.97	106.71
18	bA	4016	BCR	C20-C19-C18	7.26	146.80	126.42
18	HA	4016	BCR	C20-C19-C18	7.25	146.78	126.42
18	BA	4016	BCR	C20-C19-C18	7.24	146.76	126.42
14	bA	1212	CLA	O2D-CGD-CBD	7.24	123.91	111.25
14	BA	1201	CLA	C4A-NA-C1A	7.22	109.95	106.71
14	BA	1215	CLA	C2C-C1C-NC	7.21	116.72	109.97
14	HA	1212	CLA	O2D-CGD-CBD	7.20	123.86	111.25
14	BA	1212	CLA	O2D-CGD-CBD	7.20	123.85	111.25
14	AA	1129	CLA	O2D-CGD-CBD	7.19	123.84	111.25
14	GA	1129	CLA	O2D-CGD-CBD	7.19	123.83	111.25

Continued on next page...

PRELIMINARY VALIDATION REPORT

Continued from previous page...

Mol	Chain	Res	Type	Atoms	Z	Observed(°)	Ideal(°)
14	aA	1129	CLA	O2D-CGD-CBD	7.18	123.82	111.25
14	GA	1013	CLA	O2A-C1-C2	7.18	127.51	108.64
14	bA	1021	CLA	C4A-NA-C1A	7.18	109.93	106.71
14	HA	1215	CLA	C2C-C1C-NC	7.18	116.70	109.97
14	TA	1401	CLA	C2C-C1C-NC	7.17	116.69	109.97
14	GA	1133	CLA	C2C-C1C-NC	7.17	116.69	109.97
14	aA	1137	CLA	C2C-C1C-NC	7.17	116.69	109.97
14	bA	1215	CLA	C2C-C1C-NC	7.16	116.68	109.97
14	aA	1013	CLA	O2A-C1-C2	7.16	127.46	108.64
14	AA	1013	CLA	O2A-C1-C2	7.16	127.46	108.64
14	KA	1401	CLA	C2C-C1C-NC	7.16	116.68	109.97
14	AA	1137	CLA	C2C-C1C-NC	7.16	116.67	109.97
14	kA	1401	CLA	C2C-C1C-NC	7.15	116.67	109.97
14	HA	1021	CLA	C4A-NA-C1A	7.15	109.92	106.71
18	MA	4021	BCR	C20-C19-C18	7.14	146.48	126.42
18	VA	4021	BCR	C20-C19-C18	7.13	146.44	126.42
18	mA	4021	BCR	C20-C19-C18	7.12	146.43	126.42
14	AA	1133	CLA	C2C-C1C-NC	7.11	116.64	109.97
14	GA	1137	CLA	C2C-C1C-NC	7.11	116.63	109.97
14	aA	1133	CLA	C2C-C1C-NC	7.10	116.63	109.97
14	GA	1118	CLA	C2C-C1C-NC	7.09	116.62	109.97
14	AA	1118	CLA	C2C-C1C-NC	7.09	116.61	109.97
14	HA	1205	CLA	C4A-NA-C1A	7.07	109.89	106.71
14	BA	1226	CLA	C2C-C1C-NC	7.07	116.60	109.97
14	GA	1135	CLA	C2C-C1C-NC	7.07	116.59	109.97
14	HA	1226	CLA	C2C-C1C-NC	7.06	116.59	109.97
14	bA	1216	CLA	C2C-C1C-NC	7.06	116.59	109.97
14	AA	1123	CLA	C2C-C1C-NC	7.06	116.58	109.97
14	BA	1021	CLA	C4A-NA-C1A	7.05	109.88	106.71
14	aA	1123	CLA	C2C-C1C-NC	7.05	116.58	109.97
14	bA	1226	CLA	C2C-C1C-NC	7.05	116.58	109.97
14	AA	1135	CLA	C2C-C1C-NC	7.05	116.57	109.97
14	GA	1123	CLA	C2C-C1C-NC	7.04	116.56	109.97
14	BA	1232	CLA	C2C-C1C-NC	7.03	116.56	109.97
14	BA	1205	CLA	C4A-NA-C1A	7.03	109.86	106.71
14	HA	1232	CLA	C2C-C1C-NC	7.02	116.55	109.97
14	aA	1118	CLA	C2C-C1C-NC	7.02	116.55	109.97
14	bA	1205	CLA	O2A-C1-C2	7.02	127.07	108.64
14	HA	1223	CLA	C2C-C1C-NC	7.01	116.54	109.97
14	AA	1106	CLA	O2D-CGD-CBD	7.01	123.52	111.25
14	GA	1106	CLA	O2D-CGD-CBD	7.01	123.51	111.25
14	bA	1205	CLA	C4A-NA-C1A	7.00	109.86	106.71

Continued on next page...

PRELIMINARY VALIDATION REPORT

Continued from previous page...

Mol	Chain	Res	Type	Atoms	Z	Observed(^{o})	Ideal(^{o})
14	HA	1206	CLA	C2C-C1C-NC	7.00	116.53	109.97
14	BA	1205	CLA	O2A-C1-C2	7.00	127.03	108.64
14	bA	1232	CLA	C2C-C1C-NC	7.00	116.53	109.97
14	aA	1106	CLA	O2D-CGD-CBD	7.00	123.49	111.25
14	HA	1216	CLA	C2C-C1C-NC	6.99	116.52	109.97
14	aA	1135	CLA	C2C-C1C-NC	6.98	116.51	109.97
14	BA	1217	CLA	C2C-C1C-NC	6.98	116.51	109.97
14	HA	1217	CLA	C2C-C1C-NC	6.98	116.51	109.97
14	HA	1205	CLA	O2A-C1-C2	6.98	126.97	108.64
14	BA	1206	CLA	C2C-C1C-NC	6.98	116.51	109.97
14	BA	1223	CLA	C2C-C1C-NC	6.97	116.50	109.97
14	BA	1216	CLA	C2C-C1C-NC	6.97	116.50	109.97
14	bA	1206	CLA	C2C-C1C-NC	6.96	116.49	109.97
14	bA	1223	CLA	C2C-C1C-NC	6.96	116.49	109.97
14	bA	1217	CLA	C2C-C1C-NC	6.93	116.47	109.97
14	GA	1128	CLA	C2C-C1C-NC	6.92	116.45	109.97
14	AA	1128	CLA	C2C-C1C-NC	6.89	116.43	109.97
14	AA	1117	CLA	C2C-C1C-NC	6.88	116.42	109.97
14	bA	1233	CLA	C2C-C1C-NC	6.87	116.40	109.97
14	aA	1128	CLA	C2C-C1C-NC	6.86	116.40	109.97
14	bA	1227	CLA	C2C-C1C-NC	6.86	116.40	109.97
14	BA	1233	CLA	C2C-C1C-NC	6.86	116.40	109.97
14	GA	1117	CLA	C2C-C1C-NC	6.86	116.40	109.97
14	HA	1227	CLA	C2C-C1C-NC	6.86	116.39	109.97
14	HA	1205	CLA	O2D-CGD-CBD	6.85	123.24	111.25
14	HA	1233	CLA	C2C-C1C-NC	6.83	116.37	109.97
14	BA	1227	CLA	C2C-C1C-NC	6.83	116.37	109.97
14	aA	1117	CLA	C2C-C1C-NC	6.82	116.36	109.97
14	aA	1125	CLA	O2D-CGD-CBD	6.82	123.18	111.25
14	AA	1107	CLA	C2C-C1C-NC	6.81	116.35	109.97
14	BA	1205	CLA	O2D-CGD-CBD	6.81	123.17	111.25
14	bA	1205	CLA	O2D-CGD-CBD	6.81	123.17	111.25
18	BA	4009	BCR	C20-C19-C18	6.81	145.54	126.42
14	AA	1125	CLA	O2D-CGD-CBD	6.80	123.15	111.25
14	aA	1107	CLA	C2C-C1C-NC	6.79	116.34	109.97
18	bA	4009	BCR	C20-C19-C18	6.79	145.50	126.42
14	aA	1127	CLA	C2C-C1C-NC	6.79	116.33	109.97
14	GA	1127	CLA	C2C-C1C-NC	6.79	116.33	109.97
18	HA	4009	BCR	C20-C19-C18	6.78	145.47	126.42
14	GA	1125	CLA	O2D-CGD-CBD	6.78	123.11	111.25
14	BA	1208	CLA	C2C-C1C-NC	6.78	116.32	109.97
14	GA	1107	CLA	C2C-C1C-NC	6.77	116.32	109.97

Continued on next page...

PRELIMINARY VALIDATION REPORT

Continued from previous page...

Mol	Chain	Res	Type	Atoms	Z	Observed(^{o})	Ideal(^{o})
14	HA	1208	CLA	C2C-C1C-NC	6.77	116.31	109.97
14	bA	1208	CLA	C2C-C1C-NC	6.76	116.30	109.97
14	0	1012	CLA	O2D-CGD-CBD	6.75	123.07	111.25
14	AA	1127	CLA	C2C-C1C-NC	6.75	116.30	109.97
14	bA	1204	CLA	C2C-C1C-NC	6.75	116.30	109.97
14	bA	1022	CLA	O2D-CGD-CBD	6.75	123.06	111.25
14	GA	1132	CLA	C2C-C1C-NC	6.72	116.27	109.97
14	HA	1204	CLA	C2C-C1C-NC	6.72	116.26	109.97
14	AA	1132	CLA	C2C-C1C-NC	6.71	116.26	109.97
14	aA	1132	CLA	C2C-C1C-NC	6.71	116.26	109.97
14	HA	1218	CLA	C2C-C1C-NC	6.70	116.25	109.97
14	GA	1116	CLA	C2C-C1C-NC	6.69	116.24	109.97
14	GA	1138	CLA	O2D-CGD-CBD	6.68	122.95	111.25
14	BA	1022	CLA	O2D-CGD-CBD	6.68	122.95	111.25
14	AA	1140	CLA	C2C-C1C-NC	6.68	116.23	109.97
13	GA	1011	CL0	C4A-NA-C1A	6.68	109.71	106.71
14	AA	1138	CLA	O2D-CGD-CBD	6.67	122.93	111.25
14	aA	1138	CLA	O2D-CGD-CBD	6.67	122.93	111.25
14	aA	1012	CLA	C2C-C1C-NC	6.67	116.22	109.97
14	AA	1116	CLA	C2C-C1C-NC	6.67	116.22	109.97
14	BA	1228	CLA	C2C-C1C-NC	6.67	116.22	109.97
14	aA	1116	CLA	C2C-C1C-NC	6.67	116.22	109.97
14	BA	1204	CLA	C2C-C1C-NC	6.67	116.22	109.97
14	BA	1218	CLA	C2C-C1C-NC	6.66	116.22	109.97
14	GA	1140	CLA	C2C-C1C-NC	6.66	116.21	109.97
14	bA	1202	CLA	C2C-C1C-NC	6.66	116.21	109.97
14	GA	1012	CLA	C2C-C1C-NC	6.66	116.21	109.97
14	aA	1140	CLA	C2C-C1C-NC	6.65	116.20	109.97
14	bA	1220	CLA	C2C-C1C-NC	6.65	116.20	109.97
14	UA	1502	CLA	O2D-CGD-CBD	6.64	122.88	111.25
14	LA	1501	CLA	O2A-C1-C2	6.64	126.08	108.64
14	aA	1113	CLA	C2C-C1C-NC	6.64	116.19	109.97
14	LA	1502	CLA	O2D-CGD-CBD	6.63	122.86	111.25
14	AA	1012	CLA	C2C-C1C-NC	6.63	116.19	109.97
14	GA	1126	CLA	C2C-C1C-NC	6.63	116.18	109.97
18	LA	4022	BCR	C7-C8-C9	-6.62	116.26	126.21
14	GA	1113	CLA	C2C-C1C-NC	6.62	116.18	109.97
14	UA	1501	CLA	O2A-C1-C2	6.62	126.03	108.64
14	GA	1133	CLA	C4A-NA-C1A	6.62	109.68	106.71
14	aA	1112	CLA	C2C-C1C-NC	6.62	116.17	109.97
14	AA	1126	CLA	C2C-C1C-NC	6.62	116.17	109.97
14	bA	1228	CLA	C2C-C1C-NC	6.62	116.17	109.97

Continued on next page...

PRELIMINARY VALIDATION REPORT

Continued from previous page...

Mol	Chain	Res	Type	Atoms	Z	Observed(°)	Ideal(°)
14	AA	1112	CLA	C2C-C1C-NC	6.62	116.17	109.97
14	BA	1220	CLA	C2C-C1C-NC	6.62	116.17	109.97
14	HA	1220	CLA	C2C-C1C-NC	6.61	116.17	109.97
14	AA	1113	CLA	C2C-C1C-NC	6.61	116.17	109.97
14	GA	1112	CLA	C2C-C1C-NC	6.61	116.17	109.97
14	UA	1503	CLA	C2C-C1C-NC	6.61	116.17	109.97
18	GA	4001	BCR	C7-C8-C9	-6.61	116.28	126.21
14	bA	1218	CLA	C2C-C1C-NC	6.61	116.16	109.97
14	AA	1131	CLA	C2C-C1C-NC	6.61	116.16	109.97
18	UA	4022	BCR	C7-C8-C9	-6.60	116.29	126.21
14	HA	1202	CLA	C2C-C1C-NC	6.60	116.16	109.97
14	lA	1502	CLA	O2D-CGD-CBD	6.60	122.81	111.25
14	BA	1202	CLA	C2C-C1C-NC	6.60	116.16	109.97
14	lA	1501	CLA	O2A-C1-C2	6.60	125.99	108.64
14	GA	1131	CLA	C2C-C1C-NC	6.60	116.15	109.97
18	aA	4001	BCR	C7-C8-C9	-6.60	116.30	126.21
14	bA	1236	CLA	O2D-CGD-CBD	6.60	122.80	111.25
18	BA	4004	BCR	C24-C23-C22	-6.60	116.30	126.21
14	aA	1126	CLA	C2C-C1C-NC	6.59	116.15	109.97
14	HA	1210	CLA	C2C-C1C-NC	6.59	116.14	109.97
14	BA	1210	CLA	C2C-C1C-NC	6.59	116.14	109.97
14	HA	1228	CLA	C2C-C1C-NC	6.59	116.14	109.97
18	lA	4022	BCR	C7-C8-C9	-6.58	116.32	126.21
14	bA	1210	CLA	C2C-C1C-NC	6.58	116.14	109.97
14	lA	1503	CLA	C2C-C1C-NC	6.58	116.14	109.97
14	HA	1236	CLA	C2C-C1C-NC	6.57	116.13	109.97
14	BA	1236	CLA	O2D-CGD-CBD	6.57	122.75	111.25
18	bA	4004	BCR	C24-C23-C22	-6.57	116.34	126.21
18	AA	4001	BCR	C7-C8-C9	-6.57	116.34	126.21
14	GA	1106	CLA	C2C-C1C-NC	6.57	116.12	109.97
14	HA	1236	CLA	O2D-CGD-CBD	6.57	122.74	111.25
14	aA	1013	CLA	C4A-NA-C1A	6.56	109.66	106.71
14	BA	1201	CLA	C2C-C1C-NC	6.56	116.12	109.97
14	HA	1201	CLA	C2C-C1C-NC	6.56	116.12	109.97
14	BA	1223	CLA	O2D-CGD-CBD	6.56	122.73	111.25
14	aA	1106	CLA	C2C-C1C-NC	6.56	116.12	109.97
14	aA	1131	CLA	C2C-C1C-NC	6.56	116.12	109.97
14	GA	1013	CLA	C4A-NA-C1A	6.56	109.65	106.71
14	bA	1201	CLA	C2C-C1C-NC	6.56	116.11	109.97
14	AA	1102	CLA	C2C-C1C-NC	6.55	116.11	109.97
18	HA	4004	BCR	C24-C23-C22	-6.55	116.37	126.21
14	AA	1106	CLA	C2C-C1C-NC	6.55	116.11	109.97

Continued on next page...

PRELIMINARY VALIDATION REPORT

Continued from previous page...

Mol	Chain	Res	Type	Atoms	Z	Observed(°)	Ideal(°)
14	bA	1236	CLA	C2C-C1C-NC	6.55	116.11	109.97
14	bA	1209	CLA	C2C-C1C-NC	6.55	116.11	109.97
14	HA	1223	CLA	O2D-CGD-CBD	6.55	122.72	111.25
14	AA	1133	CLA	C4A-NA-C1A	6.55	109.65	106.71
13	aA	1011	CL0	C4A-NA-C1A	6.55	109.65	106.71
14	LA	1503	CLA	C2C-C1C-NC	6.55	116.10	109.97
18	RA	4020	BCR	C34-C9-C10	-6.54	113.76	122.92
14	AA	1101	CLA	O2D-CGD-CBD	6.54	122.69	111.25
14	GA	1101	CLA	O2D-CGD-CBD	6.54	122.69	111.25
14	bA	1223	CLA	O2D-CGD-CBD	6.54	122.69	111.25
13	AA	1011	CL0	C4A-NA-C1A	6.53	109.64	106.71
14	HA	1209	CLA	C2C-C1C-NC	6.53	116.09	109.97
14	BA	1236	CLA	C2C-C1C-NC	6.53	116.09	109.97
14	GA	1102	CLA	C2C-C1C-NC	6.53	116.09	109.97
14	GA	1109	CLA	C2C-C1C-NC	6.53	116.09	109.97
14	aA	1101	CLA	O2D-CGD-CBD	6.53	122.67	111.25
14	BA	1202	CLA	O2D-CGD-CBD	6.52	122.65	111.25
14	GA	1111	CLA	C2C-C1C-NC	6.52	116.08	109.97
14	aA	1102	CLA	C2C-C1C-NC	6.52	116.08	109.97
14	BA	1209	CLA	C2C-C1C-NC	6.52	116.08	109.97
14	aA	1133	CLA	C4A-NA-C1A	6.51	109.64	106.71
14	AA	1013	CLA	C4A-NA-C1A	6.51	109.64	106.71
18	iA	4020	BCR	C34-C9-C10	-6.51	113.80	122.92
14	AA	1110	CLA	C2C-C1C-NC	6.51	116.07	109.97
14	AA	1111	CLA	C2C-C1C-NC	6.50	116.06	109.97
14	aA	1111	CLA	C2C-C1C-NC	6.50	116.06	109.97
18	IA	4020	BCR	C34-C9-C10	-6.50	113.82	122.92
14	LA	1502	CLA	C2C-C1C-NC	6.50	116.06	109.97
14	UA	1502	CLA	C2C-C1C-NC	6.49	116.06	109.97
14	AA	1103	CLA	C4A-NA-C1A	6.49	109.62	106.71
14	aA	1110	CLA	C2C-C1C-NC	6.48	116.04	109.97
14	HA	1202	CLA	O2D-CGD-CBD	6.48	122.59	111.25
14	aA	1109	CLA	C2C-C1C-NC	6.48	116.04	109.97
14	GA	1110	CLA	C2C-C1C-NC	6.48	116.04	109.97
14	UA	1501	CLA	C2C-C1C-NC	6.48	116.04	109.97
14	AA	1109	CLA	C2C-C1C-NC	6.48	116.04	109.97
14	bA	1219	CLA	C2C-C1C-NC	6.47	116.03	109.97
14	lA	1502	CLA	C2C-C1C-NC	6.47	116.03	109.97
14	bA	1202	CLA	O2D-CGD-CBD	6.47	122.57	111.25
14	LA	1501	CLA	C2C-C1C-NC	6.45	116.01	109.97
14	aA	1103	CLA	C4A-NA-C1A	6.44	109.60	106.71
14	aA	1136	CLA	C2C-C1C-NC	6.44	116.00	109.97

Continued on next page...

PRELIMINARY VALIDATION REPORT

Continued from previous page...

Mol	Chain	Res	Type	Atoms	Z	Observed(^{o})	Ideal(^{o})
14	BA	1022	CLA	O2A-C1-C2	6.44	125.55	108.64
14	HA	1219	CLA	C2C-C1C-NC	6.44	116.00	109.97
14	0	1012	CLA	O2A-C1-C2	6.43	125.54	108.64
14	aA	1103	CLA	C2C-C1C-NC	6.43	116.00	109.97
14	bA	1022	CLA	O2A-C1-C2	6.43	125.54	108.64
14	AA	1136	CLA	C2C-C1C-NC	6.43	116.00	109.97
14	HA	1224	CLA	O2A-C1-C2	6.42	125.51	108.64
14	lA	1501	CLA	C2C-C1C-NC	6.42	115.99	109.97
14	bA	1224	CLA	O2A-C1-C2	6.42	125.51	108.64
14	AA	1103	CLA	C2C-C1C-NC	6.42	115.99	109.97
14	GA	1103	CLA	C4A-NA-C1A	6.42	109.59	106.71
14	GA	1103	CLA	C2C-C1C-NC	6.41	115.98	109.97
14	BA	1219	CLA	C2C-C1C-NC	6.41	115.98	109.97
14	BA	1224	CLA	O2A-C1-C2	6.40	125.46	108.64
14	LA	1501	CLA	O2D-CGD-CBD	6.39	122.43	111.25
14	lA	1501	CLA	O2D-CGD-CBD	6.39	122.43	111.25
14	GA	1136	CLA	O2D-CGD-CBD	6.39	122.43	111.25
14	GA	1108	CLA	C2C-C1C-NC	6.38	115.95	109.97
14	aA	1108	CLA	C2C-C1C-NC	6.38	115.95	109.97
14	GA	1127	CLA	O2A-C1-C2	6.38	125.40	108.64
14	aA	1127	CLA	O2A-C1-C2	6.38	125.40	108.64
14	AA	1136	CLA	O2D-CGD-CBD	6.37	122.41	111.25
14	UA	1501	CLA	O2D-CGD-CBD	6.37	122.41	111.25
14	AA	1127	CLA	O2A-C1-C2	6.37	125.39	108.64
14	bA	1201	CLA	O2D-CGD-CBD	6.36	122.39	111.25
18	AA	4002	BCR	C20-C19-C18	6.36	144.29	126.42
14	aA	1136	CLA	O2D-CGD-CBD	6.36	122.38	111.25
14	GA	1136	CLA	C2C-C1C-NC	6.36	115.93	109.97
14	BA	1201	CLA	O2D-CGD-CBD	6.36	122.38	111.25
18	aA	4002	BCR	C20-C19-C18	6.35	144.25	126.42
14	HA	1201	CLA	O2D-CGD-CBD	6.34	122.35	111.25
14	AA	1108	CLA	C2C-C1C-NC	6.34	115.91	109.97
18	GA	4002	BCR	C20-C19-C18	6.34	144.22	126.42
14	lA	1503	CLA	O2A-C1-C2	6.34	125.29	108.64
14	UA	1503	CLA	O2A-C1-C2	6.33	125.28	108.64
14	BA	1225	CLA	C2C-C1C-NC	6.33	115.91	109.97
14	HA	1225	CLA	C2C-C1C-NC	6.33	115.90	109.97
14	GA	1139	CLA	C2C-C1C-NC	6.32	115.89	109.97
14	LA	1503	CLA	O2A-C1-C2	6.32	125.24	108.64
14	BA	1213	CLA	C2C-C1C-NC	6.32	115.89	109.97
14	BA	1235	CLA	O2D-CGD-CBD	6.32	122.31	111.25
18	bA	4014	BCR	C20-C19-C18	6.32	144.16	126.42

Continued on next page...

PRELIMINARY VALIDATION REPORT

Continued from previous page...

Mol	Chain	Res	Type	Atoms	Z	Observed(^{o})	Ideal(^{o})
14	AA	1139	CLA	C2C-C1C-NC	6.31	115.89	109.97
14	aA	1125	CLA	C4A-NA-C1A	6.31	109.55	106.71
14	bA	1235	CLA	O2D-CGD-CBD	6.31	122.30	111.25
14	bA	1225	CLA	C2C-C1C-NC	6.31	115.88	109.97
18	HA	4014	BCR	C20-C19-C18	6.30	144.13	126.42
14	GA	1115	CLA	C2C-C1C-NC	6.30	115.88	109.97
14	HA	1213	CLA	C2C-C1C-NC	6.30	115.88	109.97
14	xA	1701	CLA	C2C-C1C-NC	6.30	115.87	109.97
14	HA	1235	CLA	O2D-CGD-CBD	6.29	122.27	111.25
14	aA	1104	CLA	C2C-C1C-NC	6.29	115.87	109.97
14	AA	1102	CLA	O2A-C1-C2	6.29	125.17	108.64
14	XA	1701	CLA	C2C-C1C-NC	6.29	115.86	109.97
14	AA	1104	CLA	C2C-C1C-NC	6.29	115.86	109.97
14	GA	1138	CLA	C2C-C1C-NC	6.29	115.86	109.97
18	BA	4014	BCR	C20-C19-C18	6.29	144.07	126.42
14	aA	1139	CLA	C2C-C1C-NC	6.29	115.86	109.97
14	bA	1213	CLA	C2C-C1C-NC	6.28	115.86	109.97
14	aA	1102	CLA	O2A-C1-C2	6.28	125.15	108.64
14	GA	1102	CLA	O2A-C1-C2	6.28	125.14	108.64
18	bA	4010	BCR	C20-C19-C18	6.27	144.04	126.42
14	AA	1138	CLA	C2C-C1C-NC	6.27	115.85	109.97
14	aA	1138	CLA	C2C-C1C-NC	6.27	115.85	109.97
14	aA	1115	CLA	C2C-C1C-NC	6.26	115.84	109.97
18	BA	4010	BCR	C20-C19-C18	6.26	144.00	126.42
14	WA	1701	CLA	C2C-C1C-NC	6.26	115.83	109.97
18	HA	4010	BCR	C20-C19-C18	6.26	143.99	126.42
14	BA	1215	CLA	C1C-C2C-C3C	-6.26	100.32	106.95
14	GA	1104	CLA	C2C-C1C-NC	6.25	115.83	109.97
14	GA	1109	CLA	C4A-NA-C1A	6.25	109.51	106.71
14	AA	1109	CLA	C4A-NA-C1A	6.25	109.51	106.71
14	GA	1128	CLA	O2D-CGD-CBD	6.24	122.17	111.25
14	AA	1115	CLA	C2C-C1C-NC	6.24	115.82	109.97
14	aA	1105	CLA	C2C-C1C-NC	6.24	115.82	109.97
14	aA	1128	CLA	O2D-CGD-CBD	6.24	122.17	111.25
14	GA	1125	CLA	C4A-NA-C1A	6.23	109.51	106.71
14	aA	1122	CLA	C2C-C1C-NC	6.23	115.81	109.97
14	bA	1215	CLA	C1C-C2C-C3C	-6.22	100.35	106.95
18	GA	4002	BCR	C11-C10-C9	6.22	136.19	127.31
14	HA	1215	CLA	C1C-C2C-C3C	-6.22	100.36	106.95
14	AA	1128	CLA	O2D-CGD-CBD	6.22	122.14	111.25
13	AA	1011	CL0	C1C-C2C-C3C	-6.22	100.36	106.95
13	aA	1011	CL0	C1C-C2C-C3C	-6.22	100.36	106.95

Continued on next page...

PRELIMINARY VALIDATION REPORT

Continued from previous page...

Mol	Chain	Res	Type	Atoms	Z	Observed(^{o})	Ideal(^{o})
18	aA	4002	BCR	C11-C10-C9	6.21	136.17	127.31
14	bA	1222	CLA	C2C-C1C-NC	6.20	115.78	109.97
13	GA	1011	CL0	C1C-C2C-C3C	-6.20	100.38	106.95
14	BA	1212	CLA	C2C-C1C-NC	6.20	115.78	109.97
14	BA	1222	CLA	C2C-C1C-NC	6.19	115.77	109.97
14	aA	1109	CLA	C4A-NA-C1A	6.19	109.49	106.71
14	AA	1125	CLA	OBD-CAD-C3D	-6.19	117.08	128.04
14	GA	1122	CLA	C2C-C1C-NC	6.18	115.76	109.97
14	GA	1105	CLA	C2C-C1C-NC	6.18	115.76	109.97
14	AA	1105	CLA	C2C-C1C-NC	6.18	115.76	109.97
18	AA	4002	BCR	C11-C10-C9	6.18	136.13	127.31
14	HA	1229	CLA	C2C-C1C-NC	6.18	115.76	109.97
14	AA	1125	CLA	C4A-NA-C1A	6.17	109.48	106.71
14	HA	1239	CLA	O2A-C1-C2	6.17	124.86	108.64
14	BA	1210	CLA	O2D-CGD-CBD	6.17	122.05	111.25
14	aA	1125	CLA	OBD-CAD-C3D	-6.17	117.11	128.04
14	bA	1239	CLA	O2A-C1-C2	6.17	124.85	108.64
14	aA	1130	CLA	C2C-C1C-NC	6.17	115.75	109.97
14	HA	1212	CLA	C2C-C1C-NC	6.17	115.75	109.97
14	BA	1239	CLA	O2A-C1-C2	6.17	124.85	108.64
18	GA	4001	BCR	C20-C19-C18	6.16	143.72	126.42
14	GA	1125	CLA	OBD-CAD-C3D	-6.16	117.13	128.04
18	aA	4001	BCR	C20-C19-C18	6.16	143.71	126.42
14	AA	1129	CLA	C2C-C1C-NC	6.15	115.73	109.97
14	bA	1212	CLA	C2C-C1C-NC	6.15	115.73	109.97
14	bA	1229	CLA	C2C-C1C-NC	6.15	115.73	109.97
14	BA	1211	CLA	C2C-C1C-NC	6.15	115.73	109.97
14	HA	1210	CLA	O2D-CGD-CBD	6.15	122.01	111.25
18	AA	4001	BCR	C20-C19-C18	6.15	143.69	126.42
14	AA	1122	CLA	C2C-C1C-NC	6.14	115.73	109.97
14	aA	1131	CLA	O2A-C1-C2	6.14	124.78	108.64
14	AA	1131	CLA	O2A-C1-C2	6.14	124.77	108.64
14	GA	1119	CLA	C2C-C1C-NC	6.14	115.72	109.97
14	HA	1222	CLA	C2C-C1C-NC	6.14	115.72	109.97
14	HA	1235	CLA	C2C-C1C-NC	6.13	115.72	109.97
14	GA	1131	CLA	O2A-C1-C2	6.13	124.75	108.64
14	bA	1210	CLA	O2D-CGD-CBD	6.13	121.98	111.25
14	BA	1235	CLA	C2C-C1C-NC	6.13	115.72	109.97
14	aA	1104	CLA	C4A-NA-C1A	6.13	109.46	106.71
14	aA	1124	CLA	O2A-C1-C2	6.13	124.73	108.64
14	BA	1229	CLA	C2C-C1C-NC	6.12	115.71	109.97
14	GA	1124	CLA	O2A-C1-C2	6.12	124.72	108.64

Continued on next page...

PRELIMINARY VALIDATION REPORT

Continued from previous page...

Mol	Chain	Res	Type	Atoms	Z	Observed(°)	Ideal(°)
14	AA	1119	CLA	C2C-C1C-NC	6.12	115.70	109.97
14	bA	1211	CLA	C2C-C1C-NC	6.11	115.70	109.97
14	AA	1124	CLA	O2A-C1-C2	6.11	124.70	108.64
14	bA	1021	CLA	C2C-C1C-NC	6.11	115.70	109.97
14	AA	1104	CLA	C4A-NA-C1A	6.11	109.45	106.71
14	HA	1021	CLA	C2C-C1C-NC	6.11	115.70	109.97
14	GA	1129	CLA	C2C-C1C-NC	6.11	115.69	109.97
14	BA	1203	CLA	C2C-C1C-NC	6.11	115.69	109.97
14	HA	1213	CLA	O2D-CGD-CBD	6.11	121.94	111.25
14	GA	1130	CLA	C2C-C1C-NC	6.10	115.69	109.97
14	aA	1119	CLA	C2C-C1C-NC	6.10	115.69	109.97
14	aA	1129	CLA	C2C-C1C-NC	6.10	115.69	109.97
14	BA	1205	CLA	O2A-CGA-O1A	-6.10	107.98	123.56
14	bA	1203	CLA	C2C-C1C-NC	6.10	115.68	109.97
14	AA	1130	CLA	C2C-C1C-NC	6.09	115.68	109.97
14	bA	1213	CLA	O2D-CGD-CBD	6.09	121.91	111.25
14	BA	1021	CLA	C2C-C1C-NC	6.09	115.68	109.97
14	AA	1124	CLA	C2C-C1C-NC	6.09	115.68	109.97
14	BA	1213	CLA	O2D-CGD-CBD	6.09	121.91	111.25
14	bA	1205	CLA	O2A-CGA-O1A	-6.09	108.00	123.56
14	HA	1203	CLA	C2C-C1C-NC	6.09	115.67	109.97
14	bA	1235	CLA	C2C-C1C-NC	6.09	115.67	109.97
14	aA	1118	CLA	O2D-CGD-CBD	6.08	121.89	111.25
14	HA	1211	CLA	C2C-C1C-NC	6.08	115.67	109.97
14	GA	1118	CLA	O2D-CGD-CBD	6.08	121.88	111.25
14	HA	1205	CLA	O2A-CGA-O1A	-6.07	108.04	123.56
14	AA	1106	CLA	C4A-NA-C1A	6.07	109.44	106.71
18	bA	4013	BCR	C24-C23-C22	-6.07	117.09	126.21
14	AA	1102	CLA	O2D-CGD-CBD	6.06	121.86	111.25
14	aA	1124	CLA	C2C-C1C-NC	6.06	115.65	109.97
14	aA	1102	CLA	O2D-CGD-CBD	6.06	121.85	111.25
14	GA	1104	CLA	C4A-NA-C1A	6.05	109.43	106.71
14	GA	1102	CLA	O2D-CGD-CBD	6.05	121.84	111.25
18	HA	4013	BCR	C24-C23-C22	-6.05	117.12	126.21
14	AA	1118	CLA	O2D-CGD-CBD	6.05	121.84	111.25
14	GA	1124	CLA	C2C-C1C-NC	6.05	115.64	109.97
14	AA	1140	CLA	O2D-CGD-CBD	6.05	121.84	111.25
14	GA	1140	CLA	O2D-CGD-CBD	6.05	121.84	111.25
14	aA	1140	CLA	O2D-CGD-CBD	6.04	121.82	111.25
14	BA	1222	CLA	O2D-CGD-CBD	6.04	121.82	111.25
18	BA	4013	BCR	C24-C23-C22	-6.04	117.14	126.21
14	HA	1222	CLA	O2D-CGD-CBD	6.03	121.81	111.25

Continued on next page...

PRELIMINARY VALIDATION REPORT

Continued from previous page...

Mol	Chain	Res	Type	Atoms	Z	Observed(^{o})	Ideal(^{o})
14	AA	1131	CLA	O2D-CGD-CBD	6.03	121.81	111.25
14	GA	1125	CLA	C2C-C1C-NC	6.03	115.62	109.97
14	bA	1222	CLA	O2D-CGD-CBD	6.03	121.80	111.25
14	bA	1224	CLA	O2D-CGD-CBD	6.03	121.80	111.25
14	AA	1136	CLA	C4A-NA-C1A	6.02	109.41	106.71
14	aA	1116	CLA	O2A-C1-C2	6.02	124.46	108.64
14	GA	1131	CLA	O2D-CGD-CBD	6.02	121.79	111.25
14	GA	1116	CLA	O2A-C1-C2	6.02	124.45	108.64
18	GA	4003	BCR	C24-C23-C22	-6.01	117.18	126.21
14	HA	1224	CLA	O2D-CGD-CBD	6.01	121.77	111.25
14	AA	1118	CLA	C1C-C2C-C3C	-6.01	100.58	106.95
14	aA	1131	CLA	O2D-CGD-CBD	6.01	121.77	111.25
18	bA	4005	BCR	C24-C23-C22	-6.01	117.18	126.21
14	aA	1122	CLA	C4A-NA-C1A	6.01	109.41	106.71
14	AA	1116	CLA	O2A-C1-C2	6.01	124.43	108.64
14	AA	1133	CLA	O2D-CGD-CBD	6.00	121.76	111.25
14	GA	1101	CLA	C2C-C1C-NC	6.00	115.60	109.97
14	bA	1239	CLA	C4A-NA-C1A	6.00	109.41	106.71
14	BA	1224	CLA	O2D-CGD-CBD	6.00	121.75	111.25
14	aA	1101	CLA	C2C-C1C-NC	6.00	115.59	109.97
14	GA	1106	CLA	C4A-NA-C1A	6.00	109.40	106.71
14	aA	1106	CLA	C4A-NA-C1A	6.00	109.40	106.71
14	HA	1239	CLA	C1C-C2C-C3C	-6.00	100.59	106.95
14	AA	1101	CLA	C2C-C1C-NC	6.00	115.59	109.97
18	AA	4003	BCR	C24-C23-C22	-6.00	117.20	126.21
18	BA	4005	BCR	C24-C23-C22	-5.99	117.21	126.21
14	LA	1502	CLA	O2A-C1-C2	5.99	124.38	108.64
18	aA	4003	BCR	C24-C23-C22	-5.99	117.22	126.21
14	bA	1239	CLA	C1C-C2C-C3C	-5.99	100.60	106.95
14	GA	1118	CLA	C1C-C2C-C3C	-5.99	100.61	106.95
14	bA	1202	CLA	O2A-CGA-O1A	-5.99	108.26	123.56
14	aA	1136	CLA	C4A-NA-C1A	5.98	109.40	106.71
13	GA	1011	CL0	O2A-C1-C2	5.98	124.36	108.64
13	AA	1011	CL0	O2A-C1-C2	5.98	124.36	108.64
14	GA	1137	CLA	C4A-NA-C1A	5.98	109.39	106.71
14	aA	1133	CLA	O2D-CGD-CBD	5.98	121.71	111.25
14	UA	1502	CLA	O2A-C1-C2	5.98	124.35	108.64
18	lA	4022	BCR	C20-C19-C18	5.98	143.21	126.42
14	aA	1126	CLA	O2D-CGD-CBD	5.98	121.71	111.25
14	aA	1104	CLA	C1C-C2C-C3C	-5.98	100.62	106.95
13	aA	1011	CL0	O2A-C1-C2	5.98	124.34	108.64
14	GA	1133	CLA	O2D-CGD-CBD	5.97	121.71	111.25

Continued on next page...

PRELIMINARY VALIDATION REPORT

Continued from previous page...

Mol	Chain	Res	Type	Atoms	Z	Observed(^{o})	Ideal(^{o})
14	lA	1502	CLA	O2A-C1-C2	5.97	124.34	108.64
14	BA	1239	CLA	C4A-NA-C1A	5.97	109.39	106.71
18	UA	4022	BCR	C20-C19-C18	5.97	143.18	126.42
14	BA	1202	CLA	O2A-CGA-O1A	-5.97	108.31	123.56
18	LA	4022	BCR	C20-C19-C18	5.97	143.18	126.42
14	HA	1202	CLA	O2A-CGA-O1A	-5.96	108.32	123.56
18	HA	4005	BCR	C24-C23-C22	-5.96	117.25	126.21
14	AA	1125	CLA	C2C-C1C-NC	5.96	115.56	109.97
14	GA	1126	CLA	O2D-CGD-CBD	5.96	121.68	111.25
14	AA	1137	CLA	C4A-NA-C1A	5.96	109.39	106.71
13	aA	1011	CL0	O2D-CGD-CBD	5.96	121.68	111.25
14	aA	1118	CLA	C1C-C2C-C3C	-5.96	100.64	106.95
14	BA	1239	CLA	C1C-C2C-C3C	-5.95	100.64	106.95
14	bA	1226	CLA	O2A-C1-C2	5.95	124.27	108.64
14	AA	1126	CLA	O2D-CGD-CBD	5.95	121.66	111.25
14	HA	1221	CLA	C2C-C1C-NC	5.95	115.54	109.97
13	AA	1011	CL0	O2D-CGD-CBD	5.95	121.66	111.25
14	AA	1104	CLA	C1C-C2C-C3C	-5.94	100.65	106.95
14	GA	1136	CLA	C4A-NA-C1A	5.94	109.38	106.71
14	HA	1226	CLA	O2A-C1-C2	5.94	124.25	108.64
14	LA	1501	CLA	C4A-NA-C1A	5.94	109.38	106.71
13	GA	1011	CL0	O2D-CGD-CBD	5.94	121.64	111.25
14	BA	1226	CLA	O2A-C1-C2	5.94	124.24	108.64
14	HA	1239	CLA	C4A-NA-C1A	5.93	109.37	106.71
14	GA	1122	CLA	C4A-NA-C1A	5.92	109.37	106.71
14	BA	1217	CLA	C4A-NA-C1A	5.92	109.37	106.71
14	UA	1501	CLA	C4A-NA-C1A	5.92	109.37	106.71
14	aA	1125	CLA	C2C-C1C-NC	5.91	115.51	109.97
14	HA	1219	CLA	C4A-NA-C1A	5.91	109.36	106.71
14	GA	1133	CLA	C1C-C2C-C3C	-5.91	100.69	106.95
14	aA	1103	CLA	O2D-CGD-CBD	5.90	121.58	111.25
14	bA	1221	CLA	C2C-C1C-NC	5.90	115.50	109.97
14	AA	1122	CLA	C4A-NA-C1A	5.90	109.36	106.71
14	BA	1221	CLA	C4A-NA-C1A	5.90	109.36	106.71
14	GA	1103	CLA	O2D-CGD-CBD	5.90	121.58	111.25
14	GA	1104	CLA	C1C-C2C-C3C	-5.90	100.69	106.95
14	GA	1118	CLA	C4A-NA-C1A	5.90	109.36	106.71
14	bA	1214	CLA	O2D-CGD-CBD	5.89	121.56	111.25
14	bA	1217	CLA	C4A-NA-C1A	5.89	109.35	106.71
14	AA	1103	CLA	O2D-CGD-CBD	5.88	121.55	111.25
14	AA	1111	CLA	O2D-CGD-CBD	5.88	121.54	111.25
14	aA	1111	CLA	O2D-CGD-CBD	5.88	121.54	111.25

Continued on next page...

PRELIMINARY VALIDATION REPORT

Continued from previous page...

Mol	Chain	Res	Type	Atoms	Z	Observed(°)	Ideal(°)
14	aA	1133	CLA	C1C-C2C-C3C	-5.88	100.72	106.95
14	0	1012	CLA	C2C-C1C-NC	5.87	115.47	109.97
14	BA	1221	CLA	C2C-C1C-NC	5.87	115.47	109.97
14	HA	1023	CLA	C2C-C1C-NC	5.87	115.47	109.97
14	aA	1137	CLA	C4A-NA-C1A	5.86	109.34	106.71
14	GA	1111	CLA	O2D-CGD-CBD	5.86	121.51	111.25
14	BA	1023	CLA	C2C-C1C-NC	5.86	115.46	109.97
14	bA	1201	CLA	O2A-CGA-O1A	-5.86	108.58	123.56
14	BA	1201	CLA	O2A-CGA-O1A	-5.85	108.60	123.56
14	bA	1219	CLA	C4A-NA-C1A	5.85	109.34	106.71
14	AA	1133	CLA	C1C-C2C-C3C	-5.85	100.75	106.95
14	bA	1238	CLA	C2C-C1C-NC	5.85	115.45	109.97
14	HA	1201	CLA	O2A-CGA-O1A	-5.85	108.62	123.56
14	aA	1126	CLA	C4A-NA-C1A	5.85	109.33	106.71
14	BA	1218	CLA	C4A-NA-C1A	5.85	109.33	106.71
14	bA	1221	CLA	C4A-NA-C1A	5.84	109.33	106.71
14	BA	1204	CLA	O2D-CGD-CBD	5.84	121.48	111.25
14	bA	1022	CLA	C2C-C1C-NC	5.84	115.44	109.97
14	BA	1214	CLA	O2D-CGD-CBD	5.84	121.47	111.25
14	HA	1214	CLA	O2D-CGD-CBD	5.84	121.47	111.25
14	aA	1118	CLA	C4A-NA-C1A	5.84	109.33	106.71
14	bA	1218	CLA	C4A-NA-C1A	5.84	109.33	106.71
14	BA	1022	CLA	C2C-C1C-NC	5.84	115.44	109.97
14	bA	1204	CLA	O2D-CGD-CBD	5.84	121.47	111.25
14	BA	1219	CLA	C4A-NA-C1A	5.83	109.33	106.71
14	bA	1023	CLA	C2C-C1C-NC	5.83	115.43	109.97
14	HA	1204	CLA	O2D-CGD-CBD	5.82	121.44	111.25
14	HA	1238	CLA	C2C-C1C-NC	5.82	115.42	109.97
14	AA	1126	CLA	C4A-NA-C1A	5.81	109.32	106.71
14	HA	1216	CLA	O2A-C1-C2	5.81	123.91	108.64
14	bA	1216	CLA	O2A-C1-C2	5.81	123.91	108.64
14	bA	1235	CLA	C4A-NA-C1A	5.80	109.31	106.71
14	bA	1209	CLA	O2D-CGD-CBD	5.80	121.41	111.25
14	HA	1209	CLA	O2D-CGD-CBD	5.80	121.40	111.25
14	GA	1123	CLA	O2D-CGD-CBD	5.80	121.40	111.25
14	AA	1123	CLA	O2D-CGD-CBD	5.80	121.40	111.25
14	HA	1217	CLA	C4A-NA-C1A	5.80	109.31	106.71
14	BA	1238	CLA	C2C-C1C-NC	5.79	115.40	109.97
14	aA	1123	CLA	O2D-CGD-CBD	5.79	121.39	111.25
14	1A	1501	CLA	C4A-NA-C1A	5.79	109.31	106.71
14	HA	1221	CLA	C4A-NA-C1A	5.79	109.31	106.71
14	BA	1209	CLA	O2D-CGD-CBD	5.78	121.37	111.25

Continued on next page...

PRELIMINARY VALIDATION REPORT

Continued from previous page...

Mol	Chain	Res	Type	Atoms	Z	Observed(^{o})	Ideal(^{o})
14	HA	1218	CLA	O2D-CGD-CBD	5.78	121.37	111.25
14	BA	1216	CLA	O2A-C1-C2	5.78	123.83	108.64
14	aA	1114	CLA	C1C-C2C-C3C	-5.78	100.83	106.95
14	HA	1235	CLA	C4A-NA-C1A	5.77	109.30	106.71
14	bA	1231	CLA	C2C-C1C-NC	5.77	115.38	109.97
14	bA	1224	CLA	C2C-C1C-NC	5.77	115.38	109.97
14	AA	1118	CLA	C4A-NA-C1A	5.77	109.30	106.71
14	HA	1218	CLA	C4A-NA-C1A	5.77	109.30	106.71
14	HA	1231	CLA	C2C-C1C-NC	5.77	115.38	109.97
14	GA	1114	CLA	C1C-C2C-C3C	-5.76	100.84	106.95
14	aA	1116	CLA	O2D-CGD-CBD	5.76	121.34	111.25
14	GA	1126	CLA	C4A-NA-C1A	5.76	109.30	106.71
14	HA	1207	CLA	C1C-C2C-C3C	-5.76	100.85	106.95
14	GA	1116	CLA	O2D-CGD-CBD	5.76	121.33	111.25
14	AA	1116	CLA	O2D-CGD-CBD	5.75	121.32	111.25
14	BA	1218	CLA	O2D-CGD-CBD	5.75	121.31	111.25
14	BA	1231	CLA	C2C-C1C-NC	5.74	115.35	109.97
14	HA	1239	CLA	O2D-CGD-CBD	5.74	121.29	111.25
18	BA	4014	BCR	C24-C23-C22	-5.74	117.59	126.21
14	GA	1012	CLA	O2A-C1-C2	5.74	123.71	108.64
14	BA	1224	CLA	C2C-C1C-NC	5.73	115.34	109.97
14	AA	1012	CLA	O2A-C1-C2	5.73	123.69	108.64
14	AA	1114	CLA	C1C-C2C-C3C	-5.73	100.88	106.95
14	aA	1012	CLA	O2A-C1-C2	5.72	123.68	108.64
18	HA	4014	BCR	C24-C23-C22	-5.72	117.61	126.21
14	bA	1218	CLA	O2D-CGD-CBD	5.72	121.27	111.25
14	AA	1119	CLA	O2A-C1-C2	5.72	123.67	108.64
14	BA	1207	CLA	C1C-C2C-C3C	-5.72	100.89	106.95
14	bA	1239	CLA	O2D-CGD-CBD	5.71	121.25	111.25
14	aA	1119	CLA	O2A-C1-C2	5.71	123.65	108.64
14	BA	1239	CLA	O2D-CGD-CBD	5.71	121.25	111.25
14	bA	1219	CLA	O2D-CGD-CBD	5.71	121.25	111.25
14	BA	1235	CLA	C4A-NA-C1A	5.71	109.27	106.71
14	HA	1224	CLA	C2C-C1C-NC	5.71	115.32	109.97
18	bA	4014	BCR	C24-C23-C22	-5.70	117.64	126.21
14	GA	1119	CLA	O2A-C1-C2	5.70	123.62	108.64
14	aA	1125	CLA	O2A-C1-C2	5.70	123.62	108.64
14	bA	1207	CLA	C1C-C2C-C3C	-5.70	100.91	106.95
14	GA	1125	CLA	O2A-C1-C2	5.69	123.60	108.64
14	AA	1013	CLA	CMB-C2B-C3B	5.69	135.47	124.80
14	bA	1023	CLA	O2A-C1-C2	5.69	123.58	108.64
14	aA	1013	CLA	CMB-C2B-C3B	5.68	135.46	124.80

Continued on next page...

PRELIMINARY VALIDATION REPORT

Continued from previous page...

Mol	Chain	Res	Type	Atoms	Z	Observed(°)	Ideal(°)
14	BA	1219	CLA	O2D-CGD-CBD	5.68	121.20	111.25
14	AA	1125	CLA	O2A-C1-C2	5.68	123.57	108.64
14	BA	1206	CLA	C4A-NA-C1A	5.68	109.26	106.71
14	HA	1206	CLA	C4A-NA-C1A	5.68	109.26	106.71
14	HA	1219	CLA	O2D-CGD-CBD	5.68	121.19	111.25
14	GA	1013	CLA	CMB-C2B-C3B	5.68	135.45	124.80
18	AA	4008	BCR	C20-C19-C18	5.68	142.36	126.42
14	HA	1023	CLA	O2A-C1-C2	5.67	123.55	108.64
14	BA	1023	CLA	O2A-C1-C2	5.67	123.53	108.64
14	bA	1214	CLA	C1C-C2C-C3C	-5.66	100.95	106.95
18	GA	4008	BCR	C20-C19-C18	5.66	142.32	126.42
14	HA	1226	CLA	O2D-CGD-CBD	5.66	121.16	111.25
14	BA	1226	CLA	O2D-CGD-CBD	5.66	121.15	111.25
18	HA	4004	BCR	C20-C19-C18	5.66	142.31	126.42
14	AA	1115	CLA	C4A-NA-C1A	5.66	109.25	106.71
14	bA	1211	CLA	C4A-NA-C1A	5.66	109.25	106.71
18	aA	4008	BCR	C20-C19-C18	5.65	142.30	126.42
14	bA	1226	CLA	O2D-CGD-CBD	5.65	121.14	111.25
14	BA	1211	CLA	C4A-NA-C1A	5.64	109.24	106.71
14	bA	1206	CLA	C4A-NA-C1A	5.64	109.24	106.71
14	BA	1214	CLA	C1C-C2C-C3C	-5.64	100.97	106.95
18	BA	4004	BCR	C20-C19-C18	5.64	142.26	126.42
14	HA	1220	CLA	O2A-C1-C2	5.64	123.46	108.64
18	bA	4004	BCR	C20-C19-C18	5.64	142.25	126.42
14	bA	1220	CLA	O2A-C1-C2	5.64	123.45	108.64
14	aA	1112	CLA	C4A-NA-C1A	5.63	109.24	106.71
14	BA	1220	CLA	O2A-C1-C2	5.63	123.43	108.64
18	RA	4018	BCR	C15-C14-C13	-5.63	119.28	127.31
18	IA	4018	BCR	C15-C14-C13	-5.62	119.28	127.31
14	HA	1214	CLA	C1C-C2C-C3C	-5.62	100.99	106.95
14	BA	1220	CLA	C4A-NA-C1A	5.62	109.23	106.71
14	HA	1220	CLA	C4A-NA-C1A	5.61	109.23	106.71
14	TA	1401	CLA	C1C-C2C-C3C	-5.61	101.00	106.95
14	HA	1204	CLA	C1C-C2C-C3C	-5.61	101.01	106.95
14	bA	1204	CLA	C1C-C2C-C3C	-5.60	101.01	106.95
14	aA	1115	CLA	C4A-NA-C1A	5.60	109.22	106.71
14	AA	1113	CLA	C4A-NA-C1A	5.60	109.22	106.71
14	KA	1401	CLA	C1C-C2C-C3C	-5.60	101.02	106.95
14	BA	1229	CLA	O2D-CGD-CBD	5.60	121.04	111.25
14	kA	1401	CLA	C1C-C2C-C3C	-5.59	101.02	106.95
18	iA	4018	BCR	C15-C14-C13	-5.59	119.33	127.31
14	bA	1207	CLA	C4A-NA-C1A	5.59	109.22	106.71

Continued on next page...

PRELIMINARY VALIDATION REPORT

Continued from previous page...

Mol	Chain	Res	Type	Atoms	Z	Observed(^{o})	Ideal(^{o})
14	bA	1229	CLA	O2D-CGD-CBD	5.59	121.03	111.25
14	HA	1208	CLA	C1C-C2C-C3C	-5.58	101.03	106.95
14	bA	1227	CLA	O2D-CGD-CBD	5.58	121.02	111.25
14	BA	1227	CLA	O2D-CGD-CBD	5.57	121.01	111.25
14	HA	1213	CLA	O2A-C1-C2	5.57	123.28	108.64
14	bA	1220	CLA	C4A-NA-C1A	5.57	109.21	106.71
14	BA	1213	CLA	O2A-C1-C2	5.57	123.27	108.64
14	bA	1208	CLA	C1C-C2C-C3C	-5.56	101.05	106.95
14	aA	1113	CLA	O2D-CGD-CBD	5.56	120.98	111.25
14	AA	1113	CLA	O2D-CGD-CBD	5.56	120.98	111.25
14	GA	1112	CLA	O2D-CGD-CBD	5.56	120.98	111.25
14	aA	1112	CLA	O2D-CGD-CBD	5.56	120.98	111.25
14	bA	1207	CLA	O2D-CGD-CBD	5.56	120.98	111.25
14	bA	1213	CLA	O2A-C1-C2	5.56	123.24	108.64
14	HA	1227	CLA	O2D-CGD-CBD	5.55	120.97	111.25
14	HA	1229	CLA	O2D-CGD-CBD	5.55	120.97	111.25
14	GA	1113	CLA	O2D-CGD-CBD	5.55	120.97	111.25
14	KA	1401	CLA	C4A-NA-C1A	5.55	109.20	106.71
14	BA	1204	CLA	C1C-C2C-C3C	-5.54	101.07	106.95
14	aA	1114	CLA	O2D-CGD-CBD	5.54	120.94	111.25
14	BA	1225	CLA	O2A-C1-C2	5.54	123.19	108.64
14	HA	1207	CLA	O2D-CGD-CBD	5.54	120.94	111.25
14	HA	1211	CLA	C4A-NA-C1A	5.53	109.19	106.71
14	BA	1208	CLA	C1C-C2C-C3C	-5.53	101.09	106.95
14	bA	1225	CLA	O2A-C1-C2	5.53	123.17	108.64
14	HA	1217	CLA	C1C-C2C-C3C	-5.53	101.09	106.95
14	aA	1113	CLA	C4A-NA-C1A	5.53	109.19	106.71
14	BA	1207	CLA	O2D-CGD-CBD	5.53	120.92	111.25
14	HA	1207	CLA	C4A-NA-C1A	5.52	109.19	106.71
14	HA	1225	CLA	O2A-C1-C2	5.52	123.14	108.64
14	GA	1115	CLA	C4A-NA-C1A	5.52	109.19	106.71
14	AA	1112	CLA	O2D-CGD-CBD	5.52	120.91	111.25
14	HA	1234	CLA	C2C-C1C-NC	5.51	115.14	109.97
14	BA	1217	CLA	C1C-C2C-C3C	-5.51	101.11	106.95
14	bA	1229	CLA	C1C-C2C-C3C	-5.51	101.11	106.95
14	GA	1126	CLA	O2A-CGA-O1A	-5.51	109.48	123.56
14	AA	1126	CLA	O2A-CGA-O1A	-5.51	109.48	123.56
14	TA	1401	CLA	C4A-NA-C1A	5.51	109.18	106.71
14	HA	1229	CLA	C1C-C2C-C3C	-5.50	101.12	106.95
14	BA	1229	CLA	C1C-C2C-C3C	-5.50	101.12	106.95
14	LA	1502	CLA	C4A-NA-C1A	5.50	109.18	106.71
14	BA	1207	CLA	C4A-NA-C1A	5.50	109.18	106.71

Continued on next page...

PRELIMINARY VALIDATION REPORT

Continued from previous page...

Mol	Chain	Res	Type	Atoms	Z	Observed(^{o})	Ideal(^{o})
14	bA	1234	CLA	C2C-C1C-NC	5.50	115.12	109.97
14	GA	1113	CLA	C4A-NA-C1A	5.49	109.18	106.71
14	aA	1126	CLA	O2A-CGA-O1A	-5.49	109.52	123.56
14	AA	1130	CLA	O2D-CGD-CBD	5.49	120.86	111.25
14	BA	1204	CLA	O2A-C1-C2	5.49	123.07	108.64
14	GA	1114	CLA	O2D-CGD-CBD	5.49	120.86	111.25
14	BA	1236	CLA	C1C-C2C-C3C	-5.49	101.13	106.95
14	aA	1131	CLA	C4A-NA-C1A	5.49	109.17	106.71
14	AA	1114	CLA	O2D-CGD-CBD	5.49	120.85	111.25
14	aA	1130	CLA	O2D-CGD-CBD	5.49	120.85	111.25
14	AA	1112	CLA	C4A-NA-C1A	5.49	109.17	106.71
14	aA	1118	CLA	O2A-C1-C2	5.48	123.04	108.64
14	HA	1204	CLA	O2A-C1-C2	5.48	123.04	108.64
14	bA	1217	CLA	C1C-C2C-C3C	-5.48	101.14	106.95
14	kA	1401	CLA	C4A-NA-C1A	5.48	109.17	106.71
14	bA	1208	CLA	O2D-CGD-CBD	5.48	120.84	111.25
14	bA	1204	CLA	O2A-C1-C2	5.48	123.03	108.64
14	GA	1118	CLA	O2A-C1-C2	5.48	123.03	108.64
14	AA	1118	CLA	O2A-C1-C2	5.47	123.02	108.64
14	GA	1109	CLA	C1C-C2C-C3C	-5.47	101.15	106.95
14	BA	1208	CLA	O2D-CGD-CBD	5.47	120.83	111.25
14	HA	1208	CLA	O2D-CGD-CBD	5.47	120.82	111.25
14	bA	1215	CLA	O2D-CGD-CBD	5.47	120.82	111.25
14	AA	1137	CLA	C1C-C2C-C3C	-5.47	101.15	106.95
14	GA	1130	CLA	O2D-CGD-CBD	5.47	120.82	111.25
14	AA	1109	CLA	C1C-C2C-C3C	-5.46	101.16	106.95
14	AA	1128	CLA	C4A-NA-C1A	5.46	109.16	106.71
14	BA	1234	CLA	C2C-C1C-NC	5.46	115.08	109.97
14	bA	1236	CLA	C1C-C2C-C3C	-5.45	101.17	106.95
14	GA	1131	CLA	C4A-NA-C1A	5.45	109.16	106.71
14	lA	1502	CLA	C4A-NA-C1A	5.45	109.16	106.71
14	HA	1236	CLA	C1C-C2C-C3C	-5.45	101.17	106.95
14	aA	1137	CLA	C1C-C2C-C3C	-5.44	101.18	106.95
14	UA	1502	CLA	C4A-NA-C1A	5.44	109.15	106.71
14	GA	1112	CLA	C4A-NA-C1A	5.44	109.15	106.71
14	AA	1123	CLA	C1C-C2C-C3C	-5.44	101.19	106.95
14	HA	1215	CLA	O2D-CGD-CBD	5.44	120.77	111.25
14	BA	1215	CLA	O2D-CGD-CBD	5.43	120.76	111.25
14	aA	1109	CLA	C1C-C2C-C3C	-5.43	101.19	106.95
14	GA	1137	CLA	C1C-C2C-C3C	-5.43	101.20	106.95
14	AA	1131	CLA	C4A-NA-C1A	5.42	109.14	106.71
18	bA	4009	BCR	C24-C23-C22	-5.42	118.07	126.21

Continued on next page...

PRELIMINARY VALIDATION REPORT

Continued from previous page...

Mol	Chain	Res	Type	Atoms	Z	Observed(^{o})	Ideal(^{o})
14	bA	1238	CLA	O2A-C1-C2	5.42	122.87	108.64
14	AA	1136	CLA	O2A-CGA-O1A	-5.42	109.72	123.56
14	aA	1128	CLA	O2A-C1-C2	5.42	122.87	108.64
14	AA	1131	CLA	C1C-C2C-C3C	-5.42	101.21	106.95
14	HA	1238	CLA	O2A-C1-C2	5.41	122.86	108.64
14	GA	1128	CLA	O2A-C1-C2	5.41	122.86	108.64
14	aA	1136	CLA	O2A-CGA-O1A	-5.41	109.73	123.56
14	UA	1502	CLA	C1C-C2C-C3C	-5.41	101.22	106.95
14	AA	1128	CLA	O2A-C1-C2	5.41	122.85	108.64
14	GA	1123	CLA	C1C-C2C-C3C	-5.41	101.22	106.95
14	WA	1701	CLA	C4A-NA-C1A	5.41	109.14	106.71
18	mA	4021	BCR	C24-C23-C22	-5.40	118.09	126.21
14	LA	1502	CLA	C1C-C2C-C3C	-5.40	101.22	106.95
14	AA	1140	CLA	C4A-NA-C1A	5.40	109.14	106.71
14	HA	1218	CLA	C1C-C2C-C3C	-5.40	101.23	106.95
14	aA	1123	CLA	C1C-C2C-C3C	-5.40	101.23	106.95
14	BA	1205	CLA	O2A-CGA-CBA	5.40	129.27	111.93
14	GA	1136	CLA	O2A-CGA-O1A	-5.40	109.76	123.56
18	MA	4021	BCR	C24-C23-C22	-5.40	118.10	126.21
14	BA	1203	CLA	C1C-C2C-C3C	-5.40	101.23	106.95
14	BA	1238	CLA	O2A-C1-C2	5.40	122.81	108.64
18	VA	4021	BCR	C24-C23-C22	-5.39	118.11	126.21
14	BA	1203	CLA	C4A-NA-C1A	5.39	109.13	106.71
14	HA	1205	CLA	O2A-CGA-CBA	5.39	129.24	111.93
14	bA	1205	CLA	O2A-CGA-CBA	5.39	129.23	111.93
18	HA	4009	BCR	C24-C23-C22	-5.39	118.12	126.21
14	GA	1117	CLA	C4A-NA-C1A	5.38	109.12	106.71
14	HA	1203	CLA	C1C-C2C-C3C	-5.38	101.25	106.95
14	HA	1203	CLA	C4A-NA-C1A	5.38	109.12	106.71
14	GA	1131	CLA	C1C-C2C-C3C	-5.38	101.25	106.95
18	mA	4021	BCR	C15-C14-C13	-5.38	119.64	127.31
14	xA	1701	CLA	C4A-NA-C1A	5.38	109.12	106.71
14	bA	1203	CLA	C1C-C2C-C3C	-5.38	101.25	106.95
14	HA	1023	CLA	O2D-CGD-CBD	5.38	120.66	111.25
14	HA	1232	CLA	C1C-C2C-C3C	-5.37	101.26	106.95
14	BA	1212	CLA	C4A-NA-C1A	5.37	109.12	106.71
14	BA	1218	CLA	C1C-C2C-C3C	-5.37	101.26	106.95
14	HA	1228	CLA	C4A-NA-C1A	5.37	109.12	106.71
14	AA	1113	CLA	C1C-C2C-C3C	-5.36	101.27	106.95
14	BA	1021	CLA	O2D-CGD-CBD	5.36	120.63	111.25
14	BA	1232	CLA	C1C-C2C-C3C	-5.36	101.27	106.95
18	BA	4009	BCR	C24-C23-C22	-5.36	118.16	126.21

Continued on next page...

PRELIMINARY VALIDATION REPORT

Continued from previous page...

Mol	Chain	Res	Type	Atoms	Z	Observed(^{o})	Ideal(^{o})
14	aA	1102	CLA	C4A-NA-C1A	5.36	109.11	106.71
14	AA	1108	CLA	C4A-NA-C1A	5.36	109.11	106.71
14	HA	1202	CLA	C4A-NA-C1A	5.36	109.11	106.71
14	HA	1212	CLA	C4A-NA-C1A	5.36	109.11	106.71
14	bA	1235	CLA	O2A-CGA-O1A	-5.36	109.87	123.56
14	HA	1021	CLA	O2D-CGD-CBD	5.36	120.63	111.25
14	HA	1235	CLA	O2A-CGA-O1A	-5.36	109.87	123.56
18	MA	4021	BCR	C15-C14-C13	-5.36	119.67	127.31
18	AA	4001	BCR	C28-C27-C26	-5.36	104.67	113.99
14	bA	1021	CLA	O2D-CGD-CBD	5.36	120.62	111.25
14	lA	1502	CLA	C1C-C2C-C3C	-5.35	101.28	106.95
14	aA	1113	CLA	C1C-C2C-C3C	-5.35	101.28	106.95
14	bA	1218	CLA	C1C-C2C-C3C	-5.35	101.28	106.95
14	aA	1128	CLA	C4A-NA-C1A	5.35	109.11	106.71
14	bA	1232	CLA	C1C-C2C-C3C	-5.35	101.28	106.95
14	BA	1023	CLA	O2D-CGD-CBD	5.35	120.61	111.25
18	GA	4001	BCR	C28-C27-C26	-5.35	104.69	113.99
14	aA	1111	CLA	O2A-C1-C2	5.34	122.68	108.64
14	HA	1223	CLA	C1C-C2C-C3C	-5.34	101.29	106.95
14	aA	1117	CLA	C4A-NA-C1A	5.34	109.11	106.71
14	HA	1201	CLA	C1C-C2C-C3C	-5.34	101.29	106.95
14	GA	1128	CLA	C4A-NA-C1A	5.34	109.11	106.71
18	VA	4021	BCR	C15-C14-C13	-5.34	119.69	127.31
18	aA	4011	BCR	C7-C8-C9	-5.34	118.19	126.21
14	BA	1201	CLA	C1C-C2C-C3C	-5.34	101.29	106.95
14	aA	1131	CLA	C1C-C2C-C3C	-5.34	101.29	106.95
14	GA	1113	CLA	C1C-C2C-C3C	-5.33	101.30	106.95
14	bA	1233	CLA	C1C-C2C-C3C	-5.33	101.30	106.95
14	GA	1128	CLA	CMB-C2B-C3B	5.33	134.80	124.80
14	BA	1235	CLA	O2A-CGA-O1A	-5.33	109.93	123.56
18	aA	4001	BCR	C28-C27-C26	-5.33	104.71	113.99
14	XA	1701	CLA	C4A-NA-C1A	5.33	109.10	106.71
14	aA	1128	CLA	CMB-C2B-C3B	5.33	134.80	124.80
14	GA	1116	CLA	C1C-C2C-C3C	-5.33	101.30	106.95
14	AA	1128	CLA	CMB-C2B-C3B	5.33	134.79	124.80
18	AA	4011	BCR	C7-C8-C9	-5.33	118.21	126.21
14	bA	1023	CLA	O2D-CGD-CBD	5.33	120.57	111.25
14	bA	1201	CLA	C1C-C2C-C3C	-5.32	101.31	106.95
14	GA	1111	CLA	O2A-C1-C2	5.32	122.61	108.64
14	bA	1223	CLA	C1C-C2C-C3C	-5.32	101.31	106.95
14	BA	1209	CLA	C1C-C2C-C3C	-5.32	101.32	106.95
14	AA	1111	CLA	O2A-C1-C2	5.31	122.60	108.64

Continued on next page...

PRELIMINARY VALIDATION REPORT

Continued from previous page...

Mol	Chain	Res	Type	Atoms	Z	Observed(^{o})	Ideal(^{o})
14	GA	1117	CLA	C1C-C2C-C3C	-5.31	101.32	106.95
14	AA	1116	CLA	C1C-C2C-C3C	-5.31	101.32	106.95
14	BA	1223	CLA	C1C-C2C-C3C	-5.31	101.32	106.95
14	aA	1116	CLA	C1C-C2C-C3C	-5.31	101.32	106.95
14	GA	1138	CLA	C4A-NA-C1A	5.31	109.09	106.71
14	aA	1136	CLA	O2A-C1-C2	5.31	122.59	108.64
14	AA	1132	CLA	C1C-C2C-C3C	-5.31	101.32	106.95
14	HA	1233	CLA	C1C-C2C-C3C	-5.31	101.33	106.95
18	GA	4011	BCR	C7-C8-C9	-5.31	118.24	126.21
14	BA	1233	CLA	C1C-C2C-C3C	-5.31	101.33	106.95
14	AA	1136	CLA	O2A-C1-C2	5.31	122.58	108.64
14	GA	1136	CLA	O2A-C1-C2	5.30	122.58	108.64
14	AA	1138	CLA	C4A-NA-C1A	5.30	109.09	106.71
14	bA	1203	CLA	C4A-NA-C1A	5.30	109.09	106.71
14	aA	1115	CLA	O2D-CGD-CBD	5.29	120.52	111.25
14	BA	1205	CLA	C2C-C1C-NC	5.29	114.93	109.97
14	HA	1209	CLA	C1C-C2C-C3C	-5.29	101.34	106.95
14	bA	1227	CLA	C1C-C2C-C3C	-5.29	101.34	106.95
14	aA	1013	CLA	O2D-CGD-CBD	5.29	120.51	111.25
14	aA	1138	CLA	C4A-NA-C1A	5.29	109.08	106.71
14	BA	1202	CLA	C4A-NA-C1A	5.29	109.08	106.71
14	HA	1234	CLA	O2D-CGD-CBD	5.29	120.51	111.25
14	HA	1227	CLA	C1C-C2C-C3C	-5.29	101.34	106.95
14	bA	1228	CLA	C4A-NA-C1A	5.29	109.08	106.71
14	GA	1132	CLA	C1C-C2C-C3C	-5.29	101.35	106.95
14	AA	1013	CLA	O2D-CGD-CBD	5.29	120.50	111.25
14	AA	1117	CLA	C1C-C2C-C3C	-5.29	101.35	106.95
14	AA	1117	CLA	C4A-NA-C1A	5.28	109.08	106.71
14	bA	1231	CLA	CMB-C2B-C3B	5.28	134.70	124.80
18	GA	4002	BCR	C34-C9-C10	-5.28	115.53	122.92
14	aA	1117	CLA	C1C-C2C-C3C	-5.28	101.36	106.95
14	AA	1123	CLA	C4A-NA-C1A	5.28	109.08	106.71
14	aA	1012	CLA	C1C-C2C-C3C	-5.28	101.36	106.95
14	bA	1202	CLA	C4A-NA-C1A	5.27	109.08	106.71
14	AA	1115	CLA	O2D-CGD-CBD	5.27	120.48	111.25
14	bA	1203	CLA	O2A-C1-C2	5.27	122.49	108.64
14	bA	1209	CLA	C1C-C2C-C3C	-5.27	101.36	106.95
14	BA	1234	CLA	O2D-CGD-CBD	5.27	120.48	111.25
14	HA	1221	CLA	O2A-C1-C2	5.27	122.49	108.64
14	bA	1234	CLA	O2D-CGD-CBD	5.27	120.48	111.25
14	GA	1115	CLA	O2D-CGD-CBD	5.27	120.47	111.25
14	AA	1119	CLA	C4A-NA-C1A	5.27	109.08	106.71

Continued on next page...

PRELIMINARY VALIDATION REPORT

Continued from previous page...

Mol	Chain	Res	Type	Atoms	Z	Observed(°)	Ideal(°)
14	HA	1233	CLA	O2D-CGD-CBD	5.27	120.47	111.25
14	BA	1221	CLA	O2A-C1-C2	5.27	122.48	108.64
14	bA	1233	CLA	O2D-CGD-CBD	5.27	120.47	111.25
14	AA	1012	CLA	C1C-C2C-C3C	-5.27	101.37	106.95
14	BA	1228	CLA	C4A-NA-C1A	5.26	109.07	106.71
14	LA	1501	CLA	C1C-C2C-C3C	-5.26	101.37	106.95
14	HA	1231	CLA	CMB-C2B-C3B	5.26	134.67	124.80
14	AA	1102	CLA	C1C-C2C-C3C	-5.26	101.37	106.95
14	bA	1221	CLA	O2A-C1-C2	5.26	122.46	108.64
14	BA	1231	CLA	CMB-C2B-C3B	5.26	134.67	124.80
14	GA	1013	CLA	O2D-CGD-CBD	5.26	120.46	111.25
18	aA	4002	BCR	C34-C9-C10	-5.26	115.56	122.92
14	HA	1203	CLA	O2A-C1-C2	5.26	122.45	108.64
14	AA	1102	CLA	C4A-NA-C1A	5.26	109.07	106.71
14	BA	1203	CLA	O2A-C1-C2	5.26	122.45	108.64
14	bA	1212	CLA	C4A-NA-C1A	5.25	109.07	106.71
14	GA	1102	CLA	C1C-C2C-C3C	-5.25	101.38	106.95
14	aA	1107	CLA	C1C-C2C-C3C	-5.25	101.39	106.95
14	aA	1102	CLA	C1C-C2C-C3C	-5.25	101.39	106.95
14	LA	1503	CLA	O2D-CGD-CBD	5.25	120.43	111.25
14	GA	1012	CLA	C1C-C2C-C3C	-5.25	101.39	106.95
14	aA	1132	CLA	C1C-C2C-C3C	-5.25	101.39	106.95
14	BA	1227	CLA	C1C-C2C-C3C	-5.25	101.39	106.95
14	BA	1214	CLA	O2A-C1-C2	5.24	122.42	108.64
14	aA	1104	CLA	O2D-CGD-CBD	5.24	120.43	111.25
14	BA	1233	CLA	O2D-CGD-CBD	5.24	120.43	111.25
14	bA	1205	CLA	C2C-C1C-NC	5.24	114.89	109.97
14	aA	1140	CLA	C4A-NA-C1A	5.24	109.06	106.71
14	UA	1501	CLA	C1C-C2C-C3C	-5.24	101.39	106.95
14	AA	1104	CLA	O2D-CGD-CBD	5.24	120.42	111.25
14	GA	1108	CLA	C4A-NA-C1A	5.24	109.06	106.71
14	GA	1140	CLA	C4A-NA-C1A	5.24	109.06	106.71
14	GA	1102	CLA	C4A-NA-C1A	5.24	109.06	106.71
18	AA	4002	BCR	C34-C9-C10	-5.24	115.58	122.92
14	GA	1132	CLA	O2A-C1-C2	5.24	122.40	108.64
14	lA	1503	CLA	O2D-CGD-CBD	5.24	120.42	111.25
14	lA	1501	CLA	C1C-C2C-C3C	-5.24	101.40	106.95
14	bA	1216	CLA	C4A-NA-C1A	5.24	109.06	106.71
14	HA	1216	CLA	C4A-NA-C1A	5.23	109.06	106.71
14	HA	1226	CLA	C1C-C2C-C3C	-5.23	101.40	106.95
14	AA	1110	CLA	C4A-NA-C1A	5.23	109.06	106.71
14	AA	1132	CLA	O2A-C1-C2	5.23	122.38	108.64

Continued on next page...

PRELIMINARY VALIDATION REPORT

Continued from previous page...

Mol	Chain	Res	Type	Atoms	Z	Observed(°)	Ideal(°)
14	HA	1205	CLA	C2C-C1C-NC	5.23	114.87	109.97
14	GA	1104	CLA	O2D-CGD-CBD	5.23	120.40	111.25
14	UA	1503	CLA	O2D-CGD-CBD	5.23	120.40	111.25
14	HA	1214	CLA	O2A-C1-C2	5.23	122.37	108.64
14	BA	1021	CLA	C1C-C2C-C3C	-5.23	101.41	106.95
14	bA	1210	CLA	CAC-C3C-C4C	5.22	131.64	124.82
14	GA	1108	CLA	O2D-CGD-CBD	5.22	120.39	111.25
14	bA	1214	CLA	O2A-C1-C2	5.22	122.35	108.64
14	AA	1107	CLA	C1C-C2C-C3C	-5.22	101.42	106.95
14	bA	1231	CLA	O2D-CGD-CBD	5.21	120.38	111.25
14	HA	1231	CLA	O2D-CGD-CBD	5.21	120.38	111.25
14	aA	1124	CLA	C1C-C2C-C3C	-5.21	101.42	106.95
14	GA	1107	CLA	C1C-C2C-C3C	-5.21	101.42	106.95
14	GA	1012	CLA	O2D-CGD-CBD	5.21	120.38	111.25
14	LA	1503	CLA	C4A-NA-C1A	5.21	109.05	106.71
14	aA	1103	CLA	O2A-C1-C2	5.21	122.33	108.64
14	aA	1012	CLA	O2D-CGD-CBD	5.21	120.37	111.25
14	aA	1132	CLA	O2A-C1-C2	5.21	122.33	108.64
14	BA	1210	CLA	CAC-C3C-C4C	5.21	131.63	124.82
14	BA	1231	CLA	O2D-CGD-CBD	5.21	120.36	111.25
14	BA	1226	CLA	C1C-C2C-C3C	-5.20	101.44	106.95
18	HA	4012	BCR	C1-C6-C5	-5.20	115.32	122.59
14	AA	1126	CLA	C1C-C2C-C3C	-5.20	101.44	106.95
14	HA	1021	CLA	C1C-C2C-C3C	-5.20	101.44	106.95
14	HA	1210	CLA	CAC-C3C-C4C	5.20	131.61	124.82
14	AA	1124	CLA	C1C-C2C-C3C	-5.20	101.44	106.95
14	bA	1021	CLA	C1C-C2C-C3C	-5.20	101.44	106.95
14	aA	1107	CLA	C4A-NA-C1A	5.20	109.04	106.71
14	AA	1107	CLA	C4A-NA-C1A	5.20	109.04	106.71
14	BA	1216	CLA	C4A-NA-C1A	5.19	109.04	106.71
18	BA	4012	BCR	C1-C6-C5	-5.19	115.33	122.59
14	GA	1110	CLA	C4A-NA-C1A	5.19	109.04	106.71
14	AA	1103	CLA	O2A-C1-C2	5.19	122.27	108.64
14	aA	1108	CLA	C4A-NA-C1A	5.18	109.04	106.71
14	bA	1226	CLA	C1C-C2C-C3C	-5.18	101.46	106.95
14	GA	1135	CLA	C1C-C2C-C3C	-5.18	101.46	106.95
18	bA	4012	BCR	C1-C6-C5	-5.18	115.35	122.59
14	aA	1108	CLA	O2D-CGD-CBD	5.18	120.32	111.25
14	GA	1123	CLA	C4A-NA-C1A	5.18	109.04	106.71
14	GA	1103	CLA	O2A-C1-C2	5.18	122.25	108.64
14	aA	1126	CLA	C1C-C2C-C3C	-5.18	101.46	106.95
14	HA	1234	CLA	C4A-NA-C1A	5.18	109.03	106.71

Continued on next page...

PRELIMINARY VALIDATION REPORT

Continued from previous page...

Mol	Chain	Res	Type	Atoms	Z	Observed(°)	Ideal(°)
14	bA	1213	CLA	C4A-NA-C1A	5.17	109.03	106.71
14	AA	1104	CLA	O2A-C1-C2	5.17	122.23	108.64
14	aA	1110	CLA	C4A-NA-C1A	5.17	109.03	106.71
14	AA	1138	CLA	C1C-C2C-C3C	-5.17	101.47	106.95
14	GA	1124	CLA	C1C-C2C-C3C	-5.17	101.47	106.95
14	aA	1127	CLA	C1C-C2C-C3C	-5.17	101.47	106.95
14	GA	1104	CLA	O2A-C1-C2	5.17	122.21	108.64
14	AA	1108	CLA	O2D-CGD-CBD	5.17	120.29	111.25
14	GA	1126	CLA	C1C-C2C-C3C	-5.16	101.48	106.95
14	AA	1012	CLA	O2D-CGD-CBD	5.16	120.29	111.25
14	GA	1138	CLA	C1C-C2C-C3C	-5.16	101.48	106.95
14	GA	1135	CLA	O2D-CGD-CBD	5.16	120.28	111.25
14	aA	1104	CLA	O2A-C1-C2	5.15	122.18	108.64
14	GA	1127	CLA	C1C-C2C-C3C	-5.15	101.49	106.95
14	BA	1211	CLA	C1C-C2C-C3C	-5.15	101.49	106.95
14	HA	1229	CLA	CAA-C2A-C3A	-5.15	98.68	112.78
14	aA	1123	CLA	C4A-NA-C1A	5.15	109.02	106.71
14	lA	1503	CLA	C4A-NA-C1A	5.15	109.02	106.71
14	AA	1136	CLA	C1C-C2C-C3C	-5.15	101.49	106.95
14	aA	1135	CLA	O2D-CGD-CBD	5.15	120.26	111.25
14	BA	1229	CLA	CAA-C2A-C3A	-5.15	98.68	112.78
14	bA	1229	CLA	CAA-C2A-C3A	-5.15	98.68	112.78
14	UA	1501	CLA	CAA-C2A-C3A	-5.15	98.68	112.78
14	aA	1139	CLA	O2D-CGD-CBD	5.15	120.26	111.25
14	BA	1234	CLA	C4A-NA-C1A	5.14	109.02	106.71
14	lA	1501	CLA	CAA-C2A-C3A	-5.14	98.69	112.78
14	aA	1138	CLA	C1C-C2C-C3C	-5.14	101.50	106.95
14	HA	1235	CLA	C1C-C2C-C3C	-5.14	101.50	106.95
14	AA	1127	CLA	C1C-C2C-C3C	-5.14	101.50	106.95
14	bA	1211	CLA	C1C-C2C-C3C	-5.14	101.50	106.95
14	bA	1213	CLA	O2A-CGA-O1A	-5.14	110.43	123.56
14	UA	1503	CLA	C4A-NA-C1A	5.14	109.02	106.71
14	HA	1217	CLA	O2A-C1-C2	5.14	122.14	108.64
14	LA	1501	CLA	CAA-C2A-C3A	-5.14	98.71	112.78
14	AA	1110	CLA	C1C-C2C-C3C	-5.14	101.51	106.95
14	AA	1135	CLA	C1C-C2C-C3C	-5.14	101.51	106.95
14	BA	1217	CLA	O2A-C1-C2	5.13	122.13	108.64
14	AA	1139	CLA	O2D-CGD-CBD	5.13	120.23	111.25
14	GA	1107	CLA	C4A-NA-C1A	5.13	109.01	106.71
14	BA	1235	CLA	C1C-C2C-C3C	-5.13	101.51	106.95
14	HA	1239	CLA	O2A-CGA-O1A	-5.13	110.45	123.56
14	bA	1208	CLA	O2A-C1-C2	5.13	122.11	108.64

Continued on next page...

PRELIMINARY VALIDATION REPORT

Continued from previous page...

Mol	Chain	Res	Type	Atoms	Z	Observed(°)	Ideal(°)
14	GA	1110	CLA	C1C-C2C-C3C	-5.13	101.52	106.95
14	bA	1235	CLA	C1C-C2C-C3C	-5.13	101.52	106.95
14	bA	1202	CLA	C1C-C2C-C3C	-5.13	101.52	106.95
14	HA	1208	CLA	O2A-C1-C2	5.13	122.11	108.64
14	BA	1208	CLA	O2A-C1-C2	5.13	122.11	108.64
14	BA	1213	CLA	O2A-CGA-O1A	-5.12	110.47	123.56
18	IA	4020	BCR	C20-C19-C18	5.12	140.81	126.42
14	aA	1136	CLA	C1C-C2C-C3C	-5.12	101.52	106.95
14	HA	1213	CLA	O2A-CGA-O1A	-5.12	110.47	123.56
14	HA	1202	CLA	C1C-C2C-C3C	-5.12	101.52	106.95
14	bA	1217	CLA	O2A-C1-C2	5.12	122.09	108.64
14	BA	1221	CLA	O2D-CGD-CBD	5.12	120.21	111.25
14	HA	1221	CLA	O2D-CGD-CBD	5.12	120.21	111.25
14	aA	1110	CLA	C1C-C2C-C3C	-5.12	101.52	106.95
14	GA	1139	CLA	O2D-CGD-CBD	5.12	120.21	111.25
14	HA	1211	CLA	C1C-C2C-C3C	-5.12	101.53	106.95
14	HA	1206	CLA	C1C-C2C-C3C	-5.11	101.53	106.95
18	RA	4020	BCR	C20-C19-C18	5.11	140.78	126.42
14	aA	1119	CLA	C4A-NA-C1A	5.11	109.00	106.71
14	BA	1223	CLA	C4A-NA-C1A	5.11	109.00	106.71
14	BA	1239	CLA	O2A-CGA-O1A	-5.11	110.49	123.56
14	GA	1135	CLA	O2A-C1-C2	5.11	122.07	108.64
14	AA	1127	CLA	C4A-NA-C1A	5.11	109.00	106.71
14	HA	1213	CLA	C4A-NA-C1A	5.11	109.00	106.71
14	BA	1202	CLA	C1C-C2C-C3C	-5.11	101.53	106.95
14	bA	1221	CLA	O2D-CGD-CBD	5.11	120.19	111.25
14	AA	1135	CLA	O2D-CGD-CBD	5.11	120.19	111.25
14	bA	1206	CLA	C1C-C2C-C3C	-5.11	101.54	106.95
14	aA	1108	CLA	C1C-C2C-C3C	-5.11	101.54	106.95
14	HA	1222	CLA	O2A-CGA-O1A	-5.10	110.52	123.56
18	LA	4022	BCR	C24-C23-C22	-5.10	118.54	126.21
18	iA	4020	BCR	C20-C19-C18	5.10	140.75	126.42
18	UA	4022	BCR	C24-C23-C22	-5.10	118.55	126.21
14	BA	1232	CLA	C4A-NA-C1A	5.10	109.00	106.71
14	bA	1208	CLA	O2A-CGA-O1A	-5.10	110.53	123.56
14	AA	1127	CLA	O2D-CGD-CBD	5.10	120.17	111.25
14	AA	1135	CLA	O2A-C1-C2	5.10	122.03	108.64
14	BA	1222	CLA	O2A-CGA-O1A	-5.10	110.54	123.56
14	aA	1135	CLA	O2A-C1-C2	5.09	122.03	108.64
18	lA	4022	BCR	C24-C23-C22	-5.09	118.56	126.21
14	bA	1234	CLA	C4A-NA-C1A	5.09	109.00	106.71
14	HA	1208	CLA	O2A-CGA-O1A	-5.09	110.54	123.56

Continued on next page...

PRELIMINARY VALIDATION REPORT

Continued from previous page...

Mol	Chain	Res	Type	Atoms	Z	Observed(^{o})	Ideal(^{o})
14	bA	1239	CLA	O2A-CGA-O1A	-5.09	110.55	123.56
14	GA	1127	CLA	O2D-CGD-CBD	5.09	120.16	111.25
14	bA	1222	CLA	O2A-CGA-O1A	-5.09	110.55	123.56
14	GA	1119	CLA	C4A-NA-C1A	5.09	108.99	106.71
14	GA	1136	CLA	C1C-C2C-C3C	-5.09	101.56	106.95
14	AA	1133	CLA	O2A-C1-C2	5.09	122.01	108.64
14	BA	1206	CLA	C1C-C2C-C3C	-5.09	101.56	106.95
14	AA	1108	CLA	C1C-C2C-C3C	-5.09	101.56	106.95
14	GA	1133	CLA	O2A-C1-C2	5.08	122.00	108.64
14	BA	1208	CLA	O2A-CGA-O1A	-5.08	110.57	123.56
14	HA	1226	CLA	O2A-CGA-O1A	-5.08	110.57	123.56
14	BA	1213	CLA	C4A-NA-C1A	5.08	108.99	106.71
14	AA	1115	CLA	C1C-C2C-C3C	-5.08	101.57	106.95
14	aA	1130	CLA	C1C-C2C-C3C	-5.08	101.57	106.95
14	aA	1133	CLA	O2A-C1-C2	5.08	121.98	108.64
14	GA	1107	CLA	O2D-CGD-CBD	5.08	120.14	111.25
14	HA	1223	CLA	C4A-NA-C1A	5.08	108.99	106.71
14	GA	1108	CLA	C1C-C2C-C3C	-5.08	101.57	106.95
14	bA	1226	CLA	O2A-CGA-O1A	-5.08	110.58	123.56
14	GA	1128	CLA	C1C-C2C-C3C	-5.08	101.57	106.95
14	HA	1213	CLA	C1C-C2C-C3C	-5.08	101.57	106.95
14	AA	1111	CLA	O2A-CGA-O1A	-5.08	110.59	123.56
14	aA	1127	CLA	O2D-CGD-CBD	5.07	120.13	111.25
14	aA	1135	CLA	C1C-C2C-C3C	-5.07	101.57	106.95
14	bA	1216	CLA	C1C-C2C-C3C	-5.07	101.57	106.95
14	BA	1229	CLA	O2A-CGA-O1A	-5.07	110.59	123.56
14	aA	1111	CLA	O2A-CGA-O1A	-5.07	110.59	123.56
14	HA	1229	CLA	O2A-CGA-O1A	-5.07	110.61	123.56
14	BA	1226	CLA	CMB-C2B-C3B	5.07	134.30	124.80
14	aA	1128	CLA	C1C-C2C-C3C	-5.07	101.58	106.95
14	bA	1229	CLA	O2A-CGA-O1A	-5.06	110.62	123.56
14	BA	1226	CLA	O2A-CGA-O1A	-5.06	110.62	123.56
14	bA	1226	CLA	CMB-C2B-C3B	5.06	134.29	124.80
14	GA	1130	CLA	C1C-C2C-C3C	-5.06	101.59	106.95
14	GA	1111	CLA	O2A-CGA-O1A	-5.06	110.63	123.56
14	AA	1128	CLA	C1C-C2C-C3C	-5.06	101.59	106.95
14	UA	1503	CLA	C1C-C2C-C3C	-5.06	101.59	106.95
14	GA	1115	CLA	C1C-C2C-C3C	-5.06	101.59	106.95
14	HA	1226	CLA	CMB-C2B-C3B	5.06	134.28	124.80
14	AA	1130	CLA	C1C-C2C-C3C	-5.05	101.59	106.95
14	bA	1225	CLA	C4A-NA-C1A	5.05	108.98	106.71
14	lA	1503	CLA	C1C-C2C-C3C	-5.05	101.60	106.95

Continued on next page...

PRELIMINARY VALIDATION REPORT

Continued from previous page...

Mol	Chain	Res	Type	Atoms	Z	Observed(^{o})	Ideal(^{o})
14	AA	1107	CLA	O2D-CGD-CBD	5.05	120.09	111.25
14	aA	1115	CLA	C1C-C2C-C3C	-5.05	101.60	106.95
14	bA	1213	CLA	C1C-C2C-C3C	-5.05	101.60	106.95
14	aA	1106	CLA	C1C-C2C-C3C	-5.04	101.61	106.95
14	GA	1115	CLA	O2A-CGA-O1A	-5.04	110.68	123.56
14	BA	1213	CLA	C1C-C2C-C3C	-5.04	101.61	106.95
14	aA	1107	CLA	O2D-CGD-CBD	5.04	120.06	111.25
14	bA	1204	CLA	O2A-CGA-O1A	-5.03	110.70	123.56
14	BA	1222	CLA	C4A-NA-C1A	5.03	108.97	106.71
14	aA	1117	CLA	O2D-CGD-CBD	5.03	120.05	111.25
14	AA	1115	CLA	O2A-CGA-O1A	-5.03	110.71	123.56
14	bA	1209	CLA	C4A-NA-C1A	5.03	108.97	106.71
14	LA	1503	CLA	C1C-C2C-C3C	-5.03	101.62	106.95
14	HA	1204	CLA	O2A-CGA-O1A	-5.03	110.72	123.56
14	GA	1117	CLA	O2D-CGD-CBD	5.02	120.04	111.25
14	BA	1204	CLA	O2A-CGA-O1A	-5.02	110.72	123.56
14	BA	1220	CLA	C1C-C2C-C3C	-5.02	101.63	106.95
14	GA	1105	CLA	O2D-CGD-CBD	5.02	120.04	111.25
14	aA	1140	CLA	C1C-C2C-C3C	-5.02	101.63	106.95
14	GA	1140	CLA	C1C-C2C-C3C	-5.02	101.63	106.95
14	aA	1115	CLA	O2A-CGA-O1A	-5.02	110.74	123.56
14	AA	1117	CLA	O2D-CGD-CBD	5.01	120.03	111.25
14	AA	1105	CLA	O2D-CGD-CBD	5.01	120.03	111.25
14	HA	1209	CLA	C4A-NA-C1A	5.01	108.96	106.71
14	bA	1232	CLA	C4A-NA-C1A	5.01	108.96	106.71
14	bA	1219	CLA	C1C-C2C-C3C	-5.01	101.64	106.95
14	GA	1106	CLA	C1C-C2C-C3C	-5.01	101.64	106.95
14	HA	1219	CLA	C1C-C2C-C3C	-5.01	101.64	106.95
14	AA	1117	CLA	O2A-C1-C2	5.01	121.80	108.64
14	AA	1106	CLA	C1C-C2C-C3C	-5.01	101.64	106.95
14	GA	1117	CLA	O2A-C1-C2	5.01	121.79	108.64
14	aA	1105	CLA	O2D-CGD-CBD	5.01	120.01	111.25
14	aA	1117	CLA	O2A-C1-C2	5.01	121.79	108.64
18	BA	4004	BCR	C3-C4-C5	-5.00	105.28	113.99
14	HA	1216	CLA	C1C-C2C-C3C	-5.00	101.65	106.95
14	bA	1220	CLA	C1C-C2C-C3C	-5.00	101.65	106.95
14	aA	1127	CLA	C4A-NA-C1A	5.00	108.95	106.71
14	bA	1233	CLA	C4A-NA-C1A	5.00	108.95	106.71
18	bA	4004	BCR	C3-C4-C5	-4.99	105.30	113.99
14	AA	1140	CLA	C1C-C2C-C3C	-4.99	101.66	106.95
14	BA	1235	CLA	CMB-C2B-C3B	4.99	134.15	124.80
18	bA	4004	BCR	C7-C8-C9	-4.99	118.72	126.21

Continued on next page...

PRELIMINARY VALIDATION REPORT

Continued from previous page...

Mol	Chain	Res	Type	Atoms	Z	Observed(^{o})	Ideal(^{o})
18	HA	4004	BCR	C3-C4-C5	-4.98	105.32	113.99
14	BA	1216	CLA	C1C-C2C-C3C	-4.98	101.67	106.95
18	HA	4004	BCR	C7-C8-C9	-4.98	118.73	126.21
14	BA	1219	CLA	C1C-C2C-C3C	-4.98	101.67	106.95
14	GA	1125	CLA	O2A-CGA-O1A	-4.98	110.84	123.56
14	AA	1129	CLA	C1C-C2C-C3C	-4.98	101.68	106.95
14	AA	1125	CLA	O2A-CGA-O1A	-4.97	110.85	123.56
18	AA	4011	BCR	C20-C19-C18	4.97	140.38	126.42
18	BA	4004	BCR	C7-C8-C9	-4.97	118.75	126.21
18	GA	4011	BCR	C20-C19-C18	4.97	140.37	126.42
14	BA	1228	CLA	O2D-CGD-CBD	4.97	119.94	111.25
14	bA	1223	CLA	C4A-NA-C1A	4.96	108.94	106.71
14	GA	1129	CLA	C1C-C2C-C3C	-4.96	101.69	106.95
14	HA	1220	CLA	C1C-C2C-C3C	-4.96	101.69	106.95
14	lA	1501	CLA	O2A-CGA-O1A	-4.96	110.88	123.56
14	aA	1125	CLA	O2A-CGA-O1A	-4.96	110.88	123.56
14	bA	1235	CLA	CMB-C2B-C3B	4.96	134.10	124.80
14	aA	1129	CLA	C1C-C2C-C3C	-4.96	101.70	106.95
14	HA	1233	CLA	C4A-NA-C1A	4.95	108.93	106.71
18	aA	4011	BCR	C20-C19-C18	4.95	140.33	126.42
14	HA	1231	CLA	O2A-CGA-O1A	-4.95	110.91	123.56
14	BA	1231	CLA	O2A-CGA-O1A	-4.95	110.91	123.56
14	xA	1701	CLA	O2D-CGD-CBD	4.95	119.91	111.25
14	BA	1209	CLA	C4A-NA-C1A	4.95	108.93	106.71
14	HA	1235	CLA	CMB-C2B-C3B	4.95	134.08	124.80
14	UA	1501	CLA	O2A-CGA-O1A	-4.95	110.92	123.56
14	HA	1231	CLA	C4A-NA-C1A	4.94	108.93	106.71
14	HA	1222	CLA	C4A-NA-C1A	4.94	108.93	106.71
14	HA	1232	CLA	C4A-NA-C1A	4.94	108.93	106.71
14	WA	1701	CLA	O2D-CGD-CBD	4.94	119.90	111.25
14	LA	1501	CLA	O2A-CGA-O1A	-4.94	110.94	123.56
14	bA	1231	CLA	O2A-CGA-O1A	-4.94	110.94	123.56
14	AA	1104	CLA	O2A-CGA-O1A	-4.94	110.94	123.56
14	aA	1013	CLA	O2A-CGA-O1A	-4.93	110.97	123.56
14	BA	1216	CLA	O2D-CGD-CBD	4.93	119.88	111.25
14	HA	1225	CLA	C4A-NA-C1A	4.93	108.92	106.71
18	HA	4010	BCR	C24-C23-C22	-4.93	118.81	126.21
14	AA	1101	CLA	C4A-NA-C1A	4.92	108.92	106.71
14	HA	1228	CLA	O2D-CGD-CBD	4.92	119.87	111.25
14	HA	1216	CLA	O2D-CGD-CBD	4.92	119.87	111.25
14	XA	1701	CLA	O2D-CGD-CBD	4.92	119.87	111.25
14	aA	1105	CLA	C1C-C2C-C3C	-4.92	101.73	106.95

Continued on next page...

PRELIMINARY VALIDATION REPORT

Continued from previous page...

Mol	Chain	Res	Type	Atoms	Z	Observed(^{o})	Ideal(^{o})
14	BA	1238	CLA	O2A-CGA-O1A	-4.92	110.98	123.56
14	bA	1222	CLA	C4A-NA-C1A	4.92	108.92	106.71
18	bA	4010	BCR	C24-C23-C22	-4.92	118.82	126.21
14	bA	1238	CLA	O2A-CGA-O1A	-4.92	110.99	123.56
14	AA	1136	CLA	O2A-CGA-CBA	4.92	127.72	111.93
14	GA	1013	CLA	O2A-CGA-O1A	-4.92	110.99	123.56
14	GA	1104	CLA	O2A-CGA-O1A	-4.92	111.00	123.56
14	AA	1109	CLA	O2D-CGD-CBD	4.91	119.85	111.25
14	HA	1238	CLA	O2A-CGA-O1A	-4.91	111.00	123.56
14	aA	1128	CLA	O2A-CGA-O1A	-4.91	111.00	123.56
14	aA	1104	CLA	O2A-CGA-O1A	-4.91	111.01	123.56
14	bA	1216	CLA	O2D-CGD-CBD	4.91	119.85	111.25
14	bA	1228	CLA	O2D-CGD-CBD	4.91	119.84	111.25
14	GA	1127	CLA	C4A-NA-C1A	4.91	108.91	106.71
14	aA	1136	CLA	O2A-CGA-CBA	4.91	127.69	111.93
18	BA	4010	BCR	C24-C23-C22	-4.91	118.84	126.21
14	GA	1136	CLA	O2A-CGA-CBA	4.91	127.68	111.93
14	GA	1128	CLA	O2A-CGA-O1A	-4.90	111.03	123.56
14	AA	1013	CLA	O2A-CGA-O1A	-4.90	111.03	123.56
14	AA	1128	CLA	O2A-CGA-O1A	-4.90	111.03	123.56
14	aA	1101	CLA	C4A-NA-C1A	4.90	108.91	106.71
14	GA	1109	CLA	O2D-CGD-CBD	4.90	119.82	111.25
18	iA	4018	BCR	C24-C23-C22	-4.90	118.86	126.21
14	BA	1225	CLA	C4A-NA-C1A	4.90	108.91	106.71
14	AA	1139	CLA	C4A-NA-C1A	4.89	108.91	106.71
14	GA	1101	CLA	C4A-NA-C1A	4.89	108.90	106.71
14	bA	1231	CLA	C4A-NA-C1A	4.89	108.90	106.71
14	AA	1105	CLA	C1C-C2C-C3C	-4.89	101.77	106.95
14	GA	1105	CLA	C1C-C2C-C3C	-4.88	101.78	106.95
14	aA	1109	CLA	O2D-CGD-CBD	4.88	119.79	111.25
14	BA	1233	CLA	C4A-NA-C1A	4.88	108.90	106.71
14	GA	1123	CLA	O2A-C1-C2	4.86	121.42	108.64
14	aA	1124	CLA	O2D-CGD-CBD	4.86	119.76	111.25
14	aA	1135	CLA	O2A-CGA-O1A	-4.86	111.14	123.56
14	HA	1223	CLA	O2A-C1-C2	4.86	121.41	108.64
14	AA	1135	CLA	O2A-CGA-O1A	-4.86	111.14	123.56
18	IA	4018	BCR	C24-C23-C22	-4.86	118.91	126.21
14	aA	1123	CLA	O2A-C1-C2	4.86	121.40	108.64
14	BA	1223	CLA	O2A-C1-C2	4.85	121.39	108.64
14	AA	1124	CLA	O2D-CGD-CBD	4.85	119.74	111.25
14	AA	1123	CLA	O2A-C1-C2	4.85	121.39	108.64
18	RA	4018	BCR	C24-C23-C22	-4.85	118.93	126.21

Continued on next page...

PRELIMINARY VALIDATION REPORT

Continued from previous page...

Mol	Chain	Res	Type	Atoms	Z	Observed(°)	Ideal(°)
14	aA	1139	CLA	C1C-C2C-C3C	-4.85	101.81	106.95
14	aA	1119	CLA	O2D-CGD-CBD	4.85	119.74	111.25
14	GA	1129	CLA	C4A-NA-C1A	4.85	108.89	106.71
14	GA	1139	CLA	C1C-C2C-C3C	-4.84	101.82	106.95
14	AA	1116	CLA	O2A-CGA-O1A	-4.84	111.18	123.56
14	GA	1135	CLA	O2A-CGA-O1A	-4.84	111.19	123.56
14	bA	1223	CLA	O2A-C1-C2	4.84	121.36	108.64
14	bA	1202	CLA	O2A-CGA-CBA	4.84	127.47	111.93
14	BA	1202	CLA	O2A-CGA-CBA	4.84	127.47	111.93
14	AA	1119	CLA	O2D-CGD-CBD	4.84	119.72	111.25
14	GA	1124	CLA	O2D-CGD-CBD	4.83	119.71	111.25
14	aA	1116	CLA	O2A-CGA-O1A	-4.83	111.21	123.56
14	BA	1220	CLA	O2D-CGD-CBD	4.83	119.71	111.25
14	GA	1116	CLA	O2A-CGA-O1A	-4.83	111.22	123.56
14	BA	1231	CLA	C4A-NA-C1A	4.83	108.88	106.71
14	HA	1202	CLA	O2A-CGA-CBA	4.83	127.43	111.93
14	AA	1112	CLA	C1C-C2C-C3C	-4.83	101.83	106.95
14	GA	1115	CLA	O2A-C1-C2	4.83	121.32	108.64
14	HA	1220	CLA	O2D-CGD-CBD	4.83	119.70	111.25
14	AA	1139	CLA	C1C-C2C-C3C	-4.82	101.84	106.95
14	aA	1115	CLA	O2A-C1-C2	4.82	121.31	108.64
14	aA	1127	CLA	O2A-CGA-O1A	-4.82	111.24	123.56
14	BA	1226	CLA	C4A-NA-C1A	4.82	108.87	106.71
14	aA	1112	CLA	C1C-C2C-C3C	-4.82	101.85	106.95
14	AA	1103	CLA	O2A-CGA-O1A	-4.82	111.25	123.56
14	AA	1115	CLA	O2A-C1-C2	4.82	121.29	108.64
14	bA	1220	CLA	O2D-CGD-CBD	4.81	119.67	111.25
14	GA	1119	CLA	O2D-CGD-CBD	4.81	119.67	111.25
14	aA	1103	CLA	O2A-CGA-O1A	-4.81	111.27	123.56
13	aA	1011	CL0	CAA-C2A-C3A	-4.81	99.61	112.78
14	GA	1103	CLA	O2A-CGA-O1A	-4.81	111.28	123.56
13	AA	1011	CL0	CAA-C2A-C3A	-4.80	99.62	112.78
14	GA	1127	CLA	O2A-CGA-O1A	-4.80	111.29	123.56
14	GA	1112	CLA	C1C-C2C-C3C	-4.80	101.86	106.95
13	GA	1011	CL0	CAA-C2A-C3A	-4.80	99.63	112.78
14	bA	1231	CLA	C1C-C2C-C3C	-4.80	101.86	106.95
14	AA	1127	CLA	O2A-CGA-O1A	-4.79	111.31	123.56
14	BA	1217	CLA	O2A-CGA-O1A	-4.79	111.31	123.56
14	HA	1236	CLA	C4A-NA-C1A	4.79	108.86	106.71
14	HA	1215	CLA	CMB-C2B-C3B	4.79	133.78	124.80
14	bA	1217	CLA	O2A-CGA-O1A	-4.79	111.32	123.56
14	HA	1217	CLA	O2A-CGA-O1A	-4.79	111.32	123.56

Continued on next page...

PRELIMINARY VALIDATION REPORT

Continued from previous page...

Mol	Chain	Res	Type	Atoms	Z	Observed(^{o})	Ideal(^{o})
14	GA	1116	CLA	C4A-NA-C1A	4.79	108.86	106.71
14	bA	1215	CLA	CMB-C2B-C3B	4.78	133.77	124.80
14	HA	1231	CLA	C1C-C2C-C3C	-4.78	101.88	106.95
14	AA	1135	CLA	C4A-NA-C1A	4.78	108.85	106.71
14	BA	1215	CLA	CMB-C2B-C3B	4.78	133.76	124.80
14	AA	1129	CLA	C4A-NA-C1A	4.78	108.85	106.71
14	BA	1224	CLA	C4A-NA-C1A	4.78	108.85	106.71
14	aA	1118	CLA	O2A-CGA-O1A	-4.77	111.36	123.56
14	AA	1102	CLA	O2A-CGA-O1A	-4.77	111.36	123.56
14	BA	1222	CLA	C1C-C2C-C3C	-4.77	101.89	106.95
14	BA	1231	CLA	C1C-C2C-C3C	-4.77	101.89	106.95
14	bA	1222	CLA	C1C-C2C-C3C	-4.77	101.90	106.95
14	XA	1701	CLA	C1C-C2C-C3C	-4.77	101.90	106.95
14	AA	1103	CLA	C1C-C2C-C3C	-4.77	101.90	106.95
14	aA	1103	CLA	C1C-C2C-C3C	-4.77	101.90	106.95
14	xA	1701	CLA	C1C-C2C-C3C	-4.77	101.90	106.95
18	aA	4002	BCR	C8-C9-C10	4.77	126.25	118.94
14	GA	1102	CLA	O2A-CGA-O1A	-4.76	111.39	123.56
14	aA	1102	CLA	O2A-CGA-O1A	-4.76	111.39	123.56
14	HA	1225	CLA	O2A-CGA-O1A	-4.76	111.40	123.56
18	GA	4002	BCR	C8-C9-C10	4.76	126.24	118.94
14	HA	1216	CLA	O2A-CGA-O1A	-4.76	111.40	123.56
14	BA	1216	CLA	O2A-CGA-O1A	-4.76	111.41	123.56
14	bA	1225	CLA	O2A-CGA-O1A	-4.75	111.41	123.56
14	AA	1118	CLA	O2A-CGA-O1A	-4.75	111.41	123.56
14	AA	1113	CLA	CMB-C2B-C3B	4.75	133.71	124.80
14	WA	1701	CLA	C1C-C2C-C3C	-4.75	101.91	106.95
14	BA	1211	CLA	O2A-CGA-O1A	-4.75	111.42	123.56
14	bA	1216	CLA	O2A-CGA-O1A	-4.75	111.42	123.56
14	aA	1129	CLA	C4A-NA-C1A	4.75	108.84	106.71
14	GA	1103	CLA	C1C-C2C-C3C	-4.75	101.92	106.95
14	bA	1224	CLA	O2A-CGA-O1A	-4.75	111.42	123.56
14	GA	1132	CLA	O2A-CGA-O1A	-4.75	111.42	123.56
14	aA	1139	CLA	C4A-NA-C1A	4.75	108.84	106.71
14	BA	1225	CLA	O2A-CGA-O1A	-4.75	111.43	123.56
14	HA	1206	CLA	O2D-CGD-CBD	4.75	119.56	111.25
14	AA	1124	CLA	C4A-NA-C1A	4.74	108.84	106.71
14	aA	1135	CLA	C4A-NA-C1A	4.74	108.84	106.71
14	GA	1135	CLA	C4A-NA-C1A	4.74	108.84	106.71
14	HA	1222	CLA	C1C-C2C-C3C	-4.74	101.92	106.95
14	GA	1118	CLA	O2A-CGA-O1A	-4.74	111.44	123.56
14	BA	1206	CLA	O2D-CGD-CBD	4.74	119.55	111.25

Continued on next page...

PRELIMINARY VALIDATION REPORT

Continued from previous page...

Mol	Chain	Res	Type	Atoms	Z	Observed(°)	Ideal(°)
14	AA	1130	CLA	OBD-CAD-C3D	-4.74	119.65	128.04
14	HA	1224	CLA	O2A-CGA-O1A	-4.73	111.47	123.56
18	AA	4002	BCR	C8-C9-C10	4.73	126.20	118.94
14	HA	1211	CLA	O2A-CGA-O1A	-4.73	111.47	123.56
14	AA	1119	CLA	O2A-CGA-O1A	-4.73	111.47	123.56
14	bA	1206	CLA	O2D-CGD-CBD	4.73	119.53	111.25
14	GA	1113	CLA	CMB-C2B-C3B	4.73	133.67	124.80
14	BA	1224	CLA	O2A-CGA-O1A	-4.73	111.48	123.56
14	AA	1132	CLA	O2A-CGA-O1A	-4.72	111.49	123.56
14	AA	1123	CLA	O2A-CGA-O1A	-4.72	111.49	123.56
14	GA	1130	CLA	OBD-CAD-C3D	-4.72	119.67	128.04
14	aA	1113	CLA	CMB-C2B-C3B	4.72	133.65	124.80
14	aA	1123	CLA	O2A-CGA-O1A	-4.72	111.50	123.56
14	aA	1130	CLA	OBD-CAD-C3D	-4.72	119.68	128.04
14	BA	1201	CLA	O2A-CGA-CBA	4.72	127.08	111.93
14	bA	1211	CLA	O2A-CGA-O1A	-4.72	111.50	123.56
14	bA	1201	CLA	O2A-CGA-CBA	4.71	127.06	111.93
14	aA	1132	CLA	O2A-CGA-O1A	-4.71	111.52	123.56
14	GA	1140	CLA	O2A-CGA-O1A	-4.71	111.52	123.56
14	aA	1119	CLA	O2A-CGA-O1A	-4.71	111.53	123.56
14	aA	1140	CLA	O2A-C1-C2	4.71	121.01	108.64
14	GA	1139	CLA	C4A-NA-C1A	4.71	108.82	106.71
14	aA	1140	CLA	O2A-CGA-O1A	-4.70	111.54	123.56
14	HA	1223	CLA	O2A-CGA-O1A	-4.70	111.54	123.56
14	AA	1116	CLA	C4A-NA-C1A	4.70	108.82	106.71
14	AA	1102	CLA	CMB-C2B-C3B	4.70	133.62	124.80
14	HA	1201	CLA	O2A-CGA-CBA	4.70	127.03	111.93
14	GA	1123	CLA	O2A-CGA-O1A	-4.70	111.54	123.56
14	GA	1119	CLA	O2A-CGA-O1A	-4.70	111.55	123.56
14	BA	1223	CLA	O2A-CGA-O1A	-4.70	111.55	123.56
14	HA	1212	CLA	C1C-C2C-C3C	-4.70	101.97	106.95
14	aA	1102	CLA	CMB-C2B-C3B	4.70	133.61	124.80
14	BA	1212	CLA	C1C-C2C-C3C	-4.70	101.97	106.95
14	AA	1140	CLA	O2A-CGA-O1A	-4.70	111.56	123.56
14	AA	1140	CLA	O2A-C1-C2	4.69	120.97	108.64
18	BA	4005	BCR	C34-C9-C10	-4.69	116.35	122.92
14	BA	1203	CLA	O2A-CGA-O1A	-4.69	111.57	123.56
14	bA	1223	CLA	O2A-CGA-O1A	-4.69	111.57	123.56
14	GA	1140	CLA	O2A-C1-C2	4.69	120.97	108.64
14	bA	1203	CLA	O2A-CGA-O1A	-4.69	111.57	123.56
14	HA	1203	CLA	O2A-CGA-O1A	-4.69	111.57	123.56
14	bA	1212	CLA	C1C-C2C-C3C	-4.69	101.98	106.95

Continued on next page...

PRELIMINARY VALIDATION REPORT

Continued from previous page...

Mol	Chain	Res	Type	Atoms	Z	Observed(°)	Ideal(°)
14	bA	1229	CLA	CMB-C2B-C3B	4.68	133.58	124.80
14	AA	1012	CLA	O2A-CGA-O1A	-4.68	111.60	123.56
14	aA	1116	CLA	C4A-NA-C1A	4.68	108.81	106.71
14	aA	1012	CLA	O2A-CGA-O1A	-4.68	111.61	123.56
14	GA	1102	CLA	CMB-C2B-C3B	4.68	133.57	124.80
18	HA	4005	BCR	C34-C9-C10	-4.67	116.38	122.92
14	GA	1126	CLA	O2A-C1-C2	4.67	120.92	108.64
14	GA	1012	CLA	O2A-CGA-O1A	-4.67	111.62	123.56
14	HA	1229	CLA	CMB-C2B-C3B	4.67	133.56	124.80
14	aA	1126	CLA	O2A-C1-C2	4.67	120.90	108.64
14	HA	1021	CLA	O2A-CGA-O1A	-4.67	111.63	123.56
14	HA	1226	CLA	C4A-NA-C1A	4.67	108.80	106.71
14	HA	1217	CLA	O2D-CGD-CBD	4.67	119.42	111.25
14	bA	1214	CLA	O2A-CGA-O1A	-4.66	111.64	123.56
14	bA	1217	CLA	O2D-CGD-CBD	4.66	119.41	111.25
14	BA	1214	CLA	O2A-CGA-O1A	-4.66	111.64	123.56
13	aA	1011	CL0	O2A-CGA-O1A	-4.66	111.65	123.56
14	GA	1117	CLA	CMB-C2B-C3B	4.66	133.54	124.80
14	BA	1211	CLA	O2A-C1-C2	4.66	120.88	108.64
14	HA	1214	CLA	O2A-CGA-O1A	-4.66	111.66	123.56
14	BA	1228	CLA	C1C-C2C-C3C	-4.66	102.02	106.95
14	BA	1021	CLA	O2A-CGA-O1A	-4.66	111.66	123.56
14	bA	1021	CLA	O2A-CGA-O1A	-4.66	111.66	123.56
14	BA	1229	CLA	CMB-C2B-C3B	4.65	133.53	124.80
14	BA	1217	CLA	O2D-CGD-CBD	4.65	119.39	111.25
14	AA	1101	CLA	C1C-C2C-C3C	-4.65	102.02	106.95
13	AA	1011	CL0	O2A-CGA-O1A	-4.65	111.67	123.56
14	HA	1224	CLA	C4A-NA-C1A	4.65	108.80	106.71
14	bA	1211	CLA	O2A-C1-C2	4.65	120.86	108.64
14	bA	1224	CLA	C4A-NA-C1A	4.65	108.80	106.71
14	AA	1126	CLA	O2A-C1-C2	4.64	120.84	108.64
14	bA	1220	CLA	O2A-CGA-O1A	-4.64	111.70	123.56
14	BA	1220	CLA	O2A-CGA-O1A	-4.64	111.70	123.56
13	GA	1011	CL0	O2A-CGA-O1A	-4.64	111.71	123.56
18	bA	4005	BCR	C34-C9-C10	-4.64	116.43	122.92
14	bA	1226	CLA	C4A-NA-C1A	4.63	108.79	106.71
14	HA	1220	CLA	O2A-CGA-O1A	-4.63	111.73	123.56
14	HA	1211	CLA	O2A-C1-C2	4.63	120.80	108.64
14	GA	1101	CLA	C1C-C2C-C3C	-4.62	102.05	106.95
14	bA	1228	CLA	C1C-C2C-C3C	-4.62	102.05	106.95
14	aA	1101	CLA	C1C-C2C-C3C	-4.62	102.06	106.95
14	aA	1117	CLA	CMB-C2B-C3B	4.62	133.46	124.80

Continued on next page...

PRELIMINARY VALIDATION REPORT

Continued from previous page...

Mol	Chain	Res	Type	Atoms	Z	Observed(^{o})	Ideal(^{o})
14	GA	1111	CLA	C1C-C2C-C3C	-4.62	102.06	106.95
14	BA	1021	CLA	O2A-C1-C2	4.62	120.76	108.64
14	aA	1124	CLA	C4A-NA-C1A	4.61	108.78	106.71
14	BA	1021	CLA	O2A-CGA-CBA	4.61	126.74	111.93
14	aA	1111	CLA	C1C-C2C-C3C	-4.61	102.07	106.95
14	bA	1021	CLA	O2A-CGA-CBA	4.61	126.72	111.93
14	HA	1021	CLA	O2A-CGA-CBA	4.60	126.72	111.93
14	AA	1117	CLA	CMB-C2B-C3B	4.60	133.44	124.80
14	BA	1236	CLA	C4A-NA-C1A	4.60	108.78	106.71
14	AA	1122	CLA	O2A-CGA-O1A	-4.60	111.81	123.56
14	bA	1021	CLA	O2A-C1-C2	4.60	120.72	108.64
14	aA	1122	CLA	O2A-CGA-O1A	-4.60	111.81	123.56
14	HA	1228	CLA	C1C-C2C-C3C	-4.60	102.08	106.95
14	BA	1208	CLA	C4A-NA-C1A	4.59	108.77	106.71
14	aA	1138	CLA	O2A-CGA-O1A	-4.59	111.82	123.56
14	BA	1225	CLA	C1C-C2C-C3C	-4.59	102.09	106.95
14	HA	1021	CLA	O2A-C1-C2	4.59	120.69	108.64
14	bA	1022	CLA	C1C-C2C-C3C	-4.59	102.09	106.95
14	HA	1210	CLA	C4A-NA-C1A	4.58	108.77	106.71
14	BA	1022	CLA	C4A-NA-C1A	4.58	108.77	106.71
14	GA	1138	CLA	O2A-CGA-O1A	-4.58	111.85	123.56
14	AA	1111	CLA	C1C-C2C-C3C	-4.58	102.09	106.95
14	HA	1023	CLA	O2A-CGA-O1A	-4.58	111.85	123.56
14	aA	1130	CLA	O2A-CGA-O1A	-4.58	111.86	123.56
14	BA	1023	CLA	O2A-CGA-O1A	-4.58	111.86	123.56
14	GA	1124	CLA	C4A-NA-C1A	4.58	108.76	106.71
14	AA	1130	CLA	O2A-CGA-O1A	-4.57	111.87	123.56
14	AA	1138	CLA	O2A-CGA-O1A	-4.57	111.87	123.56
14	LA	1502	CLA	O2A-CGA-O1A	-4.57	111.88	123.56
14	bA	1023	CLA	O2A-CGA-O1A	-4.57	111.88	123.56
14	GA	1122	CLA	O2A-CGA-O1A	-4.57	111.88	123.56
14	HA	1229	CLA	C4A-NA-C1A	4.57	108.76	106.71
14	bA	1236	CLA	C4A-NA-C1A	4.57	108.76	106.71
14	BA	1022	CLA	C1C-C2C-C3C	-4.57	102.11	106.95
14	BA	1225	CLA	O2D-CGD-CBD	4.57	119.24	111.25
14	GA	1130	CLA	O2A-CGA-O1A	-4.56	111.91	123.56
14	0	1012	CLA	C4A-NA-C1A	4.55	108.75	106.71
14	bA	1225	CLA	C1C-C2C-C3C	-4.55	102.12	106.95
14	UA	1502	CLA	O2A-CGA-O1A	-4.55	111.93	123.56
14	lA	1502	CLA	O2A-CGA-O1A	-4.55	111.93	123.56
14	0	1012	CLA	C1C-C2C-C3C	-4.55	102.13	106.95
14	HA	1238	CLA	O2D-CGD-CBD	4.55	119.21	111.25

Continued on next page...

PRELIMINARY VALIDATION REPORT

Continued from previous page...

Mol	Chain	Res	Type	Atoms	Z	Observed(°)	Ideal(°)
14	bA	1208	CLA	C4A-NA-C1A	4.55	108.75	106.71
14	BA	1205	CLA	C1C-C2C-C3C	-4.55	102.13	106.95
14	HA	1225	CLA	O2D-CGD-CBD	4.54	119.20	111.25
14	BA	1238	CLA	O2D-CGD-CBD	4.54	119.20	111.25
14	bA	1225	CLA	O2D-CGD-CBD	4.54	119.19	111.25
18	aA	4011	BCR	C3-C4-C5	-4.54	106.10	113.99
14	HA	1205	CLA	C1C-C2C-C3C	-4.54	102.14	106.95
18	AA	4011	BCR	C3-C4-C5	-4.54	106.10	113.99
14	bA	1238	CLA	O2D-CGD-CBD	4.53	119.18	111.25
14	HA	1221	CLA	O2A-CGA-O1A	-4.53	111.98	123.56
14	BA	1215	CLA	O2A-CGA-CBA	4.53	126.46	111.93
14	bA	1205	CLA	C1C-C2C-C3C	-4.52	102.16	106.95
14	HA	1225	CLA	C1C-C2C-C3C	-4.52	102.16	106.95
14	HA	1221	CLA	C1C-C2C-C3C	-4.52	102.16	106.95
14	HA	1208	CLA	C4A-NA-C1A	4.52	108.74	106.71
16	HA	2002	PQN	C14-C13-C15	4.51	123.06	115.29
18	GA	4011	BCR	C3-C4-C5	-4.51	106.14	113.99
14	BA	1221	CLA	C1C-C2C-C3C	-4.51	102.17	106.95
14	AA	1131	CLA	O2A-CGA-O1A	-4.51	112.03	123.56
14	HA	1215	CLA	O2A-CGA-CBA	4.51	126.41	111.93
14	bA	1232	CLA	O2D-CGD-CBD	4.51	119.14	111.25
14	bA	1221	CLA	C1C-C2C-C3C	-4.51	102.17	106.95
14	bA	1221	CLA	O2A-CGA-O1A	-4.51	112.05	123.56
14	BA	1221	CLA	O2A-CGA-O1A	-4.51	112.05	123.56
18	GA	4011	BCR	C24-C23-C22	-4.50	119.45	126.21
16	bA	2002	PQN	C14-C13-C15	4.50	123.04	115.29
14	GA	1133	CLA	O2A-CGA-O1A	-4.50	112.06	123.56
14	GA	1124	CLA	O2A-CGA-O1A	-4.50	112.07	123.56
14	GA	1131	CLA	O2A-CGA-O1A	-4.49	112.07	123.56
14	bA	1215	CLA	O2A-CGA-CBA	4.49	126.36	111.93
14	AA	1124	CLA	O2A-CGA-O1A	-4.49	112.08	123.56
14	HA	1222	CLA	CMB-C2B-C3B	4.49	133.22	124.80
16	BA	2002	PQN	C14-C13-C15	4.49	123.02	115.29
13	aA	1011	CL0	O2A-CGA-CBA	4.49	126.34	111.93
14	aA	1124	CLA	O2A-CGA-O1A	-4.49	112.09	123.56
14	BA	1222	CLA	CMB-C2B-C3B	4.49	133.22	124.80
14	HA	1232	CLA	O2D-CGD-CBD	4.49	119.11	111.25
18	LA	4019	BCR	C33-C5-C6	-4.49	119.51	124.51
14	bA	1222	CLA	CMB-C2B-C3B	4.49	133.21	124.80
18	GA	4002	BCR	C24-C23-C22	-4.48	119.47	126.21
14	BA	1210	CLA	C4A-NA-C1A	4.48	108.72	106.71
14	aA	1131	CLA	O2A-CGA-O1A	-4.48	112.10	123.56

Continued on next page...

PRELIMINARY VALIDATION REPORT

Continued from previous page...

Mol	Chain	Res	Type	Atoms	Z	Observed(^{o})	Ideal(^{o})
14	HA	1227	CLA	C4A-NA-C1A	4.48	108.72	106.71
14	aA	1133	CLA	O2A-CGA-O1A	-4.48	112.11	123.56
14	BA	1232	CLA	O2D-CGD-CBD	4.48	119.09	111.25
18	aA	4011	BCR	C24-C23-C22	-4.48	119.48	126.21
18	AA	4011	BCR	C24-C23-C22	-4.48	119.48	126.21
14	aA	1122	CLA	O2A-C1-C2	4.48	120.40	108.64
14	GA	1122	CLA	O2A-C1-C2	4.47	120.39	108.64
14	bA	1022	CLA	C4A-NA-C1A	4.47	108.72	106.71
14	bA	1022	CLA	O2A-CGA-O1A	-4.47	112.14	123.56
18	lA	4019	BCR	C33-C5-C6	-4.47	119.53	124.51
13	GA	1011	CL0	O2A-CGA-CBA	4.47	126.27	111.93
14	AA	1133	CLA	O2A-CGA-O1A	-4.47	112.14	123.56
14	GA	1013	CLA	CAA-C2A-C3A	-4.47	100.55	112.78
14	AA	1122	CLA	O2A-C1-C2	4.47	120.37	108.64
14	BA	1022	CLA	O2A-CGA-O1A	-4.46	112.16	123.56
14	AA	1013	CLA	CAA-C2A-C3A	-4.46	100.56	112.78
14	aA	1132	CLA	C4A-NA-C1A	4.46	108.71	106.71
14	0	1012	CLA	O2A-CGA-O1A	-4.46	112.17	123.56
13	AA	1011	CL0	O2A-CGA-CBA	4.46	126.24	111.93
14	UA	1503	CLA	O2A-CGA-O1A	-4.45	112.18	123.56
18	aA	4002	BCR	C24-C23-C22	-4.45	119.52	126.21
14	BA	1238	CLA	CMB-C2B-C3B	4.45	133.15	124.80
14	aA	1013	CLA	CAA-C2A-C3A	-4.45	100.59	112.78
14	AA	1105	CLA	C4A-NA-C1A	4.45	108.70	106.71
14	AA	1132	CLA	C4A-NA-C1A	4.44	108.70	106.71
18	UA	4019	BCR	C33-C5-C6	-4.44	119.56	124.51
14	GA	1114	CLA	CMB-C2B-C3B	4.44	133.13	124.80
14	bA	1238	CLA	CMB-C2B-C3B	4.44	133.13	124.80
14	lA	1503	CLA	O2A-CGA-O1A	-4.44	112.22	123.56
14	HA	1238	CLA	CMB-C2B-C3B	4.44	133.12	124.80
14	AA	1114	CLA	CMB-C2B-C3B	4.43	133.12	124.80
14	BA	1210	CLA	O2A-C1-C2	4.43	120.29	108.64
14	LA	1503	CLA	O2A-CGA-O1A	-4.43	112.23	123.56
14	HA	1226	CLA	O2A-CGA-CBA	4.43	126.15	111.93
14	aA	1117	CLA	O2A-CGA-O1A	-4.43	112.24	123.56
14	GA	1117	CLA	O2A-CGA-O1A	-4.43	112.24	123.56
14	bA	1225	CLA	CMB-C2B-C3B	4.43	133.10	124.80
14	bA	1210	CLA	O2A-C1-C2	4.43	120.27	108.64
14	aA	1114	CLA	CMB-C2B-C3B	4.43	133.10	124.80
14	AA	1117	CLA	O2A-CGA-O1A	-4.42	112.25	123.56
14	BA	1226	CLA	O2A-CGA-CBA	4.42	126.14	111.93
14	GA	1132	CLA	C4A-NA-C1A	4.42	108.69	106.71

Continued on next page...

PRELIMINARY VALIDATION REPORT

Continued from previous page...

Mol	Chain	Res	Type	Atoms	Z	Observed(^{o})	Ideal(^{o})
14	BA	1229	CLA	C4A-NA-C1A	4.42	108.69	106.71
14	HA	1225	CLA	CMB-C2B-C3B	4.42	133.09	124.80
18	AA	4002	BCR	C24-C23-C22	-4.42	119.57	126.21
14	bA	1226	CLA	O2A-CGA-CBA	4.42	126.11	111.93
14	BA	1225	CLA	CMB-C2B-C3B	4.41	133.08	124.80
14	HA	1210	CLA	O2A-C1-C2	4.41	120.23	108.64
14	HA	1211	CLA	O2D-CGD-CBD	4.41	118.97	111.25
14	bA	1229	CLA	C4A-NA-C1A	4.41	108.69	106.71
14	BA	1211	CLA	O2D-CGD-CBD	4.40	118.95	111.25
14	bA	1210	CLA	C4A-NA-C1A	4.40	108.68	106.71
14	BA	1224	CLA	C1C-C2C-C3C	-4.39	102.30	106.95
14	bA	1211	CLA	O2D-CGD-CBD	4.39	118.93	111.25
14	GA	1135	CLA	CMB-C2B-C3B	4.39	133.03	124.80
14	bA	1224	CLA	C1C-C2C-C3C	-4.39	102.30	106.95
14	GA	1119	CLA	C1C-C2C-C3C	-4.38	102.31	106.95
14	AA	1119	CLA	C1C-C2C-C3C	-4.38	102.31	106.95
18	AA	4001	BCR	C36-C18-C17	-4.38	116.79	122.92
14	AA	1135	CLA	CMB-C2B-C3B	4.38	133.01	124.80
18	GA	4001	BCR	C36-C18-C17	-4.37	116.80	122.92
20	iA	5006	LMG	O7-C10-C11	4.37	121.05	111.51
14	HA	1224	CLA	C1C-C2C-C3C	-4.37	102.32	106.95
14	AA	1114	CLA	C4A-NA-C1A	4.37	108.67	106.71
14	BA	1214	CLA	C4A-NA-C1A	4.37	108.67	106.71
18	aA	4001	BCR	C36-C18-C17	-4.37	116.81	122.92
14	BA	1215	CLA	C4A-NA-C1A	4.36	108.67	106.71
20	RA	5006	LMG	O7-C10-C11	4.36	121.03	111.51
14	bA	1211	CLA	CMB-C2B-C3B	4.36	132.98	124.80
14	aA	1135	CLA	CMB-C2B-C3B	4.36	132.97	124.80
14	aA	1119	CLA	C1C-C2C-C3C	-4.36	102.33	106.95
14	HA	1211	CLA	CMB-C2B-C3B	4.35	132.96	124.80
20	GA	5005	LMG	O7-C10-C11	4.35	121.01	111.51
20	IA	5006	LMG	O7-C10-C11	4.35	121.01	111.51
18	GA	4007	BCR	C31-C1-C6	-4.35	103.26	110.30
18	BA	4017	BCR	C3-C4-C5	-4.35	106.42	113.99
20	AA	5005	LMG	O7-C10-C11	4.35	121.01	111.51
18	iA	4018	BCR	C7-C8-C9	-4.34	119.68	126.21
14	GA	1105	CLA	C4A-NA-C1A	4.34	108.66	106.71
14	BA	1211	CLA	CMB-C2B-C3B	4.34	132.93	124.80
18	aA	4007	BCR	C31-C1-C6	-4.34	103.29	110.30
18	RA	4018	BCR	C7-C8-C9	-4.34	119.70	126.21
18	AA	4007	BCR	C31-C1-C6	-4.34	103.29	110.30
18	HA	4017	BCR	C3-C4-C5	-4.34	106.45	113.99

Continued on next page...

PRELIMINARY VALIDATION REPORT

Continued from previous page...

Mol	Chain	Res	Type	Atoms	Z	Observed(°)	Ideal(°)
20	aA	5005	LMG	O7-C10-C11	4.34	120.97	111.51
18	bA	4013	BCR	C3-C4-C5	-4.32	106.47	113.99
18	bA	4017	BCR	C3-C4-C5	-4.32	106.47	113.99
14	HA	1221	CLA	O2A-CGA-CBA	4.32	125.80	111.93
14	BA	1222	CLA	O2A-CGA-CBA	4.32	125.79	111.93
14	aA	1130	CLA	O2A-C1-C2	4.31	119.97	108.64
18	GA	4011	BCR	C34-C9-C10	-4.31	116.88	122.92
14	aA	1105	CLA	C4A-NA-C1A	4.31	108.64	106.71
14	bA	1222	CLA	O2A-CGA-CBA	4.31	125.77	111.93
14	HA	1222	CLA	O2A-CGA-CBA	4.31	125.77	111.93
14	GA	1130	CLA	O2A-C1-C2	4.31	119.96	108.64
14	BA	1222	CLA	O2A-C1-C2	4.31	119.96	108.64
14	BA	1231	CLA	O2A-C1-C2	4.31	119.95	108.64
14	bA	1215	CLA	C4A-NA-C1A	4.31	108.64	106.71
18	IA	4018	BCR	C7-C8-C9	-4.31	119.74	126.21
18	HA	4013	BCR	C3-C4-C5	-4.30	106.50	113.99
14	AA	1130	CLA	O2A-C1-C2	4.30	119.95	108.64
14	BA	1221	CLA	O2A-CGA-CBA	4.30	125.75	111.93
14	HA	1215	CLA	C4A-NA-C1A	4.30	108.64	106.71
14	bA	1221	CLA	O2A-CGA-CBA	4.30	125.75	111.93
14	bA	1222	CLA	O2A-C1-C2	4.30	119.94	108.64
14	HA	1238	CLA	C1C-C2C-C3C	-4.30	102.39	106.95
18	AA	4011	BCR	C34-C9-C10	-4.30	116.91	122.92
14	HA	1222	CLA	O2A-C1-C2	4.29	119.92	108.64
14	bA	1231	CLA	O2A-C1-C2	4.29	119.92	108.64
14	bA	1238	CLA	C1C-C2C-C3C	-4.29	102.40	106.95
14	HA	1231	CLA	O2A-C1-C2	4.29	119.92	108.64
18	BA	4013	BCR	C3-C4-C5	-4.29	106.53	113.99
14	BA	1238	CLA	C1C-C2C-C3C	-4.29	102.41	106.95
14	bA	1227	CLA	C4A-NA-C1A	4.29	108.63	106.71
14	bA	1210	CLA	C1C-C2C-C3C	-4.28	102.41	106.95
14	GA	1128	CLA	CMB-C2B-C1B	-4.27	121.89	128.46
18	HA	4009	BCR	C15-C14-C13	-4.27	121.21	127.31
14	HA	1214	CLA	C4A-NA-C1A	4.27	108.63	106.71
18	aA	4008	BCR	C15-C14-C13	-4.27	121.22	127.31
18	AA	4003	BCR	C7-C8-C9	-4.26	119.80	126.21
18	GA	4008	BCR	C15-C14-C13	-4.26	121.23	127.31
14	bA	1214	CLA	C4A-NA-C1A	4.26	108.62	106.71
14	aA	1114	CLA	C4A-NA-C1A	4.26	108.62	106.71
14	aA	1128	CLA	CMB-C2B-C1B	-4.26	121.92	128.46
14	HA	1202	CLA	O2A-C1-C2	4.26	119.82	108.64
14	HA	1204	CLA	O2A-CGA-CBA	4.26	125.60	111.93

Continued on next page...

PRELIMINARY VALIDATION REPORT

Continued from previous page...

Mol	Chain	Res	Type	Atoms	Z	Observed(^{o})	Ideal(^{o})
14	AA	1128	CLA	CMB-C2B-C1B	-4.26	121.92	128.46
14	bA	1204	CLA	O2A-CGA-CBA	4.26	125.59	111.93
14	BA	1210	CLA	C1C-C2C-C3C	-4.25	102.44	106.95
18	BA	4013	BCR	C15-C14-C13	-4.25	121.24	127.31
18	aA	4011	BCR	C34-C9-C10	-4.25	116.96	122.92
14	bA	1202	CLA	O2A-C1-C2	4.25	119.81	108.64
14	GA	1114	CLA	C4A-NA-C1A	4.25	108.62	106.71
18	bA	4013	BCR	C15-C14-C13	-4.25	121.25	127.31
14	BA	1202	CLA	O2A-C1-C2	4.25	119.80	108.64
18	bA	4009	BCR	C15-C14-C13	-4.24	121.25	127.31
14	AA	1111	CLA	C4A-NA-C1A	4.24	108.61	106.71
14	AA	1128	CLA	O2A-CGA-CBA	4.24	125.55	111.93
14	HA	1210	CLA	C1C-C2C-C3C	-4.24	102.46	106.95
14	GA	1119	CLA	CMB-C2B-C3B	4.24	132.75	124.80
14	BA	1204	CLA	O2A-CGA-CBA	4.24	125.54	111.93
18	iA	4018	BCR	C37-C22-C21	-4.24	116.99	122.92
18	RA	4018	BCR	C37-C22-C21	-4.24	116.99	122.92
14	aA	1128	CLA	O2A-CGA-CBA	4.24	125.53	111.93
14	AA	1119	CLA	CMB-C2B-C3B	4.23	132.74	124.80
14	BA	1227	CLA	C4A-NA-C1A	4.23	108.61	106.71
18	HA	4017	BCR	C24-C23-C22	-4.23	119.86	126.21
18	bA	4017	BCR	C24-C23-C22	-4.23	119.86	126.21
18	aA	4003	BCR	C7-C8-C9	-4.23	119.86	126.21
18	HA	4013	BCR	C15-C14-C13	-4.23	121.28	127.31
18	GA	4003	BCR	C7-C8-C9	-4.23	119.86	126.21
18	AA	4008	BCR	C15-C14-C13	-4.22	121.28	127.31
18	BA	4009	BCR	C15-C14-C13	-4.22	121.28	127.31
14	GA	1128	CLA	O2A-CGA-CBA	4.22	125.49	111.93
18	HA	4006	BCR	C24-C23-C22	-4.22	119.87	126.21
18	BA	4017	BCR	C24-C23-C22	-4.22	119.87	126.21
18	GA	4001	BCR	C19-C18-C17	4.22	125.42	118.94
14	aA	1119	CLA	CMB-C2B-C3B	4.22	132.71	124.80
14	BA	1203	CLA	CAA-C2A-C3A	-4.22	101.23	112.78
14	GA	1122	CLA	C1C-C2C-C3C	-4.21	102.48	106.95
14	bA	1234	CLA	O2A-CGA-O1A	-4.21	112.79	123.56
14	GA	1103	CLA	CAA-C2A-C3A	-4.21	101.24	112.78
18	BA	4006	BCR	C24-C23-C22	-4.21	119.88	126.21
14	aA	1122	CLA	C1C-C2C-C3C	-4.21	102.49	106.95
14	BA	1229	CLA	O2A-CGA-CBA	4.21	125.45	111.93
14	AA	1103	CLA	CAA-C2A-C3A	-4.21	101.25	112.78
18	IA	4018	BCR	C37-C22-C21	-4.21	117.03	122.92
14	aA	1103	CLA	CAA-C2A-C3A	-4.21	101.25	112.78

Continued on next page...

PRELIMINARY VALIDATION REPORT

Continued from previous page...

Mol	Chain	Res	Type	Atoms	Z	Observed(°)	Ideal(°)
18	bA	4006	BCR	C24-C23-C22	-4.20	119.90	126.21
14	aA	1127	CLA	CMB-C2B-C3B	4.20	132.68	124.80
14	GA	1127	CLA	CMB-C2B-C3B	4.20	132.68	124.80
18	aA	4011	BCR	C33-C5-C6	-4.20	119.83	124.51
14	HA	1239	CLA	O2A-CGA-CBA	4.20	125.41	111.93
18	GA	4011	BCR	C33-C5-C6	-4.20	119.84	124.51
14	HA	1234	CLA	O2A-CGA-O1A	-4.19	112.84	123.56
14	bA	1229	CLA	O2A-CGA-CBA	4.19	125.39	111.93
14	HA	1229	CLA	O2A-CGA-CBA	4.19	125.39	111.93
18	AA	4001	BCR	C19-C18-C17	4.19	125.37	118.94
14	BA	1234	CLA	O2A-CGA-O1A	-4.19	112.86	123.56
14	bA	1203	CLA	CAA-C2A-C3A	-4.18	101.32	112.78
18	aA	4001	BCR	C19-C18-C17	4.18	125.36	118.94
14	BA	1239	CLA	O2A-CGA-CBA	4.18	125.36	111.93
14	bA	1239	CLA	O2A-CGA-CBA	4.18	125.35	111.93
14	HA	1203	CLA	CAA-C2A-C3A	-4.18	101.33	112.78
18	AA	4011	BCR	C33-C5-C6	-4.18	119.86	124.51
14	AA	1122	CLA	C1C-C2C-C3C	-4.18	102.52	106.95
14	bA	1235	CLA	O2A-CGA-CBA	4.18	125.34	111.93
14	GA	1111	CLA	C4A-NA-C1A	4.18	108.58	106.71
14	bA	1235	CLA	O2A-C1-C2	4.17	119.61	108.64
14	HA	1235	CLA	O2A-C1-C2	4.17	119.60	108.64
18	AA	4002	BCR	C19-C18-C17	4.17	125.34	118.94
14	BA	1235	CLA	O2A-CGA-CBA	4.17	125.31	111.93
19	AA	5001	LHG	O7-C7-C8	4.17	120.61	111.51
14	BA	1235	CLA	O2A-C1-C2	4.17	119.58	108.64
14	BA	1238	CLA	O2A-CGA-CBA	4.17	125.30	111.93
14	GA	1111	CLA	O2A-CGA-CBA	4.16	125.30	111.93
14	AA	1127	CLA	CMB-C2B-C3B	4.16	132.61	124.80
14	AA	1111	CLA	O2A-CGA-CBA	4.16	125.29	111.93
14	HA	1235	CLA	O2A-CGA-CBA	4.16	125.28	111.93
18	aA	4002	BCR	C19-C18-C17	4.16	125.32	118.94
18	MA	4021	BCR	C36-C18-C17	-4.16	117.10	122.92
14	aA	1111	CLA	O2A-CGA-CBA	4.15	125.27	111.93
18	IA	4020	BCR	C7-C8-C9	-4.15	119.97	126.21
18	iA	4020	BCR	C7-C8-C9	-4.15	119.97	126.21
18	RA	4020	BCR	C7-C8-C9	-4.15	119.97	126.21
14	AA	1012	CLA	O2A-CGA-CBA	4.15	125.26	111.93
14	bA	1238	CLA	O2A-CGA-CBA	4.15	125.25	111.93
14	HA	1238	CLA	O2A-CGA-CBA	4.15	125.25	111.93
14	aA	1130	CLA	CMB-C2B-C3B	4.15	132.58	124.80
19	GA	5001	LHG	O7-C7-C8	4.15	120.56	111.51

Continued on next page...

PRELIMINARY VALIDATION REPORT

Continued from previous page...

Mol	Chain	Res	Type	Atoms	Z	Observed(°)	Ideal(°)
14	bA	1208	CLA	O2A-CGA-CBA	4.14	125.24	111.93
14	aA	1012	CLA	O2A-CGA-CBA	4.14	125.23	111.93
14	BA	1208	CLA	O2A-CGA-CBA	4.13	125.20	111.93
18	mA	4021	BCR	C36-C18-C17	-4.13	117.13	122.92
14	GA	1012	CLA	O2A-CGA-CBA	4.13	125.20	111.93
18	GA	4001	BCR	C24-C23-C22	-4.13	120.01	126.21
14	AA	1130	CLA	CMB-C2B-C3B	4.13	132.54	124.80
18	GA	4003	BCR	C15-C14-C13	-4.13	121.42	127.31
18	GA	4002	BCR	C19-C18-C17	4.13	125.27	118.94
18	RA	4020	BCR	C19-C18-C17	4.13	125.27	118.94
19	aA	5001	LHG	O7-C7-C8	4.12	120.51	111.51
18	AA	4003	BCR	C15-C14-C13	-4.12	121.43	127.31
14	HA	1203	CLA	O2D-CGD-CBD	4.12	118.46	111.25
18	VA	4021	BCR	C36-C18-C17	-4.12	117.16	122.92
14	HA	1208	CLA	O2A-CGA-CBA	4.12	125.14	111.93
14	bA	1203	CLA	O2D-CGD-CBD	4.11	118.45	111.25
19	bA	5004	LHG	O7-C7-C8	4.11	120.48	111.51
18	IA	4020	BCR	C19-C18-C17	4.11	125.25	118.94
14	GA	1125	CLA	CMB-C2B-C3B	4.11	132.51	124.80
18	aA	4003	BCR	C15-C14-C13	-4.11	121.45	127.31
19	BA	5004	LHG	O7-C7-C8	4.11	120.47	111.51
14	GA	1130	CLA	CMB-C2B-C3B	4.11	132.50	124.80
18	iA	4018	BCR	C33-C5-C6	-4.11	119.94	124.51
14	BA	1203	CLA	O2D-CGD-CBD	4.10	118.43	111.25
14	HA	1203	CLA	O2A-CGA-CBA	4.10	125.10	111.93
14	bA	1203	CLA	O2A-CGA-CBA	4.10	125.10	111.93
19	HA	5004	LHG	O7-C7-C8	4.10	120.46	111.51
18	iA	4020	BCR	C19-C18-C17	4.10	125.23	118.94
14	BA	1203	CLA	O2A-CGA-CBA	4.10	125.09	111.93
18	AA	4001	BCR	C24-C23-C22	-4.09	120.06	126.21
18	aA	4001	BCR	C24-C23-C22	-4.09	120.06	126.21
14	HA	1222	CLA	CAA-C2A-C3A	-4.09	101.58	112.78
18	HA	4012	BCR	C24-C23-C22	-4.09	120.07	126.21
14	aA	1125	CLA	CMB-C2B-C3B	4.09	132.46	124.80
14	bA	1222	CLA	CAA-C2A-C3A	-4.08	101.59	112.78
18	bA	4012	BCR	C24-C23-C22	-4.08	120.08	126.21
18	UA	4022	BCR	C33-C5-C6	-4.08	119.96	124.51
18	LA	4022	BCR	C33-C5-C6	-4.08	119.97	124.51
14	AA	1125	CLA	CMB-C2B-C3B	4.08	132.45	124.80
18	aA	4008	BCR	C34-C9-C10	-4.07	117.22	122.92
18	AA	4008	BCR	C34-C9-C10	-4.07	117.22	122.92
14	BA	1222	CLA	CAA-C2A-C3A	-4.07	101.63	112.78

Continued on next page...

PRELIMINARY VALIDATION REPORT

Continued from previous page...

Mol	Chain	Res	Type	Atoms	Z	Observed(^{o})	Ideal(^{o})
18	GA	4008	BCR	C24-C23-C22	-4.07	120.10	126.21
18	BA	4012	BCR	C24-C23-C22	-4.05	120.12	126.21
14	GA	1111	CLA	CAC-C3C-C4C	4.05	130.12	124.82
18	RA	4018	BCR	C33-C5-C6	-4.05	120.00	124.51
18	GA	4008	BCR	C34-C9-C10	-4.05	117.25	122.92
18	IA	4018	BCR	C33-C5-C6	-4.04	120.01	124.51
14	GA	1125	CLA	O2A-CGA-CBA	4.04	124.92	111.93
18	aA	4008	BCR	C24-C23-C22	-4.04	120.14	126.21
14	AA	1111	CLA	CAC-C3C-C4C	4.04	130.10	124.82
14	KA	1401	CLA	O2D-CGD-CBD	4.04	118.32	111.25
18	lA	4022	BCR	C33-C5-C6	-4.04	120.01	124.51
14	HA	1201	CLA	O2A-C1-C2	4.04	119.25	108.64
14	BA	1213	CLA	O2A-CGA-CBA	4.04	124.89	111.93
18	AA	4008	BCR	C24-C23-C22	-4.03	120.15	126.21
14	aA	1125	CLA	O2A-CGA-CBA	4.03	124.87	111.93
14	bA	1220	CLA	O2A-CGA-CBA	4.03	124.87	111.93
14	BA	1220	CLA	O2A-CGA-CBA	4.03	124.87	111.93
14	AA	1125	CLA	O2A-CGA-CBA	4.03	124.86	111.93
14	bA	1201	CLA	O2A-C1-C2	4.03	119.22	108.64
14	bA	1213	CLA	O2A-CGA-CBA	4.03	124.86	111.93
14	AA	1140	CLA	O2A-CGA-CBA	4.03	124.86	111.93
14	HA	1213	CLA	O2A-CGA-CBA	4.03	124.86	111.93
14	TA	1401	CLA	O2D-CGD-CBD	4.02	118.30	111.25
14	BA	1023	CLA	O2A-CGA-CBA	4.02	124.84	111.93
14	BA	1201	CLA	O2A-C1-C2	4.02	119.20	108.64
14	bA	1023	CLA	O2A-CGA-CBA	4.02	124.84	111.93
14	aA	1127	CLA	O2A-CGA-CBA	4.02	124.83	111.93
18	aA	4007	BCR	C33-C5-C6	-4.02	120.04	124.51
14	aA	1140	CLA	O2A-CGA-CBA	4.01	124.82	111.93
14	HA	1023	CLA	O2A-CGA-CBA	4.01	124.82	111.93
18	GA	4007	BCR	C33-C5-C6	-4.01	120.05	124.51
14	HA	1220	CLA	O2A-CGA-CBA	4.01	124.80	111.93
14	aA	1111	CLA	C4A-NA-C1A	4.01	108.51	106.71
14	BA	1223	CLA	CMB-C2B-C3B	4.01	132.32	124.80
14	GA	1127	CLA	O2A-CGA-CBA	4.01	124.80	111.93
14	AA	1127	CLA	O2A-CGA-CBA	4.01	124.80	111.93
18	AA	4007	BCR	C33-C5-C6	-4.00	120.05	124.51
14	kA	1401	CLA	O2D-CGD-CBD	4.00	118.25	111.25
14	GA	1140	CLA	O2A-CGA-CBA	4.00	124.77	111.93
14	aA	1111	CLA	CAC-C3C-C4C	4.00	130.04	124.82
14	GA	1126	CLA	O2A-CGA-CBA	3.98	124.72	111.93
18	GA	4007	BCR	C3-C4-C5	-3.98	107.06	113.99

Continued on next page...

PRELIMINARY VALIDATION REPORT

Continued from previous page...

Mol	Chain	Res	Type	Atoms	Z	Observed(°)	Ideal(°)
14	BA	1022	CLA	OBD-CAD-C3D	-3.98	120.99	128.04
18	AA	4007	BCR	C3-C4-C5	-3.98	107.07	113.99
14	bA	1220	CLA	CAA-C2A-C3A	-3.98	101.89	112.78
18	aA	4007	BCR	C3-C4-C5	-3.98	107.07	113.99
14	bA	1223	CLA	CMB-C2B-C3B	3.97	132.25	124.80
14	HA	1223	CLA	CMB-C2B-C3B	3.97	132.24	124.80
14	GA	1129	CLA	O2D-CGD-O1D	-3.97	115.99	123.83
14	BA	1220	CLA	CAA-C2A-C3A	-3.97	101.92	112.78
14	AA	1126	CLA	O2A-CGA-CBA	3.96	124.65	111.93
14	bA	1022	CLA	OBD-CAD-C3D	-3.96	121.02	128.04
14	AA	1129	CLA	O2D-CGD-O1D	-3.96	116.01	123.83
14	LA	1501	CLA	CMB-C2B-C3B	3.96	132.22	124.80
14	BA	1217	CLA	O2A-CGA-CBA	3.96	124.64	111.93
14	aA	1126	CLA	O2A-CGA-CBA	3.96	124.64	111.93
14	0	1012	CLA	OBD-CAD-C3D	-3.96	121.03	128.04
14	aA	1129	CLA	O2D-CGD-O1D	-3.96	116.02	123.83
14	bA	1204	CLA	C4A-NA-C1A	3.95	108.48	106.71
18	HA	4016	BCR	C15-C14-C13	-3.95	121.67	127.31
14	HA	1220	CLA	CAA-C2A-C3A	-3.95	101.97	112.78
14	bA	1217	CLA	O2A-CGA-CBA	3.94	124.59	111.93
14	UA	1501	CLA	CMB-C2B-C3B	3.94	132.20	124.80
14	HA	1229	CLA	C4-C3-C5	3.94	122.08	115.29
14	HA	1217	CLA	O2A-CGA-CBA	3.94	124.58	111.93
14	GA	1115	CLA	O2A-CGA-CBA	3.93	124.56	111.93
14	AA	1115	CLA	O2A-CGA-CBA	3.93	124.56	111.93
18	BA	4009	BCR	C34-C9-C10	-3.93	117.41	122.92
14	aA	1115	CLA	O2A-CGA-CBA	3.93	124.56	111.93
18	bA	4016	BCR	C15-C14-C13	-3.93	121.70	127.31
18	HA	4009	BCR	C34-C9-C10	-3.93	117.42	122.92
14	lA	1501	CLA	CMB-C2B-C3B	3.93	132.17	124.80
18	bA	4009	BCR	C34-C9-C10	-3.93	117.42	122.92
14	bA	1231	CLA	O2A-CGA-CBA	3.93	124.53	111.93
18	MA	4021	BCR	C19-C18-C17	3.92	124.96	118.94
14	aA	1135	CLA	O2A-CGA-CBA	3.92	124.52	111.93
14	BA	1231	CLA	O2A-CGA-CBA	3.92	124.51	111.93
14	BA	1204	CLA	C4A-NA-C1A	3.91	108.47	106.71
14	HA	1231	CLA	O2A-CGA-CBA	3.91	124.48	111.93
14	AA	1124	CLA	CMB-C2B-C3B	3.91	132.12	124.80
14	GA	1124	CLA	CMB-C2B-C3B	3.91	132.12	124.80
14	GA	1132	CLA	O2A-CGA-CBA	3.90	124.47	111.93
18	BA	4016	BCR	C15-C14-C13	-3.90	121.74	127.31
14	BA	1229	CLA	C4-C3-C5	3.90	122.01	115.29

Continued on next page...

PRELIMINARY VALIDATION REPORT

Continued from previous page...

Mol	Chain	Res	Type	Atoms	Z	Observed(°)	Ideal(°)
14	AA	1135	CLA	O2A-CGA-CBA	3.90	124.46	111.93
14	GA	1135	CLA	O2A-CGA-CBA	3.90	124.46	111.93
14	aA	1132	CLA	O2A-CGA-CBA	3.90	124.45	111.93
14	bA	1229	CLA	C4-C3-C5	3.90	122.00	115.29
18	GA	4011	BCR	C33-C5-C4	3.90	120.86	113.57
14	aA	1124	CLA	CMB-C2B-C3B	3.89	132.10	124.80
18	mA	4021	BCR	C19-C18-C17	3.89	124.92	118.94
14	AA	1132	CLA	O2A-CGA-CBA	3.89	124.42	111.93
14	aA	1116	CLA	O2A-CGA-CBA	3.89	124.41	111.93
18	aA	4011	BCR	C33-C5-C4	3.88	120.84	113.57
14	AA	1138	CLA	CMB-C2B-C3B	3.88	132.08	124.80
14	GA	1138	CLA	CMB-C2B-C3B	3.88	132.08	124.80
18	VA	4021	BCR	C19-C18-C17	3.88	124.89	118.94
14	GA	1133	CLA	O2A-CGA-CBA	3.88	124.38	111.93
14	AA	1131	CLA	O2A-CGA-CBA	3.88	124.38	111.93
14	HA	1021	CLA	CAA-C2A-C3A	-3.88	102.17	112.78
14	AA	1116	CLA	O2A-CGA-CBA	3.87	124.37	111.93
20	lA	5007	LMG	O7-C10-C11	3.87	119.95	111.51
14	aA	1138	CLA	CMB-C2B-C3B	3.87	132.05	124.80
14	GA	1116	CLA	O2A-CGA-CBA	3.87	124.35	111.93
14	aA	1133	CLA	O2A-CGA-CBA	3.87	124.34	111.93
18	HA	4004	BCR	C19-C18-C17	3.86	124.87	118.94
14	HA	1226	CLA	CMB-C2B-C1B	-3.86	122.53	128.46
14	bA	1021	CLA	CAA-C2A-C3A	-3.86	102.20	112.78
20	LA	5007	LMG	O7-C10-C11	3.86	119.94	111.51
14	HA	1238	CLA	C4A-NA-C1A	3.86	108.44	106.71
14	aA	1122	CLA	O2D-CGD-CBD	3.86	118.01	111.25
14	GA	1131	CLA	O2A-CGA-CBA	3.86	124.33	111.93
14	BA	1021	CLA	CAA-C2A-C3A	-3.86	102.21	112.78
20	UA	5007	LMG	O7-C10-C11	3.86	119.94	111.51
18	AA	4011	BCR	C33-C5-C4	3.86	120.79	113.57
14	AA	1133	CLA	O2A-CGA-CBA	3.86	124.32	111.93
14	aA	1103	CLA	O2A-CGA-CBA	3.86	124.31	111.93
14	GA	1104	CLA	O2A-CGA-CBA	3.86	124.31	111.93
18	bA	4005	BCR	C7-C8-C9	-3.85	120.42	126.21
14	aA	1131	CLA	O2A-CGA-CBA	3.85	124.31	111.93
18	bA	4009	BCR	C38-C26-C25	-3.85	120.22	124.51
18	BA	4009	BCR	C38-C26-C25	-3.85	120.22	124.51
14	AA	1103	CLA	O2A-CGA-CBA	3.85	124.28	111.93
18	BA	4004	BCR	C19-C18-C17	3.85	124.84	118.94
14	BA	1226	CLA	CMB-C2B-C1B	-3.85	122.55	128.46
14	bA	1226	CLA	CMB-C2B-C1B	-3.84	122.56	128.46

Continued on next page...

PRELIMINARY VALIDATION REPORT

Continued from previous page...

Mol	Chain	Res	Type	Atoms	Z	Observed(^{o})	Ideal(^{o})
14	AA	1104	CLA	O2A-CGA-CBA	3.84	124.27	111.93
18	HA	4005	BCR	C7-C8-C9	-3.84	120.44	126.21
18	lA	4022	BCR	C28-C27-C26	-3.84	107.31	113.99
14	BA	1023	CLA	C4C-C3C-C2C	-3.84	101.33	106.89
18	bA	4004	BCR	C19-C18-C17	3.84	124.83	118.94
14	aA	1104	CLA	O2A-CGA-CBA	3.84	124.25	111.93
18	HA	4009	BCR	C38-C26-C25	-3.83	120.24	124.51
14	HA	1204	CLA	C4A-NA-C1A	3.83	108.43	106.71
18	BA	4005	BCR	C7-C8-C9	-3.83	120.45	126.21
18	GA	4007	BCR	C24-C23-C22	-3.83	120.46	126.21
18	UA	4022	BCR	C28-C27-C26	-3.83	107.33	113.99
14	BA	1205	CLA	CMB-C2B-C3B	3.83	131.98	124.80
14	GA	1103	CLA	O2A-CGA-CBA	3.82	124.21	111.93
14	GA	1122	CLA	O2D-CGD-CBD	3.82	117.95	111.25
14	GA	1117	CLA	O2A-CGA-CBA	3.82	124.21	111.93
18	bA	4016	BCR	C24-C23-C22	-3.82	120.47	126.21
18	aA	4002	BCR	C36-C18-C17	-3.82	117.57	122.92
18	AA	4007	BCR	C24-C23-C22	-3.82	120.47	126.21
14	bA	1023	CLA	C4C-C3C-C2C	-3.82	101.36	106.89
14	GA	1013	CLA	C2C-C1C-NC	3.82	113.55	109.97
14	bA	1205	CLA	CMB-C2B-C3B	3.82	131.96	124.80
18	VA	4021	BCR	C34-C9-C10	-3.81	117.58	122.92
14	HA	1023	CLA	C4C-C3C-C2C	-3.81	101.37	106.89
14	AA	1122	CLA	O2D-CGD-CBD	3.81	117.92	111.25
18	AA	4002	BCR	C36-C18-C17	-3.81	117.58	122.92
14	bA	1215	CLA	O2A-C1-C2	3.81	118.65	108.64
18	BA	4016	BCR	C24-C23-C22	-3.81	120.49	126.21
18	GA	4008	BCR	C7-C8-C9	-3.81	120.49	126.21
18	BA	4006	BCR	C4-C5-C6	-3.81	117.19	122.74
14	bA	1201	CLA	CMB-C2B-C3B	3.81	131.94	124.80
18	GA	4002	BCR	C36-C18-C17	-3.80	117.59	122.92
14	HA	1205	CLA	CMB-C2B-C3B	3.80	131.94	124.80
14	aA	1117	CLA	O2A-CGA-CBA	3.80	124.14	111.93
18	mA	4021	BCR	C34-C9-C10	-3.80	117.60	122.92
14	AA	1117	CLA	O2A-CGA-CBA	3.80	124.13	111.93
14	BA	1215	CLA	O2A-C1-C2	3.80	118.62	108.64
14	bA	1210	CLA	O2A-CGA-O1A	-3.80	113.85	123.56
18	LA	4022	BCR	C28-C27-C26	-3.80	107.38	113.99
14	HA	1215	CLA	O2A-C1-C2	3.80	118.61	108.64
18	BA	4009	BCR	C33-C5-C6	-3.80	120.28	124.51
18	AA	4008	BCR	C7-C8-C9	-3.80	120.51	126.21
14	bA	1225	CLA	O2A-CGA-CBA	3.79	124.11	111.93

Continued on next page...

PRELIMINARY VALIDATION REPORT

Continued from previous page...

Mol	Chain	Res	Type	Atoms	Z	Observed(^{o})	Ideal(^{o})
14	HA	1201	CLA	CMB-C2B-C3B	3.79	131.91	124.80
18	HA	4006	BCR	C4-C5-C6	-3.79	117.20	122.74
14	HA	1210	CLA	O2A-CGA-O1A	-3.79	113.87	123.56
14	HA	1225	CLA	O2A-CGA-CBA	3.79	124.08	111.93
14	LA	1501	CLA	CBA-CAA-C2A	3.78	125.11	113.85
14	BA	1201	CLA	CMB-C2B-C3B	3.78	131.89	124.80
14	BA	1210	CLA	O2A-CGA-O1A	-3.78	113.89	123.56
18	MA	4021	BCR	C34-C9-C10	-3.78	117.63	122.92
18	aA	4007	BCR	C24-C23-C22	-3.78	120.53	126.21
18	HA	4006	BCR	C7-C8-C9	3.78	131.89	126.21
14	UA	1501	CLA	CBA-CAA-C2A	3.78	125.09	113.85
14	AA	1118	CLA	C4-C3-C5	3.78	121.80	115.29
14	BA	1224	CLA	CMB-C2B-C3B	3.78	131.88	124.80
14	bA	1226	CLA	O2D-CGD-O1D	-3.78	116.37	123.83
14	aA	1118	CLA	C4-C3-C5	3.78	121.79	115.29
18	BA	4012	BCR	C33-C5-C6	-3.78	120.30	124.51
18	iA	4018	BCR	C34-C9-C10	-3.78	117.63	122.92
14	BA	1225	CLA	O2A-CGA-CBA	3.78	124.05	111.93
18	aA	4008	BCR	C7-C8-C9	-3.77	120.54	126.21
14	BA	1226	CLA	O2D-CGD-O1D	-3.77	116.38	123.83
14	GA	1132	CLA	O2D-CGD-O1D	-3.77	116.38	123.83
14	GA	1118	CLA	C4-C3-C5	3.77	121.79	115.29
18	lA	4019	BCR	C38-C26-C25	-3.77	120.31	124.51
18	bA	4006	BCR	C4-C5-C6	-3.77	117.24	122.74
14	aA	1132	CLA	O2D-CGD-O1D	-3.77	116.39	123.83
20	bA	5002	LMG	O7-C10-C11	3.77	119.74	111.51
20	HA	5002	LMG	O7-C10-C11	3.77	119.74	111.51
18	HA	4016	BCR	C24-C23-C22	-3.77	120.55	126.21
18	HA	4012	BCR	C33-C5-C6	-3.77	120.31	124.51
14	HA	1228	CLA	CMB-C2B-C3B	3.77	131.87	124.80
14	aA	1013	CLA	C2C-C1C-NC	3.77	113.50	109.97
18	IA	4018	BCR	C34-C9-C10	-3.77	117.65	122.92
20	BA	5002	LMG	O7-C10-C11	3.77	119.73	111.51
14	HA	1226	CLA	O2D-CGD-O1D	-3.76	116.39	123.83
14	AA	1110	CLA	O2D-CGD-O1D	-3.76	116.40	123.83
14	BA	1216	CLA	O2A-CGA-CBA	3.76	124.02	111.93
14	AA	1132	CLA	O2D-CGD-O1D	-3.76	116.40	123.83
14	BA	1022	CLA	O2A-CGA-CBA	3.76	124.01	111.93
18	bA	4006	BCR	C7-C8-C9	3.76	131.86	126.21
14	lA	1501	CLA	CBA-CAA-C2A	3.76	125.04	113.85
14	bA	1216	CLA	O2A-CGA-CBA	3.76	123.99	111.93
18	BA	4013	BCR	C7-C8-C9	-3.76	120.57	126.21

Continued on next page...

PRELIMINARY VALIDATION REPORT

Continued from previous page...

Mol	Chain	Res	Type	Atoms	Z	Observed(°)	Ideal(°)
14	bA	1238	CLA	C4A-NA-C1A	3.76	108.39	106.71
18	bA	4012	BCR	C38-C26-C25	-3.76	120.33	124.51
14	aA	1110	CLA	O2D-CGD-O1D	-3.76	116.41	123.83
18	BA	4006	BCR	C7-C8-C9	3.76	131.85	126.21
14	bA	1022	CLA	O2A-CGA-CBA	3.75	123.98	111.93
18	bA	4013	BCR	C7-C8-C9	-3.75	120.57	126.21
14	HA	1216	CLA	O2A-CGA-CBA	3.75	123.98	111.93
14	HA	1224	CLA	CMB-C2B-C3B	3.75	131.84	124.80
18	bA	4012	BCR	C33-C5-C6	-3.75	120.33	124.51
14	AA	1013	CLA	C2C-C1C-NC	3.75	113.49	109.97
14	0	1012	CLA	O2A-CGA-CBA	3.75	123.98	111.93
18	RA	4018	BCR	C34-C9-C10	-3.75	117.67	122.92
18	BA	4012	BCR	C38-C26-C25	-3.75	120.33	124.51
18	UA	4019	BCR	C38-C26-C25	-3.75	120.33	124.51
14	BA	1228	CLA	CMB-C2B-C3B	3.75	131.83	124.80
14	BA	1238	CLA	C4A-NA-C1A	3.75	108.39	106.71
14	BA	1234	CLA	O2A-C1-C2	3.75	118.49	108.64
14	HA	1234	CLA	O2A-C1-C2	3.75	118.49	108.64
18	bA	4017	BCR	C15-C14-C13	-3.75	121.96	127.31
14	bA	1228	CLA	CMB-C2B-C3B	3.75	131.83	124.80
14	bA	1224	CLA	CMB-C2B-C3B	3.75	131.83	124.80
14	bA	1234	CLA	O2A-C1-C2	3.75	118.48	108.64
14	aA	1013	CLA	O2A-CGA-CBA	3.74	123.95	111.93
14	GA	1110	CLA	O2D-CGD-O1D	-3.74	116.44	123.83
18	bA	4009	BCR	C33-C5-C6	-3.74	120.34	124.51
18	HA	4009	BCR	C33-C5-C6	-3.74	120.34	124.51
14	bA	1214	CLA	O2A-CGA-CBA	3.74	123.94	111.93
14	BA	1214	CLA	O2A-CGA-CBA	3.74	123.94	111.93
18	HA	4013	BCR	C7-C8-C9	-3.74	120.59	126.21
18	bA	4005	BCR	C33-C5-C6	-3.74	120.35	124.51
14	HA	1214	CLA	O2A-CGA-CBA	3.74	123.92	111.93
14	AA	1013	CLA	O2A-CGA-CBA	3.73	123.92	111.93
18	RA	4020	BCR	C35-C13-C12	3.73	124.00	118.09
14	HA	1023	CLA	CAA-C2A-C3A	-3.73	102.57	112.78
14	bA	1023	CLA	CAA-C2A-C3A	-3.73	102.58	112.78
14	GA	1013	CLA	O2A-CGA-CBA	3.72	123.89	111.93
18	IA	4020	BCR	C35-C13-C12	3.72	123.99	118.09
14	BA	1023	CLA	CAA-C2A-C3A	-3.72	102.60	112.78
18	HA	4005	BCR	C33-C5-C6	-3.72	120.37	124.51
14	AA	1122	CLA	O2A-CGA-CBA	3.72	123.86	111.93
18	BA	4017	BCR	C15-C14-C13	-3.72	122.01	127.31
18	BA	4005	BCR	C37-C22-C21	-3.71	117.72	122.92

Continued on next page...

PRELIMINARY VALIDATION REPORT

Continued from previous page...

Mol	Chain	Res	Type	Atoms	Z	Observed(°)	Ideal(°)
18	iA	4020	BCR	C35-C13-C12	3.71	123.98	118.09
18	BA	4005	BCR	C33-C5-C6	-3.71	120.38	124.51
14	bA	1231	CLA	CMC-C2C-C1C	3.70	130.67	125.03
18	HA	4012	BCR	C38-C26-C25	-3.70	120.39	124.51
14	BA	1210	CLA	CMB-C2B-C3B	3.70	131.74	124.80
14	aA	1122	CLA	O2A-CGA-CBA	3.70	123.82	111.93
14	HA	1210	CLA	CMB-C2B-C3B	3.70	131.74	124.80
14	bA	1224	CLA	O2A-CGA-CBA	3.70	123.81	111.93
18	LA	4019	BCR	C38-C26-C25	-3.70	120.39	124.51
14	bA	1210	CLA	CMB-C2B-C3B	3.70	131.74	124.80
14	GA	1115	CLA	CMB-C2B-C3B	3.70	131.74	124.80
14	AA	1125	CLA	O2D-CGD-O1D	-3.70	116.53	123.83
14	HA	1224	CLA	O2A-CGA-CBA	3.70	123.80	111.93
18	HA	4005	BCR	C37-C22-C21	-3.69	117.75	122.92
18	HA	4017	BCR	C7-C8-C9	-3.69	120.67	126.21
14	AA	1115	CLA	CMB-C2B-C3B	3.69	131.72	124.80
14	BA	1224	CLA	O2A-CGA-CBA	3.69	123.77	111.93
14	HA	1234	CLA	C1C-C2C-C3C	-3.68	103.05	106.95
14	HA	1223	CLA	O2A-CGA-CBA	3.68	123.75	111.93
14	GA	1125	CLA	O2D-CGD-O1D	-3.68	116.56	123.83
18	bA	4017	BCR	C7-C8-C9	-3.68	120.68	126.21
14	HA	1234	CLA	C3C-C4C-NC	3.68	114.70	110.57
18	bA	4005	BCR	C37-C22-C21	-3.68	117.77	122.92
14	BA	1231	CLA	CMC-C2C-C1C	3.68	130.63	125.03
14	GA	1122	CLA	O2A-CGA-CBA	3.68	123.74	111.93
18	BA	4017	BCR	C7-C8-C9	-3.67	120.69	126.21
14	bA	1223	CLA	O2A-CGA-CBA	3.67	123.71	111.93
14	bA	1234	CLA	C1C-C2C-C3C	-3.67	103.06	106.95
14	BA	1223	CLA	O2A-CGA-CBA	3.67	123.70	111.93
14	bA	1234	CLA	C3C-C4C-NC	3.67	114.68	110.57
14	HA	1231	CLA	CMC-C2C-C1C	3.67	130.61	125.03
14	BA	1234	CLA	C1C-C2C-C3C	-3.66	103.07	106.95
14	aA	1115	CLA	CMB-C2B-C3B	3.66	131.66	124.80
14	aA	1125	CLA	O2D-CGD-O1D	-3.66	116.61	123.83
14	bA	1235	CLA	CAA-C2A-C3A	-3.65	102.77	112.78
14	BA	1234	CLA	C3C-C4C-NC	3.65	114.67	110.57
18	bA	4009	BCR	C3-C4-C5	-3.65	107.63	113.99
14	HA	1235	CLA	CAA-C2A-C3A	-3.65	102.78	112.78
14	aA	1118	CLA	O2A-CGA-CBA	3.65	123.65	111.93
18	HA	4017	BCR	C15-C14-C13	-3.64	122.11	127.31
14	AA	1123	CLA	CMB-C2B-C3B	3.64	131.63	124.80
14	bA	1021	CLA	CAA-CBA-CGA	-3.64	102.52	113.26

Continued on next page...

PRELIMINARY VALIDATION REPORT

Continued from previous page...

Mol	Chain	Res	Type	Atoms	Z	Observed(^{o})	Ideal(^{o})
14	HA	1021	CLA	CAA-CBA-CGA	-3.64	102.53	113.26
18	RA	4020	BCR	C3-C4-C5	-3.64	107.66	113.99
18	BA	4009	BCR	C3-C4-C5	-3.64	107.66	113.99
18	bA	4006	BCR	C3-C4-C5	-3.64	107.66	113.99
14	BA	1223	CLA	C3C-C4C-NC	3.64	114.65	110.57
14	GA	1132	CLA	CAA-C2A-C3A	-3.64	102.82	112.78
14	AA	1105	CLA	CMB-C2B-C3B	3.64	131.62	124.80
14	AA	1132	CLA	CAA-C2A-C3A	-3.63	102.83	112.78
14	BA	1235	CLA	CAA-C2A-C3A	-3.63	102.83	112.78
14	AA	1118	CLA	O2A-CGA-CBA	3.63	123.59	111.93
14	BA	1021	CLA	CAA-CBA-CGA	-3.63	102.56	113.26
18	AA	4002	BCR	C38-C26-C25	-3.63	120.47	124.51
18	HA	4006	BCR	C3-C4-C5	-3.63	107.68	113.99
14	aA	1132	CLA	CAA-C2A-C3A	-3.63	102.84	112.78
14	HA	1223	CLA	C3C-C4C-NC	3.63	114.64	110.57
14	GA	1123	CLA	CMB-C2B-C3B	3.63	131.60	124.80
18	HA	4009	BCR	C3-C4-C5	-3.62	107.69	113.99
14	GA	1118	CLA	O2A-CGA-CBA	3.62	123.56	111.93
18	BA	4010	BCR	C15-C14-C13	-3.62	122.14	127.31
14	aA	1105	CLA	CMB-C2B-C3B	3.62	131.59	124.80
18	iA	4020	BCR	C3-C4-C5	-3.62	107.69	113.99
18	lA	4019	BCR	C36-C18-C17	-3.62	117.86	122.92
14	BA	1208	CLA	CAA-C2A-C3A	-3.62	102.88	112.78
18	IA	4020	BCR	C3-C4-C5	-3.61	107.70	113.99
14	bA	1220	CLA	CMB-C2B-C3B	3.61	131.58	124.80
18	bA	4006	BCR	C34-C9-C8	3.61	123.82	118.09
14	aA	1123	CLA	CMB-C2B-C3B	3.61	131.58	124.80
14	GA	1105	CLA	CMB-C2B-C3B	3.61	131.57	124.80
18	bA	4010	BCR	C15-C14-C13	-3.61	122.16	127.31
14	AA	1102	CLA	O2A-CGA-CBA	3.61	123.52	111.93
18	GA	4003	BCR	C28-C27-C26	-3.61	107.71	113.99
18	BA	4006	BCR	C34-C9-C8	3.61	123.81	118.09
14	GA	1123	CLA	O2A-CGA-CBA	3.61	123.51	111.93
14	HA	1208	CLA	CAA-C2A-C3A	-3.61	102.91	112.78
18	LA	4019	BCR	C36-C18-C17	-3.61	117.87	122.92
18	GA	4002	BCR	C38-C26-C25	-3.60	120.50	124.51
18	aA	4003	BCR	C28-C27-C26	-3.60	107.72	113.99
18	BA	4006	BCR	C3-C4-C5	-3.60	107.72	113.99
14	HA	1220	CLA	CMB-C2B-C3B	3.60	131.55	124.80
14	aA	1102	CLA	O2A-CGA-CBA	3.60	123.49	111.93
14	bA	1208	CLA	CAA-C2A-C3A	-3.60	102.92	112.78
18	HA	4010	BCR	C15-C14-C13	-3.60	122.17	127.31

Continued on next page...

PRELIMINARY VALIDATION REPORT

Continued from previous page...

Mol	Chain	Res	Type	Atoms	Z	Observed(°)	Ideal(°)
14	UA	1503	CLA	CAA-C2A-C3A	-3.60	102.93	112.78
14	AA	1123	CLA	O2A-CGA-CBA	3.60	123.48	111.93
14	lA	1503	CLA	CAA-C2A-C3A	-3.60	102.93	112.78
14	bA	1212	CLA	O2D-CGD-O1D	-3.59	116.73	123.83
14	LA	1503	CLA	CAA-C2A-C3A	-3.59	102.94	112.78
18	AA	4003	BCR	C28-C27-C26	-3.59	107.74	113.99
14	bA	1223	CLA	C3C-C4C-NC	3.59	114.60	110.57
14	AA	1139	CLA	CAA-C2A-C3A	-3.59	102.94	112.78
18	HA	4006	BCR	C34-C9-C8	3.59	123.78	118.09
14	GA	1137	CLA	O2D-CGD-O1D	-3.59	116.74	123.83
14	BA	1220	CLA	CMB-C2B-C3B	3.59	131.53	124.80
14	aA	1123	CLA	O2A-CGA-CBA	3.59	123.45	111.93
14	0	1012	CLA	O2D-CGD-O1D	-3.59	116.75	123.83
18	HA	4005	BCR	C38-C26-C25	-3.59	120.52	124.51
14	GA	1102	CLA	O2A-CGA-CBA	3.59	123.44	111.93
14	GA	1125	CLA	C1C-C2C-C3C	-3.58	103.15	106.95
14	0	1012	CLA	C3C-C4C-NC	3.58	114.59	110.57
14	GA	1139	CLA	CAA-C2A-C3A	-3.58	102.99	112.78
18	mA	4021	BCR	C7-C8-C9	3.58	131.58	126.21
14	aA	1139	CLA	CAA-C2A-C3A	-3.57	102.99	112.78
14	bA	1021	CLA	CMB-C2B-C3B	3.57	131.50	124.80
14	AA	1124	CLA	O2A-CGA-CBA	3.57	123.40	111.93
14	GA	1124	CLA	O2A-CGA-CBA	3.57	123.40	111.93
14	HA	1212	CLA	O2D-CGD-O1D	-3.57	116.77	123.83
14	aA	1119	CLA	O2A-CGA-CBA	3.57	123.40	111.93
14	aA	1012	CLA	C4A-NA-C1A	3.57	108.31	106.71
14	BA	1239	CLA	C1-C2-C3	-3.57	119.86	126.04
14	AA	1129	CLA	CMB-C2B-C3B	3.57	131.49	124.80
18	VA	4021	BCR	C7-C8-C9	3.57	131.57	126.21
14	aA	1124	CLA	O2A-CGA-CBA	3.57	123.38	111.93
18	aA	4002	BCR	C38-C26-C25	-3.57	120.54	124.51
14	AA	1119	CLA	O2A-CGA-CBA	3.57	123.38	111.93
14	GA	1111	CLA	CMB-C2B-C3B	3.56	131.49	124.80
14	BA	1021	CLA	CMB-C2B-C3B	3.56	131.48	124.80
14	AA	1137	CLA	O2D-CGD-O1D	-3.56	116.79	123.83
18	UA	4019	BCR	C36-C18-C17	-3.56	117.93	122.92
14	GA	1119	CLA	O2A-CGA-CBA	3.56	123.36	111.93
14	BA	1212	CLA	O2D-CGD-O1D	-3.56	116.80	123.83
18	aA	4003	BCR	C3-C4-C5	-3.56	107.80	113.99
14	HA	1021	CLA	CMB-C2B-C3B	3.56	131.47	124.80
18	AA	4003	BCR	C3-C4-C5	-3.56	107.80	113.99
14	bA	1022	CLA	C3C-C4C-NC	3.56	114.56	110.57

Continued on next page...

PRELIMINARY VALIDATION REPORT

Continued from previous page...

Mol	Chain	Res	Type	Atoms	Z	Observed(^{o})	Ideal(^{o})
14	bA	1239	CLA	C1-C2-C3	-3.56	119.89	126.04
14	bA	1210	CLA	O2D-CGD-O1D	-3.55	116.81	123.83
14	aA	1137	CLA	O2D-CGD-O1D	-3.55	116.81	123.83
18	bA	4005	BCR	C38-C26-C25	-3.55	120.56	124.51
14	BA	1022	CLA	O2D-CGD-O1D	-3.55	116.82	123.83
14	bA	1022	CLA	O2D-CGD-O1D	-3.55	116.82	123.83
14	HA	1210	CLA	O2D-CGD-O1D	-3.55	116.83	123.83
18	BA	4005	BCR	C38-C26-C25	-3.54	120.56	124.51
14	GA	1012	CLA	C4A-NA-C1A	3.54	108.30	106.71
18	GA	4003	BCR	C3-C4-C5	-3.54	107.83	113.99
14	aA	1129	CLA	CMB-C2B-C3B	3.54	131.44	124.80
14	GA	1129	CLA	CMB-C2B-C3B	3.54	131.44	124.80
14	aA	1111	CLA	CMB-C2B-C3B	3.54	131.43	124.80
14	AA	1125	CLA	C1C-C2C-C3C	-3.53	103.20	106.95
14	AA	1130	CLA	C4A-NA-C1A	3.53	108.30	106.71
14	AA	1111	CLA	CMB-C2B-C3B	3.53	131.43	124.80
14	GA	1109	CLA	CAA-C2A-C3A	-3.53	103.11	112.78
14	AA	1109	CLA	CAA-C2A-C3A	-3.53	103.11	112.78
14	BA	1022	CLA	C3C-C4C-NC	3.53	114.53	110.57
18	MA	4021	BCR	C7-C8-C9	3.53	131.51	126.21
14	HA	1239	CLA	C1-C2-C3	-3.53	119.95	126.04
14	AA	1115	CLA	C4-C3-C5	3.52	121.36	115.29
14	aA	1109	CLA	CAA-C2A-C3A	-3.52	103.13	112.78
21	aA	6002	LMT	O5B-C5B-C4B	3.52	116.11	109.68
14	BA	1210	CLA	O2D-CGD-O1D	-3.52	116.87	123.83
14	GA	1138	CLA	O2A-CGA-CBA	3.52	123.23	111.93
19	bA	5004	LHG	C5-O7-C7	-3.52	109.04	117.82
14	aA	1130	CLA	C4A-NA-C1A	3.52	108.29	106.71
19	BA	5004	LHG	C5-O7-C7	-3.52	109.05	117.82
14	GA	1130	CLA	C4A-NA-C1A	3.51	108.28	106.71
19	HA	5004	LHG	C5-O7-C7	-3.51	109.06	117.82
14	aA	1138	CLA	O2A-CGA-CBA	3.51	123.20	111.93
14	AA	1138	CLA	O2A-CGA-CBA	3.51	123.19	111.93
21	GA	6002	LMT	O5B-C5B-C4B	3.51	116.08	109.68
14	GA	1138	CLA	CAA-C2A-C3A	-3.50	103.18	112.78
14	aA	1125	CLA	C1C-C2C-C3C	-3.50	103.24	106.95
14	GA	1115	CLA	C4-C3-C5	3.50	121.32	115.29
14	BA	1221	CLA	CMB-C2B-C3B	3.50	131.37	124.80
14	AA	1138	CLA	CAA-C2A-C3A	-3.50	103.19	112.78
14	aA	1138	CLA	CAA-C2A-C3A	-3.50	103.19	112.78
14	HA	1221	CLA	CMB-C2B-C3B	3.50	131.36	124.80
14	BA	1211	CLA	O2A-CGA-CBA	3.50	123.16	111.93

Continued on next page...

PRELIMINARY VALIDATION REPORT

Continued from previous page...

Mol	Chain	Res	Type	Atoms	Z	Observed(°)	Ideal(°)
14	lA	1501	CLA	O2A-CGA-CBA	3.50	123.16	111.93
14	bA	1221	CLA	CMB-C2B-C3B	3.50	131.36	124.80
14	aA	1130	CLA	O2A-CGA-CBA	3.50	123.15	111.93
14	AA	1130	CLA	O2A-CGA-CBA	3.49	123.15	111.93
21	AA	6002	LMT	O5B-C5B-C4B	3.49	116.05	109.68
14	HA	1211	CLA	O2A-CGA-CBA	3.49	123.13	111.93
13	aA	1011	CL0	CBC-CAC-C3C	-3.49	102.75	112.43
14	aA	1115	CLA	C4-C3-C5	3.49	121.29	115.29
14	bA	1211	CLA	O2A-CGA-CBA	3.48	123.12	111.93
14	UA	1501	CLA	O2A-CGA-CBA	3.48	123.11	111.93
13	GA	1011	CL0	CBC-CAC-C3C	-3.48	102.77	112.43
14	GA	1130	CLA	O2A-CGA-CBA	3.48	123.09	111.93
14	AA	1012	CLA	C4A-NA-C1A	3.47	108.27	106.71
14	LA	1501	CLA	O2A-CGA-CBA	3.47	123.08	111.93
13	AA	1011	CL0	CBC-CAC-C3C	-3.47	102.79	112.43
14	aA	1012	CLA	OBD-CAD-C3D	-3.47	121.90	128.04
18	HA	4012	BCR	C7-C8-C9	-3.46	121.01	126.21
14	AA	1012	CLA	OBD-CAD-C3D	-3.45	121.92	128.04
14	GA	1012	CLA	OBD-CAD-C3D	-3.45	121.92	128.04
18	BA	4012	BCR	C7-C8-C9	-3.45	121.02	126.21
14	LA	1502	CLA	O2A-CGA-CBA	3.45	123.01	111.93
14	aA	1122	CLA	CAC-C3C-C4C	3.45	129.33	124.82
14	aA	1101	CLA	CAA-C2A-C3A	-3.45	103.34	112.78
14	GA	1122	CLA	CAC-C3C-C4C	3.45	129.32	124.82
18	bA	4012	BCR	C7-C8-C9	-3.45	121.03	126.21
14	lA	1501	CLA	C1-C2-C3	-3.45	120.08	126.04
14	UA	1502	CLA	O2A-CGA-CBA	3.44	122.98	111.93
14	GA	1126	CLA	CMB-C2B-C3B	3.44	131.25	124.80
14	lA	1502	CLA	O2A-CGA-CBA	3.44	122.97	111.93
14	GA	1012	CLA	CAA-C2A-C3A	-3.44	103.37	112.78
14	HA	1214	CLA	OBD-CAD-C3D	-3.43	121.95	128.04
14	AA	1101	CLA	CAA-C2A-C3A	-3.43	103.37	112.78
14	BA	1220	CLA	CMA-C3A-C4A	3.43	121.00	111.77
14	HA	1220	CLA	CMA-C3A-C4A	3.43	121.00	111.77
14	aA	1012	CLA	CAA-C2A-C3A	-3.43	103.38	112.78
14	aA	1126	CLA	CMB-C2B-C3B	3.43	131.23	124.80
14	BA	1022	CLA	CAA-C2A-C3A	-3.43	103.38	112.78
14	bA	1022	CLA	CAA-C2A-C3A	-3.43	103.39	112.78
14	AA	1126	CLA	CMB-C2B-C3B	3.43	131.23	124.80
14	GA	1101	CLA	CAA-C2A-C3A	-3.43	103.39	112.78
18	GA	4007	BCR	C15-C14-C13	-3.43	122.42	127.31
14	GA	1136	CLA	O2D-CGD-O1D	-3.43	117.06	123.83

Continued on next page...

PRELIMINARY VALIDATION REPORT

Continued from previous page...

Mol	Chain	Res	Type	Atoms	Z	Observed(^{o})	Ideal(^{o})
14	BA	1214	CLA	OBD-CAD-C3D	-3.43	121.97	128.04
14	bA	1220	CLA	CMA-C3A-C4A	3.43	120.98	111.77
14	GA	1125	CLA	C3C-C4C-NC	3.42	114.41	110.57
14	0	1012	CLA	CAA-C2A-C3A	-3.42	103.41	112.78
14	AA	1122	CLA	CAC-C3C-C4C	3.42	129.29	124.82
14	bA	1214	CLA	OBD-CAD-C3D	-3.42	121.99	128.04
14	bA	1206	CLA	OBD-CAD-CBD	-3.42	120.89	125.91
18	AA	4007	BCR	C15-C14-C13	-3.41	122.44	127.31
14	HA	1206	CLA	OBD-CAD-CBD	-3.41	120.90	125.91
14	aA	1125	CLA	C3C-C4C-NC	3.41	114.40	110.57
14	HA	1210	CLA	C4-C3-C5	3.41	121.16	115.29
14	AA	1012	CLA	CAA-C2A-C3A	-3.41	103.45	112.78
14	GA	1013	CLA	C1C-C2C-C3C	-3.41	103.34	106.95
14	LA	1501	CLA	C1-C2-C3	-3.41	120.15	126.04
14	bA	1210	CLA	C4-C3-C5	3.40	121.15	115.29
14	UA	1501	CLA	C1-C2-C3	-3.40	120.16	126.04
14	LA	1501	CLA	OBD-CAD-C3D	-3.40	122.01	128.04
14	AA	1125	CLA	C3C-C4C-NC	3.40	114.39	110.57
14	AA	1135	CLA	CAA-C2A-C3A	-3.40	103.46	112.78
18	HA	4006	BCR	C1-C6-C5	-3.40	117.84	122.59
18	bA	4006	BCR	C1-C6-C5	-3.40	117.84	122.59
14	aA	1135	CLA	CAA-C2A-C3A	-3.40	103.47	112.78
14	BA	1210	CLA	C4-C3-C5	3.40	121.14	115.29
14	lA	1501	CLA	OBD-CAD-C3D	-3.40	122.02	128.04
18	aA	4007	BCR	C15-C14-C13	-3.40	122.46	127.31
14	UA	1501	CLA	OBD-CAD-C3D	-3.39	122.03	128.04
14	AA	1136	CLA	O2D-CGD-O1D	-3.39	117.14	123.83
14	bA	1227	CLA	CAA-C2A-C3A	-3.39	103.50	112.78
14	aA	1105	CLA	CAA-C2A-C3A	-3.39	103.50	112.78
14	GA	1135	CLA	CAA-C2A-C3A	-3.38	103.51	112.78
14	BA	1206	CLA	OBD-CAD-CBD	-3.38	120.94	125.91
14	BA	1215	CLA	C4-C3-C5	3.38	121.11	115.29
14	LA	1503	CLA	O2A-CGA-CBA	3.38	122.78	111.93
14	BA	1225	CLA	C3C-C4C-NC	3.38	114.36	110.57
14	GA	1105	CLA	CAA-C2A-C3A	-3.38	103.53	112.78
14	UA	1503	CLA	O2A-CGA-CBA	3.38	122.77	111.93
14	BA	1227	CLA	CAA-C2A-C3A	-3.38	103.53	112.78
14	AA	1105	CLA	CAA-C2A-C3A	-3.38	103.53	112.78
14	bA	1225	CLA	C3C-C4C-NC	3.37	114.35	110.57
14	GA	1103	CLA	O2D-CGD-O1D	-3.37	117.17	123.83
14	HA	1021	CLA	CMC-C2C-C1C	3.37	130.16	125.03
18	BA	4006	BCR	C1-C6-C5	-3.37	117.88	122.59

Continued on next page...

PRELIMINARY VALIDATION REPORT

Continued from previous page...

Mol	Chain	Res	Type	Atoms	Z	Observed(°)	Ideal(°)
14	HA	1205	CLA	O2D-CGD-O1D	-3.37	117.18	123.83
14	aA	1136	CLA	O2D-CGD-O1D	-3.37	117.18	123.83
14	lA	1503	CLA	O2A-CGA-CBA	3.37	122.74	111.93
14	HA	1225	CLA	C3C-C4C-NC	3.36	114.34	110.57
13	aA	1011	CL0	C4-C3-C5	3.36	121.08	115.29
18	BA	4012	BCR	C33-C5-C4	3.36	119.87	113.57
14	HA	1227	CLA	CAA-C2A-C3A	-3.36	103.57	112.78
18	BA	4009	BCR	C33-C5-C4	3.36	119.86	113.57
18	bA	4012	BCR	C33-C5-C4	3.36	119.86	113.57
18	HA	4009	BCR	C33-C5-C4	3.36	119.86	113.57
14	BA	1205	CLA	O2D-CGD-O1D	-3.36	117.20	123.83
14	BA	1021	CLA	CMC-C2C-C1C	3.36	130.14	125.03
14	aA	1013	CLA	C1C-C2C-C3C	-3.36	103.39	106.95
14	HA	1215	CLA	C4-C3-C5	3.35	121.07	115.29
13	GA	1011	CL0	C4-C3-C5	3.35	121.06	115.29
14	GA	1113	CLA	OBD-CAD-C3D	-3.35	122.10	128.04
14	AA	1103	CLA	O2D-CGD-O1D	-3.35	117.21	123.83
18	HA	4012	BCR	C33-C5-C4	3.35	119.84	113.57
14	bA	1021	CLA	CMC-C2C-C1C	3.35	130.13	125.03
18	bA	4009	BCR	C33-C5-C4	3.35	119.84	113.57
14	HA	1213	CLA	CAA-C2A-C3A	-3.35	103.61	112.78
14	bA	1215	CLA	C4-C3-C5	3.35	121.05	115.29
14	AA	1113	CLA	OBD-CAD-C3D	-3.34	122.11	128.04
14	bA	1219	CLA	CMB-C2B-C3B	3.34	131.07	124.80
14	HA	1219	CLA	CMB-C2B-C3B	3.34	131.07	124.80
14	GA	1115	CLA	CAA-C2A-C3A	-3.34	103.63	112.78
14	aA	1101	CLA	O2D-CGD-O1D	-3.34	117.23	123.83
14	aA	1113	CLA	OBD-CAD-C3D	-3.34	122.12	128.04
14	aA	1103	CLA	O2D-CGD-O1D	-3.34	117.24	123.83
13	AA	1011	CL0	C4-C3-C5	3.33	121.03	115.29
14	BA	1223	CLA	O2D-CGD-O1D	-3.33	117.25	123.83
14	AA	1115	CLA	CAA-C2A-C3A	-3.33	103.65	112.78
18	MA	4021	BCR	C8-C7-C6	3.33	136.61	127.28
14	GA	1101	CLA	O2D-CGD-O1D	-3.33	117.25	123.83
18	VA	4021	BCR	C8-C7-C6	3.33	136.60	127.28
14	aA	1118	CLA	CAA-C2A-C3A	-3.33	103.66	112.78
18	RA	4020	BCR	C36-C18-C17	-3.33	118.26	122.92
14	BA	1213	CLA	CAA-C2A-C3A	-3.33	103.67	112.78
18	mA	4021	BCR	C8-C7-C6	3.33	136.59	127.28
14	bA	1205	CLA	O2D-CGD-O1D	-3.33	117.26	123.83
14	AA	1013	CLA	C1C-C2C-C3C	-3.33	103.43	106.95
14	AA	1101	CLA	O2D-CGD-O1D	-3.33	117.26	123.83

Continued on next page...

PRELIMINARY VALIDATION REPORT

Continued from previous page...

Mol	Chain	Res	Type	Atoms	Z	Observed(°)	Ideal(°)
18	iA	4020	BCR	C36-C18-C17	-3.32	118.27	122.92
14	aA	1115	CLA	CAA-C2A-C3A	-3.32	103.68	112.78
14	GA	1118	CLA	CAA-C2A-C3A	-3.32	103.68	112.78
14	AA	1118	CLA	CAA-C2A-C3A	-3.32	103.68	112.78
14	bA	1213	CLA	CAA-C2A-C3A	-3.32	103.68	112.78
18	IA	4020	BCR	C36-C18-C17	-3.32	118.27	122.92
18	bA	4013	BCR	C28-C27-C26	-3.32	108.22	113.99
18	BA	4009	BCR	C34-C9-C8	3.32	123.35	118.09
18	BA	4013	BCR	C28-C27-C26	-3.31	108.23	113.99
14	bA	1224	CLA	CMC-C2C-C1C	3.31	130.07	125.03
14	BA	1224	CLA	CMC-C2C-C1C	3.31	130.07	125.03
14	GA	1140	CLA	C3C-C4C-NC	3.31	114.28	110.57
14	AA	1140	CLA	C3C-C4C-NC	3.31	114.28	110.57
18	HA	4013	BCR	C28-C27-C26	-3.31	108.23	113.99
14	BA	1219	CLA	CMB-C2B-C3B	3.31	131.00	124.80
14	AA	1117	CLA	CBA-CAA-C2A	3.31	123.69	113.85
18	BA	4005	BCR	C38-C26-C27	3.31	119.76	113.57
14	aA	1117	CLA	CBA-CAA-C2A	3.30	123.69	113.85
18	HA	4005	BCR	C38-C26-C27	3.30	119.76	113.57
18	bA	4005	BCR	C38-C26-C27	3.30	119.76	113.57
14	GA	1123	CLA	CMA-C3A-C4A	3.30	120.65	111.77
14	aA	1123	CLA	CMA-C3A-C4A	3.30	120.65	111.77
14	bA	1223	CLA	O2D-CGD-O1D	-3.30	117.31	123.83
14	GA	1117	CLA	CBA-CAA-C2A	3.30	123.67	113.85
14	HA	1224	CLA	CMC-C2C-C1C	3.30	130.05	125.03
18	aA	4007	BCR	C38-C26-C25	-3.29	120.84	124.51
14	AA	1106	CLA	O2D-CGD-O1D	-3.29	117.33	123.83
14	HA	1223	CLA	O2D-CGD-O1D	-3.29	117.33	123.83
14	AA	1123	CLA	CMA-C3A-C4A	3.29	120.62	111.77
18	AA	4007	BCR	C38-C26-C25	-3.29	120.84	124.51
14	GA	1106	CLA	O2D-CGD-O1D	-3.29	117.33	123.83
14	BA	1238	CLA	C4-C3-C5	3.29	120.95	115.29
14	HA	1201	CLA	C4-C3-C5	3.29	120.95	115.29
14	aA	1106	CLA	O2D-CGD-O1D	-3.28	117.34	123.83
14	AA	1013	CLA	C3C-C4C-NC	3.28	114.25	110.57
18	GA	4002	BCR	C28-C27-C26	-3.28	108.28	113.99
14	bA	1238	CLA	C4-C3-C5	3.27	120.93	115.29
18	bA	4009	BCR	C34-C9-C8	3.27	123.28	118.09
18	LA	4019	BCR	C33-C5-C4	3.27	119.69	113.57
18	AA	4002	BCR	C28-C27-C26	-3.27	108.30	113.99
18	aA	4002	BCR	C28-C27-C26	-3.27	108.30	113.99
14	aA	1013	CLA	C1-C2-C3	-3.27	120.39	126.04

Continued on next page...

PRELIMINARY VALIDATION REPORT

Continued from previous page...

Mol	Chain	Res	Type	Atoms	Z	Observed(^{o})	Ideal(^{o})
18	HA	4009	BCR	C34-C9-C8	3.27	123.27	118.09
14	bA	1201	CLA	C4-C3-C5	3.27	120.92	115.29
14	HA	1238	CLA	C4-C3-C5	3.27	120.92	115.29
14	bA	1204	CLA	CAA-C2A-C3A	-3.27	103.83	112.78
14	HA	1224	CLA	C3C-C4C-NC	3.27	114.23	110.57
14	bA	1021	CLA	C3C-C4C-NC	3.27	114.23	110.57
14	AA	1013	CLA	C1-C2-C3	-3.26	120.40	126.04
14	bA	1209	CLA	O2D-CGD-O1D	-3.26	117.39	123.83
14	aA	1140	CLA	C3C-C4C-NC	3.26	114.23	110.57
19	aA	5001	LHG	O8-C23-C24	3.26	122.40	111.93
14	BA	1204	CLA	CAA-C2A-C3A	-3.26	103.85	112.78
14	BA	1209	CLA	O2D-CGD-O1D	-3.26	117.39	123.83
13	AA	1011	CL0	CAA-CBA-CGA	-3.26	103.65	113.26
14	HA	1204	CLA	CAA-C2A-C3A	-3.26	103.86	112.78
14	BA	1201	CLA	C4-C3-C5	3.26	120.90	115.29
14	aA	1013	CLA	C3C-C4C-NC	3.26	114.22	110.57
18	bA	4017	BCR	C34-C9-C10	-3.26	118.36	122.92
18	mA	4021	BCR	C3-C4-C5	-3.26	108.33	113.99
18	GA	4007	BCR	C38-C26-C25	-3.26	120.89	124.51
14	bA	1213	CLA	CMB-C2B-C3B	3.25	130.90	124.80
14	bA	1022	CLA	OBD-CAD-CBD	-3.25	121.14	125.91
18	BA	4017	BCR	C34-C9-C10	-3.25	118.37	122.92
14	UA	1501	CLA	C3C-C4C-NC	3.25	114.21	110.57
13	GA	1011	CL0	CAA-CBA-CGA	-3.25	103.69	113.26
18	lA	4019	BCR	C33-C5-C4	3.25	119.65	113.57
18	MA	4021	BCR	C3-C4-C5	-3.25	108.34	113.99
14	GA	1013	CLA	C3C-C4C-NC	3.24	114.21	110.57
14	HA	1209	CLA	O2D-CGD-O1D	-3.24	117.42	123.83
18	VA	4021	BCR	C3-C4-C5	-3.24	108.35	113.99
19	AA	5001	LHG	O8-C23-C24	3.24	122.34	111.93
14	BA	1022	CLA	OBD-CAD-CBD	-3.24	121.15	125.91
14	aA	1136	CLA	C4-C3-C5	3.24	120.86	115.29
18	bA	4010	BCR	C7-C8-C9	-3.24	121.35	126.21
14	GA	1013	CLA	C1-C2-C3	-3.24	120.44	126.04
19	GA	5001	LHG	O8-C23-C24	3.24	122.33	111.93
18	UA	4019	BCR	C33-C5-C4	3.24	119.63	113.57
14	0	1012	CLA	OBD-CAD-CBD	-3.24	121.16	125.91
13	aA	1011	CL0	CAA-CBA-CGA	-3.23	103.73	113.26
14	bA	1224	CLA	C3C-C4C-NC	3.23	114.19	110.57
14	AA	1103	CLA	CMB-C2B-C3B	3.23	130.86	124.80
14	BA	1224	CLA	C3C-C4C-NC	3.23	114.19	110.57
14	bA	1222	CLA	CAA-C2A-C1A	-3.23	101.40	111.97

Continued on next page...

PRELIMINARY VALIDATION REPORT

Continued from previous page...

Mol	Chain	Res	Type	Atoms	Z	Observed(^{o})	Ideal(^{o})
14	BA	1213	CLA	CMB-C2B-C3B	3.23	130.85	124.80
16	GA	2001	PQN	C14-C13-C15	3.23	120.84	115.29
14	HA	1213	CLA	CMB-C2B-C3B	3.23	130.85	124.80
18	HA	4010	BCR	C7-C8-C9	-3.23	121.36	126.21
14	bA	1218	CLA	CMB-C2B-C3B	3.23	130.85	124.80
14	HA	1222	CLA	CAA-C2A-C1A	-3.22	101.41	111.97
14	aA	1103	CLA	CMB-C2B-C3B	3.22	130.84	124.80
18	HA	4004	BCR	C38-C26-C27	3.22	119.60	113.57
14	GA	1104	CLA	CMB-C2B-C3B	3.22	130.84	124.80
14	aA	1104	CLA	CMB-C2B-C3B	3.22	130.84	124.80
18	BA	4010	BCR	C7-C8-C9	-3.22	121.37	126.21
14	BA	1222	CLA	CAA-C2A-C1A	-3.22	101.42	111.97
14	BA	1021	CLA	C3C-C4C-NC	3.22	114.18	110.57
14	GA	1136	CLA	C4-C3-C5	3.22	120.83	115.29
14	BA	1205	CLA	CBA-CAA-C2A	3.22	123.43	113.85
14	lA	1501	CLA	C3C-C4C-NC	3.22	114.18	110.57
18	GA	4001	BCR	C3-C4-C5	-3.21	108.40	113.99
14	HA	1229	CLA	O2A-C1-C2	3.21	117.08	108.64
14	AA	1104	CLA	CMB-C2B-C3B	3.21	130.82	124.80
14	BA	1218	CLA	CMB-C2B-C3B	3.21	130.82	124.80
16	aA	2001	PQN	C14-C13-C15	3.21	120.82	115.29
14	aA	1131	CLA	CMB-C2B-C3B	3.21	130.82	124.80
14	HA	1218	CLA	CMB-C2B-C3B	3.21	130.82	124.80
16	AA	2001	PQN	C14-C13-C15	3.21	120.81	115.29
18	AA	4001	BCR	C3-C4-C5	-3.21	108.41	113.99
18	bA	4004	BCR	C38-C26-C27	3.21	119.58	113.57
14	HA	1205	CLA	CBA-CAA-C2A	3.21	123.39	113.85
14	LA	1501	CLA	C3C-C4C-NC	3.21	114.17	110.57
18	bA	4009	BCR	C38-C26-C27	3.21	119.57	113.57
14	bA	1205	CLA	CBA-CAA-C2A	3.21	123.39	113.85
18	HA	4017	BCR	C34-C9-C10	-3.21	118.43	122.92
18	BA	4006	BCR	C33-C5-C4	3.21	119.57	113.57
14	bA	1229	CLA	O2A-C1-C2	3.20	117.06	108.64
18	BA	4009	BCR	C38-C26-C27	3.20	119.57	113.57
14	BA	1212	CLA	C3C-C4C-NC	3.20	114.16	110.57
14	AA	1131	CLA	CMB-C2B-C3B	3.20	130.80	124.80
14	HA	1021	CLA	C3C-C4C-NC	3.20	114.16	110.57
14	AA	1129	CLA	CMC-C2C-C1C	3.20	129.90	125.03
18	UA	4022	BCR	C33-C5-C4	3.20	119.56	113.57
14	AA	1116	CLA	CMC-C2C-C1C	3.20	129.90	125.03
14	BA	1021	CLA	C4-C3-C5	3.20	120.80	115.29
14	aA	1130	CLA	CAA-C2A-C3A	-3.20	104.02	112.78

Continued on next page...

PRELIMINARY VALIDATION REPORT

Continued from previous page...

Mol	Chain	Res	Type	Atoms	Z	Observed(^{o})	Ideal(^{o})
14	bA	1222	CLA	CAA-CBA-CGA	-3.20	103.84	113.26
14	GA	1130	CLA	CAA-C2A-C3A	-3.20	104.03	112.78
14	aA	1116	CLA	CMC-C2C-C1C	3.20	129.89	125.03
14	HA	1222	CLA	CAA-CBA-CGA	-3.19	103.84	113.26
14	HA	1021	CLA	C4-C3-C5	3.19	120.79	115.29
18	HA	4006	BCR	C33-C5-C4	3.19	119.55	113.57
18	lA	4022	BCR	C33-C5-C4	3.19	119.55	113.57
14	AA	1136	CLA	CMA-C3A-C4A	3.19	120.36	111.77
14	BA	1229	CLA	O2A-C1-C2	3.19	117.03	108.64
14	GA	1131	CLA	CMB-C2B-C3B	3.19	130.79	124.80
14	aA	1136	CLA	CMA-C3A-C4A	3.19	120.35	111.77
18	RA	4018	BCR	C33-C5-C4	3.19	119.54	113.57
14	GA	1103	CLA	CMB-C2B-C3B	3.19	130.78	124.80
14	HA	1202	CLA	O2D-CGD-O1D	-3.19	117.53	123.83
18	BA	4004	BCR	C38-C26-C27	3.19	119.54	113.57
14	AA	1135	CLA	C1-C2-C3	-3.19	121.59	126.75
14	BA	1202	CLA	O2D-CGD-O1D	-3.19	117.54	123.83
14	BA	1222	CLA	CAA-CBA-CGA	-3.19	103.87	113.26
18	HA	4009	BCR	C38-C26-C27	3.19	119.54	113.57
13	aA	1011	CL0	O2D-CGD-O1D	-3.19	117.54	123.83
14	AA	1136	CLA	C4-C3-C5	3.19	120.78	115.29
14	GA	1136	CLA	CMA-C3A-C4A	3.19	120.34	111.77
14	HA	1232	CLA	CMB-C2B-C3B	3.18	130.77	124.80
14	AA	1104	CLA	CAA-C2A-C3A	-3.18	104.06	112.78
18	bA	4010	BCR	C36-C18-C17	-3.18	118.46	122.92
14	GA	1116	CLA	CMC-C2C-C1C	3.18	129.87	125.03
18	bA	4006	BCR	C33-C5-C4	3.18	119.53	113.57
14	GA	1138	CLA	O2D-CGD-O1D	-3.18	117.55	123.83
18	LA	4022	BCR	C33-C5-C4	3.18	119.52	113.57
14	GA	1104	CLA	CAA-C2A-C3A	-3.18	104.07	112.78
18	aA	4001	BCR	C3-C4-C5	-3.18	108.46	113.99
18	iA	4018	BCR	C33-C5-C4	3.18	119.52	113.57
14	AA	1130	CLA	CAA-C2A-C3A	-3.17	104.09	112.78
14	aA	1104	CLA	CAA-C2A-C3A	-3.17	104.09	112.78
18	GA	4003	BCR	C27-C26-C25	-3.17	118.11	122.74
14	GA	1129	CLA	CMC-C2C-C1C	3.17	129.86	125.03
13	AA	1011	CL0	O2D-CGD-O1D	-3.17	117.57	123.83
14	bA	1021	CLA	C4-C3-C5	3.17	120.74	115.29
14	GA	1135	CLA	C1-C2-C3	-3.17	121.63	126.75
14	WA	1701	CLA	CAA-C2A-C3A	-3.17	104.11	112.78
14	bA	1224	CLA	CAA-C2A-C3A	-3.17	104.11	112.78
14	xA	1701	CLA	CAA-C2A-C3A	-3.17	104.11	112.78

Continued on next page...

PRELIMINARY VALIDATION REPORT

Continued from previous page...

Mol	Chain	Res	Type	Atoms	Z	Observed(°)	Ideal(°)
18	IA	4018	BCR	C33-C5-C4	3.17	119.50	113.57
14	aA	1135	CLA	C1-C2-C3	-3.17	121.63	126.75
14	AA	1138	CLA	O2D-CGD-O1D	-3.17	117.58	123.83
14	AA	1102	CLA	CAA-C2A-C3A	-3.17	104.11	112.78
14	bA	1203	CLA	OBD-CAD-C3D	-3.17	122.43	128.04
14	aA	1138	CLA	O2D-CGD-O1D	-3.16	117.58	123.83
14	aA	1129	CLA	CMC-C2C-C1C	3.16	129.84	125.03
14	bA	1232	CLA	CMB-C2B-C3B	3.16	130.73	124.80
14	BA	1232	CLA	CMB-C2B-C3B	3.16	130.72	124.80
14	bA	1202	CLA	O2D-CGD-O1D	-3.16	117.59	123.83
14	HA	1224	CLA	CAA-C2A-C3A	-3.16	104.13	112.78
14	AA	1138	CLA	O2A-C1-C2	3.16	116.93	108.64
14	GA	1102	CLA	C1-C2-C3	-3.16	120.58	126.04
14	HA	1203	CLA	OBD-CAD-C3D	-3.16	122.45	128.04
14	XA	1701	CLA	CAA-C2A-C3A	-3.16	104.14	112.78
14	AA	1102	CLA	C1-C2-C3	-3.16	120.59	126.04
14	GA	1102	CLA	CAA-C2A-C3A	-3.15	104.14	112.78
14	BA	1224	CLA	CAA-C2A-C3A	-3.15	104.14	112.78
13	GA	1011	CL0	O2D-CGD-O1D	-3.15	117.60	123.83
14	HA	1207	CLA	O2D-CGD-O1D	-3.15	117.60	123.83
14	aA	1104	CLA	C1-C2-C3	-3.15	120.59	126.04
14	BA	1206	CLA	CMB-C2B-C3B	3.15	130.71	124.80
14	HA	1212	CLA	CMB-C2B-C3B	3.15	130.71	124.80
14	aA	1102	CLA	C1-C2-C3	-3.15	120.59	126.04
14	bA	1206	CLA	CMB-C2B-C3B	3.15	130.70	124.80
14	aA	1102	CLA	CAA-C2A-C3A	-3.15	104.16	112.78
14	HA	1206	CLA	CMB-C2B-C3B	3.15	130.70	124.80
14	bA	1207	CLA	O2D-CGD-O1D	-3.15	117.62	123.83
18	HA	4010	BCR	C36-C18-C17	-3.15	118.52	122.92
14	bA	1212	CLA	CMB-C2B-C3B	3.15	130.70	124.80
14	GA	1138	CLA	O2A-C1-C2	3.14	116.90	108.64
14	aA	1138	CLA	O2A-C1-C2	3.14	116.90	108.64
18	IA	4020	BCR	C12-C13-C14	-3.14	114.12	118.94
14	AA	1104	CLA	C1-C2-C3	-3.14	120.61	126.04
18	aA	4003	BCR	C27-C26-C25	-3.14	118.16	122.74
18	bA	4014	BCR	C37-C22-C21	-3.14	118.52	122.92
18	BA	4010	BCR	C36-C18-C17	-3.14	118.52	122.92
18	iA	4020	BCR	C12-C13-C14	-3.14	114.12	118.94
14	BA	1220	CLA	C1-C2-C3	-3.14	120.61	126.04
18	AA	4003	BCR	C27-C26-C25	-3.14	118.16	122.74
14	AA	1124	CLA	CAA-C2A-C3A	-3.14	104.19	112.78
18	RA	4020	BCR	C12-C13-C14	-3.14	114.13	118.94

Continued on next page...

PRELIMINARY VALIDATION REPORT

Continued from previous page...

Mol	Chain	Res	Type	Atoms	Z	Observed(°)	Ideal(°)
18	HA	4014	BCR	C37-C22-C21	-3.14	118.53	122.92
14	BA	1212	CLA	CMB-C2B-C3B	3.14	130.68	124.80
14	aA	1140	CLA	O2D-CGD-O1D	-3.14	117.64	123.83
14	GA	1104	CLA	C1-C2-C3	-3.13	120.62	126.04
18	aA	4008	BCR	C38-C26-C25	-3.13	121.02	124.51
14	GA	1013	CLA	CMC-C2C-C1C	3.13	129.80	125.03
18	HA	4004	BCR	C36-C18-C17	-3.13	118.53	122.92
14	aA	1013	CLA	CMC-C2C-C1C	3.13	129.80	125.03
14	bA	1023	CLA	CBC-CAC-C3C	3.13	121.12	112.43
14	aA	1125	CLA	C4C-C3C-C2C	-3.13	102.36	106.89
14	HA	1220	CLA	C1-C2-C3	-3.13	120.63	126.04
14	AA	1140	CLA	O2D-CGD-O1D	-3.13	117.65	123.83
14	HA	1023	CLA	CBC-CAC-C3C	3.13	121.11	112.43
14	BA	1023	CLA	CBC-CAC-C3C	3.13	121.11	112.43
14	bA	1220	CLA	C1-C2-C3	-3.13	120.63	126.04
14	xA	1701	CLA	C3C-C4C-NC	3.13	114.08	110.57
14	XA	1701	CLA	C3C-C4C-NC	3.13	114.08	110.57
14	GA	1140	CLA	O2D-CGD-O1D	-3.12	117.66	123.83
14	UA	1503	CLA	C1-C2-C3	-3.12	120.64	126.04
18	LA	4022	BCR	C36-C18-C17	-3.12	118.55	122.92
18	bA	4004	BCR	C36-C18-C17	-3.12	118.55	122.92
14	aA	1124	CLA	CAA-C2A-C3A	-3.12	104.23	112.78
14	BA	1207	CLA	O2D-CGD-O1D	-3.12	117.67	123.83
14	GA	1124	CLA	CAA-C2A-C3A	-3.12	104.23	112.78
14	WA	1701	CLA	C3C-C4C-NC	3.12	114.07	110.57
18	BA	4014	BCR	C37-C22-C21	-3.12	118.56	122.92
14	bA	1212	CLA	C3C-C4C-NC	3.12	114.07	110.57
18	lA	4022	BCR	C36-C18-C17	-3.12	118.56	122.92
14	BA	1203	CLA	OBD-CAD-C3D	-3.11	122.52	128.04
14	GA	1125	CLA	C4C-C3C-C2C	-3.11	102.38	106.89
14	aA	1137	CLA	CMB-C2B-C3B	3.11	130.64	124.80
14	AA	1013	CLA	CMC-C2C-C1C	3.11	129.77	125.03
14	AA	1012	CLA	OBD-CAD-CBD	-3.11	121.34	125.91
14	LA	1503	CLA	C1-C2-C3	-3.11	120.67	126.04
14	HA	1212	CLA	C3C-C4C-NC	3.11	114.06	110.57
18	BA	4004	BCR	C36-C18-C17	-3.11	118.57	122.92
14	aA	1126	CLA	C1-C2-C3	-3.11	120.67	126.04
14	lA	1503	CLA	C1-C2-C3	-3.11	120.67	126.04
14	GA	1135	CLA	CAC-C3C-C4C	3.10	128.88	124.82
18	bA	4013	BCR	C34-C9-C10	-3.10	118.58	122.92
14	HA	1238	CLA	CAC-C3C-C4C	3.10	128.87	124.82
18	AA	4003	BCR	C30-C25-C26	-3.10	118.26	122.59

Continued on next page...

PRELIMINARY VALIDATION REPORT

Continued from previous page...

Mol	Chain	Res	Type	Atoms	Z	Observed(^{o})	Ideal(^{o})
14	aA	1140	CLA	C4-C3-C5	3.10	120.62	115.29
14	AA	1125	CLA	C4C-C3C-C2C	-3.10	102.40	106.89
14	HA	1023	CLA	O2D-CGD-O1D	-3.10	117.71	123.83
14	bA	1238	CLA	CAC-C3C-C4C	3.10	128.87	124.82
14	GA	1137	CLA	CMB-C2B-C3B	3.10	130.61	124.80
14	AA	1110	CLA	C3C-C4C-NC	3.09	114.04	110.57
18	HA	4013	BCR	C34-C9-C10	-3.09	118.59	122.92
14	GA	1012	CLA	OBD-CAD-CBD	-3.09	121.37	125.91
14	AA	1137	CLA	CMB-C2B-C3B	3.09	130.60	124.80
14	kA	1401	CLA	CMA-C3A-C4A	3.09	120.08	111.77
18	bA	4010	BCR	C19-C18-C17	3.09	123.68	118.94
14	TA	1401	CLA	CMA-C3A-C4A	3.09	120.07	111.77
14	bA	1023	CLA	O2D-CGD-O1D	-3.09	117.73	123.83
14	aA	1135	CLA	CAC-C3C-C4C	3.08	128.85	124.82
14	aA	1012	CLA	OBD-CAD-CBD	-3.08	121.38	125.91
14	KA	1401	CLA	CMA-C3A-C4A	3.08	120.06	111.77
18	UA	4022	BCR	C36-C18-C17	-3.08	118.61	122.92
18	IA	4020	BCR	C24-C23-C22	-3.08	121.58	126.21
14	HA	1224	CLA	O1D-CGD-CBD	-3.08	118.10	124.48
14	BA	1224	CLA	O1D-CGD-CBD	-3.08	118.10	124.48
14	AA	1126	CLA	C1-C2-C3	-3.08	120.72	126.04
14	GA	1140	CLA	C4-C3-C5	3.08	120.59	115.29
18	GA	4003	BCR	C30-C25-C26	-3.08	118.29	122.59
14	AA	1135	CLA	CAC-C3C-C4C	3.07	128.84	124.82
14	BA	1238	CLA	CAC-C3C-C4C	3.07	128.84	124.82
14	GA	1102	CLA	O2D-CGD-O1D	-3.07	117.76	123.83
14	AA	1140	CLA	C4-C3-C5	3.07	120.58	115.29
14	BA	1023	CLA	O2D-CGD-O1D	-3.07	117.76	123.83
14	AA	1123	CLA	O2D-CGD-O1D	-3.07	117.77	123.83
18	bA	4014	BCR	C7-C8-C9	-3.07	121.60	126.21
14	bA	1224	CLA	O1D-CGD-CBD	-3.07	118.12	124.48
14	aA	1123	CLA	O2D-CGD-O1D	-3.07	117.77	123.83
18	HA	4014	BCR	C7-C8-C9	-3.07	121.61	126.21
14	aA	1102	CLA	O2D-CGD-O1D	-3.07	117.78	123.83
18	RA	4020	BCR	C24-C23-C22	-3.06	121.61	126.21
18	iA	4020	BCR	C24-C23-C22	-3.06	121.61	126.21
18	BA	4013	BCR	C34-C9-C10	-3.06	118.63	122.92
14	GA	1110	CLA	C3C-C4C-NC	3.06	114.01	110.57
14	aA	1128	CLA	O2D-CGD-O1D	-3.06	117.78	123.83
14	HA	1205	CLA	C1-C2-C3	-3.06	120.74	126.04
14	GA	1123	CLA	O2D-CGD-O1D	-3.06	117.78	123.83
18	HA	4010	BCR	C19-C18-C17	3.06	123.64	118.94

Continued on next page...

PRELIMINARY VALIDATION REPORT

Continued from previous page...

Mol	Chain	Res	Type	Atoms	Z	Observed(°)	Ideal(°)
14	aA	1123	CLA	OBD-CAD-C3D	-3.06	122.62	128.04
14	lA	1501	CLA	O2D-CGD-O1D	-3.06	117.78	123.83
18	BA	4010	BCR	C19-C18-C17	3.06	123.64	118.94
14	bA	1234	CLA	O2A-CGA-CBA	3.06	121.75	111.93
14	AA	1126	CLA	C3C-C4C-NC	3.06	114.00	110.57
14	aA	1115	CLA	CMC-C2C-C1C	3.06	129.68	125.03
14	BA	1234	CLA	O2A-CGA-CBA	3.05	121.74	111.93
18	UA	4022	BCR	C37-C22-C21	-3.05	118.64	122.92
14	GA	1126	CLA	C1-C2-C3	-3.05	120.76	126.04
18	aA	4003	BCR	C30-C25-C26	-3.05	118.32	122.59
14	LA	1501	CLA	O2D-CGD-O1D	-3.05	117.80	123.83
14	AA	1115	CLA	CMC-C2C-C1C	3.05	129.68	125.03
14	lA	1501	CLA	C1-O2A-CGA	3.05	124.88	116.54
18	BA	4014	BCR	C7-C8-C9	-3.05	121.63	126.21
14	BA	1205	CLA	C1-C2-C3	-3.05	120.77	126.04
14	AA	1123	CLA	C3C-C4C-NC	3.05	113.99	110.57
18	LA	4022	BCR	C37-C22-C21	-3.05	118.65	122.92
18	IA	4020	BCR	C34-C9-C8	3.05	122.92	118.09
14	LA	1502	CLA	O2D-CGD-O1D	-3.05	117.81	123.83
14	GA	1123	CLA	C3C-C4C-NC	3.05	113.99	110.57
14	GA	1115	CLA	CMC-C2C-C1C	3.05	129.66	125.03
14	bA	1234	CLA	C4C-C3C-C2C	-3.04	102.48	106.89
14	HA	1215	CLA	O2A-CGA-O1A	-3.04	115.78	123.56
20	BA	5002	LMG	O8-C28-C29	3.04	121.70	111.93
14	GA	1123	CLA	OBD-CAD-C3D	-3.04	122.65	128.04
14	aA	1112	CLA	CMB-C2B-C3B	3.04	130.50	124.80
14	AA	1123	CLA	OBD-CAD-C3D	-3.04	122.65	128.04
14	bA	1235	CLA	CMA-C3A-C4A	3.04	119.94	111.77
14	AA	1102	CLA	O2D-CGD-O1D	-3.04	117.83	123.83
14	aA	1118	CLA	O2D-CGD-O1D	-3.04	117.83	123.83
14	GA	1126	CLA	C3C-C4C-NC	3.04	113.98	110.57
18	lA	4022	BCR	C37-C22-C21	-3.04	118.67	122.92
14	UA	1501	CLA	C1-O2A-CGA	3.04	124.84	116.54
14	HA	1234	CLA	O2A-CGA-CBA	3.04	121.68	111.93
14	HA	1235	CLA	CMA-C3A-C4A	3.04	119.94	111.77
20	bA	5002	LMG	O8-C28-C29	3.04	121.68	111.93
14	AA	1128	CLA	O2D-CGD-O1D	-3.04	117.83	123.83
14	HA	1236	CLA	O2D-CGD-O1D	-3.03	117.84	123.83
14	bA	1239	CLA	C3C-C4C-NC	3.03	113.97	110.57
18	AA	4008	BCR	C38-C26-C25	-3.03	121.13	124.51
14	BA	1235	CLA	CMA-C3A-C4A	3.03	119.93	111.77
14	UA	1501	CLA	O2D-CGD-O1D	-3.03	117.84	123.83

Continued on next page...

PRELIMINARY VALIDATION REPORT

Continued from previous page...

Mol	Chain	Res	Type	Atoms	Z	Observed(°)	Ideal(°)
20	HA	5002	LMG	O8-C28-C29	3.03	121.67	111.93
14	GA	1128	CLA	O2D-CGD-O1D	-3.03	117.84	123.83
14	aA	1123	CLA	C3C-C4C-NC	3.03	113.97	110.57
18	iA	4020	BCR	C34-C9-C8	3.03	122.90	118.09
14	BA	1215	CLA	O2A-CGA-O1A	-3.03	115.81	123.56
18	RA	4020	BCR	C34-C9-C8	3.03	122.90	118.09
14	lA	1502	CLA	O2D-CGD-O1D	-3.03	117.85	123.83
14	bA	1205	CLA	C1-C2-C3	-3.03	120.80	126.04
14	UA	1502	CLA	O2D-CGD-O1D	-3.03	117.85	123.83
14	BA	1234	CLA	C4C-C3C-C2C	-3.03	102.50	106.89
14	GA	1129	CLA	C3C-C4C-NC	3.03	113.97	110.57
14	bA	1214	CLA	C1-C2-C3	-3.03	120.81	126.04
14	GA	1118	CLA	O2D-CGD-O1D	-3.02	117.86	123.83
14	BA	1022	CLA	C1-C2-C3	-3.02	120.81	126.04
14	bA	1236	CLA	O2D-CGD-O1D	-3.02	117.86	123.83
14	bA	1238	CLA	C3C-C4C-NC	3.02	113.96	110.57
14	HA	1238	CLA	C3C-C4C-NC	3.02	113.96	110.57
14	LA	1501	CLA	C1-O2A-CGA	3.02	124.80	116.54
14	BA	1236	CLA	O2D-CGD-O1D	-3.02	117.86	123.83
14	aA	1113	CLA	O2D-CGD-O1D	-3.02	117.87	123.83
18	GA	4008	BCR	C38-C26-C25	-3.02	121.15	124.51
14	aA	1129	CLA	C3C-C4C-NC	3.02	113.96	110.57
14	BA	1211	CLA	C1-C2-C3	-3.02	120.82	126.04
14	bA	1210	CLA	O2A-CGA-CBA	3.02	121.62	111.93
18	BA	4013	BCR	C37-C22-C21	-3.02	118.70	122.92
14	BA	1235	CLA	O2D-CGD-O1D	-3.02	117.87	123.83
14	bA	1215	CLA	O2A-CGA-O1A	-3.02	115.85	123.56
14	BA	1238	CLA	C3C-C4C-NC	3.01	113.95	110.57
14	BA	1210	CLA	O2A-CGA-CBA	3.01	121.61	111.93
14	BA	1228	CLA	CAC-C3C-C4C	3.01	128.76	124.82
14	HA	1234	CLA	C4C-C3C-C2C	-3.01	102.53	106.89
14	BA	1214	CLA	C1-C2-C3	-3.01	120.84	126.04
14	bA	1221	CLA	C3C-C4C-NC	3.01	113.95	110.57
13	aA	1011	CL0	CMB-C2B-C3B	3.01	130.44	124.80
14	bA	1022	CLA	C1-C2-C3	-3.01	120.84	126.04
14	HA	1211	CLA	C1-C2-C3	-3.01	120.84	126.04
14	bA	1228	CLA	CAC-C3C-C4C	3.01	128.75	124.82
14	HA	1214	CLA	C1-C2-C3	-3.01	120.84	126.04
14	0	1012	CLA	C1-C2-C3	-3.01	120.84	126.04
14	HA	1210	CLA	O2A-CGA-CBA	3.01	121.58	111.93
14	GA	1123	CLA	CAA-C2A-C3A	-3.01	104.55	112.78
14	lA	1503	CLA	CMB-C2B-C3B	3.01	130.44	124.80

Continued on next page...

PRELIMINARY VALIDATION REPORT

Continued from previous page...

Mol	Chain	Res	Type	Atoms	Z	Observed(°)	Ideal(°)
14	aA	1123	CLA	CAA-C2A-C3A	-3.00	104.55	112.78
14	UA	1503	CLA	CMB-C2B-C3B	3.00	130.43	124.80
14	aA	1110	CLA	C3C-C4C-NC	3.00	113.94	110.57
14	AA	1113	CLA	O2D-CGD-O1D	-3.00	117.90	123.83
14	aA	1127	CLA	C3C-C4C-NC	3.00	113.94	110.57
14	HA	1228	CLA	CAC-C3C-C4C	3.00	128.75	124.82
14	BA	1227	CLA	CMB-C2B-C3B	3.00	130.43	124.80
14	GA	1112	CLA	CMB-C2B-C3B	3.00	130.43	124.80
18	HA	4013	BCR	C37-C22-C21	-3.00	118.72	122.92
14	AA	1123	CLA	CAA-C2A-C3A	-3.00	104.57	112.78
14	aA	1126	CLA	C3C-C4C-NC	3.00	113.93	110.57
14	BA	1233	CLA	CMA-C3A-C4A	3.00	119.83	111.77
18	bA	4013	BCR	C37-C22-C21	-3.00	118.72	122.92
14	aA	1101	CLA	C3C-C4C-NC	3.00	113.93	110.57
14	bA	1229	CLA	O2D-CGD-O1D	-2.99	117.92	123.83
14	aA	1013	CLA	O2D-CGD-O1D	-2.99	117.92	123.83
14	LA	1503	CLA	CMB-C2B-C3B	2.99	130.41	124.80
14	GA	1101	CLA	C3C-C4C-NC	2.99	113.93	110.57
14	BA	1239	CLA	C3C-C4C-NC	2.99	113.93	110.57
14	AA	1118	CLA	O2D-CGD-O1D	-2.99	117.92	123.83
14	bA	1235	CLA	O2D-CGD-O1D	-2.99	117.93	123.83
14	HA	1239	CLA	C3C-C4C-NC	2.99	113.92	110.57
14	bA	1211	CLA	C1-C2-C3	-2.99	120.88	126.04
13	AA	1011	CL0	CMB-C2B-C3B	2.99	130.40	124.80
14	AA	1112	CLA	CMB-C2B-C3B	2.99	130.40	124.80
13	GA	1011	CL0	CMB-C2B-C3B	2.98	130.40	124.80
14	HA	1233	CLA	CMA-C3A-C4A	2.98	119.79	111.77
14	HA	1210	CLA	CAA-C2A-C3A	-2.98	104.62	112.78
18	GA	4002	BCR	C37-C22-C21	-2.98	118.75	122.92
14	BA	1023	CLA	CHB-C4A-NA	2.98	128.63	124.51
14	AA	1122	CLA	CMB-C2B-C3B	2.98	130.39	124.80
14	HA	1023	CLA	CHB-C4A-NA	2.98	128.63	124.51
14	bA	1227	CLA	CMB-C2B-C3B	2.98	130.38	124.80
14	AA	1129	CLA	C3C-C4C-NC	2.98	113.91	110.57
14	GA	1113	CLA	O2D-CGD-O1D	-2.97	117.96	123.83
14	bA	1233	CLA	CMA-C3A-C4A	2.97	119.76	111.77
14	BA	1229	CLA	O2D-CGD-O1D	-2.97	117.96	123.83
14	aA	1126	CLA	CAA-C2A-C3A	-2.97	104.64	112.78
14	aA	1111	CLA	O2D-CGD-O1D	-2.97	117.96	123.83
14	AA	1101	CLA	C3C-C4C-NC	2.97	113.90	110.57
14	bA	1210	CLA	CAA-C2A-C3A	-2.97	104.64	112.78
14	bA	1218	CLA	CMA-C3A-C4A	2.97	119.75	111.77

Continued on next page...

PRELIMINARY VALIDATION REPORT

Continued from previous page...

Mol	Chain	Res	Type	Atoms	Z	Observed(°)	Ideal(°)
18	HA	4010	BCR	C3-C4-C5	-2.97	108.83	113.99
14	AA	1127	CLA	C3C-C4C-NC	2.97	113.90	110.57
18	aA	4002	BCR	C37-C22-C21	-2.97	118.77	122.92
18	bA	4013	BCR	C38-C26-C25	-2.97	121.20	124.51
14	HA	1235	CLA	O2D-CGD-O1D	-2.97	117.97	123.83
14	GA	1126	CLA	CAA-C2A-C3A	-2.97	104.66	112.78
14	AA	1013	CLA	O2D-CGD-O1D	-2.96	117.97	123.83
14	bA	1023	CLA	CHB-C4A-NA	2.96	128.61	124.51
14	HA	1227	CLA	CMB-C2B-C3B	2.96	130.36	124.80
14	aA	1137	CLA	C3C-C4C-NC	2.96	113.89	110.57
14	bA	1201	CLA	O2D-CGD-O1D	-2.96	117.98	123.83
18	AA	4002	BCR	C37-C22-C21	-2.96	118.77	122.92
14	BA	1023	CLA	CMB-C2B-C3B	2.96	130.36	124.80
14	BA	1218	CLA	CMA-C3A-C4A	2.96	119.73	111.77
18	bA	4006	BCR	C36-C18-C17	-2.96	118.78	122.92
14	BA	1203	CLA	C1-C2-C3	-2.96	120.92	126.04
14	aA	1130	CLA	O2D-CGD-O1D	-2.96	117.98	123.83
14	BA	1210	CLA	CAA-C2A-C3A	-2.96	104.67	112.78
14	BA	1202	CLA	C4-C3-C5	2.96	120.38	115.29
14	UA	1502	CLA	CAA-C2A-C3A	-2.96	104.68	112.78
14	HA	1229	CLA	O2D-CGD-O1D	-2.96	117.99	123.83
14	GA	1122	CLA	CMB-C2B-C3B	2.96	130.34	124.80
14	HA	1023	CLA	CMB-C2B-C3B	2.95	130.34	124.80
14	bA	1233	CLA	CMC-C2C-C1C	2.95	129.53	125.03
14	bA	1023	CLA	CMB-C2B-C3B	2.95	130.34	124.80
18	RA	4018	BCR	C34-C9-C8	2.95	122.77	118.09
14	AA	1126	CLA	CAA-C2A-C3A	-2.95	104.69	112.78
14	HA	1221	CLA	C3C-C4C-NC	2.95	113.88	110.57
14	HA	1201	CLA	O2D-CGD-O1D	-2.95	118.00	123.83
14	BA	1201	CLA	O2D-CGD-O1D	-2.95	118.00	123.83
14	GA	1127	CLA	C3C-C4C-NC	2.95	113.88	110.57
14	GA	1111	CLA	O2D-CGD-O1D	-2.95	118.00	123.83
18	bA	4010	BCR	C3-C4-C5	-2.95	108.86	113.99
14	GA	1013	CLA	O2D-CGD-O1D	-2.95	118.00	123.83
14	BA	1233	CLA	CMC-C2C-C1C	2.95	129.52	125.03
14	HA	1203	CLA	C1-C2-C3	-2.95	120.95	126.04
18	GA	4008	BCR	C28-C27-C26	-2.95	108.86	113.99
14	AA	1139	CLA	CMC-C2C-C1C	2.95	129.51	125.03
18	BA	4006	BCR	C36-C18-C17	-2.95	118.80	122.92
14	HA	1213	CLA	CMC-C2C-C1C	2.95	129.51	125.03
14	aA	1122	CLA	CMB-C2B-C3B	2.94	130.32	124.80
14	LA	1502	CLA	CAA-C2A-C3A	-2.94	104.72	112.78

Continued on next page...

PRELIMINARY VALIDATION REPORT

Continued from previous page...

Mol	Chain	Res	Type	Atoms	Z	Observed(°)	Ideal(°)
18	BA	4010	BCR	C3-C4-C5	-2.94	108.87	113.99
14	AA	1130	CLA	O2D-CGD-O1D	-2.94	118.02	123.83
14	HA	1217	CLA	C3C-C4C-NC	2.94	113.87	110.57
14	AA	1125	CLA	CAA-C2A-C3A	-2.94	104.73	112.78
14	HA	1207	CLA	CAA-C2A-C3A	-2.94	104.73	112.78
14	AA	1111	CLA	O2D-CGD-O1D	-2.94	118.03	123.83
14	GA	1130	CLA	O2D-CGD-O1D	-2.94	118.03	123.83
14	AA	1136	CLA	C3C-C4C-NC	2.94	113.87	110.57
14	GA	1125	CLA	CAA-C2A-C3A	-2.94	104.73	112.78
18	aA	4008	BCR	C39-C30-C25	-2.94	105.55	110.30
18	aA	4008	BCR	C28-C27-C26	-2.94	108.88	113.99
14	GA	1139	CLA	CMC-C2C-C1C	2.94	129.50	125.03
14	HA	1218	CLA	CMA-C3A-C4A	2.94	119.67	111.77
18	HA	4006	BCR	C36-C18-C17	-2.94	118.81	122.92
18	HA	4013	BCR	C38-C26-C25	-2.94	121.24	124.51
14	aA	1125	CLA	CAA-C2A-C3A	-2.93	104.74	112.78
14	BA	1221	CLA	C3C-C4C-NC	2.93	113.86	110.57
14	HA	1202	CLA	C4-C3-C5	2.93	120.34	115.29
14	BA	1222	CLA	O2D-CGD-O1D	-2.93	118.04	123.83
18	BA	4013	BCR	C38-C26-C25	-2.93	121.24	124.51
14	AA	1131	CLA	O2D-CGD-O1D	-2.93	118.04	123.83
14	BA	1213	CLA	CMC-C2C-C1C	2.93	129.49	125.03
14	lA	1502	CLA	CAA-C2A-C3A	-2.93	104.75	112.78
14	AA	1137	CLA	C3C-C4C-NC	2.93	113.86	110.57
14	aA	1131	CLA	O2D-CGD-O1D	-2.93	118.04	123.83
14	bA	1213	CLA	CMC-C2C-C1C	2.93	129.49	125.03
18	lA	4019	BCR	C19-C18-C17	2.93	123.44	118.94
14	aA	1139	CLA	CMC-C2C-C1C	2.93	129.49	125.03
14	HA	1234	CLA	CAC-C3C-C4C	2.93	128.65	124.82
18	iA	4018	BCR	C34-C9-C8	2.93	122.74	118.09
14	bA	1202	CLA	C4-C3-C5	2.93	120.33	115.29
14	BA	1207	CLA	CAA-C2A-C3A	-2.93	104.76	112.78
18	AA	4008	BCR	C28-C27-C26	-2.93	108.89	113.99
14	HA	1233	CLA	CMC-C2C-C1C	2.93	129.49	125.03
18	HA	4005	BCR	C40-C30-C25	-2.93	105.57	110.30
18	GA	4008	BCR	C39-C30-C25	-2.93	105.57	110.30
14	HA	1222	CLA	O2D-CGD-O1D	-2.93	118.05	123.83
14	bA	1222	CLA	O2D-CGD-O1D	-2.93	118.05	123.83
14	aA	1135	CLA	CMA-C3A-C4A	2.93	119.64	111.77
18	IA	4018	BCR	C34-C9-C8	2.92	122.73	118.09
14	bA	1239	CLA	CAA-CBA-CGA	-2.92	104.64	113.26
14	bA	1207	CLA	CAA-C2A-C3A	-2.92	104.77	112.78

Continued on next page...

PRELIMINARY VALIDATION REPORT

Continued from previous page...

Mol	Chain	Res	Type	Atoms	Z	Observed(°)	Ideal(°)
14	bA	1203	CLA	C1-C2-C3	-2.92	120.98	126.04
18	AA	4008	BCR	C39-C30-C25	-2.92	105.58	110.30
14	HA	1239	CLA	CAA-CBA-CGA	-2.92	104.65	113.26
14	aA	1136	CLA	C3C-C4C-NC	2.92	113.85	110.57
14	HA	1205	CLA	OBD-CAD-C3D	-2.92	122.86	128.04
18	aA	4007	BCR	C33-C5-C4	2.92	119.03	113.57
14	bA	1234	CLA	CAC-C3C-C4C	2.92	128.63	124.82
14	BA	1239	CLA	CAA-CBA-CGA	-2.92	104.66	113.26
14	LA	1501	CLA	OBD-CAD-CBD	-2.92	121.63	125.91
18	bA	4005	BCR	C40-C30-C25	-2.91	105.59	110.30
14	GA	1107	CLA	C3C-C4C-NC	2.91	113.84	110.57
14	GA	1136	CLA	C3C-C4C-NC	2.91	113.84	110.57
18	HA	4004	BCR	C38-C26-C25	-2.91	121.27	124.51
14	UA	1501	CLA	OBD-CAD-CBD	-2.91	121.64	125.91
14	BA	1217	CLA	C3C-C4C-NC	2.91	113.83	110.57
14	bA	1023	CLA	C3C-C4C-NC	2.91	113.83	110.57
18	bA	4009	BCR	C37-C22-C21	-2.91	118.85	122.92
18	bA	4004	BCR	C38-C26-C25	-2.91	121.27	124.51
18	LA	4019	BCR	C19-C18-C17	2.91	123.40	118.94
14	BA	1222	CLA	C4-C3-C5	2.91	120.29	115.29
14	GA	1131	CLA	O2D-CGD-O1D	-2.90	118.09	123.83
14	HA	1222	CLA	C4-C3-C5	2.90	120.29	115.29
18	AA	4002	BCR	C38-C26-C27	2.90	119.01	113.57
14	BA	1023	CLA	C3C-C4C-NC	2.90	113.83	110.57
14	bA	1205	CLA	OBD-CAD-C3D	-2.90	122.90	128.04
14	GA	1135	CLA	CMA-C3A-C4A	2.90	119.57	111.77
14	aA	1106	CLA	C3C-C4C-NC	2.90	113.83	110.57
14	BA	1205	CLA	OBD-CAD-C3D	-2.90	122.90	128.04
14	GA	1137	CLA	C3C-C4C-NC	2.90	113.82	110.57
14	bA	1217	CLA	C3C-C4C-NC	2.90	113.82	110.57
18	AA	4007	BCR	C33-C5-C4	2.90	119.00	113.57
14	BA	1226	CLA	C1-C2-C3	-2.90	121.03	126.04
14	AA	1135	CLA	CMA-C3A-C4A	2.90	119.56	111.77
14	bA	1023	CLA	C1D-CHD-C4C	2.90	126.44	122.48
14	BA	1232	CLA	CMA-C3A-C4A	2.90	119.55	111.77
14	BA	1234	CLA	CAC-C3C-C4C	2.89	128.60	124.82
14	BA	1206	CLA	C3C-C4C-NC	2.89	113.82	110.57
14	HA	1023	CLA	C1D-CHD-C4C	2.89	126.44	122.48
14	GA	1106	CLA	C3C-C4C-NC	2.89	113.82	110.57
14	HA	1232	CLA	CMA-C3A-C4A	2.89	119.55	111.77
14	HA	1206	CLA	C3C-C4C-NC	2.89	113.81	110.57
14	bA	1226	CLA	C1-C2-C3	-2.89	121.04	126.04

Continued on next page...

PRELIMINARY VALIDATION REPORT

Continued from previous page...

Mol	Chain	Res	Type	Atoms	Z	Observed(°)	Ideal(°)
18	AA	4011	BCR	C38-C26-C25	-2.89	121.29	124.51
14	lA	1501	CLA	OBD-CAD-CBD	-2.89	121.67	125.91
14	aA	1103	CLA	C4-C3-C5	2.89	120.26	115.29
14	HA	1203	CLA	C4-C3-C5	2.89	120.26	115.29
18	GA	4002	BCR	C38-C26-C27	2.89	118.97	113.57
18	GA	4007	BCR	C33-C5-C4	2.89	118.97	113.57
14	HA	1226	CLA	C1-C2-C3	-2.89	121.05	126.04
18	MA	4021	BCR	C37-C22-C21	-2.88	118.88	122.92
14	AA	1122	CLA	C4-C3-C5	2.88	120.26	115.29
18	BA	4017	BCR	C34-C9-C8	2.88	122.67	118.09
18	aA	4002	BCR	C38-C26-C27	2.88	118.97	113.57
14	bA	1222	CLA	C4-C3-C5	2.88	120.25	115.29
14	BA	1203	CLA	C4-C3-C5	2.88	120.25	115.29
18	HA	4009	BCR	C37-C22-C21	-2.88	118.89	122.92
14	aA	1107	CLA	C3C-C4C-NC	2.88	113.80	110.57
14	bA	1201	CLA	CMC-C2C-C1C	2.88	129.41	125.03
18	BA	4005	BCR	C40-C30-C25	-2.88	105.64	110.30
14	aA	1108	CLA	CAA-C2A-C3A	-2.88	104.89	112.78
14	AA	1107	CLA	C3C-C4C-NC	2.88	113.80	110.57
14	AA	1139	CLA	CAC-C3C-C4C	2.88	128.58	124.82
14	bA	1206	CLA	C3C-C4C-NC	2.88	113.80	110.57
14	HA	1234	CLA	O2D-CGD-O1D	-2.88	118.14	123.83
14	GA	1108	CLA	CAA-C2A-C3A	-2.88	104.89	112.78
14	AA	1108	CLA	CAA-C2A-C3A	-2.88	104.90	112.78
18	UA	4019	BCR	C19-C18-C17	2.88	123.36	118.94
18	bA	4010	BCR	C34-C9-C10	-2.88	118.89	122.92
14	BA	1023	CLA	C1D-CHD-C4C	2.88	126.42	122.48
18	BA	4009	BCR	C37-C22-C21	-2.88	118.89	122.92
14	bA	1232	CLA	CMA-C3A-C4A	2.88	119.50	111.77
18	bA	4017	BCR	C34-C9-C8	2.87	122.65	118.09
14	HA	1201	CLA	CMC-C2C-C1C	2.87	129.40	125.03
14	AA	1106	CLA	C3C-C4C-NC	2.87	113.79	110.57
18	BA	4004	BCR	C38-C26-C25	-2.87	121.31	124.51
14	GA	1126	CLA	C4-C3-C5	2.87	120.23	115.29
14	AA	1131	CLA	CMC-C2C-C1C	2.87	129.40	125.03
14	bA	1203	CLA	C4-C3-C5	2.87	120.23	115.29
18	bA	4014	BCR	C33-C5-C4	2.87	118.94	113.57
14	HA	1207	CLA	CMC-C2C-C1C	2.87	129.40	125.03
14	bA	1215	CLA	CBA-CAA-C2A	2.87	122.39	113.85
14	AA	1013	CLA	CHB-C4A-NA	2.87	128.48	124.51
18	RA	4020	BCR	C8-C9-C10	2.87	123.34	118.94
14	HA	1205	CLA	C3C-C4C-NC	2.87	113.79	110.57

Continued on next page...

PRELIMINARY VALIDATION REPORT

Continued from previous page...

Mol	Chain	Res	Type	Atoms	Z	Observed(^{o})	Ideal(^{o})
14	HA	1228	CLA	OBD-CAD-C3D	-2.87	122.96	128.04
14	aA	1013	CLA	CHB-C4A-NA	2.87	128.47	124.51
18	HA	4017	BCR	C34-C9-C8	2.87	122.64	118.09
14	aA	1139	CLA	CAC-C3C-C4C	2.87	128.56	124.82
14	HA	1224	CLA	CMA-C3A-C4A	2.86	119.47	111.77
14	bA	1234	CLA	O2D-CGD-O1D	-2.86	118.17	123.83
14	BA	1201	CLA	CMC-C2C-C1C	2.86	129.39	125.03
13	aA	1011	CL0	C1-O2A-CGA	2.86	124.37	116.54
14	BA	1234	CLA	O2D-CGD-O1D	-2.86	118.17	123.83
18	mA	4021	BCR	C37-C22-C21	-2.86	118.91	122.92
14	BA	1221	CLA	CAA-C2A-C3A	-2.86	104.94	112.78
14	aA	1126	CLA	C4-C3-C5	2.86	120.22	115.29
14	GA	1131	CLA	CMC-C2C-C1C	2.86	129.38	125.03
14	HA	1219	CLA	C3C-C4C-NC	2.86	113.78	110.57
14	HA	1023	CLA	C3C-C4C-NC	2.86	113.78	110.57
14	GA	1109	CLA	CMA-C3A-C4A	2.86	119.46	111.77
18	HA	4010	BCR	C34-C9-C10	-2.86	118.92	122.92
14	BA	1207	CLA	CMC-C2C-C1C	2.86	129.38	125.03
14	HA	1215	CLA	CBA-CAA-C2A	2.86	122.35	113.85
14	BA	1228	CLA	OBD-CAD-C3D	-2.86	122.98	128.04
18	VA	4021	BCR	C37-C22-C21	-2.86	118.92	122.92
14	bA	1224	CLA	CMA-C3A-C4A	2.86	119.45	111.77
18	BA	4014	BCR	C33-C5-C4	2.86	118.92	113.57
14	AA	1113	CLA	CAA-C2A-C3A	-2.86	104.96	112.78
18	aA	4008	BCR	C34-C9-C8	2.86	122.62	118.09
14	GA	1139	CLA	CAC-C3C-C4C	2.85	128.55	124.82
14	aA	1111	CLA	C4-C3-C5	2.85	120.20	115.29
14	aA	1109	CLA	CMA-C3A-C4A	2.85	119.44	111.77
14	bA	1221	CLA	CAA-C2A-C3A	-2.85	104.96	112.78
14	BA	1021	CLA	C11-C12-C13	-2.85	106.87	115.77
18	aA	4011	BCR	C38-C26-C25	-2.85	121.33	124.51
14	AA	1126	CLA	C4-C3-C5	2.85	120.20	115.29
18	BA	4012	BCR	C37-C22-C21	-2.85	118.93	122.92
14	aA	1122	CLA	C4-C3-C5	2.85	120.20	115.29
14	BA	1219	CLA	C3C-C4C-NC	2.85	113.77	110.57
14	aA	1113	CLA	CAA-C2A-C3A	-2.85	104.97	112.78
14	bA	1021	CLA	C11-C12-C13	-2.85	106.88	115.77
14	HA	1021	CLA	C11-C12-C13	-2.85	106.88	115.77
14	BA	1215	CLA	CBA-CAA-C2A	2.85	122.33	113.85
14	GA	1122	CLA	C4-C3-C5	2.85	120.20	115.29
18	BA	4010	BCR	C34-C9-C10	-2.85	118.93	122.92
14	HA	1231	CLA	C1-C2-C3	-2.85	121.11	126.04

Continued on next page...

PRELIMINARY VALIDATION REPORT

Continued from previous page...

Mol	Chain	Res	Type	Atoms	Z	Observed(°)	Ideal(°)
14	bA	1207	CLA	CMC-C2C-C1C	2.85	129.37	125.03
14	bA	1231	CLA	C1-C2-C3	-2.85	121.11	126.04
14	aA	1131	CLA	CMC-C2C-C1C	2.85	129.37	125.03
14	aA	1122	CLA	C3C-C4C-NC	2.85	113.77	110.57
14	BA	1224	CLA	CMA-C3A-C4A	2.85	119.43	111.77
14	AA	1109	CLA	CMA-C3A-C4A	2.85	119.43	111.77
18	AA	4008	BCR	C34-C9-C8	2.85	122.61	118.09
14	BA	1212	CLA	CAA-C2A-C3A	-2.85	104.98	112.78
18	BA	4004	BCR	C2-C1-C6	2.85	114.89	110.48
14	HA	1221	CLA	CAA-C2A-C3A	-2.85	104.98	112.78
14	BA	1231	CLA	C1-C2-C3	-2.85	121.12	126.04
14	bA	1208	CLA	C1-C2-C3	-2.85	121.12	126.04
14	HA	1212	CLA	CAA-C2A-C3A	-2.85	104.98	112.78
13	GA	1011	CL0	C1-O2A-CGA	2.85	124.32	116.54
14	bA	1023	CLA	CHD-C4C-NC	-2.85	119.72	124.20
18	bA	4017	BCR	C38-C26-C25	-2.84	121.34	124.51
14	bA	1022	CLA	CMC-C2C-C1C	2.84	129.36	125.03
14	GA	1122	CLA	C3C-C4C-NC	2.84	113.76	110.57
14	bA	1228	CLA	OBD-CAD-C3D	-2.84	123.00	128.04
14	GA	1013	CLA	CHB-C4A-NA	2.84	128.44	124.51
14	bA	1205	CLA	C3C-C4C-NC	2.84	113.76	110.57
18	HA	4017	BCR	C38-C26-C25	-2.84	121.34	124.51
14	GA	1113	CLA	CAA-C2A-C3A	-2.84	105.00	112.78
18	bA	4014	BCR	C28-C27-C26	-2.84	109.05	113.99
14	BA	1022	CLA	CMC-C2C-C1C	2.84	129.35	125.03
13	AA	1011	CL0	C1-O2A-CGA	2.84	124.31	116.54
18	BA	4009	BCR	C7-C8-C9	-2.84	121.94	126.21
14	BA	1204	CLA	O2D-CGD-O1D	-2.84	118.22	123.83
14	AA	1122	CLA	C3C-C4C-NC	2.84	113.76	110.57
14	HA	1201	CLA	C3C-C4C-NC	2.84	113.76	110.57
14	BA	1205	CLA	C3C-C4C-NC	2.84	113.76	110.57
18	bA	4006	BCR	C34-C9-C10	-2.84	118.95	122.92
14	IA	1502	CLA	C1-C2-C3	-2.84	121.13	126.04
18	HA	4014	BCR	C33-C5-C4	2.84	118.88	113.57
18	iA	4020	BCR	C8-C9-C10	2.84	123.30	118.94
18	GA	4008	BCR	C34-C9-C8	2.84	122.59	118.09
14	bA	1204	CLA	O2D-CGD-O1D	-2.84	118.23	123.83
14	bA	1212	CLA	CAA-C2A-C3A	-2.84	105.01	112.78
14	bA	1217	CLA	O2D-CGD-O1D	-2.83	118.23	123.83
14	GA	1103	CLA	C4-C3-C5	2.83	120.17	115.29
14	bA	1201	CLA	C3C-C4C-NC	2.83	113.75	110.57
18	GA	4011	BCR	C38-C26-C25	-2.83	121.36	124.51

Continued on next page...

PRELIMINARY VALIDATION REPORT

Continued from previous page...

Mol	Chain	Res	Type	Atoms	Z	Observed(°)	Ideal(°)
14	HA	1217	CLA	O2D-CGD-O1D	-2.83	118.24	123.83
14	GA	1136	CLA	CMB-C2B-C3B	2.83	130.11	124.80
14	UA	1502	CLA	C1-C2-C3	-2.83	121.15	126.04
14	AA	1111	CLA	C4-C3-C5	2.83	120.16	115.29
18	HA	4006	BCR	C34-C9-C10	-2.83	118.96	122.92
14	GA	1111	CLA	C4-C3-C5	2.83	120.16	115.29
14	HA	1023	CLA	CHD-C4C-NC	-2.83	119.75	124.20
14	BA	1023	CLA	CHD-C4C-NC	-2.83	119.75	124.20
18	BA	4006	BCR	C34-C9-C10	-2.83	118.97	122.92
14	HA	1208	CLA	C1-C2-C3	-2.83	121.16	126.04
14	LA	1502	CLA	C1-C2-C3	-2.83	121.16	126.04
14	GA	1115	CLA	CBA-CAA-C2A	2.83	122.26	113.85
14	HA	1204	CLA	O2D-CGD-O1D	-2.82	118.25	123.83
14	0	1012	CLA	CMC-C2C-C1C	2.82	129.33	125.03
18	bA	4012	BCR	C37-C22-C21	-2.82	118.97	122.92
14	BA	1201	CLA	C3C-C4C-NC	2.82	113.74	110.57
18	HA	4014	BCR	C28-C27-C26	-2.82	109.08	113.99
14	AA	1103	CLA	C4-C3-C5	2.82	120.15	115.29
18	HA	4004	BCR	C2-C1-C6	2.82	114.85	110.48
18	bA	4009	BCR	C7-C8-C9	-2.82	121.97	126.21
14	aA	1115	CLA	CBA-CAA-C2A	2.82	122.25	113.85
20	AA	5005	LMG	O8-C28-C29	2.82	120.99	111.93
20	aA	5005	LMG	O8-C28-C29	2.82	120.98	111.93
18	bA	4004	BCR	C2-C1-C6	2.82	114.84	110.48
14	aA	1136	CLA	CMB-C2B-C3B	2.82	130.08	124.80
14	HA	1223	CLA	OBD-CAD-C3D	-2.82	123.05	128.04
14	AA	1115	CLA	CBA-CAA-C2A	2.82	122.23	113.85
14	HA	1233	CLA	CAA-C2A-C3A	-2.82	105.07	112.78
20	GA	5005	LMG	O8-C28-C29	2.82	120.97	111.93
18	BA	4016	BCR	C7-C8-C9	-2.81	121.98	126.21
14	BA	1208	CLA	C1-C2-C3	-2.81	121.17	126.04
14	aA	1132	CLA	C3C-C4C-NC	2.81	113.73	110.57
14	AA	1136	CLA	CMB-C2B-C3B	2.81	130.07	124.80
14	aA	1109	CLA	CMB-C2B-C3B	2.81	130.07	124.80
14	GA	1127	CLA	CAA-C2A-C3A	-2.81	105.08	112.78
14	aA	1127	CLA	CAA-C2A-C3A	-2.81	105.08	112.78
14	BA	1217	CLA	O2D-CGD-O1D	-2.81	118.29	123.83
14	BA	1233	CLA	CAA-C2A-C3A	-2.81	105.09	112.78
18	IA	4020	BCR	C8-C9-C10	2.81	123.25	118.94
14	bA	1233	CLA	CAA-C2A-C3A	-2.81	105.09	112.78
18	HA	4012	BCR	C37-C22-C21	-2.81	118.99	122.92
18	BA	4014	BCR	C28-C27-C26	-2.81	109.11	113.99

Continued on next page...

PRELIMINARY VALIDATION REPORT

Continued from previous page...

Mol	Chain	Res	Type	Atoms	Z	Observed(^{o})	Ideal(^{o})
14	bA	1221	CLA	CMC-C2C-C1C	2.80	129.30	125.03
14	HA	1221	CLA	CMC-C2C-C1C	2.80	129.30	125.03
14	AA	1129	CLA	CAA-C2A-C3A	-2.80	105.10	112.78
14	AA	1133	CLA	O2D-CGD-O1D	-2.80	118.30	123.83
14	AA	1127	CLA	CAA-C2A-C3A	-2.80	105.10	112.78
14	aA	1133	CLA	O2D-CGD-O1D	-2.80	118.30	123.83
14	BA	1223	CLA	OBD-CAD-C3D	-2.80	123.08	128.04
14	bA	1219	CLA	C3C-C4C-NC	2.80	113.71	110.57
14	GA	1101	CLA	CMC-C2C-C1C	2.80	129.29	125.03
14	aA	1107	CLA	CMC-C2C-C1C	2.80	129.29	125.03
18	AA	4007	BCR	C34-C9-C8	2.80	122.53	118.09
14	AA	1132	CLA	C3C-C4C-NC	2.80	113.71	110.57
13	AA	1011	CL0	CMA-C3A-C2A	-2.80	102.51	113.78
14	HA	1224	CLA	C4-C3-C5	2.80	120.10	115.29
14	aA	1101	CLA	CMC-C2C-C1C	2.80	129.28	125.03
14	GA	1111	CLA	C1-O2A-CGA	2.80	124.18	116.54
14	XA	1701	CLA	CMA-C3A-C4A	2.80	119.29	111.77
14	GA	1133	CLA	O2D-CGD-O1D	-2.80	118.31	123.83
14	AA	1109	CLA	O2D-CGD-O1D	-2.80	118.31	123.83
14	AA	1107	CLA	CMC-C2C-C1C	2.79	129.28	125.03
14	WA	1701	CLA	CMA-C3A-C4A	2.79	119.28	111.77
14	aA	1126	CLA	O1D-CGD-CBD	-2.79	118.69	124.48
14	bA	1223	CLA	OBD-CAD-C3D	-2.79	123.09	128.04
14	GA	1129	CLA	CAA-C2A-C3A	-2.79	105.13	112.78
14	xA	1701	CLA	CMA-C3A-C4A	2.79	119.28	111.77
18	aA	4007	BCR	C34-C9-C8	2.79	122.52	118.09
18	HA	4016	BCR	C7-C8-C9	-2.79	122.02	126.21
14	aA	1129	CLA	CAA-C2A-C3A	-2.79	105.14	112.78
14	AA	1111	CLA	C1-O2A-CGA	2.79	124.17	116.54
14	lA	1503	CLA	O2D-CGD-O1D	-2.79	118.32	123.83
14	AA	1101	CLA	CMC-C2C-C1C	2.79	129.28	125.03
20	IA	5006	LMG	O8-C28-C29	2.79	120.89	111.93
14	GA	1132	CLA	C3C-C4C-NC	2.79	113.70	110.57
18	GA	4003	BCR	C1-C6-C5	-2.78	118.70	122.59
14	aA	1107	CLA	CAA-C2A-C3A	-2.78	105.15	112.78
18	bA	4010	BCR	C33-C5-C6	-2.78	121.41	124.51
13	GA	1011	CL0	CMA-C3A-C2A	-2.78	102.56	113.78
14	BA	1203	CLA	C3C-C4C-NC	2.78	113.69	110.57
14	BA	1221	CLA	CMC-C2C-C1C	2.78	129.26	125.03
14	GA	1126	CLA	O1D-CGD-CBD	-2.78	118.71	124.48
14	aA	1137	CLA	O1D-CGD-CBD	-2.78	118.71	124.48
14	BA	1224	CLA	C4-C3-C5	2.78	120.08	115.29

Continued on next page...

PRELIMINARY VALIDATION REPORT

Continued from previous page...

Mol	Chain	Res	Type	Atoms	Z	Observed(°)	Ideal(°)
20	lA	5007	LMG	O8-C28-C29	2.78	120.86	111.93
14	AA	1136	CLA	CMC-C2C-C1C	2.78	129.26	125.03
20	iA	5006	LMG	O8-C28-C29	2.78	120.86	111.93
14	AA	1109	CLA	CMB-C2B-C3B	2.78	130.02	124.80
20	UA	5007	LMG	O8-C28-C29	2.78	120.86	111.93
14	GA	1107	CLA	CAA-C2A-C3A	-2.78	105.16	112.78
14	AA	1107	CLA	CAA-C2A-C3A	-2.78	105.16	112.78
14	BA	1211	CLA	CMC-C2C-C1C	2.78	129.26	125.03
13	aA	1011	CL0	CMA-C3A-C2A	-2.78	102.57	113.78
18	bA	4016	BCR	C7-C8-C9	-2.78	122.03	126.21
14	GA	1109	CLA	CMB-C2B-C3B	2.78	130.01	124.80
14	HA	1211	CLA	O2D-CGD-O1D	-2.78	118.34	123.83
18	aA	4007	BCR	C38-C26-C27	2.78	118.78	113.57
18	HA	4005	BCR	C30-C25-C26	-2.78	118.70	122.59
14	AA	1118	CLA	CBC-CAC-C3C	-2.78	104.71	112.43
14	aA	1108	CLA	CMA-C3A-C4A	2.78	119.24	111.77
14	AA	1130	CLA	C4-C3-C5	2.78	120.07	115.29
14	AA	1124	CLA	C1-C2-C3	-2.78	121.24	126.04
20	RA	5006	LMG	O8-C28-C29	2.78	120.85	111.93
14	HA	1203	CLA	C3C-C4C-NC	2.78	113.69	110.57
14	bA	1211	CLA	CMC-C2C-C1C	2.78	129.26	125.03
14	aA	1130	CLA	C4-C3-C5	2.78	120.07	115.29
18	AA	4003	BCR	C1-C6-C5	-2.78	118.71	122.59
18	bA	4014	BCR	C33-C5-C6	-2.78	121.42	124.51
18	bA	4014	BCR	C34-C9-C8	2.78	122.49	118.09
18	iA	4018	BCR	C38-C26-C25	-2.77	121.42	124.51
14	GA	1108	CLA	CMA-C3A-C4A	2.77	119.23	111.77
14	GA	1107	CLA	CMC-C2C-C1C	2.77	129.25	125.03
14	HA	1211	CLA	CMC-C2C-C1C	2.77	129.25	125.03
14	aA	1122	CLA	CMA-C3A-C4A	2.77	119.23	111.77
18	HA	4009	BCR	C7-C8-C9	-2.77	122.05	126.21
14	AA	1122	CLA	CMA-C3A-C4A	2.77	119.22	111.77
14	AA	1126	CLA	O1D-CGD-CBD	-2.77	118.73	124.48
18	BA	4005	BCR	C30-C25-C26	-2.77	118.72	122.59
14	bA	1211	CLA	O2D-CGD-O1D	-2.77	118.36	123.83
14	aA	1111	CLA	C1-O2A-CGA	2.77	124.11	116.54
14	BA	1214	CLA	O2D-CGD-O1D	-2.77	118.36	123.83
18	GA	4007	BCR	C34-C9-C8	2.77	122.48	118.09
14	GA	1122	CLA	CMA-C3A-C4A	2.77	119.22	111.77
18	bA	4012	BCR	C30-C25-C24	2.77	123.65	115.78
18	BA	4017	BCR	C38-C26-C25	-2.77	121.43	124.51
14	GA	1124	CLA	C1-C2-C3	-2.77	121.26	126.04

Continued on next page...

PRELIMINARY VALIDATION REPORT

Continued from previous page...

Mol	Chain	Res	Type	Atoms	Z	Observed(°)	Ideal(°)
18	BA	4014	BCR	C33-C5-C6	-2.76	121.43	124.51
14	AA	1103	CLA	CAC-C3C-C4C	2.76	128.43	124.82
20	LA	5007	LMG	O8-C28-C29	2.76	120.81	111.93
14	HA	1214	CLA	O2D-CGD-O1D	-2.76	118.37	123.83
14	AA	1139	CLA	C3C-C4C-NC	2.76	113.67	110.57
14	AA	1131	CLA	C1-C2-C3	-2.76	121.26	126.04
14	LA	1503	CLA	O2D-CGD-O1D	-2.76	118.38	123.83
14	BA	1226	CLA	CAA-C2A-C3A	-2.76	105.21	112.78
14	AA	1108	CLA	CMA-C3A-C4A	2.76	119.20	111.77
14	bA	1203	CLA	CMB-C2B-C3B	2.76	129.98	124.80
18	BA	4010	BCR	C33-C5-C6	-2.76	121.43	124.51
18	HA	4014	BCR	C35-C13-C12	2.76	122.47	118.09
14	GA	1109	CLA	O2D-CGD-O1D	-2.76	118.38	123.83
14	BA	1211	CLA	O2D-CGD-O1D	-2.76	118.38	123.83
14	GA	1137	CLA	O1D-CGD-CBD	-2.76	118.76	124.48
18	BA	4012	BCR	C30-C25-C24	2.76	123.63	115.78
14	UA	1503	CLA	O2D-CGD-O1D	-2.76	118.38	123.83
14	HA	1203	CLA	CMB-C2B-C3B	2.76	129.97	124.80
14	AA	1012	CLA	C3C-C4C-NC	2.76	113.67	110.57
14	GA	1118	CLA	CBC-CAC-C3C	-2.76	104.77	112.43
14	AA	1140	CLA	CMC-C2C-C1C	2.76	129.23	125.03
14	GA	1140	CLA	CMC-C2C-C1C	2.76	129.23	125.03
18	HA	4014	BCR	C34-C9-C8	2.76	122.47	118.09
14	bA	1224	CLA	C4-C3-C5	2.76	120.04	115.29
14	bA	1214	CLA	O2D-CGD-O1D	-2.76	118.39	123.83
14	BA	1224	CLA	C6-C7-C8	-2.76	107.17	115.77
14	GA	1130	CLA	C4-C3-C5	2.76	120.04	115.29
14	HA	1226	CLA	CAA-C2A-C3A	-2.76	105.23	112.78
14	AA	1137	CLA	O1D-CGD-CBD	-2.76	118.77	124.48
14	bA	1226	CLA	CAA-C2A-C3A	-2.76	105.23	112.78
18	HA	4010	BCR	C33-C5-C6	-2.75	121.44	124.51
18	GA	4008	BCR	C33-C5-C6	-2.75	121.44	124.51
18	RA	4018	BCR	C38-C26-C25	-2.75	121.44	124.51
14	aA	1118	CLA	CBC-CAC-C3C	-2.75	104.78	112.43
14	GA	1012	CLA	C3C-C4C-NC	2.75	113.66	110.57
14	aA	1109	CLA	O2D-CGD-O1D	-2.75	118.39	123.83
18	IA	4018	BCR	C38-C26-C25	-2.75	121.45	124.51
14	aA	1108	CLA	C3C-C4C-NC	2.75	113.66	110.57
14	GA	1013	CLA	CMB-C2B-C1B	-2.75	124.24	128.46
19	BA	5004	LHG	O8-C23-C24	2.75	120.76	111.93
18	HA	4012	BCR	C30-C25-C24	2.75	123.60	115.78
14	aA	1137	CLA	OBD-CAD-C3D	-2.75	123.17	128.04

Continued on next page...

PRELIMINARY VALIDATION REPORT

Continued from previous page...

Mol	Chain	Res	Type	Atoms	Z	Observed(^{o})	Ideal(^{o})
14	HA	1239	CLA	CMC-C2C-C3C	2.75	133.60	126.11
14	bA	1214	CLA	C4-C3-C5	2.75	120.02	115.29
18	bA	4005	BCR	C30-C25-C26	-2.75	118.75	122.59
18	GA	4007	BCR	C38-C26-C27	2.75	118.72	113.57
14	aA	1103	CLA	CAC-C3C-C4C	2.75	128.41	124.82
14	aA	1013	CLA	CMB-C2B-C1B	-2.75	124.24	128.46
14	aA	1116	CLA	CMB-C2B-C3B	2.75	129.95	124.80
14	aA	1135	CLA	C3C-C4C-NC	2.75	113.65	110.57
18	aA	4003	BCR	C1-C6-C5	-2.75	118.75	122.59
14	HA	1214	CLA	C4-C3-C5	2.75	120.02	115.29
18	bA	4014	BCR	C35-C13-C12	2.75	122.45	118.09
21	AA	6002	LMT	O1'-C1'-C2'	2.75	112.64	108.26
14	GA	1108	CLA	C3C-C4C-NC	2.75	113.65	110.57
14	bA	1203	CLA	C3C-C4C-NC	2.75	113.65	110.57
14	XA	1701	CLA	CMB-C2B-C3B	2.75	129.95	124.80
14	HA	1224	CLA	C6-C7-C8	-2.75	107.20	115.77
14	aA	1140	CLA	CMC-C2C-C1C	2.75	129.21	125.03
14	aA	1136	CLA	CMC-C2C-C1C	2.75	129.21	125.03
18	AA	4007	BCR	C38-C26-C27	2.75	118.71	113.57
14	HA	1217	CLA	CMC-C2C-C1C	2.74	129.21	125.03
14	WA	1701	CLA	CMB-C2B-C3B	2.74	129.95	124.80
14	HA	1235	CLA	CMC-C2C-C1C	2.74	129.21	125.03
14	GA	1116	CLA	CMB-C2B-C3B	2.74	129.95	124.80
14	aA	1113	CLA	CBC-CAC-C3C	-2.74	104.81	112.43
14	BA	1214	CLA	C4-C3-C5	2.74	120.01	115.29
18	BA	4016	BCR	C27-C26-C25	-2.74	118.74	122.74
18	HA	4014	BCR	C36-C18-C17	-2.74	119.08	122.92
14	BA	1239	CLA	CMC-C2C-C3C	2.74	133.59	126.11
14	BA	1203	CLA	CMB-C2B-C3B	2.74	129.94	124.80
14	bA	1239	CLA	CMC-C2C-C3C	2.74	133.59	126.11
18	BA	4014	BCR	C34-C9-C8	2.74	122.44	118.09
14	xA	1701	CLA	CMB-C2B-C3B	2.74	129.94	124.80
14	GA	1138	CLA	CMC-C2C-C1C	2.74	129.20	125.03
14	bA	1235	CLA	CMC-C2C-C1C	2.74	129.20	125.03
14	GA	1116	CLA	C1-C2-C3	-2.74	121.30	126.04
14	AA	1013	CLA	C4C-C3C-C2C	-2.74	102.92	106.89
14	AA	1137	CLA	OBD-CAD-C3D	-2.74	123.19	128.04
14	aA	1012	CLA	C3C-C4C-NC	2.74	113.64	110.57
14	GA	1135	CLA	C3C-C4C-NC	2.74	113.64	110.57
14	aA	1131	CLA	C1-C2-C3	-2.74	121.31	126.04
21	aA	6002	LMT	O1'-C1'-C2'	2.74	112.62	108.26
14	HA	1218	CLA	O2D-CGD-O1D	-2.74	118.42	123.83

Continued on next page...

PRELIMINARY VALIDATION REPORT

Continued from previous page...

Mol	Chain	Res	Type	Atoms	Z	Observed(°)	Ideal(°)
14	GA	1139	CLA	C3C-C4C-NC	2.74	113.64	110.57
14	GA	1131	CLA	C1-C2-C3	-2.74	121.31	126.04
18	HA	4014	BCR	C33-C5-C6	-2.74	121.46	124.51
14	BA	1217	CLA	CMC-C2C-C1C	2.74	129.19	125.03
14	aA	1124	CLA	C1-C2-C3	-2.74	121.31	126.04
14	BA	1222	CLA	C3C-C4C-NC	2.74	113.64	110.57
18	bA	4016	BCR	C27-C26-C25	-2.74	118.75	122.74
18	AA	4001	BCR	C34-C9-C10	-2.74	119.09	122.92
19	HA	5004	LHG	O8-C23-C24	2.73	120.71	111.93
14	kA	1401	CLA	C3C-C4C-NC	2.73	113.64	110.57
14	AA	1116	CLA	CMB-C2B-C3B	2.73	129.93	124.80
19	bA	5004	LHG	O8-C23-C24	2.73	120.70	111.93
14	GA	1111	CLA	CMC-C2C-C1C	2.73	129.19	125.03
18	aA	4001	BCR	C34-C9-C10	-2.73	119.09	122.92
14	aA	1138	CLA	C4-C3-C5	2.73	119.99	115.29
14	AA	1013	CLA	CMB-C2B-C1B	-2.73	124.27	128.46
14	bA	1224	CLA	C6-C7-C8	-2.73	107.25	115.77
14	AA	1125	CLA	C1-C2-C3	-2.73	121.32	126.04
14	aA	1111	CLA	OBD-CAD-C3D	-2.73	123.20	128.04
14	UA	1502	CLA	CMB-C2B-C3B	2.73	129.92	124.80
14	AA	1108	CLA	C3C-C4C-NC	2.73	113.63	110.57
14	aA	1139	CLA	C3C-C4C-NC	2.73	113.63	110.57
14	BA	1208	CLA	O2D-CGD-O1D	-2.73	118.44	123.83
14	aA	1122	CLA	CAA-C2A-C3A	-2.73	105.31	112.78
14	HA	1209	CLA	CAA-C2A-C3A	-2.73	105.31	112.78
14	GA	1137	CLA	OBD-CAD-C3D	-2.73	123.21	128.04
14	bA	1208	CLA	O2D-CGD-O1D	-2.73	118.45	123.83
18	GA	4001	BCR	C34-C9-C10	-2.73	119.10	122.92
14	AA	1113	CLA	CBC-CAC-C3C	-2.73	104.86	112.43
14	BA	1205	CLA	CAA-C2A-C3A	-2.72	105.32	112.78
14	GA	1136	CLA	CMC-C2C-C1C	2.72	129.17	125.03
14	AA	1116	CLA	C1-C2-C3	-2.72	121.33	126.04
14	AA	1123	CLA	CMC-C2C-C1C	2.72	129.17	125.03
14	lA	1502	CLA	CMB-C2B-C3B	2.72	129.90	124.80
14	GA	1122	CLA	CAA-C2A-C3A	-2.72	105.33	112.78
14	KA	1401	CLA	C3C-C4C-NC	2.72	113.62	110.57
14	BA	1021	CLA	O2D-CGD-O1D	-2.72	118.46	123.83
14	AA	1138	CLA	CMC-C2C-C1C	2.72	129.17	125.03
14	aA	1111	CLA	CMC-C2C-C1C	2.72	129.17	125.03
18	HA	4004	BCR	C37-C22-C21	-2.72	119.11	122.92
14	HA	1205	CLA	CAA-C2A-C3A	-2.72	105.33	112.78
14	GA	1103	CLA	CAC-C3C-C4C	2.72	128.37	124.82

Continued on next page...

PRELIMINARY VALIDATION REPORT

Continued from previous page...

Mol	Chain	Res	Type	Atoms	Z	Observed(°)	Ideal(°)
21	GA	6002	LMT	O1'-C1'-C2'	2.72	112.59	108.26
14	GA	1128	CLA	CAA-C2A-C3A	-2.72	105.33	112.78
14	HA	1208	CLA	O2D-CGD-O1D	-2.72	118.46	123.83
14	TA	1401	CLA	C3C-C4C-NC	2.72	113.62	110.57
18	BA	4014	BCR	C36-C18-C17	-2.72	119.12	122.92
14	aA	1138	CLA	CMC-C2C-C1C	2.72	129.17	125.03
14	bA	1217	CLA	CMC-C2C-C1C	2.72	129.16	125.03
18	bA	4014	BCR	C36-C18-C17	-2.72	119.12	122.92
18	bA	4004	BCR	C37-C22-C21	-2.72	119.12	122.92
14	bA	1209	CLA	CAA-C2A-C3A	-2.72	105.34	112.78
14	GA	1012	CLA	O2D-CGD-O1D	-2.72	118.47	123.83
14	bA	1204	CLA	C1-C2-C3	-2.72	121.35	126.04
14	aA	1116	CLA	C1-C2-C3	-2.72	121.35	126.04
14	HA	1234	CLA	C1-C2-C3	-2.71	121.35	126.04
14	AA	1135	CLA	C3C-C4C-NC	2.71	113.61	110.57
14	BA	1209	CLA	CAA-C2A-C3A	-2.71	105.35	112.78
14	AA	1111	CLA	OBD-CAD-C3D	-2.71	123.23	128.04
14	GA	1113	CLA	CBC-CAC-C3C	-2.71	104.90	112.43
14	GA	1138	CLA	C4-C3-C5	2.71	119.96	115.29
14	HA	1207	CLA	CAC-C3C-C4C	2.71	128.36	124.82
14	HA	1218	CLA	C3C-C4C-NC	2.71	113.61	110.57
14	aA	1128	CLA	CAA-C2A-C3A	-2.71	105.35	112.78
14	BA	1235	CLA	CMC-C2C-C1C	2.71	129.16	125.03
18	BA	4014	BCR	C35-C13-C12	2.71	122.39	118.09
13	AA	1011	CL0	C3B-C4B-NB	2.71	112.71	109.21
14	HA	1235	CLA	C4-C3-C5	2.71	119.95	115.29
14	bA	1234	CLA	C1-C2-C3	-2.71	121.36	126.04
14	bA	1218	CLA	C3C-C4C-NC	2.71	113.61	110.57
14	LA	1502	CLA	CMB-C2B-C3B	2.71	129.88	124.80
14	HA	1021	CLA	O2D-CGD-O1D	-2.71	118.48	123.83
14	AA	1128	CLA	CAA-C2A-C3A	-2.71	105.36	112.78
14	GA	1111	CLA	OBD-CAD-C3D	-2.71	123.24	128.04
14	aA	1013	CLA	C4C-C3C-C2C	-2.71	102.97	106.89
14	AA	1138	CLA	C4-C3-C5	2.71	119.95	115.29
14	GA	1123	CLA	CMC-C2C-C1C	2.71	129.15	125.03
18	RA	4018	BCR	C38-C26-C27	2.71	118.64	113.57
14	aA	1125	CLA	C1-C2-C3	-2.71	121.36	126.04
14	BA	1218	CLA	C3C-C4C-NC	2.71	113.61	110.57
14	AA	1012	CLA	O2D-CGD-O1D	-2.71	118.49	123.83
14	BA	1204	CLA	C1-O2A-CGA	2.70	123.93	116.54
14	BA	1224	CLA	C1-C2-C3	-2.70	121.37	126.04
14	bA	1205	CLA	CAA-C2A-C3A	-2.70	105.37	112.78

Continued on next page...

PRELIMINARY VALIDATION REPORT

Continued from previous page...

Mol	Chain	Res	Type	Atoms	Z	Observed(^{o})	Ideal(^{o})
18	BA	4004	BCR	C37-C22-C21	-2.70	119.14	122.92
14	bA	1204	CLA	C1-O2A-CGA	2.70	123.93	116.54
18	HA	4016	BCR	C27-C26-C25	-2.70	118.79	122.74
14	bA	1233	CLA	CAC-C3C-C4C	2.70	128.35	124.82
18	AA	4008	BCR	C33-C5-C6	-2.70	121.50	124.51
18	GA	4008	BCR	C36-C18-C17	-2.70	119.14	122.92
14	GA	1128	CLA	C1-C2-C3	-2.70	121.37	126.04
14	aA	1012	CLA	O2D-CGD-O1D	-2.70	118.50	123.83
14	bA	1021	CLA	O2D-CGD-O1D	-2.70	118.50	123.83
14	AA	1111	CLA	C3C-C4C-NC	2.70	113.60	110.57
14	BA	1218	CLA	CMC-C2C-C1C	2.70	129.14	125.03
18	AA	4008	BCR	C36-C18-C17	-2.70	119.14	122.92
14	AA	1118	CLA	OBD-CAD-C3D	-2.70	123.26	128.04
18	lA	4022	BCR	C19-C18-C17	2.70	123.08	118.94
18	BA	4012	BCR	C39-C30-C25	2.70	114.67	110.30
18	aA	4008	BCR	C36-C18-C17	-2.70	119.15	122.92
18	RA	4020	BCR	C38-C26-C25	-2.70	121.51	124.51
14	AA	1111	CLA	CMC-C2C-C1C	2.70	129.13	125.03
14	AA	1128	CLA	C1-C2-C3	-2.69	121.38	126.04
14	BA	1218	CLA	O2D-CGD-O1D	-2.69	118.51	123.83
18	lA	4019	BCR	C34-C9-C10	-2.69	119.15	122.92
14	aA	1123	CLA	CMC-C2C-C1C	2.69	129.13	125.03
14	HA	1224	CLA	C1-C2-C3	-2.69	121.38	126.04
14	HA	1218	CLA	CMC-C2C-C1C	2.69	129.13	125.03
18	iA	4018	BCR	C38-C26-C27	2.69	118.61	113.57
18	IA	4020	BCR	C38-C26-C25	-2.69	121.51	124.51
14	BA	1205	CLA	C1-O2A-CGA	2.69	123.90	116.54
14	AA	1122	CLA	CAA-C2A-C3A	-2.69	105.41	112.78
14	bA	1224	CLA	C1-C2-C3	-2.69	121.39	126.04
14	HA	1222	CLA	C3C-C4C-NC	2.69	113.59	110.57
14	bA	1202	CLA	C3C-C4C-NC	2.69	113.59	110.57
18	bA	4012	BCR	C34-C9-C10	-2.69	119.15	122.92
14	bA	1207	CLA	CAC-C3C-C4C	2.69	128.34	124.82
14	GA	1118	CLA	OBD-CAD-C3D	-2.69	123.28	128.04
18	iA	4018	BCR	C8-C7-C6	-2.69	119.75	127.28
14	aA	1128	CLA	C1-C2-C3	-2.69	121.39	126.04
14	aA	1132	CLA	C1-C2-C3	-2.69	121.39	126.04
14	GA	1111	CLA	C3C-C4C-NC	2.69	113.58	110.57
14	HA	1231	CLA	C3C-C4C-NC	2.69	113.58	110.57
14	BA	1204	CLA	C1-C2-C3	-2.69	121.40	126.04
14	bA	1209	CLA	CMB-C2B-C3B	2.69	129.84	124.80
14	bA	1222	CLA	C3C-C4C-NC	2.68	113.58	110.57

Continued on next page...

PRELIMINARY VALIDATION REPORT

Continued from previous page...

Mol	Chain	Res	Type	Atoms	Z	Observed(°)	Ideal(°)
14	bA	1218	CLA	O2D-CGD-O1D	-2.68	118.53	123.83
14	bA	1205	CLA	C1-O2A-CGA	2.68	123.88	116.54
14	HA	1239	CLA	CAA-C2A-C3A	-2.68	105.43	112.78
14	GA	1013	CLA	C4C-C3C-C2C	-2.68	103.00	106.89
14	BA	1233	CLA	CAC-C3C-C4C	2.68	128.33	124.82
14	BA	1221	CLA	O2D-CGD-O1D	-2.68	118.53	123.83
18	HA	4012	BCR	C39-C30-C25	2.68	114.64	110.30
18	IA	4018	BCR	C38-C26-C27	2.68	118.59	113.57
14	BA	1239	CLA	CAA-C2A-C3A	-2.68	105.44	112.78
14	HA	1204	CLA	C1-C2-C3	-2.68	121.41	126.04
14	HA	1214	CLA	C3C-C4C-NC	2.68	113.58	110.57
18	aA	4008	BCR	C33-C5-C6	-2.68	121.53	124.51
13	GA	1011	CL0	C3B-C4B-NB	2.68	112.67	109.21
14	GA	1125	CLA	C1-C2-C3	-2.68	121.41	126.04
14	BA	1234	CLA	C1-C2-C3	-2.68	121.41	126.04
14	HA	1205	CLA	C1-O2A-CGA	2.68	123.86	116.54
14	HA	1206	CLA	O2D-CGD-O1D	-2.68	118.54	123.83
14	GA	1133	CLA	CMC-C2C-C1C	2.68	129.11	125.03
14	bA	1234	CLA	C4-C3-C5	2.68	119.90	115.29
18	bA	4012	BCR	C39-C30-C25	2.68	114.64	110.30
14	HA	1202	CLA	C3C-C4C-NC	2.68	113.57	110.57
14	bA	1233	CLA	C3C-C4C-NC	2.68	113.57	110.57
14	aA	1118	CLA	OBD-CAD-C3D	-2.68	123.30	128.04
13	aA	1011	CL0	C3B-C4B-NB	2.68	112.67	109.21
14	HA	1233	CLA	CAC-C3C-C4C	2.68	128.32	124.82
18	IA	4018	BCR	C8-C7-C6	-2.68	119.78	127.28
14	bA	1221	CLA	O2D-CGD-O1D	-2.68	118.55	123.83
14	bA	1236	CLA	CAA-C2A-C3A	-2.68	105.45	112.78
18	RA	4018	BCR	C8-C7-C6	-2.68	119.79	127.28
14	BA	1231	CLA	C3C-C4C-NC	2.68	113.57	110.57
14	HA	1221	CLA	O2D-CGD-O1D	-2.67	118.55	123.83
14	AA	1132	CLA	C1-C2-C3	-2.67	121.42	126.04
14	GA	1110	CLA	CMC-C2C-C1C	2.67	129.10	125.03
14	bA	1239	CLA	CAA-C2A-C3A	-2.67	105.46	112.78
18	UA	4022	BCR	C19-C18-C17	2.67	123.04	118.94
14	bA	1231	CLA	C3C-C4C-NC	2.67	113.57	110.57
14	AA	1124	CLA	C3C-C4C-NC	2.67	113.57	110.57
18	LA	4022	BCR	C19-C18-C17	2.67	123.04	118.94
14	AA	1133	CLA	CMC-C2C-C1C	2.67	129.09	125.03
14	HA	1204	CLA	C1-O2A-CGA	2.67	123.84	116.54
14	aA	1133	CLA	CMC-C2C-C1C	2.67	129.09	125.03
14	AA	1128	CLA	C3C-C4C-NC	2.67	113.56	110.57

Continued on next page...

PRELIMINARY VALIDATION REPORT

Continued from previous page...

Mol	Chain	Res	Type	Atoms	Z	Observed(^{o})	Ideal(^{o})
14	AA	1110	CLA	CMC-C2C-C1C	2.67	129.09	125.03
14	GA	1119	CLA	CAC-C3C-C4C	2.67	128.31	124.82
14	BA	1236	CLA	CAA-C2A-C3A	-2.67	105.47	112.78
14	aA	1110	CLA	CMC-C2C-C1C	2.67	129.09	125.03
14	BA	1202	CLA	C3C-C4C-NC	2.67	113.56	110.57
14	BA	1209	CLA	CMB-C2B-C3B	2.67	129.80	124.80
14	HA	1236	CLA	CAA-C2A-C3A	-2.67	105.48	112.78
14	HA	1234	CLA	C4-C3-C5	2.67	119.88	115.29
14	aA	1119	CLA	CAC-C3C-C4C	2.66	128.30	124.82
14	HA	1226	CLA	CMC-C2C-C3C	2.66	133.37	126.11
14	AA	1119	CLA	CAC-C3C-C4C	2.66	128.30	124.82
18	iA	4020	BCR	C38-C26-C25	-2.66	121.54	124.51
18	AA	4011	BCR	C19-C18-C17	2.66	123.03	118.94
14	AA	1115	CLA	C3C-C4C-NC	2.66	113.56	110.57
14	HA	1233	CLA	C3C-C4C-NC	2.66	113.56	110.57
14	bA	1218	CLA	CMC-C2C-C1C	2.66	129.08	125.03
14	bA	1235	CLA	C4-C3-C5	2.66	119.87	115.29
14	BA	1233	CLA	C3C-C4C-NC	2.66	113.55	110.57
14	aA	1117	CLA	O2D-CGD-O1D	-2.66	118.58	123.83
18	bA	4006	BCR	C38-C26-C25	-2.66	121.55	124.51
18	LA	4019	BCR	C34-C9-C10	-2.66	119.20	122.92
14	bA	1213	CLA	C1-C2-C3	-2.66	121.45	126.04
14	GA	1122	CLA	C1-C2-C3	-2.66	121.45	126.04
14	BA	1235	CLA	C4-C3-C5	2.66	119.86	115.29
18	AA	4011	BCR	C36-C18-C19	-2.66	113.88	118.09
14	GA	1103	CLA	C1-C2-C3	-2.65	121.45	126.04
14	GA	1132	CLA	C1-C2-C3	-2.65	121.45	126.04
14	GA	1115	CLA	C3C-C4C-NC	2.65	113.55	110.57
14	BA	1226	CLA	CMC-C2C-C3C	2.65	133.34	126.11
14	GA	1130	CLA	C3C-C4C-NC	2.65	113.55	110.57
14	AA	1117	CLA	O2D-CGD-O1D	-2.65	118.59	123.83
14	GA	1111	CLA	CAA-C2A-C3A	-2.65	105.52	112.78
14	AA	1136	CLA	OBD-CAD-C3D	-2.65	123.34	128.04
14	AA	1130	CLA	C3C-C4C-NC	2.65	113.55	110.57
18	HA	4016	BCR	C30-C25-C26	-2.65	118.89	122.59
18	GA	4011	BCR	C36-C18-C19	-2.65	113.89	118.09
14	aA	1111	CLA	C3C-C4C-NC	2.65	113.54	110.57
18	HA	4012	BCR	C34-C9-C10	-2.65	119.21	122.92
14	GA	1117	CLA	O2D-CGD-O1D	-2.65	118.60	123.83
14	GA	1136	CLA	OBD-CAD-C3D	-2.65	123.35	128.04
14	bA	1203	CLA	CMC-C2C-C1C	2.65	129.06	125.03
14	GA	1128	CLA	C3C-C4C-NC	2.65	113.54	110.57

Continued on next page...

PRELIMINARY VALIDATION REPORT

Continued from previous page...

Mol	Chain	Res	Type	Atoms	Z	Observed(°)	Ideal(°)
14	AA	1127	CLA	CAC-C3C-C4C	2.65	128.28	124.82
14	bA	1238	CLA	CMA-C3A-C4A	2.65	118.89	111.77
14	bA	1206	CLA	O2D-CGD-O1D	-2.65	118.60	123.83
14	BA	1214	CLA	C3C-C4C-NC	2.65	113.54	110.57
14	bA	1204	CLA	CMC-C2C-C1C	2.65	129.06	125.03
14	GA	1105	CLA	CMC-C2C-C1C	2.64	129.06	125.03
14	HA	1203	CLA	CMC-C2C-C1C	2.64	129.05	125.03
14	BA	1203	CLA	CMC-C2C-C1C	2.64	129.05	125.03
14	GA	1135	CLA	O2D-CGD-O1D	-2.64	118.61	123.83
14	AA	1111	CLA	CAA-C2A-C3A	-2.64	105.54	112.78
14	aA	1111	CLA	CAA-C2A-C3A	-2.64	105.54	112.78
18	UA	4019	BCR	C34-C9-C10	-2.64	119.22	122.92
18	aA	4011	BCR	C19-C18-C17	2.64	123.00	118.94
14	BA	1234	CLA	C4-C3-C5	2.64	119.84	115.29
14	AA	1103	CLA	C1-C2-C3	-2.64	121.47	126.04
14	aA	1135	CLA	O2D-CGD-O1D	-2.64	118.61	123.83
14	bA	1233	CLA	O2D-CGD-O1D	-2.64	118.62	123.83
14	bA	1214	CLA	C3C-C4C-NC	2.64	113.53	110.57
14	aA	1105	CLA	CMC-C2C-C1C	2.64	129.04	125.03
14	GA	1110	CLA	CMB-C2B-C3B	2.64	129.75	124.80
18	LA	4019	BCR	C7-C8-C9	-2.64	122.25	126.21
14	aA	1130	CLA	C3C-C4C-NC	2.64	113.53	110.57
14	GA	1124	CLA	C3C-C4C-NC	2.64	113.53	110.57
14	bA	1226	CLA	CMC-C2C-C3C	2.64	133.30	126.11
14	BA	1206	CLA	O2D-CGD-O1D	-2.64	118.62	123.83
14	bA	1214	CLA	CMB-C2B-C3B	2.64	129.74	124.80
18	aA	4011	BCR	C36-C18-C19	-2.64	113.91	118.09
14	BA	1207	CLA	CAC-C3C-C4C	2.64	128.26	124.82
18	BA	4016	BCR	C30-C25-C26	-2.63	118.91	122.59
14	aA	1122	CLA	C1-C2-C3	-2.63	121.49	126.04
14	aA	1136	CLA	OBD-CAD-C3D	-2.63	123.37	128.04
14	aA	1116	CLA	O2D-CGD-O1D	-2.63	118.63	123.83
18	UA	4019	BCR	C7-C8-C9	-2.63	122.26	126.21
18	lA	4019	BCR	C37-C22-C21	-2.63	119.23	122.92
14	bA	1023	CLA	C4-C3-C5	2.63	119.82	115.29
14	HA	1226	CLA	CHC-C1C-C2C	-2.63	119.45	126.71
14	BA	1238	CLA	CMA-C3A-C4A	2.63	118.85	111.77
18	BA	4004	BCR	C15-C14-C13	-2.63	123.56	127.31
14	BA	1214	CLA	CMB-C2B-C3B	2.63	129.73	124.80
14	aA	1113	CLA	CMA-C3A-C4A	2.63	118.84	111.77
18	bA	4016	BCR	C30-C25-C26	-2.63	118.92	122.59
14	HA	1238	CLA	CMA-C3A-C4A	2.63	118.84	111.77

Continued on next page...

PRELIMINARY VALIDATION REPORT

Continued from previous page...

Mol	Chain	Res	Type	Atoms	Z	Observed(°)	Ideal(°)
14	AA	1135	CLA	O2D-CGD-O1D	-2.63	118.64	123.83
14	HA	1233	CLA	O2D-CGD-O1D	-2.63	118.64	123.83
14	BA	1023	CLA	C4-C3-C5	2.63	119.81	115.29
18	VA	4021	BCR	C39-C30-C25	-2.63	106.05	110.30
18	LA	4019	BCR	C37-C22-C21	-2.63	119.24	122.92
14	GA	1113	CLA	CMA-C3A-C4A	2.63	118.83	111.77
18	BA	4012	BCR	C34-C9-C10	-2.63	119.24	122.92
14	aA	1114	CLA	O2D-CGD-O1D	-2.63	118.64	123.83
14	BA	1213	CLA	C1-C2-C3	-2.63	121.50	126.04
14	aA	1127	CLA	CAC-C3C-C4C	2.63	128.25	124.82
14	BA	1234	CLA	CGD-CBD-CAD	-2.63	102.23	110.73
18	GA	4003	BCR	C36-C18-C17	-2.63	119.25	122.92
14	HA	1214	CLA	CMB-C2B-C3B	2.63	129.72	124.80
18	bA	4004	BCR	C15-C14-C13	-2.63	123.56	127.31
18	UA	4019	BCR	C37-C22-C21	-2.62	119.25	122.92
14	AA	1112	CLA	C3C-C4C-NC	2.62	113.51	110.57
14	AA	1113	CLA	CMA-C3A-C4A	2.62	118.82	111.77
14	HA	1023	CLA	C4-C3-C5	2.62	119.81	115.29
14	HA	1234	CLA	CGD-CBD-CAD	-2.62	102.24	110.73
14	HA	1209	CLA	CMB-C2B-C3B	2.62	129.72	124.80
14	aA	1103	CLA	C1-C2-C3	-2.62	121.51	126.04
14	aA	1110	CLA	CMB-C2B-C3B	2.62	129.72	124.80
14	BA	1226	CLA	CHC-C1C-C2C	-2.62	119.48	126.71
14	aA	1117	CLA	CHC-C1C-C2C	-2.62	119.48	126.71
18	HA	4013	BCR	C36-C18-C17	-2.62	119.25	122.92
14	bA	1226	CLA	CHC-C1C-C2C	-2.62	119.48	126.71
14	HA	1219	CLA	CMC-C2C-C1C	2.62	129.02	125.03
14	BA	1205	CLA	CMC-C2C-C1C	2.62	129.02	125.03
14	AA	1110	CLA	CMB-C2B-C3B	2.62	129.72	124.80
18	bA	4005	BCR	C36-C18-C17	-2.62	119.25	122.92
14	GA	1127	CLA	CAC-C3C-C4C	2.62	128.24	124.82
14	GA	1101	CLA	CMA-C3A-C4A	2.62	118.81	111.77
14	bA	1234	CLA	CGD-CBD-CAD	-2.62	102.26	110.73
14	HA	1204	CLA	CMC-C2C-C1C	2.62	129.01	125.03
18	aA	4011	BCR	C1-C6-C5	-2.62	118.93	122.59
18	HA	4004	BCR	C15-C14-C13	-2.62	123.58	127.31
14	aA	1119	CLA	C3C-C4C-NC	2.62	113.50	110.57
14	bA	1236	CLA	CMC-C2C-C1C	2.62	129.01	125.03
14	BA	1233	CLA	O2D-CGD-O1D	-2.62	118.67	123.83
14	aA	1105	CLA	CAC-C3C-C4C	2.61	128.24	124.82
14	aA	1128	CLA	C3C-C4C-NC	2.61	113.50	110.57
14	aA	1012	CLA	C1-O2A-CGA	2.61	123.69	116.54

Continued on next page...

PRELIMINARY VALIDATION REPORT

Continued from previous page...

Mol	Chain	Res	Type	Atoms	Z	Observed(^{o})	Ideal(^{o})
14	AA	1122	CLA	C1-C2-C3	-2.61	121.52	126.04
14	BA	1210	CLA	C3C-C4C-NC	2.61	113.50	110.57
14	bA	1210	CLA	CAA-CBA-CGA	-2.61	105.56	113.26
14	AA	1101	CLA	CMA-C3A-C4A	2.61	118.79	111.77
14	GA	1117	CLA	CHC-C1C-C2C	-2.61	119.51	126.71
18	GA	4011	BCR	C19-C18-C17	2.61	122.95	118.94
14	BA	1236	CLA	CMC-C2C-C1C	2.61	129.00	125.03
18	bA	4013	BCR	C36-C18-C17	-2.61	119.27	122.92
14	GA	1117	CLA	CMA-C3A-C4A	2.61	118.79	111.77
14	HA	1236	CLA	CMC-C2C-C1C	2.61	129.00	125.03
14	AA	1012	CLA	C1-O2A-CGA	2.61	123.68	116.54
14	GA	1119	CLA	C3C-C4C-NC	2.61	113.50	110.57
18	lA	4022	BCR	C15-C14-C13	-2.61	123.58	127.31
14	AA	1117	CLA	CHC-C1C-C2C	-2.61	119.52	126.71
14	aA	1117	CLA	CMA-C3A-C4A	2.61	118.79	111.77
14	BA	1210	CLA	CAA-CBA-CGA	-2.61	105.57	113.26
18	lA	4019	BCR	C7-C8-C9	-2.61	122.29	126.21
18	HA	4006	BCR	C38-C26-C25	-2.61	121.61	124.51
14	HA	1231	CLA	CAC-C3C-C4C	2.61	128.23	124.82
14	AA	1114	CLA	O2D-CGD-O1D	-2.61	118.68	123.83
14	HA	1213	CLA	C1-C2-C3	-2.61	121.53	126.04
14	HA	1239	CLA	O2D-CGD-O1D	-2.61	118.68	123.83
14	bA	1219	CLA	CMC-C2C-C1C	2.61	129.00	125.03
14	BA	1231	CLA	O2D-CGD-O1D	-2.61	118.68	123.83
18	BA	4006	BCR	C38-C26-C25	-2.61	121.61	124.51
14	HA	1229	CLA	CMC-C2C-C1C	2.61	129.00	125.03
14	GA	1112	CLA	O2D-CGD-O1D	-2.60	118.69	123.83
14	aA	1101	CLA	CMA-C3A-C4A	2.60	118.77	111.77
14	bA	1239	CLA	O2D-CGD-O1D	-2.60	118.69	123.83
18	GA	4002	BCR	C3-C4-C5	-2.60	109.46	113.99
18	HA	4016	BCR	C33-C5-C6	-2.60	121.61	124.51
14	BA	1227	CLA	C3C-C4C-NC	2.60	113.49	110.57
14	AA	1105	CLA	CMC-C2C-C1C	2.60	128.99	125.03
14	bA	1227	CLA	C3C-C4C-NC	2.60	113.49	110.57
18	aA	4002	BCR	C3-C4-C5	-2.60	109.46	113.99
18	LA	4019	BCR	C1-C6-C5	-2.60	118.95	122.59
14	GA	1012	CLA	C1-O2A-CGA	2.60	123.65	116.54
14	BA	1204	CLA	CMC-C2C-C1C	2.60	128.99	125.03
14	GA	1105	CLA	CAC-C3C-C4C	2.60	128.22	124.82
14	aA	1115	CLA	C3C-C4C-NC	2.60	113.49	110.57
14	BA	1219	CLA	CMC-C2C-C1C	2.60	128.99	125.03
14	HA	1210	CLA	C3C-C4C-NC	2.60	113.49	110.57

Continued on next page...

PRELIMINARY VALIDATION REPORT

Continued from previous page...

Mol	Chain	Res	Type	Atoms	Z	Observed(°)	Ideal(°)
18	lA	4019	BCR	C1-C6-C5	-2.60	118.96	122.59
14	HA	1210	CLA	CAA-CBA-CGA	-2.60	105.60	113.26
14	bA	1224	CLA	CHD-C4C-C3C	-2.60	121.08	124.87
14	aA	1124	CLA	C3C-C4C-NC	2.60	113.48	110.57
14	GA	1102	CLA	C3C-C4C-NC	2.60	113.48	110.57
14	aA	1112	CLA	C3C-C4C-NC	2.60	113.48	110.57
18	LA	4022	BCR	C15-C14-C13	-2.60	123.60	127.31
14	BA	1227	CLA	CMA-C3A-C4A	2.60	118.75	111.77
18	GA	4011	BCR	C1-C6-C5	-2.60	118.96	122.59
14	bA	1231	CLA	CAC-C3C-C4C	2.60	128.21	124.82
18	mA	4021	BCR	C39-C30-C25	-2.60	106.11	110.30
14	HA	1216	CLA	CMB-C2B-C3B	2.59	129.67	124.80
14	bA	1210	CLA	C3C-C4C-NC	2.59	113.48	110.57
14	BA	1216	CLA	C4-C3-C5	2.59	119.75	115.29
14	AA	1116	CLA	O2D-CGD-O1D	-2.59	118.71	123.83
18	AA	4007	BCR	C7-C8-C9	-2.59	122.32	126.21
14	AA	1119	CLA	C3C-C4C-NC	2.59	113.48	110.57
14	GA	1114	CLA	O2D-CGD-O1D	-2.59	118.71	123.83
14	bA	1227	CLA	CMA-C3A-C4A	2.59	118.74	111.77
14	aA	1112	CLA	O2D-CGD-O1D	-2.59	118.71	123.83
14	HA	1224	CLA	CHD-C4C-C3C	-2.59	121.09	124.87
14	HA	1217	CLA	CMA-C3A-C4A	2.59	118.73	111.77
14	BA	1220	CLA	O2D-CGD-O1D	-2.59	118.72	123.83
14	HA	1227	CLA	CMA-C3A-C4A	2.59	118.73	111.77
14	bA	1216	CLA	CMB-C2B-C3B	2.59	129.66	124.80
18	AA	4011	BCR	C1-C6-C5	-2.59	118.97	122.59
14	BA	1231	CLA	CAC-C3C-C4C	2.59	128.20	124.82
14	bA	1231	CLA	O2D-CGD-O1D	-2.59	118.72	123.83
14	BA	1229	CLA	CAA-C2A-C1A	-2.59	103.49	111.97
14	AA	1139	CLA	CMB-C2B-C3B	2.59	129.65	124.80
14	HA	1231	CLA	O2D-CGD-O1D	-2.59	118.72	123.83
14	HA	1220	CLA	O2D-CGD-O1D	-2.59	118.72	123.83
14	BA	1216	CLA	CAA-C2A-C3A	-2.59	105.70	112.78
14	BA	1217	CLA	CMA-C3A-C4A	2.58	118.72	111.77
14	AA	1117	CLA	CMA-C3A-C4A	2.58	118.72	111.77
18	MA	4021	BCR	C39-C30-C25	-2.58	106.12	110.30
14	AA	1112	CLA	O2D-CGD-O1D	-2.58	118.73	123.83
14	GA	1105	CLA	O2D-CGD-O1D	-2.58	118.73	123.83
14	GA	1112	CLA	C3C-C4C-NC	2.58	113.47	110.57
14	GA	1116	CLA	O2D-CGD-O1D	-2.58	118.73	123.83
14	BA	1023	CLA	C1-C2-C3	-2.58	121.58	126.04
18	BA	4012	BCR	C3-C4-C5	-2.58	109.50	113.99

Continued on next page...

PRELIMINARY VALIDATION REPORT

Continued from previous page...

Mol	Chain	Res	Type	Atoms	Z	Observed(^{o})	Ideal(^{o})
14	aA	1102	CLA	C3C-C4C-NC	2.58	113.47	110.57
14	HA	1208	CLA	CMC-C2C-C1C	2.58	128.96	125.03
14	bA	1229	CLA	CMC-C2C-C1C	2.58	128.96	125.03
14	bA	1208	CLA	CMC-C2C-C1C	2.58	128.96	125.03
18	HA	4014	BCR	C34-C9-C10	-2.58	119.31	122.92
18	aA	4003	BCR	C36-C18-C17	-2.58	119.31	122.92
14	lA	1501	CLA	CMC-C2C-C1C	2.58	128.96	125.03
14	UA	1501	CLA	CMC-C2C-C1C	2.58	128.96	125.03
18	BA	4016	BCR	C34-C9-C8	2.58	122.18	118.09
14	HA	1227	CLA	C3C-C4C-NC	2.58	113.46	110.57
18	AA	4002	BCR	C3-C4-C5	-2.58	109.50	113.99
14	bA	1216	CLA	CAA-C2A-C3A	-2.58	105.72	112.78
14	GA	1107	CLA	CAC-C3C-C4C	2.58	128.19	124.82
14	AA	1105	CLA	CAC-C3C-C4C	2.58	128.19	124.82
14	BA	1239	CLA	O2D-CGD-O1D	-2.58	118.74	123.83
14	HA	1229	CLA	CAA-C2A-C1A	-2.58	103.53	111.97
18	HA	4012	BCR	C3-C4-C5	-2.58	109.51	113.99
18	UA	4022	BCR	C15-C14-C13	-2.58	123.63	127.31
14	BA	1208	CLA	CMC-C2C-C1C	2.58	128.95	125.03
18	BA	4005	BCR	C36-C18-C17	-2.57	119.32	122.92
14	AA	1115	CLA	O2D-CGD-O1D	-2.57	118.75	123.83
14	bA	1217	CLA	CMA-C3A-C4A	2.57	118.69	111.77
14	BA	1216	CLA	CMB-C2B-C3B	2.57	129.63	124.80
14	BA	1228	CLA	CAA-C2A-C3A	-2.57	105.73	112.78
14	AA	1106	CLA	O1D-CGD-CBD	-2.57	119.15	124.48
14	GA	1115	CLA	O2D-CGD-O1D	-2.57	118.75	123.83
14	GA	1106	CLA	O1D-CGD-CBD	-2.57	119.15	124.48
18	aA	4007	BCR	C7-C8-C9	-2.57	122.35	126.21
14	HA	1216	CLA	CAA-C2A-C3A	-2.57	105.74	112.78
18	BA	4013	BCR	C36-C18-C17	-2.57	119.32	122.92
14	HA	1216	CLA	C4-C3-C5	2.57	119.71	115.29
18	bA	4010	BCR	C34-C9-C8	2.57	122.17	118.09
18	IA	4020	BCR	C33-C5-C6	-2.57	121.65	124.51
14	bA	1229	CLA	CAA-C2A-C1A	-2.57	103.56	111.97
14	BA	1224	CLA	CHD-C4C-C3C	-2.57	121.12	124.87
14	aA	1115	CLA	O2D-CGD-O1D	-2.57	118.76	123.83
18	BA	4012	BCR	C36-C18-C17	-2.57	119.33	122.92
14	aA	1106	CLA	O1D-CGD-CBD	-2.57	119.16	124.48
14	HA	1228	CLA	CAA-C2A-C3A	-2.57	105.75	112.78
14	AA	1105	CLA	O2D-CGD-O1D	-2.57	118.76	123.83
18	bA	4012	BCR	C36-C18-C17	-2.57	119.33	122.92
14	BA	1229	CLA	CMC-C2C-C1C	2.57	128.94	125.03

Continued on next page...

PRELIMINARY VALIDATION REPORT

Continued from previous page...

Mol	Chain	Res	Type	Atoms	Z	Observed(^{o})	Ideal(^{o})
18	UA	4019	BCR	C1-C6-C5	-2.57	119.00	122.59
18	GA	4001	BCR	C33-C5-C6	-2.57	121.65	124.51
14	bA	1228	CLA	CAA-C2A-C3A	-2.56	105.75	112.78
18	AA	4003	BCR	C36-C18-C17	-2.56	119.33	122.92
14	bA	1023	CLA	C1-C2-C3	-2.56	121.61	126.04
18	BA	4016	BCR	C33-C5-C6	-2.56	121.66	124.51
18	bA	4016	BCR	C33-C5-C6	-2.56	121.66	124.51
18	GA	4007	BCR	C7-C8-C9	-2.56	122.36	126.21
14	bA	1205	CLA	CMC-C2C-C1C	2.56	128.93	125.03
18	AA	4001	BCR	C33-C5-C6	-2.56	121.66	124.51
14	aA	1105	CLA	O2D-CGD-O1D	-2.56	118.77	123.83
14	LA	1501	CLA	CMC-C2C-C1C	2.56	128.93	125.03
18	HA	4010	BCR	C34-C9-C8	2.56	122.15	118.09
18	lA	4019	BCR	C34-C9-C8	2.56	122.15	118.09
18	AA	4008	BCR	C19-C18-C17	2.56	122.87	118.94
14	bA	1216	CLA	C4-C3-C5	2.56	119.70	115.29
14	HA	1231	CLA	CMB-C2B-C1B	-2.56	124.53	128.46
14	HA	1209	CLA	CMC-C2C-C1C	2.56	128.93	125.03
14	bA	1220	CLA	O2D-CGD-O1D	-2.56	118.78	123.83
18	HA	4005	BCR	C36-C18-C17	-2.56	119.34	122.92
14	bA	1204	CLA	OBD-CAD-C3D	-2.55	123.52	128.04
18	GA	4008	BCR	C19-C18-C17	2.55	122.86	118.94
18	aA	4001	BCR	C33-C5-C6	-2.55	121.67	124.51
14	aA	1139	CLA	CMB-C2B-C3B	2.55	129.59	124.80
18	HA	4016	BCR	C34-C9-C8	2.55	122.14	118.09
18	RA	4020	BCR	C33-C5-C6	-2.55	121.67	124.51
18	AA	4003	BCR	C39-C30-C25	-2.55	106.17	110.30
14	bA	1231	CLA	CMB-C2B-C1B	-2.55	124.54	128.46
18	UA	4019	BCR	C34-C9-C8	2.55	122.14	118.09
14	bA	1213	CLA	O2D-CGD-O1D	-2.55	118.79	123.83
14	HA	1217	CLA	C1-C2-C3	-2.55	121.63	126.04
14	BA	1212	CLA	CMC-C2C-C1C	2.55	128.91	125.03
14	AA	1117	CLA	CAA-C2A-C1A	-2.55	103.62	111.97
14	BA	1214	CLA	CAA-C2A-C3A	-2.55	105.80	112.78
18	HA	4004	BCR	C34-C9-C10	-2.55	119.35	122.92
14	GA	1117	CLA	CAA-C2A-C1A	-2.55	103.62	111.97
18	GA	4003	BCR	C39-C30-C25	-2.55	106.18	110.30
14	aA	1107	CLA	CAC-C3C-C4C	2.55	128.15	124.82
18	HA	4013	BCR	C33-C5-C6	-2.55	121.67	124.51
14	AA	1115	CLA	C1-C2-C3	-2.55	121.64	126.04
14	aA	1117	CLA	CAA-C2A-C1A	-2.55	103.62	111.97
14	aA	1108	CLA	CMC-C2C-C1C	2.55	128.91	125.03

Continued on next page...

PRELIMINARY VALIDATION REPORT

Continued from previous page...

Mol	Chain	Res	Type	Atoms	Z	Observed(^{o})	Ideal(^{o})
14	bA	1238	CLA	CMC-C2C-C1C	2.55	128.91	125.03
14	bA	1235	CLA	CBA-CAA-C2A	2.55	121.43	113.85
14	bA	1209	CLA	CMC-C2C-C1C	2.55	128.91	125.03
18	LA	4019	BCR	C34-C9-C8	2.55	122.13	118.09
14	BA	1217	CLA	C1-C2-C3	-2.55	121.64	126.04
14	AA	1107	CLA	CAC-C3C-C4C	2.55	128.15	124.82
14	HA	1205	CLA	CMC-C2C-C1C	2.55	128.91	125.03
18	BA	4010	BCR	C34-C9-C8	2.55	122.13	118.09
14	HA	1235	CLA	CBA-CAA-C2A	2.55	121.43	113.85
14	GA	1117	CLA	CMB-C2B-C1B	-2.55	124.55	128.46
18	bA	4016	BCR	C34-C9-C8	2.55	122.13	118.09
18	aA	4003	BCR	C39-C30-C25	-2.55	106.19	110.30
14	HA	1213	CLA	O2D-CGD-O1D	-2.54	118.81	123.83
14	HA	1214	CLA	CAA-C2A-C3A	-2.54	105.81	112.78
18	bA	4014	BCR	C34-C9-C10	-2.54	119.36	122.92
18	bA	4012	BCR	C3-C4-C5	-2.54	109.56	113.99
14	bA	1225	CLA	C1-C2-C3	-2.54	121.64	126.04
14	AA	1127	CLA	C1-O2A-CGA	2.54	123.49	116.54
14	bA	1214	CLA	CAA-C2A-C3A	-2.54	105.81	112.78
14	AA	1013	CLA	C4-C3-C5	2.54	119.67	115.29
14	bA	1212	CLA	CMC-C2C-C1C	2.54	128.90	125.03
14	GA	1127	CLA	C1-C2-C3	-2.54	121.65	126.04
14	HA	1023	CLA	C1-C2-C3	-2.54	121.65	126.04
18	IA	4020	BCR	C23-C22-C21	-2.54	115.04	118.94
14	BA	1235	CLA	CBA-CAA-C2A	2.54	121.41	113.85
14	aA	1123	CLA	C4-C3-C5	2.54	119.66	115.29
18	iA	4020	BCR	C33-C5-C6	-2.54	121.68	124.51
14	GA	1139	CLA	CMB-C2B-C3B	2.54	129.56	124.80
14	BA	1228	CLA	O2D-CGD-O1D	-2.54	118.82	123.83
14	aA	1127	CLA	C1-C2-C3	-2.54	121.66	126.04
14	BA	1220	CLA	C3C-C4C-NC	2.54	113.42	110.57
14	HA	1225	CLA	C1-C2-C3	-2.54	121.66	126.04
14	GA	1127	CLA	C1-O2A-CGA	2.54	123.47	116.54
14	AA	1117	CLA	CMB-C2B-C1B	-2.54	124.57	128.46
14	HA	1235	CLA	C3C-C4C-NC	2.54	113.42	110.57
14	AA	1131	CLA	C3C-C4C-NC	2.54	113.41	110.57
14	HA	1212	CLA	CMC-C2C-C1C	2.53	128.89	125.03
14	GA	1115	CLA	C1-C2-C3	-2.53	121.66	126.04
14	HA	1238	CLA	CMC-C2C-C1C	2.53	128.89	125.03
14	AA	1102	CLA	C3C-C4C-NC	2.53	113.41	110.57
14	BA	1225	CLA	C1-C2-C3	-2.53	121.66	126.04
18	RA	4020	BCR	C23-C22-C21	-2.53	115.06	118.94

Continued on next page...

PRELIMINARY VALIDATION REPORT

Continued from previous page...

Mol	Chain	Res	Type	Atoms	Z	Observed(^{o})	Ideal(^{o})
14	AA	1108	CLA	CMC-C2C-C1C	2.53	128.88	125.03
14	GA	1108	CLA	CMC-C2C-C1C	2.53	128.88	125.03
14	AA	1133	CLA	C3C-C4C-NC	2.53	113.41	110.57
18	HA	4012	BCR	C36-C18-C17	-2.53	119.38	122.92
18	BA	4004	BCR	C34-C9-C10	-2.53	119.38	122.92
18	iA	4020	BCR	C23-C22-C21	-2.53	115.06	118.94
14	HA	1023	CLA	CHC-C1C-C2C	-2.53	119.74	126.71
14	BA	1231	CLA	CMB-C2B-C1B	-2.53	124.58	128.46
14	BA	1213	CLA	O2D-CGD-O1D	-2.53	118.83	123.83
14	AA	1123	CLA	C4-C3-C5	2.53	119.64	115.29
18	bA	4004	BCR	C34-C9-C10	-2.53	119.38	122.92
18	BA	4013	BCR	C33-C5-C6	-2.53	121.69	124.51
14	aA	1117	CLA	CMB-C2B-C1B	-2.53	124.58	128.46
14	BA	1209	CLA	CMC-C2C-C1C	2.53	128.88	125.03
14	aA	1124	CLA	CAA-C2A-C1A	-2.53	103.69	111.97
14	BA	1238	CLA	CMC-C2C-C1C	2.53	128.88	125.03
14	AA	1127	CLA	C1-C2-C3	-2.53	121.67	126.04
18	aA	4008	BCR	C19-C18-C17	2.53	122.82	118.94
14	HA	1228	CLA	O2D-CGD-O1D	-2.53	118.84	123.83
14	BA	1213	CLA	C3C-C4C-NC	2.53	113.40	110.57
14	AA	1124	CLA	CAA-C2A-C1A	-2.53	103.70	111.97
14	LA	1502	CLA	CMC-C2C-C1C	2.53	128.87	125.03
14	aA	1127	CLA	C1-O2A-CGA	2.52	123.44	116.54
14	BA	1222	CLA	CMA-C3A-C4A	2.52	118.56	111.77
14	HA	1220	CLA	C3C-C4C-NC	2.52	113.40	110.57
14	HA	1213	CLA	C4-C3-C5	2.52	119.63	115.29
14	bA	1215	CLA	O2D-CGD-O1D	-2.52	118.85	123.83
14	bA	1023	CLA	CHC-C1C-C2C	-2.52	119.75	126.71
14	AA	1138	CLA	C1-O2A-CGA	2.52	123.44	116.54
14	bA	1217	CLA	C1-C2-C3	-2.52	121.68	126.04
14	BA	1204	CLA	OBD-CAD-C3D	-2.52	123.57	128.04
14	BA	1235	CLA	C1-O2A-CGA	2.52	123.43	116.54
14	bA	1235	CLA	C1-O2A-CGA	2.52	123.43	116.54
14	aA	1105	CLA	C3C-C4C-NC	2.52	113.40	110.57
14	aA	1137	CLA	CMC-C2C-C1C	2.52	128.87	125.03
14	aA	1115	CLA	C1-C2-C3	-2.52	121.68	126.04
14	BA	1215	CLA	O2D-CGD-O1D	-2.52	118.85	123.83
14	BA	1213	CLA	O1D-CGD-CBD	-2.52	119.25	124.48
14	GA	1124	CLA	CAA-C2A-C1A	-2.52	103.71	111.97
14	HA	1213	CLA	O1D-CGD-CBD	-2.52	119.26	124.48
14	GA	1140	CLA	CMB-C2B-C3B	2.52	129.53	124.80
14	bA	1213	CLA	C4-C3-C5	2.52	119.63	115.29

Continued on next page...

PRELIMINARY VALIDATION REPORT

Continued from previous page...

Mol	Chain	Res	Type	Atoms	Z	Observed(°)	Ideal(°)
14	bA	1222	CLA	CMA-C3A-C4A	2.52	118.55	111.77
14	HA	1235	CLA	C1-O2A-CGA	2.52	123.43	116.54
14	bA	1228	CLA	O2D-CGD-O1D	-2.52	118.86	123.83
14	HA	1223	CLA	CMC-C2C-C1C	2.52	128.86	125.03
14	bA	1235	CLA	C3C-C4C-NC	2.52	113.39	110.57
14	GA	1013	CLA	C4-C3-C5	2.52	119.62	115.29
14	HA	1215	CLA	O2D-CGD-O1D	-2.52	118.86	123.83
14	bA	1211	CLA	C4-C3-C5	2.52	119.62	115.29
14	aA	1101	CLA	CMB-C2B-C3B	2.51	129.52	124.80
14	AA	1101	CLA	CMB-C2B-C3B	2.51	129.51	124.80
14	BA	1023	CLA	CHC-C1C-C2C	-2.51	119.78	126.71
14	UA	1502	CLA	CMC-C2C-C1C	2.51	128.86	125.03
18	BA	4014	BCR	C34-C9-C10	-2.51	119.40	122.92
18	AA	4007	BCR	C1-C6-C5	-2.51	119.08	122.59
18	GA	4007	BCR	C1-C6-C5	-2.51	119.08	122.59
14	aA	1122	CLA	CED-O2D-CGD	2.51	121.69	115.95
14	GA	1123	CLA	C4-C3-C5	2.51	119.61	115.29
14	UA	1502	CLA	O1D-CGD-CBD	-2.51	119.28	124.48
18	bA	4006	BCR	C15-C14-C13	-2.51	123.73	127.31
14	aA	1012	CLA	CMC-C2C-C1C	2.51	128.85	125.03
14	aA	1138	CLA	C1-O2A-CGA	2.51	123.40	116.54
14	BA	1213	CLA	C4-C3-C5	2.51	119.61	115.29
14	HA	1213	CLA	C3C-C4C-NC	2.51	113.39	110.57
14	UA	1503	CLA	C4-C3-C5	2.51	119.61	115.29
14	BA	1223	CLA	CMC-C2C-C1C	2.51	128.85	125.03
14	HA	1211	CLA	C4-C3-C5	2.51	119.61	115.29
14	GA	1116	CLA	CBA-CAA-C2A	2.51	121.32	113.85
14	bA	1213	CLA	C3C-C4C-NC	2.51	113.38	110.57
14	BA	1210	CLA	OBD-CAD-C3D	-2.51	123.60	128.04
18	bA	4013	BCR	C2-C1-C6	2.51	114.36	110.48
14	lA	1503	CLA	C4-C3-C5	2.51	119.61	115.29
14	HA	1204	CLA	OBD-CAD-C3D	-2.51	123.60	128.04
18	AA	4011	BCR	C28-C27-C26	-2.51	109.63	113.99
18	AA	4008	BCR	C35-C13-C12	2.51	122.07	118.09
21	aA	6001	LMT	C1'-O5'-C5'	2.51	118.64	113.70
21	AA	6001	LMT	C1'-O5'-C5'	2.51	118.64	113.70
14	GA	1122	CLA	CED-O2D-CGD	2.51	121.67	115.95
14	GA	1133	CLA	C3C-C4C-NC	2.51	113.38	110.57
14	HA	1222	CLA	CMA-C3A-C4A	2.51	118.51	111.77
14	HA	1201	CLA	CHB-C4A-NA	2.51	127.98	124.51
14	GA	1116	CLA	C3C-C4C-NC	2.50	113.38	110.57
14	bA	1223	CLA	CMC-C2C-C1C	2.50	128.84	125.03

Continued on next page...

PRELIMINARY VALIDATION REPORT

Continued from previous page...

Mol	Chain	Res	Type	Atoms	Z	Observed(^{o})	Ideal(^{o})
14	bA	1213	CLA	O1D-CGD-CBD	-2.50	119.29	124.48
14	aA	1013	CLA	C4-C3-C5	2.50	119.60	115.29
14	aA	1131	CLA	C3C-C4C-NC	2.50	113.38	110.57
14	bA	1023	CLA	C1C-C2C-C3C	-2.50	104.30	106.95
14	GA	1101	CLA	CMB-C2B-C3B	2.50	129.49	124.80
14	aA	1133	CLA	C3C-C4C-NC	2.50	113.38	110.57
14	LA	1503	CLA	C4-C3-C5	2.50	119.59	115.29
14	AA	1012	CLA	CHA-C1A-NA	-2.50	120.67	126.40
18	HA	4006	BCR	C15-C14-C13	-2.50	123.74	127.31
14	lA	1502	CLA	CMC-C2C-C1C	2.50	128.83	125.03
14	GA	1131	CLA	C3C-C4C-NC	2.50	113.37	110.57
18	GA	4011	BCR	C28-C27-C26	-2.50	109.64	113.99
14	GA	1137	CLA	CMC-C2C-C1C	2.50	128.83	125.03
18	BA	4013	BCR	C2-C1-C6	2.50	114.35	110.48
14	AA	1116	CLA	CBA-CAA-C2A	2.50	121.28	113.85
14	AA	1135	CLA	OBD-CAD-C3D	-2.50	123.62	128.04
14	HA	1023	CLA	C1C-C2C-C3C	-2.50	104.30	106.95
14	aA	1116	CLA	CBA-CAA-C2A	2.50	121.28	113.85
18	BA	4006	BCR	C15-C14-C13	-2.50	123.75	127.31
14	AA	1122	CLA	CED-O2D-CGD	2.50	121.65	115.95
18	aA	4007	BCR	C1-C6-C5	-2.50	119.10	122.59
14	bA	1210	CLA	OBD-CAD-C3D	-2.50	123.62	128.04
14	BA	1232	CLA	C3C-C4C-NC	2.49	113.37	110.57
14	aA	1135	CLA	OBD-CAD-C3D	-2.49	123.62	128.04
14	AA	1140	CLA	CMB-C2B-C3B	2.49	129.48	124.80
14	aA	1118	CLA	CMB-C2B-C3B	2.49	129.47	124.80
18	IA	4020	BCR	C23-C24-C25	-2.49	120.30	127.28
14	GA	1135	CLA	OBD-CAD-C3D	-2.49	123.62	128.04
14	BA	1203	CLA	C4-C3-C2	-2.49	117.20	123.68
21	GA	6001	LMT	C1'-O5'-C5'	2.49	118.61	113.70
14	bA	1231	CLA	C4-C3-C5	2.49	119.58	115.29
14	AA	1116	CLA	C3C-C4C-NC	2.49	113.36	110.57
14	HA	1232	CLA	C3C-C4C-NC	2.49	113.36	110.57
14	BA	1210	CLA	CMA-C3A-C4A	2.49	118.47	111.77
14	aA	1140	CLA	CMB-C2B-C3B	2.49	129.47	124.80
18	iA	4018	BCR	C1-C6-C5	-2.49	119.11	122.59
18	bA	4013	BCR	C33-C5-C6	-2.49	121.74	124.51
14	BA	1235	CLA	C3C-C4C-NC	2.49	113.36	110.57
14	AA	1118	CLA	CMB-C2B-C3B	2.49	129.47	124.80
14	GA	1138	CLA	C1-O2A-CGA	2.49	123.34	116.54
14	BA	1212	CLA	CMA-C3A-C4A	2.49	118.46	111.77
18	RA	4020	BCR	C23-C24-C25	-2.49	120.31	127.28

Continued on next page...

PRELIMINARY VALIDATION REPORT

Continued from previous page...

Mol	Chain	Res	Type	Atoms	Z	Observed(°)	Ideal(°)
18	aA	4008	BCR	C35-C13-C12	2.49	122.04	118.09
14	bA	1216	CLA	CHC-C1C-C2C	-2.49	119.85	126.71
14	HA	1203	CLA	C4-C3-C2	-2.49	117.21	123.68
14	LA	1502	CLA	O1D-CGD-CBD	-2.49	119.33	124.48
14	aA	1110	CLA	CAA-C2A-C3A	-2.48	105.97	112.78
18	aA	4011	BCR	C28-C27-C26	-2.48	109.67	113.99
14	aA	1132	CLA	O1D-CGD-CBD	-2.48	119.33	124.48
14	AA	1110	CLA	CAA-C2A-C3A	-2.48	105.98	112.78
14	bA	1201	CLA	CHB-C4A-NA	2.48	127.94	124.51
14	AA	1137	CLA	CMC-C2C-C1C	2.48	128.81	125.03
14	AA	1012	CLA	CMC-C2C-C1C	2.48	128.81	125.03
14	GA	1012	CLA	CMC-C2C-C1C	2.48	128.81	125.03
14	GA	1012	CLA	CHA-C1A-NA	-2.48	120.72	126.40
14	HA	1210	CLA	OBD-CAD-C3D	-2.48	123.64	128.04
14	aA	1012	CLA	C6-C5-C3	-2.48	107.05	113.01
14	bA	1236	CLA	O1D-CGD-CBD	-2.48	119.34	124.48
18	iA	4020	BCR	C23-C24-C25	-2.48	120.33	127.28
14	aA	1012	CLA	CHA-C1A-NA	-2.48	120.72	126.40
18	HA	4013	BCR	C2-C1-C6	2.48	114.32	110.48
14	HA	1236	CLA	C3C-C4C-NC	2.48	113.35	110.57
14	bA	1232	CLA	C3C-C4C-NC	2.48	113.35	110.57
14	lA	1502	CLA	O1D-CGD-CBD	-2.48	119.34	124.48
14	bA	1203	CLA	C4-C3-C2	-2.48	117.24	123.68
14	BA	1236	CLA	C3C-C4C-NC	2.48	113.35	110.57
14	aA	1127	CLA	CMC-C2C-C1C	2.48	128.80	125.03
14	GA	1110	CLA	O1D-CGD-CBD	-2.48	119.35	124.48
14	aA	1116	CLA	C4-C3-C5	2.48	119.55	115.29
18	GA	4008	BCR	C35-C13-C12	2.48	122.02	118.09
14	AA	1127	CLA	CMC-C2C-C1C	2.48	128.80	125.03
14	bA	1212	CLA	CMA-C3A-C4A	2.48	118.43	111.77
14	bA	1021	CLA	CHD-C4C-C3C	-2.48	121.26	124.87
18	HA	4004	BCR	C27-C26-C25	-2.48	119.13	122.74
14	BA	1212	CLA	O1D-CGD-CBD	-2.48	119.35	124.48
13	aA	1011	CL0	C3C-C4C-NC	2.48	113.35	110.57
14	HA	1210	CLA	C4C-C3C-C2C	-2.47	103.31	106.89
18	bA	4012	BCR	C38-C26-C27	2.47	118.20	113.57
14	AA	1110	CLA	O1D-CGD-CBD	-2.47	119.35	124.48
14	HA	1212	CLA	CMA-C3A-C4A	2.47	118.42	111.77
14	GA	1138	CLA	CBA-CAA-C2A	2.47	121.22	113.85
14	GA	1110	CLA	CAA-C2A-C3A	-2.47	106.00	112.78
14	aA	1114	CLA	CHC-C1C-C2C	-2.47	119.89	126.71
14	bA	1212	CLA	O1D-CGD-CBD	-2.47	119.35	124.48

Continued on next page...

PRELIMINARY VALIDATION REPORT

Continued from previous page...

Mol	Chain	Res	Type	Atoms	Z	Observed(°)	Ideal(°)
14	HA	1231	CLA	C4-C3-C5	2.47	119.55	115.29
14	bA	1210	CLA	CMA-C3A-C4A	2.47	118.42	111.77
14	HA	1209	CLA	CMA-C3A-C4A	2.47	118.42	111.77
14	BA	1209	CLA	CMA-C3A-C4A	2.47	118.42	111.77
18	BA	4013	BCR	C34-C9-C8	2.47	122.01	118.09
14	GA	1127	CLA	CMC-C2C-C1C	2.47	128.79	125.03
18	HA	4005	BCR	C23-C22-C21	2.47	122.73	118.94
14	HA	1216	CLA	CHB-C4A-NA	2.47	127.93	124.51
14	bA	1209	CLA	CMA-C3A-C4A	2.47	118.41	111.77
14	HA	1210	CLA	CMA-C3A-C4A	2.47	118.41	111.77
14	AA	1129	CLA	OBD-CAD-C3D	-2.47	123.66	128.04
14	aA	1116	CLA	C3C-C4C-NC	2.47	113.34	110.57
14	bA	1220	CLA	C3C-C4C-NC	2.47	113.34	110.57
14	aA	1138	CLA	CBA-CAA-C2A	2.47	121.20	113.85
13	AA	1011	CL0	CMC-C2C-C1C	2.47	128.79	125.03
14	AA	1132	CLA	O1D-CGD-CBD	-2.47	119.36	124.48
14	HA	1212	CLA	O1D-CGD-CBD	-2.47	119.36	124.48
14	BA	1231	CLA	C4-C3-C5	2.47	119.54	115.29
14	AA	1012	CLA	C6-C5-C3	-2.47	107.08	113.01
14	BA	1211	CLA	C4-C3-C5	2.47	119.54	115.29
14	AA	1138	CLA	CBA-CAA-C2A	2.47	121.19	113.85
18	IA	4018	BCR	C1-C6-C5	-2.47	119.14	122.59
14	bA	1216	CLA	CHB-C4A-NA	2.47	127.92	124.51
14	BA	1210	CLA	C4C-C3C-C2C	-2.47	103.32	106.89
14	HA	1235	CLA	C4-C3-C2	-2.46	117.27	123.68
14	BA	1023	CLA	C1C-C2C-C3C	-2.46	104.34	106.95
14	GA	1114	CLA	CHC-C1C-C2C	-2.46	119.92	126.71
14	AA	1102	CLA	CMC-C2C-C1C	2.46	128.78	125.03
14	GA	1118	CLA	CMB-C2B-C3B	2.46	129.42	124.80
14	BA	1213	CLA	CAC-C3C-C4C	2.46	128.04	124.82
18	BA	4005	BCR	C23-C22-C21	2.46	122.72	118.94
14	aA	1110	CLA	O1D-CGD-CBD	-2.46	119.38	124.48
14	BA	1227	CLA	O2D-CGD-O1D	-2.46	118.97	123.83
18	BA	4004	BCR	C27-C26-C25	-2.46	119.15	122.74
14	HA	1208	CLA	C4-C3-C5	2.46	119.53	115.29
14	AA	1116	CLA	C4-C3-C5	2.46	119.52	115.29
14	bA	1236	CLA	C3C-C4C-NC	2.46	113.33	110.57
14	aA	1102	CLA	CMC-C2C-C1C	2.46	128.77	125.03
18	bA	4004	BCR	C27-C26-C25	-2.46	119.15	122.74
14	BA	1236	CLA	O1D-CGD-CBD	-2.46	119.38	124.48
18	HA	4012	BCR	C38-C26-C27	2.46	118.17	113.57
18	BA	4010	BCR	C40-C30-C25	2.46	114.28	110.30

Continued on next page...

PRELIMINARY VALIDATION REPORT

Continued from previous page...

Mol	Chain	Res	Type	Atoms	Z	Observed(^{o})	Ideal(^{o})
14	BA	1208	CLA	C3C-C4C-NC	2.46	113.33	110.57
14	GA	1132	CLA	O1D-CGD-CBD	-2.46	119.39	124.48
14	BA	1021	CLA	CHD-C4C-C3C	-2.46	121.28	124.87
14	AA	1114	CLA	CHC-C1C-C2C	-2.46	119.94	126.71
14	GA	1102	CLA	CMC-C2C-C1C	2.46	128.77	125.03
14	GA	1105	CLA	C3C-C4C-NC	2.46	113.33	110.57
14	aA	1106	CLA	CMB-C2B-C3B	2.45	129.40	124.80
14	GA	1116	CLA	C4-C3-C5	2.45	119.52	115.29
14	bA	1210	CLA	C4C-C3C-C2C	-2.45	103.34	106.89
18	bA	4010	BCR	C40-C30-C25	2.45	114.27	110.30
18	GA	4003	BCR	C38-C26-C27	2.45	118.16	113.57
14	AA	1105	CLA	C3C-C4C-NC	2.45	113.32	110.57
14	HA	1216	CLA	CHC-C1C-C2C	-2.45	119.95	126.71
14	bA	1227	CLA	O2D-CGD-O1D	-2.45	118.99	123.83
14	HA	1225	CLA	C4C-C3C-C2C	-2.45	103.34	106.89
13	AA	1011	CL0	C3C-C4C-NC	2.45	113.32	110.57
13	aA	1011	CL0	CMC-C2C-C1C	2.45	128.76	125.03
14	GA	1012	CLA	C6-C5-C3	-2.45	107.12	113.01
18	GA	4007	BCR	C23-C24-C25	-2.45	120.42	127.28
14	aA	1117	CLA	CAA-C2A-C3A	-2.45	106.07	112.78
14	GA	1106	CLA	CMB-C2B-C3B	2.45	129.39	124.80
14	aA	1138	CLA	CED-O2D-CGD	2.45	121.54	115.95
18	bA	4013	BCR	C34-C9-C8	2.45	121.97	118.09
14	GA	1133	CLA	C4-C3-C5	2.45	119.50	115.29
18	UA	4019	BCR	C15-C14-C13	-2.45	123.82	127.31
18	aA	4003	BCR	C38-C26-C27	2.45	118.15	113.57
14	aA	1129	CLA	OBD-CAD-C3D	-2.44	123.71	128.04
18	RA	4018	BCR	C1-C6-C5	-2.44	119.17	122.59
14	HA	1209	CLA	C3C-C4C-NC	2.44	113.31	110.57
18	BA	4016	BCR	C34-C9-C10	-2.44	119.50	122.92
14	GA	1129	CLA	OBD-CAD-C3D	-2.44	123.71	128.04
14	AA	1106	CLA	CMB-C2B-C3B	2.44	129.38	124.80
14	GA	1117	CLA	CAA-C2A-C3A	-2.44	106.09	112.78
18	AA	4007	BCR	C23-C24-C25	-2.44	120.44	127.28
14	aA	1102	CLA	CHB-C4A-NA	2.44	127.89	124.51
14	AA	1117	CLA	CAA-C2A-C3A	-2.44	106.09	112.78
14	BA	1209	CLA	C3C-C4C-NC	2.44	113.31	110.57
14	bA	1213	CLA	CAC-C3C-C4C	2.44	128.01	124.82
14	bA	1235	CLA	C4-C3-C2	-2.44	117.33	123.68
18	BA	4012	BCR	C38-C26-C27	2.44	118.14	113.57
14	GA	1107	CLA	O2D-CGD-O1D	-2.44	119.01	123.83
14	bA	1208	CLA	C3C-C4C-NC	2.44	113.31	110.57

Continued on next page...

PRELIMINARY VALIDATION REPORT

Continued from previous page...

Mol	Chain	Res	Type	Atoms	Z	Observed(^{o})	Ideal(^{o})
14	HA	1236	CLA	O1D-CGD-CBD	-2.44	119.42	124.48
14	AA	1107	CLA	O2D-CGD-O1D	-2.44	119.01	123.83
14	HA	1021	CLA	CHD-C4C-C3C	-2.44	121.31	124.87
14	HA	1213	CLA	CAC-C3C-C4C	2.44	128.01	124.82
18	AA	4003	BCR	C38-C26-C27	2.44	118.14	113.57
14	BA	1235	CLA	C4-C3-C2	-2.44	117.34	123.68
13	GA	1011	CL0	CMC-C2C-C1C	2.44	128.74	125.03
18	HA	4016	BCR	C34-C9-C10	-2.44	119.51	122.92
14	bA	1209	CLA	C3C-C4C-NC	2.44	113.31	110.57
14	BA	1201	CLA	CHB-C4A-NA	2.44	127.88	124.51
14	BA	1216	CLA	CHC-C1C-C2C	-2.44	119.99	126.71
18	bA	4005	BCR	C23-C22-C21	2.44	122.68	118.94
14	bA	1239	CLA	CHC-C1C-C2C	-2.43	120.00	126.71
14	GA	1138	CLA	CED-O2D-CGD	2.43	121.51	115.95
14	aA	1107	CLA	O2D-CGD-O1D	-2.43	119.02	123.83
14	aA	1112	CLA	CAC-C3C-C4C	2.43	128.00	124.82
14	AA	1118	CLA	CMA-C3A-C4A	2.43	118.31	111.77
14	aA	1118	CLA	CMA-C3A-C4A	2.43	118.31	111.77
14	bA	1208	CLA	C4-C3-C5	2.43	119.48	115.29
18	HA	4010	BCR	C40-C30-C25	2.43	114.24	110.30
14	AA	1133	CLA	C4-C3-C5	2.43	119.48	115.29
14	HA	1239	CLA	CHC-C1C-C2C	-2.43	120.01	126.71
14	AA	1127	CLA	O2D-CGD-O1D	-2.43	119.03	123.83
18	aA	4007	BCR	C23-C24-C25	-2.43	120.47	127.28
14	HA	1208	CLA	C3C-C4C-NC	2.43	113.30	110.57
14	HA	1227	CLA	O2D-CGD-O1D	-2.43	119.03	123.83
18	HA	4013	BCR	C34-C9-C8	2.43	121.94	118.09
18	bA	4016	BCR	C34-C9-C10	-2.43	119.52	122.92
14	BA	1208	CLA	C4-C3-C5	2.43	119.47	115.29
14	GA	1119	CLA	C1-C2-C3	-2.43	121.84	126.04
14	GA	1127	CLA	O2D-CGD-O1D	-2.43	119.03	123.83
14	aA	1133	CLA	C4-C3-C5	2.43	119.47	115.29
13	GA	1011	CL0	C3C-C4C-NC	2.43	113.29	110.57
14	aA	1122	CLA	CAA-CBA-CGA	-2.43	106.11	113.26
14	GA	1122	CLA	CAA-CBA-CGA	-2.43	106.11	113.26
14	GA	1127	CLA	C6-C5-C3	-2.43	107.18	113.01
14	GA	1125	CLA	CMC-C2C-C3C	2.43	132.72	126.11
14	AA	1138	CLA	CED-O2D-CGD	2.43	121.49	115.95
14	BA	1021	CLA	CBC-CAC-C3C	-2.43	105.69	112.43
14	AA	1125	CLA	CMC-C2C-C3C	2.43	132.72	126.11
14	1A	1502	CLA	C3C-C4C-NC	2.43	113.29	110.57
14	bA	1021	CLA	CBC-CAC-C3C	-2.43	105.70	112.43

Continued on next page...

PRELIMINARY VALIDATION REPORT

Continued from previous page...

Mol	Chain	Res	Type	Atoms	Z	Observed(°)	Ideal(°)
14	bA	1023	CLA	CAC-C3C-C2C	-2.42	123.39	127.53
14	BA	1225	CLA	C4C-C3C-C2C	-2.42	103.38	106.89
14	AA	1013	CLA	CHD-C4C-C3C	-2.42	121.34	124.87
14	bA	1225	CLA	C4C-C3C-C2C	-2.42	103.38	106.89
18	lA	4019	BCR	C15-C14-C13	-2.42	123.85	127.31
14	aA	1125	CLA	CMC-C2C-C3C	2.42	132.71	126.11
14	aA	1127	CLA	C6-C5-C3	-2.42	107.19	113.01
18	HA	4005	BCR	C15-C14-C13	-2.42	123.86	127.31
14	AA	1122	CLA	CAA-CBA-CGA	-2.42	106.12	113.26
14	AA	1139	CLA	O1D-CGD-CBD	-2.42	119.46	124.48
14	GA	1139	CLA	CMA-C3A-C4A	2.42	118.28	111.77
18	GA	4003	BCR	C37-C22-C21	-2.42	119.53	122.92
14	GA	1112	CLA	CAC-C3C-C4C	2.42	127.98	124.82
14	BA	1216	CLA	CHB-C4A-NA	2.42	127.86	124.51
18	LA	4019	BCR	C15-C14-C13	-2.42	123.86	127.31
14	BA	1023	CLA	CAC-C3C-C2C	-2.42	123.40	127.53
14	BA	1239	CLA	CHC-C1C-C2C	-2.42	120.05	126.71
14	GA	1127	CLA	C11-C10-C8	-2.42	108.23	115.77
14	GA	1118	CLA	CMA-C3A-C4A	2.42	118.27	111.77
14	HA	1021	CLA	CBC-CAC-C3C	-2.42	105.72	112.43
18	HA	4005	BCR	C33-C5-C4	2.42	118.09	113.57
18	GA	4008	BCR	C37-C22-C21	-2.42	119.54	122.92
18	aA	4008	BCR	C37-C22-C21	-2.42	119.54	122.92
18	bA	4005	BCR	C33-C5-C4	2.41	118.09	113.57
14	AA	1119	CLA	C1-C2-C3	-2.41	121.87	126.04
14	AA	1112	CLA	CAC-C3C-C4C	2.41	127.97	124.82
14	AA	1127	CLA	C6-C5-C3	-2.41	107.21	113.01
14	aA	1139	CLA	O1D-CGD-CBD	-2.41	119.48	124.48
14	aA	1119	CLA	C1-C2-C3	-2.41	121.87	126.04
14	GA	1122	CLA	C4C-C3C-C2C	-2.41	103.40	106.89
14	HA	1232	CLA	CMC-C2C-C1C	2.41	128.70	125.03
14	aA	1138	CLA	O1D-CGD-CBD	-2.41	119.49	124.48
14	XA	1701	CLA	CMC-C2C-C1C	2.41	128.70	125.03
14	HA	1023	CLA	CAC-C3C-C2C	-2.41	123.42	127.53
14	AA	1102	CLA	CHB-C4A-NA	2.41	127.84	124.51
18	VA	4021	BCR	C8-C9-C10	2.41	122.64	118.94
18	AA	4008	BCR	C37-C22-C21	-2.41	119.55	122.92
14	xA	1701	CLA	CMC-C2C-C1C	2.41	128.69	125.03
14	AA	1138	CLA	O1D-CGD-CBD	-2.41	119.49	124.48
18	aA	4003	BCR	C37-C22-C21	-2.41	119.55	122.92
14	aA	1127	CLA	C11-C10-C8	-2.41	108.26	115.77
14	aA	1139	CLA	CMA-C3A-C4A	2.41	118.24	111.77

Continued on next page...

PRELIMINARY VALIDATION REPORT

Continued from previous page...

Mol	Chain	Res	Type	Atoms	Z	Observed(^{o})	Ideal(^{o})
14	AA	1127	CLA	C11-C10-C8	-2.41	108.26	115.77
18	mA	4021	BCR	C8-C9-C10	2.40	122.63	118.94
14	GA	1013	CLA	CHD-C4C-C3C	-2.40	121.36	124.87
18	AA	4003	BCR	C37-C22-C21	-2.40	119.56	122.92
14	aA	1127	CLA	O2D-CGD-O1D	-2.40	119.08	123.83
14	BA	1226	CLA	CAA-CBA-CGA	-2.40	106.17	113.26
14	BA	1215	CLA	CMA-C3A-C4A	2.40	118.23	111.77
14	GA	1138	CLA	O1D-CGD-CBD	-2.40	119.50	124.48
18	LA	4022	BCR	C34-C9-C10	-2.40	119.56	122.92
14	HA	1219	CLA	CAC-C3C-C4C	2.40	127.96	124.82
14	GA	1102	CLA	CHB-C4A-NA	2.40	127.83	124.51
14	aA	1013	CLA	CHD-C4C-C3C	-2.40	121.37	124.87
14	aA	1122	CLA	C4C-C3C-C2C	-2.40	103.42	106.89
18	BA	4005	BCR	C33-C5-C4	2.40	118.06	113.57
14	HA	1226	CLA	CAA-CBA-CGA	-2.40	106.19	113.26
18	LA	4022	BCR	C3-C4-C5	-2.40	109.82	113.99
14	AA	1139	CLA	CMA-C3A-C4A	2.40	118.22	111.77
14	aA	1012	CLA	C11-C10-C8	-2.40	108.30	115.77
14	bA	1215	CLA	CMA-C3A-C4A	2.40	118.21	111.77
14	HA	1220	CLA	CED-O2D-CGD	2.39	121.42	115.95
14	GA	1012	CLA	C11-C10-C8	-2.39	108.30	115.77
14	bA	1223	CLA	C1-C2-C3	-2.39	121.90	126.04
14	BA	1223	CLA	C4-C3-C5	2.39	119.41	115.29
18	BA	4014	BCR	C29-C28-C27	-2.39	105.98	111.37
14	BA	1210	CLA	C4-C3-C2	-2.39	117.47	123.68
14	bA	1226	CLA	CAA-CBA-CGA	-2.39	106.22	113.26
18	bA	4005	BCR	C15-C14-C13	-2.39	123.90	127.31
14	HA	1215	CLA	CMB-C2B-C1B	-2.39	124.79	128.46
14	BA	1022	CLA	C4-C3-C5	2.39	119.40	115.29
14	0	1012	CLA	C4-C3-C5	2.39	119.40	115.29
18	UA	4022	BCR	C3-C4-C5	-2.39	109.84	113.99
14	AA	1012	CLA	C11-C10-C8	-2.39	108.32	115.77
14	BA	1220	CLA	CED-O2D-CGD	2.39	121.40	115.95
14	HA	1215	CLA	CMA-C3A-C4A	2.39	118.19	111.77
14	AA	1122	CLA	C4C-C3C-C2C	-2.38	103.44	106.89
14	GA	1139	CLA	O1D-CGD-CBD	-2.38	119.54	124.48
14	LA	1502	CLA	C3C-C4C-NC	2.38	113.24	110.57
18	HA	4014	BCR	C29-C28-C27	-2.38	106.00	111.37
18	BA	4005	BCR	C15-C14-C13	-2.38	123.91	127.31
14	bA	1238	CLA	C4C-C3C-C2C	-2.38	103.44	106.89
14	GA	1109	CLA	CMC-C2C-C1C	2.38	128.66	125.03
14	HA	1210	CLA	C4-C3-C2	-2.38	117.49	123.68

Continued on next page...

PRELIMINARY VALIDATION REPORT

Continued from previous page...

Mol	Chain	Res	Type	Atoms	Z	Observed(^{o})	Ideal(^{o})
18	lA	4022	BCR	C29-C30-C25	2.38	114.17	110.48
18	aA	4003	BCR	C33-C5-C4	2.38	118.03	113.57
14	BA	1232	CLA	CMC-C2C-C1C	2.38	128.65	125.03
14	UA	1502	CLA	C3C-C4C-NC	2.38	113.24	110.57
14	bA	1022	CLA	C4-C3-C5	2.38	119.39	115.29
14	BA	1217	CLA	CMB-C2B-C3B	2.38	129.26	124.80
14	AA	1012	CLA	C4-C3-C5	2.38	119.39	115.29
14	bA	1210	CLA	C4-C3-C2	-2.38	117.49	123.68
18	MA	4021	BCR	C8-C9-C10	2.38	122.59	118.94
14	bA	1215	CLA	CMB-C2B-C1B	-2.38	124.81	128.46
14	AA	1109	CLA	CMC-C2C-C1C	2.38	128.65	125.03
18	bA	4014	BCR	C29-C28-C27	-2.38	106.01	111.37
18	LA	4022	BCR	C29-C30-C25	2.38	114.16	110.48
18	AA	4003	BCR	C33-C5-C4	2.38	118.02	113.57
14	bA	1205	CLA	O1D-CGD-CBD	-2.38	119.55	124.48
14	bA	1227	CLA	CMC-C2C-C1C	2.38	128.65	125.03
14	GA	1012	CLA	C4-C3-C5	2.38	119.38	115.29
14	bA	1214	CLA	OBD-CAD-CBD	-2.38	122.42	125.91
18	lA	4022	BCR	C3-C4-C5	-2.38	109.86	113.99
14	bA	1222	CLA	C1-C2-C3	-2.38	121.93	126.04
18	GA	4003	BCR	C33-C5-C4	2.38	118.02	113.57
14	HA	1205	CLA	O1D-CGD-CBD	-2.37	119.56	124.48
14	bA	1223	CLA	C4-C3-C5	2.37	119.38	115.29
14	BA	1227	CLA	CMC-C2C-C1C	2.37	128.64	125.03
18	lA	4022	BCR	C34-C9-C10	-2.37	119.60	122.92
14	bA	1220	CLA	CED-O2D-CGD	2.37	121.37	115.95
18	aA	4001	BCR	C29-C30-C25	2.37	114.15	110.48
14	BA	1215	CLA	CMB-C2B-C1B	-2.37	124.82	128.46
18	UA	4022	BCR	C34-C9-C10	-2.37	119.60	122.92
14	BA	1219	CLA	O1D-CGD-CBD	-2.37	119.57	124.48
14	HA	1219	CLA	O1D-CGD-CBD	-2.37	119.57	124.48
14	bA	1219	CLA	CAC-C3C-C4C	2.37	127.92	124.82
14	AA	1110	CLA	CMA-C3A-C4A	2.37	118.14	111.77
14	GA	1131	CLA	CAA-C2A-C3A	-2.37	106.29	112.78
14	bA	1219	CLA	O1D-CGD-CBD	-2.37	119.57	124.48
18	LA	4019	BCR	C32-C1-C6	-2.37	106.47	110.30
14	HA	1217	CLA	CMB-C2B-C3B	2.37	129.24	124.80
14	HA	1223	CLA	C4-C3-C5	2.37	119.37	115.29
14	aA	1108	CLA	CMB-C2B-C3B	2.37	129.24	124.80
18	bA	4013	BCR	C31-C1-C6	-2.37	106.47	110.30
14	aA	1109	CLA	CMC-C2C-C1C	2.37	128.63	125.03
14	bA	1232	CLA	CMC-C2C-C1C	2.37	128.63	125.03

Continued on next page...

PRELIMINARY VALIDATION REPORT

Continued from previous page...

Mol	Chain	Res	Type	Atoms	Z	Observed(°)	Ideal(°)
14	HA	1211	CLA	C3C-C4C-NC	2.37	113.23	110.57
21	AA	6001	LMT	O5'-C5'-C6'	2.37	112.33	106.43
14	HA	1226	CLA	CAC-C3C-C4C	2.37	127.92	124.82
14	bA	1207	CLA	CMA-C3A-C4A	2.37	118.14	111.77
18	AA	4007	BCR	C34-C9-C10	-2.37	119.61	122.92
14	BA	1223	CLA	C1-C2-C3	-2.37	121.95	126.04
18	UA	4022	BCR	C29-C30-C25	2.37	114.14	110.48
14	HA	1227	CLA	CMC-C2C-C1C	2.37	128.63	125.03
14	GA	1110	CLA	CMA-C3A-C4A	2.37	118.13	111.77
14	BA	1222	CLA	C1-C2-C3	-2.37	121.95	126.04
14	aA	1110	CLA	CMA-C3A-C4A	2.37	118.13	111.77
14	GA	1116	CLA	CAC-C3C-C4C	2.37	127.91	124.82
14	BA	1219	CLA	CAC-C3C-C4C	2.37	127.91	124.82
14	AA	1103	CLA	CHB-C4A-NA	2.37	127.78	124.51
14	HA	1223	CLA	C1-C2-C3	-2.36	121.95	126.04
14	AA	1115	CLA	CMA-C3A-C4A	2.36	118.13	111.77
14	WA	1701	CLA	CMC-C2C-C1C	2.36	128.63	125.03
14	BA	1228	CLA	CHC-C1C-C2C	-2.36	120.19	126.71
18	HA	4004	BCR	C33-C5-C4	2.36	118.00	113.57
14	lA	1503	CLA	C3C-C4C-NC	2.36	113.22	110.57
14	aA	1131	CLA	CAA-C2A-C3A	-2.36	106.31	112.78
14	BA	1216	CLA	CAC-C3C-C4C	2.36	127.91	124.82
14	xA	1701	CLA	O2D-CGD-O1D	-2.36	119.17	123.83
18	BA	4005	BCR	C34-C9-C8	2.36	121.84	118.09
21	GA	6001	LMT	O5'-C5'-C6'	2.36	112.32	106.43
14	aA	1012	CLA	C4-C3-C5	2.36	119.35	115.29
18	GA	4011	BCR	C4-C5-C6	-2.36	119.29	122.74
14	HA	1238	CLA	C4C-C3C-C2C	-2.36	103.47	106.89
14	AA	1013	CLA	CAA-C2A-C1A	-2.36	104.24	111.97
14	HA	1228	CLA	CHC-C1C-C2C	-2.36	120.21	126.71
14	GA	1103	CLA	CHB-C4A-NA	2.36	127.77	124.51
14	bA	1211	CLA	C3C-C4C-NC	2.36	113.22	110.57
14	BA	1207	CLA	CMA-C3A-C4A	2.36	118.11	111.77
18	bA	4009	BCR	C8-C7-C6	-2.36	120.68	127.28
14	AA	1116	CLA	CAC-C3C-C4C	2.36	127.90	124.82
18	BA	4004	BCR	C33-C5-C4	2.36	117.98	113.57
14	WA	1701	CLA	O2D-CGD-O1D	-2.36	119.18	123.83
14	bA	1219	CLA	O2D-CGD-O1D	-2.36	119.18	123.83
18	BA	4009	BCR	C8-C7-C6	-2.36	120.68	127.28
18	MA	4021	BCR	C38-C26-C25	-2.35	121.89	124.51
18	bA	4004	BCR	C33-C5-C4	2.35	117.98	113.57
14	HA	1214	CLA	OBD-CAD-CBD	-2.35	122.45	125.91

Continued on next page...

PRELIMINARY VALIDATION REPORT

Continued from previous page...

Mol	Chain	Res	Type	Atoms	Z	Observed(°)	Ideal(°)
14	AA	1131	CLA	CAA-C2A-C3A	-2.35	106.33	112.78
18	BA	4013	BCR	C31-C1-C6	-2.35	106.50	110.30
14	aA	1115	CLA	CMA-C3A-C4A	2.35	118.10	111.77
14	HA	1222	CLA	C1-C2-C3	-2.35	121.97	126.04
14	aA	1013	CLA	CAA-C2A-C1A	-2.35	104.26	111.97
14	BA	1214	CLA	OBD-CAD-CBD	-2.35	122.46	125.91
18	lA	4019	BCR	C3-C4-C5	-2.35	109.90	113.99
14	GA	1108	CLA	CMB-C2B-C3B	2.35	129.21	124.80
18	aA	4007	BCR	C34-C9-C10	-2.35	119.63	122.92
14	GA	1013	CLA	CAA-C2A-C1A	-2.35	104.27	111.97
18	HA	4005	BCR	C34-C9-C8	2.35	121.82	118.09
18	bA	4005	BCR	C34-C9-C8	2.35	121.82	118.09
14	bA	1228	CLA	CHC-C1C-C2C	-2.35	120.23	126.71
14	bA	1217	CLA	CMB-C2B-C3B	2.35	129.21	124.80
14	BA	1215	CLA	CAA-C2A-C3A	-2.35	106.34	112.78
18	HA	4012	BCR	C32-C1-C6	-2.35	106.50	110.30
14	HA	1207	CLA	CMA-C3A-C4A	2.35	118.09	111.77
14	GA	1115	CLA	CMA-C3A-C4A	2.35	118.09	111.77
14	BA	1205	CLA	O1D-CGD-CBD	-2.35	119.61	124.48
14	aA	1133	CLA	C6-C5-C3	-2.35	107.36	113.01
18	BA	4017	BCR	C33-C5-C6	-2.35	121.89	124.51
14	BA	1201	CLA	O1D-CGD-CBD	-2.35	119.61	124.48
14	HA	1238	CLA	C11-C12-C13	-2.35	108.44	115.77
21	GA	6002	LMT	C1'-O5'-C5'	-2.35	109.07	113.70
14	aA	1119	CLA	CHB-C4A-NA	2.35	127.76	124.51
14	bA	1215	CLA	CAA-C2A-C3A	-2.35	106.35	112.78
14	BA	1238	CLA	C4C-C3C-C2C	-2.35	103.49	106.89
18	HA	4013	BCR	C31-C1-C6	-2.35	106.51	110.30
21	aA	6001	LMT	O5'-C5'-C6'	2.35	112.28	106.43
14	AA	1108	CLA	CMB-C2B-C3B	2.35	129.20	124.80
14	GA	1133	CLA	C6-C5-C3	-2.35	107.37	113.01
14	BA	1216	CLA	C1-C2-C3	-2.35	121.98	126.04
18	GA	4007	BCR	C34-C9-C10	-2.35	119.64	122.92
14	bA	1238	CLA	C11-C12-C13	-2.35	108.45	115.77
14	HA	1216	CLA	CAC-C3C-C4C	2.35	127.89	124.82
18	RA	4020	BCR	C37-C22-C23	2.35	121.81	118.09
18	iA	4020	BCR	C37-C22-C23	2.34	121.81	118.09
14	AA	1133	CLA	C6-C5-C3	-2.34	107.37	113.01
18	GA	4001	BCR	C29-C30-C25	2.34	114.11	110.48
14	BA	1238	CLA	C11-C12-C13	-2.34	108.46	115.77
18	LA	4019	BCR	C3-C4-C5	-2.34	109.91	113.99
14	bA	1201	CLA	O1D-CGD-CBD	-2.34	119.62	124.48

Continued on next page...

PRELIMINARY VALIDATION REPORT

Continued from previous page...

Mol	Chain	Res	Type	Atoms	Z	Observed(°)	Ideal(°)
14	XA	1701	CLA	O2D-CGD-O1D	-2.34	119.20	123.83
14	AA	1119	CLA	CHB-C4A-NA	2.34	127.75	124.51
18	IA	4020	BCR	C37-C22-C23	2.34	121.80	118.09
18	aA	4011	BCR	C4-C5-C6	-2.34	119.32	122.74
18	HA	4009	BCR	C8-C7-C6	-2.34	120.73	127.28
18	UA	4019	BCR	C3-C4-C5	-2.34	109.92	113.99
14	bA	1238	CLA	C16-C15-C13	-2.34	108.47	115.77
14	HA	1215	CLA	CAA-C2A-C3A	-2.34	106.37	112.78
14	HA	1201	CLA	O1D-CGD-CBD	-2.34	119.63	124.48
18	mA	4021	BCR	C38-C26-C25	-2.34	121.91	124.51
18	HA	4017	BCR	C33-C5-C6	-2.34	121.91	124.51
14	aA	1103	CLA	CHB-C4A-NA	2.34	127.74	124.51
18	BA	4012	BCR	C32-C1-C6	-2.34	106.52	110.30
14	AA	1104	CLA	CMC-C2C-C1C	2.34	128.59	125.03
14	aA	1116	CLA	CAC-C3C-C4C	2.34	127.87	124.82
14	BA	1211	CLA	C3C-C4C-NC	2.33	113.19	110.57
18	UA	4019	BCR	C32-C1-C6	-2.33	106.53	110.30
21	aA	6002	LMT	C1'-O5'-C5'	-2.33	109.10	113.70
14	bA	1225	CLA	CMB-C2B-C1B	-2.33	124.88	128.46
14	GA	1102	CLA	CHA-C1A-NA	-2.33	121.06	126.40
14	BA	1226	CLA	CAC-C3C-C4C	2.33	127.86	124.82
14	BA	1219	CLA	O2D-CGD-O1D	-2.33	119.23	123.83
14	AA	1103	CLA	C3C-C4C-NC	2.33	113.18	110.57
14	HA	1216	CLA	C1-C2-C3	-2.33	122.02	126.04
14	AA	1102	CLA	CHA-C1A-NA	-2.33	121.07	126.40
14	bA	1221	CLA	CAC-C3C-C4C	2.33	127.86	124.82
14	bA	1226	CLA	CAC-C3C-C4C	2.33	127.86	124.82
18	AA	4011	BCR	C4-C5-C6	-2.33	119.34	122.74
14	HA	1225	CLA	CMB-C2B-C1B	-2.33	124.89	128.46
14	HA	1219	CLA	O2D-CGD-O1D	-2.33	119.23	123.83
14	GA	1103	CLA	C3C-C4C-NC	2.33	113.18	110.57
18	bA	4017	BCR	C33-C5-C6	-2.33	121.92	124.51
21	AA	6002	LMT	C1'-O5'-C5'	-2.33	109.11	113.70
18	bA	4012	BCR	C32-C1-C6	-2.33	106.54	110.30
14	bA	1216	CLA	C1-C2-C3	-2.33	122.02	126.04
14	GA	1132	CLA	C11-C10-C8	-2.33	108.51	115.77
14	aA	1103	CLA	C3C-C4C-NC	2.33	113.18	110.57
18	bA	4005	BCR	C3-C4-C5	-2.32	109.94	113.99
14	LA	1503	CLA	C3C-C4C-NC	2.32	113.18	110.57
14	HA	1225	CLA	CAC-C3C-C4C	2.32	127.86	124.82
14	aA	1131	CLA	C4-C3-C5	2.32	119.29	115.29
14	BA	1225	CLA	CAC-C3C-C4C	2.32	127.86	124.82

Continued on next page...

PRELIMINARY VALIDATION REPORT

Continued from previous page...

Mol	Chain	Res	Type	Atoms	Z	Observed(^{o})	Ideal(^{o})
14	aA	1104	CLA	CMC-C2C-C1C	2.32	128.56	125.03
18	lA	4019	BCR	C32-C1-C6	-2.32	106.55	110.30
18	AA	4002	BCR	C30-C25-C24	2.32	122.38	115.78
14	HA	1238	CLA	C16-C15-C13	-2.32	108.53	115.77
14	bA	1214	CLA	CMA-C3A-C4A	2.32	118.01	111.77
14	BA	1238	CLA	C16-C15-C13	-2.32	108.53	115.77
18	lA	4022	BCR	C1-C6-C5	-2.32	119.35	122.59
18	BA	4006	BCR	C35-C13-C12	2.32	121.77	118.09
14	aA	1102	CLA	CHA-C1A-NA	-2.32	121.09	126.40
14	GA	1128	CLA	CHC-C1C-C2C	-2.32	120.32	126.71
18	UA	4022	BCR	C1-C6-C5	-2.32	119.35	122.59
14	BA	1225	CLA	CMB-C2B-C1B	-2.32	124.90	128.46
14	HA	1229	CLA	C3C-C4C-NC	2.32	113.17	110.57
14	HA	1214	CLA	CMA-C3A-C4A	2.32	118.00	111.77
18	AA	4001	BCR	C29-C30-C25	2.32	114.06	110.48
14	aA	1128	CLA	CHC-C1C-C2C	-2.32	120.33	126.71
14	GA	1135	CLA	CMC-C2C-C1C	2.31	128.55	125.03
14	bA	1211	CLA	CAA-C2A-C3A	-2.31	106.44	112.78
14	HA	1239	CLA	CMA-C3A-C4A	2.31	117.99	111.77
14	aA	1118	CLA	C1-O2A-CGA	2.31	122.86	116.54
14	bA	1216	CLA	CAC-C3C-C4C	2.31	127.84	124.82
18	BA	4005	BCR	C3-C4-C5	-2.31	109.97	113.99
14	AA	1132	CLA	C11-C10-C8	-2.31	108.56	115.77
14	HA	1232	CLA	CAC-C3C-C4C	2.31	127.84	124.82
14	AA	1118	CLA	C1-O2A-CGA	2.31	122.86	116.54
14	BA	1207	CLA	CMB-C2B-C3B	2.31	129.13	124.80
14	AA	1104	CLA	CHB-C4A-NA	2.31	127.71	124.51
14	HA	1217	CLA	C4-C3-C5	2.31	119.27	115.29
18	VA	4021	BCR	C38-C26-C25	-2.31	121.94	124.51
14	GA	1119	CLA	CHB-C4A-NA	2.31	127.70	124.51
14	bA	1218	CLA	CAA-C2A-C3A	-2.31	106.46	112.78
14	BA	1217	CLA	C4-C3-C5	2.31	119.26	115.29
14	BA	1218	CLA	CAA-C2A-C3A	-2.31	106.46	112.78
18	GA	4002	BCR	C30-C25-C24	2.31	122.34	115.78
18	bA	4016	BCR	C3-C4-C5	-2.31	109.97	113.99
18	HA	4005	BCR	C3-C4-C5	-2.31	109.98	113.99
14	GA	1118	CLA	C1-O2A-CGA	2.31	122.84	116.54
14	BA	1239	CLA	CMA-C3A-C4A	2.31	117.97	111.77
18	GA	4011	BCR	C8-C9-C10	2.31	122.48	118.94
14	bA	1239	CLA	CMA-C3A-C4A	2.31	117.97	111.77
14	aA	1132	CLA	C11-C10-C8	-2.31	108.58	115.77
14	GA	1138	CLA	C3C-C4C-NC	2.31	113.16	110.57

Continued on next page...

PRELIMINARY VALIDATION REPORT

Continued from previous page...

Mol	Chain	Res	Type	Atoms	Z	Observed(°)	Ideal(°)
18	BA	4016	BCR	C37-C22-C21	-2.31	119.69	122.92
14	BA	1212	CLA	CAC-C3C-C4C	2.31	127.83	124.82
14	HA	1222	CLA	C11-C12-C13	-2.31	108.58	115.77
14	bA	1207	CLA	CMB-C2B-C3B	2.30	129.12	124.80
14	GA	1131	CLA	C4-C3-C5	2.30	119.26	115.29
18	bA	4006	BCR	C35-C13-C12	2.30	121.75	118.09
14	BA	1214	CLA	CMA-C3A-C4A	2.30	117.97	111.77
14	bA	1225	CLA	CAC-C3C-C4C	2.30	127.83	124.82
14	HA	1221	CLA	OBD-CAD-C3D	-2.30	123.96	128.04
14	HA	1204	CLA	CAC-C3C-C4C	2.30	127.83	124.82
18	BA	4016	BCR	C3-C4-C5	-2.30	109.98	113.99
14	AA	1131	CLA	C4-C3-C5	2.30	119.25	115.29
14	UA	1503	CLA	C3C-C4C-NC	2.30	113.15	110.57
14	bA	1214	CLA	CHC-C1C-C2C	-2.30	120.37	126.71
14	bA	1021	CLA	C11-C10-C8	-2.30	108.59	115.77
18	HA	4006	BCR	C35-C13-C12	2.30	121.74	118.09
14	aA	1106	CLA	CMC-C2C-C1C	2.30	128.53	125.03
14	BA	1221	CLA	CAC-C3C-C4C	2.30	127.83	124.82
14	AA	1128	CLA	CHC-C1C-C2C	-2.30	120.37	126.71
14	BA	1232	CLA	CAC-C3C-C4C	2.30	127.83	124.82
14	GA	1104	CLA	CMC-C2C-C1C	2.30	128.53	125.03
14	AA	1127	CLA	C4-C3-C5	2.30	119.25	115.29
14	aA	1103	CLA	CMC-C2C-C1C	2.30	128.53	125.03
14	bA	1214	CLA	CMC-C2C-C3C	2.30	132.37	126.11
18	LA	4022	BCR	C1-C6-C5	-2.30	119.38	122.59
18	HA	4016	BCR	C37-C22-C21	-2.30	119.71	122.92
14	bA	1217	CLA	C4-C3-C5	2.29	119.24	115.29
14	BA	1238	CLA	C1-C2-C3	-2.29	122.08	126.04
14	BA	1222	CLA	C11-C12-C13	-2.29	108.61	115.77
18	AA	4011	BCR	C8-C9-C10	2.29	122.46	118.94
14	HA	1218	CLA	CAA-C2A-C3A	-2.29	106.50	112.78
14	BA	1226	CLA	C1-O2A-CGA	2.29	122.81	116.54
14	BA	1211	CLA	CAA-C2A-C3A	-2.29	106.50	112.78
14	HA	1204	CLA	CMB-C2B-C3B	2.29	129.10	124.80
14	AA	1132	CLA	CMA-C3A-C4A	2.29	117.93	111.77
14	bA	1204	CLA	CAC-C3C-C4C	2.29	127.81	124.82
14	HA	1211	CLA	CAA-C2A-C3A	-2.29	106.51	112.78
14	bA	1232	CLA	CAC-C3C-C4C	2.29	127.81	124.82
14	bA	1222	CLA	C11-C12-C13	-2.29	108.63	115.77
14	HA	1207	CLA	C3C-C4C-NC	2.29	113.14	110.57
18	aA	4002	BCR	C30-C25-C24	2.29	122.29	115.78
14	BA	1214	CLA	CMC-C2C-C3C	2.29	132.35	126.11

Continued on next page...

PRELIMINARY VALIDATION REPORT

Continued from previous page...

Mol	Chain	Res	Type	Atoms	Z	Observed(°)	Ideal(°)
14	aA	1117	CLA	CAC-C3C-C4C	2.29	127.81	124.82
14	aA	1132	CLA	CMA-C3A-C4A	2.29	117.92	111.77
14	AA	1138	CLA	C3C-C4C-NC	2.29	113.14	110.57
14	HA	1220	CLA	CAC-C3C-C4C	2.29	127.81	124.82
14	BA	1204	CLA	CMB-C2B-C3B	2.29	129.09	124.80
14	HA	1214	CLA	CHC-C1C-C2C	-2.29	120.41	126.71
14	HA	1236	CLA	CMB-C2B-C3B	2.29	129.09	124.80
14	BA	1021	CLA	C11-C10-C8	-2.29	108.64	115.77
14	HA	1226	CLA	C1-O2A-CGA	2.29	122.79	116.54
14	aA	1104	CLA	CHB-C4A-NA	2.29	127.67	124.51
14	AA	1138	CLA	CAC-C3C-C4C	2.28	127.81	124.82
14	HA	1221	CLA	CAC-C3C-C4C	2.28	127.81	124.82
18	GA	4003	BCR	C4-C5-C6	-2.28	119.41	122.74
14	bA	1236	CLA	CMB-C2B-C3B	2.28	129.08	124.80
14	BA	1214	CLA	CHC-C1C-C2C	-2.28	120.42	126.71
14	HA	1207	CLA	CMB-C2B-C3B	2.28	129.08	124.80
14	bA	1201	CLA	CAA-C2A-C3A	-2.28	106.53	112.78
14	GA	1138	CLA	CMA-C3A-C4A	2.28	117.91	111.77
14	HA	1214	CLA	CMC-C2C-C3C	2.28	132.33	126.11
14	bA	1204	CLA	CMB-C2B-C3B	2.28	129.08	124.80
14	HA	1234	CLA	C4-C3-C2	-2.28	117.75	123.68
14	GA	1117	CLA	CAC-C3C-C4C	2.28	127.80	124.82
14	HA	1021	CLA	C11-C10-C8	-2.28	108.65	115.77
18	UA	4022	BCR	C35-C13-C12	2.28	121.71	118.09
14	AA	1107	CLA	CMA-C3A-C4A	2.28	117.91	111.77
14	GA	1106	CLA	CMC-C2C-C1C	2.28	128.50	125.03
14	bA	1207	CLA	C3C-C4C-NC	2.28	113.13	110.57
14	BA	1201	CLA	CAA-C2A-C3A	-2.28	106.53	112.78
14	bA	1226	CLA	C1-O2A-CGA	2.28	122.77	116.54
14	BA	1236	CLA	CMB-C2B-C3B	2.28	129.08	124.80
14	HA	1238	CLA	C1-C2-C3	-2.28	122.10	126.04
18	aA	4003	BCR	C4-C5-C6	-2.28	119.41	122.74
18	bA	4016	BCR	C37-C22-C21	-2.28	119.73	122.92
14	UA	1501	CLA	O1D-CGD-CBD	-2.28	119.75	124.48
14	HA	1235	CLA	O1D-CGD-CBD	-2.28	119.75	124.48
14	BA	1220	CLA	CAC-C3C-C4C	2.28	127.80	124.82
18	HA	4016	BCR	C3-C4-C5	-2.28	110.02	113.99
14	bA	1202	CLA	CMB-C2B-C3B	2.28	129.07	124.80
14	aA	1117	CLA	C1-C2-C3	-2.28	122.10	126.04
14	0	1012	CLA	C4C-C3C-C2C	-2.28	103.59	106.89
18	aA	4011	BCR	C8-C9-C10	2.28	122.44	118.94
14	GA	1104	CLA	CHB-C4A-NA	2.28	127.66	124.51

Continued on next page...

PRELIMINARY VALIDATION REPORT

Continued from previous page...

Mol	Chain	Res	Type	Atoms	Z	Observed(^{o})	Ideal(^{o})
14	bA	1235	CLA	O1D-CGD-CBD	-2.28	119.76	124.48
14	AA	1103	CLA	CMC-C2C-C1C	2.28	128.49	125.03
14	HA	1201	CLA	CAA-C2A-C3A	-2.28	106.55	112.78
14	AA	1106	CLA	CMC-C2C-C1C	2.27	128.49	125.03
14	bA	1223	CLA	CHA-C1A-NA	-2.27	121.19	126.40
14	aA	1138	CLA	CMA-C3A-C4A	2.27	117.88	111.77
14	GA	1132	CLA	CMA-C3A-C4A	2.27	117.88	111.77
14	AA	1135	CLA	CMC-C2C-C1C	2.27	128.49	125.03
14	LA	1501	CLA	O1D-CGD-CBD	-2.27	119.77	124.48
14	BA	1202	CLA	CMB-C2B-C3B	2.27	129.06	124.80
14	GA	1103	CLA	CMC-C2C-C1C	2.27	128.49	125.03
14	aA	1013	CLA	C1-O2A-CGA	2.27	122.74	116.54
14	GA	1138	CLA	CAC-C3C-C4C	2.27	127.79	124.82
14	aA	1127	CLA	C4-C3-C5	2.27	119.19	115.29
14	aA	1135	CLA	CMC-C2C-C1C	2.27	128.48	125.03
21	GA	6002	LMT	C1B-O1B-C4'	2.27	123.63	117.97
14	HA	1202	CLA	CMB-C2B-C3B	2.27	129.05	124.80
14	bA	1221	CLA	OBD-CAD-C3D	-2.27	124.02	128.04
14	BA	1204	CLA	CAC-C3C-C4C	2.27	127.78	124.82
14	aA	1138	CLA	CAC-C3C-C4C	2.27	127.78	124.82
14	BA	1223	CLA	CHA-C1A-NA	-2.27	121.21	126.40
14	BA	1229	CLA	C3C-C4C-NC	2.27	113.11	110.57
14	aA	1116	CLA	CMA-C3A-C4A	2.27	117.86	111.77
18	HA	4004	BCR	C33-C5-C6	-2.27	121.99	124.51
14	BA	1022	CLA	C4C-C3C-C2C	-2.26	103.61	106.89
14	bA	1238	CLA	C1-C2-C3	-2.26	122.13	126.04
14	bA	1220	CLA	CAC-C3C-C4C	2.26	127.78	124.82
14	GA	1117	CLA	C1-C2-C3	-2.26	122.13	126.04
14	lA	1501	CLA	O1D-CGD-CBD	-2.26	119.79	124.48
14	bA	1234	CLA	C4-C3-C2	-2.26	117.80	123.68
14	AA	1118	CLA	CMC-C2C-C1C	2.26	128.47	125.03
14	AA	1117	CLA	C1-C2-C3	-2.26	122.13	126.04
18	AA	4003	BCR	C4-C5-C6	-2.26	119.44	122.74
14	aA	1107	CLA	CMA-C3A-C4A	2.26	117.85	111.77
14	AA	1138	CLA	CMA-C3A-C4A	2.26	117.85	111.77
14	BA	1221	CLA	OBD-CAD-C3D	-2.26	124.04	128.04
14	BA	1216	CLA	C3C-C4C-NC	2.26	113.11	110.57
14	aA	1119	CLA	C4C-C3C-C2C	-2.26	103.62	106.89
14	GA	1118	CLA	CMC-C2C-C1C	2.26	128.47	125.03
14	GA	1127	CLA	C4-C3-C5	2.26	119.18	115.29
14	BA	1202	CLA	O1D-CGD-CBD	-2.26	119.80	124.48
14	BA	1207	CLA	C3C-C4C-NC	2.26	113.10	110.57

Continued on next page...

PRELIMINARY VALIDATION REPORT

Continued from previous page...

Mol	Chain	Res	Type	Atoms	Z	Observed(°)	Ideal(°)
14	AA	1104	CLA	O2D-CGD-O1D	-2.26	119.37	123.83
14	HA	1208	CLA	CAA-CBA-CGA	-2.26	106.61	113.26
14	aA	1132	CLA	CMB-C2B-C3B	2.26	129.03	124.80
14	HA	1223	CLA	CHA-C1A-NA	-2.26	121.23	126.40
14	bA	1216	CLA	CMC-C2C-C3C	2.25	132.26	126.11
14	aA	1138	CLA	C3C-C4C-NC	2.25	113.10	110.57
18	aA	4011	BCR	C1-C6-C7	2.25	122.19	115.78
14	bA	1229	CLA	C3C-C4C-NC	2.25	113.10	110.57
14	GA	1132	CLA	CMB-C2B-C3B	2.25	129.03	124.80
14	AA	1119	CLA	OBD-CAD-CBD	-2.25	122.60	125.91
14	GA	1107	CLA	CMA-C3A-C4A	2.25	117.83	111.77
14	bA	1022	CLA	C4C-C3C-C2C	-2.25	103.63	106.89
18	GA	4011	BCR	C1-C6-C7	2.25	122.18	115.78
14	AA	1117	CLA	CAC-C3C-C4C	2.25	127.76	124.82
14	BA	1235	CLA	O1D-CGD-CBD	-2.25	119.81	124.48
14	bA	1206	CLA	OBD-CAD-C3D	-2.25	124.05	128.04
14	AA	1132	CLA	CMB-C2B-C3B	2.25	129.02	124.80
14	bA	1206	CLA	CHC-C1C-C2C	-2.25	120.51	126.71
14	HA	1216	CLA	C3C-C4C-NC	2.25	113.09	110.57
14	AA	1104	CLA	C7-C6-C5	-2.25	107.20	113.25
14	HA	1206	CLA	OBD-CAD-C3D	-2.25	124.06	128.04
18	aA	4001	BCR	C34-C9-C8	2.25	121.66	118.09
14	BA	1206	CLA	OBD-CAD-C3D	-2.25	124.06	128.04
14	BA	1234	CLA	C4-C3-C2	-2.25	117.83	123.68
14	HA	1224	CLA	C4C-C3C-C2C	-2.25	103.63	106.89
18	RA	4018	BCR	C23-C22-C21	2.25	122.39	118.94
14	HA	1205	CLA	C16-C15-C13	-2.25	108.75	115.77
14	aA	1104	CLA	C7-C6-C5	-2.25	107.21	113.25
14	bA	1212	CLA	CAC-C3C-C4C	2.25	127.76	124.82
21	GA	6002	LMT	C3B-C4B-C5B	-2.25	106.19	110.23
14	aA	1111	CLA	CHA-C1A-NA	-2.25	121.25	126.40
14	GA	1125	CLA	CHC-C1C-C2C	-2.25	120.52	126.71
14	AA	1013	CLA	C1-O2A-CGA	2.25	122.68	116.54
14	GA	1013	CLA	C1-O2A-CGA	2.25	122.68	116.54
14	bA	1210	CLA	CMC-C2C-C1C	2.25	128.45	125.03
14	GA	1119	CLA	OBD-CAD-CBD	-2.25	122.61	125.91
18	LA	4022	BCR	C35-C13-C12	2.24	121.65	118.09
14	HA	1212	CLA	CAC-C3C-C4C	2.24	127.75	124.82
14	GA	1140	CLA	CMA-C3A-C4A	2.24	117.81	111.77
18	GA	4001	BCR	C34-C9-C8	2.24	121.65	118.09
14	GA	1123	CLA	C1-O2A-CGA	2.24	122.67	116.54
14	bA	1202	CLA	O1D-CGD-CBD	-2.24	119.83	124.48

Continued on next page...

PRELIMINARY VALIDATION REPORT

Continued from previous page...

Mol	Chain	Res	Type	Atoms	Z	Observed(°)	Ideal(°)
18	aA	4001	BCR	C38-C26-C27	2.24	117.77	113.57
14	aA	1116	CLA	C1-O2A-CGA	2.24	122.67	116.54
21	AA	6002	LMT	C1B-O1B-C4'	2.24	123.57	117.97
14	bA	1205	CLA	C16-C15-C13	-2.24	108.78	115.77
14	AA	1119	CLA	C4C-C3C-C2C	-2.24	103.64	106.89
14	aA	1103	CLA	CHC-C1C-C2C	-2.24	120.53	126.71
14	AA	1111	CLA	CHA-C1A-NA	-2.24	121.27	126.40
14	BA	1208	CLA	CAA-CBA-CGA	-2.24	106.66	113.26
14	GA	1104	CLA	C7-C6-C5	-2.24	107.23	113.25
14	HA	1216	CLA	CMC-C2C-C3C	2.24	132.22	126.11
14	BA	1216	CLA	CMC-C2C-C3C	2.24	132.22	126.11
21	aA	6002	LMT	C3B-C4B-C5B	-2.24	106.20	110.23
21	aA	6002	LMT	C1B-O1B-C4'	2.24	123.56	117.97
14	AA	1116	CLA	CMA-C3A-C4A	2.24	117.79	111.77
14	LA	1502	CLA	C1-O2A-CGA	2.24	122.66	116.54
14	AA	1125	CLA	CHC-C1C-C2C	-2.24	120.54	126.71
18	BA	4004	BCR	C33-C5-C6	-2.24	122.02	124.51
14	AA	1116	CLA	C1-O2A-CGA	2.24	122.66	116.54
14	AA	1103	CLA	CHC-C1C-C2C	-2.24	120.54	126.71
14	HA	1206	CLA	CHC-C1C-C2C	-2.24	120.54	126.71
14	bA	1219	CLA	CAA-C2A-C3A	-2.24	106.65	112.78
14	GA	1119	CLA	C4C-C3C-C2C	-2.24	103.65	106.89
14	GA	1116	CLA	CMA-C3A-C4A	2.24	117.78	111.77
18	lA	4022	BCR	C35-C13-C12	2.24	121.64	118.09
14	AA	1123	CLA	C1-O2A-CGA	2.24	122.65	116.54
14	BA	1205	CLA	C16-C15-C13	-2.24	108.80	115.77
18	AA	4011	BCR	C1-C6-C7	2.23	122.13	115.78
14	bA	1208	CLA	CAA-CBA-CGA	-2.23	106.67	113.26
14	BA	1206	CLA	CHC-C1C-C2C	-2.23	120.56	126.71
18	AA	4001	BCR	C34-C9-C8	2.23	121.63	118.09
18	bA	4004	BCR	C33-C5-C6	-2.23	122.03	124.51
14	GA	1111	CLA	CHA-C1A-NA	-2.23	121.29	126.40
14	aA	1125	CLA	CHC-C1C-C2C	-2.23	120.56	126.71
14	BA	1219	CLA	CAA-C2A-C3A	-2.23	106.67	112.78
14	HA	1210	CLA	CMC-C2C-C1C	2.23	128.42	125.03
14	bA	1224	CLA	C4C-C3C-C2C	-2.23	103.66	106.89
14	UA	1502	CLA	C1-O2A-CGA	2.23	122.63	116.54
14	lA	1502	CLA	C1-O2A-CGA	2.23	122.63	116.54
18	IA	4018	BCR	C23-C22-C21	2.23	122.36	118.94
18	HA	4017	BCR	C36-C18-C17	-2.23	119.81	122.92
18	bA	4017	BCR	C36-C18-C17	-2.23	119.81	122.92
14	aA	1123	CLA	C1-O2A-CGA	2.23	122.62	116.54

Continued on next page...

PRELIMINARY VALIDATION REPORT

Continued from previous page...

Mol	Chain	Res	Type	Atoms	Z	Observed(^{o})	Ideal(^{o})
14	BA	1212	CLA	C4C-C3C-C2C	-2.23	103.67	106.89
14	HA	1202	CLA	O1D-CGD-CBD	-2.23	119.87	124.48
18	iA	4018	BCR	C23-C22-C21	2.22	122.36	118.94
14	HA	1201	CLA	C4-C3-C2	-2.22	117.89	123.68
14	HA	1219	CLA	CAA-C2A-C3A	-2.22	106.69	112.78
14	BA	1203	CLA	CHD-C4C-C3C	-2.22	121.63	124.87
14	GA	1116	CLA	C1-O2A-CGA	2.22	122.62	116.54
14	HA	1202	CLA	CMC-C2C-C1C	2.22	128.41	125.03
14	BA	1210	CLA	CMC-C2C-C1C	2.22	128.41	125.03
14	bA	1221	CLA	OBD-CAD-CBD	-2.22	122.65	125.91
14	BA	1229	CLA	CMA-C3A-C4A	2.22	117.75	111.77
14	aA	1118	CLA	CMC-C2C-C1C	2.22	128.41	125.03
14	BA	1224	CLA	C4C-C3C-C2C	-2.22	103.67	106.89
18	AA	4001	BCR	C38-C26-C27	2.22	117.73	113.57
14	bA	1201	CLA	C4-C3-C2	-2.22	117.90	123.68
14	AA	1140	CLA	CMA-C3A-C4A	2.22	117.74	111.77
14	AA	1137	CLA	CAC-C3C-C4C	2.22	127.72	124.82
14	bA	1217	CLA	OBD-CAD-C3D	-2.22	124.11	128.04
14	GA	1103	CLA	CHC-C1C-C2C	-2.22	120.59	126.71
14	aA	1119	CLA	OBD-CAD-CBD	-2.22	122.65	125.91
14	aA	1108	CLA	O1D-CGD-CBD	-2.22	119.88	124.48
14	aA	1104	CLA	O2D-CGD-O1D	-2.22	119.45	123.83
14	bA	1210	CLA	C1-O2A-CGA	2.22	122.60	116.54
14	bA	1216	CLA	C3C-C4C-NC	2.22	113.06	110.57
18	GA	4002	BCR	C33-C5-C6	-2.22	122.04	124.51
14	BA	1204	CLA	C1D-CHD-C4C	2.22	125.52	122.48
14	GA	1104	CLA	O2D-CGD-O1D	-2.22	119.45	123.83
14	KA	1401	CLA	CMC-C2C-C1C	2.22	128.40	125.03
14	HA	1232	CLA	CHC-C1C-C2C	-2.22	120.60	126.71
14	GA	1124	CLA	C1-O2A-CGA	2.22	122.60	116.54
18	GA	4001	BCR	C38-C26-C27	2.22	117.72	113.57
14	HA	1021	CLA	C1-C2-C3	-2.22	122.21	126.04
14	bA	1021	CLA	C1-C2-C3	-2.21	122.21	126.04
21	AA	6002	LMT	C3B-C4B-C5B	-2.21	106.25	110.23
14	HA	1204	CLA	C1D-CHD-C4C	2.21	125.51	122.48
14	AA	1106	CLA	CMA-C3A-C4A	2.21	117.72	111.77
14	BA	1217	CLA	OBD-CAD-C3D	-2.21	124.12	128.04
14	BA	1210	CLA	C1-O2A-CGA	2.21	122.59	116.54
14	aA	1140	CLA	CMA-C3A-C4A	2.21	117.72	111.77
18	BA	4009	BCR	C31-C1-C6	-2.21	106.72	110.30
14	bA	1202	CLA	CMC-C2C-C1C	2.21	128.40	125.03
18	AA	4001	BCR	C35-C13-C12	2.21	121.60	118.09

Continued on next page...

PRELIMINARY VALIDATION REPORT

Continued from previous page...

Mol	Chain	Res	Type	Atoms	Z	Observed(°)	Ideal(°)
18	AA	4002	BCR	C33-C5-C6	-2.21	122.05	124.51
18	BA	4017	BCR	C36-C18-C17	-2.21	119.83	122.92
14	bA	1229	CLA	CMA-C3A-C4A	2.21	117.72	111.77
14	aA	1137	CLA	CAC-C3C-C4C	2.21	127.71	124.82
14	TA	1401	CLA	CMC-C2C-C1C	2.21	128.39	125.03
14	HA	1221	CLA	OBD-CAD-CBD	-2.21	122.67	125.91
14	aA	1106	CLA	CMA-C3A-C4A	2.21	117.71	111.77
14	BA	1222	CLA	C4-C3-C2	-2.21	117.94	123.68
14	bA	1215	CLA	CHC-C1C-C2C	-2.21	120.63	126.71
18	bA	4005	BCR	C28-C27-C26	-2.21	110.15	113.99
14	bA	1207	CLA	OBD-CAD-C3D	-2.21	124.13	128.04
14	bA	1232	CLA	CHC-C1C-C2C	-2.21	120.63	126.71
14	bA	1204	CLA	C1D-CHD-C4C	2.21	125.50	122.48
14	bA	1023	CLA	CMC-C2C-C3C	2.21	132.12	126.11
14	BA	1232	CLA	CHC-C1C-C2C	-2.20	120.63	126.71
14	HA	1217	CLA	OBD-CAD-C3D	-2.20	124.13	128.04
14	AA	1117	CLA	C3B-C4B-NB	2.20	112.06	109.21
14	BA	1201	CLA	C4-C3-C2	-2.20	117.95	123.68
14	bA	1203	CLA	CHD-C4C-C3C	-2.20	121.66	124.87
14	GA	1108	CLA	O1D-CGD-CBD	-2.20	119.91	124.48
14	BA	1023	CLA	CMC-C2C-C3C	2.20	132.12	126.11
20	bA	5002	LMG	C8-O7-C10	-2.20	112.32	117.82
14	AA	1119	CLA	C1-O2A-CGA	2.20	122.56	116.54
18	aA	4001	BCR	C35-C13-C12	2.20	121.58	118.09
14	BA	1203	CLA	O2D-CGD-O1D	-2.20	119.48	123.83
21	GA	6002	LMT	O5B-C5B-C6B	2.20	111.92	106.43
14	bA	1021	CLA	C7-C6-C5	-2.20	107.33	113.25
14	BA	1221	CLA	OBD-CAD-CBD	-2.20	122.68	125.91
14	bA	1228	CLA	C3C-C4C-NC	2.20	113.04	110.57
14	GA	1106	CLA	CMA-C3A-C4A	2.20	117.69	111.77
14	kA	1401	CLA	CMC-C2C-C1C	2.20	128.38	125.03
14	BA	1202	CLA	CMC-C2C-C1C	2.20	128.38	125.03
18	bA	4009	BCR	C31-C1-C6	-2.20	106.74	110.30
18	BA	4005	BCR	C28-C27-C26	-2.20	110.16	113.99
14	HA	1210	CLA	C1-O2A-CGA	2.20	122.55	116.54
14	BA	1228	CLA	C3C-C4C-NC	2.20	113.04	110.57
18	LA	4022	BCR	C31-C1-C6	-2.20	106.75	110.30
14	AA	1108	CLA	O1D-CGD-CBD	-2.20	119.92	124.48
14	HA	1023	CLA	CMC-C2C-C3C	2.20	132.10	126.11
14	BA	1207	CLA	OBD-CAD-C3D	-2.20	124.14	128.04
21	GA	6001	LMT	O5'-C5'-C4'	2.20	114.41	109.76
14	BA	1021	CLA	C1-C2-C3	-2.20	122.24	126.04

Continued on next page...

PRELIMINARY VALIDATION REPORT

Continued from previous page...

Mol	Chain	Res	Type	Atoms	Z	Observed(^{o})	Ideal(^{o})
14	bA	1222	CLA	C4-C3-C2	-2.20	117.97	123.68
18	aA	4001	BCR	C38-C26-C25	-2.20	122.06	124.51
21	aA	6001	LMT	O5'-C5'-C4'	2.20	114.41	109.76
20	HA	5002	LMG	C8-O7-C10	-2.20	112.34	117.82
14	BA	1215	CLA	CHC-C1C-C2C	-2.20	120.65	126.71
21	AA	6001	LMT	O5'-C5'-C4'	2.20	114.41	109.76
14	HA	1229	CLA	CMA-C3A-C4A	2.20	117.67	111.77
14	HA	1203	CLA	CHD-C4C-C3C	-2.20	121.67	124.87
14	bA	1212	CLA	C4C-C3C-C2C	-2.20	103.71	106.89
20	BA	5002	LMG	C8-O7-C10	-2.20	112.34	117.82
14	BA	1021	CLA	C7-C6-C5	-2.19	107.35	113.25
14	GA	1117	CLA	CMC-C2C-C3C	2.19	132.09	126.11
14	GA	1012	CLA	C1-C2-C3	-2.19	122.25	126.04
14	AA	1124	CLA	C1-O2A-CGA	2.19	122.53	116.54
18	mA	4021	BCR	C40-C30-C25	2.19	113.85	110.30
14	aA	1119	CLA	C1-O2A-CGA	2.19	122.53	116.54
18	HA	4005	BCR	C28-C27-C26	-2.19	110.18	113.99
14	HA	1021	CLA	C7-C6-C5	-2.19	107.36	113.25
14	GA	1116	CLA	O1D-CGD-CBD	-2.19	119.94	124.48
14	bA	1203	CLA	O2D-CGD-O1D	-2.19	119.50	123.83
14	aA	1124	CLA	C1-O2A-CGA	2.19	122.53	116.54
14	bA	1226	CLA	C4-C3-C5	2.19	119.06	115.29
14	HA	1207	CLA	OBD-CAD-C3D	-2.19	124.16	128.04
14	HA	1224	CLA	CAC-C3C-C4C	2.19	127.68	124.82
14	GA	1119	CLA	C1-O2A-CGA	2.19	122.53	116.54
14	AA	1133	CLA	O1D-CGD-CBD	-2.19	119.94	124.48
14	aA	1130	CLA	CMC-C2C-C1C	2.19	128.36	125.03
14	GA	1137	CLA	CAC-C3C-C4C	2.19	127.68	124.82
14	HA	1222	CLA	C4-C3-C2	-2.19	117.99	123.68
18	GA	4001	BCR	C35-C13-C12	2.19	121.56	118.09
14	bA	1220	CLA	CHC-C1C-C2C	-2.19	120.68	126.71
14	aA	1127	CLA	CMA-C3A-C4A	2.19	117.65	111.77
14	bA	1023	CLA	CAA-CBA-CGA	-2.19	106.81	113.26
14	aA	1012	CLA	C1-C2-C3	-2.19	122.26	126.04
18	BA	4012	BCR	C23-C22-C21	2.19	122.30	118.94
14	HA	1226	CLA	C4-C3-C5	2.19	119.05	115.29
14	BA	1220	CLA	CHC-C1C-C2C	-2.18	120.69	126.71
14	BA	1239	CLA	C4-C3-C5	2.18	119.05	115.29
14	HA	1223	CLA	O1D-CGD-CBD	-2.18	119.95	124.48
14	bA	1238	CLA	CED-O2D-CGD	2.18	120.94	115.95
21	aA	6002	LMT	O5B-C5B-C6B	2.18	111.87	106.43
18	HA	4009	BCR	C31-C1-C6	-2.18	106.77	110.30

Continued on next page...

PRELIMINARY VALIDATION REPORT

Continued from previous page...

Mol	Chain	Res	Type	Atoms	Z	Observed(^{o})	Ideal(^{o})
14	BA	1023	CLA	CAA-CBA-CGA	-2.18	106.82	113.26
14	BA	1238	CLA	CED-O2D-CGD	2.18	120.94	115.95
18	lA	4022	BCR	C31-C1-C6	-2.18	106.77	110.30
14	HA	1215	CLA	CHC-C1C-C2C	-2.18	120.69	126.71
18	aA	4002	BCR	C33-C5-C6	-2.18	122.08	124.51
14	GA	1130	CLA	C1-O2A-CGA	2.18	122.50	116.54
14	GA	1130	CLA	CMC-C2C-C1C	2.18	128.35	125.03
14	AA	1126	CLA	C4C-C3C-C2C	-2.18	103.73	106.89
14	UA	1503	CLA	C11-C10-C8	-2.18	108.96	115.77
14	GA	1127	CLA	CMA-C3A-C4A	2.18	117.64	111.77
14	HA	1203	CLA	O2D-CGD-O1D	-2.18	119.52	123.83
18	HA	4013	BCR	C33-C5-C4	2.18	117.65	113.57
14	KA	1401	CLA	CMB-C2B-C3B	2.18	128.89	124.80
14	AA	1130	CLA	CMC-C2C-C1C	2.18	128.35	125.03
18	BA	4013	BCR	C33-C5-C4	2.18	117.65	113.57
14	AA	1117	CLA	CMC-C2C-C3C	2.18	132.05	126.11
14	HA	1201	CLA	C1-O2A-CGA	2.18	122.50	116.54
21	AA	6002	LMT	O5B-C5B-C6B	2.18	111.86	106.43
14	AA	1116	CLA	O1D-CGD-CBD	-2.18	119.96	124.48
14	HA	1239	CLA	C1-O2A-CGA	2.18	122.50	116.54
14	HA	1228	CLA	C3C-C4C-NC	2.18	113.01	110.57
14	bA	1239	CLA	C1-O2A-CGA	2.18	122.49	116.54
14	GA	1126	CLA	C4C-C3C-C2C	-2.18	103.74	106.89
14	aA	1130	CLA	C1-O2A-CGA	2.18	122.49	116.54
18	bA	4017	BCR	C2-C1-C6	2.18	113.85	110.48
14	AA	1101	CLA	CAC-C3C-C4C	2.18	127.66	124.82
18	HA	4017	BCR	C2-C1-C6	2.17	113.85	110.48
14	BA	1239	CLA	C1-O2A-CGA	2.17	122.48	116.54
14	lA	1503	CLA	CHC-C1C-C2C	-2.17	120.72	126.71
14	GA	1118	CLA	C3C-C4C-NC	2.17	113.01	110.57
14	GA	1123	CLA	C1-C2-C3	-2.17	122.28	126.04
14	aA	1117	CLA	C3B-C4B-NB	2.17	112.02	109.21
14	AA	1012	CLA	C1-C2-C3	-2.17	122.29	126.04
20	RA	5006	LMG	C8-O7-C10	-2.17	112.40	117.82
14	HA	1216	CLA	C1-O2A-CGA	2.17	122.48	116.54
14	GA	1133	CLA	O1D-CGD-CBD	-2.17	119.98	124.48
14	BA	1224	CLA	CAC-C3C-C4C	2.17	127.66	124.82
14	AA	1127	CLA	CMA-C3A-C4A	2.17	117.61	111.77
14	lA	1503	CLA	C11-C10-C8	-2.17	109.00	115.77
14	AA	1140	CLA	C4C-C3C-C2C	-2.17	103.75	106.89
18	UA	4022	BCR	C31-C1-C6	-2.17	106.79	110.30
18	AA	4001	BCR	C37-C22-C21	-2.17	119.88	122.92

Continued on next page...

PRELIMINARY VALIDATION REPORT

Continued from previous page...

Mol	Chain	Res	Type	Atoms	Z	Observed(^{o})	Ideal(^{o})
18	bA	4013	BCR	C33-C5-C4	2.17	117.63	113.57
14	HA	1023	CLA	CAA-CBA-CGA	-2.17	106.86	113.26
14	aA	1118	CLA	C3C-C4C-NC	2.17	113.00	110.57
14	AA	1114	CLA	OBD-CAD-C3D	-2.17	124.20	128.04
18	HA	4012	BCR	C23-C22-C21	2.17	122.27	118.94
14	AA	1106	CLA	CHB-C4A-NA	2.17	127.51	124.51
14	BA	1216	CLA	C1-O2A-CGA	2.17	122.47	116.54
14	aA	1133	CLA	O1D-CGD-CBD	-2.17	119.99	124.48
14	LA	1503	CLA	C11-C10-C8	-2.17	109.00	115.77
14	AA	1013	CLA	CAC-C3C-C4C	2.17	127.65	124.82
14	bA	1215	CLA	C11-C10-C8	-2.17	109.01	115.77
14	HA	1238	CLA	CED-O2D-CGD	2.17	120.90	115.95
14	GA	1128	CLA	O1D-CGD-CBD	-2.17	119.99	124.48
14	bA	1227	CLA	O1D-CGD-CBD	-2.17	119.99	124.48
14	HA	1239	CLA	C4-C3-C5	2.17	119.02	115.29
14	BA	1215	CLA	C11-C10-C8	-2.17	109.01	115.77
14	HA	1220	CLA	CHC-C1C-C2C	-2.17	120.74	126.71
14	UA	1503	CLA	CHC-C1C-C2C	-2.17	120.74	126.71
14	aA	1126	CLA	C4C-C3C-C2C	-2.17	103.75	106.89
18	bA	4006	BCR	C2-C1-C6	-2.17	107.14	110.48
18	BA	4006	BCR	C2-C1-C6	-2.17	107.14	110.48
14	bA	1239	CLA	C4-C3-C5	2.17	119.02	115.29
18	VA	4021	BCR	C40-C30-C25	2.17	113.81	110.30
14	HA	1215	CLA	C11-C10-C8	-2.16	109.02	115.77
14	HA	1212	CLA	C4C-C3C-C2C	-2.16	103.76	106.89
14	AA	1124	CLA	O2D-CGD-O1D	-2.16	119.56	123.83
14	TA	1401	CLA	CMB-C2B-C3B	2.16	128.86	124.80
14	AA	1133	CLA	C1-O2A-CGA	2.16	122.45	116.54
14	bA	1223	CLA	O1D-CGD-CBD	-2.16	120.00	124.48
14	aA	1123	CLA	C1-C2-C3	-2.16	122.30	126.04
18	BA	4017	BCR	C2-C1-C6	2.16	113.83	110.48
14	GA	1117	CLA	C3B-C4B-NB	2.16	112.01	109.21
14	bA	1022	CLA	C1-O2A-CGA	2.16	122.45	116.54
14	AA	1130	CLA	C1-O2A-CGA	2.16	122.45	116.54
18	AA	4011	BCR	C35-C13-C12	2.16	121.52	118.09
14	kA	1401	CLA	CMB-C2B-C3B	2.16	128.85	124.80
14	GA	1140	CLA	C4C-C3C-C2C	-2.16	103.76	106.89
14	HA	1227	CLA	O1D-CGD-CBD	-2.16	120.00	124.48
18	HA	4016	BCR	C35-C13-C14	-2.16	119.90	122.92
14	0	1012	CLA	C1-O2A-CGA	2.16	122.44	116.54
14	aA	1117	CLA	CMC-C2C-C3C	2.16	132.00	126.11
14	bA	1201	CLA	C1-O2A-CGA	2.16	122.44	116.54

Continued on next page...

PRELIMINARY VALIDATION REPORT

Continued from previous page...

Mol	Chain	Res	Type	Atoms	Z	Observed(°)	Ideal(°)
18	GA	4001	BCR	C38-C26-C25	-2.16	122.11	124.51
14	GA	1118	CLA	CHC-C1C-C2C	-2.16	120.76	126.71
14	BA	1022	CLA	C1-O2A-CGA	2.16	122.44	116.54
14	aA	1124	CLA	O2D-CGD-O1D	-2.16	119.57	123.83
14	AA	1104	CLA	C3C-C4C-NC	2.16	112.99	110.57
14	bA	1220	CLA	OBD-CAD-C3D	-2.16	124.22	128.04
20	IA	5006	LMG	C8-O7-C10	-2.16	112.44	117.82
14	AA	1118	CLA	CHC-C1C-C2C	-2.16	120.76	126.71
14	bA	1231	CLA	CAA-C2A-C3A	-2.16	106.87	112.78
14	aA	1133	CLA	C1-O2A-CGA	2.16	122.44	116.54
14	BA	1239	CLA	O1D-CGD-CBD	-2.16	120.01	124.48
14	LA	1503	CLA	CHC-C1C-C2C	-2.16	120.77	126.71
14	AA	1123	CLA	C1-C2-C3	-2.15	122.32	126.04
14	BA	1226	CLA	C4-C3-C5	2.15	119.00	115.29
14	bA	1227	CLA	CAC-C3C-C4C	2.15	127.64	124.82
14	GA	1106	CLA	CHB-C4A-NA	2.15	127.49	124.51
14	bA	1216	CLA	C1-O2A-CGA	2.15	122.43	116.54
14	bA	1224	CLA	CAC-C3C-C4C	2.15	127.64	124.82
14	GA	1126	CLA	CAC-C3C-C2C	2.15	131.21	127.53
14	bA	1022	CLA	CHA-C1A-NA	-2.15	121.47	126.40
14	bA	1238	CLA	O2D-CGD-O1D	-2.15	119.58	123.83
14	HA	1219	CLA	CHB-C4A-NA	2.15	127.49	124.51
14	bA	1223	CLA	CMA-C3A-C4A	2.15	117.56	111.77
20	iA	5006	LMG	C8-O7-C10	-2.15	112.45	117.82
14	HA	1220	CLA	OBD-CAD-C3D	-2.15	124.23	128.04
14	BA	1223	CLA	O1D-CGD-CBD	-2.15	120.02	124.48
18	MA	4021	BCR	C40-C30-C25	2.15	113.79	110.30
14	GA	1124	CLA	O2D-CGD-O1D	-2.15	119.58	123.83
18	bA	4016	BCR	C35-C13-C14	-2.15	119.91	122.92
14	aA	1106	CLA	CHB-C4A-NA	2.15	127.49	124.51
14	bA	1219	CLA	CHB-C4A-NA	2.15	127.49	124.51
14	AA	1113	CLA	CHC-C1C-C2C	-2.15	120.78	126.71
14	BA	1201	CLA	C1-O2A-CGA	2.15	122.42	116.54
14	GA	1111	CLA	C4C-C3C-C2C	-2.15	103.78	106.89
14	BA	1227	CLA	O1D-CGD-CBD	-2.15	120.02	124.48
14	HA	1239	CLA	O1D-CGD-CBD	-2.15	120.03	124.48
14	aA	1116	CLA	O1D-CGD-CBD	-2.15	120.03	124.48
14	aA	1113	CLA	CHC-C1C-C2C	-2.15	120.79	126.71
14	GA	1114	CLA	CAA-C2A-C3A	-2.15	106.89	112.78
18	BA	4004	BCR	C31-C1-C6	-2.15	106.83	110.30
18	GA	4001	BCR	C37-C22-C21	-2.15	119.91	122.92
14	GA	1133	CLA	C1-O2A-CGA	2.15	122.41	116.54

Continued on next page...

PRELIMINARY VALIDATION REPORT

Continued from previous page...

Mol	Chain	Res	Type	Atoms	Z	Observed(°)	Ideal(°)
18	iA	4018	BCR	C23-C24-C25	-2.15	121.26	127.28
14	AA	1128	CLA	O1D-CGD-CBD	-2.15	120.03	124.48
14	AA	1119	CLA	O2D-CGD-O1D	-2.15	119.59	123.83
18	bA	4012	BCR	C23-C22-C21	2.15	122.23	118.94
14	aA	1126	CLA	O2D-CGD-O1D	-2.15	119.59	123.83
18	IA	4018	BCR	C23-C24-C25	-2.15	121.27	127.28
14	GA	1128	CLA	C4C-C3C-C2C	-2.15	103.78	106.89
14	BA	1231	CLA	CAA-C2A-C3A	-2.14	106.91	112.78
14	aA	1104	CLA	C3C-C4C-NC	2.14	112.98	110.57
14	aA	1118	CLA	CHC-C1C-C2C	-2.14	120.80	126.71
14	GA	1126	CLA	O2D-CGD-O1D	-2.14	119.60	123.83
14	AA	1101	CLA	O1D-CGD-CBD	-2.14	120.04	124.48
14	GA	1013	CLA	CAC-C3C-C4C	2.14	127.62	124.82
14	GA	1113	CLA	CHC-C1C-C2C	-2.14	120.80	126.71
14	AA	1126	CLA	O2D-CGD-O1D	-2.14	119.60	123.83
14	BA	1222	CLA	C4C-C3C-C2C	-2.14	103.79	106.89
14	BA	1021	CLA	CMA-C3A-C2A	-2.14	105.14	113.78
14	GA	1114	CLA	OBD-CAD-C3D	-2.14	124.24	128.04
14	HA	1223	CLA	CMA-C3A-C4A	2.14	117.53	111.77
14	BA	1227	CLA	CAC-C3C-C4C	2.14	127.62	124.82
14	AA	1114	CLA	C3C-C4C-NC	2.14	112.97	110.57
14	BA	1223	CLA	CMA-C3A-C4A	2.14	117.53	111.77
18	BA	4012	BCR	C35-C13-C12	2.14	121.49	118.09
18	RA	4018	BCR	C23-C24-C25	-2.14	121.28	127.28
14	HA	1228	CLA	C4C-C3C-C2C	-2.14	103.79	106.89
14	aA	1140	CLA	C4C-C3C-C2C	-2.14	103.79	106.89
14	BA	1219	CLA	CHB-C4A-NA	2.14	127.47	124.51
18	aA	4011	BCR	C35-C13-C12	2.14	121.48	118.09
14	AA	1111	CLA	C4C-C3C-C2C	-2.14	103.79	106.89
14	aA	1013	CLA	CAC-C3C-C4C	2.14	127.61	124.82
14	AA	1118	CLA	C3C-C4C-NC	2.14	112.97	110.57
14	aA	1114	CLA	CAA-C2A-C3A	-2.14	106.92	112.78
14	BA	1238	CLA	O2D-CGD-O1D	-2.14	119.61	123.83
18	HA	4004	BCR	C31-C1-C6	-2.14	106.84	110.30
14	AA	1114	CLA	CAA-C2A-C3A	-2.14	106.92	112.78
14	BA	1022	CLA	CHA-C1A-NA	-2.14	121.50	126.40
18	AA	4011	BCR	C37-C22-C21	-2.14	119.93	122.92
14	HA	1021	CLA	CMA-C3A-C2A	-2.14	105.17	113.78
14	bA	1021	CLA	CMA-C3A-C2A	-2.14	105.17	113.78
14	HA	1231	CLA	CAA-C2A-C3A	-2.14	106.93	112.78
14	HA	1238	CLA	O2D-CGD-O1D	-2.14	119.61	123.83
14	AA	1128	CLA	C4C-C3C-C2C	-2.14	103.80	106.89

Continued on next page...

PRELIMINARY VALIDATION REPORT

Continued from previous page...

Mol	Chain	Res	Type	Atoms	Z	Observed(^{o})	Ideal(^{o})
14	aA	1128	CLA	O1D-CGD-CBD	-2.14	120.05	124.48
14	bA	1214	CLA	O1D-CGD-CBD	-2.14	120.06	124.48
14	GA	1101	CLA	O1D-CGD-CBD	-2.14	120.06	124.48
14	BA	1220	CLA	OBD-CAD-C3D	-2.14	124.26	128.04
14	aA	1126	CLA	CAC-C3C-C2C	2.13	131.18	127.53
14	HA	1221	CLA	C1-C2-C3	-2.13	122.35	126.04
18	AA	4001	BCR	C38-C26-C25	-2.13	122.13	124.51
14	aA	1119	CLA	O2D-CGD-O1D	-2.13	119.62	123.83
14	GA	1104	CLA	C3C-C4C-NC	2.13	112.96	110.57
14	bA	1239	CLA	O1D-CGD-CBD	-2.13	120.06	124.48
14	0	1012	CLA	CHA-C1A-NA	-2.13	121.52	126.40
14	HA	1232	CLA	CHA-C1A-NA	-2.13	121.52	126.40
14	aA	1101	CLA	CAC-C3C-C4C	2.13	127.61	124.82
14	HA	1221	CLA	CAA-CBA-CGA	-2.13	106.98	113.26
14	AA	1119	CLA	C4-C3-C5	2.13	118.96	115.29
14	GA	1119	CLA	O2D-CGD-O1D	-2.13	119.62	123.83
14	LA	1501	CLA	CMA-C3A-C4A	2.13	117.50	111.77
14	lA	1501	CLA	CMA-C3A-C4A	2.13	117.50	111.77
14	bA	1205	CLA	OBD-CAD-CBD	-2.13	122.78	125.91
14	BA	1239	CLA	CAC-C3C-C2C	2.13	131.17	127.53
14	HA	1227	CLA	CAC-C3C-C4C	2.13	127.60	124.82
18	aA	4001	BCR	C33-C5-C4	2.13	117.56	113.57
14	bA	1221	CLA	CAA-CBA-CGA	-2.13	106.98	113.26
14	HA	1222	CLA	C4C-C3C-C2C	-2.13	103.81	106.89
14	GA	1101	CLA	CAC-C3C-C4C	2.13	127.60	124.82
18	bA	4014	BCR	C4-C5-C6	-2.13	119.63	122.74
14	bA	1202	CLA	CHA-C1A-NA	-2.13	121.53	126.40
14	aA	1128	CLA	C4C-C3C-C2C	-2.13	103.81	106.89
14	UA	1501	CLA	CMA-C3A-C4A	2.13	117.48	111.77
18	bA	4012	BCR	C35-C13-C12	2.13	121.46	118.09
18	GA	4011	BCR	C35-C13-C12	2.13	121.46	118.09
14	aA	1111	CLA	C4C-C3C-C2C	-2.12	103.81	106.89
18	GA	4002	BCR	C35-C13-C12	2.12	121.46	118.09
18	HA	4006	BCR	C2-C1-C6	-2.12	107.20	110.48
14	HA	1214	CLA	C11-C10-C8	-2.12	109.14	115.77
14	BA	1221	CLA	CAA-CBA-CGA	-2.12	107.00	113.26
14	aA	1114	CLA	OBD-CAD-C3D	-2.12	124.28	128.04
14	AA	1126	CLA	CAC-C3C-C2C	2.12	131.16	127.53
18	aA	4001	BCR	C37-C22-C21	-2.12	119.95	122.92
14	bA	1202	CLA	CHC-C1C-C2C	-2.12	120.86	126.71
14	BA	1214	CLA	C11-C10-C8	-2.12	109.15	115.77
14	GA	1119	CLA	CHC-C1C-C2C	-2.12	120.86	126.71

Continued on next page...

PRELIMINARY VALIDATION REPORT

Continued from previous page...

Mol	Chain	Res	Type	Atoms	Z	Observed(^{o})	Ideal(^{o})
14	AA	1119	CLA	CHC-C1C-C2C	-2.12	120.86	126.71
14	AA	1133	CLA	CMA-C3A-C4A	2.12	117.47	111.77
14	lA	1503	CLA	C11-C12-C13	-2.12	109.15	115.77
14	bA	1228	CLA	C4C-C3C-C2C	-2.12	103.82	106.89
18	BA	4014	BCR	C4-C5-C6	-2.12	119.64	122.74
14	aA	1114	CLA	C3C-C4C-NC	2.12	112.95	110.57
14	AA	1124	CLA	CMA-C3A-C4A	2.12	117.47	111.77
14	bA	1214	CLA	C11-C10-C8	-2.12	109.15	115.77
14	GA	1119	CLA	C4-C3-C5	2.12	118.94	115.29
14	bA	1221	CLA	C1-C2-C3	-2.12	122.38	126.04
18	bA	4005	BCR	C1-C6-C5	-2.12	119.63	122.59
14	aA	1132	CLA	C4-C3-C5	2.12	118.94	115.29
18	HA	4014	BCR	C4-C5-C6	-2.12	119.65	122.74
14	BA	1223	CLA	C4C-C3C-C2C	-2.12	103.82	106.89
18	AA	4002	BCR	C35-C13-C12	2.12	121.45	118.09
14	GA	1114	CLA	CHA-C1A-NA	-2.12	121.55	126.40
14	bA	1206	CLA	CMA-C3A-C4A	2.12	117.47	111.77
14	BA	1228	CLA	C4C-C3C-C2C	-2.12	103.82	106.89
14	bA	1239	CLA	CAC-C3C-C2C	2.12	131.15	127.53
18	BA	4016	BCR	C35-C13-C14	-2.12	119.96	122.92
14	AA	1137	CLA	CAA-C2A-C3A	-2.12	106.98	112.78
14	BA	1205	CLA	OBD-CAD-CBD	-2.12	122.80	125.91
14	GA	1133	CLA	CMA-C3A-C4A	2.12	117.47	111.77
14	aA	1101	CLA	O1D-CGD-CBD	-2.12	120.09	124.48
14	LA	1502	CLA	OBD-CAD-C3D	-2.12	124.29	128.04
14	xA	1701	CLA	C4C-C3C-C2C	-2.12	103.82	106.89
14	HA	1202	CLA	CHC-C1C-C2C	-2.12	120.88	126.71
14	aA	1123	CLA	OBD-CAD-CBD	-2.12	122.80	125.91
18	GA	4011	BCR	C37-C22-C21	-2.11	119.96	122.92
14	aA	1119	CLA	CHC-C1C-C2C	-2.11	120.88	126.71
18	BA	4009	BCR	C1-C6-C5	-2.11	119.64	122.59
14	LA	1503	CLA	C11-C12-C13	-2.11	109.17	115.77
14	bA	1232	CLA	CHA-C1A-NA	-2.11	121.56	126.40
14	GA	1124	CLA	CMA-C3A-C4A	2.11	117.45	111.77
18	GA	4007	BCR	C35-C13-C12	2.11	121.44	118.09
14	BA	1202	CLA	CHC-C1C-C2C	-2.11	120.89	126.71
14	BA	1202	CLA	CHA-C1A-NA	-2.11	121.56	126.40
18	BA	4010	BCR	C37-C22-C21	-2.11	119.97	122.92
14	HA	1221	CLA	CHB-C4A-NA	2.11	127.43	124.51
14	aA	1124	CLA	CMA-C3A-C4A	2.11	117.45	111.77
14	GA	1137	CLA	CAA-C2A-C3A	-2.11	107.00	112.78
18	HA	4012	BCR	C35-C13-C12	2.11	121.44	118.09

Continued on next page...

PRELIMINARY VALIDATION REPORT

Continued from previous page...

Mol	Chain	Res	Type	Atoms	Z	Observed(^{o})	Ideal(^{o})
14	UA	1502	CLA	OBD-CAD-C3D	-2.11	124.30	128.04
14	bA	1022	CLA	O1D-CGD-CBD	-2.11	120.11	124.48
18	MA	4021	BCR	C2-C1-C6	2.11	113.75	110.48
14	BA	1206	CLA	CMA-C3A-C4A	2.11	117.44	111.77
14	bA	1222	CLA	C4C-C3C-C2C	-2.11	103.84	106.89
14	HA	1234	CLA	OBD-CAD-C3D	-2.11	124.30	128.04
14	GA	1133	CLA	CHC-C1C-C2C	-2.11	120.90	126.71
14	GA	1125	CLA	OBD-CAD-CBD	-2.11	122.81	125.91
14	UA	1503	CLA	C11-C12-C13	-2.11	109.19	115.77
14	aA	1137	CLA	CAA-C2A-C3A	-2.11	107.01	112.78
18	aA	4011	BCR	C37-C22-C21	-2.11	119.97	122.92
18	GA	4001	BCR	C33-C5-C4	2.11	117.52	113.57
18	HA	4009	BCR	C1-C6-C5	-2.11	119.65	122.59
18	bA	4014	BCR	C12-C13-C14	-2.11	115.71	118.94
14	AA	1128	CLA	CMC-C2C-C3C	2.11	131.85	126.11
18	aA	4002	BCR	C35-C13-C12	2.11	121.43	118.09
14	bA	1221	CLA	CHB-C4A-NA	2.11	127.42	124.51
18	bA	4004	BCR	C23-C22-C21	2.10	122.17	118.94
14	aA	1104	CLA	O1D-CGD-CBD	-2.10	120.12	124.48
14	XA	1701	CLA	C4C-C3C-C2C	-2.10	103.84	106.89
18	VA	4021	BCR	C2-C1-C6	2.10	113.74	110.48
14	GA	1131	CLA	O1D-CGD-CBD	-2.10	120.12	124.48
14	aA	1125	CLA	OBD-CAD-CBD	-2.10	122.82	125.91
14	GA	1129	CLA	CAC-C3C-C4C	2.10	127.57	124.82
18	BA	4005	BCR	C1-C6-C5	-2.10	119.65	122.59
14	BA	1221	CLA	CHB-C4A-NA	2.10	127.42	124.51
14	AA	1123	CLA	OBD-CAD-CBD	-2.10	122.82	125.91
14	aA	1133	CLA	CHC-C1C-C2C	-2.10	120.91	126.71
18	AA	4011	BCR	C38-C26-C27	2.10	117.51	113.57
14	GA	1132	CLA	C4-C3-C5	2.10	118.91	115.29
14	aA	1133	CLA	CMA-C3A-C4A	2.10	117.42	111.77
18	BA	4005	BCR	C27-C26-C25	-2.10	119.67	122.74
14	aA	1114	CLA	CHA-C1A-NA	-2.10	121.58	126.40
18	bA	4004	BCR	C31-C1-C6	-2.10	106.90	110.30
18	mA	4021	BCR	C2-C1-C6	2.10	113.73	110.48
20	RA	5006	LMG	O8-C28-O10	-2.10	118.19	123.56
18	HA	4010	BCR	C28-C27-C26	-2.10	110.33	113.99
14	BA	1221	CLA	C1-C2-C3	-2.10	122.41	126.04
18	AA	4001	BCR	C33-C5-C4	2.10	117.50	113.57
14	BA	1232	CLA	CHA-C1A-NA	-2.10	121.59	126.40
14	AA	1133	CLA	CHC-C1C-C2C	-2.10	120.92	126.71
18	BA	4012	BCR	C24-C25-C26	-2.10	116.38	121.48

Continued on next page...

PRELIMINARY VALIDATION REPORT

Continued from previous page...

Mol	Chain	Res	Type	Atoms	Z	Observed(°)	Ideal(°)
14	aA	1128	CLA	CMC-C2C-C3C	2.10	131.83	126.11
18	HA	4005	BCR	C1-C6-C5	-2.10	119.66	122.59
14	HA	1226	CLA	C3C-C4C-NC	2.10	112.92	110.57
14	GA	1123	CLA	OBD-CAD-CBD	-2.10	122.83	125.91
18	aA	4001	BCR	C15-C14-C13	-2.10	124.31	127.31
14	HA	1239	CLA	CAC-C3C-C2C	2.10	131.12	127.53
18	HA	4014	BCR	C12-C13-C14	-2.10	115.72	118.94
18	bA	4012	BCR	C24-C25-C26	-2.10	116.39	121.48
14	BA	1235	CLA	CMB-C2B-C1B	-2.10	125.24	128.46
14	bA	1022	CLA	CMB-C2B-C3B	2.10	128.73	124.80
14	bA	1226	CLA	C3C-C4C-NC	2.10	112.92	110.57
14	HA	1202	CLA	CHA-C1A-NA	-2.10	121.60	126.40
18	bA	4005	BCR	C27-C26-C25	-2.10	119.68	122.74
14	AA	1128	CLA	C4-C3-C5	2.10	118.90	115.29
18	HA	4012	BCR	C24-C25-C26	-2.10	116.39	121.48
14	BA	1234	CLA	OBD-CAD-C3D	-2.10	124.33	128.04
18	bA	4010	BCR	C28-C27-C26	-2.10	110.34	113.99
18	HA	4016	BCR	C36-C18-C17	-2.10	119.99	122.92
14	aA	1119	CLA	C4-C3-C5	2.09	118.89	115.29
18	aA	4011	BCR	C38-C26-C27	2.09	117.49	113.57
18	BA	4014	BCR	C12-C13-C14	-2.09	115.73	118.94
18	BA	4004	BCR	C23-C22-C21	2.09	122.15	118.94
14	GA	1108	CLA	O2D-CGD-O1D	-2.09	119.70	123.83
18	bA	4013	BCR	C38-C26-C27	2.09	117.49	113.57
20	IA	5006	LMG	O8-C28-O10	-2.09	118.21	123.56
14	AA	1114	CLA	CHA-C1A-NA	-2.09	121.61	126.40
14	HA	1222	CLA	O1D-CGD-CBD	-2.09	120.14	124.48
14	GA	1104	CLA	O1D-CGD-CBD	-2.09	120.14	124.48
14	BA	1222	CLA	O1D-CGD-CBD	-2.09	120.15	124.48
14	aA	1128	CLA	C4-C3-C5	2.09	118.89	115.29
14	BA	1217	CLA	CBA-CAA-C2A	2.09	120.07	113.85
14	GA	1128	CLA	CMC-C2C-C3C	2.09	131.81	126.11
18	bA	4009	BCR	C1-C6-C5	-2.09	119.67	122.59
14	AA	1129	CLA	O1D-CGD-CBD	-2.09	120.15	124.48
14	BA	1215	CLA	CMC-C2C-C3C	2.09	131.81	126.11
14	GA	1131	CLA	C1-O2A-CGA	2.09	122.25	116.54
14	bA	1217	CLA	CBA-CAA-C2A	2.09	120.07	113.85
14	bA	1234	CLA	CMB-C2B-C3B	2.09	128.72	124.80
14	HA	1234	CLA	CMB-C2B-C3B	2.09	128.72	124.80
13	aA	1011	CL0	CHC-C1C-C2C	-2.09	120.95	126.71
14	bA	1222	CLA	O1D-CGD-CBD	-2.09	120.15	124.48
18	AA	4001	BCR	C15-C14-C13	-2.09	124.33	127.31

Continued on next page...

PRELIMINARY VALIDATION REPORT

Continued from previous page...

Mol	Chain	Res	Type	Atoms	Z	Observed(^{o})	Ideal(^{o})
14	HA	1217	CLA	CBA-CAA-C2A	2.09	120.07	113.85
14	HA	1206	CLA	CMA-C3A-C4A	2.09	117.38	111.77
14	HA	1223	CLA	C4C-C3C-C2C	-2.09	103.87	106.89
14	HA	1214	CLA	O1D-CGD-CBD	-2.09	120.15	124.48
14	aA	1114	CLA	CMC-C2C-C3C	2.09	131.80	126.11
14	GA	1114	CLA	C3C-C4C-NC	2.09	112.91	110.57
14	bA	1211	CLA	CHB-C4A-NA	2.09	127.40	124.51
20	iA	5006	LMG	O8-C28-O10	-2.09	118.23	123.56
14	AA	1131	CLA	O1D-CGD-CBD	-2.09	120.16	124.48
14	aA	1129	CLA	O1D-CGD-CBD	-2.09	120.16	124.48
18	BA	4010	BCR	C28-C27-C26	-2.09	110.36	113.99
14	HA	1205	CLA	OBD-CAD-CBD	-2.09	122.85	125.91
14	AA	1125	CLA	OBD-CAD-CBD	-2.09	122.85	125.91
13	AA	1011	CL0	CHC-C1C-C2C	-2.08	120.96	126.71
18	HA	4004	BCR	C23-C22-C21	2.08	122.14	118.94
14	bA	1223	CLA	C4C-C3C-C2C	-2.08	103.87	106.89
14	AA	1132	CLA	C4-C3-C5	2.08	118.87	115.29
14	BA	1022	CLA	CMB-C2B-C3B	2.08	128.71	124.80
14	bA	1221	CLA	C4C-C3C-C2C	-2.08	103.87	106.89
14	BA	1214	CLA	O1D-CGD-CBD	-2.08	120.17	124.48
14	HA	1215	CLA	CMC-C2C-C3C	2.08	131.79	126.11
14	bA	1233	CLA	OBD-CAD-C3D	-2.08	124.35	128.04
18	aA	4007	BCR	C35-C13-C12	2.08	121.39	118.09
14	aA	1104	CLA	C4-C3-C5	2.08	118.87	115.29
18	GA	4002	BCR	C30-C25-C26	-2.08	119.68	122.59
14	AA	1131	CLA	C1-O2A-CGA	2.08	122.23	116.54
14	WA	1701	CLA	C4C-C3C-C2C	-2.08	103.88	106.89
14	GA	1129	CLA	O1D-CGD-CBD	-2.08	120.17	124.48
18	GA	4001	BCR	C15-C14-C13	-2.08	124.34	127.31
14	0	1012	CLA	O1D-CGD-CBD	-2.08	120.17	124.48
14	bA	1215	CLA	CMC-C2C-C3C	2.08	131.78	126.11
18	AA	4011	BCR	C31-C1-C6	-2.08	106.94	110.30
13	GA	1011	CL0	CHC-C1C-C2C	-2.08	120.98	126.71
14	aA	1131	CLA	C1-O2A-CGA	2.08	122.22	116.54
14	BA	1022	CLA	CHD-C4C-C3C	-2.08	121.84	124.87
14	GA	1132	CLA	CMC-C2C-C1C	2.08	128.19	125.03
14	BA	1211	CLA	CHB-C4A-NA	2.08	127.38	124.51
14	bA	1239	CLA	C4C-C3C-C2C	-2.08	103.88	106.89
14	bA	1234	CLA	OBD-CAD-C3D	-2.08	124.36	128.04
14	aA	1132	CLA	CMC-C2C-C1C	2.08	128.19	125.03
14	aA	1129	CLA	CAC-C3C-C4C	2.07	127.53	124.82
14	aA	1122	CLA	CMC-C2C-C1C	2.07	128.19	125.03

Continued on next page...

PRELIMINARY VALIDATION REPORT

Continued from previous page...

Mol	Chain	Res	Type	Atoms	Z	Observed(°)	Ideal(°)
18	BA	4013	BCR	C38-C26-C27	2.07	117.45	113.57
14	0	1012	CLA	CHD-C4C-C3C	-2.07	121.84	124.87
14	GA	1109	CLA	CHC-C1C-C2C	-2.07	120.99	126.71
14	BA	1218	CLA	O1D-CGD-CBD	-2.07	120.18	124.48
14	GA	1114	CLA	CMC-C2C-C3C	2.07	131.76	126.11
14	bA	1235	CLA	CMB-C2B-C1B	-2.07	125.28	128.46
14	lA	1502	CLA	OBD-CAD-C3D	-2.07	124.37	128.04
18	bA	4014	BCR	C39-C30-C25	-2.07	106.95	110.30
18	AA	4007	BCR	C35-C13-C12	2.07	121.38	118.09
14	0	1012	CLA	CMB-C2B-C3B	2.07	128.69	124.80
18	BA	4016	BCR	C36-C18-C17	-2.07	120.02	122.92
14	AA	1125	CLA	CAC-C3C-C2C	2.07	131.07	127.53
14	kA	1401	CLA	CHC-C1C-C2C	-2.07	121.00	126.71
14	BA	1239	CLA	C4C-C3C-C2C	-2.07	103.89	106.89
14	AA	1104	CLA	C4-C3-C5	2.07	118.85	115.29
18	AA	4002	BCR	C30-C25-C26	-2.07	119.69	122.59
18	HA	4005	BCR	C27-C26-C25	-2.07	119.72	122.74
18	bA	4016	BCR	C36-C18-C17	-2.07	120.02	122.92
14	aA	1131	CLA	O1D-CGD-CBD	-2.07	120.19	124.48
14	AA	1114	CLA	CMC-C2C-C3C	2.07	131.75	126.11
14	GA	1124	CLA	CMC-C2C-C1C	2.07	128.18	125.03
14	aA	1125	CLA	CAC-C3C-C2C	2.07	131.06	127.53
14	GA	1104	CLA	C4-C3-C5	2.07	118.85	115.29
14	AA	1124	CLA	CMC-C2C-C1C	2.07	128.18	125.03
14	GA	1129	CLA	CMA-C3A-C4A	2.07	117.33	111.77
18	bA	4010	BCR	C37-C22-C21	-2.07	120.03	122.92
14	AA	1104	CLA	O1D-CGD-CBD	-2.07	120.20	124.48
18	GA	4007	BCR	C8-C7-C6	-2.07	121.49	127.28
14	AA	1132	CLA	CMC-C2C-C1C	2.07	128.17	125.03
14	bA	1022	CLA	CHD-C4C-C3C	-2.06	121.86	124.87
14	HA	1218	CLA	O1D-CGD-CBD	-2.06	120.20	124.48
18	AA	4007	BCR	C8-C7-C6	-2.06	121.50	127.28
14	AA	1109	CLA	CHC-C1C-C2C	-2.06	121.02	126.71
14	BA	1226	CLA	C3C-C4C-NC	2.06	112.89	110.57
14	AA	1129	CLA	CMA-C3A-C4A	2.06	117.32	111.77
14	bA	1218	CLA	O1D-CGD-CBD	-2.06	120.20	124.48
14	aA	1129	CLA	CMA-C3A-C4A	2.06	117.32	111.77
18	HA	4013	BCR	C38-C26-C27	2.06	117.43	113.57
18	GA	4011	BCR	C31-C1-C6	-2.06	106.97	110.30
14	aA	1135	CLA	C5-C3-C4	2.06	119.22	114.59
14	TA	1401	CLA	CHC-C1C-C2C	-2.06	121.03	126.71
14	bA	1214	CLA	CHA-C1A-NA	-2.06	121.68	126.40

Continued on next page...

PRELIMINARY VALIDATION REPORT

Continued from previous page...

Mol	Chain	Res	Type	Atoms	Z	Observed(^{o})	Ideal(^{o})
14	aA	1125	CLA	O1D-CGD-CBD	-2.06	120.21	124.48
18	GA	4011	BCR	C38-C26-C27	2.06	117.43	113.57
14	aA	1130	CLA	CHA-C1A-NA	-2.06	121.68	126.40
13	GA	1011	CL0	OBD-CAD-C3D	-2.06	124.39	128.04
14	BA	1233	CLA	OBD-CAD-C3D	-2.06	124.39	128.04
14	HA	1222	CLA	CAC-C3C-C4C	2.06	127.51	124.82
13	aA	1011	CL0	OBD-CAD-C3D	-2.06	124.39	128.04
14	aA	1109	CLA	CHC-C1C-C2C	-2.06	121.04	126.71
14	AA	1135	CLA	C5-C3-C4	2.06	119.21	114.59
14	AA	1129	CLA	CAC-C3C-C4C	2.06	127.51	124.82
14	BA	1214	CLA	CHA-C1A-NA	-2.06	121.69	126.40
14	AA	1112	CLA	CHC-C1C-C2C	-2.06	121.04	126.71
14	aA	1112	CLA	CHC-C1C-C2C	-2.06	121.04	126.71
14	GA	1128	CLA	C4-C3-C5	2.06	118.83	115.29
21	AA	6002	LMT	C2'-C3'-C4'	2.05	114.37	109.67
14	AA	1124	CLA	C4-C3-C5	2.05	118.83	115.29
14	HA	1214	CLA	CHA-C1A-NA	-2.05	121.69	126.40
14	BA	1022	CLA	O1D-CGD-CBD	-2.05	120.22	124.48
14	HA	1235	CLA	CMB-C2B-C1B	-2.05	125.31	128.46
18	BA	4016	BCR	C38-C26-C27	2.05	117.41	113.57
18	bA	4010	BCR	C35-C13-C12	2.05	121.34	118.09
14	AA	1108	CLA	O2D-CGD-O1D	-2.05	119.78	123.83
14	AA	1118	CLA	O1D-CGD-CBD	-2.05	120.23	124.48
18	HA	4010	BCR	C37-C22-C21	-2.05	120.05	122.92
13	GA	1011	CL0	CHB-C4A-NA	2.05	127.35	124.51
14	aA	1124	CLA	CMC-C2C-C1C	2.05	128.15	125.03
18	aA	4007	BCR	C8-C7-C6	-2.05	121.54	127.28
14	GA	1123	CLA	CHA-C1A-NA	-2.05	121.70	126.40
18	GA	4003	BCR	C34-C9-C10	-2.05	120.05	122.92
14	aA	1137	CLA	CHC-C1C-C2C	-2.05	121.06	126.71
14	KA	1401	CLA	CHC-C1C-C2C	-2.05	121.06	126.71
14	bA	1023	CLA	CMA-C3A-C4A	2.05	117.28	111.77
21	aA	6002	LMT	C2'-C3'-C4'	2.05	114.35	109.67
14	bA	1232	CLA	OBD-CAD-C3D	-2.05	124.41	128.04
14	AA	1140	CLA	C1-O2A-CGA	2.05	122.14	116.54
18	HA	4014	BCR	C39-C30-C25	-2.05	106.99	110.30
14	AA	1114	CLA	CMB-C2B-C1B	-2.05	125.32	128.46
18	BA	4010	BCR	C35-C13-C12	2.05	121.34	118.09
14	UA	1502	CLA	CBC-CAC-C3C	-2.05	106.75	112.43
14	AA	1137	CLA	CHC-C1C-C2C	-2.05	121.07	126.71
18	bA	4012	BCR	C4-C5-C6	-2.05	119.75	122.74
14	BA	1221	CLA	CGD-CBD-CAD	-2.05	104.10	110.73

Continued on next page...

PRELIMINARY VALIDATION REPORT

Continued from previous page...

Mol	Chain	Res	Type	Atoms	Z	Observed(^{o})	Ideal(^{o})
14	HA	1232	CLA	OBD-CAD-C3D	-2.05	124.41	128.04
14	GA	1122	CLA	CMC-C2C-C1C	2.05	128.15	125.03
14	HA	1211	CLA	CHB-C4A-NA	2.05	127.34	124.51
14	GA	1125	CLA	CAC-C3C-C2C	2.05	131.03	127.53
14	GA	1130	CLA	CHA-C1A-NA	-2.05	121.71	126.40
14	BA	1221	CLA	C4C-C3C-C2C	-2.05	103.93	106.89
14	BA	1239	CLA	C6-C7-C8	-2.05	109.39	115.77
14	BA	1206	CLA	C4C-C3C-C2C	-2.05	103.93	106.89
14	bA	1239	CLA	C6-C7-C8	-2.05	109.39	115.77
14	BA	1234	CLA	CMB-C2B-C3B	2.04	128.63	124.80
14	aA	1108	CLA	O2D-CGD-O1D	-2.04	119.79	123.83
14	HA	1239	CLA	C4C-C3C-C2C	-2.04	103.93	106.89
14	GA	1137	CLA	CHC-C1C-C2C	-2.04	121.08	126.71
14	BA	1205	CLA	C4C-C3C-C2C	-2.04	103.93	106.89
14	GA	1135	CLA	C5-C3-C4	2.04	119.18	114.59
14	GA	1114	CLA	CMB-C2B-C1B	-2.04	125.33	128.46
14	aA	1140	CLA	C1-O2A-CGA	2.04	122.12	116.54
14	BA	1216	CLA	O2D-CGD-O1D	-2.04	119.80	123.83
14	GA	1101	CLA	C4C-C3C-C2C	-2.04	103.93	106.89
14	bA	1233	CLA	CHC-C1C-C2C	-2.04	121.08	126.71
14	BA	1228	CLA	CMC-C2C-C3C	2.04	131.67	126.11
14	HA	1202	CLA	CAA-C2A-C3A	-2.04	107.19	112.78
14	AA	1130	CLA	CHA-C1A-NA	-2.04	121.72	126.40
14	lA	1502	CLA	CBC-CAC-C3C	-2.04	106.76	112.43
14	aA	1109	CLA	OBD-CAD-C3D	-2.04	124.43	128.04
14	HA	1221	CLA	C4C-C3C-C2C	-2.04	103.94	106.89
14	HA	1238	CLA	CAA-CBA-CGA	-2.04	107.25	113.26
14	HA	1233	CLA	OBD-CAD-C3D	-2.04	124.43	128.04
13	AA	1011	CL0	OBD-CAD-C3D	-2.04	124.43	128.04
14	GA	1140	CLA	C1-O2A-CGA	2.04	122.11	116.54
18	aA	4011	BCR	C31-C1-C6	-2.04	107.01	110.30
18	GA	4002	BCR	C23-C24-C25	-2.04	121.57	127.28
14	HA	1216	CLA	O1D-CGD-CBD	-2.04	120.26	124.48
14	GA	1118	CLA	O1D-CGD-CBD	-2.04	120.26	124.48
14	BA	1023	CLA	CMA-C3A-C4A	2.04	117.25	111.77
14	HA	1239	CLA	C6-C7-C8	-2.04	109.42	115.77
14	HA	1204	CLA	C3C-C4C-NC	2.04	112.85	110.57
14	GA	1109	CLA	OBD-CAD-C3D	-2.04	124.43	128.04
14	bA	1205	CLA	C4C-C3C-C2C	-2.03	103.94	106.89
14	GA	1138	CLA	OBD-CAD-C3D	-2.03	124.44	128.04
14	GA	1013	CLA	CMD-C2D-C3D	2.03	128.62	124.80
14	bA	1238	CLA	CAA-CBA-CGA	-2.03	107.26	113.26

Continued on next page...

PRELIMINARY VALIDATION REPORT

Continued from previous page...

Mol	Chain	Res	Type	Atoms	Z	Observed(^{o})	Ideal(^{o})
14	LA	1502	CLA	CBC-CAC-C3C	-2.03	106.78	112.43
18	BA	4012	BCR	C4-C5-C6	-2.03	119.77	122.74
18	bA	4005	BCR	C19-C18-C17	2.03	122.06	118.94
14	HA	1221	CLA	CGD-CBD-CAD	-2.03	104.15	110.73
14	BA	1238	CLA	CAA-CBA-CGA	-2.03	107.27	113.26
14	GA	1112	CLA	CHC-C1C-C2C	-2.03	121.11	126.71
14	HA	1227	CLA	CHC-C1C-C2C	-2.03	121.11	126.71
18	aA	4003	BCR	C34-C9-C10	-2.03	120.08	122.92
14	AA	1123	CLA	CHA-C1A-NA	-2.03	121.74	126.40
14	bA	1228	CLA	CMC-C2C-C3C	2.03	131.65	126.11
14	aA	1101	CLA	C4C-C3C-C2C	-2.03	103.95	106.89
14	BA	1222	CLA	CAC-C3C-C4C	2.03	127.48	124.82
14	bA	1202	CLA	CAA-C2A-C3A	-2.03	107.21	112.78
21	GA	6002	LMT	C2'-C3'-C4'	2.03	114.32	109.67
14	aA	1123	CLA	CHA-C1A-NA	-2.03	121.75	126.40
14	bA	1221	CLA	CGD-CBD-CAD	-2.03	104.16	110.73
18	AA	4003	BCR	C34-C9-C10	-2.03	120.08	122.92
14	bA	1227	CLA	CHC-C1C-C2C	-2.03	121.11	126.71
14	HA	1228	CLA	CMC-C2C-C3C	2.03	131.65	126.11
14	BA	1215	CLA	C1-O2A-CGA	2.03	122.09	116.54
14	BA	1233	CLA	CHC-C1C-C2C	-2.03	121.11	126.71
14	BA	1227	CLA	CHC-C1C-C2C	-2.03	121.11	126.71
14	BA	1202	CLA	CAA-C2A-C3A	-2.03	107.22	112.78
14	AA	1101	CLA	C4C-C3C-C2C	-2.03	103.95	106.89
14	GA	1013	CLA	C6-C7-C8	-2.03	109.44	115.77
14	aA	1118	CLA	O1D-CGD-CBD	-2.03	120.28	124.48
14	bA	1216	CLA	O2D-CGD-O1D	-2.03	119.83	123.83
14	bA	1206	CLA	C4C-C3C-C2C	-2.03	103.95	106.89
14	bA	1023	CLA	OBD-CAD-C3D	-2.03	124.45	128.04
14	bA	1204	CLA	C3C-C4C-NC	2.03	112.84	110.57
14	BA	1206	CLA	CAA-C2A-C3A	-2.03	107.23	112.78
14	HA	1205	CLA	C4C-C3C-C2C	-2.03	103.96	106.89
14	HA	1023	CLA	CMA-C3A-C4A	2.03	117.22	111.77
18	BA	4014	BCR	C39-C30-C25	-2.02	107.03	110.30
14	bA	1202	CLA	CHB-C4A-NA	2.02	127.31	124.51
18	BA	4013	BCR	C23-C22-C21	2.02	122.05	118.94
18	bA	4016	BCR	C38-C26-C27	2.02	117.36	113.57
14	AA	1013	CLA	C6-C7-C8	-2.02	109.46	115.77
18	HA	4012	BCR	C4-C5-C6	-2.02	119.79	122.74
14	GA	1106	CLA	C4C-C3C-C2C	-2.02	103.96	106.89
14	BA	1232	CLA	OBD-CAD-C3D	-2.02	124.46	128.04
14	aA	1114	CLA	CMB-C2B-C1B	-2.02	125.36	128.46

Continued on next page...

PRELIMINARY VALIDATION REPORT

Continued from previous page...

Mol	Chain	Res	Type	Atoms	Z	Observed(°)	Ideal(°)
18	RA	4020	BCR	C28-C29-C30	-2.02	107.42	114.55
14	HA	1233	CLA	CHC-C1C-C2C	-2.02	121.14	126.71
14	aA	1013	CLA	C6-C7-C8	-2.02	109.46	115.77
14	GA	1125	CLA	CHD-C4C-C3C	-2.02	121.92	124.87
14	AA	1122	CLA	CMC-C2C-C1C	2.02	128.11	125.03
14	bA	1232	CLA	O2D-CGD-O1D	-2.02	119.84	123.83
14	HA	1206	CLA	C4C-C3C-C2C	-2.02	103.97	106.89
18	HA	4010	BCR	C35-C13-C12	2.02	121.29	118.09
14	HA	1023	CLA	OBD-CAD-C3D	-2.02	124.46	128.04
21	aA	6001	LMT	O1'-C1'-C2'	2.02	111.48	108.26
14	AA	1109	CLA	CAC-C3C-C4C	2.02	127.46	124.82
14	HA	1216	CLA	O2D-CGD-O1D	-2.02	119.85	123.83
14	AA	1109	CLA	OBD-CAD-C3D	-2.02	124.47	128.04
14	lA	1503	CLA	C6-C5-C3	-2.02	108.16	113.01
14	BA	1202	CLA	CAC-C3C-C4C	2.02	127.46	124.82
14	BA	1204	CLA	O1D-CGD-CBD	-2.02	120.30	124.48
14	BA	1216	CLA	O1D-CGD-CBD	-2.02	120.30	124.48
14	BA	1204	CLA	C3C-C4C-NC	2.02	112.83	110.57
14	GA	1109	CLA	CAC-C3C-C4C	2.02	127.46	124.82
14	bA	1206	CLA	CAA-C2A-C3A	-2.02	107.25	112.78
14	aA	1124	CLA	C4-C3-C5	2.02	118.76	115.29
14	GA	1124	CLA	C4-C3-C5	2.02	118.76	115.29
14	HA	1206	CLA	CAA-C2A-C3A	-2.02	107.26	112.78
14	bA	1204	CLA	O1D-CGD-CBD	-2.02	120.30	124.48
14	GA	1135	CLA	CHC-C1C-C2C	-2.02	121.15	126.71
14	aA	1125	CLA	CHD-C4C-C3C	-2.02	121.93	124.87
14	BA	1202	CLA	CHB-C4A-NA	2.02	127.30	124.51
14	bA	1216	CLA	O1D-CGD-CBD	-2.02	120.30	124.48
14	bA	1222	CLA	CAC-C3C-C4C	2.01	127.45	124.82
14	HA	1204	CLA	O1D-CGD-CBD	-2.01	120.31	124.48
18	iA	4020	BCR	C28-C29-C30	-2.01	107.44	114.55
14	UA	1503	CLA	C6-C5-C3	-2.01	108.17	113.01
14	GA	1013	CLA	OBD-CAD-C3D	-2.01	124.47	128.04
18	HA	4009	BCR	C4-C5-C6	-2.01	119.80	122.74
14	BA	1223	CLA	CHD-C4C-C3C	-2.01	121.93	124.87
14	HA	1215	CLA	C1-O2A-CGA	2.01	122.05	116.54
14	aA	1112	CLA	O1D-CGD-CBD	-2.01	120.31	124.48
14	xA	1701	CLA	CHD-C4C-C3C	-2.01	121.93	124.87
14	BA	1216	CLA	C4C-C3C-C2C	-2.01	103.97	106.89
14	aA	1106	CLA	C4C-C3C-C2C	-2.01	103.97	106.89
14	aA	1013	CLA	OBD-CAD-C3D	-2.01	124.47	128.04
18	aA	4002	BCR	C30-C25-C26	-2.01	119.78	122.59

Continued on next page...

PRELIMINARY VALIDATION REPORT

Continued from previous page...

Mol	Chain	Res	Type	Atoms	Z	Observed(°)	Ideal(°)
14	AA	1102	CLA	O1D-CGD-CBD	-2.01	120.31	124.48
14	HA	1223	CLA	CHD-C4C-C3C	-2.01	121.94	124.87
14	AA	1013	CLA	CMD-C2D-C3D	2.01	128.57	124.80
14	LA	1503	CLA	C6-C5-C3	-2.01	108.18	113.01
18	IA	4020	BCR	C28-C29-C30	-2.01	107.46	114.55
18	aA	4002	BCR	C23-C24-C25	-2.01	121.65	127.28
14	bA	1202	CLA	CAC-C3C-C4C	2.01	127.45	124.82
14	HA	1210	CLA	C1-C2-C3	-2.01	122.57	126.04
14	BA	1232	CLA	O2D-CGD-O1D	-2.01	119.86	123.83
14	AA	1125	CLA	O1D-CGD-CBD	-2.01	120.32	124.48
18	bA	4014	BCR	C19-C18-C17	2.01	122.02	118.94
18	HA	4014	BCR	C19-C18-C17	2.01	122.02	118.94
14	aA	1013	CLA	CMD-C2D-C3D	2.01	128.57	124.80
18	HA	4016	BCR	C38-C26-C27	2.01	117.33	113.57
14	XA	1701	CLA	OBD-CAD-C3D	-2.01	124.48	128.04
13	aA	1011	CL0	CHB-C4A-NA	2.01	127.29	124.51
14	GA	1125	CLA	O1D-CGD-CBD	-2.01	120.32	124.48
18	AA	4002	BCR	C23-C24-C25	-2.01	121.66	127.28
14	HA	1216	CLA	C4C-C3C-C2C	-2.01	103.99	106.89
14	bA	1214	CLA	C4-C3-C2	-2.00	118.47	123.68
14	AA	1123	CLA	CAC-C3C-C4C	2.00	127.44	124.82
14	bA	1215	CLA	O1D-CGD-CBD	-2.00	120.33	124.48
14	aA	1133	CLA	CMB-C2B-C3B	2.00	128.56	124.80
14	GA	1133	CLA	CMB-C2B-C3B	2.00	128.56	124.80
14	aA	1135	CLA	OBD-CAD-CBD	-2.00	122.97	125.91
18	HA	4005	BCR	C19-C18-C17	2.00	122.02	118.94
14	AA	1106	CLA	C4C-C3C-C2C	-2.00	103.99	106.89
21	aA	6002	LMT	O1B-C4'-C5'	-2.00	103.92	109.43
14	AA	1135	CLA	OBD-CAD-CBD	-2.00	122.97	125.91
14	HA	1232	CLA	O2D-CGD-O1D	-2.00	119.88	123.83
14	XA	1701	CLA	CHD-C4C-C3C	-2.00	121.95	124.87
14	BA	1214	CLA	C4-C3-C2	-2.00	118.47	123.68
14	GA	1131	CLA	CHB-C4A-NA	2.00	127.28	124.51
14	bA	1209	CLA	CAC-C3C-C4C	2.00	127.44	124.82
14	GA	1112	CLA	O1D-CGD-CBD	-2.00	120.33	124.48
18	HA	4009	BCR	C1-C6-C7	2.00	121.47	115.78
14	BA	1023	CLA	OBD-CAD-C3D	-2.00	124.50	128.04
14	AA	1013	CLA	OBD-CAD-C3D	-2.00	124.50	128.04

All (759) chirality outliers are listed below:

Continued on next page...

PRELIMINARY VALIDATION REPORT

Continued from previous page...

Mol	Chain	Res	Type	Atom
14	LA	1501	CLA	NC
14	LA	1501	CLA	ND
14	LA	1501	CLA	NA
14	AA	1115	CLA	NC
14	AA	1115	CLA	ND
14	AA	1115	CLA	NA
14	bA	1234	CLA	NC
14	bA	1234	CLA	ND
14	bA	1234	CLA	NA
14	bA	1022	CLA	NC
14	bA	1022	CLA	ND
14	bA	1022	CLA	NA
14	BA	1225	CLA	NC
14	BA	1225	CLA	ND
14	BA	1225	CLA	NA
14	UA	1502	CLA	NC
14	UA	1502	CLA	ND
14	UA	1502	CLA	NA
14	GA	1116	CLA	NC
14	GA	1116	CLA	ND
14	GA	1116	CLA	NA
14	bA	1211	CLA	NC
14	bA	1211	CLA	ND
14	bA	1211	CLA	NA
14	BA	1228	CLA	NC
14	BA	1228	CLA	ND
14	BA	1228	CLA	NA
13	GA	1011	CL0	NC
13	GA	1011	CL0	ND
13	GA	1011	CL0	NA
14	GA	1103	CLA	ND
14	GA	1103	CLA	NA
14	GA	1111	CLA	NC
14	GA	1111	CLA	ND
14	GA	1111	CLA	NA
14	aA	1119	CLA	NC
14	aA	1119	CLA	ND
14	aA	1119	CLA	NA
14	WA	1701	CLA	NC
14	WA	1701	CLA	ND
14	WA	1701	CLA	NA

Continued on next page...

PRELIMINARY VALIDATION REPORT

Continued from previous page...

Mol	Chain	Res	Type	Atom
14	AA	1118	CLA	NC
14	AA	1118	CLA	ND
14	AA	1118	CLA	NA
14	HA	1225	CLA	NC
14	HA	1225	CLA	ND
14	HA	1225	CLA	NA
14	aA	1124	CLA	NC
14	aA	1124	CLA	ND
14	aA	1124	CLA	NA
14	HA	1208	CLA	NC
14	HA	1208	CLA	ND
14	HA	1208	CLA	NA
14	aA	1102	CLA	NC
14	aA	1102	CLA	ND
14	aA	1102	CLA	NA
14	HA	1215	CLA	NC
14	HA	1215	CLA	ND
14	HA	1215	CLA	NA
14	bA	1221	CLA	NC
14	bA	1221	CLA	ND
14	bA	1221	CLA	NA
14	bA	1231	CLA	NC
14	bA	1231	CLA	ND
14	bA	1231	CLA	NA
14	HA	1216	CLA	NC
14	HA	1216	CLA	ND
14	HA	1216	CLA	NA
14	BA	1219	CLA	NC
14	BA	1219	CLA	ND
14	BA	1219	CLA	NA
14	bA	1214	CLA	NC
14	bA	1214	CLA	ND
14	bA	1214	CLA	NA
14	aA	1123	CLA	NC
14	aA	1123	CLA	ND
14	aA	1123	CLA	NA
14	AA	1135	CLA	NC
14	AA	1135	CLA	ND
14	AA	1135	CLA	NA
14	GA	1101	CLA	NC
14	GA	1101	CLA	ND
14	GA	1101	CLA	NA

Continued on next page...

PRELIMINARY VALIDATION REPORT

Continued from previous page...

Mol	Chain	Res	Type	Atom
14	GA	1137	CLA	NC
14	GA	1137	CLA	ND
14	GA	1137	CLA	NA
14	bA	1223	CLA	NC
14	bA	1223	CLA	ND
14	bA	1223	CLA	NA
14	BA	1212	CLA	NC
14	BA	1212	CLA	ND
14	BA	1212	CLA	NA
14	BA	1238	CLA	NC
14	BA	1238	CLA	ND
14	BA	1238	CLA	NA
14	BA	1217	CLA	NC
14	BA	1217	CLA	ND
14	BA	1217	CLA	NA
14	AA	1140	CLA	NC
14	AA	1140	CLA	ND
14	AA	1140	CLA	NA
14	aA	1012	CLA	NC
14	aA	1012	CLA	NA
14	aA	1012	CLA	ND
14	AA	1122	CLA	NC
14	AA	1122	CLA	ND
14	AA	1122	CLA	NA
14	BA	1204	CLA	NC
14	BA	1204	CLA	ND
14	BA	1204	CLA	NA
14	aA	1133	CLA	NC
14	aA	1133	CLA	ND
14	aA	1133	CLA	NA
14	aA	1107	CLA	NC
14	aA	1107	CLA	ND
14	aA	1107	CLA	NA
14	AA	1124	CLA	NC
14	AA	1124	CLA	ND
14	AA	1124	CLA	NA
14	bA	1225	CLA	NC
14	bA	1225	CLA	ND
14	bA	1225	CLA	NA
14	AA	1132	CLA	NC
14	AA	1132	CLA	ND
14	AA	1132	CLA	NA

Continued on next page...

PRELIMINARY VALIDATION REPORT

Continued from previous page...

Mol	Chain	Res	Type	Atom
14	GA	1108	CLA	NC
14	GA	1108	CLA	ND
14	GA	1108	CLA	NA
14	bA	1226	CLA	NC
14	bA	1226	CLA	ND
14	bA	1226	CLA	NA
14	AA	1106	CLA	NC
14	AA	1106	CLA	ND
14	AA	1106	CLA	NA
14	AA	1138	CLA	NC
14	AA	1138	CLA	ND
14	AA	1138	CLA	NA
14	AA	1133	CLA	NC
14	AA	1133	CLA	ND
14	AA	1133	CLA	NA
14	HA	1231	CLA	NC
14	HA	1231	CLA	ND
14	HA	1231	CLA	NA
14	HA	1219	CLA	NC
14	HA	1219	CLA	ND
14	HA	1219	CLA	NA
14	BA	1221	CLA	NC
14	BA	1221	CLA	ND
14	BA	1221	CLA	NA
14	bA	1210	CLA	NC
14	bA	1210	CLA	ND
14	bA	1210	CLA	NA
14	bA	1204	CLA	NC
14	bA	1204	CLA	ND
14	bA	1204	CLA	NA
14	aA	1135	CLA	NC
14	aA	1135	CLA	ND
14	aA	1135	CLA	NA
14	HA	1229	CLA	NC
14	HA	1229	CLA	ND
14	HA	1229	CLA	NA
14	aA	1114	CLA	NC
14	aA	1114	CLA	ND
14	aA	1114	CLA	NA
14	HA	1222	CLA	NC
14	HA	1222	CLA	ND
14	HA	1222	CLA	NA

Continued on next page...

PRELIMINARY VALIDATION REPORT

Continued from previous page...

Mol	Chain	Res	Type	Atom
14	HA	1209	CLA	NC
14	HA	1209	CLA	ND
14	HA	1209	CLA	NA
14	BA	1235	CLA	NC
14	BA	1235	CLA	ND
14	BA	1235	CLA	NA
14	aA	1101	CLA	NC
14	aA	1101	CLA	ND
14	aA	1101	CLA	NA
14	HA	1233	CLA	NC
14	HA	1233	CLA	ND
14	HA	1233	CLA	NA
14	aA	1108	CLA	NC
14	aA	1108	CLA	ND
14	aA	1108	CLA	NA
14	GA	1140	CLA	NC
14	GA	1140	CLA	ND
14	GA	1140	CLA	NA
14	bA	1217	CLA	NC
14	bA	1217	CLA	ND
14	bA	1217	CLA	NA
14	AA	1127	CLA	NC
14	AA	1127	CLA	ND
14	AA	1127	CLA	NA
14	bA	1236	CLA	NC
14	bA	1236	CLA	ND
14	bA	1236	CLA	NA
14	GA	1102	CLA	NC
14	GA	1102	CLA	ND
14	GA	1102	CLA	NA
13	AA	1011	CL0	NC
13	AA	1011	CL0	ND
13	AA	1011	CL0	NA
14	aA	1122	CLA	NC
14	aA	1122	CLA	ND
14	aA	1122	CLA	NA
14	AA	1112	CLA	NC
14	AA	1112	CLA	ND
14	AA	1112	CLA	NA
14	AA	1101	CLA	NC
14	AA	1101	CLA	ND
14	AA	1101	CLA	NA

Continued on next page...

PRELIMINARY VALIDATION REPORT

Continued from previous page...

Mol	Chain	Res	Type	Atom
14	GA	1114	CLA	NC
14	GA	1114	CLA	ND
14	GA	1114	CLA	NA
14	GA	1107	CLA	NC
14	GA	1107	CLA	ND
14	GA	1107	CLA	NA
14	HA	1224	CLA	ND
14	HA	1224	CLA	NA
14	aA	1117	CLA	NC
14	aA	1117	CLA	ND
14	aA	1117	CLA	NA
14	HA	1205	CLA	NC
14	HA	1205	CLA	ND
14	HA	1205	CLA	NA
14	aA	1128	CLA	NC
14	aA	1128	CLA	ND
14	aA	1128	CLA	NA
14	aA	1125	CLA	NC
14	aA	1125	CLA	ND
14	aA	1125	CLA	NA
14	BA	1223	CLA	NC
14	BA	1223	CLA	ND
14	BA	1223	CLA	NA
14	aA	1115	CLA	NC
14	aA	1115	CLA	ND
14	aA	1115	CLA	NA
14	bA	1023	CLA	NC
14	bA	1023	CLA	ND
14	bA	1023	CLA	NA
14	AA	1114	CLA	NC
14	AA	1114	CLA	ND
14	AA	1114	CLA	NA
14	aA	1104	CLA	NC
14	aA	1104	CLA	ND
14	aA	1104	CLA	NA
14	aA	1132	CLA	NC
14	aA	1132	CLA	ND
14	aA	1132	CLA	NA
14	bA	1205	CLA	NC
14	bA	1205	CLA	ND
14	bA	1205	CLA	NA
14	GA	1113	CLA	NC

Continued on next page...

PRELIMINARY VALIDATION REPORT

Continued from previous page...

Mol	Chain	Res	Type	Atom
14	GA	1113	CLA	ND
14	GA	1113	CLA	NA
14	BA	1208	CLA	NC
14	BA	1208	CLA	ND
14	BA	1208	CLA	NA
14	HA	1021	CLA	NC
14	HA	1021	CLA	ND
14	HA	1021	CLA	NA
14	HA	1228	CLA	NC
14	HA	1228	CLA	ND
14	HA	1228	CLA	NA
14	HA	1207	CLA	NC
14	HA	1207	CLA	ND
14	HA	1207	CLA	NA
14	BA	1220	CLA	NC
14	BA	1220	CLA	ND
14	BA	1220	CLA	NA
14	GA	1105	CLA	NC
14	GA	1105	CLA	ND
14	GA	1105	CLA	NA
14	GA	1127	CLA	NC
14	GA	1127	CLA	ND
14	GA	1127	CLA	NA
14	bA	1218	CLA	NC
14	bA	1218	CLA	ND
14	bA	1218	CLA	NA
14	aA	1130	CLA	NC
14	aA	1130	CLA	ND
14	aA	1130	CLA	NA
14	GA	1128	CLA	NC
14	GA	1128	CLA	ND
14	GA	1128	CLA	NA
14	HA	1239	CLA	NC
14	HA	1239	CLA	ND
14	HA	1239	CLA	NA
14	HA	1214	CLA	NC
14	HA	1214	CLA	ND
14	HA	1214	CLA	NA
14	AA	1128	CLA	NC
14	AA	1128	CLA	ND
14	AA	1128	CLA	NA
14	0	1012	CLA	NC

Continued on next page...

PRELIMINARY VALIDATION REPORT

Continued from previous page...

Mol	Chain	Res	Type	Atom
14	0	1012	CLA	ND
14	0	1012	CLA	NA
14	bA	1216	CLA	NC
14	bA	1216	CLA	ND
14	bA	1216	CLA	NA
14	AA	1107	CLA	NC
14	AA	1107	CLA	ND
14	AA	1107	CLA	NA
14	BA	1236	CLA	NC
14	BA	1236	CLA	ND
14	BA	1236	CLA	NA
14	BA	1213	CLA	NC
14	BA	1213	CLA	ND
14	BA	1213	CLA	NA
14	GA	1125	CLA	NC
14	GA	1125	CLA	ND
14	GA	1125	CLA	NA
14	AA	1108	CLA	NC
14	AA	1108	CLA	ND
14	AA	1108	CLA	NA
14	HA	1201	CLA	NC
14	HA	1201	CLA	ND
14	HA	1201	CLA	NA
14	HA	1202	CLA	NC
14	HA	1202	CLA	ND
14	HA	1202	CLA	NA
14	aA	1109	CLA	NC
14	aA	1109	CLA	ND
14	aA	1109	CLA	NA
14	lA	1503	CLA	NC
14	lA	1503	CLA	ND
14	lA	1503	CLA	NA
14	aA	1103	CLA	ND
14	aA	1103	CLA	NA
14	lA	1501	CLA	NC
14	lA	1501	CLA	ND
14	lA	1501	CLA	NA
14	aA	1140	CLA	NC
14	aA	1140	CLA	ND
14	aA	1140	CLA	NA
14	BA	1231	CLA	NC
14	BA	1231	CLA	ND

Continued on next page...

PRELIMINARY VALIDATION REPORT

Continued from previous page...

Mol	Chain	Res	Type	Atom
14	BA	1231	CLA	NA
14	GA	1110	CLA	NC
14	GA	1110	CLA	ND
14	GA	1110	CLA	NA
14	GA	1138	CLA	NC
14	GA	1138	CLA	ND
14	GA	1138	CLA	NA
14	GA	1139	CLA	NC
14	GA	1139	CLA	ND
14	GA	1139	CLA	NA
14	aA	1136	CLA	NC
14	aA	1136	CLA	ND
14	aA	1136	CLA	NA
14	GA	1117	CLA	NC
14	GA	1117	CLA	ND
14	GA	1117	CLA	NA
14	BA	1232	CLA	NC
14	BA	1232	CLA	ND
14	BA	1232	CLA	NA
14	AA	1103	CLA	ND
14	AA	1103	CLA	NA
14	aA	1112	CLA	NC
14	aA	1112	CLA	ND
14	aA	1112	CLA	NA
14	GA	1106	CLA	NC
14	GA	1106	CLA	ND
14	GA	1106	CLA	NA
14	AA	1129	CLA	NC
14	AA	1129	CLA	ND
14	AA	1129	CLA	NA
14	AA	1123	CLA	NC
14	AA	1123	CLA	ND
14	AA	1123	CLA	NA
14	BA	1021	CLA	NC
14	BA	1021	CLA	ND
14	BA	1021	CLA	NA
14	GA	1131	CLA	NC
14	GA	1131	CLA	ND
14	GA	1131	CLA	NA
14	bA	1229	CLA	NC
14	bA	1229	CLA	ND
14	bA	1229	CLA	NA

Continued on next page...

PRELIMINARY VALIDATION REPORT

Continued from previous page...

Mol	Chain	Res	Type	Atom
14	AA	1116	CLA	NC
14	AA	1116	CLA	ND
14	AA	1116	CLA	NA
14	aA	1106	CLA	NC
14	aA	1106	CLA	ND
14	aA	1106	CLA	NA
14	BA	1209	CLA	NC
14	BA	1209	CLA	ND
14	BA	1209	CLA	NA
14	GA	1129	CLA	NC
14	GA	1129	CLA	ND
14	GA	1129	CLA	NA
14	bA	1233	CLA	NC
14	bA	1233	CLA	ND
14	bA	1233	CLA	NA
14	AA	1137	CLA	NC
14	AA	1137	CLA	ND
14	AA	1137	CLA	NA
14	GA	1133	CLA	NC
14	GA	1133	CLA	ND
14	GA	1133	CLA	NA
14	AA	1111	CLA	NC
14	AA	1111	CLA	ND
14	AA	1111	CLA	NA
14	HA	1203	CLA	NC
14	HA	1203	CLA	ND
14	HA	1203	CLA	NA
14	HA	1234	CLA	NC
14	HA	1234	CLA	ND
14	HA	1234	CLA	NA
14	aA	1139	CLA	NC
14	aA	1139	CLA	ND
14	aA	1139	CLA	NA
14	BA	1022	CLA	NC
14	BA	1022	CLA	ND
14	BA	1022	CLA	NA
14	BA	1216	CLA	NC
14	BA	1216	CLA	ND
14	BA	1216	CLA	NA
14	GA	1115	CLA	NC
14	GA	1115	CLA	ND
14	GA	1115	CLA	NA

Continued on next page...

PRELIMINARY VALIDATION REPORT

Continued from previous page...

Mol	Chain	Res	Type	Atom
14	AA	1136	CLA	NC
14	AA	1136	CLA	ND
14	AA	1136	CLA	NA
14	aA	1126	CLA	NC
14	aA	1126	CLA	ND
14	aA	1126	CLA	NA
14	bA	1238	CLA	NC
14	bA	1238	CLA	ND
14	bA	1238	CLA	NA
14	BA	1205	CLA	NC
14	BA	1205	CLA	ND
14	BA	1205	CLA	NA
14	UA	1501	CLA	NC
14	UA	1501	CLA	ND
14	UA	1501	CLA	NA
14	bA	1202	CLA	NC
14	bA	1202	CLA	ND
14	bA	1202	CLA	NA
14	HA	1211	CLA	NC
14	HA	1211	CLA	ND
14	HA	1211	CLA	NA
14	GA	1109	CLA	NC
14	GA	1109	CLA	ND
14	GA	1109	CLA	NA
14	GA	1126	CLA	NC
14	GA	1126	CLA	ND
14	GA	1126	CLA	NA
14	HA	1232	CLA	NC
14	HA	1232	CLA	ND
14	HA	1232	CLA	NA
14	HA	1238	CLA	NC
14	HA	1238	CLA	ND
14	HA	1238	CLA	NA
14	bA	1213	CLA	NC
14	bA	1213	CLA	ND
14	bA	1213	CLA	NA
14	BA	1215	CLA	NC
14	BA	1215	CLA	ND
14	BA	1215	CLA	NA
14	BA	1210	CLA	NC
14	BA	1210	CLA	ND
14	BA	1210	CLA	NA

Continued on next page...

PRELIMINARY VALIDATION REPORT

Continued from previous page...

Mol	Chain	Res	Type	Atom
14	BA	1239	CLA	NC
14	BA	1239	CLA	ND
14	BA	1239	CLA	NA
14	XA	1701	CLA	NC
14	XA	1701	CLA	ND
14	XA	1701	CLA	NA
14	bA	1224	CLA	ND
14	bA	1224	CLA	NA
14	HA	1221	CLA	NC
14	HA	1221	CLA	ND
14	HA	1221	CLA	NA
14	AA	1110	CLA	NC
14	AA	1110	CLA	ND
14	AA	1110	CLA	NA
14	GA	1123	CLA	NC
14	GA	1123	CLA	ND
14	GA	1123	CLA	NA
14	AA	1131	CLA	NC
14	AA	1131	CLA	ND
14	AA	1131	CLA	NA
14	AA	1126	CLA	NC
14	AA	1126	CLA	ND
14	AA	1126	CLA	NA
14	aA	1116	CLA	NC
14	aA	1116	CLA	ND
14	aA	1116	CLA	NA
14	bA	1235	CLA	NC
14	bA	1235	CLA	ND
14	bA	1235	CLA	NA
14	GA	1132	CLA	NC
14	GA	1132	CLA	ND
14	GA	1132	CLA	NA
14	AA	1102	CLA	NC
14	AA	1102	CLA	ND
14	AA	1102	CLA	NA
14	BA	1233	CLA	NC
14	BA	1233	CLA	ND
14	BA	1233	CLA	NA
14	AA	1113	CLA	NC
14	AA	1113	CLA	ND
14	AA	1113	CLA	NA
14	bA	1208	CLA	NC

Continued on next page...

PRELIMINARY VALIDATION REPORT

Continued from previous page...

Mol	Chain	Res	Type	Atom
14	bA	1208	CLA	ND
14	bA	1208	CLA	NA
14	bA	1207	CLA	NC
14	bA	1207	CLA	ND
14	bA	1207	CLA	NA
14	bA	1220	CLA	NC
14	bA	1220	CLA	ND
14	bA	1220	CLA	NA
14	LA	1503	CLA	NC
14	LA	1503	CLA	ND
14	LA	1503	CLA	NA
14	BA	1206	CLA	NC
14	BA	1206	CLA	ND
14	BA	1206	CLA	NA
14	HA	1023	CLA	NC
14	HA	1023	CLA	ND
14	HA	1023	CLA	NA
14	HA	1210	CLA	NC
14	HA	1210	CLA	ND
14	HA	1210	CLA	NA
14	aA	1137	CLA	NC
14	aA	1137	CLA	ND
14	aA	1137	CLA	NA
14	BA	1224	CLA	ND
14	BA	1224	CLA	NA
14	AA	1104	CLA	NC
14	AA	1104	CLA	ND
14	AA	1104	CLA	NA
14	bA	1203	CLA	NC
14	bA	1203	CLA	ND
14	bA	1203	CLA	NA
14	BA	1226	CLA	NC
14	BA	1226	CLA	ND
14	BA	1226	CLA	NA
14	GA	1136	CLA	NC
14	GA	1136	CLA	ND
14	GA	1136	CLA	NA
14	bA	1227	CLA	NC
14	bA	1227	CLA	ND
14	bA	1227	CLA	NA
14	HA	1213	CLA	NC
14	HA	1213	CLA	ND

Continued on next page...

PRELIMINARY VALIDATION REPORT

Continued from previous page...

Mol	Chain	Res	Type	Atom
14	HA	1213	CLA	NA
14	bA	1222	CLA	NC
14	bA	1222	CLA	ND
14	bA	1222	CLA	NA
14	GA	1013	CLA	NC
14	GA	1013	CLA	ND
14	GA	1013	CLA	NA
14	GA	1104	CLA	NC
14	GA	1104	CLA	ND
14	GA	1104	CLA	NA
14	AA	1109	CLA	NC
14	AA	1109	CLA	ND
14	AA	1109	CLA	NA
14	lA	1502	CLA	NC
14	lA	1502	CLA	ND
14	lA	1502	CLA	NA
14	HA	1217	CLA	NC
14	HA	1217	CLA	ND
14	HA	1217	CLA	NA
14	AA	1012	CLA	NC
14	AA	1012	CLA	NA
14	AA	1012	CLA	ND
14	BA	1229	CLA	NC
14	BA	1229	CLA	ND
14	BA	1229	CLA	NA
14	BA	1218	CLA	NC
14	BA	1218	CLA	ND
14	BA	1218	CLA	NA
14	HA	1220	CLA	NC
14	HA	1220	CLA	ND
14	HA	1220	CLA	NA
14	KA	1401	CLA	NC
14	KA	1401	CLA	ND
14	KA	1401	CLA	NA
14	bA	1201	CLA	NC
14	bA	1201	CLA	ND
14	bA	1201	CLA	NA
14	GA	1112	CLA	NC
14	GA	1112	CLA	ND
14	GA	1112	CLA	NA
14	GA	1118	CLA	NC
14	GA	1118	CLA	ND

Continued on next page...

PRELIMINARY VALIDATION REPORT

Continued from previous page...

Mol	Chain	Res	Type	Atom
14	GA	1118	CLA	NA
14	GA	1130	CLA	NC
14	GA	1130	CLA	ND
14	GA	1130	CLA	NA
14	aA	1129	CLA	NC
14	aA	1129	CLA	ND
14	aA	1129	CLA	NA
14	aA	1118	CLA	NC
14	aA	1118	CLA	ND
14	aA	1118	CLA	NA
14	BA	1227	CLA	NC
14	BA	1227	CLA	ND
14	BA	1227	CLA	NA
14	kA	1401	CLA	NC
14	kA	1401	CLA	ND
14	kA	1401	CLA	NA
14	BA	1201	CLA	NC
14	BA	1201	CLA	ND
14	BA	1201	CLA	NA
14	GA	1012	CLA	NC
14	GA	1012	CLA	NA
14	GA	1012	CLA	ND
14	aA	1131	CLA	NC
14	aA	1131	CLA	ND
14	aA	1131	CLA	NA
14	xA	1701	CLA	NC
14	xA	1701	CLA	ND
14	xA	1701	CLA	NA
14	bA	1209	CLA	NC
14	bA	1209	CLA	ND
14	bA	1209	CLA	NA
14	HA	1226	CLA	NC
14	HA	1226	CLA	ND
14	HA	1226	CLA	NA
14	AA	1105	CLA	NC
14	AA	1105	CLA	ND
14	AA	1105	CLA	NA
14	bA	1219	CLA	NC
14	bA	1219	CLA	ND
14	bA	1219	CLA	NA
14	aA	1105	CLA	NC
14	aA	1105	CLA	ND

Continued on next page...

PRELIMINARY VALIDATION REPORT

Continued from previous page...

Mol	Chain	Res	Type	Atom
14	aA	1105	CLA	NA
14	AA	1125	CLA	NC
14	AA	1125	CLA	ND
14	AA	1125	CLA	NA
14	GA	1124	CLA	NC
14	GA	1124	CLA	ND
14	GA	1124	CLA	NA
14	AA	1130	CLA	NC
14	AA	1130	CLA	ND
14	AA	1130	CLA	NA
14	HA	1218	CLA	NC
14	HA	1218	CLA	ND
14	HA	1218	CLA	NA
14	GA	1122	CLA	NC
14	GA	1122	CLA	ND
14	GA	1122	CLA	NA
14	GA	1119	CLA	NC
14	GA	1119	CLA	ND
14	GA	1119	CLA	NA
14	aA	1013	CLA	NC
14	aA	1013	CLA	ND
14	aA	1013	CLA	NA
14	HA	1212	CLA	NC
14	HA	1212	CLA	ND
14	HA	1212	CLA	NA
14	aA	1138	CLA	NC
14	aA	1138	CLA	ND
14	aA	1138	CLA	NA
14	UA	1503	CLA	NC
14	UA	1503	CLA	ND
14	UA	1503	CLA	NA
14	HA	1236	CLA	NC
14	HA	1236	CLA	ND
14	HA	1236	CLA	NA
14	bA	1228	CLA	NC
14	bA	1228	CLA	ND
14	bA	1228	CLA	NA
14	AA	1119	CLA	NC
14	AA	1119	CLA	ND
14	AA	1119	CLA	NA
14	AA	1139	CLA	NC
14	AA	1139	CLA	ND

Continued on next page...

PRELIMINARY VALIDATION REPORT

Continued from previous page...

Mol	Chain	Res	Type	Atom
14	AA	1139	CLA	NA
14	bA	1232	CLA	NC
14	bA	1232	CLA	ND
14	bA	1232	CLA	NA
14	BA	1211	CLA	NC
14	BA	1211	CLA	ND
14	BA	1211	CLA	NA
14	HA	1235	CLA	NC
14	HA	1235	CLA	ND
14	HA	1235	CLA	NA
14	HA	1223	CLA	NC
14	HA	1223	CLA	ND
14	HA	1223	CLA	NA
14	aA	1111	CLA	NC
14	aA	1111	CLA	ND
14	aA	1111	CLA	NA
14	HA	1204	CLA	NC
14	HA	1204	CLA	ND
14	HA	1204	CLA	NA
14	BA	1214	CLA	NC
14	BA	1214	CLA	ND
14	BA	1214	CLA	NA
14	TA	1401	CLA	NC
14	TA	1401	CLA	ND
14	TA	1401	CLA	NA
14	BA	1222	CLA	NC
14	BA	1222	CLA	ND
14	BA	1222	CLA	NA
14	aA	1127	CLA	NC
14	aA	1127	CLA	ND
14	aA	1127	CLA	NA
14	bA	1212	CLA	NC
14	bA	1212	CLA	ND
14	bA	1212	CLA	NA
14	BA	1023	CLA	NC
14	BA	1023	CLA	ND
14	BA	1023	CLA	NA
14	HA	1227	CLA	NC
14	HA	1227	CLA	ND
14	HA	1227	CLA	NA
14	AA	1117	CLA	NC
14	AA	1117	CLA	ND

Continued on next page...

PRELIMINARY VALIDATION REPORT

Continued from previous page...

Mol	Chain	Res	Type	Atom
14	AA	1117	CLA	NA
14	bA	1021	CLA	NC
14	bA	1021	CLA	ND
14	bA	1021	CLA	NA
14	BA	1234	CLA	NC
14	BA	1234	CLA	ND
14	BA	1234	CLA	NA
14	bA	1215	CLA	NC
14	bA	1215	CLA	ND
14	bA	1215	CLA	NA
14	aA	1110	CLA	NC
14	aA	1110	CLA	ND
14	aA	1110	CLA	NA
14	AA	1013	CLA	NC
14	AA	1013	CLA	ND
14	AA	1013	CLA	NA
14	BA	1202	CLA	NC
14	BA	1202	CLA	ND
14	BA	1202	CLA	NA
14	BA	1203	CLA	NC
14	BA	1203	CLA	ND
14	BA	1203	CLA	NA
13	aA	1011	CL0	NC
13	aA	1011	CL0	ND
13	aA	1011	CL0	NA
14	bA	1239	CLA	NC
14	bA	1239	CLA	ND
14	bA	1239	CLA	NA
14	bA	1206	CLA	NC
14	bA	1206	CLA	ND
14	bA	1206	CLA	NA
14	HA	1206	CLA	NC
14	HA	1206	CLA	ND
14	HA	1206	CLA	NA
14	GA	1135	CLA	NC
14	GA	1135	CLA	ND
14	GA	1135	CLA	NA
14	LA	1502	CLA	NC
14	LA	1502	CLA	ND
14	LA	1502	CLA	NA
14	aA	1113	CLA	NC
14	aA	1113	CLA	ND

Continued on next page...

PRELIMINARY VALIDATION REPORT

Continued from previous page...

Mol	Chain	Res	Type	Atom
14	aA	1113	CLA	NA
14	BA	1207	CLA	NC
14	BA	1207	CLA	ND
14	BA	1207	CLA	NA

All (3872) torsion outliers are listed below:

Mol	Chain	Res	Type	Atoms
14	LA	1501	CLA	C1A-C2A-CAA-CBA
14	AA	1115	CLA	C1A-C2A-CAA-CBA
14	AA	1115	CLA	C3A-C2A-CAA-CBA
14	AA	1115	CLA	CBD-CGD-O2D-CED
14	bA	1234	CLA	C2A-CAA-CBA-CGA
14	bA	1234	CLA	CHA-CBD-CGD-O2D
14	bA	1022	CLA	CHA-CBD-CGD-O1D
14	bA	1022	CLA	CHA-CBD-CGD-O2D
14	BA	1225	CLA	C1A-C2A-CAA-CBA
14	BA	1225	CLA	C3A-C2A-CAA-CBA
14	BA	1225	CLA	CHA-CBD-CGD-O1D
14	BA	1225	CLA	CHA-CBD-CGD-O2D
18	aA	4003	BCR	C7-C8-C9-C10
18	aA	4003	BCR	C7-C8-C9-C34
18	aA	4003	BCR	C11-C10-C9-C8
18	aA	4003	BCR	C11-C10-C9-C34
18	aA	4003	BCR	C9-C10-C11-C12
18	aA	4003	BCR	C10-C11-C12-C13
18	aA	4003	BCR	C21-C22-C23-C24
18	aA	4003	BCR	C37-C22-C23-C24
18	aA	4003	BCR	C23-C24-C25-C30
14	GA	1116	CLA	C1A-C2A-CAA-CBA
14	GA	1116	CLA	C3A-C2A-CAA-CBA
14	GA	1116	CLA	C2-C3-C5-C6
14	GA	1116	CLA	C4-C3-C5-C6
14	bA	1211	CLA	CBD-CGD-O2D-CED
14	BA	1228	CLA	CBD-CGD-O2D-CED
13	GA	1011	CL0	C2-C1-O2A-CGA
14	GA	1103	CLA	C2-C3-C5-C6
14	GA	1103	CLA	C4-C3-C5-C6
14	GA	1111	CLA	C2-C3-C5-C6
14	GA	1111	CLA	C4-C3-C5-C6
14	aA	1119	CLA	CHA-CBD-CGD-O1D
14	aA	1119	CLA	CHA-CBD-CGD-O2D

Continued on next page...

PRELIMINARY VALIDATION REPORT

Continued from previous page...

Mol	Chain	Res	Type	Atoms
14	AA	1118	CLA	C2-C1-O2A-CGA
14	AA	1118	CLA	C2-C3-C5-C6
14	AA	1118	CLA	C4-C3-C5-C6
19	AA	5001	LHG	O1-C1-C2-C3
19	AA	5001	LHG	C4-O6-P-O3
19	AA	5001	LHG	C4-O6-P-O4
19	AA	5001	LHG	C4-O6-P-O5
14	HA	1225	CLA	C1A-C2A-CAA-CBA
14	HA	1225	CLA	C3A-C2A-CAA-CBA
14	HA	1225	CLA	CHA-CBD-CGD-O1D
14	HA	1225	CLA	CHA-CBD-CGD-O2D
18	HA	4016	BCR	C11-C10-C9-C34
18	HA	4016	BCR	C17-C18-C19-C20
18	HA	4016	BCR	C36-C18-C19-C20
18	aA	4011	BCR	C7-C8-C9-C10
18	aA	4011	BCR	C7-C8-C9-C34
18	aA	4011	BCR	C10-C11-C12-C13
18	aA	4011	BCR	C11-C12-C13-C14
18	aA	4011	BCR	C11-C12-C13-C35
18	aA	4011	BCR	C23-C24-C25-C26
18	aA	4011	BCR	C23-C24-C25-C30
14	aA	1102	CLA	C1A-C2A-CAA-CBA
14	aA	1102	CLA	C2A-CAA-CBA-CGA
18	BA	4016	BCR	C11-C10-C9-C34
18	BA	4016	BCR	C17-C18-C19-C20
18	BA	4016	BCR	C36-C18-C19-C20
18	IA	4020	BCR	C11-C10-C9-C34
18	IA	4020	BCR	C23-C24-C25-C30
19	bA	5004	LHG	O1-C1-C2-C3
19	bA	5004	LHG	C4-O6-P-O4
14	HA	1215	CLA	C3A-C2A-CAA-CBA
14	HA	1215	CLA	CBD-CGD-O2D-CED
18	BA	4012	BCR	C7-C8-C9-C34
18	BA	4012	BCR	C11-C10-C9-C8
18	BA	4012	BCR	C11-C10-C9-C34
18	BA	4012	BCR	C10-C11-C12-C13
18	BA	4012	BCR	C11-C12-C13-C14
18	BA	4012	BCR	C11-C12-C13-C35
18	BA	4012	BCR	C17-C18-C19-C20
18	BA	4012	BCR	C36-C18-C19-C20
18	BA	4012	BCR	C19-C20-C21-C22
18	BA	4012	BCR	C21-C22-C23-C24

Continued on next page...

PRELIMINARY VALIDATION REPORT

Continued from previous page...

Mol	Chain	Res	Type	Atoms
18	BA	4012	BCR	C37-C22-C23-C24
18	BA	4012	BCR	C23-C24-C25-C26
18	BA	4012	BCR	C23-C24-C25-C30
18	HA	4005	BCR	C11-C10-C9-C8
18	HA	4005	BCR	C11-C10-C9-C34
14	HA	1216	CLA	CHA-CBD-CGD-O1D
14	HA	1216	CLA	CHA-CBD-CGD-O2D
20	bA	5002	LMG	O6-C1-O1-C7
18	MA	4021	BCR	C7-C8-C9-C10
18	MA	4021	BCR	C7-C8-C9-C34
18	MA	4021	BCR	C11-C10-C9-C8
18	MA	4021	BCR	C11-C10-C9-C34
18	MA	4021	BCR	C17-C18-C19-C20
18	MA	4021	BCR	C36-C18-C19-C20
18	MA	4021	BCR	C23-C24-C25-C30
18	bA	4014	BCR	C11-C10-C9-C8
18	bA	4014	BCR	C11-C10-C9-C34
18	bA	4014	BCR	C10-C11-C12-C13
18	bA	4014	BCR	C11-C12-C13-C14
18	bA	4014	BCR	C11-C12-C13-C35
18	bA	4014	BCR	C17-C18-C19-C20
18	bA	4014	BCR	C36-C18-C19-C20
14	bA	1214	CLA	CBA-CGA-O2A-C1
14	bA	1214	CLA	O1A-CGA-O2A-C1
14	bA	1214	CLA	C2-C3-C5-C6
14	bA	1214	CLA	C4-C3-C5-C6
14	GA	1101	CLA	CHA-CBD-CGD-O1D
14	GA	1101	CLA	CHA-CBD-CGD-O2D
18	AA	4003	BCR	C7-C8-C9-C10
18	AA	4003	BCR	C7-C8-C9-C34
18	AA	4003	BCR	C11-C10-C9-C8
18	AA	4003	BCR	C11-C10-C9-C34
18	AA	4003	BCR	C9-C10-C11-C12
18	AA	4003	BCR	C10-C11-C12-C13
18	AA	4003	BCR	C21-C22-C23-C24
18	AA	4003	BCR	C37-C22-C23-C24
18	AA	4003	BCR	C23-C24-C25-C30
14	BA	1212	CLA	C2A-CAA-CBA-CGA
14	BA	1212	CLA	CHA-CBD-CGD-O1D
14	BA	1212	CLA	CHA-CBD-CGD-O2D
14	BA	1217	CLA	C3A-C2A-CAA-CBA
14	BA	1217	CLA	CBD-CGD-O2D-CED

Continued on next page...

PRELIMINARY VALIDATION REPORT

Continued from previous page...

Mol	Chain	Res	Type	Atoms
14	AA	1140	CLA	C2-C3-C5-C6
14	AA	1140	CLA	C4-C3-C5-C6
18	RA	4020	BCR	C11-C10-C9-C34
18	RA	4020	BCR	C23-C24-C25-C30
18	bA	4005	BCR	C11-C10-C9-C8
18	bA	4005	BCR	C11-C10-C9-C34
18	LA	4019	BCR	C21-C22-C23-C24
18	LA	4019	BCR	C37-C22-C23-C24
18	LA	4019	BCR	C23-C24-C25-C26
18	LA	4019	BCR	C23-C24-C25-C30
14	aA	1012	CLA	CHA-CBD-CGD-O1D
14	aA	1012	CLA	CHA-CBD-CGD-O2D
18	iA	4020	BCR	C11-C10-C9-C34
18	iA	4020	BCR	C23-C24-C25-C30
14	AA	1122	CLA	CBA-CGA-O2A-C1
14	AA	1122	CLA	CHA-CBD-CGD-O1D
14	AA	1122	CLA	CHA-CBD-CGD-O2D
14	BA	1204	CLA	CHA-CBD-CGD-O1D
14	BA	1204	CLA	CHA-CBD-CGD-O2D
14	aA	1133	CLA	C1A-C2A-CAA-CBA
14	aA	1133	CLA	C3A-C2A-CAA-CBA
14	bA	1225	CLA	C1A-C2A-CAA-CBA
14	bA	1225	CLA	C3A-C2A-CAA-CBA
14	bA	1225	CLA	CHA-CBD-CGD-O1D
14	bA	1225	CLA	CHA-CBD-CGD-O2D
14	GA	1108	CLA	CHA-CBD-CGD-O1D
14	GA	1108	CLA	CHA-CBD-CGD-O2D
14	bA	1226	CLA	C2-C1-O2A-CGA
20	BA	5002	LMG	O6-C1-O1-C7
14	AA	1106	CLA	C3A-C2A-CAA-CBA
14	AA	1106	CLA	C2A-CAA-CBA-CGA
14	AA	1106	CLA	CHA-CBD-CGD-O1D
14	AA	1106	CLA	CHA-CBD-CGD-O2D
14	AA	1138	CLA	C1A-C2A-CAA-CBA
14	AA	1138	CLA	CBD-CGD-O2D-CED
18	HA	4014	BCR	C11-C10-C9-C8
18	HA	4014	BCR	C11-C10-C9-C34
18	HA	4014	BCR	C10-C11-C12-C13
18	HA	4014	BCR	C11-C12-C13-C14
18	HA	4014	BCR	C11-C12-C13-C35
18	HA	4014	BCR	C17-C18-C19-C20
18	HA	4014	BCR	C36-C18-C19-C20

Continued on next page...

PRELIMINARY VALIDATION REPORT

Continued from previous page...

Mol	Chain	Res	Type	Atoms
14	AA	1133	CLA	C1A-C2A-CAA-CBA
14	AA	1133	CLA	C3A-C2A-CAA-CBA
19	BA	5004	LHG	O1-C1-C2-C3
19	BA	5004	LHG	C4-O6-P-O4
18	BA	4009	BCR	C11-C10-C9-C8
18	BA	4009	BCR	C11-C10-C9-C34
18	BA	4009	BCR	C10-C11-C12-C13
18	bA	4012	BCR	C7-C8-C9-C34
18	bA	4012	BCR	C11-C10-C9-C8
18	bA	4012	BCR	C11-C10-C9-C34
18	bA	4012	BCR	C10-C11-C12-C13
18	bA	4012	BCR	C11-C12-C13-C14
18	bA	4012	BCR	C11-C12-C13-C35
18	bA	4012	BCR	C17-C18-C19-C20
18	bA	4012	BCR	C36-C18-C19-C20
18	bA	4012	BCR	C19-C20-C21-C22
18	bA	4012	BCR	C21-C22-C23-C24
18	bA	4012	BCR	C37-C22-C23-C24
18	bA	4012	BCR	C23-C24-C25-C26
18	bA	4012	BCR	C23-C24-C25-C30
14	bA	1210	CLA	C1A-C2A-CAA-CBA
14	bA	1210	CLA	CHA-CBD-CGD-O1D
14	bA	1210	CLA	CHA-CBD-CGD-O2D
14	bA	1204	CLA	CHA-CBD-CGD-O1D
14	bA	1204	CLA	CHA-CBD-CGD-O2D
14	HA	1229	CLA	C3A-C2A-CAA-CBA
14	HA	1229	CLA	C2-C1-O2A-CGA
14	HA	1229	CLA	CBD-CGD-O2D-CED
14	HA	1229	CLA	C2-C3-C5-C6
14	HA	1229	CLA	C4-C3-C5-C6
14	HA	1222	CLA	C6-C7-C8-C9
18	lA	4019	BCR	C21-C22-C23-C24
18	lA	4019	BCR	C37-C22-C23-C24
18	lA	4019	BCR	C23-C24-C25-C26
18	lA	4019	BCR	C23-C24-C25-C30
14	aA	1101	CLA	CHA-CBD-CGD-O1D
14	aA	1101	CLA	CHA-CBD-CGD-O2D
19	HA	5004	LHG	O1-C1-C2-C3
19	HA	5004	LHG	C4-O6-P-O4
14	HA	1233	CLA	CBD-CGD-O2D-CED
14	aA	1108	CLA	CHA-CBD-CGD-O1D
14	aA	1108	CLA	CHA-CBD-CGD-O2D

Continued on next page...

PRELIMINARY VALIDATION REPORT

Continued from previous page...

Mol	Chain	Res	Type	Atoms
14	GA	1140	CLA	C2-C3-C5-C6
14	GA	1140	CLA	C4-C3-C5-C6
14	bA	1217	CLA	C3A-C2A-CAA-CBA
14	bA	1217	CLA	CBD-CGD-O2D-CED
14	AA	1127	CLA	CHA-CBD-CGD-O1D
14	AA	1127	CLA	CHA-CBD-CGD-O2D
14	GA	1102	CLA	C1A-C2A-CAA-CBA
14	GA	1102	CLA	C2A-CAA-CBA-CGA
13	AA	1011	CL0	C2-C1-O2A-CGA
14	aA	1122	CLA	CBA-CGA-O2A-C1
14	aA	1122	CLA	CHA-CBD-CGD-O1D
14	aA	1122	CLA	CHA-CBD-CGD-O2D
18	bA	4009	BCR	C11-C10-C9-C8
18	bA	4009	BCR	C11-C10-C9-C34
18	bA	4009	BCR	C10-C11-C12-C13
14	AA	1101	CLA	CHA-CBD-CGD-O1D
14	AA	1101	CLA	CHA-CBD-CGD-O2D
14	HA	1224	CLA	CHA-CBD-CGD-O1D
14	HA	1224	CLA	CHA-CBD-CGD-O2D
14	HA	1224	CLA	CBD-CGD-O2D-CED
14	aA	1117	CLA	C1A-C2A-CAA-CBA
14	aA	1117	CLA	C3A-C2A-CAA-CBA
14	aA	1117	CLA	CHA-CBD-CGD-O1D
14	aA	1117	CLA	CHA-CBD-CGD-O2D
14	HA	1205	CLA	CHA-CBD-CGD-O2D
14	aA	1128	CLA	CHA-CBD-CGD-O1D
14	aA	1128	CLA	CHA-CBD-CGD-O2D
18	bA	4010	BCR	C11-C10-C9-C8
18	bA	4010	BCR	C11-C10-C9-C34
18	bA	4010	BCR	C17-C18-C19-C20
18	bA	4010	BCR	C36-C18-C19-C20
14	aA	1115	CLA	C1A-C2A-CAA-CBA
14	aA	1115	CLA	C3A-C2A-CAA-CBA
14	aA	1115	CLA	CBD-CGD-O2D-CED
18	AA	4001	BCR	C11-C10-C9-C8
18	AA	4001	BCR	C11-C10-C9-C34
18	AA	4001	BCR	C17-C18-C19-C20
18	AA	4001	BCR	C36-C18-C19-C20
14	bA	1023	CLA	CHA-CBD-CGD-O1D
14	bA	1023	CLA	CHA-CBD-CGD-O2D
18	aA	4007	BCR	C11-C10-C9-C8
18	aA	4007	BCR	C11-C10-C9-C34

Continued on next page...

PRELIMINARY VALIDATION REPORT

Continued from previous page...

Mol	Chain	Res	Type	Atoms
18	aA	4007	BCR	C10-C11-C12-C13
18	aA	4007	BCR	C21-C22-C23-C24
18	aA	4007	BCR	C37-C22-C23-C24
18	aA	4007	BCR	C23-C24-C25-C30
14	bA	1205	CLA	CHA-CBD-CGD-O2D
14	GA	1113	CLA	CHA-CBD-CGD-O1D
14	GA	1113	CLA	CHA-CBD-CGD-O2D
14	GA	1113	CLA	CAD-CBD-CGD-O1D
14	HA	1021	CLA	C2-C1-O2A-CGA
14	HA	1021	CLA	CHA-CBD-CGD-O1D
14	HA	1021	CLA	CHA-CBD-CGD-O2D
14	HA	1021	CLA	CBD-CGD-O2D-CED
14	HA	1228	CLA	CBD-CGD-O2D-CED
14	HA	1207	CLA	C1A-C2A-CAA-CBA
14	HA	1207	CLA	C3A-C2A-CAA-CBA
14	HA	1207	CLA	CBD-CGD-O2D-CED
14	BA	1220	CLA	CHA-CBD-CGD-O1D
14	BA	1220	CLA	CHA-CBD-CGD-O2D
18	bA	4016	BCR	C11-C10-C9-C34
18	bA	4016	BCR	C17-C18-C19-C20
18	bA	4016	BCR	C36-C18-C19-C20
14	GA	1127	CLA	CHA-CBD-CGD-O1D
14	GA	1127	CLA	CHA-CBD-CGD-O2D
14	bA	1218	CLA	CHA-CBD-CGD-O1D
14	bA	1218	CLA	CHA-CBD-CGD-O2D
21	AA	6001	LMT	C2'-C1'-O1'-C1
21	AA	6001	LMT	O5'-C1'-O1'-C1
14	GA	1128	CLA	CHA-CBD-CGD-O1D
14	GA	1128	CLA	CHA-CBD-CGD-O2D
14	HA	1239	CLA	C2-C1-O2A-CGA
14	HA	1214	CLA	CBA-CGA-O2A-C1
14	HA	1214	CLA	O1A-CGA-O2A-C1
14	HA	1214	CLA	C2-C3-C5-C6
14	HA	1214	CLA	C4-C3-C5-C6
14	AA	1128	CLA	CHA-CBD-CGD-O1D
14	AA	1128	CLA	CHA-CBD-CGD-O2D
14	0	1012	CLA	CHA-CBD-CGD-O1D
14	0	1012	CLA	CHA-CBD-CGD-O2D
20	HA	5002	LMG	O6-C1-O1-C7
14	bA	1216	CLA	CHA-CBD-CGD-O1D
14	bA	1216	CLA	CHA-CBD-CGD-O2D
18	aA	4001	BCR	C11-C10-C9-C8

Continued on next page...

PRELIMINARY VALIDATION REPORT

Continued from previous page...

Mol	Chain	Res	Type	Atoms
18	aA	4001	BCR	C11-C10-C9-C34
18	aA	4001	BCR	C17-C18-C19-C20
18	aA	4001	BCR	C36-C18-C19-C20
14	BA	1213	CLA	C1A-C2A-CAA-CBA
14	BA	1213	CLA	CBA-CGA-O2A-C1
14	BA	1213	CLA	O1A-CGA-O2A-C1
14	BA	1213	CLA	CHA-CBD-CGD-O1D
14	BA	1213	CLA	CHA-CBD-CGD-O2D
14	AA	1108	CLA	CHA-CBD-CGD-O1D
14	AA	1108	CLA	CHA-CBD-CGD-O2D
14	HA	1201	CLA	C2-C3-C5-C6
14	HA	1201	CLA	C4-C3-C5-C6
14	HA	1202	CLA	CHA-CBD-CGD-O1D
14	HA	1202	CLA	CHA-CBD-CGD-O2D
14	aA	1103	CLA	C2-C3-C5-C6
14	aA	1103	CLA	C4-C3-C5-C6
14	lA	1501	CLA	C1A-C2A-CAA-CBA
14	aA	1140	CLA	C2-C3-C5-C6
14	aA	1140	CLA	C4-C3-C5-C6
14	GA	1110	CLA	CHA-CBD-CGD-O1D
14	GA	1110	CLA	CHA-CBD-CGD-O2D
14	GA	1138	CLA	C1A-C2A-CAA-CBA
14	GA	1138	CLA	CBD-CGD-O2D-CED
18	HA	4010	BCR	C11-C10-C9-C8
18	HA	4010	BCR	C11-C10-C9-C34
18	HA	4010	BCR	C17-C18-C19-C20
18	HA	4010	BCR	C36-C18-C19-C20
20	RA	5006	LMG	C2-C1-O1-C7
20	RA	5006	LMG	O6-C1-O1-C7
19	GA	5001	LHG	O1-C1-C2-C3
19	GA	5001	LHG	C4-O6-P-O3
19	GA	5001	LHG	C4-O6-P-O4
19	GA	5001	LHG	C4-O6-P-O5
14	GA	1139	CLA	CBD-CGD-O2D-CED
14	aA	1136	CLA	C2-C3-C5-C6
14	aA	1136	CLA	C4-C3-C5-C6
14	GA	1117	CLA	C1A-C2A-CAA-CBA
14	GA	1117	CLA	C3A-C2A-CAA-CBA
14	GA	1117	CLA	CHA-CBD-CGD-O1D
14	GA	1117	CLA	CHA-CBD-CGD-O2D
14	BA	1232	CLA	CBD-CGD-O2D-CED
14	AA	1103	CLA	C2-C3-C5-C6

Continued on next page...

PRELIMINARY VALIDATION REPORT

Continued from previous page...

Mol	Chain	Res	Type	Atoms
14	AA	1103	CLA	C4-C3-C5-C6
14	GA	1106	CLA	C3A-C2A-CAA-CBA
14	GA	1106	CLA	C2A-CAA-CBA-CGA
14	GA	1106	CLA	CHA-CBD-CGD-O1D
14	GA	1106	CLA	CHA-CBD-CGD-O2D
18	AA	4002	BCR	C5-C6-C7-C8
18	AA	4002	BCR	C37-C22-C23-C24
14	BA	1021	CLA	C2-C1-O2A-CGA
14	BA	1021	CLA	CHA-CBD-CGD-O1D
14	BA	1021	CLA	CHA-CBD-CGD-O2D
14	BA	1021	CLA	CBD-CGD-O2D-CED
14	GA	1131	CLA	C2A-CAA-CBA-CGA
18	GA	4008	BCR	C1-C6-C7-C8
18	GA	4008	BCR	C7-C8-C9-C10
18	GA	4008	BCR	C7-C8-C9-C34
18	GA	4008	BCR	C11-C10-C9-C8
18	GA	4008	BCR	C11-C10-C9-C34
18	GA	4008	BCR	C10-C11-C12-C13
21	GA	6001	LMT	C2'-C1'-O1'-C1
21	GA	6001	LMT	O5'-C1'-O1'-C1
14	bA	1229	CLA	C3A-C2A-CAA-CBA
14	bA	1229	CLA	C2-C1-O2A-CGA
14	bA	1229	CLA	CBD-CGD-O2D-CED
14	bA	1229	CLA	C2-C3-C5-C6
14	bA	1229	CLA	C4-C3-C5-C6
18	IA	4018	BCR	C7-C8-C9-C10
18	IA	4018	BCR	C7-C8-C9-C34
18	IA	4018	BCR	C11-C10-C9-C8
18	IA	4018	BCR	C11-C10-C9-C34
18	IA	4018	BCR	C36-C18-C19-C20
18	IA	4018	BCR	C19-C20-C21-C22
14	AA	1116	CLA	C1A-C2A-CAA-CBA
14	AA	1116	CLA	C3A-C2A-CAA-CBA
14	AA	1116	CLA	C2-C3-C5-C6
14	AA	1116	CLA	C4-C3-C5-C6
14	aA	1106	CLA	C3A-C2A-CAA-CBA
14	aA	1106	CLA	C2A-CAA-CBA-CGA
14	aA	1106	CLA	CHA-CBD-CGD-O1D
14	aA	1106	CLA	CHA-CBD-CGD-O2D
14	bA	1233	CLA	CBD-CGD-O2D-CED
18	BA	4005	BCR	C11-C10-C9-C8
18	BA	4005	BCR	C11-C10-C9-C34

Continued on next page...

PRELIMINARY VALIDATION REPORT

Continued from previous page...

Mol	Chain	Res	Type	Atoms
14	GA	1133	CLA	C1A-C2A-CAA-CBA
14	GA	1133	CLA	C3A-C2A-CAA-CBA
14	AA	1111	CLA	C2-C3-C5-C6
14	AA	1111	CLA	C4-C3-C5-C6
18	bA	4013	BCR	C11-C10-C9-C8
18	bA	4013	BCR	C11-C10-C9-C34
18	bA	4013	BCR	C10-C11-C12-C13
18	bA	4013	BCR	C11-C12-C13-C14
18	bA	4013	BCR	C11-C12-C13-C35
18	bA	4013	BCR	C17-C18-C19-C20
18	bA	4013	BCR	C36-C18-C19-C20
18	bA	4013	BCR	C21-C22-C23-C24
18	bA	4013	BCR	C37-C22-C23-C24
18	bA	4013	BCR	C23-C24-C25-C26
14	HA	1203	CLA	C1A-C2A-CAA-CBA
14	HA	1203	CLA	CBD-CGD-O2D-CED
14	HA	1234	CLA	C2A-CAA-CBA-CGA
14	HA	1234	CLA	CHA-CBD-CGD-O2D
14	aA	1139	CLA	CBD-CGD-O2D-CED
18	LA	4022	BCR	C11-C10-C9-C8
18	LA	4022	BCR	C11-C10-C9-C34
14	BA	1022	CLA	CHA-CBD-CGD-O1D
14	BA	1022	CLA	CHA-CBD-CGD-O2D
14	BA	1216	CLA	CHA-CBD-CGD-O1D
14	BA	1216	CLA	CHA-CBD-CGD-O2D
14	GA	1115	CLA	C1A-C2A-CAA-CBA
14	GA	1115	CLA	C3A-C2A-CAA-CBA
14	GA	1115	CLA	CBD-CGD-O2D-CED
14	AA	1136	CLA	C2-C3-C5-C6
14	AA	1136	CLA	C4-C3-C5-C6
18	BA	4014	BCR	C11-C10-C9-C8
18	BA	4014	BCR	C11-C10-C9-C34
18	BA	4014	BCR	C10-C11-C12-C13
18	BA	4014	BCR	C11-C12-C13-C14
18	BA	4014	BCR	C11-C12-C13-C35
18	BA	4014	BCR	C17-C18-C19-C20
18	BA	4014	BCR	C36-C18-C19-C20
14	aA	1126	CLA	CHA-CBD-CGD-O1D
14	aA	1126	CLA	CHA-CBD-CGD-O2D
14	aA	1126	CLA	CBD-CGD-O2D-CED
14	BA	1205	CLA	CHA-CBD-CGD-O2D
14	UA	1501	CLA	C1A-C2A-CAA-CBA

Continued on next page...

PRELIMINARY VALIDATION REPORT

Continued from previous page...

Mol	Chain	Res	Type	Atoms
14	bA	1202	CLA	CHA-CBD-CGD-O1D
14	bA	1202	CLA	CHA-CBD-CGD-O2D
14	HA	1211	CLA	CBD-CGD-O2D-CED
18	aA	4002	BCR	C5-C6-C7-C8
18	aA	4002	BCR	C37-C22-C23-C24
14	GA	1126	CLA	CHA-CBD-CGD-O1D
14	GA	1126	CLA	CHA-CBD-CGD-O2D
14	GA	1126	CLA	CBD-CGD-O2D-CED
14	HA	1232	CLA	CBD-CGD-O2D-CED
20	GA	5005	LMG	O10-C28-O8-C9
14	bA	1213	CLA	C1A-C2A-CAA-CBA
14	bA	1213	CLA	CBA-CGA-O2A-C1
14	bA	1213	CLA	O1A-CGA-O2A-C1
14	bA	1213	CLA	CHA-CBD-CGD-O1D
14	bA	1213	CLA	CHA-CBD-CGD-O2D
18	bA	4004	BCR	C11-C10-C9-C8
18	bA	4004	BCR	C11-C10-C9-C34
18	UA	4019	BCR	C21-C22-C23-C24
18	UA	4019	BCR	C37-C22-C23-C24
18	UA	4019	BCR	C23-C24-C25-C26
18	UA	4019	BCR	C23-C24-C25-C30
14	BA	1215	CLA	C3A-C2A-CAA-CBA
14	BA	1215	CLA	CBD-CGD-O2D-CED
14	BA	1210	CLA	C1A-C2A-CAA-CBA
14	BA	1210	CLA	CHA-CBD-CGD-O1D
14	BA	1210	CLA	CHA-CBD-CGD-O2D
18	AA	4008	BCR	C1-C6-C7-C8
18	AA	4008	BCR	C7-C8-C9-C10
18	AA	4008	BCR	C7-C8-C9-C34
18	AA	4008	BCR	C11-C10-C9-C8
18	AA	4008	BCR	C11-C10-C9-C34
18	AA	4008	BCR	C10-C11-C12-C13
14	BA	1239	CLA	C2-C1-O2A-CGA
18	HA	4017	BCR	C11-C10-C9-C8
18	HA	4017	BCR	C11-C10-C9-C34
18	HA	4017	BCR	C10-C11-C12-C13
18	HA	4017	BCR	C21-C22-C23-C24
18	HA	4017	BCR	C37-C22-C23-C24
14	bA	1224	CLA	CHA-CBD-CGD-O1D
14	bA	1224	CLA	CHA-CBD-CGD-O2D
14	bA	1224	CLA	CBD-CGD-O2D-CED
18	GA	4011	BCR	C7-C8-C9-C10

Continued on next page...

PRELIMINARY VALIDATION REPORT

Continued from previous page...

Mol	Chain	Res	Type	Atoms
18	GA	4011	BCR	C7-C8-C9-C34
18	GA	4011	BCR	C10-C11-C12-C13
18	GA	4011	BCR	C11-C12-C13-C14
18	GA	4011	BCR	C11-C12-C13-C35
18	GA	4011	BCR	C23-C24-C25-C26
18	GA	4011	BCR	C23-C24-C25-C30
14	AA	1110	CLA	CHA-CBD-CGD-O1D
14	AA	1110	CLA	CHA-CBD-CGD-O2D
14	AA	1131	CLA	C2A-CAA-CBA-CGA
14	AA	1126	CLA	CHA-CBD-CGD-O1D
14	AA	1126	CLA	CHA-CBD-CGD-O2D
14	AA	1126	CLA	CBD-CGD-O2D-CED
18	HA	4004	BCR	C11-C10-C9-C8
18	HA	4004	BCR	C11-C10-C9-C34
18	GA	4002	BCR	C5-C6-C7-C8
18	GA	4002	BCR	C37-C22-C23-C24
14	aA	1116	CLA	C1A-C2A-CAA-CBA
14	aA	1116	CLA	C3A-C2A-CAA-CBA
14	aA	1116	CLA	C2-C3-C5-C6
14	aA	1116	CLA	C4-C3-C5-C6
14	AA	1102	CLA	C1A-C2A-CAA-CBA
14	AA	1102	CLA	C2A-CAA-CBA-CGA
18	HA	4012	BCR	C7-C8-C9-C34
18	HA	4012	BCR	C11-C10-C9-C8
18	HA	4012	BCR	C11-C10-C9-C34
18	HA	4012	BCR	C10-C11-C12-C13
18	HA	4012	BCR	C11-C12-C13-C14
18	HA	4012	BCR	C11-C12-C13-C35
18	HA	4012	BCR	C17-C18-C19-C20
18	HA	4012	BCR	C36-C18-C19-C20
18	HA	4012	BCR	C19-C20-C21-C22
18	HA	4012	BCR	C21-C22-C23-C24
18	HA	4012	BCR	C37-C22-C23-C24
18	HA	4012	BCR	C23-C24-C25-C26
18	HA	4012	BCR	C23-C24-C25-C30
14	BA	1233	CLA	CBD-CGD-O2D-CED
14	AA	1113	CLA	CHA-CBD-CGD-O1D
14	AA	1113	CLA	CHA-CBD-CGD-O2D
14	AA	1113	CLA	CAD-CBD-CGD-O1D
14	bA	1207	CLA	C1A-C2A-CAA-CBA
14	bA	1207	CLA	C3A-C2A-CAA-CBA
14	bA	1207	CLA	CBD-CGD-O2D-CED

Continued on next page...

PRELIMINARY VALIDATION REPORT

Continued from previous page...

Mol	Chain	Res	Type	Atoms
18	GA	4001	BCR	C11-C10-C9-C8
18	GA	4001	BCR	C11-C10-C9-C34
18	GA	4001	BCR	C17-C18-C19-C20
18	GA	4001	BCR	C36-C18-C19-C20
14	bA	1220	CLA	CHA-CBD-CGD-O1D
14	bA	1220	CLA	CHA-CBD-CGD-O2D
20	iA	5006	LMG	C2-C1-O1-C7
20	iA	5006	LMG	O6-C1-O1-C7
14	BA	1206	CLA	C1A-C2A-CAA-CBA
14	BA	1206	CLA	C3A-C2A-CAA-CBA
14	BA	1206	CLA	C2A-CAA-CBA-CGA
14	HA	1023	CLA	CHA-CBD-CGD-O1D
14	HA	1023	CLA	CHA-CBD-CGD-O2D
14	HA	1210	CLA	C1A-C2A-CAA-CBA
14	HA	1210	CLA	CHA-CBD-CGD-O1D
14	HA	1210	CLA	CHA-CBD-CGD-O2D
14	BA	1224	CLA	CHA-CBD-CGD-O1D
14	BA	1224	CLA	CHA-CBD-CGD-O2D
14	BA	1224	CLA	CBD-CGD-O2D-CED
14	bA	1203	CLA	C1A-C2A-CAA-CBA
14	bA	1203	CLA	CBD-CGD-O2D-CED
14	BA	1226	CLA	C2-C1-O2A-CGA
14	GA	1136	CLA	C2-C3-C5-C6
14	GA	1136	CLA	C4-C3-C5-C6
20	AA	5005	LMG	O10-C28-O8-C9
18	AA	4007	BCR	C11-C10-C9-C8
18	AA	4007	BCR	C11-C10-C9-C34
18	AA	4007	BCR	C10-C11-C12-C13
18	AA	4007	BCR	C21-C22-C23-C24
18	AA	4007	BCR	C37-C22-C23-C24
18	AA	4007	BCR	C23-C24-C25-C30
18	RA	4018	BCR	C7-C8-C9-C10
18	RA	4018	BCR	C7-C8-C9-C34
18	RA	4018	BCR	C11-C10-C9-C8
18	RA	4018	BCR	C11-C10-C9-C34
18	RA	4018	BCR	C36-C18-C19-C20
18	lA	4022	BCR	C11-C10-C9-C8
18	lA	4022	BCR	C11-C10-C9-C34
14	HA	1213	CLA	C1A-C2A-CAA-CBA
14	HA	1213	CLA	CBA-CGA-O2A-C1
14	HA	1213	CLA	O1A-CGA-O2A-C1
14	HA	1213	CLA	CHA-CBD-CGD-O1D

Continued on next page...

PRELIMINARY VALIDATION REPORT

Continued from previous page...

Mol	Chain	Res	Type	Atoms
14	HA	1213	CLA	CHA-CBD-CGD-O2D
14	bA	1222	CLA	C6-C7-C8-C9
18	HA	4009	BCR	C11-C10-C9-C8
18	HA	4009	BCR	C11-C10-C9-C34
18	HA	4009	BCR	C10-C11-C12-C13
14	GA	1013	CLA	C2-C1-O2A-CGA
18	GA	4003	BCR	C7-C8-C9-C10
18	GA	4003	BCR	C7-C8-C9-C34
18	GA	4003	BCR	C11-C10-C9-C8
18	GA	4003	BCR	C11-C10-C9-C34
18	GA	4003	BCR	C9-C10-C11-C12
18	GA	4003	BCR	C10-C11-C12-C13
18	GA	4003	BCR	C21-C22-C23-C24
18	GA	4003	BCR	C37-C22-C23-C24
18	GA	4003	BCR	C23-C24-C25-C30
21	aA	6001	LMT	C2'-C1'-O1'-C1
21	aA	6001	LMT	O5'-C1'-O1'-C1
19	aA	5001	LHG	O1-C1-C2-C3
19	aA	5001	LHG	C4-O6-P-O3
19	aA	5001	LHG	C4-O6-P-O4
19	aA	5001	LHG	C4-O6-P-O5
14	HA	1217	CLA	C3A-C2A-CAA-CBA
14	HA	1217	CLA	CBD-CGD-O2D-CED
14	AA	1012	CLA	CHA-CBD-CGD-O1D
14	AA	1012	CLA	CHA-CBD-CGD-O2D
14	BA	1229	CLA	C3A-C2A-CAA-CBA
14	BA	1229	CLA	C2-C1-O2A-CGA
14	BA	1229	CLA	CBD-CGD-O2D-CED
14	BA	1229	CLA	C2-C3-C5-C6
14	BA	1229	CLA	C4-C3-C5-C6
14	BA	1218	CLA	CHA-CBD-CGD-O1D
14	BA	1218	CLA	CHA-CBD-CGD-O2D
14	HA	1220	CLA	CHA-CBD-CGD-O1D
14	HA	1220	CLA	CHA-CBD-CGD-O2D
14	KA	1401	CLA	C2A-CAA-CBA-CGA
14	KA	1401	CLA	CHA-CBD-CGD-O1D
14	KA	1401	CLA	CHA-CBD-CGD-O2D
14	KA	1401	CLA	CBD-CGD-O2D-CED
14	bA	1201	CLA	C2-C3-C5-C6
14	bA	1201	CLA	C4-C3-C5-C6
18	mA	4021	BCR	C7-C8-C9-C10
18	mA	4021	BCR	C7-C8-C9-C34

Continued on next page...

PRELIMINARY VALIDATION REPORT

Continued from previous page...

Mol	Chain	Res	Type	Atoms
18	mA	4021	BCR	C11-C10-C9-C8
18	mA	4021	BCR	C11-C10-C9-C34
18	mA	4021	BCR	C17-C18-C19-C20
18	mA	4021	BCR	C36-C18-C19-C20
18	mA	4021	BCR	C23-C24-C25-C30
14	GA	1118	CLA	C2-C1-O2A-CGA
14	GA	1118	CLA	C2-C3-C5-C6
14	GA	1118	CLA	C4-C3-C5-C6
14	aA	1118	CLA	C2-C1-O2A-CGA
14	aA	1118	CLA	C2-C3-C5-C6
14	aA	1118	CLA	C4-C3-C5-C6
14	kA	1401	CLA	C2A-CAA-CBA-CGA
14	kA	1401	CLA	CHA-CBD-CGD-O1D
14	kA	1401	CLA	CHA-CBD-CGD-O2D
14	kA	1401	CLA	CBD-CGD-O2D-CED
14	BA	1201	CLA	C2-C3-C5-C6
14	BA	1201	CLA	C4-C3-C5-C6
14	GA	1012	CLA	CHA-CBD-CGD-O1D
14	GA	1012	CLA	CHA-CBD-CGD-O2D
14	aA	1131	CLA	C2A-CAA-CBA-CGA
18	aA	4008	BCR	C1-C6-C7-C8
18	aA	4008	BCR	C7-C8-C9-C10
18	aA	4008	BCR	C7-C8-C9-C34
18	aA	4008	BCR	C11-C10-C9-C8
18	aA	4008	BCR	C11-C10-C9-C34
18	aA	4008	BCR	C10-C11-C12-C13
18	bA	4017	BCR	C11-C10-C9-C8
18	bA	4017	BCR	C11-C10-C9-C34
18	bA	4017	BCR	C10-C11-C12-C13
18	bA	4017	BCR	C21-C22-C23-C24
18	bA	4017	BCR	C37-C22-C23-C24
14	HA	1226	CLA	C2-C1-O2A-CGA
18	iA	4018	BCR	C7-C8-C9-C10
18	iA	4018	BCR	C7-C8-C9-C34
18	iA	4018	BCR	C11-C10-C9-C8
18	iA	4018	BCR	C11-C10-C9-C34
18	iA	4018	BCR	C36-C18-C19-C20
18	iA	4018	BCR	C19-C20-C21-C22
14	AA	1130	CLA	C4-C3-C5-C6
14	HA	1218	CLA	CHA-CBD-CGD-O1D
14	HA	1218	CLA	CHA-CBD-CGD-O2D
14	GA	1122	CLA	CBA-CGA-O2A-C1

Continued on next page...

PRELIMINARY VALIDATION REPORT

Continued from previous page...

Mol	Chain	Res	Type	Atoms
14	GA	1122	CLA	CHA-CBD-CGD-O1D
14	GA	1122	CLA	CHA-CBD-CGD-O2D
14	GA	1119	CLA	CHA-CBD-CGD-O1D
14	GA	1119	CLA	CHA-CBD-CGD-O2D
14	aA	1013	CLA	C2-C1-O2A-CGA
14	HA	1212	CLA	C2A-CAA-CBA-CGA
14	HA	1212	CLA	CHA-CBD-CGD-O1D
14	HA	1212	CLA	CHA-CBD-CGD-O2D
18	BA	4017	BCR	C11-C10-C9-C8
18	BA	4017	BCR	C11-C10-C9-C34
18	BA	4017	BCR	C10-C11-C12-C13
18	BA	4017	BCR	C21-C22-C23-C24
18	BA	4017	BCR	C37-C22-C23-C24
20	aA	5005	LMG	O10-C28-O8-C9
14	aA	1138	CLA	C1A-C2A-CAA-CBA
14	aA	1138	CLA	CBD-CGD-O2D-CED
18	BA	4010	BCR	C11-C10-C9-C8
18	BA	4010	BCR	C11-C10-C9-C34
18	BA	4010	BCR	C17-C18-C19-C20
18	BA	4010	BCR	C36-C18-C19-C20
14	bA	1228	CLA	CBD-CGD-O2D-CED
14	AA	1119	CLA	CHA-CBD-CGD-O1D
14	AA	1119	CLA	CHA-CBD-CGD-O2D
18	BA	4013	BCR	C11-C10-C9-C8
18	BA	4013	BCR	C11-C10-C9-C34
18	BA	4013	BCR	C10-C11-C12-C13
18	BA	4013	BCR	C11-C12-C13-C14
18	BA	4013	BCR	C11-C12-C13-C35
18	BA	4013	BCR	C17-C18-C19-C20
18	BA	4013	BCR	C36-C18-C19-C20
18	BA	4013	BCR	C21-C22-C23-C24
18	BA	4013	BCR	C37-C22-C23-C24
18	BA	4013	BCR	C23-C24-C25-C26
14	AA	1139	CLA	CBD-CGD-O2D-CED
18	AA	4011	BCR	C7-C8-C9-C10
18	AA	4011	BCR	C7-C8-C9-C34
18	AA	4011	BCR	C10-C11-C12-C13
18	AA	4011	BCR	C11-C12-C13-C14
18	AA	4011	BCR	C11-C12-C13-C35
18	AA	4011	BCR	C23-C24-C25-C26
18	AA	4011	BCR	C23-C24-C25-C30
14	bA	1232	CLA	CBD-CGD-O2D-CED

Continued on next page...

PRELIMINARY VALIDATION REPORT

Continued from previous page...

Mol	Chain	Res	Type	Atoms
14	BA	1211	CLA	CBD-CGD-O2D-CED
20	IA	5006	LMG	C2-C1-O1-C7
20	IA	5006	LMG	O6-C1-O1-C7
14	aA	1111	CLA	C2-C3-C5-C6
14	aA	1111	CLA	C4-C3-C5-C6
18	UA	4022	BCR	C11-C10-C9-C8
18	UA	4022	BCR	C11-C10-C9-C34
14	HA	1204	CLA	CHA-CBD-CGD-O1D
14	HA	1204	CLA	CHA-CBD-CGD-O2D
14	BA	1214	CLA	CBA-CGA-O2A-C1
14	BA	1214	CLA	O1A-CGA-O2A-C1
14	BA	1214	CLA	C2-C3-C5-C6
14	BA	1214	CLA	C4-C3-C5-C6
14	TA	1401	CLA	C2A-CAA-CBA-CGA
14	TA	1401	CLA	CHA-CBD-CGD-O1D
14	TA	1401	CLA	CHA-CBD-CGD-O2D
14	TA	1401	CLA	CBD-CGD-O2D-CED
14	BA	1222	CLA	C6-C7-C8-C9
14	aA	1127	CLA	CHA-CBD-CGD-O1D
14	aA	1127	CLA	CHA-CBD-CGD-O2D
14	bA	1212	CLA	C2A-CAA-CBA-CGA
14	bA	1212	CLA	CHA-CBD-CGD-O1D
14	bA	1212	CLA	CHA-CBD-CGD-O2D
14	BA	1023	CLA	CHA-CBD-CGD-O1D
14	BA	1023	CLA	CHA-CBD-CGD-O2D
14	AA	1117	CLA	C1A-C2A-CAA-CBA
14	AA	1117	CLA	C3A-C2A-CAA-CBA
14	AA	1117	CLA	CHA-CBD-CGD-O1D
14	AA	1117	CLA	CHA-CBD-CGD-O2D
18	BA	4004	BCR	C11-C10-C9-C8
18	BA	4004	BCR	C11-C10-C9-C34
14	bA	1021	CLA	C2-C1-O2A-CGA
14	bA	1021	CLA	CHA-CBD-CGD-O1D
14	bA	1021	CLA	CHA-CBD-CGD-O2D
14	bA	1021	CLA	CBD-CGD-O2D-CED
18	HA	4013	BCR	C11-C10-C9-C8
18	HA	4013	BCR	C11-C10-C9-C34
18	HA	4013	BCR	C10-C11-C12-C13
18	HA	4013	BCR	C11-C12-C13-C14
18	HA	4013	BCR	C11-C12-C13-C35
18	HA	4013	BCR	C17-C18-C19-C20
18	HA	4013	BCR	C36-C18-C19-C20

Continued on next page...

PRELIMINARY VALIDATION REPORT

Continued from previous page...

Mol	Chain	Res	Type	Atoms
18	HA	4013	BCR	C21-C22-C23-C24
18	HA	4013	BCR	C37-C22-C23-C24
18	HA	4013	BCR	C23-C24-C25-C26
14	BA	1234	CLA	C2A-CAA-CBA-CGA
14	BA	1234	CLA	CHA-CBD-CGD-O2D
14	bA	1215	CLA	C3A-C2A-CAA-CBA
14	bA	1215	CLA	CBD-CGD-O2D-CED
14	aA	1110	CLA	CHA-CBD-CGD-O1D
14	aA	1110	CLA	CHA-CBD-CGD-O2D
14	AA	1013	CLA	C2-C1-O2A-CGA
14	BA	1202	CLA	CHA-CBD-CGD-O1D
14	BA	1202	CLA	CHA-CBD-CGD-O2D
14	BA	1203	CLA	C1A-C2A-CAA-CBA
14	BA	1203	CLA	O1A-CGA-O2A-C1
14	BA	1203	CLA	CBD-CGD-O2D-CED
13	aA	1011	CL0	C2-C1-O2A-CGA
14	bA	1239	CLA	C2-C1-O2A-CGA
18	GA	4007	BCR	C11-C10-C9-C8
18	GA	4007	BCR	C11-C10-C9-C34
18	GA	4007	BCR	C10-C11-C12-C13
18	GA	4007	BCR	C21-C22-C23-C24
18	GA	4007	BCR	C37-C22-C23-C24
18	GA	4007	BCR	C23-C24-C25-C30
14	bA	1206	CLA	C1A-C2A-CAA-CBA
14	bA	1206	CLA	C3A-C2A-CAA-CBA
14	bA	1206	CLA	C2A-CAA-CBA-CGA
14	HA	1206	CLA	C1A-C2A-CAA-CBA
14	HA	1206	CLA	C3A-C2A-CAA-CBA
14	HA	1206	CLA	C2A-CAA-CBA-CGA
18	VA	4021	BCR	C7-C8-C9-C10
18	VA	4021	BCR	C7-C8-C9-C34
18	VA	4021	BCR	C11-C10-C9-C8
18	VA	4021	BCR	C11-C10-C9-C34
18	VA	4021	BCR	C17-C18-C19-C20
18	VA	4021	BCR	C36-C18-C19-C20
18	VA	4021	BCR	C23-C24-C25-C30
14	aA	1113	CLA	CHA-CBD-CGD-O1D
14	aA	1113	CLA	CHA-CBD-CGD-O2D
14	aA	1113	CLA	CAD-CBD-CGD-O1D
14	BA	1207	CLA	C1A-C2A-CAA-CBA
14	BA	1207	CLA	C3A-C2A-CAA-CBA
14	BA	1207	CLA	CBD-CGD-O2D-CED

Continued on next page...

PRELIMINARY VALIDATION REPORT

Continued from previous page...

Mol	Chain	Res	Type	Atoms
14	bA	1023	CLA	C2C-C3C-CAC-CBC
14	HA	1023	CLA	C2C-C3C-CAC-CBC
14	BA	1023	CLA	C2C-C3C-CAC-CBC
14	aA	1119	CLA	O1D-CGD-O2D-CED
14	HA	1216	CLA	O1D-CGD-O2D-CED
14	bA	1216	CLA	O1D-CGD-O2D-CED
14	BA	1216	CLA	O1D-CGD-O2D-CED
14	GA	1119	CLA	O1D-CGD-O2D-CED
14	AA	1119	CLA	O1D-CGD-O2D-CED
14	WA	1701	CLA	O1D-CGD-O2D-CED
14	BA	1217	CLA	O1D-CGD-O2D-CED
14	bA	1217	CLA	O1D-CGD-O2D-CED
14	HA	1021	CLA	O1D-CGD-O2D-CED
14	GA	1139	CLA	O1D-CGD-O2D-CED
14	BA	1021	CLA	O1D-CGD-O2D-CED
14	HA	1203	CLA	O1D-CGD-O2D-CED
14	aA	1139	CLA	O1D-CGD-O2D-CED
14	XA	1701	CLA	O1D-CGD-O2D-CED
14	bA	1203	CLA	O1D-CGD-O2D-CED
14	HA	1217	CLA	O1D-CGD-O2D-CED
14	KA	1401	CLA	O1D-CGD-O2D-CED
14	kA	1401	CLA	O1D-CGD-O2D-CED
14	xA	1701	CLA	O1D-CGD-O2D-CED
14	AA	1139	CLA	O1D-CGD-O2D-CED
14	TA	1401	CLA	O1D-CGD-O2D-CED
14	BA	1203	CLA	O1D-CGD-O2D-CED
14	LA	1501	CLA	CBD-CGD-O2D-CED
14	GA	1116	CLA	CBD-CGD-O2D-CED
14	GA	1111	CLA	CBD-CGD-O2D-CED
14	aA	1119	CLA	CBD-CGD-O2D-CED
14	WA	1701	CLA	CBD-CGD-O2D-CED
14	AA	1118	CLA	CBD-CGD-O2D-CED
14	aA	1124	CLA	CBD-CGD-O2D-CED
14	aA	1102	CLA	CBD-CGD-O2D-CED
14	HA	1216	CLA	CBD-CGD-O2D-CED
14	GA	1137	CLA	CBD-CGD-O2D-CED
14	aA	1107	CLA	CBD-CGD-O2D-CED
14	AA	1124	CLA	CBD-CGD-O2D-CED
14	AA	1132	CLA	CBD-CGD-O2D-CED
14	GA	1108	CLA	CBD-CGD-O2D-CED
14	aA	1114	CLA	CBD-CGD-O2D-CED
14	aA	1108	CLA	CBD-CGD-O2D-CED

Continued on next page...

PRELIMINARY VALIDATION REPORT

Continued from previous page...

Mol	Chain	Res	Type	Atoms
14	GA	1102	CLA	CBD-CGD-O2D-CED
14	GA	1114	CLA	CBD-CGD-O2D-CED
14	GA	1107	CLA	CBD-CGD-O2D-CED
14	AA	1114	CLA	CBD-CGD-O2D-CED
14	aA	1132	CLA	CBD-CGD-O2D-CED
14	HA	1239	CLA	CBD-CGD-O2D-CED
14	bA	1216	CLA	CBD-CGD-O2D-CED
14	AA	1107	CLA	CBD-CGD-O2D-CED
14	BA	1213	CLA	CBD-CGD-O2D-CED
14	AA	1108	CLA	CBD-CGD-O2D-CED
14	aA	1109	CLA	CBD-CGD-O2D-CED
14	lA	1501	CLA	CBD-CGD-O2D-CED
14	GA	1131	CLA	CBD-CGD-O2D-CED
14	AA	1116	CLA	CBD-CGD-O2D-CED
14	AA	1137	CLA	CBD-CGD-O2D-CED
14	AA	1111	CLA	CBD-CGD-O2D-CED
14	BA	1216	CLA	CBD-CGD-O2D-CED
14	UA	1501	CLA	CBD-CGD-O2D-CED
14	GA	1109	CLA	CBD-CGD-O2D-CED
14	bA	1213	CLA	CBD-CGD-O2D-CED
14	BA	1239	CLA	CBD-CGD-O2D-CED
14	XA	1701	CLA	CBD-CGD-O2D-CED
14	AA	1131	CLA	CBD-CGD-O2D-CED
14	aA	1116	CLA	CBD-CGD-O2D-CED
14	GA	1132	CLA	CBD-CGD-O2D-CED
14	AA	1102	CLA	CBD-CGD-O2D-CED
14	BA	1206	CLA	CBD-CGD-O2D-CED
14	aA	1137	CLA	CBD-CGD-O2D-CED
14	bA	1227	CLA	CBD-CGD-O2D-CED
14	HA	1213	CLA	CBD-CGD-O2D-CED
14	AA	1109	CLA	CBD-CGD-O2D-CED
14	GA	1118	CLA	CBD-CGD-O2D-CED
14	aA	1118	CLA	CBD-CGD-O2D-CED
14	BA	1227	CLA	CBD-CGD-O2D-CED
14	aA	1131	CLA	CBD-CGD-O2D-CED
14	xA	1701	CLA	CBD-CGD-O2D-CED
14	GA	1124	CLA	CBD-CGD-O2D-CED
14	GA	1119	CLA	CBD-CGD-O2D-CED
14	AA	1119	CLA	CBD-CGD-O2D-CED
14	aA	1111	CLA	CBD-CGD-O2D-CED
14	HA	1227	CLA	CBD-CGD-O2D-CED
14	bA	1239	CLA	CBD-CGD-O2D-CED

Continued on next page...

PRELIMINARY VALIDATION REPORT

Continued from previous page...

Mol	Chain	Res	Type	Atoms
14	bA	1206	CLA	CBD-CGD-O2D-CED
14	HA	1206	CLA	CBD-CGD-O2D-CED
14	AA	1118	CLA	O1A-CGA-O2A-C1
14	aA	1102	CLA	O1A-CGA-O2A-C1
14	AA	1122	CLA	O1A-CGA-O2A-C1
14	aA	1133	CLA	O1A-CGA-O2A-C1
14	AA	1138	CLA	O1A-CGA-O2A-C1
14	AA	1133	CLA	O1A-CGA-O2A-C1
14	HA	1222	CLA	O1A-CGA-O2A-C1
14	AA	1127	CLA	O1A-CGA-O2A-C1
14	GA	1102	CLA	O1A-CGA-O2A-C1
14	aA	1122	CLA	O1A-CGA-O2A-C1
14	GA	1127	CLA	O1A-CGA-O2A-C1
14	GA	1138	CLA	O1A-CGA-O2A-C1
14	GA	1133	CLA	O1A-CGA-O2A-C1
14	HA	1203	CLA	O1A-CGA-O2A-C1
14	AA	1102	CLA	O1A-CGA-O2A-C1
14	bA	1203	CLA	O1A-CGA-O2A-C1
14	bA	1222	CLA	O1A-CGA-O2A-C1
14	GA	1118	CLA	O1A-CGA-O2A-C1
14	aA	1118	CLA	O1A-CGA-O2A-C1
14	GA	1122	CLA	O1A-CGA-O2A-C1
14	aA	1138	CLA	O1A-CGA-O2A-C1
14	BA	1222	CLA	O1A-CGA-O2A-C1
14	aA	1127	CLA	O1A-CGA-O2A-C1
14	bA	1023	CLA	C4C-C3C-CAC-CBC
14	HA	1023	CLA	C4C-C3C-CAC-CBC
14	BA	1023	CLA	C4C-C3C-CAC-CBC
14	GA	1108	CLA	O1D-CGD-O2D-CED
14	AA	1138	CLA	O1D-CGD-O2D-CED
14	aA	1108	CLA	O1D-CGD-O2D-CED
14	AA	1108	CLA	O1D-CGD-O2D-CED
14	GA	1138	CLA	O1D-CGD-O2D-CED
14	aA	1138	CLA	O1D-CGD-O2D-CED
14	bA	1021	CLA	O1D-CGD-O2D-CED
21	GA	6002	LMT	O5B-C1B-O1B-C4’
21	aA	6002	LMT	O5B-C1B-O1B-C4’
21	AA	6002	LMT	O5B-C1B-O1B-C4’
14	bA	1211	CLA	O1D-CGD-O2D-CED
14	BA	1228	CLA	O1D-CGD-O2D-CED
14	HA	1215	CLA	O1D-CGD-O2D-CED
14	HA	1233	CLA	O1D-CGD-O2D-CED

Continued on next page...

PRELIMINARY VALIDATION REPORT

Continued from previous page...

Mol	Chain	Res	Type	Atoms
14	HA	1224	CLA	O1D-CGD-O2D-CED
14	HA	1228	CLA	O1D-CGD-O2D-CED
14	HA	1207	CLA	O1D-CGD-O2D-CED
14	bA	1233	CLA	O1D-CGD-O2D-CED
14	aA	1126	CLA	O1D-CGD-O2D-CED
14	HA	1211	CLA	O1D-CGD-O2D-CED
14	GA	1126	CLA	O1D-CGD-O2D-CED
14	BA	1215	CLA	O1D-CGD-O2D-CED
14	bA	1224	CLA	O1D-CGD-O2D-CED
14	AA	1126	CLA	O1D-CGD-O2D-CED
14	BA	1233	CLA	O1D-CGD-O2D-CED
14	bA	1207	CLA	O1D-CGD-O2D-CED
14	BA	1224	CLA	O1D-CGD-O2D-CED
14	bA	1228	CLA	O1D-CGD-O2D-CED
14	BA	1211	CLA	O1D-CGD-O2D-CED
14	bA	1215	CLA	O1D-CGD-O2D-CED
14	BA	1207	CLA	O1D-CGD-O2D-CED
20	bA	5002	LMG	C29-C28-O8-C9
14	aA	1133	CLA	CBA-CGA-O2A-C1
20	BA	5002	LMG	C29-C28-O8-C9
14	AA	1133	CLA	CBA-CGA-O2A-C1
14	HA	1222	CLA	CBA-CGA-O2A-C1
14	BA	1235	CLA	CBA-CGA-O2A-C1
14	AA	1127	CLA	CBA-CGA-O2A-C1
14	HA	1224	CLA	CBA-CGA-O2A-C1
14	GA	1127	CLA	CBA-CGA-O2A-C1
20	HA	5002	LMG	C29-C28-O8-C9
14	GA	1133	CLA	CBA-CGA-O2A-C1
14	HA	1203	CLA	CBA-CGA-O2A-C1
14	bA	1224	CLA	CBA-CGA-O2A-C1
14	bA	1235	CLA	CBA-CGA-O2A-C1
14	BA	1224	CLA	CBA-CGA-O2A-C1
14	bA	1203	CLA	CBA-CGA-O2A-C1
14	bA	1222	CLA	CBA-CGA-O2A-C1
14	HA	1235	CLA	CBA-CGA-O2A-C1
14	BA	1222	CLA	CBA-CGA-O2A-C1
14	aA	1127	CLA	CBA-CGA-O2A-C1
14	BA	1203	CLA	CBA-CGA-O2A-C1
14	BA	1219	CLA	CBD-CGD-O2D-CED
14	bA	1214	CLA	CBD-CGD-O2D-CED
14	GA	1101	CLA	CBD-CGD-O2D-CED
14	bA	1223	CLA	CBD-CGD-O2D-CED

Continued on next page...

PRELIMINARY VALIDATION REPORT

Continued from previous page...

Mol	Chain	Res	Type	Atoms
14	BA	1212	CLA	CBD-CGD-O2D-CED
14	BA	1238	CLA	CBD-CGD-O2D-CED
14	aA	1012	CLA	CBD-CGD-O2D-CED
14	AA	1106	CLA	CBD-CGD-O2D-CED
14	HA	1219	CLA	CBD-CGD-O2D-CED
14	HA	1222	CLA	CBD-CGD-O2D-CED
14	BA	1235	CLA	CBD-CGD-O2D-CED
14	aA	1101	CLA	CBD-CGD-O2D-CED
14	bA	1236	CLA	CBD-CGD-O2D-CED
14	AA	1112	CLA	CBD-CGD-O2D-CED
14	AA	1101	CLA	CBD-CGD-O2D-CED
14	aA	1128	CLA	CBD-CGD-O2D-CED
14	BA	1223	CLA	CBD-CGD-O2D-CED
14	bA	1023	CLA	CBD-CGD-O2D-CED
14	GA	1105	CLA	CBD-CGD-O2D-CED
14	bA	1218	CLA	CBD-CGD-O2D-CED
14	GA	1128	CLA	CBD-CGD-O2D-CED
14	HA	1214	CLA	CBD-CGD-O2D-CED
14	AA	1128	CLA	CBD-CGD-O2D-CED
14	BA	1236	CLA	CBD-CGD-O2D-CED
14	HA	1201	CLA	CBD-CGD-O2D-CED
14	lA	1503	CLA	CBD-CGD-O2D-CED
14	aA	1112	CLA	CBD-CGD-O2D-CED
14	GA	1106	CLA	CBD-CGD-O2D-CED
14	aA	1106	CLA	CBD-CGD-O2D-CED
14	bA	1238	CLA	CBD-CGD-O2D-CED
14	HA	1238	CLA	CBD-CGD-O2D-CED
14	bA	1235	CLA	CBD-CGD-O2D-CED
14	LA	1503	CLA	CBD-CGD-O2D-CED
14	HA	1023	CLA	CBD-CGD-O2D-CED
14	bA	1222	CLA	CBD-CGD-O2D-CED
14	GA	1013	CLA	CBD-CGD-O2D-CED
14	AA	1012	CLA	CBD-CGD-O2D-CED
14	BA	1218	CLA	CBD-CGD-O2D-CED
14	bA	1201	CLA	CBD-CGD-O2D-CED
14	GA	1112	CLA	CBD-CGD-O2D-CED
14	BA	1201	CLA	CBD-CGD-O2D-CED
14	GA	1012	CLA	CBD-CGD-O2D-CED
14	AA	1105	CLA	CBD-CGD-O2D-CED
14	bA	1219	CLA	CBD-CGD-O2D-CED
14	aA	1105	CLA	CBD-CGD-O2D-CED
14	HA	1218	CLA	CBD-CGD-O2D-CED

Continued on next page...

PRELIMINARY VALIDATION REPORT

Continued from previous page...

Mol	Chain	Res	Type	Atoms
14	aA	1013	CLA	CBD-CGD-O2D-CED
14	HA	1212	CLA	CBD-CGD-O2D-CED
14	UA	1503	CLA	CBD-CGD-O2D-CED
14	HA	1236	CLA	CBD-CGD-O2D-CED
14	HA	1235	CLA	CBD-CGD-O2D-CED
14	HA	1223	CLA	CBD-CGD-O2D-CED
14	BA	1214	CLA	CBD-CGD-O2D-CED
14	BA	1222	CLA	CBD-CGD-O2D-CED
14	bA	1212	CLA	CBD-CGD-O2D-CED
14	BA	1023	CLA	CBD-CGD-O2D-CED
14	AA	1013	CLA	CBD-CGD-O2D-CED
14	GA	1111	CLA	O1A-CGA-O2A-C1
14	bA	1221	CLA	O1A-CGA-O2A-C1
20	bA	5002	LMG	O10-C28-O8-C9
14	BA	1204	CLA	O1A-CGA-O2A-C1
14	bA	1226	CLA	O1A-CGA-O2A-C1
20	BA	5002	LMG	O10-C28-O8-C9
14	BA	1221	CLA	O1A-CGA-O2A-C1
14	bA	1204	CLA	O1A-CGA-O2A-C1
14	BA	1235	CLA	O1A-CGA-O2A-C1
14	HA	1224	CLA	O1A-CGA-O2A-C1
20	HA	5002	LMG	O10-C28-O8-C9
14	HA	1202	CLA	O1A-CGA-O2A-C1
20	RA	5006	LMG	O10-C28-O8-C9
14	GA	1131	CLA	O1A-CGA-O2A-C1
14	AA	1111	CLA	O1A-CGA-O2A-C1
14	bA	1202	CLA	O1A-CGA-O2A-C1
14	bA	1224	CLA	O1A-CGA-O2A-C1
14	HA	1221	CLA	O1A-CGA-O2A-C1
14	AA	1131	CLA	O1A-CGA-O2A-C1
14	bA	1235	CLA	O1A-CGA-O2A-C1
20	iA	5006	LMG	O10-C28-O8-C9
14	BA	1224	CLA	O1A-CGA-O2A-C1
14	BA	1226	CLA	O1A-CGA-O2A-C1
14	aA	1131	CLA	O1A-CGA-O2A-C1
14	HA	1226	CLA	O1A-CGA-O2A-C1
14	HA	1235	CLA	O1A-CGA-O2A-C1
20	IA	5006	LMG	O10-C28-O8-C9
14	aA	1111	CLA	O1A-CGA-O2A-C1
14	HA	1204	CLA	O1A-CGA-O2A-C1
14	BA	1202	CLA	O1A-CGA-O2A-C1
14	AA	1115	CLA	O1D-CGD-O2D-CED

Continued on next page...

PRELIMINARY VALIDATION REPORT

Continued from previous page...

Mol	Chain	Res	Type	Atoms
14	HA	1229	CLA	O1D-CGD-O2D-CED
14	aA	1115	CLA	O1D-CGD-O2D-CED
14	BA	1232	CLA	O1D-CGD-O2D-CED
14	bA	1229	CLA	O1D-CGD-O2D-CED
14	GA	1115	CLA	O1D-CGD-O2D-CED
14	HA	1232	CLA	O1D-CGD-O2D-CED
14	BA	1229	CLA	O1D-CGD-O2D-CED
14	bA	1232	CLA	O1D-CGD-O2D-CED
14	BA	1204	CLA	CBD-CGD-O2D-CED
14	aA	1133	CLA	CBD-CGD-O2D-CED
14	AA	1133	CLA	CBD-CGD-O2D-CED
14	bA	1204	CLA	CBD-CGD-O2D-CED
14	AA	1127	CLA	CBD-CGD-O2D-CED
14	GA	1127	CLA	CBD-CGD-O2D-CED
14	GA	1133	CLA	CBD-CGD-O2D-CED
14	HA	1204	CLA	CBD-CGD-O2D-CED
14	aA	1127	CLA	CBD-CGD-O2D-CED
14	GA	1131	CLA	O1D-CGD-O2D-CED
14	AA	1131	CLA	O1D-CGD-O2D-CED
14	aA	1131	CLA	O1D-CGD-O2D-CED
14	bA	1234	CLA	C3-C5-C6-C7
14	AA	1118	CLA	C3-C5-C6-C7
14	HA	1208	CLA	C3-C5-C6-C7
14	aA	1102	CLA	C3-C5-C6-C7
14	AA	1140	CLA	C3-C5-C6-C7
14	BA	1204	CLA	C3-C5-C6-C7
14	bA	1210	CLA	C3-C5-C6-C7
14	bA	1204	CLA	C3-C5-C6-C7
14	BA	1235	CLA	C3-C5-C6-C7
14	GA	1140	CLA	C3-C5-C6-C7
14	AA	1127	CLA	C3-C5-C6-C7
14	GA	1102	CLA	C3-C5-C6-C7
14	aA	1128	CLA	C3-C5-C6-C7
14	BA	1208	CLA	C3-C5-C6-C7
14	GA	1127	CLA	C3-C5-C6-C7
14	aA	1130	CLA	C3-C5-C6-C7
14	GA	1128	CLA	C3-C5-C6-C7
14	HA	1239	CLA	C3-C5-C6-C7
14	AA	1128	CLA	C3-C5-C6-C7
14	aA	1140	CLA	C3-C5-C6-C7
14	GA	1131	CLA	C3-C5-C6-C7
14	HA	1234	CLA	C3-C5-C6-C7

Continued on next page...

PRELIMINARY VALIDATION REPORT

Continued from previous page...

Mol	Chain	Res	Type	Atoms
14	BA	1210	CLA	C3-C5-C6-C7
14	BA	1239	CLA	C3-C5-C6-C7
14	AA	1131	CLA	C3-C5-C6-C7
14	bA	1235	CLA	C3-C5-C6-C7
14	AA	1102	CLA	C3-C5-C6-C7
14	bA	1208	CLA	C3-C5-C6-C7
14	HA	1210	CLA	C3-C5-C6-C7
14	GA	1118	CLA	C3-C5-C6-C7
14	GA	1130	CLA	C3-C5-C6-C7
14	aA	1118	CLA	C3-C5-C6-C7
14	aA	1131	CLA	C3-C5-C6-C7
14	AA	1130	CLA	C3-C5-C6-C7
14	HA	1235	CLA	C3-C5-C6-C7
14	HA	1204	CLA	C3-C5-C6-C7
14	aA	1127	CLA	C3-C5-C6-C7
14	BA	1234	CLA	C3-C5-C6-C7
14	bA	1239	CLA	C3-C5-C6-C7
14	AA	1118	CLA	CBA-CGA-O2A-C1
14	aA	1102	CLA	CBA-CGA-O2A-C1
14	AA	1138	CLA	CBA-CGA-O2A-C1
14	GA	1102	CLA	CBA-CGA-O2A-C1
14	GA	1138	CLA	CBA-CGA-O2A-C1
14	GA	1131	CLA	CBA-CGA-O2A-C1
20	GA	5005	LMG	C29-C28-O8-C9
14	AA	1131	CLA	CBA-CGA-O2A-C1
14	AA	1102	CLA	CBA-CGA-O2A-C1
20	AA	5005	LMG	C29-C28-O8-C9
14	GA	1118	CLA	CBA-CGA-O2A-C1
14	aA	1118	CLA	CBA-CGA-O2A-C1
14	aA	1131	CLA	CBA-CGA-O2A-C1
20	aA	5005	LMG	C29-C28-O8-C9
14	aA	1138	CLA	CBA-CGA-O2A-C1
14	BA	1206	CLA	O1D-CGD-O2D-CED
14	bA	1206	CLA	O1D-CGD-O2D-CED
14	HA	1206	CLA	O1D-CGD-O2D-CED
14	HA	1208	CLA	CBD-CGD-O2D-CED
14	aA	1104	CLA	CBD-CGD-O2D-CED
14	GA	1113	CLA	CBD-CGD-O2D-CED
14	BA	1208	CLA	CBD-CGD-O2D-CED
14	aA	1130	CLA	CBD-CGD-O2D-CED
14	AA	1113	CLA	CBD-CGD-O2D-CED
14	bA	1208	CLA	CBD-CGD-O2D-CED

Continued on next page...

PRELIMINARY VALIDATION REPORT

Continued from previous page...

Mol	Chain	Res	Type	Atoms
14	AA	1104	CLA	CBD-CGD-O2D-CED
14	GA	1104	CLA	CBD-CGD-O2D-CED
14	GA	1130	CLA	CBD-CGD-O2D-CED
14	AA	1130	CLA	CBD-CGD-O2D-CED
14	aA	1113	CLA	CBD-CGD-O2D-CED
14	aA	1128	CLA	O1A-CGA-O2A-C1
14	GA	1128	CLA	O1A-CGA-O2A-C1
14	AA	1128	CLA	O1A-CGA-O2A-C1
14	bA	1022	CLA	C2A-CAA-CBA-CGA
14	BA	1225	CLA	C2A-CAA-CBA-CGA
14	GA	1111	CLA	C2A-CAA-CBA-CGA
14	HA	1225	CLA	C2A-CAA-CBA-CGA
14	aA	1123	CLA	C2A-CAA-CBA-CGA
14	AA	1135	CLA	C2A-CAA-CBA-CGA
14	bA	1223	CLA	C2A-CAA-CBA-CGA
14	BA	1238	CLA	C2A-CAA-CBA-CGA
14	bA	1225	CLA	C2A-CAA-CBA-CGA
14	bA	1210	CLA	C2A-CAA-CBA-CGA
14	aA	1135	CLA	C2A-CAA-CBA-CGA
14	BA	1223	CLA	C2A-CAA-CBA-CGA
14	0	1012	CLA	C2A-CAA-CBA-CGA
14	AA	1123	CLA	C2A-CAA-CBA-CGA
14	AA	1111	CLA	C2A-CAA-CBA-CGA
14	BA	1022	CLA	C2A-CAA-CBA-CGA
14	bA	1238	CLA	C2A-CAA-CBA-CGA
14	HA	1238	CLA	C2A-CAA-CBA-CGA
14	BA	1210	CLA	C2A-CAA-CBA-CGA
14	GA	1123	CLA	C2A-CAA-CBA-CGA
14	HA	1210	CLA	C2A-CAA-CBA-CGA
14	HA	1223	CLA	C2A-CAA-CBA-CGA
14	aA	1111	CLA	C2A-CAA-CBA-CGA
14	GA	1135	CLA	C2A-CAA-CBA-CGA
20	bA	5002	LMG	C17-C18-C19-C20
20	LA	5007	LMG	C35-C36-C37-C38
20	BA	5002	LMG	C17-C18-C19-C20
20	HA	5002	LMG	C17-C18-C19-C20
20	GA	5005	LMG	C35-C36-C37-C38
20	AA	5005	LMG	C35-C36-C37-C38
20	UA	5007	LMG	C35-C36-C37-C38
20	lA	5007	LMG	C35-C36-C37-C38
20	aA	5005	LMG	C35-C36-C37-C38
13	GA	1011	CL0	C4-C3-C5-C6

Continued on next page...

PRELIMINARY VALIDATION REPORT

Continued from previous page...

Mol	Chain	Res	Type	Atoms
14	HA	1216	CLA	C4-C3-C5-C6
14	aA	1130	CLA	C4-C3-C5-C6
14	bA	1216	CLA	C4-C3-C5-C6
14	BA	1216	CLA	C4-C3-C5-C6
14	GA	1130	CLA	C4-C3-C5-C6
14	HA	1216	CLA	C2-C3-C5-C6
14	aA	1130	CLA	C2-C3-C5-C6
14	bA	1216	CLA	C2-C3-C5-C6
14	BA	1216	CLA	C2-C3-C5-C6
14	GA	1130	CLA	C2-C3-C5-C6
14	AA	1130	CLA	C2-C3-C5-C6
14	bA	1022	CLA	C3-C5-C6-C7
14	AA	1138	CLA	C3-C5-C6-C7
14	0	1012	CLA	C3-C5-C6-C7
14	BA	1213	CLA	C3-C5-C6-C7
14	HA	1202	CLA	C3-C5-C6-C7
14	GA	1138	CLA	C3-C5-C6-C7
14	BA	1022	CLA	C3-C5-C6-C7
14	bA	1202	CLA	C3-C5-C6-C7
14	bA	1213	CLA	C3-C5-C6-C7
14	HA	1213	CLA	C3-C5-C6-C7
14	GA	1013	CLA	C3-C5-C6-C7
14	aA	1013	CLA	C3-C5-C6-C7
14	aA	1138	CLA	C3-C5-C6-C7
14	AA	1013	CLA	C3-C5-C6-C7
14	BA	1202	CLA	C3-C5-C6-C7
14	GA	1111	CLA	CBA-CGA-O2A-C1
14	HA	1208	CLA	CBA-CGA-O2A-C1
14	bA	1221	CLA	CBA-CGA-O2A-C1
14	BA	1204	CLA	CBA-CGA-O2A-C1
14	bA	1226	CLA	CBA-CGA-O2A-C1
14	BA	1221	CLA	CBA-CGA-O2A-C1
14	bA	1204	CLA	CBA-CGA-O2A-C1
14	BA	1208	CLA	CBA-CGA-O2A-C1
14	HA	1202	CLA	CBA-CGA-O2A-C1
20	RA	5006	LMG	C29-C28-O8-C9
14	AA	1111	CLA	CBA-CGA-O2A-C1
14	bA	1202	CLA	CBA-CGA-O2A-C1
14	HA	1221	CLA	CBA-CGA-O2A-C1
14	bA	1208	CLA	CBA-CGA-O2A-C1
20	iA	5006	LMG	C29-C28-O8-C9
14	BA	1226	CLA	CBA-CGA-O2A-C1

Continued on next page...

PRELIMINARY VALIDATION REPORT

Continued from previous page...

Mol	Chain	Res	Type	Atoms
14	HA	1226	CLA	CBA-CGA-O2A-C1
20	IA	5006	LMG	C29-C28-O8-C9
14	aA	1111	CLA	CBA-CGA-O2A-C1
14	HA	1204	CLA	CBA-CGA-O2A-C1
14	BA	1202	CLA	CBA-CGA-O2A-C1
14	GA	1116	CLA	O1D-CGD-O2D-CED
14	aA	1109	CLA	O1D-CGD-O2D-CED
14	AA	1116	CLA	O1D-CGD-O2D-CED
14	GA	1109	CLA	O1D-CGD-O2D-CED
14	aA	1116	CLA	O1D-CGD-O2D-CED
14	AA	1109	CLA	O1D-CGD-O2D-CED
14	bA	1234	CLA	CBD-CGD-O2D-CED
14	BA	1225	CLA	CBD-CGD-O2D-CED
14	GA	1103	CLA	CBD-CGD-O2D-CED
14	HA	1225	CLA	CBD-CGD-O2D-CED
14	bA	1225	CLA	CBD-CGD-O2D-CED
14	aA	1103	CLA	CBD-CGD-O2D-CED
14	AA	1103	CLA	CBD-CGD-O2D-CED
14	HA	1234	CLA	CBD-CGD-O2D-CED
14	BA	1234	CLA	CBD-CGD-O2D-CED
21	AA	6001	LMT	C4B-C5B-C6B-O6B
21	GA	6001	LMT	C4B-C5B-C6B-O6B
21	aA	6001	LMT	C4B-C5B-C6B-O6B
14	aA	1102	CLA	O1D-CGD-O2D-CED
14	aA	1107	CLA	O1D-CGD-O2D-CED
14	AA	1132	CLA	O1D-CGD-O2D-CED
14	GA	1102	CLA	O1D-CGD-O2D-CED
14	GA	1107	CLA	O1D-CGD-O2D-CED
14	aA	1132	CLA	O1D-CGD-O2D-CED
14	HA	1239	CLA	O1D-CGD-O2D-CED
14	AA	1107	CLA	O1D-CGD-O2D-CED
14	BA	1239	CLA	O1D-CGD-O2D-CED
14	GA	1132	CLA	O1D-CGD-O2D-CED
14	AA	1102	CLA	O1D-CGD-O2D-CED
14	bA	1227	CLA	O1D-CGD-O2D-CED
14	BA	1227	CLA	O1D-CGD-O2D-CED
14	HA	1227	CLA	O1D-CGD-O2D-CED
14	bA	1239	CLA	O1D-CGD-O2D-CED
14	aA	1130	CLA	O1A-CGA-O2A-C1
14	GA	1130	CLA	O1A-CGA-O2A-C1
14	AA	1130	CLA	O1A-CGA-O2A-C1
18	IA	4020	BCR	C19-C20-C21-C22

Continued on next page...

PRELIMINARY VALIDATION REPORT

Continued from previous page...

Mol	Chain	Res	Type	Atoms
18	RA	4020	BCR	C19-C20-C21-C22
18	iA	4020	BCR	C19-C20-C21-C22
18	IA	4018	BCR	C9-C10-C11-C12
18	IA	4018	BCR	C15-C16-C17-C18
18	bA	4013	BCR	C15-C16-C17-C18
18	HA	4017	BCR	C9-C10-C11-C12
18	HA	4017	BCR	C19-C20-C21-C22
18	RA	4018	BCR	C9-C10-C11-C12
18	RA	4018	BCR	C15-C16-C17-C18
18	RA	4018	BCR	C19-C20-C21-C22
18	bA	4017	BCR	C9-C10-C11-C12
18	bA	4017	BCR	C19-C20-C21-C22
18	iA	4018	BCR	C9-C10-C11-C12
18	iA	4018	BCR	C15-C16-C17-C18
18	BA	4017	BCR	C9-C10-C11-C12
18	BA	4017	BCR	C19-C20-C21-C22
18	BA	4013	BCR	C15-C16-C17-C18
18	HA	4013	BCR	C15-C16-C17-C18
14	BA	1213	CLA	O1D-CGD-O2D-CED
14	bA	1213	CLA	O1D-CGD-O2D-CED
14	HA	1213	CLA	O1D-CGD-O2D-CED
14	GA	1111	CLA	C3-C5-C6-C7
14	BA	1220	CLA	C3-C5-C6-C7
14	lA	1503	CLA	C3-C5-C6-C7
14	AA	1111	CLA	C3-C5-C6-C7
14	bA	1220	CLA	C3-C5-C6-C7
14	LA	1503	CLA	C3-C5-C6-C7
14	HA	1220	CLA	C3-C5-C6-C7
14	UA	1503	CLA	C3-C5-C6-C7
14	aA	1111	CLA	C3-C5-C6-C7
21	AA	6001	LMT	O5B-C5B-C6B-O6B
21	GA	6001	LMT	O5B-C5B-C6B-O6B
21	aA	6001	LMT	O5B-C5B-C6B-O6B
14	bA	1211	CLA	CBA-CGA-O2A-C1
14	bA	1231	CLA	CBA-CGA-O2A-C1
14	HA	1231	CLA	CBA-CGA-O2A-C1
14	aA	1117	CLA	CBA-CGA-O2A-C1
14	aA	1104	CLA	CBA-CGA-O2A-C1
14	HA	1021	CLA	CBA-CGA-O2A-C1
14	aA	1130	CLA	CBA-CGA-O2A-C1
14	AA	1128	CLA	CBA-CGA-O2A-C1
14	BA	1231	CLA	CBA-CGA-O2A-C1

Continued on next page...

PRELIMINARY VALIDATION REPORT

Continued from previous page...

Mol	Chain	Res	Type	Atoms
14	GA	1117	CLA	CBA-CGA-O2A-C1
14	BA	1021	CLA	CBA-CGA-O2A-C1
14	HA	1211	CLA	CBA-CGA-O2A-C1
14	AA	1104	CLA	CBA-CGA-O2A-C1
14	GA	1104	CLA	CBA-CGA-O2A-C1
14	GA	1130	CLA	CBA-CGA-O2A-C1
14	AA	1130	CLA	CBA-CGA-O2A-C1
14	BA	1211	CLA	CBA-CGA-O2A-C1
14	AA	1117	CLA	CBA-CGA-O2A-C1
14	bA	1021	CLA	CBA-CGA-O2A-C1
14	LA	1501	CLA	O1D-CGD-O2D-CED
14	aA	1124	CLA	O1D-CGD-O2D-CED
14	GA	1137	CLA	O1D-CGD-O2D-CED
14	AA	1124	CLA	O1D-CGD-O2D-CED
14	lA	1501	CLA	O1D-CGD-O2D-CED
14	AA	1137	CLA	O1D-CGD-O2D-CED
14	UA	1501	CLA	O1D-CGD-O2D-CED
14	aA	1137	CLA	O1D-CGD-O2D-CED
14	GA	1124	CLA	O1D-CGD-O2D-CED
20	bA	5002	LMG	C11-C10-O7-C8
20	BA	5002	LMG	C11-C10-O7-C8
20	HA	5002	LMG	C11-C10-O7-C8
21	AA	6001	LMT	C4'-C5'-C6'-O6'
21	GA	6001	LMT	C4'-C5'-C6'-O6'
21	aA	6001	LMT	C4'-C5'-C6'-O6'
14	AA	1118	CLA	O1D-CGD-O2D-CED
14	aA	1114	CLA	O1D-CGD-O2D-CED
14	GA	1114	CLA	O1D-CGD-O2D-CED
14	AA	1114	CLA	O1D-CGD-O2D-CED
14	GA	1118	CLA	O1D-CGD-O2D-CED
14	aA	1118	CLA	O1D-CGD-O2D-CED
14	AA	1104	CLA	O1A-CGA-O2A-C1
14	HA	1239	CLA	C2C-C3C-CAC-CBC
14	BA	1239	CLA	C2C-C3C-CAC-CBC
14	bA	1239	CLA	C2C-C3C-CAC-CBC
14	HA	1203	CLA	C3-C5-C6-C7
14	bA	1203	CLA	C3-C5-C6-C7
14	BA	1203	CLA	C3-C5-C6-C7
19	AA	5001	LHG	C11-C12-C13-C14
19	GA	5001	LHG	C11-C12-C13-C14
19	aA	5001	LHG	C11-C12-C13-C14
14	aA	1124	CLA	CBA-CGA-O2A-C1

Continued on next page...

PRELIMINARY VALIDATION REPORT

Continued from previous page...

Mol	Chain	Res	Type	Atoms
14	AA	1124	CLA	CBA-CGA-O2A-C1
14	aA	1128	CLA	CBA-CGA-O2A-C1
14	GA	1128	CLA	CBA-CGA-O2A-C1
14	GA	1124	CLA	CBA-CGA-O2A-C1
14	bA	1211	CLA	O1A-CGA-O2A-C1
14	HA	1208	CLA	O1A-CGA-O2A-C1
14	aA	1104	CLA	O1A-CGA-O2A-C1
14	BA	1208	CLA	O1A-CGA-O2A-C1
14	HA	1211	CLA	O1A-CGA-O2A-C1
14	bA	1208	CLA	O1A-CGA-O2A-C1
14	GA	1104	CLA	O1A-CGA-O2A-C1
14	BA	1211	CLA	O1A-CGA-O2A-C1
14	AA	1140	CLA	C2A-CAA-CBA-CGA
14	GA	1140	CLA	C2A-CAA-CBA-CGA
14	bA	1023	CLA	C2A-CAA-CBA-CGA
14	aA	1140	CLA	C2A-CAA-CBA-CGA
14	HA	1023	CLA	C2A-CAA-CBA-CGA
14	BA	1023	CLA	C2A-CAA-CBA-CGA
14	aA	1125	CLA	CBD-CGD-O2D-CED
14	BA	1220	CLA	CBD-CGD-O2D-CED
14	AA	1115	CLA	C4-C3-C5-C6
14	bA	1022	CLA	C4-C3-C5-C6
16	bA	2002	PQN	C14-C13-C15-C16
13	AA	1011	CL0	C4-C3-C5-C6
14	aA	1115	CLA	C4-C3-C5-C6
16	BA	2002	PQN	C14-C13-C15-C16
14	0	1012	CLA	C4-C3-C5-C6
14	HA	1203	CLA	C4-C3-C5-C6
14	BA	1022	CLA	C4-C3-C5-C6
14	GA	1115	CLA	C4-C3-C5-C6
14	bA	1203	CLA	C4-C3-C5-C6
14	BA	1203	CLA	C4-C3-C5-C6
13	aA	1011	CL0	C4-C3-C5-C6
16	HA	2002	PQN	C14-C13-C15-C16
14	AA	1115	CLA	C2-C3-C5-C6
14	bA	1022	CLA	C2-C3-C5-C6
16	bA	2002	PQN	C12-C13-C15-C16
14	aA	1115	CLA	C2-C3-C5-C6
16	BA	2002	PQN	C12-C13-C15-C16
14	0	1012	CLA	C2-C3-C5-C6
14	HA	1203	CLA	C2-C3-C5-C6
14	BA	1022	CLA	C2-C3-C5-C6

Continued on next page...

PRELIMINARY VALIDATION REPORT

Continued from previous page...

Mol	Chain	Res	Type	Atoms
14	GA	1115	CLA	C2-C3-C5-C6
14	bA	1203	CLA	C2-C3-C5-C6
14	BA	1203	CLA	C2-C3-C5-C6
16	HA	2002	PQN	C12-C13-C15-C16
14	HA	1239	CLA	O1A-CGA-O2A-C1
14	BA	1239	CLA	O1A-CGA-O2A-C1
14	bA	1239	CLA	O1A-CGA-O2A-C1
14	GA	1111	CLA	O1D-CGD-O2D-CED
14	BA	1219	CLA	O1D-CGD-O2D-CED
14	HA	1219	CLA	O1D-CGD-O2D-CED
14	bA	1218	CLA	O1D-CGD-O2D-CED
14	lA	1503	CLA	O1D-CGD-O2D-CED
14	LA	1503	CLA	O1D-CGD-O2D-CED
14	BA	1218	CLA	O1D-CGD-O2D-CED
14	bA	1219	CLA	O1D-CGD-O2D-CED
14	HA	1218	CLA	O1D-CGD-O2D-CED
14	UA	1503	CLA	O1D-CGD-O2D-CED
14	aA	1111	CLA	O1D-CGD-O2D-CED
14	bA	1214	CLA	O1D-CGD-O2D-CED
14	BA	1212	CLA	O1D-CGD-O2D-CED
14	AA	1106	CLA	O1D-CGD-O2D-CED
14	HA	1214	CLA	O1D-CGD-O2D-CED
14	GA	1106	CLA	O1D-CGD-O2D-CED
14	aA	1106	CLA	O1D-CGD-O2D-CED
14	AA	1111	CLA	O1D-CGD-O2D-CED
14	HA	1212	CLA	O1D-CGD-O2D-CED
14	BA	1214	CLA	O1D-CGD-O2D-CED
14	bA	1212	CLA	O1D-CGD-O2D-CED
14	GA	1125	CLA	CBD-CGD-O2D-CED
14	bA	1220	CLA	CBD-CGD-O2D-CED
14	HA	1220	CLA	CBD-CGD-O2D-CED
14	AA	1125	CLA	CBD-CGD-O2D-CED
14	HA	1229	CLA	CBA-CGA-O2A-C1
14	HA	1239	CLA	CBA-CGA-O2A-C1
14	bA	1229	CLA	CBA-CGA-O2A-C1
14	BA	1239	CLA	CBA-CGA-O2A-C1
14	BA	1229	CLA	CBA-CGA-O2A-C1
14	bA	1239	CLA	CBA-CGA-O2A-C1
14	HA	1222	CLA	C13-C15-C16-C17
14	HA	1222	CLA	C15-C16-C17-C18
14	bA	1222	CLA	C13-C15-C16-C17
14	bA	1222	CLA	C15-C16-C17-C18

Continued on next page...

PRELIMINARY VALIDATION REPORT

Continued from previous page...

Mol	Chain	Res	Type	Atoms
14	BA	1222	CLA	C13-C15-C16-C17
14	BA	1222	CLA	C15-C16-C17-C18
14	aA	1124	CLA	O1A-CGA-O2A-C1
14	bA	1231	CLA	O1A-CGA-O2A-C1
14	AA	1124	CLA	O1A-CGA-O2A-C1
14	HA	1231	CLA	O1A-CGA-O2A-C1
14	HA	1021	CLA	O1A-CGA-O2A-C1
14	BA	1231	CLA	O1A-CGA-O2A-C1
14	BA	1021	CLA	O1A-CGA-O2A-C1
14	GA	1124	CLA	O1A-CGA-O2A-C1
14	bA	1021	CLA	O1A-CGA-O2A-C1
14	GA	1101	CLA	O1D-CGD-O2D-CED
14	aA	1101	CLA	O1D-CGD-O2D-CED
14	AA	1112	CLA	O1D-CGD-O2D-CED
14	AA	1101	CLA	O1D-CGD-O2D-CED
14	aA	1112	CLA	O1D-CGD-O2D-CED
14	GA	1112	CLA	O1D-CGD-O2D-CED
20	bA	5002	LMG	O9-C10-O7-C8
20	BA	5002	LMG	O9-C10-O7-C8
20	HA	5002	LMG	O9-C10-O7-C8
14	aA	1119	CLA	C10-C11-C12-C13
14	aA	1117	CLA	C15-C16-C17-C18
14	aA	1125	CLA	C10-C11-C12-C13
14	GA	1125	CLA	C10-C11-C12-C13
14	GA	1117	CLA	C15-C16-C17-C18
14	AA	1125	CLA	C10-C11-C12-C13
14	GA	1119	CLA	C10-C11-C12-C13
14	AA	1119	CLA	C10-C11-C12-C13
14	AA	1117	CLA	C15-C16-C17-C18
14	HA	1215	CLA	C3-C5-C6-C7
14	HA	1201	CLA	C3-C5-C6-C7
14	BA	1215	CLA	C3-C5-C6-C7
14	bA	1201	CLA	C3-C5-C6-C7
14	BA	1201	CLA	C3-C5-C6-C7
14	bA	1215	CLA	C3-C5-C6-C7
14	HA	1229	CLA	O1A-CGA-O2A-C1
14	aA	1117	CLA	O1A-CGA-O2A-C1
14	GA	1117	CLA	O1A-CGA-O2A-C1
14	bA	1229	CLA	O1A-CGA-O2A-C1
14	BA	1229	CLA	O1A-CGA-O2A-C1
14	AA	1117	CLA	O1A-CGA-O2A-C1
14	AA	1115	CLA	CBA-CGA-O2A-C1

Continued on next page...

PRELIMINARY VALIDATION REPORT

Continued from previous page...

Mol	Chain	Res	Type	Atoms
14	bA	1234	CLA	CBA-CGA-O2A-C1
14	UA	1502	CLA	CBA-CGA-O2A-C1
14	aA	1119	CLA	CBA-CGA-O2A-C1
14	HA	1215	CLA	CBA-CGA-O2A-C1
14	HA	1216	CLA	CBA-CGA-O2A-C1
14	bA	1223	CLA	CBA-CGA-O2A-C1
14	BA	1223	CLA	CBA-CGA-O2A-C1
14	aA	1115	CLA	CBA-CGA-O2A-C1
14	bA	1216	CLA	CBA-CGA-O2A-C1
14	HA	1201	CLA	CBA-CGA-O2A-C1
14	HA	1234	CLA	CBA-CGA-O2A-C1
14	BA	1216	CLA	CBA-CGA-O2A-C1
14	GA	1115	CLA	CBA-CGA-O2A-C1
14	BA	1215	CLA	CBA-CGA-O2A-C1
14	lA	1502	CLA	CBA-CGA-O2A-C1
14	bA	1201	CLA	CBA-CGA-O2A-C1
14	BA	1201	CLA	CBA-CGA-O2A-C1
14	GA	1119	CLA	CBA-CGA-O2A-C1
14	AA	1119	CLA	CBA-CGA-O2A-C1
14	HA	1223	CLA	CBA-CGA-O2A-C1
14	BA	1234	CLA	CBA-CGA-O2A-C1
14	bA	1215	CLA	CBA-CGA-O2A-C1
14	LA	1502	CLA	CBA-CGA-O2A-C1
19	bA	5004	LHG	C28-C29-C30-C31
14	AA	1138	CLA	C10-C11-C12-C13
19	HA	5004	LHG	C28-C29-C30-C31
14	GA	1138	CLA	C10-C11-C12-C13
14	aA	1138	CLA	C10-C11-C12-C13
14	bA	1023	CLA	O1D-CGD-O2D-CED
14	HA	1023	CLA	O1D-CGD-O2D-CED
14	GA	1013	CLA	O1D-CGD-O2D-CED
14	aA	1013	CLA	O1D-CGD-O2D-CED
14	BA	1023	CLA	O1D-CGD-O2D-CED
14	AA	1013	CLA	O1D-CGD-O2D-CED
19	BA	5004	LHG	C28-C29-C30-C31
14	aA	1125	CLA	C3-C5-C6-C7
14	GA	1125	CLA	C3-C5-C6-C7
14	AA	1125	CLA	C3-C5-C6-C7
16	HA	2002	PQN	C13-C15-C16-C17
14	aA	1123	CLA	C6-C7-C8-C9
16	AA	2001	PQN	C19-C18-C20-C21
14	AA	1140	CLA	C6-C7-C8-C9

Continued on next page...

PRELIMINARY VALIDATION REPORT

Continued from previous page...

Mol	Chain	Res	Type	Atoms
14	aA	1133	CLA	C11-C10-C8-C9
14	AA	1133	CLA	C11-C10-C8-C9
16	GA	2001	PQN	C19-C18-C20-C21
14	GA	1140	CLA	C6-C7-C8-C9
14	aA	1104	CLA	C6-C7-C8-C9
14	aA	1140	CLA	C6-C7-C8-C9
14	AA	1123	CLA	C6-C7-C8-C9
14	GA	1133	CLA	C11-C10-C8-C9
14	aA	1126	CLA	C6-C7-C8-C9
14	GA	1126	CLA	C6-C7-C8-C9
14	GA	1123	CLA	C6-C7-C8-C9
14	AA	1126	CLA	C6-C7-C8-C9
14	AA	1104	CLA	C6-C7-C8-C9
14	GA	1104	CLA	C6-C7-C8-C9
16	aA	2001	PQN	C19-C18-C20-C21
16	bA	2002	PQN	C18-C20-C21-C22
14	bA	1023	CLA	C13-C15-C16-C17
16	BA	2002	PQN	C18-C20-C21-C22
14	HA	1023	CLA	C13-C15-C16-C17
14	BA	1023	CLA	C13-C15-C16-C17
16	HA	2002	PQN	C18-C20-C21-C22
18	aA	4011	BCR	C37-C22-C23-C24
18	HA	4005	BCR	C36-C18-C19-C20
20	bA	5002	LMG	C2-C1-O1-C7
18	bA	4005	BCR	C36-C18-C19-C20
20	BA	5002	LMG	C2-C1-O1-C7
18	aA	4007	BCR	C36-C18-C19-C20
20	HA	5002	LMG	C2-C1-O1-C7
18	AA	4002	BCR	C7-C8-C9-C34
18	BA	4005	BCR	C36-C18-C19-C20
18	LA	4022	BCR	C7-C8-C9-C34
18	LA	4022	BCR	C11-C12-C13-C35
18	aA	4002	BCR	C7-C8-C9-C34
18	bA	4004	BCR	C7-C8-C9-C34
18	bA	4004	BCR	C37-C22-C23-C24
18	GA	4011	BCR	C37-C22-C23-C24
18	HA	4004	BCR	C7-C8-C9-C34
18	HA	4004	BCR	C37-C22-C23-C24
18	GA	4002	BCR	C7-C8-C9-C34
18	bA	4006	BCR	C7-C8-C9-C34
18	bA	4006	BCR	C37-C22-C23-C24
18	AA	4007	BCR	C36-C18-C19-C20

Continued on next page...

PRELIMINARY VALIDATION REPORT

Continued from previous page...

Mol	Chain	Res	Type	Atoms
18	HA	4006	BCR	C7-C8-C9-C34
18	HA	4006	BCR	C37-C22-C23-C24
18	lA	4022	BCR	C7-C8-C9-C34
18	lA	4022	BCR	C11-C12-C13-C35
18	BA	4006	BCR	C7-C8-C9-C34
18	BA	4006	BCR	C37-C22-C23-C24
18	AA	4011	BCR	C37-C22-C23-C24
18	UA	4022	BCR	C7-C8-C9-C34
18	UA	4022	BCR	C11-C12-C13-C35
18	BA	4004	BCR	C7-C8-C9-C34
18	BA	4004	BCR	C37-C22-C23-C24
18	GA	4007	BCR	C36-C18-C19-C20
14	bA	1223	CLA	O1A-CGA-O2A-C1
14	HA	1223	CLA	O1A-CGA-O2A-C1
18	aA	4011	BCR	C21-C22-C23-C24
18	BA	4012	BCR	C7-C8-C9-C10
18	bA	4012	BCR	C7-C8-C9-C10
18	AA	4001	BCR	C7-C8-C9-C10
18	aA	4001	BCR	C7-C8-C9-C10
18	AA	4002	BCR	C7-C8-C9-C10
18	LA	4022	BCR	C7-C8-C9-C10
18	aA	4002	BCR	C7-C8-C9-C10
18	bA	4004	BCR	C7-C8-C9-C10
18	bA	4004	BCR	C21-C22-C23-C24
18	GA	4011	BCR	C21-C22-C23-C24
18	HA	4004	BCR	C7-C8-C9-C10
18	HA	4004	BCR	C21-C22-C23-C24
18	GA	4002	BCR	C7-C8-C9-C10
18	HA	4012	BCR	C7-C8-C9-C10
18	GA	4001	BCR	C7-C8-C9-C10
18	bA	4006	BCR	C7-C8-C9-C10
18	bA	4006	BCR	C21-C22-C23-C24
18	HA	4006	BCR	C7-C8-C9-C10
18	HA	4006	BCR	C21-C22-C23-C24
18	lA	4022	BCR	C7-C8-C9-C10
18	BA	4006	BCR	C7-C8-C9-C10
18	BA	4006	BCR	C21-C22-C23-C24
18	AA	4011	BCR	C21-C22-C23-C24
18	UA	4022	BCR	C7-C8-C9-C10
18	BA	4004	BCR	C7-C8-C9-C10
18	BA	4004	BCR	C21-C22-C23-C24
14	BA	1238	CLA	O1D-CGD-O2D-CED

Continued on next page...

PRELIMINARY VALIDATION REPORT

Continued from previous page...

Mol	Chain	Res	Type	Atoms
14	aA	1128	CLA	O1D-CGD-O2D-CED
14	GA	1105	CLA	O1D-CGD-O2D-CED
14	GA	1128	CLA	O1D-CGD-O2D-CED
14	AA	1128	CLA	O1D-CGD-O2D-CED
14	bA	1238	CLA	O1D-CGD-O2D-CED
14	HA	1238	CLA	O1D-CGD-O2D-CED
14	GA	1012	CLA	O1D-CGD-O2D-CED
14	AA	1105	CLA	O1D-CGD-O2D-CED
14	aA	1105	CLA	O1D-CGD-O2D-CED
14	aA	1012	CLA	O1D-CGD-O2D-CED
14	AA	1012	CLA	O1D-CGD-O2D-CED
14	BA	1225	CLA	C5-C6-C7-C8
14	aA	1119	CLA	C15-C16-C17-C18
14	HA	1225	CLA	C5-C6-C7-C8
14	aA	1102	CLA	C10-C11-C12-C13
14	HA	1215	CLA	C10-C11-C12-C13
14	bA	1221	CLA	C10-C11-C12-C13
14	bA	1214	CLA	C8-C10-C11-C12
16	bA	2002	PQN	C20-C21-C22-C23
14	BA	1204	CLA	C5-C6-C7-C8
14	BA	1204	CLA	C10-C11-C12-C13
14	bA	1225	CLA	C5-C6-C7-C8
14	AA	1132	CLA	C5-C6-C7-C8
14	BA	1221	CLA	C10-C11-C12-C13
14	bA	1210	CLA	C10-C11-C12-C13
14	bA	1204	CLA	C5-C6-C7-C8
14	bA	1204	CLA	C10-C11-C12-C13
14	GA	1102	CLA	C10-C11-C12-C13
14	aA	1125	CLA	C8-C10-C11-C12
14	aA	1125	CLA	C15-C16-C17-C18
14	aA	1132	CLA	C5-C6-C7-C8
16	BA	2002	PQN	C20-C21-C22-C23
14	HA	1214	CLA	C8-C10-C11-C12
14	BA	1213	CLA	C5-C6-C7-C8
14	GA	1125	CLA	C8-C10-C11-C12
14	GA	1125	CLA	C15-C16-C17-C18
14	bA	1213	CLA	C5-C6-C7-C8
14	BA	1215	CLA	C10-C11-C12-C13
14	BA	1210	CLA	C10-C11-C12-C13
14	HA	1221	CLA	C10-C11-C12-C13
14	GA	1132	CLA	C5-C6-C7-C8
14	AA	1102	CLA	C10-C11-C12-C13

Continued on next page...

PRELIMINARY VALIDATION REPORT

Continued from previous page...

Mol	Chain	Res	Type	Atoms
14	HA	1210	CLA	C10-C11-C12-C13
14	HA	1213	CLA	C5-C6-C7-C8
14	AA	1125	CLA	C8-C10-C11-C12
14	AA	1125	CLA	C15-C16-C17-C18
14	GA	1119	CLA	C15-C16-C17-C18
14	AA	1119	CLA	C15-C16-C17-C18
14	HA	1204	CLA	C5-C6-C7-C8
14	HA	1204	CLA	C10-C11-C12-C13
14	BA	1214	CLA	C8-C10-C11-C12
14	bA	1215	CLA	C10-C11-C12-C13
16	HA	2002	PQN	C20-C21-C22-C23
20	bA	5002	LMG	C10-C11-C12-C13
20	BA	5002	LMG	C10-C11-C12-C13
20	HA	5002	LMG	C10-C11-C12-C13
14	BA	1223	CLA	O1A-CGA-O2A-C1
14	HA	1201	CLA	O1A-CGA-O2A-C1
14	bA	1201	CLA	O1A-CGA-O2A-C1
14	BA	1201	CLA	O1A-CGA-O2A-C1
14	bA	1211	CLA	C5-C6-C7-C8
14	bA	1211	CLA	C15-C16-C17-C18
14	bA	1214	CLA	C5-C6-C7-C8
14	AA	1140	CLA	C5-C6-C7-C8
14	BA	1204	CLA	C13-C15-C16-C17
14	bA	1226	CLA	C13-C15-C16-C17
14	bA	1204	CLA	C13-C15-C16-C17
14	GA	1140	CLA	C5-C6-C7-C8
14	aA	1117	CLA	C10-C11-C12-C13
14	HA	1239	CLA	C10-C11-C12-C13
14	HA	1214	CLA	C5-C6-C7-C8
14	aA	1140	CLA	C5-C6-C7-C8
14	GA	1117	CLA	C10-C11-C12-C13
14	HA	1211	CLA	C5-C6-C7-C8
14	BA	1239	CLA	C10-C11-C12-C13
14	BA	1226	CLA	C13-C15-C16-C17
14	HA	1226	CLA	C13-C15-C16-C17
14	BA	1211	CLA	C5-C6-C7-C8
14	BA	1211	CLA	C15-C16-C17-C18
14	HA	1204	CLA	C13-C15-C16-C17
14	BA	1214	CLA	C5-C6-C7-C8
14	AA	1117	CLA	C10-C11-C12-C13
14	bA	1239	CLA	C10-C11-C12-C13
16	bA	2002	PQN	C13-C15-C16-C17

Continued on next page...

PRELIMINARY VALIDATION REPORT

Continued from previous page...

Mol	Chain	Res	Type	Atoms
14	BA	1217	CLA	C3-C5-C6-C7
14	bA	1217	CLA	C3-C5-C6-C7
16	BA	2002	PQN	C13-C15-C16-C17
14	HA	1217	CLA	C3-C5-C6-C7
14	HA	1239	CLA	C4C-C3C-CAC-CBC
14	BA	1239	CLA	C4C-C3C-CAC-CBC
14	bA	1239	CLA	C4C-C3C-CAC-CBC
13	GA	1011	CL0	CBA-CGA-O2A-C1
13	AA	1011	CL0	CBA-CGA-O2A-C1
13	aA	1011	CL0	CBA-CGA-O2A-C1
14	HA	1222	CLA	O1D-CGD-O2D-CED
14	bA	1236	CLA	O1D-CGD-O2D-CED
14	BA	1236	CLA	O1D-CGD-O2D-CED
14	bA	1222	CLA	O1D-CGD-O2D-CED
14	HA	1236	CLA	O1D-CGD-O2D-CED
14	BA	1222	CLA	O1D-CGD-O2D-CED
14	LA	1501	CLA	C10-C11-C12-C13
14	AA	1122	CLA	C5-C6-C7-C8
14	aA	1122	CLA	C5-C6-C7-C8
14	lA	1501	CLA	C10-C11-C12-C13
14	UA	1501	CLA	C10-C11-C12-C13
14	HA	1211	CLA	C15-C16-C17-C18
14	AA	1104	CLA	C15-C16-C17-C18
14	GA	1013	CLA	C5-C6-C7-C8
14	GA	1122	CLA	C5-C6-C7-C8
14	aA	1013	CLA	C5-C6-C7-C8
14	AA	1013	CLA	C5-C6-C7-C8
19	AA	5001	LHG	C23-C24-C25-C26
19	GA	5001	LHG	C23-C24-C25-C26
19	aA	5001	LHG	C23-C24-C25-C26
14	bA	1223	CLA	C8-C10-C11-C12
14	HA	1229	CLA	C8-C10-C11-C12
14	BA	1223	CLA	C8-C10-C11-C12
14	aA	1104	CLA	C15-C16-C17-C18
14	bA	1229	CLA	C8-C10-C11-C12
14	GA	1104	CLA	C15-C16-C17-C18
14	HA	1223	CLA	C8-C10-C11-C12
14	bA	1221	CLA	C3-C5-C6-C7
14	BA	1221	CLA	C3-C5-C6-C7
14	HA	1221	CLA	C3-C5-C6-C7
14	lA	1503	CLA	C8-C10-C11-C12
14	LA	1503	CLA	C8-C10-C11-C12

Continued on next page...

PRELIMINARY VALIDATION REPORT

Continued from previous page...

Mol	Chain	Res	Type	Atoms
14	BA	1229	CLA	C8-C10-C11-C12
14	UA	1503	CLA	C8-C10-C11-C12
14	UA	1502	CLA	C2-C1-O2A-CGA
14	GA	1111	CLA	C2-C1-O2A-CGA
14	aA	1119	CLA	C2-C1-O2A-CGA
14	AA	1140	CLA	C2-C1-O2A-CGA
14	GA	1140	CLA	C2-C1-O2A-CGA
14	aA	1140	CLA	C2-C1-O2A-CGA
14	AA	1111	CLA	C2-C1-O2A-CGA
14	lA	1502	CLA	C2-C1-O2A-CGA
14	GA	1119	CLA	C2-C1-O2A-CGA
14	AA	1119	CLA	C2-C1-O2A-CGA
14	aA	1111	CLA	C2-C1-O2A-CGA
14	LA	1502	CLA	C2-C1-O2A-CGA
14	HA	1208	CLA	O1D-CGD-O2D-CED
14	bA	1204	CLA	O1D-CGD-O2D-CED
14	BA	1208	CLA	O1D-CGD-O2D-CED
14	bA	1208	CLA	O1D-CGD-O2D-CED
14	UA	1502	CLA	C6-C7-C8-C10
14	HA	1229	CLA	C6-C7-C8-C10
14	HA	1222	CLA	C6-C7-C8-C10
14	AA	1127	CLA	C6-C7-C8-C10
14	HA	1205	CLA	C11-C12-C13-C15
14	aA	1125	CLA	C11-C12-C13-C15
14	bA	1205	CLA	C11-C12-C13-C15
14	GA	1113	CLA	C2A-CAA-CBA-CGA
14	HA	1021	CLA	C6-C7-C8-C10
14	GA	1127	CLA	C6-C7-C8-C10
14	GA	1125	CLA	C11-C12-C13-C15
14	BA	1021	CLA	C6-C7-C8-C10
14	bA	1229	CLA	C6-C7-C8-C10
14	HA	1203	CLA	C12-C13-C15-C16
14	BA	1205	CLA	C11-C12-C13-C15
14	AA	1113	CLA	C2A-CAA-CBA-CGA
14	bA	1203	CLA	C12-C13-C15-C16
14	bA	1227	CLA	C2A-CAA-CBA-CGA
14	bA	1222	CLA	C6-C7-C8-C10
14	lA	1502	CLA	C6-C7-C8-C10
14	BA	1229	CLA	C6-C7-C8-C10
14	BA	1227	CLA	C2A-CAA-CBA-CGA
14	AA	1125	CLA	C11-C12-C13-C15
14	BA	1222	CLA	C6-C7-C8-C10

Continued on next page...

PRELIMINARY VALIDATION REPORT

Continued from previous page...

Mol	Chain	Res	Type	Atoms
14	aA	1127	CLA	C6-C7-C8-C10
14	HA	1227	CLA	C2A-CAA-CBA-CGA
14	bA	1021	CLA	C6-C7-C8-C10
14	BA	1203	CLA	C12-C13-C15-C16
14	LA	1502	CLA	C6-C7-C8-C10
14	aA	1113	CLA	C2A-CAA-CBA-CGA
16	bA	2002	PQN	C25-C26-C27-C28
14	aA	1133	CLA	C10-C11-C12-C13
14	AA	1133	CLA	C10-C11-C12-C13
16	GA	2001	PQN	C23-C25-C26-C27
14	aA	1128	CLA	C10-C11-C12-C13
14	HA	1021	CLA	C8-C10-C11-C12
16	BA	2002	PQN	C25-C26-C27-C28
14	GA	1128	CLA	C10-C11-C12-C13
14	AA	1128	CLA	C10-C11-C12-C13
14	BA	1021	CLA	C8-C10-C11-C12
14	GA	1133	CLA	C10-C11-C12-C13
16	aA	2001	PQN	C23-C25-C26-C27
14	bA	1021	CLA	C8-C10-C11-C12
16	HA	2002	PQN	C25-C26-C27-C28
14	bA	1223	CLA	O1D-CGD-O2D-CED
14	BA	1204	CLA	O1D-CGD-O2D-CED
14	BA	1235	CLA	O1D-CGD-O2D-CED
14	AA	1127	CLA	O1D-CGD-O2D-CED
14	BA	1223	CLA	O1D-CGD-O2D-CED
14	GA	1127	CLA	O1D-CGD-O2D-CED
14	HA	1201	CLA	O1D-CGD-O2D-CED
14	bA	1235	CLA	O1D-CGD-O2D-CED
14	bA	1201	CLA	O1D-CGD-O2D-CED
14	BA	1201	CLA	O1D-CGD-O2D-CED
14	HA	1235	CLA	O1D-CGD-O2D-CED
14	HA	1223	CLA	O1D-CGD-O2D-CED
14	HA	1204	CLA	O1D-CGD-O2D-CED
14	aA	1127	CLA	O1D-CGD-O2D-CED
14	AA	1132	CLA	C2A-CAA-CBA-CGA
14	aA	1132	CLA	C2A-CAA-CBA-CGA
14	BA	1213	CLA	C2A-CAA-CBA-CGA
14	bA	1213	CLA	C2A-CAA-CBA-CGA
14	GA	1132	CLA	C2A-CAA-CBA-CGA
14	HA	1213	CLA	C2A-CAA-CBA-CGA
14	aA	1136	CLA	C3-C5-C6-C7
14	AA	1136	CLA	C3-C5-C6-C7

Continued on next page...

PRELIMINARY VALIDATION REPORT

Continued from previous page...

Mol	Chain	Res	Type	Atoms
14	GA	1136	CLA	C3-C5-C6-C7
18	aA	4003	BCR	C19-C20-C21-C22
18	IA	4020	BCR	C15-C16-C17-C18
18	bA	4014	BCR	C9-C10-C11-C12
18	AA	4003	BCR	C19-C20-C21-C22
18	RA	4020	BCR	C15-C16-C17-C18
18	iA	4020	BCR	C15-C16-C17-C18
18	HA	4014	BCR	C9-C10-C11-C12
18	aA	4007	BCR	C19-C20-C21-C22
18	bA	4013	BCR	C19-C20-C21-C22
18	BA	4014	BCR	C9-C10-C11-C12
18	AA	4007	BCR	C19-C20-C21-C22
18	GA	4003	BCR	C19-C20-C21-C22
18	BA	4013	BCR	C19-C20-C21-C22
18	HA	4013	BCR	C19-C20-C21-C22
18	GA	4007	BCR	C19-C20-C21-C22
16	AA	2001	PQN	C23-C25-C26-C27
14	bA	1234	CLA	O1A-CGA-O2A-C1
14	HA	1216	CLA	O1A-CGA-O2A-C1
14	bA	1216	CLA	O1A-CGA-O2A-C1
14	HA	1234	CLA	O1A-CGA-O2A-C1
14	GA	1115	CLA	O1A-CGA-O2A-C1
14	BA	1234	CLA	O1A-CGA-O2A-C1
14	AA	1138	CLA	C4-C3-C5-C6
14	GA	1138	CLA	C4-C3-C5-C6
14	aA	1138	CLA	C4-C3-C5-C6
14	AA	1138	CLA	C2-C3-C5-C6
14	GA	1138	CLA	C2-C3-C5-C6
14	aA	1138	CLA	C2-C3-C5-C6
14	bA	1234	CLA	C5-C6-C7-C8
14	aA	1119	CLA	C5-C6-C7-C8
14	BA	1221	CLA	C5-C6-C7-C8
14	aA	1104	CLA	C10-C11-C12-C13
14	HA	1021	CLA	C15-C16-C17-C18
14	BA	1021	CLA	C15-C16-C17-C18
14	HA	1203	CLA	C15-C16-C17-C18
14	HA	1234	CLA	C5-C6-C7-C8
14	AA	1104	CLA	C10-C11-C12-C13
14	bA	1203	CLA	C15-C16-C17-C18
14	GA	1104	CLA	C10-C11-C12-C13
14	GA	1119	CLA	C5-C6-C7-C8
14	AA	1119	CLA	C5-C6-C7-C8

Continued on next page...

PRELIMINARY VALIDATION REPORT

Continued from previous page...

Mol	Chain	Res	Type	Atoms
14	bA	1021	CLA	C15-C16-C17-C18
14	BA	1234	CLA	C5-C6-C7-C8
14	BA	1203	CLA	C15-C16-C17-C18
14	AA	1115	CLA	O1A-CGA-O2A-C1
14	aA	1119	CLA	O1A-CGA-O2A-C1
14	aA	1115	CLA	O1A-CGA-O2A-C1
14	BA	1216	CLA	O1A-CGA-O2A-C1
14	GA	1119	CLA	O1A-CGA-O2A-C1
14	AA	1119	CLA	O1A-CGA-O2A-C1
14	aA	1130	CLA	O1D-CGD-O2D-CED
14	AA	1130	CLA	O1D-CGD-O2D-CED
18	MA	4021	BCR	C10-C11-C12-C13
18	AA	4002	BCR	C10-C11-C12-C13
18	LA	4022	BCR	C10-C11-C12-C13
18	aA	4002	BCR	C10-C11-C12-C13
18	bA	4004	BCR	C10-C11-C12-C13
18	HA	4004	BCR	C10-C11-C12-C13
18	GA	4002	BCR	C10-C11-C12-C13
18	bA	4006	BCR	C10-C11-C12-C13
18	HA	4006	BCR	C10-C11-C12-C13
18	RA	4018	BCR	C10-C11-C12-C13
18	lA	4022	BCR	C10-C11-C12-C13
18	BA	4006	BCR	C10-C11-C12-C13
18	mA	4021	BCR	C10-C11-C12-C13
18	UA	4022	BCR	C10-C11-C12-C13
18	BA	4004	BCR	C10-C11-C12-C13
18	VA	4021	BCR	C10-C11-C12-C13
14	GA	1130	CLA	O1D-CGD-O2D-CED
14	bA	1022	CLA	C13-C15-C16-C17
14	aA	1119	CLA	C8-C10-C11-C12
14	bA	1221	CLA	C5-C6-C7-C8
20	LA	5007	LMG	C20-C21-C22-C23
14	aA	1012	CLA	C15-C16-C17-C18
14	0	1012	CLA	C13-C15-C16-C17
14	lA	1503	CLA	C13-C15-C16-C17
14	HA	1203	CLA	C8-C10-C11-C12
14	BA	1022	CLA	C13-C15-C16-C17
14	aA	1126	CLA	C10-C11-C12-C13
14	HA	1221	CLA	C5-C6-C7-C8
14	LA	1503	CLA	C13-C15-C16-C17
14	bA	1203	CLA	C8-C10-C11-C12
20	UA	5007	LMG	C20-C21-C22-C23

Continued on next page...

PRELIMINARY VALIDATION REPORT

Continued from previous page...

Mol	Chain	Res	Type	Atoms
14	AA	1012	CLA	C15-C16-C17-C18
20	lA	5007	LMG	C20-C21-C22-C23
14	GA	1012	CLA	C15-C16-C17-C18
14	GA	1119	CLA	C8-C10-C11-C12
14	UA	1503	CLA	C13-C15-C16-C17
14	AA	1119	CLA	C8-C10-C11-C12
14	BA	1203	CLA	C8-C10-C11-C12
14	aA	1133	CLA	O1D-CGD-O2D-CED
14	GA	1133	CLA	O1D-CGD-O2D-CED
19	bA	5004	LHG	C4-O6-P-O3
19	BA	5004	LHG	C4-O6-P-O3
19	HA	5004	LHG	C4-O6-P-O3
14	UA	1502	CLA	O1A-CGA-O2A-C1
14	lA	1502	CLA	O1A-CGA-O2A-C1
14	LA	1502	CLA	O1A-CGA-O2A-C1
14	bA	1022	CLA	C8-C10-C11-C12
14	BA	1225	CLA	C15-C16-C17-C18
14	HA	1225	CLA	C15-C16-C17-C18
14	bA	1225	CLA	C15-C16-C17-C18
14	HA	1222	CLA	C5-C6-C7-C8
14	AA	1127	CLA	C10-C11-C12-C13
14	AA	1127	CLA	C13-C15-C16-C17
14	HA	1205	CLA	C10-C11-C12-C13
14	HA	1021	CLA	C13-C15-C16-C17
14	GA	1127	CLA	C10-C11-C12-C13
14	GA	1127	CLA	C13-C15-C16-C17
14	0	1012	CLA	C8-C10-C11-C12
14	BA	1021	CLA	C13-C15-C16-C17
14	BA	1022	CLA	C8-C10-C11-C12
14	BA	1205	CLA	C10-C11-C12-C13
14	GA	1126	CLA	C10-C11-C12-C13
14	AA	1126	CLA	C10-C11-C12-C13
14	bA	1222	CLA	C5-C6-C7-C8
14	BA	1222	CLA	C5-C6-C7-C8
14	aA	1127	CLA	C10-C11-C12-C13
14	aA	1127	CLA	C13-C15-C16-C17
14	bA	1021	CLA	C13-C15-C16-C17
14	AA	1133	CLA	O1D-CGD-O2D-CED
14	GA	1113	CLA	O1D-CGD-O2D-CED
14	AA	1113	CLA	O1D-CGD-O2D-CED
14	aA	1113	CLA	O1D-CGD-O2D-CED
14	bA	1211	CLA	C8-C10-C11-C12

Continued on next page...

PRELIMINARY VALIDATION REPORT

Continued from previous page...

Mol	Chain	Res	Type	Atoms
14	bA	1205	CLA	C10-C11-C12-C13
14	HA	1211	CLA	C8-C10-C11-C12
14	BA	1211	CLA	C8-C10-C11-C12
14	HA	1215	CLA	O1A-CGA-O2A-C1
14	BA	1215	CLA	O1A-CGA-O2A-C1
14	bA	1215	CLA	O1A-CGA-O2A-C1
14	aA	1104	CLA	O1D-CGD-O2D-CED
14	GA	1104	CLA	O1D-CGD-O2D-CED
14	AA	1104	CLA	O1D-CGD-O2D-CED
14	BA	1238	CLA	CBA-CGA-O2A-C1
14	bA	1238	CLA	CBA-CGA-O2A-C1
14	HA	1238	CLA	CBA-CGA-O2A-C1
14	aA	1012	CLA	C2A-CAA-CBA-CGA
14	HA	1224	CLA	C2A-CAA-CBA-CGA
14	bA	1224	CLA	C2A-CAA-CBA-CGA
14	BA	1224	CLA	C2A-CAA-CBA-CGA
14	AA	1012	CLA	C2A-CAA-CBA-CGA
14	GA	1012	CLA	C2A-CAA-CBA-CGA
14	HA	1205	CLA	C3-C5-C6-C7
14	bA	1205	CLA	C3-C5-C6-C7
14	aA	1126	CLA	C3-C5-C6-C7
14	BA	1205	CLA	C3-C5-C6-C7
14	GA	1126	CLA	C3-C5-C6-C7
14	AA	1126	CLA	C3-C5-C6-C7
14	GA	1103	CLA	C11-C12-C13-C15
14	HA	1215	CLA	C11-C12-C13-C15
14	aA	1103	CLA	C11-C12-C13-C15
14	AA	1103	CLA	C11-C12-C13-C15
14	BA	1215	CLA	C11-C12-C13-C15
14	bA	1215	CLA	C11-C12-C13-C15
14	BA	1238	CLA	C4-C3-C5-C6
14	bA	1238	CLA	C4-C3-C5-C6
14	HA	1238	CLA	C4-C3-C5-C6
14	GA	1110	CLA	C2A-CAA-CBA-CGA
14	AA	1110	CLA	C2A-CAA-CBA-CGA
14	aA	1110	CLA	C2A-CAA-CBA-CGA
18	LA	4019	BCR	C11-C10-C9-C34
18	lA	4019	BCR	C11-C10-C9-C34
18	UA	4019	BCR	C11-C10-C9-C34
20	LA	5007	LMG	C29-C28-O8-C9
20	UA	5007	LMG	C29-C28-O8-C9
20	lA	5007	LMG	C29-C28-O8-C9

Continued on next page...

PRELIMINARY VALIDATION REPORT

Continued from previous page...

Mol	Chain	Res	Type	Atoms
19	AA	5001	LHG	C29-C30-C31-C32
19	bA	5004	LHG	C11-C12-C13-C14
19	BA	5004	LHG	C11-C12-C13-C14
19	HA	5004	LHG	C11-C12-C13-C14
14	aA	1117	CLA	C13-C15-C16-C17
19	GA	5001	LHG	C29-C30-C31-C32
14	GA	1117	CLA	C13-C15-C16-C17
20	GA	5005	LMG	C13-C14-C15-C16
20	AA	5005	LMG	C13-C14-C15-C16
19	aA	5001	LHG	C29-C30-C31-C32
20	aA	5005	LMG	C13-C14-C15-C16
14	AA	1117	CLA	C13-C15-C16-C17
18	AA	4008	BCR	C9-C10-C11-C12
19	bA	5004	LHG	C25-C26-C27-C28
19	BA	5004	LHG	C25-C26-C27-C28
19	HA	5004	LHG	C25-C26-C27-C28
19	AA	5001	LHG	C8-C7-O7-C5
19	GA	5001	LHG	C8-C7-O7-C5
19	aA	5001	LHG	C8-C7-O7-C5
14	AA	1122	CLA	C3-C5-C6-C7
14	aA	1122	CLA	C3-C5-C6-C7
14	GA	1122	CLA	C3-C5-C6-C7
19	AA	5001	LHG	O9-C7-O7-C5
19	GA	5001	LHG	O9-C7-O7-C5
19	aA	5001	LHG	O9-C7-O7-C5
14	BA	1225	CLA	C16-C17-C18-C19
13	GA	1011	CL0	C16-C17-C18-C20
14	HA	1225	CLA	C16-C17-C18-C19
14	bA	1225	CLA	C16-C17-C18-C19
13	AA	1011	CL0	C16-C17-C18-C20
13	aA	1011	CL0	C16-C17-C18-C20
18	HA	4016	BCR	C11-C10-C9-C8
18	BA	4016	BCR	C11-C10-C9-C8
18	IA	4020	BCR	C11-C10-C9-C8
18	RA	4020	BCR	C11-C10-C9-C8
18	LA	4019	BCR	C11-C10-C9-C8
18	iA	4020	BCR	C11-C10-C9-C8
18	lA	4019	BCR	C11-C10-C9-C8
18	bA	4016	BCR	C11-C10-C9-C8
18	UA	4019	BCR	C11-C10-C9-C8
13	AA	1011	CL0	O1A-CGA-O2A-C1
13	aA	1011	CL0	O1A-CGA-O2A-C1

Continued on next page...

PRELIMINARY VALIDATION REPORT

Continued from previous page...

Mol	Chain	Res	Type	Atoms
20	iA	5006	LMG	C32-C33-C34-C35
20	IA	5006	LMG	C32-C33-C34-C35
14	BA	1225	CLA	O1D-CGD-O2D-CED
14	HA	1225	CLA	O1D-CGD-O2D-CED
14	bA	1225	CLA	O1D-CGD-O2D-CED
19	AA	5001	LHG	C30-C31-C32-C33
20	RA	5006	LMG	C32-C33-C34-C35
19	GA	5001	LHG	C30-C31-C32-C33
19	aA	5001	LHG	C30-C31-C32-C33
14	HA	1215	CLA	C6-C7-C8-C9
14	bA	1210	CLA	C11-C12-C13-C14
14	aA	1125	CLA	C11-C12-C13-C14
14	HA	1021	CLA	C6-C7-C8-C9
14	GA	1125	CLA	C11-C12-C13-C14
14	BA	1021	CLA	C6-C7-C8-C9
14	HA	1203	CLA	C14-C13-C15-C16
14	BA	1215	CLA	C6-C7-C8-C9
14	BA	1210	CLA	C11-C12-C13-C14
14	HA	1210	CLA	C11-C12-C13-C14
14	bA	1203	CLA	C14-C13-C15-C16
14	AA	1125	CLA	C11-C12-C13-C14
14	bA	1021	CLA	C6-C7-C8-C9
14	bA	1215	CLA	C6-C7-C8-C9
14	BA	1203	CLA	C14-C13-C15-C16
18	AA	4001	BCR	C7-C8-C9-C34
18	aA	4001	BCR	C7-C8-C9-C34
18	GA	4001	BCR	C7-C8-C9-C34
14	bA	1022	CLA	C16-C17-C18-C20
14	aA	1133	CLA	C11-C12-C13-C14
14	AA	1133	CLA	C11-C12-C13-C14
14	0	1012	CLA	C16-C17-C18-C20
14	aA	1136	CLA	C6-C7-C8-C10
14	GA	1133	CLA	C11-C12-C13-C14
14	BA	1022	CLA	C16-C17-C18-C20
14	AA	1136	CLA	C6-C7-C8-C10
14	HA	1238	CLA	C16-C17-C18-C20
14	GA	1136	CLA	C6-C7-C8-C10
14	bA	1214	CLA	C2A-CAA-CBA-CGA
14	HA	1214	CLA	C2A-CAA-CBA-CGA
14	BA	1214	CLA	C2A-CAA-CBA-CGA
13	GA	1011	CL0	O1A-CGA-O2A-C1
18	AA	4002	BCR	C21-C22-C23-C24

Continued on next page...

PRELIMINARY VALIDATION REPORT

Continued from previous page...

Mol	Chain	Res	Type	Atoms
18	aA	4002	BCR	C21-C22-C23-C24
18	GA	4002	BCR	C21-C22-C23-C24
19	bA	5004	LHG	C32-C33-C34-C35
20	bA	5002	LMG	C34-C35-C36-C37
20	BA	5002	LMG	C34-C35-C36-C37
19	BA	5004	LHG	C32-C33-C34-C35
19	HA	5004	LHG	C32-C33-C34-C35
20	HA	5002	LMG	C34-C35-C36-C37
19	bA	5004	LHG	C23-C24-C25-C26
19	BA	5004	LHG	C23-C24-C25-C26
19	HA	5004	LHG	C23-C24-C25-C26
20	GA	5005	LMG	O9-C10-O7-C8
20	AA	5005	LMG	O9-C10-O7-C8
20	aA	5005	LMG	O9-C10-O7-C8
14	LA	1501	CLA	C4-C3-C5-C6
14	HA	1205	CLA	C4-C3-C5-C6
14	bA	1205	CLA	C4-C3-C5-C6
14	lA	1501	CLA	C4-C3-C5-C6
14	BA	1205	CLA	C4-C3-C5-C6
14	UA	1501	CLA	C4-C3-C5-C6
13	GA	1011	CL0	C2-C3-C5-C6
14	BA	1238	CLA	C2-C3-C5-C6
14	HA	1205	CLA	C2-C3-C5-C6
14	bA	1205	CLA	C2-C3-C5-C6
14	BA	1205	CLA	C2-C3-C5-C6
13	aA	1011	CL0	C2-C3-C5-C6
19	bA	5004	LHG	C33-C34-C35-C36
19	BA	5004	LHG	C33-C34-C35-C36
19	HA	5004	LHG	C33-C34-C35-C36
14	bA	1223	CLA	C3-C5-C6-C7
14	BA	1223	CLA	C3-C5-C6-C7
14	HA	1223	CLA	C3-C5-C6-C7
21	AA	6001	LMT	C2-C3-C4-C5
21	GA	6001	LMT	C2-C3-C4-C5
21	aA	6001	LMT	C2-C3-C4-C5
14	bA	1022	CLA	C16-C17-C18-C19
14	bA	1211	CLA	C16-C17-C18-C19
14	HA	1215	CLA	C11-C12-C13-C14
14	BA	1238	CLA	C16-C17-C18-C20
14	0	1012	CLA	C16-C17-C18-C19
14	BA	1022	CLA	C16-C17-C18-C19
14	bA	1238	CLA	C16-C17-C18-C20

Continued on next page...

PRELIMINARY VALIDATION REPORT

Continued from previous page...

Mol	Chain	Res	Type	Atoms
14	HA	1211	CLA	C16-C17-C18-C19
14	BA	1215	CLA	C11-C12-C13-C14
14	BA	1211	CLA	C16-C17-C18-C19
14	bA	1215	CLA	C11-C12-C13-C14
19	AA	5001	LHG	C28-C29-C30-C31
19	GA	5001	LHG	C28-C29-C30-C31
19	aA	5001	LHG	C28-C29-C30-C31
14	GA	1103	CLA	O1D-CGD-O2D-CED
14	aA	1103	CLA	O1D-CGD-O2D-CED
14	LA	1501	CLA	C3A-C2A-CAA-CBA
14	bA	1022	CLA	C3A-C2A-CAA-CBA
14	GA	1103	CLA	C3A-C2A-CAA-CBA
14	aA	1102	CLA	C3A-C2A-CAA-CBA
14	AA	1135	CLA	C3A-C2A-CAA-CBA
14	BA	1212	CLA	C3A-C2A-CAA-CBA
14	AA	1138	CLA	C3A-C2A-CAA-CBA
14	bA	1210	CLA	C3A-C2A-CAA-CBA
14	aA	1135	CLA	C3A-C2A-CAA-CBA
14	HA	1209	CLA	C3A-C2A-CAA-CBA
14	HA	1233	CLA	C3A-C2A-CAA-CBA
14	GA	1102	CLA	C3A-C2A-CAA-CBA
14	aA	1125	CLA	C3A-C2A-CAA-CBA
14	BA	1220	CLA	C3A-C2A-CAA-CBA
14	0	1012	CLA	C3A-C2A-CAA-CBA
14	BA	1213	CLA	C3A-C2A-CAA-CBA
14	GA	1125	CLA	C3A-C2A-CAA-CBA
14	aA	1103	CLA	C3A-C2A-CAA-CBA
14	lA	1501	CLA	C3A-C2A-CAA-CBA
14	GA	1138	CLA	C3A-C2A-CAA-CBA
14	GA	1139	CLA	C3A-C2A-CAA-CBA
14	AA	1103	CLA	C3A-C2A-CAA-CBA
14	BA	1209	CLA	C3A-C2A-CAA-CBA
14	bA	1233	CLA	C3A-C2A-CAA-CBA
14	aA	1139	CLA	C3A-C2A-CAA-CBA
14	BA	1022	CLA	C3A-C2A-CAA-CBA
14	UA	1501	CLA	C3A-C2A-CAA-CBA
14	bA	1213	CLA	C3A-C2A-CAA-CBA
14	BA	1210	CLA	C3A-C2A-CAA-CBA
14	AA	1102	CLA	C3A-C2A-CAA-CBA
14	BA	1233	CLA	C3A-C2A-CAA-CBA
14	bA	1220	CLA	C3A-C2A-CAA-CBA
14	HA	1210	CLA	C3A-C2A-CAA-CBA

Continued on next page...

PRELIMINARY VALIDATION REPORT

Continued from previous page...

Mol	Chain	Res	Type	Atoms
14	bA	1227	CLA	C3A-C2A-CAA-CBA
14	HA	1213	CLA	C3A-C2A-CAA-CBA
14	GA	1013	CLA	C3A-C2A-CAA-CBA
14	HA	1220	CLA	C3A-C2A-CAA-CBA
14	BA	1227	CLA	C3A-C2A-CAA-CBA
14	bA	1209	CLA	C3A-C2A-CAA-CBA
14	AA	1125	CLA	C3A-C2A-CAA-CBA
14	aA	1013	CLA	C3A-C2A-CAA-CBA
14	HA	1212	CLA	C3A-C2A-CAA-CBA
14	aA	1138	CLA	C3A-C2A-CAA-CBA
14	AA	1139	CLA	C3A-C2A-CAA-CBA
14	bA	1212	CLA	C3A-C2A-CAA-CBA
14	HA	1227	CLA	C3A-C2A-CAA-CBA
14	AA	1013	CLA	C3A-C2A-CAA-CBA
14	GA	1135	CLA	C3A-C2A-CAA-CBA
21	GA	6001	LMT	O5'-C5'-C6'-O6'
21	aA	6001	LMT	O5'-C5'-C6'-O6'
14	AA	1140	CLA	C8-C10-C11-C12
14	GA	1140	CLA	C8-C10-C11-C12
14	AA	1103	CLA	O1D-CGD-O2D-CED
21	AA	6001	LMT	O5'-C5'-C6'-O6'
14	bA	1223	CLA	C13-C15-C16-C17
14	BA	1223	CLA	C13-C15-C16-C17
14	aA	1140	CLA	C8-C10-C11-C12
14	HA	1223	CLA	C13-C15-C16-C17
19	bA	5004	LHG	O9-C7-O7-C5
19	BA	5004	LHG	O9-C7-O7-C5
19	HA	5004	LHG	O9-C7-O7-C5
14	BA	1225	CLA	C16-C17-C18-C20
13	GA	1011	CL0	C16-C17-C18-C19
14	GA	1103	CLA	C11-C12-C13-C14
14	HA	1225	CLA	C16-C17-C18-C20
14	aA	1133	CLA	C11-C12-C13-C15
14	bA	1225	CLA	C16-C17-C18-C20
14	AA	1133	CLA	C11-C12-C13-C15
13	AA	1011	CL0	C16-C17-C18-C19
14	aA	1103	CLA	C11-C12-C13-C14
14	AA	1103	CLA	C11-C12-C13-C14
14	GA	1133	CLA	C11-C12-C13-C15
13	aA	1011	CL0	C16-C17-C18-C19
14	BA	1224	CLA	C10-C11-C12-C13
19	bA	5004	LHG	C8-C7-O7-C5

Continued on next page...

PRELIMINARY VALIDATION REPORT

Continued from previous page...

Mol	Chain	Res	Type	Atoms
19	BA	5004	LHG	C8-C7-O7-C5
19	HA	5004	LHG	C8-C7-O7-C5
20	GA	5005	LMG	C11-C10-O7-C8
20	AA	5005	LMG	C11-C10-O7-C8
20	aA	5005	LMG	C11-C10-O7-C8
19	AA	5001	LHG	O1-C1-C2-O2
19	GA	5001	LHG	O1-C1-C2-O2
19	aA	5001	LHG	O1-C1-C2-O2
14	AA	1138	CLA	C5-C6-C7-C8
14	HA	1224	CLA	C10-C11-C12-C13
14	GA	1138	CLA	C5-C6-C7-C8
14	bA	1224	CLA	C10-C11-C12-C13
14	aA	1138	CLA	C5-C6-C7-C8
14	LA	1501	CLA	C2-C3-C5-C6
13	AA	1011	CL0	C2-C3-C5-C6
14	lA	1501	CLA	C2-C3-C5-C6
14	bA	1238	CLA	C2-C3-C5-C6
14	UA	1501	CLA	C2-C3-C5-C6
14	HA	1238	CLA	C2-C3-C5-C6
14	bA	1211	CLA	C16-C17-C18-C20
14	BA	1238	CLA	C16-C17-C18-C19
14	bA	1238	CLA	C16-C17-C18-C19
14	HA	1211	CLA	C16-C17-C18-C20
14	HA	1238	CLA	C16-C17-C18-C19
14	BA	1211	CLA	C16-C17-C18-C20
20	LA	5007	LMG	O10-C28-O8-C9
20	UA	5007	LMG	O10-C28-O8-C9
20	lA	5007	LMG	O10-C28-O8-C9
18	aA	4003	BCR	C1-C6-C7-C8
18	aA	4003	BCR	C5-C6-C7-C8
18	aA	4003	BCR	C23-C24-C25-C26
18	HA	4016	BCR	C1-C6-C7-C8
18	HA	4016	BCR	C5-C6-C7-C8
18	HA	4016	BCR	C23-C24-C25-C26
18	BA	4016	BCR	C1-C6-C7-C8
18	BA	4016	BCR	C5-C6-C7-C8
18	BA	4016	BCR	C23-C24-C25-C26
18	IA	4020	BCR	C23-C24-C25-C26
18	MA	4021	BCR	C23-C24-C25-C26
18	AA	4003	BCR	C1-C6-C7-C8
18	AA	4003	BCR	C5-C6-C7-C8
18	AA	4003	BCR	C23-C24-C25-C26

Continued on next page...

PRELIMINARY VALIDATION REPORT

Continued from previous page...

Mol	Chain	Res	Type	Atoms
18	RA	4020	BCR	C23-C24-C25-C26
18	iA	4020	BCR	C23-C24-C25-C26
18	bA	4012	BCR	C5-C6-C7-C8
18	bA	4010	BCR	C23-C24-C25-C26
18	AA	4001	BCR	C1-C6-C7-C8
18	AA	4001	BCR	C5-C6-C7-C8
18	AA	4001	BCR	C23-C24-C25-C26
18	aA	4007	BCR	C23-C24-C25-C26
18	bA	4016	BCR	C1-C6-C7-C8
18	bA	4016	BCR	C5-C6-C7-C8
18	bA	4016	BCR	C23-C24-C25-C26
18	aA	4001	BCR	C1-C6-C7-C8
18	aA	4001	BCR	C5-C6-C7-C8
18	aA	4001	BCR	C23-C24-C25-C26
18	HA	4010	BCR	C23-C24-C25-C26
18	AA	4002	BCR	C1-C6-C7-C8
18	GA	4008	BCR	C5-C6-C7-C8
18	GA	4008	BCR	C23-C24-C25-C26
18	GA	4008	BCR	C23-C24-C25-C30
18	bA	4013	BCR	C1-C6-C7-C8
18	bA	4013	BCR	C5-C6-C7-C8
18	bA	4013	BCR	C23-C24-C25-C30
18	aA	4002	BCR	C1-C6-C7-C8
18	AA	4008	BCR	C5-C6-C7-C8
18	AA	4008	BCR	C23-C24-C25-C26
18	AA	4008	BCR	C23-C24-C25-C30
18	HA	4017	BCR	C23-C24-C25-C26
18	HA	4017	BCR	C23-C24-C25-C30
18	GA	4002	BCR	C1-C6-C7-C8
18	HA	4012	BCR	C5-C6-C7-C8
18	GA	4001	BCR	C1-C6-C7-C8
18	GA	4001	BCR	C5-C6-C7-C8
18	GA	4001	BCR	C23-C24-C25-C26
18	bA	4006	BCR	C23-C24-C25-C26
18	bA	4006	BCR	C23-C24-C25-C30
18	AA	4007	BCR	C23-C24-C25-C26
18	HA	4006	BCR	C23-C24-C25-C26
18	HA	4006	BCR	C23-C24-C25-C30
18	GA	4003	BCR	C1-C6-C7-C8
18	GA	4003	BCR	C5-C6-C7-C8
18	GA	4003	BCR	C23-C24-C25-C26
18	BA	4006	BCR	C23-C24-C25-C26

Continued on next page...

PRELIMINARY VALIDATION REPORT

Continued from previous page...

Mol	Chain	Res	Type	Atoms
18	BA	4006	BCR	C23-C24-C25-C30
18	mA	4021	BCR	C23-C24-C25-C26
18	aA	4008	BCR	C5-C6-C7-C8
18	aA	4008	BCR	C23-C24-C25-C26
18	aA	4008	BCR	C23-C24-C25-C30
18	bA	4017	BCR	C23-C24-C25-C26
18	bA	4017	BCR	C23-C24-C25-C30
18	BA	4017	BCR	C23-C24-C25-C26
18	BA	4017	BCR	C23-C24-C25-C30
18	BA	4010	BCR	C23-C24-C25-C26
18	BA	4013	BCR	C1-C6-C7-C8
18	BA	4013	BCR	C5-C6-C7-C8
18	BA	4013	BCR	C23-C24-C25-C30
18	HA	4013	BCR	C1-C6-C7-C8
18	HA	4013	BCR	C5-C6-C7-C8
18	HA	4013	BCR	C23-C24-C25-C30
18	GA	4007	BCR	C23-C24-C25-C26
18	VA	4021	BCR	C23-C24-C25-C26
20	GA	5005	LMG	C36-C37-C38-C39
20	AA	5005	LMG	C36-C37-C38-C39
20	aA	5005	LMG	C36-C37-C38-C39
14	BA	1225	CLA	C13-C15-C16-C17
14	HA	1225	CLA	C13-C15-C16-C17
14	bA	1225	CLA	C13-C15-C16-C17
14	aA	1126	CLA	C2C-C3C-CAC-CBC
14	aA	1125	CLA	CBA-CGA-O2A-C1
14	GA	1125	CLA	CBA-CGA-O2A-C1
14	AA	1125	CLA	CBA-CGA-O2A-C1
14	GA	1126	CLA	C2C-C3C-CAC-CBC
14	GA	1103	CLA	C2-C1-O2A-CGA
14	aA	1102	CLA	C2-C1-O2A-CGA
14	aA	1012	CLA	C2-C1-O2A-CGA
14	aA	1133	CLA	C2-C1-O2A-CGA
14	AA	1133	CLA	C2-C1-O2A-CGA
14	GA	1102	CLA	C2-C1-O2A-CGA
14	aA	1128	CLA	C2-C1-O2A-CGA
14	GA	1128	CLA	C2-C1-O2A-CGA
14	AA	1128	CLA	C2-C1-O2A-CGA
14	BA	1213	CLA	C2-C1-O2A-CGA
14	lA	1503	CLA	C2-C1-O2A-CGA
14	aA	1136	CLA	C2-C1-O2A-CGA
14	GA	1133	CLA	C2-C1-O2A-CGA

Continued on next page...

PRELIMINARY VALIDATION REPORT

Continued from previous page...

Mol	Chain	Res	Type	Atoms
14	AA	1136	CLA	C2-C1-O2A-CGA
14	bA	1213	CLA	C2-C1-O2A-CGA
14	AA	1102	CLA	C2-C1-O2A-CGA
14	LA	1503	CLA	C2-C1-O2A-CGA
14	GA	1136	CLA	C2-C1-O2A-CGA
14	HA	1213	CLA	C2-C1-O2A-CGA
14	AA	1012	CLA	C2-C1-O2A-CGA
14	GA	1012	CLA	C2-C1-O2A-CGA
14	UA	1503	CLA	C2-C1-O2A-CGA
14	BA	1204	CLA	C15-C16-C17-C18
14	bA	1204	CLA	C15-C16-C17-C18
14	HA	1204	CLA	C15-C16-C17-C18
14	aA	1136	CLA	C6-C7-C8-C9
14	AA	1136	CLA	C6-C7-C8-C9
14	GA	1136	CLA	C6-C7-C8-C9
14	HA	1215	CLA	C6-C7-C8-C10
14	aA	1012	CLA	C11-C10-C8-C7
14	HA	1224	CLA	C12-C13-C15-C16
14	BA	1215	CLA	C6-C7-C8-C10
14	bA	1224	CLA	C12-C13-C15-C16
14	BA	1224	CLA	C12-C13-C15-C16
14	AA	1012	CLA	C11-C10-C8-C7
14	GA	1012	CLA	C11-C10-C8-C7
14	bA	1215	CLA	C6-C7-C8-C10
14	BA	1238	CLA	O1A-CGA-O2A-C1
14	bA	1238	CLA	O1A-CGA-O2A-C1
14	HA	1238	CLA	O1A-CGA-O2A-C1
14	AA	1126	CLA	C2C-C3C-CAC-CBC
14	AA	1140	CLA	C10-C11-C12-C13
14	GA	1140	CLA	C10-C11-C12-C13
14	HA	1239	CLA	C5-C6-C7-C8
14	aA	1140	CLA	C10-C11-C12-C13
14	BA	1239	CLA	C5-C6-C7-C8
14	AA	1012	CLA	C13-C15-C16-C17
14	bA	1239	CLA	C5-C6-C7-C8
14	bA	1234	CLA	O1D-CGD-O2D-CED
14	HA	1234	CLA	O1D-CGD-O2D-CED
14	BA	1234	CLA	O1D-CGD-O2D-CED
14	aA	1126	CLA	CBA-CGA-O2A-C1
14	GA	1126	CLA	CBA-CGA-O2A-C1
14	AA	1126	CLA	CBA-CGA-O2A-C1
14	aA	1012	CLA	C13-C15-C16-C17

Continued on next page...

PRELIMINARY VALIDATION REPORT

Continued from previous page...

Mol	Chain	Res	Type	Atoms
14	aA	1126	CLA	C5-C6-C7-C8
14	GA	1126	CLA	C5-C6-C7-C8
14	AA	1126	CLA	C5-C6-C7-C8
14	GA	1012	CLA	C13-C15-C16-C17
14	BA	1217	CLA	C2A-CAA-CBA-CGA
14	BA	1204	CLA	C2A-CAA-CBA-CGA
14	bA	1204	CLA	C2A-CAA-CBA-CGA
14	bA	1217	CLA	C2A-CAA-CBA-CGA
14	HA	1217	CLA	C2A-CAA-CBA-CGA
14	HA	1204	CLA	C2A-CAA-CBA-CGA
18	GA	4008	BCR	C9-C10-C11-C12
18	aA	4008	BCR	C9-C10-C11-C12
14	BA	1220	CLA	O1D-CGD-O2D-CED
14	bA	1220	CLA	O1D-CGD-O2D-CED
14	HA	1220	CLA	O1D-CGD-O2D-CED
14	AA	1132	CLA	C10-C11-C12-C13
14	aA	1132	CLA	C10-C11-C12-C13
14	GA	1132	CLA	C10-C11-C12-C13
14	aA	1119	CLA	C4-C3-C5-C6
14	GA	1119	CLA	C4-C3-C5-C6
14	AA	1119	CLA	C4-C3-C5-C6
14	BA	1204	CLA	C8-C10-C11-C12
14	bA	1204	CLA	C8-C10-C11-C12
14	HA	1204	CLA	C8-C10-C11-C12
19	bA	5004	LHG	C13-C14-C15-C16
16	AA	2001	PQN	C18-C20-C21-C22
19	BA	5004	LHG	C13-C14-C15-C16
16	GA	2001	PQN	C18-C20-C21-C22
19	HA	5004	LHG	C13-C14-C15-C16
14	bA	1238	CLA	C10-C11-C12-C13
14	BA	1238	CLA	C10-C11-C12-C13
14	HA	1238	CLA	C10-C11-C12-C13
16	aA	2001	PQN	C18-C20-C21-C22
14	HA	1214	CLA	C2C-C3C-CAC-CBC
14	BA	1214	CLA	C2C-C3C-CAC-CBC
18	HA	4017	BCR	C18-C19-C20-C21
18	bA	4017	BCR	C18-C19-C20-C21
18	BA	4017	BCR	C18-C19-C20-C21
18	IA	4018	BCR	C14-C15-C16-C17
18	RA	4018	BCR	C14-C15-C16-C17
18	iA	4018	BCR	C14-C15-C16-C17
14	aA	1012	CLA	C11-C10-C8-C9

Continued on next page...

PRELIMINARY VALIDATION REPORT

Continued from previous page...

Mol	Chain	Res	Type	Atoms
14	bA	1210	CLA	C11-C10-C8-C9
14	HA	1229	CLA	C6-C7-C8-C9
14	AA	1127	CLA	C6-C7-C8-C9
14	HA	1224	CLA	C14-C13-C15-C16
14	HA	1205	CLA	C11-C12-C13-C14
14	bA	1023	CLA	C11-C10-C8-C9
14	bA	1205	CLA	C11-C12-C13-C14
14	GA	1127	CLA	C6-C7-C8-C9
14	GA	1131	CLA	C6-C7-C8-C9
14	bA	1229	CLA	C6-C7-C8-C9
14	BA	1205	CLA	C11-C12-C13-C14
14	BA	1210	CLA	C11-C10-C8-C9
14	bA	1224	CLA	C14-C13-C15-C16
14	AA	1131	CLA	C6-C7-C8-C9
14	HA	1023	CLA	C11-C10-C8-C9
14	HA	1210	CLA	C11-C10-C8-C9
14	BA	1224	CLA	C14-C13-C15-C16
14	AA	1012	CLA	C11-C10-C8-C9
14	BA	1229	CLA	C6-C7-C8-C9
14	GA	1012	CLA	C11-C10-C8-C9
14	aA	1131	CLA	C6-C7-C8-C9
14	aA	1127	CLA	C6-C7-C8-C9
14	BA	1023	CLA	C11-C10-C8-C9
14	UA	1502	CLA	C1A-C2A-CAA-CBA
14	GA	1103	CLA	C1A-C2A-CAA-CBA
14	AA	1118	CLA	C1A-C2A-CAA-CBA
14	HA	1215	CLA	C1A-C2A-CAA-CBA
14	BA	1219	CLA	C1A-C2A-CAA-CBA
14	AA	1135	CLA	C1A-C2A-CAA-CBA
14	GA	1137	CLA	C1A-C2A-CAA-CBA
14	BA	1212	CLA	C1A-C2A-CAA-CBA
14	BA	1217	CLA	C1A-C2A-CAA-CBA
14	AA	1122	CLA	C1A-C2A-CAA-CBA
14	BA	1204	CLA	C1A-C2A-CAA-CBA
14	aA	1107	CLA	C1A-C2A-CAA-CBA
14	AA	1132	CLA	C1A-C2A-CAA-CBA
14	AA	1106	CLA	C1A-C2A-CAA-CBA
14	HA	1219	CLA	C1A-C2A-CAA-CBA
14	bA	1204	CLA	C1A-C2A-CAA-CBA
14	aA	1135	CLA	C1A-C2A-CAA-CBA
14	HA	1229	CLA	C1A-C2A-CAA-CBA
14	HA	1209	CLA	C1A-C2A-CAA-CBA

Continued on next page...

PRELIMINARY VALIDATION REPORT

Continued from previous page...

Mol	Chain	Res	Type	Atoms
14	HA	1233	CLA	C1A-C2A-CAA-CBA
14	bA	1217	CLA	C1A-C2A-CAA-CBA
14	AA	1127	CLA	C1A-C2A-CAA-CBA
14	bA	1236	CLA	C1A-C2A-CAA-CBA
14	aA	1122	CLA	C1A-C2A-CAA-CBA
14	GA	1107	CLA	C1A-C2A-CAA-CBA
14	aA	1125	CLA	C1A-C2A-CAA-CBA
14	aA	1132	CLA	C1A-C2A-CAA-CBA
14	GA	1113	CLA	C1A-C2A-CAA-CBA
14	BA	1220	CLA	C1A-C2A-CAA-CBA
14	GA	1105	CLA	C1A-C2A-CAA-CBA
14	GA	1127	CLA	C1A-C2A-CAA-CBA
14	aA	1130	CLA	C1A-C2A-CAA-CBA
14	HA	1239	CLA	C1A-C2A-CAA-CBA
14	AA	1107	CLA	C1A-C2A-CAA-CBA
14	BA	1236	CLA	C1A-C2A-CAA-CBA
14	GA	1125	CLA	C1A-C2A-CAA-CBA
14	aA	1109	CLA	C1A-C2A-CAA-CBA
14	aA	1103	CLA	C1A-C2A-CAA-CBA
14	GA	1110	CLA	C1A-C2A-CAA-CBA
14	GA	1139	CLA	C1A-C2A-CAA-CBA
14	AA	1103	CLA	C1A-C2A-CAA-CBA
14	GA	1106	CLA	C1A-C2A-CAA-CBA
14	AA	1129	CLA	C1A-C2A-CAA-CBA
14	bA	1229	CLA	C1A-C2A-CAA-CBA
14	aA	1106	CLA	C1A-C2A-CAA-CBA
14	BA	1209	CLA	C1A-C2A-CAA-CBA
14	GA	1129	CLA	C1A-C2A-CAA-CBA
14	bA	1233	CLA	C1A-C2A-CAA-CBA
14	AA	1137	CLA	C1A-C2A-CAA-CBA
14	aA	1139	CLA	C1A-C2A-CAA-CBA
14	GA	1109	CLA	C1A-C2A-CAA-CBA
14	BA	1215	CLA	C1A-C2A-CAA-CBA
14	BA	1239	CLA	C1A-C2A-CAA-CBA
14	AA	1110	CLA	C1A-C2A-CAA-CBA
14	GA	1132	CLA	C1A-C2A-CAA-CBA
14	BA	1233	CLA	C1A-C2A-CAA-CBA
14	AA	1113	CLA	C1A-C2A-CAA-CBA
14	bA	1220	CLA	C1A-C2A-CAA-CBA
14	aA	1137	CLA	C1A-C2A-CAA-CBA
14	bA	1227	CLA	C1A-C2A-CAA-CBA
14	AA	1109	CLA	C1A-C2A-CAA-CBA

Continued on next page...

PRELIMINARY VALIDATION REPORT

Continued from previous page...

Mol	Chain	Res	Type	Atoms
14	lA	1502	CLA	C1A-C2A-CAA-CBA
14	HA	1217	CLA	C1A-C2A-CAA-CBA
14	BA	1229	CLA	C1A-C2A-CAA-CBA
14	HA	1220	CLA	C1A-C2A-CAA-CBA
14	GA	1118	CLA	C1A-C2A-CAA-CBA
14	GA	1130	CLA	C1A-C2A-CAA-CBA
14	aA	1129	CLA	C1A-C2A-CAA-CBA
14	aA	1118	CLA	C1A-C2A-CAA-CBA
14	BA	1227	CLA	C1A-C2A-CAA-CBA
14	bA	1209	CLA	C1A-C2A-CAA-CBA
14	AA	1105	CLA	C1A-C2A-CAA-CBA
14	bA	1219	CLA	C1A-C2A-CAA-CBA
14	aA	1105	CLA	C1A-C2A-CAA-CBA
14	AA	1125	CLA	C1A-C2A-CAA-CBA
14	AA	1130	CLA	C1A-C2A-CAA-CBA
14	GA	1122	CLA	C1A-C2A-CAA-CBA
14	HA	1212	CLA	C1A-C2A-CAA-CBA
14	HA	1236	CLA	C1A-C2A-CAA-CBA
14	AA	1139	CLA	C1A-C2A-CAA-CBA
14	HA	1204	CLA	C1A-C2A-CAA-CBA
14	aA	1127	CLA	C1A-C2A-CAA-CBA
14	bA	1212	CLA	C1A-C2A-CAA-CBA
14	HA	1227	CLA	C1A-C2A-CAA-CBA
14	bA	1215	CLA	C1A-C2A-CAA-CBA
14	aA	1110	CLA	C1A-C2A-CAA-CBA
14	bA	1239	CLA	C1A-C2A-CAA-CBA
14	GA	1135	CLA	C1A-C2A-CAA-CBA
14	LA	1502	CLA	C1A-C2A-CAA-CBA
14	aA	1113	CLA	C1A-C2A-CAA-CBA
14	HA	1208	CLA	C15-C16-C17-C18
14	bA	1214	CLA	C2C-C3C-CAC-CBC
14	BA	1208	CLA	C15-C16-C17-C18
14	bA	1208	CLA	C15-C16-C17-C18
14	lA	1503	CLA	C2A-CAA-CBA-CGA
14	LA	1503	CLA	C2A-CAA-CBA-CGA
14	UA	1503	CLA	C2A-CAA-CBA-CGA
19	bA	5004	LHG	C3-O3-P-O6
19	BA	5004	LHG	C3-O3-P-O6
19	HA	5004	LHG	C3-O3-P-O6
14	aA	1125	CLA	O1A-CGA-O2A-C1
14	GA	1125	CLA	O1A-CGA-O2A-C1
14	AA	1125	CLA	O1A-CGA-O2A-C1

Continued on next page...

PRELIMINARY VALIDATION REPORT

Continued from previous page...

Mol	Chain	Res	Type	Atoms
14	aA	1119	CLA	C2-C3-C5-C6
14	GA	1119	CLA	C2-C3-C5-C6
14	AA	1119	CLA	C2-C3-C5-C6
21	GA	6002	LMT	O5B-C5B-C6B-O6B
21	aA	6002	LMT	O5B-C5B-C6B-O6B
14	aA	1102	CLA	C8-C10-C11-C12
16	AA	2001	PQN	C25-C26-C27-C28
14	GA	1102	CLA	C8-C10-C11-C12
14	AA	1102	CLA	C8-C10-C11-C12
16	aA	2001	PQN	C25-C26-C27-C28
18	HA	4016	BCR	C15-C16-C17-C18
18	BA	4016	BCR	C15-C16-C17-C18
18	bA	4016	BCR	C15-C16-C17-C18
14	aA	1125	CLA	O1D-CGD-O2D-CED
14	AA	1125	CLA	O1D-CGD-O2D-CED
21	AA	6002	LMT	O5B-C5B-C6B-O6B
14	UA	1502	CLA	C3-C5-C6-C7
14	lA	1502	CLA	C3-C5-C6-C7
14	LA	1502	CLA	C3-C5-C6-C7
16	GA	2001	PQN	C25-C26-C27-C28
16	AA	2001	PQN	C15-C16-C17-C18
16	GA	2001	PQN	C15-C16-C17-C18
16	aA	2001	PQN	C15-C16-C17-C18
20	LA	5007	LMG	O6-C5-C6-O5
20	UA	5007	LMG	O6-C5-C6-O5
20	lA	5007	LMG	O6-C5-C6-O5
14	aA	1126	CLA	O1A-CGA-O2A-C1
14	GA	1126	CLA	O1A-CGA-O2A-C1
14	AA	1126	CLA	O1A-CGA-O2A-C1
14	GA	1125	CLA	O1D-CGD-O2D-CED
19	AA	5001	LHG	C25-C26-C27-C28
19	GA	5001	LHG	C25-C26-C27-C28
19	aA	5001	LHG	C25-C26-C27-C28
20	bA	5002	LMG	C7-C8-C9-O8
20	BA	5002	LMG	C7-C8-C9-O8
20	HA	5002	LMG	C7-C8-C9-O8
14	aA	1125	CLA	C5-C6-C7-C8
14	GA	1125	CLA	C5-C6-C7-C8
14	aA	1140	CLA	C15-C16-C17-C18
14	AA	1125	CLA	C5-C6-C7-C8
20	GA	5005	LMG	O6-C5-C6-O5
20	AA	5005	LMG	O6-C5-C6-O5

Continued on next page...

PRELIMINARY VALIDATION REPORT

Continued from previous page...

Mol	Chain	Res	Type	Atoms
20	aA	5005	LMG	O6-C5-C6-O5
14	AA	1140	CLA	C15-C16-C17-C18
14	GA	1140	CLA	C15-C16-C17-C18
20	bA	5002	LMG	O6-C5-C6-O5
20	BA	5002	LMG	O6-C5-C6-O5
20	HA	5002	LMG	O6-C5-C6-O5
20	RA	5006	LMG	O6-C5-C6-O5
20	iA	5006	LMG	O6-C5-C6-O5
20	IA	5006	LMG	O6-C5-C6-O5
20	GA	5005	LMG	C30-C31-C32-C33
20	AA	5005	LMG	C30-C31-C32-C33
20	aA	5005	LMG	C30-C31-C32-C33
19	bA	5004	LHG	O1-C1-C2-O2
19	BA	5004	LHG	O1-C1-C2-O2
19	HA	5004	LHG	O1-C1-C2-O2
14	AA	1140	CLA	CBA-CGA-O2A-C1
14	GA	1140	CLA	CBA-CGA-O2A-C1
14	aA	1140	CLA	CBA-CGA-O2A-C1
14	BA	1220	CLA	CAA-CBA-CGA-O2A
14	bA	1220	CLA	CAA-CBA-CGA-O2A
14	HA	1220	CLA	CAA-CBA-CGA-O2A
14	BA	1220	CLA	CBA-CGA-O2A-C1
14	bA	1220	CLA	CBA-CGA-O2A-C1
14	HA	1220	CLA	CBA-CGA-O2A-C1
14	BA	1225	CLA	C4-C3-C5-C6
14	HA	1225	CLA	C4-C3-C5-C6
14	bA	1225	CLA	C4-C3-C5-C6
20	bA	5002	LMG	O7-C8-C9-O8
20	BA	5002	LMG	O7-C8-C9-O8
20	HA	5002	LMG	O7-C8-C9-O8
14	HA	1205	CLA	C2-C1-O2A-CGA
14	bA	1205	CLA	C2-C1-O2A-CGA
14	HA	1202	CLA	C2-C1-O2A-CGA
14	aA	1103	CLA	C2-C1-O2A-CGA
14	AA	1103	CLA	C2-C1-O2A-CGA
14	BA	1205	CLA	C2-C1-O2A-CGA
14	bA	1202	CLA	C2-C1-O2A-CGA
14	BA	1202	CLA	C2-C1-O2A-CGA
14	UA	1502	CLA	C8-C10-C11-C12
16	bA	2002	PQN	C15-C16-C17-C18
16	BA	2002	PQN	C15-C16-C17-C18
14	lA	1502	CLA	C8-C10-C11-C12

Continued on next page...

PRELIMINARY VALIDATION REPORT

Continued from previous page...

Mol	Chain	Res	Type	Atoms
16	HA	2002	PQN	C15-C16-C17-C18
14	LA	1502	CLA	C8-C10-C11-C12
14	bA	1211	CLA	C12-C13-C15-C16
14	bA	1221	CLA	C11-C10-C8-C7
14	AA	1140	CLA	C6-C7-C8-C10
14	BA	1221	CLA	C11-C10-C8-C7
14	bA	1210	CLA	C11-C10-C8-C7
14	GA	1140	CLA	C6-C7-C8-C10
14	aA	1117	CLA	C6-C7-C8-C10
14	bA	1023	CLA	C11-C10-C8-C7
14	HA	1021	CLA	C12-C13-C15-C16
14	HA	1207	CLA	C2A-CAA-CBA-CGA
14	lA	1503	CLA	C11-C10-C8-C7
14	aA	1140	CLA	C6-C7-C8-C10
14	GA	1117	CLA	C6-C7-C8-C10
14	BA	1021	CLA	C12-C13-C15-C16
14	GA	1131	CLA	C6-C7-C8-C10
14	HA	1211	CLA	C12-C13-C15-C16
14	BA	1210	CLA	C11-C10-C8-C7
14	HA	1221	CLA	C11-C10-C8-C7
14	AA	1131	CLA	C6-C7-C8-C10
14	bA	1207	CLA	C2A-CAA-CBA-CGA
14	LA	1503	CLA	C11-C10-C8-C7
14	HA	1023	CLA	C11-C10-C8-C7
14	HA	1210	CLA	C11-C10-C8-C7
14	aA	1131	CLA	C6-C7-C8-C10
14	UA	1503	CLA	C11-C10-C8-C7
14	BA	1211	CLA	C12-C13-C15-C16
14	BA	1023	CLA	C11-C10-C8-C7
14	AA	1117	CLA	C6-C7-C8-C10
14	bA	1021	CLA	C12-C13-C15-C16
14	BA	1207	CLA	C2A-CAA-CBA-CGA
14	aA	1131	CLA	C5-C6-C7-C8
14	GA	1131	CLA	C5-C6-C7-C8
14	bA	1022	CLA	C14-C13-C15-C16
14	bA	1221	CLA	C11-C10-C8-C9
16	bA	2002	PQN	C19-C18-C20-C21
16	bA	2002	PQN	C21-C22-C23-C24
14	BA	1221	CLA	C11-C10-C8-C9
14	AA	1127	CLA	C14-C13-C15-C16
14	aA	1117	CLA	C6-C7-C8-C9
14	GA	1127	CLA	C14-C13-C15-C16

Continued on next page...

PRELIMINARY VALIDATION REPORT

Continued from previous page...

Mol	Chain	Res	Type	Atoms
16	BA	2002	PQN	C19-C18-C20-C21
16	BA	2002	PQN	C21-C22-C23-C24
14	HA	1239	CLA	C11-C10-C8-C9
14	0	1012	CLA	C14-C13-C15-C16
14	GA	1117	CLA	C6-C7-C8-C9
14	BA	1022	CLA	C14-C13-C15-C16
14	BA	1239	CLA	C11-C10-C8-C9
14	HA	1221	CLA	C11-C10-C8-C9
14	aA	1127	CLA	C14-C13-C15-C16
14	AA	1117	CLA	C6-C7-C8-C9
14	bA	1239	CLA	C11-C10-C8-C9
16	HA	2002	PQN	C19-C18-C20-C21
16	HA	2002	PQN	C21-C22-C23-C24
14	AA	1131	CLA	C5-C6-C7-C8
14	aA	1119	CLA	C2A-CAA-CBA-CGA
14	HA	1201	CLA	C2A-CAA-CBA-CGA
14	bA	1201	CLA	C2A-CAA-CBA-CGA
14	BA	1201	CLA	C2A-CAA-CBA-CGA
14	GA	1119	CLA	C2A-CAA-CBA-CGA
14	AA	1119	CLA	C2A-CAA-CBA-CGA
18	IA	4018	BCR	C17-C18-C19-C20
18	RA	4018	BCR	C17-C18-C19-C20
18	iA	4018	BCR	C17-C18-C19-C20
14	bA	1211	CLA	C13-C15-C16-C17
14	HA	1211	CLA	C13-C15-C16-C17
14	BA	1211	CLA	C13-C15-C16-C17
14	BA	1238	CLA	C3-C5-C6-C7
21	GA	6001	LMT	C3-C4-C5-C6
14	aA	1133	CLA	C8-C10-C11-C12
14	AA	1133	CLA	C8-C10-C11-C12
21	AA	6001	LMT	C3-C4-C5-C6
14	GA	1133	CLA	C8-C10-C11-C12
21	aA	6001	LMT	C3-C4-C5-C6
14	GA	1140	CLA	O1A-CGA-O2A-C1
14	aA	1140	CLA	O1A-CGA-O2A-C1
14	aA	1126	CLA	C11-C12-C13-C15
14	GA	1126	CLA	C11-C12-C13-C15
14	AA	1126	CLA	C11-C12-C13-C15
14	AA	1140	CLA	O1A-CGA-O2A-C1
14	bA	1238	CLA	C3-C5-C6-C7
14	HA	1238	CLA	C3-C5-C6-C7
14	lA	1503	CLA	C10-C11-C12-C13

Continued on next page...

PRELIMINARY VALIDATION REPORT

Continued from previous page...

Mol	Chain	Res	Type	Atoms
14	AA	1132	CLA	C13-C15-C16-C17
14	GA	1132	CLA	C13-C15-C16-C17
14	LA	1503	CLA	C10-C11-C12-C13
14	UA	1503	CLA	C10-C11-C12-C13
14	aA	1126	CLA	C11-C12-C13-C14
14	GA	1126	CLA	C11-C12-C13-C14
14	AA	1126	CLA	C11-C12-C13-C14
14	aA	1132	CLA	C13-C15-C16-C17
14	aA	1126	CLA	C4C-C3C-CAC-CBC
14	GA	1126	CLA	C4C-C3C-CAC-CBC
14	AA	1126	CLA	C4C-C3C-CAC-CBC
14	AA	1140	CLA	C3A-C2A-CAA-CBA
14	GA	1140	CLA	C3A-C2A-CAA-CBA
14	HA	1205	CLA	C3A-C2A-CAA-CBA
14	bA	1205	CLA	C3A-C2A-CAA-CBA
14	aA	1140	CLA	C3A-C2A-CAA-CBA
14	HA	1203	CLA	C3A-C2A-CAA-CBA
14	BA	1205	CLA	C3A-C2A-CAA-CBA
14	bA	1203	CLA	C3A-C2A-CAA-CBA
14	BA	1203	CLA	C3A-C2A-CAA-CBA
14	bA	1023	CLA	C8-C10-C11-C12
14	HA	1023	CLA	C8-C10-C11-C12
14	BA	1023	CLA	C8-C10-C11-C12
14	GA	1103	CLA	CBA-CGA-O2A-C1
14	aA	1103	CLA	CBA-CGA-O2A-C1
14	GA	1013	CLA	CBA-CGA-O2A-C1
14	aA	1013	CLA	CBA-CGA-O2A-C1
14	AA	1013	CLA	CBA-CGA-O2A-C1
21	AA	6001	LMT	C2-C1-O1'-C1'
21	GA	6001	LMT	C2-C1-O1'-C1'
21	aA	6001	LMT	C2-C1-O1'-C1'
14	BA	1225	CLA	C2-C3-C5-C6
14	HA	1225	CLA	C2-C3-C5-C6
14	bA	1225	CLA	C2-C3-C5-C6
14	GA	1116	CLA	C3-C5-C6-C7
14	AA	1116	CLA	C3-C5-C6-C7
14	aA	1116	CLA	C3-C5-C6-C7
14	bA	1023	CLA	CBA-CGA-O2A-C1
14	AA	1103	CLA	CBA-CGA-O2A-C1
14	HA	1023	CLA	CBA-CGA-O2A-C1
14	BA	1023	CLA	CBA-CGA-O2A-C1
14	bA	1211	CLA	C10-C11-C12-C13

Continued on next page...

PRELIMINARY VALIDATION REPORT

Continued from previous page...

Mol	Chain	Res	Type	Atoms
13	GA	1011	CL0	C5-C6-C7-C8
13	AA	1011	CL0	C5-C6-C7-C8
14	aA	1126	CLA	C8-C10-C11-C12
14	HA	1211	CLA	C10-C11-C12-C13
14	GA	1126	CLA	C8-C10-C11-C12
14	AA	1126	CLA	C8-C10-C11-C12
14	BA	1211	CLA	C10-C11-C12-C13
13	aA	1011	CL0	C5-C6-C7-C8
14	BA	1220	CLA	O1A-CGA-O2A-C1
14	bA	1220	CLA	O1A-CGA-O2A-C1
14	HA	1220	CLA	O1A-CGA-O2A-C1
20	GA	5005	LMG	O7-C8-C9-O8
20	AA	5005	LMG	O7-C8-C9-O8
20	aA	5005	LMG	O7-C8-C9-O8
14	bA	1222	CLA	C10-C11-C12-C13
14	BA	1204	CLA	C4-C3-C5-C6
14	bA	1204	CLA	C4-C3-C5-C6
14	HA	1204	CLA	C4-C3-C5-C6
14	GA	1111	CLA	C10-C11-C12-C13
14	HA	1222	CLA	C10-C11-C12-C13
14	BA	1204	CLA	C2-C3-C5-C6
14	bA	1204	CLA	C2-C3-C5-C6
14	HA	1204	CLA	C2-C3-C5-C6
14	BA	1208	CLA	C16-C17-C18-C20
14	bA	1208	CLA	C16-C17-C18-C20
14	AA	1111	CLA	C10-C11-C12-C13
14	aA	1111	CLA	C10-C11-C12-C13
14	BA	1222	CLA	C10-C11-C12-C13
14	GA	1111	CLA	C8-C10-C11-C12
14	bA	1211	CLA	C14-C13-C15-C16
18	HA	4016	BCR	C23-C24-C25-C30
14	HA	1208	CLA	C14-C13-C15-C16
18	BA	4016	BCR	C23-C24-C25-C30
14	bA	1221	CLA	C6-C7-C8-C9
18	BA	4012	BCR	C1-C6-C7-C8
18	BA	4012	BCR	C5-C6-C7-C8
14	BA	1221	CLA	C6-C7-C8-C9
18	bA	4012	BCR	C1-C6-C7-C8
14	aA	1117	CLA	C14-C13-C15-C16
18	bA	4010	BCR	C23-C24-C25-C30
18	AA	4001	BCR	C23-C24-C25-C30
14	BA	1208	CLA	C14-C13-C15-C16

Continued on next page...

PRELIMINARY VALIDATION REPORT

Continued from previous page...

Mol	Chain	Res	Type	Atoms
18	bA	4016	BCR	C23-C24-C25-C30
18	aA	4001	BCR	C23-C24-C25-C30
18	HA	4010	BCR	C23-C24-C25-C30
14	GA	1117	CLA	C14-C13-C15-C16
14	HA	1203	CLA	C11-C10-C8-C9
14	aA	1126	CLA	C11-C10-C8-C9
14	HA	1211	CLA	C14-C13-C15-C16
14	GA	1126	CLA	C11-C10-C8-C9
18	HA	4017	BCR	C1-C6-C7-C8
14	HA	1221	CLA	C6-C7-C8-C9
14	AA	1126	CLA	C11-C10-C8-C9
18	HA	4012	BCR	C1-C6-C7-C8
14	bA	1208	CLA	C14-C13-C15-C16
18	GA	4001	BCR	C23-C24-C25-C30
14	bA	1203	CLA	C11-C10-C8-C9
18	bA	4017	BCR	C1-C6-C7-C8
18	BA	4017	BCR	C1-C6-C7-C8
18	BA	4010	BCR	C23-C24-C25-C30
14	BA	1211	CLA	C14-C13-C15-C16
14	AA	1117	CLA	C14-C13-C15-C16
14	BA	1203	CLA	C11-C10-C8-C9
14	HA	1229	CLA	C10-C11-C12-C13
14	bA	1229	CLA	C10-C11-C12-C13
14	HA	1208	CLA	C16-C17-C18-C20
14	bA	1214	CLA	C4C-C3C-CAC-CBC
14	HA	1214	CLA	C4C-C3C-CAC-CBC
14	BA	1229	CLA	C10-C11-C12-C13
14	BA	1214	CLA	C4C-C3C-CAC-CBC
19	AA	5001	LHG	O8-C23-C24-C25
19	GA	5001	LHG	O8-C23-C24-C25
19	aA	5001	LHG	O8-C23-C24-C25
18	HA	4005	BCR	C17-C18-C19-C20
18	bA	4005	BCR	C17-C18-C19-C20
18	aA	4007	BCR	C17-C18-C19-C20
18	BA	4005	BCR	C17-C18-C19-C20
18	LA	4022	BCR	C11-C12-C13-C14
18	AA	4007	BCR	C17-C18-C19-C20
18	lA	4022	BCR	C11-C12-C13-C14
18	UA	4022	BCR	C11-C12-C13-C14
18	GA	4007	BCR	C17-C18-C19-C20
14	AA	1111	CLA	C8-C10-C11-C12
14	aA	1111	CLA	C8-C10-C11-C12

Continued on next page...

PRELIMINARY VALIDATION REPORT

Continued from previous page...

Mol	Chain	Res	Type	Atoms
14	bA	1221	CLA	C2-C1-O2A-CGA
14	BA	1221	CLA	C2-C1-O2A-CGA
14	HA	1221	CLA	C2-C1-O2A-CGA
14	bA	1022	CLA	C12-C13-C15-C16
14	bA	1214	CLA	C11-C10-C8-C7
16	bA	2002	PQN	C21-C22-C23-C25
16	AA	2001	PQN	C17-C18-C20-C21
14	BA	1204	CLA	C6-C7-C8-C10
14	bA	1226	CLA	C6-C7-C8-C10
14	bA	1204	CLA	C6-C7-C8-C10
16	GA	2001	PQN	C17-C18-C20-C21
14	AA	1127	CLA	C12-C13-C15-C16
14	aA	1117	CLA	C12-C13-C15-C16
14	aA	1104	CLA	C6-C7-C8-C10
14	GA	1127	CLA	C12-C13-C15-C16
16	BA	2002	PQN	C21-C22-C23-C25
14	HA	1239	CLA	C11-C10-C8-C7
14	HA	1214	CLA	C11-C10-C8-C7
14	0	1012	CLA	C12-C13-C15-C16
14	GA	1117	CLA	C12-C13-C15-C16
14	HA	1203	CLA	C6-C7-C8-C10
14	BA	1022	CLA	C12-C13-C15-C16
14	aA	1126	CLA	C11-C10-C8-C7
14	GA	1126	CLA	C6-C7-C8-C10
14	GA	1126	CLA	C11-C10-C8-C7
14	BA	1239	CLA	C11-C10-C8-C7
14	AA	1126	CLA	C11-C10-C8-C7
14	AA	1104	CLA	C6-C7-C8-C10
14	bA	1203	CLA	C6-C7-C8-C10
14	BA	1226	CLA	C6-C7-C8-C10
14	GA	1104	CLA	C6-C7-C8-C10
14	HA	1226	CLA	C6-C7-C8-C10
16	aA	2001	PQN	C17-C18-C20-C21
14	HA	1204	CLA	C6-C7-C8-C10
14	BA	1214	CLA	C11-C10-C8-C7
14	aA	1127	CLA	C12-C13-C15-C16
14	AA	1117	CLA	C12-C13-C15-C16
14	BA	1203	CLA	C6-C7-C8-C10
14	bA	1239	CLA	C11-C10-C8-C7
16	HA	2002	PQN	C21-C22-C23-C25
19	AA	5001	LHG	O6-C4-C5-C6
19	GA	5001	LHG	O6-C4-C5-C6

Continued on next page...

PRELIMINARY VALIDATION REPORT

Continued from previous page...

Mol	Chain	Res	Type	Atoms
19	aA	5001	LHG	O6-C4-C5-C6
14	bA	1223	CLA	C5-C6-C7-C8
14	BA	1223	CLA	C5-C6-C7-C8
14	HA	1223	CLA	C5-C6-C7-C8
20	GA	5005	LMG	C12-C13-C14-C15
20	AA	5005	LMG	C12-C13-C14-C15
20	aA	5005	LMG	C12-C13-C14-C15
18	bA	4014	BCR	C19-C20-C21-C22
18	HA	4014	BCR	C19-C20-C21-C22
18	BA	4014	BCR	C19-C20-C21-C22
14	HA	1216	CLA	C5-C6-C7-C8
14	bA	1216	CLA	C5-C6-C7-C8
14	BA	1216	CLA	C5-C6-C7-C8
14	AA	1118	CLA	CAD-CBD-CGD-O2D
14	bA	1221	CLA	CAD-CBD-CGD-O2D
14	AA	1135	CLA	CAD-CBD-CGD-O2D
14	bA	1226	CLA	CAD-CBD-CGD-O2D
14	AA	1138	CLA	CAD-CBD-CGD-O2D
14	BA	1221	CLA	CAD-CBD-CGD-O2D
14	aA	1135	CLA	CAD-CBD-CGD-O2D
14	GA	1138	CLA	CAD-CBD-CGD-O2D
14	HA	1221	CLA	CAD-CBD-CGD-O2D
14	BA	1226	CLA	CAD-CBD-CGD-O2D
14	GA	1118	CLA	CAD-CBD-CGD-O2D
14	aA	1118	CLA	CAD-CBD-CGD-O2D
14	HA	1226	CLA	CAD-CBD-CGD-O2D
14	aA	1138	CLA	CAD-CBD-CGD-O2D
14	GA	1135	CLA	CAD-CBD-CGD-O2D
20	bA	5002	LMG	C19-C20-C21-C22
20	BA	5002	LMG	C19-C20-C21-C22
20	HA	5002	LMG	C19-C20-C21-C22
13	GA	1011	CL0	C10-C11-C12-C13
14	BA	1235	CLA	C8-C10-C11-C12
13	AA	1011	CL0	C10-C11-C12-C13
14	bA	1235	CLA	C8-C10-C11-C12
14	HA	1235	CLA	C8-C10-C11-C12
13	aA	1011	CL0	C10-C11-C12-C13
19	AA	5001	LHG	O6-C4-C5-O7
19	GA	5001	LHG	O6-C4-C5-O7
19	aA	5001	LHG	O6-C4-C5-O7
14	aA	1122	CLA	CBD-CGD-O2D-CED
14	GA	1122	CLA	CBD-CGD-O2D-CED

Continued on next page...

PRELIMINARY VALIDATION REPORT

Continued from previous page...

Mol	Chain	Res	Type	Atoms
20	RA	5006	LMG	O7-C10-C11-C12
20	iA	5006	LMG	O7-C10-C11-C12
20	IA	5006	LMG	O7-C10-C11-C12
14	bA	1023	CLA	O1A-CGA-O2A-C1
14	HA	1023	CLA	O1A-CGA-O2A-C1
14	BA	1023	CLA	O1A-CGA-O2A-C1
14	bA	1211	CLA	C2A-CAA-CBA-CGA
14	HA	1211	CLA	C2A-CAA-CBA-CGA
14	BA	1211	CLA	C2A-CAA-CBA-CGA
14	AA	1122	CLA	CBD-CGD-O2D-CED
14	bA	1226	CLA	CBD-CGD-O2D-CED
14	BA	1226	CLA	CBD-CGD-O2D-CED
14	HA	1226	CLA	CBD-CGD-O2D-CED
14	GA	1103	CLA	O1A-CGA-O2A-C1
14	aA	1103	CLA	O1A-CGA-O2A-C1
14	AA	1103	CLA	O1A-CGA-O2A-C1
14	GA	1103	CLA	CHA-CBD-CGD-O1D
14	GA	1103	CLA	CHA-CBD-CGD-O2D
14	GA	1111	CLA	CHA-CBD-CGD-O1D
14	GA	1137	CLA	CHA-CBD-CGD-O1D
14	GA	1137	CLA	CHA-CBD-CGD-O2D
14	aA	1133	CLA	CHA-CBD-CGD-O1D
14	aA	1133	CLA	CHA-CBD-CGD-O2D
14	AA	1132	CLA	CHA-CBD-CGD-O1D
14	AA	1132	CLA	CHA-CBD-CGD-O2D
14	AA	1133	CLA	CHA-CBD-CGD-O1D
14	AA	1133	CLA	CHA-CBD-CGD-O2D
14	HA	1222	CLA	CHA-CBD-CGD-O1D
14	HA	1222	CLA	CHA-CBD-CGD-O2D
14	HA	1205	CLA	CHA-CBD-CGD-O1D
14	aA	1132	CLA	CHA-CBD-CGD-O1D
14	aA	1132	CLA	CHA-CBD-CGD-O2D
14	bA	1205	CLA	CHA-CBD-CGD-O1D
14	HA	1207	CLA	CHA-CBD-CGD-O1D
14	HA	1207	CLA	CHA-CBD-CGD-O2D
14	HA	1201	CLA	CHA-CBD-CGD-O1D
14	HA	1201	CLA	CHA-CBD-CGD-O2D
14	aA	1103	CLA	CHA-CBD-CGD-O1D
14	aA	1103	CLA	CHA-CBD-CGD-O2D
14	aA	1136	CLA	CHA-CBD-CGD-O1D
14	aA	1136	CLA	CHA-CBD-CGD-O2D
14	AA	1103	CLA	CHA-CBD-CGD-O1D

Continued on next page...

PRELIMINARY VALIDATION REPORT

Continued from previous page...

Mol	Chain	Res	Type	Atoms
14	AA	1103	CLA	CHA-CBD-CGD-O2D
14	AA	1137	CLA	CHA-CBD-CGD-O1D
14	AA	1137	CLA	CHA-CBD-CGD-O2D
14	GA	1133	CLA	CHA-CBD-CGD-O1D
14	GA	1133	CLA	CHA-CBD-CGD-O2D
14	AA	1111	CLA	CHA-CBD-CGD-O1D
14	AA	1136	CLA	CHA-CBD-CGD-O1D
14	AA	1136	CLA	CHA-CBD-CGD-O2D
14	BA	1205	CLA	CHA-CBD-CGD-O1D
14	GA	1132	CLA	CHA-CBD-CGD-O1D
14	GA	1132	CLA	CHA-CBD-CGD-O2D
14	bA	1207	CLA	CHA-CBD-CGD-O1D
14	bA	1207	CLA	CHA-CBD-CGD-O2D
14	aA	1137	CLA	CHA-CBD-CGD-O1D
14	aA	1137	CLA	CHA-CBD-CGD-O2D
14	GA	1136	CLA	CHA-CBD-CGD-O1D
14	GA	1136	CLA	CHA-CBD-CGD-O2D
14	bA	1222	CLA	CHA-CBD-CGD-O1D
14	bA	1222	CLA	CHA-CBD-CGD-O2D
14	bA	1201	CLA	CHA-CBD-CGD-O1D
14	bA	1201	CLA	CHA-CBD-CGD-O2D
14	BA	1201	CLA	CHA-CBD-CGD-O1D
14	BA	1201	CLA	CHA-CBD-CGD-O2D
14	aA	1111	CLA	CHA-CBD-CGD-O1D
14	BA	1222	CLA	CHA-CBD-CGD-O1D
14	BA	1222	CLA	CHA-CBD-CGD-O2D
14	BA	1207	CLA	CHA-CBD-CGD-O1D
14	BA	1207	CLA	CHA-CBD-CGD-O2D
14	AA	1118	CLA	C6-C7-C8-C9
14	AA	1118	CLA	C6-C7-C8-C10
14	GA	1118	CLA	C6-C7-C8-C9
14	GA	1118	CLA	C6-C7-C8-C10
14	aA	1118	CLA	C6-C7-C8-C9
14	aA	1118	CLA	C6-C7-C8-C10
14	aA	1117	CLA	C5-C6-C7-C8
14	GA	1117	CLA	C5-C6-C7-C8
14	AA	1117	CLA	C5-C6-C7-C8
14	aA	1125	CLA	C6-C7-C8-C9
14	GA	1125	CLA	C6-C7-C8-C9
14	GA	1013	CLA	C11-C10-C8-C9
14	AA	1125	CLA	C6-C7-C8-C9
14	aA	1013	CLA	C11-C10-C8-C9

Continued on next page...

PRELIMINARY VALIDATION REPORT

Continued from previous page...

Mol	Chain	Res	Type	Atoms
14	AA	1013	CLA	C11-C10-C8-C9
14	bA	1022	CLA	C1A-C2A-CAA-CBA
14	aA	1124	CLA	C1A-C2A-CAA-CBA
14	AA	1124	CLA	C1A-C2A-CAA-CBA
14	0	1012	CLA	C1A-C2A-CAA-CBA
14	BA	1022	CLA	C1A-C2A-CAA-CBA
14	GA	1124	CLA	C1A-C2A-CAA-CBA
18	IA	4020	BCR	C37-C22-C23-C24
18	bA	4014	BCR	C7-C8-C9-C34
18	RA	4020	BCR	C37-C22-C23-C24
18	iA	4020	BCR	C37-C22-C23-C24
18	HA	4014	BCR	C7-C8-C9-C34
18	BA	4014	BCR	C7-C8-C9-C34
14	HA	1222	CLA	C3-C5-C6-C7
14	bA	1222	CLA	C3-C5-C6-C7
14	BA	1222	CLA	C3-C5-C6-C7
19	BA	5004	LHG	C11-C10-C9-C8
19	HA	5004	LHG	C11-C10-C9-C8
19	bA	5004	LHG	C2-C3-O3-P
19	BA	5004	LHG	C2-C3-O3-P
19	HA	5004	LHG	C2-C3-O3-P
19	bA	5004	LHG	C11-C10-C9-C8
20	bA	5002	LMG	C12-C13-C14-C15
20	BA	5002	LMG	C12-C13-C14-C15
20	HA	5002	LMG	C12-C13-C14-C15
14	aA	1123	CLA	C11-C12-C13-C15
14	AA	1123	CLA	C11-C12-C13-C15
14	GA	1123	CLA	C11-C12-C13-C15
20	lA	5007	LMG	O9-C10-O7-C8
14	bA	1022	CLA	CAD-CBD-CGD-O1D
14	GA	1103	CLA	CAD-CBD-CGD-O1D
14	GA	1111	CLA	CAD-CBD-CGD-O1D
14	0	1012	CLA	CAD-CBD-CGD-O1D
14	aA	1103	CLA	CAD-CBD-CGD-O1D
14	aA	1136	CLA	CAD-CBD-CGD-O1D
14	AA	1103	CLA	CAD-CBD-CGD-O1D
14	AA	1111	CLA	CAD-CBD-CGD-O1D
14	BA	1022	CLA	CAD-CBD-CGD-O1D
14	AA	1136	CLA	CAD-CBD-CGD-O1D
14	GA	1136	CLA	CAD-CBD-CGD-O1D
14	aA	1111	CLA	CAD-CBD-CGD-O1D
14	aA	1136	CLA	CBA-CGA-O2A-C1

Continued on next page...

PRELIMINARY VALIDATION REPORT

Continued from previous page...

Mol	Chain	Res	Type	Atoms
14	AA	1136	CLA	CBA-CGA-O2A-C1
14	GA	1136	CLA	CBA-CGA-O2A-C1
14	aA	1124	CLA	C2-C1-O2A-CGA
14	HA	1215	CLA	C2-C1-O2A-CGA
14	AA	1124	CLA	C2-C1-O2A-CGA
14	HA	1201	CLA	C2-C1-O2A-CGA
14	GA	1131	CLA	C2-C1-O2A-CGA
14	aA	1126	CLA	C2-C1-O2A-CGA
14	GA	1126	CLA	C2-C1-O2A-CGA
14	BA	1215	CLA	C2-C1-O2A-CGA
14	AA	1131	CLA	C2-C1-O2A-CGA
14	AA	1126	CLA	C2-C1-O2A-CGA
14	bA	1201	CLA	C2-C1-O2A-CGA
14	BA	1201	CLA	C2-C1-O2A-CGA
14	aA	1131	CLA	C2-C1-O2A-CGA
14	GA	1124	CLA	C2-C1-O2A-CGA
14	bA	1215	CLA	C2-C1-O2A-CGA
19	BA	5004	LHG	C7-C8-C9-C10
20	LA	5007	LMG	O9-C10-O7-C8
20	UA	5007	LMG	O9-C10-O7-C8
19	bA	5004	LHG	C7-C8-C9-C10
19	HA	5004	LHG	C7-C8-C9-C10
14	aA	1123	CLA	C6-C7-C8-C10
16	bA	2002	PQN	C17-C18-C20-C21
16	AA	2001	PQN	C21-C22-C23-C25
14	aA	1133	CLA	C11-C10-C8-C7
14	AA	1132	CLA	C11-C10-C8-C7
14	AA	1133	CLA	C11-C10-C8-C7
16	GA	2001	PQN	C21-C22-C23-C25
14	aA	1117	CLA	C11-C12-C13-C15
14	HA	1205	CLA	C11-C10-C8-C7
14	aA	1125	CLA	C6-C7-C8-C10
14	aA	1104	CLA	C12-C13-C15-C16
14	aA	1132	CLA	C11-C10-C8-C7
14	bA	1205	CLA	C11-C10-C8-C7
16	BA	2002	PQN	C17-C18-C20-C21
14	HA	1239	CLA	C11-C12-C13-C15
14	GA	1125	CLA	C6-C7-C8-C10
14	GA	1117	CLA	C11-C12-C13-C15
14	AA	1123	CLA	C6-C7-C8-C10
14	GA	1133	CLA	C11-C10-C8-C7
14	aA	1126	CLA	C6-C7-C8-C10

Continued on next page...

PRELIMINARY VALIDATION REPORT

Continued from previous page...

Mol	Chain	Res	Type	Atoms
14	BA	1205	CLA	C11-C10-C8-C7
14	BA	1239	CLA	C11-C12-C13-C15
14	GA	1123	CLA	C6-C7-C8-C10
14	AA	1126	CLA	C6-C7-C8-C10
14	GA	1132	CLA	C11-C10-C8-C7
14	AA	1104	CLA	C12-C13-C15-C16
14	GA	1104	CLA	C12-C13-C15-C16
14	AA	1125	CLA	C6-C7-C8-C10
16	aA	2001	PQN	C21-C22-C23-C25
14	AA	1117	CLA	C11-C12-C13-C15
14	bA	1239	CLA	C11-C12-C13-C15
16	HA	2002	PQN	C17-C18-C20-C21
14	BA	1226	CLA	O1D-CGD-O2D-CED
14	GA	1131	CLA	C4-C3-C5-C6
14	aA	1126	CLA	C4-C3-C5-C6
14	GA	1126	CLA	C4-C3-C5-C6
14	AA	1131	CLA	C4-C3-C5-C6
14	AA	1126	CLA	C4-C3-C5-C6
14	aA	1131	CLA	C4-C3-C5-C6
14	bA	1226	CLA	O1D-CGD-O2D-CED
14	bA	1226	CLA	C16-C17-C18-C20
14	HA	1021	CLA	C16-C17-C18-C20
14	BA	1021	CLA	C16-C17-C18-C20
14	BA	1226	CLA	C16-C17-C18-C20
14	bA	1021	CLA	C16-C17-C18-C20
14	aA	1012	CLA	C10-C11-C12-C13
14	AA	1012	CLA	C10-C11-C12-C13
14	GA	1012	CLA	C10-C11-C12-C13
14	HA	1229	CLA	C3-C5-C6-C7
20	GA	5005	LMG	C7-C8-C9-O8
20	AA	5005	LMG	C7-C8-C9-O8
20	aA	5005	LMG	C7-C8-C9-O8
14	HA	1226	CLA	O1D-CGD-O2D-CED
14	bA	1226	CLA	C10-C11-C12-C13
14	BA	1226	CLA	C10-C11-C12-C13
14	BA	1226	CLA	C15-C16-C17-C18
14	HA	1226	CLA	C10-C11-C12-C13
14	HA	1226	CLA	C16-C17-C18-C20
14	bA	1229	CLA	C3-C5-C6-C7
14	BA	1229	CLA	C3-C5-C6-C7
14	BA	1217	CLA	CAA-CBA-CGA-O2A
14	GA	1140	CLA	CAA-CBA-CGA-O2A

Continued on next page...

PRELIMINARY VALIDATION REPORT

Continued from previous page...

Mol	Chain	Res	Type	Atoms
14	aA	1140	CLA	CAA-CBA-CGA-O2A
14	GA	1013	CLA	O1A-CGA-O2A-C1
14	aA	1013	CLA	O1A-CGA-O2A-C1
14	AA	1013	CLA	O1A-CGA-O2A-C1
14	bA	1226	CLA	C15-C16-C17-C18
14	HA	1226	CLA	C15-C16-C17-C18
14	AA	1140	CLA	CAA-CBA-CGA-O2A
14	bA	1217	CLA	CAA-CBA-CGA-O2A
14	aA	1116	CLA	CAA-CBA-CGA-O2A
14	HA	1217	CLA	CAA-CBA-CGA-O2A
14	HA	1222	CLA	C16-C17-C18-C20
14	bA	1222	CLA	C16-C17-C18-C20
14	UA	1502	CLA	C6-C7-C8-C9
14	bA	1214	CLA	C11-C10-C8-C9
14	BA	1204	CLA	C6-C7-C8-C9
14	bA	1204	CLA	C6-C7-C8-C9
14	HA	1021	CLA	C14-C13-C15-C16
14	HA	1214	CLA	C11-C10-C8-C9
14	BA	1021	CLA	C14-C13-C15-C16
14	HA	1203	CLA	C6-C7-C8-C9
14	bA	1203	CLA	C6-C7-C8-C9
14	lA	1502	CLA	C6-C7-C8-C9
14	HA	1204	CLA	C6-C7-C8-C9
14	BA	1214	CLA	C11-C10-C8-C9
14	bA	1021	CLA	C14-C13-C15-C16
14	BA	1203	CLA	C6-C7-C8-C9
14	LA	1502	CLA	C6-C7-C8-C9
14	AA	1116	CLA	CAA-CBA-CGA-O2A
18	HA	4016	BCR	C7-C8-C9-C34
18	AA	4001	BCR	C37-C22-C23-C24
18	aA	4001	BCR	C37-C22-C23-C24
18	GA	4001	BCR	C37-C22-C23-C24
14	BA	1222	CLA	C16-C17-C18-C20
14	bA	1214	CLA	C10-C11-C12-C13
14	BA	1235	CLA	C10-C11-C12-C13
14	bA	1235	CLA	C10-C11-C12-C13
14	HA	1235	CLA	C10-C11-C12-C13
14	aA	1136	CLA	O1A-CGA-O2A-C1
14	GA	1136	CLA	O1A-CGA-O2A-C1
14	GA	1116	CLA	CAA-CBA-CGA-O2A
14	BA	1214	CLA	C10-C11-C12-C13
14	AA	1136	CLA	O1A-CGA-O2A-C1

Continued on next page...

PRELIMINARY VALIDATION REPORT

Continued from previous page...

Mol	Chain	Res	Type	Atoms
14	BA	1211	CLA	C3-C5-C6-C7
14	HA	1214	CLA	C10-C11-C12-C13
20	RA	5006	LMG	O9-C10-O7-C8
20	iA	5006	LMG	O9-C10-O7-C8
20	IA	5006	LMG	O9-C10-O7-C8
14	bA	1211	CLA	C3-C5-C6-C7
14	HA	1211	CLA	C3-C5-C6-C7
14	bA	1202	CLA	CAA-CBA-CGA-O2A
14	BA	1202	CLA	CAA-CBA-CGA-O2A
18	aA	4003	BCR	C18-C19-C20-C21
18	IA	4020	BCR	C18-C19-C20-C21
18	BA	4012	BCR	C18-C19-C20-C21
18	MA	4021	BCR	C18-C19-C20-C21
18	AA	4003	BCR	C18-C19-C20-C21
18	RA	4020	BCR	C18-C19-C20-C21
18	iA	4020	BCR	C18-C19-C20-C21
18	bA	4012	BCR	C18-C19-C20-C21
18	bA	4013	BCR	C18-C19-C20-C21
18	HA	4012	BCR	C18-C19-C20-C21
18	bA	4006	BCR	C18-C19-C20-C21
18	HA	4006	BCR	C18-C19-C20-C21
18	GA	4003	BCR	C18-C19-C20-C21
18	BA	4006	BCR	C18-C19-C20-C21
18	mA	4021	BCR	C18-C19-C20-C21
18	BA	4013	BCR	C18-C19-C20-C21
18	HA	4013	BCR	C18-C19-C20-C21
18	VA	4021	BCR	C18-C19-C20-C21
14	AA	1140	CLA	O1D-CGD-O2D-CED
14	HA	1202	CLA	CAA-CBA-CGA-O2A
14	AA	1013	CLA	C8-C10-C11-C12
14	aA	1140	CLA	O1D-CGD-O2D-CED
18	HA	4017	BCR	C5-C6-C7-C8
18	bA	4017	BCR	C5-C6-C7-C8
18	BA	4017	BCR	C5-C6-C7-C8
14	bA	1221	CLA	C11-C12-C13-C14
14	BA	1221	CLA	C11-C12-C13-C14
14	HA	1221	CLA	C11-C12-C13-C14
14	GA	1013	CLA	C8-C10-C11-C12
14	aA	1013	CLA	C8-C10-C11-C12
20	IA	5006	LMG	C28-C29-C30-C31
14	GA	1140	CLA	O1D-CGD-O2D-CED
19	AA	5001	LHG	C3-O3-P-O6

Continued on next page...

PRELIMINARY VALIDATION REPORT

Continued from previous page...

Mol	Chain	Res	Type	Atoms
19	GA	5001	LHG	C3-O3-P-O6
19	aA	5001	LHG	C3-O3-P-O6
20	RA	5006	LMG	C28-C29-C30-C31
20	iA	5006	LMG	C28-C29-C30-C31
20	iA	5006	LMG	C30-C31-C32-C33
14	bA	1226	CLA	C3-C5-C6-C7
14	bA	1022	CLA	C15-C16-C17-C18
20	IA	5006	LMG	C30-C31-C32-C33
14	bA	1231	CLA	C2-C1-O2A-CGA
14	BA	1217	CLA	C2-C1-O2A-CGA
14	HA	1231	CLA	C2-C1-O2A-CGA
14	bA	1217	CLA	C2-C1-O2A-CGA
14	BA	1231	CLA	C2-C1-O2A-CGA
14	HA	1217	CLA	C2-C1-O2A-CGA
14	0	1012	CLA	C15-C16-C17-C18
14	BA	1022	CLA	C15-C16-C17-C18
18	BA	4012	BCR	C13-C14-C15-C16
18	bA	4012	BCR	C13-C14-C15-C16
18	HA	4012	BCR	C13-C14-C15-C16
20	RA	5006	LMG	C30-C31-C32-C33
14	BA	1226	CLA	C3-C5-C6-C7
14	HA	1226	CLA	C3-C5-C6-C7
14	bA	1226	CLA	C16-C17-C18-C19
14	BA	1226	CLA	C16-C17-C18-C19
14	HA	1226	CLA	C16-C17-C18-C19
14	BA	1238	CLA	C12-C13-C15-C16
14	HA	1233	CLA	C2A-CAA-CBA-CGA
14	aA	1125	CLA	C12-C13-C15-C16
14	GA	1125	CLA	C12-C13-C15-C16
14	AA	1129	CLA	C2A-CAA-CBA-CGA
14	GA	1129	CLA	C2A-CAA-CBA-CGA
14	bA	1233	CLA	C2A-CAA-CBA-CGA
14	bA	1238	CLA	C12-C13-C15-C16
14	HA	1238	CLA	C12-C13-C15-C16
14	BA	1233	CLA	C2A-CAA-CBA-CGA
14	aA	1129	CLA	C2A-CAA-CBA-CGA
14	AA	1125	CLA	C12-C13-C15-C16
14	AA	1123	CLA	O1D-CGD-O2D-CED
14	aA	1125	CLA	C16-C17-C18-C20
14	GA	1125	CLA	C16-C17-C18-C20
14	AA	1125	CLA	C16-C17-C18-C20
14	GA	1123	CLA	O1D-CGD-O2D-CED

Continued on next page...

PRELIMINARY VALIDATION REPORT

Continued from previous page...

Mol	Chain	Res	Type	Atoms
14	bA	1226	CLA	C6-C7-C8-C9
14	HA	1205	CLA	C11-C10-C8-C9
14	aA	1104	CLA	C14-C13-C15-C16
14	bA	1205	CLA	C11-C10-C8-C9
14	lA	1503	CLA	C11-C10-C8-C9
14	BA	1205	CLA	C11-C10-C8-C9
14	LA	1503	CLA	C11-C10-C8-C9
14	AA	1104	CLA	C14-C13-C15-C16
14	BA	1226	CLA	C6-C7-C8-C9
14	GA	1104	CLA	C14-C13-C15-C16
14	HA	1226	CLA	C6-C7-C8-C9
14	UA	1503	CLA	C11-C10-C8-C9
14	HA	1216	CLA	C3-C5-C6-C7
14	bA	1216	CLA	C3-C5-C6-C7
18	BA	4016	BCR	C7-C8-C9-C34
18	MA	4021	BCR	C37-C22-C23-C24
18	bA	4016	BCR	C7-C8-C9-C34
18	mA	4021	BCR	C37-C22-C23-C24
18	VA	4021	BCR	C37-C22-C23-C24
14	bA	1214	CLA	C11-C12-C13-C15
14	HA	1214	CLA	C11-C12-C13-C15
14	BA	1214	CLA	C11-C12-C13-C15
20	LA	5007	LMG	C11-C10-O7-C8
20	iA	5006	LMG	C11-C10-O7-C8
20	IA	5006	LMG	C11-C10-O7-C8
14	GA	1140	CLA	CBD-CGD-O2D-CED
14	aA	1140	CLA	CBD-CGD-O2D-CED
14	BA	1216	CLA	C3-C5-C6-C7
18	LA	4019	BCR	C13-C14-C15-C16
18	lA	4019	BCR	C13-C14-C15-C16
18	UA	4019	BCR	C13-C14-C15-C16
14	HA	1229	CLA	C5-C6-C7-C8
14	HA	1205	CLA	C5-C6-C7-C8
14	bA	1205	CLA	C5-C6-C7-C8
14	bA	1229	CLA	C5-C6-C7-C8
14	BA	1205	CLA	C5-C6-C7-C8
14	BA	1229	CLA	C5-C6-C7-C8
14	AA	1140	CLA	CBD-CGD-O2D-CED
14	aA	1123	CLA	O1D-CGD-O2D-CED
20	RA	5006	LMG	C11-C10-O7-C8
14	HA	1208	CLA	C16-C17-C18-C19
14	BA	1208	CLA	C16-C17-C18-C19

Continued on next page...

PRELIMINARY VALIDATION REPORT

Continued from previous page...

Mol	Chain	Res	Type	Atoms
14	bA	1208	CLA	C16-C17-C18-C19
20	RA	5006	LMG	C33-C34-C35-C36
20	iA	5006	LMG	C33-C34-C35-C36
20	IA	5006	LMG	C33-C34-C35-C36
14	aA	1123	CLA	C10-C11-C12-C13
14	bA	1023	CLA	C10-C11-C12-C13
14	AA	1123	CLA	C10-C11-C12-C13
14	GA	1131	CLA	C10-C11-C12-C13
14	GA	1123	CLA	C10-C11-C12-C13
14	AA	1131	CLA	C10-C11-C12-C13
14	HA	1023	CLA	C10-C11-C12-C13
14	aA	1131	CLA	C10-C11-C12-C13
14	BA	1023	CLA	C10-C11-C12-C13
20	lA	5007	LMG	C11-C10-O7-C8
18	aA	4011	BCR	C19-C20-C21-C22
18	BA	4012	BCR	C15-C16-C17-C18
18	GA	4011	BCR	C19-C20-C21-C22
18	AA	4011	BCR	C19-C20-C21-C22
14	WA	1701	CLA	C3A-C2A-CAA-CBA
14	XA	1701	CLA	C3A-C2A-CAA-CBA
14	KA	1401	CLA	C3A-C2A-CAA-CBA
14	kA	1401	CLA	C3A-C2A-CAA-CBA
14	xA	1701	CLA	C3A-C2A-CAA-CBA
14	TA	1401	CLA	C3A-C2A-CAA-CBA
14	aA	1125	CLA	C2A-CAA-CBA-CGA
14	GA	1125	CLA	C2A-CAA-CBA-CGA
14	AA	1125	CLA	C2A-CAA-CBA-CGA
18	HA	4016	BCR	C35-C13-C14-C15
18	aA	4011	BCR	C11-C10-C9-C34
18	aA	4011	BCR	C16-C17-C18-C36
18	BA	4016	BCR	C35-C13-C14-C15
18	IA	4020	BCR	C20-C21-C22-C37
18	RA	4020	BCR	C20-C21-C22-C37
18	iA	4020	BCR	C20-C21-C22-C37
18	bA	4010	BCR	C16-C17-C18-C36
18	aA	4007	BCR	C16-C17-C18-C36
18	bA	4016	BCR	C35-C13-C14-C15
18	HA	4010	BCR	C16-C17-C18-C36
18	GA	4011	BCR	C11-C10-C9-C34
18	GA	4011	BCR	C16-C17-C18-C36
18	AA	4007	BCR	C16-C17-C18-C36
18	BA	4010	BCR	C16-C17-C18-C36

Continued on next page...

PRELIMINARY VALIDATION REPORT

Continued from previous page...

Mol	Chain	Res	Type	Atoms
18	AA	4011	BCR	C11-C10-C9-C34
18	AA	4011	BCR	C16-C17-C18-C36
18	GA	4007	BCR	C16-C17-C18-C36
14	bA	1210	CLA	CAA-CBA-CGA-O2A
14	HA	1210	CLA	CAA-CBA-CGA-O2A
18	iA	4018	BCR	C10-C11-C12-C13
20	UA	5007	LMG	C11-C10-O7-C8
14	BA	1210	CLA	CAA-CBA-CGA-O2A
14	bA	1221	CLA	C11-C12-C13-C15
14	BA	1221	CLA	C11-C12-C13-C15
14	HA	1221	CLA	C11-C12-C13-C15
14	bA	1211	CLA	C11-C12-C13-C14
14	BA	1238	CLA	C14-C13-C15-C16
14	aA	1117	CLA	C11-C12-C13-C14
14	HA	1239	CLA	C14-C13-C15-C16
14	GA	1117	CLA	C11-C12-C13-C14
14	bA	1238	CLA	C14-C13-C15-C16
14	HA	1211	CLA	C11-C12-C13-C14
14	HA	1238	CLA	C14-C13-C15-C16
14	BA	1239	CLA	C14-C13-C15-C16
14	BA	1211	CLA	C11-C12-C13-C14
14	AA	1117	CLA	C11-C12-C13-C14
14	bA	1239	CLA	C14-C13-C15-C16
14	bA	1201	CLA	C5-C6-C7-C8
14	BA	1201	CLA	C5-C6-C7-C8
14	BA	1228	CLA	C1A-C2A-CAA-CBA
14	BA	1238	CLA	C1A-C2A-CAA-CBA
14	AA	1140	CLA	C1A-C2A-CAA-CBA
14	GA	1140	CLA	C1A-C2A-CAA-CBA
14	HA	1228	CLA	C1A-C2A-CAA-CBA
14	aA	1140	CLA	C1A-C2A-CAA-CBA
14	bA	1238	CLA	C1A-C2A-CAA-CBA
14	HA	1238	CLA	C1A-C2A-CAA-CBA
14	bA	1228	CLA	C1A-C2A-CAA-CBA
20	GA	5005	LMG	C28-C29-C30-C31
20	AA	5005	LMG	C28-C29-C30-C31
20	aA	5005	LMG	C28-C29-C30-C31
18	bA	4012	BCR	C15-C16-C17-C18
18	bA	4013	BCR	C13-C14-C15-C16
18	HA	4012	BCR	C15-C16-C17-C18
18	BA	4013	BCR	C13-C14-C15-C16
18	HA	4013	BCR	C13-C14-C15-C16

Continued on next page...

PRELIMINARY VALIDATION REPORT

Continued from previous page...

Mol	Chain	Res	Type	Atoms
14	aA	1128	CLA	C11-C12-C13-C15
14	GA	1128	CLA	C11-C12-C13-C15
14	AA	1128	CLA	C11-C12-C13-C15
14	HA	1201	CLA	C5-C6-C7-C8
14	GA	1116	CLA	C2A-CAA-CBA-CGA
14	BA	1220	CLA	C2A-CAA-CBA-CGA
14	AA	1116	CLA	C2A-CAA-CBA-CGA
14	aA	1126	CLA	C2A-CAA-CBA-CGA
14	GA	1126	CLA	C2A-CAA-CBA-CGA
14	AA	1126	CLA	C2A-CAA-CBA-CGA
14	aA	1116	CLA	C2A-CAA-CBA-CGA
14	bA	1220	CLA	C2A-CAA-CBA-CGA
14	HA	1220	CLA	C2A-CAA-CBA-CGA
14	GA	1103	CLA	C11-C10-C8-C7
14	HA	1208	CLA	C12-C13-C15-C16
14	AA	1132	CLA	C11-C12-C13-C15
14	bA	1226	CLA	C12-C13-C15-C16
14	aA	1132	CLA	C11-C12-C13-C15
14	BA	1208	CLA	C12-C13-C15-C16
14	aA	1103	CLA	C11-C10-C8-C7
14	AA	1103	CLA	C11-C10-C8-C7
14	GA	1132	CLA	C11-C12-C13-C15
14	bA	1208	CLA	C12-C13-C15-C16
14	BA	1226	CLA	C12-C13-C15-C16
14	HA	1226	CLA	C12-C13-C15-C16
14	HA	1205	CLA	CAA-CBA-CGA-O2A
14	BA	1205	CLA	CAA-CBA-CGA-O2A
14	bA	1205	CLA	CAA-CBA-CGA-O2A
21	aA	6002	LMT	C5'-C4'-O1B-C1B
21	AA	6002	LMT	C5'-C4'-O1B-C1B
21	GA	6002	LMT	C5'-C4'-O1B-C1B
14	aA	1012	CLA	C4-C3-C5-C6
14	AA	1012	CLA	C4-C3-C5-C6
14	GA	1012	CLA	C4-C3-C5-C6
20	LA	5007	LMG	C28-C29-C30-C31
20	UA	5007	LMG	C28-C29-C30-C31
20	lA	5007	LMG	C28-C29-C30-C31
18	HA	4016	BCR	C12-C13-C14-C15
18	aA	4011	BCR	C11-C10-C9-C8
18	aA	4011	BCR	C16-C17-C18-C19
18	BA	4016	BCR	C12-C13-C14-C15
18	IA	4020	BCR	C20-C21-C22-C23

Continued on next page...

PRELIMINARY VALIDATION REPORT

Continued from previous page...

Mol	Chain	Res	Type	Atoms
18	RA	4020	BCR	C20-C21-C22-C23
18	iA	4020	BCR	C20-C21-C22-C23
18	aA	4007	BCR	C16-C17-C18-C19
18	bA	4016	BCR	C12-C13-C14-C15
18	GA	4011	BCR	C11-C10-C9-C8
18	GA	4011	BCR	C16-C17-C18-C19
18	bA	4006	BCR	C11-C10-C9-C8
18	AA	4007	BCR	C16-C17-C18-C19
18	HA	4006	BCR	C11-C10-C9-C8
18	BA	4006	BCR	C11-C10-C9-C8
18	AA	4011	BCR	C11-C10-C9-C8
18	AA	4011	BCR	C16-C17-C18-C19
18	GA	4007	BCR	C16-C17-C18-C19
14	GA	1119	CLA	C13-C15-C16-C17
14	AA	1119	CLA	C13-C15-C16-C17
18	HA	4016	BCR	C19-C20-C21-C22
18	BA	4009	BCR	C9-C10-C11-C12
14	aA	1119	CLA	C13-C15-C16-C17
19	AA	5001	LHG	O7-C5-C6-O8
19	GA	5001	LHG	O7-C5-C6-O8
19	aA	5001	LHG	O7-C5-C6-O8
14	GA	1116	CLA	C5-C6-C7-C8
14	AA	1116	CLA	C5-C6-C7-C8
14	aA	1116	CLA	C5-C6-C7-C8
14	aA	1125	CLA	C4-C3-C5-C6
14	GA	1125	CLA	C4-C3-C5-C6
14	AA	1125	CLA	C4-C3-C5-C6
14	GA	1131	CLA	C2-C3-C5-C6
14	AA	1131	CLA	C2-C3-C5-C6
14	aA	1131	CLA	C2-C3-C5-C6
14	aA	1136	CLA	CAA-CBA-CGA-O2A
14	AA	1136	CLA	CAA-CBA-CGA-O2A
14	GA	1136	CLA	CAA-CBA-CGA-O2A
14	AA	1132	CLA	C6-C7-C8-C9
18	BA	4009	BCR	C23-C24-C25-C30
18	bA	4009	BCR	C23-C24-C25-C30
14	aA	1132	CLA	C6-C7-C8-C9
18	AA	4002	BCR	C23-C24-C25-C30
18	aA	4002	BCR	C23-C24-C25-C30
18	GA	4002	BCR	C23-C24-C25-C26
18	GA	4002	BCR	C23-C24-C25-C30
14	GA	1132	CLA	C6-C7-C8-C9

Continued on next page...

PRELIMINARY VALIDATION REPORT

Continued from previous page...

Mol	Chain	Res	Type	Atoms
18	HA	4009	BCR	C23-C24-C25-C30
18	HA	4016	BCR	C9-C10-C11-C12
18	BA	4016	BCR	C19-C20-C21-C22
18	bA	4009	BCR	C9-C10-C11-C12
18	bA	4016	BCR	C9-C10-C11-C12
18	bA	4016	BCR	C19-C20-C21-C22
18	HA	4009	BCR	C9-C10-C11-C12
14	BA	1220	CLA	CAA-CBA-CGA-O1A
14	bA	1220	CLA	CAA-CBA-CGA-O1A
14	HA	1220	CLA	CAA-CBA-CGA-O1A
14	AA	1132	CLA	C4-C3-C5-C6
14	aA	1132	CLA	C4-C3-C5-C6
14	HA	1202	CLA	C4-C3-C5-C6
14	bA	1202	CLA	C4-C3-C5-C6
14	GA	1132	CLA	C4-C3-C5-C6
14	BA	1202	CLA	C4-C3-C5-C6
20	lA	5007	LMG	C33-C34-C35-C36
20	LA	5007	LMG	C33-C34-C35-C36
20	UA	5007	LMG	C33-C34-C35-C36
18	HA	4016	BCR	C7-C8-C9-C10
18	BA	4016	BCR	C7-C8-C9-C10
18	bA	4016	BCR	C7-C8-C9-C10
14	LA	1501	CLA	C8-C10-C11-C12
14	BA	1225	CLA	C10-C11-C12-C13
14	HA	1225	CLA	C10-C11-C12-C13
14	bA	1225	CLA	C10-C11-C12-C13
14	lA	1501	CLA	C8-C10-C11-C12
14	UA	1501	CLA	C8-C10-C11-C12
14	HA	1222	CLA	C16-C17-C18-C19
14	bA	1222	CLA	C16-C17-C18-C19
14	BA	1222	CLA	C16-C17-C18-C19
20	BA	5002	LMG	C29-C30-C31-C32
14	HA	1203	CLA	C11-C10-C8-C7
14	bA	1203	CLA	C11-C10-C8-C7
14	BA	1203	CLA	C11-C10-C8-C7
14	AA	1122	CLA	O1D-CGD-O2D-CED
14	HA	1215	CLA	C2-C3-C5-C6
14	aA	1012	CLA	C2-C3-C5-C6
14	BA	1215	CLA	C2-C3-C5-C6
14	AA	1012	CLA	C2-C3-C5-C6
14	GA	1012	CLA	C2-C3-C5-C6
14	bA	1215	CLA	C2-C3-C5-C6

Continued on next page...

PRELIMINARY VALIDATION REPORT

Continued from previous page...

Mol	Chain	Res	Type	Atoms
20	bA	5002	LMG	C29-C30-C31-C32
20	HA	5002	LMG	C29-C30-C31-C32
20	GA	5005	LMG	C34-C35-C36-C37
18	BA	4016	BCR	C9-C10-C11-C12
18	GA	4011	BCR	C9-C10-C11-C12
18	AA	4011	BCR	C9-C10-C11-C12
14	aA	1122	CLA	O1D-CGD-O2D-CED
20	AA	5005	LMG	C34-C35-C36-C37
20	aA	5005	LMG	C34-C35-C36-C37
14	GA	1138	CLA	CAA-CBA-CGA-O2A
14	aA	1138	CLA	CAA-CBA-CGA-O2A
18	bA	4006	BCR	C11-C10-C9-C34
18	HA	4006	BCR	C11-C10-C9-C34
18	BA	4006	BCR	C11-C10-C9-C34
14	AA	1138	CLA	CAA-CBA-CGA-O2A
14	HA	1239	CLA	CAA-CBA-CGA-O2A
14	GA	1131	CLA	CAA-CBA-CGA-O2A
14	aA	1126	CLA	CAA-CBA-CGA-O2A
14	GA	1126	CLA	CAA-CBA-CGA-O2A
14	BA	1239	CLA	CAA-CBA-CGA-O2A
14	AA	1131	CLA	CAA-CBA-CGA-O2A
14	aA	1131	CLA	CAA-CBA-CGA-O2A
14	bA	1239	CLA	CAA-CBA-CGA-O2A
14	GA	1122	CLA	O1D-CGD-O2D-CED
14	LA	1502	CLA	C5-C6-C7-C8
14	AA	1127	CLA	C4-C3-C5-C6
14	GA	1127	CLA	C4-C3-C5-C6
14	aA	1127	CLA	C4-C3-C5-C6
14	bA	1231	CLA	CAA-CBA-CGA-O2A
14	HA	1231	CLA	CAA-CBA-CGA-O2A
14	aA	1125	CLA	CAA-CBA-CGA-O2A
14	GA	1125	CLA	CAA-CBA-CGA-O2A
14	lA	1503	CLA	CAA-CBA-CGA-O2A
14	BA	1231	CLA	CAA-CBA-CGA-O2A
14	AA	1126	CLA	CAA-CBA-CGA-O2A
14	bA	1235	CLA	CAA-CBA-CGA-O2A
14	LA	1503	CLA	CAA-CBA-CGA-O2A
14	AA	1125	CLA	CAA-CBA-CGA-O2A
14	UA	1503	CLA	CAA-CBA-CGA-O2A
14	lA	1502	CLA	C5-C6-C7-C8
14	HA	1215	CLA	C2A-CAA-CBA-CGA
14	BA	1215	CLA	C2A-CAA-CBA-CGA

Continued on next page...

PRELIMINARY VALIDATION REPORT

Continued from previous page...

Mol	Chain	Res	Type	Atoms
14	bA	1215	CLA	C2A-CAA-CBA-CGA
18	aA	4011	BCR	C9-C10-C11-C12
14	aA	1124	CLA	C3A-C2A-CAA-CBA
14	aA	1012	CLA	C3A-C2A-CAA-CBA
14	AA	1124	CLA	C3A-C2A-CAA-CBA
14	AA	1112	CLA	C3A-C2A-CAA-CBA
14	aA	1136	CLA	C3A-C2A-CAA-CBA
14	aA	1112	CLA	C3A-C2A-CAA-CBA
14	AA	1136	CLA	C3A-C2A-CAA-CBA
14	GA	1136	CLA	C3A-C2A-CAA-CBA
14	AA	1012	CLA	C3A-C2A-CAA-CBA
14	GA	1112	CLA	C3A-C2A-CAA-CBA
14	GA	1012	CLA	C3A-C2A-CAA-CBA
14	GA	1124	CLA	C3A-C2A-CAA-CBA
14	UA	1502	CLA	C5-C6-C7-C8
14	aA	1119	CLA	C16-C17-C18-C19
14	aA	1117	CLA	C16-C17-C18-C20
14	GA	1117	CLA	C16-C17-C18-C20
14	AA	1125	CLA	C16-C17-C18-C19
14	AA	1117	CLA	C16-C17-C18-C20
14	BA	1235	CLA	CAA-CBA-CGA-O2A
14	HA	1235	CLA	CAA-CBA-CGA-O2A
14	GA	1103	CLA	C11-C10-C8-C9
14	AA	1132	CLA	C11-C10-C8-C9
14	AA	1132	CLA	C11-C12-C13-C14
14	bA	1226	CLA	C14-C13-C15-C16
14	aA	1125	CLA	C14-C13-C15-C16
14	aA	1132	CLA	C11-C10-C8-C9
14	aA	1132	CLA	C11-C12-C13-C14
14	HA	1239	CLA	C11-C12-C13-C14
14	GA	1125	CLA	C14-C13-C15-C16
14	aA	1103	CLA	C11-C10-C8-C9
14	AA	1103	CLA	C11-C10-C8-C9
14	BA	1239	CLA	C11-C12-C13-C14
14	GA	1132	CLA	C11-C10-C8-C9
14	GA	1132	CLA	C11-C12-C13-C14
14	BA	1226	CLA	C14-C13-C15-C16
14	HA	1226	CLA	C14-C13-C15-C16
14	AA	1125	CLA	C14-C13-C15-C16
14	bA	1239	CLA	C11-C12-C13-C14
14	bA	1211	CLA	CAD-CBD-CGD-O2D
14	aA	1123	CLA	CAD-CBD-CGD-O2D

Continued on next page...

PRELIMINARY VALIDATION REPORT

Continued from previous page...

Mol	Chain	Res	Type	Atoms
14	BA	1238	CLA	CAD-CBD-CGD-O2D
14	aA	1104	CLA	CAD-CBD-CGD-O2D
14	AA	1123	CLA	CAD-CBD-CGD-O2D
14	bA	1238	CLA	CAD-CBD-CGD-O2D
14	HA	1211	CLA	CAD-CBD-CGD-O2D
14	GA	1123	CLA	CAD-CBD-CGD-O2D
14	AA	1104	CLA	CAD-CBD-CGD-O2D
14	bA	1227	CLA	CAD-CBD-CGD-O2D
14	GA	1104	CLA	CAD-CBD-CGD-O2D
14	BA	1227	CLA	CAD-CBD-CGD-O2D
14	BA	1211	CLA	CAD-CBD-CGD-O2D
14	HA	1227	CLA	CAD-CBD-CGD-O2D
14	AA	1123	CLA	CBD-CGD-O2D-CED
14	LA	1501	CLA	CAA-CBA-CGA-O2A
14	aA	1133	CLA	CAA-CBA-CGA-O2A
14	AA	1133	CLA	CAA-CBA-CGA-O2A
14	lA	1501	CLA	CAA-CBA-CGA-O2A
14	GA	1133	CLA	CAA-CBA-CGA-O2A
14	UA	1501	CLA	CAA-CBA-CGA-O2A
14	UA	1502	CLA	C4-C3-C5-C6
14	GA	1104	CLA	C4-C3-C5-C6
14	lA	1502	CLA	C4-C3-C5-C6
14	LA	1502	CLA	C4-C3-C5-C6
14	aA	1125	CLA	C16-C17-C18-C19
14	GA	1119	CLA	C16-C17-C18-C19
14	AA	1119	CLA	C16-C17-C18-C19
14	aA	1123	CLA	CBD-CGD-O2D-CED
14	GA	1123	CLA	CBD-CGD-O2D-CED
14	aA	1136	CLA	C2A-CAA-CBA-CGA
14	AA	1136	CLA	C2A-CAA-CBA-CGA
14	GA	1136	CLA	C2A-CAA-CBA-CGA
18	IA	4020	BCR	C21-C22-C23-C24
18	bA	4014	BCR	C7-C8-C9-C10
18	RA	4020	BCR	C21-C22-C23-C24
18	iA	4020	BCR	C21-C22-C23-C24
18	HA	4014	BCR	C7-C8-C9-C10
18	AA	4001	BCR	C21-C22-C23-C24
18	aA	4001	BCR	C21-C22-C23-C24
18	bA	4013	BCR	C7-C8-C9-C10
18	BA	4014	BCR	C7-C8-C9-C10
18	GA	4001	BCR	C21-C22-C23-C24
18	BA	4013	BCR	C7-C8-C9-C10

Continued on next page...

PRELIMINARY VALIDATION REPORT

Continued from previous page...

Mol	Chain	Res	Type	Atoms
18	HA	4013	BCR	C7-C8-C9-C10
20	LA	5007	LMG	O1-C7-C8-C9
20	UA	5007	LMG	O1-C7-C8-C9
20	lA	5007	LMG	O1-C7-C8-C9
14	aA	1128	CLA	C11-C12-C13-C14
14	GA	1128	CLA	C11-C12-C13-C14
14	AA	1128	CLA	C11-C12-C13-C14
14	GA	1125	CLA	C16-C17-C18-C19
20	GA	5005	LMG	O8-C28-C29-C30
20	AA	5005	LMG	O8-C28-C29-C30
20	aA	5005	LMG	O8-C28-C29-C30
18	bA	4010	BCR	C16-C17-C18-C19
18	HA	4010	BCR	C16-C17-C18-C19
18	BA	4010	BCR	C16-C17-C18-C19
14	aA	1104	CLA	C4-C3-C5-C6
14	AA	1104	CLA	C4-C3-C5-C6
14	BA	1021	CLA	C2A-CAA-CBA-CGA
14	BA	1213	CLA	CAA-CBA-CGA-O2A
14	bA	1213	CLA	CAA-CBA-CGA-O2A
14	HA	1213	CLA	CAA-CBA-CGA-O2A
14	AA	1132	CLA	C2-C3-C5-C6
14	aA	1132	CLA	C2-C3-C5-C6
14	aA	1126	CLA	C2-C3-C5-C6
14	GA	1126	CLA	C2-C3-C5-C6
14	AA	1126	CLA	C2-C3-C5-C6
14	GA	1132	CLA	C2-C3-C5-C6
20	RA	5006	LMG	C31-C32-C33-C34
19	bA	5004	LHG	O7-C5-C6-O8
19	BA	5004	LHG	O7-C5-C6-O8
19	HA	5004	LHG	O7-C5-C6-O8
14	GA	1140	CLA	C16-C17-C18-C19
14	GA	1127	CLA	C8-C10-C11-C12
20	iA	5006	LMG	C31-C32-C33-C34
20	IA	5006	LMG	C31-C32-C33-C34
14	aA	1127	CLA	C8-C10-C11-C12
14	AA	1127	CLA	C8-C10-C11-C12
14	AA	1140	CLA	C16-C17-C18-C19
14	aA	1132	CLA	C2C-C3C-CAC-CBC
14	LA	1501	CLA	CHA-CBD-CGD-O1D
14	LA	1501	CLA	CHA-CBD-CGD-O2D
14	bA	1234	CLA	CHA-CBD-CGD-O1D
14	UA	1502	CLA	CHA-CBD-CGD-O1D

Continued on next page...

PRELIMINARY VALIDATION REPORT

Continued from previous page...

Mol	Chain	Res	Type	Atoms
14	UA	1502	CLA	CHA-CBD-CGD-O2D
14	BA	1228	CLA	CHA-CBD-CGD-O1D
14	BA	1228	CLA	CHA-CBD-CGD-O2D
14	aA	1102	CLA	CHA-CBD-CGD-O1D
14	aA	1102	CLA	CHA-CBD-CGD-O2D
14	HA	1215	CLA	CHA-CBD-CGD-O1D
14	HA	1215	CLA	CHA-CBD-CGD-O2D
14	BA	1219	CLA	CHA-CBD-CGD-O1D
14	BA	1219	CLA	CHA-CBD-CGD-O2D
14	BA	1217	CLA	CHA-CBD-CGD-O1D
14	BA	1217	CLA	CHA-CBD-CGD-O2D
14	HA	1219	CLA	CHA-CBD-CGD-O1D
14	HA	1219	CLA	CHA-CBD-CGD-O2D
14	aA	1114	CLA	CHA-CBD-CGD-O1D
14	aA	1114	CLA	CHA-CBD-CGD-O2D
14	bA	1217	CLA	CHA-CBD-CGD-O1D
14	bA	1217	CLA	CHA-CBD-CGD-O2D
14	bA	1236	CLA	CHA-CBD-CGD-O1D
14	bA	1236	CLA	CHA-CBD-CGD-O2D
14	GA	1102	CLA	CHA-CBD-CGD-O1D
14	GA	1102	CLA	CHA-CBD-CGD-O2D
14	GA	1114	CLA	CHA-CBD-CGD-O1D
14	GA	1114	CLA	CHA-CBD-CGD-O2D
14	AA	1114	CLA	CHA-CBD-CGD-O1D
14	AA	1114	CLA	CHA-CBD-CGD-O2D
14	HA	1228	CLA	CHA-CBD-CGD-O1D
14	HA	1228	CLA	CHA-CBD-CGD-O2D
14	BA	1236	CLA	CHA-CBD-CGD-O1D
14	BA	1236	CLA	CHA-CBD-CGD-O2D
14	lA	1501	CLA	CHA-CBD-CGD-O1D
14	lA	1501	CLA	CHA-CBD-CGD-O2D
14	BA	1232	CLA	CHA-CBD-CGD-O2D
14	AA	1129	CLA	CHA-CBD-CGD-O1D
14	AA	1129	CLA	CHA-CBD-CGD-O2D
14	GA	1129	CLA	CHA-CBD-CGD-O1D
14	GA	1129	CLA	CHA-CBD-CGD-O2D
14	HA	1234	CLA	CHA-CBD-CGD-O1D
14	UA	1501	CLA	CHA-CBD-CGD-O1D
14	UA	1501	CLA	CHA-CBD-CGD-O2D
14	HA	1232	CLA	CHA-CBD-CGD-O2D
14	BA	1215	CLA	CHA-CBD-CGD-O1D
14	BA	1215	CLA	CHA-CBD-CGD-O2D

Continued on next page...

PRELIMINARY VALIDATION REPORT

Continued from previous page...

Mol	Chain	Res	Type	Atoms
14	AA	1102	CLA	CHA-CBD-CGD-O1D
14	AA	1102	CLA	CHA-CBD-CGD-O2D
14	BA	1206	CLA	CHA-CBD-CGD-O2D
14	lA	1502	CLA	CHA-CBD-CGD-O1D
14	lA	1502	CLA	CHA-CBD-CGD-O2D
14	HA	1217	CLA	CHA-CBD-CGD-O1D
14	HA	1217	CLA	CHA-CBD-CGD-O2D
14	aA	1129	CLA	CHA-CBD-CGD-O1D
14	aA	1129	CLA	CHA-CBD-CGD-O2D
14	bA	1219	CLA	CHA-CBD-CGD-O1D
14	bA	1219	CLA	CHA-CBD-CGD-O2D
14	HA	1236	CLA	CHA-CBD-CGD-O1D
14	HA	1236	CLA	CHA-CBD-CGD-O2D
14	bA	1228	CLA	CHA-CBD-CGD-O1D
14	bA	1228	CLA	CHA-CBD-CGD-O2D
14	bA	1232	CLA	CHA-CBD-CGD-O2D
14	BA	1234	CLA	CHA-CBD-CGD-O1D
14	bA	1215	CLA	CHA-CBD-CGD-O1D
14	bA	1215	CLA	CHA-CBD-CGD-O2D
14	bA	1206	CLA	CHA-CBD-CGD-O2D
14	HA	1206	CLA	CHA-CBD-CGD-O2D
14	LA	1502	CLA	CHA-CBD-CGD-O1D
14	LA	1502	CLA	CHA-CBD-CGD-O2D
14	AA	1132	CLA	C2C-C3C-CAC-CBC
14	GA	1132	CLA	C2C-C3C-CAC-CBC
14	HA	1021	CLA	C2A-CAA-CBA-CGA
14	bA	1021	CLA	C2A-CAA-CBA-CGA
18	HA	4012	BCR	C9-C10-C11-C12
14	aA	1132	CLA	C4C-C3C-CAC-CBC
14	aA	1140	CLA	C16-C17-C18-C19
14	bA	1224	CLA	C16-C17-C18-C19
14	GA	1132	CLA	C4C-C3C-CAC-CBC
20	bA	5002	LMG	C11-C12-C13-C14
14	bA	1231	CLA	CAA-CBA-CGA-O1A
14	BA	1231	CLA	CAA-CBA-CGA-O1A
20	BA	5002	LMG	C30-C31-C32-C33
14	BA	1225	CLA	CBA-CGA-O2A-C1
16	AA	2001	PQN	C21-C22-C23-C24
14	AA	1140	CLA	C11-C10-C8-C9
16	GA	2001	PQN	C21-C22-C23-C24
14	GA	1140	CLA	C11-C10-C8-C9
14	HA	1202	CLA	C6-C7-C8-C9

Continued on next page...

PRELIMINARY VALIDATION REPORT

Continued from previous page...

Mol	Chain	Res	Type	Atoms
14	aA	1140	CLA	C11-C10-C8-C9
14	bA	1202	CLA	C6-C7-C8-C9
16	aA	2001	PQN	C21-C22-C23-C24
14	BA	1202	CLA	C6-C7-C8-C9
14	AA	1132	CLA	C4C-C3C-CAC-CBC
14	HA	1224	CLA	C16-C17-C18-C19
14	BA	1224	CLA	C16-C17-C18-C19
20	bA	5002	LMG	C30-C31-C32-C33
20	HA	5002	LMG	C11-C12-C13-C14
20	HA	5002	LMG	C30-C31-C32-C33
14	WA	1701	CLA	C1A-C2A-CAA-CBA
14	bA	1223	CLA	C1A-C2A-CAA-CBA
14	aA	1012	CLA	C1A-C2A-CAA-CBA
14	aA	1114	CLA	C1A-C2A-CAA-CBA
14	AA	1112	CLA	C1A-C2A-CAA-CBA
14	GA	1114	CLA	C1A-C2A-CAA-CBA
14	HA	1205	CLA	C1A-C2A-CAA-CBA
14	BA	1223	CLA	C1A-C2A-CAA-CBA
14	AA	1114	CLA	C1A-C2A-CAA-CBA
14	bA	1205	CLA	C1A-C2A-CAA-CBA
14	HA	1202	CLA	C1A-C2A-CAA-CBA
14	aA	1112	CLA	C1A-C2A-CAA-CBA
14	BA	1205	CLA	C1A-C2A-CAA-CBA
14	bA	1202	CLA	C1A-C2A-CAA-CBA
14	XA	1701	CLA	C1A-C2A-CAA-CBA
14	GA	1013	CLA	C1A-C2A-CAA-CBA
14	AA	1012	CLA	C1A-C2A-CAA-CBA
14	KA	1401	CLA	C1A-C2A-CAA-CBA
14	GA	1112	CLA	C1A-C2A-CAA-CBA
14	kA	1401	CLA	C1A-C2A-CAA-CBA
14	GA	1012	CLA	C1A-C2A-CAA-CBA
14	xA	1701	CLA	C1A-C2A-CAA-CBA
14	aA	1013	CLA	C1A-C2A-CAA-CBA
14	HA	1223	CLA	C1A-C2A-CAA-CBA
14	TA	1401	CLA	C1A-C2A-CAA-CBA
14	AA	1013	CLA	C1A-C2A-CAA-CBA
14	BA	1202	CLA	C1A-C2A-CAA-CBA
20	BA	5002	LMG	C11-C12-C13-C14
14	HA	1225	CLA	CBA-CGA-O2A-C1
14	bA	1225	CLA	CBA-CGA-O2A-C1
14	AA	1138	CLA	CAA-CBA-CGA-O1A
14	HA	1231	CLA	CAA-CBA-CGA-O1A

Continued on next page...

PRELIMINARY VALIDATION REPORT

Continued from previous page...

Mol	Chain	Res	Type	Atoms
14	GA	1138	CLA	CAA-CBA-CGA-O1A
14	aA	1138	CLA	CAA-CBA-CGA-O1A
14	aA	1133	CLA	CAA-CBA-CGA-O1A
14	AA	1133	CLA	CAA-CBA-CGA-O1A
14	BA	1213	CLA	CAA-CBA-CGA-O1A
14	GA	1131	CLA	CAA-CBA-CGA-O1A
14	GA	1133	CLA	CAA-CBA-CGA-O1A
14	UA	1501	CLA	CAA-CBA-CGA-O1A
14	bA	1213	CLA	CAA-CBA-CGA-O1A
14	AA	1131	CLA	CAA-CBA-CGA-O1A
14	HA	1213	CLA	CAA-CBA-CGA-O1A
14	aA	1131	CLA	CAA-CBA-CGA-O1A
14	HA	1235	CLA	CAA-CBA-CGA-O1A
14	HA	1222	CLA	C8-C10-C11-C12
14	bA	1222	CLA	C8-C10-C11-C12
14	BA	1222	CLA	C8-C10-C11-C12
18	MA	4021	BCR	C21-C22-C23-C24
18	aA	4007	BCR	C7-C8-C9-C10
18	AA	4007	BCR	C7-C8-C9-C10
18	mA	4021	BCR	C21-C22-C23-C24
18	GA	4007	BCR	C7-C8-C9-C10
18	VA	4021	BCR	C21-C22-C23-C24
14	LA	1501	CLA	CAA-CBA-CGA-O1A
14	lA	1501	CLA	CAA-CBA-CGA-O1A
16	AA	2001	PQN	C13-C15-C16-C17
16	GA	2001	PQN	C13-C15-C16-C17
16	aA	2001	PQN	C13-C15-C16-C17
18	BA	4012	BCR	C9-C10-C11-C12
20	LA	5007	LMG	O8-C28-C29-C30
20	UA	5007	LMG	O8-C28-C29-C30
20	lA	5007	LMG	O8-C28-C29-C30
19	AA	5001	LHG	C3-O3-P-O5
19	bA	5004	LHG	C3-O3-P-O5
19	BA	5004	LHG	C3-O3-P-O5
19	HA	5004	LHG	C3-O3-P-O5
19	GA	5001	LHG	C3-O3-P-O5
19	aA	5001	LHG	C3-O3-P-O5
14	BA	1235	CLA	CAA-CBA-CGA-O1A
14	bA	1235	CLA	CAA-CBA-CGA-O1A
14	HA	1239	CLA	CAA-CBA-CGA-O1A
14	BA	1239	CLA	CAA-CBA-CGA-O1A
14	AA	1126	CLA	CAA-CBA-CGA-O1A

Continued on next page...

PRELIMINARY VALIDATION REPORT

Continued from previous page...

Mol	Chain	Res	Type	Atoms
14	bA	1239	CLA	CAA-CBA-CGA-O1A
14	aA	1013	CLA	CAA-CBA-CGA-O2A
14	AA	1013	CLA	CAA-CBA-CGA-O2A
14	BA	1226	CLA	C5-C6-C7-C8
14	HA	1226	CLA	C5-C6-C7-C8
14	aA	1126	CLA	CAA-CBA-CGA-O1A
14	GA	1126	CLA	CAA-CBA-CGA-O1A
20	GA	5005	LMG	O10-C28-C29-C30
18	AA	4002	BCR	C23-C24-C25-C26
18	aA	4002	BCR	C23-C24-C25-C26
18	HA	4004	BCR	C1-C6-C7-C8
18	bA	4006	BCR	C5-C6-C7-C8
18	HA	4006	BCR	C5-C6-C7-C8
18	BA	4006	BCR	C5-C6-C7-C8
16	bA	2002	PQN	C23-C25-C26-C27
14	bA	1226	CLA	C5-C6-C7-C8
14	aA	1119	CLA	C16-C17-C18-C20
14	GA	1119	CLA	C16-C17-C18-C20
14	AA	1119	CLA	C16-C17-C18-C20
16	BA	2002	PQN	C23-C25-C26-C27
16	HA	2002	PQN	C23-C25-C26-C27
14	bA	1214	CLA	CAD-CBD-CGD-O1D
14	AA	1138	CLA	CAD-CBD-CGD-O1D
14	HA	1214	CLA	CAD-CBD-CGD-O1D
14	GA	1138	CLA	CAD-CBD-CGD-O1D
14	aA	1138	CLA	CAD-CBD-CGD-O1D
14	BA	1214	CLA	CAD-CBD-CGD-O1D
20	AA	5005	LMG	O10-C28-C29-C30
20	aA	5005	LMG	O10-C28-C29-C30
20	LA	5007	LMG	O10-C28-C29-C30
20	UA	5007	LMG	O10-C28-C29-C30
20	lA	5007	LMG	O10-C28-C29-C30
14	bA	1023	CLA	C5-C6-C7-C8
19	HA	5004	LHG	O7-C7-C8-C9
14	GA	1013	CLA	CAA-CBA-CGA-O2A
19	AA	5001	LHG	O10-C23-C24-C25
14	lA	1503	CLA	CAA-CBA-CGA-O1A
19	GA	5001	LHG	O10-C23-C24-C25
14	LA	1503	CLA	CAA-CBA-CGA-O1A
19	aA	5001	LHG	O10-C23-C24-C25
14	UA	1503	CLA	CAA-CBA-CGA-O1A
19	bA	5004	LHG	O7-C7-C8-C9

Continued on next page...

PRELIMINARY VALIDATION REPORT

Continued from previous page...

Mol	Chain	Res	Type	Atoms
19	BA	5004	LHG	O7-C7-C8-C9
14	GA	1013	CLA	C4-C3-C5-C6
14	aA	1013	CLA	C4-C3-C5-C6
14	AA	1013	CLA	C4-C3-C5-C6
19	bA	5004	LHG	O9-C7-C8-C9
19	BA	5004	LHG	O9-C7-C8-C9
19	HA	5004	LHG	O9-C7-C8-C9
20	LA	5007	LMG	C19-C20-C21-C22
14	HA	1023	CLA	C5-C6-C7-C8
20	UA	5007	LMG	C19-C20-C21-C22
20	lA	5007	LMG	C19-C20-C21-C22
14	BA	1023	CLA	C5-C6-C7-C8
14	AA	1127	CLA	CAA-CBA-CGA-O2A
14	GA	1127	CLA	CAA-CBA-CGA-O2A
14	aA	1127	CLA	CAA-CBA-CGA-O2A
14	BA	1204	CLA	CAA-CBA-CGA-O2A
14	bA	1204	CLA	CAA-CBA-CGA-O2A
14	BA	1226	CLA	CAA-CBA-CGA-O2A
14	HA	1204	CLA	CAA-CBA-CGA-O2A
14	aA	1103	CLA	C5-C6-C7-C8
14	bA	1223	CLA	C11-C10-C8-C7
14	BA	1223	CLA	C11-C10-C8-C7
14	HA	1202	CLA	C6-C7-C8-C10
14	bA	1202	CLA	C6-C7-C8-C10
14	HA	1223	CLA	C11-C10-C8-C7
14	BA	1202	CLA	C6-C7-C8-C10
14	GA	1103	CLA	C5-C6-C7-C8
14	AA	1103	CLA	C5-C6-C7-C8
19	aA	5001	LHG	C31-C32-C33-C34
14	BA	1204	CLA	CAA-CBA-CGA-O1A
14	bA	1204	CLA	CAA-CBA-CGA-O1A
14	AA	1127	CLA	CAA-CBA-CGA-O1A
14	GA	1127	CLA	CAA-CBA-CGA-O1A
14	HA	1204	CLA	CAA-CBA-CGA-O1A
14	aA	1127	CLA	CAA-CBA-CGA-O1A
14	AA	1122	CLA	CAA-CBA-CGA-O2A
14	bA	1226	CLA	CAA-CBA-CGA-O2A
14	aA	1122	CLA	CAA-CBA-CGA-O2A
14	HA	1226	CLA	CAA-CBA-CGA-O2A
14	GA	1122	CLA	CAA-CBA-CGA-O2A
14	BA	1238	CLA	C3A-C2A-CAA-CBA
14	bA	1238	CLA	C3A-C2A-CAA-CBA

Continued on next page...

PRELIMINARY VALIDATION REPORT

Continued from previous page...

Mol	Chain	Res	Type	Atoms
14	HA	1238	CLA	C3A-C2A-CAA-CBA
14	AA	1115	CLA	C5-C6-C7-C8
19	AA	5001	LHG	C31-C32-C33-C34
14	aA	1115	CLA	C5-C6-C7-C8
14	GA	1115	CLA	C5-C6-C7-C8
18	aA	4007	BCR	C7-C8-C9-C34
18	AA	4007	BCR	C7-C8-C9-C34
18	GA	4007	BCR	C7-C8-C9-C34
19	GA	5001	LHG	C31-C32-C33-C34
18	bA	4013	BCR	C9-C10-C11-C12
14	HA	1215	CLA	C4-C3-C5-C6
14	BA	1215	CLA	C4-C3-C5-C6
14	bA	1215	CLA	C4-C3-C5-C6
20	GA	5005	LMG	C29-C30-C31-C32
20	AA	5005	LMG	C29-C30-C31-C32
20	aA	5005	LMG	C29-C30-C31-C32

There are no ring outliers.

201 monomers are involved in 527 short contacts:

Mol	Chain	Res	Type	Clashes	Symm-Clashes
14	0	1012	CLA	6	0
13	AA	1011	CL0	1	0
14	AA	1012	CLA	4	0
14	AA	1013	CLA	5	0
14	AA	1102	CLA	2	0
14	AA	1103	CLA	5	0
14	AA	1104	CLA	3	0
14	AA	1106	CLA	2	0
14	AA	1107	CLA	1	0
14	AA	1108	CLA	1	0
14	AA	1109	CLA	4	0
14	AA	1110	CLA	1	0
14	AA	1111	CLA	2	0
14	AA	1112	CLA	1	0
14	AA	1115	CLA	1	0
14	AA	1116	CLA	3	0
14	AA	1117	CLA	3	0
14	AA	1118	CLA	1	0
14	AA	1119	CLA	6	0
14	AA	1122	CLA	1	0
14	AA	1123	CLA	3	0

Continued on next page...

PRELIMINARY VALIDATION REPORT

Continued from previous page...

Mol	Chain	Res	Type	Clashes	Symm-Clashes
14	AA	1124	CLA	5	0
14	AA	1125	CLA	3	0
14	AA	1126	CLA	8	0
14	AA	1127	CLA	3	0
14	AA	1128	CLA	2	0
14	AA	1130	CLA	3	0
14	AA	1131	CLA	4	0
14	AA	1132	CLA	4	0
14	AA	1133	CLA	5	0
14	AA	1136	CLA	6	0
14	AA	1137	CLA	1	0
14	AA	1139	CLA	1	0
14	AA	1140	CLA	8	0
16	AA	2001	PQN	7	0
18	AA	4001	BCR	3	0
18	AA	4002	BCR	1	0
18	AA	4003	BCR	6	0
18	AA	4007	BCR	4	0
18	AA	4008	BCR	2	0
18	AA	4011	BCR	6	0
19	AA	5001	LHG	1	0
20	AA	5005	LMG	1	0
14	BA	1021	CLA	6	0
14	BA	1022	CLA	6	0
14	BA	1023	CLA	3	0
14	BA	1201	CLA	1	0
14	BA	1202	CLA	7	0
14	BA	1203	CLA	5	0
14	BA	1204	CLA	6	0
14	BA	1205	CLA	6	0
14	BA	1206	CLA	2	0
14	BA	1207	CLA	2	0
14	BA	1208	CLA	3	0
14	BA	1209	CLA	1	0
14	BA	1210	CLA	7	0
14	BA	1211	CLA	3	0
14	BA	1212	CLA	1	0
14	BA	1213	CLA	1	0
14	BA	1214	CLA	4	0
14	BA	1215	CLA	8	0
14	BA	1216	CLA	2	0
14	BA	1217	CLA	2	0

Continued on next page...

PRELIMINARY VALIDATION REPORT

Continued from previous page...

Mol	Chain	Res	Type	Clashes	Symm-Clashes
14	BA	1219	CLA	4	0
14	BA	1220	CLA	1	0
14	BA	1221	CLA	3	0
14	BA	1222	CLA	5	0
14	BA	1223	CLA	4	0
14	BA	1224	CLA	7	0
14	BA	1225	CLA	4	0
14	BA	1226	CLA	3	0
14	BA	1227	CLA	1	0
14	BA	1229	CLA	3	0
14	BA	1231	CLA	2	0
14	BA	1232	CLA	3	0
14	BA	1234	CLA	2	0
14	BA	1235	CLA	2	0
14	BA	1236	CLA	2	0
14	BA	1238	CLA	8	0
14	BA	1239	CLA	5	0
16	BA	2002	PQN	4	0
18	BA	4004	BCR	1	0
18	BA	4005	BCR	4	0
18	BA	4006	BCR	4	0
18	BA	4009	BCR	7	0
18	BA	4010	BCR	7	0
18	BA	4012	BCR	5	0
18	BA	4014	BCR	3	0
18	BA	4017	BCR	1	0
20	BA	5002	LMG	3	0
13	GA	1011	CL0	2	0
14	GA	1012	CLA	5	0
14	GA	1013	CLA	4	0
14	GA	1102	CLA	1	0
14	GA	1103	CLA	6	0
14	GA	1104	CLA	2	0
14	GA	1106	CLA	2	0
14	GA	1107	CLA	1	0
14	GA	1108	CLA	1	0
14	GA	1109	CLA	2	0
14	GA	1110	CLA	1	0
14	GA	1111	CLA	3	0
14	GA	1112	CLA	1	0
14	GA	1116	CLA	3	0
14	GA	1117	CLA	4	0

Continued on next page...

PRELIMINARY VALIDATION REPORT

Continued from previous page...

Mol	Chain	Res	Type	Clashes	Symm-Clashes
14	GA	1118	CLA	1	0
14	GA	1119	CLA	4	0
14	GA	1122	CLA	1	0
14	GA	1123	CLA	3	0
14	GA	1124	CLA	5	0
14	GA	1125	CLA	3	0
14	GA	1126	CLA	5	0
14	GA	1127	CLA	3	0
14	GA	1128	CLA	2	0
14	GA	1130	CLA	3	0
14	GA	1131	CLA	1	0
14	GA	1132	CLA	3	0
14	GA	1133	CLA	5	0
14	GA	1136	CLA	4	0
14	GA	1137	CLA	1	0
14	GA	1138	CLA	1	0
14	GA	1140	CLA	7	0
16	GA	2001	PQN	6	0
18	GA	4002	BCR	1	0
18	GA	4003	BCR	6	0
18	GA	4007	BCR	3	0
18	GA	4008	BCR	1	0
18	GA	4011	BCR	6	0
19	GA	5001	LHG	1	0
20	GA	5005	LMG	1	0
21	GA	6001	LMT	1	0
14	HA	1021	CLA	5	0
14	HA	1023	CLA	3	0
14	HA	1201	CLA	1	0
14	HA	1202	CLA	8	0
14	HA	1203	CLA	3	0
14	HA	1204	CLA	5	0
14	HA	1205	CLA	6	0
14	HA	1206	CLA	2	0
14	HA	1207	CLA	1	0
14	HA	1208	CLA	3	0
14	HA	1209	CLA	1	0
14	HA	1210	CLA	3	0
14	HA	1211	CLA	3	0
14	HA	1212	CLA	1	0
14	HA	1214	CLA	3	0
14	HA	1215	CLA	7	0

Continued on next page...

PRELIMINARY VALIDATION REPORT

Continued from previous page...

Mol	Chain	Res	Type	Clashes	Symm-Clashes
14	HA	1216	CLA	2	0
14	HA	1217	CLA	2	0
14	HA	1219	CLA	4	0
14	HA	1220	CLA	1	0
14	HA	1221	CLA	2	0
14	HA	1222	CLA	4	0
14	HA	1223	CLA	5	0
14	HA	1224	CLA	7	0
14	HA	1225	CLA	4	0
14	HA	1226	CLA	4	0
14	HA	1227	CLA	1	0
14	HA	1229	CLA	3	0
14	HA	1231	CLA	1	0
14	HA	1232	CLA	3	0
14	HA	1234	CLA	2	0
14	HA	1235	CLA	3	0
14	HA	1236	CLA	2	0
14	HA	1238	CLA	7	0
14	HA	1239	CLA	4	0
16	HA	2002	PQN	4	0
18	HA	4004	BCR	1	0
18	HA	4005	BCR	4	0
18	HA	4006	BCR	3	0
18	HA	4009	BCR	7	0
18	HA	4010	BCR	7	0
18	HA	4012	BCR	4	0
18	HA	4016	BCR	1	0
18	HA	4017	BCR	1	0
20	HA	5002	LMG	3	0
19	HA	5004	LHG	1	0
18	IA	4018	BCR	6	0
18	IA	4020	BCR	5	0
20	IA	5006	LMG	4	0
14	KA	1401	CLA	1	0
14	LA	1501	CLA	5	0
14	LA	1502	CLA	5	0
14	LA	1503	CLA	7	0
18	LA	4019	BCR	2	0
18	LA	4022	BCR	6	0
20	LA	5007	LMG	4	0
18	MA	4021	BCR	3	0
18	RA	4018	BCR	4	0

Continued on next page...

PRELIMINARY VALIDATION REPORT

Continued from previous page...

Mol	Chain	Res	Type	Clashes	Symm-Clashes
18	RA	4020	BCR	4	0
20	RA	5006	LMG	5	0
14	TA	1401	CLA	1	0
14	UA	1501	CLA	5	0
14	UA	1502	CLA	5	0
14	UA	1503	CLA	5	0
18	UA	4019	BCR	1	0
18	UA	4022	BCR	5	0
20	UA	5007	LMG	3	0
18	VA	4021	BCR	4	0
14	WA	1701	CLA	2	0
14	XA	1701	CLA	1	0

The following is a two-dimensional graphical depiction of Mogul quality analysis of bond lengths, bond angles, torsion angles, and ring geometry for all instances of the Ligand of Interest. In addition, ligands with molecular weight > 250 and outliers as shown on the validation Tables will also be included. For torsion angles, if less then 5% of the Mogul distribution of torsion angles is within 10 degrees of the torsion angle in question, then that torsion angle is considered an outlier. Any bond that is central to one or more torsion angles identified as an outlier by Mogul will be highlighted in the graph. For rings, the root-mean-square deviation (RMSD) between the ring in question and similar rings identified by Mogul is calculated over all ring torsion angles. If the average RMSD is greater than 60 degrees and the minimal RMSD between the ring in question and any Mogul-identified rings is also greater than 60 degrees, then that ring is considered an outlier. The outliers are highlighted in purple. The color gray indicates Mogul did not find sufficient equivalents in the CSD to analyse the geometry.

Ligand CLA 0 1012

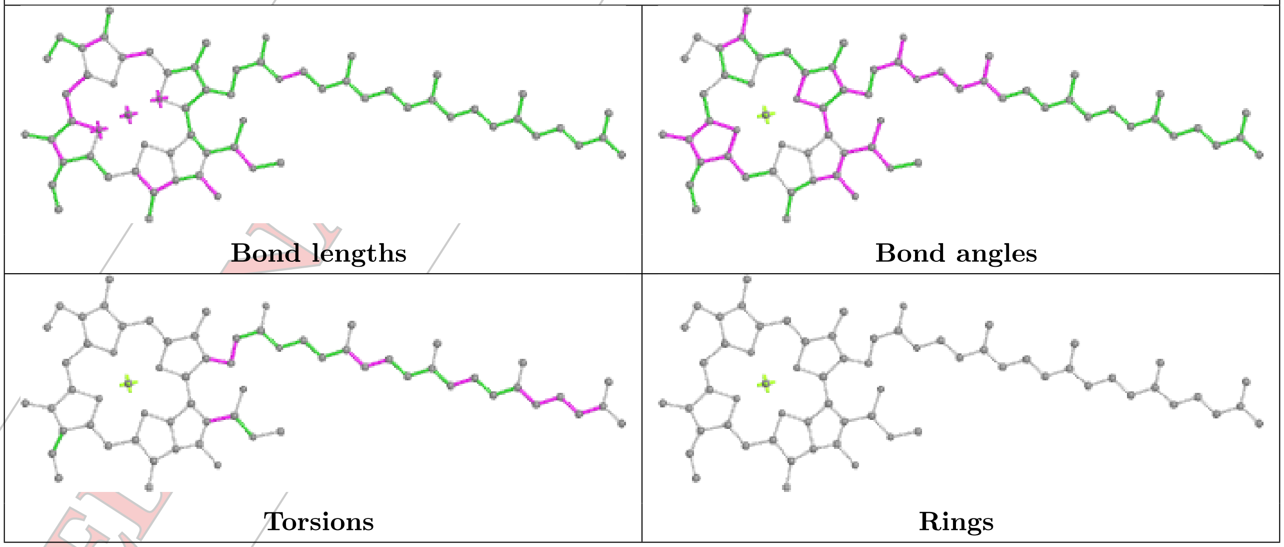

PRELIMINARY VALIDATION REPORT

Ligand CL0 AA 1011

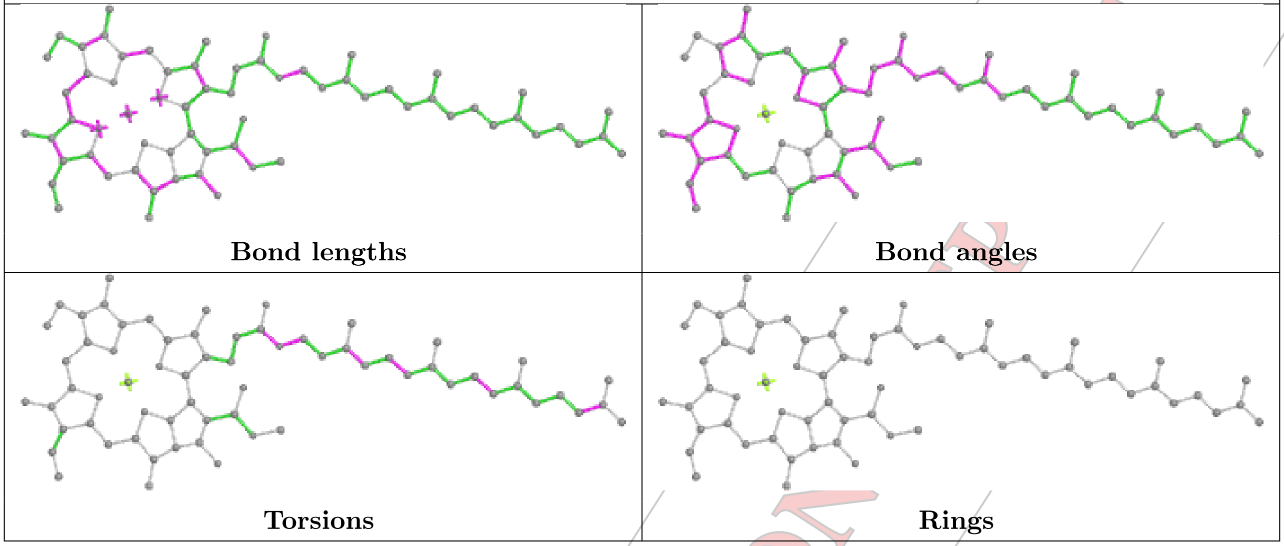

Ligand CLA AA 1012

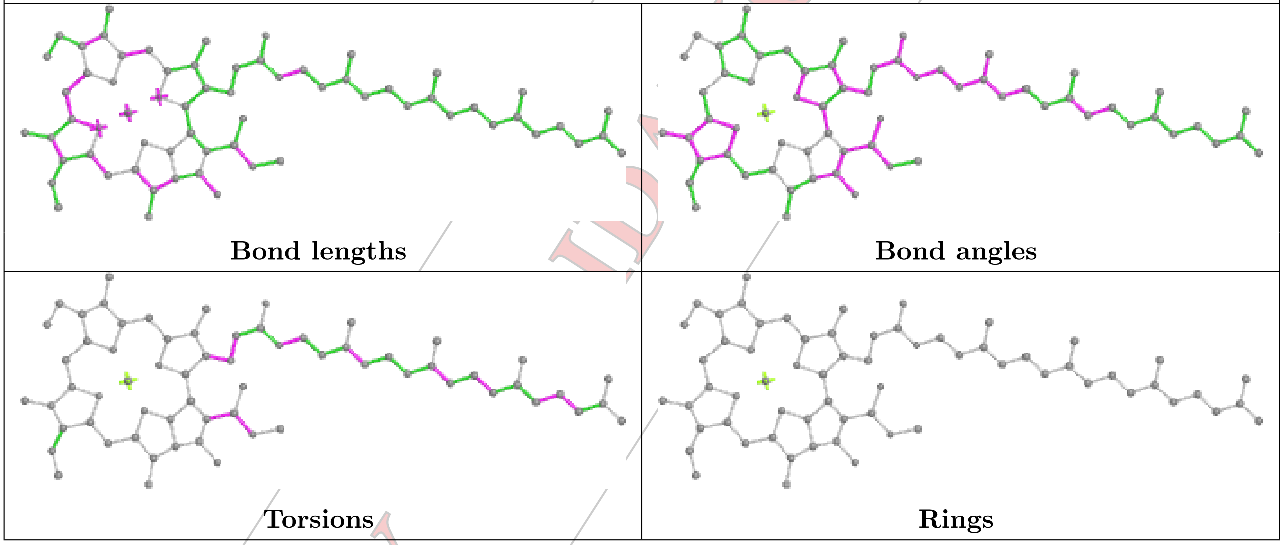

PRELIMINARY

Ligand CLA AA 1013

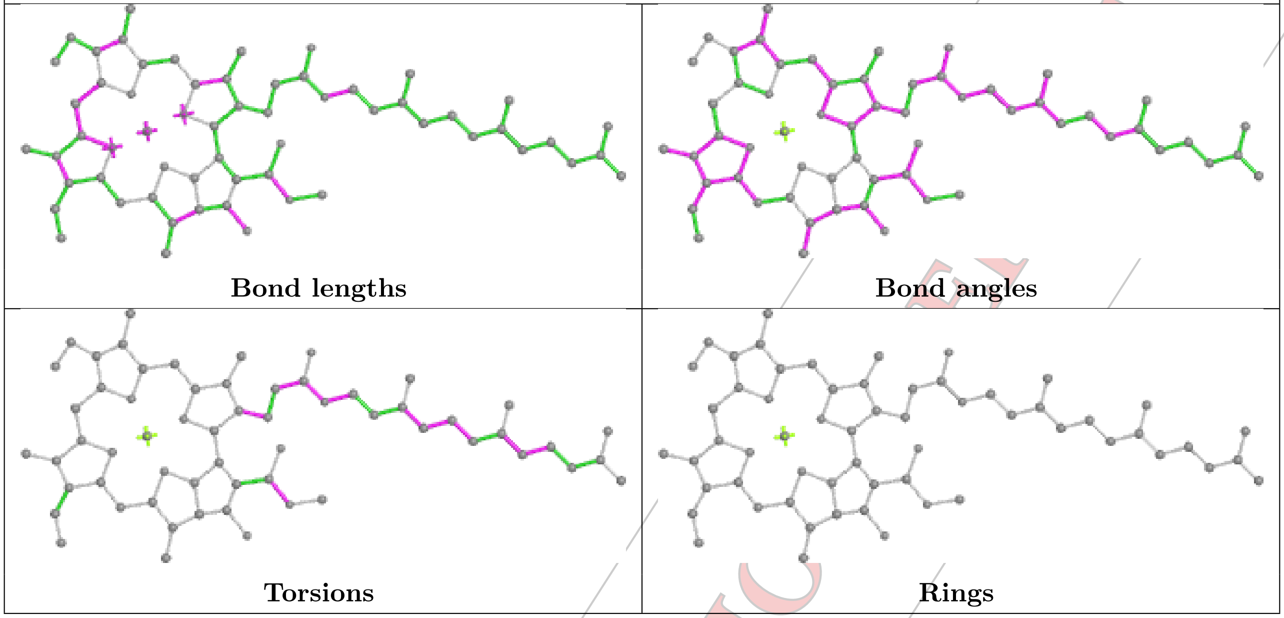

Ligand CLA AA 1101

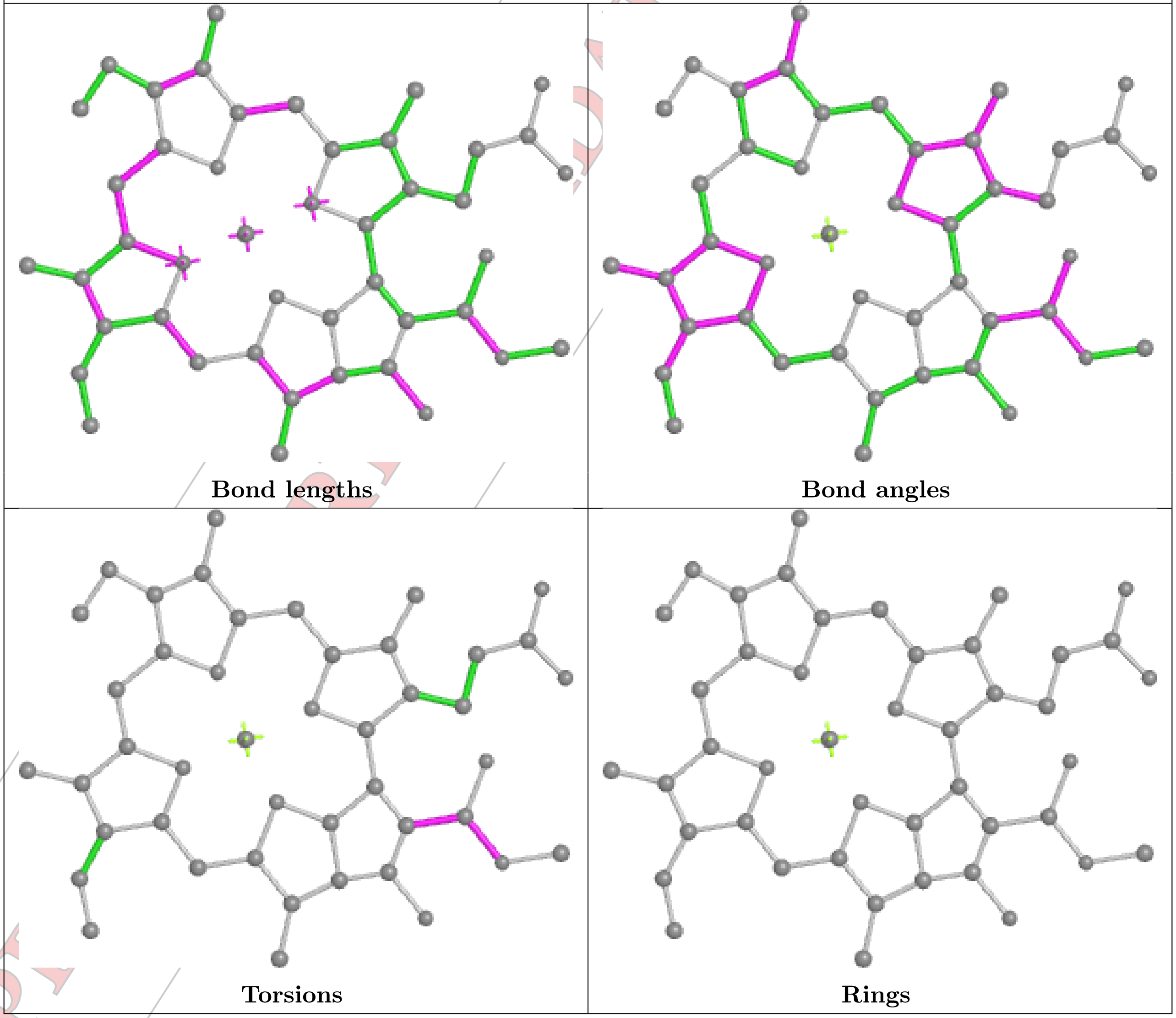

Ligand CLA AA 1102

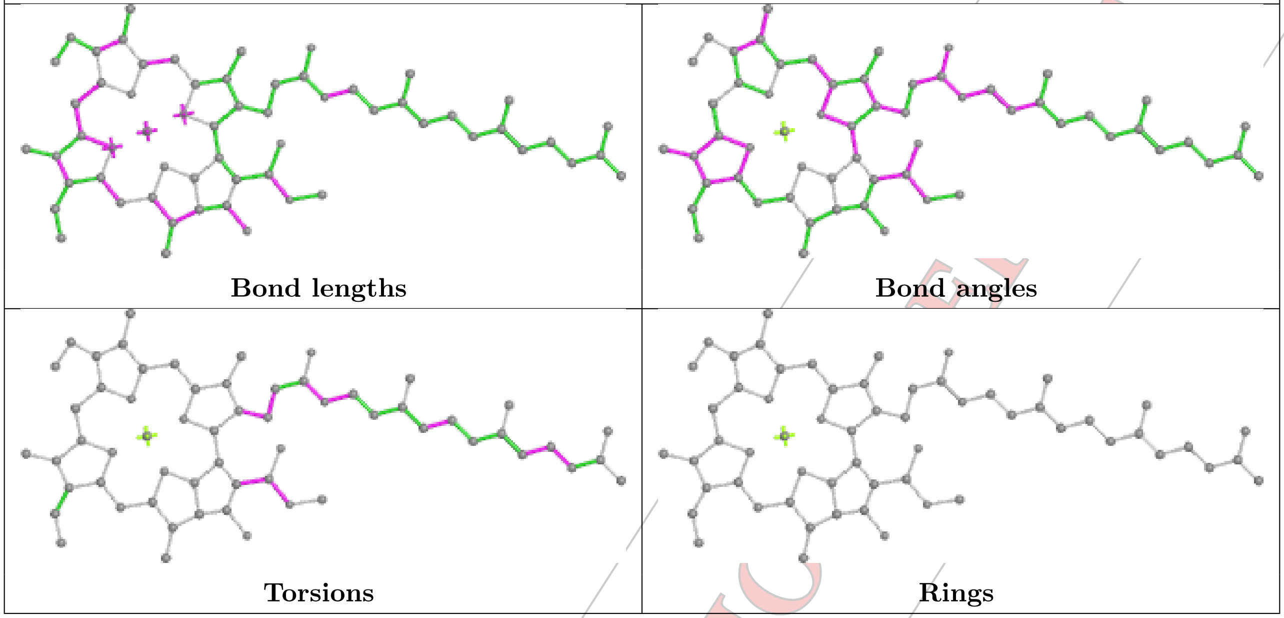

Ligand CLA AA 1103

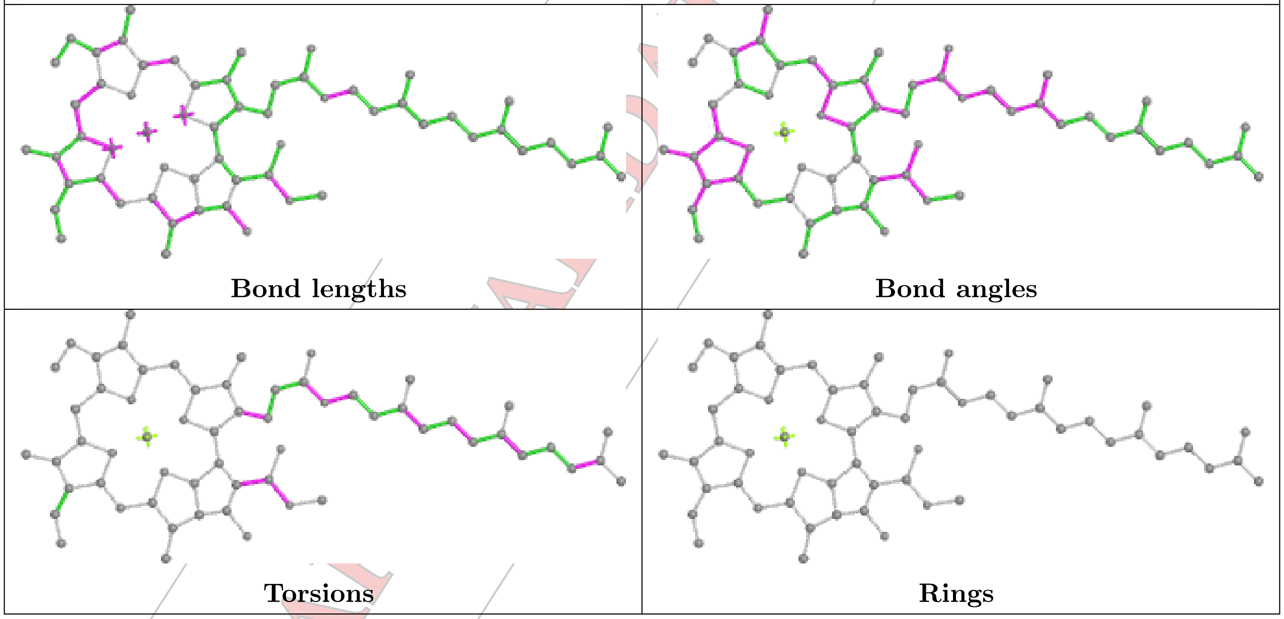

PRELIMINARY

Ligand CLA AA 1104

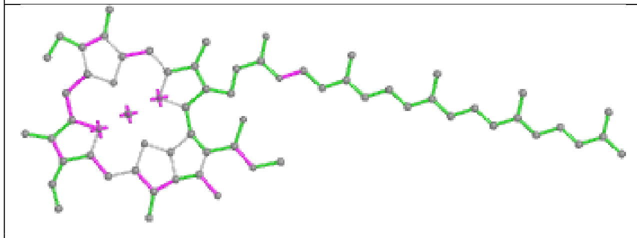

Bond lengths

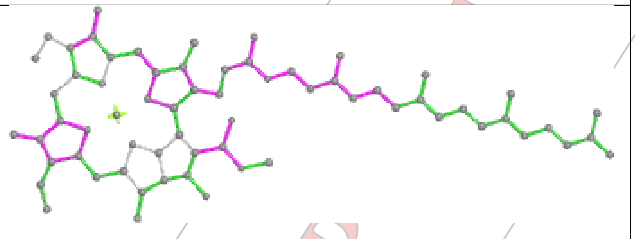

Bond angles

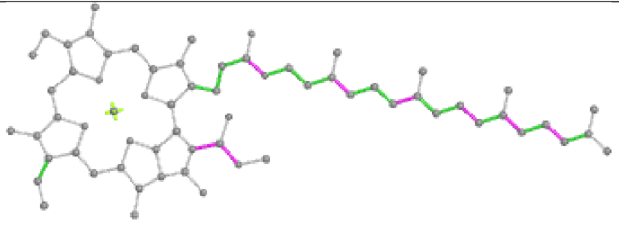

Torsions

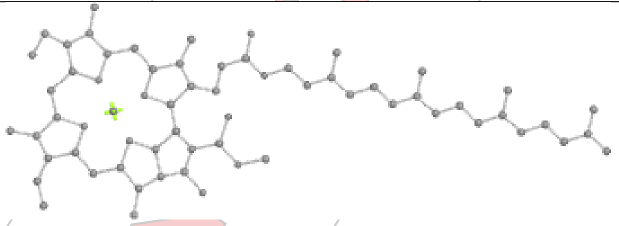

Rings

Ligand CLA AA 1105

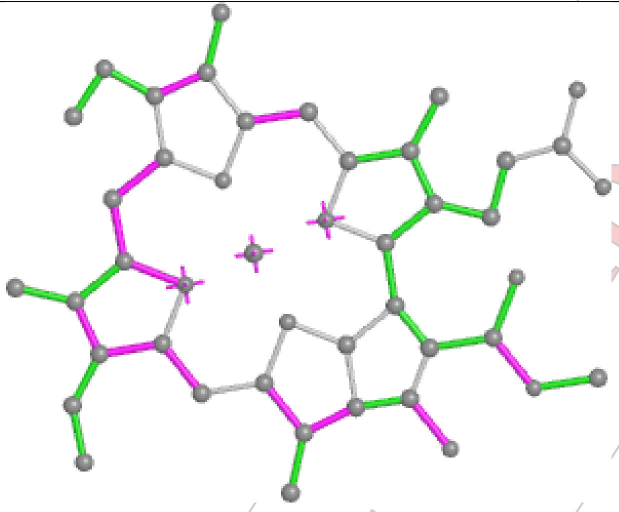

Bond lengths

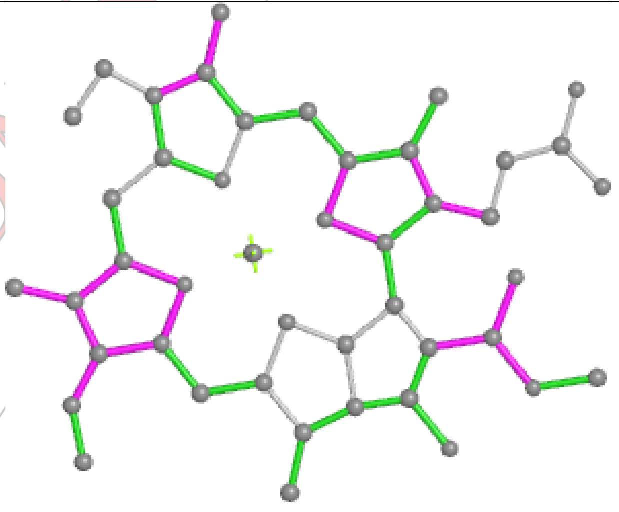

Bond angles

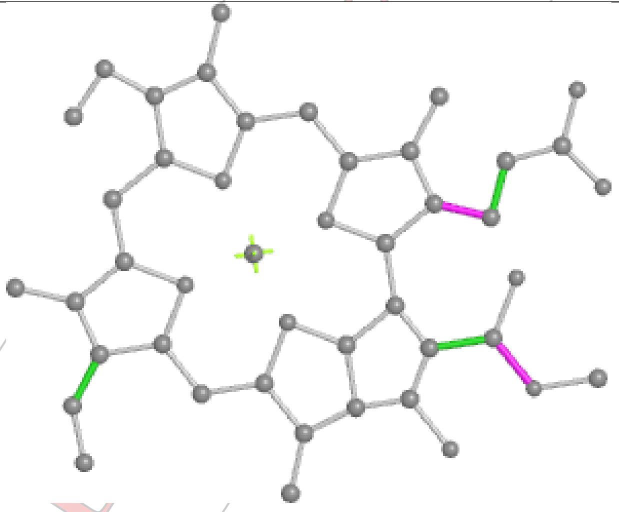

Torsions

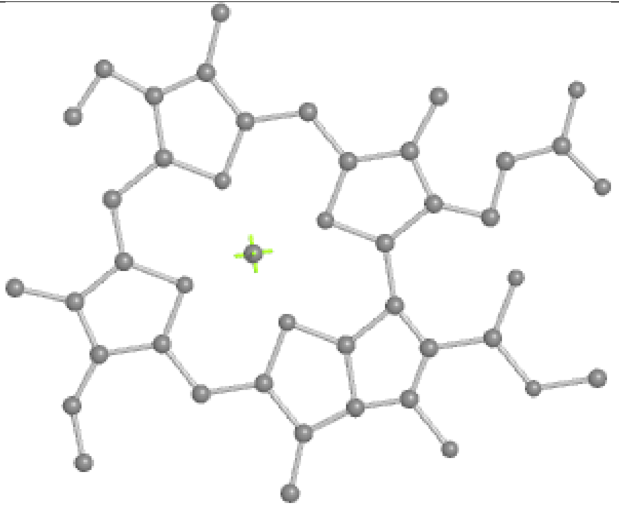

Rings

Ligand CLA AA 1106

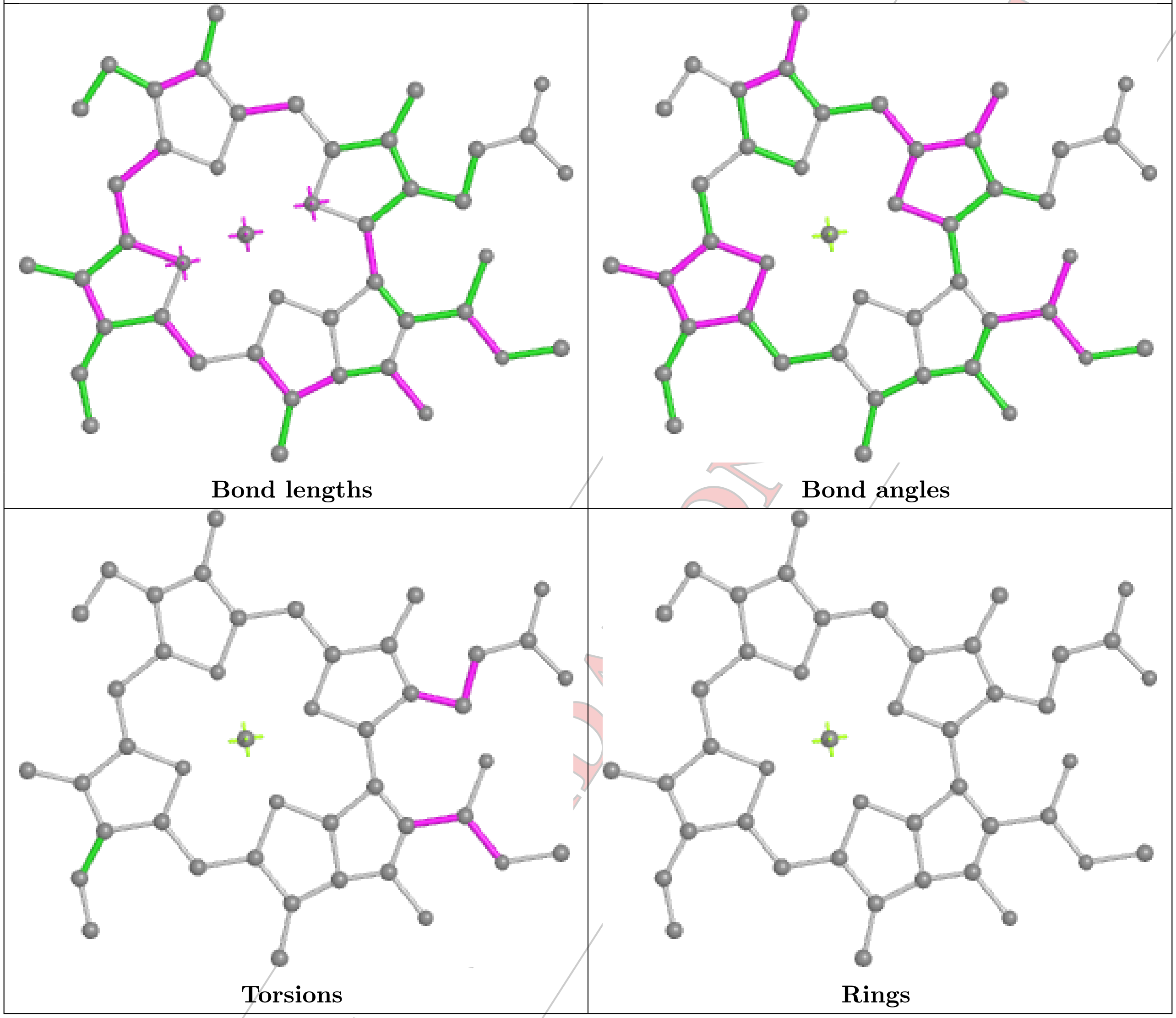

PRELIMINARY

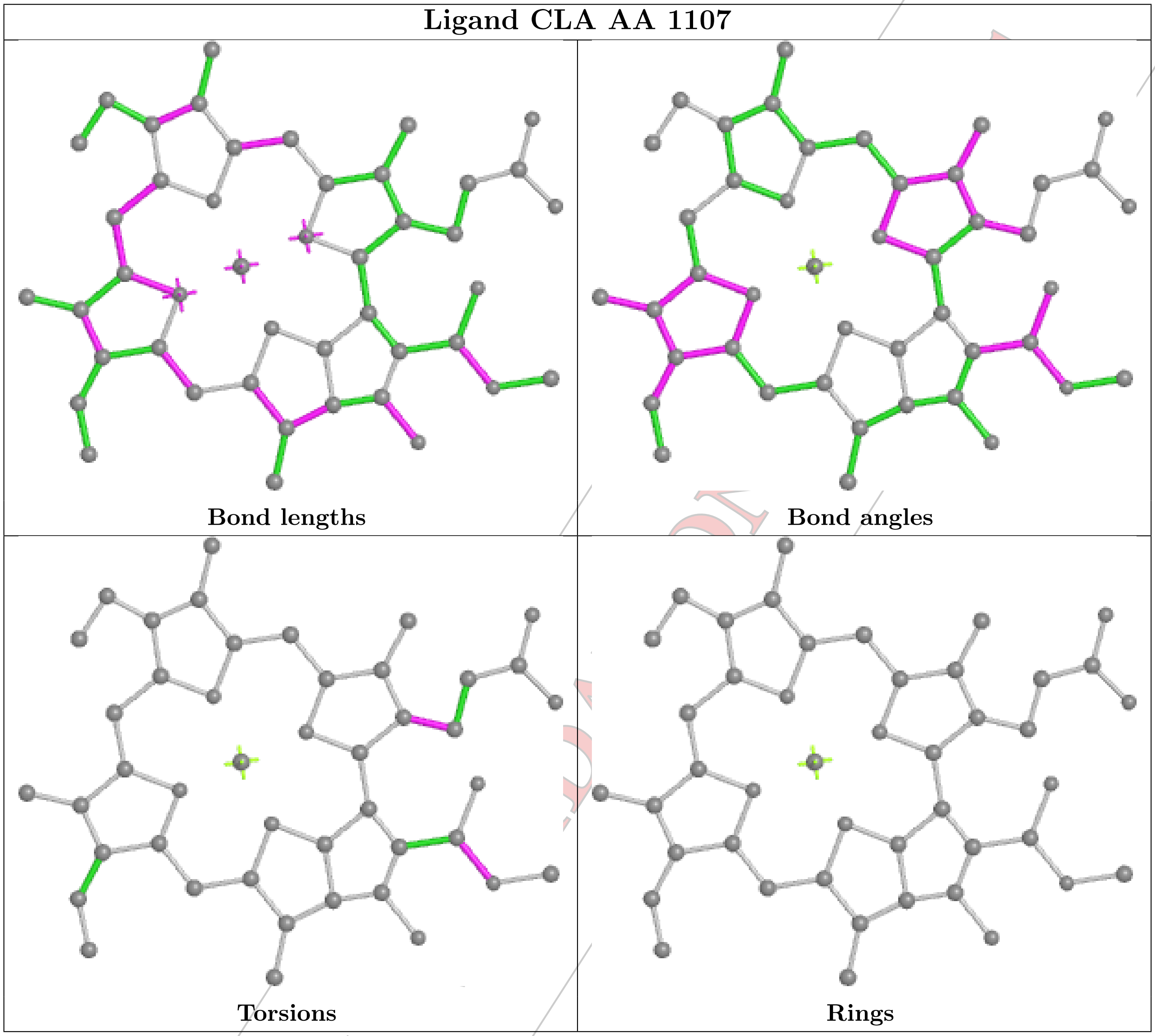

PRELIMINARY

Ligand CLA AA 1108

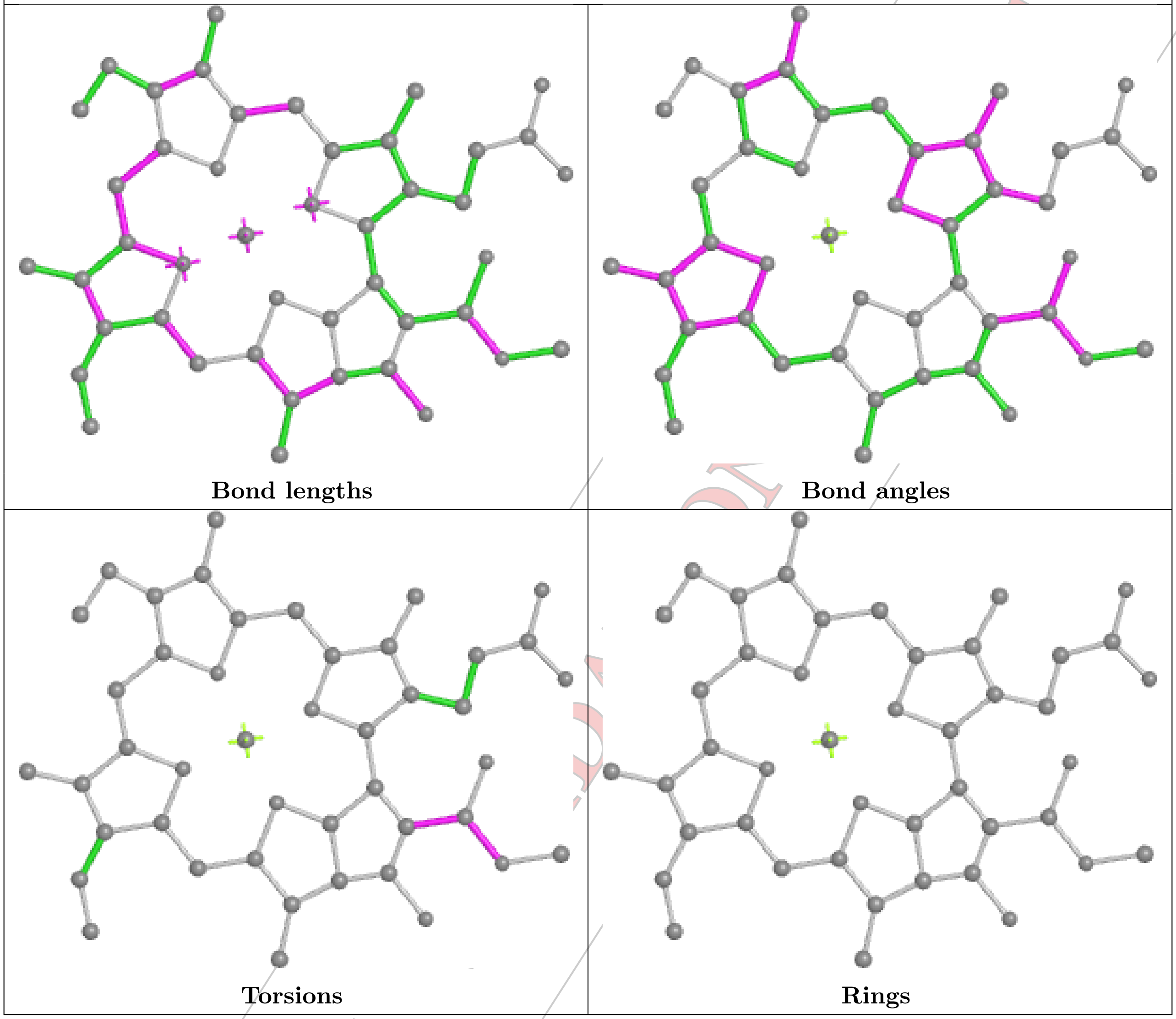

PRELIMINARY

Ligand CLA AA 1109

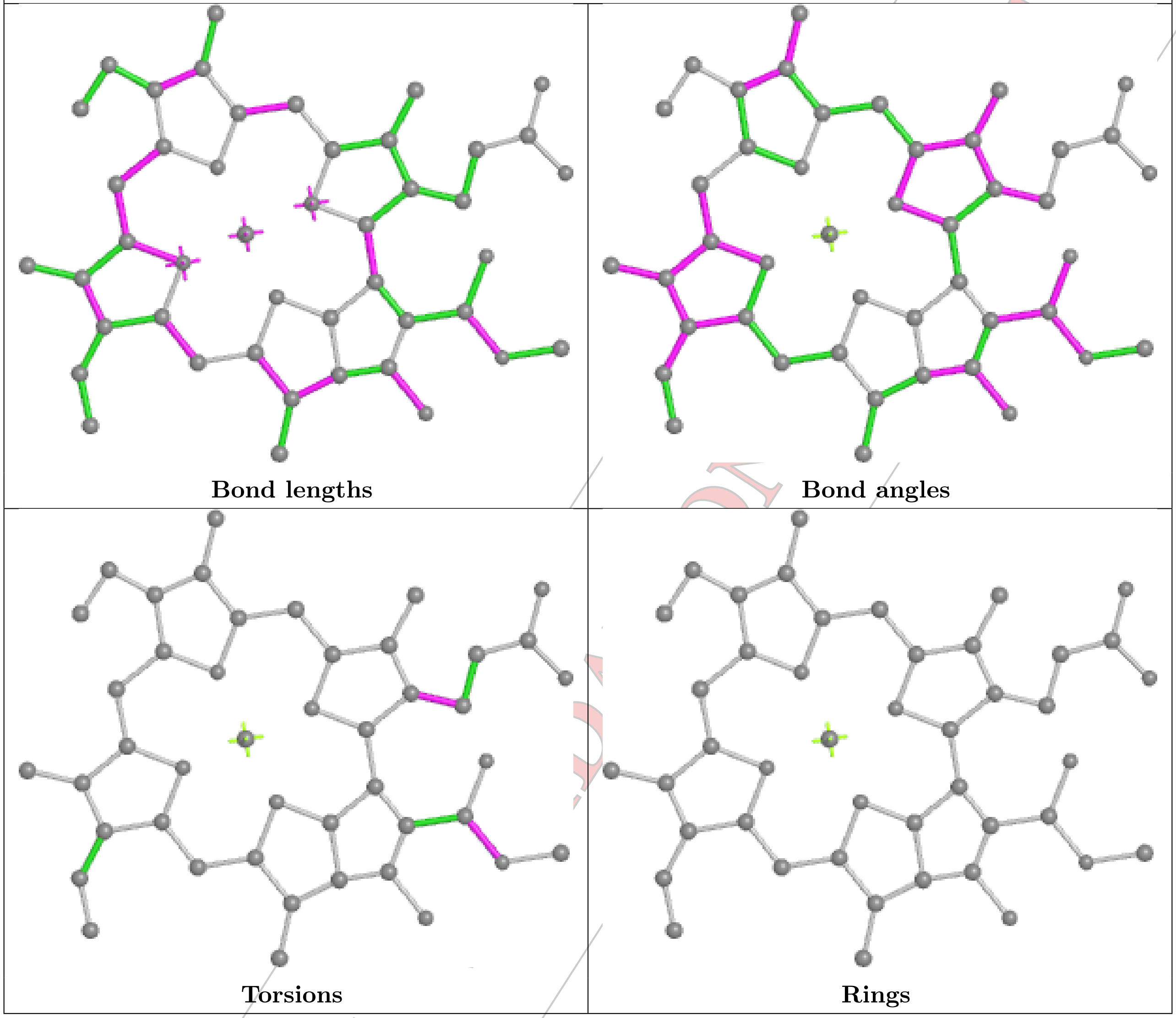

PRELIMINARY

Ligand CLA AA 1110

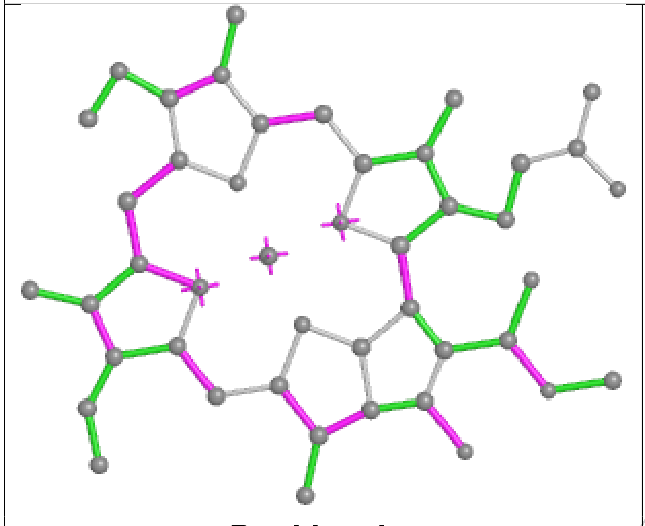

Bond lengths

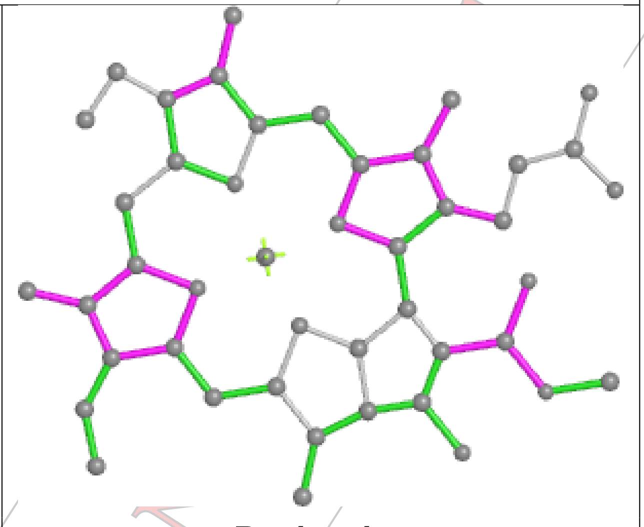

Bond angles

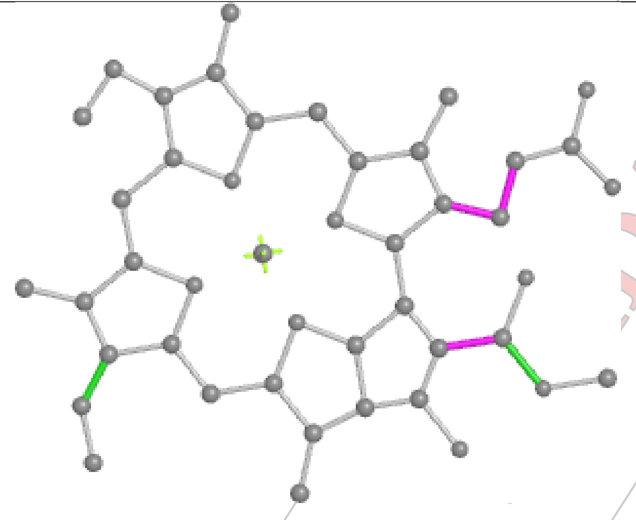

Torsions

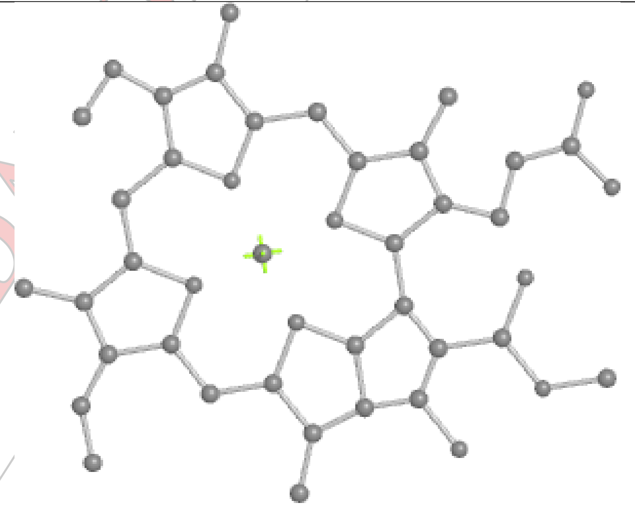

Rings

Ligand CLA AA 1111

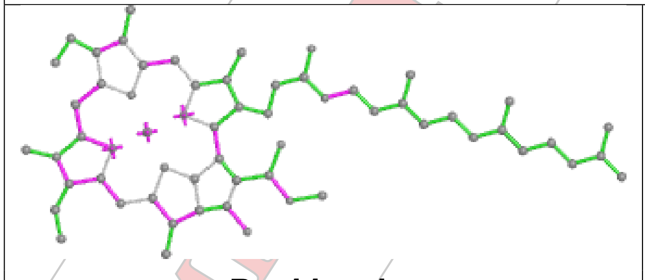

Bond lengths

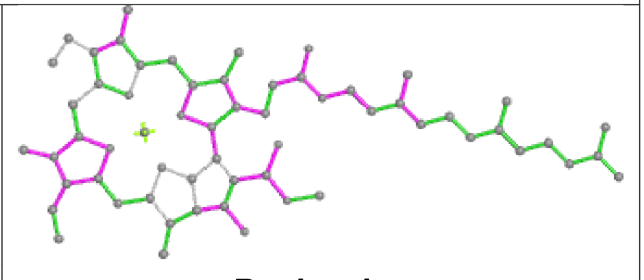

Bond angles

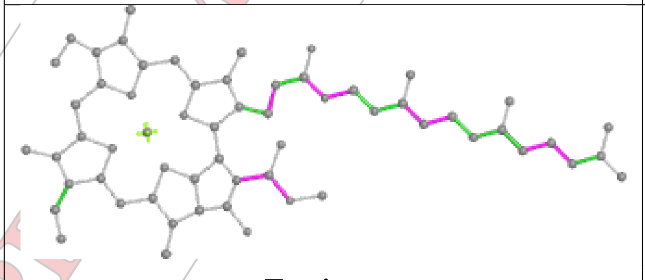

Torsions

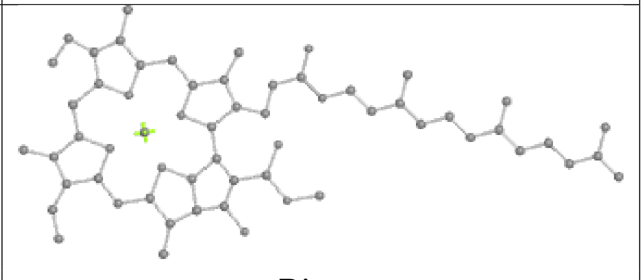

Rings

Ligand CLA AA 1112

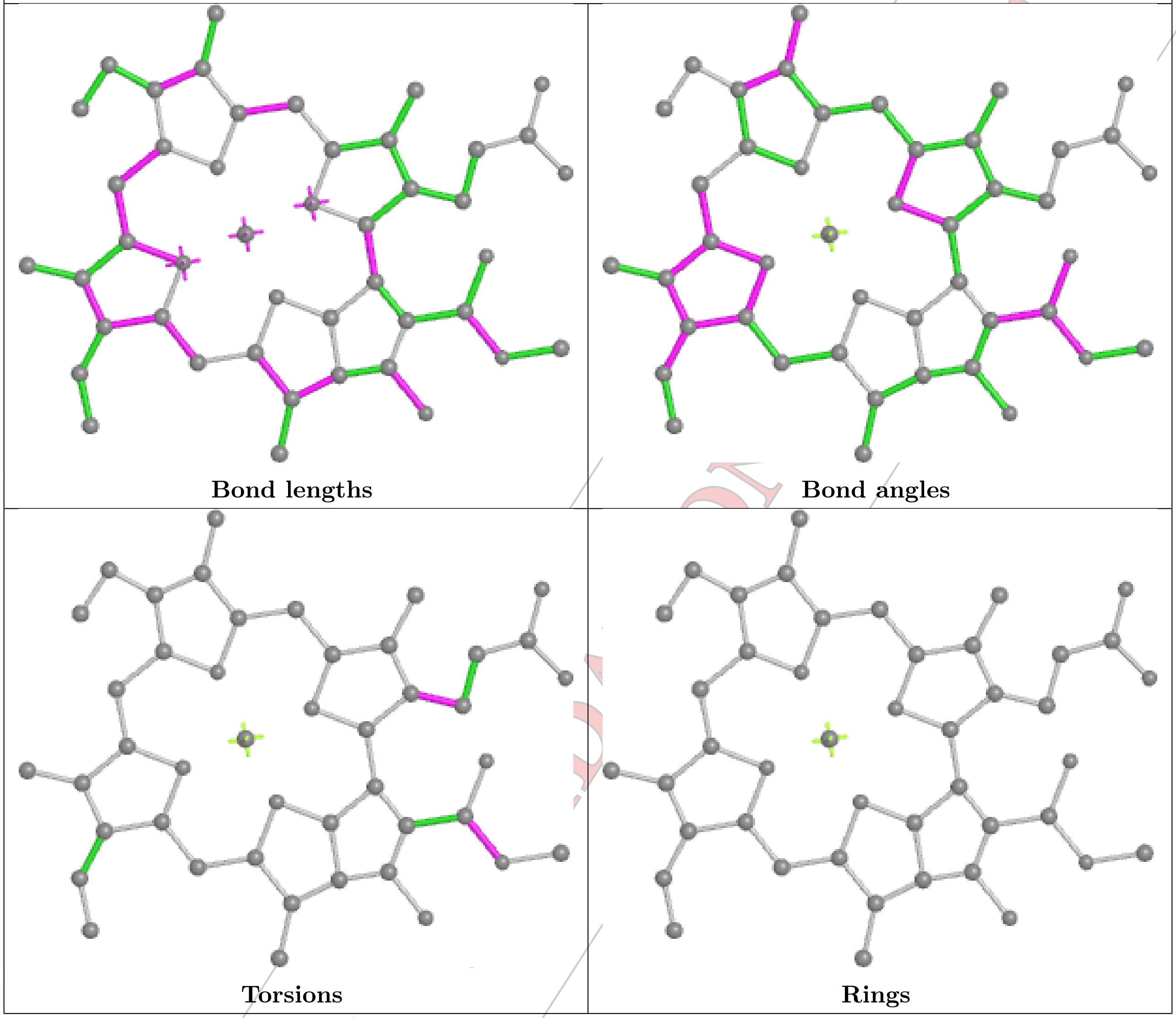

PRELIMINARY

Ligand CLA AA 1113

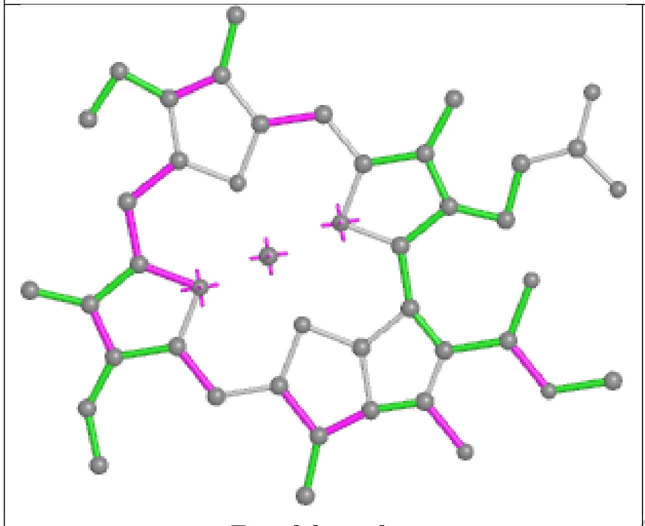

Bond lengths

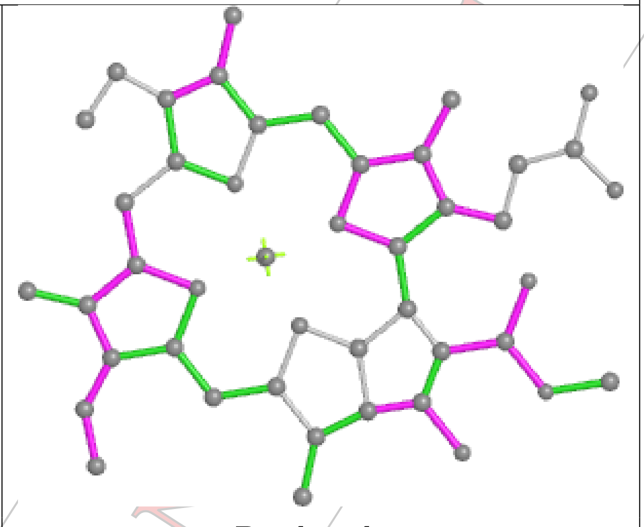

Bond angles

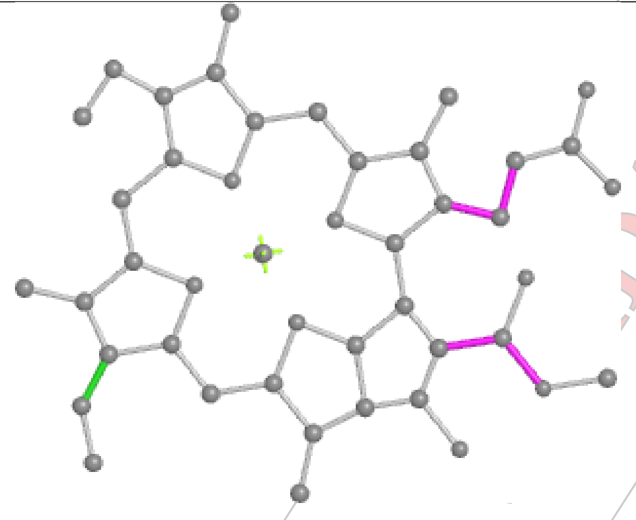

Torsions

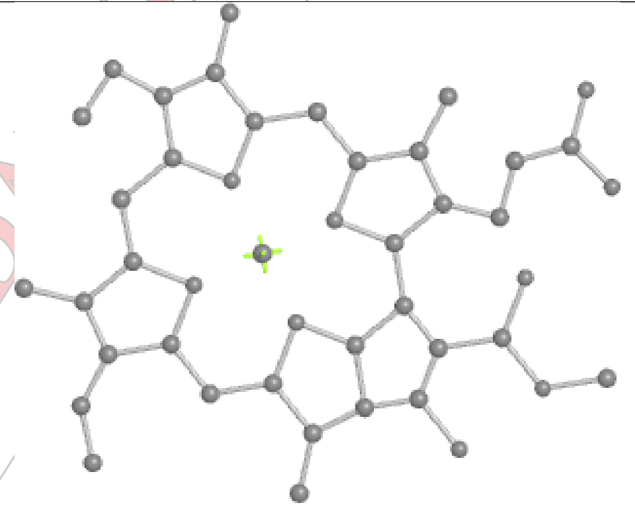

Rings

PRELIMINARY

Ligand CLA AA 1114

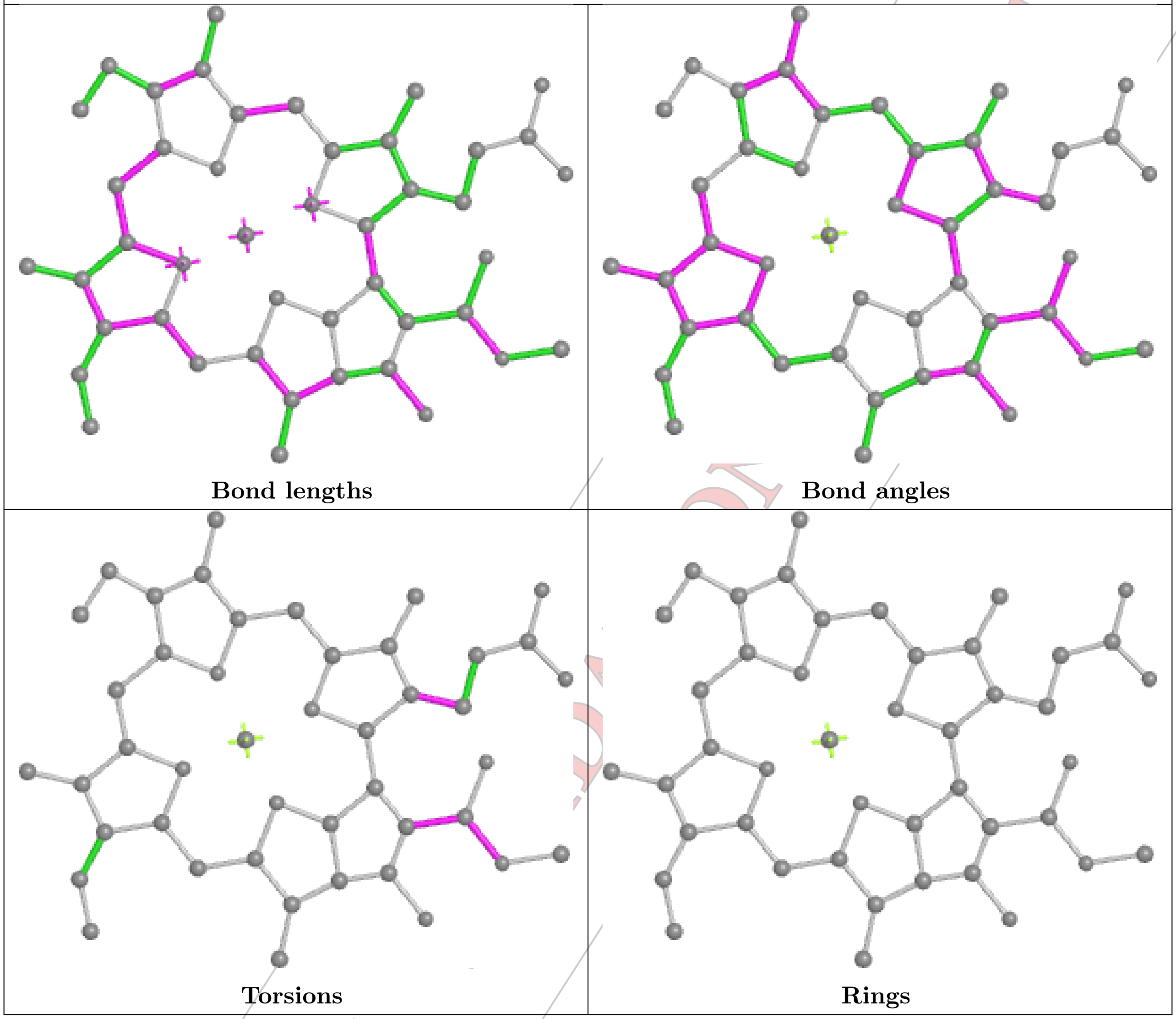

PRELIMINARY

Ligand CLA AA 1115

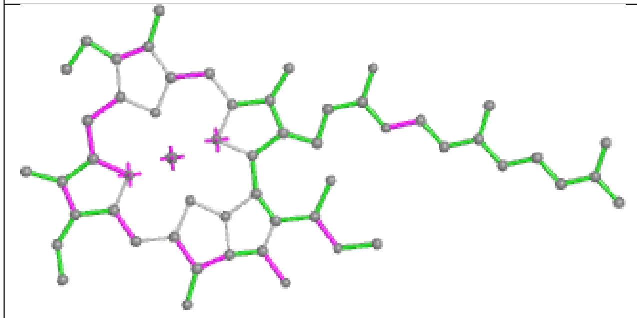

Bond lengths

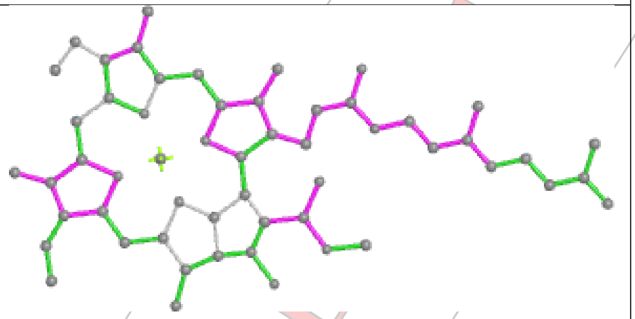

Bond angles

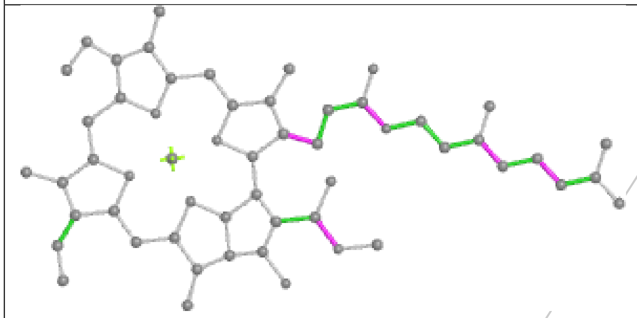

Torsions

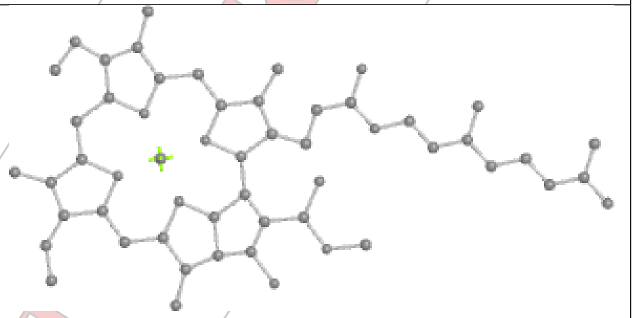

Rings

Ligand CLA AA 1116

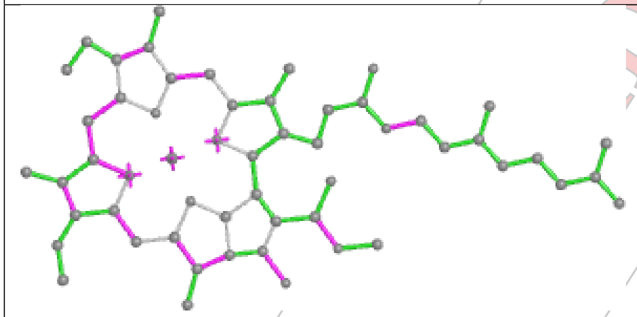

Bond lengths

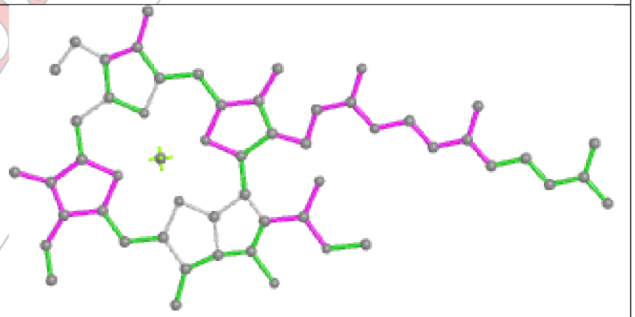

Bond angles

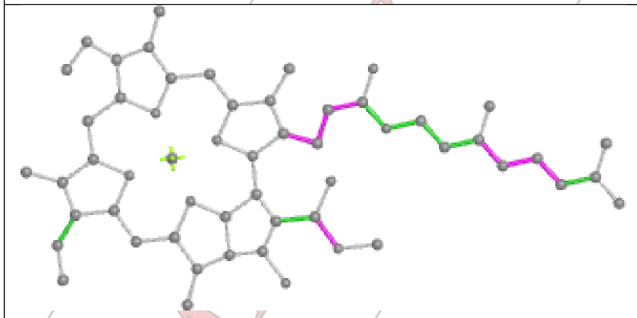

Torsions

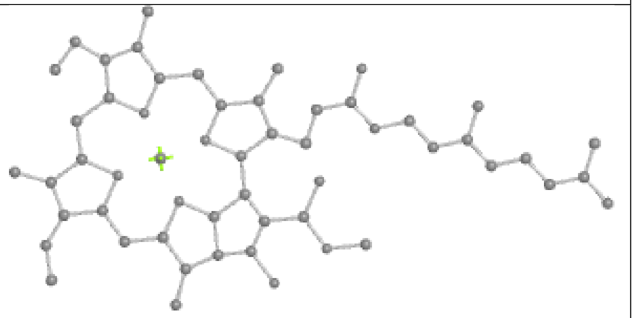

Rings

Ligand CLA AA 1117

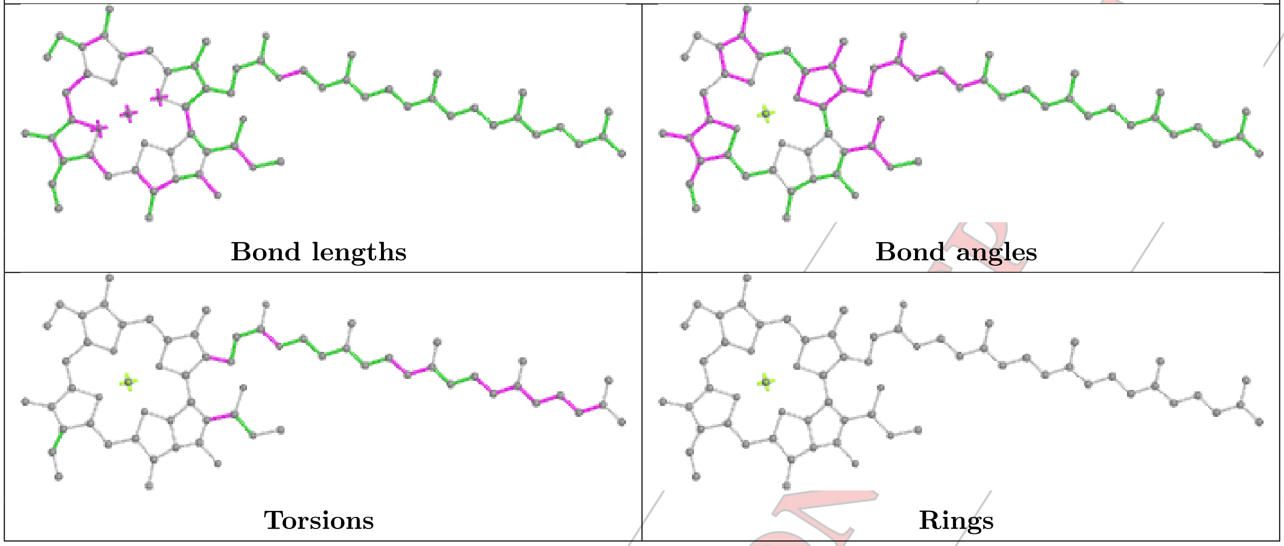

Ligand CLA AA 1118

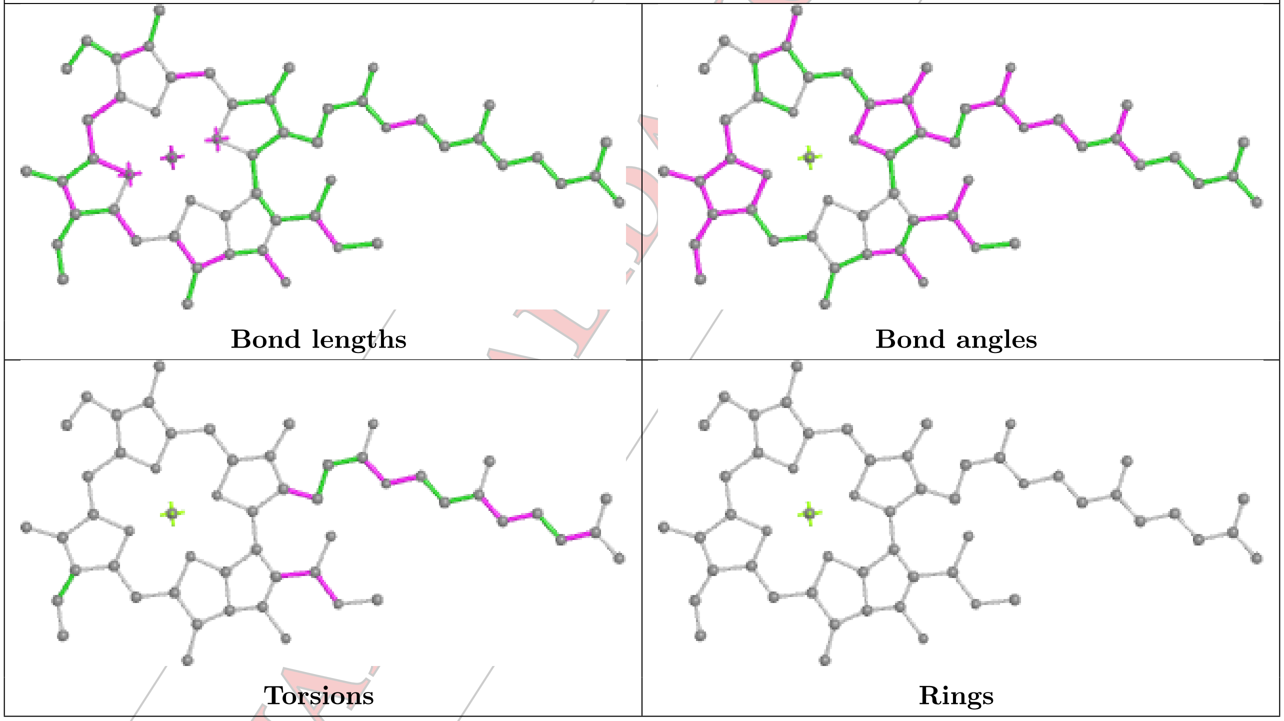

PRELIMINARY

Ligand CLA AA 1119

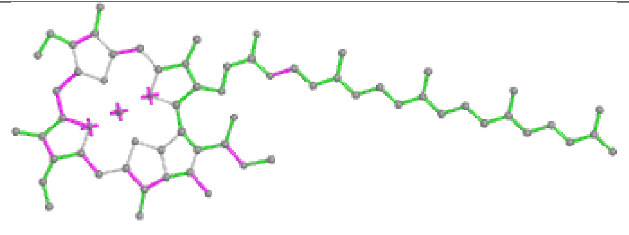

Bond lengths

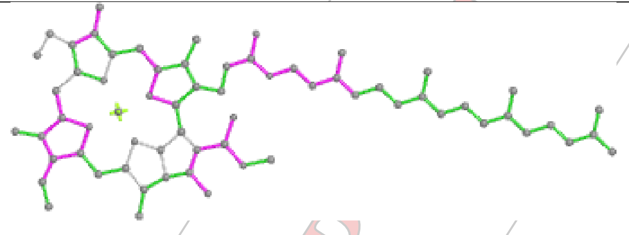

Bond angles

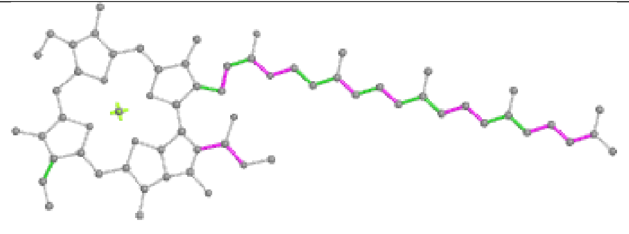

Torsions

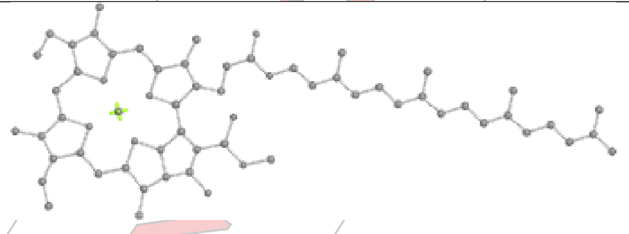

Rings

Ligand CLA AA 1122

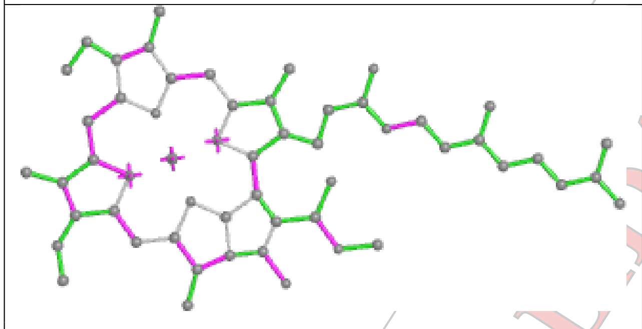

Bond lengths

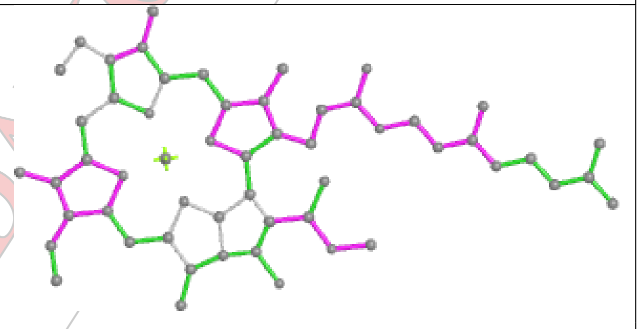

Bond angles

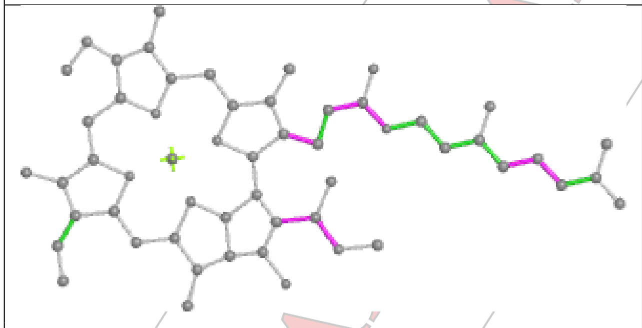

Torsions

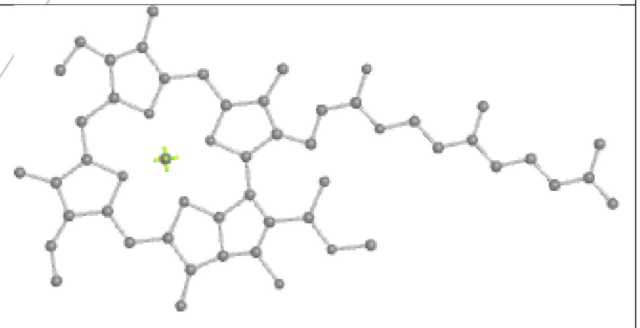

Rings

PRELIMINARY

Ligand CLA AA 1123

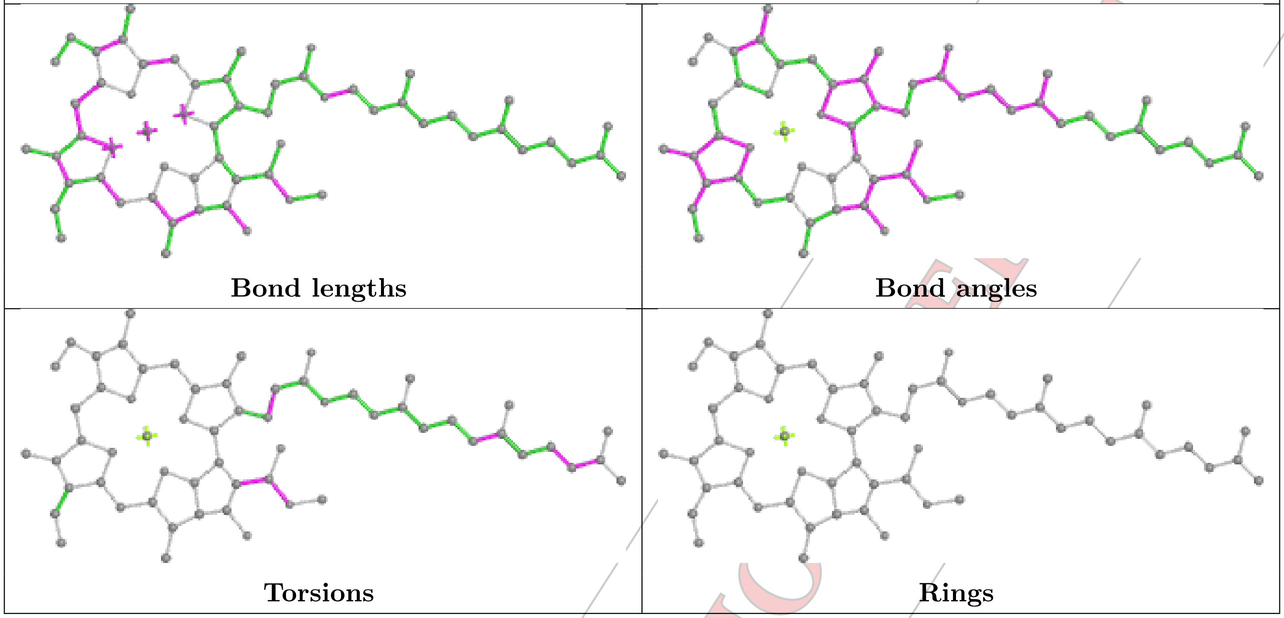

Ligand CLA AA 1124

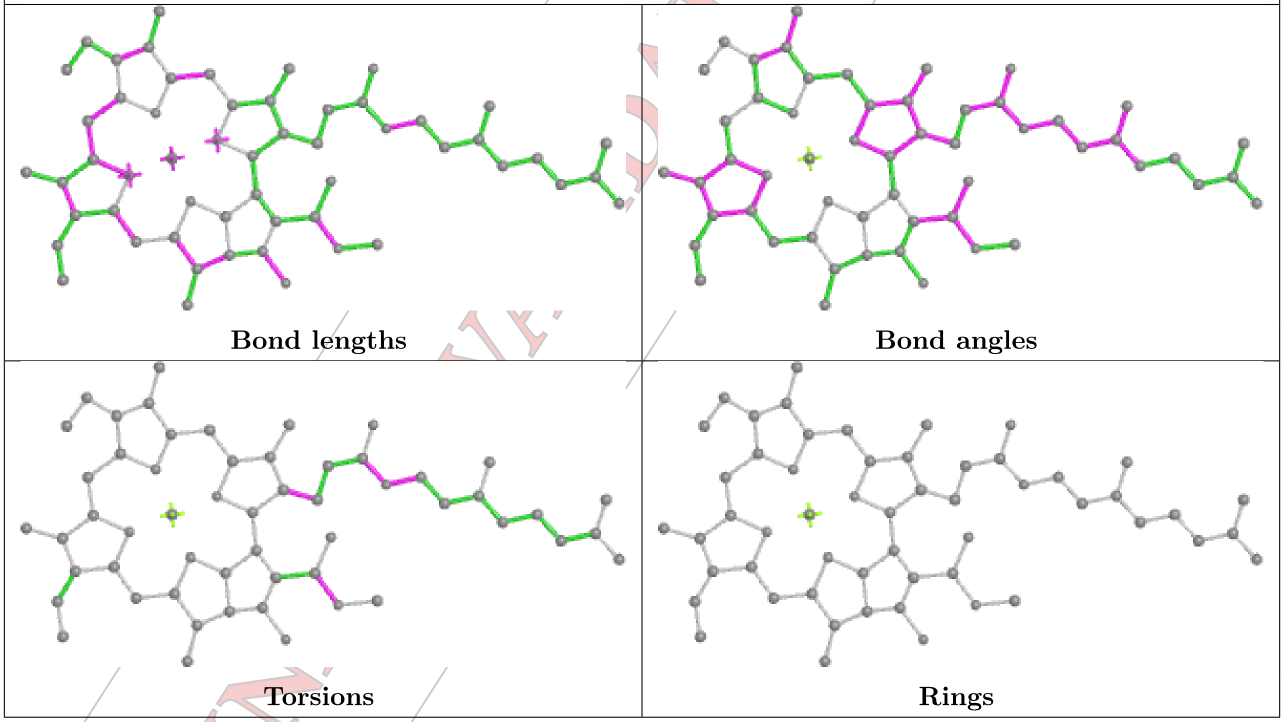

Ligand CLA AA 1125

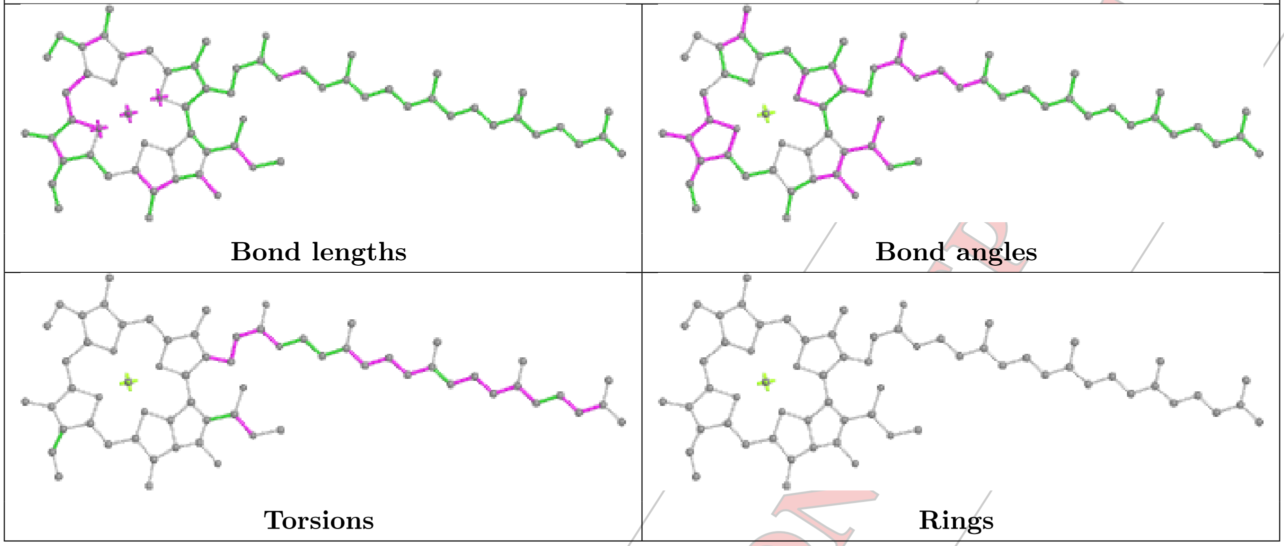

Ligand CLA AA 1126

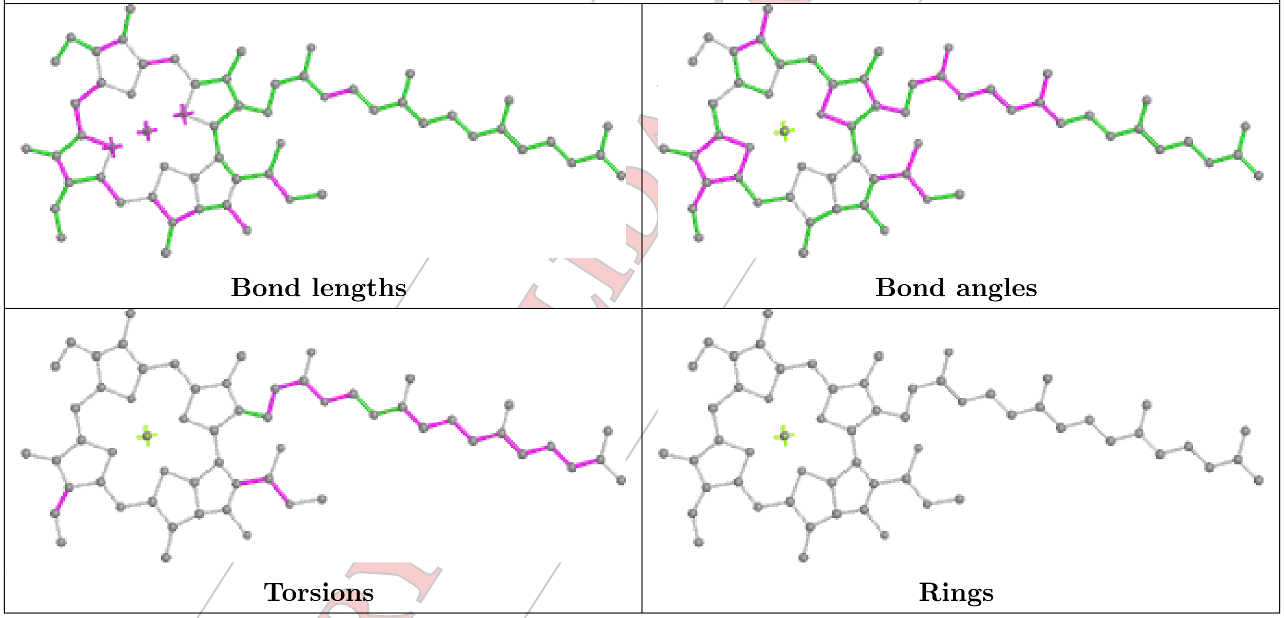

PRELIMINARY

Ligand CLA AA 1127

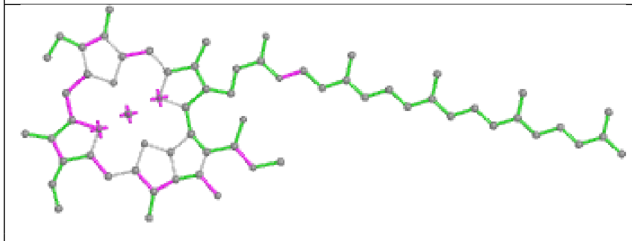 Bond lengths	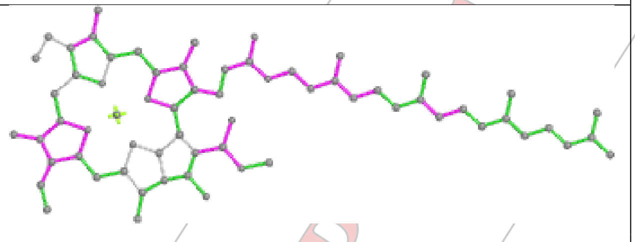 Bond angles
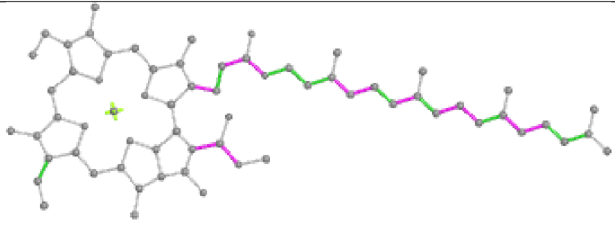 Torsions	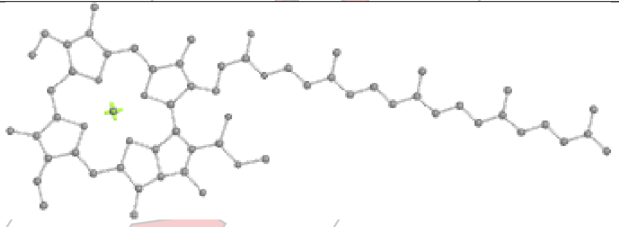 Rings

Ligand CLA AA 1128

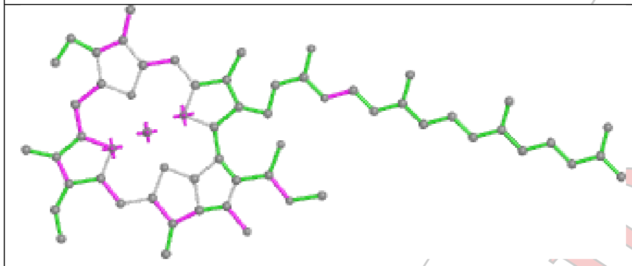 Bond lengths	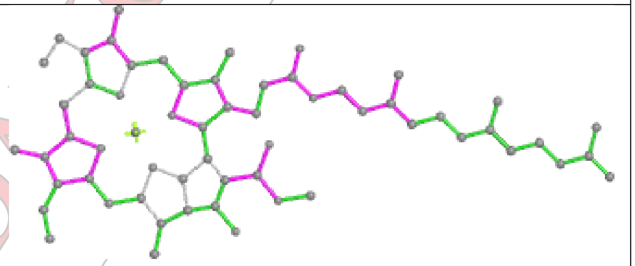 Bond angles
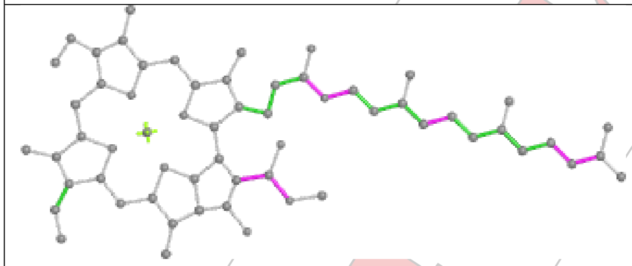 Torsions	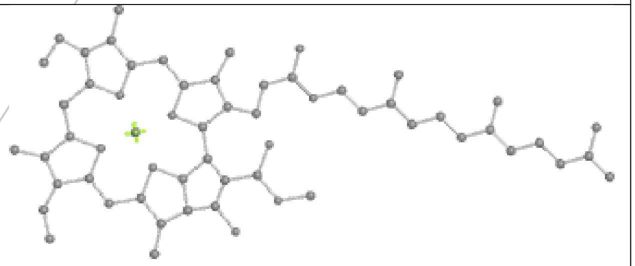 Rings

PRELIMINARY

Ligand CLA AA 1129

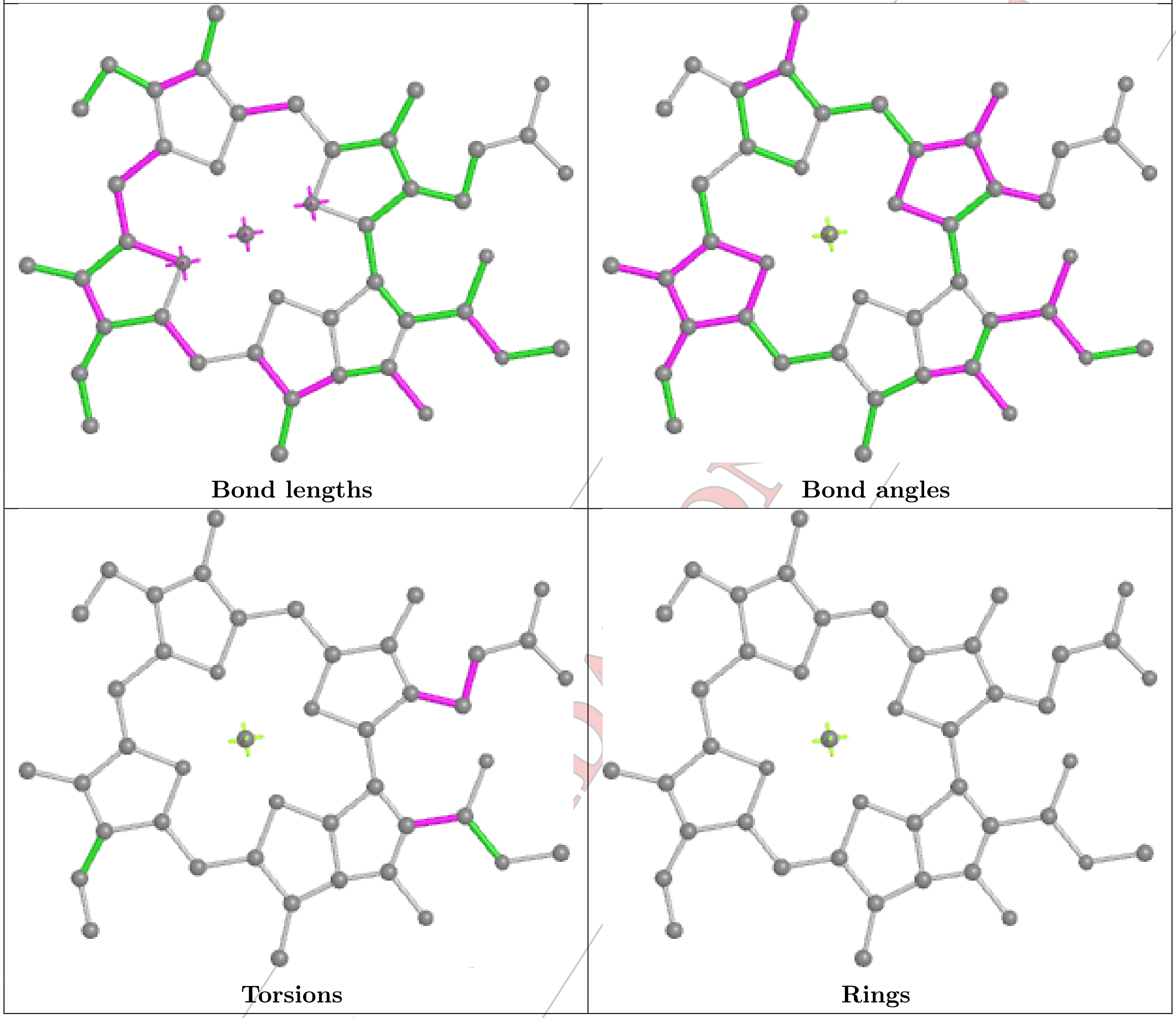

PRELIMINARY

Ligand CLA AA 1130

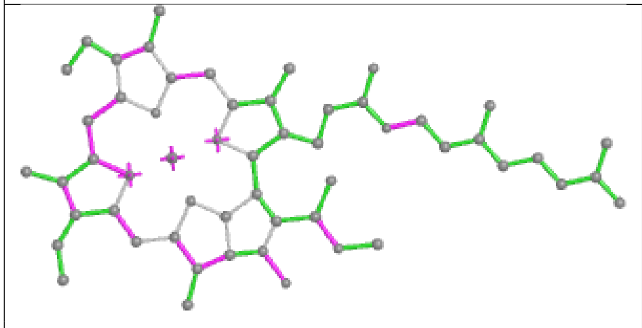

Bond lengths

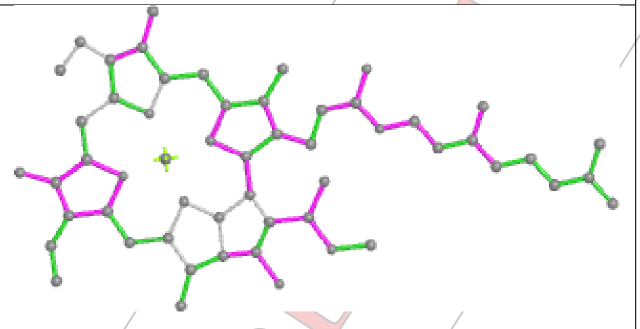

Bond angles

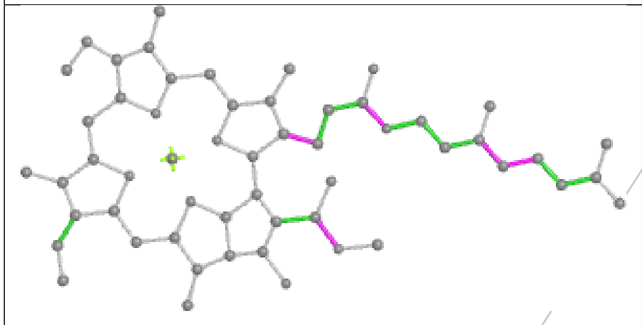

Torsions

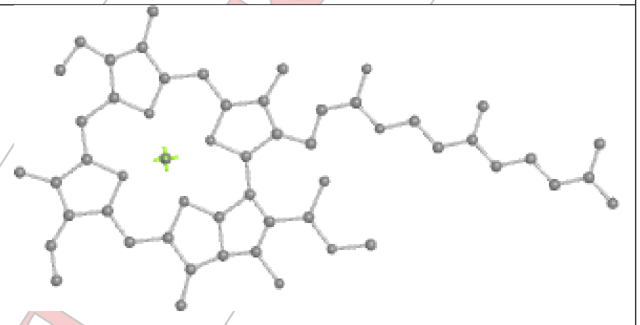

Rings

Ligand CLA AA 1131

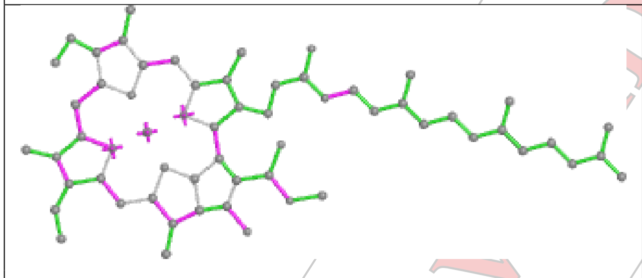

Bond lengths

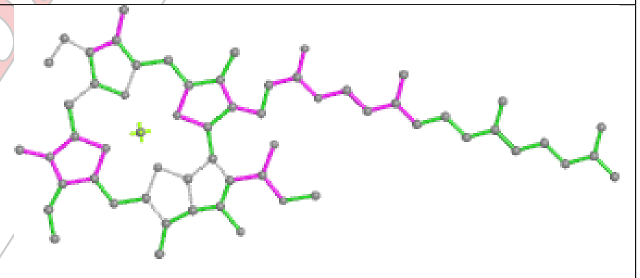

Bond angles

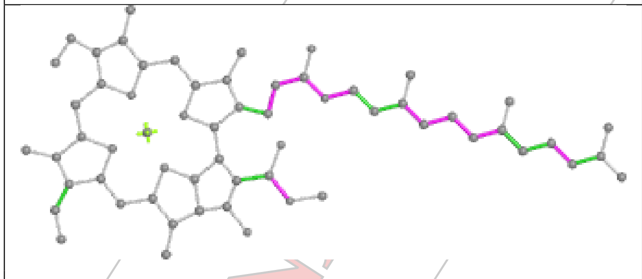

Torsions

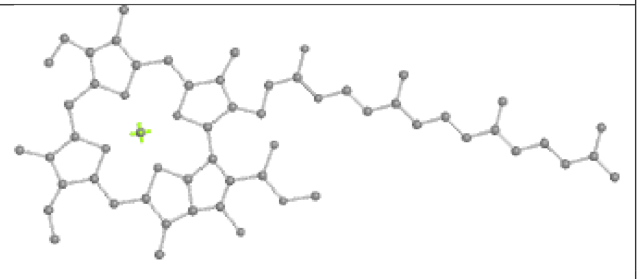

Rings

Ligand CLA AA 1132

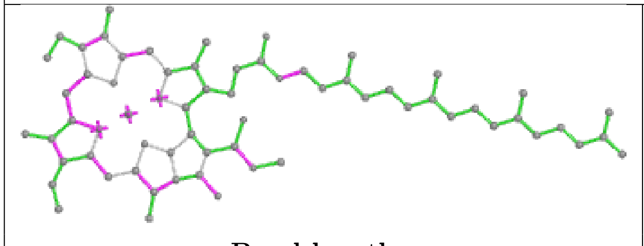
Bond lengths

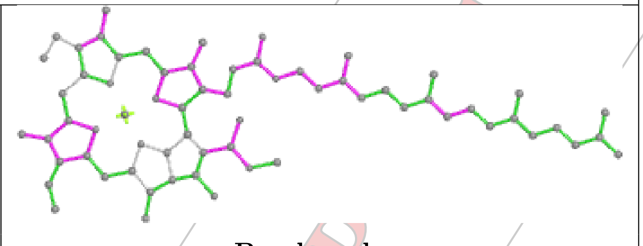
Bond angles

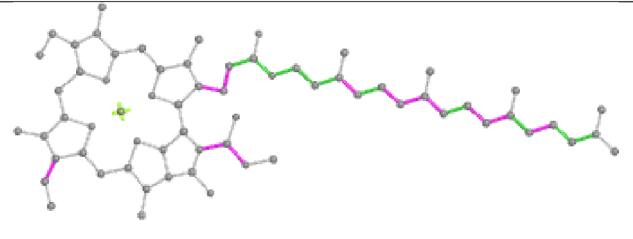
Torsions

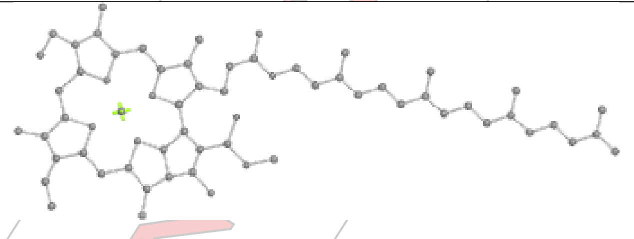
Rings

Ligand CLA AA 1133

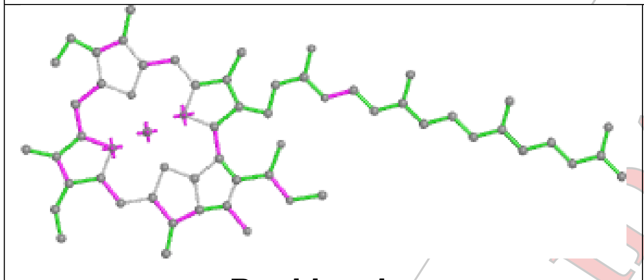
Bond lengths

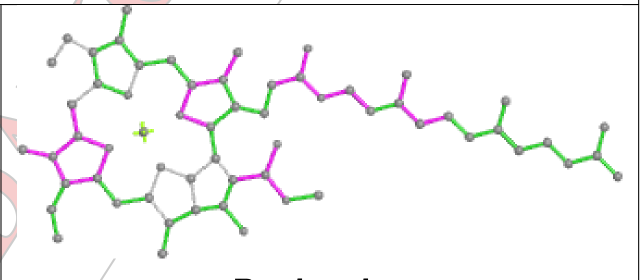
Bond angles

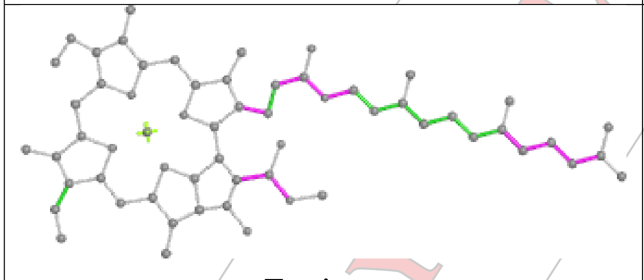
Torsions

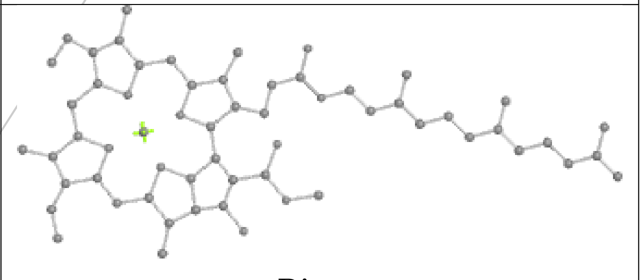
Rings

PRELIMINARY VERSION

WORLDWIDE PDB PROTEIN DATA BANK
EMDataBank Unified Data Resource for 3DEM

Ligand CLA AA 1135

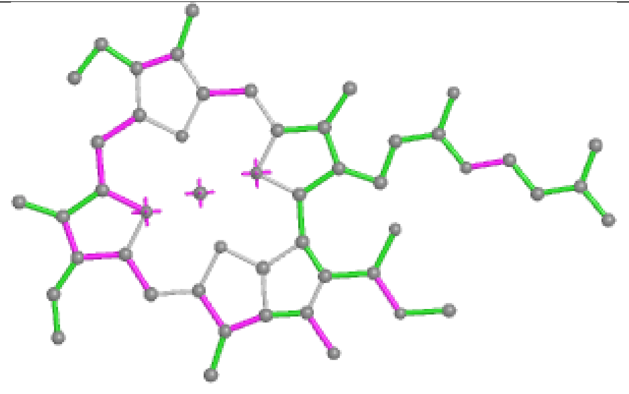

Bond lengths

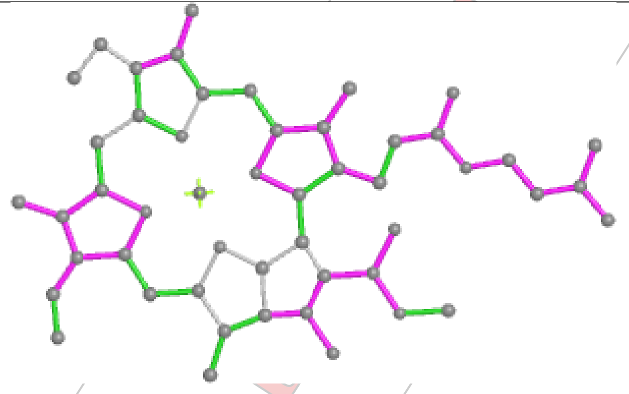

Bond angles

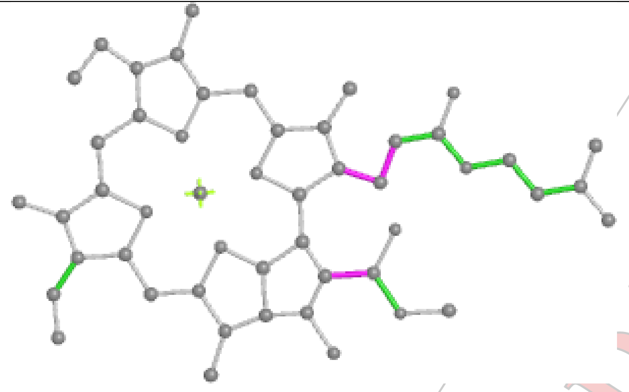

Torsions

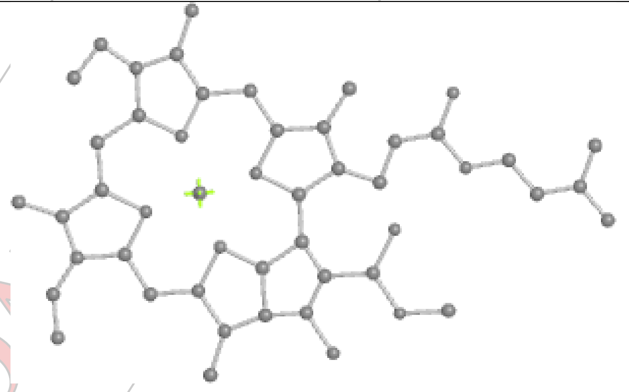

Rings

Ligand CLA AA 1136

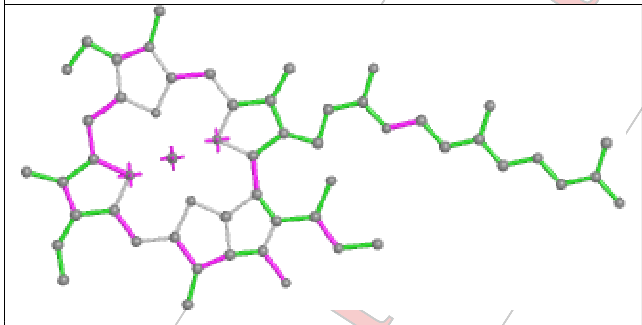

Bond lengths

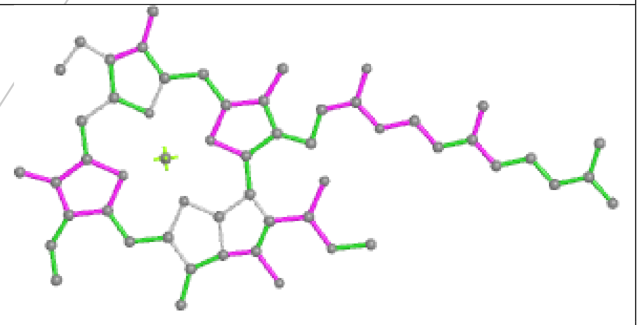

Bond angles

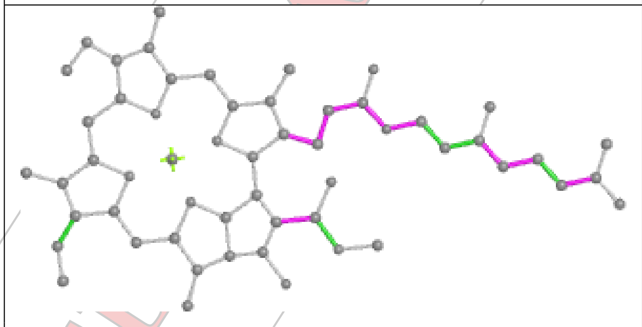

Torsions

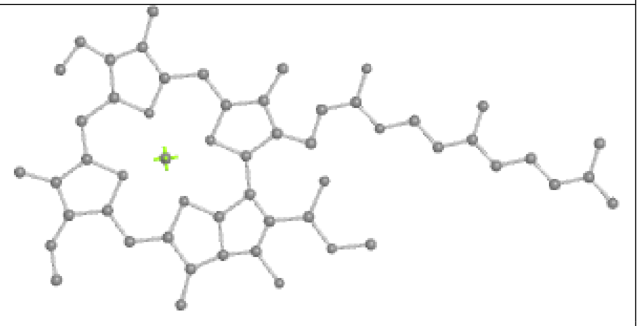

Rings

Ligand CLA AA 1137

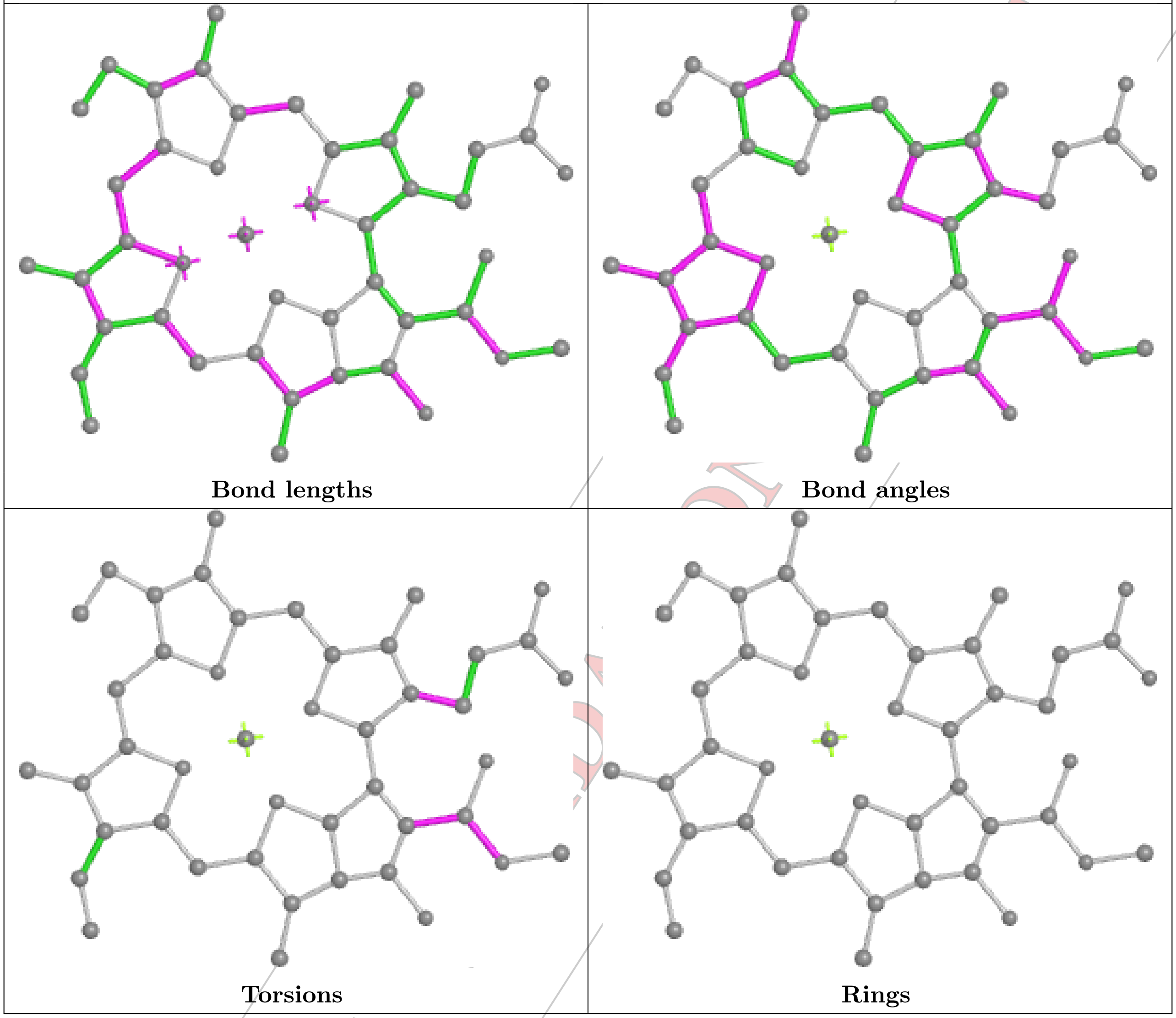

Ligand CLA AA 1138

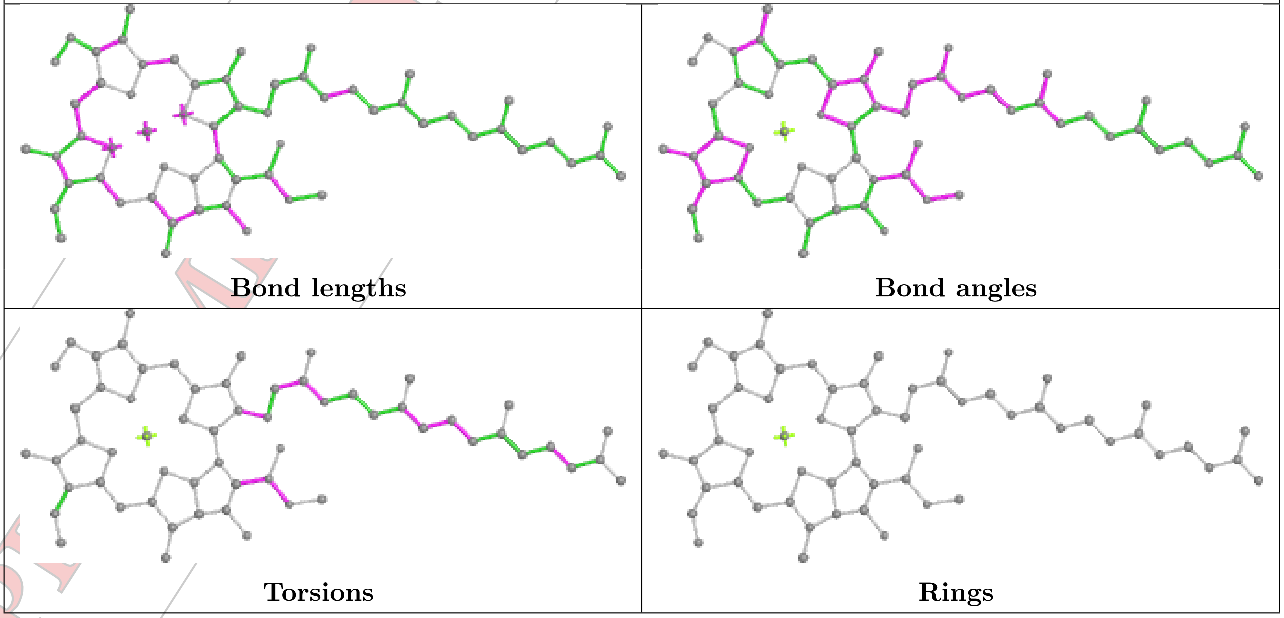

Ligand CLA AA 1139

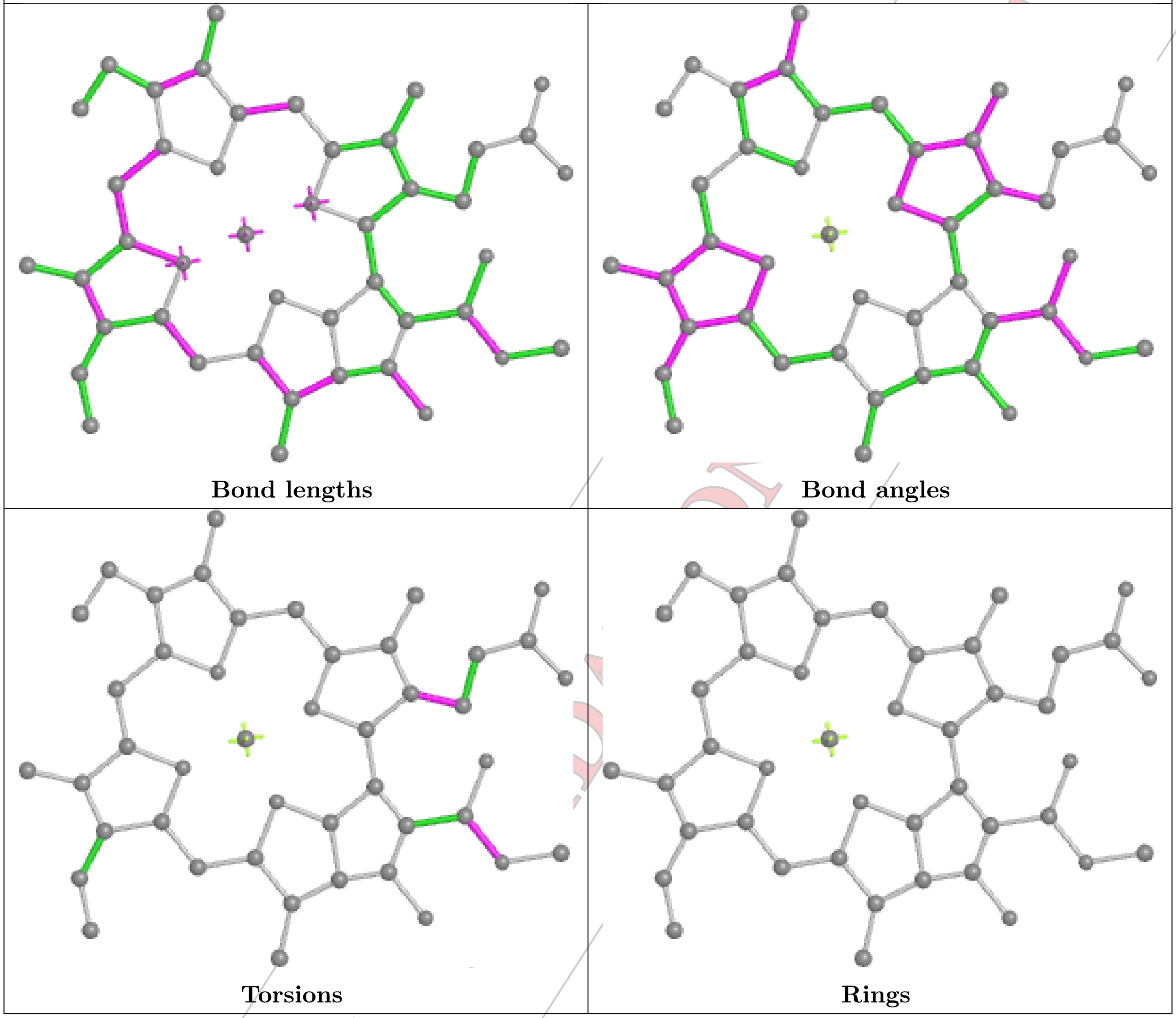

Ligand CLA AA 1140

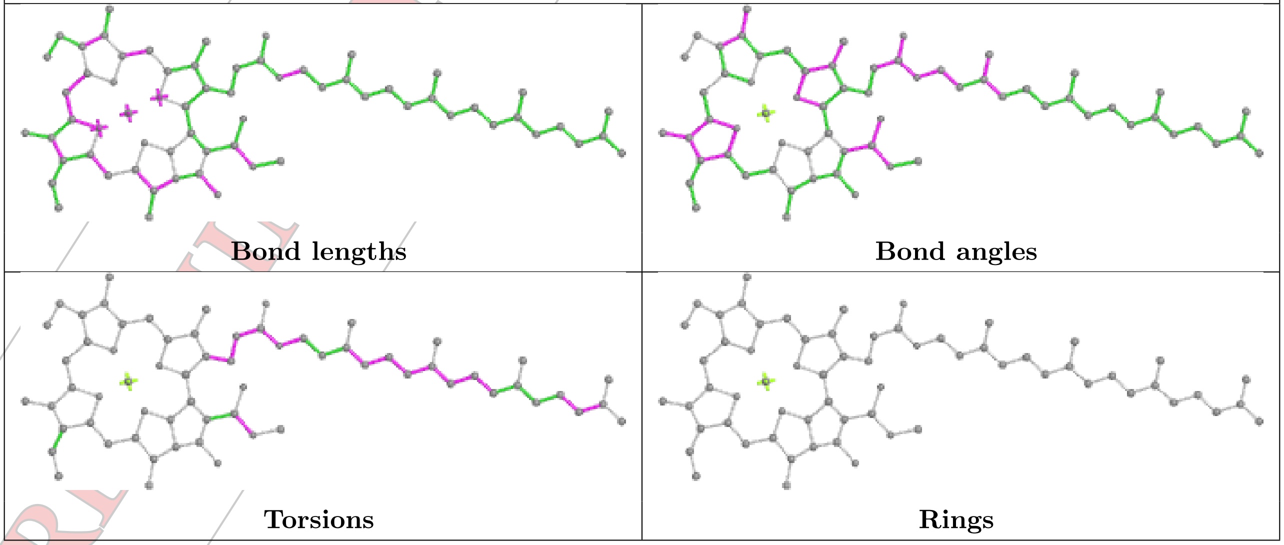

Ligand PQN AA 2001

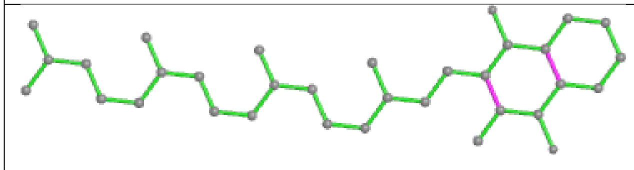
Bond lengths

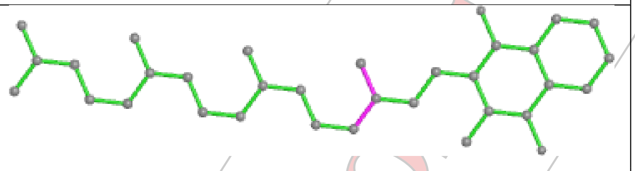
Bond angles

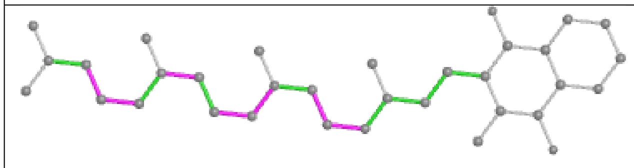
Torsions

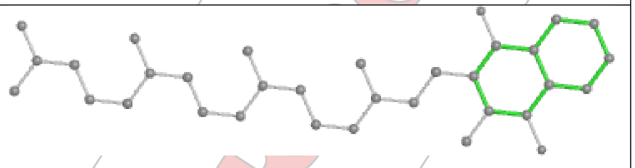
Rings

Ligand BCR AA 4001

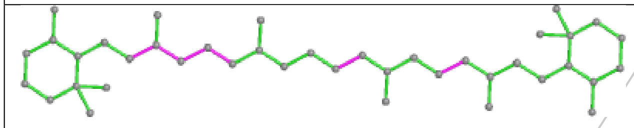
Bond lengths

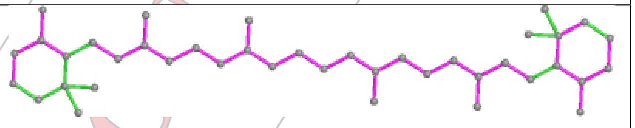
Bond angles

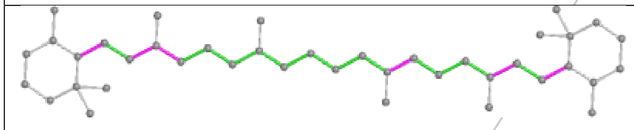
Torsions

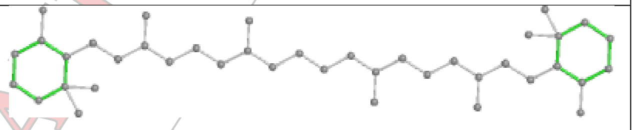
Rings

Ligand BCR AA 4002

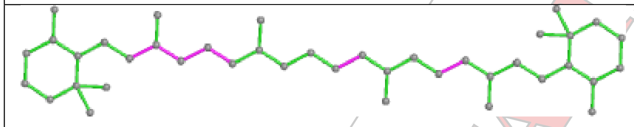
Bond lengths

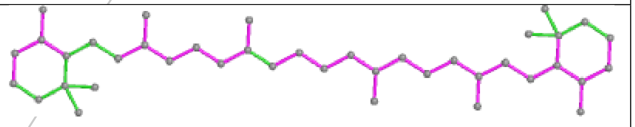
Bond angles

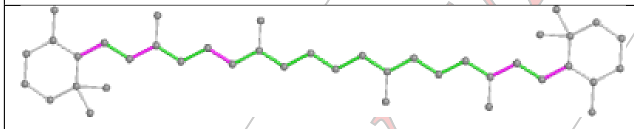
Torsions

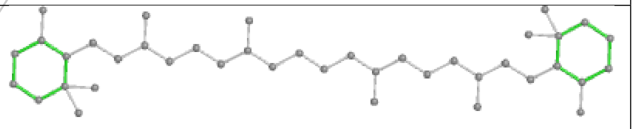
Rings

Ligand BCR AA 4003

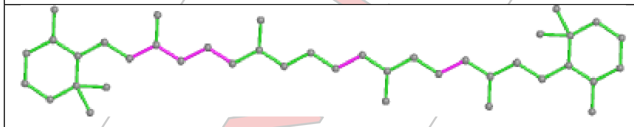
Bond lengths

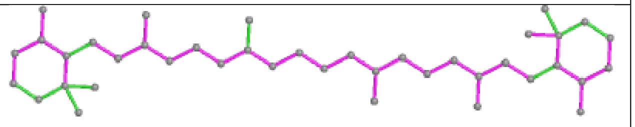
Bond angles

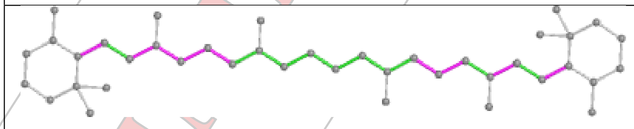
Torsions

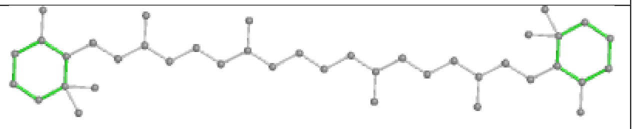
Rings

PRELIMINARY DATA

Ligand BCR AA 4007	
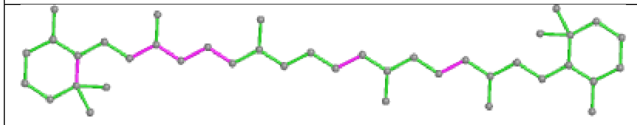 Bond lengths	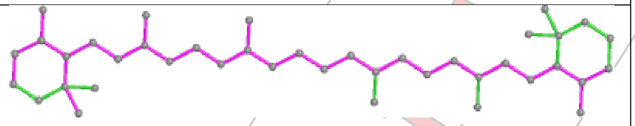 Bond angles
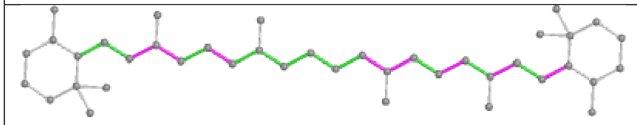 Torsions	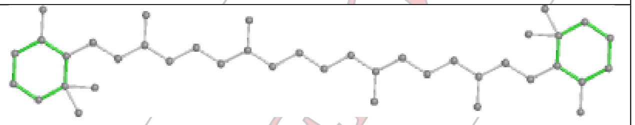 Rings

Ligand BCR AA 4008	
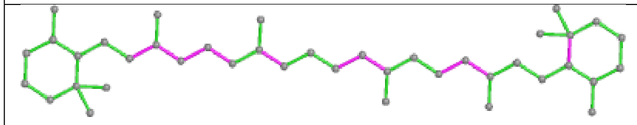 Bond lengths	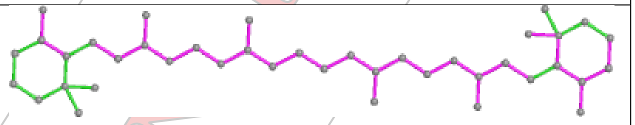 Bond angles
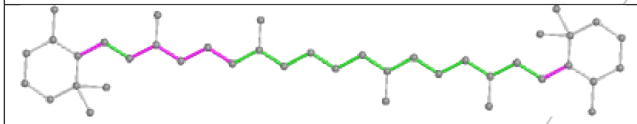 Torsions	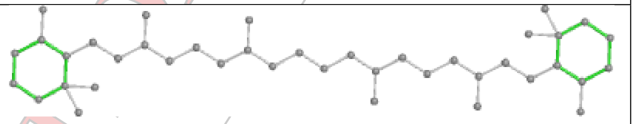 Rings

Ligand BCR AA 4011	
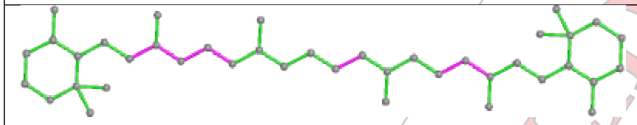 Bond lengths	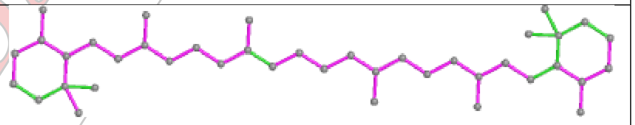 Bond angles
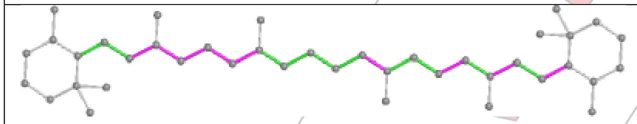 Torsions	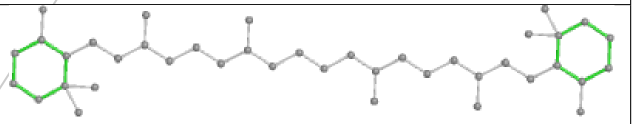 Rings

Ligand LHG AA 5001

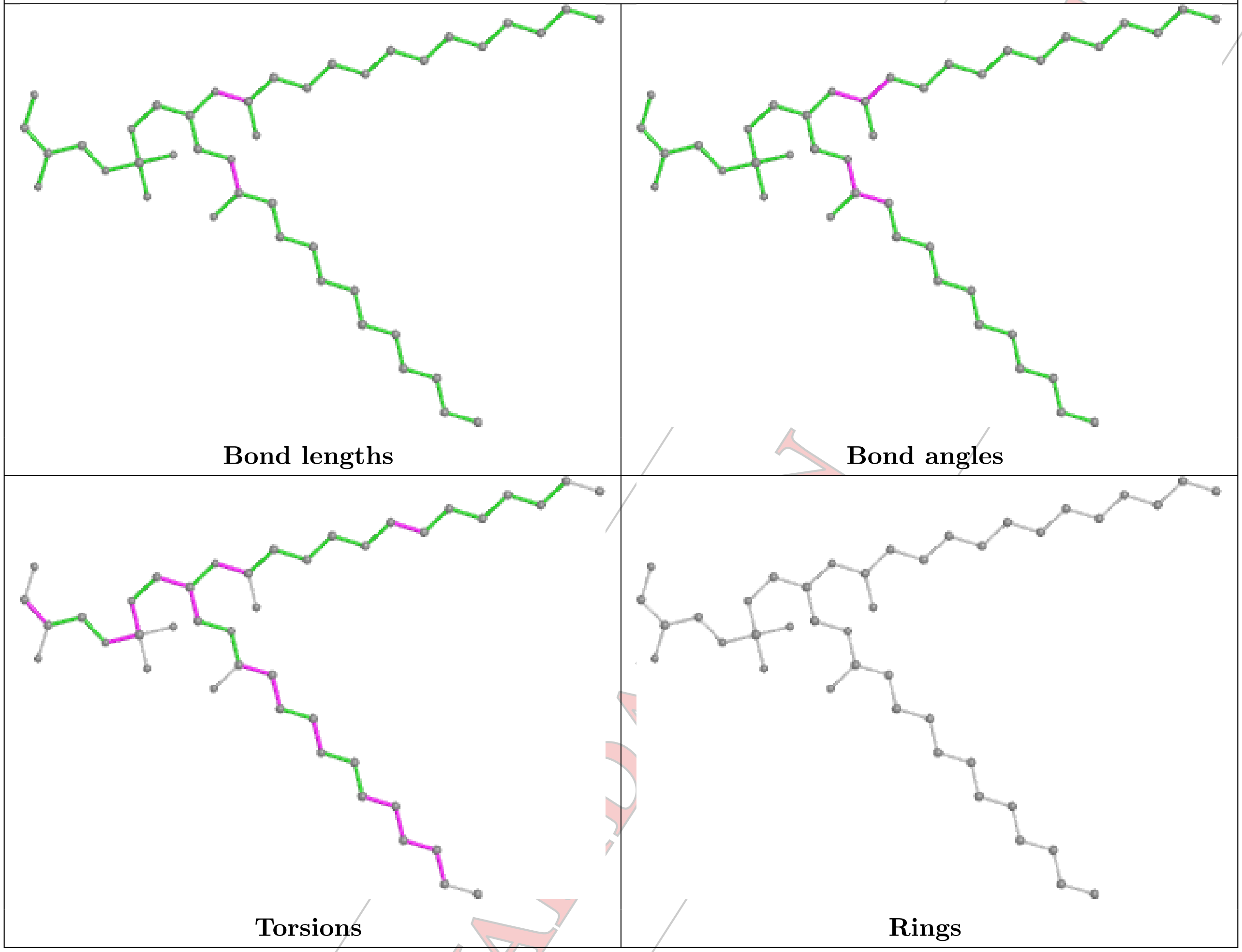

Ligand LMG AA 5005

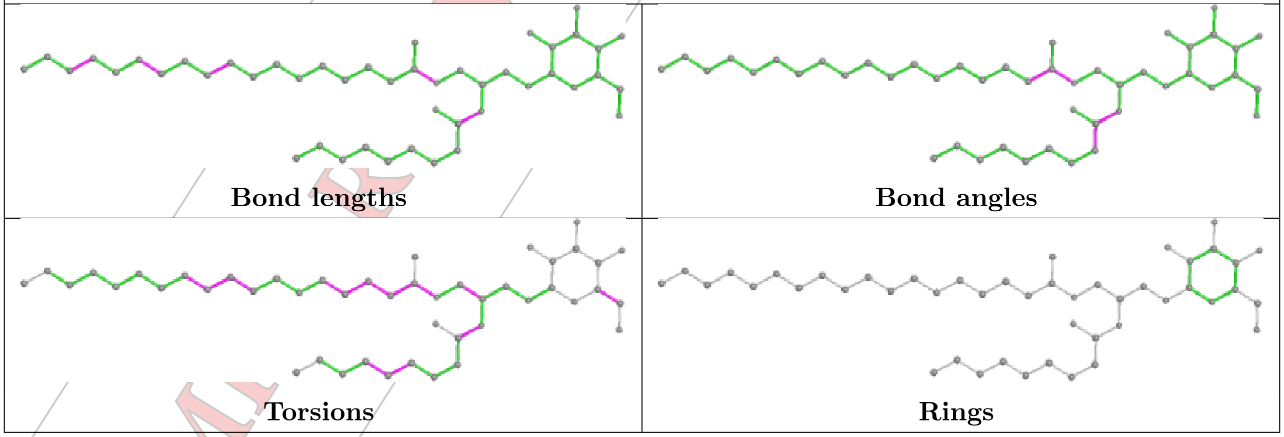

Ligand LMT AA 6001

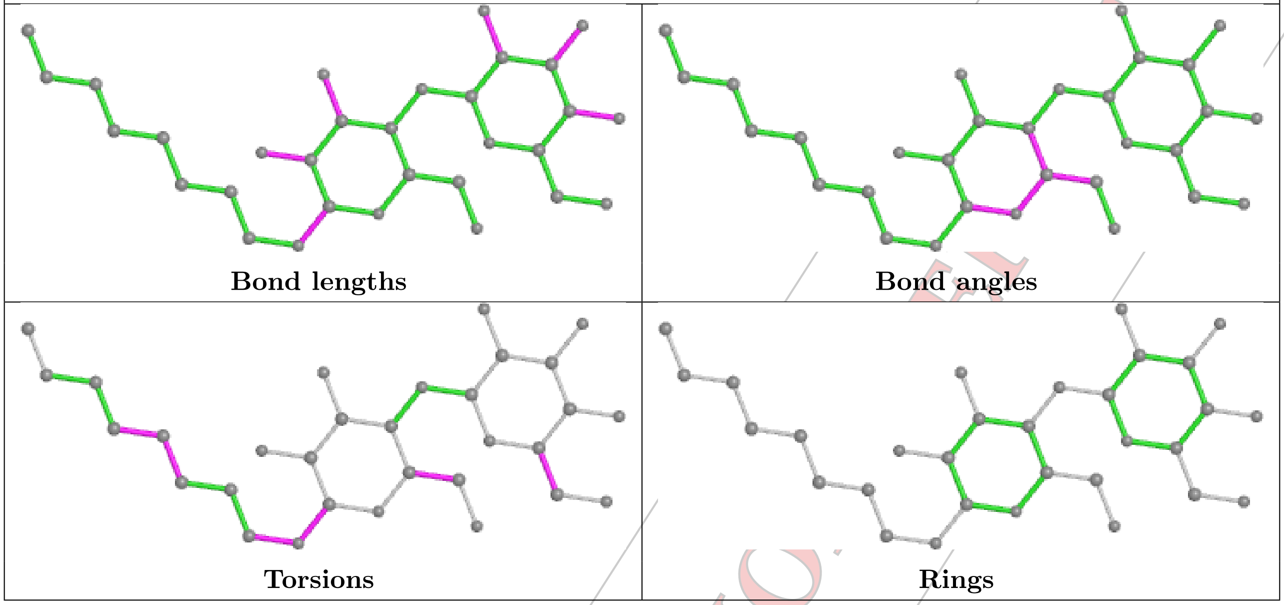

Ligand LMT AA 6002

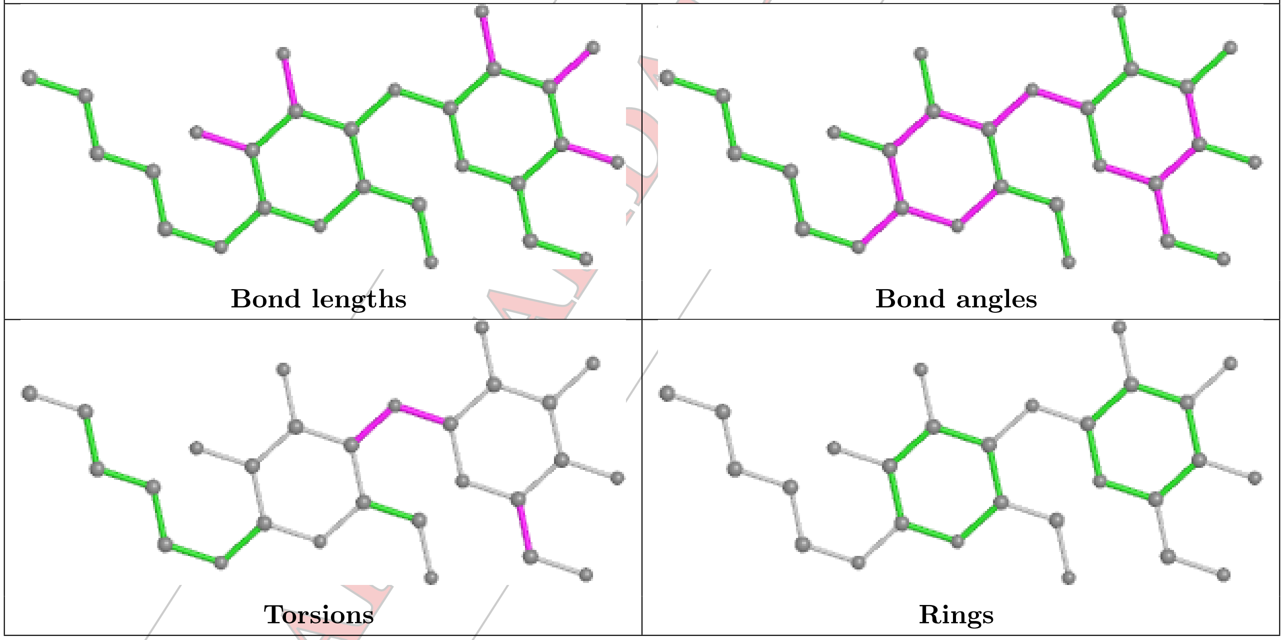

Ligand CLA BA 1021

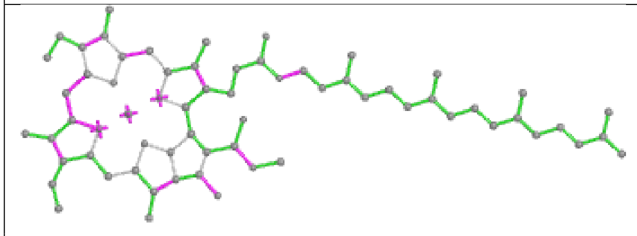
Bond lengths

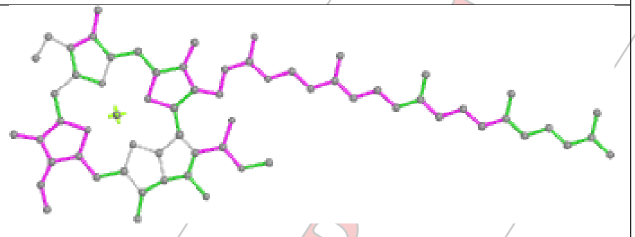
Bond angles

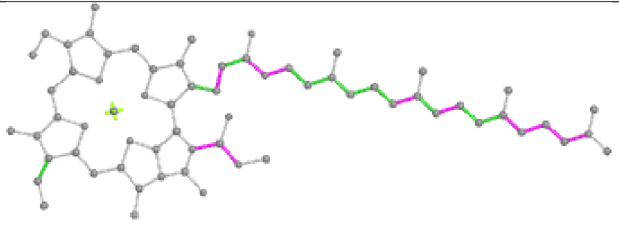
Torsions

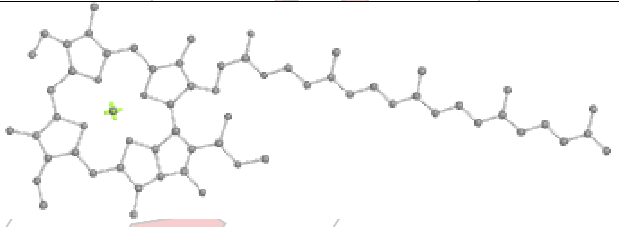
Rings

Ligand CLA BA 1022

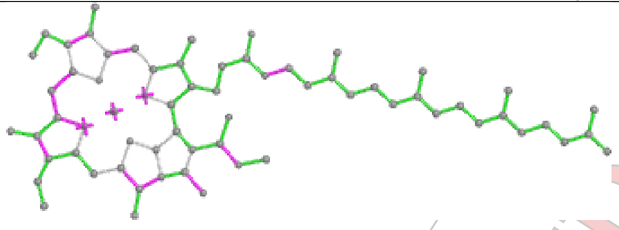
Bond lengths

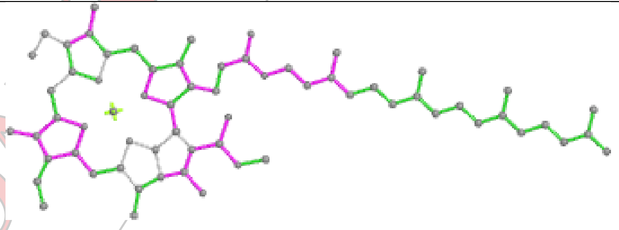
Bond angles

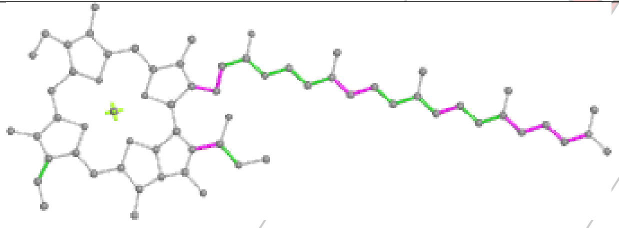
Torsions

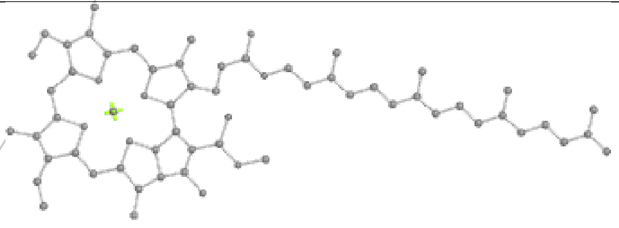
Rings

Ligand CLA BA 1023

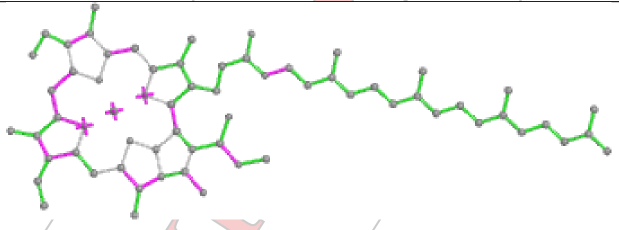
Bond lengths

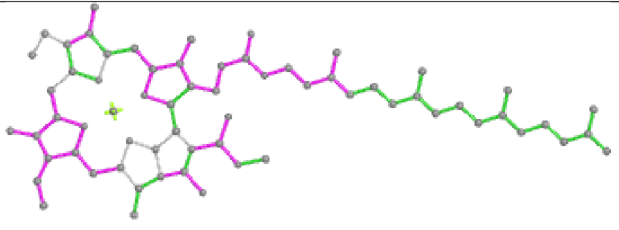
Bond angles

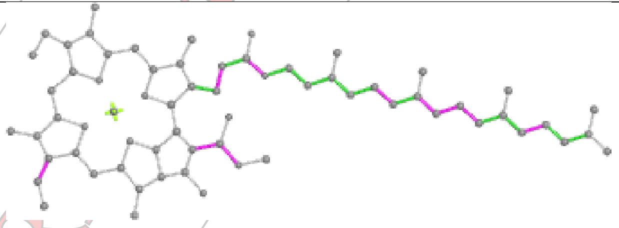
Torsions

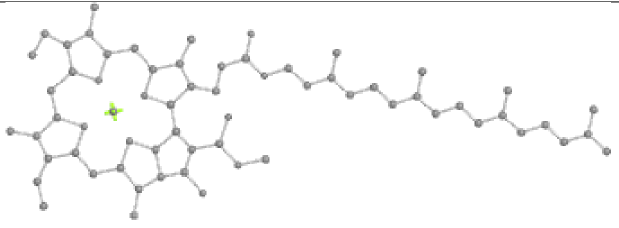
Rings

Ligand CLA BA 1201

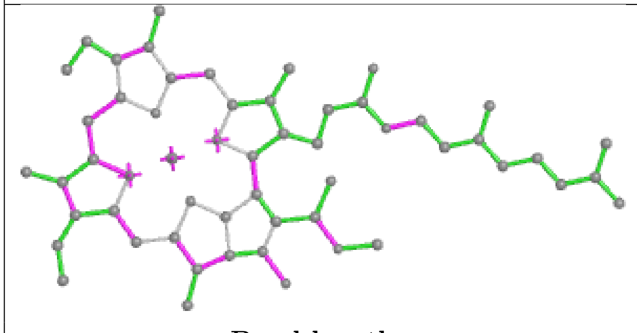

Bond lengths

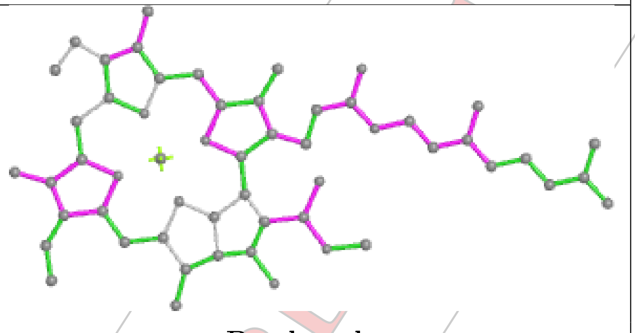

Bond angles

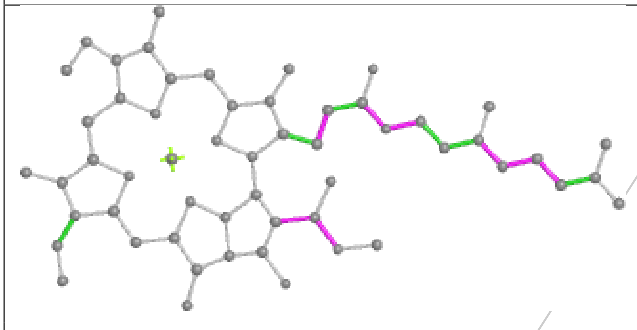

Torsions

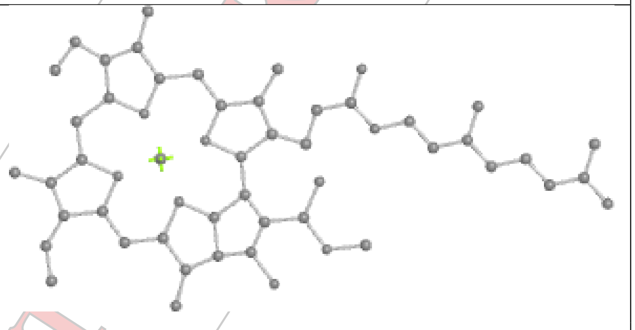

Rings

Ligand CLA BA 1202

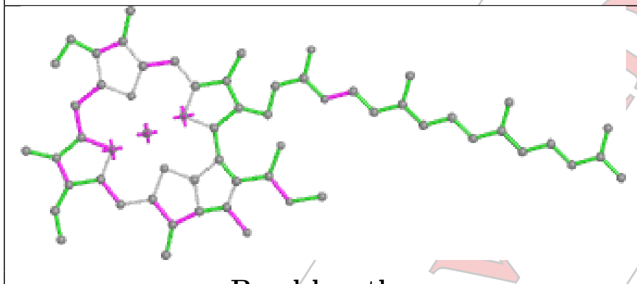

Bond lengths

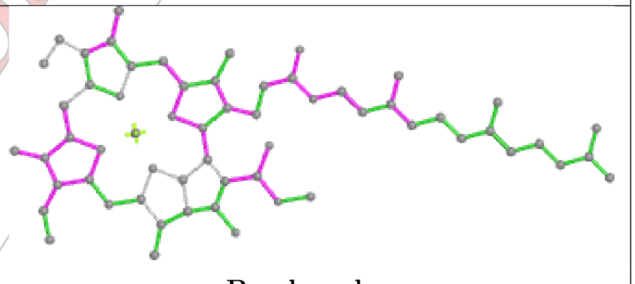

Bond angles

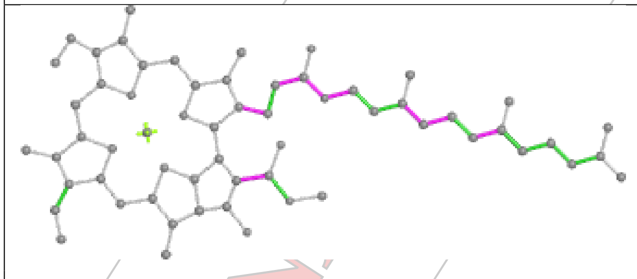

Torsions

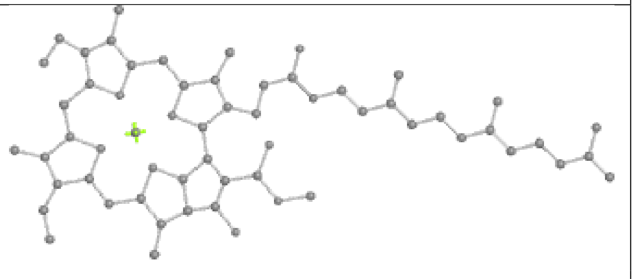

Rings

PRELIMINARY

Ligand CLA BA 1203

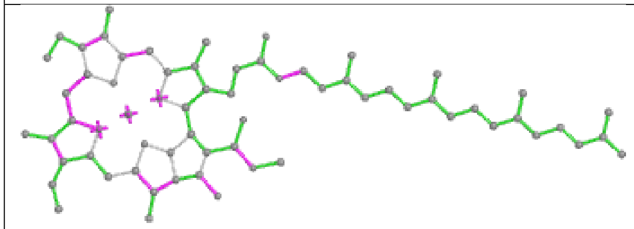
Bond lengths

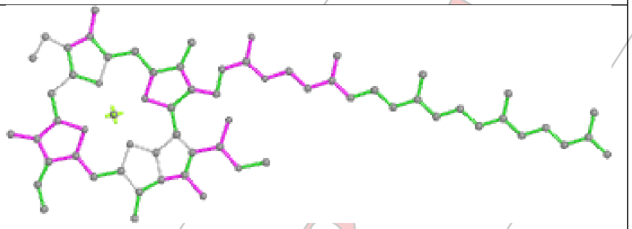
Bond angles

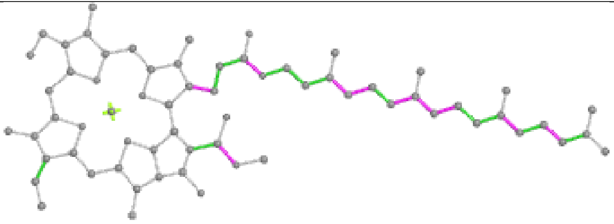
Torsions

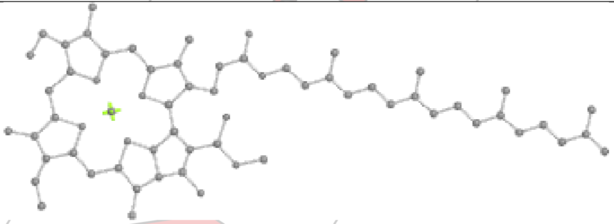
Rings

Ligand CLA BA 1204

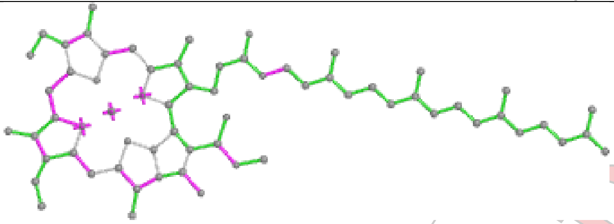
Bond lengths

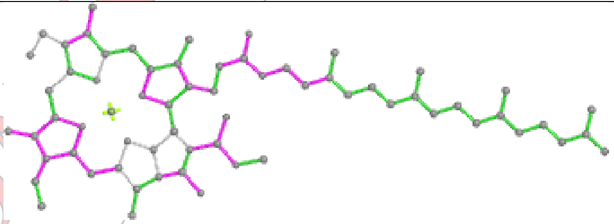
Bond angles

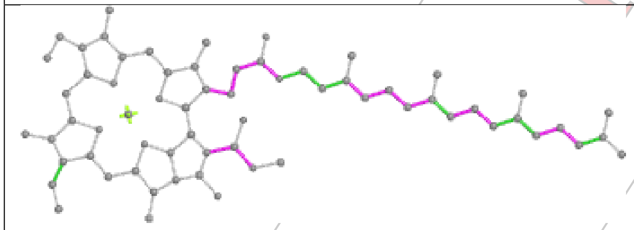
Torsions

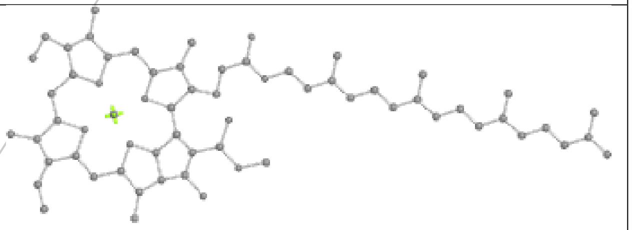
Rings

Ligand CLA BA 1205

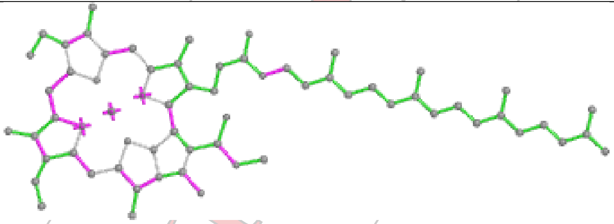
Bond lengths

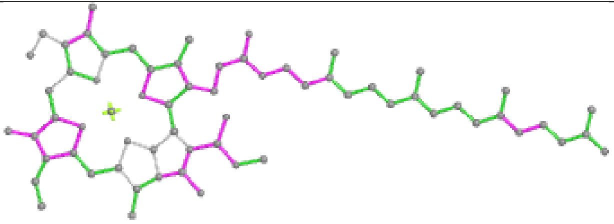
Bond angles

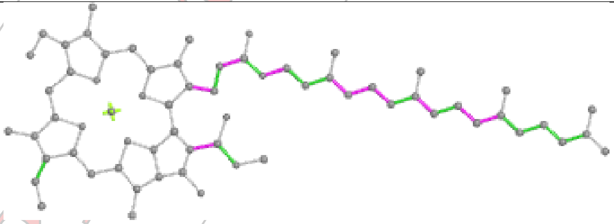
Torsions

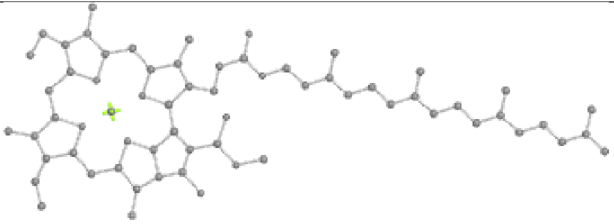
Rings

Ligand CLA BA 1206

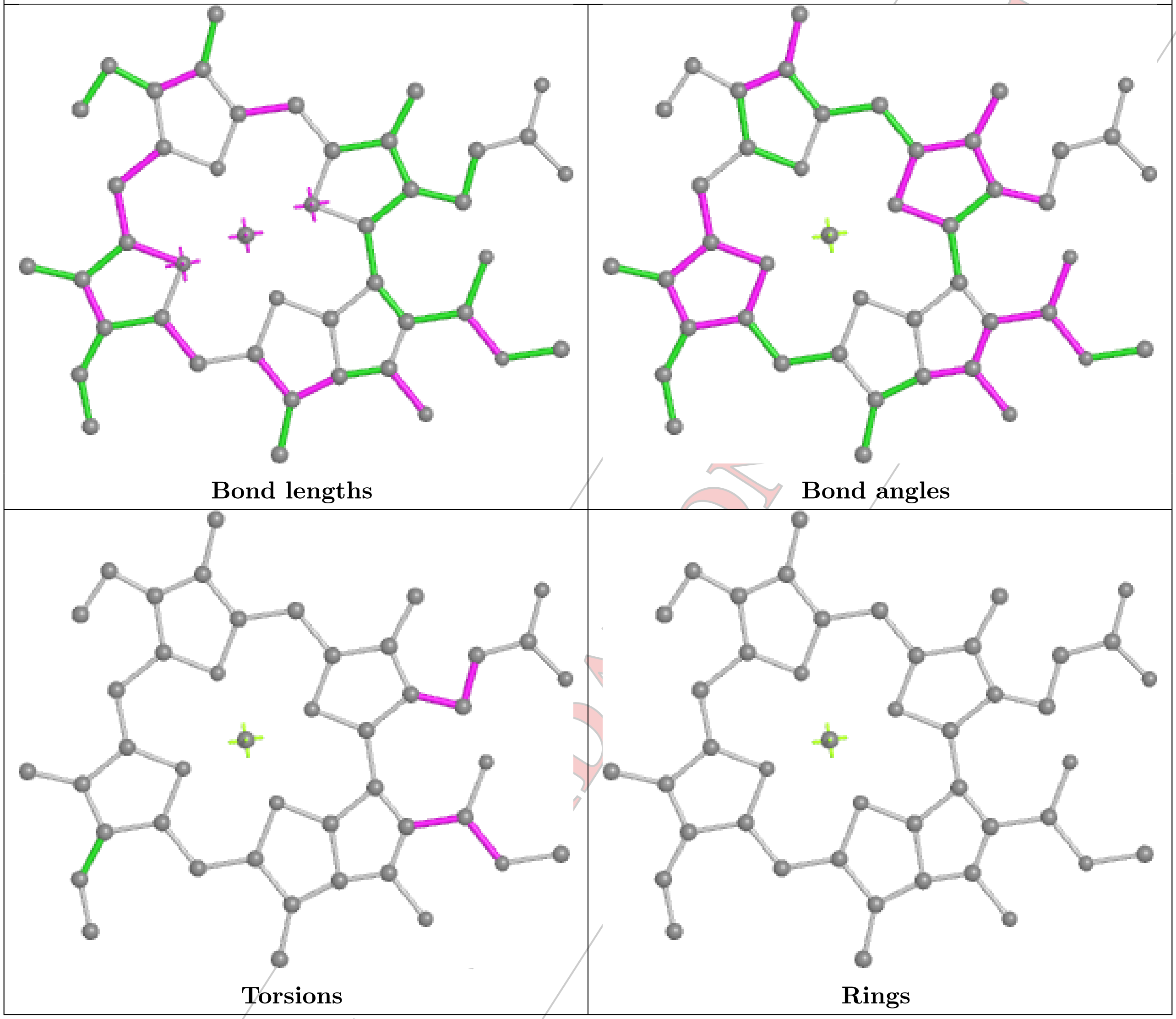

PRELIMINARY

Ligand CLA BA 1207

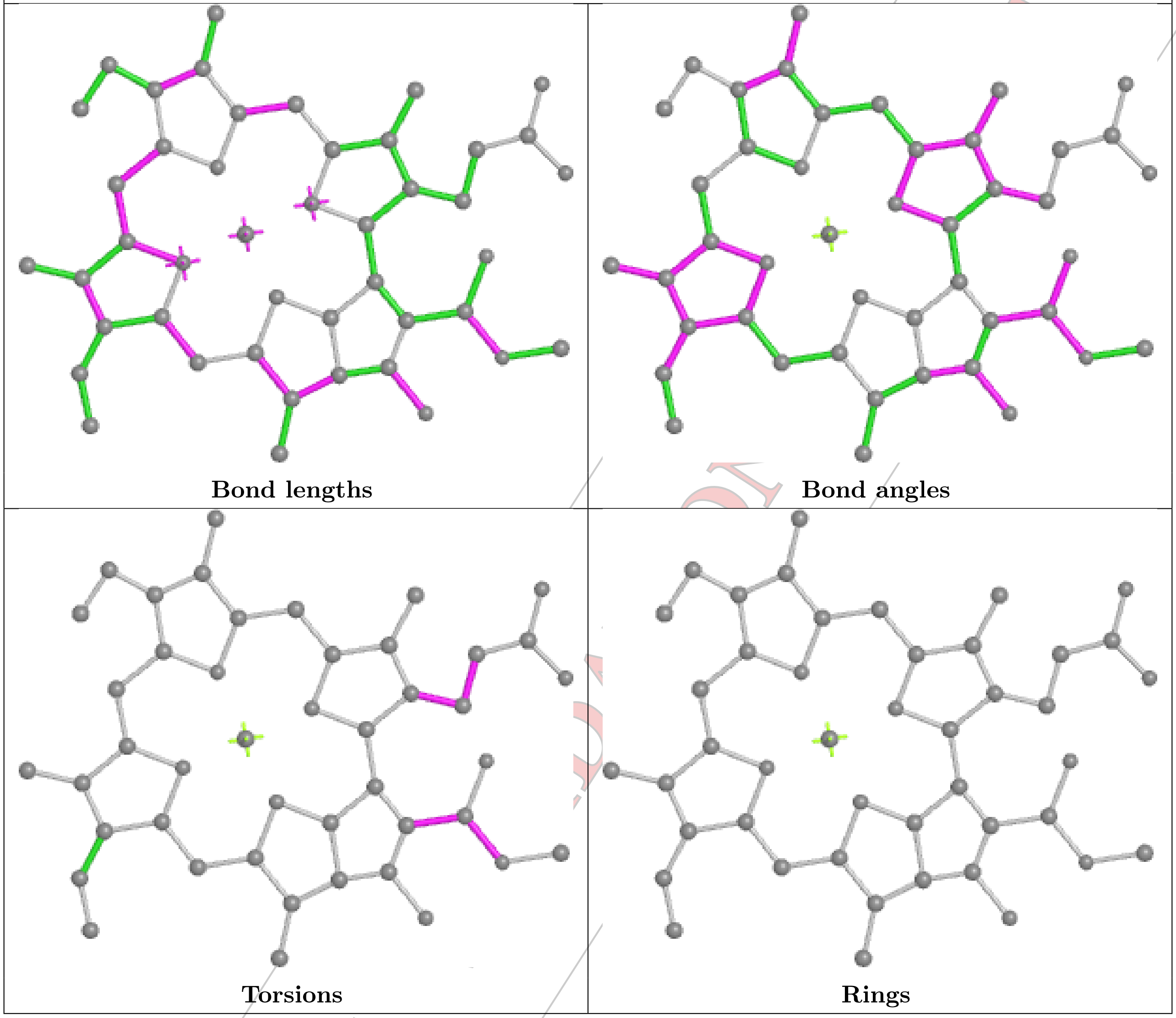

Ligand CLA BA 1208

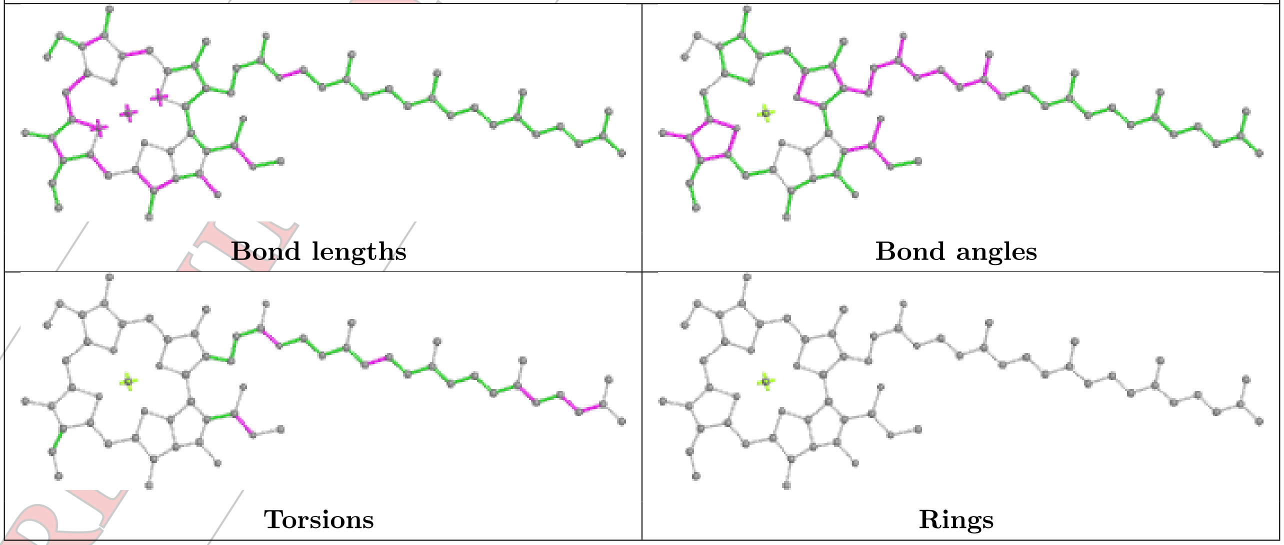

Ligand CLA BA 1209

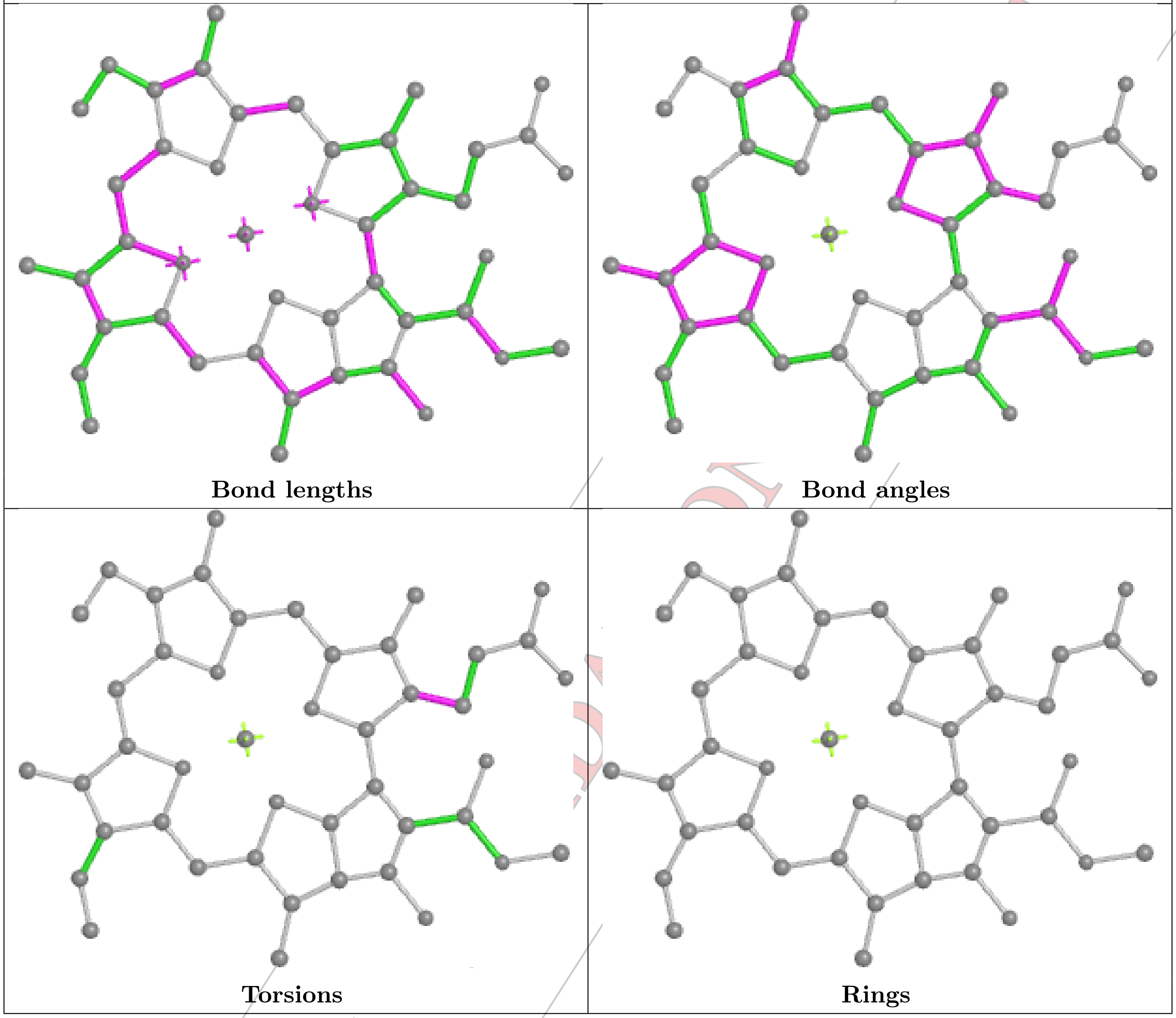

Ligand CLA BA 1210

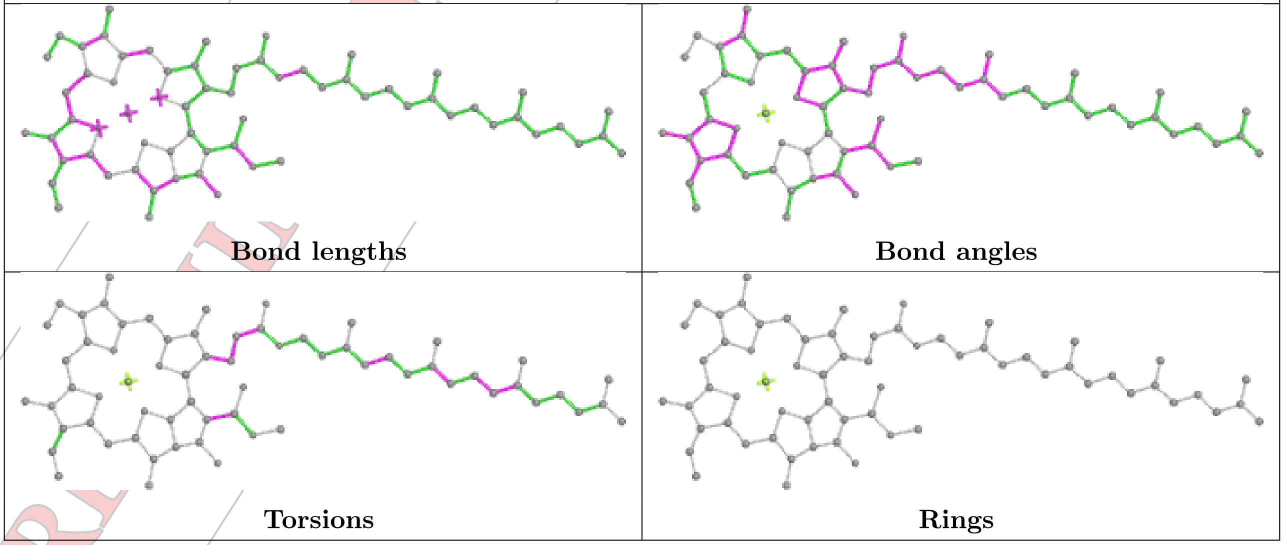

Ligand CLA BA 1211

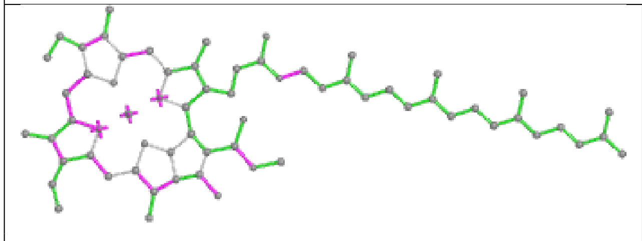

Bond lengths

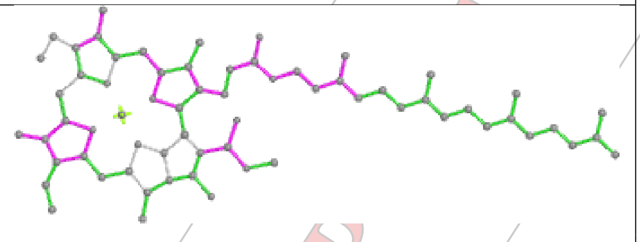

Bond angles

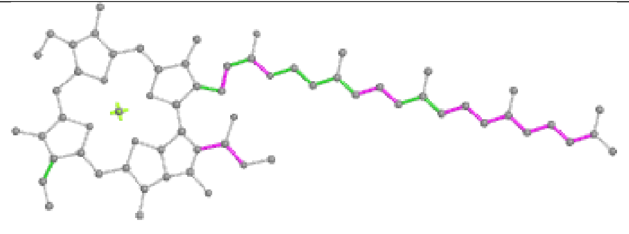

Torsions

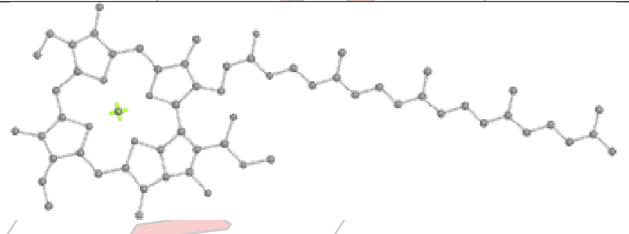

Rings

Ligand CLA BA 1212

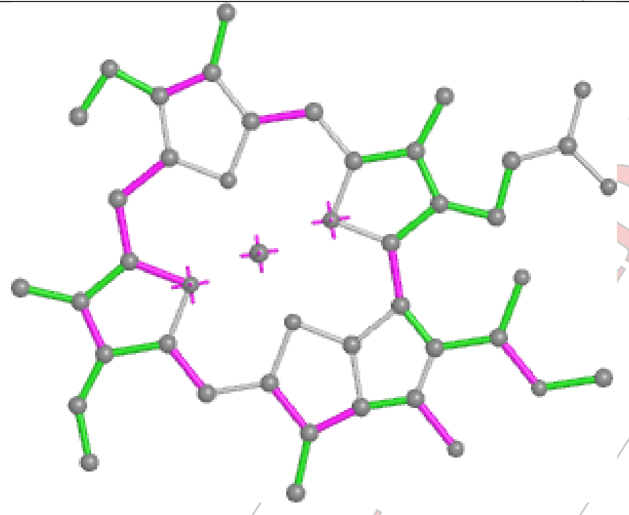

Bond lengths

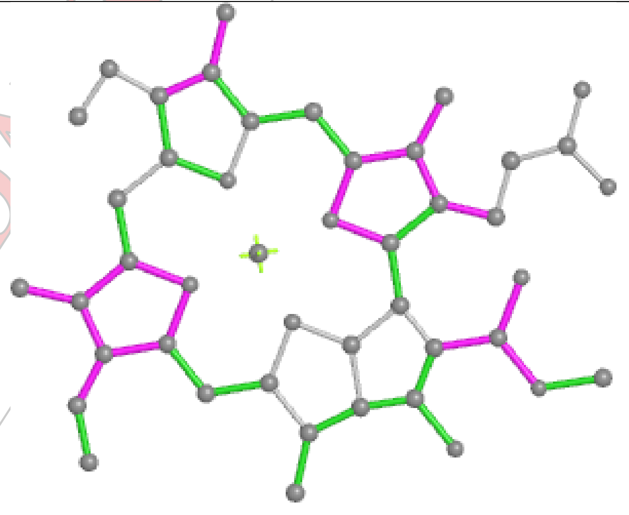

Bond angles

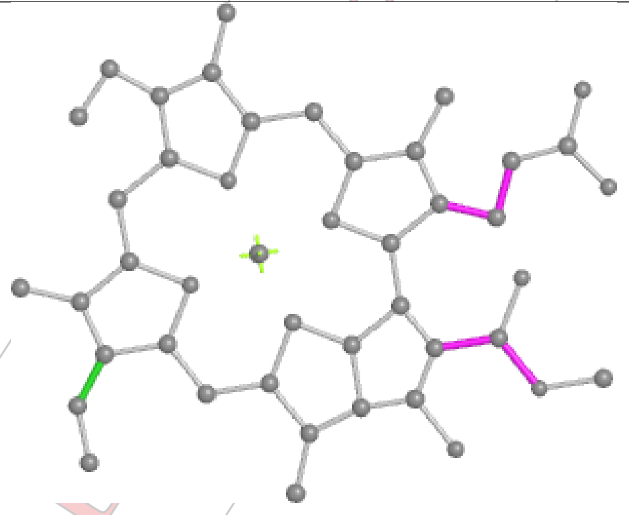

Torsions

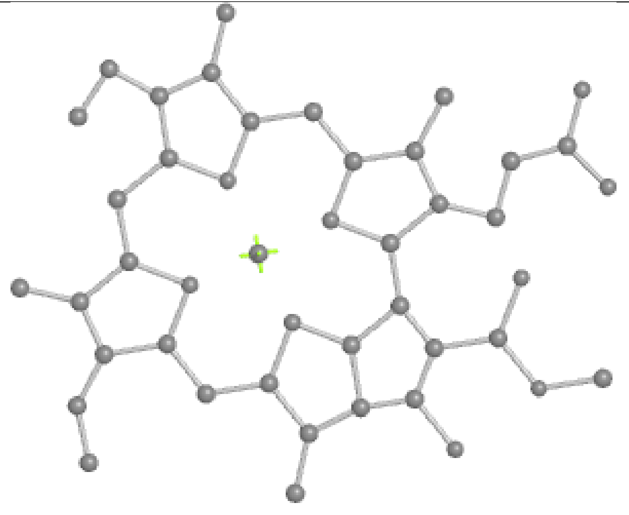

Rings

Ligand CLA BA 1213

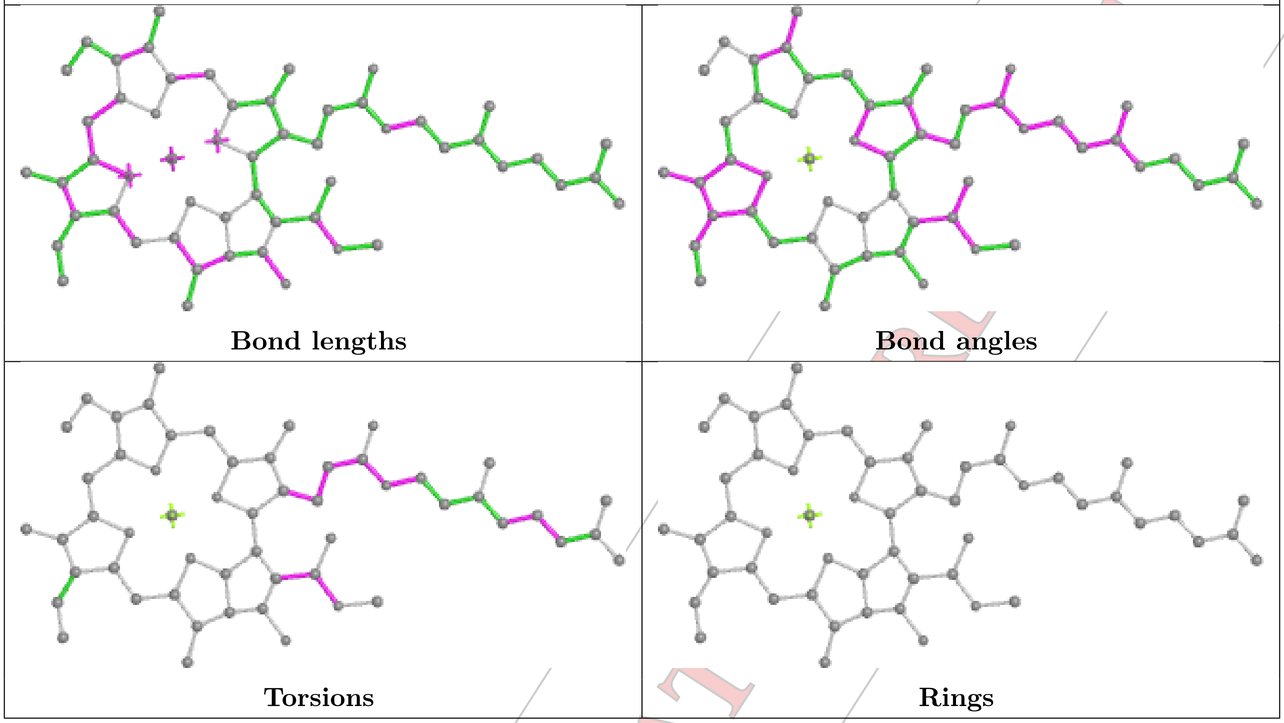

Ligand CLA BA 1214

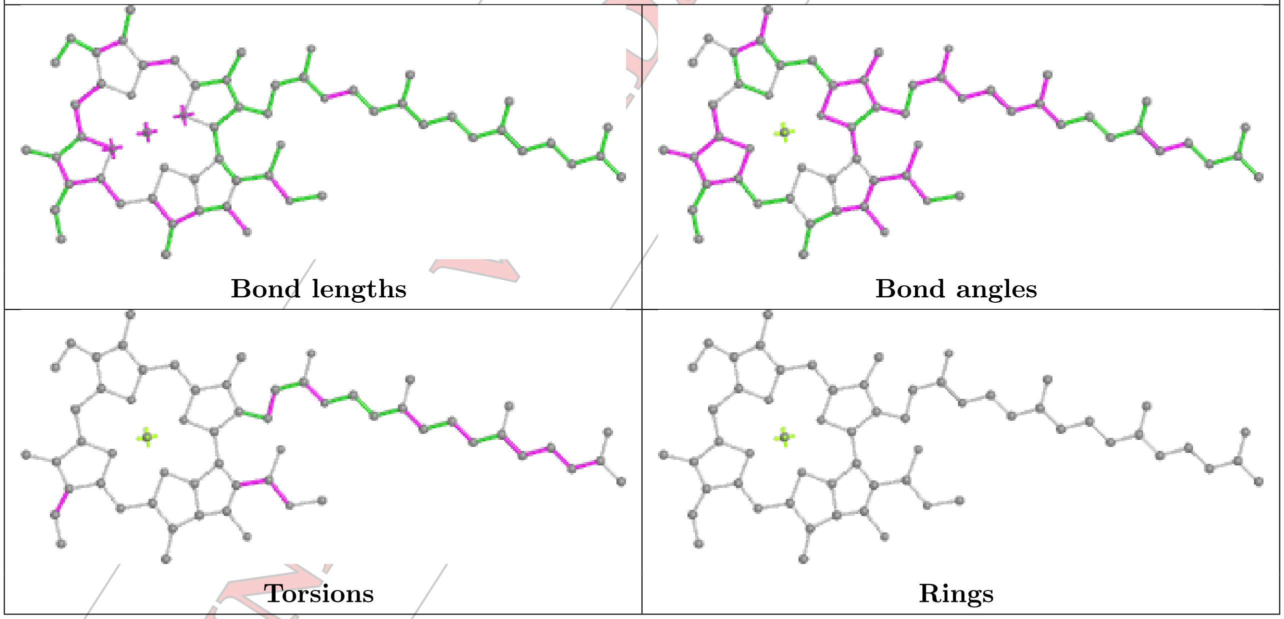

Ligand CLA BA 1215

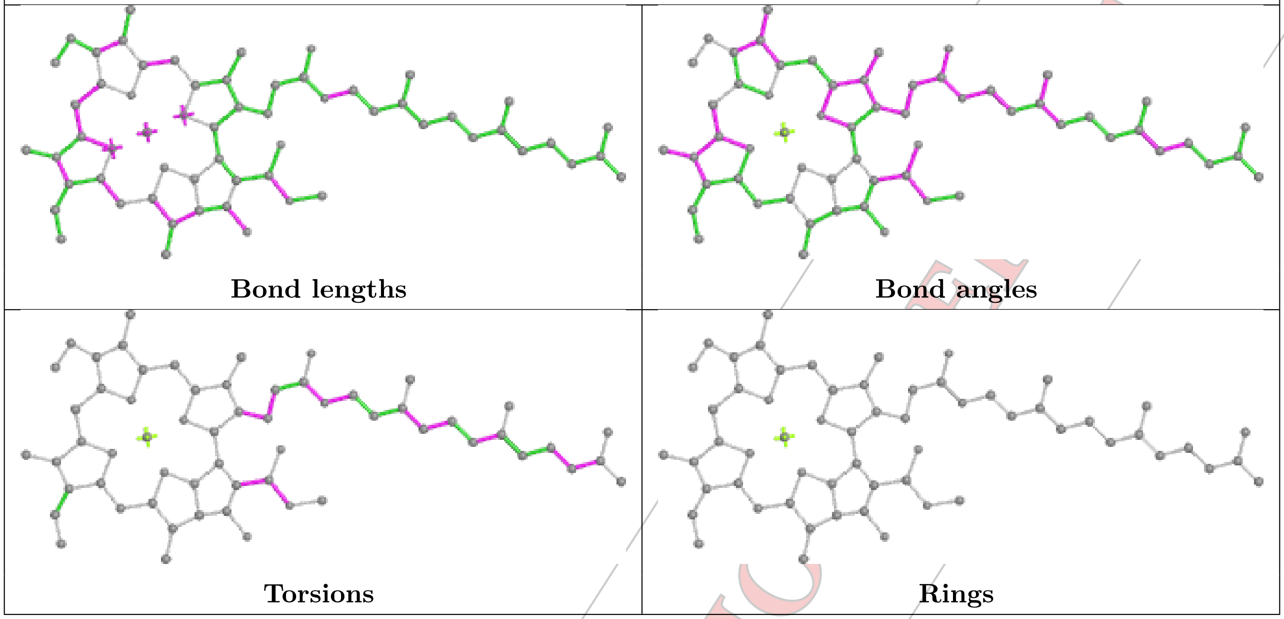

Ligand CLA BA 1216

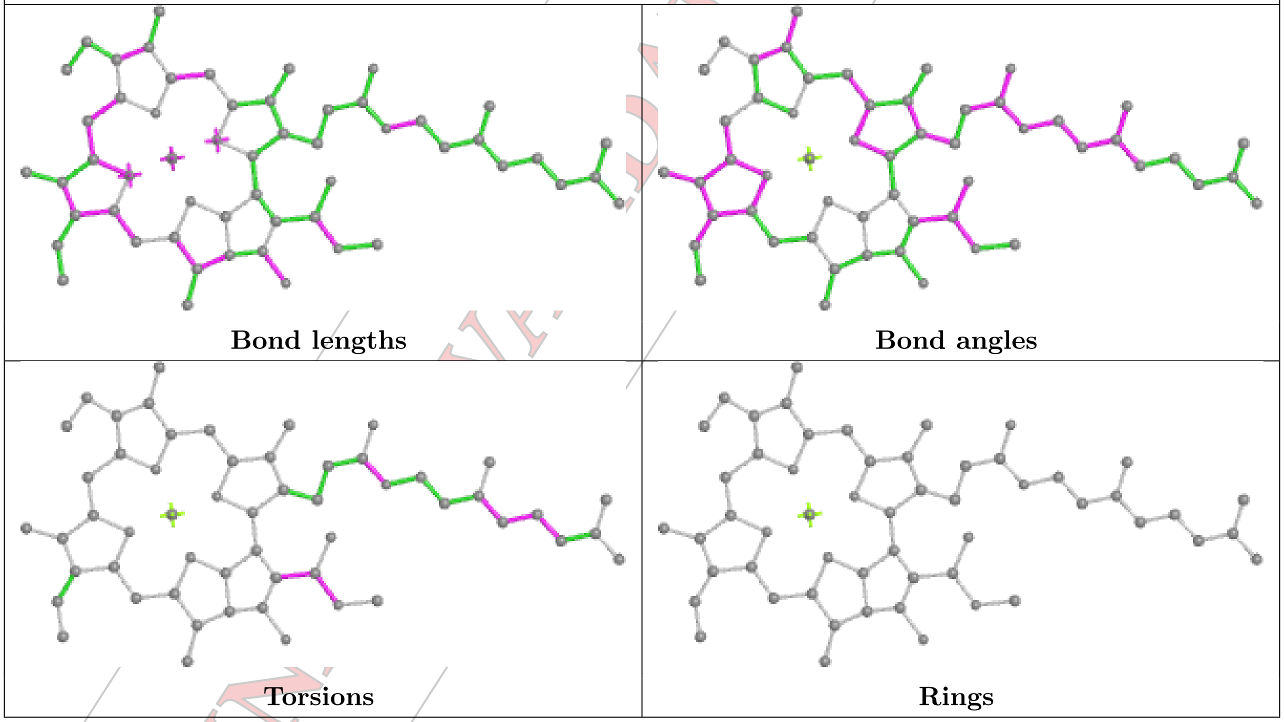

Ligand CLA BA 1217

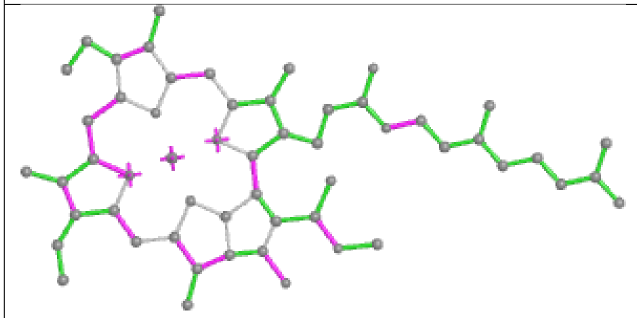

Bond lengths

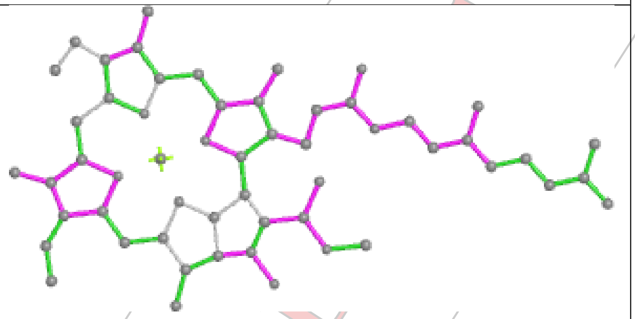

Bond angles

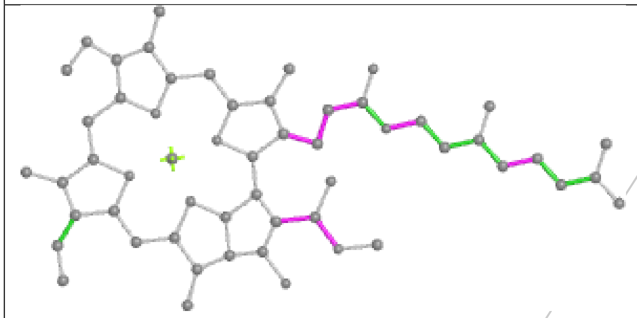

Torsions

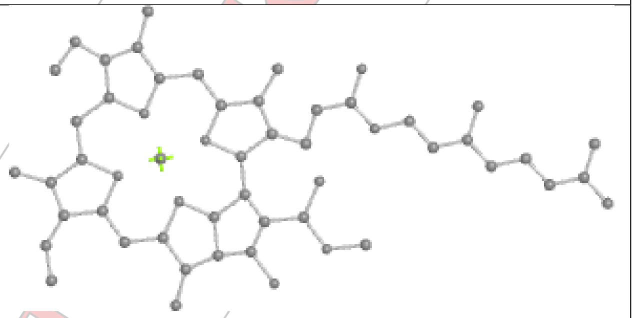

Rings

PRELIMINARY VALIDATION

Ligand CLA BA 1218

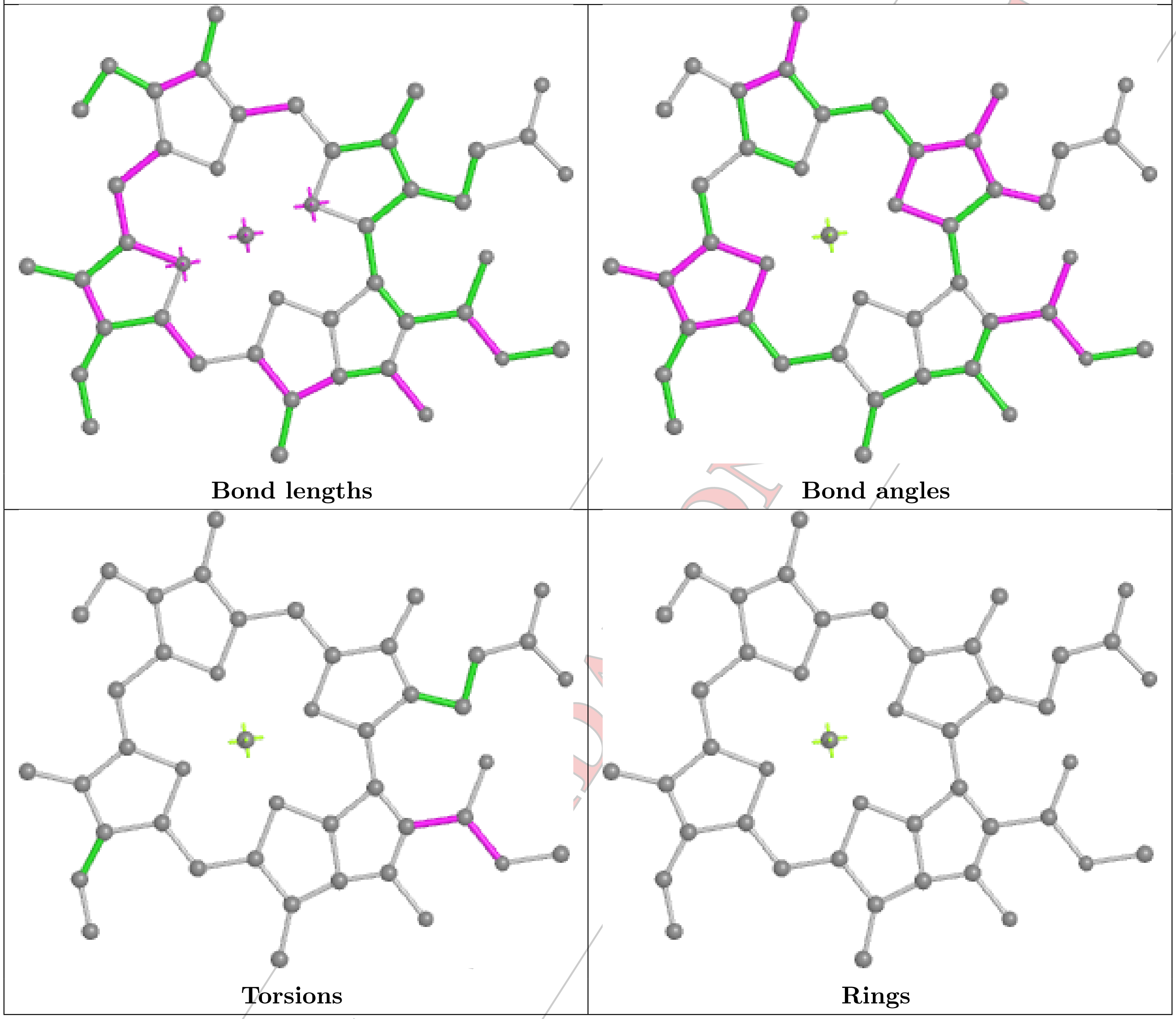

PRELIMINARY

Ligand CLA BA 1219

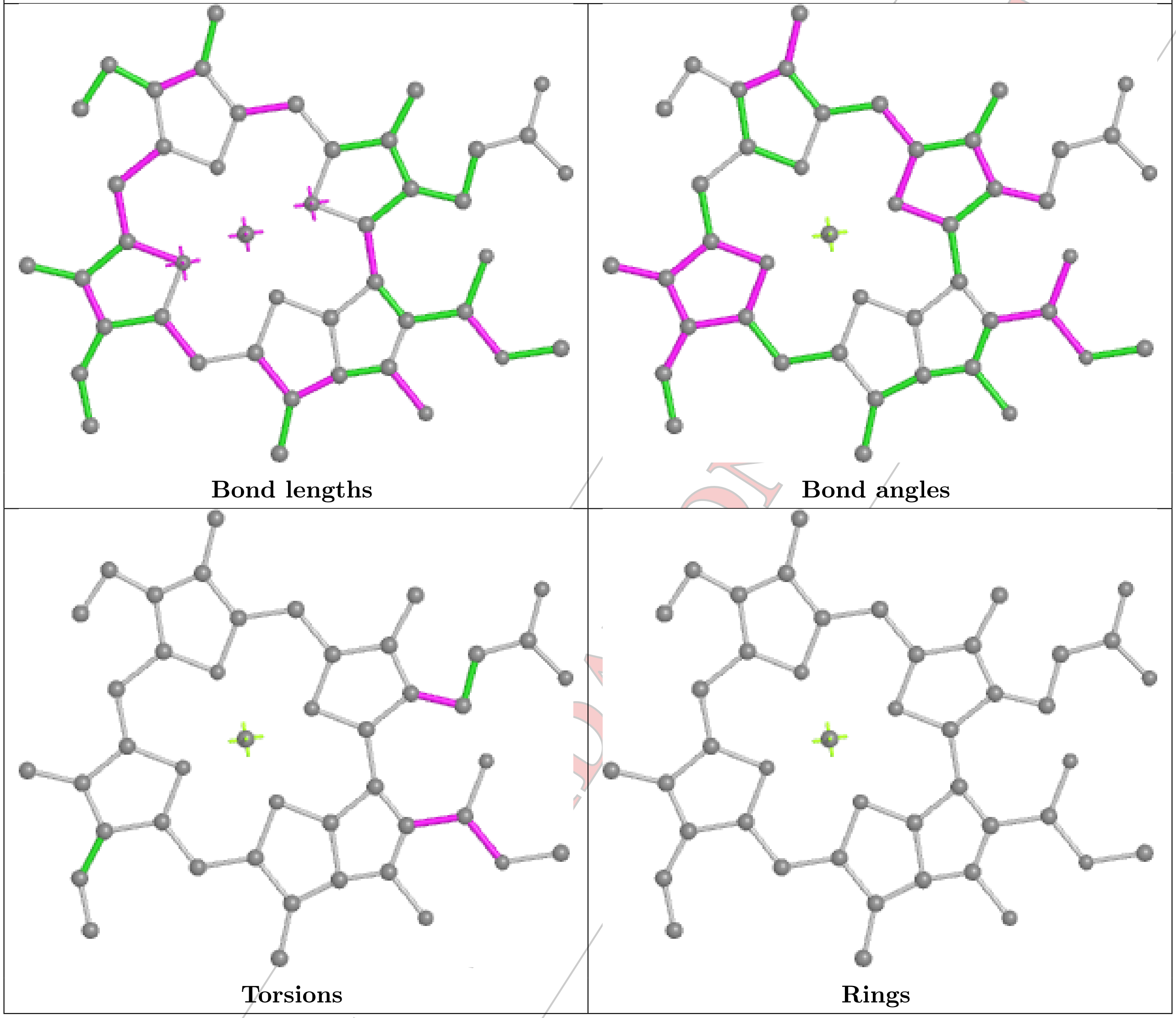

PRELIMINARY

Ligand CLA BA 1220

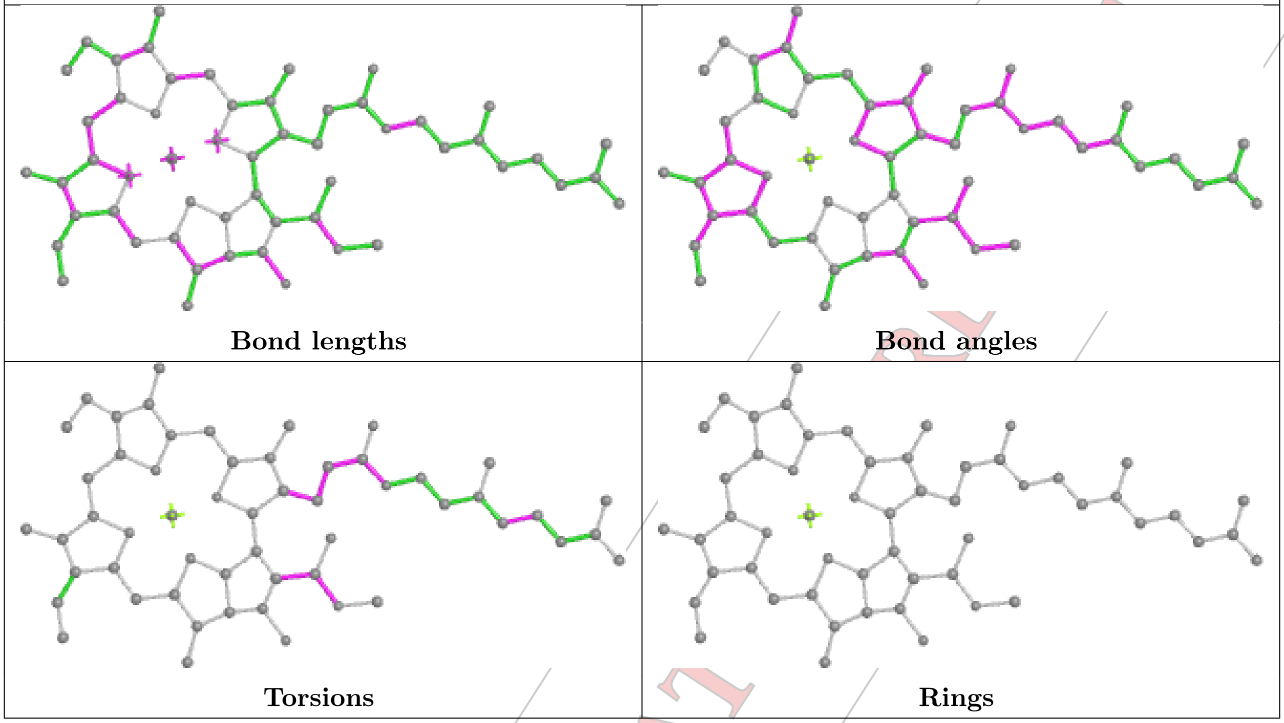

Ligand CLA BA 1221

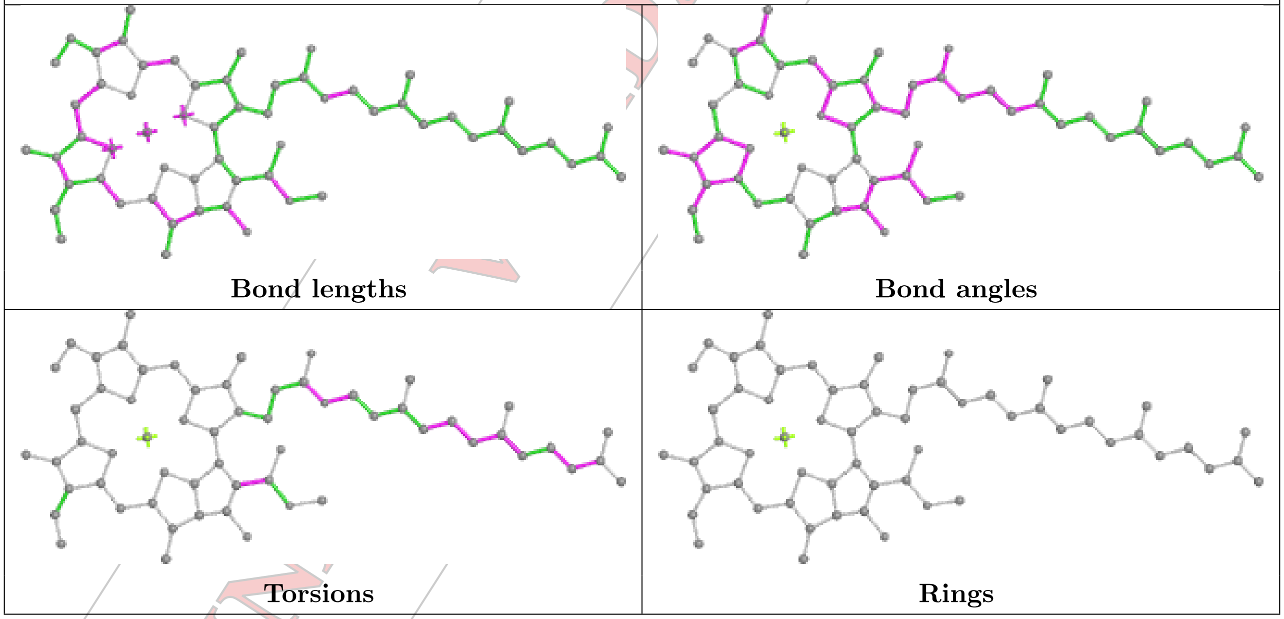

Ligand CLA BA 1222

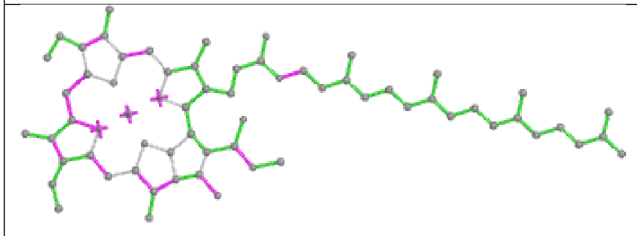
Bond lengths

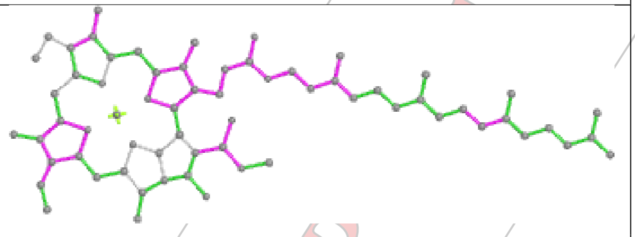
Bond angles

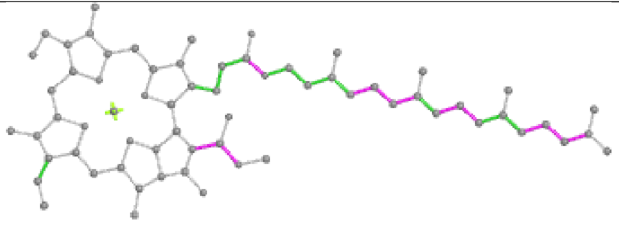
Torsions

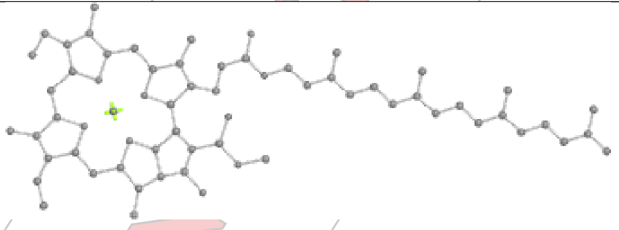
Rings

Ligand CLA BA 1223

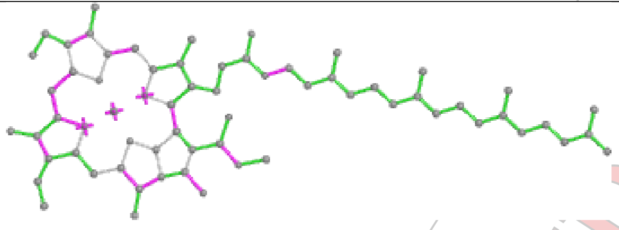
Bond lengths

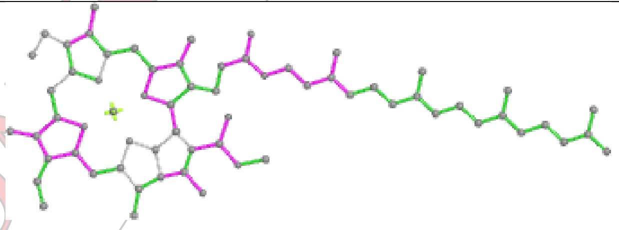
Bond angles

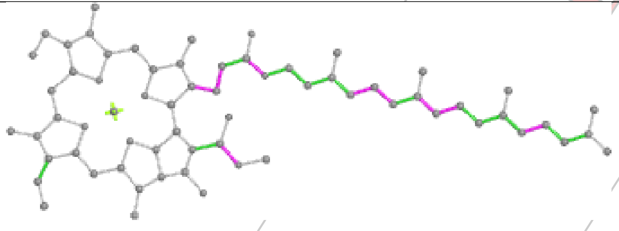
Torsions

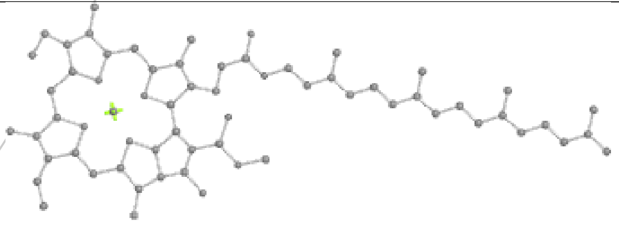
Rings

Ligand CLA BA 1224

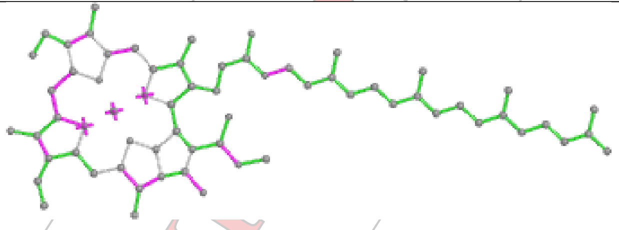
Bond lengths

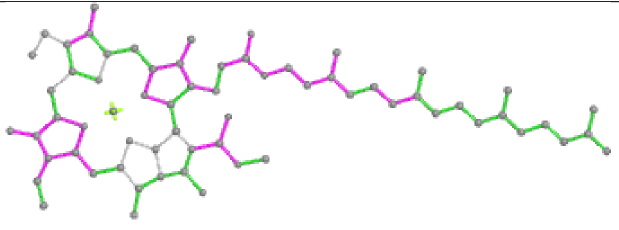
Bond angles

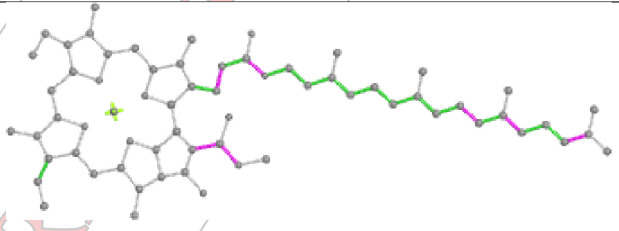
Torsions

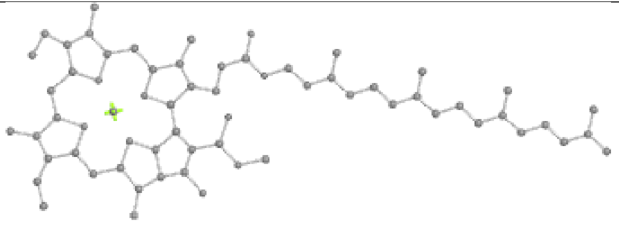
Rings

Ligand CLA BA 1225

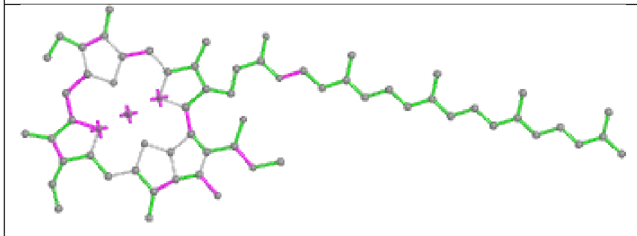

Bond lengths

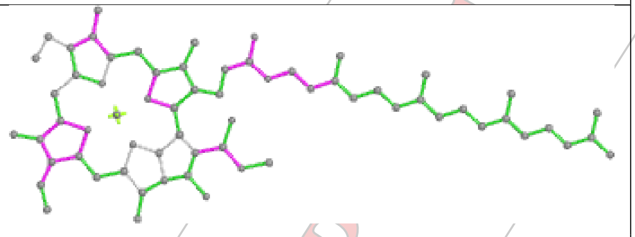

Bond angles

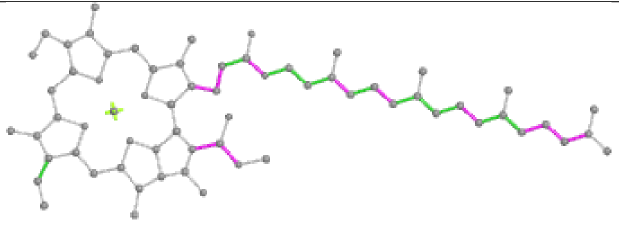

Torsions

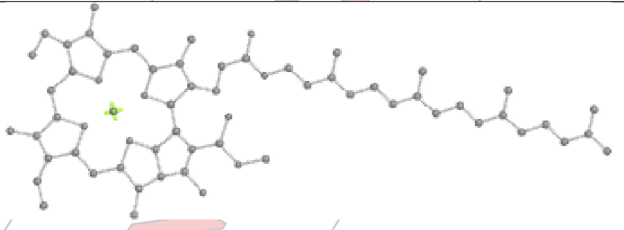

Rings

Ligand CLA BA 1226

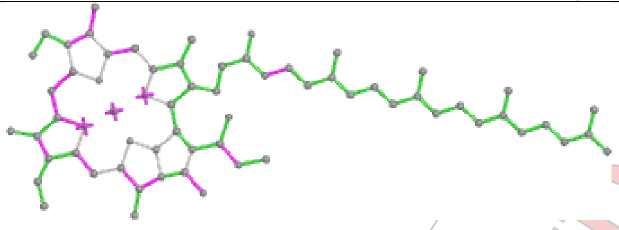

Bond lengths

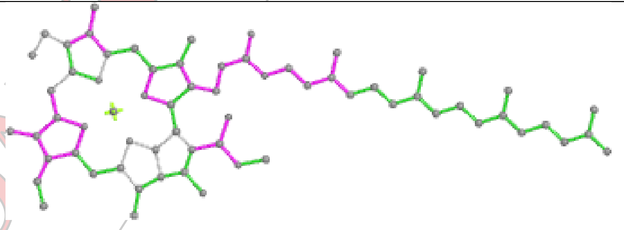

Bond angles

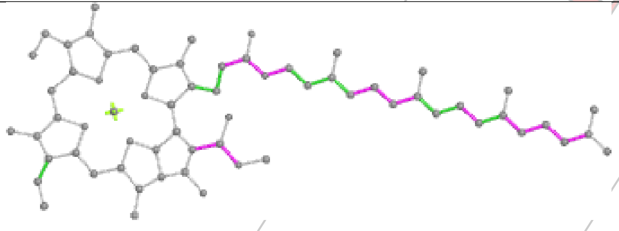

Torsions

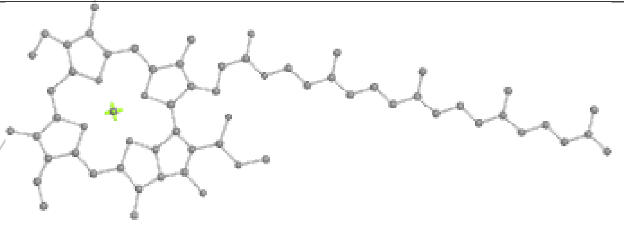

Rings

PRELIMINARY

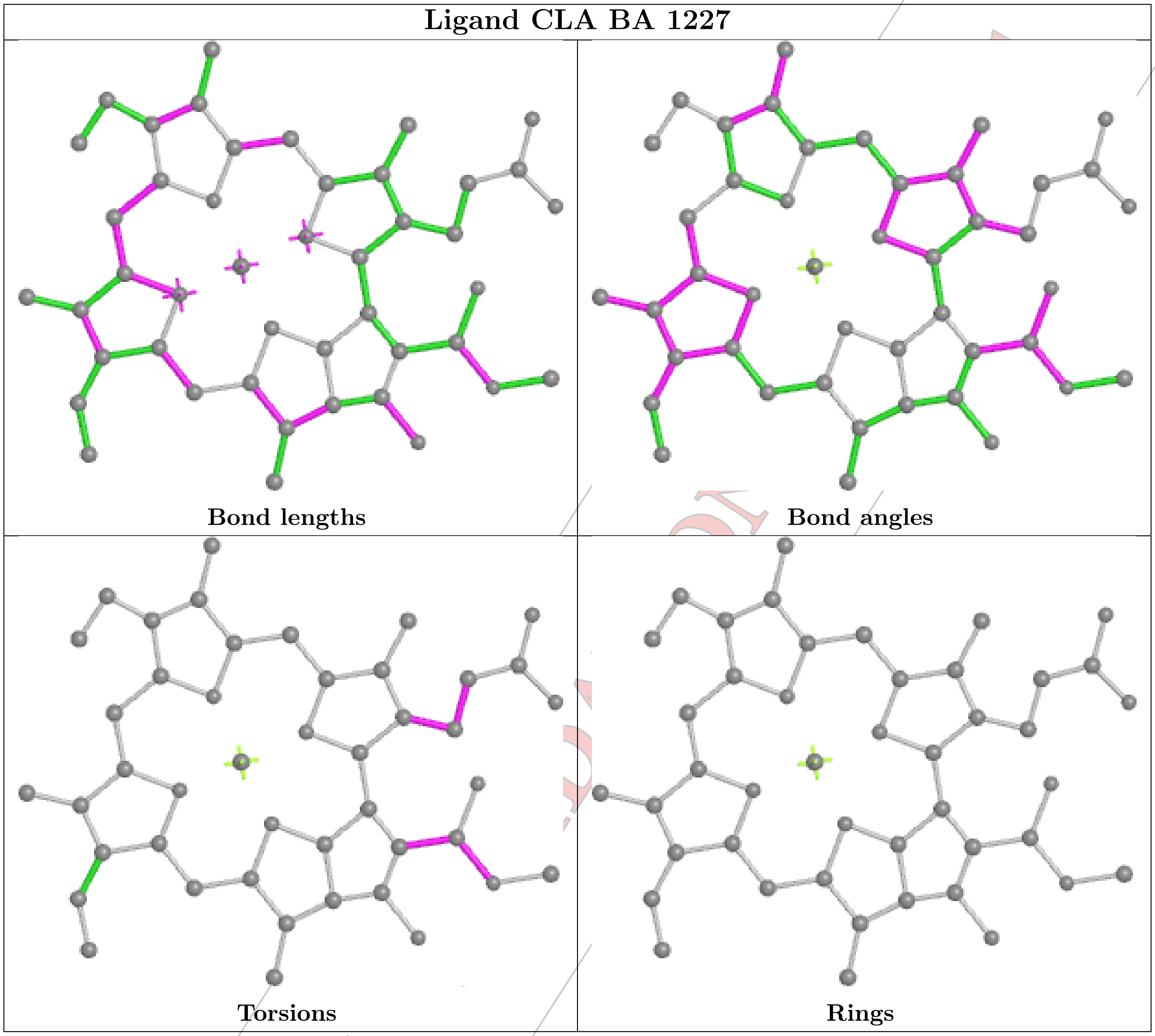

PRELIMINARY

Ligand CLA BA 1228

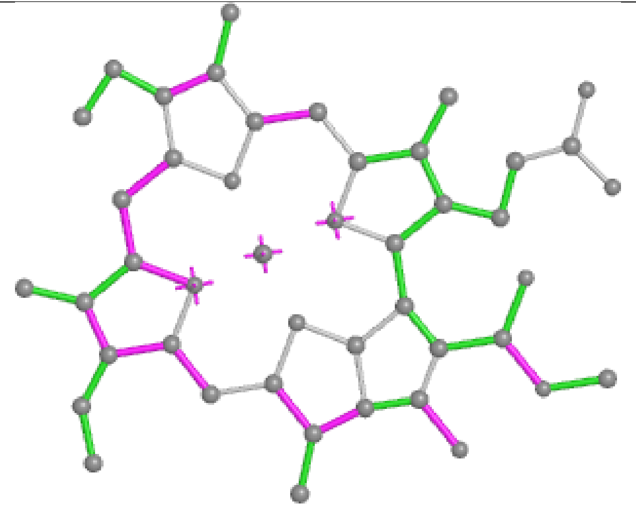
Bond lengths

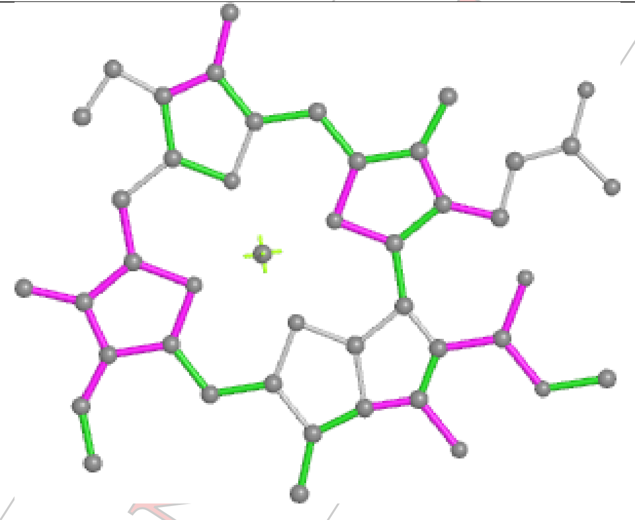
Bond angles

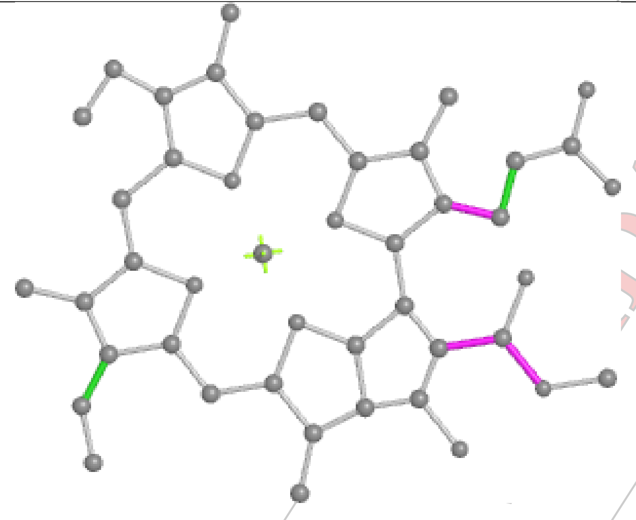
Torsions

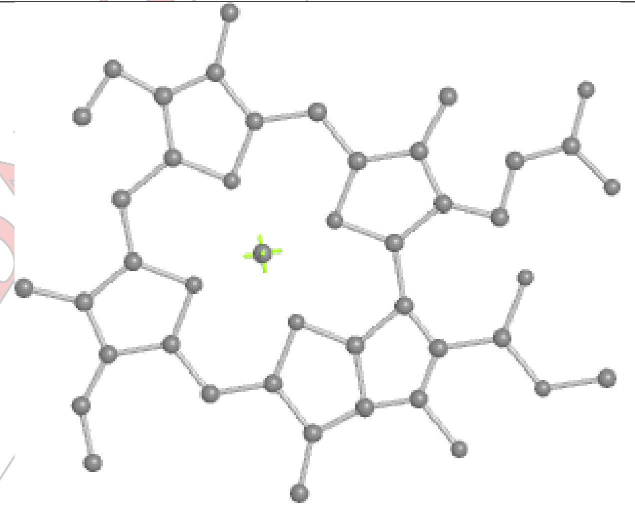
Rings

Ligand CLA BA 1229

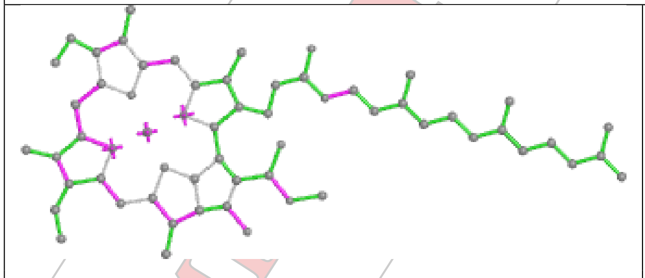
Bond lengths

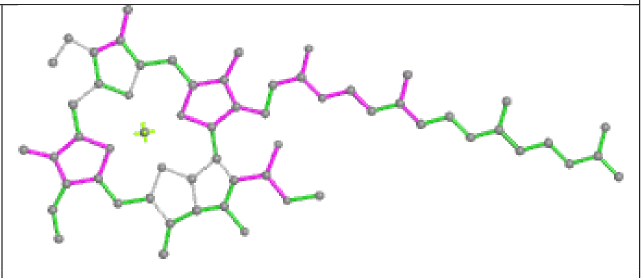
Bond angles

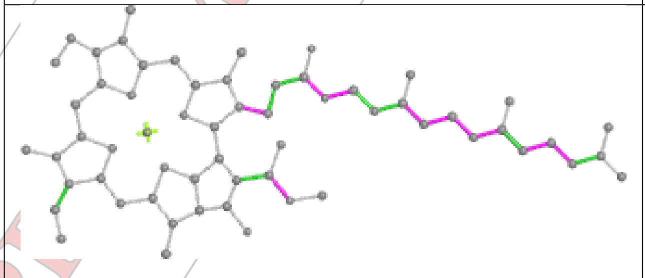
Torsions

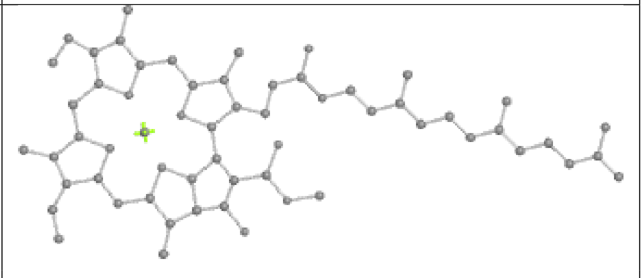
Rings

Ligand CLA BA 1231	
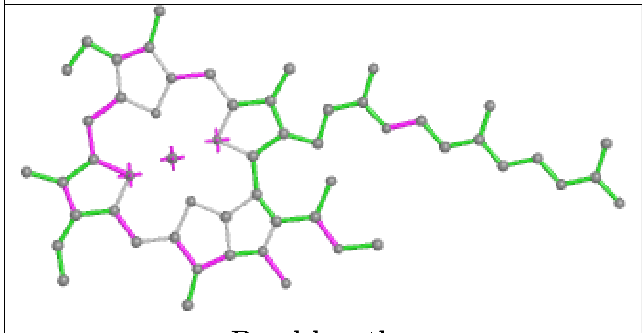 Bond lengths	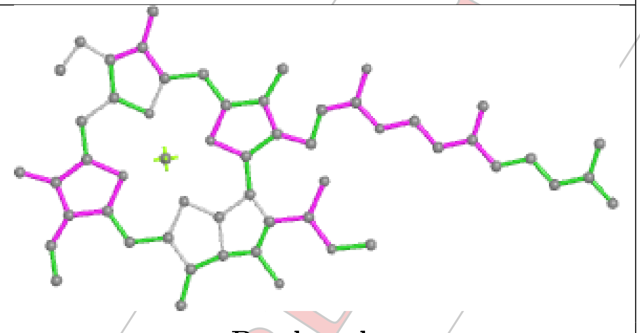 Bond angles
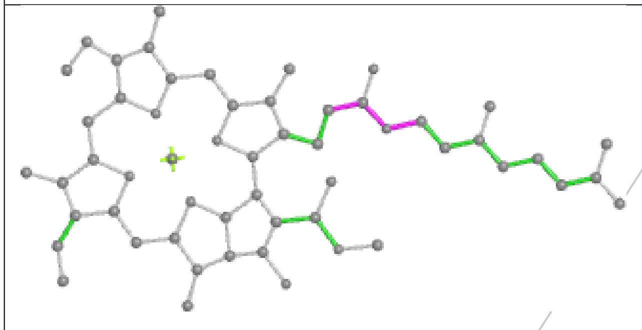 Torsions	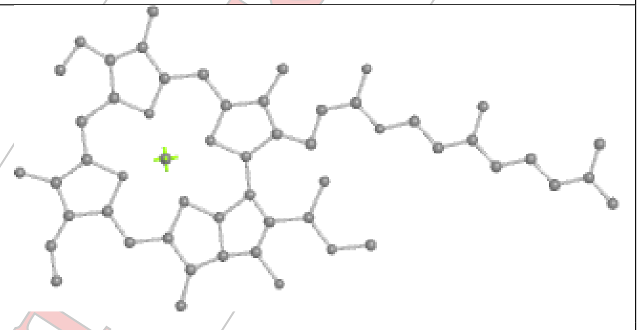 Rings

PRELIMINARY VALIDATION

Ligand CLA BA 1232

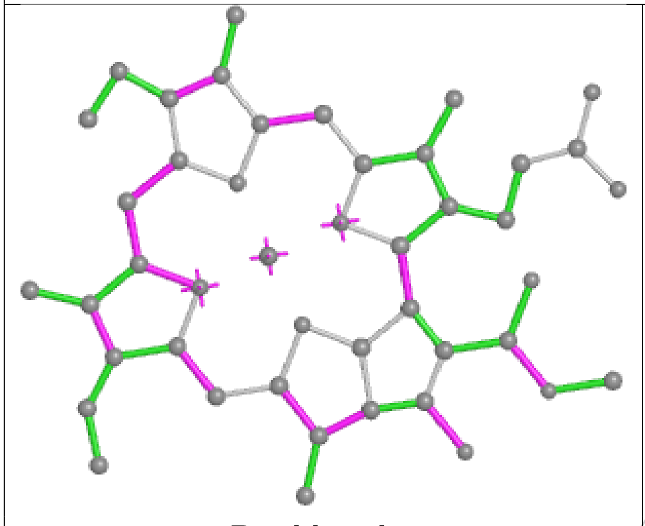
Bond lengths

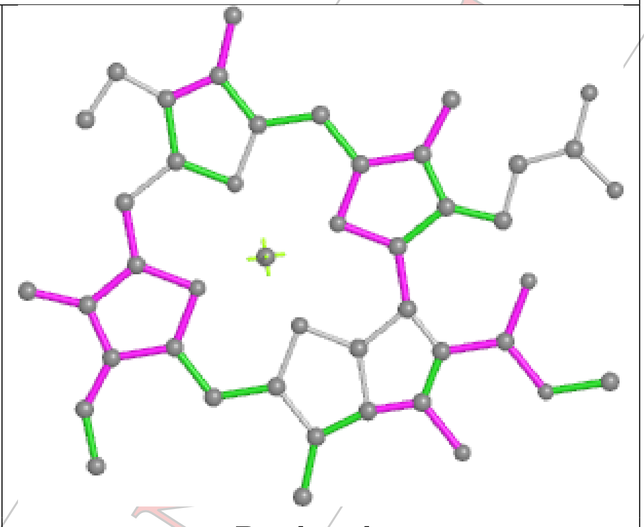
Bond angles

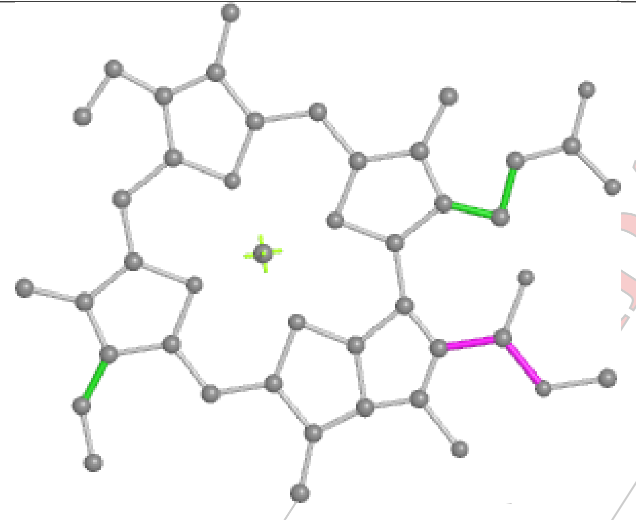
Torsions

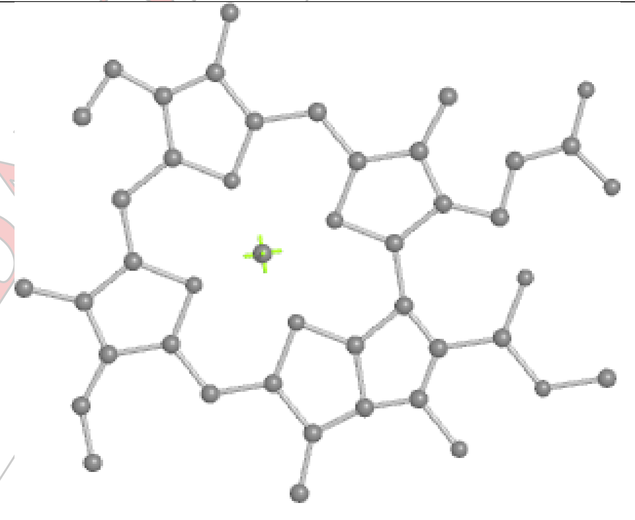
Rings

PRELIMINARY

Ligand CLA BA 1233

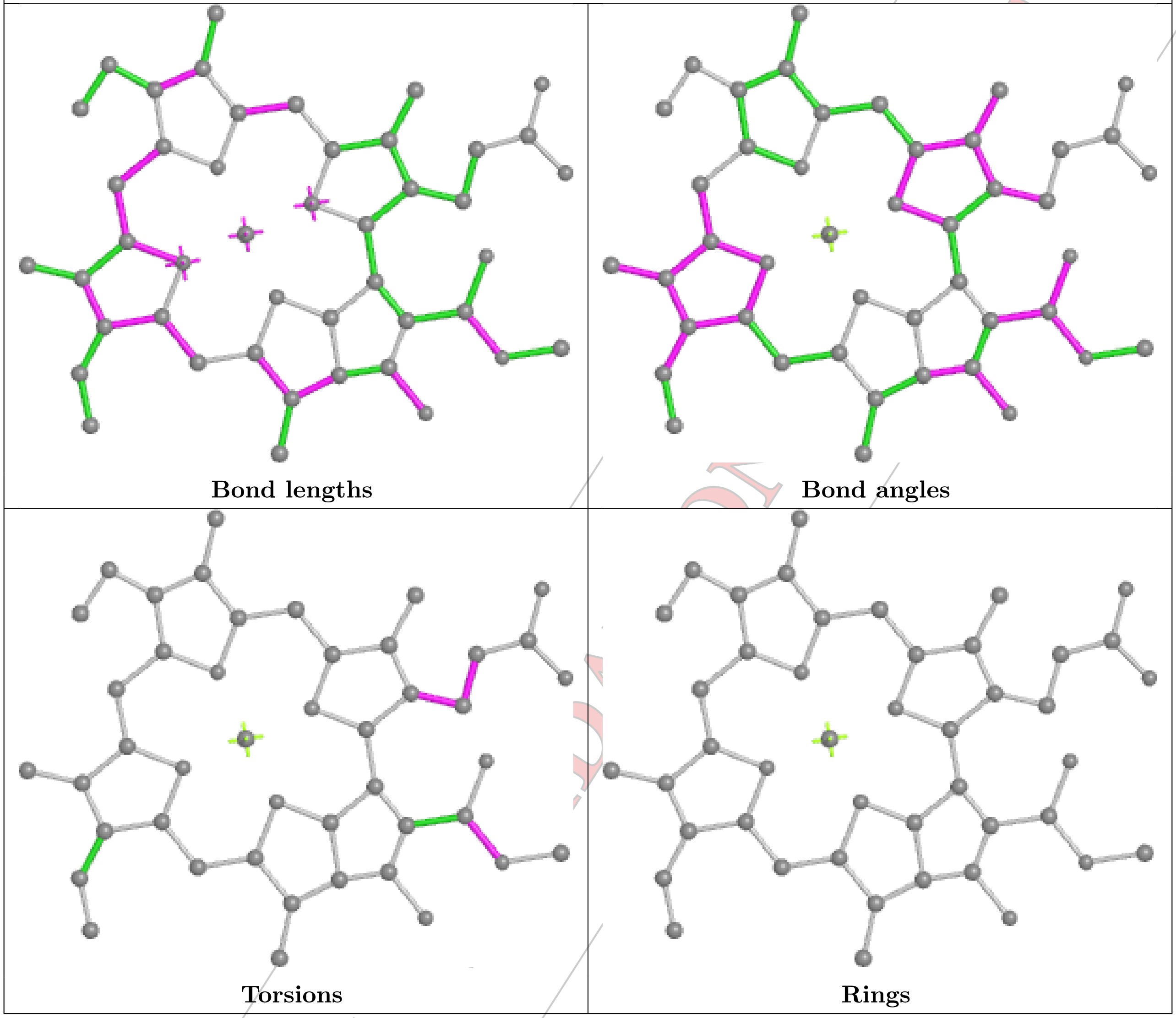

Bond lengths | Bond angles | Torsions | Rings

PRELIMINARY

Ligand CLA BA 1234

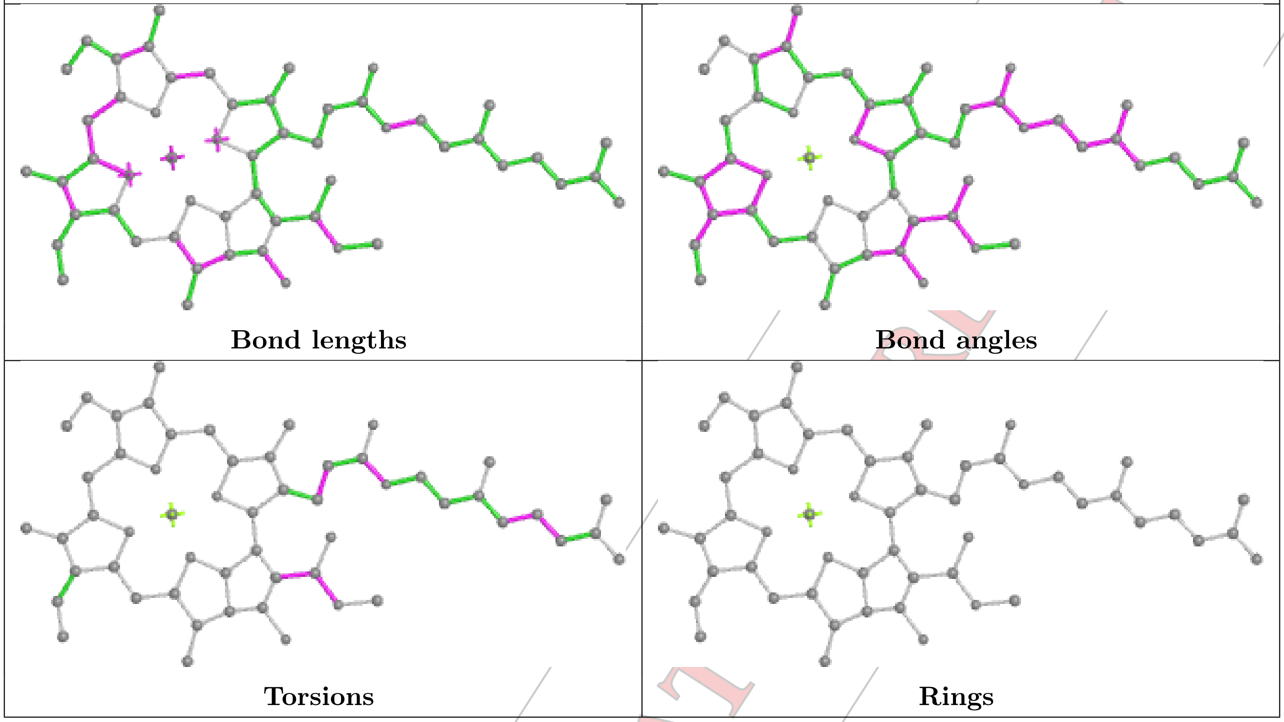

Ligand CLA BA 1235

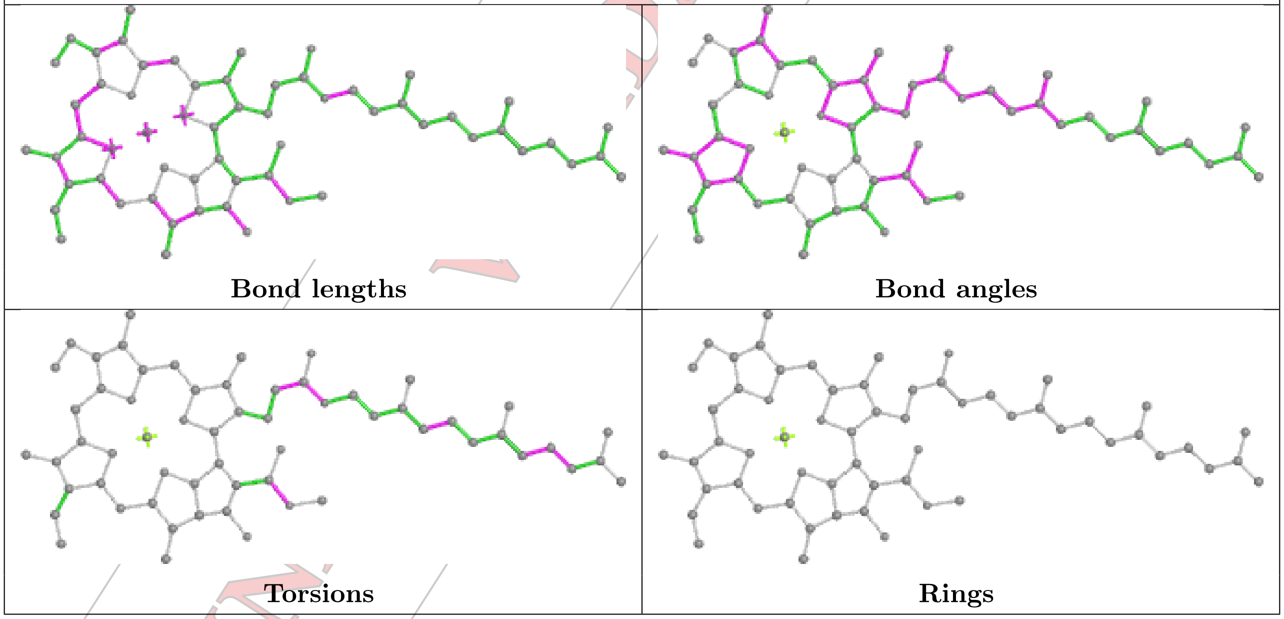

Ligand CLA BA 1236

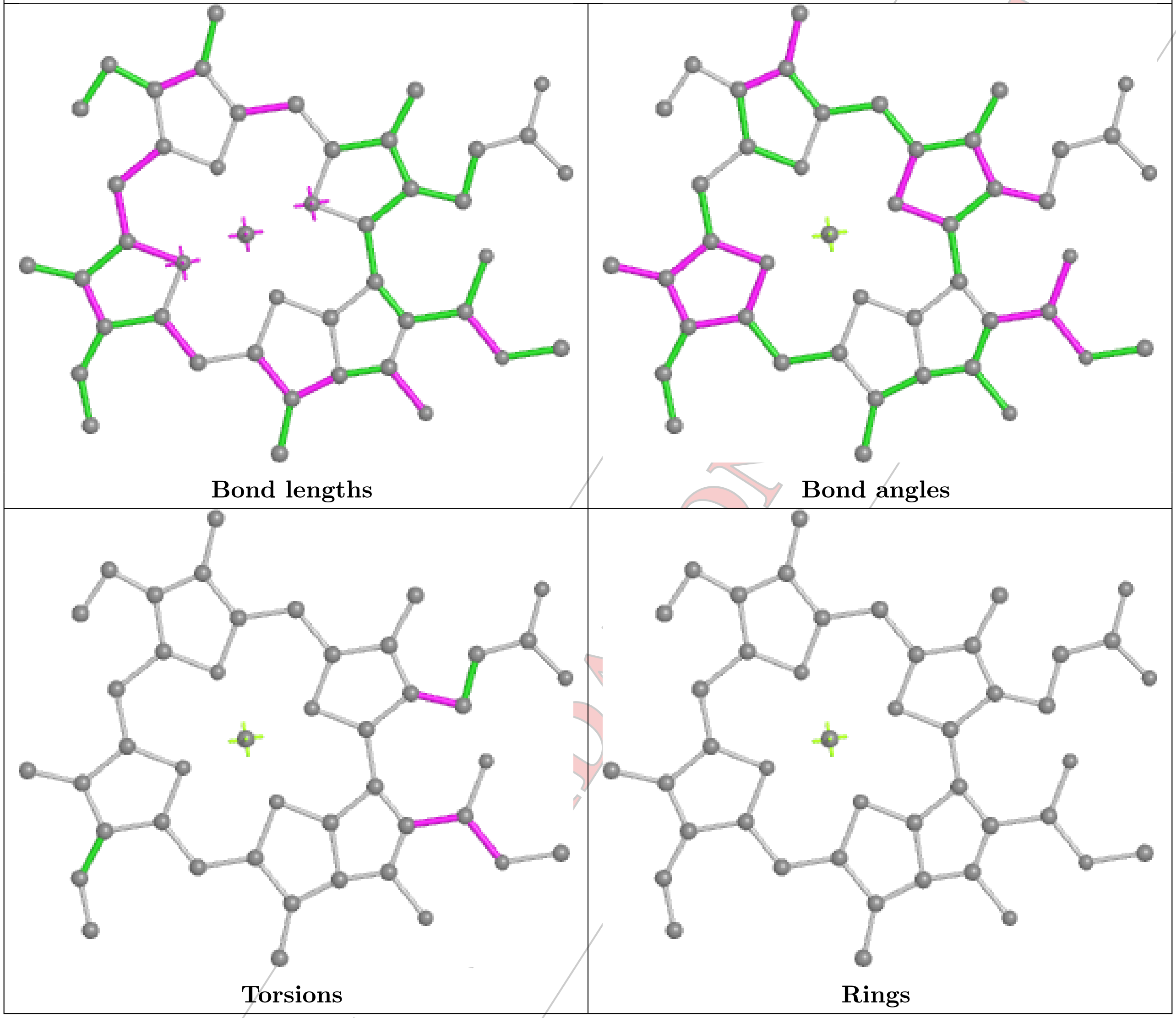

Ligand CLA BA 1238

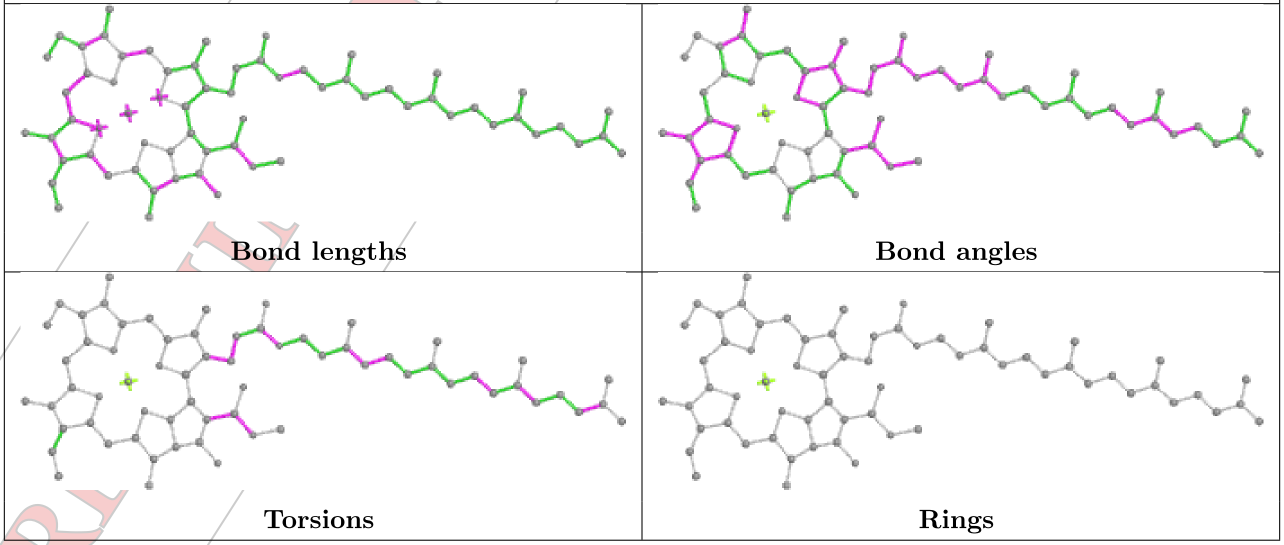

Ligand CLA BA 1239

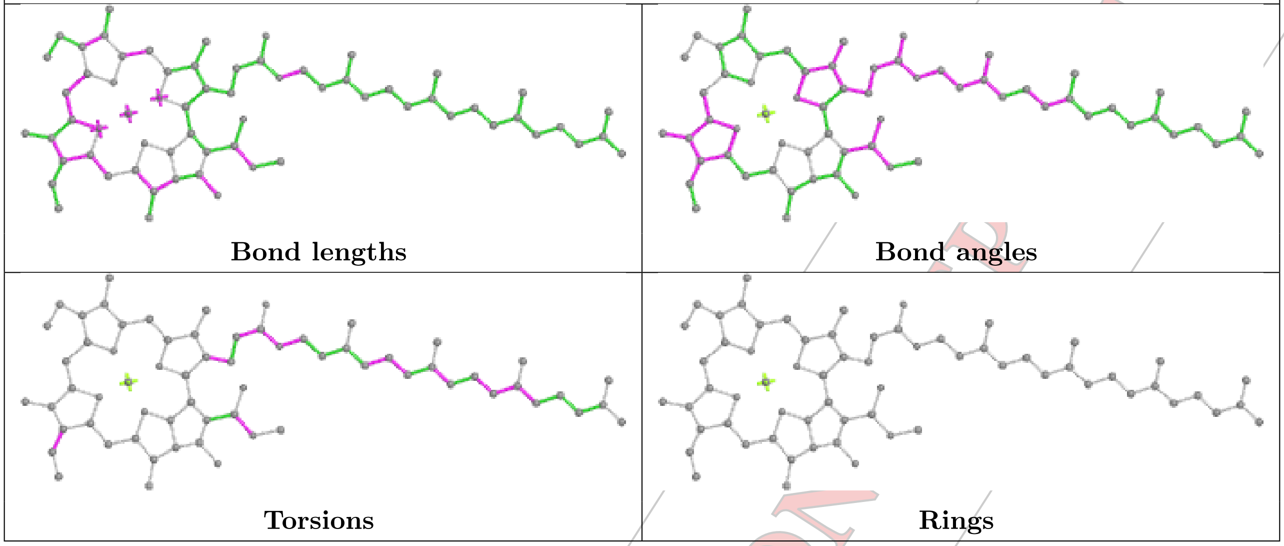

Ligand PQN BA 2002

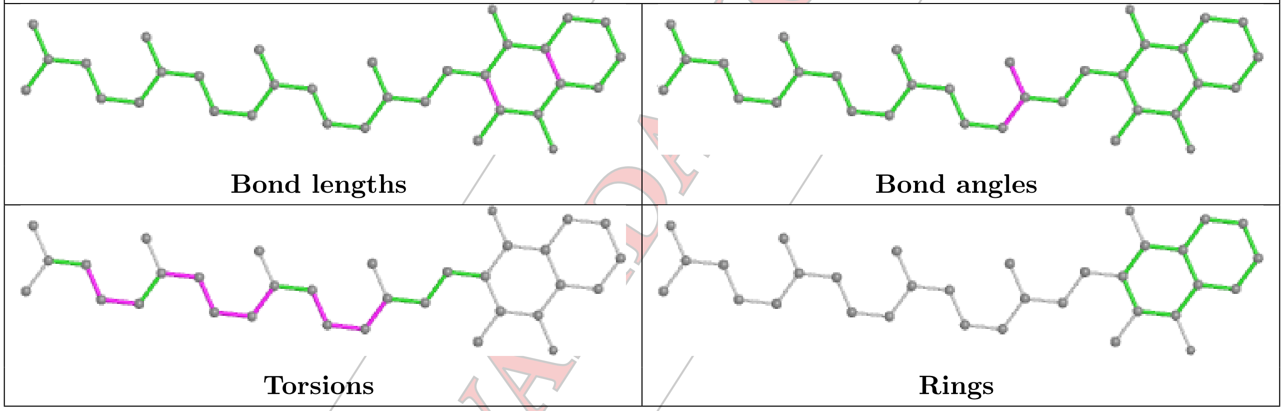

Ligand BCR BA 4004

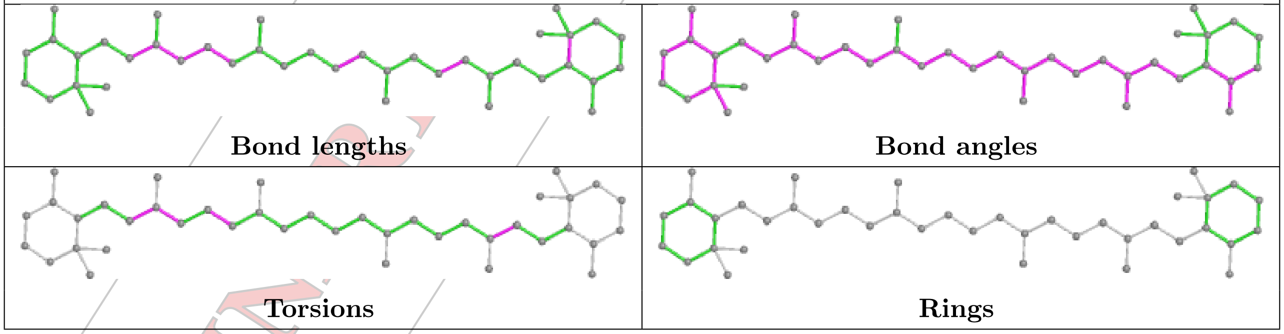

Ligand BCR BA 4005

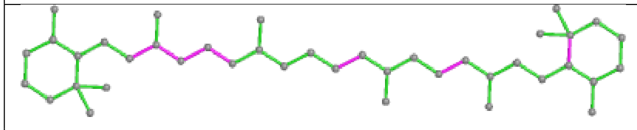

Bond lengths

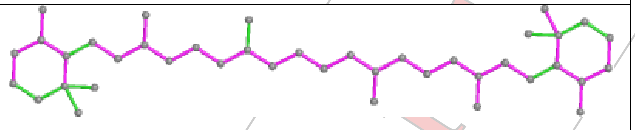

Bond angles

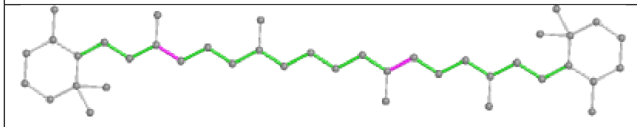

Torsions

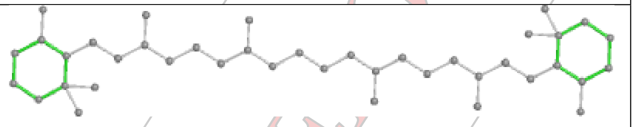

Rings

Ligand BCR BA 4006

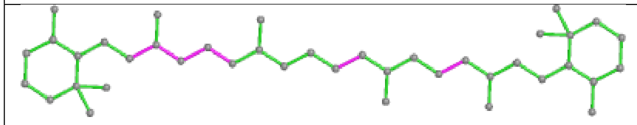

Bond lengths

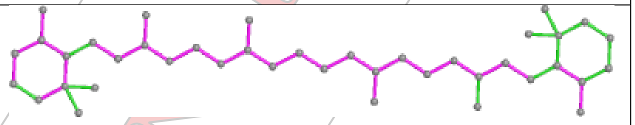

Bond angles

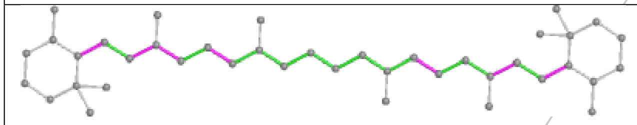

Torsions

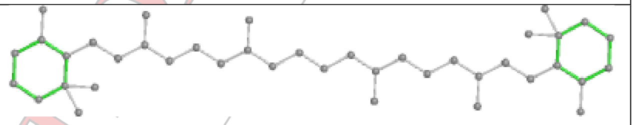

Rings

Ligand BCR BA 4009

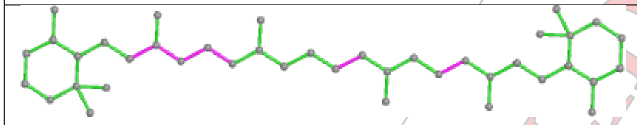

Bond lengths

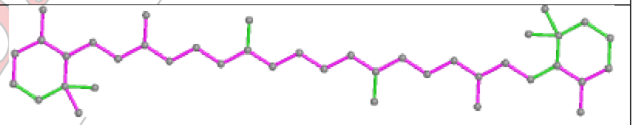

Bond angles

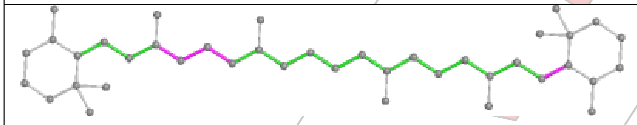

Torsions

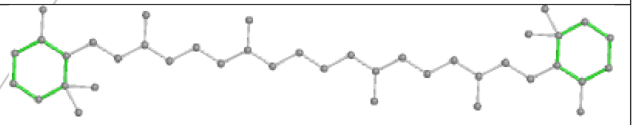

Rings

Ligand BCR BA 4010

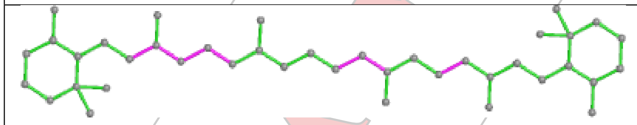

Bond lengths

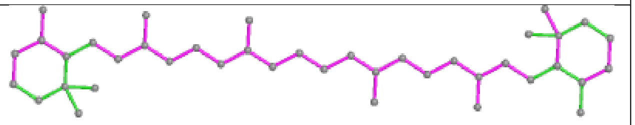

Bond angles

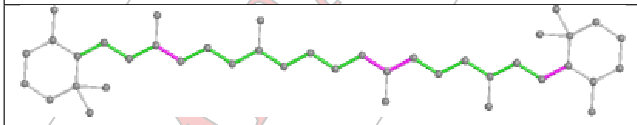

Torsions

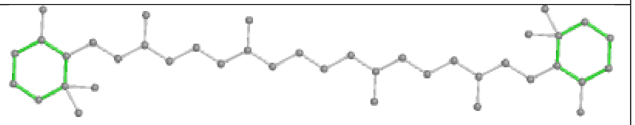

Rings

PRELIMINARY REPORT

Ligand BCR BA 4012

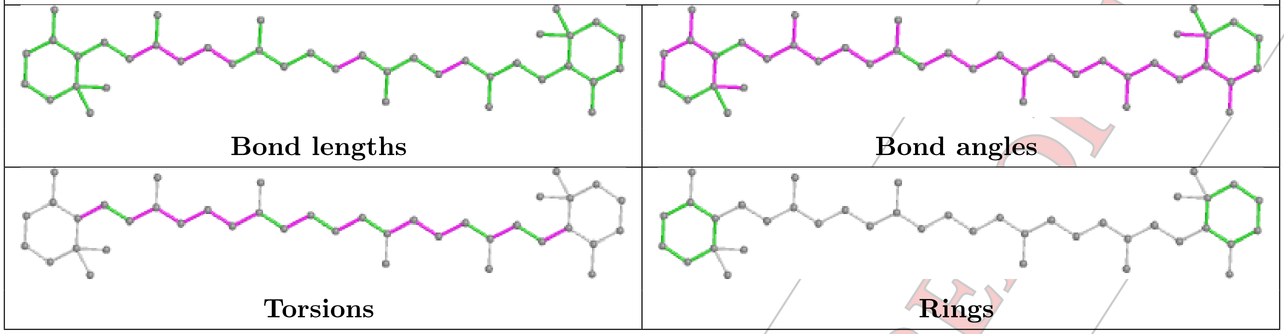

Ligand BCR BA 4013

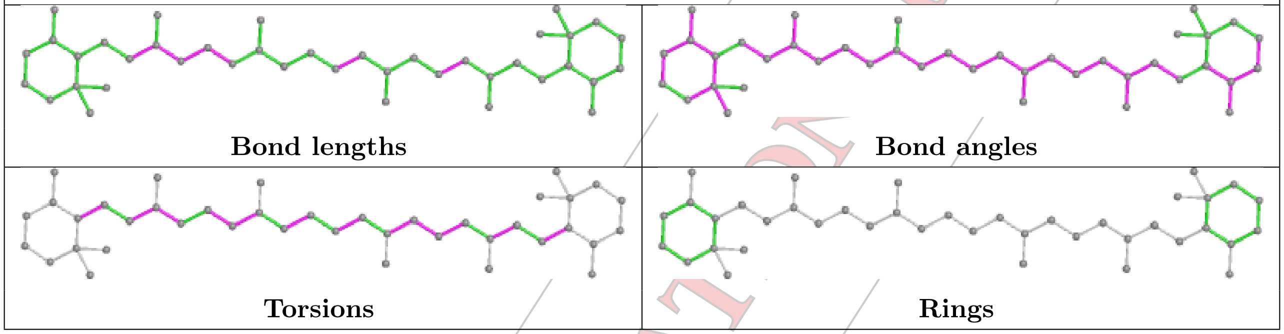

Ligand BCR BA 4014

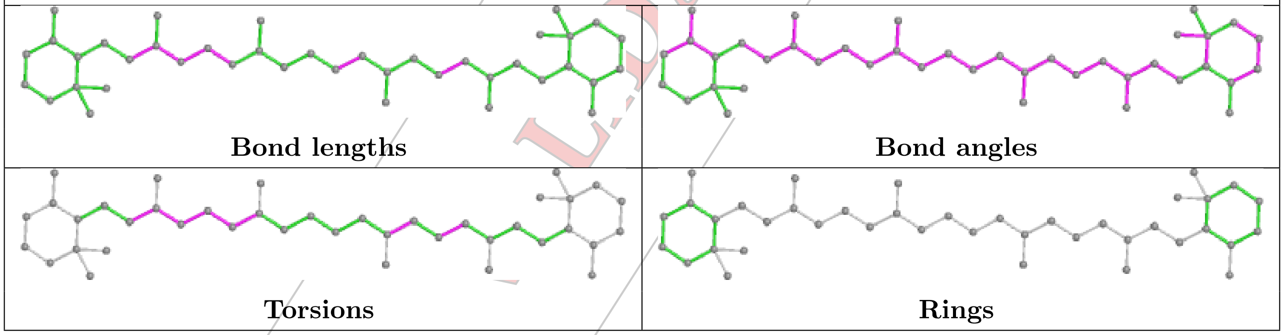

Ligand BCR BA 4016

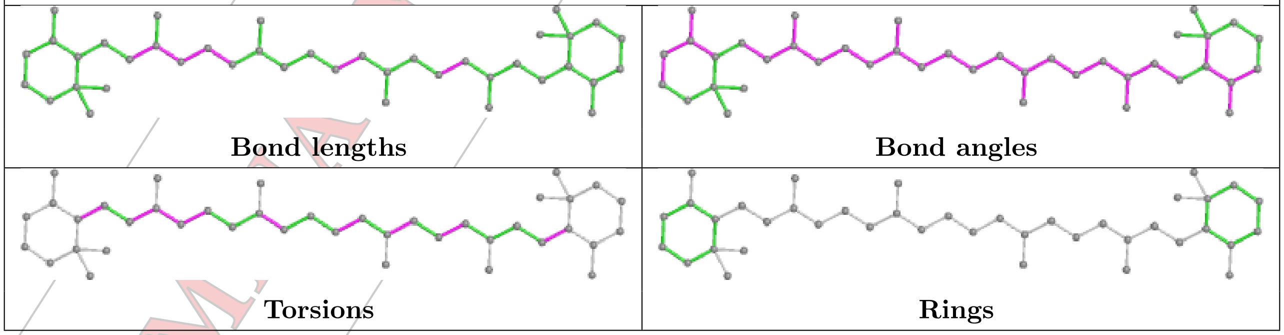

Ligand BCR BA 4017

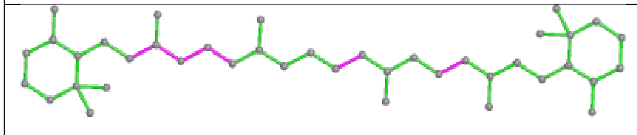

Bond lengths

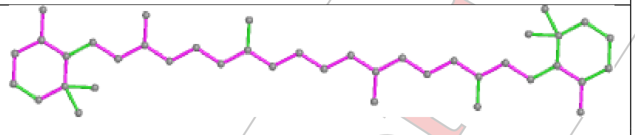

Bond angles

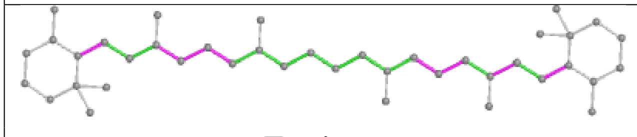

Torsions

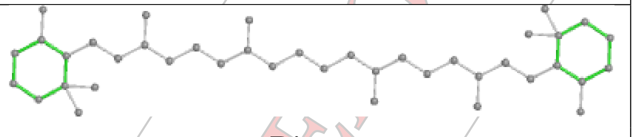

Rings

Ligand LMG BA 5002

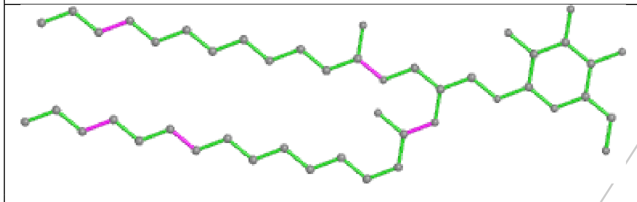

Bond lengths

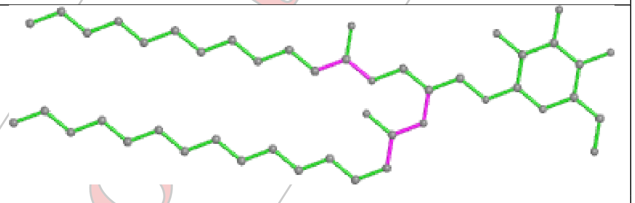

Bond angles

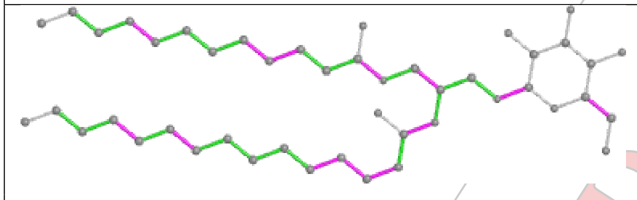

Torsions

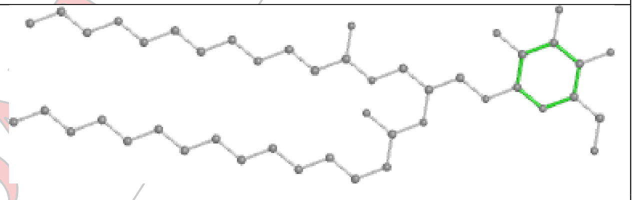

Rings

Ligand LHG BA 5004

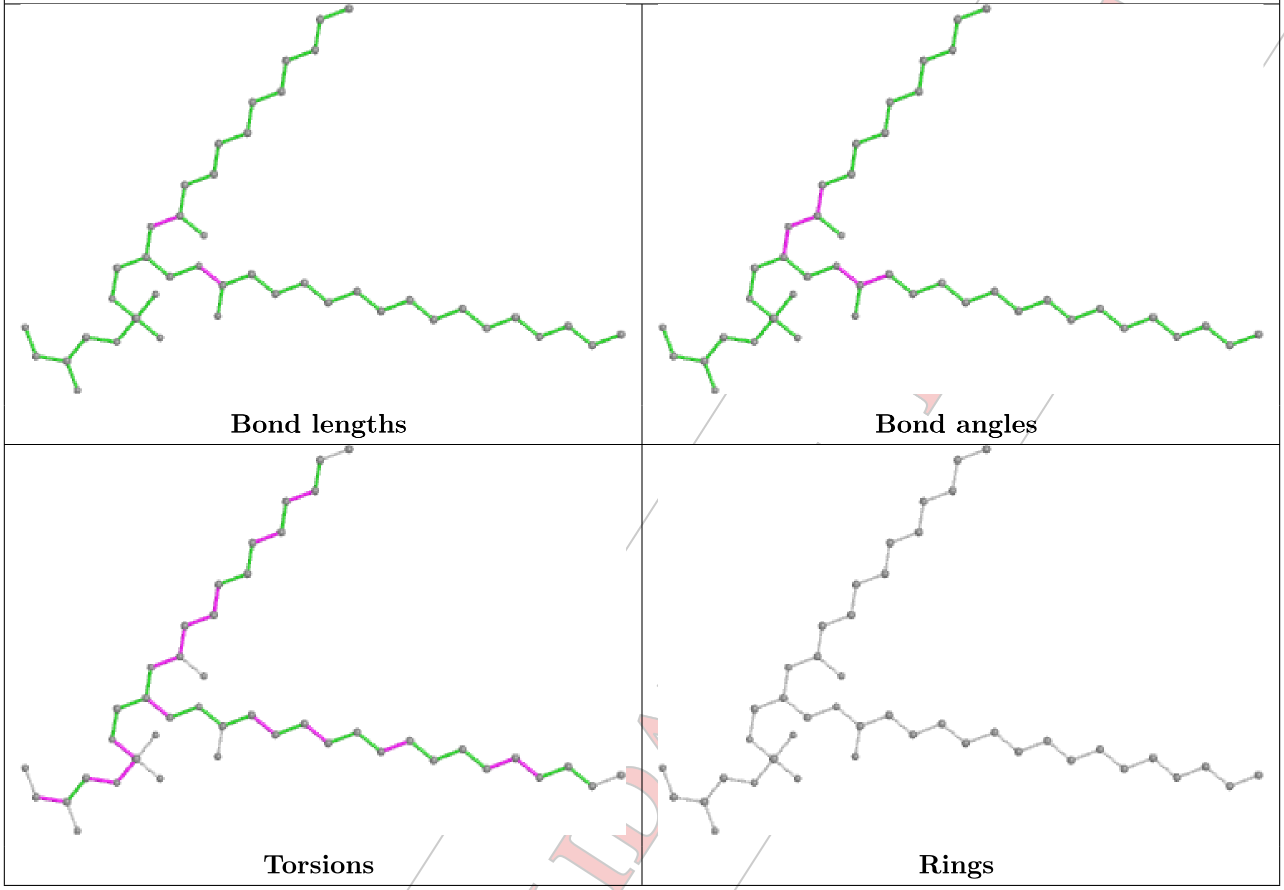

Ligand CL0 GA 1011

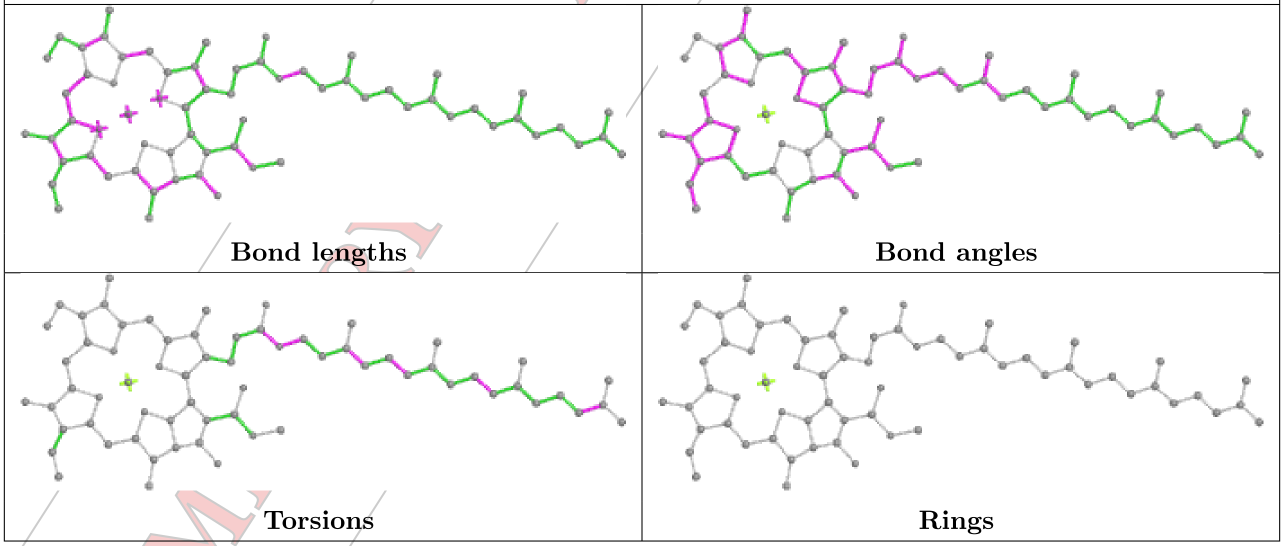

Ligand CLA GA 1012

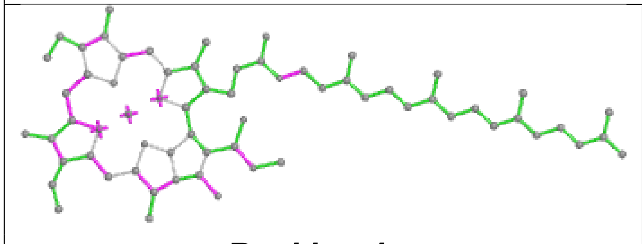

Bond lengths

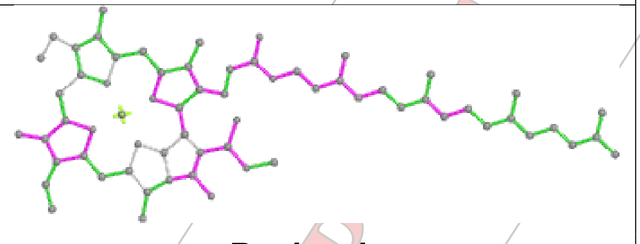

Bond angles

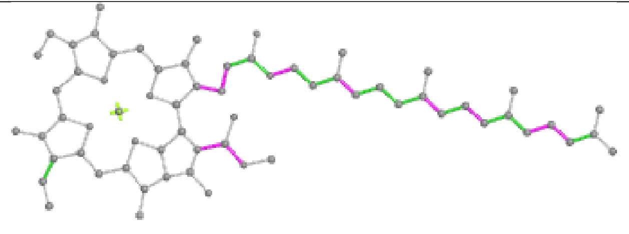

Torsions

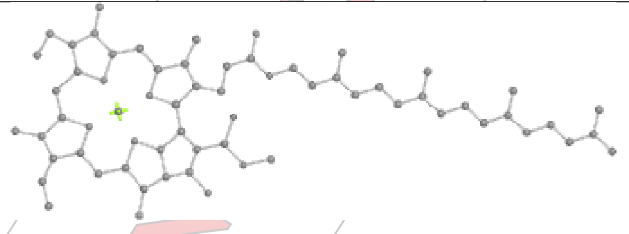

Rings

Ligand CLA GA 1013

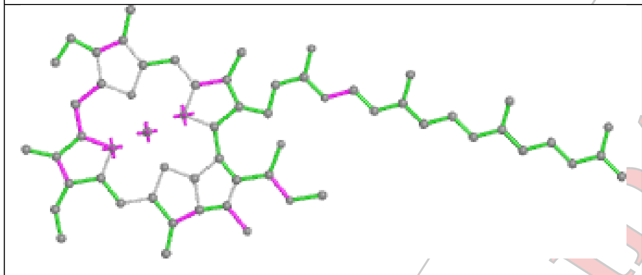

Bond lengths

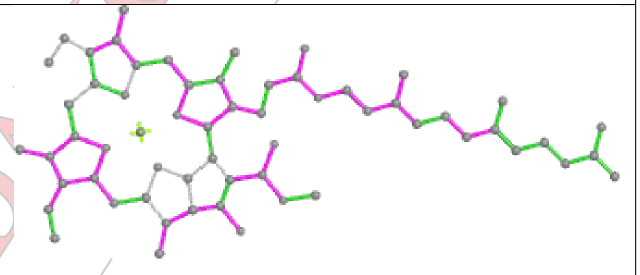

Bond angles

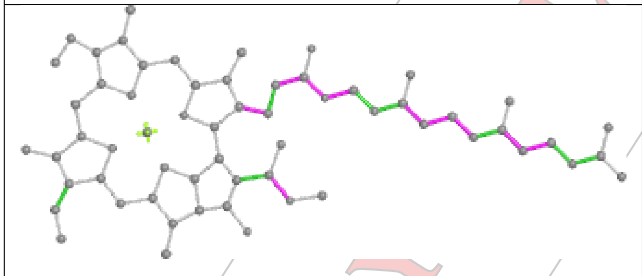

Torsions

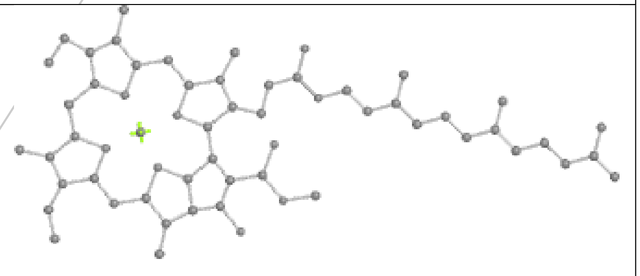

Rings

PRELIMINARY VERSION

Ligand CLA GA 1101

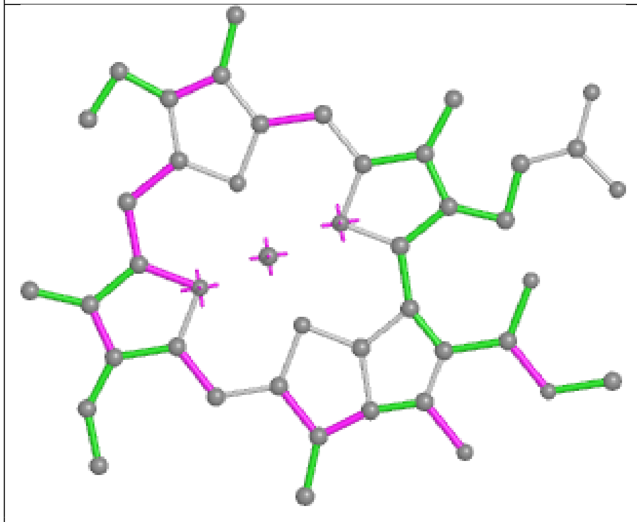

Bond lengths

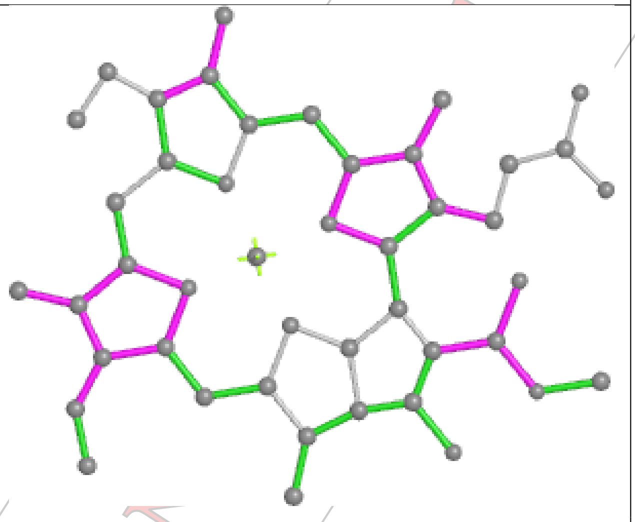

Bond angles

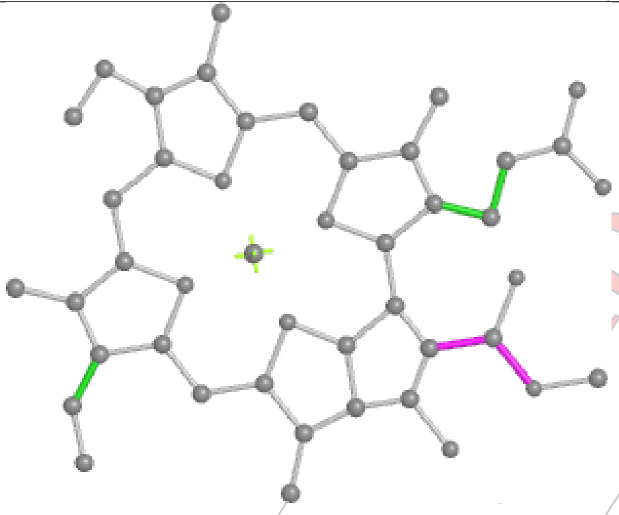

Torsions

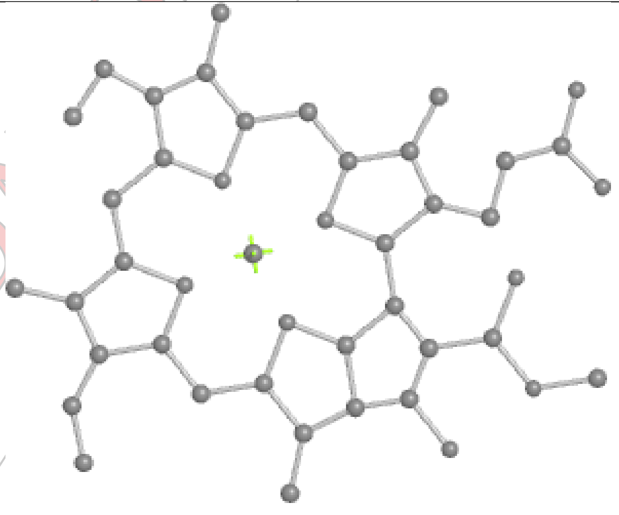

Rings

Ligand CLA GA 1102

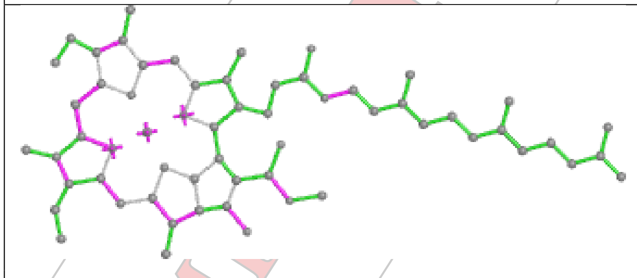

Bond lengths

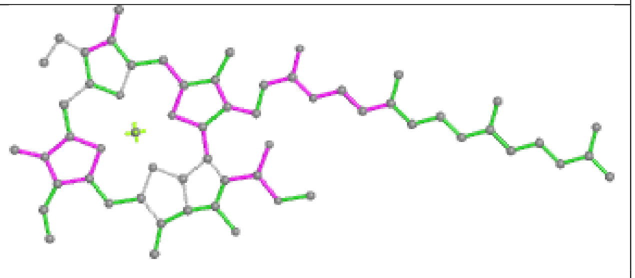

Bond angles

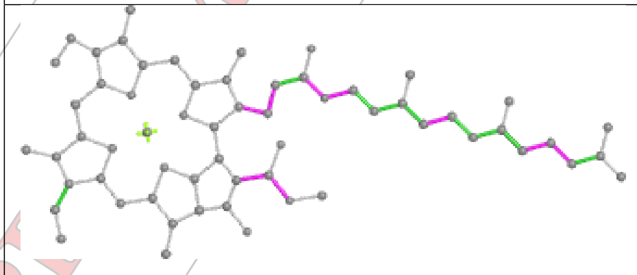

Torsions

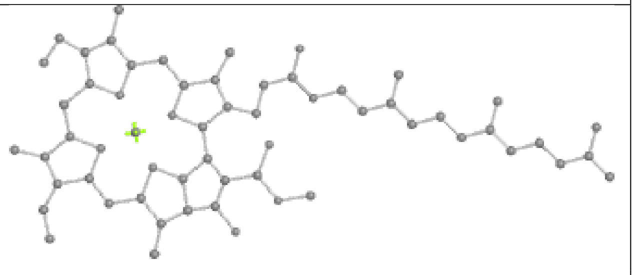

Rings

Ligand CLA GA 1103

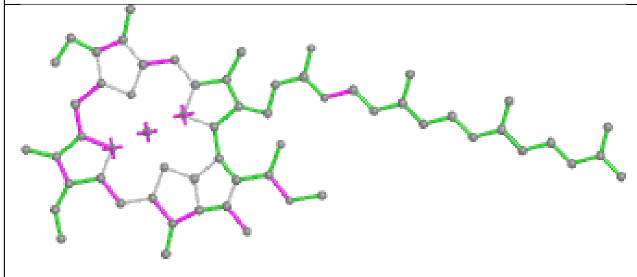

Bond lengths

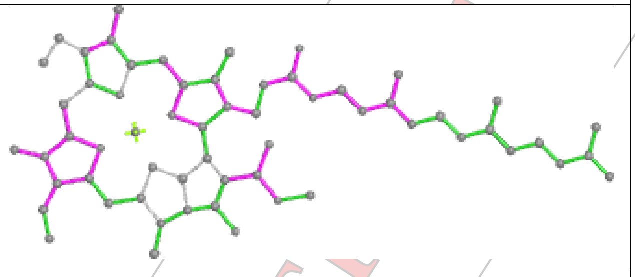

Bond angles

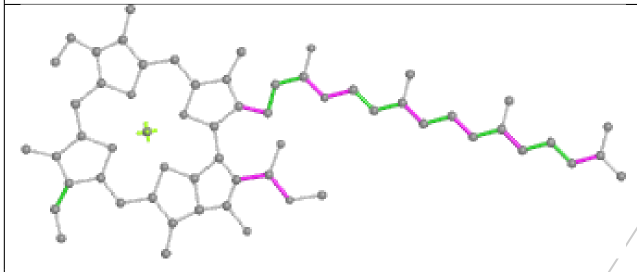

Torsions

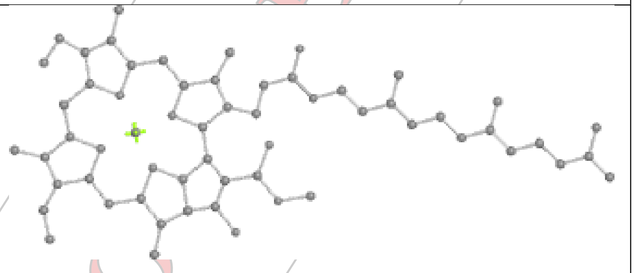

Rings

Ligand CLA GA 1104

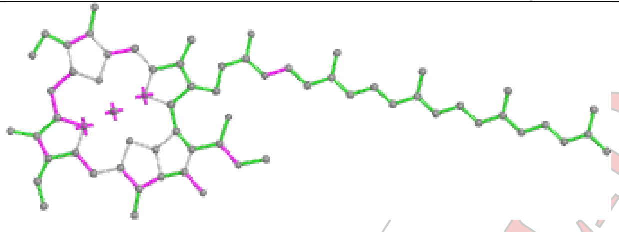

Bond lengths

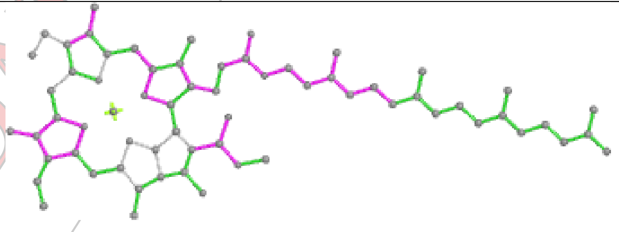

Bond angles

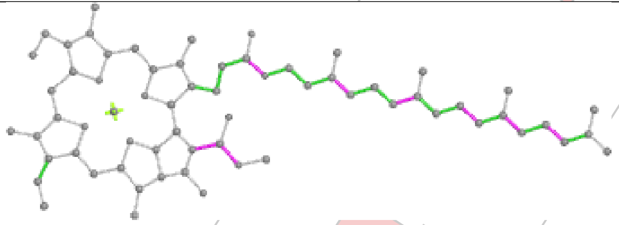

Torsions

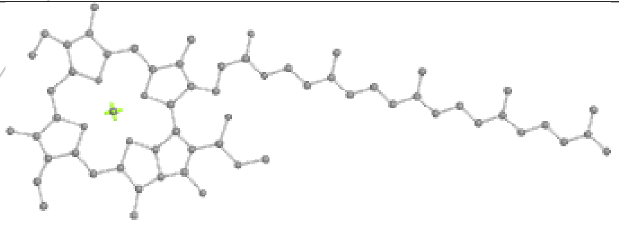

Rings

PRELIMINARY

Ligand CLA GA 1105

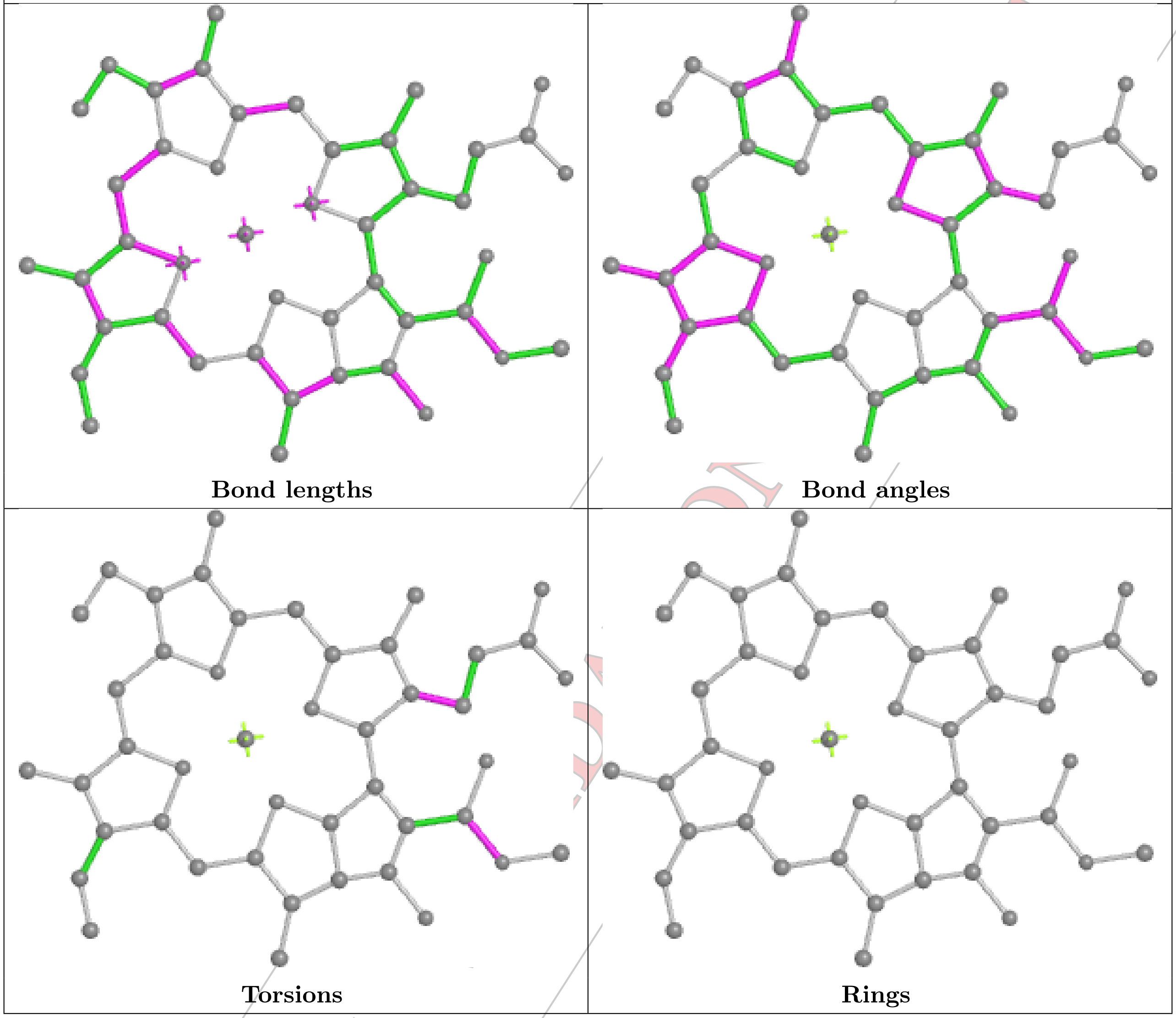

PRELIMINARY

Ligand CLA GA 1106

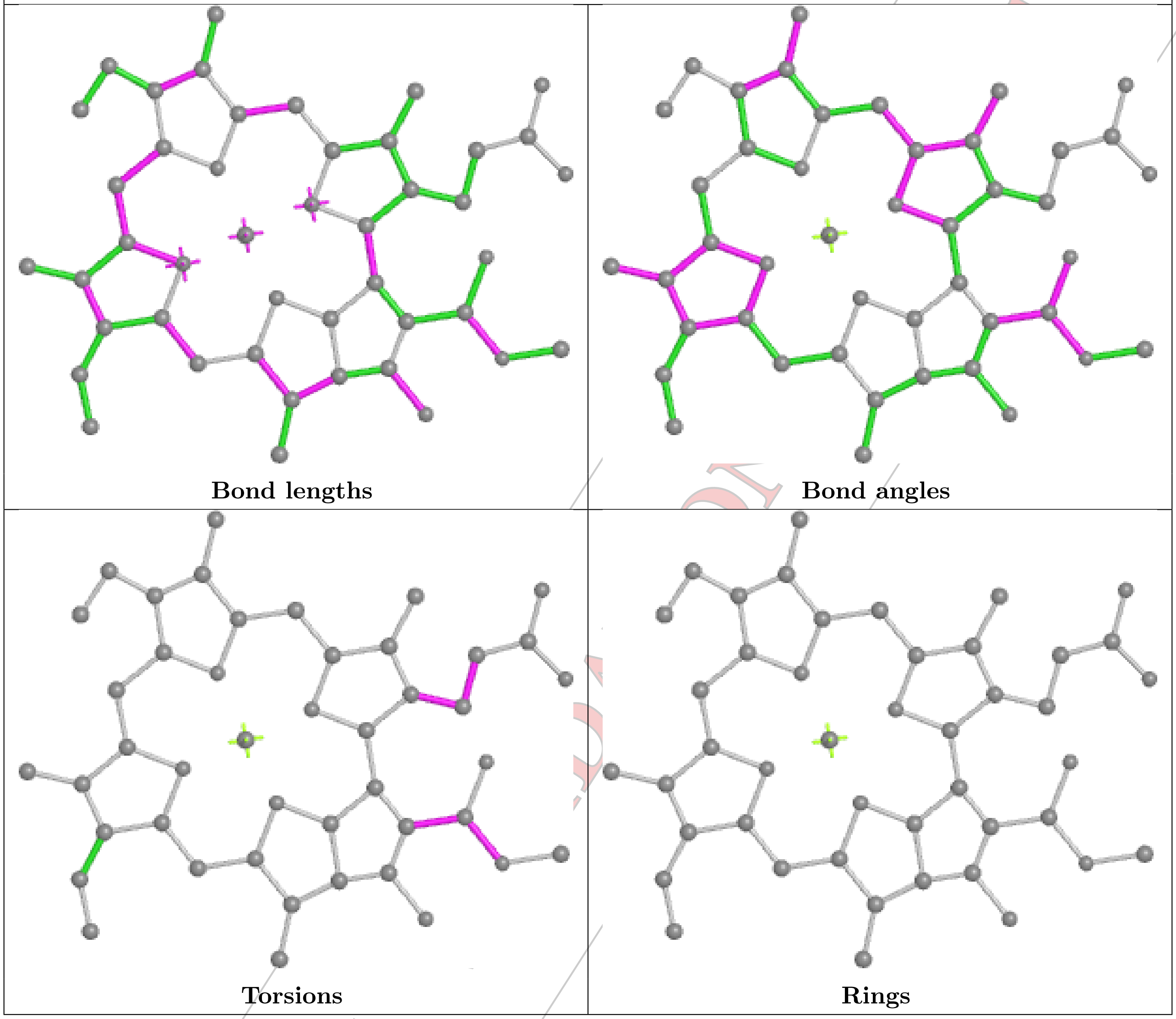

PRELIMINARY

Ligand CLA GA 1107

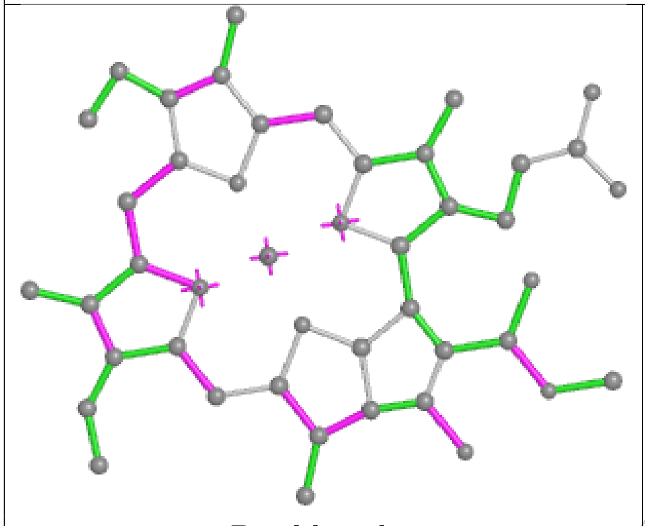

Bond lengths

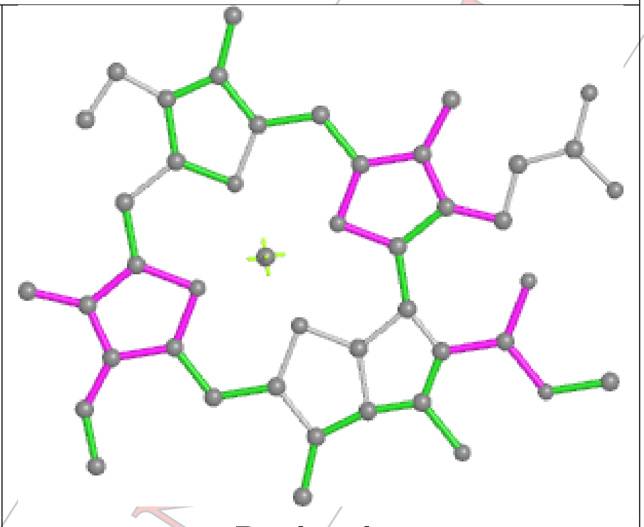

Bond angles

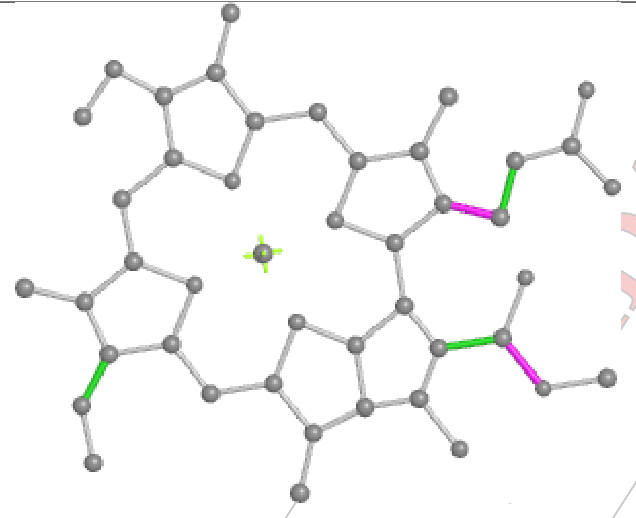

Torsions

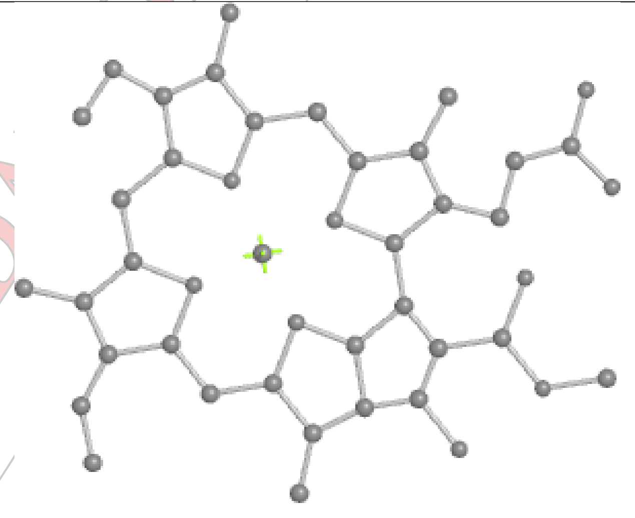

Rings

PRELIMINARY

Ligand CLA GA 1108

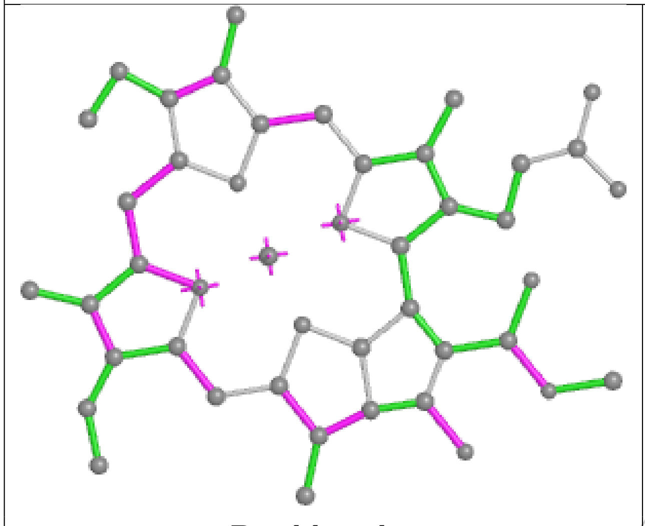

Bond lengths

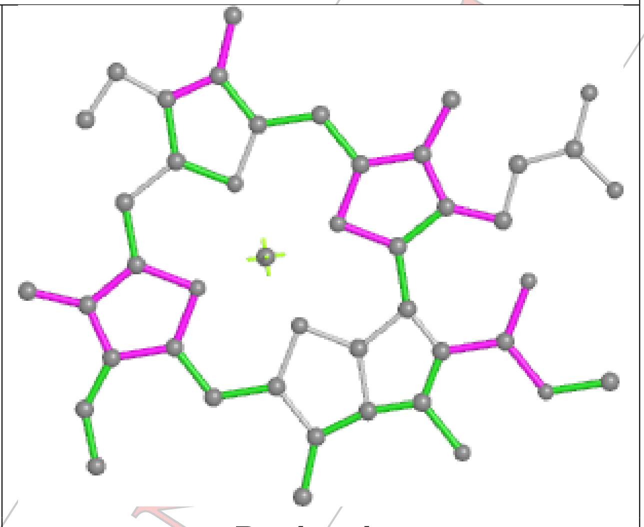

Bond angles

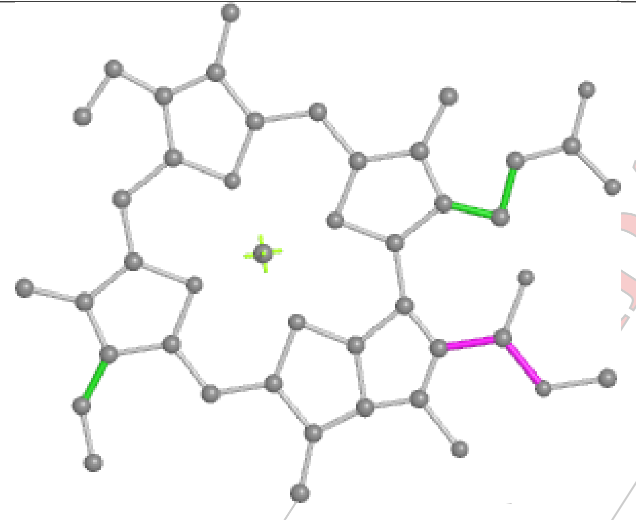

Torsions

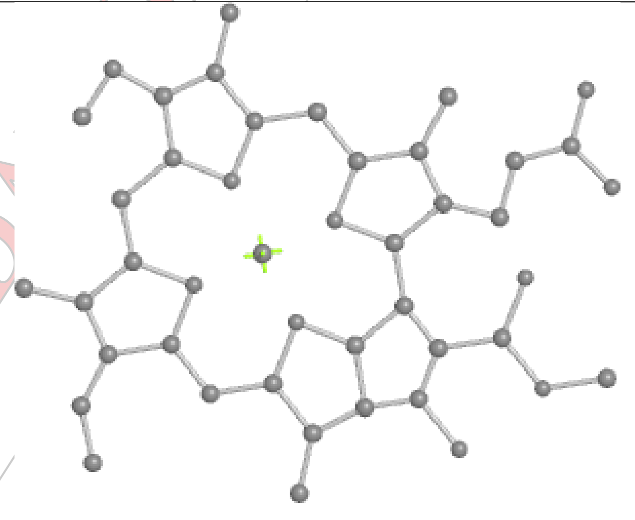

Rings

PRELIMINARY

Ligand CLA GA 1109

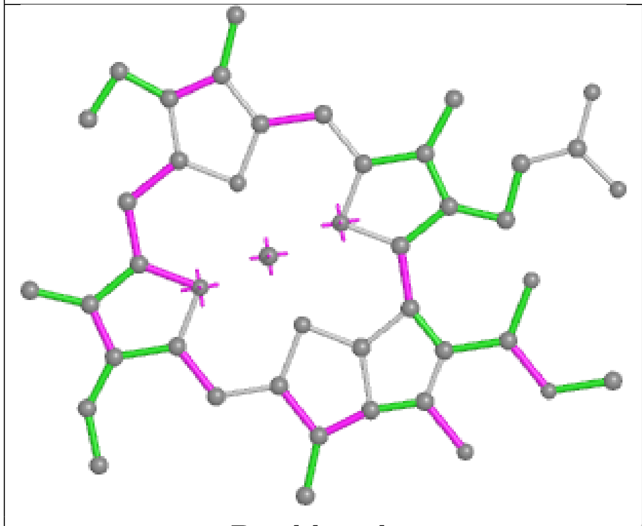

Bond lengths

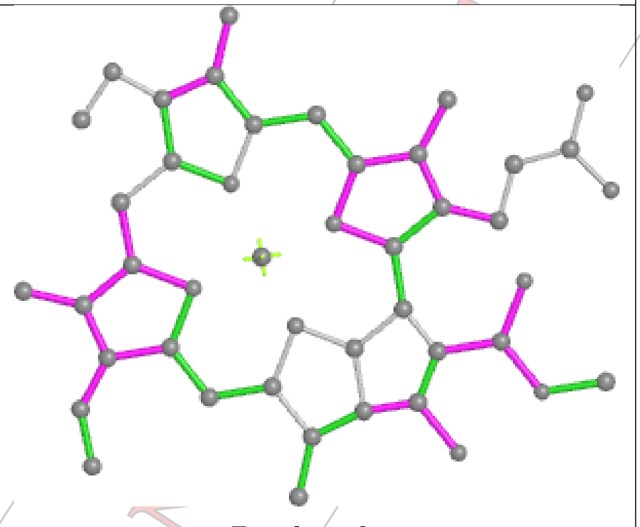

Bond angles

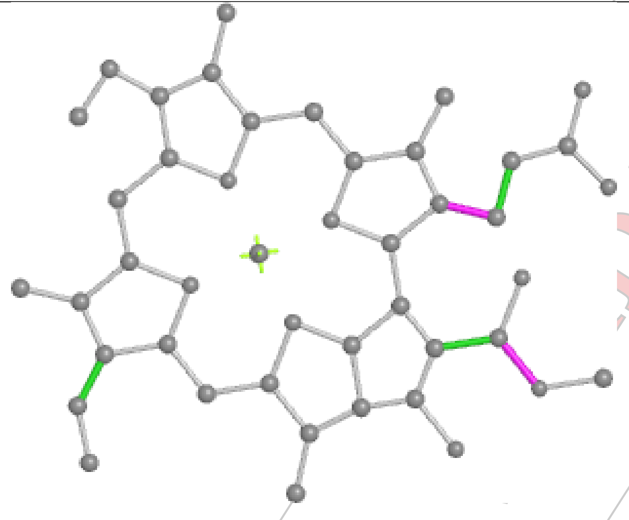

Torsions

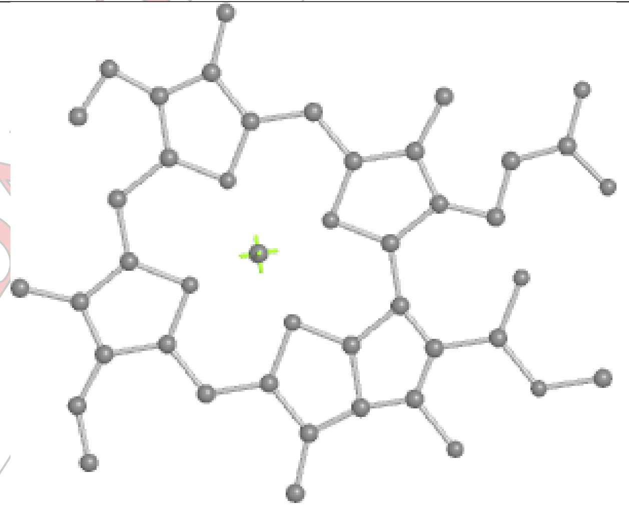

Rings

PRELIMINARY

Ligand CLA GA 1110

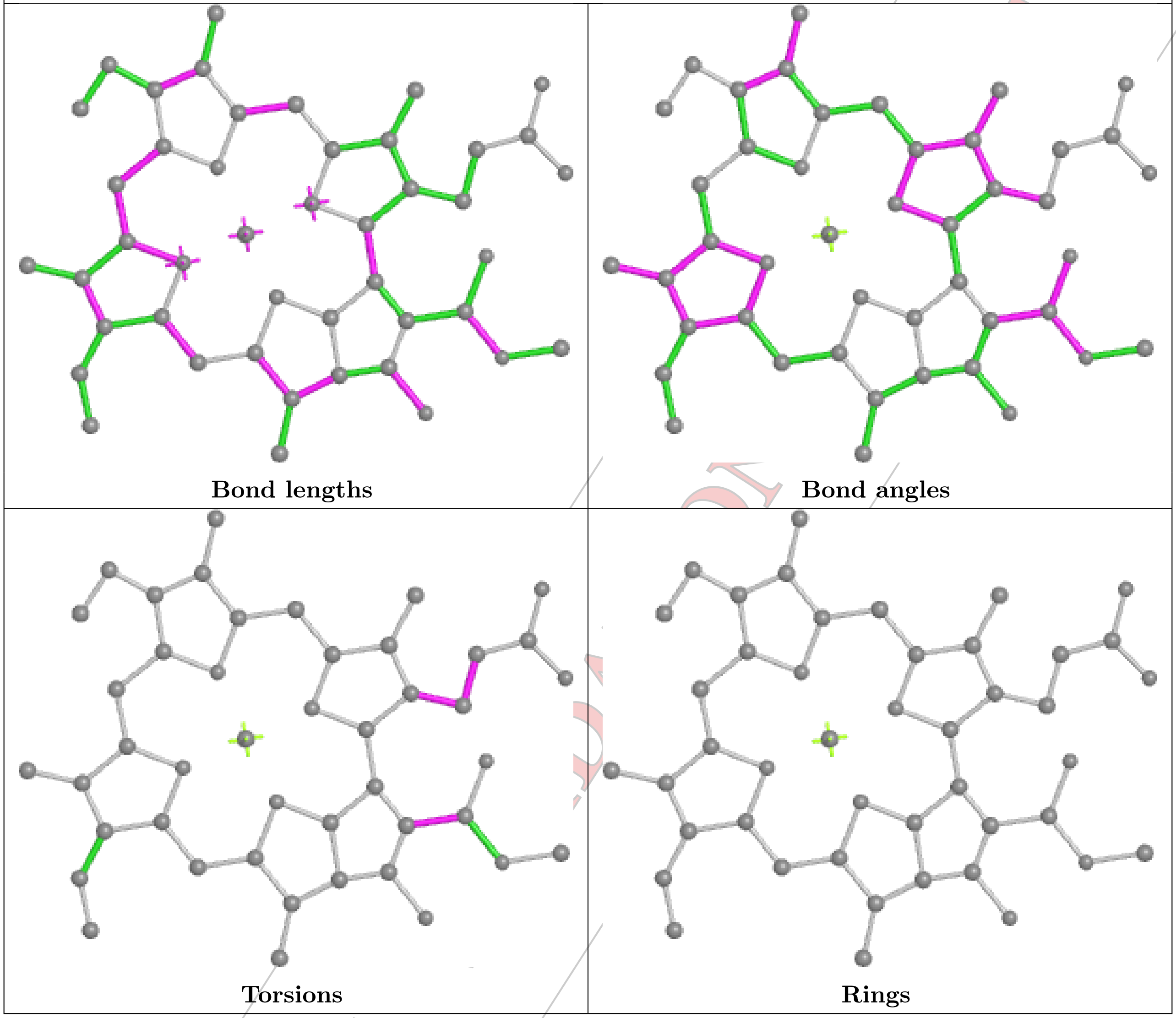

Ligand CLA GA 1111

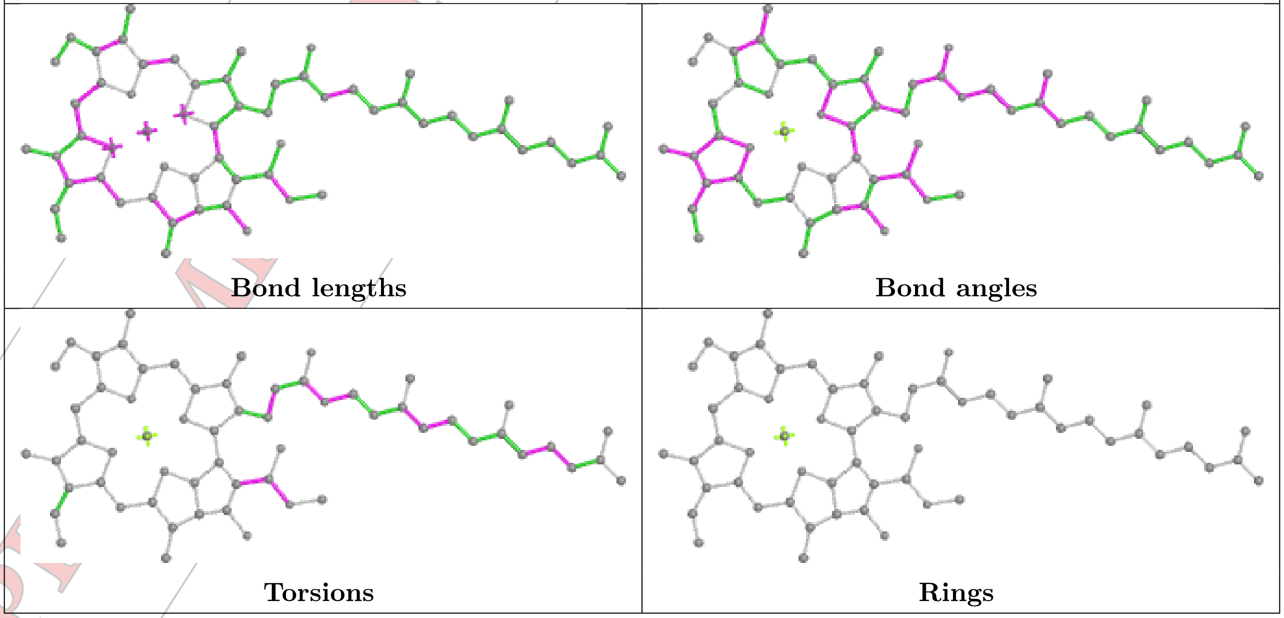

Ligand CLA GA 1112

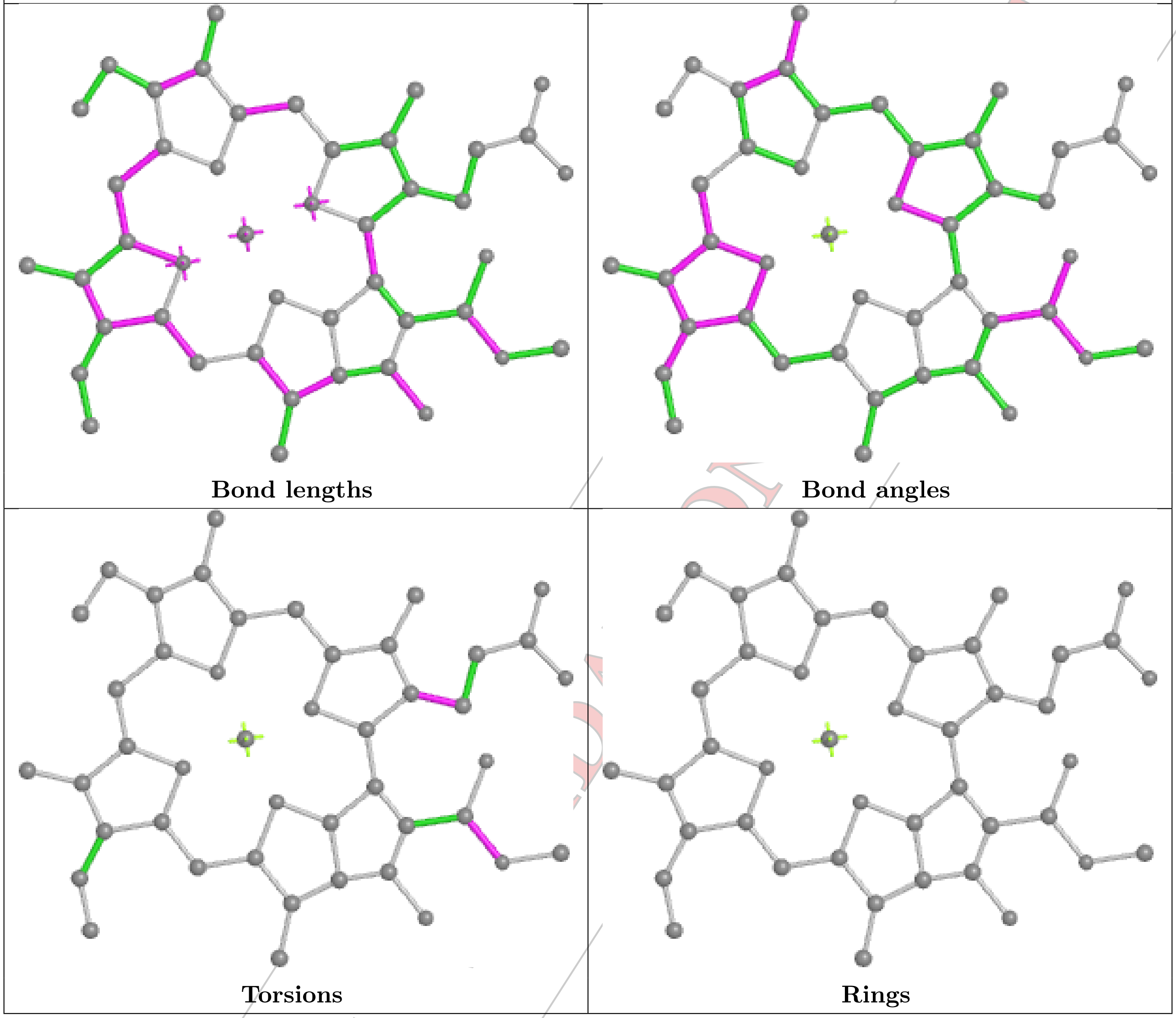

PRELIMINARY

Ligand CLA GA 1113	
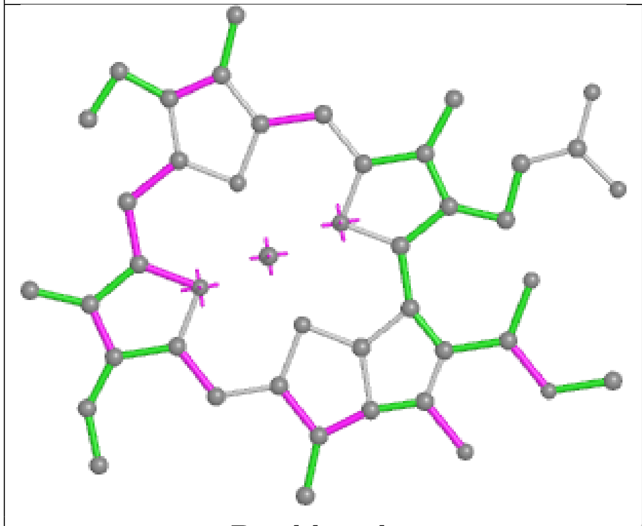 Bond lengths	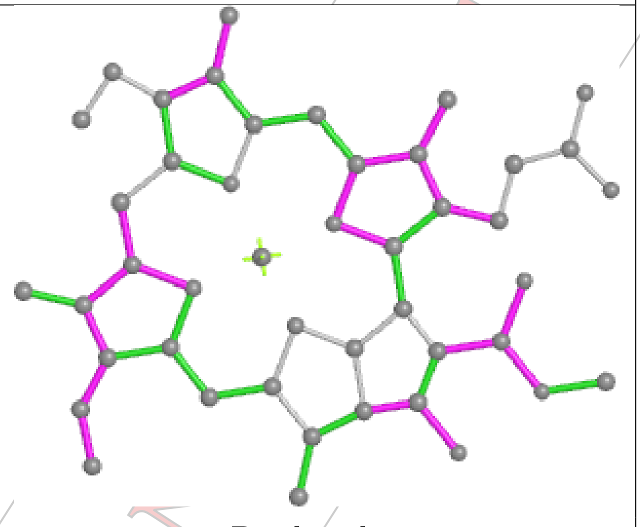 Bond angles
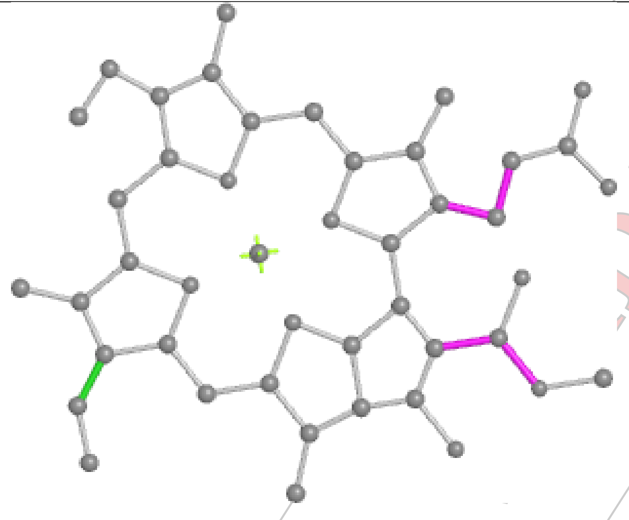 Torsions	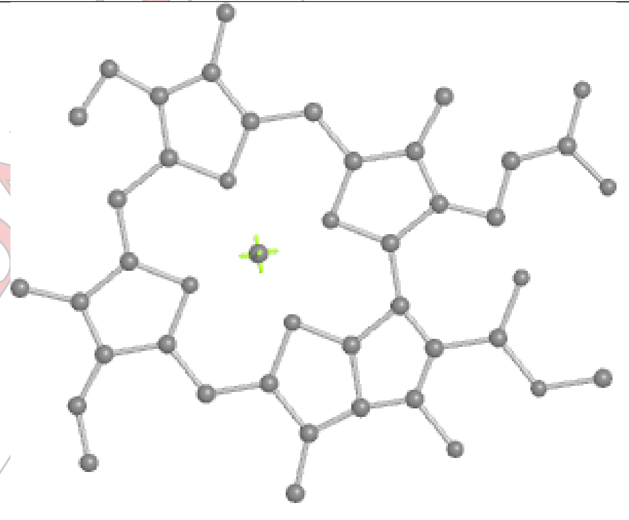 Rings

PRELIMINARY

Ligand CLA GA 1114

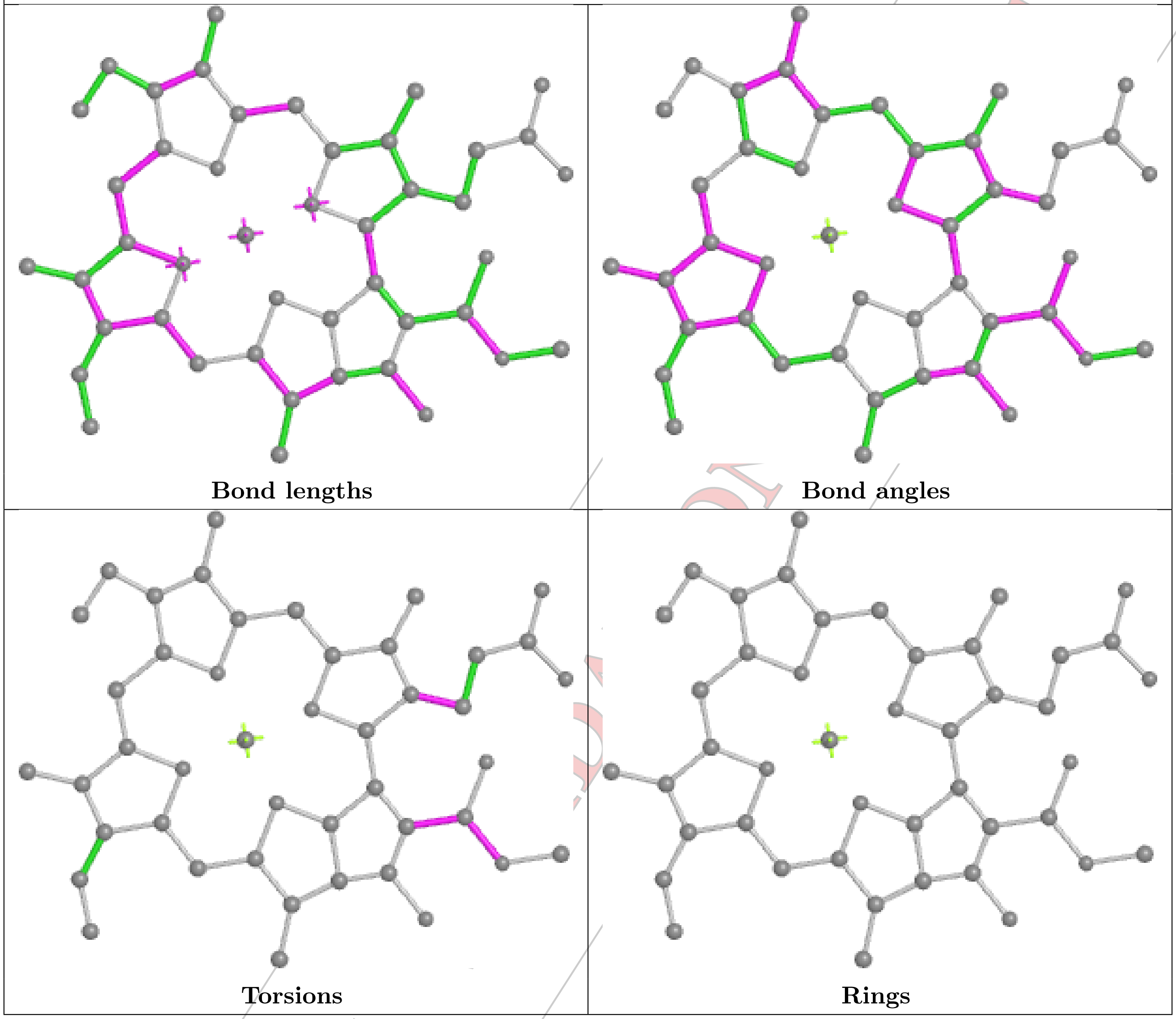

PRELIMINARY

Ligand CLA GA 1115

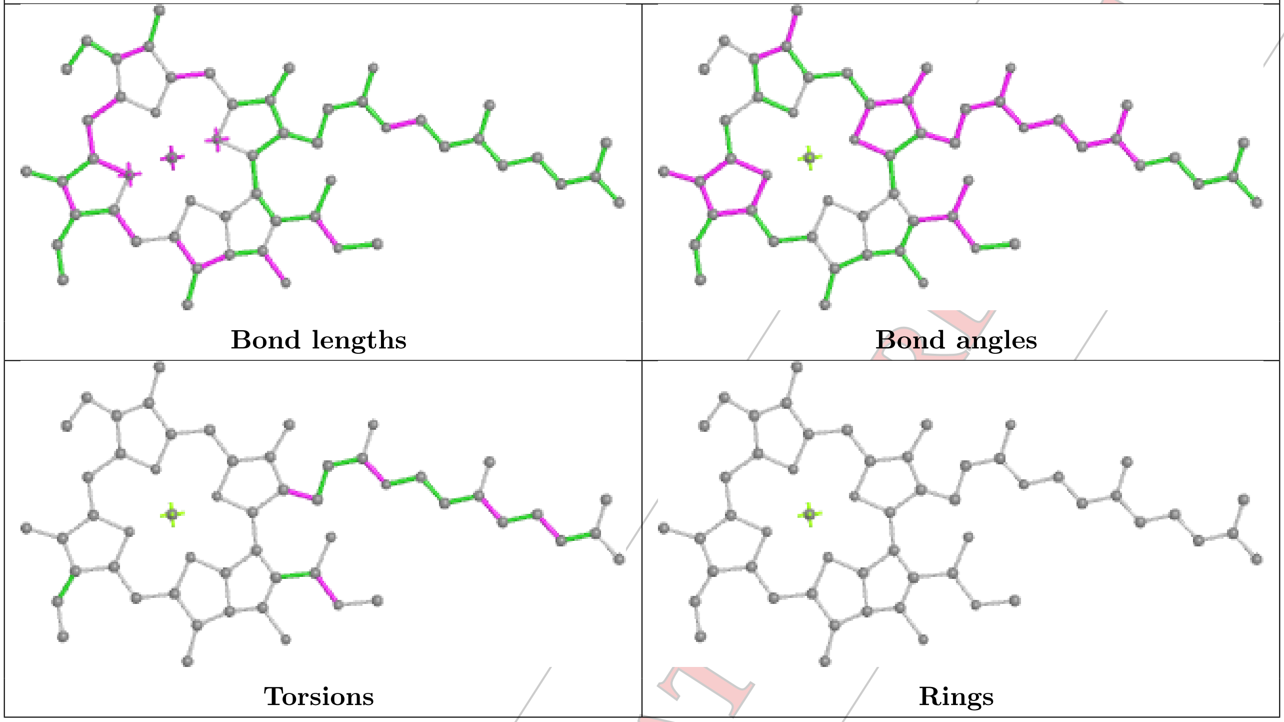

Ligand CLA GA 1116

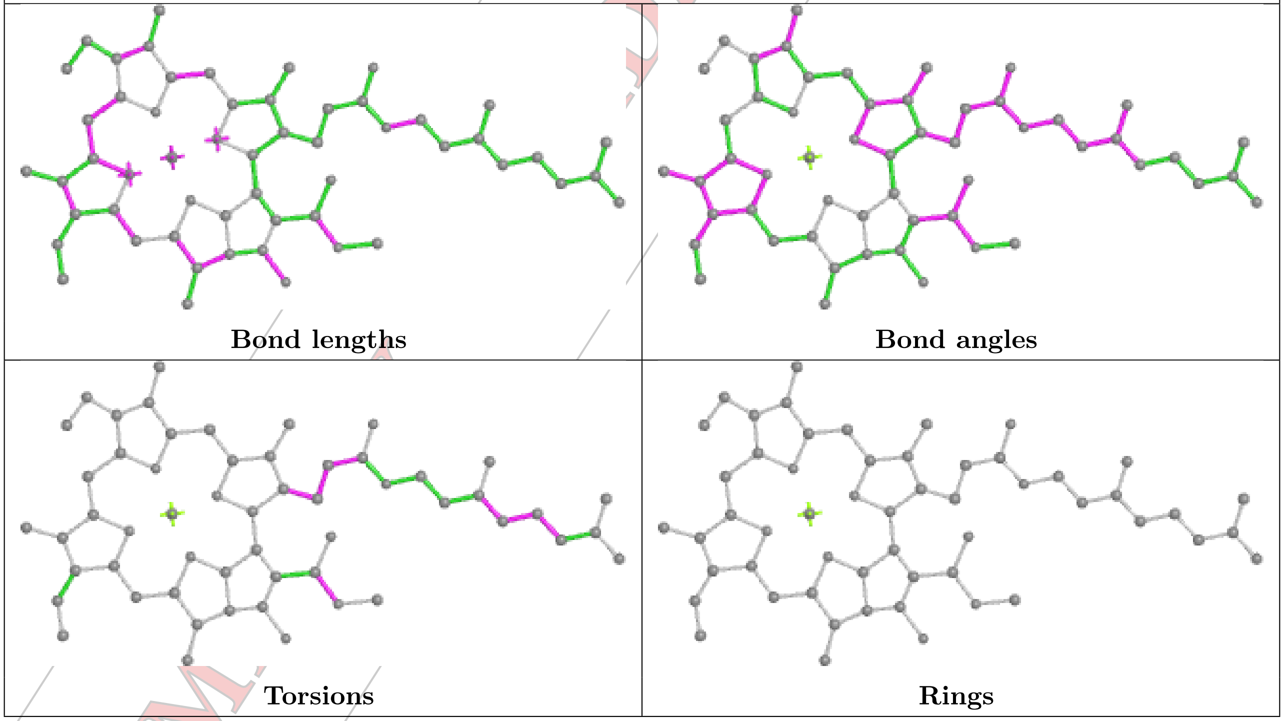

Ligand CLA GA 1117

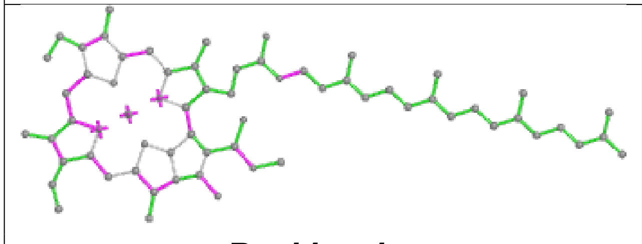

Bond lengths

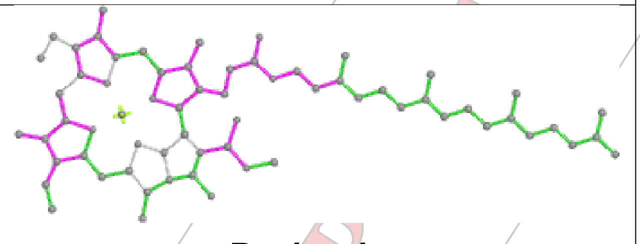

Bond angles

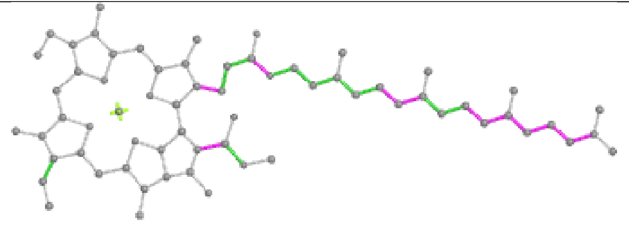

Torsions

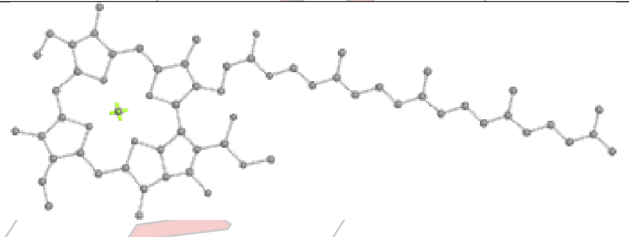

Rings

Ligand CLA GA 1118

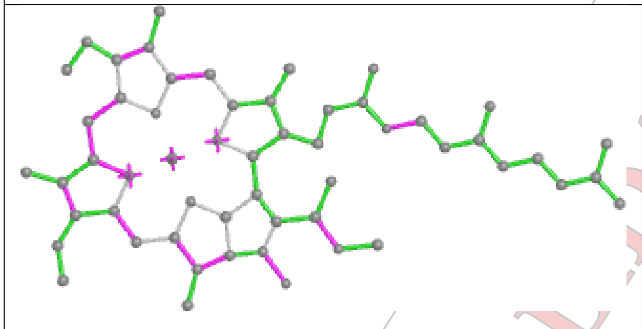

Bond lengths

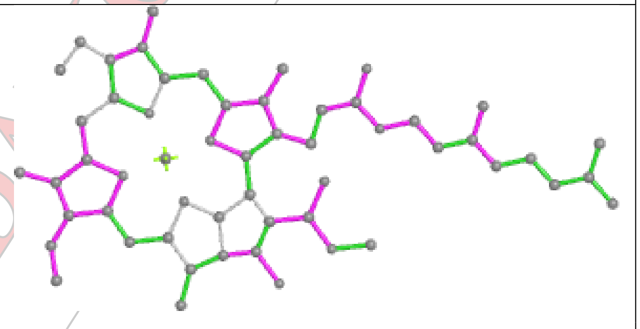

Bond angles

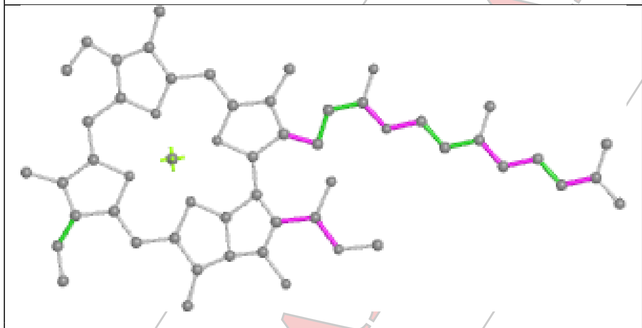

Torsions

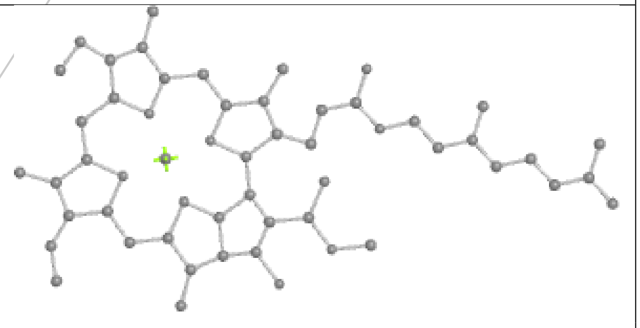

Rings

Ligand CLA GA 1119

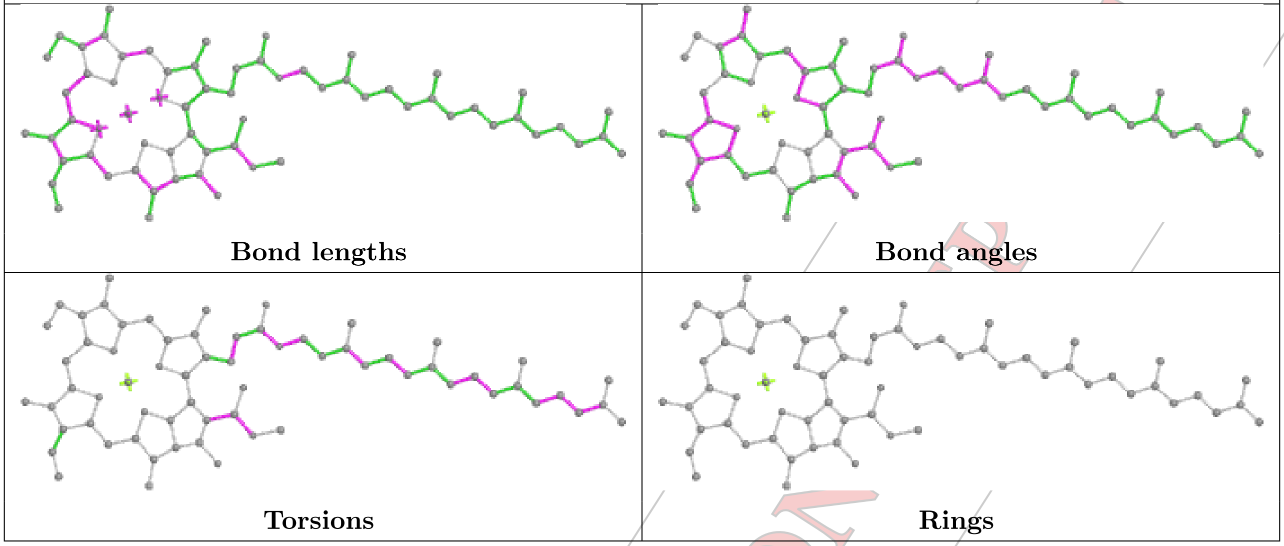

Ligand CLA GA 1122

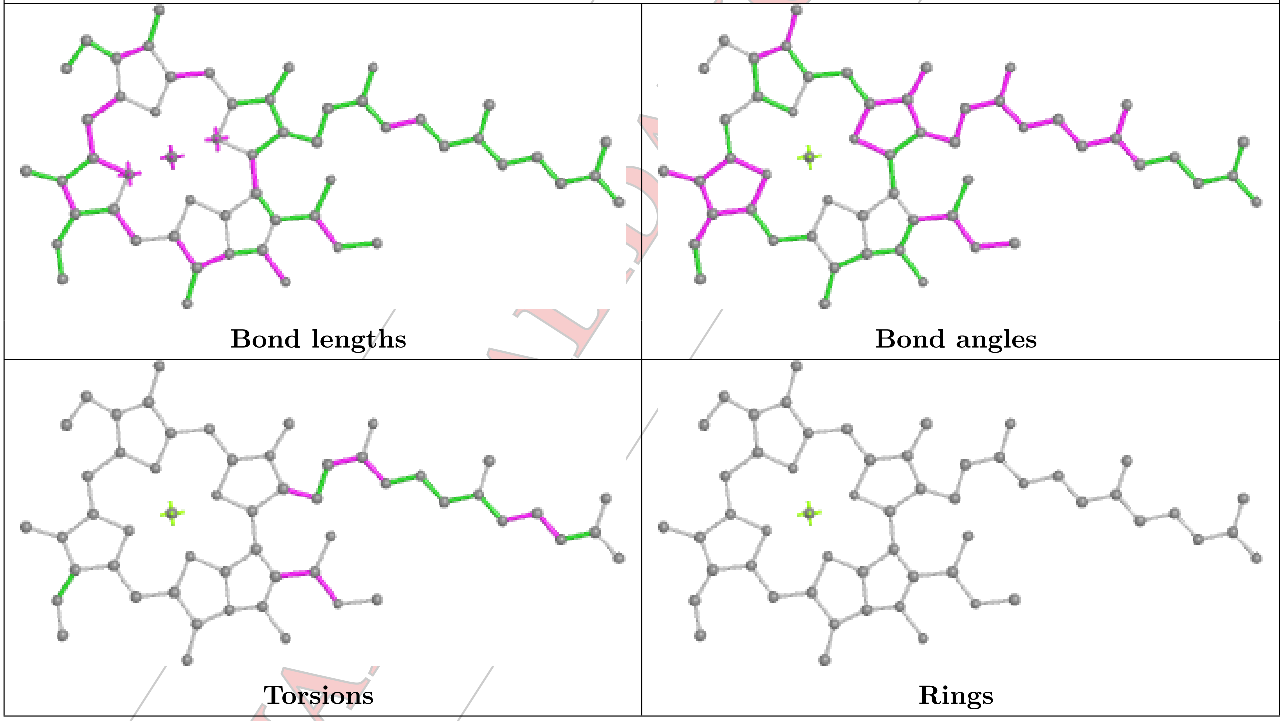

Ligand CLA GA 1123

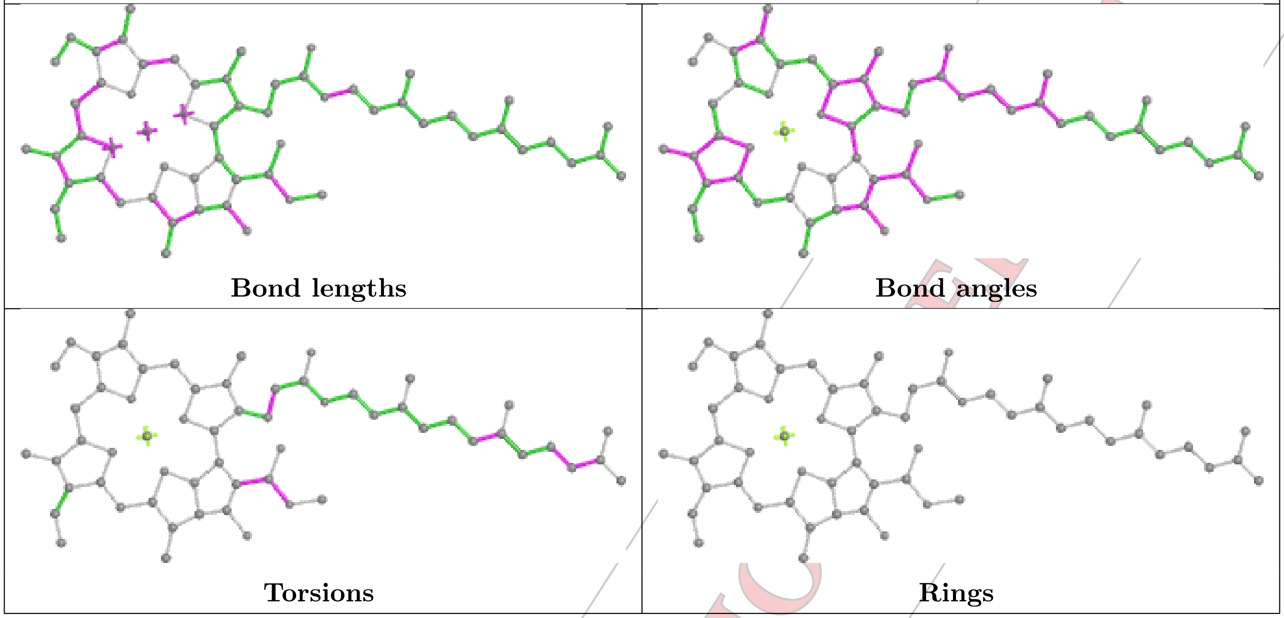

Ligand CLA GA 1124

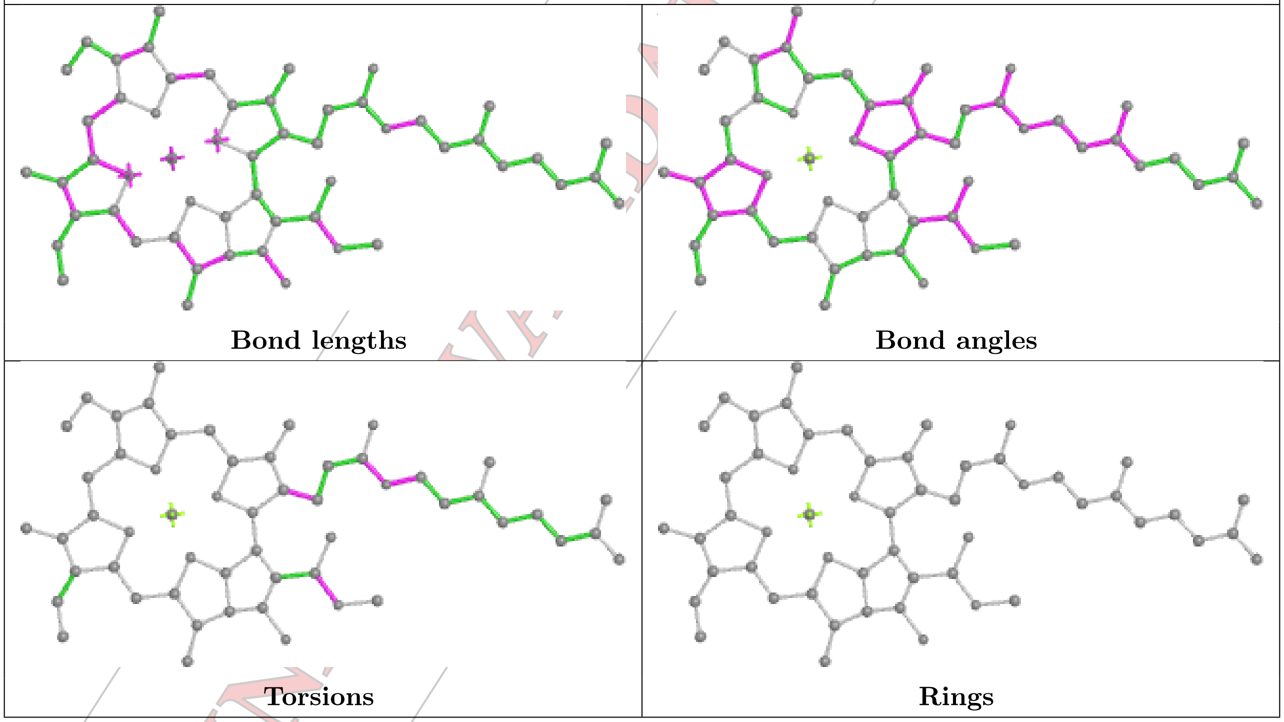

Ligand CLA GA 1125

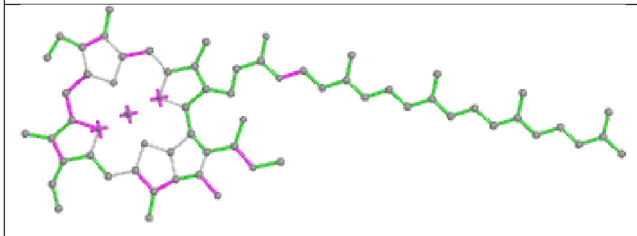
Bond lengths

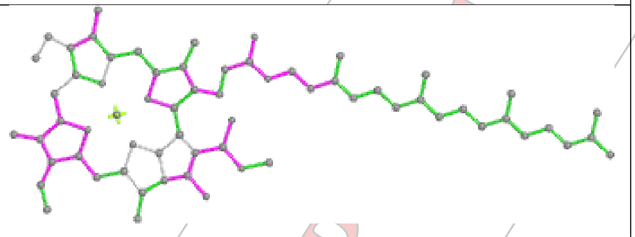
Bond angles

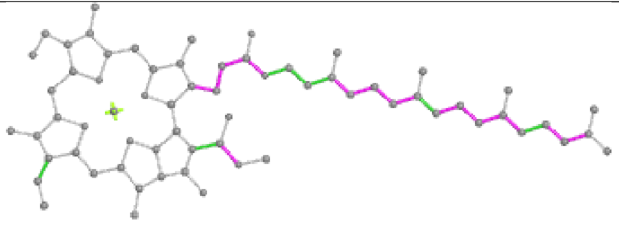
Torsions

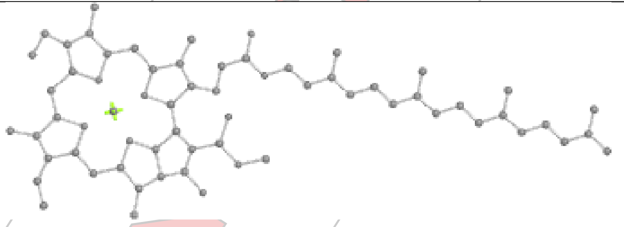
Rings

Ligand CLA GA 1126

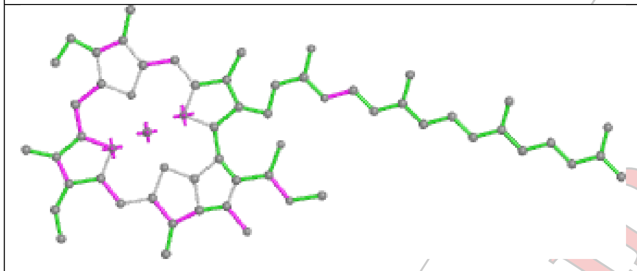
Bond lengths

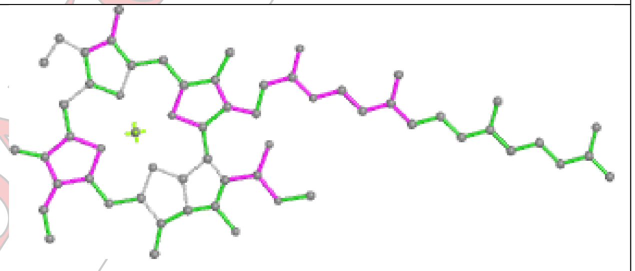
Bond angles

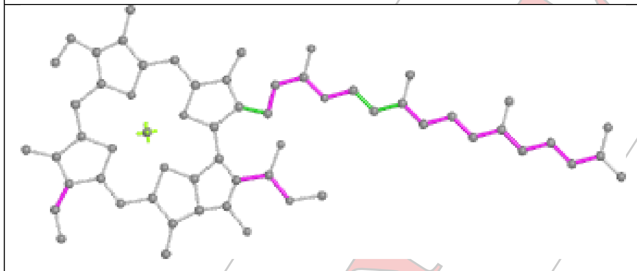
Torsions

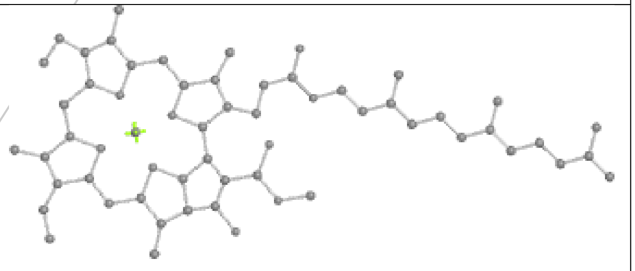
Rings

PRELIMINARY

Ligand CLA GA 1127

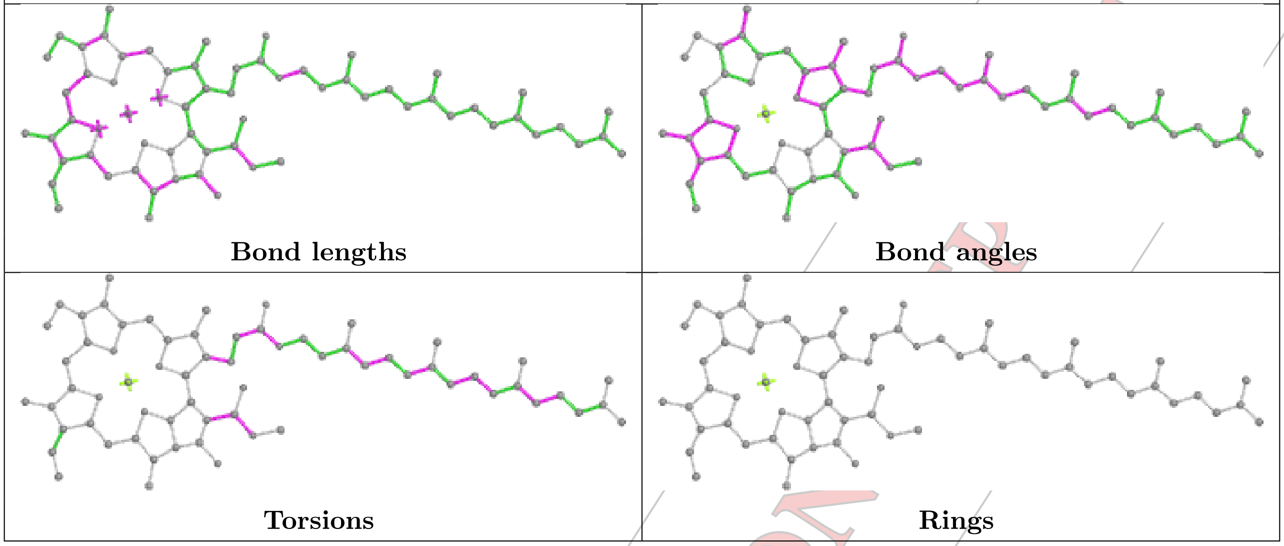

Ligand CLA GA 1128

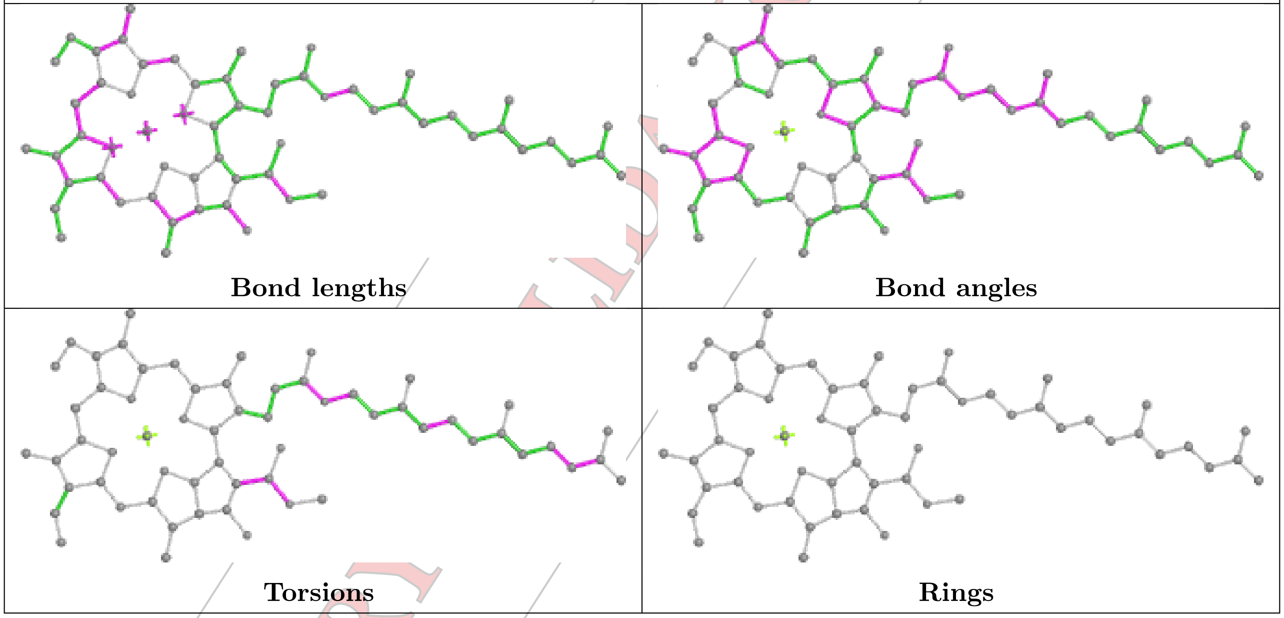

PRELIMINARY

Ligand CLA GA 1129

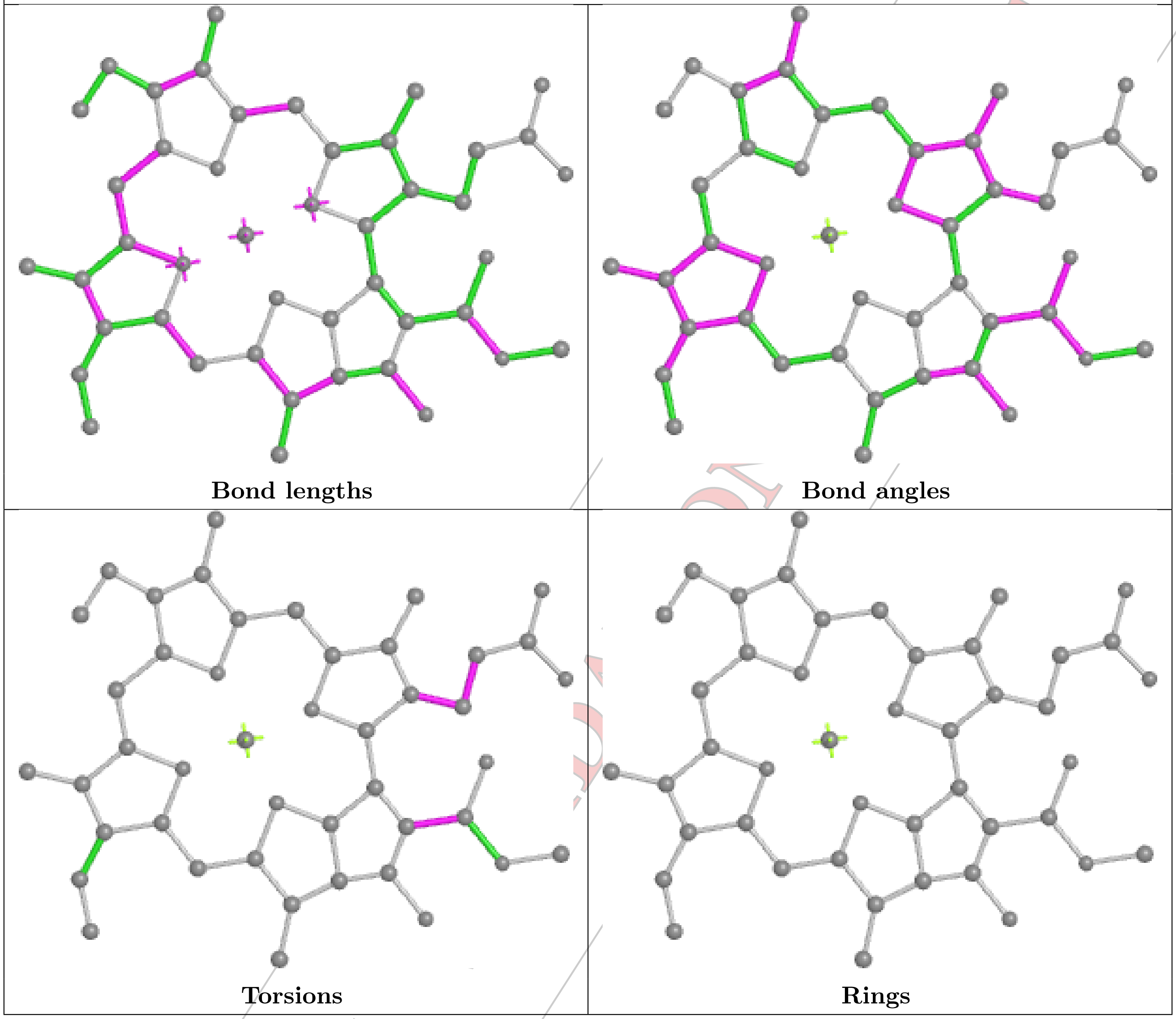

Bond lengths	Bond angles
Torsions	Rings

PRELIMINARY

Ligand CLA GA 1130

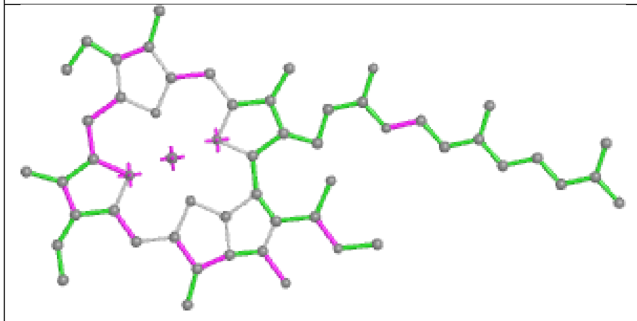

Bond lengths

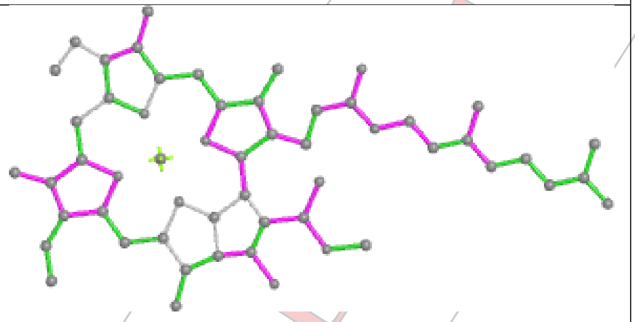

Bond angles

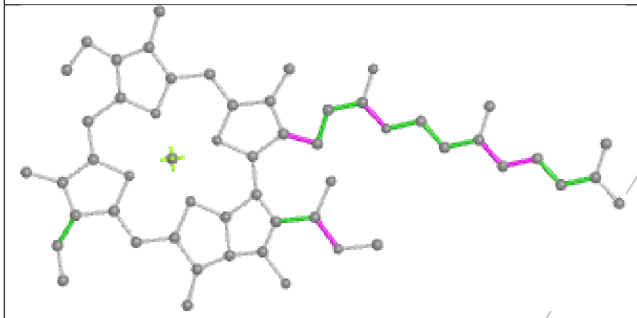

Torsions

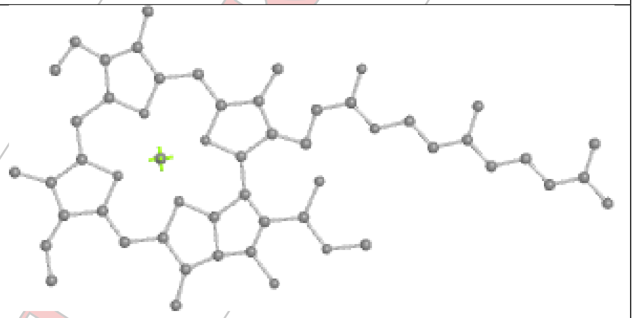

Rings

Ligand CLA GA 1131

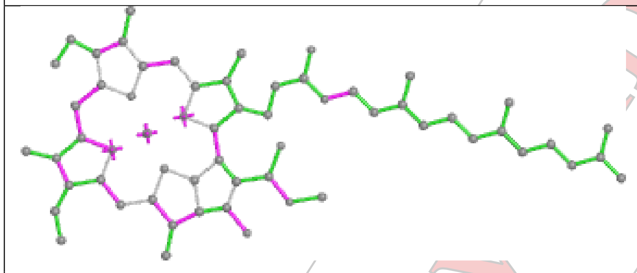

Bond lengths

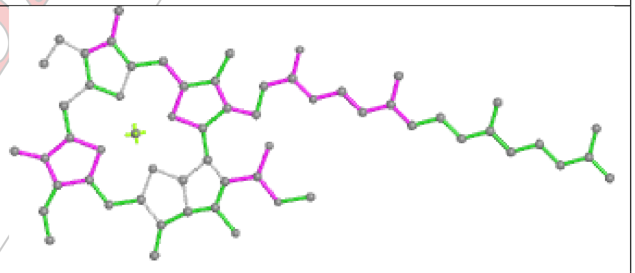

Bond angles

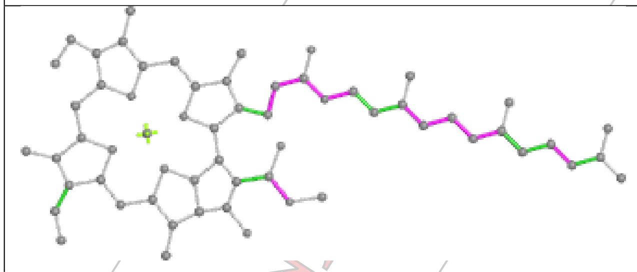

Torsions

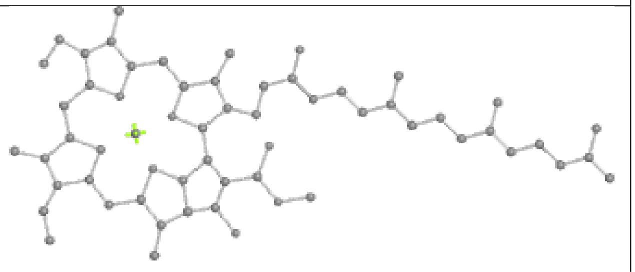

Rings

PRELIMINARY

Ligand CLA GA 1132

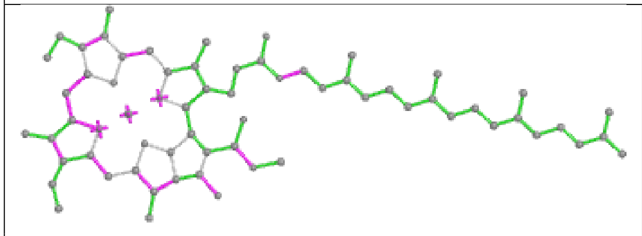

Bond lengths

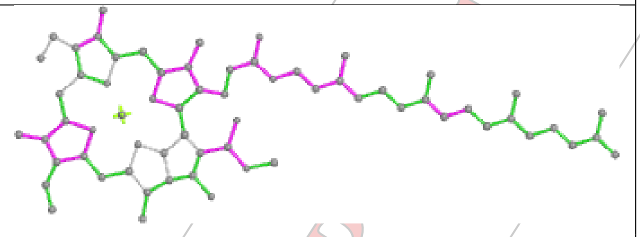

Bond angles

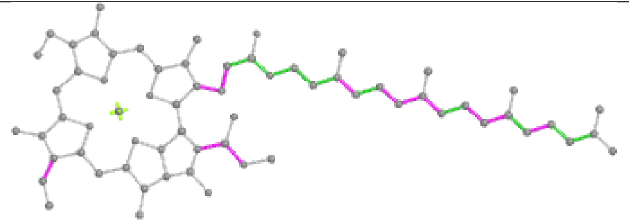

Torsions

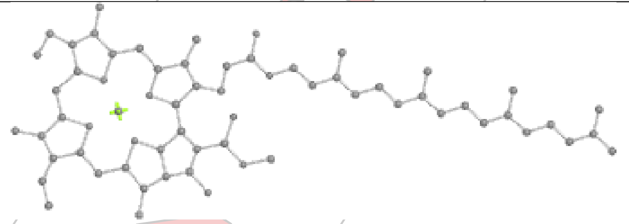

Rings

Ligand CLA GA 1133

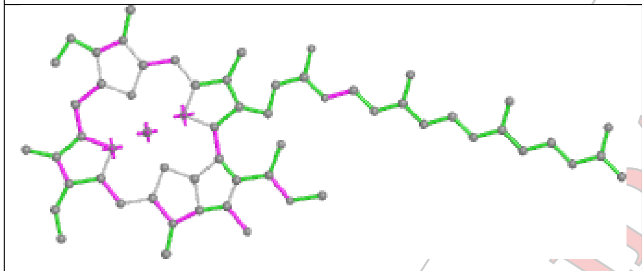

Bond lengths

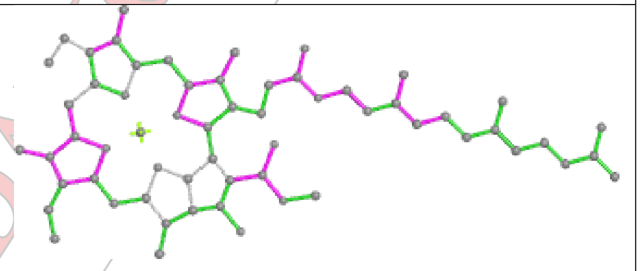

Bond angles

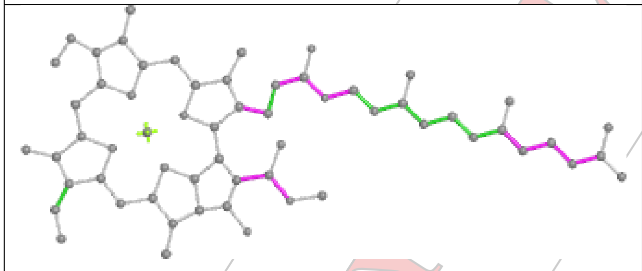

Torsions

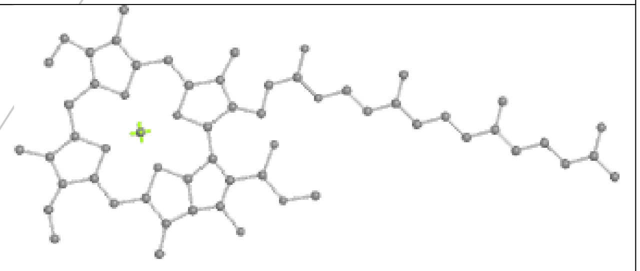

Rings

PRELIMINARY

Ligand CLA GA 1135

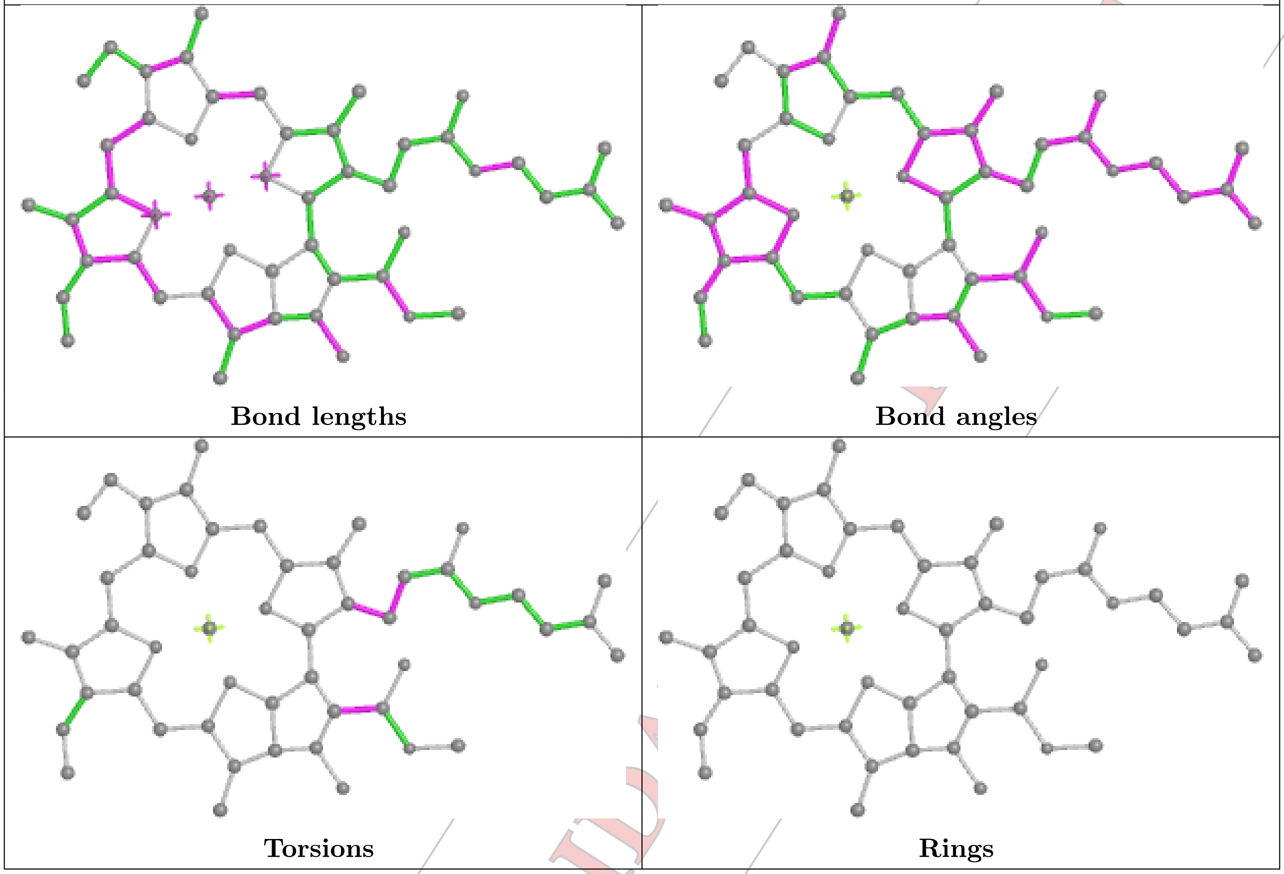

Ligand CLA GA 1136

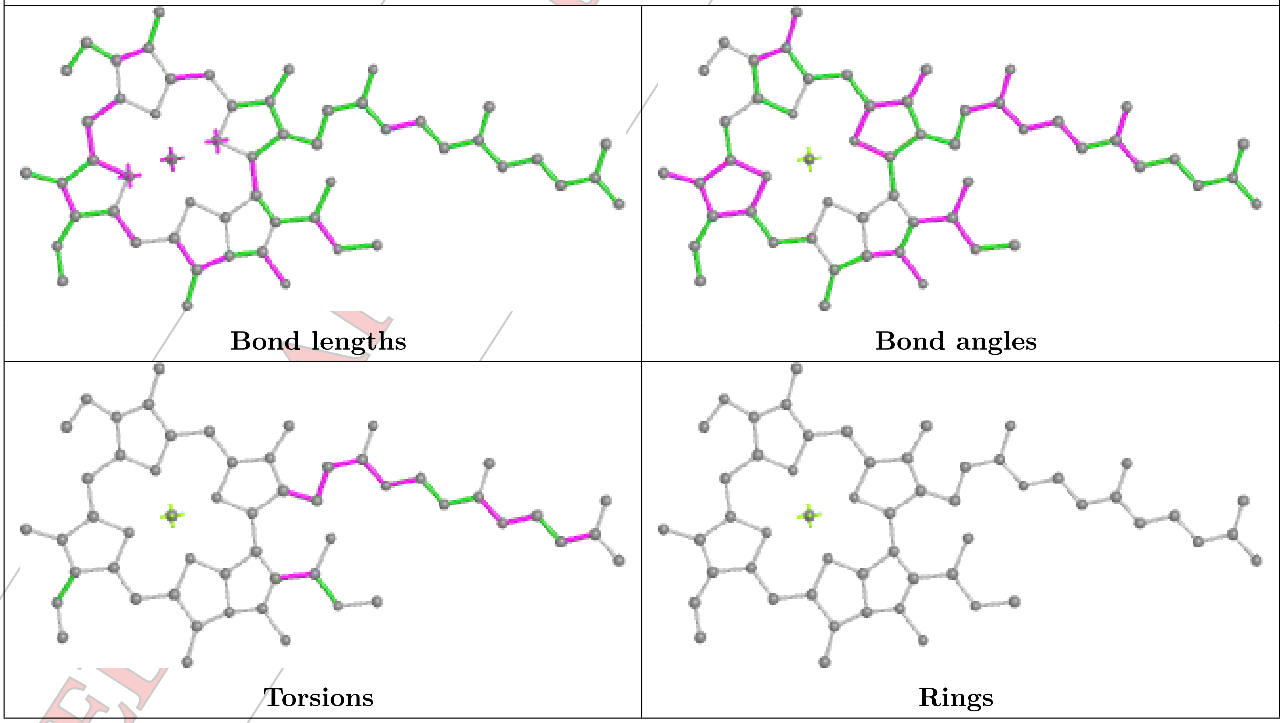

Ligand CLA GA 1137

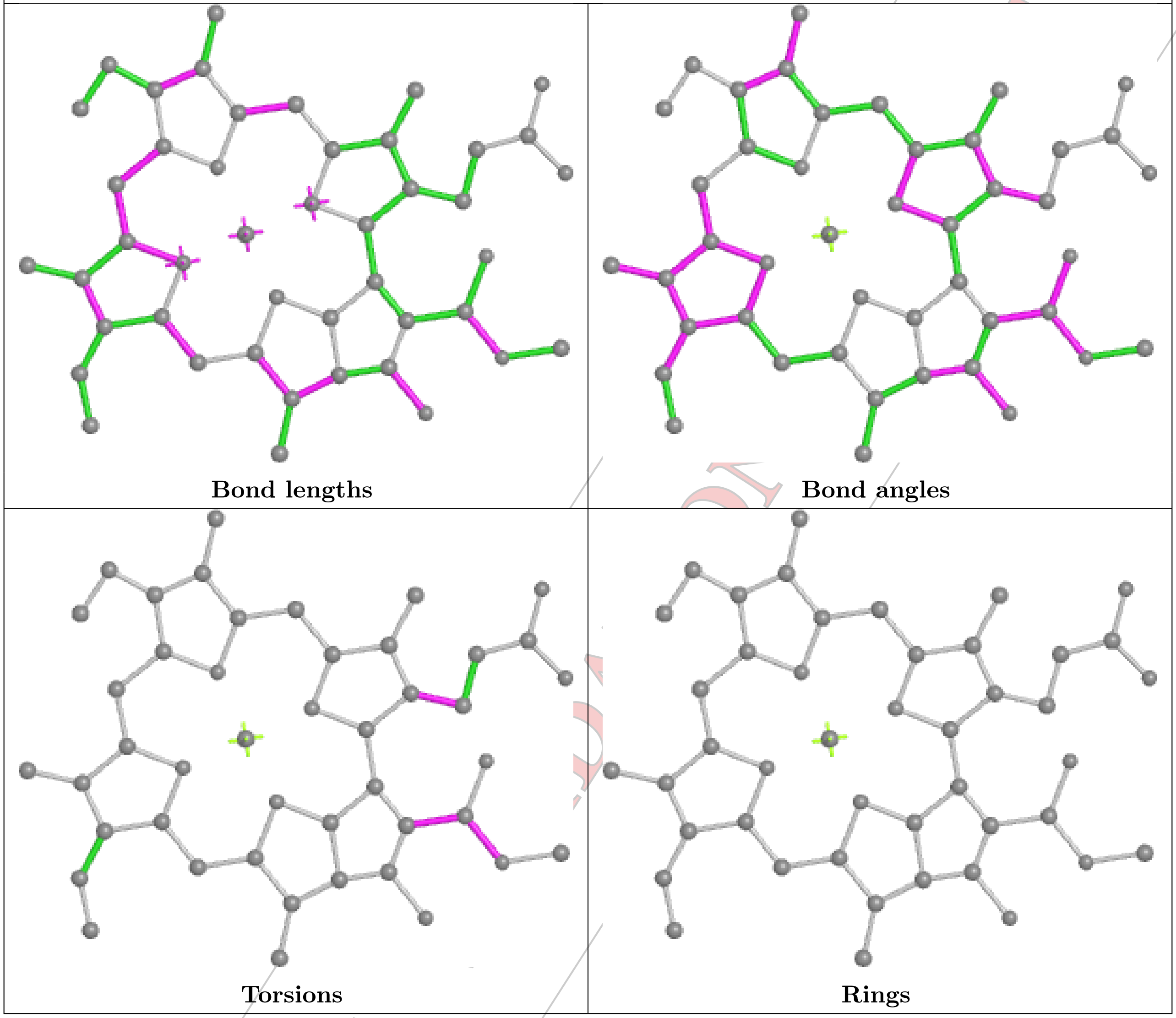

Ligand CLA GA 1138

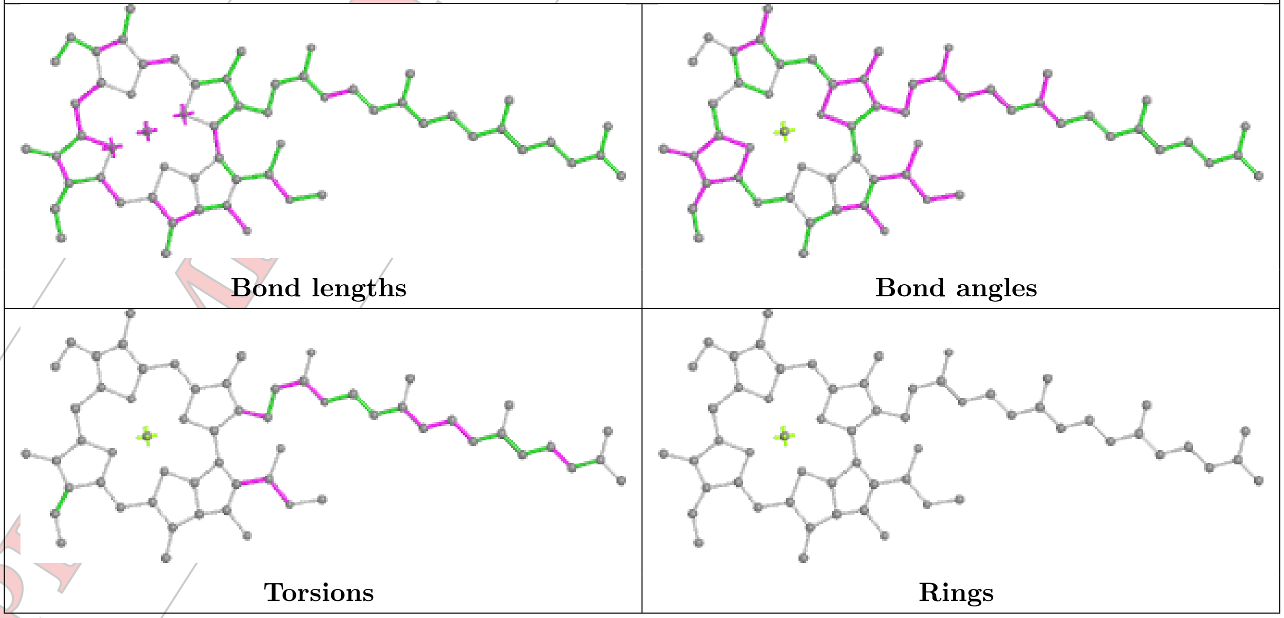

Ligand CLA GA 1139

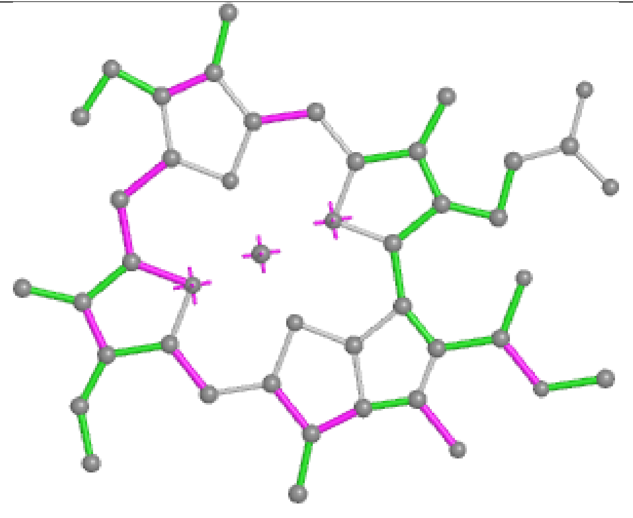

Bond lengths

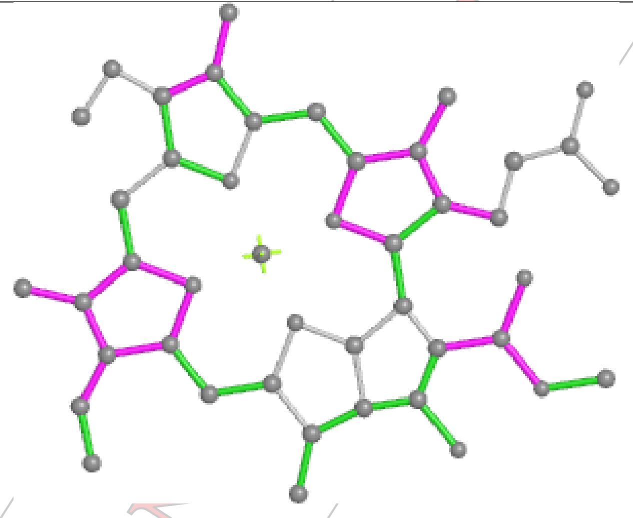

Bond angles

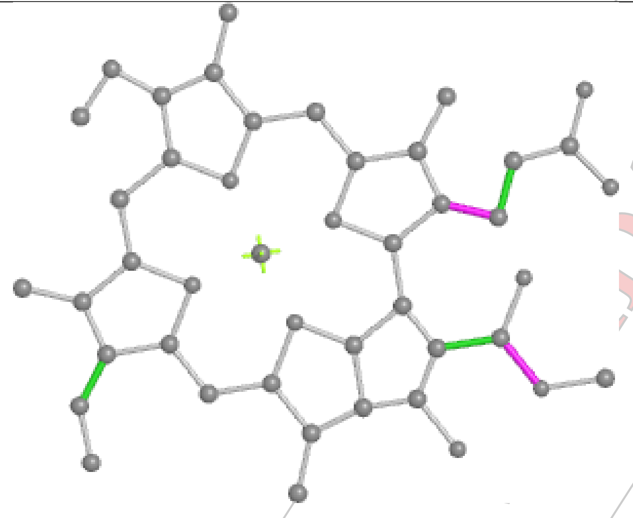

Torsions

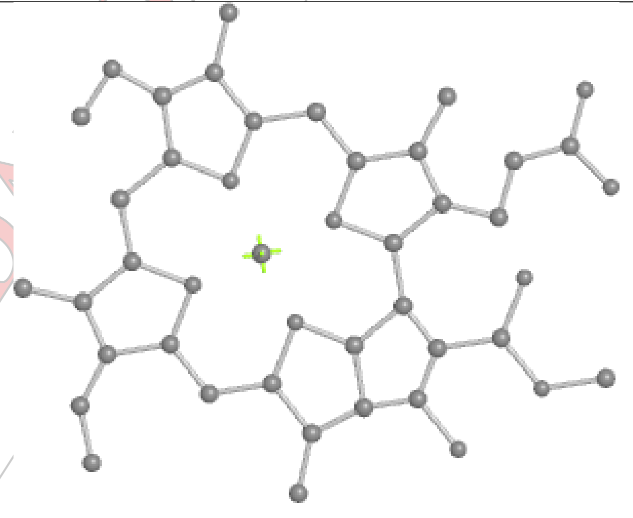

Rings

Ligand CLA GA 1140

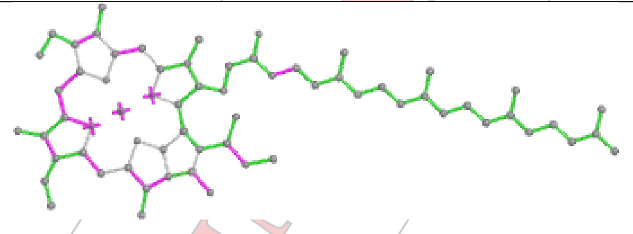

Bond lengths

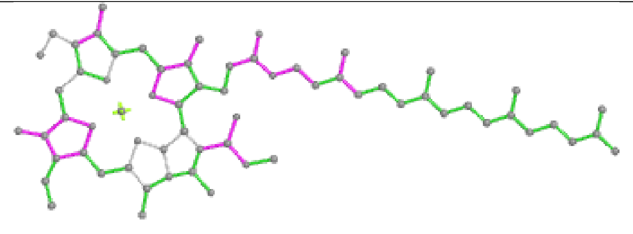

Bond angles

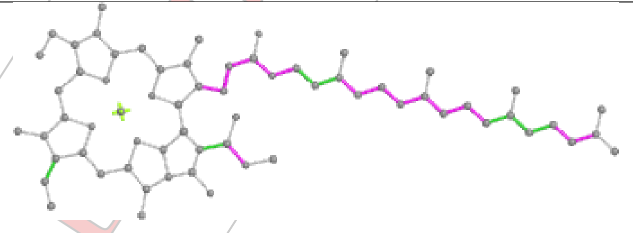

Torsions

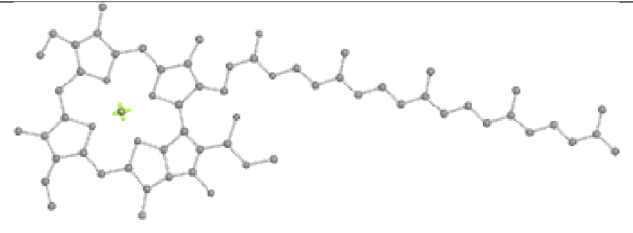

Rings

Ligand PQN GA 2001

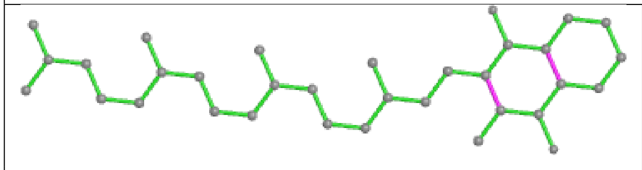

Bond lengths

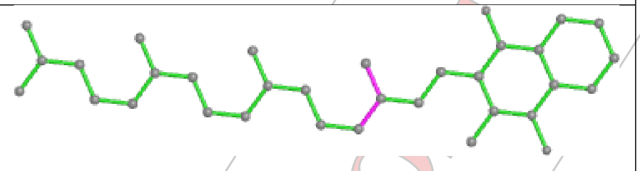

Bond angles

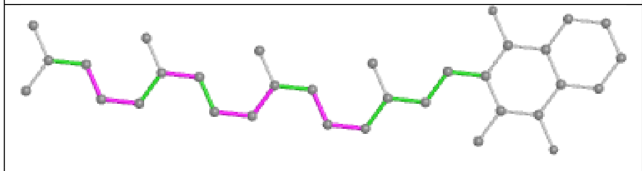

Torsions

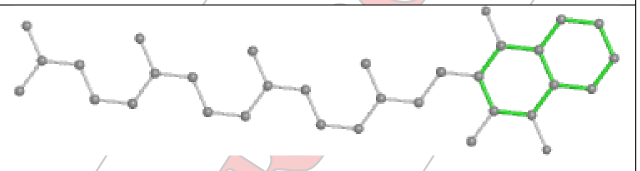

Rings

Ligand BCR GA 4001

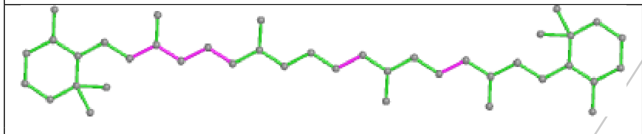

Bond lengths

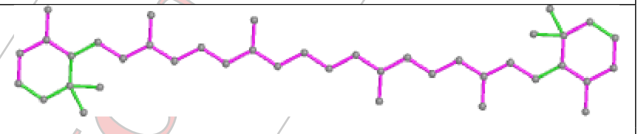

Bond angles

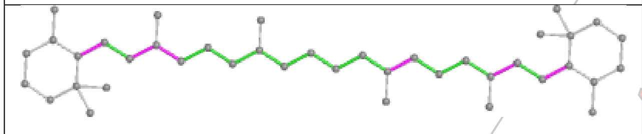

Torsions

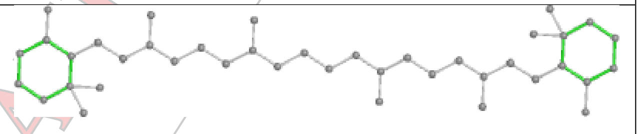

Rings

Ligand BCR GA 4002

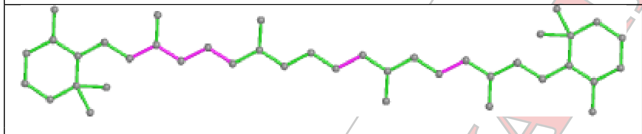

Bond lengths

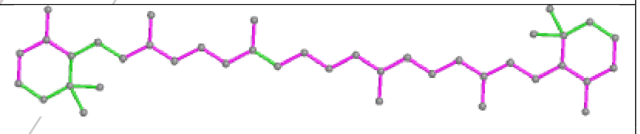

Bond angles

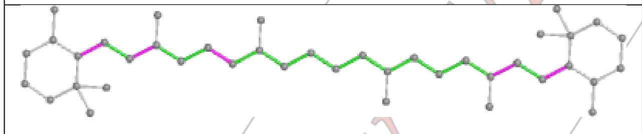

Torsions

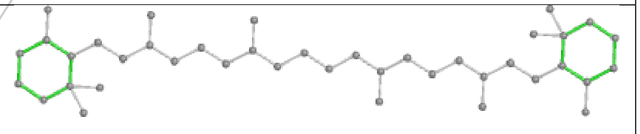

Rings

Ligand BCR GA 4003

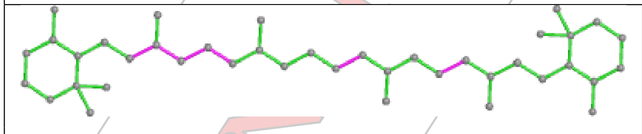

Bond lengths

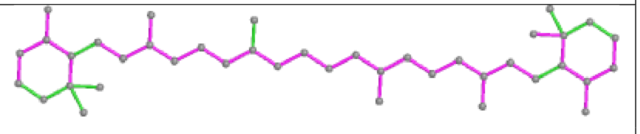

Bond angles

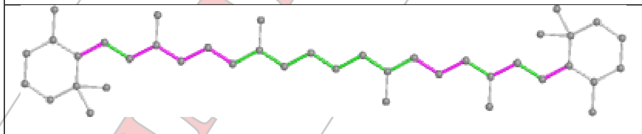

Torsions

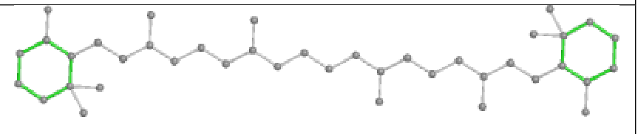

Rings

Ligand BCR GA 4007

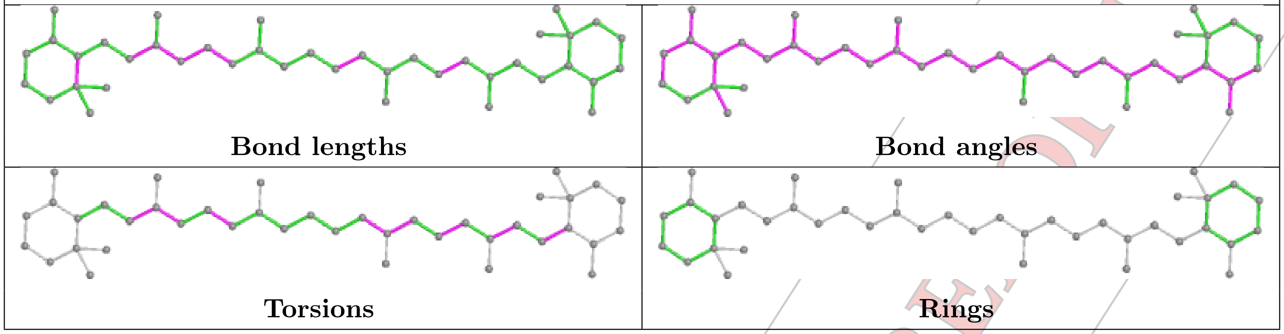

Ligand BCR GA 4008

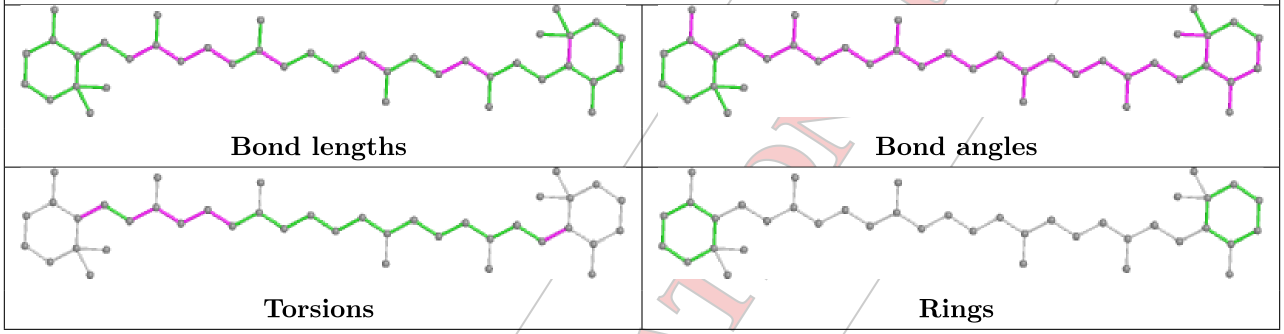

Ligand BCR GA 4011

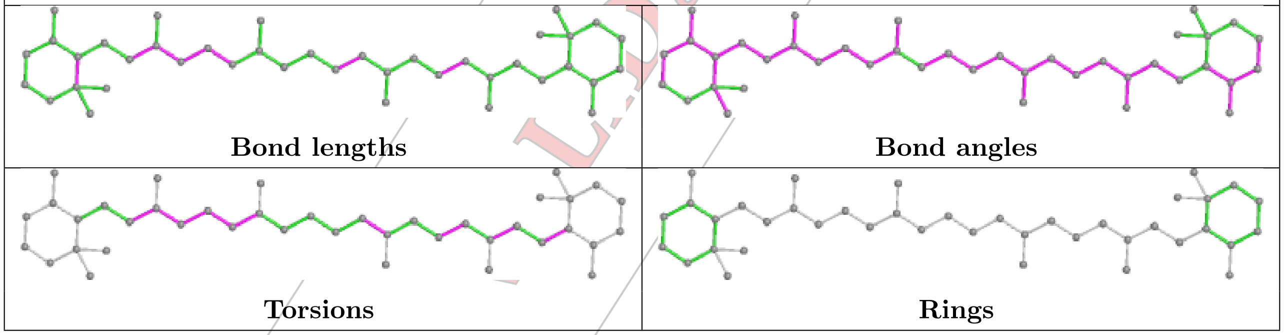

PRELIMINARY

Ligand LHG GA 5001

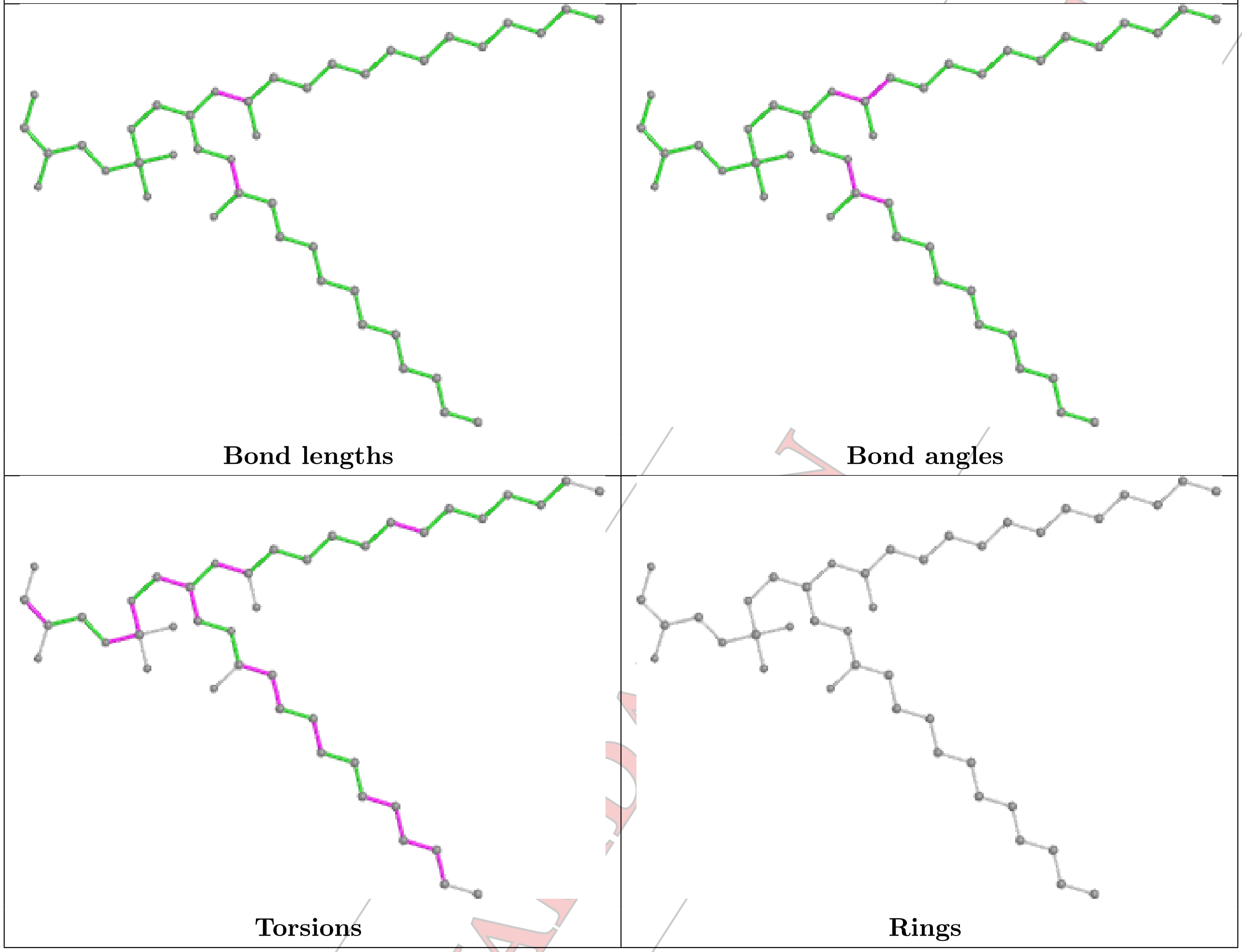

Ligand LMG GA 5005

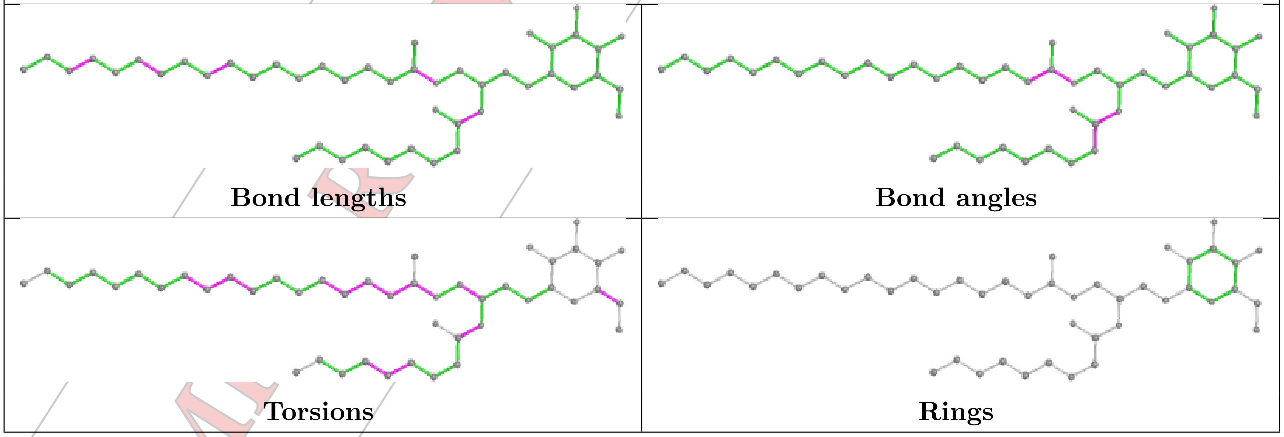

Ligand LMT GA 6001

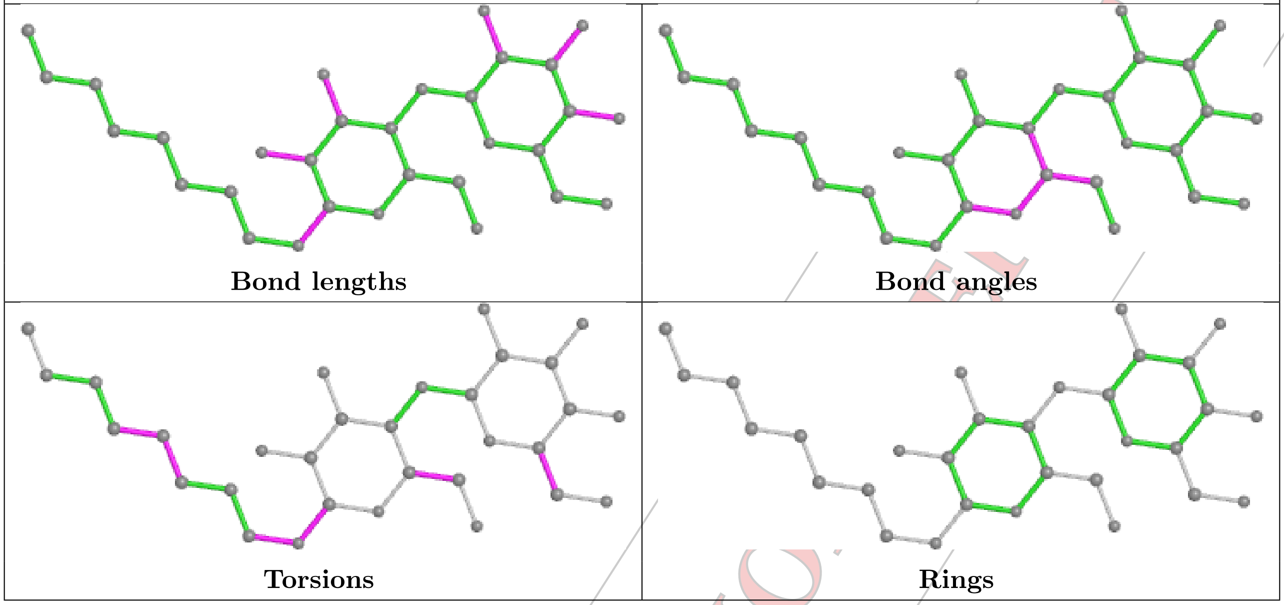

Ligand LMT GA 6002

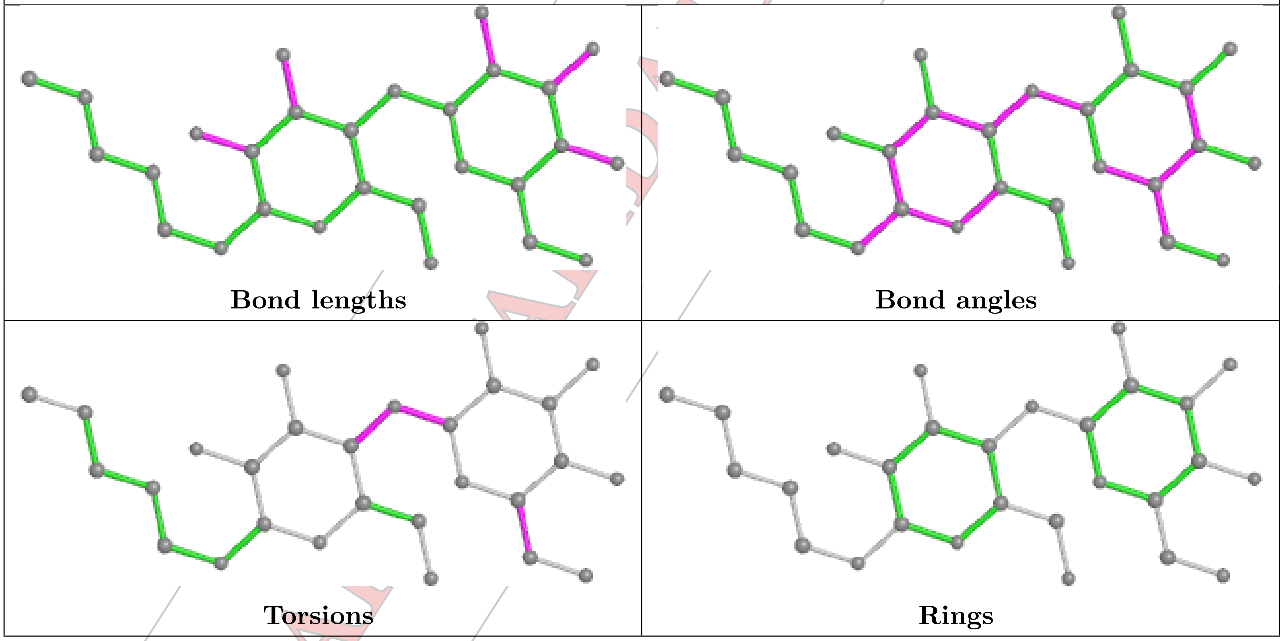

Ligand CLA HA 1021

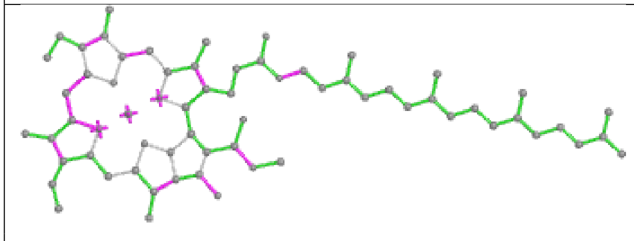

Bond lengths

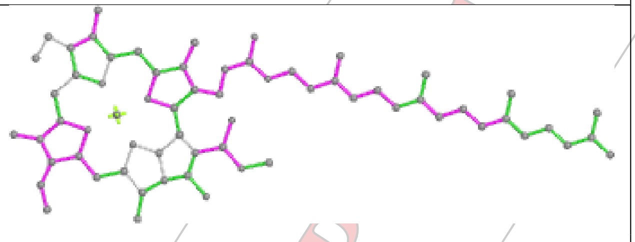

Bond angles

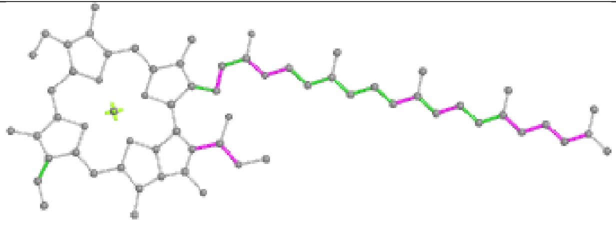

Torsions

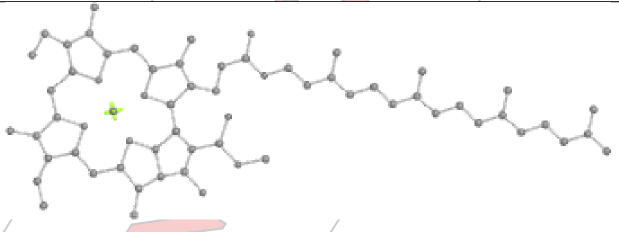

Rings

Ligand CLA HA 1023

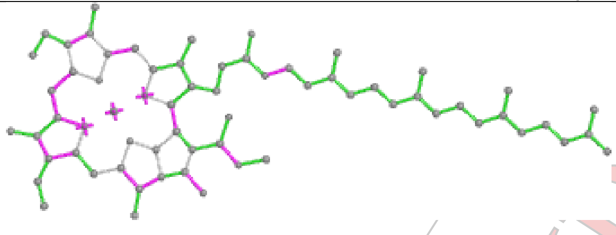

Bond lengths

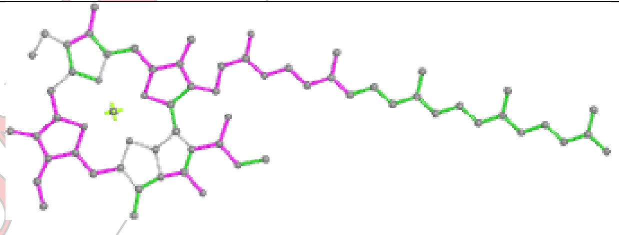

Bond angles

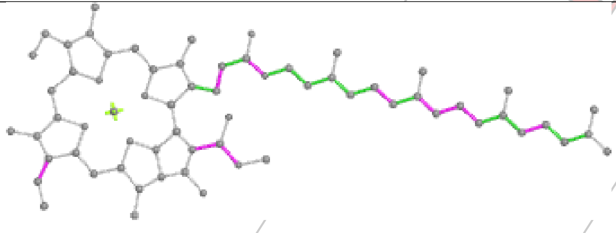

Torsions

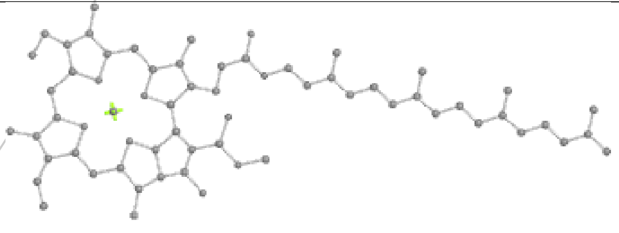

Rings

PRELIMINARY

Ligand CLA HA 1201

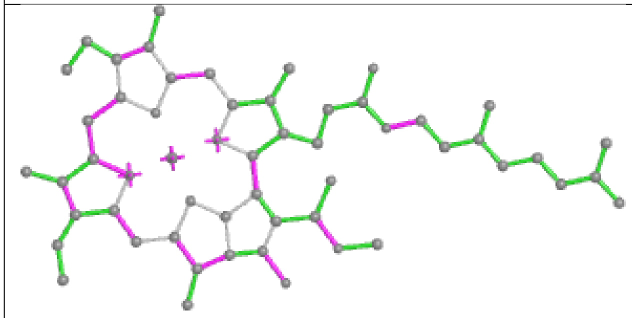

Bond lengths

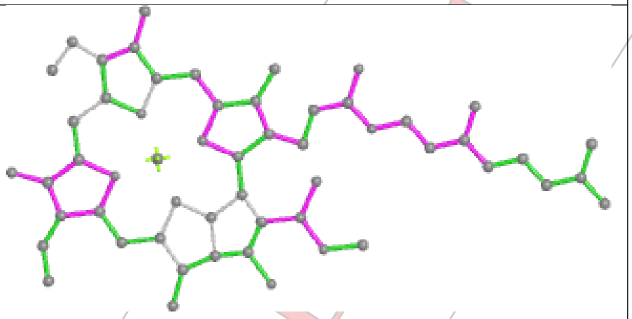

Bond angles

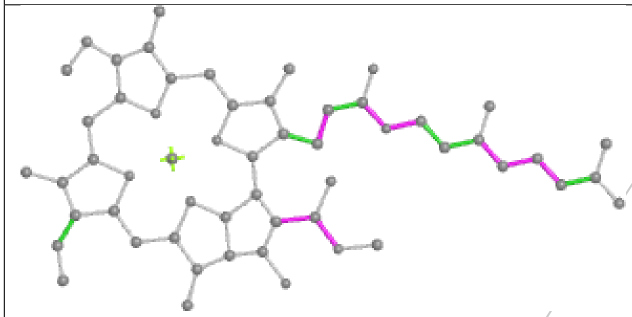

Torsions

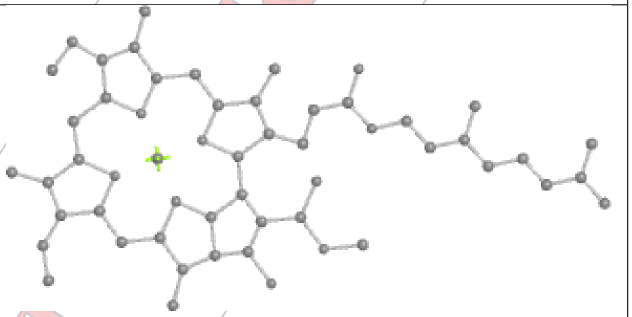

Rings

Ligand CLA HA 1202

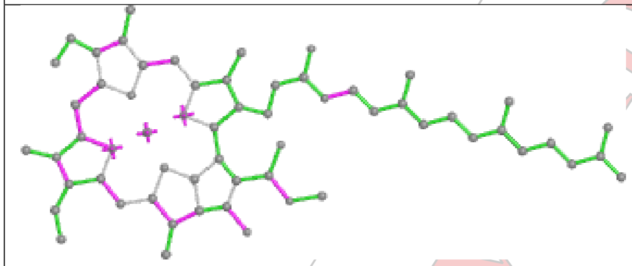

Bond lengths

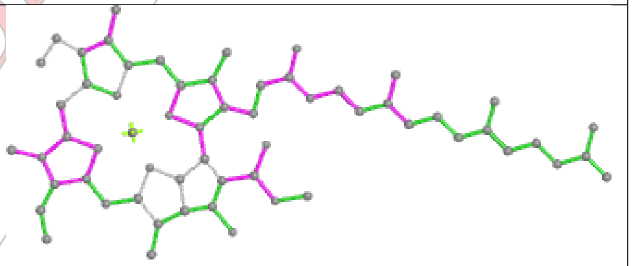

Bond angles

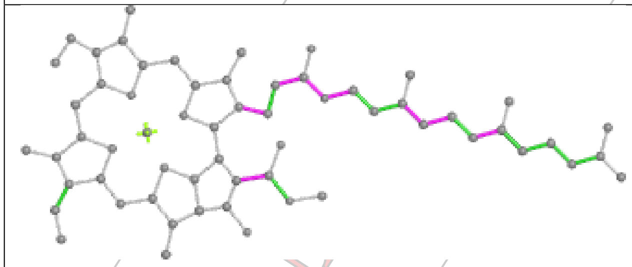

Torsions

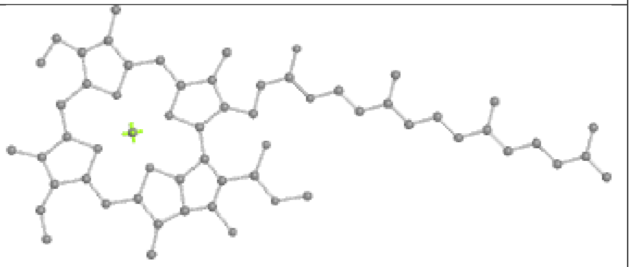

Rings

PRELIMINARY

Ligand CLA HA 1203

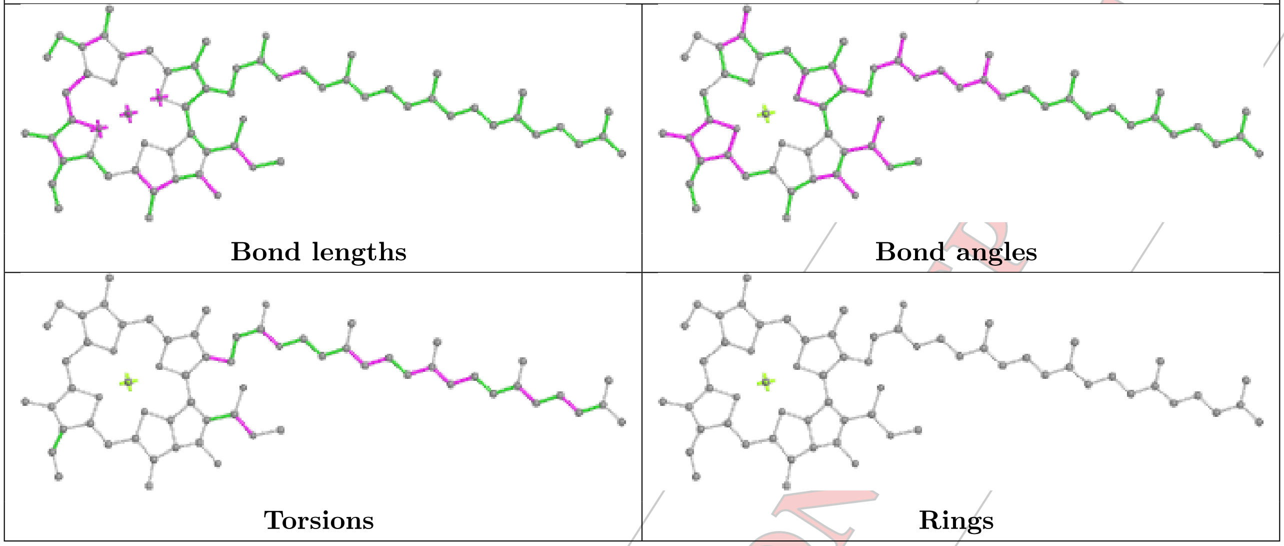

Ligand CLA HA 1204

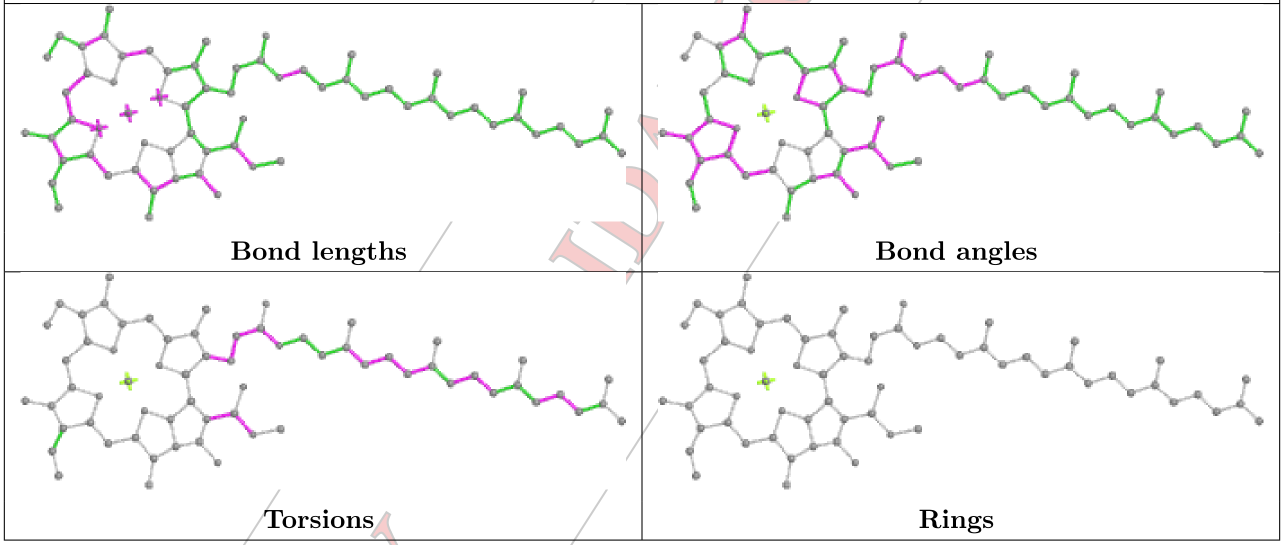

Ligand CLA HA 1205

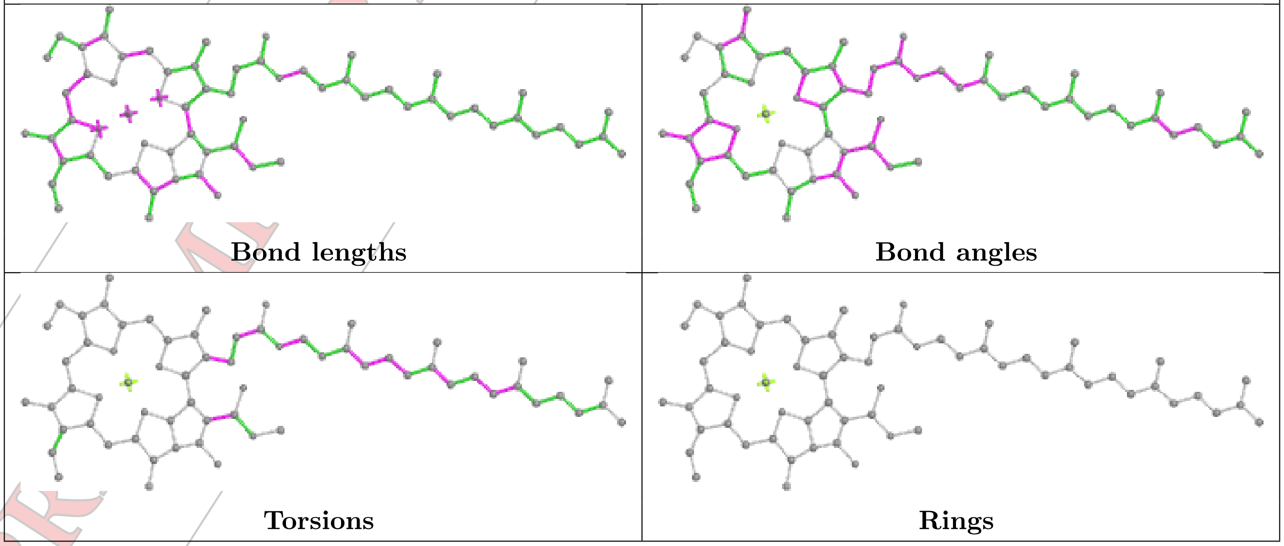

Ligand CLA HA 1206

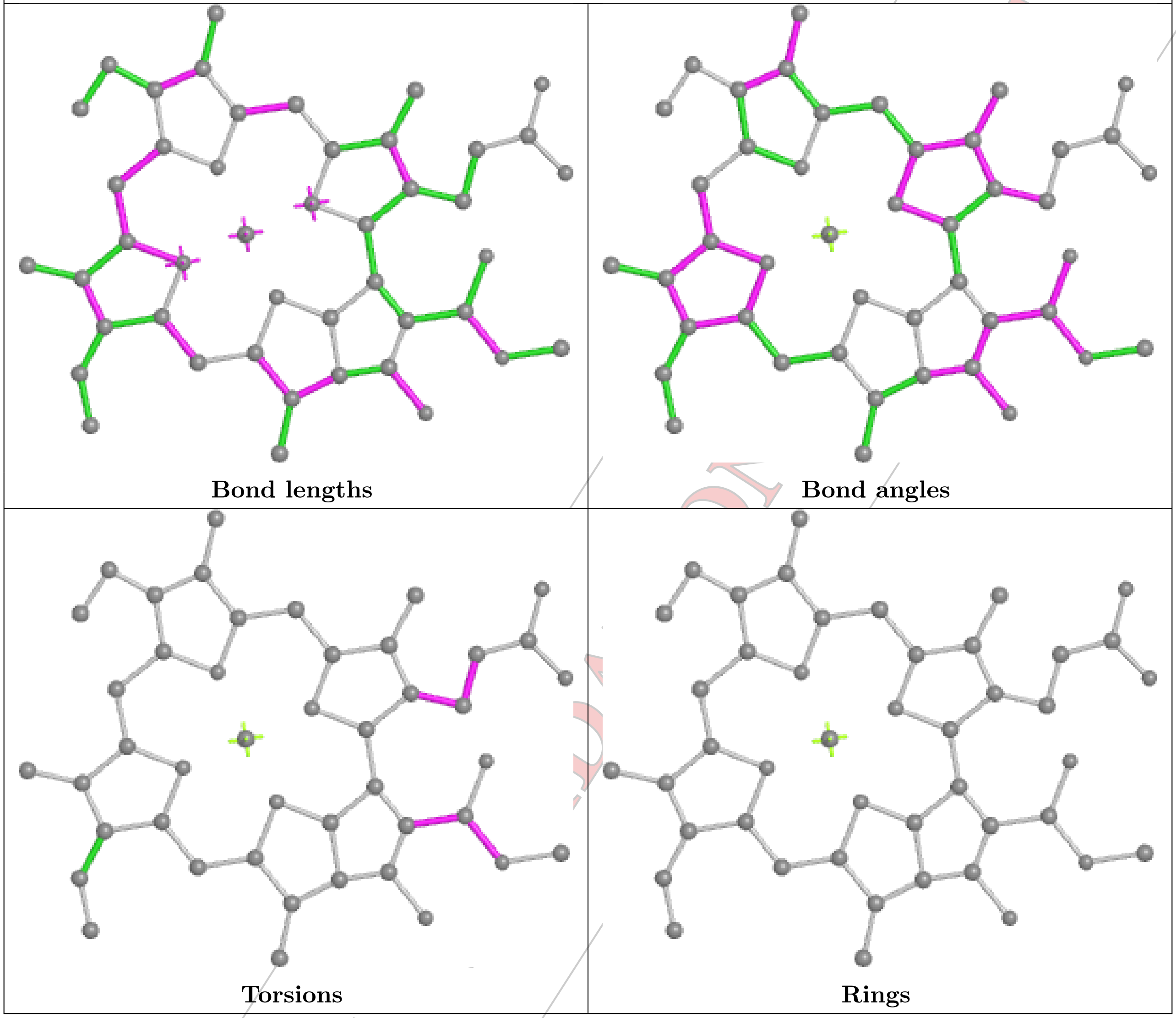

PRELIMINARY

Ligand CLA HA 1207

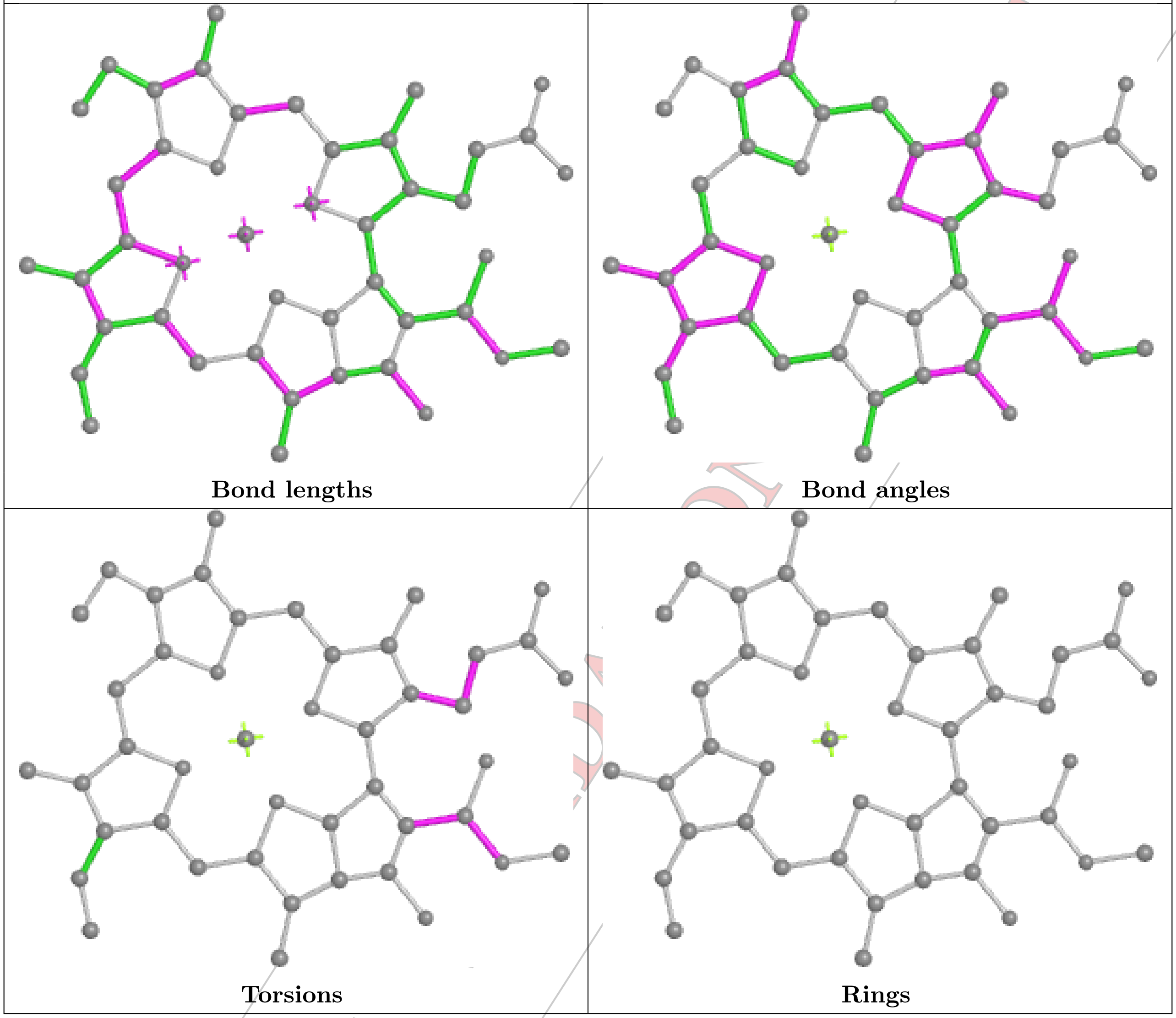

Ligand CLA HA 1208

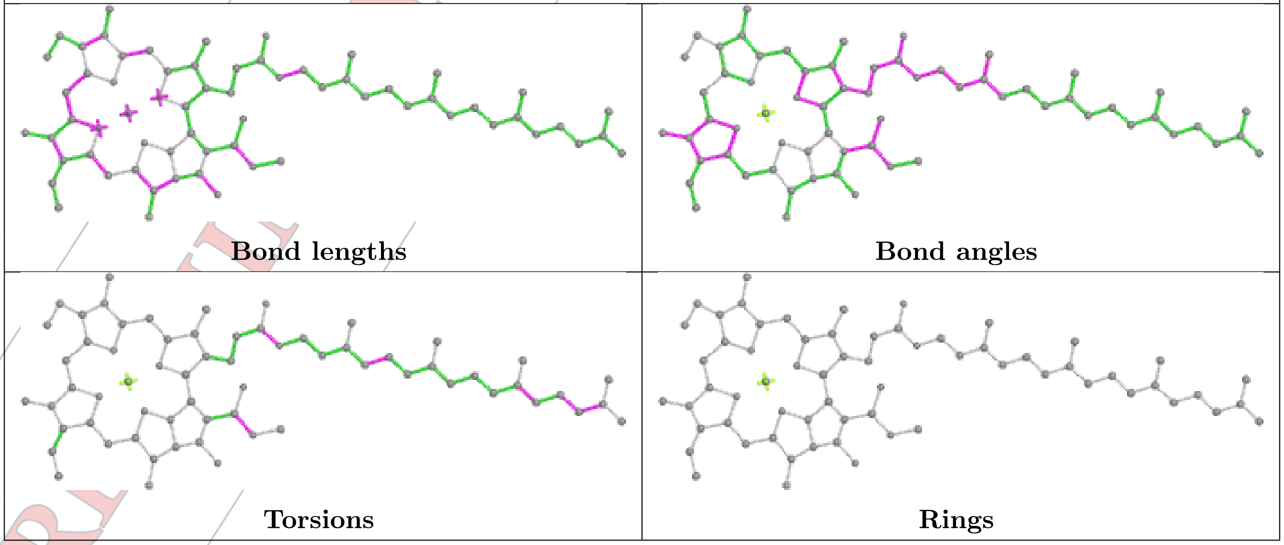

Ligand CLA HA 1209

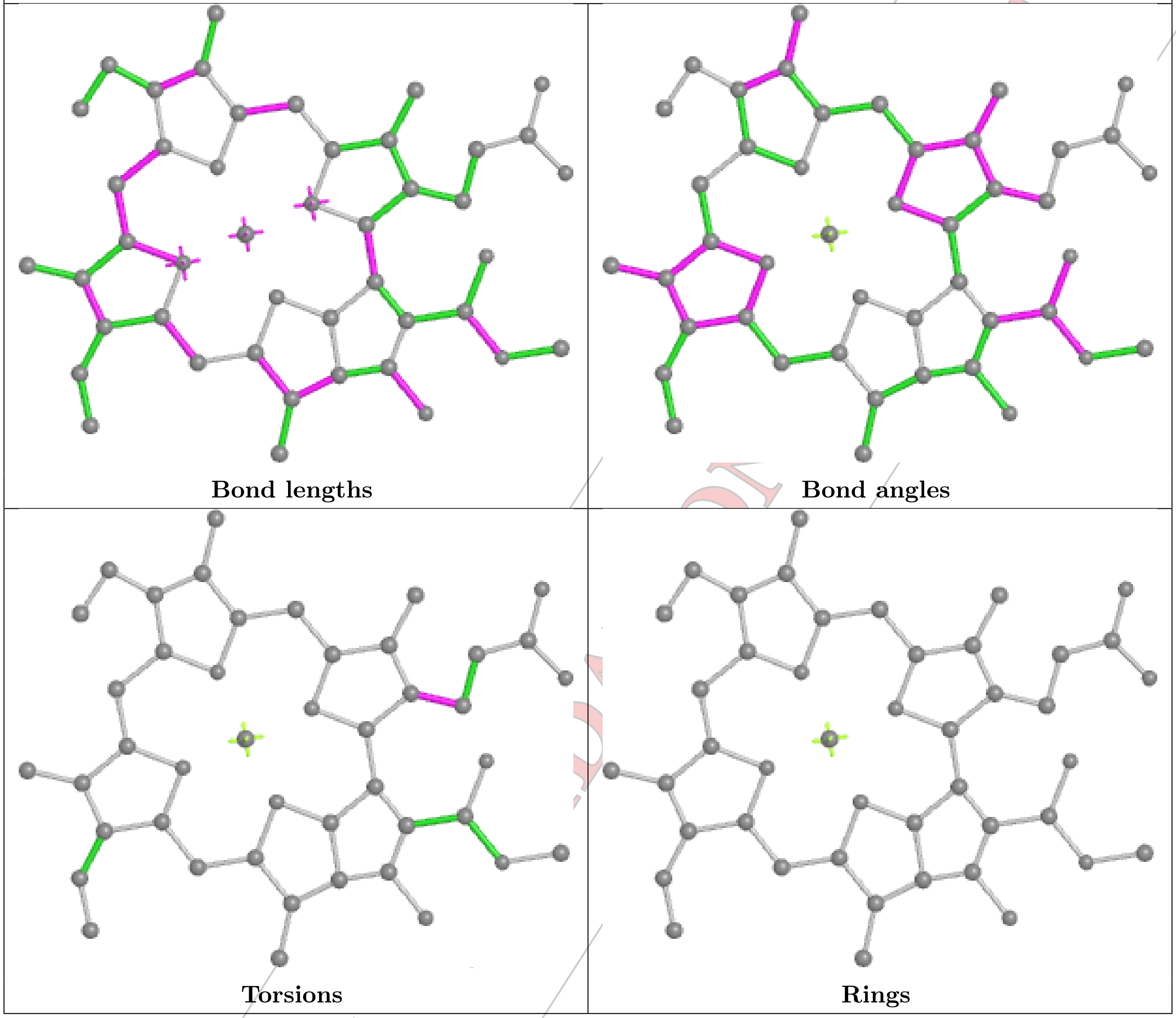

Ligand CLA HA 1210

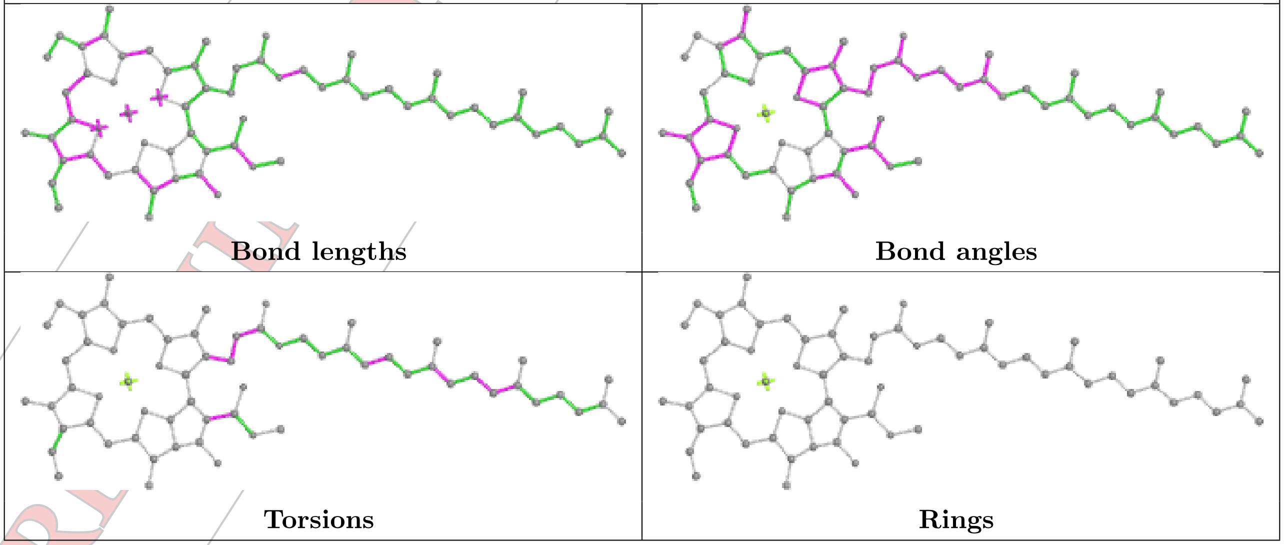

Ligand CLA HA 1211

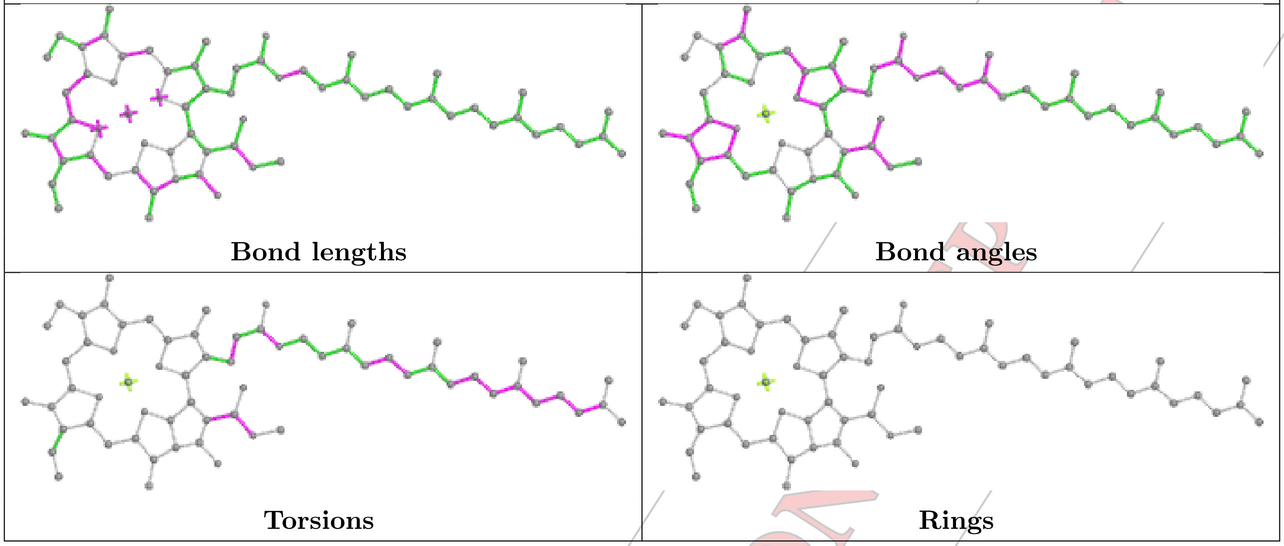

Ligand CLA HA 1212

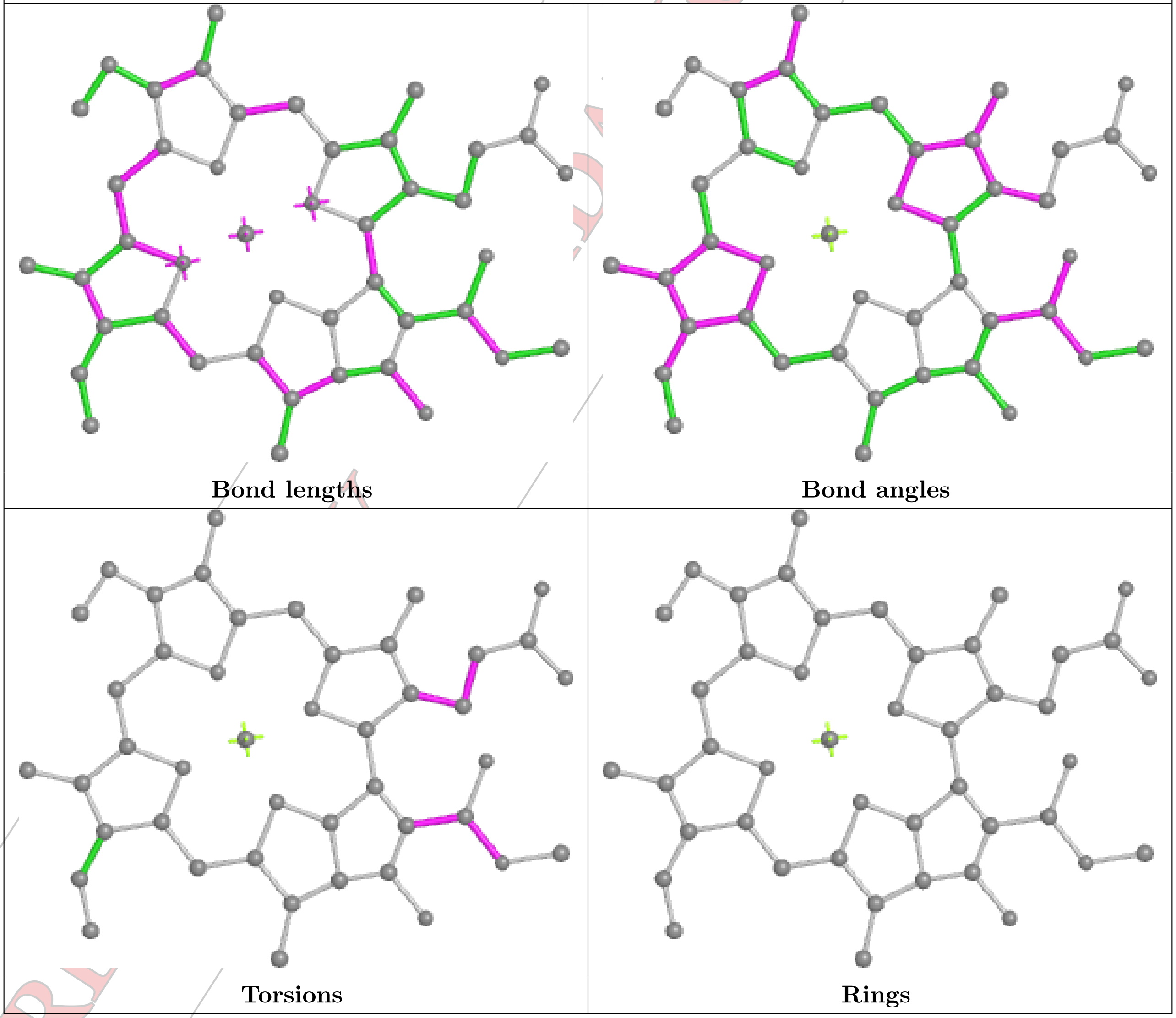

Ligand CLA HA 1213

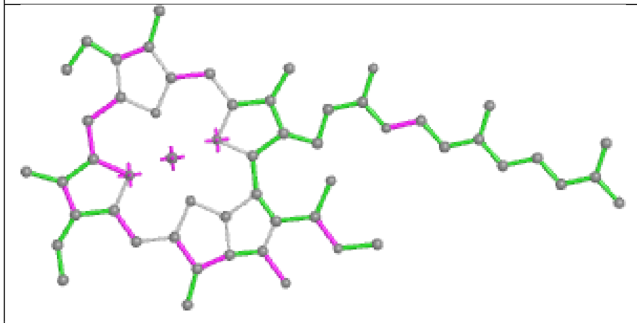

Bond lengths

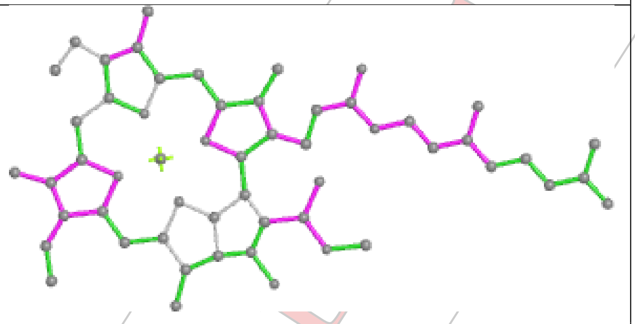

Bond angles

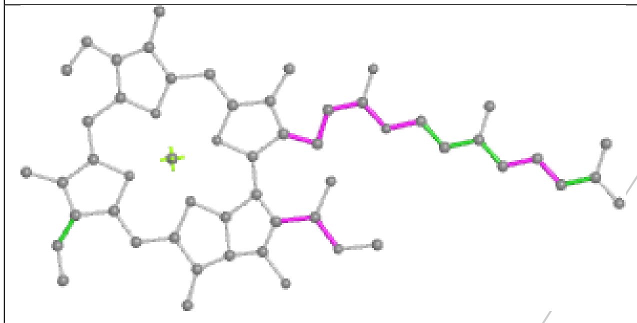

Torsions

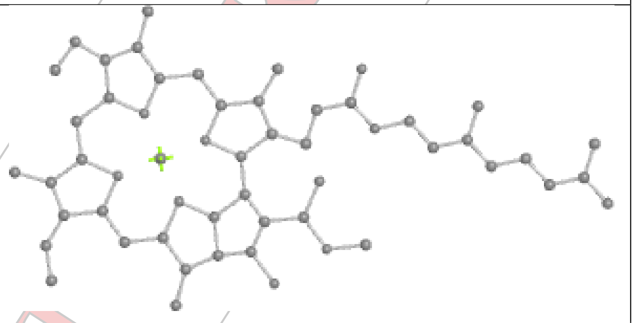

Rings

Ligand CLA HA 1214

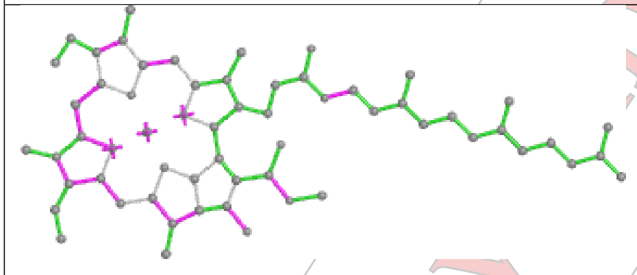

Bond lengths

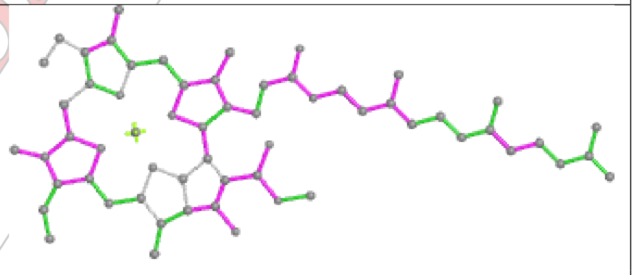

Bond angles

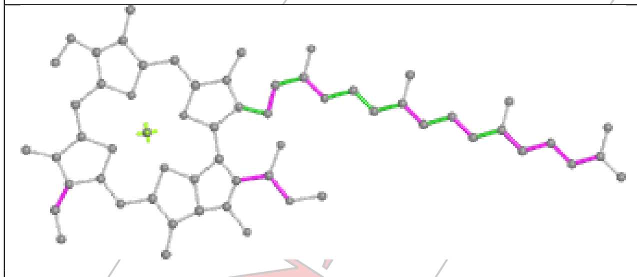

Torsions

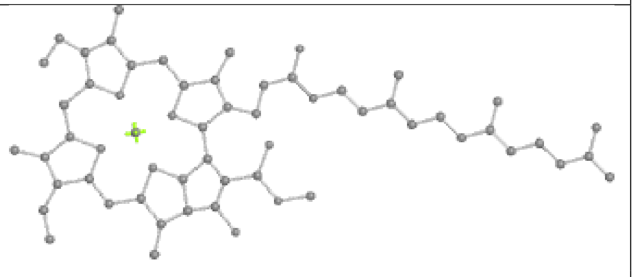

Rings

Ligand CLA HA 1215

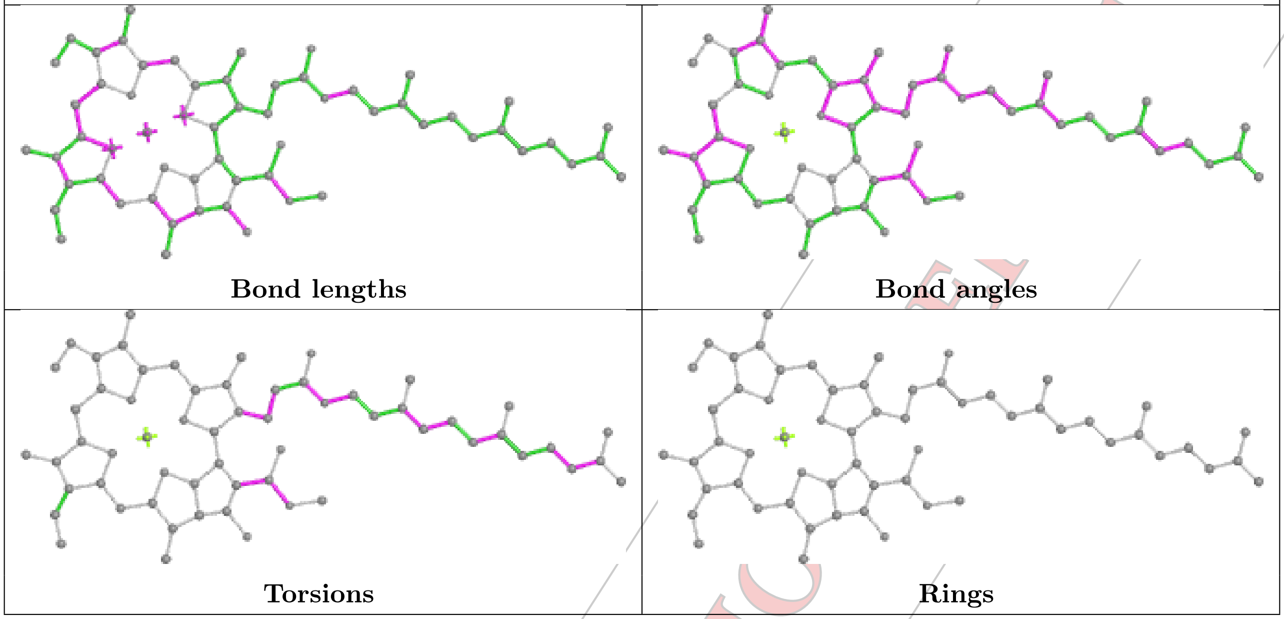

Ligand CLA HA 1216

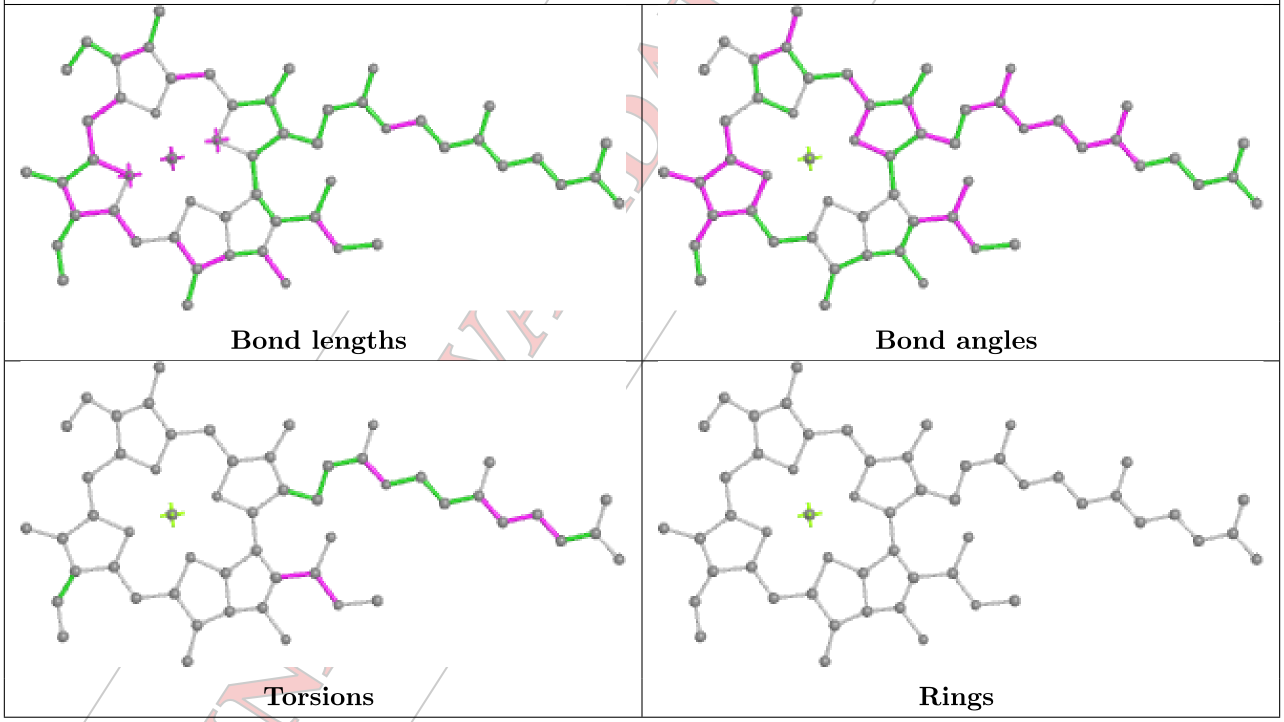

Ligand CLA HA 1217

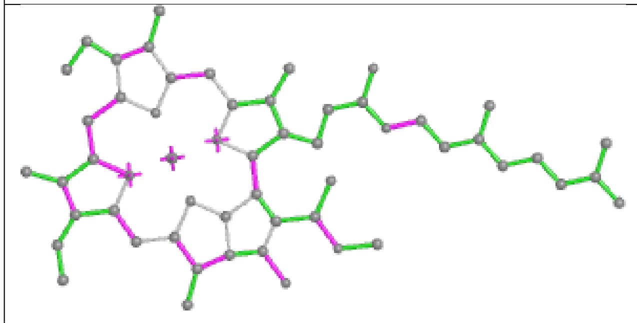

Bond lengths

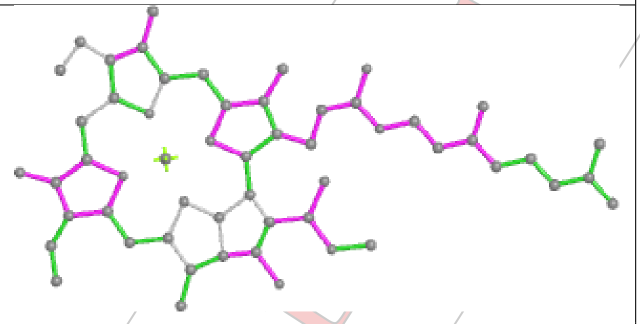

Bond angles

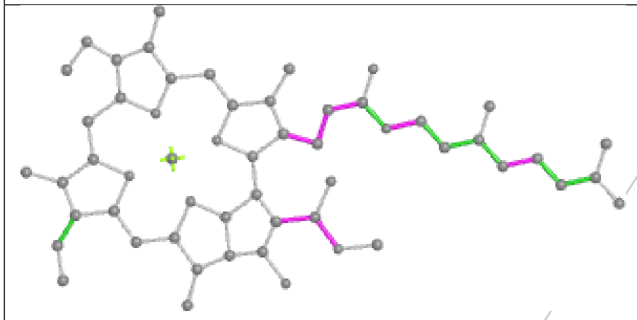

Torsions

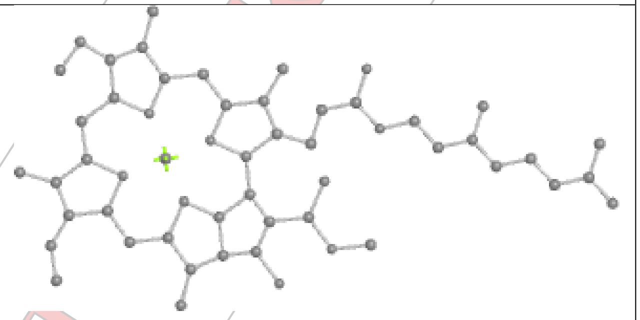

Rings

PRELIMINARY VALIDATION

Ligand CLA HA 1218

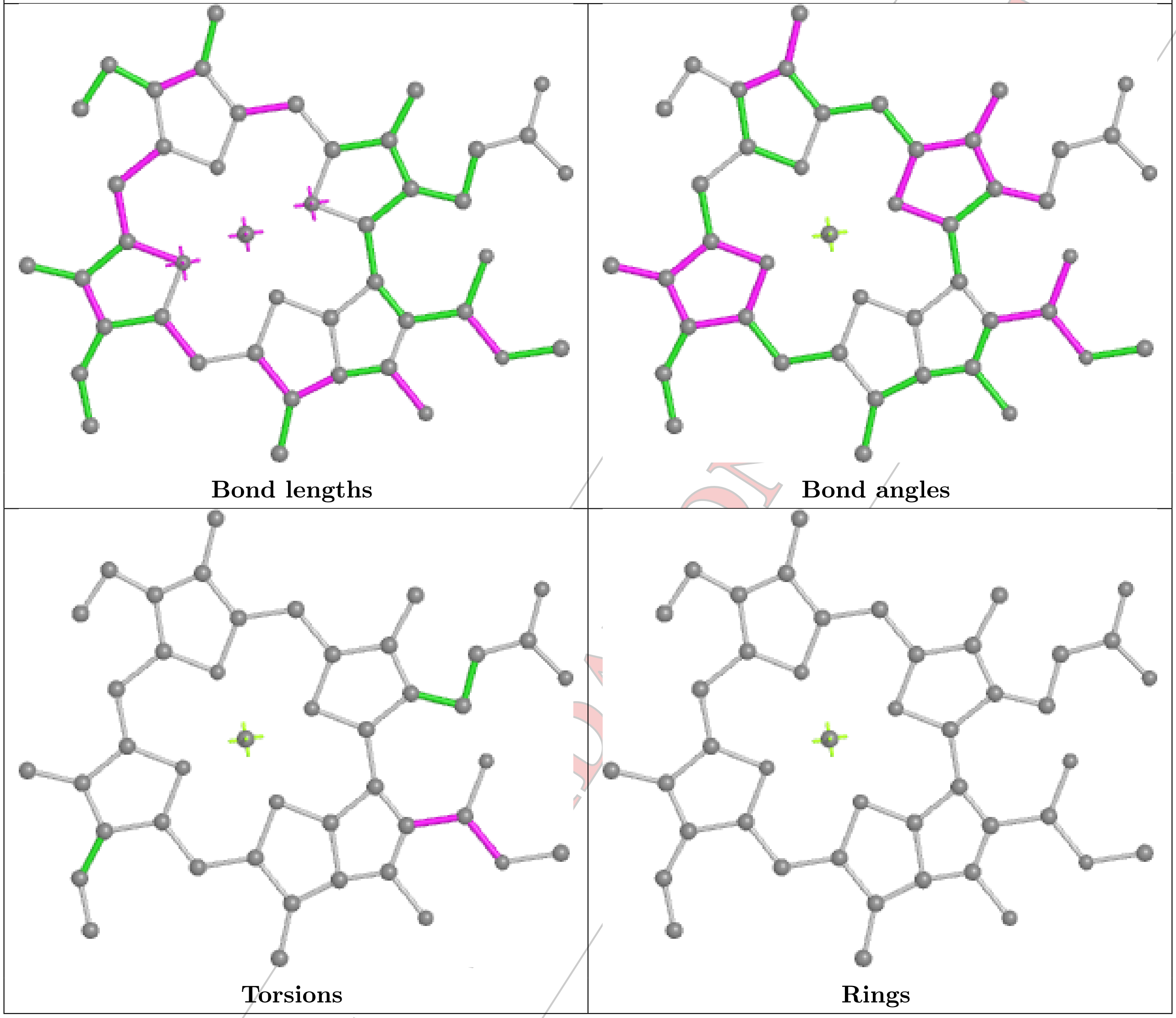

PRELIMINARY

Ligand CLA HA 1219

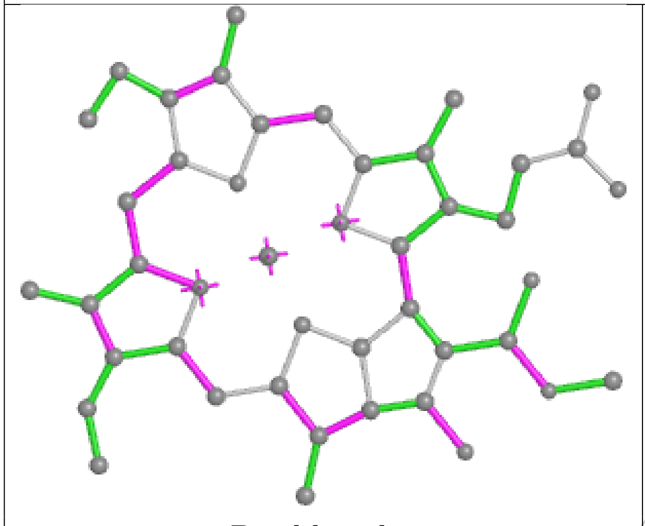

Bond lengths

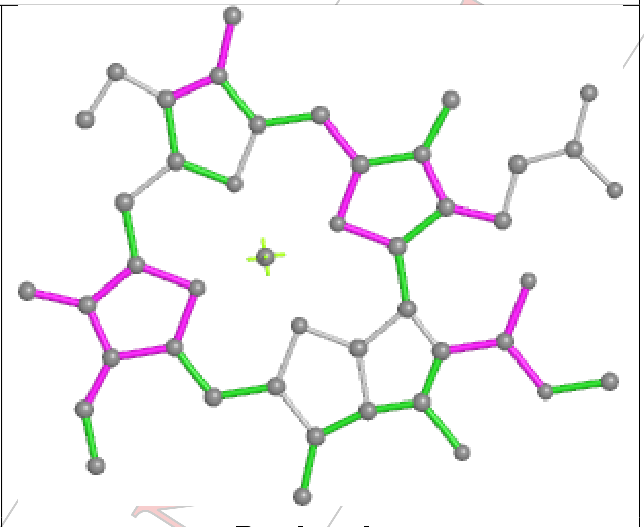

Bond angles

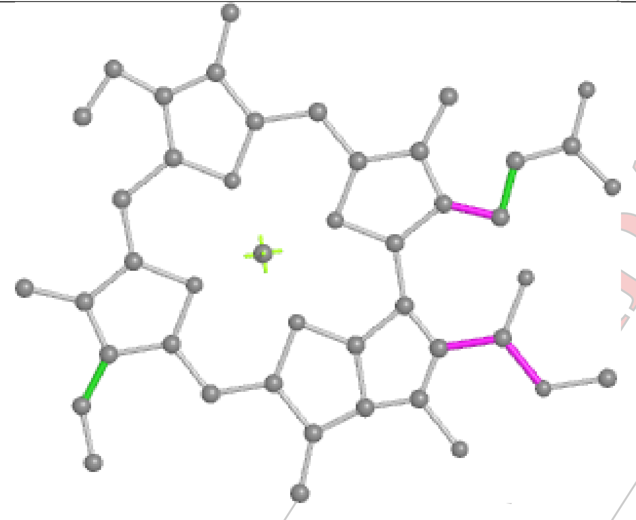

Torsions

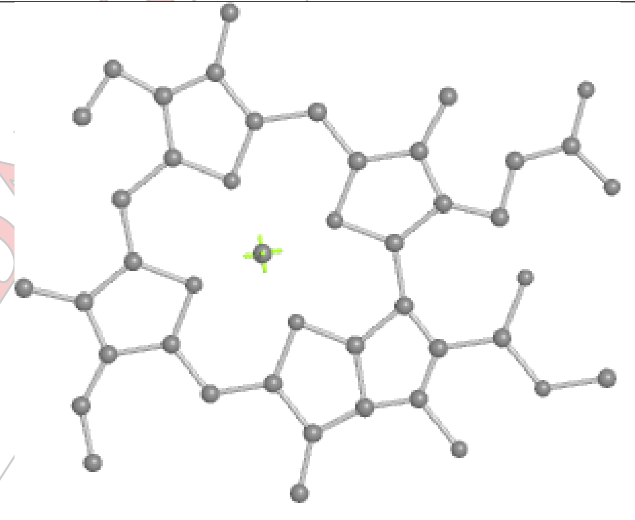

Rings

PRELIMINARY

Ligand CLA HA 1220

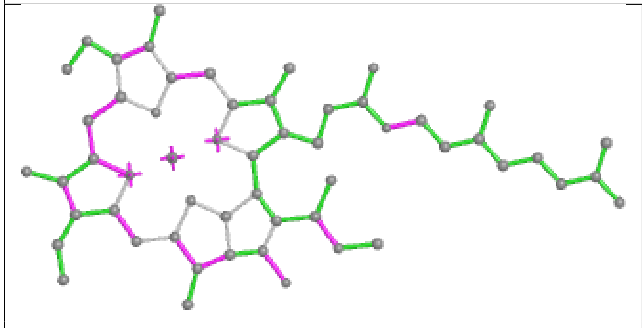
Bond lengths

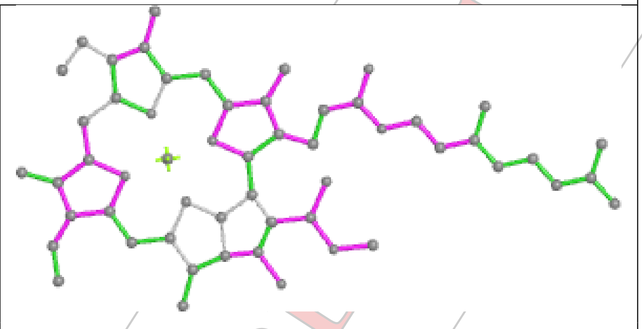
Bond angles

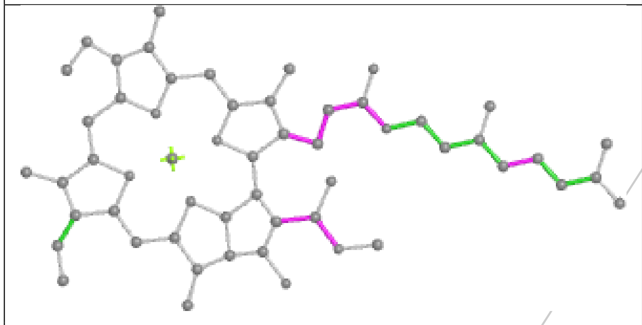
Torsions

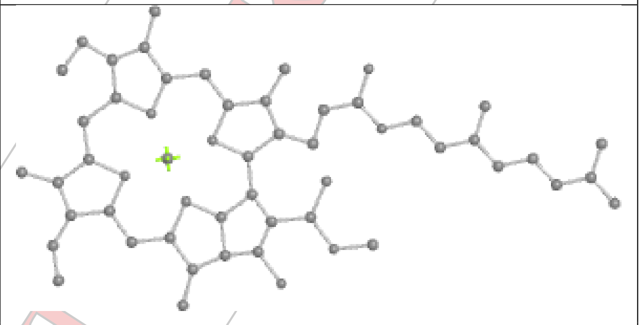
Rings

Ligand CLA HA 1221

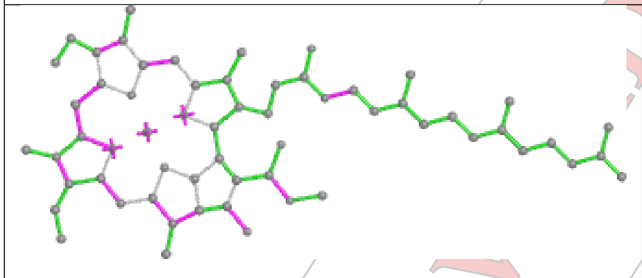
Bond lengths

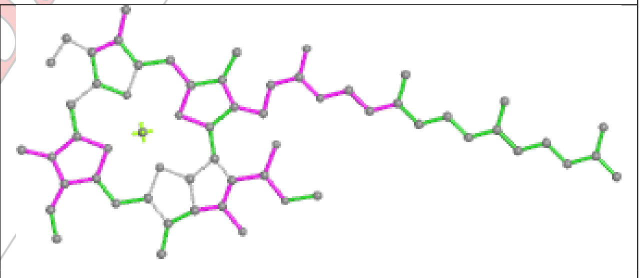
Bond angles

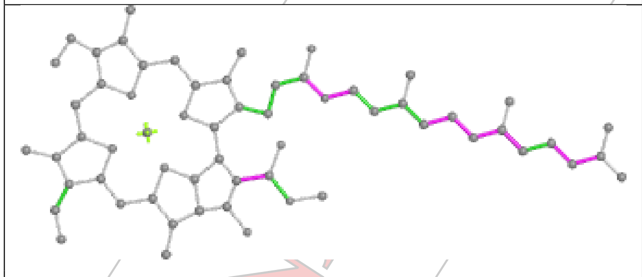
Torsions

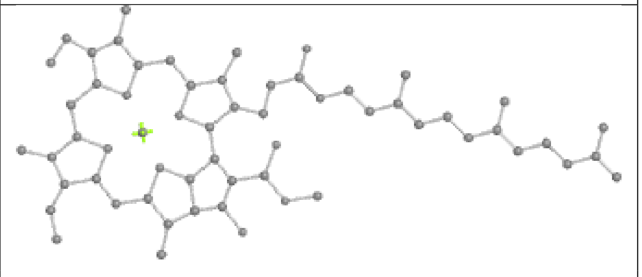
Rings

PRELIMINARY

Ligand CLA HA 1222

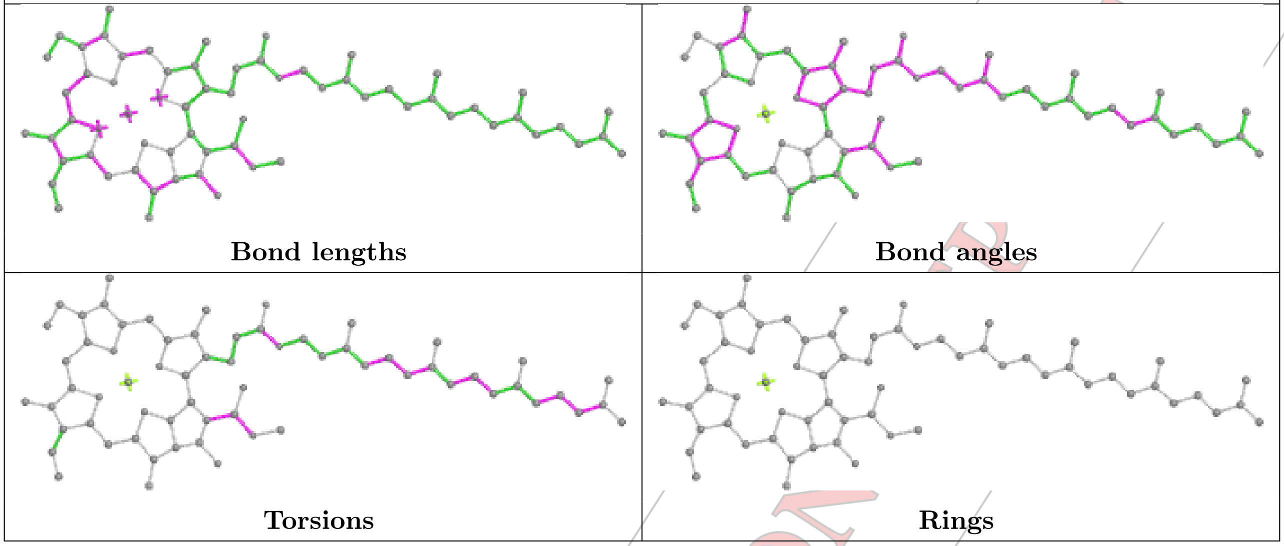

Ligand CLA HA 1223

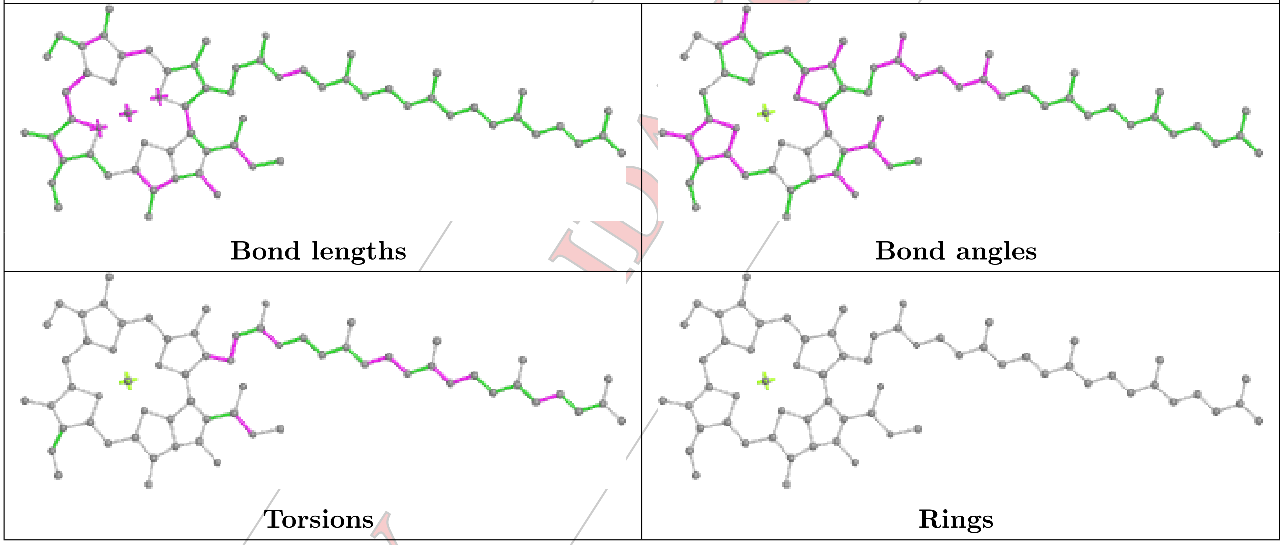

Ligand CLA HA 1224

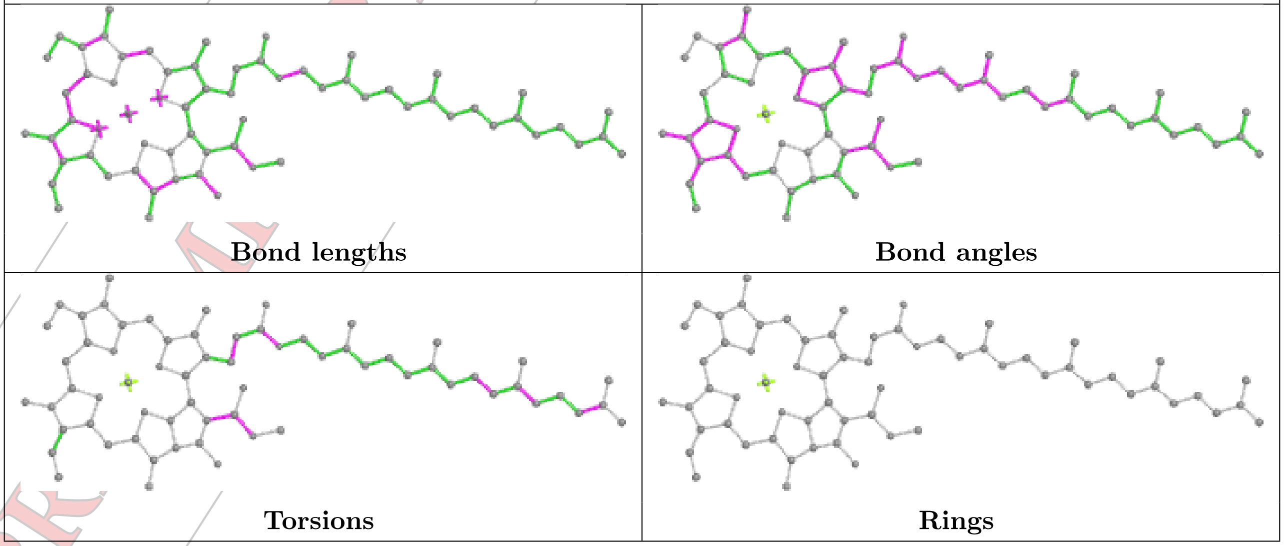

Ligand CLA HA 1225

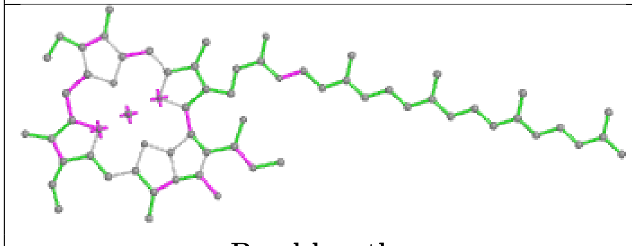
Bond lengths

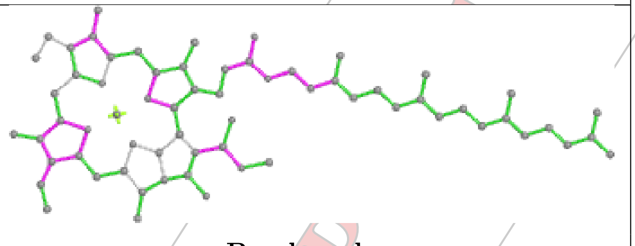
Bond angles

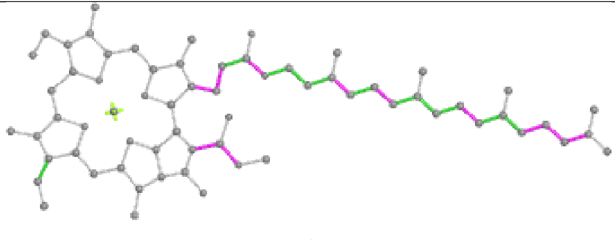
Torsions

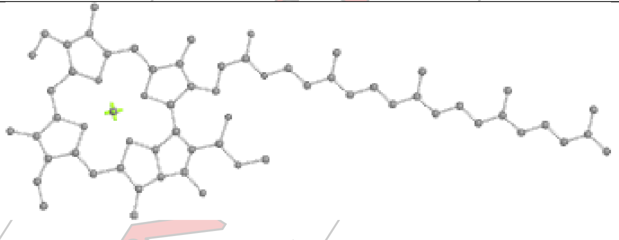
Rings

Ligand CLA HA 1226

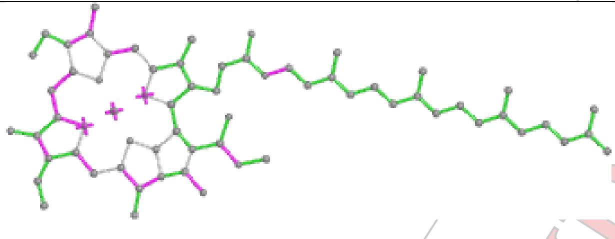
Bond lengths

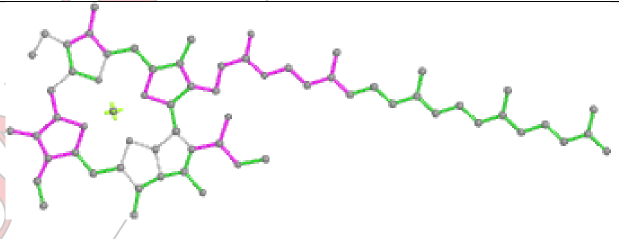
Bond angles

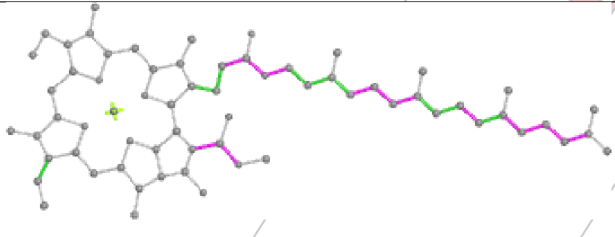
Torsions

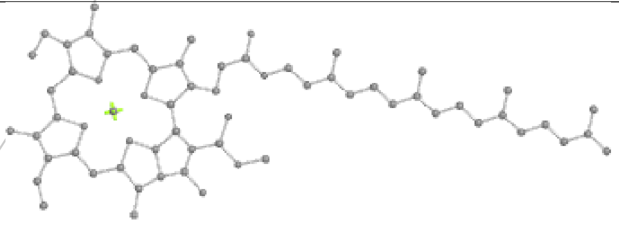
Rings

PRELIMINARY

Ligand CLA HA 1227

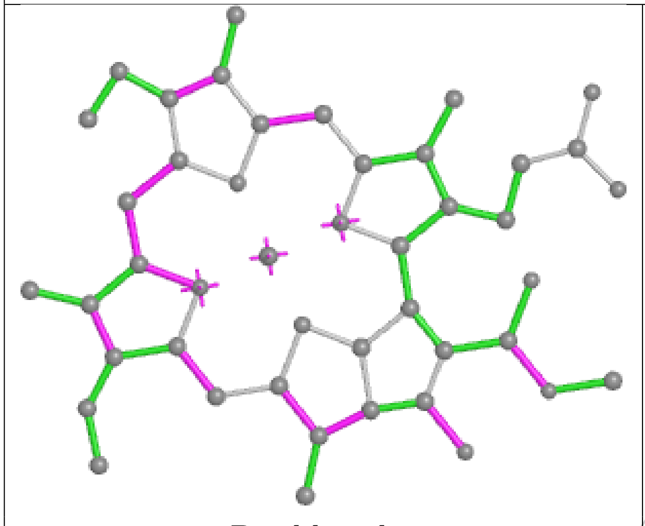

Bond lengths

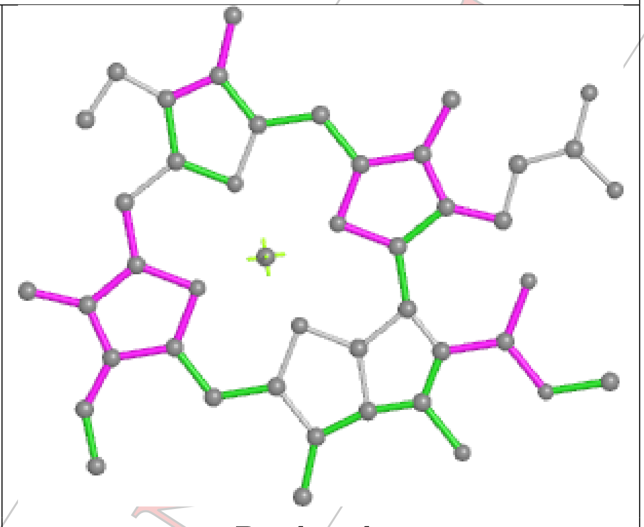

Bond angles

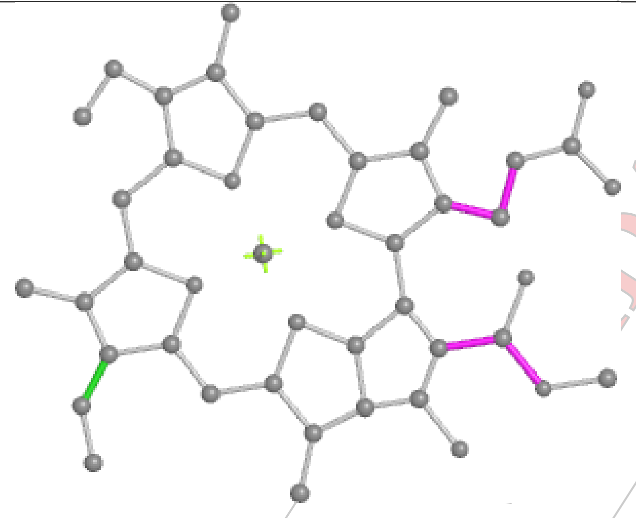

Torsions

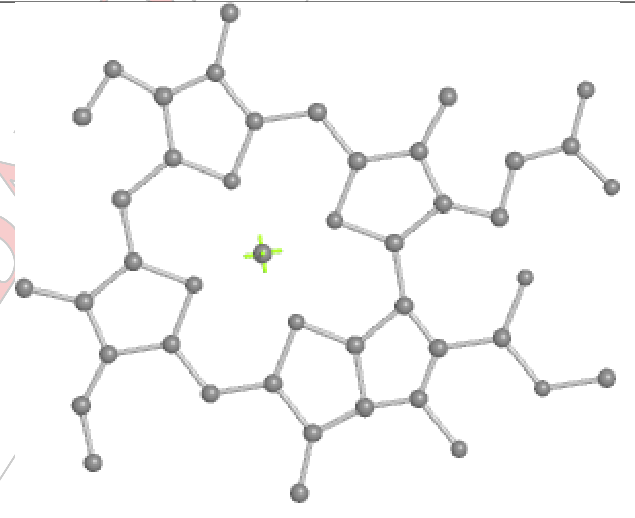

Rings

PRELIMINARY

Ligand CLA HA 1228

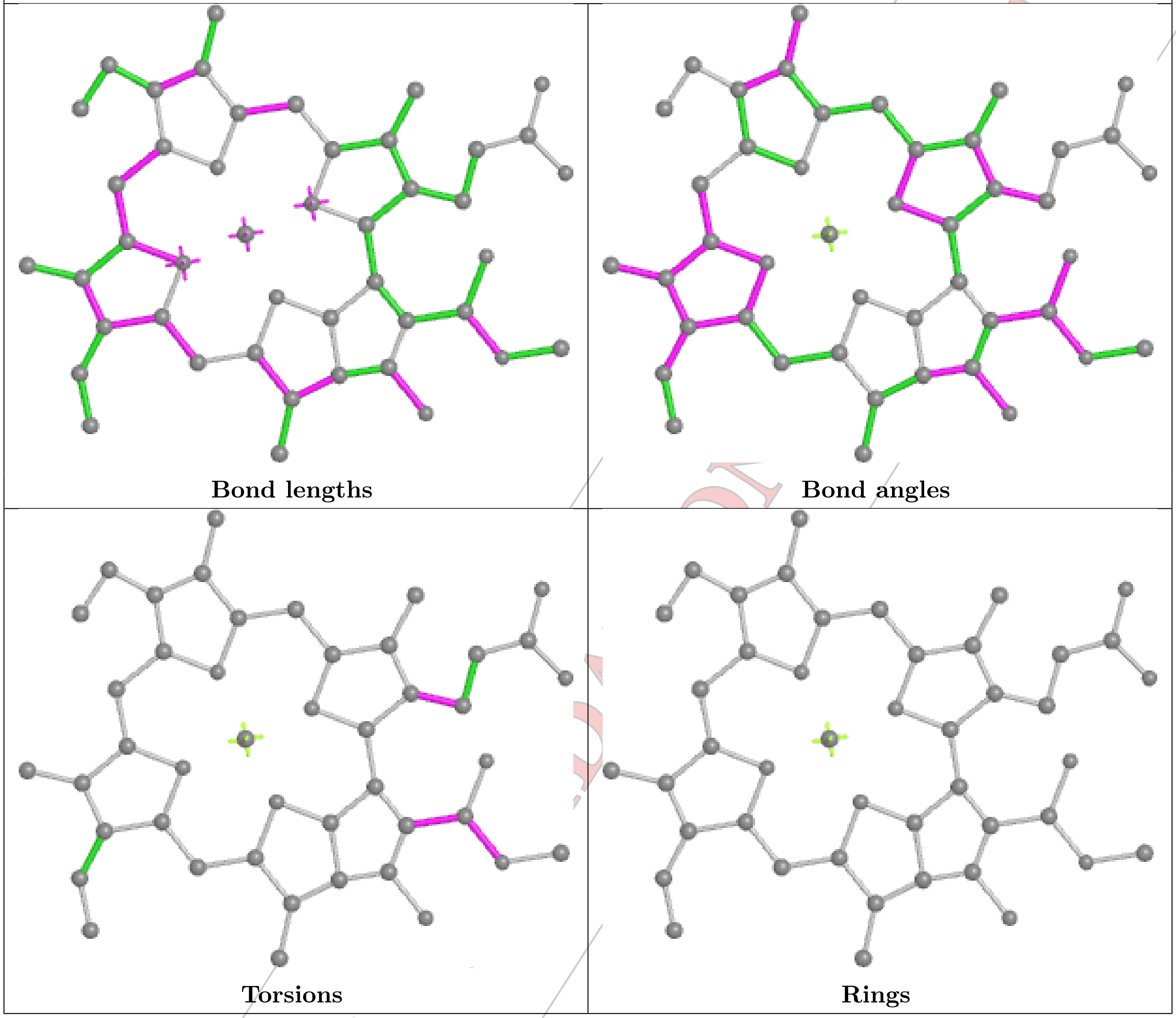

Ligand CLA HA 1229

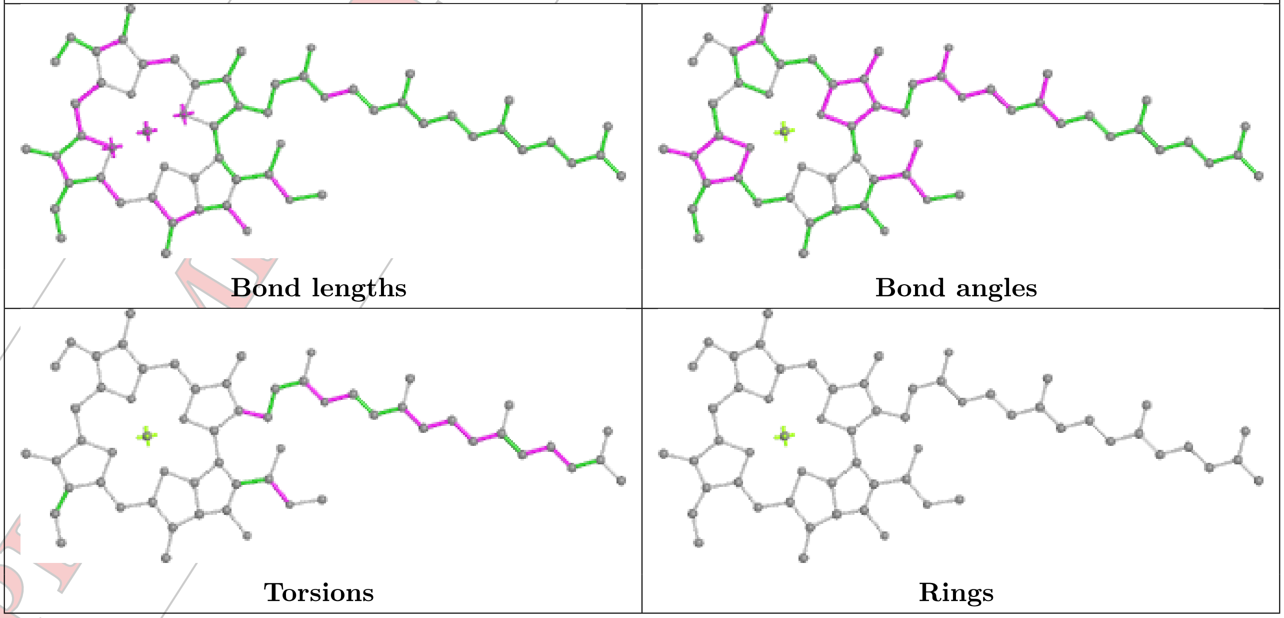

Ligand CLA HA 1231

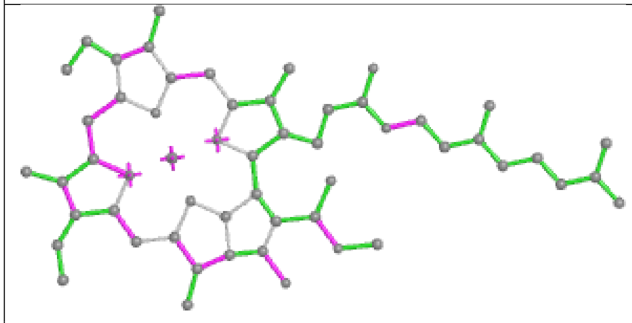

Bond lengths

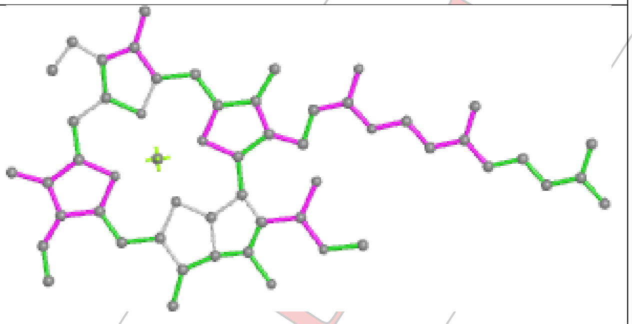

Bond angles

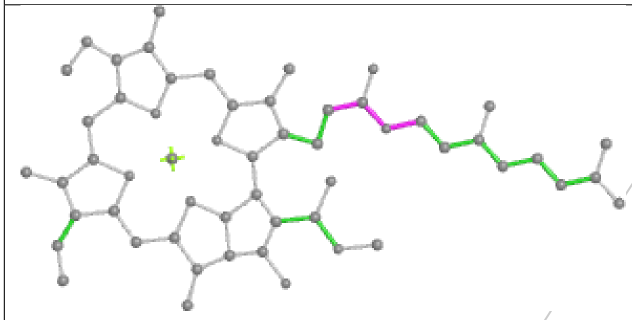

Torsions

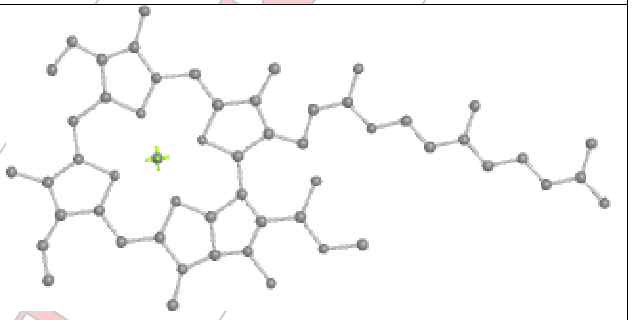

Rings

PRELIMINARY VALIDATION

Ligand CLA HA 1232

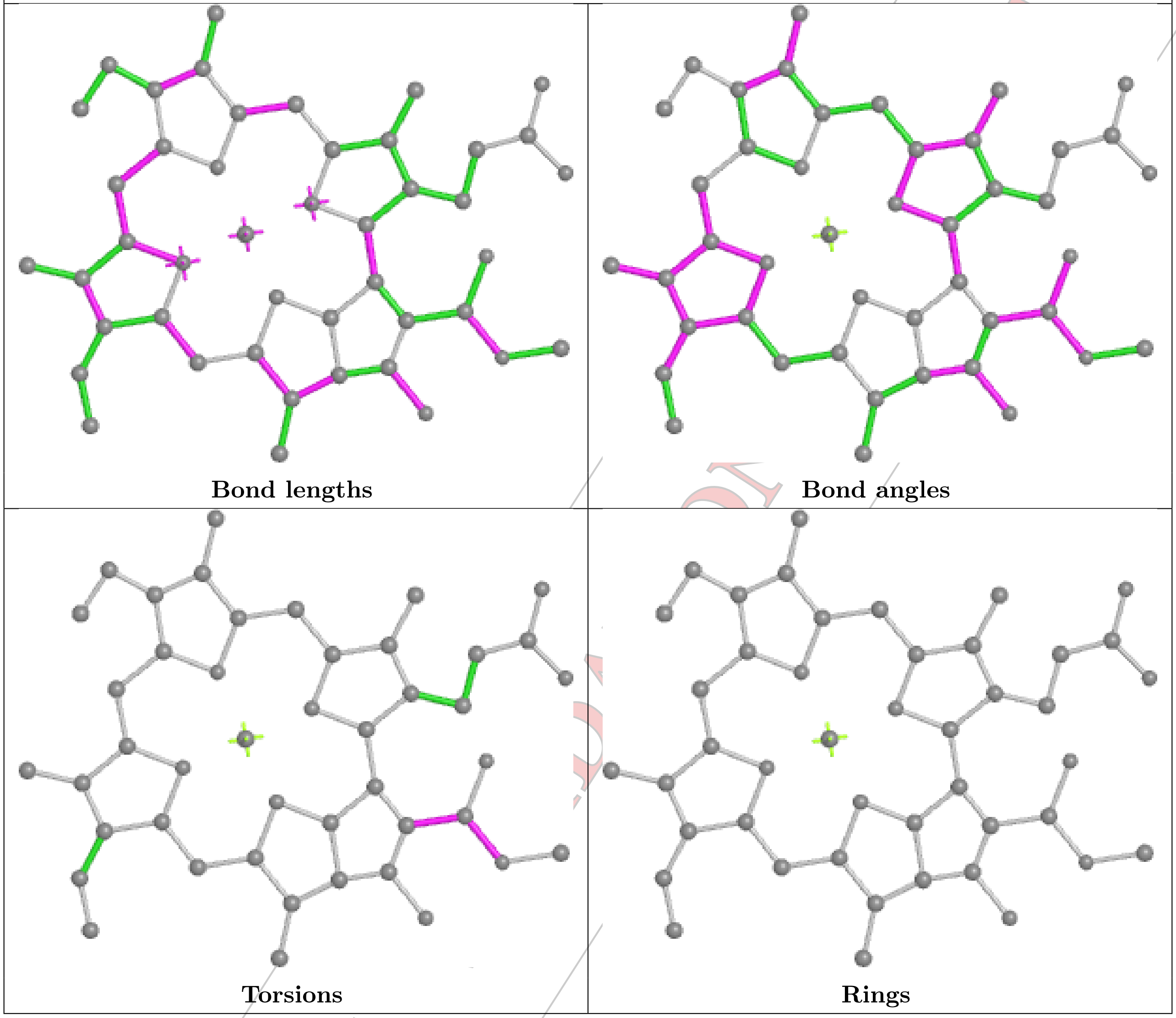

PRELIMINARY

Ligand CLA HA 1233

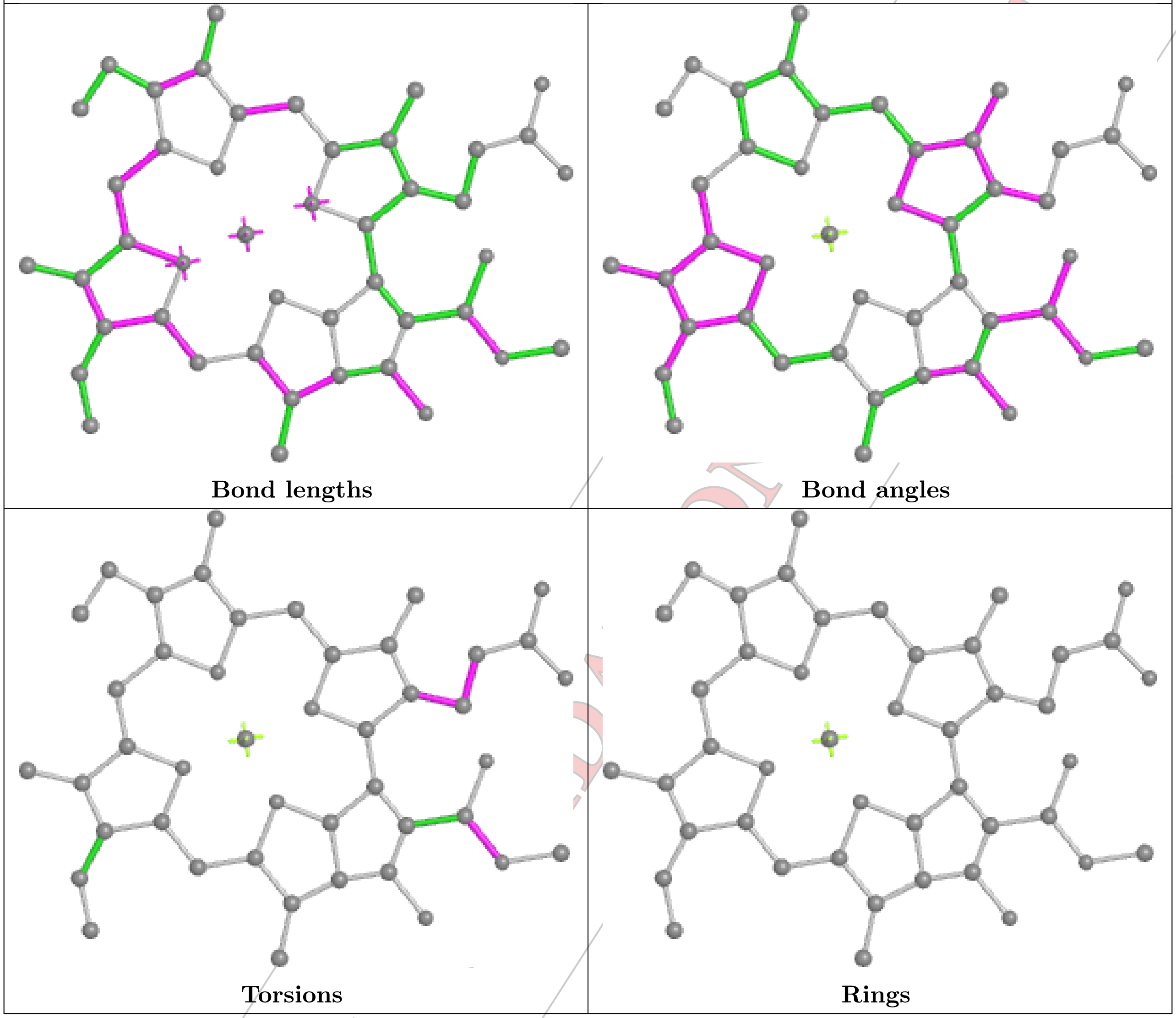

PRELIMINARY

Ligand CLA HA 1234

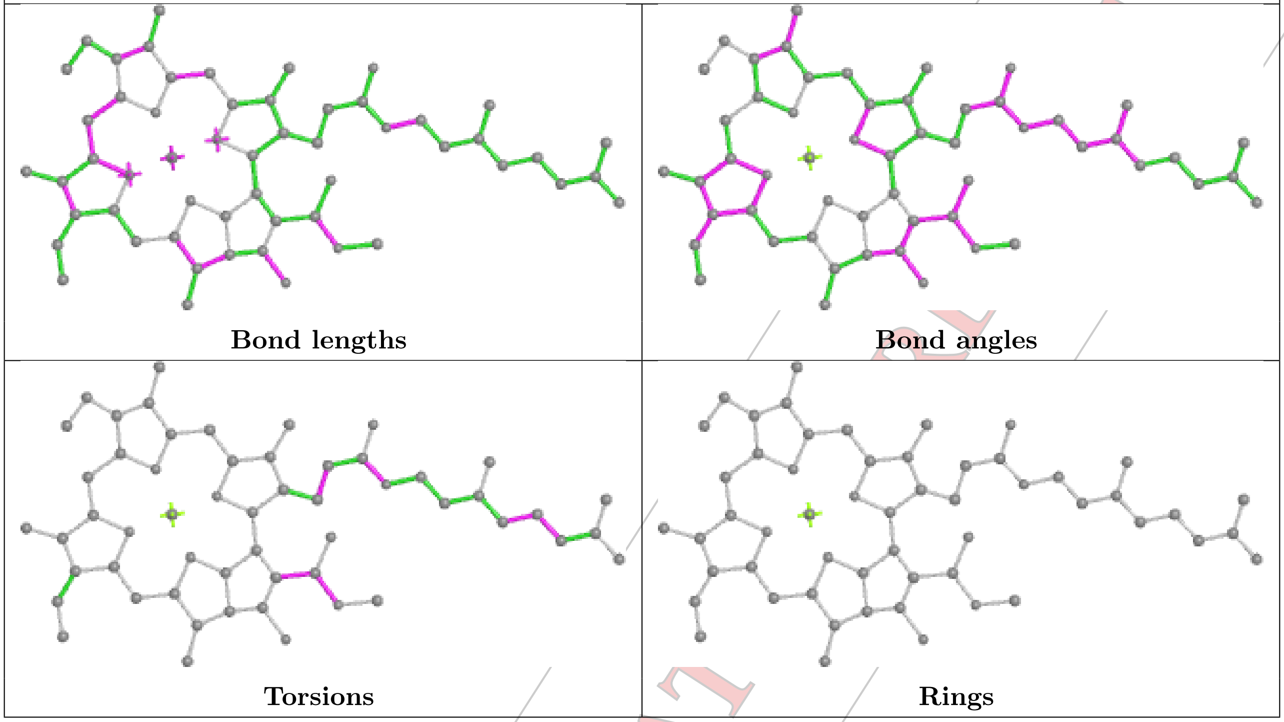

Ligand CLA HA 1235

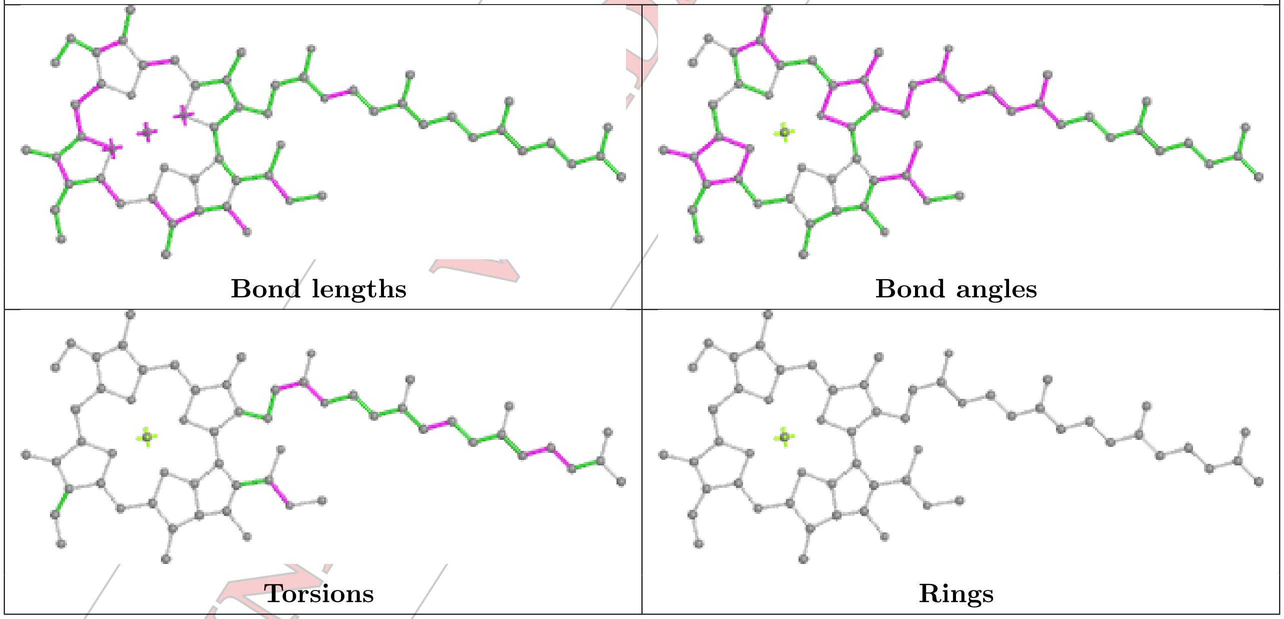

Ligand CLA HA 1236

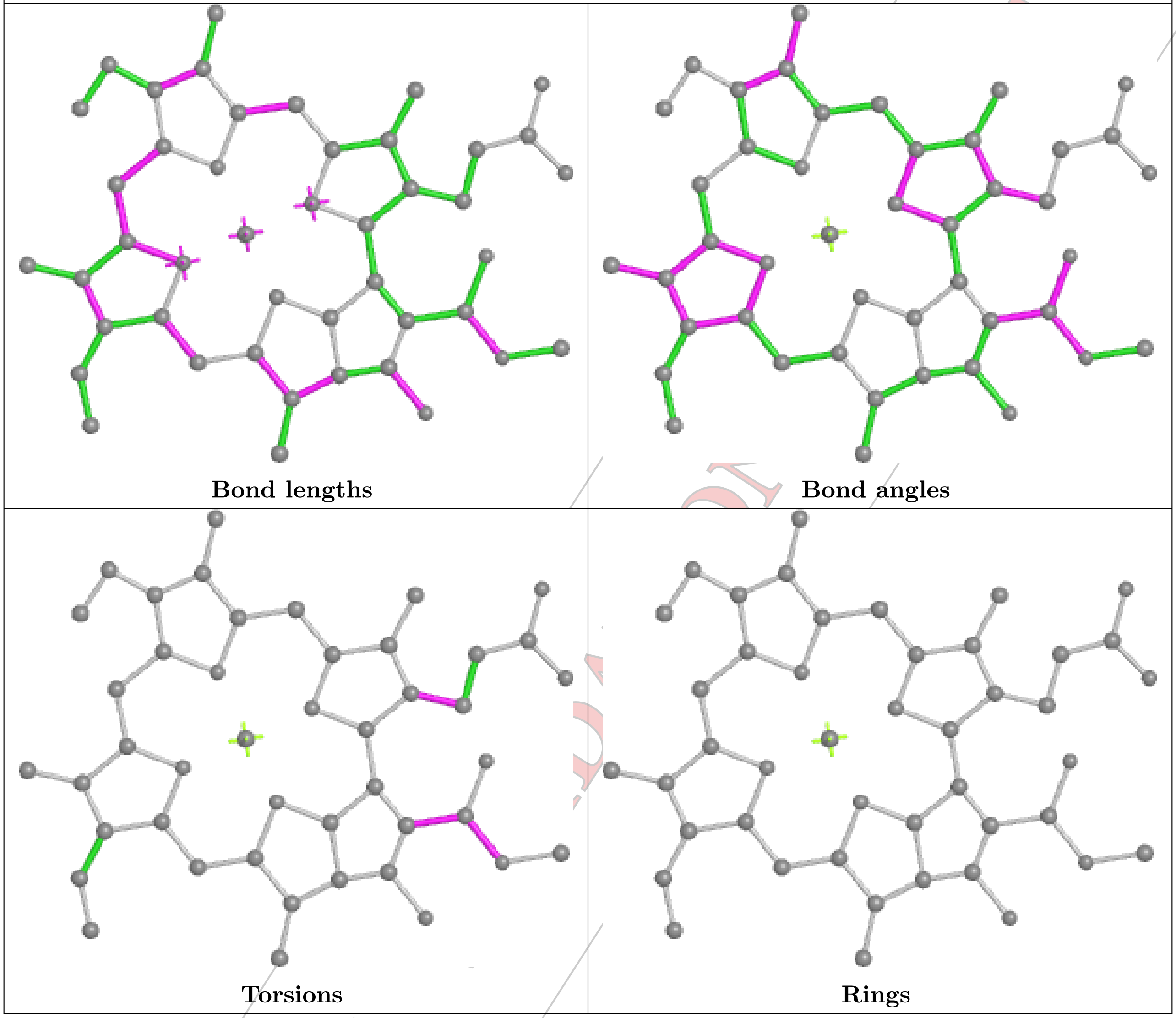

Ligand CLA HA 1238

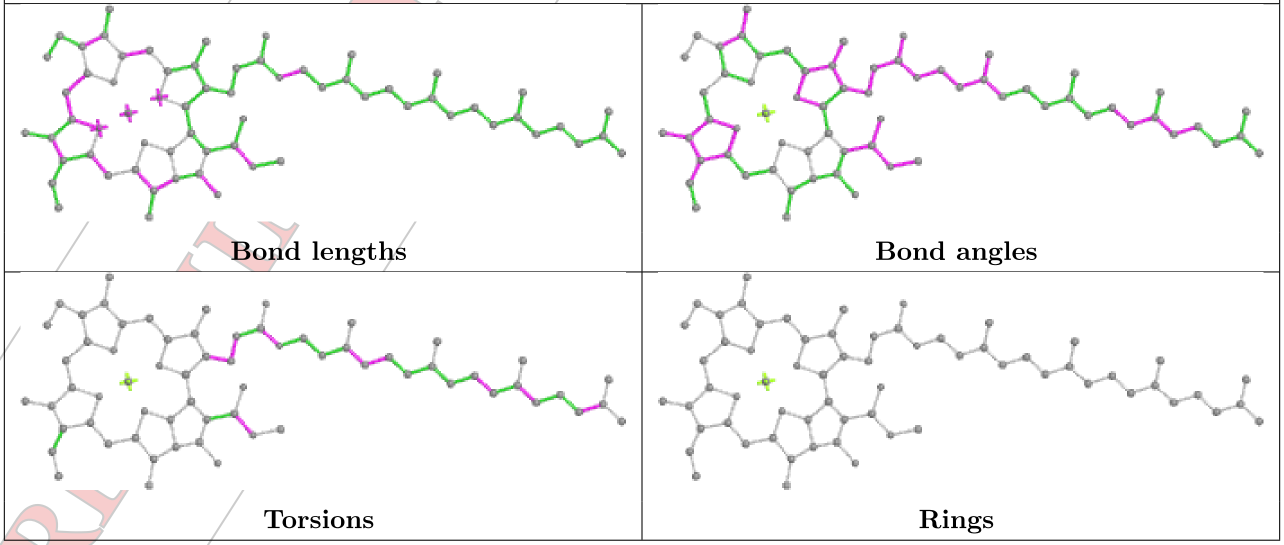

Ligand CLA HA 1239

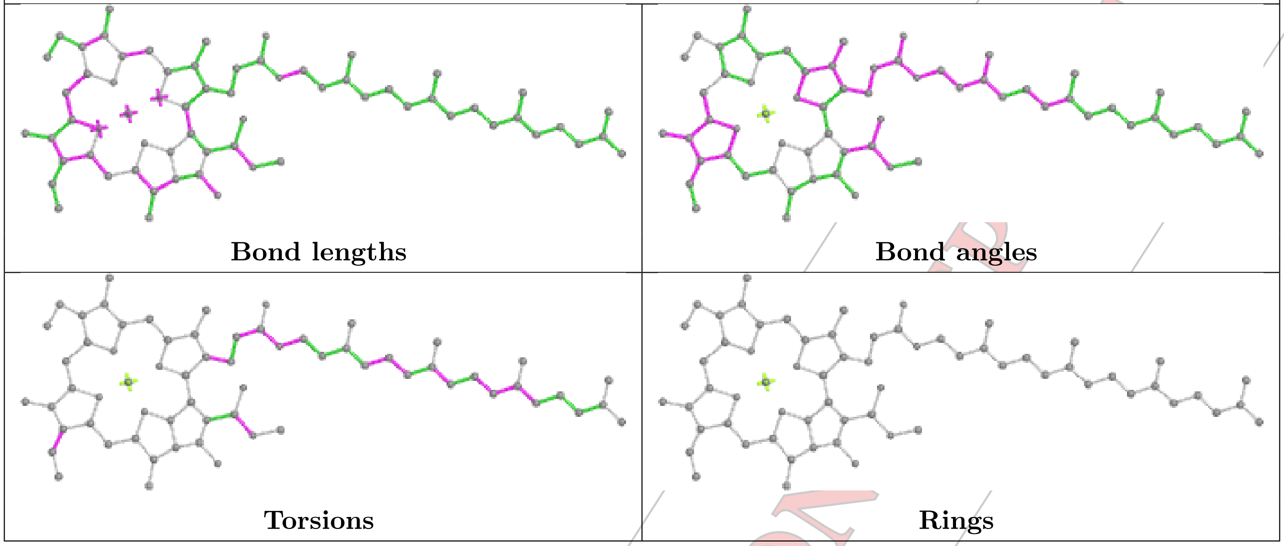

Ligand PQN HA 2002

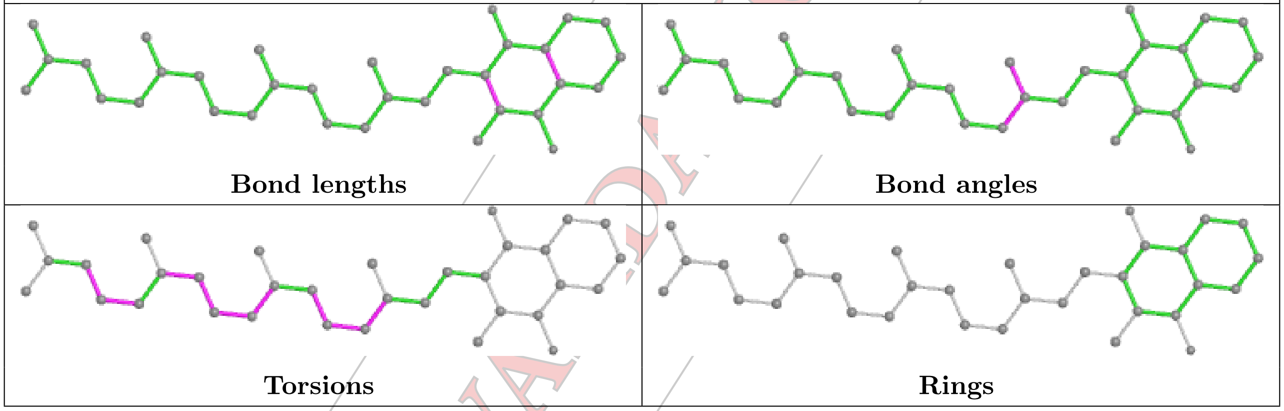

Ligand BCR HA 4004

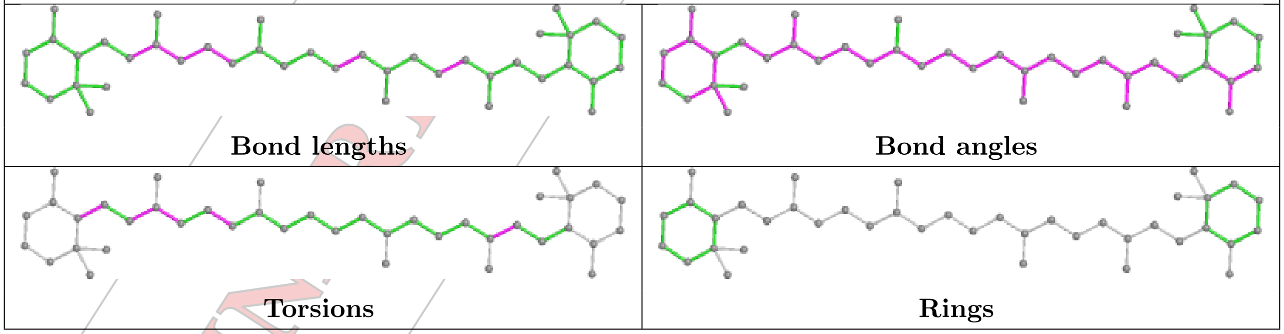

Ligand BCR HA 4005

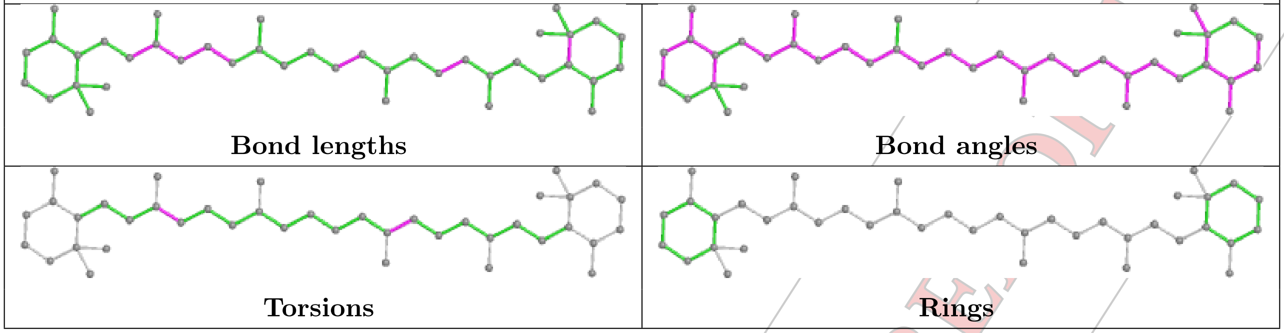

Ligand BCR HA 4006

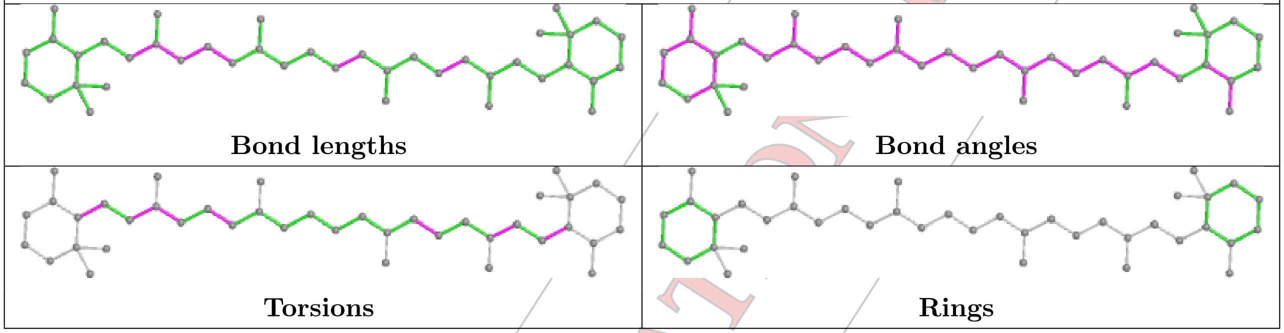

Ligand BCR HA 4009

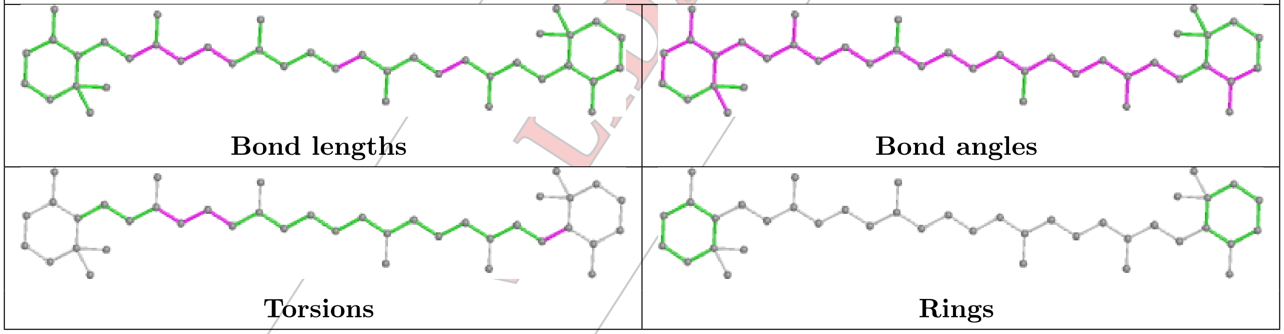

Ligand BCR HA 4010

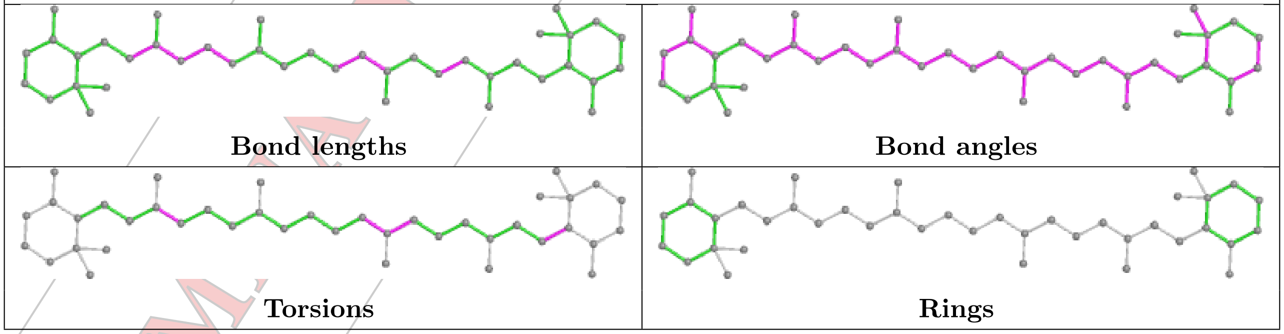

Ligand BCR HA 4012

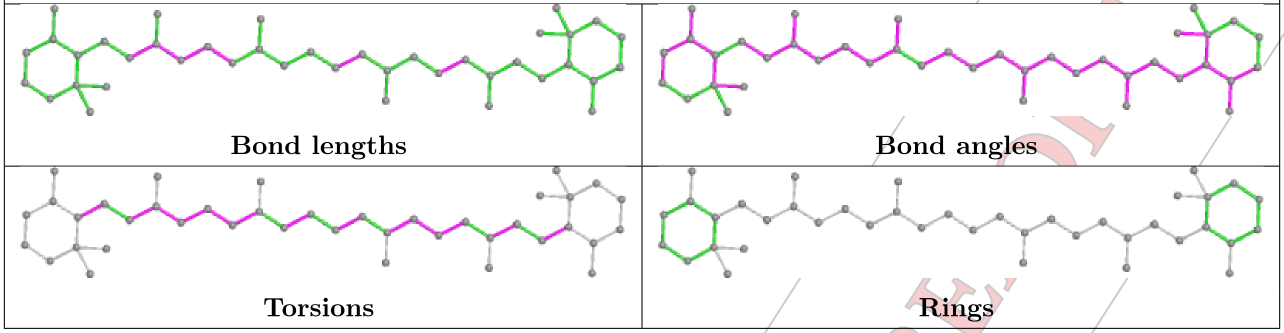

Ligand BCR HA 4013

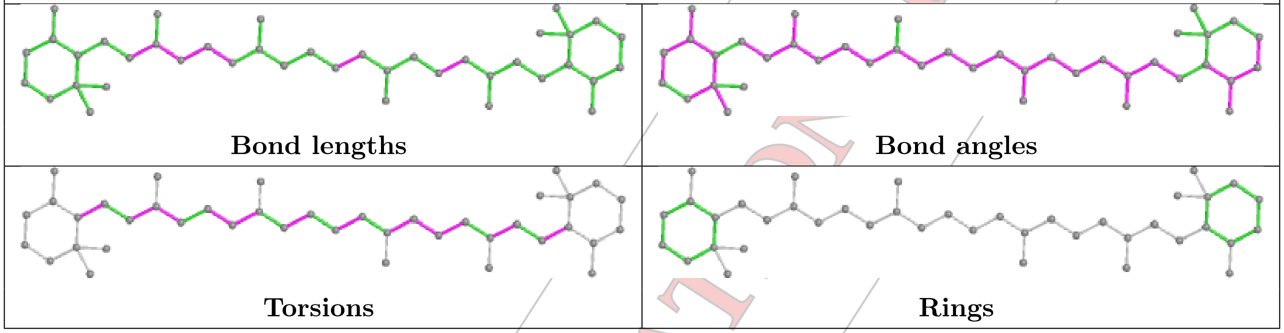

Ligand BCR HA 4014

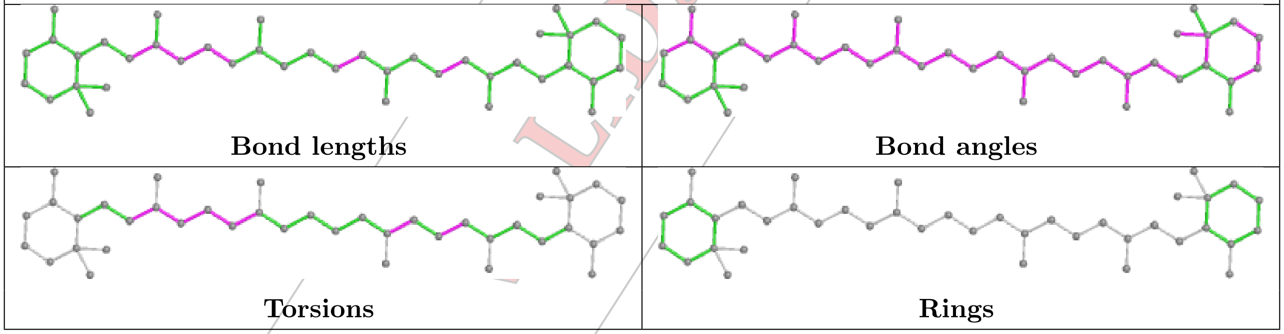

Ligand BCR HA 4016

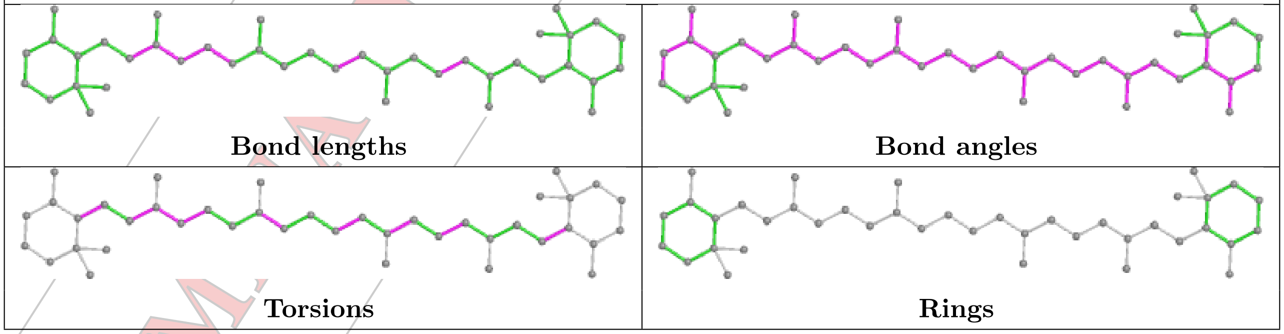

Ligand BCR HA 4017

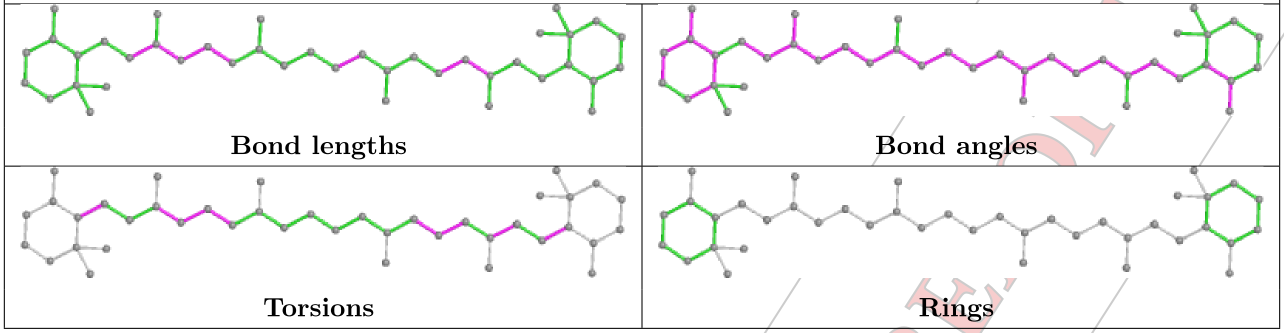

Ligand LMG HA 5002

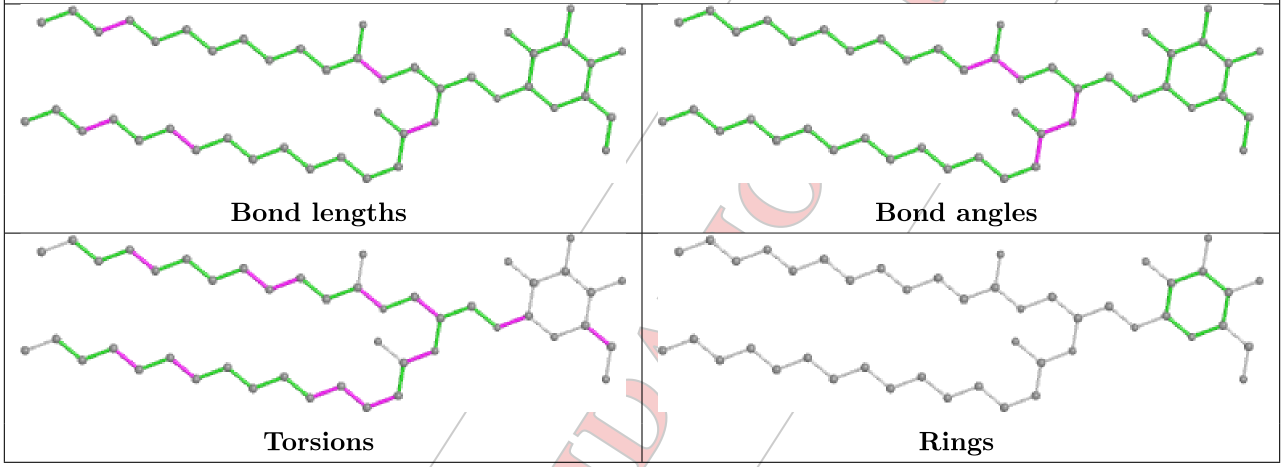

Ligand LHG HA 5004

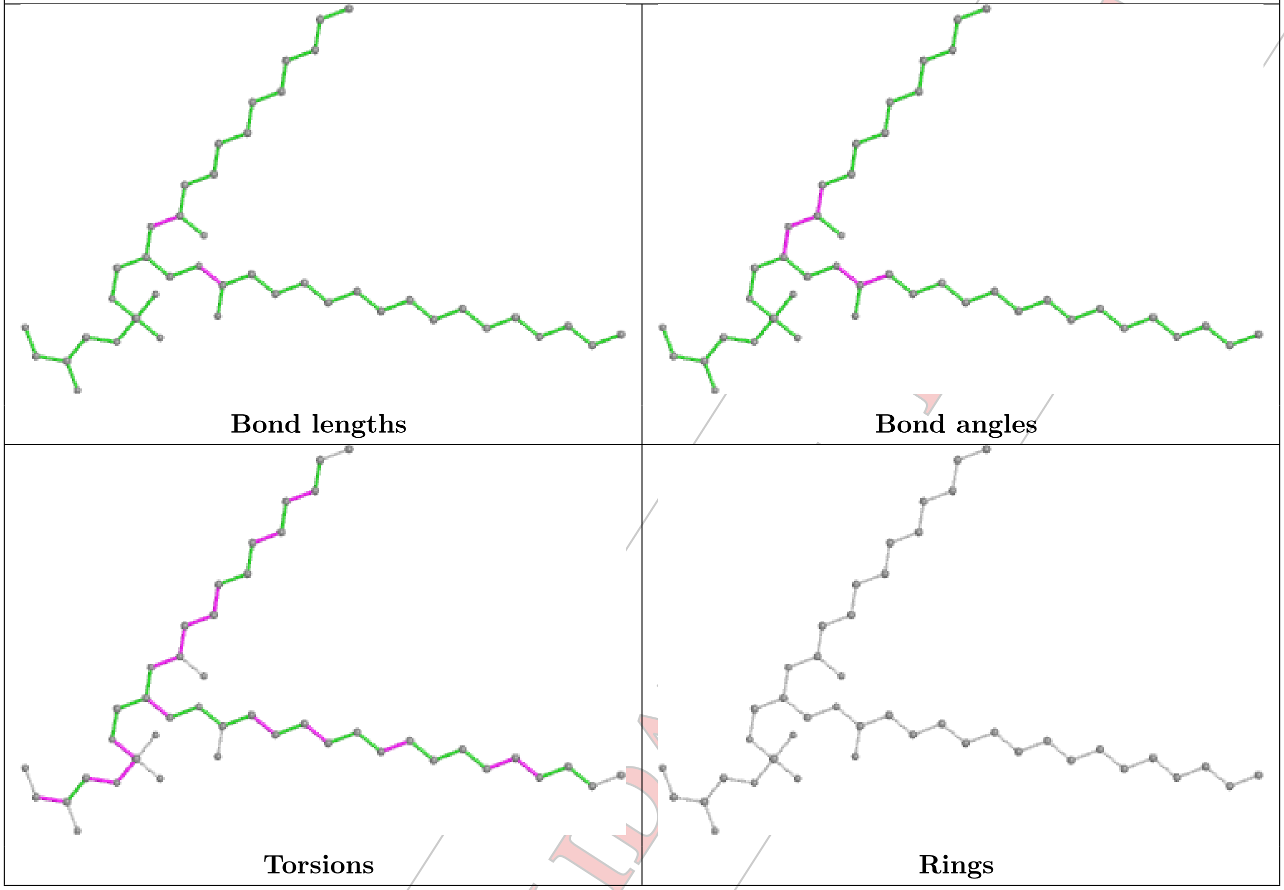

Ligand BCR IA 4018

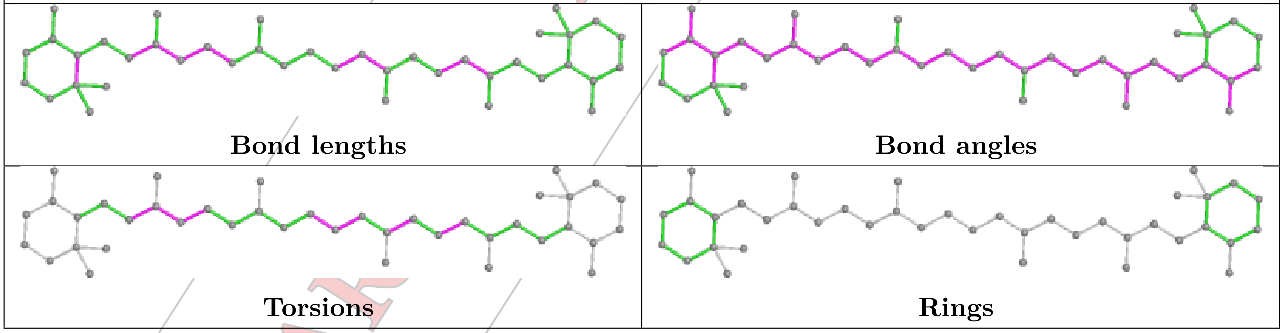

Ligand BCR IA 4020

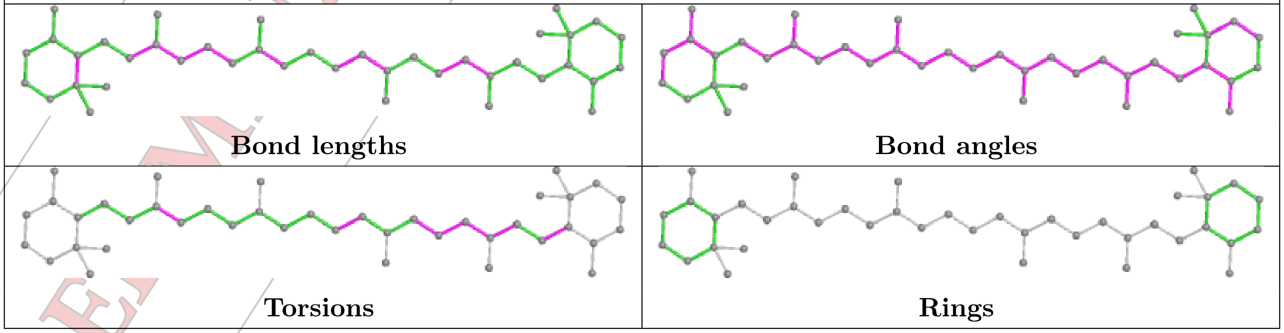

Ligand LMG IA 5006

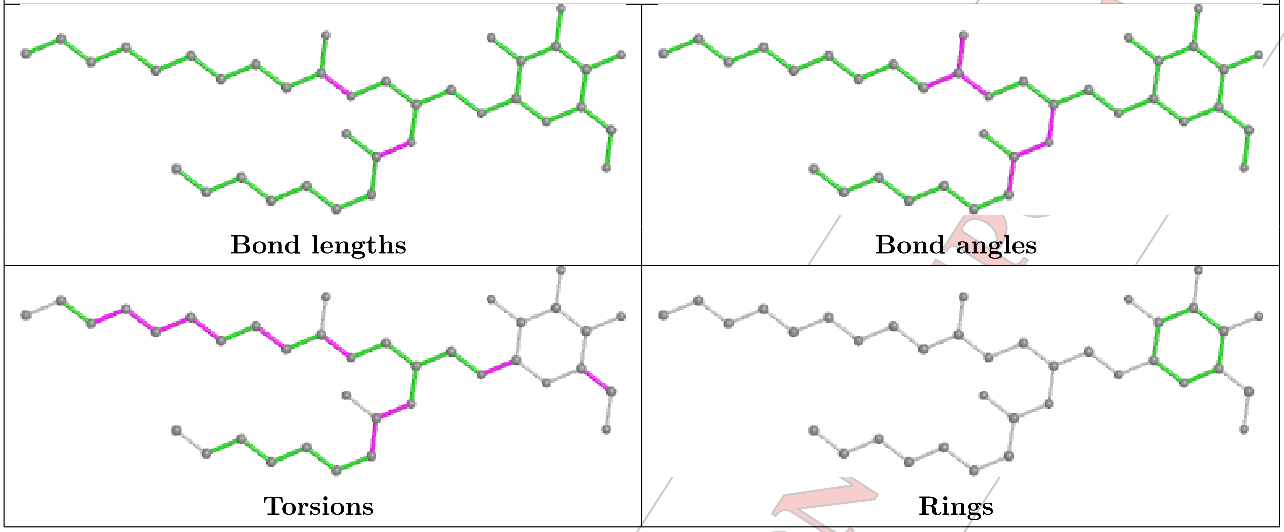

Ligand CLA KA 1401

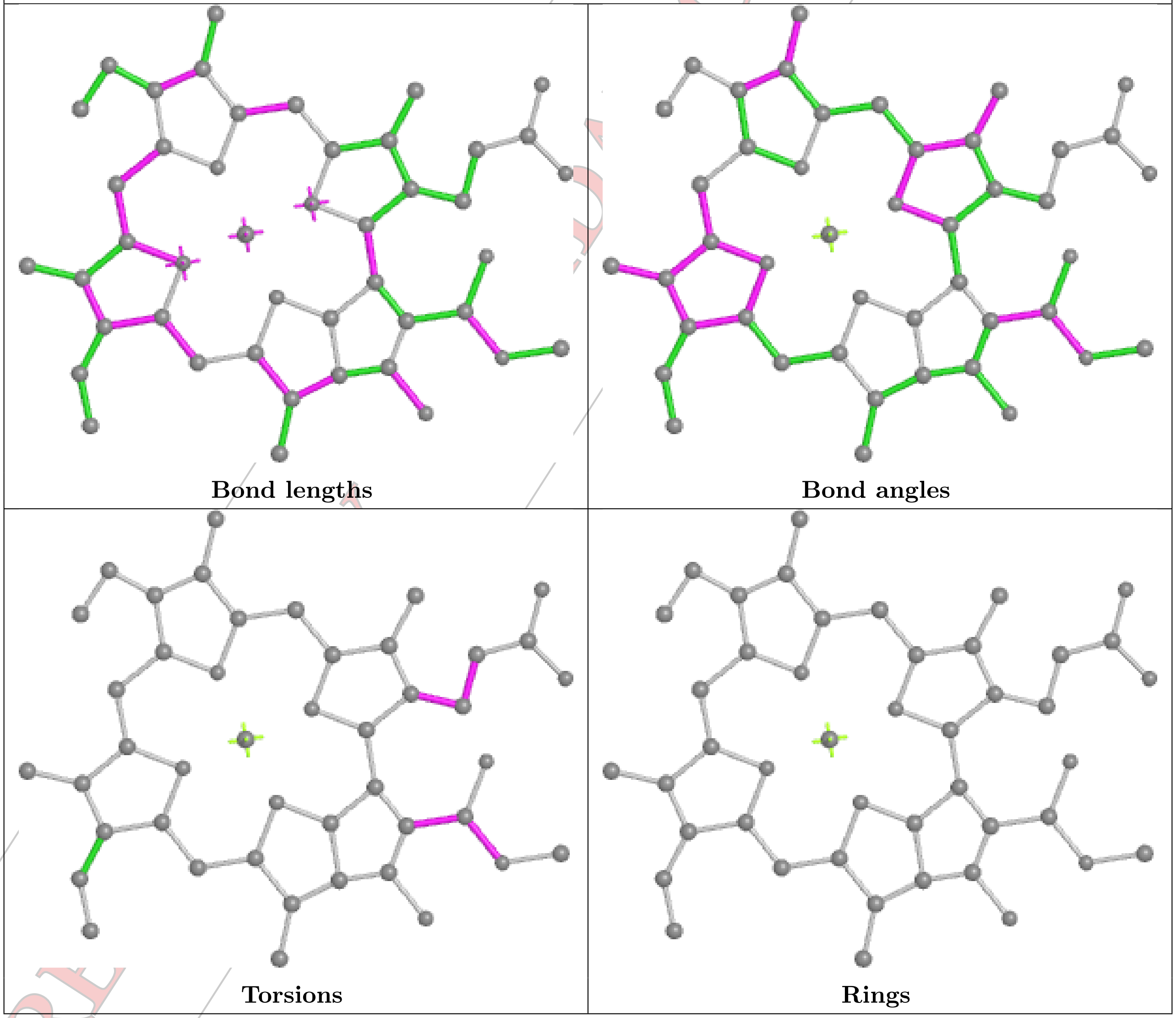

Ligand CLA LA 1501

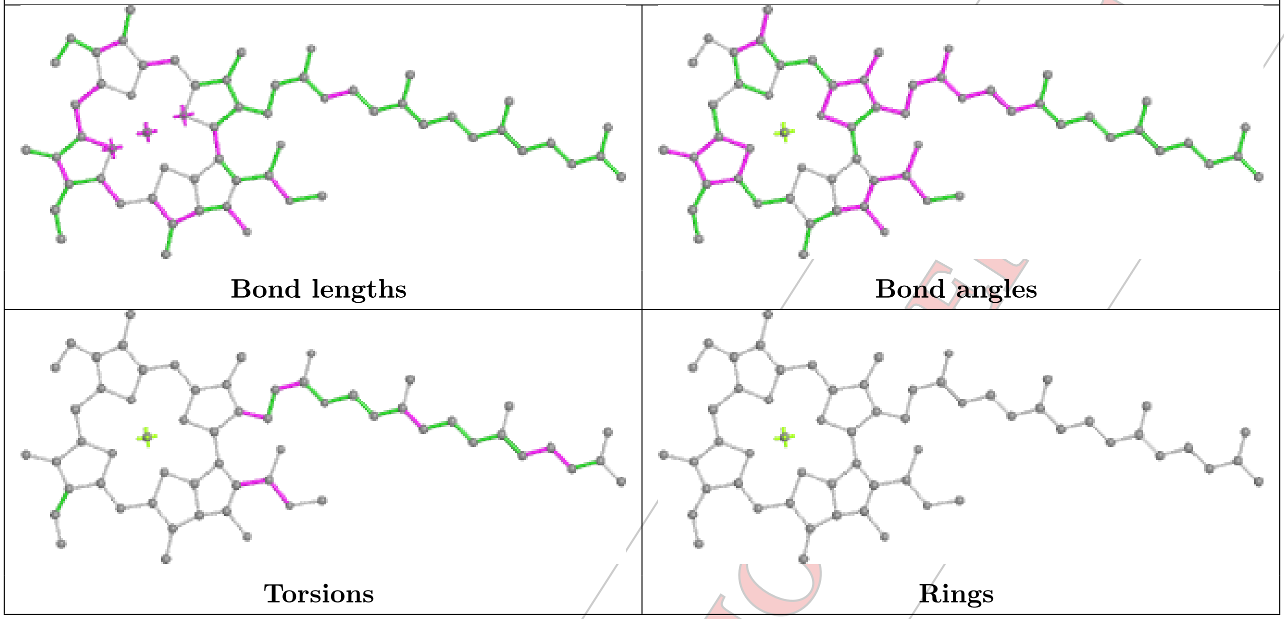

Ligand CLA LA 1502

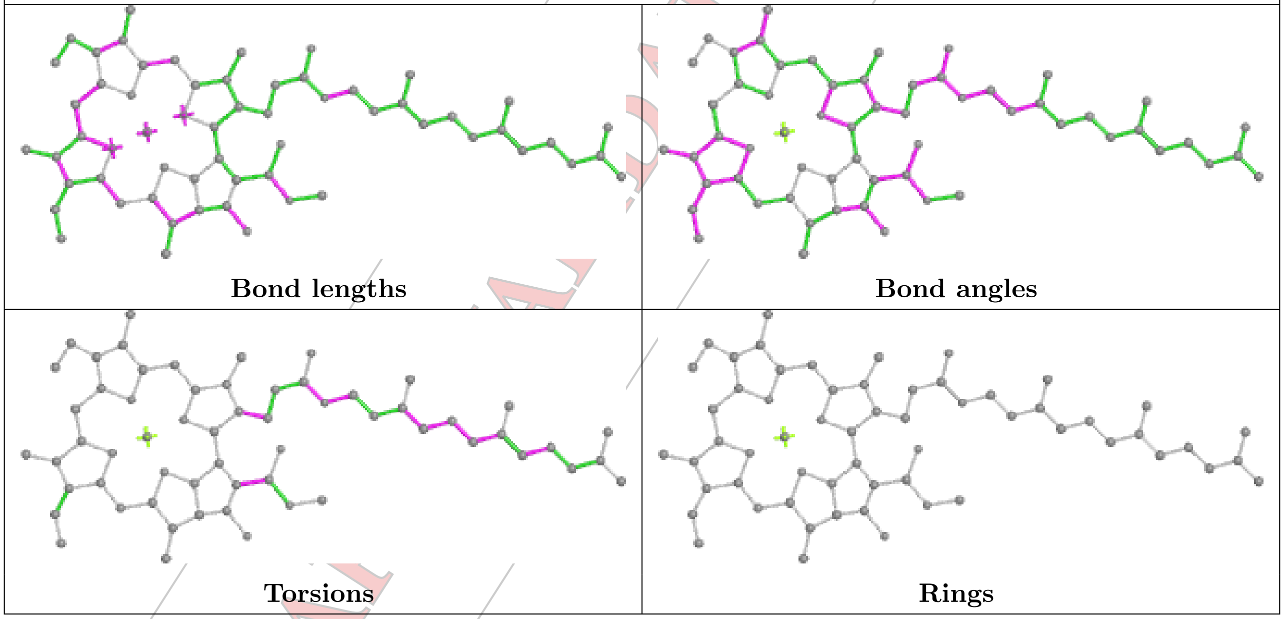

Ligand CLA LA 1503

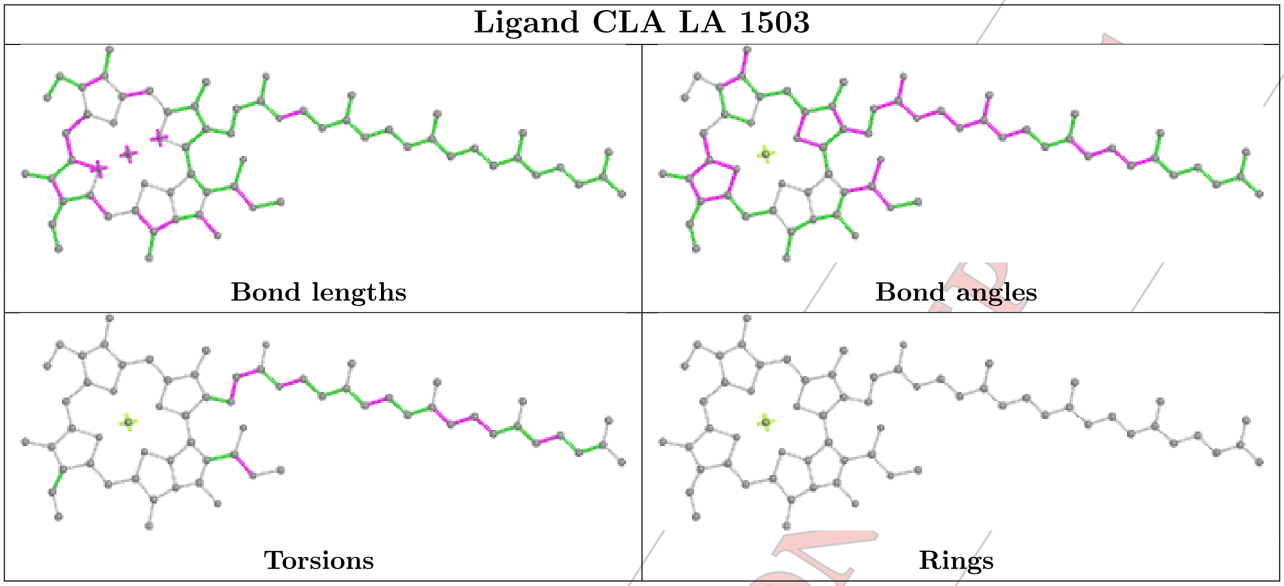

Ligand BCR LA 4019

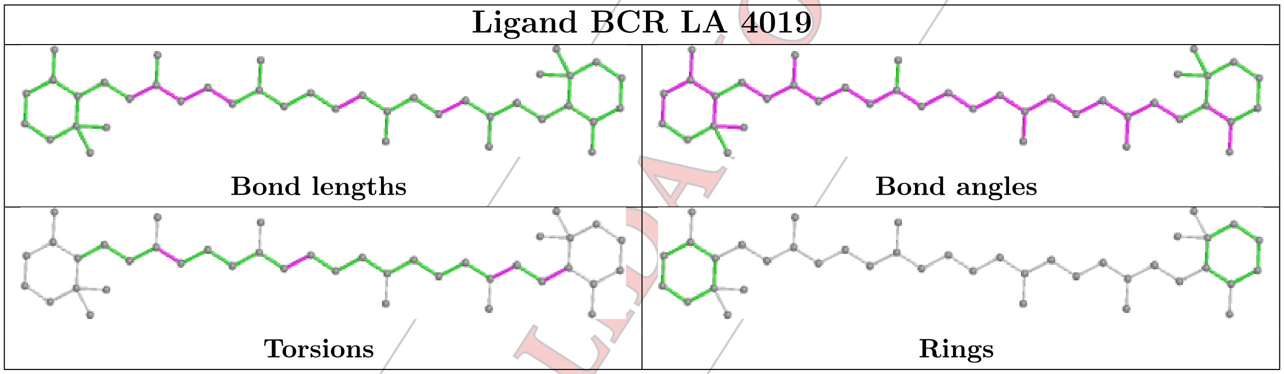

Ligand BCR LA 4022

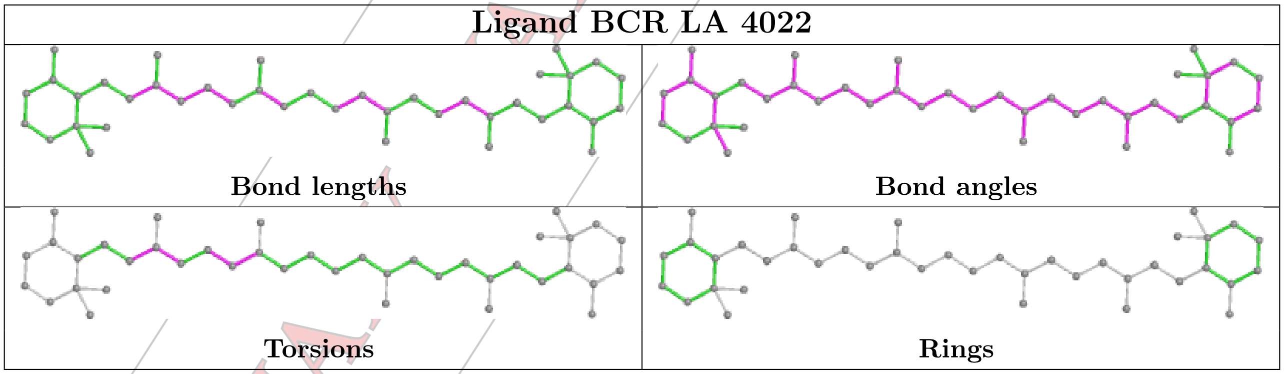

Ligand LMG LA 5007

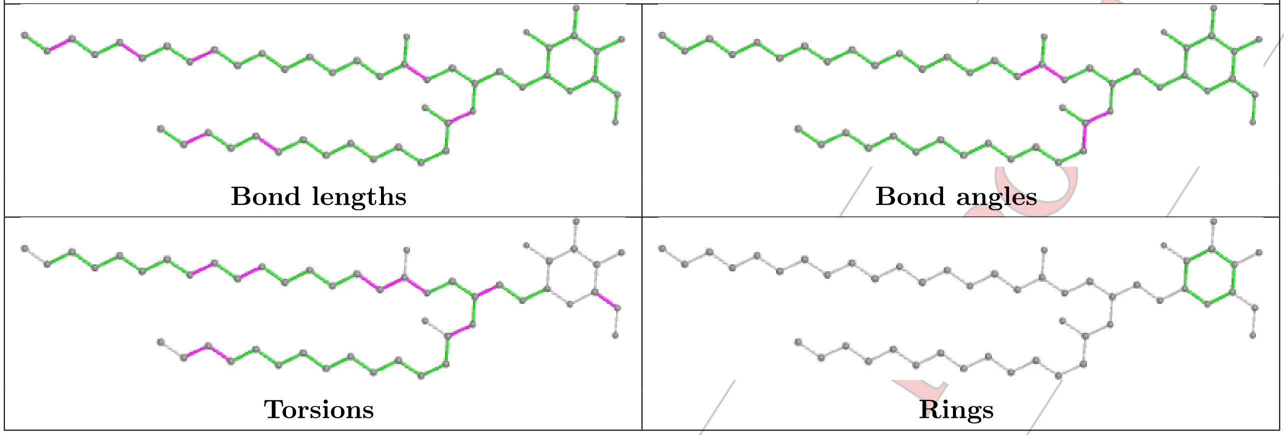

Ligand BCR MA 4021

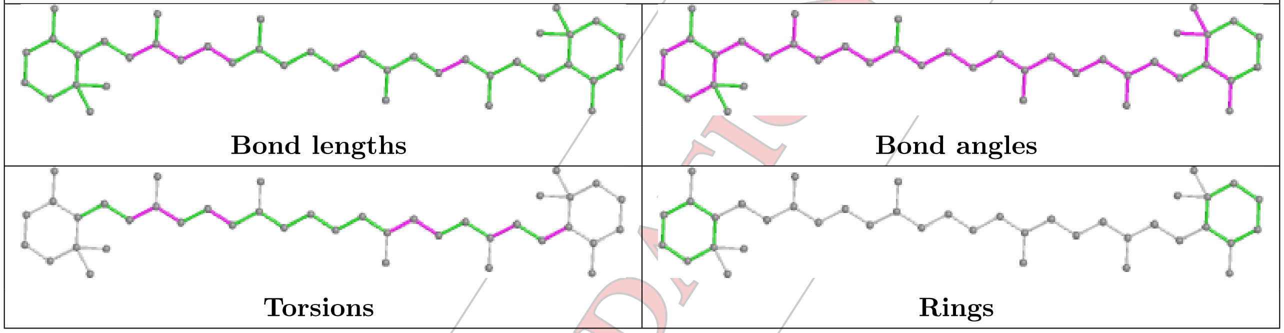

Ligand BCR RA 4018

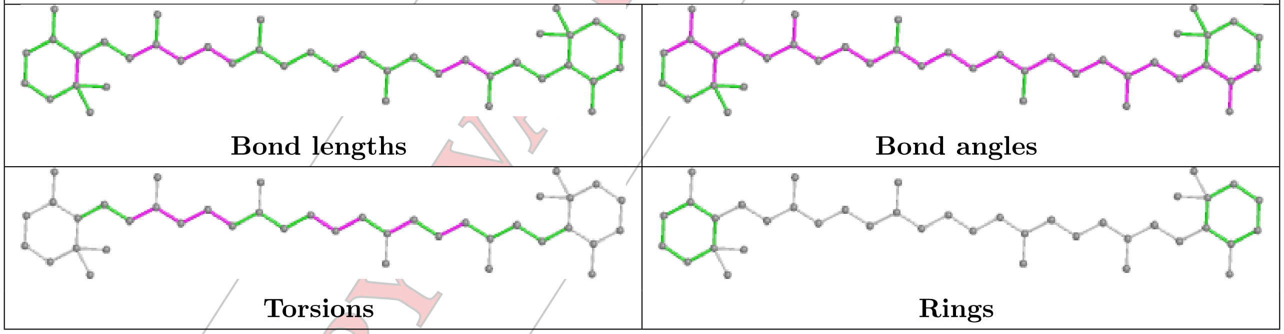

Ligand BCR RA 4020

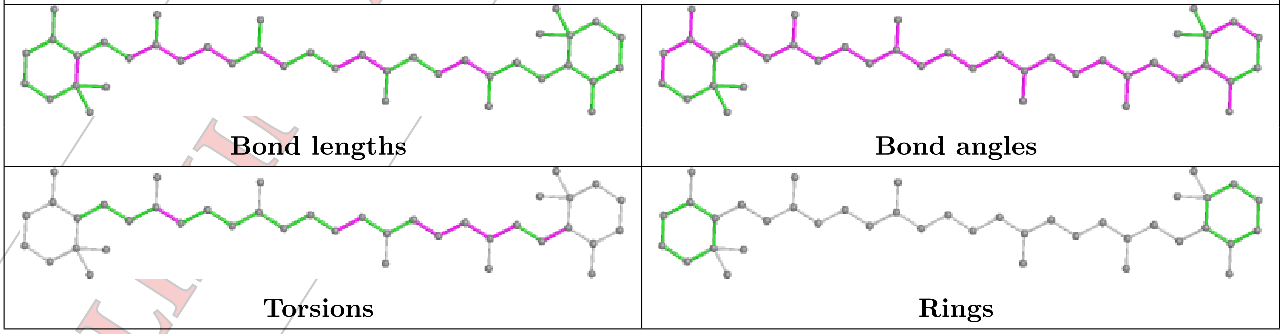

Ligand LMG RA 5006

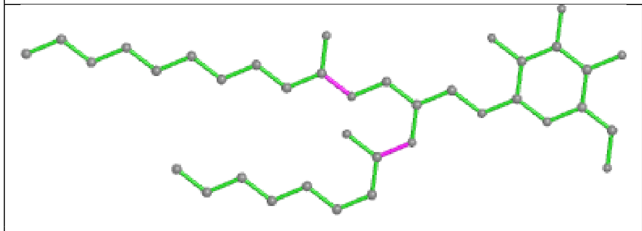
Bond lengths

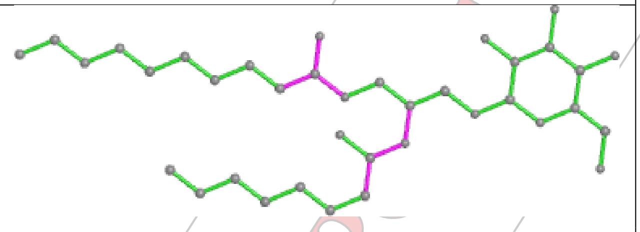
Bond angles

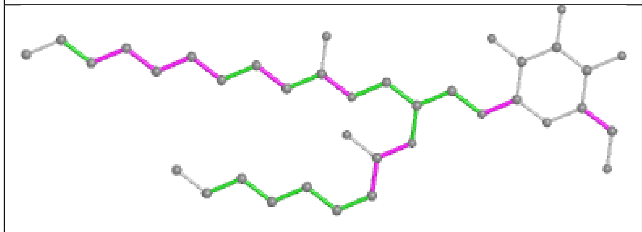
Torsions

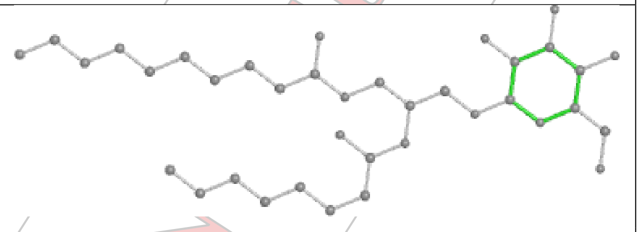
Rings

Ligand CLA TA 1401

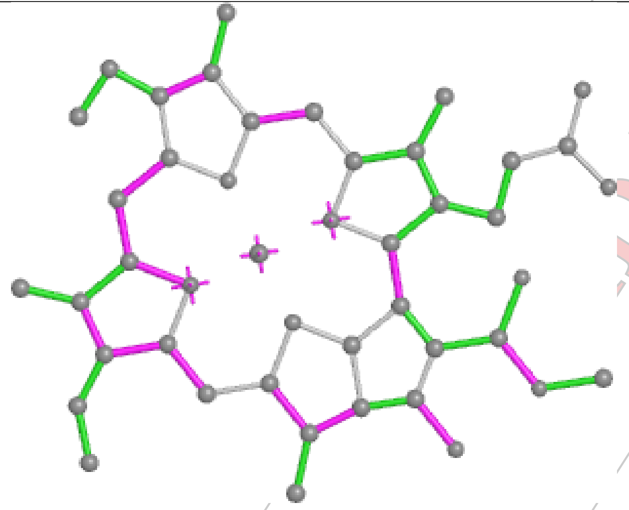
Bond lengths

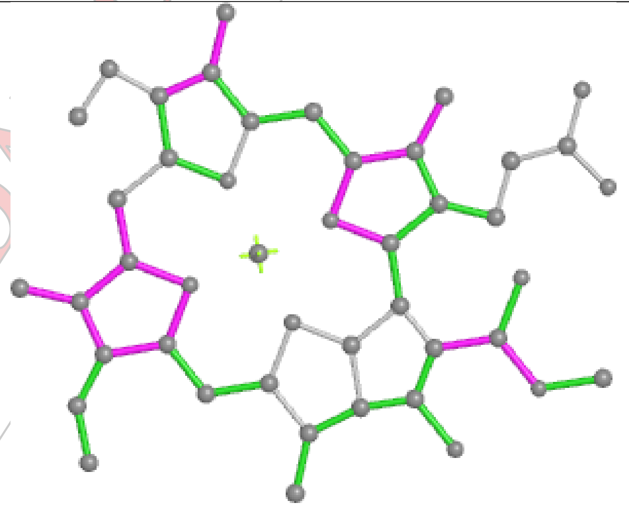
Bond angles

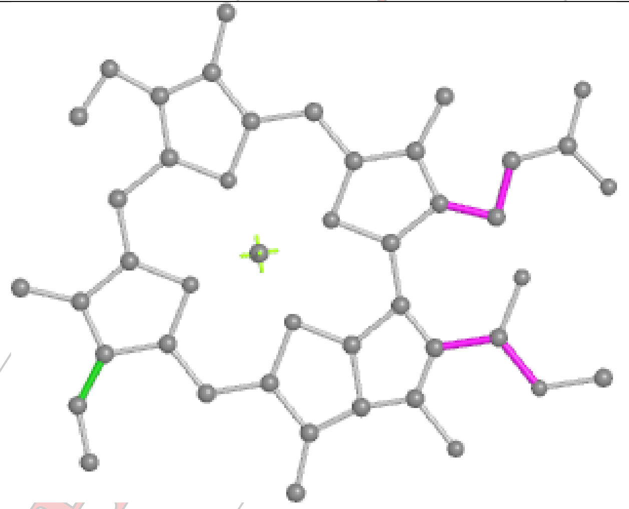
Torsions

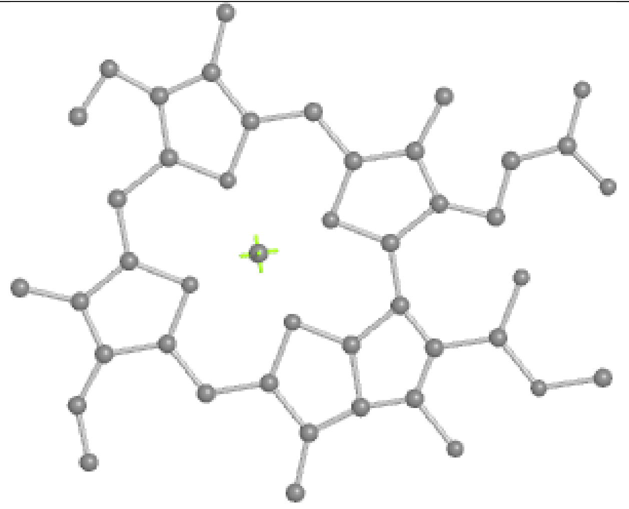
Rings

Ligand CLA UA 1501

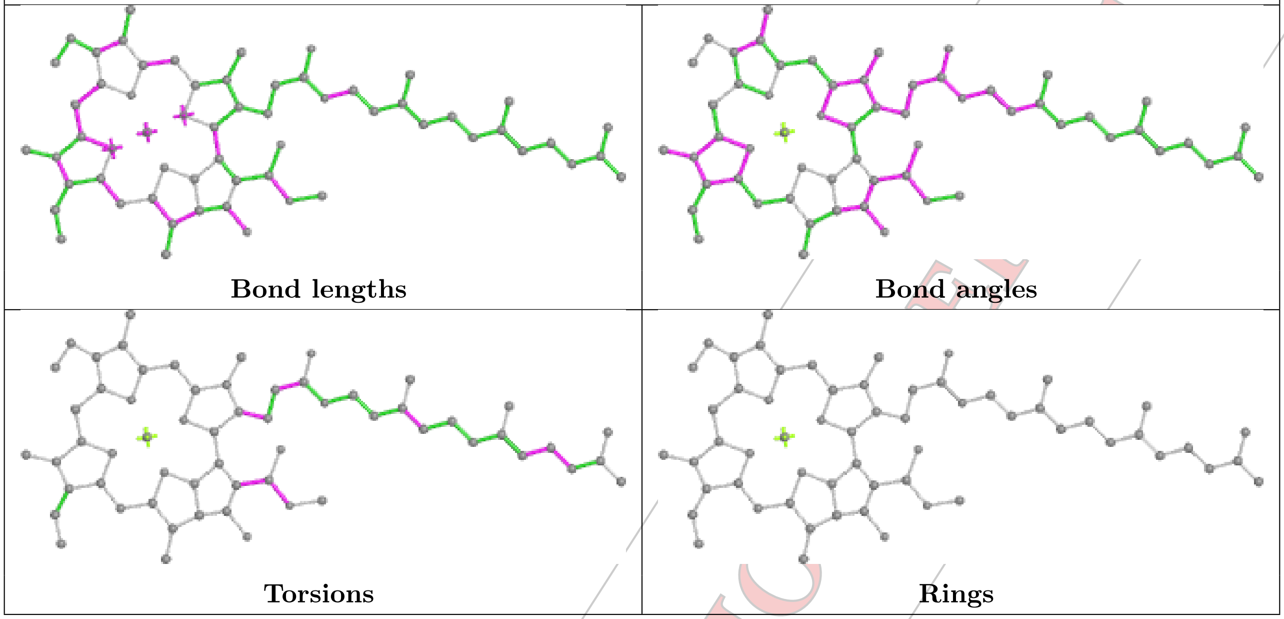

Ligand CLA UA 1502

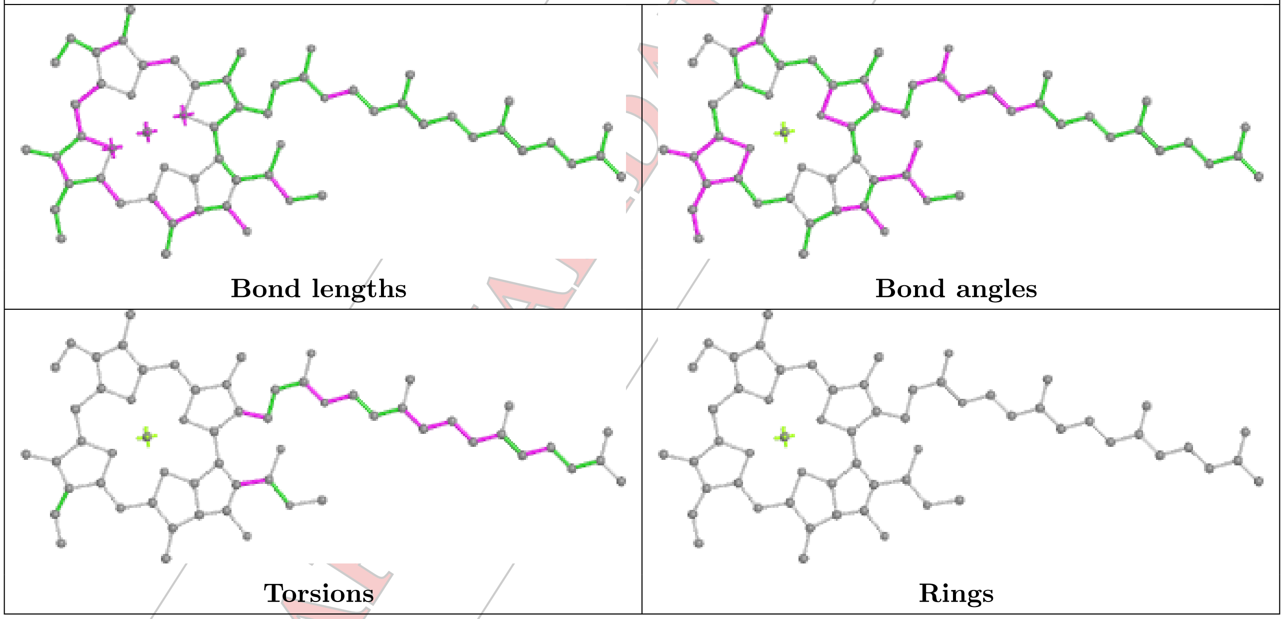

PRELIMINARY OFFICIAL

Ligand CLA UA 1503

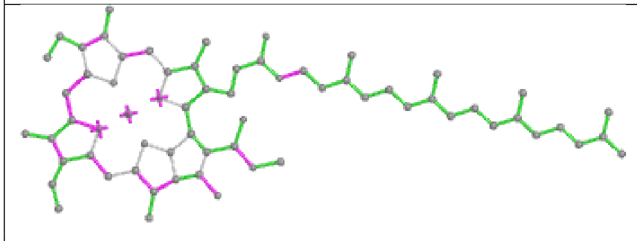

Bond lengths

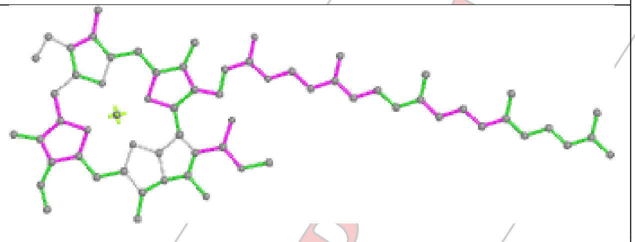

Bond angles

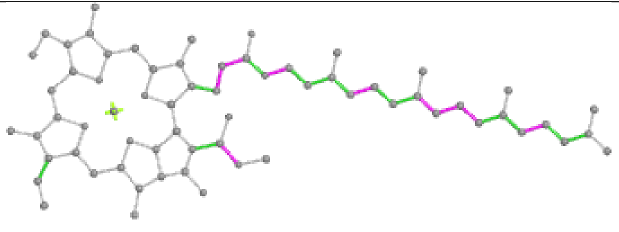

Torsions

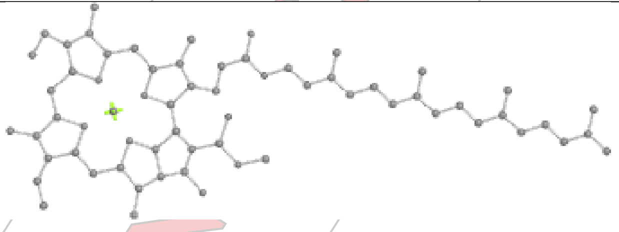

Rings

Ligand BCR UA 4019

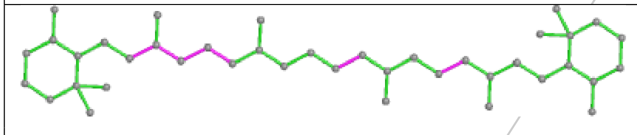

Bond lengths

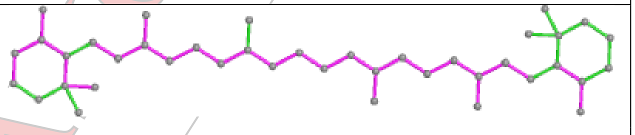

Bond angles

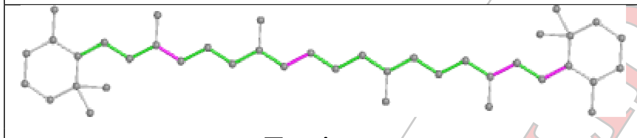

Torsions

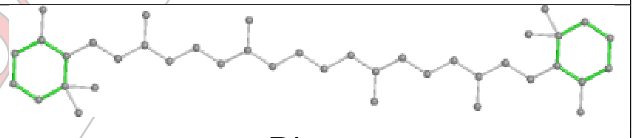

Rings

Ligand BCR UA 4022

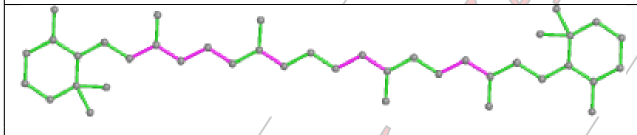

Bond lengths

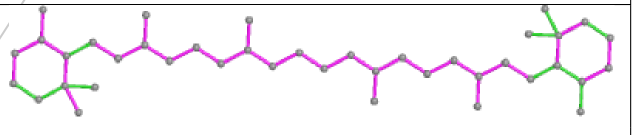

Bond angles

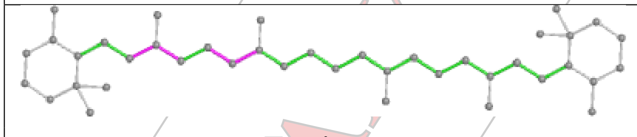

Torsions

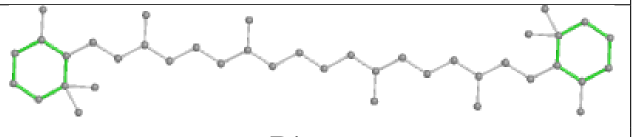

Rings

Ligand LMG UA 5007

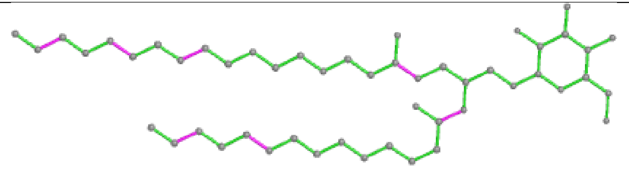

Bond lengths

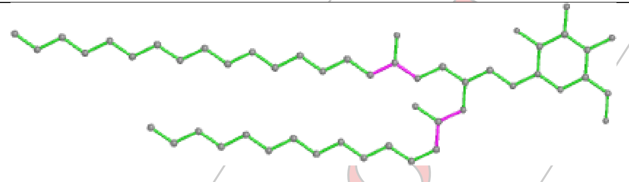

Bond angles

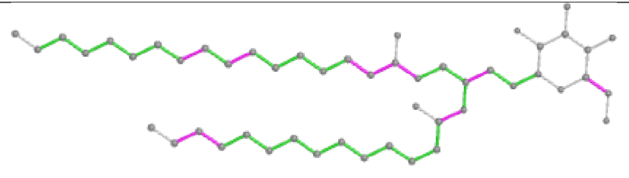

Torsions

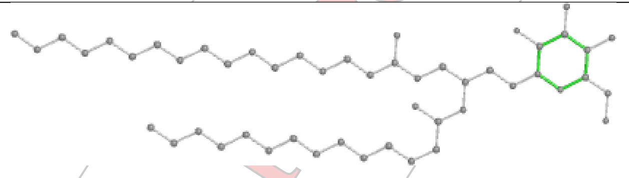

Rings

Ligand BCR VA 4021

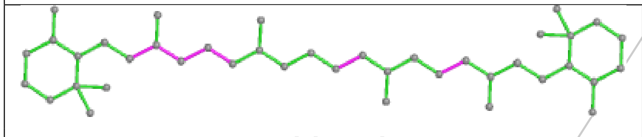

Bond lengths

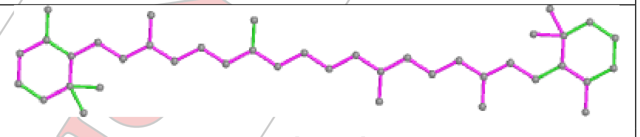

Bond angles

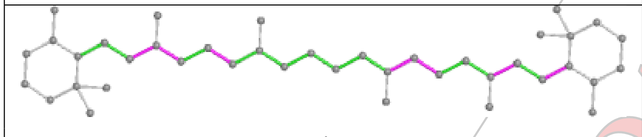

Torsions

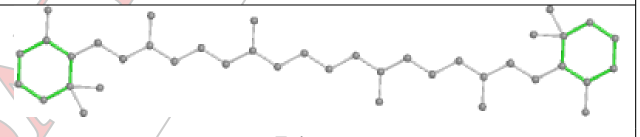

Rings

PRELIMINARY VALID

Ligand CLA WA 1701

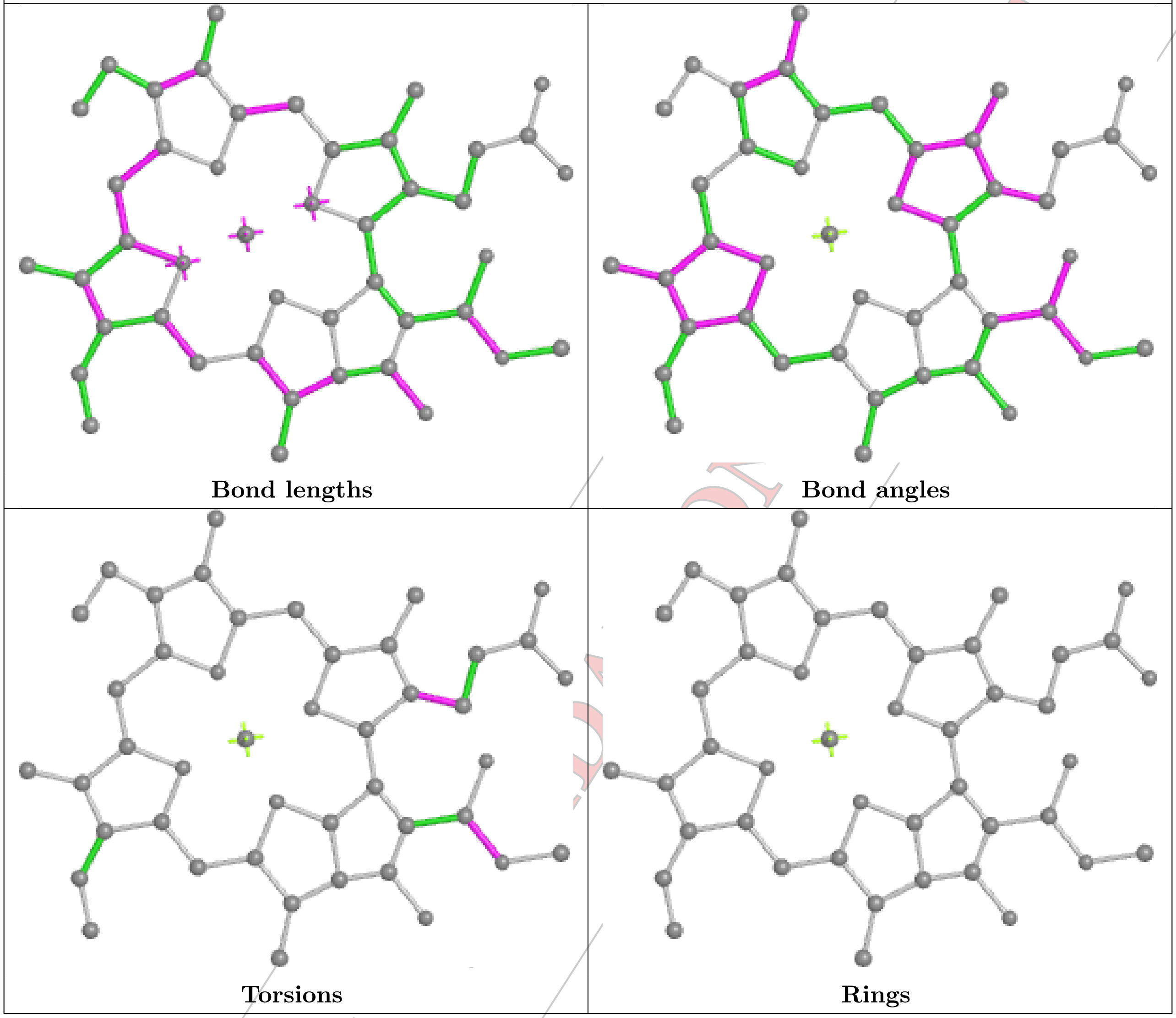

PRELIMINARY

Ligand CLA XA 1701

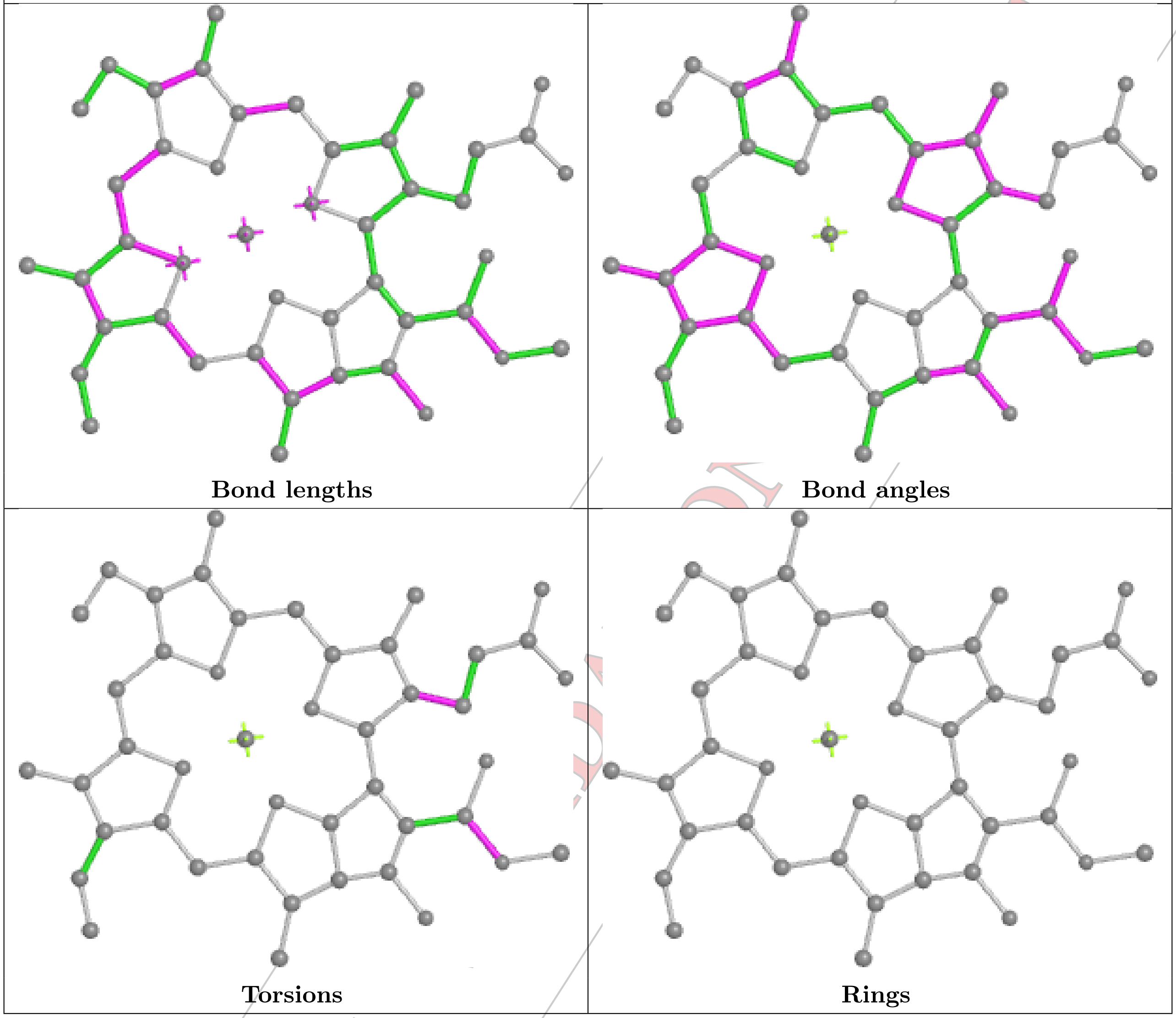

Ligand CL0 aA 1011

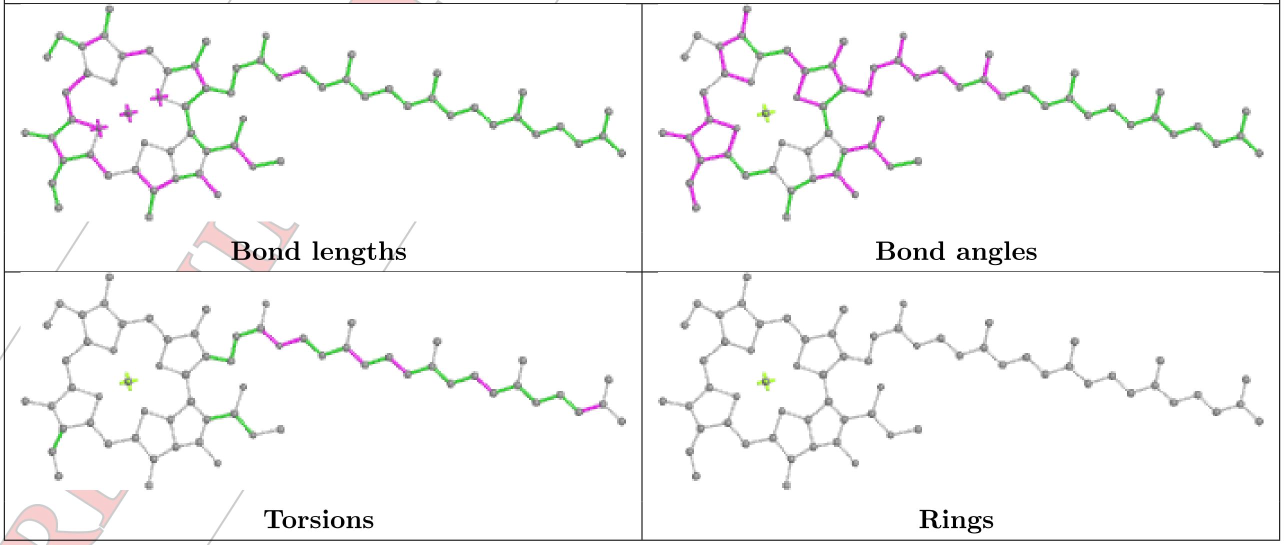

Ligand CLA aA 1012

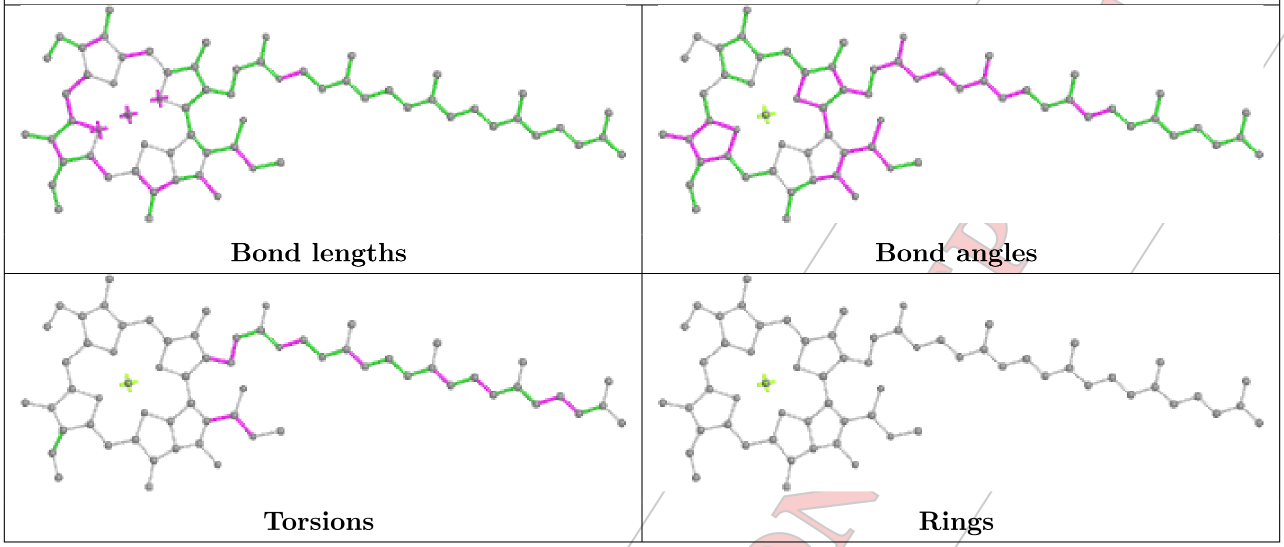

Ligand CLA aA 1013

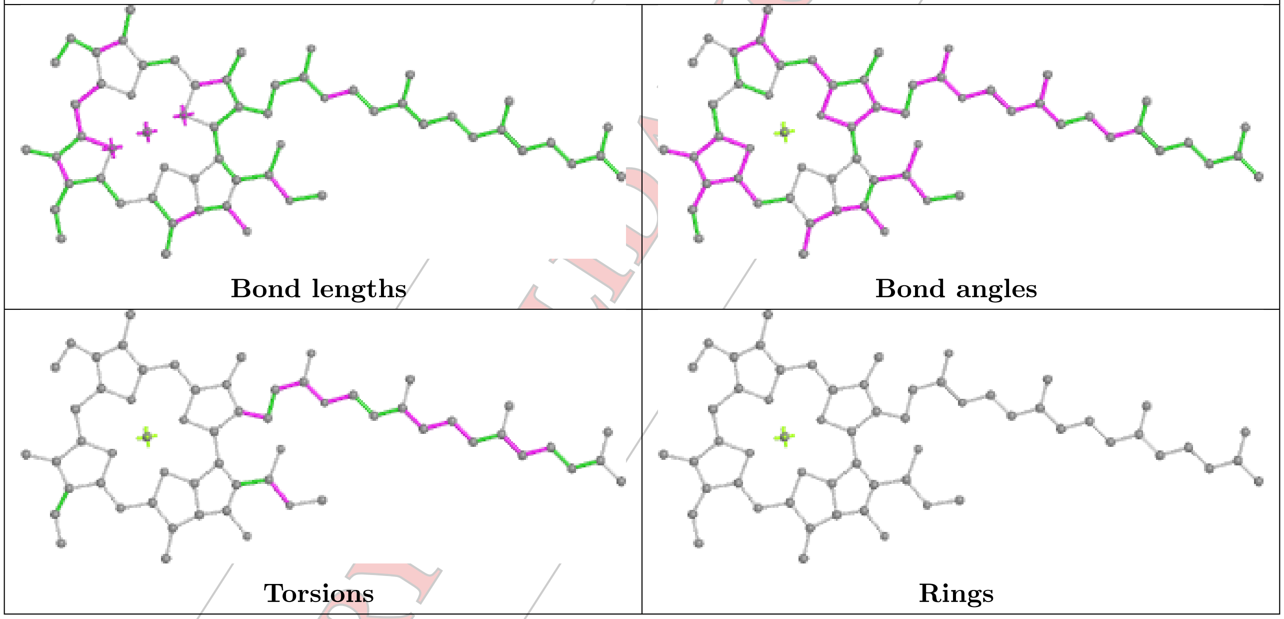

PRELIMINARY

Ligand CLA aA 1101

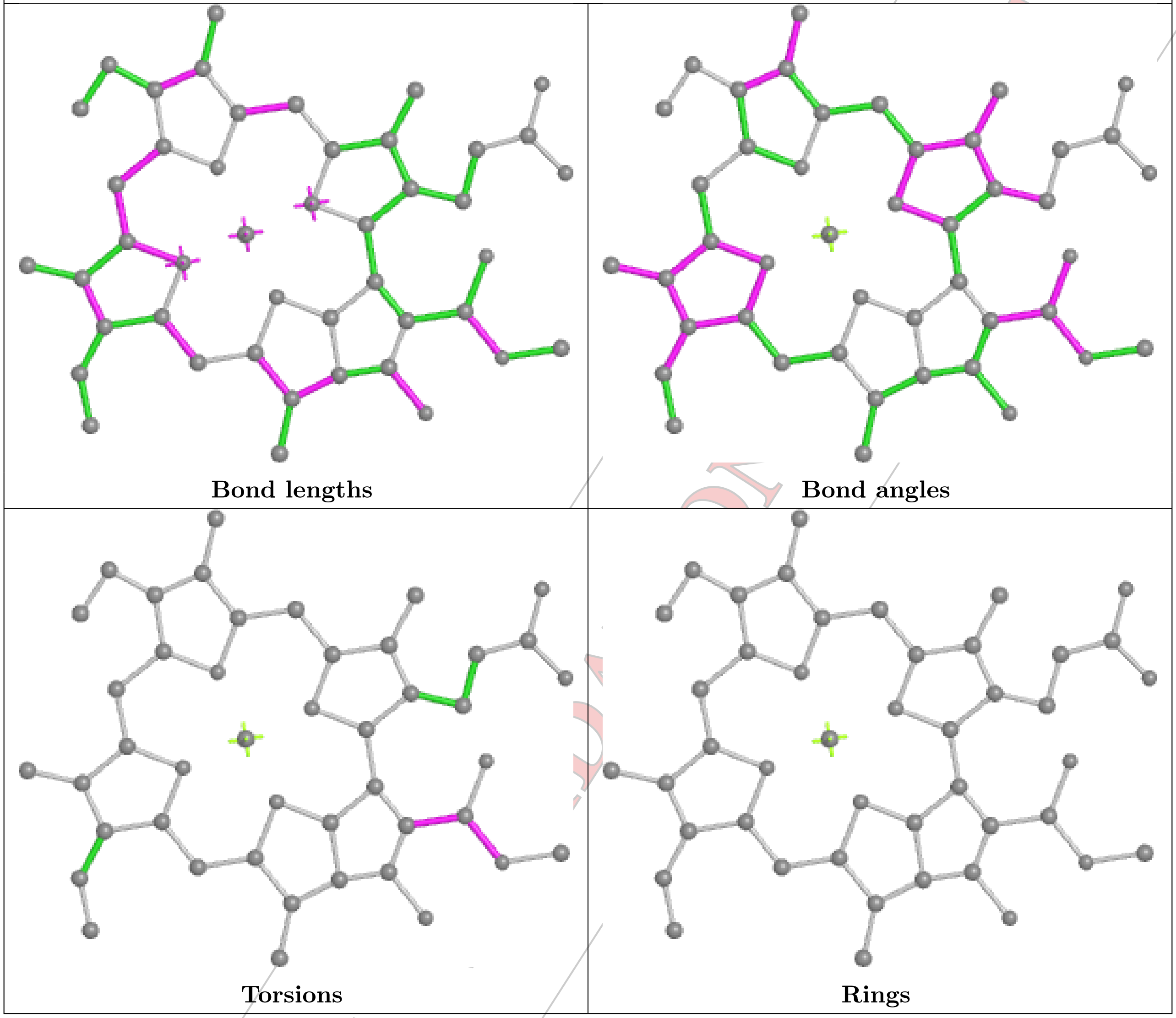

Ligand CLA aA 1102

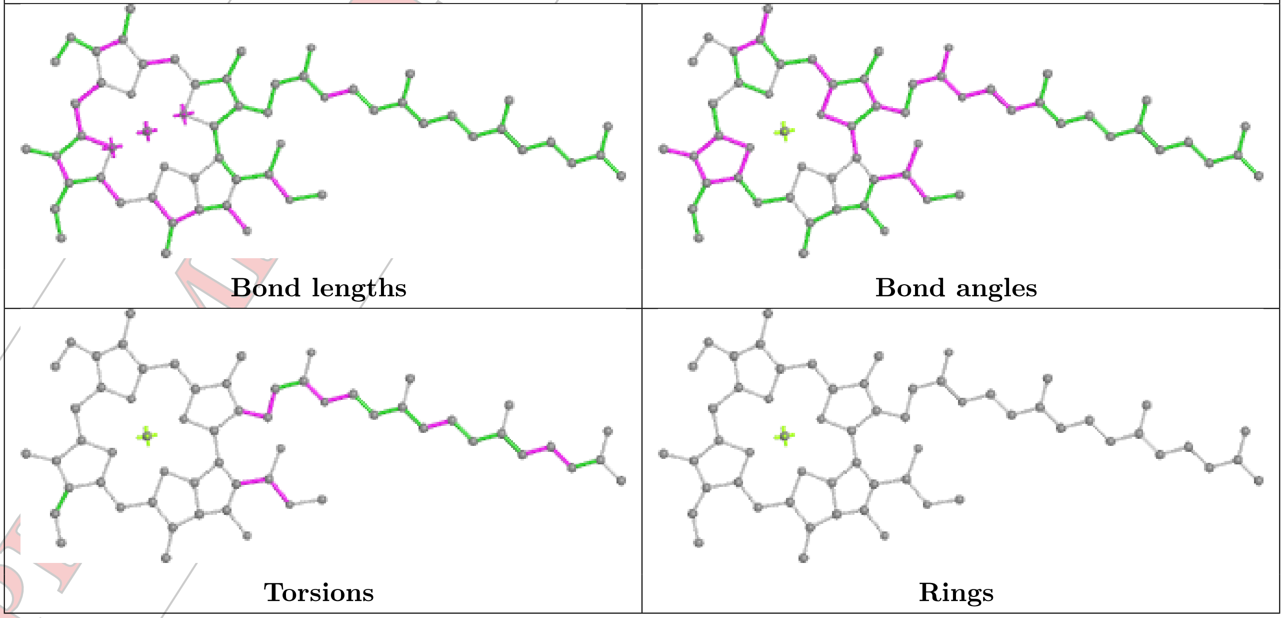

Ligand CLA aA 1103

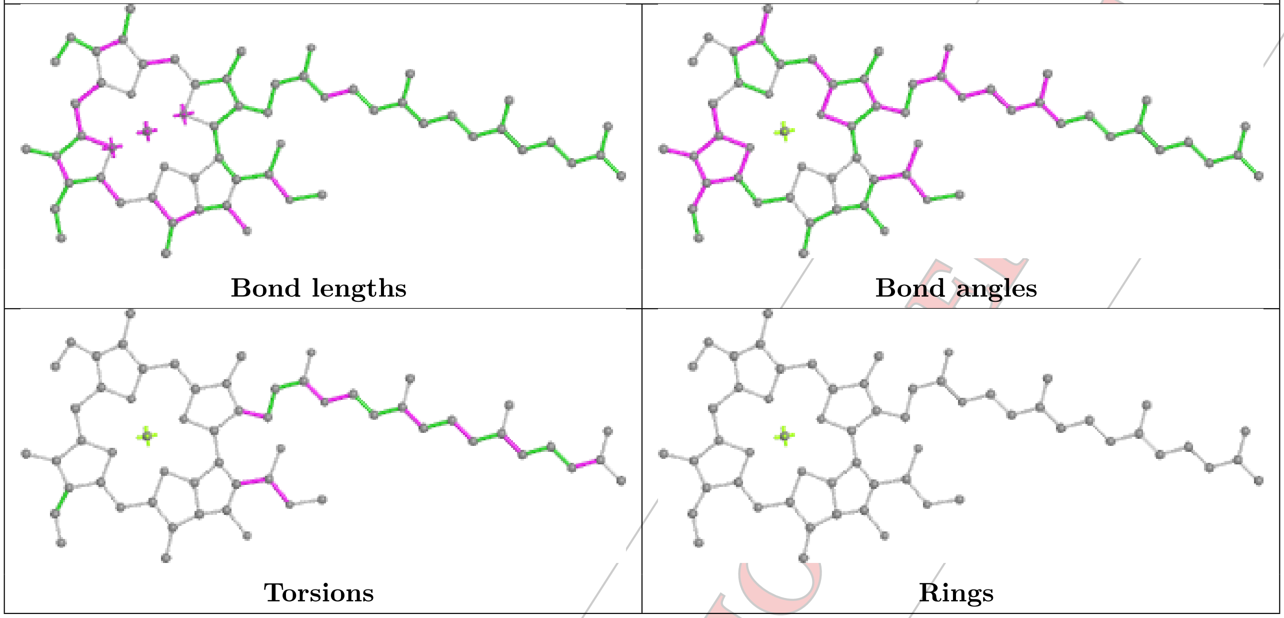

Ligand CLA aA 1104

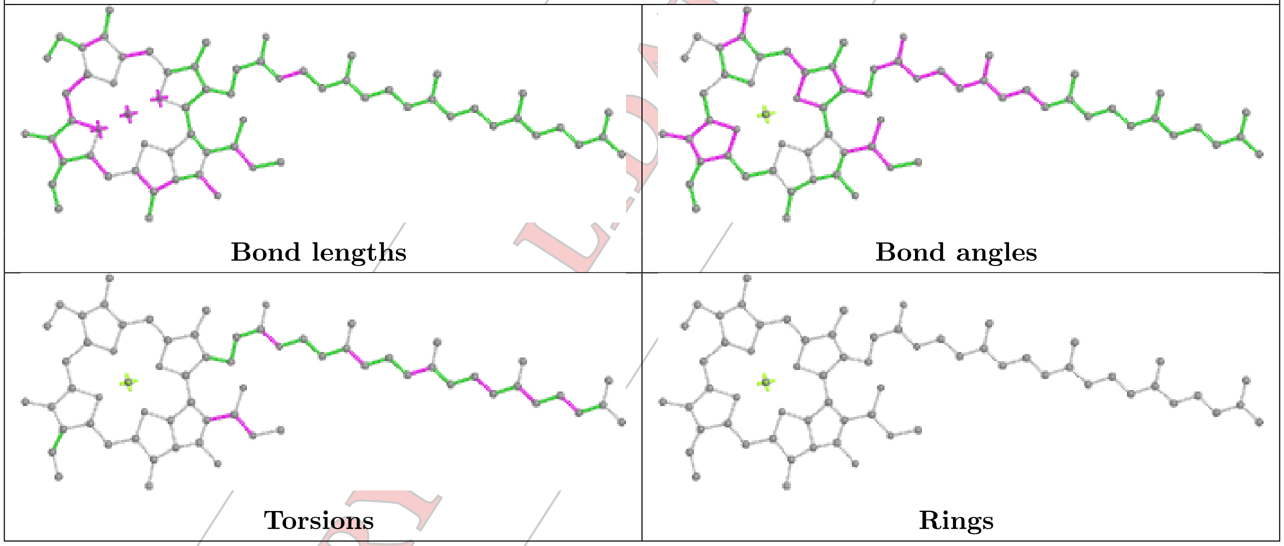

PRELIMINARY

Ligand CLA aA 1105

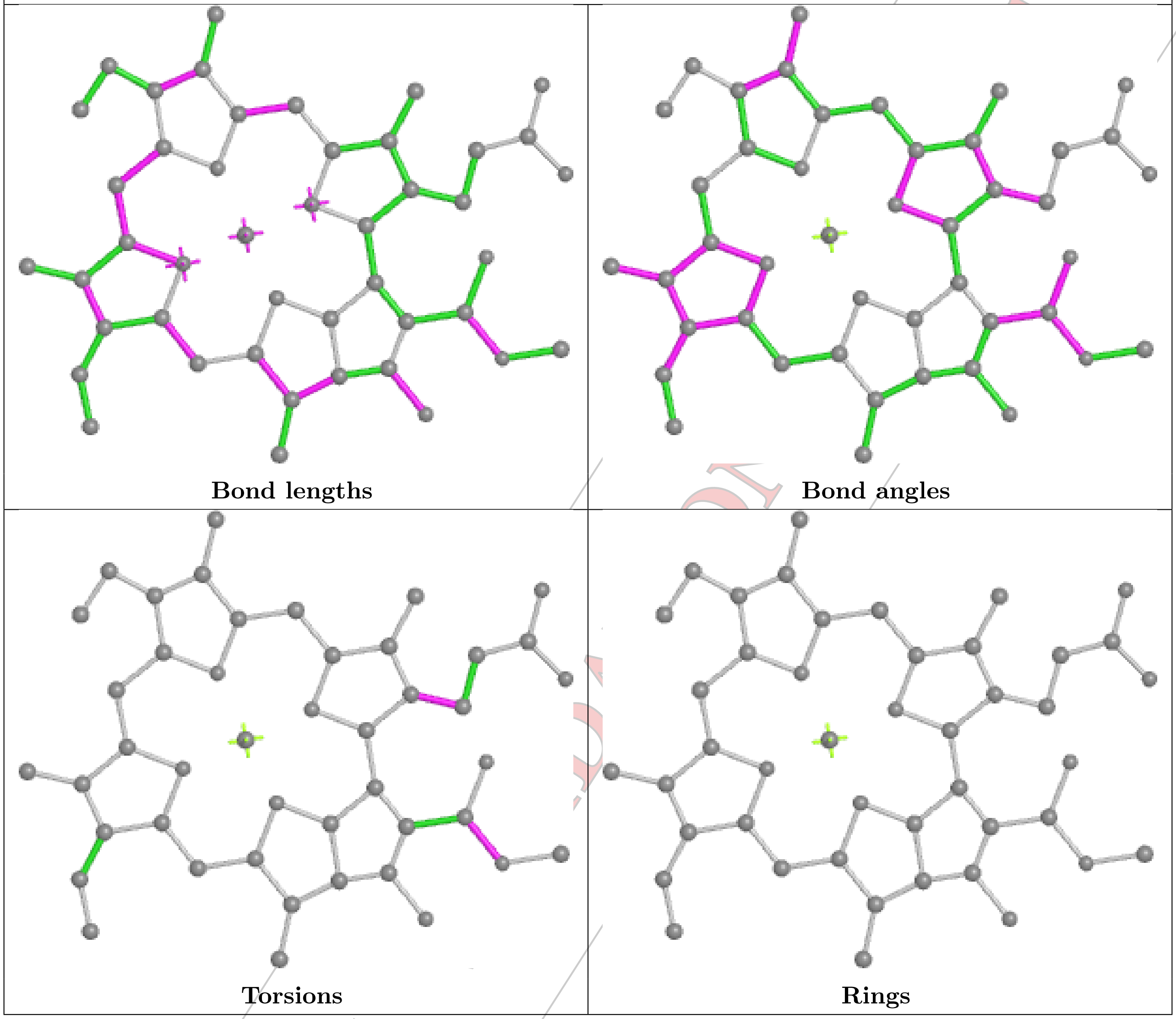

PRELIMINARY

Ligand CLA aA 1106

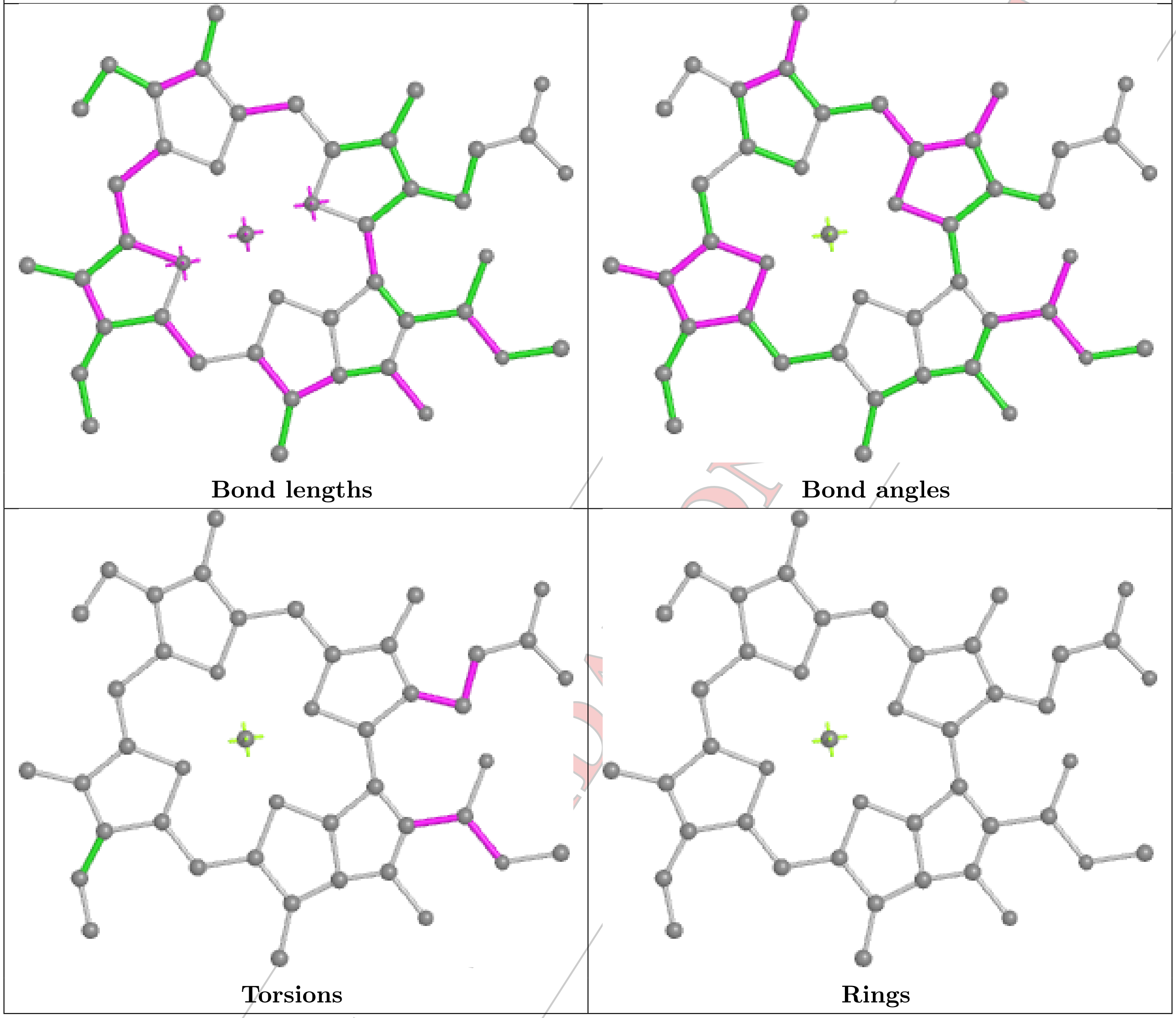

PRELIMINARY

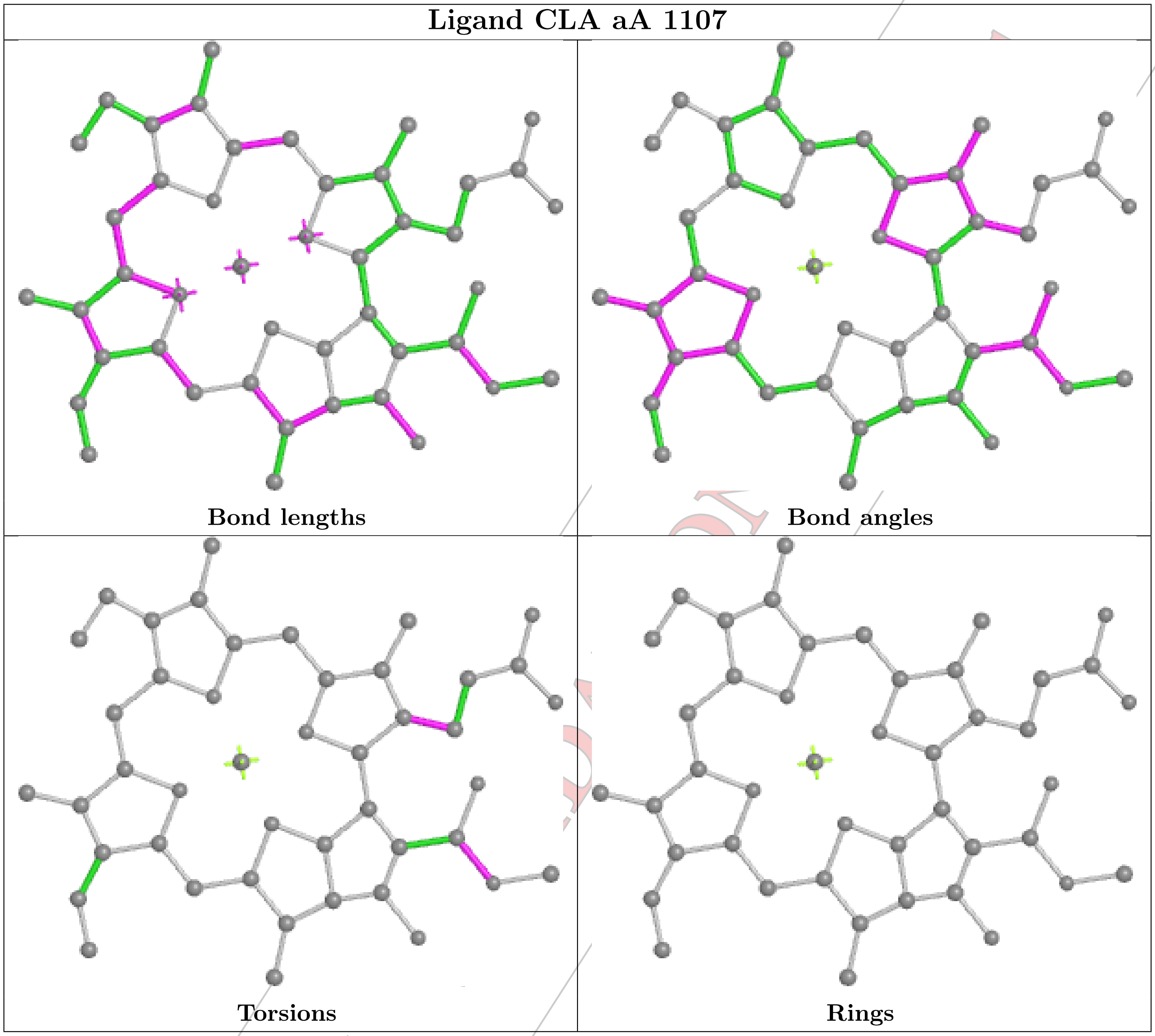

PRELIMINARY

Ligand CLA aA 1108

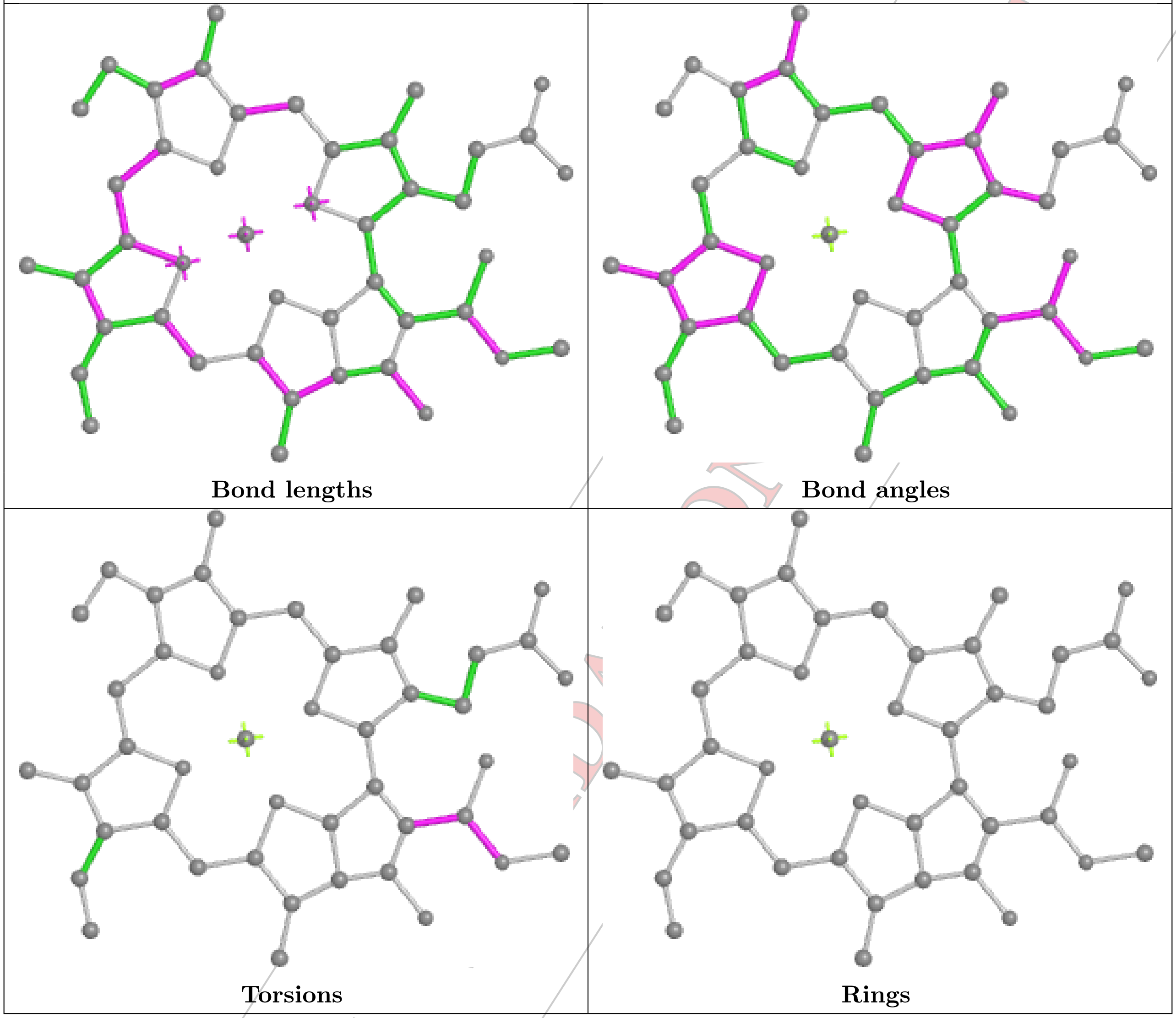

PRELIMINARY

Ligand CLA aA 1109

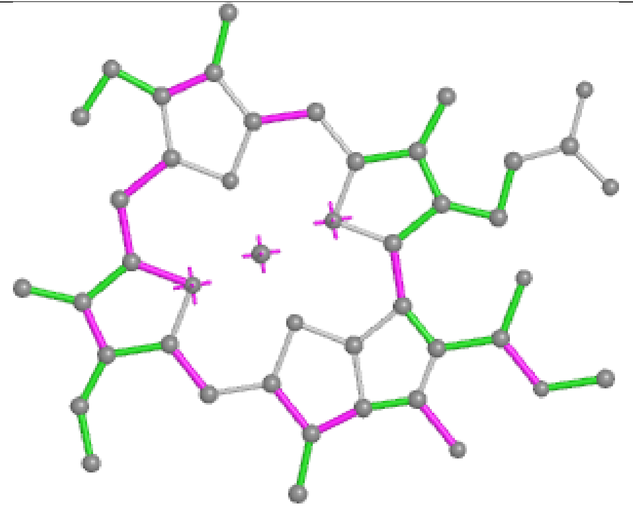

Bond lengths

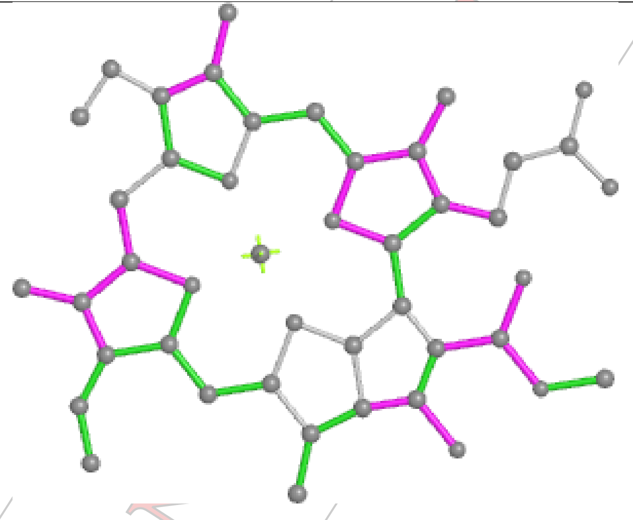

Bond angles

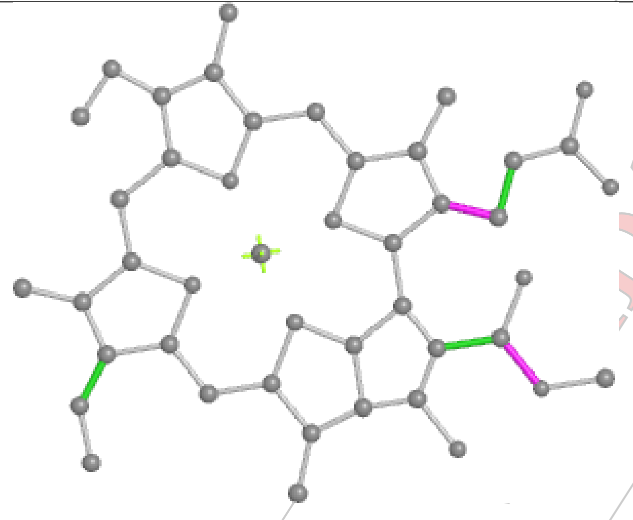

Torsions

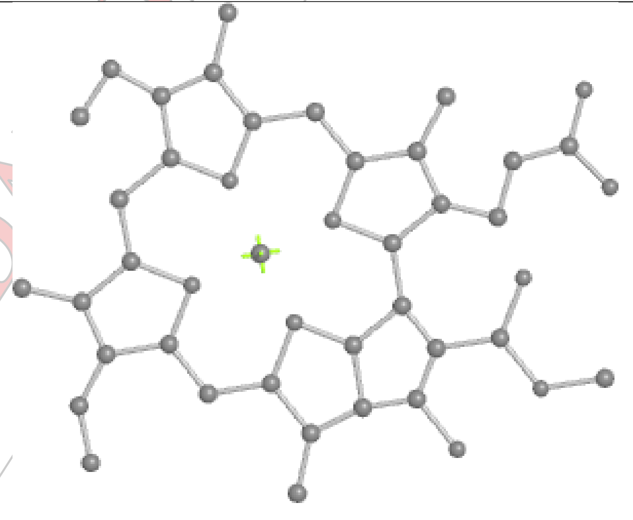

Rings

PRELIMINARY

Ligand CLA aA 1110

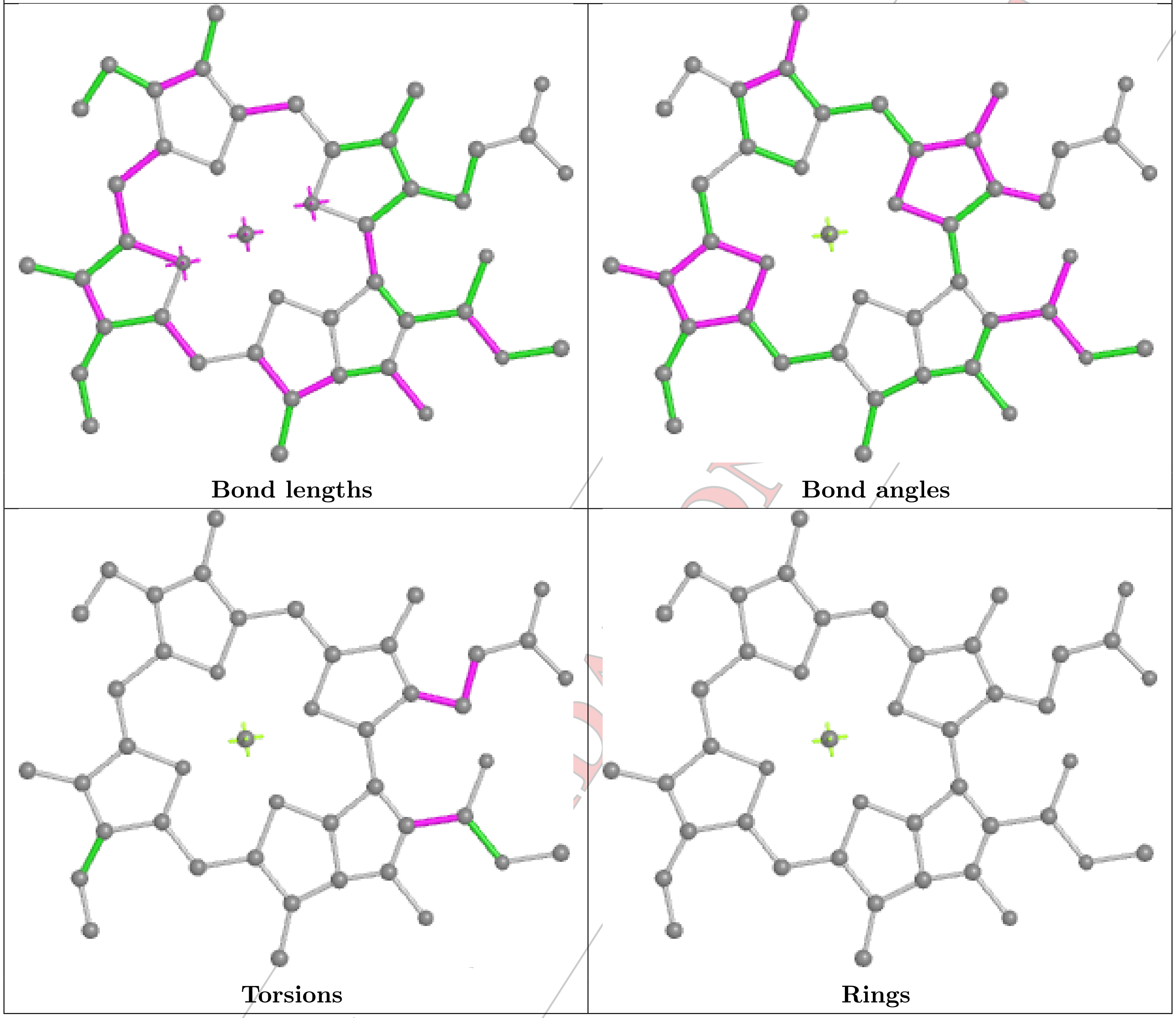

Ligand CLA aA 1111

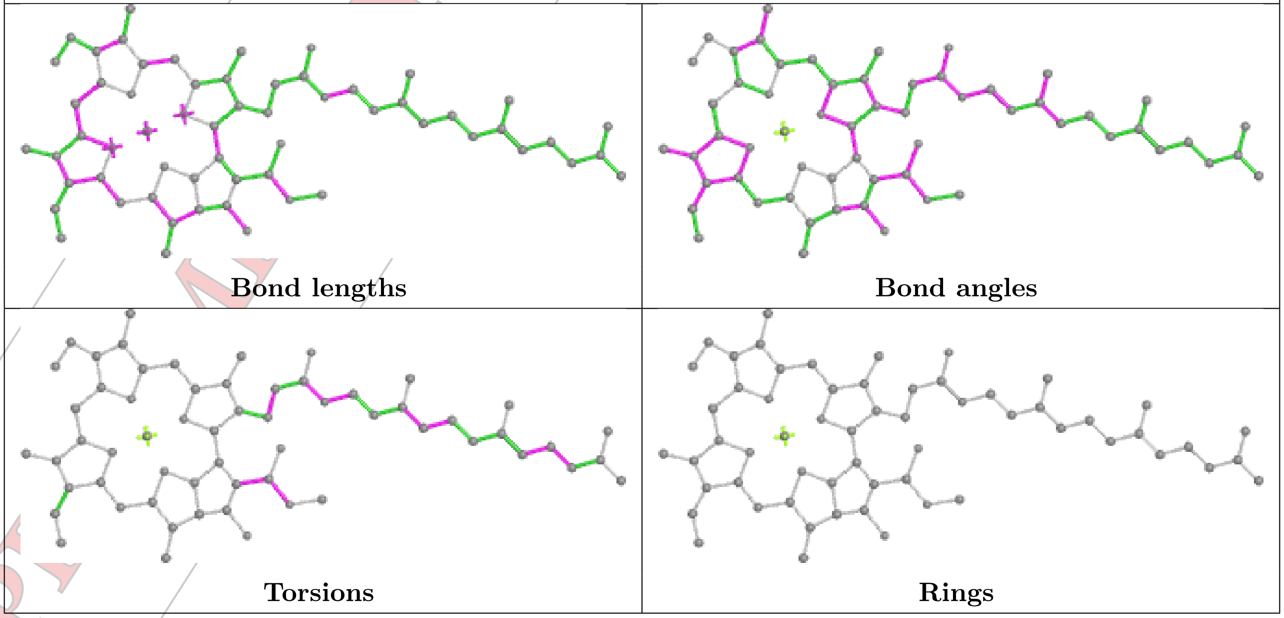

Ligand CLA aA 1112

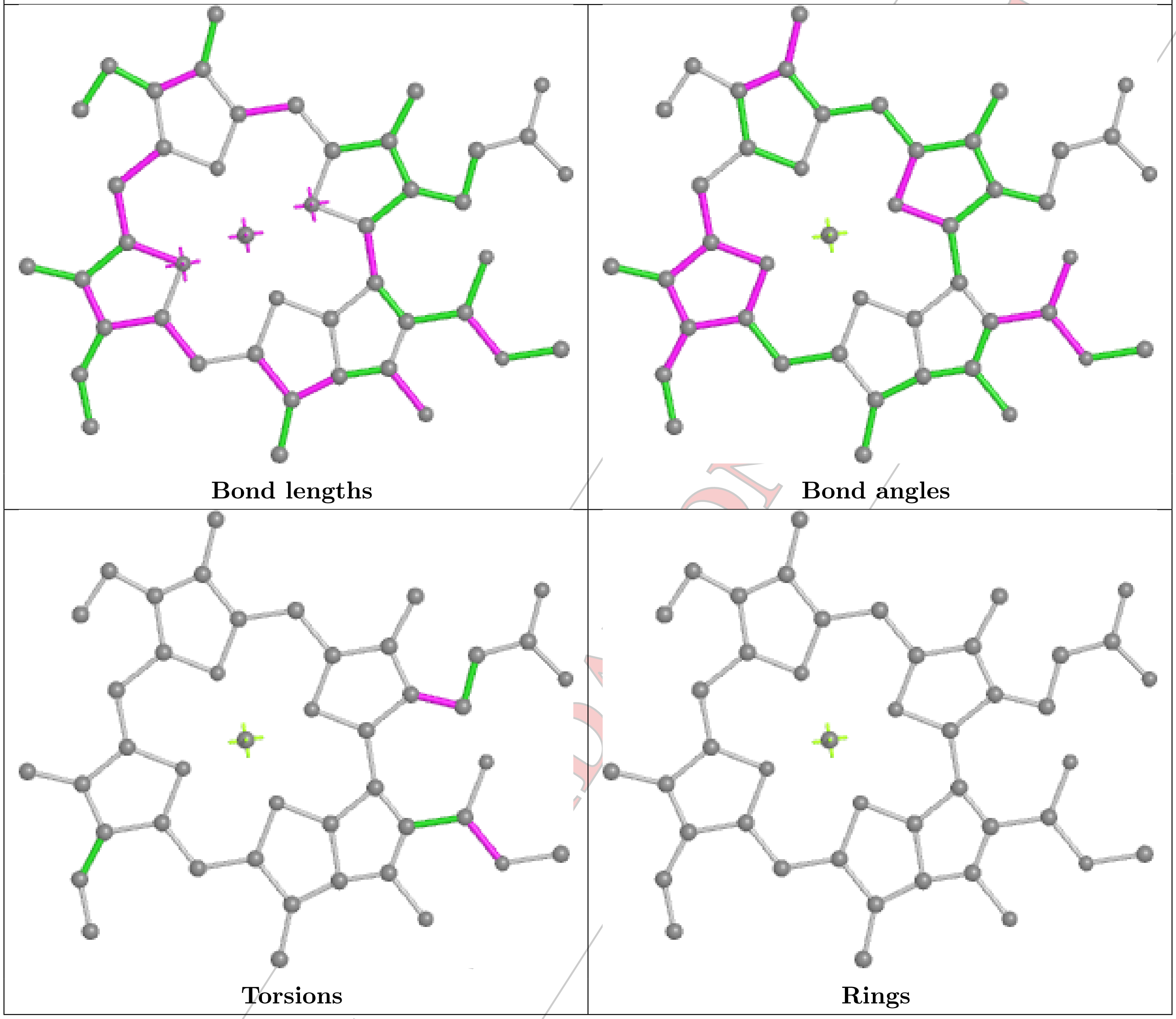

PRELIMINARY

Ligand CLA aA 1113	
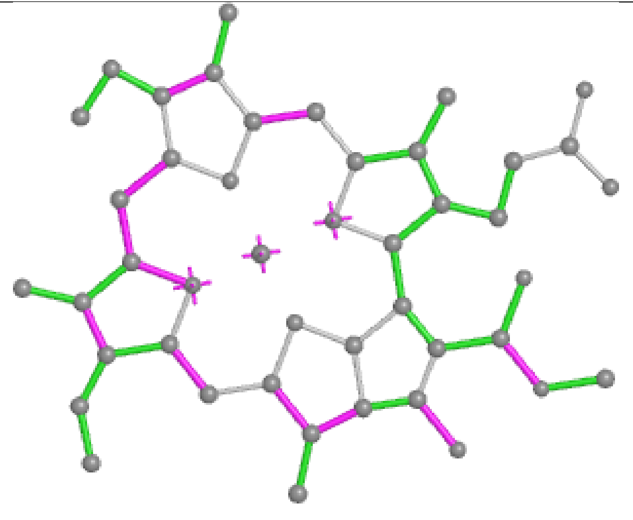 Bond lengths	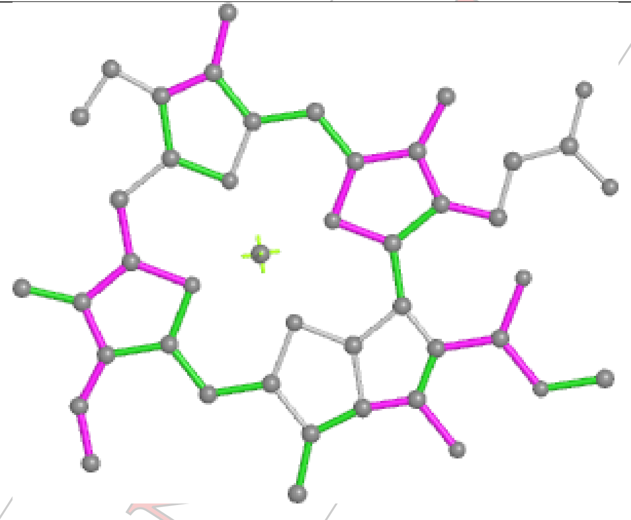 Bond angles
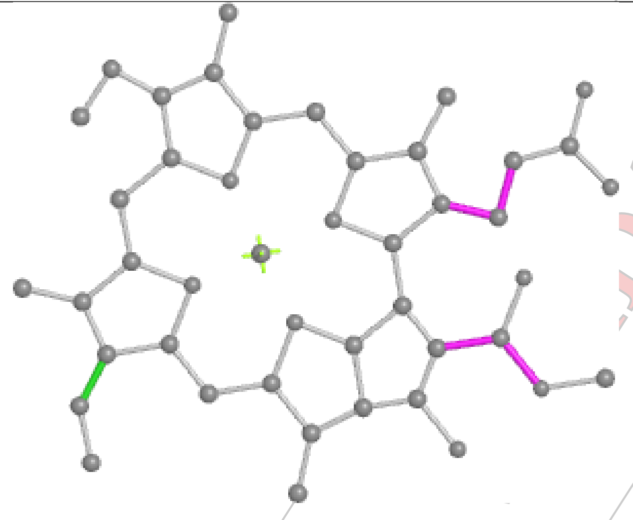 Torsions	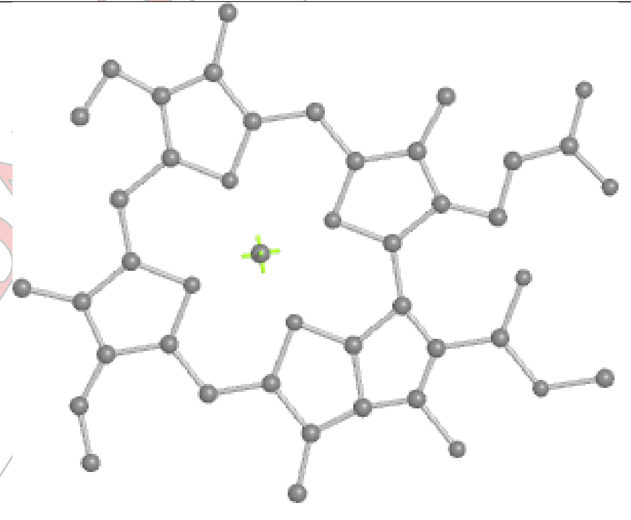 Rings

PRELIMINARY

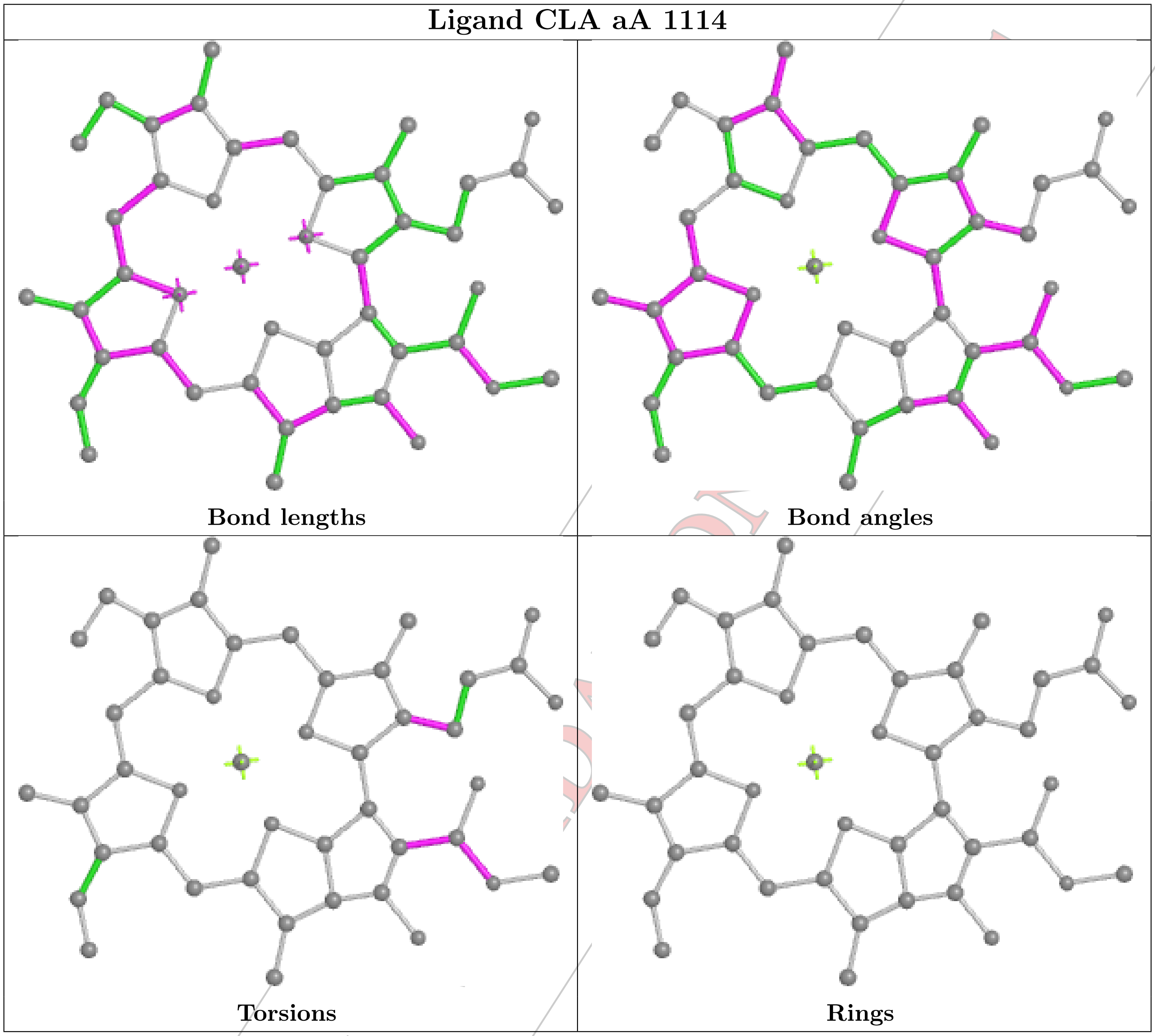

PRELIMINARY

Ligand CLA aA 1115

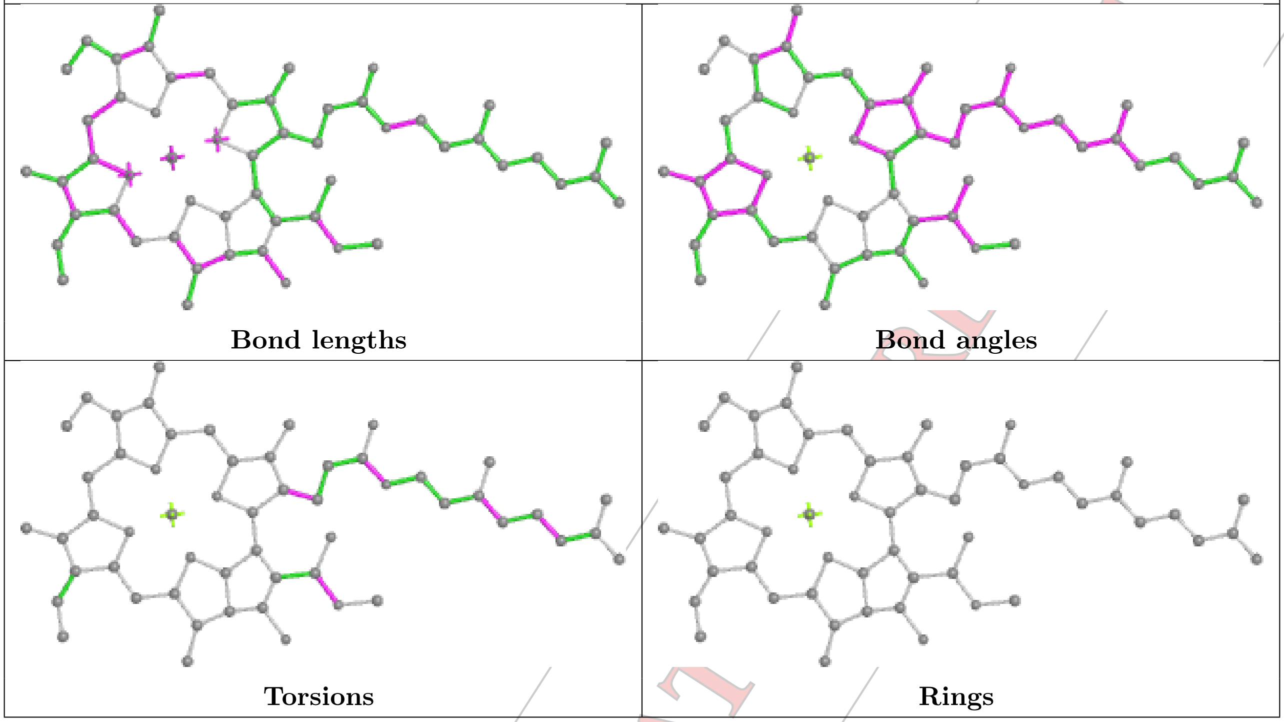

Ligand CLA aA 1116

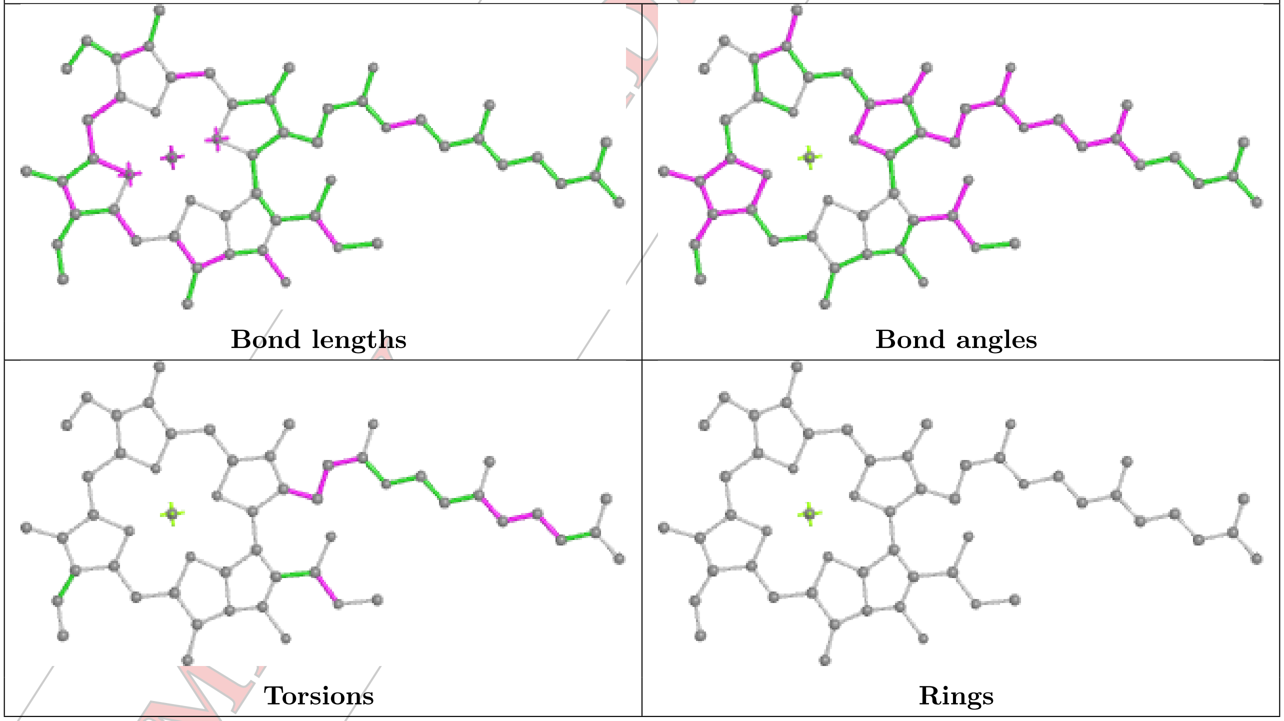

Ligand CLA aA 1117

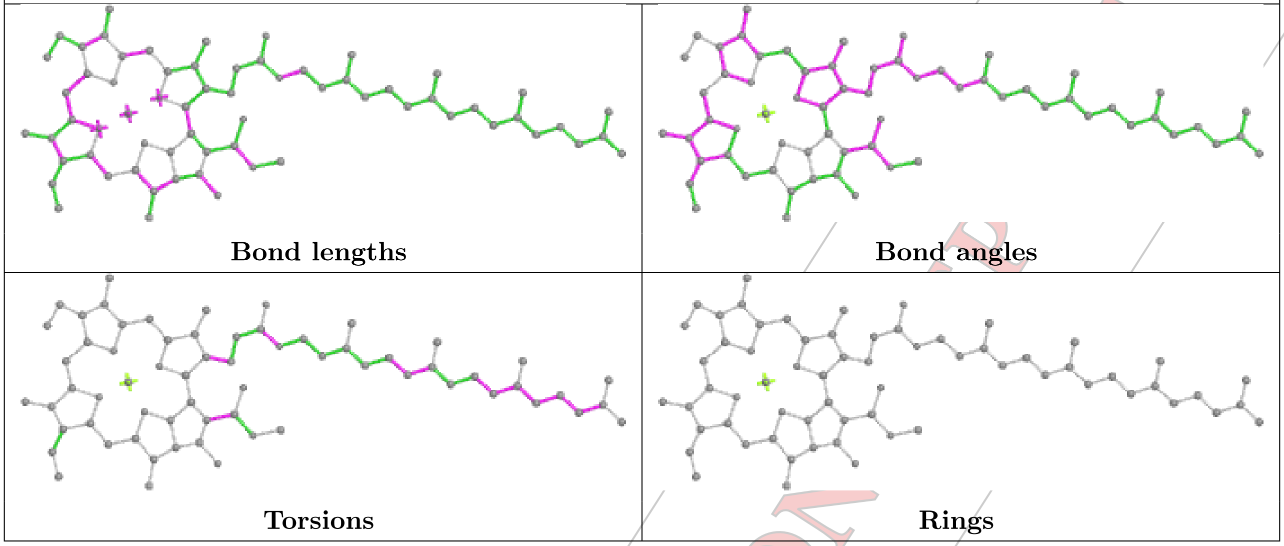

Ligand CLA aA 1118

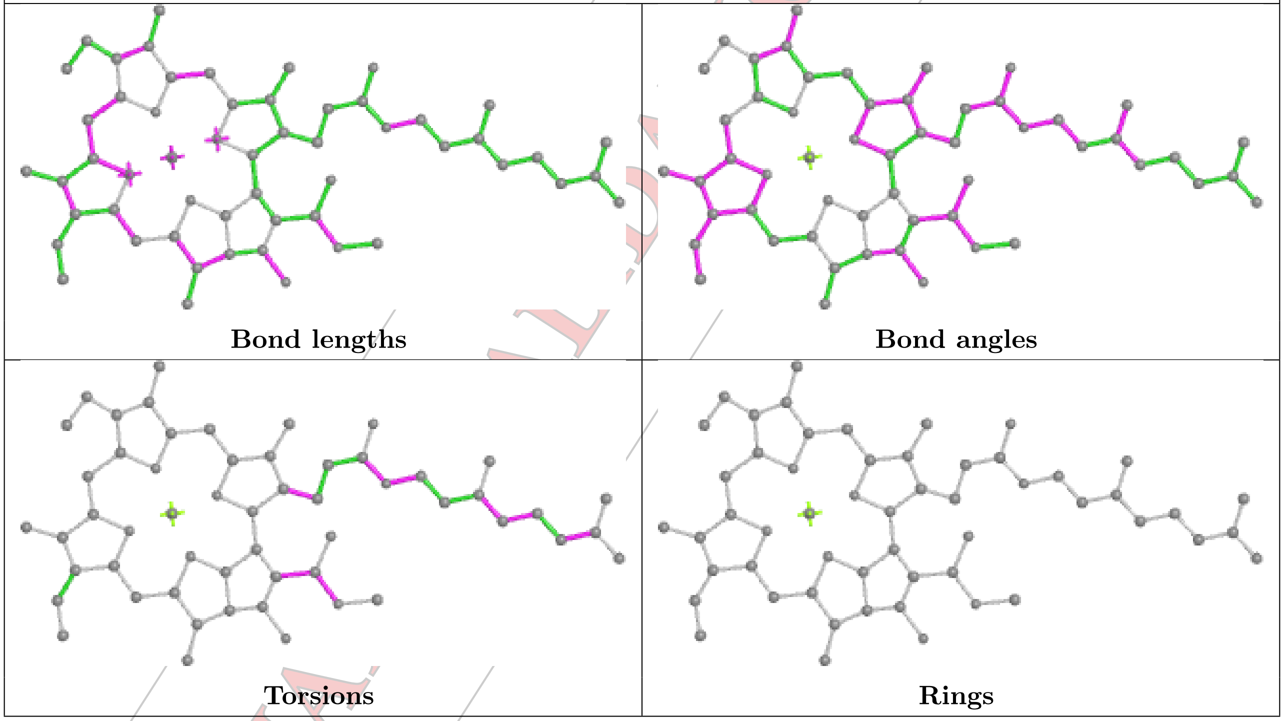

Ligand CLA aA 1119

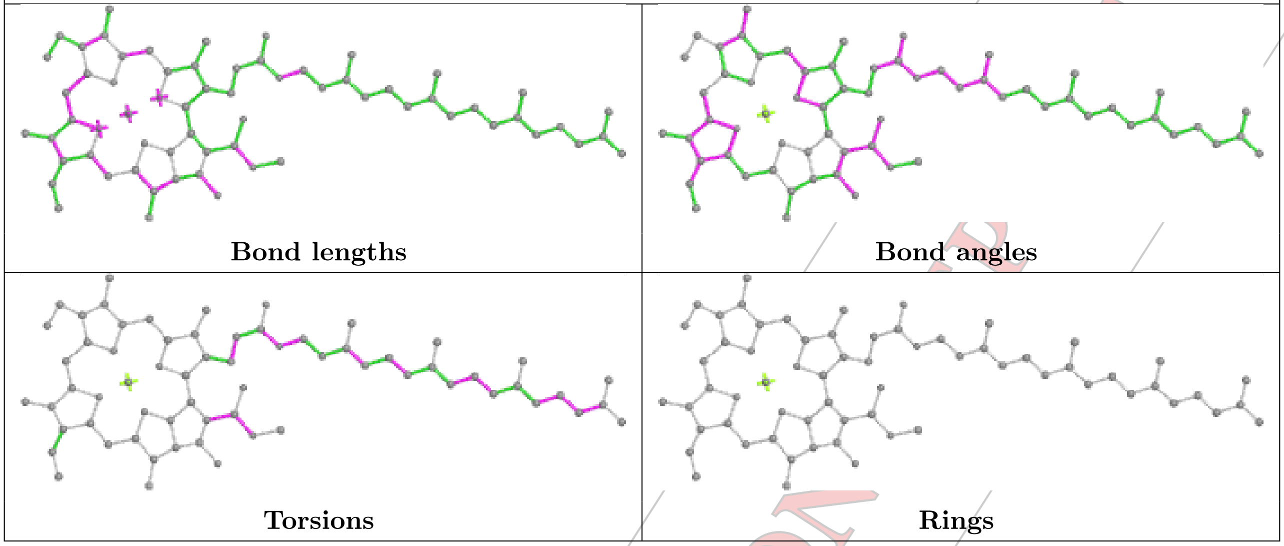

Ligand CLA aA 1122

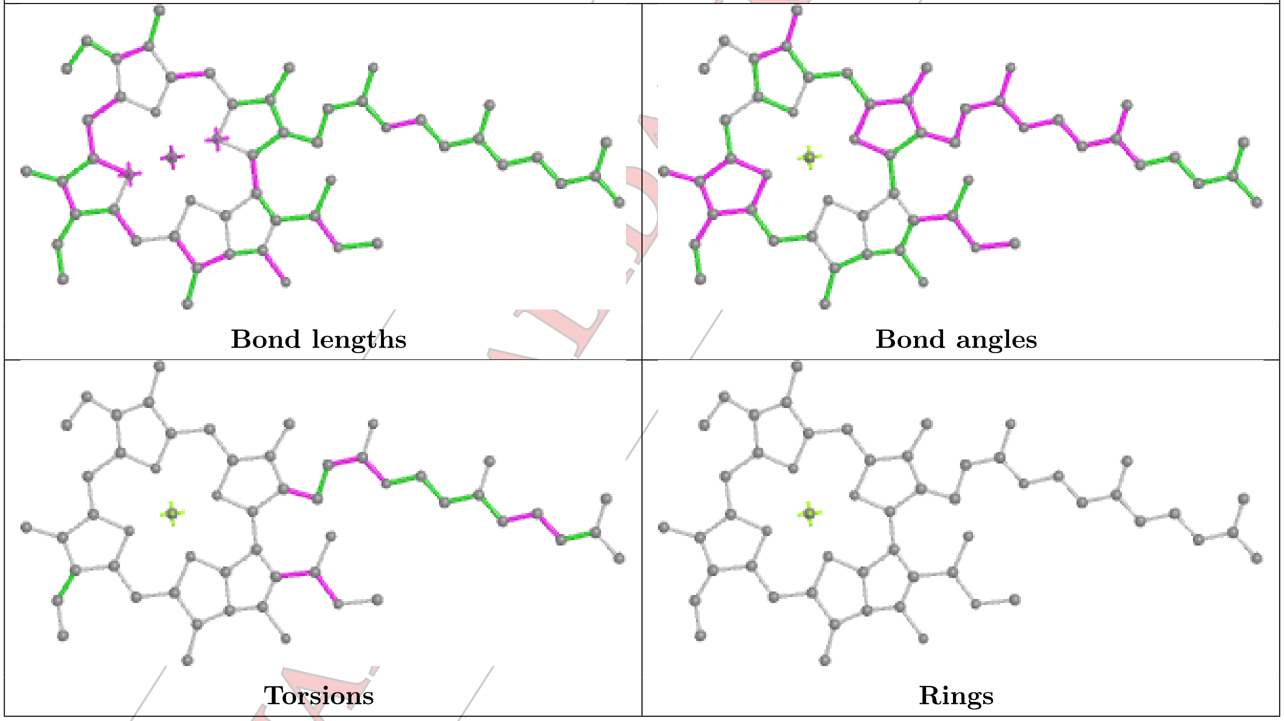

Ligand CLA aA 1123

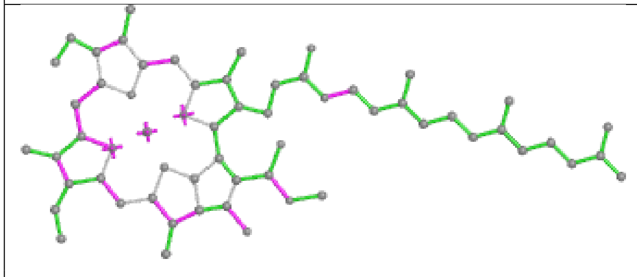
Bond lengths

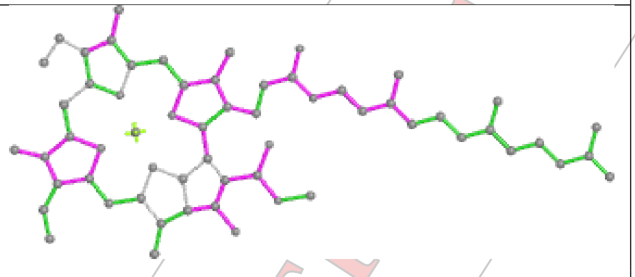
Bond angles

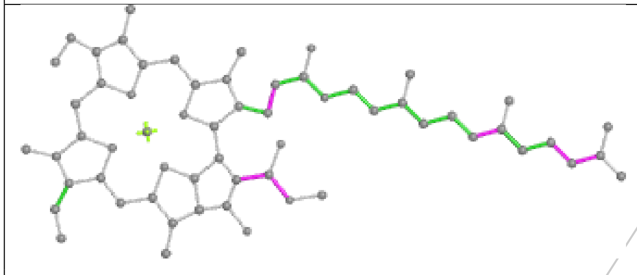
Torsions

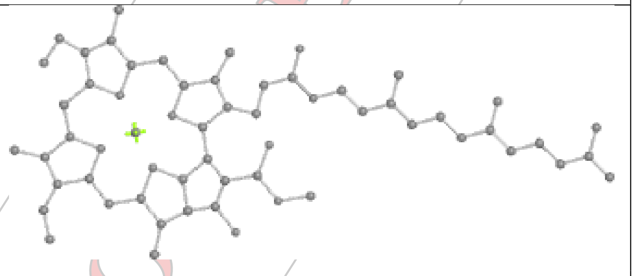
Rings

Ligand CLA aA 1124

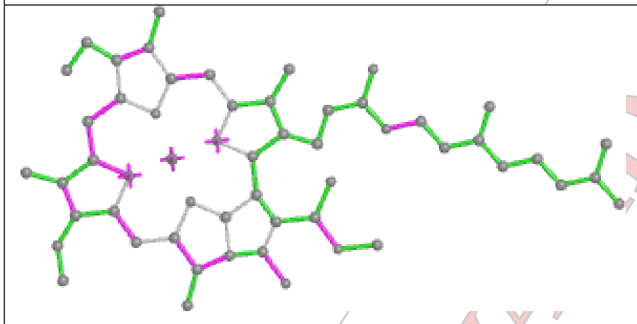
Bond lengths

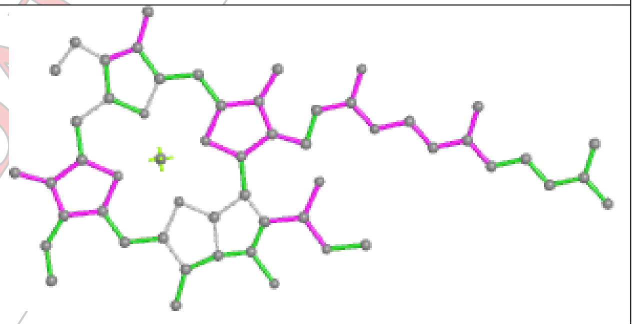
Bond angles

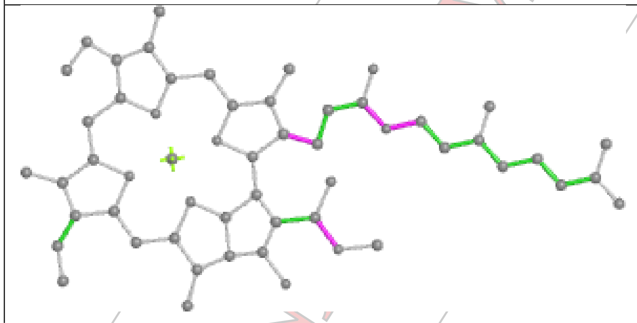
Torsions

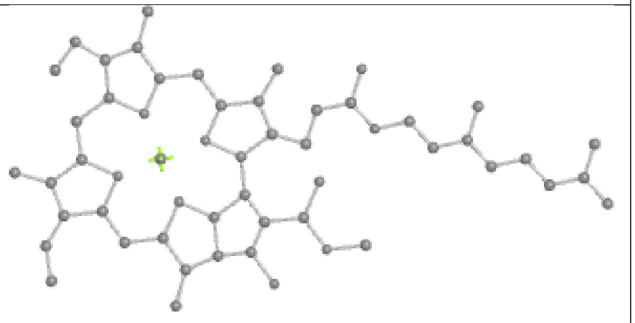
Rings

Ligand CLA aA 1125

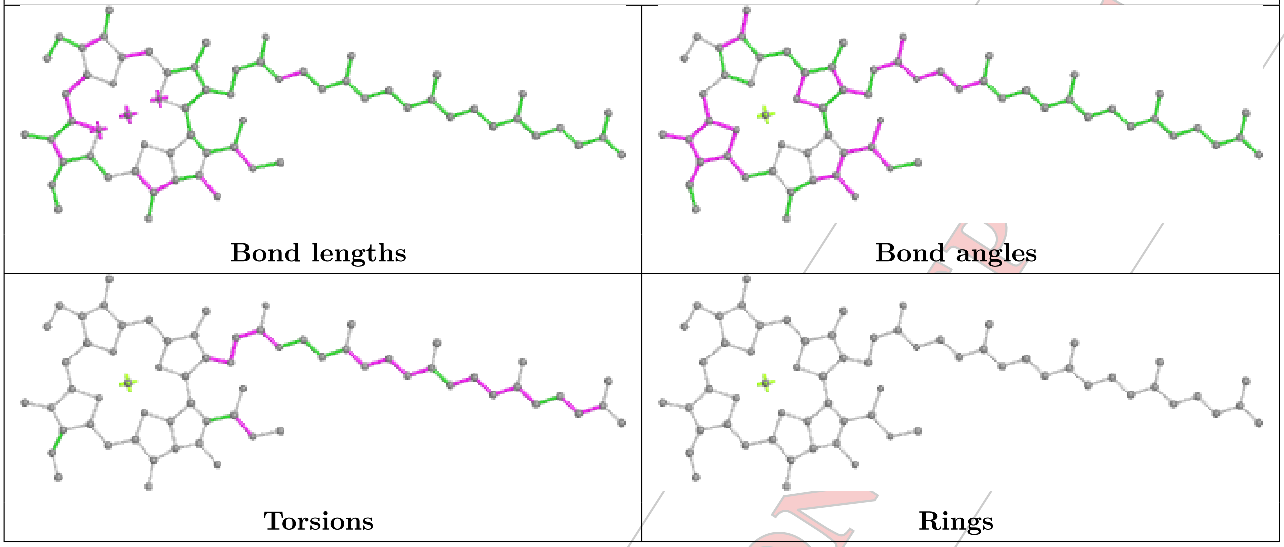

Bond lengths | Bond angles | Torsions | Rings

Ligand CLA aA 1126

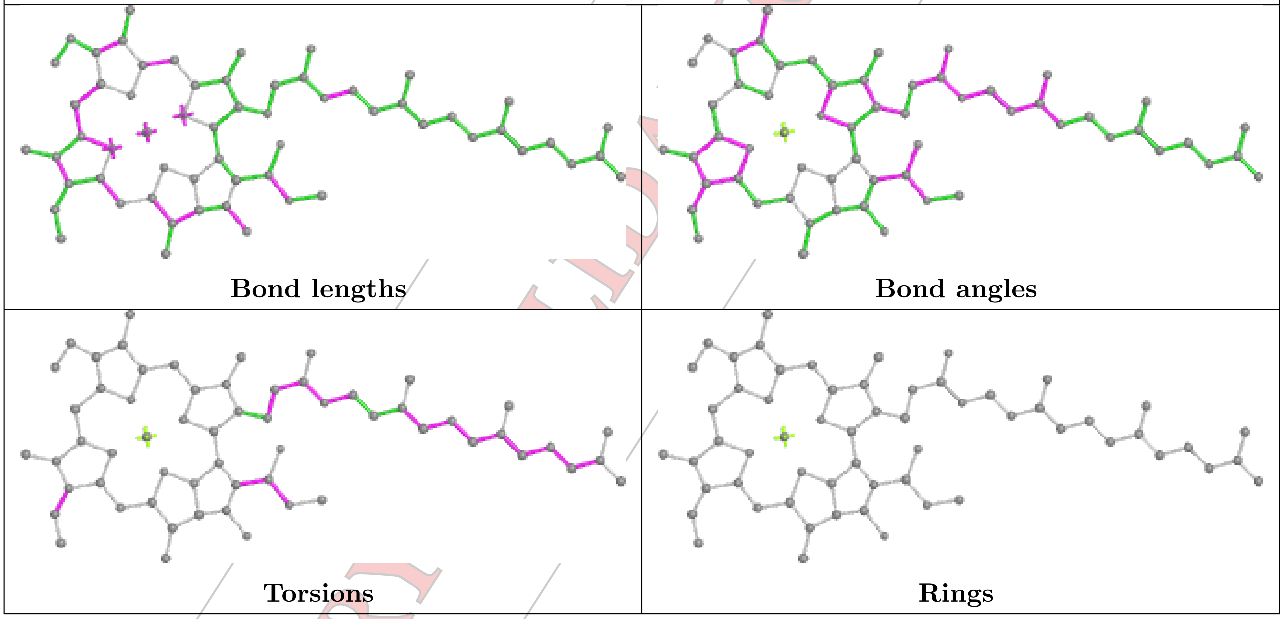

Bond lengths | Bond angles | Torsions | Rings

PRELIMINARY

Ligand CLA aA 1127

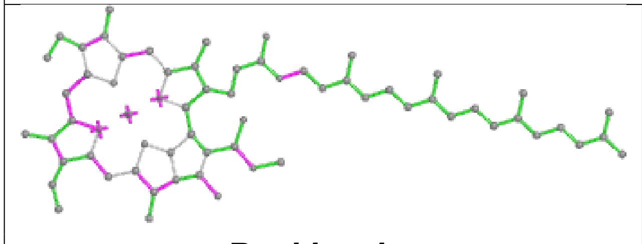
Bond lengths

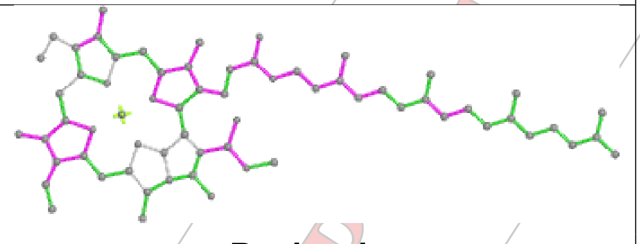
Bond angles

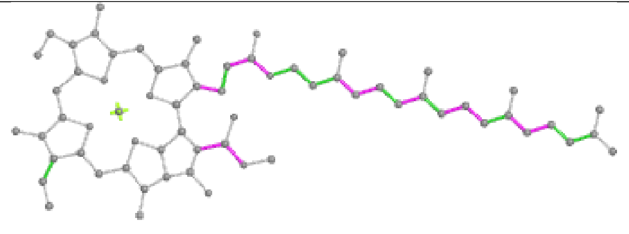
Torsions

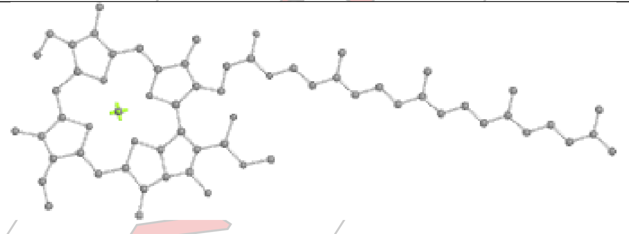
Rings

Ligand CLA aA 1128

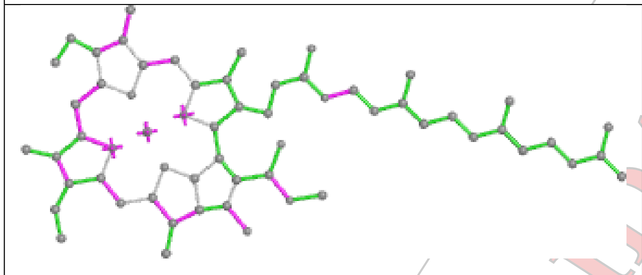
Bond lengths

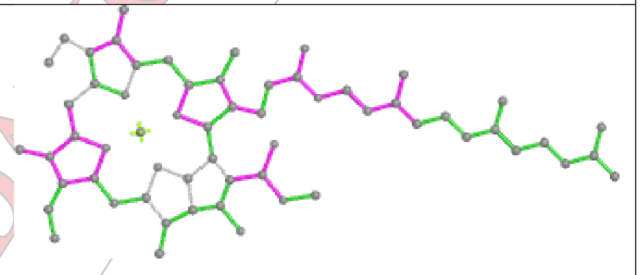
Bond angles

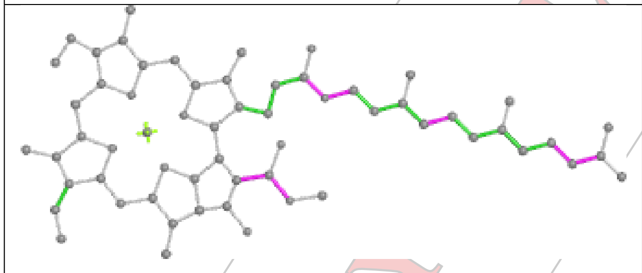
Torsions

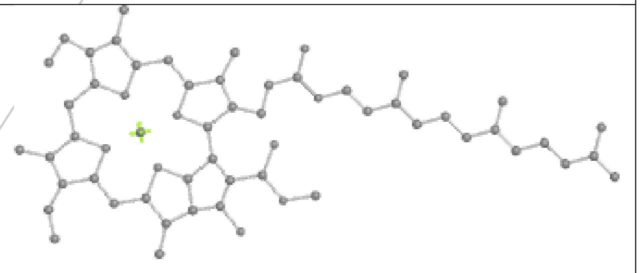
Rings

PRELIMINARY

Ligand CLA aA 1129

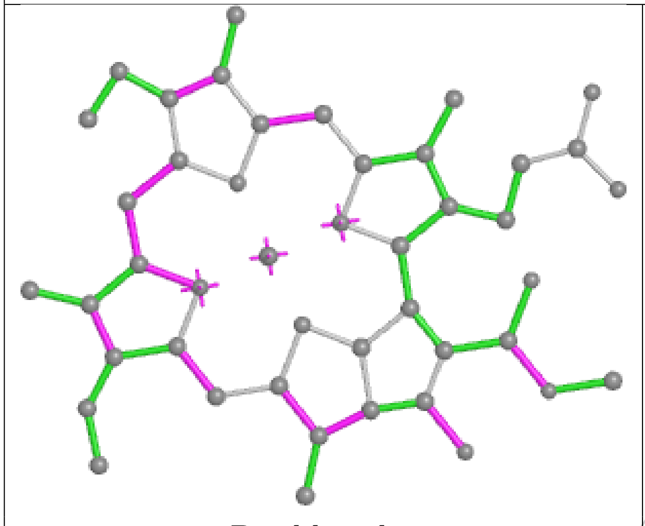

Bond lengths

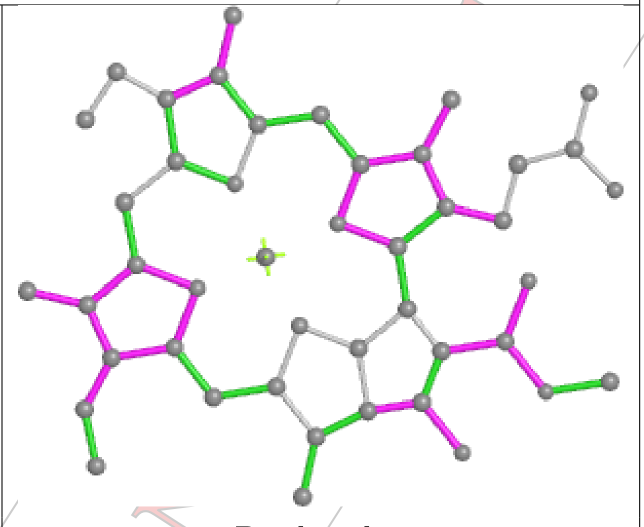

Bond angles

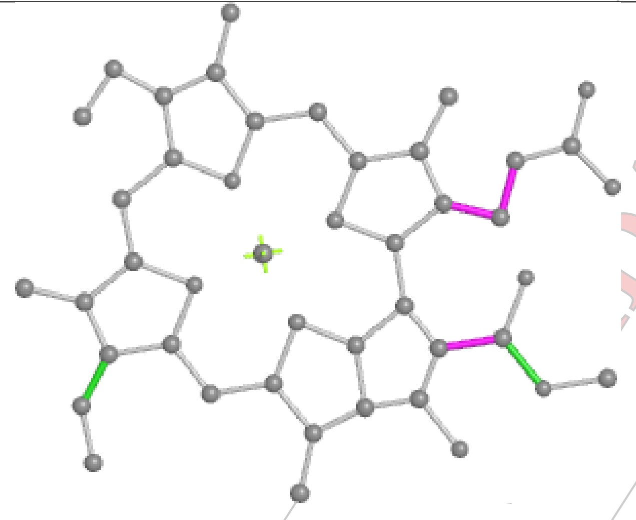

Torsions

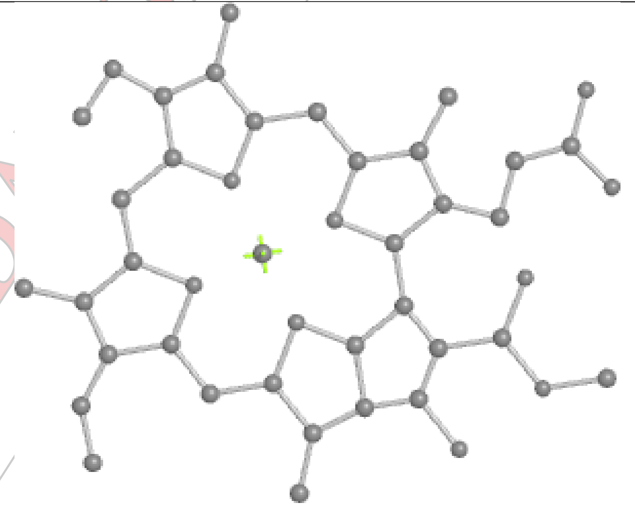

Rings

PRELIMINARY

Ligand CLA aA 1130

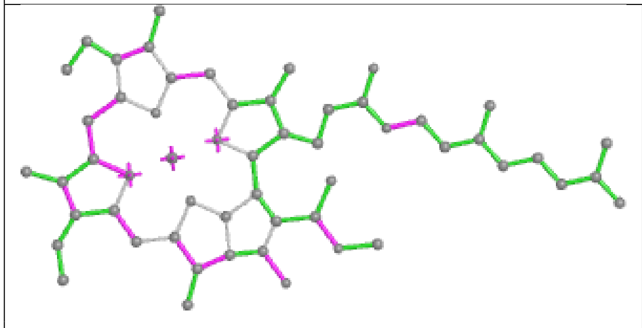

Bond lengths

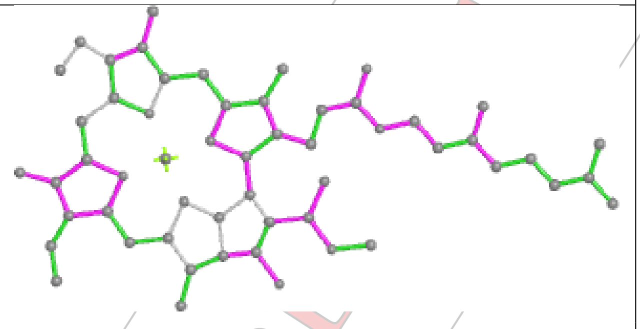

Bond angles

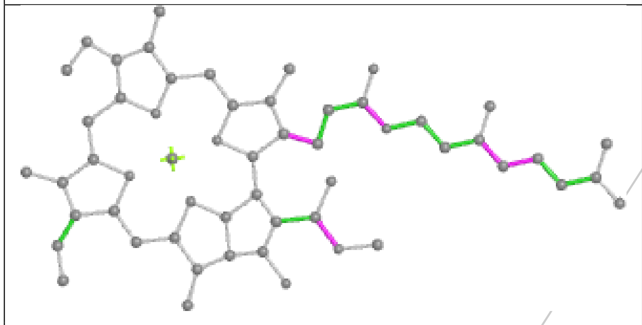

Torsions

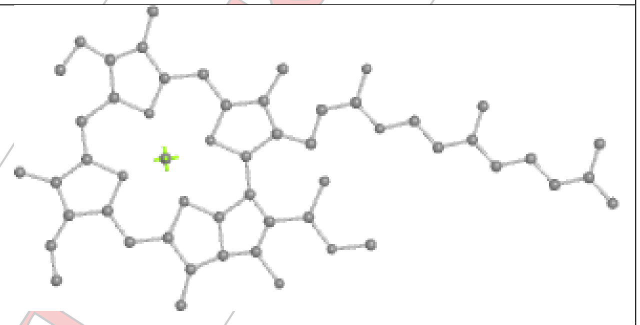

Rings

Ligand CLA aA 1131

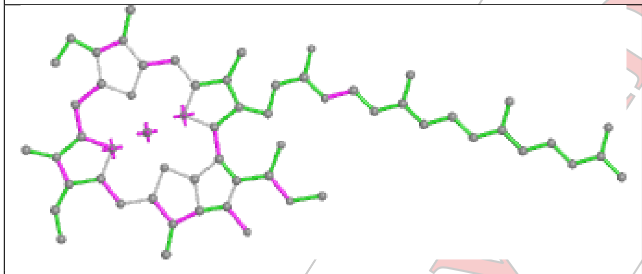

Bond lengths

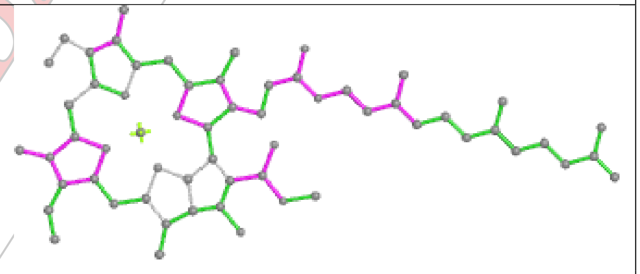

Bond angles

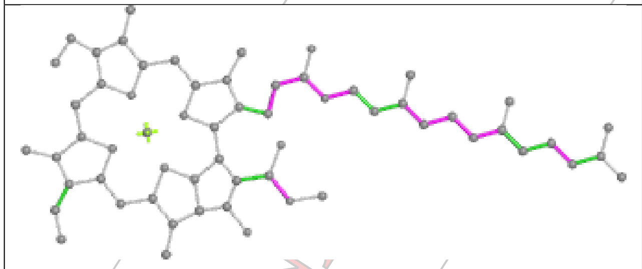

Torsions

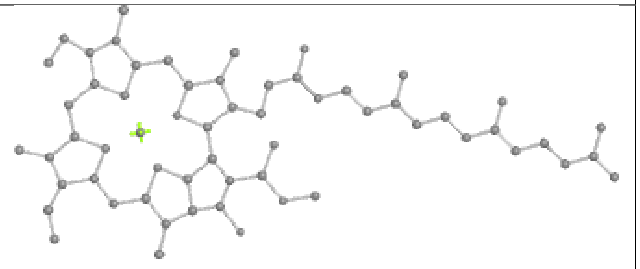

Rings

Ligand CLA aA 1132

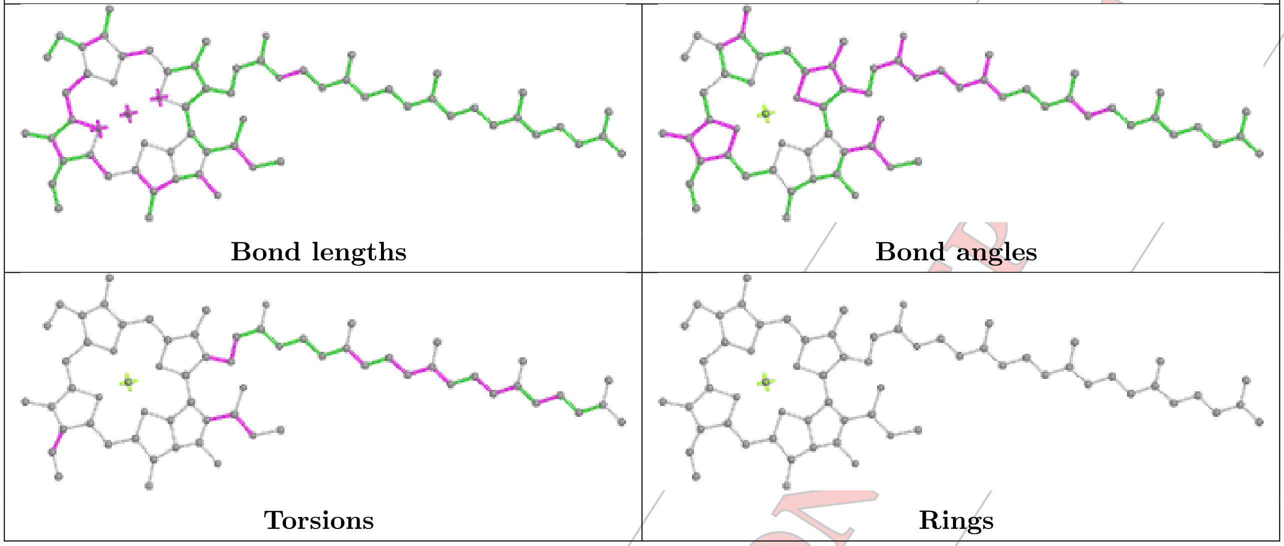

Ligand CLA aA 1133

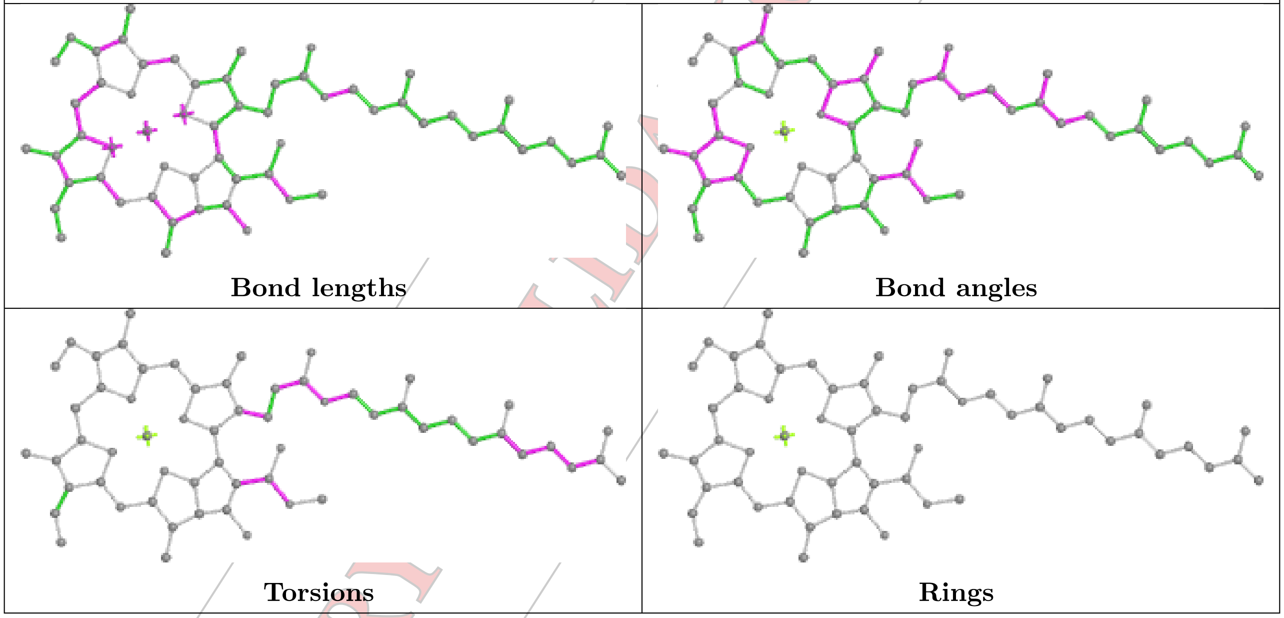

PRELIMINARY

Ligand CLA aA 1135

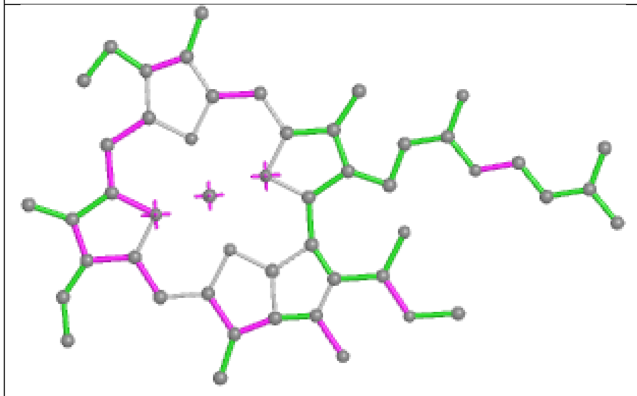

Bond lengths

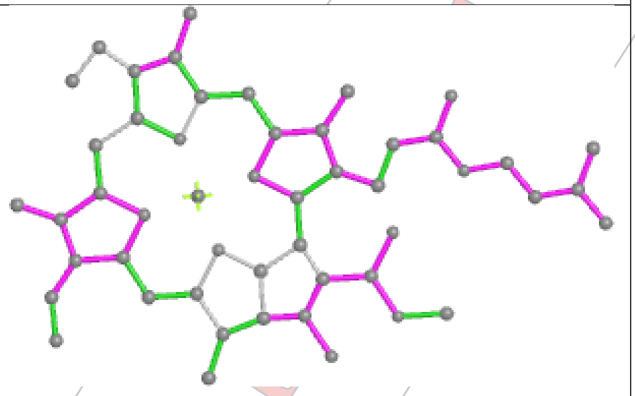

Bond angles

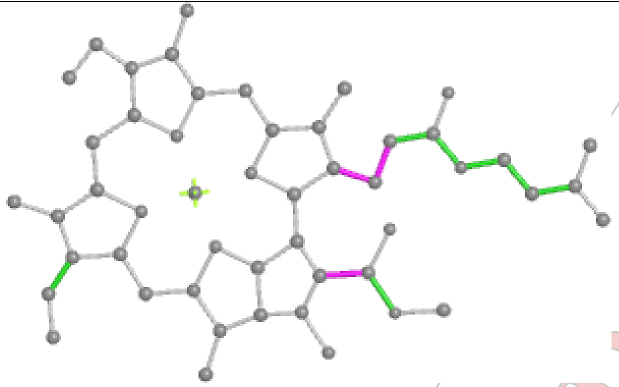

Torsions

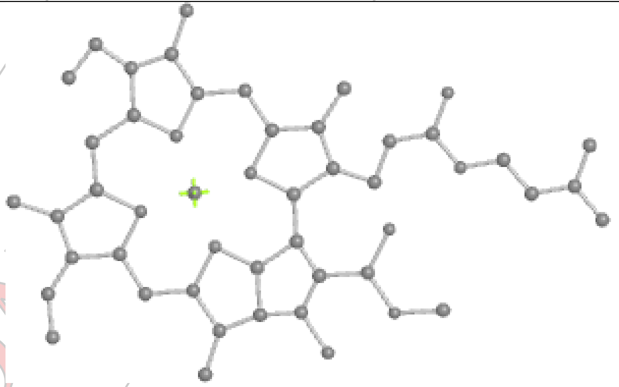

Rings

Ligand CLA aA 1136

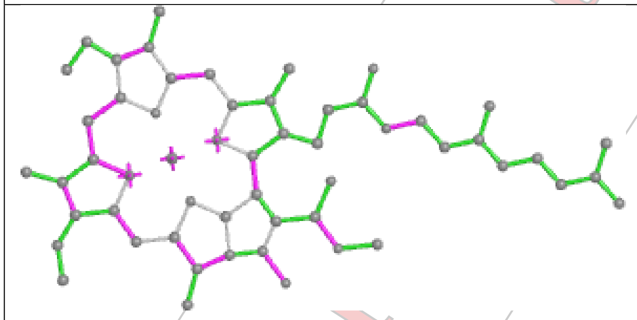

Bond lengths

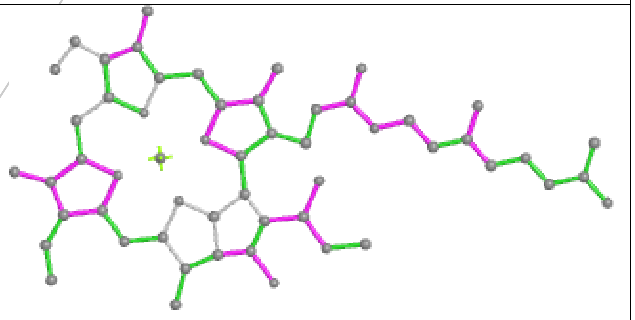

Bond angles

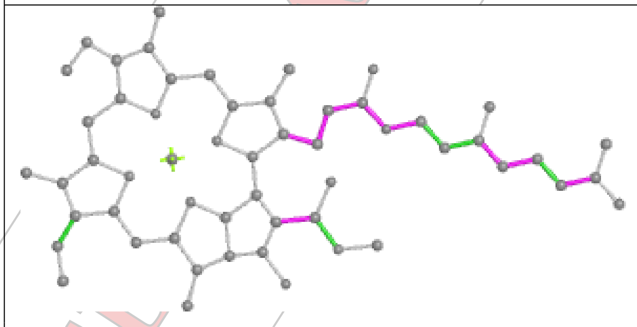

Torsions

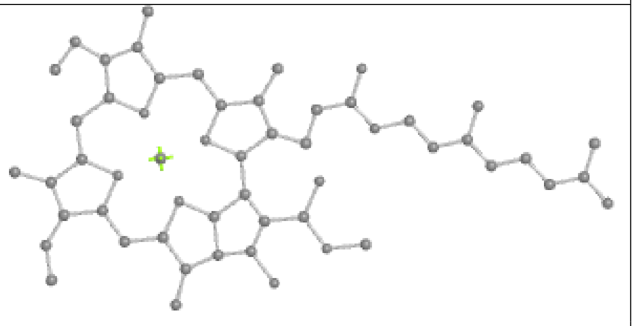

Rings

Ligand CLA aA 1137

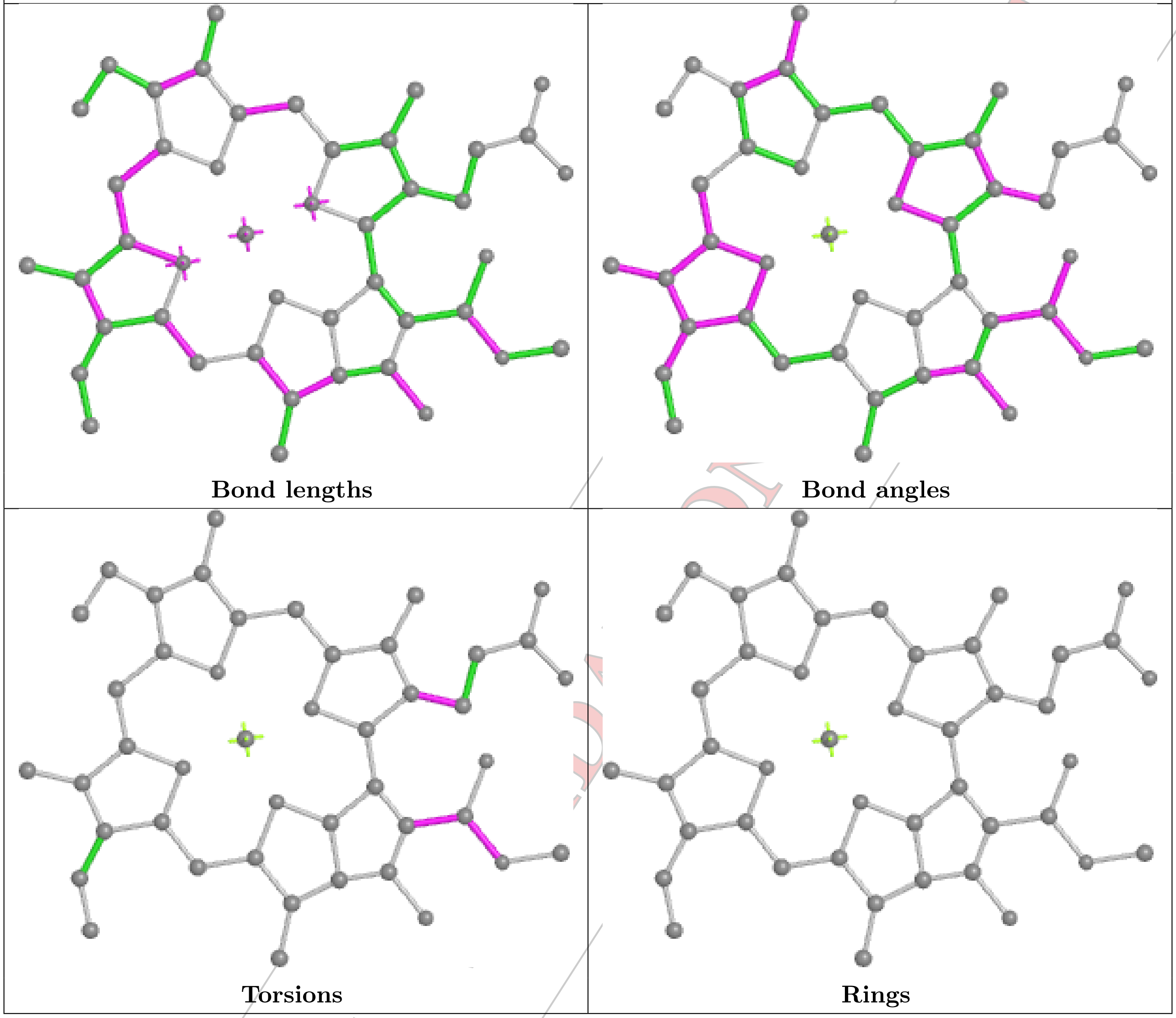

Ligand CLA aA 1138

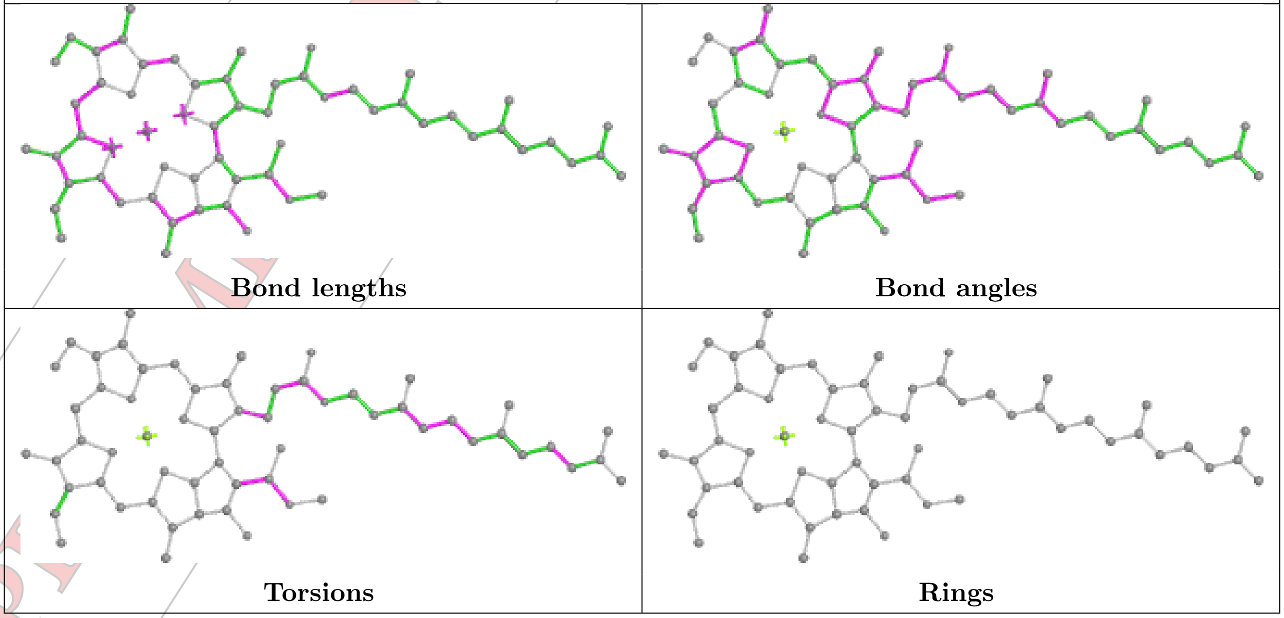

Ligand CLA aA 1139

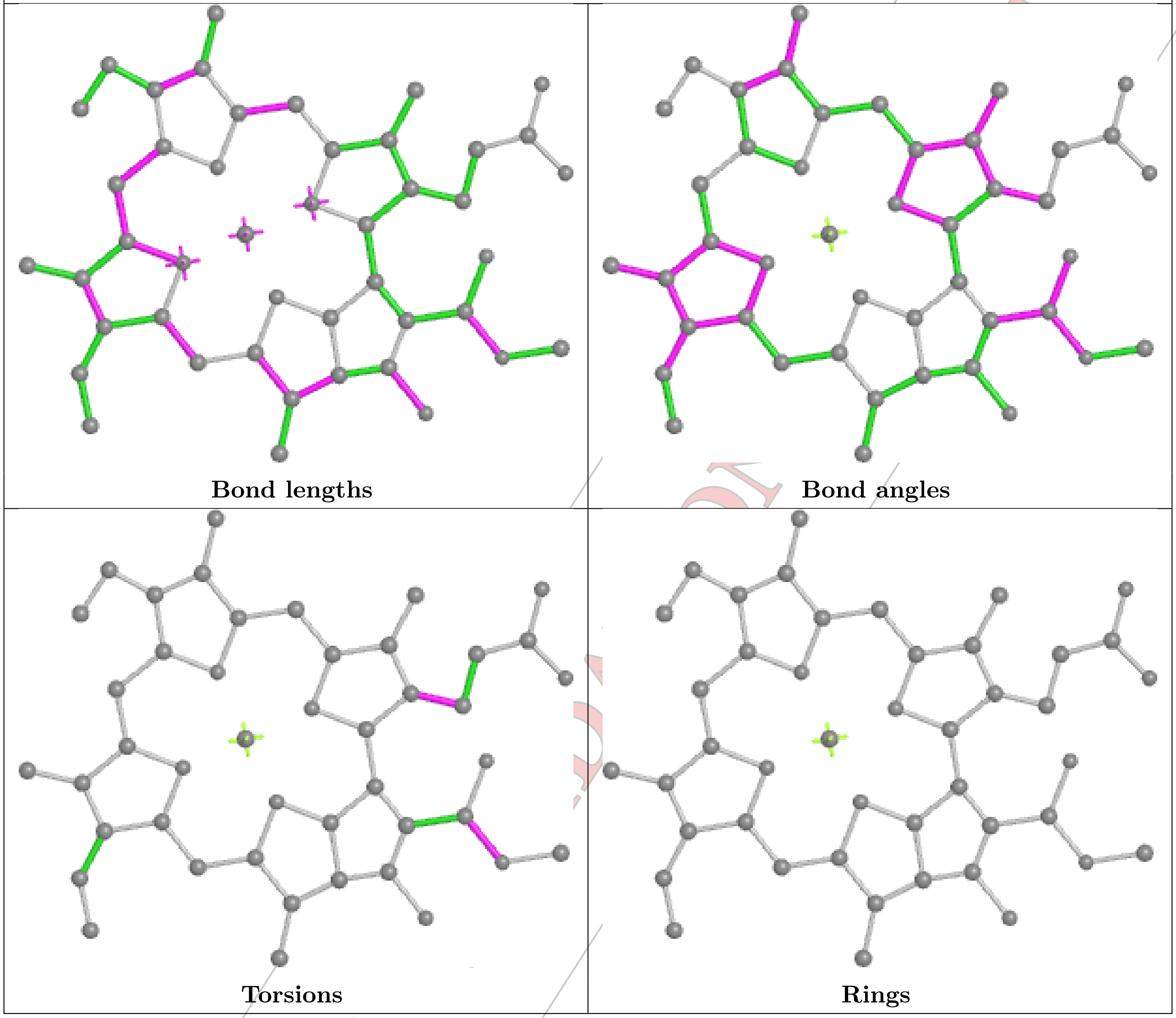

Ligand CLA aA 1140

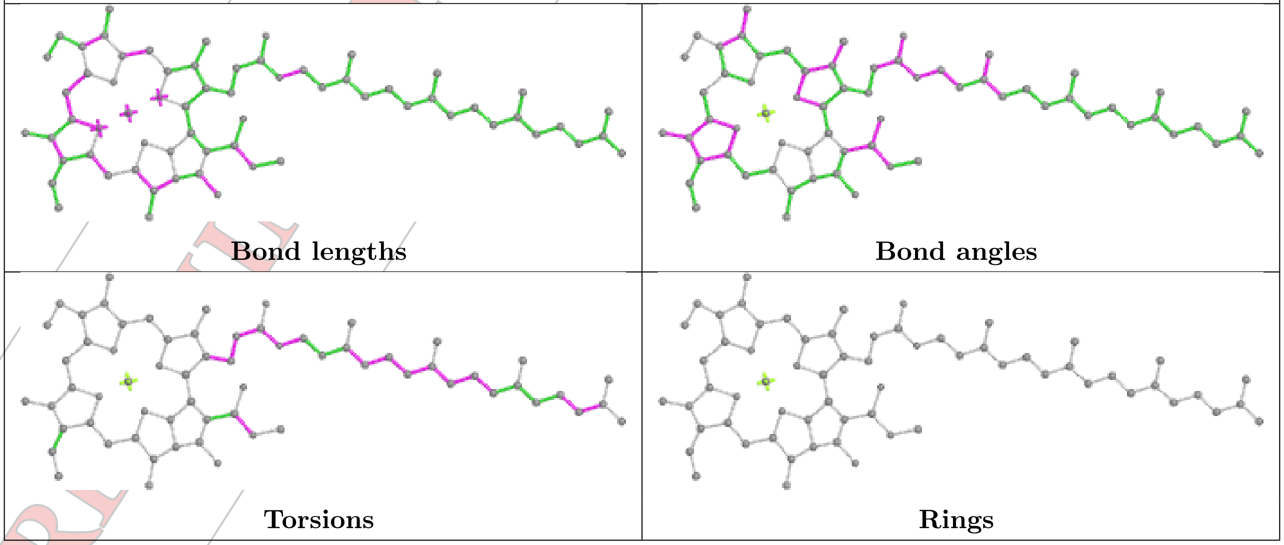

Ligand PQN aA 2001

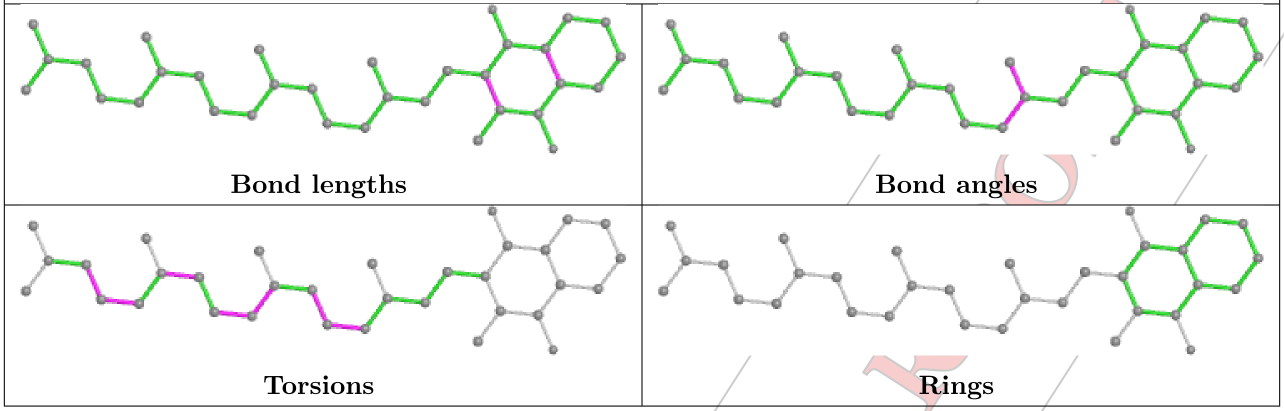

Ligand BCR aA 4001

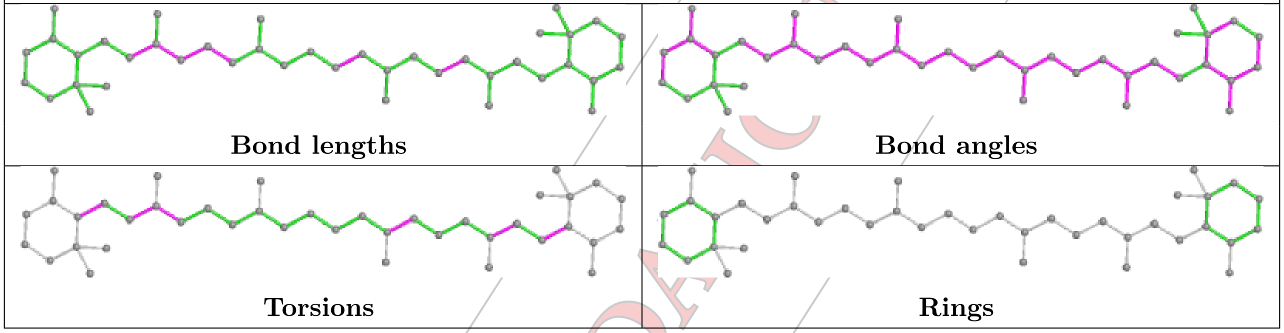

Ligand BCR aA 4002

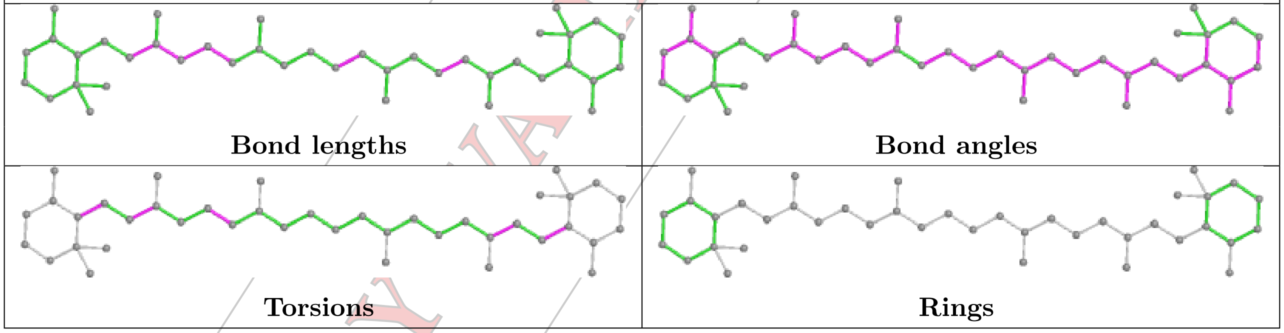

Ligand BCR aA 4003

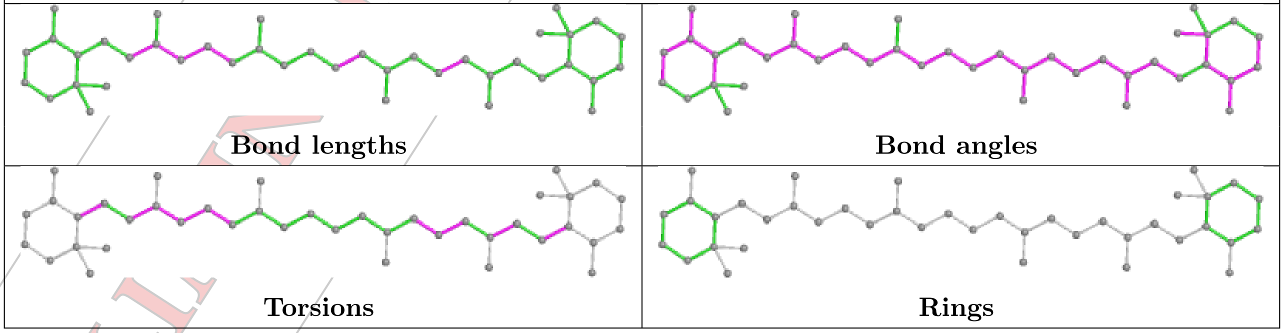

PRELIMINARY DATA

Ligand BCR aA 4007

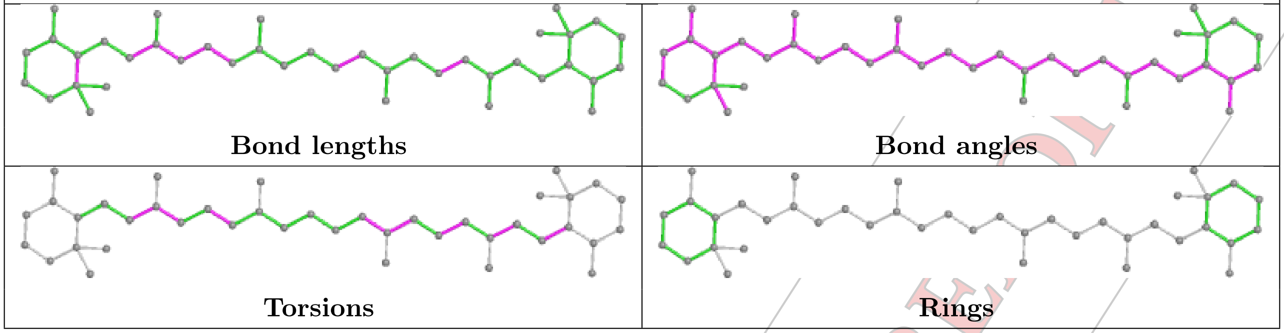

Bond lengths | Bond angles | Torsions | Rings

Ligand BCR aA 4008

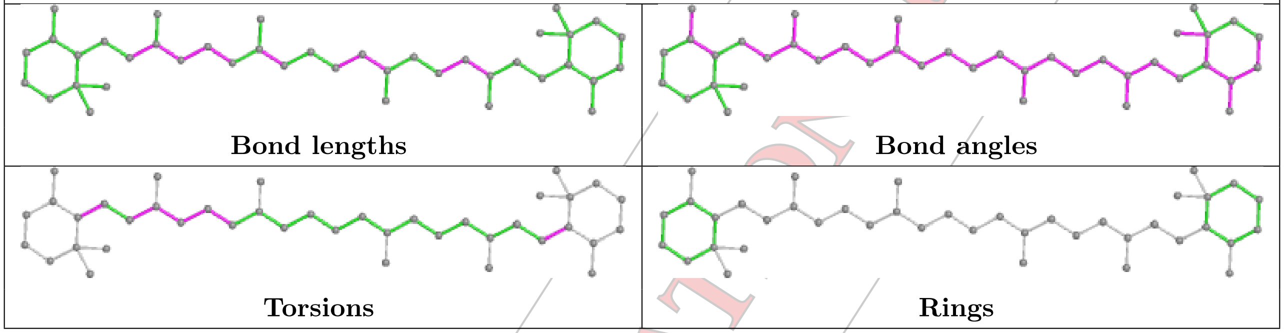

Bond lengths | Bond angles | Torsions | Rings

Ligand BCR aA 4011

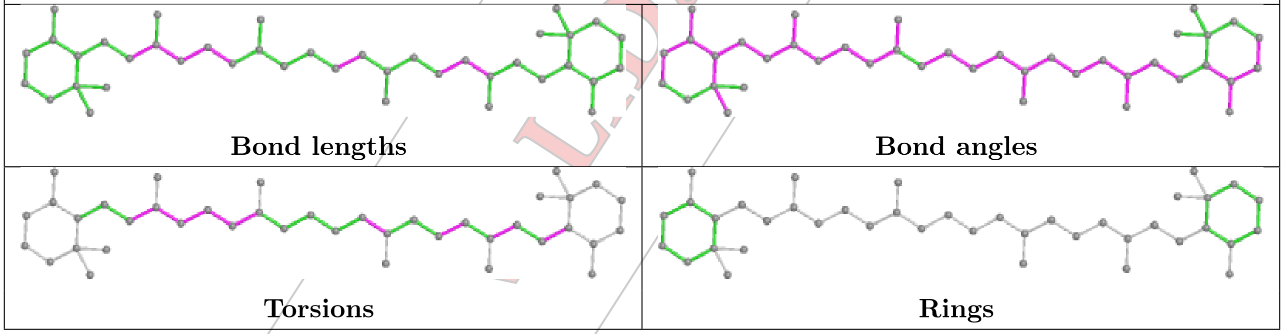

Bond lengths | Bond angles | Torsions | Rings

PRELIMINARY

Ligand LHG aA 5001

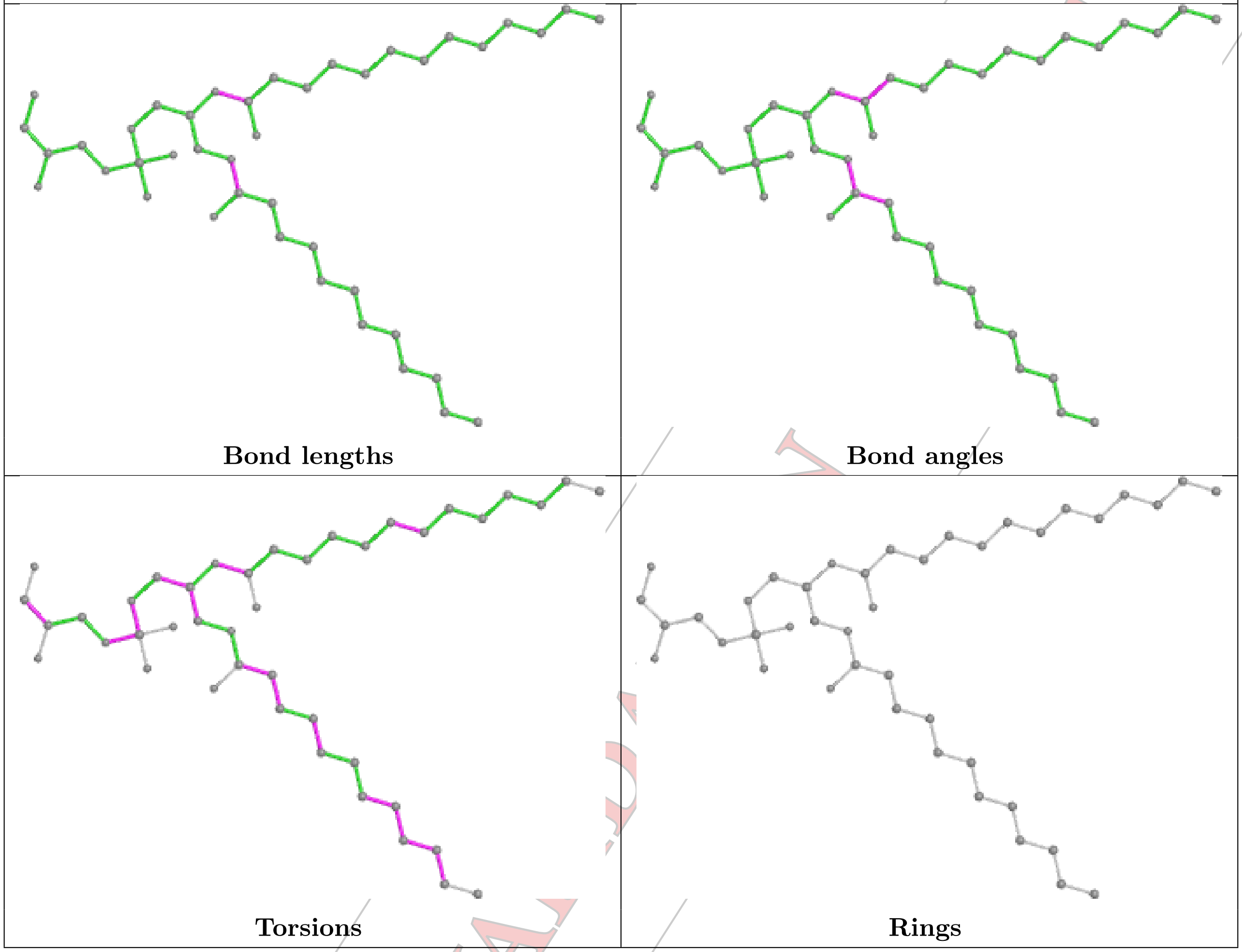

Ligand LMG aA 5005

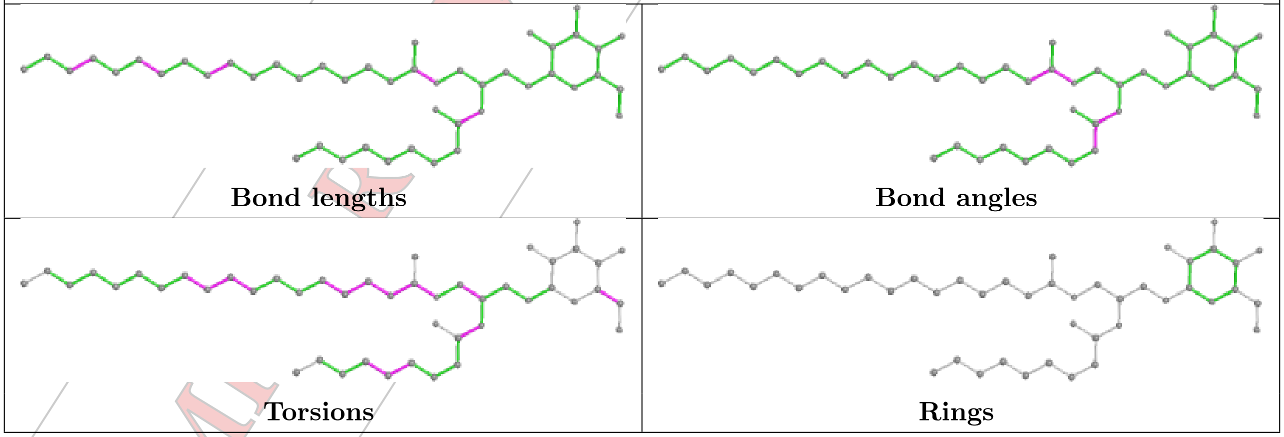

Ligand LMT aA 6001

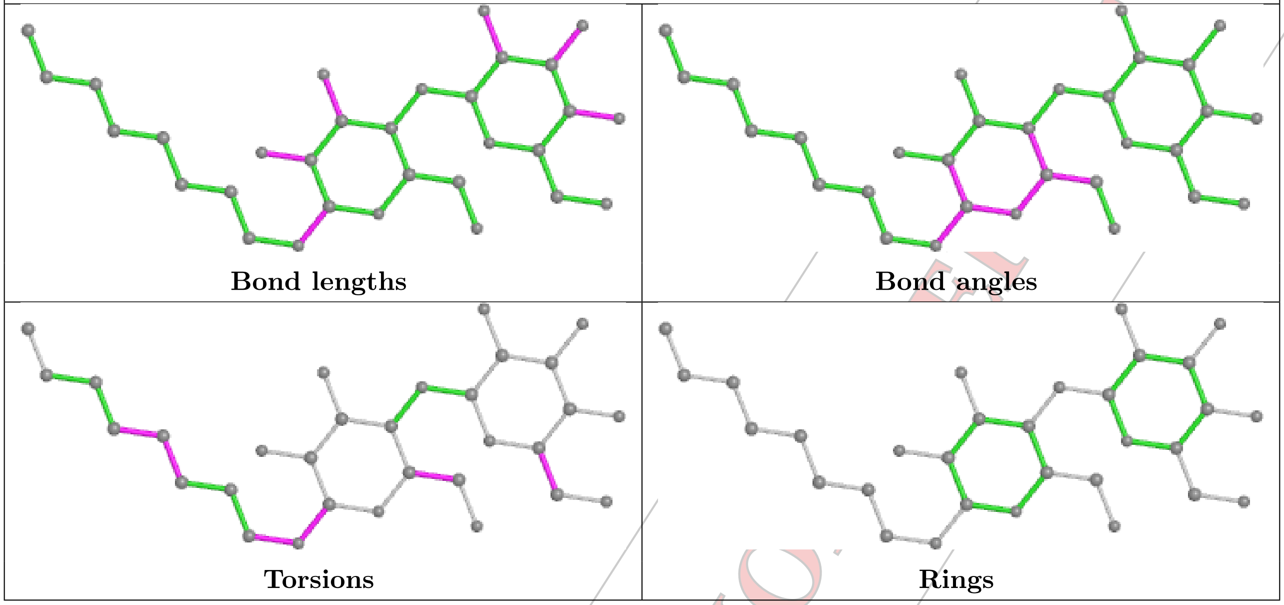

Ligand LMT aA 6002

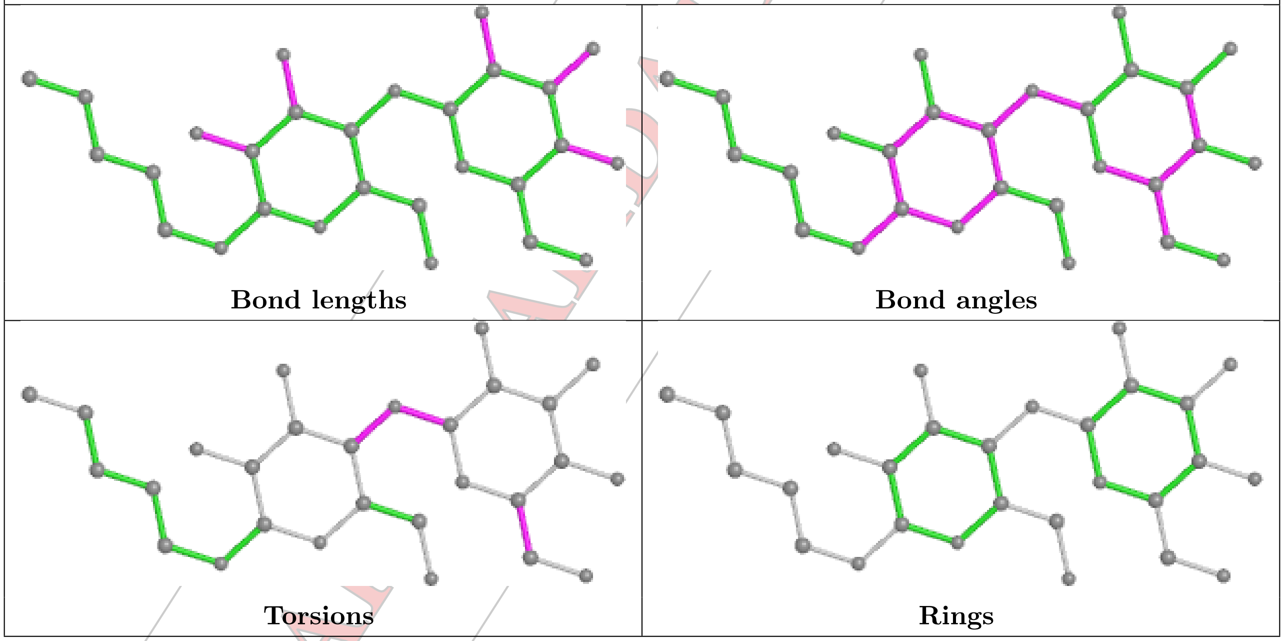

PRELIMINARY

Ligand CLA bA 1021

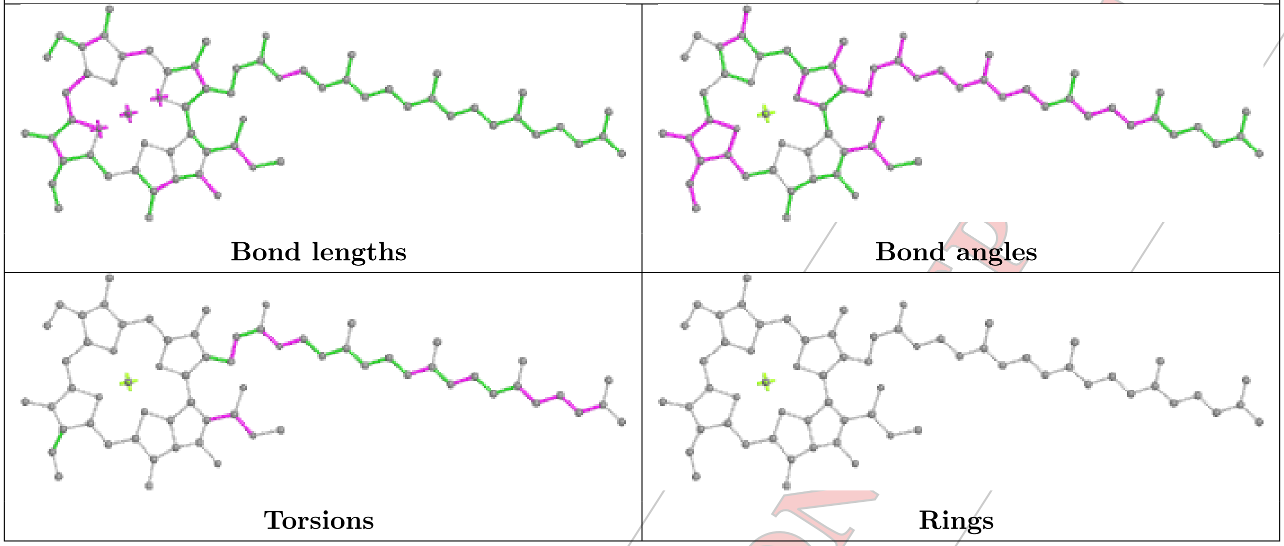

Ligand CLA bA 1022

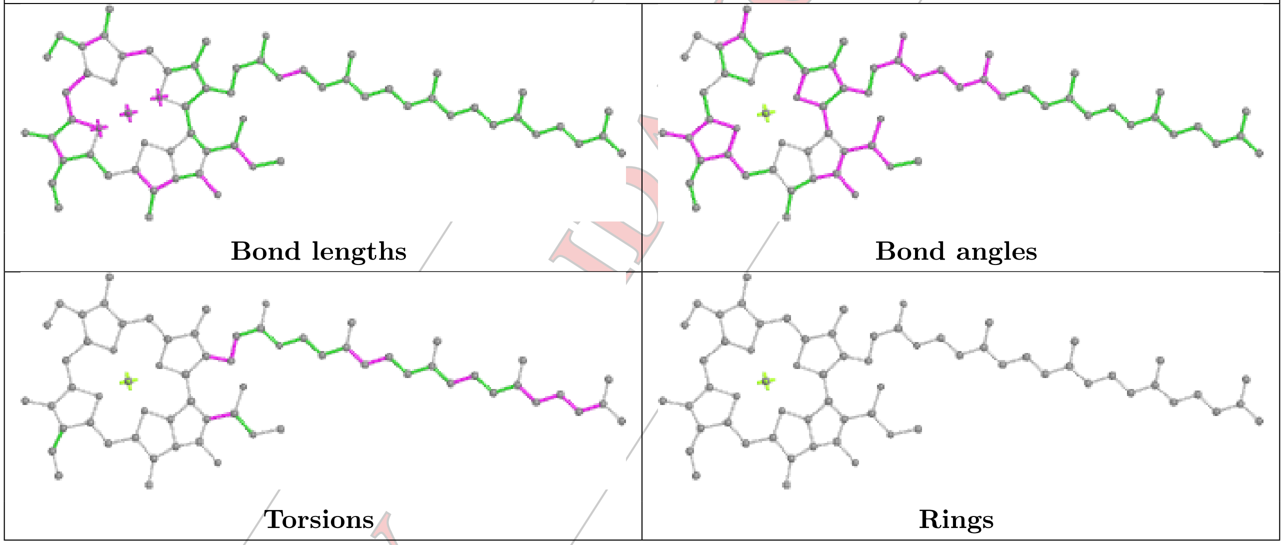

Ligand CLA bA 1023

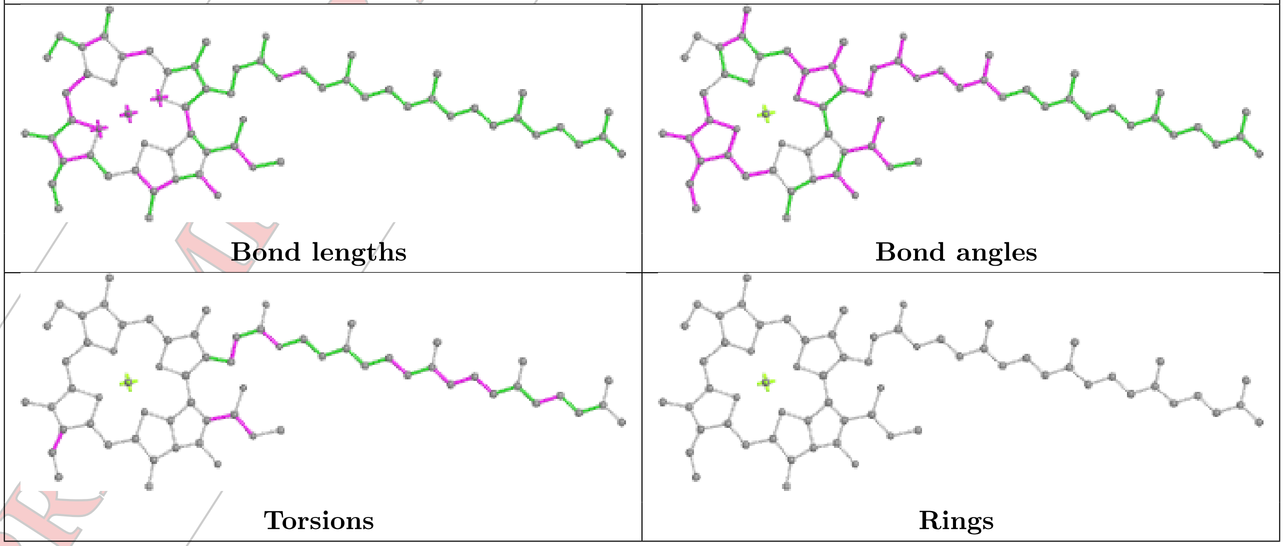

Ligand CLA bA 1201

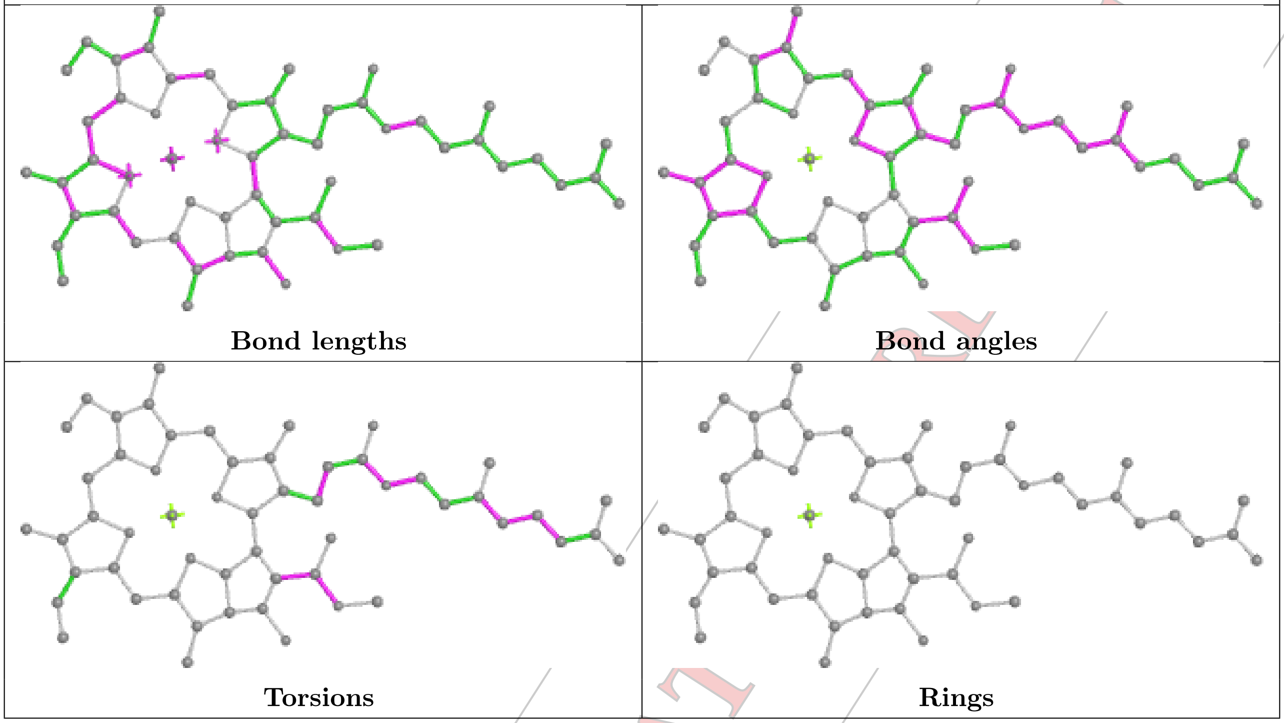

Ligand CLA bA 1202

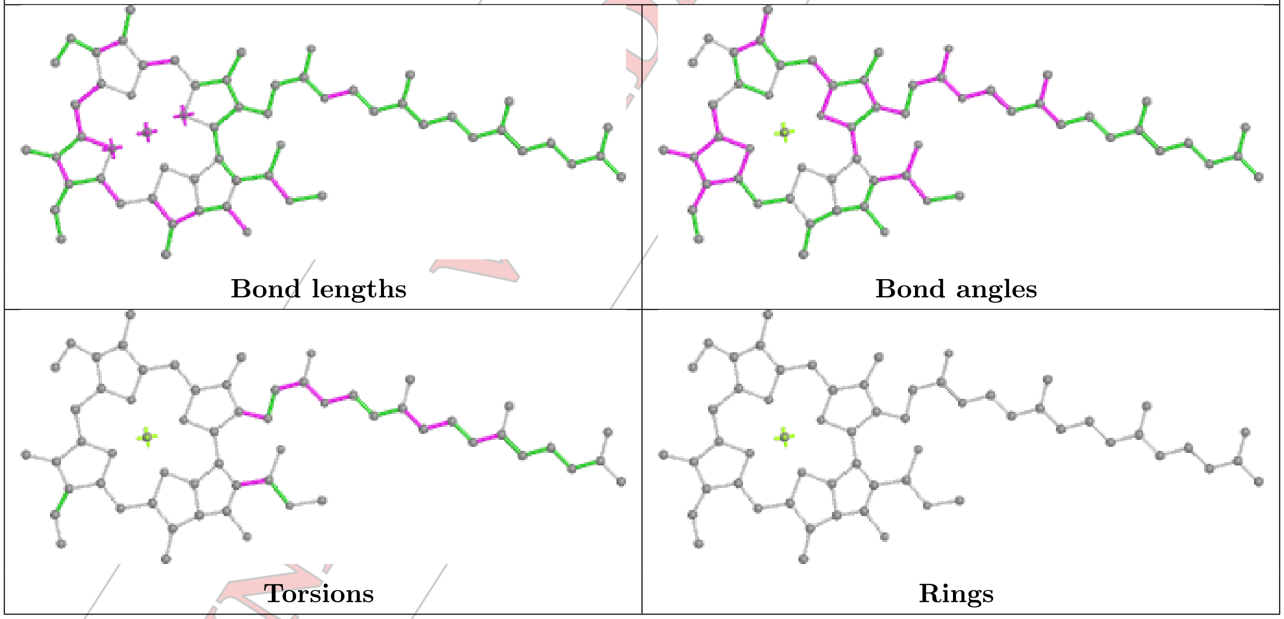

PRELIMINARY

Ligand CLA bA 1203

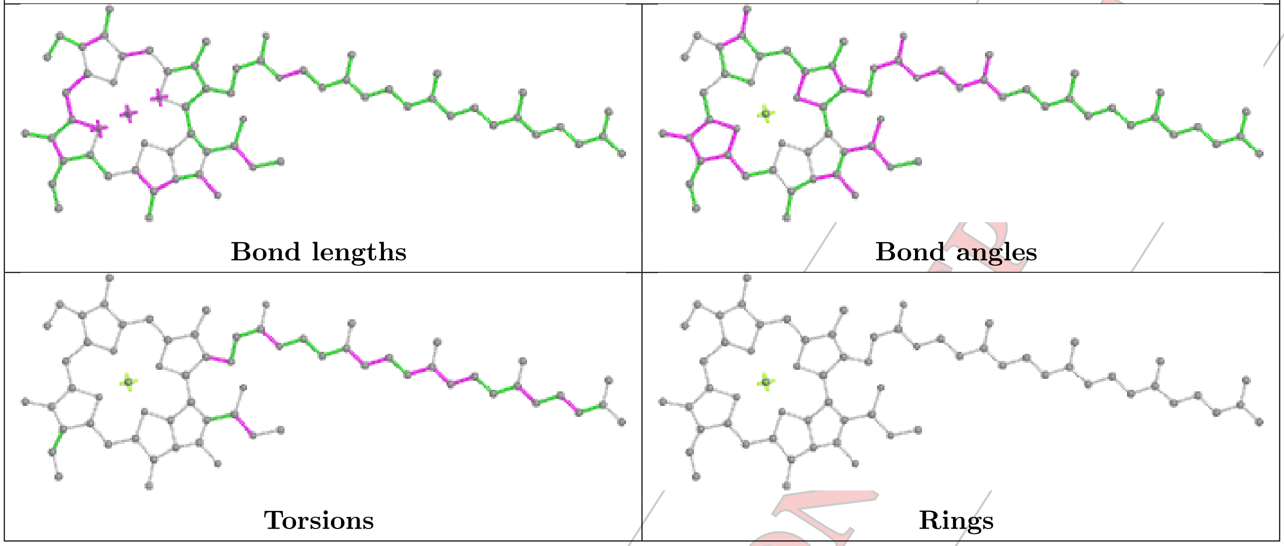

Ligand CLA bA 1204

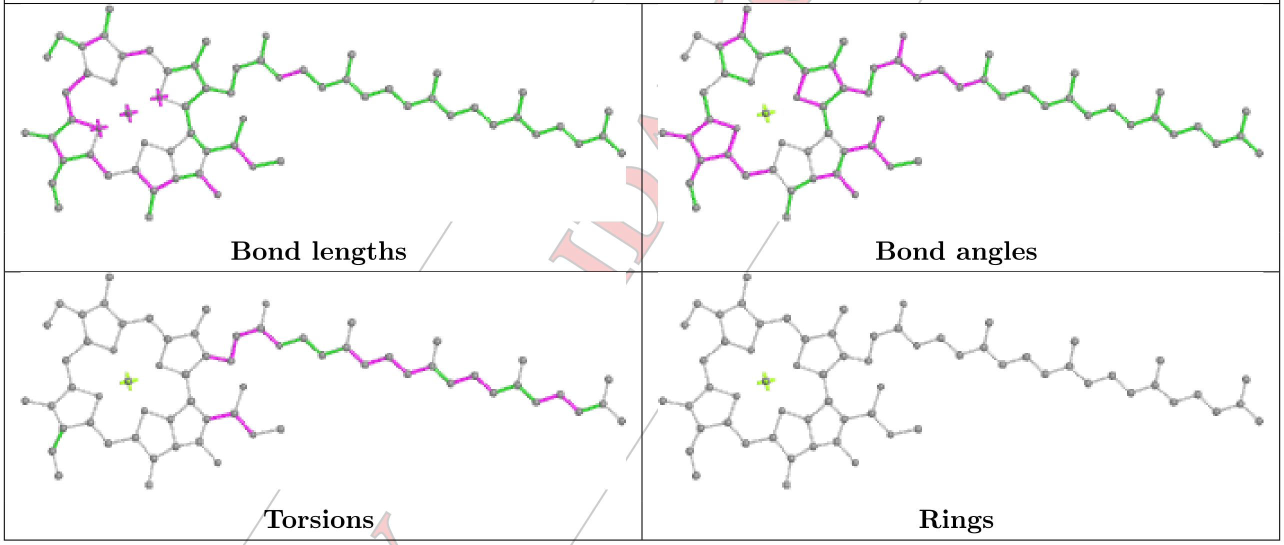

Ligand CLA bA 1205

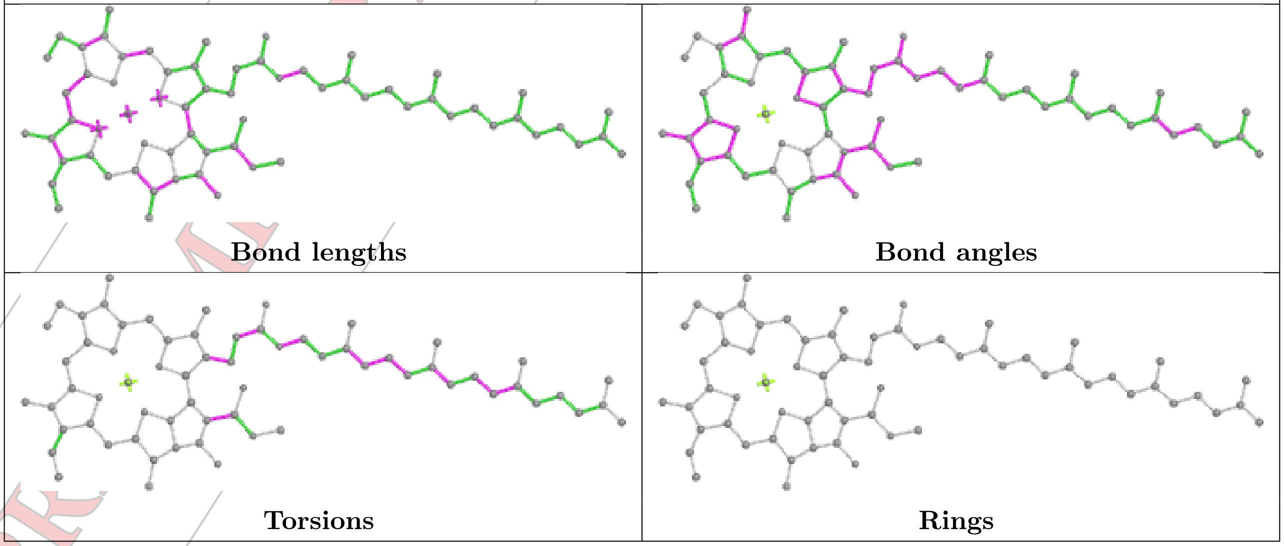

Ligand CLA bA 1206

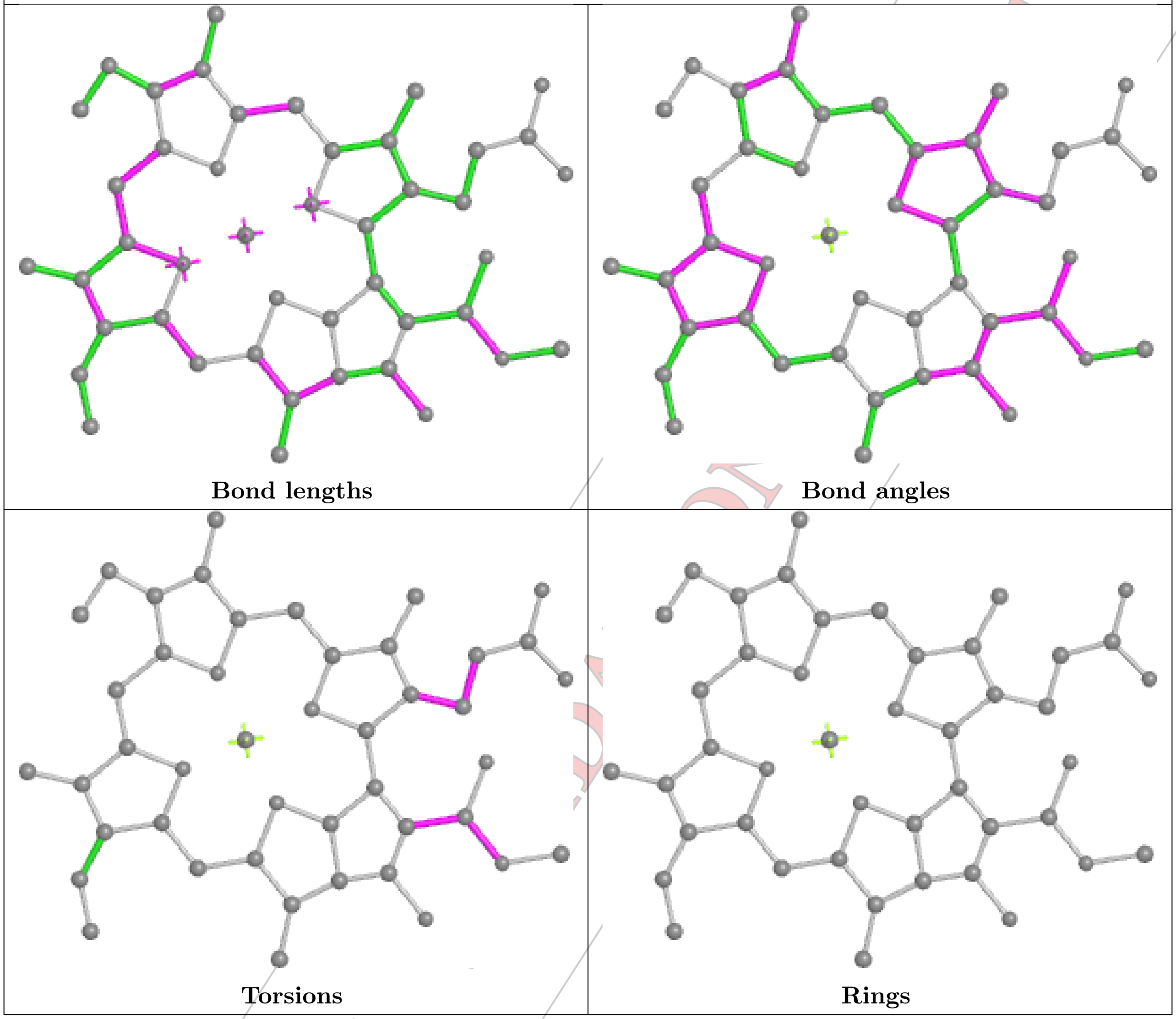

PRELIMINARY

Ligand CLA bA 1207

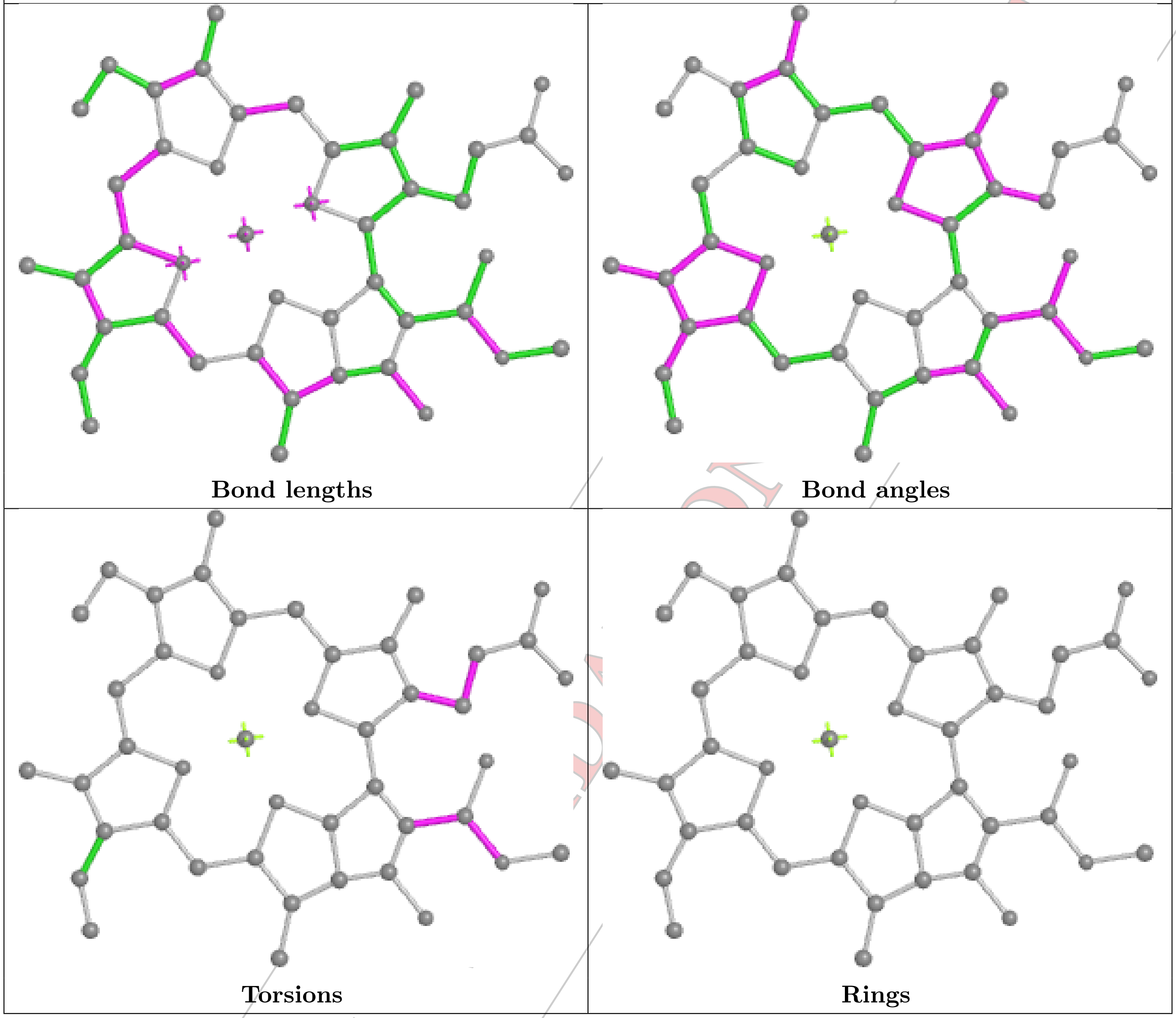

Ligand CLA bA 1208

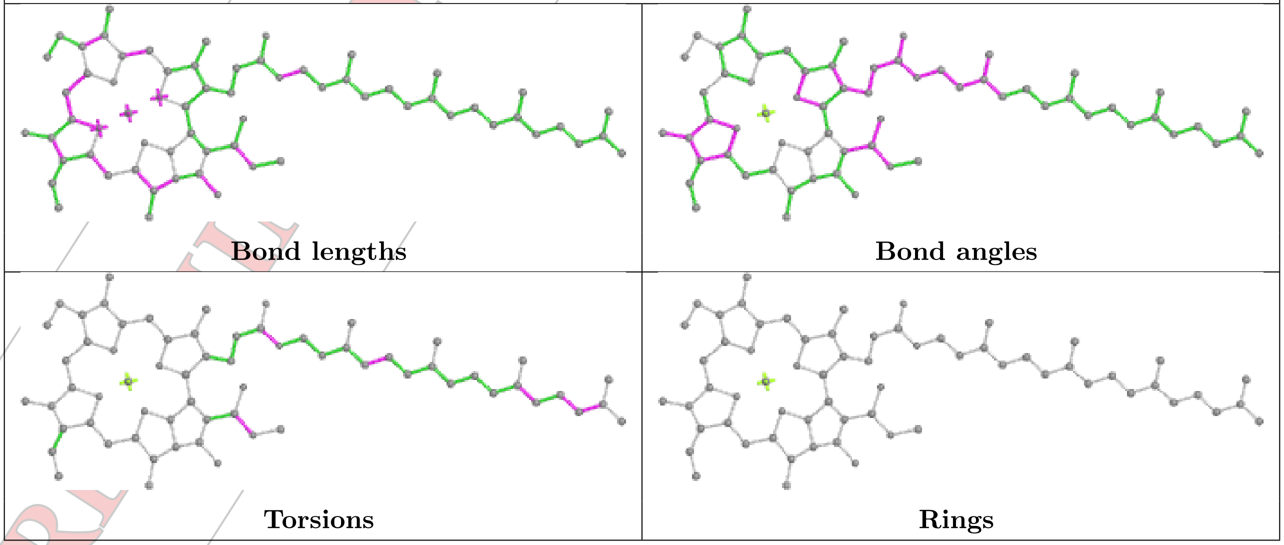

Ligand CLA bA 1209

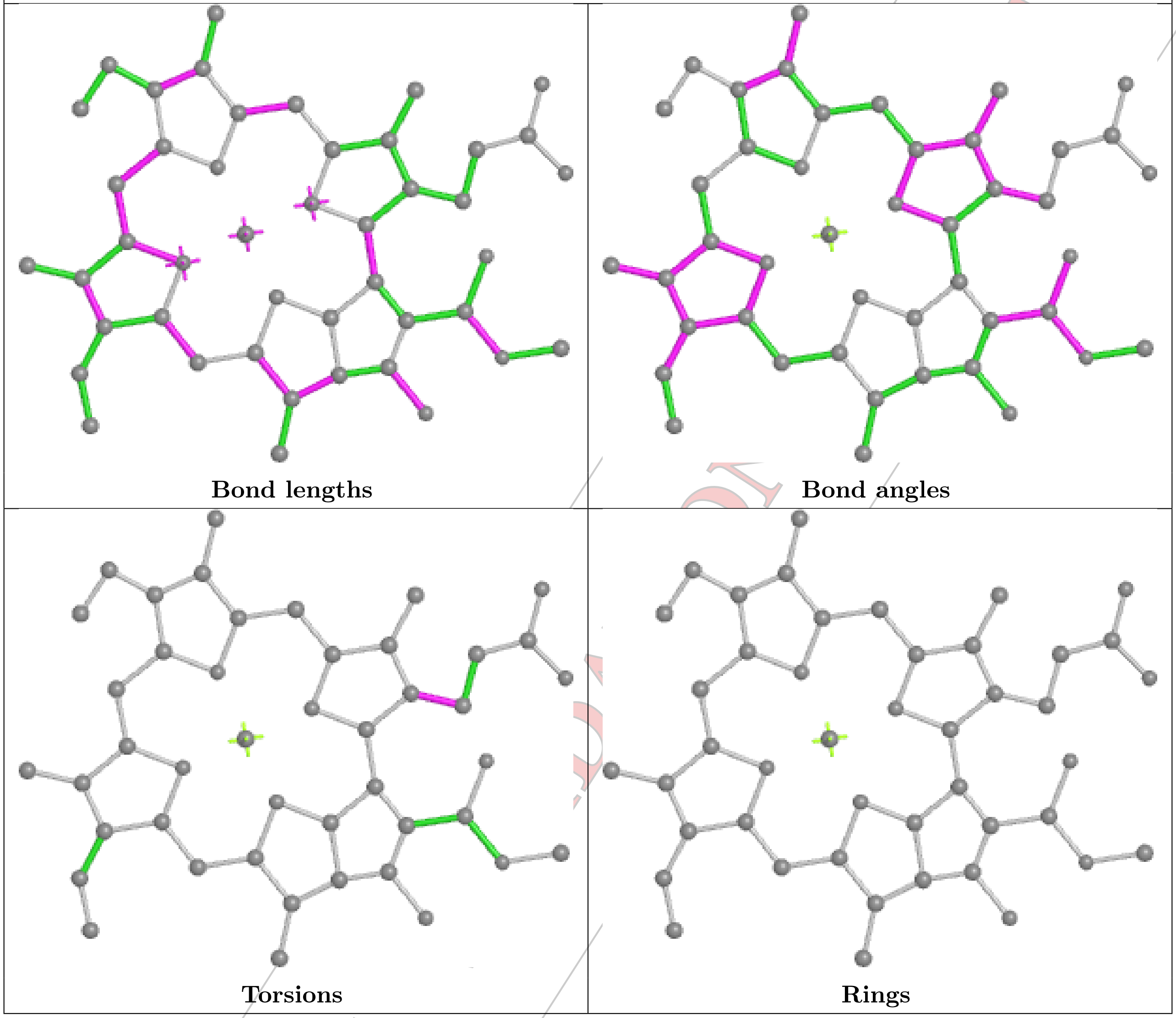

Ligand CLA bA 1210

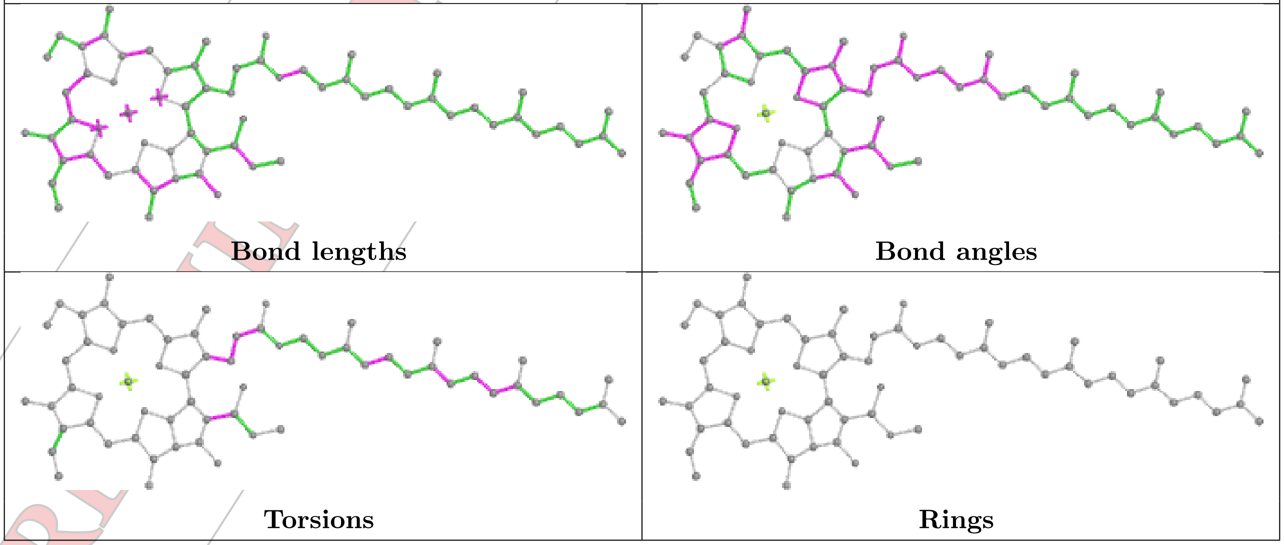

Ligand CLA bA 1211

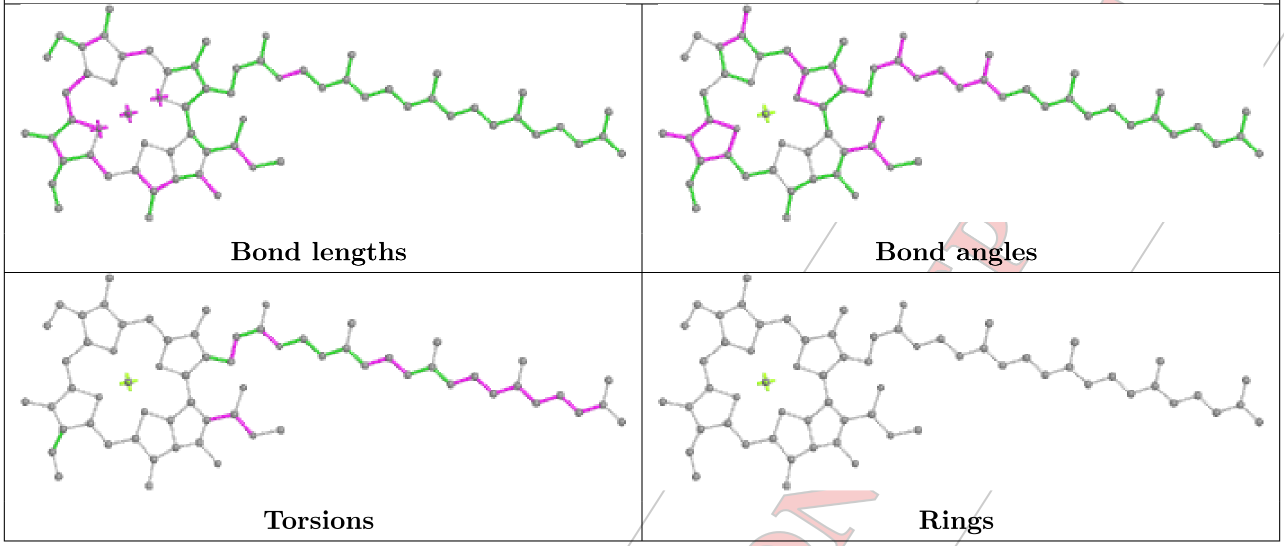

Ligand CLA bA 1212

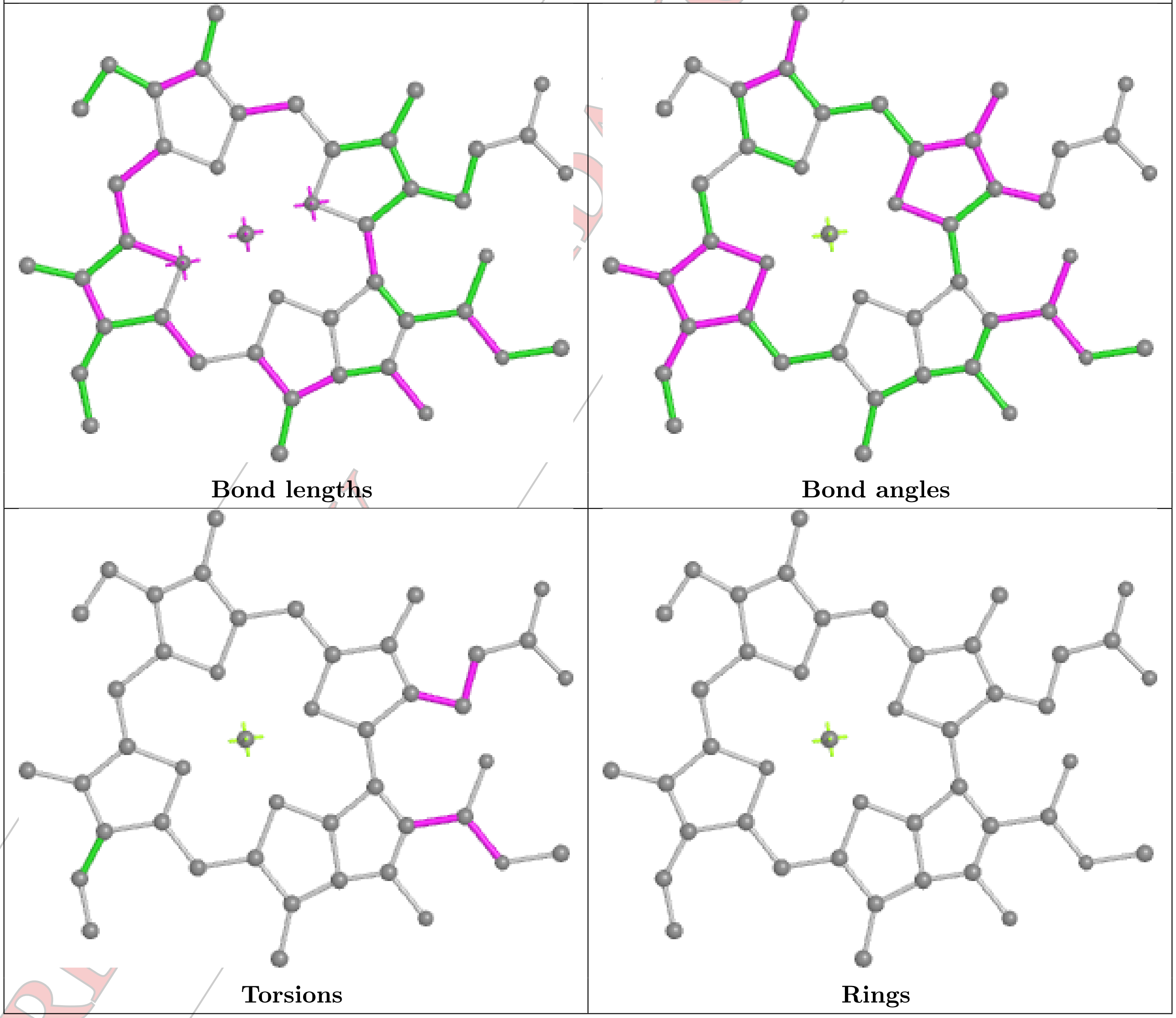

Ligand CLA bA 1213

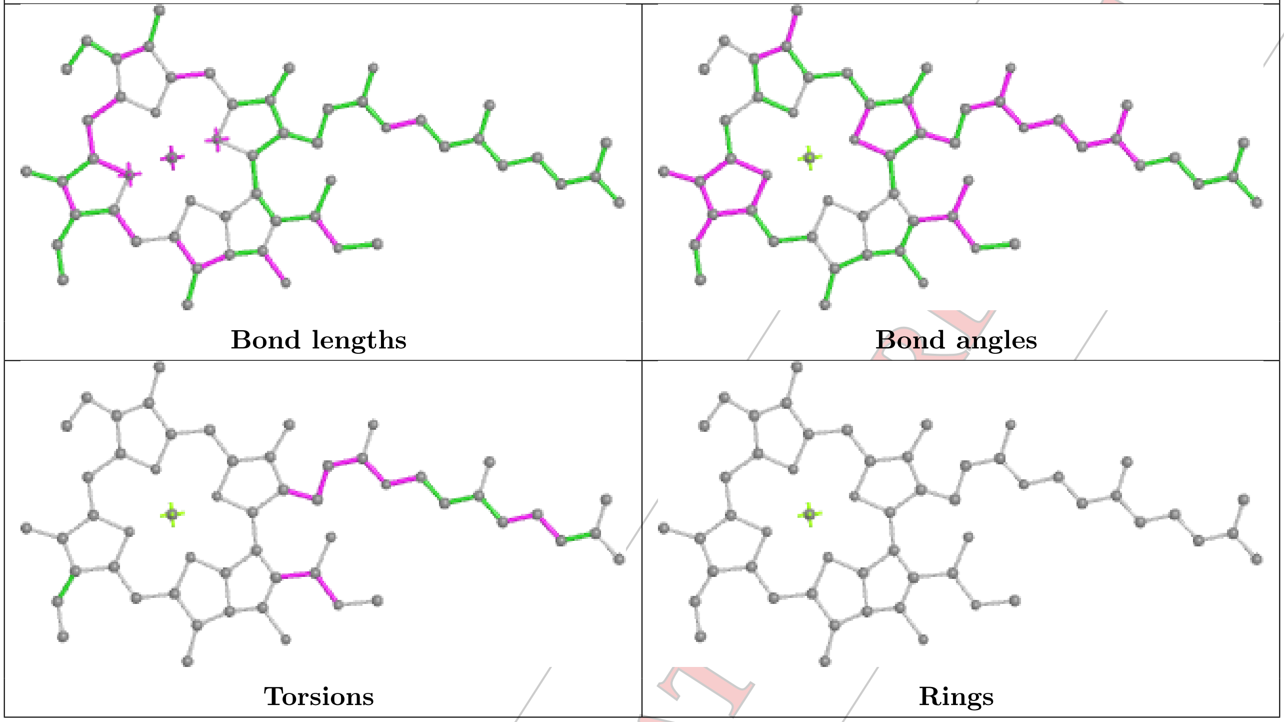

Ligand CLA bA 1214

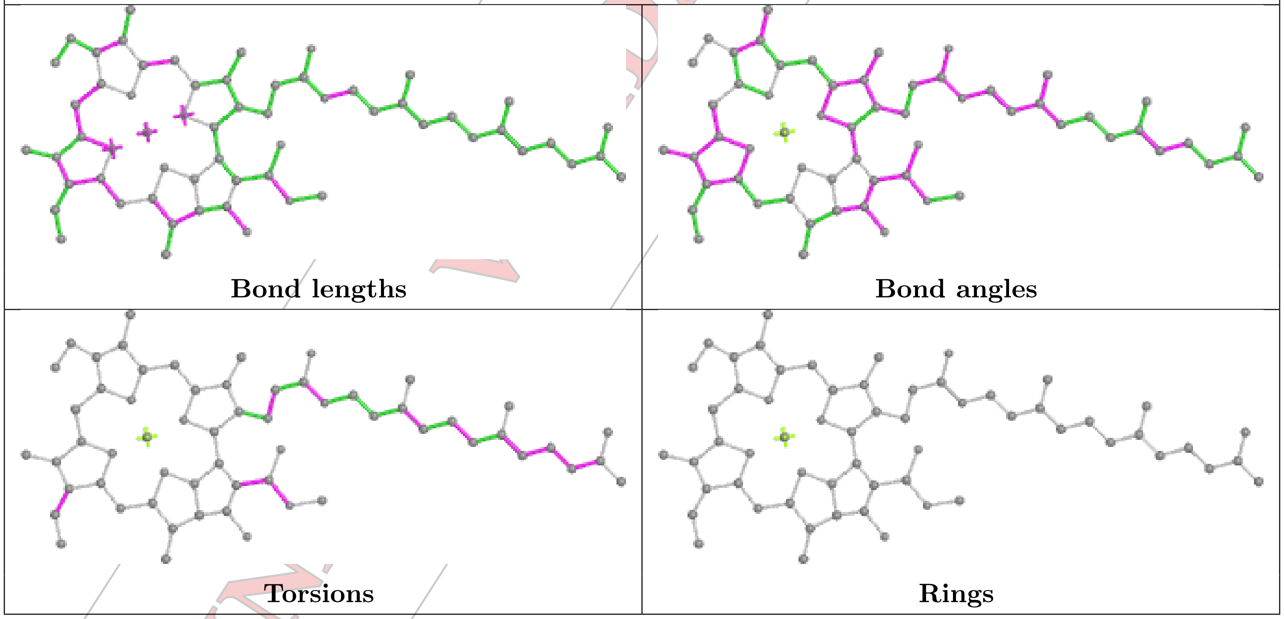

Ligand CLA bA 1215

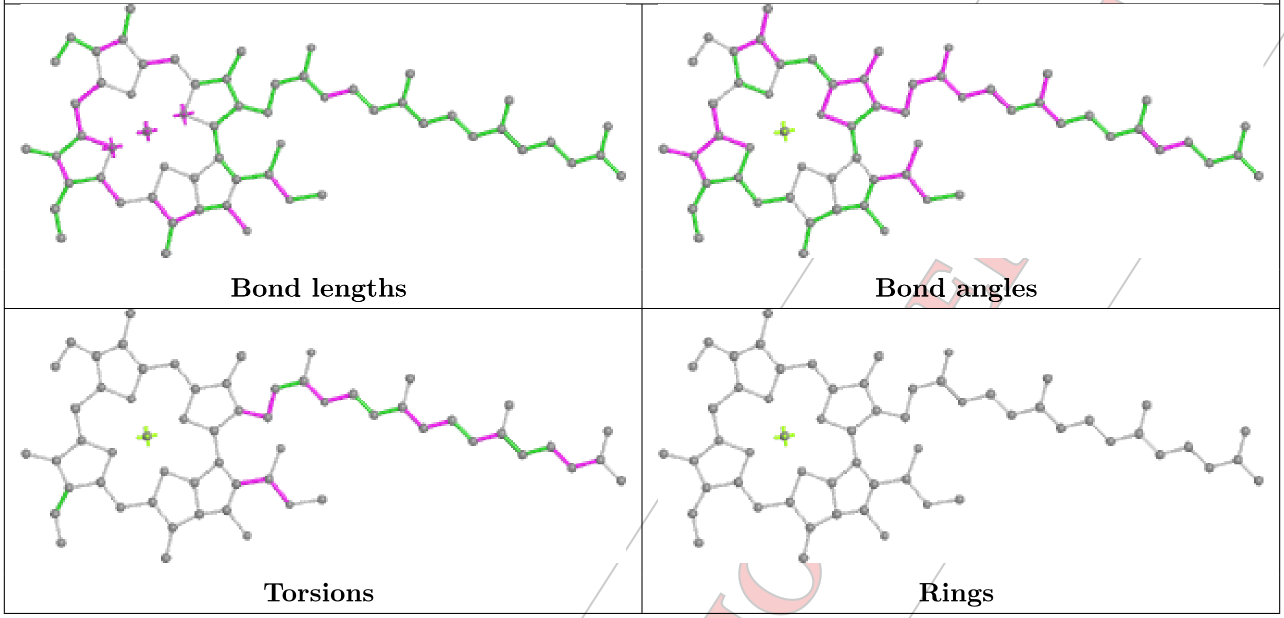

Ligand CLA bA 1216

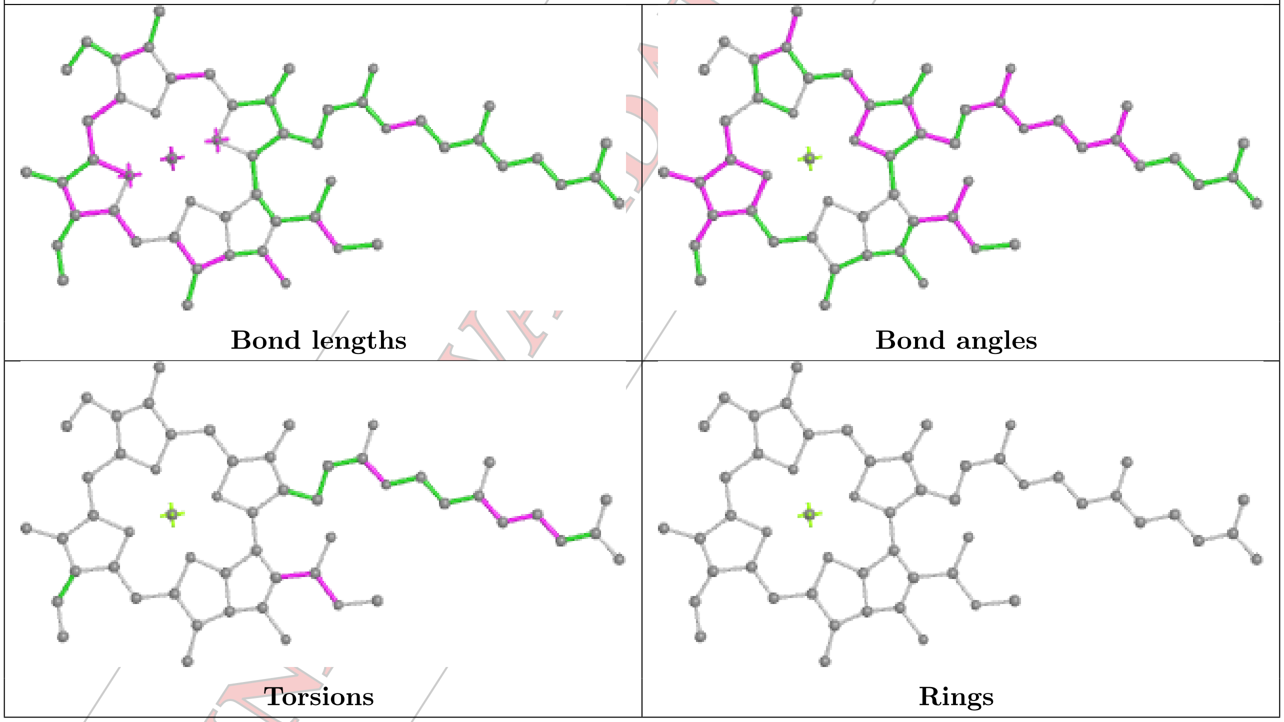

Ligand CLA bA 1217

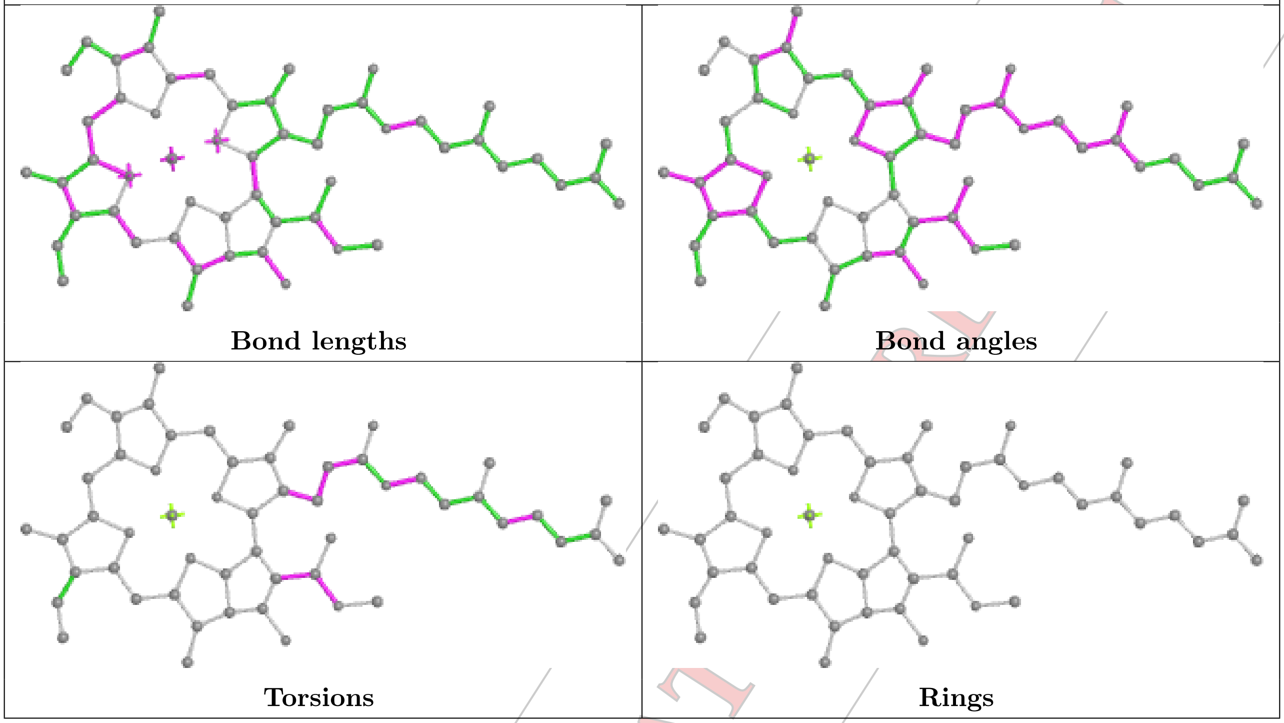

PRELIMINARY VALIDATION

Ligand CLA bA 1218

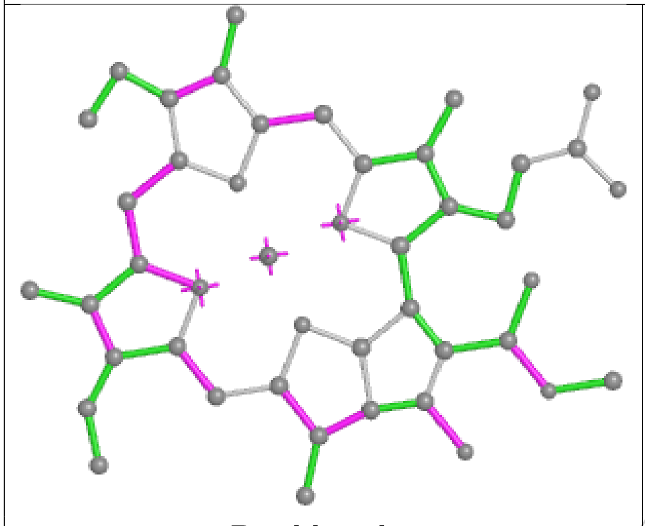

Bond lengths

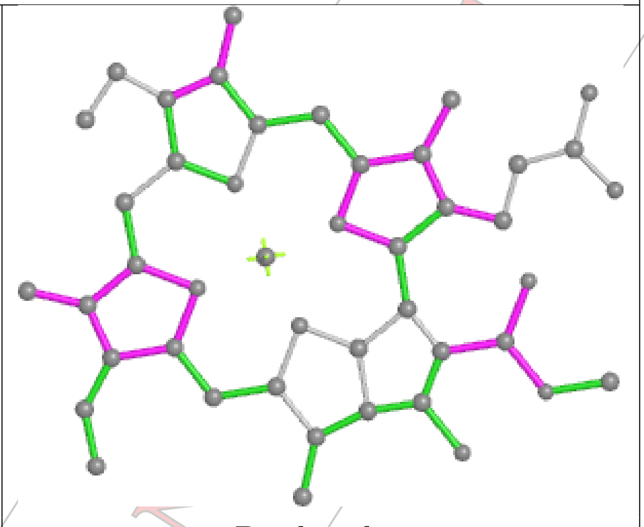

Bond angles

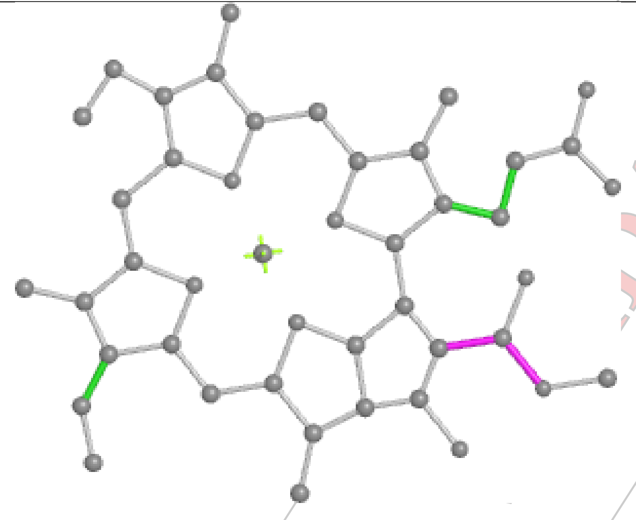

Torsions

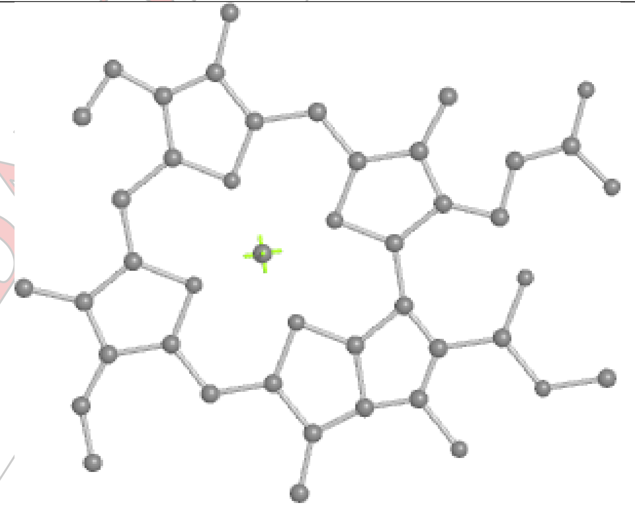

Rings

PRELIMINARY

Ligand CLA bA 1219

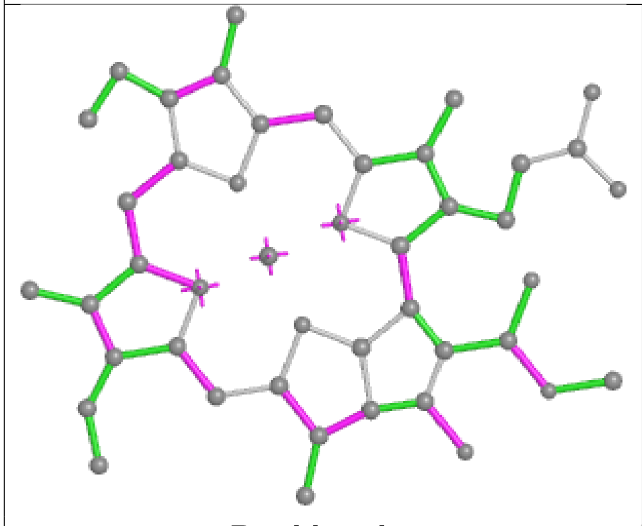

Bond lengths

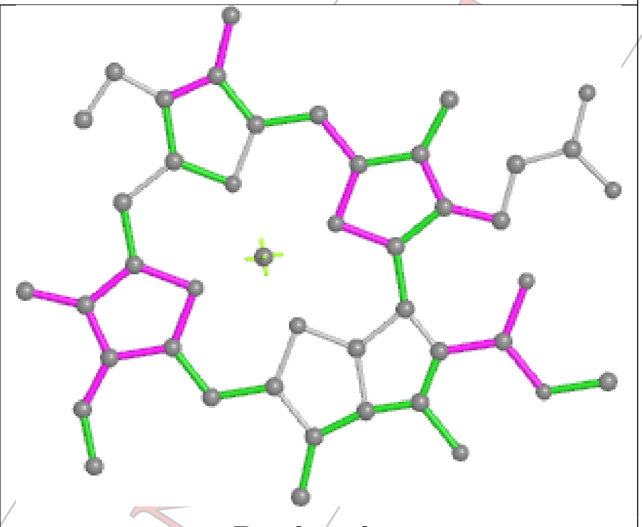

Bond angles

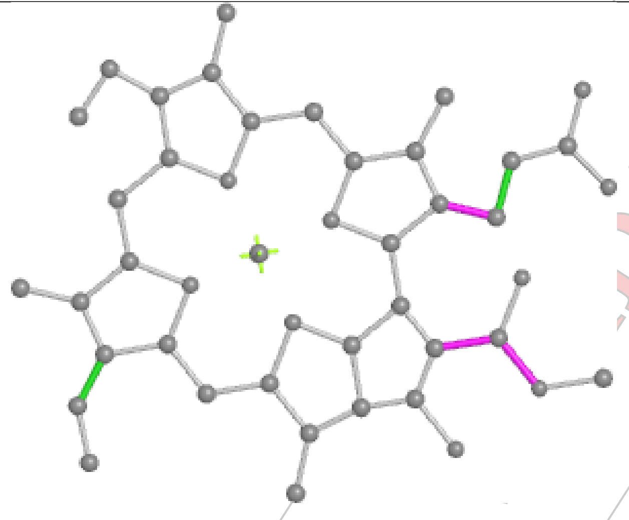

Torsions

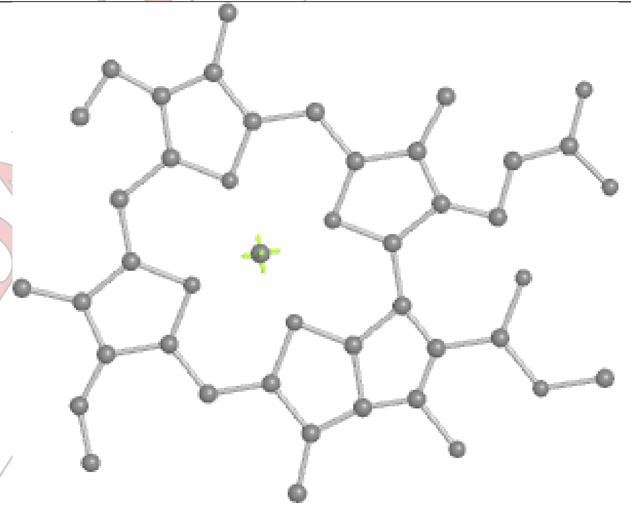

Rings

PRELIMINARY

Ligand CLA bA 1220

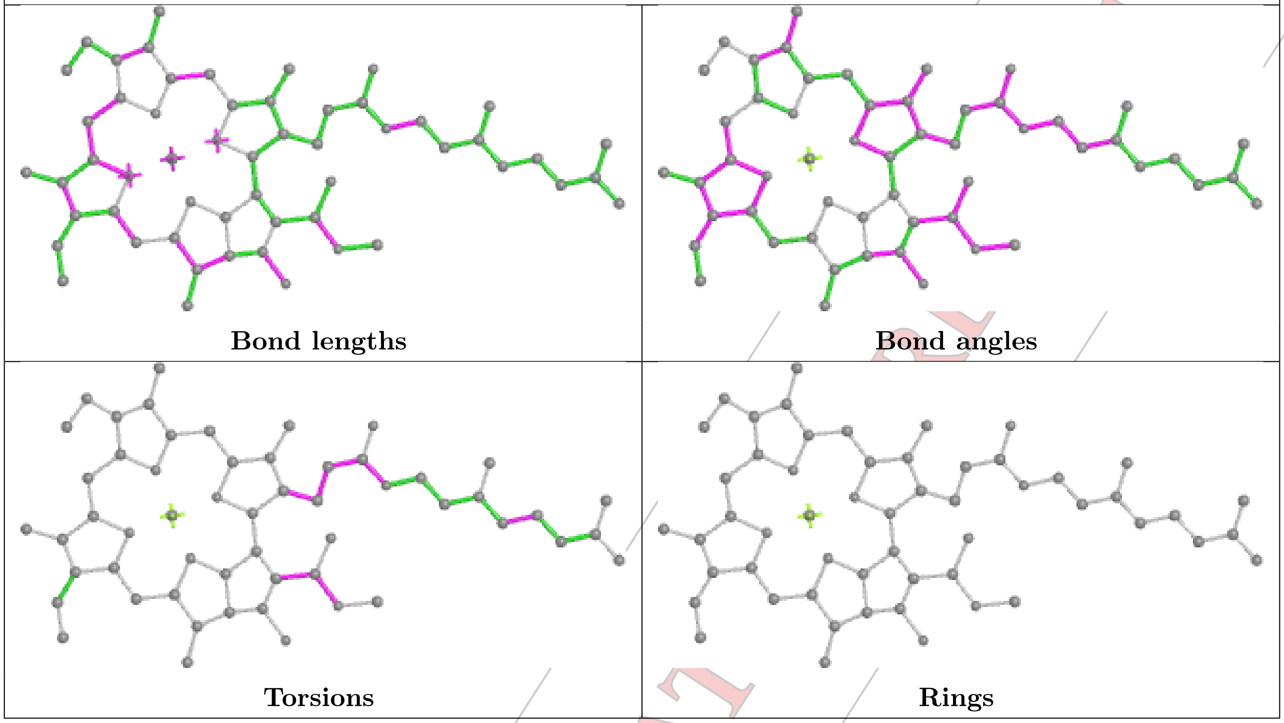

Ligand CLA bA 1221

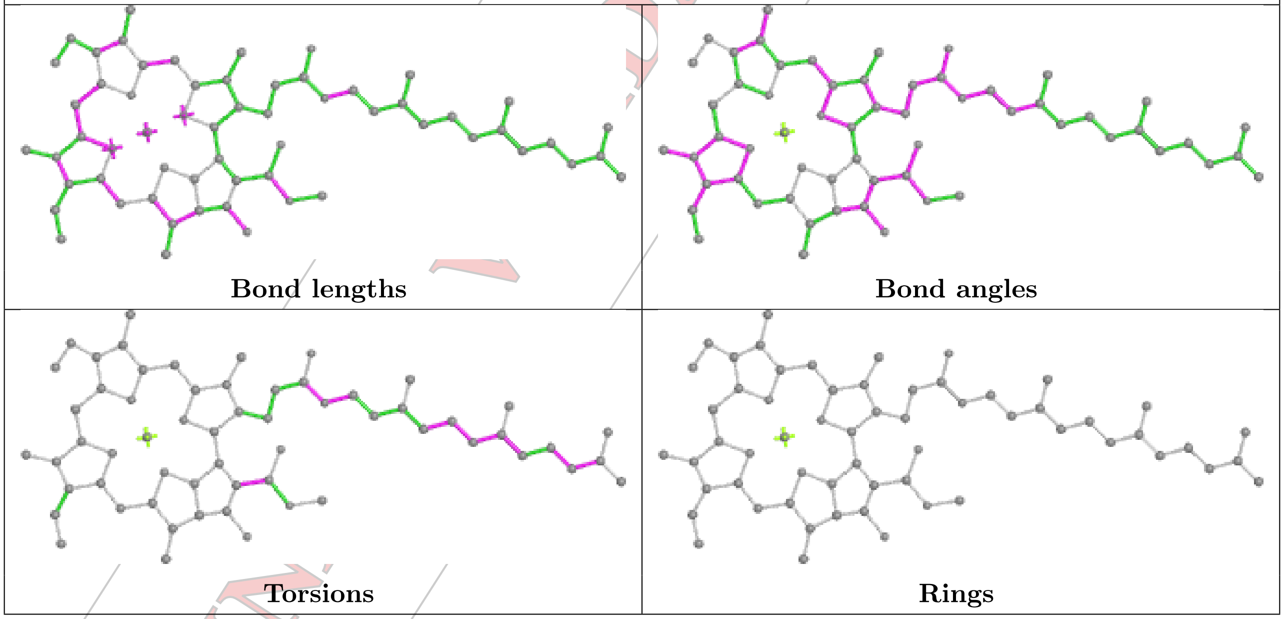

Ligand CLA bA 1222

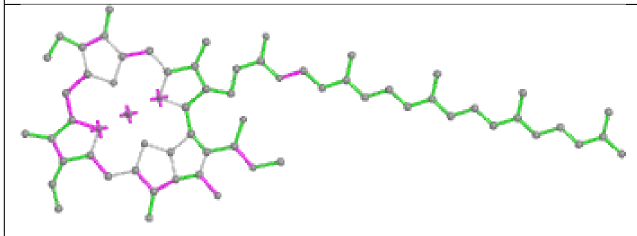
Bond lengths

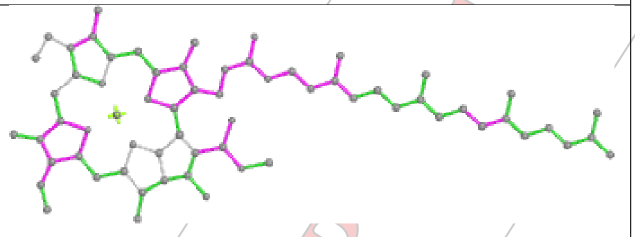
Bond angles

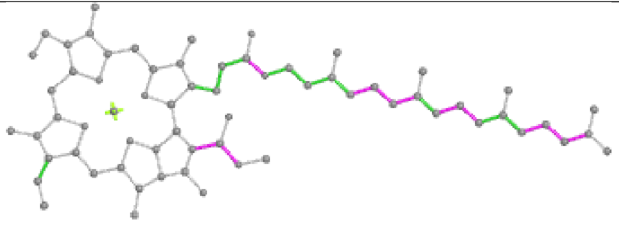
Torsions

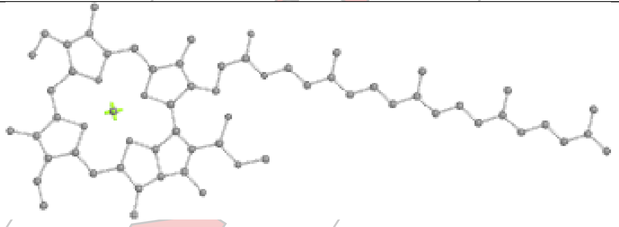
Rings

Ligand CLA bA 1223

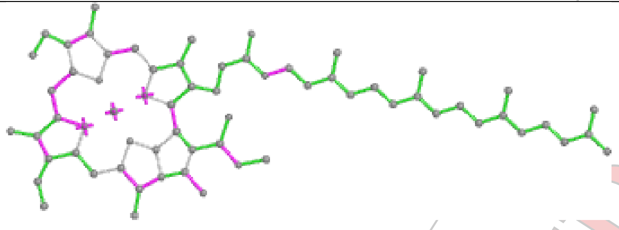
Bond lengths

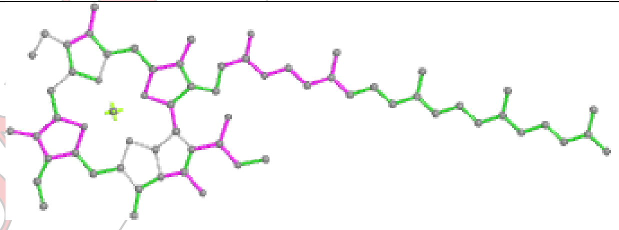
Bond angles

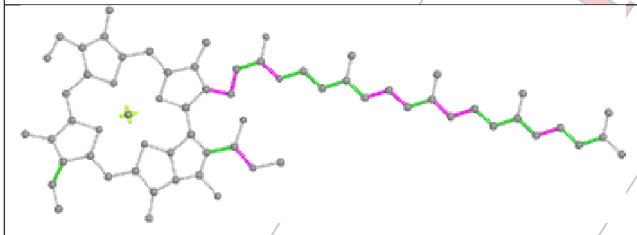
Torsions

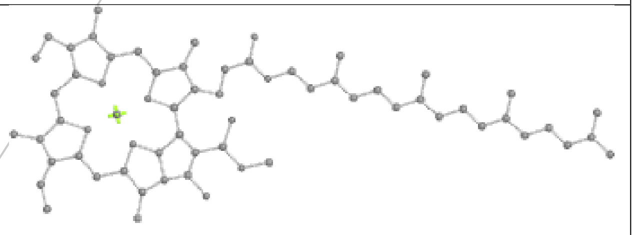
Rings

Ligand CLA bA 1224

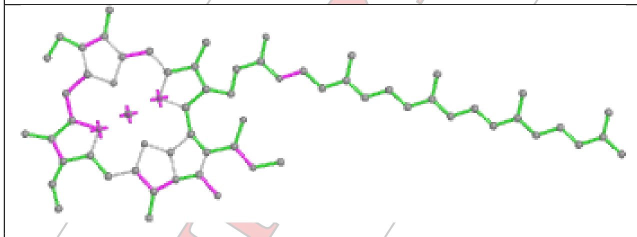
Bond lengths

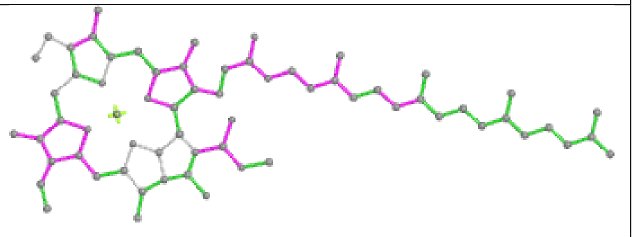
Bond angles

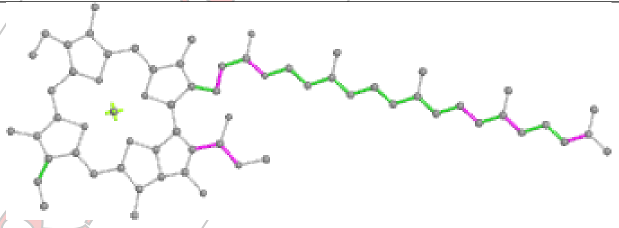
Torsions

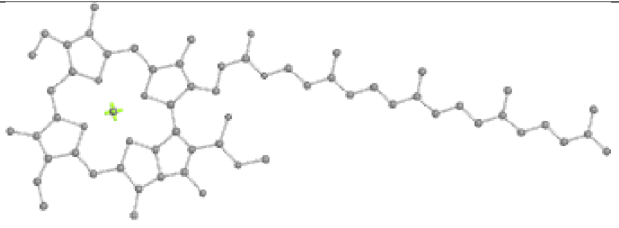
Rings

Ligand CLA bA 1225

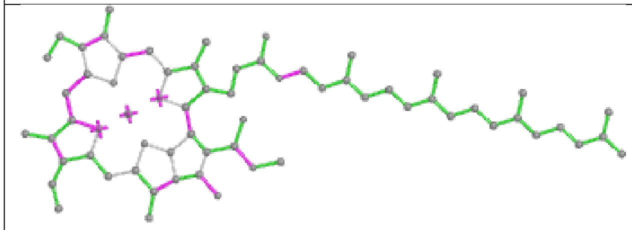
Bond lengths

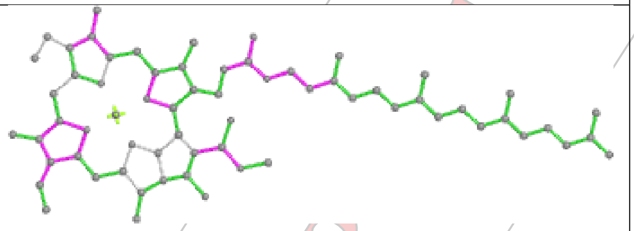
Bond angles

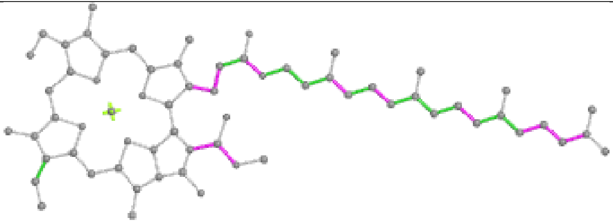
Torsions

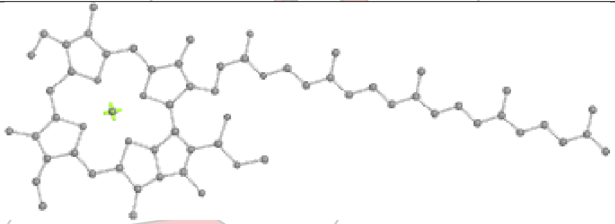
Rings

Ligand CLA bA 1226

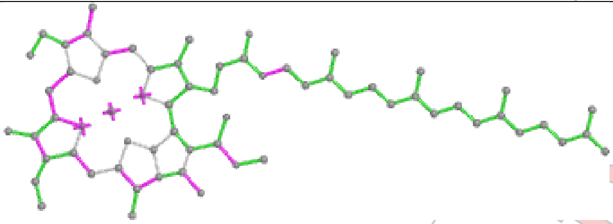
Bond lengths

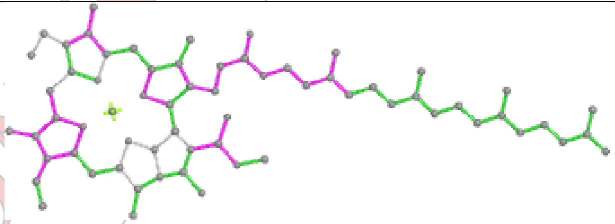
Bond angles

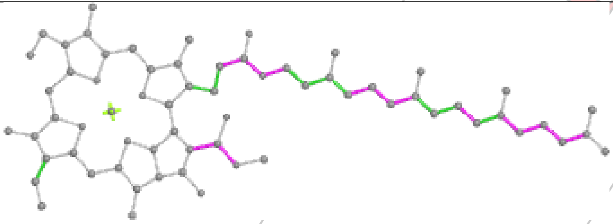
Torsions

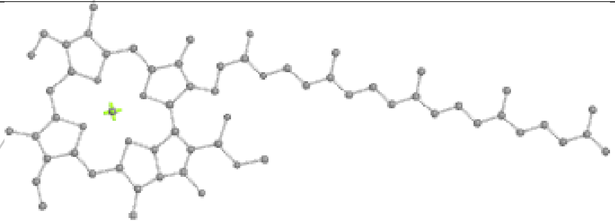
Rings

PRELIMINARY

Ligand CLA bA 1227

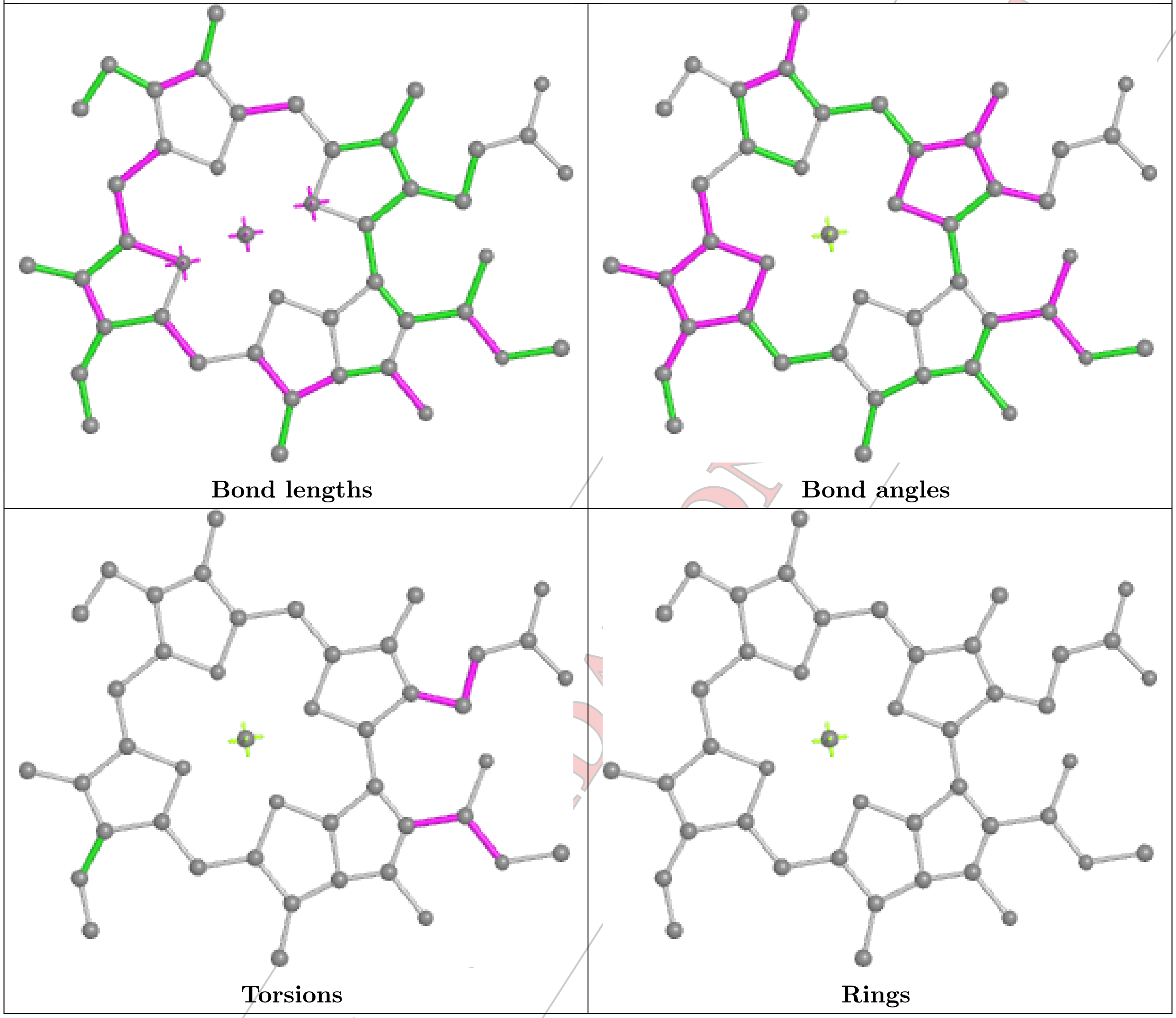

Bond lengths / Bond angles / Torsions / Rings

PRELIMINARY

Ligand CLA bA 1228

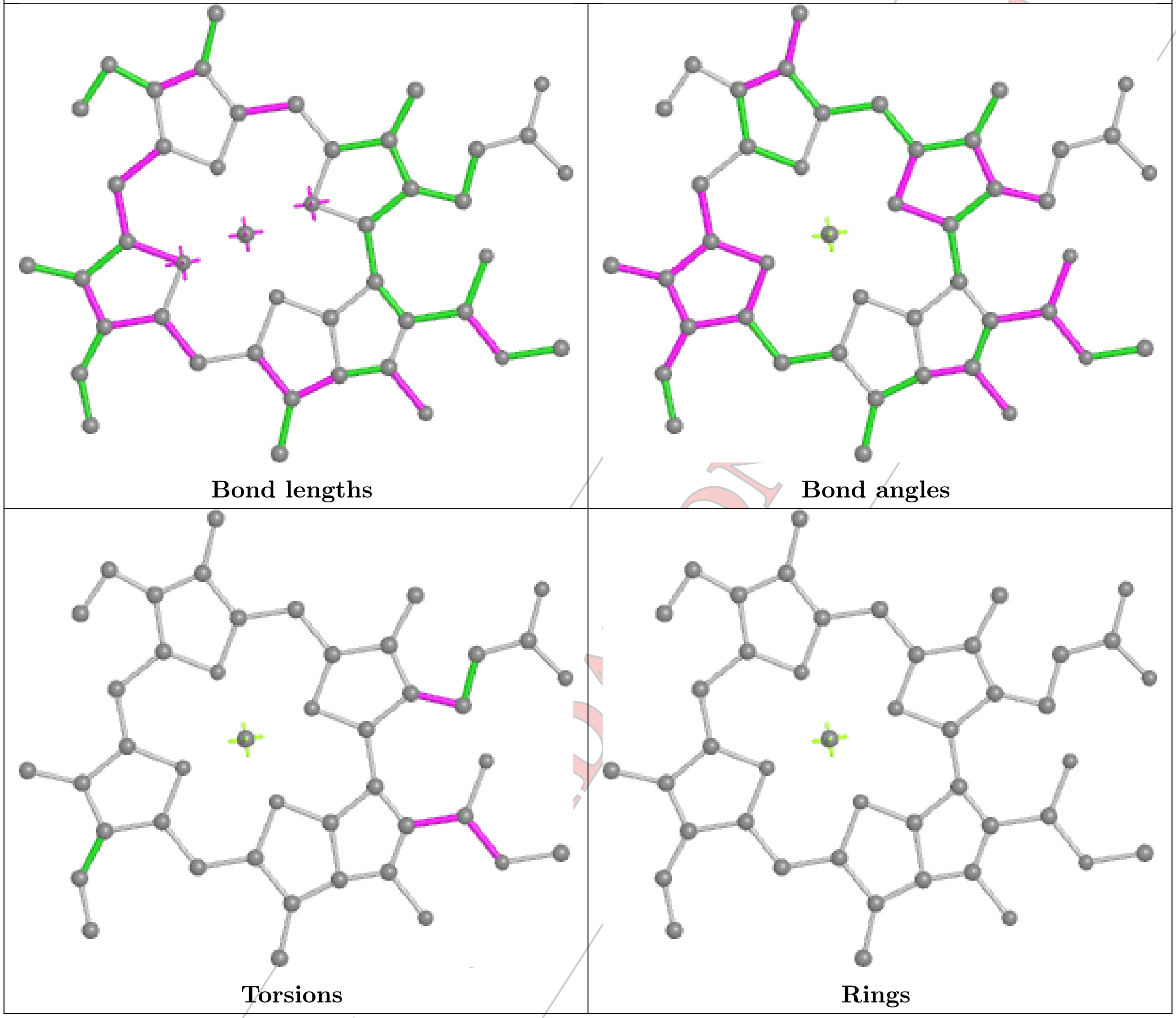

Ligand CLA bA 1229

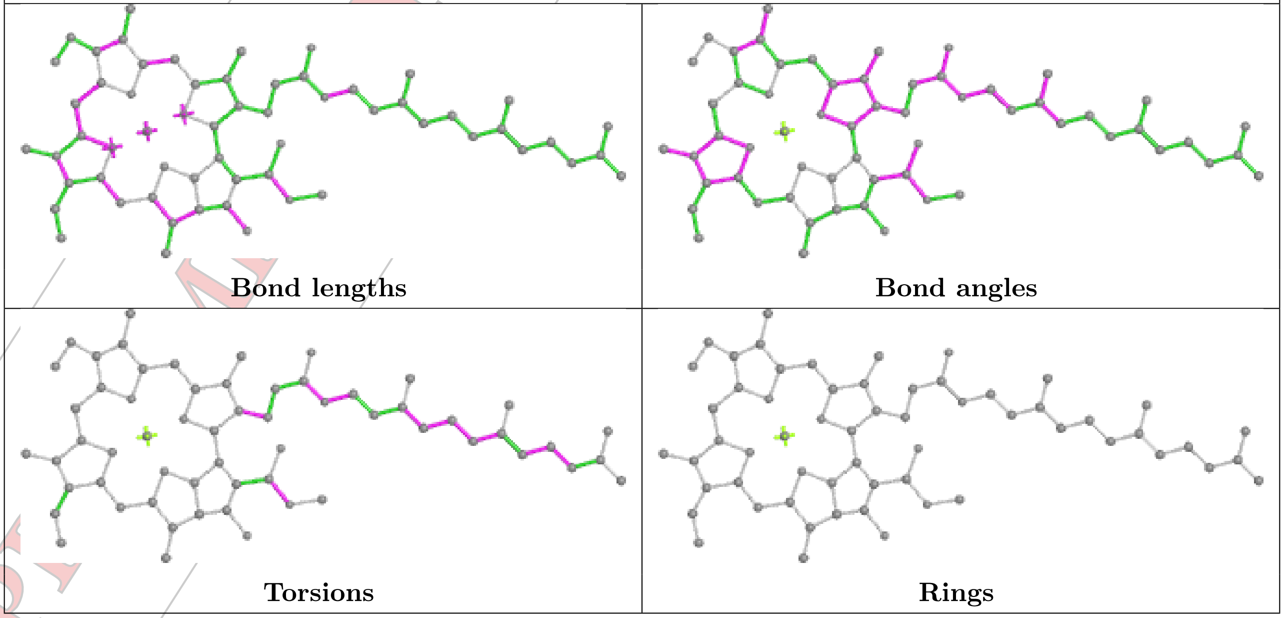

Ligand CLA bA 1231	
Bond lengths	Bond angles
Torsions	Rings

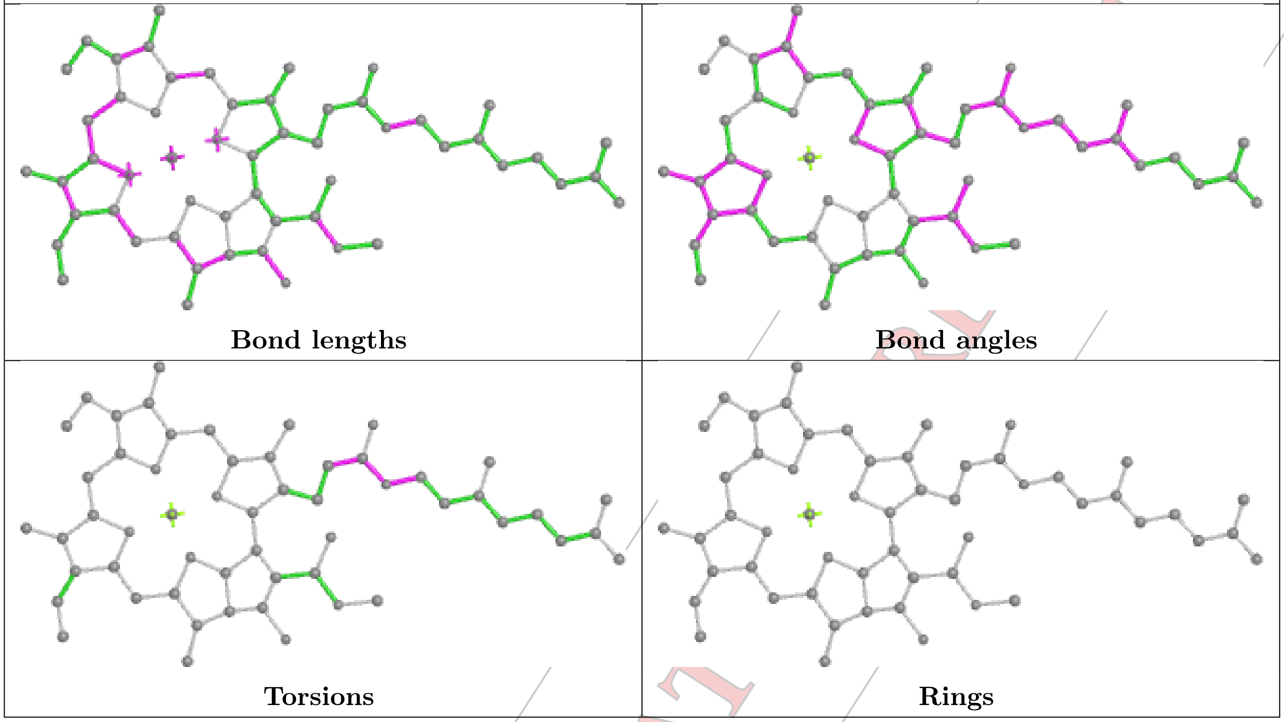

PRELIMINARY VALIDATION

Ligand CLA bA 1232

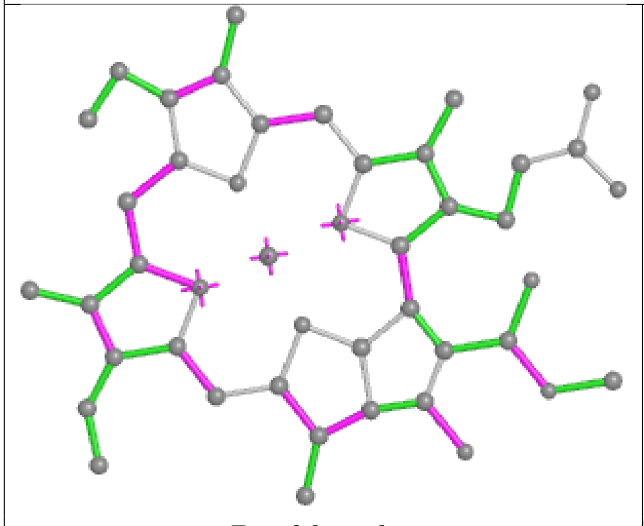

Bond lengths

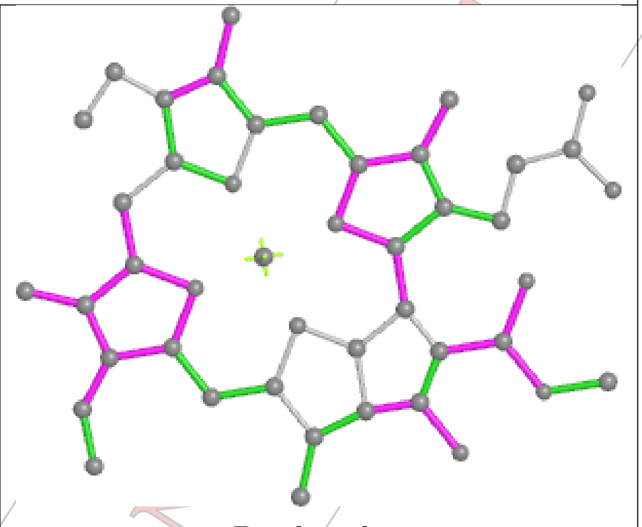

Bond angles

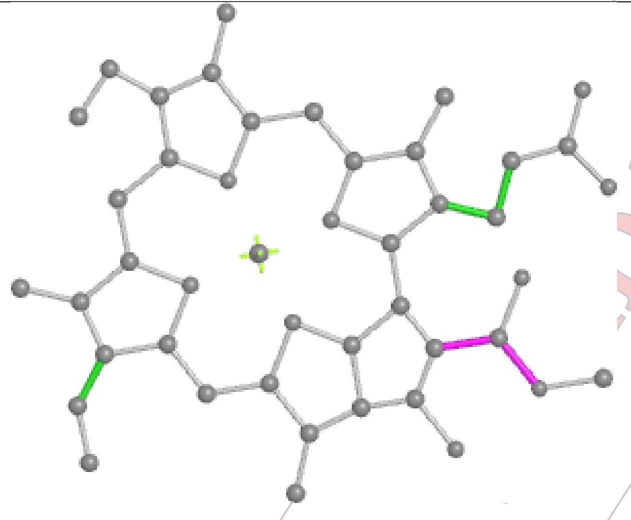

Torsions

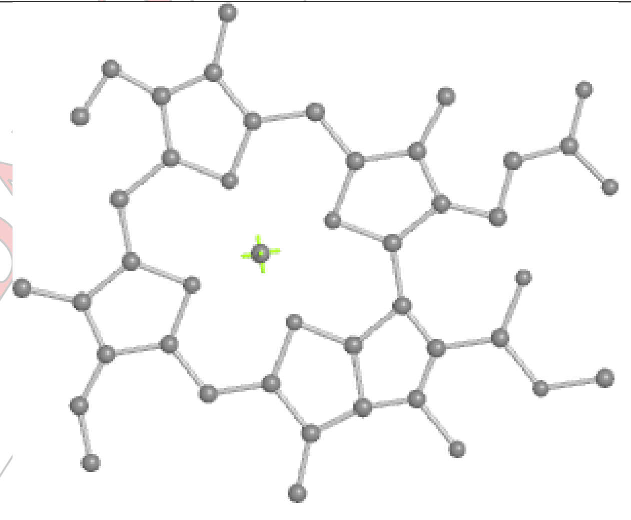

Rings

PRELIMINARY

Ligand CLA bA 1233

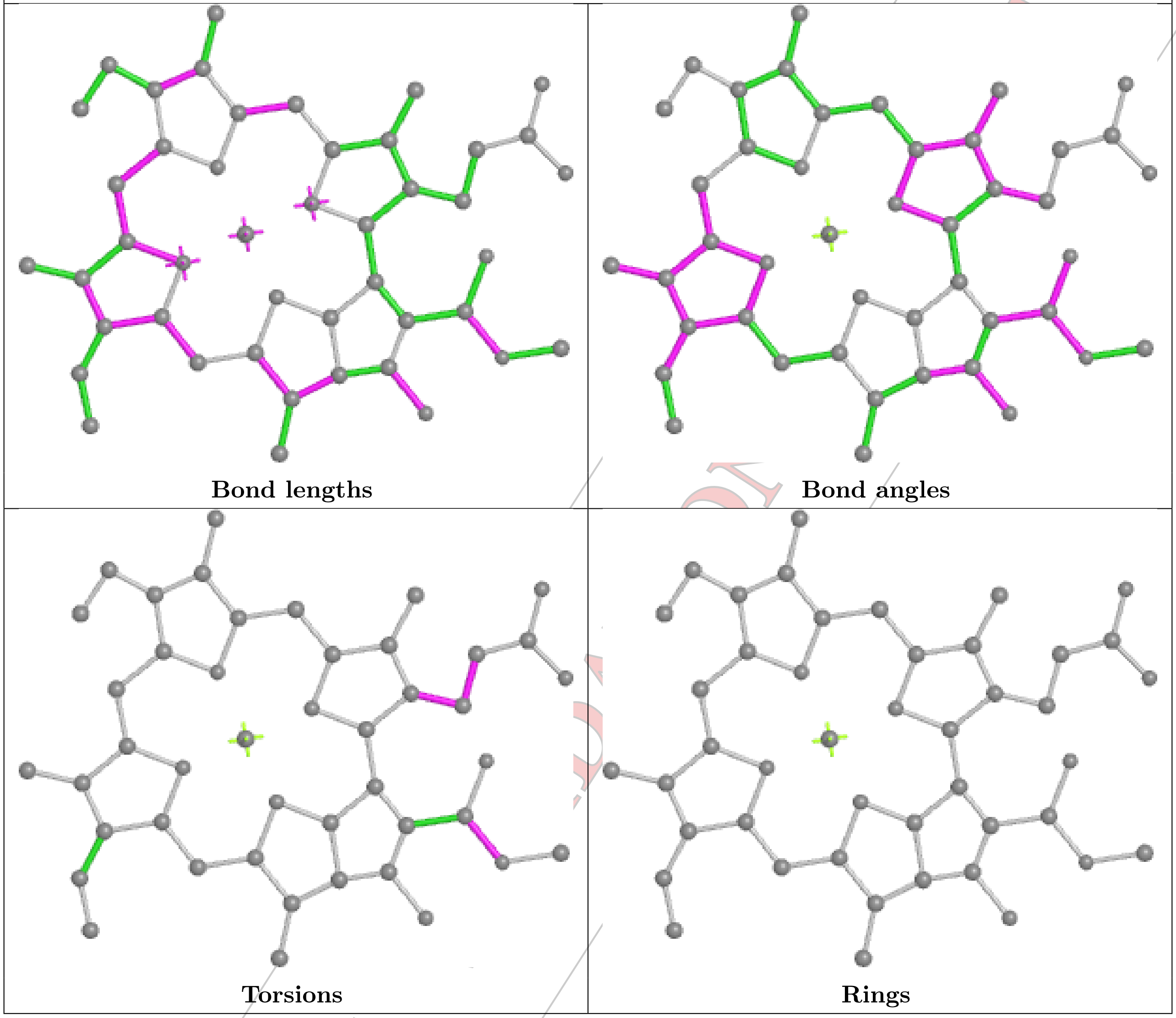

PRELIMINARY

Ligand CLA bA 1234

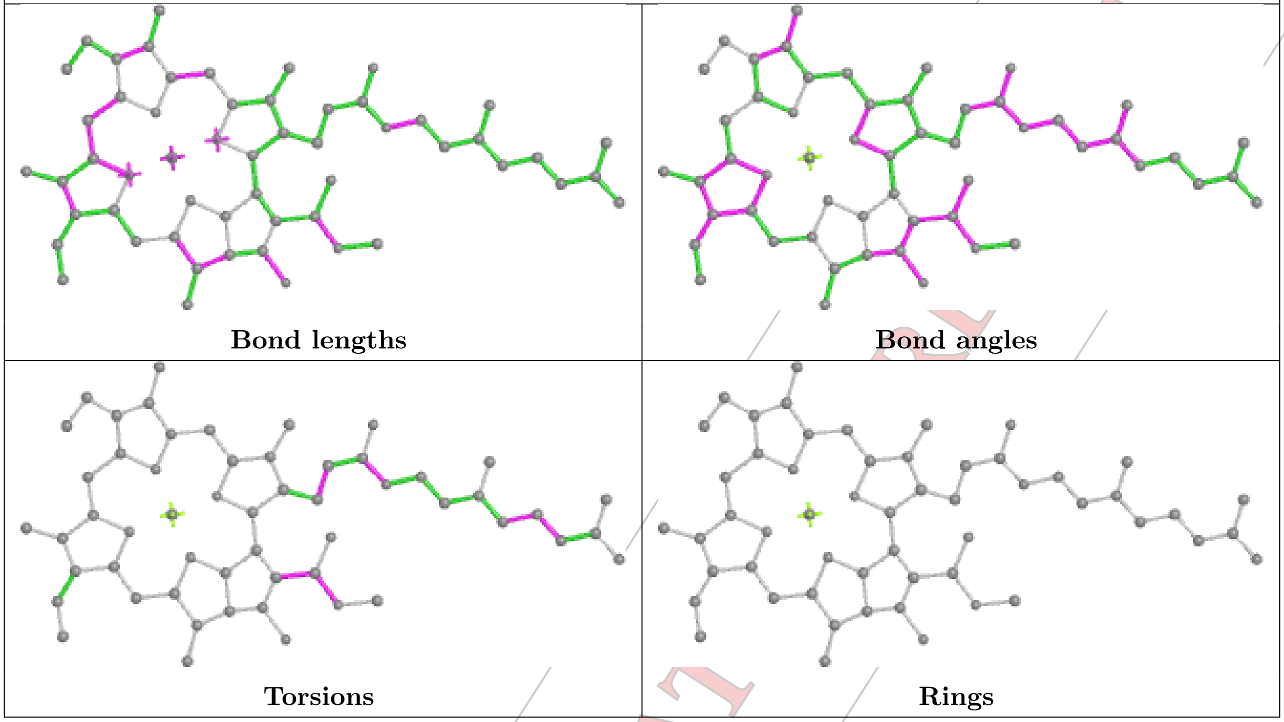

Ligand CLA bA 1235

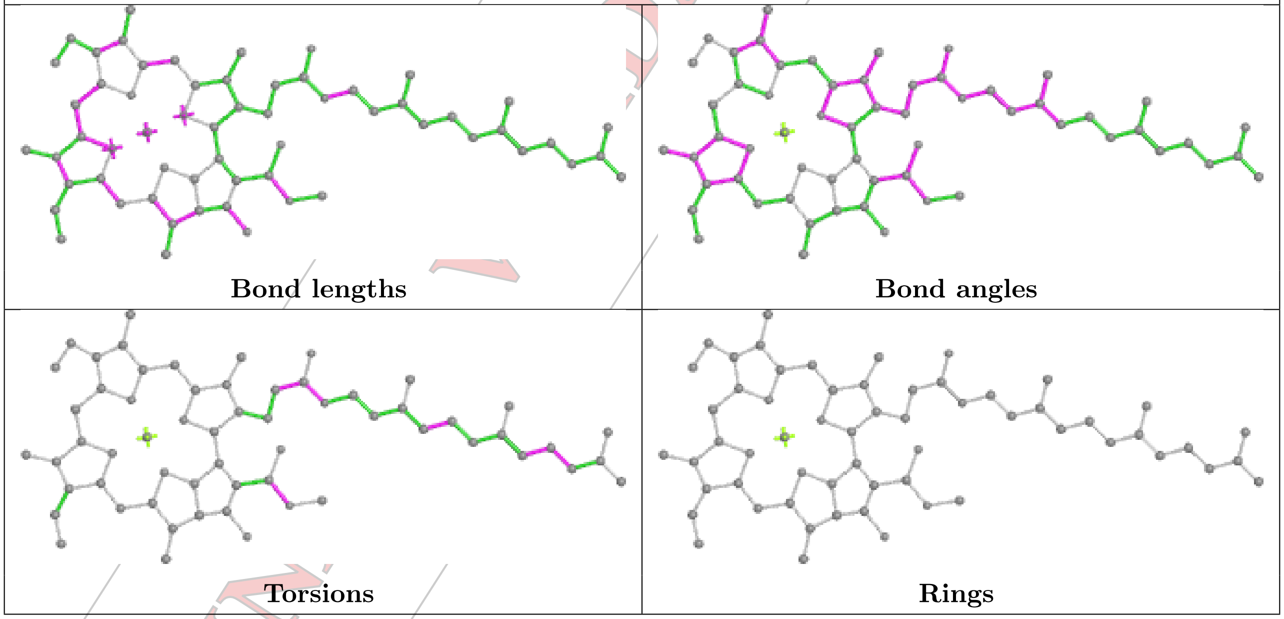

Ligand CLA bA 1236

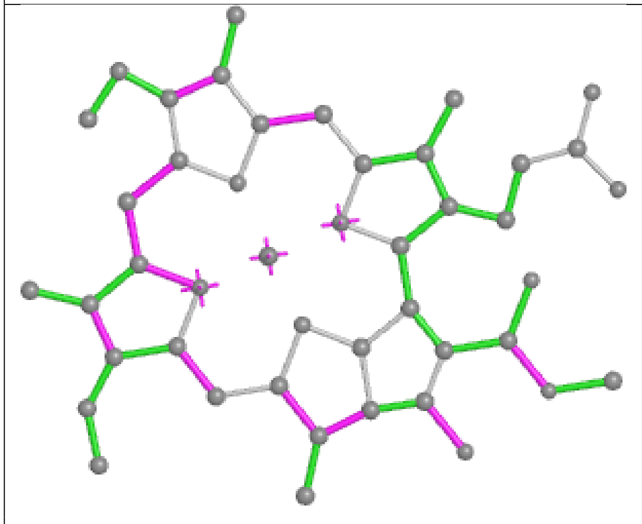

Bond lengths

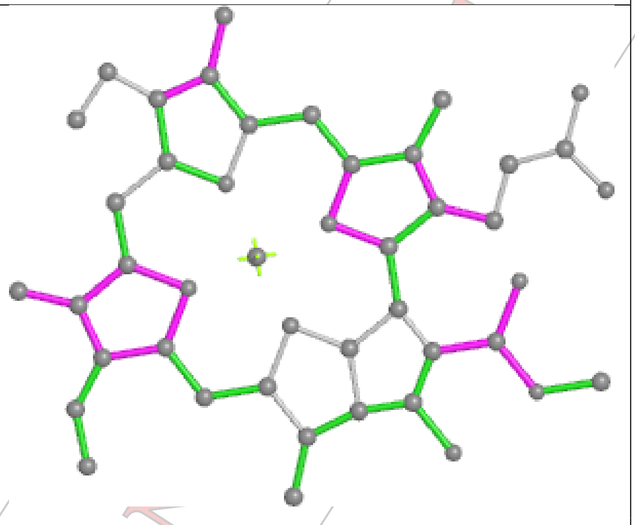

Bond angles

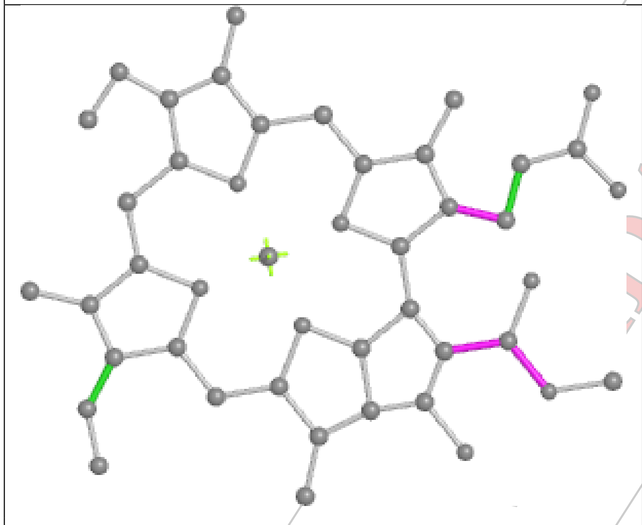

Torsions

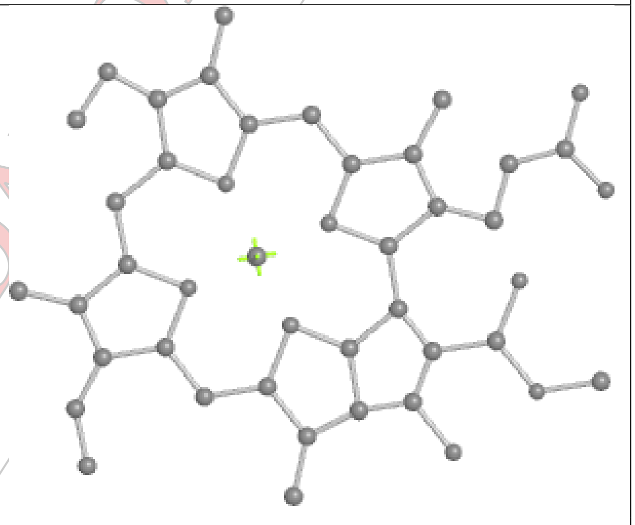

Rings

Ligand CLA bA 1238

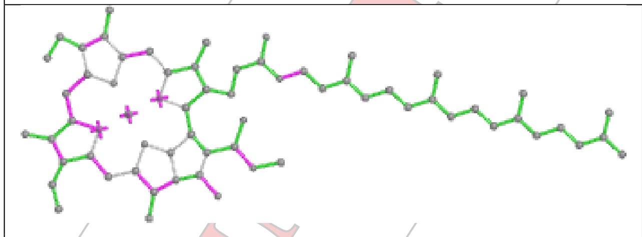

Bond lengths

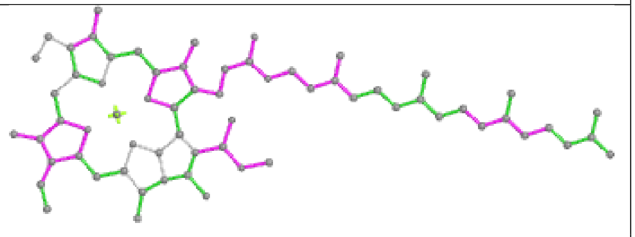

Bond angles

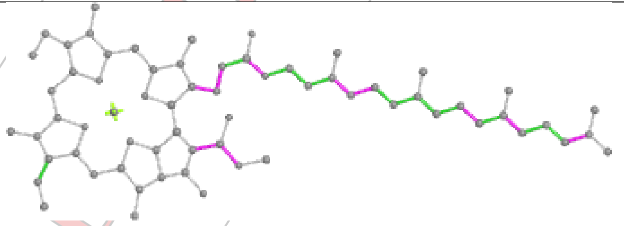

Torsions

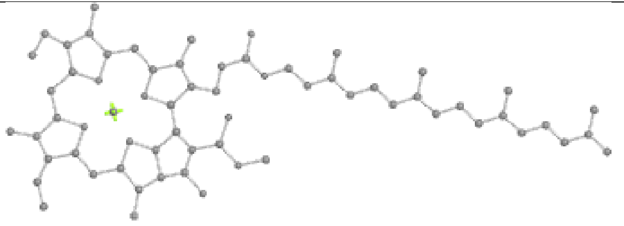

Rings

Ligand CLA bA 1239

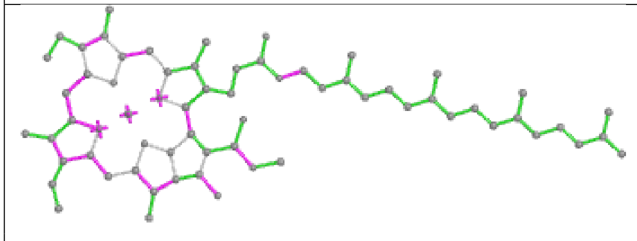

Bond lengths

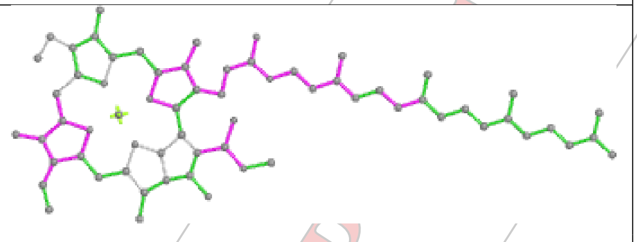

Bond angles

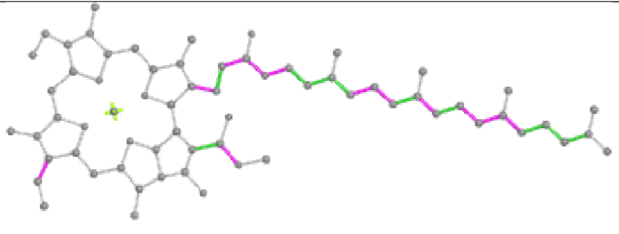

Torsions

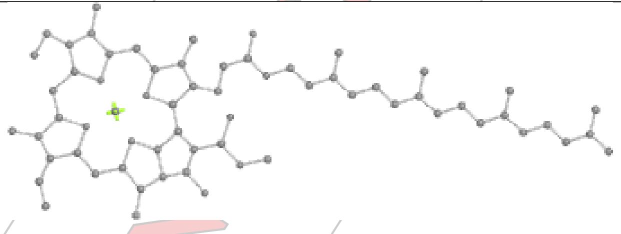

Rings

Ligand PQN bA 2002

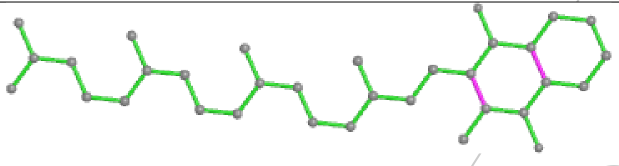

Bond lengths

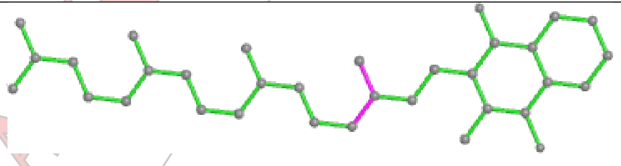

Bond angles

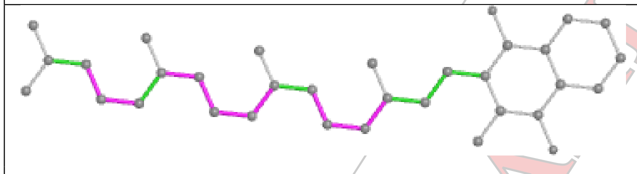

Torsions

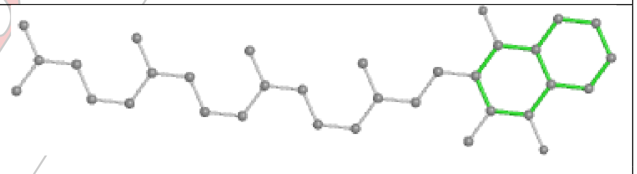

Rings

Ligand BCR bA 4004

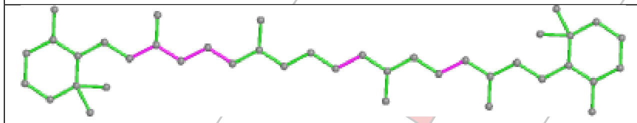

Bond lengths

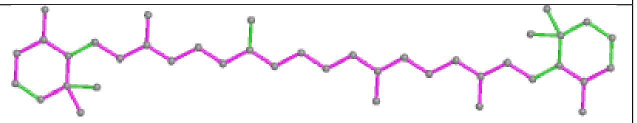

Bond angles

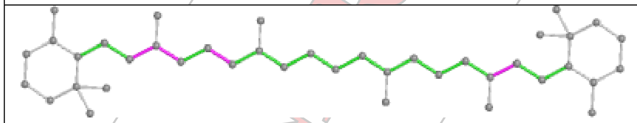

Torsions

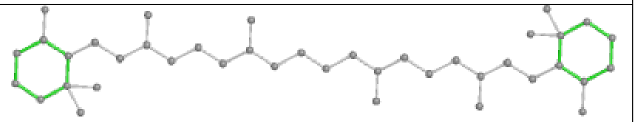

Rings

Ligand BCR bA 4005

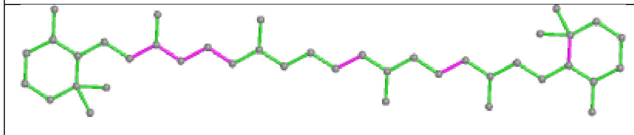

Bond lengths

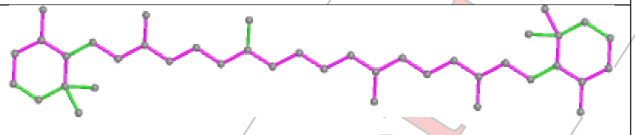

Bond angles

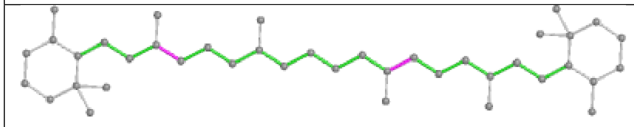

Torsions

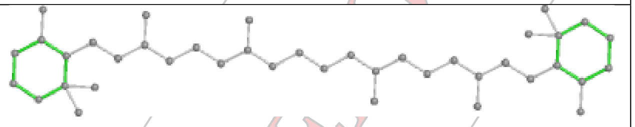

Rings

Ligand BCR bA 4006

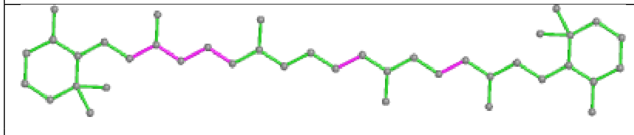

Bond lengths

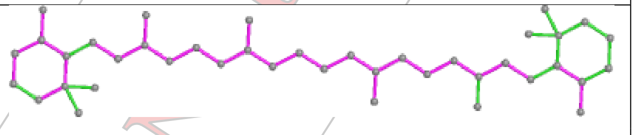

Bond angles

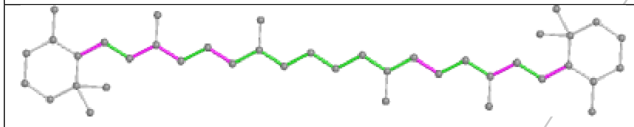

Torsions

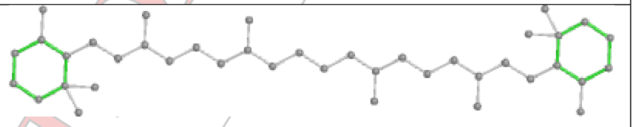

Rings

Ligand BCR bA 4009

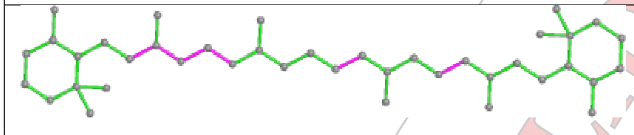

Bond lengths

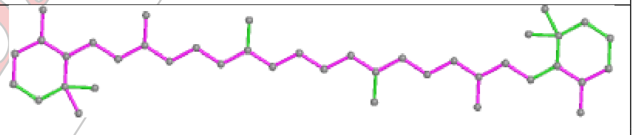

Bond angles

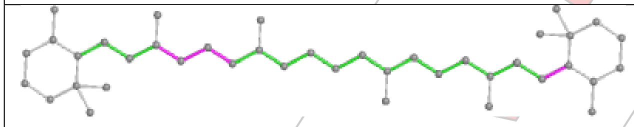

Torsions

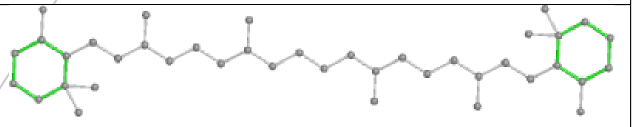

Rings

Ligand BCR bA 4010

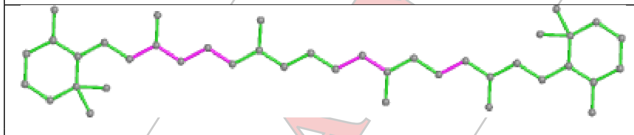

Bond lengths

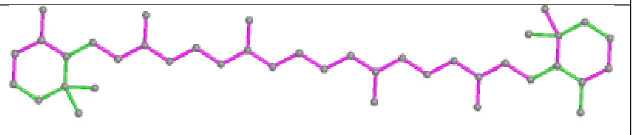

Bond angles

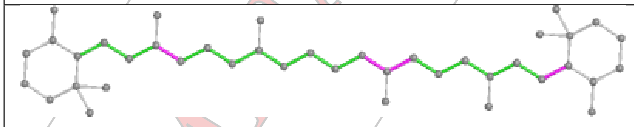

Torsions

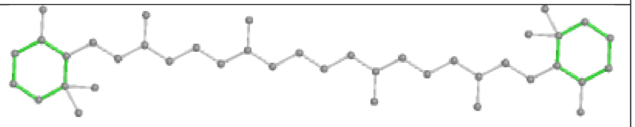

Rings

Ligand BCR bA 4012

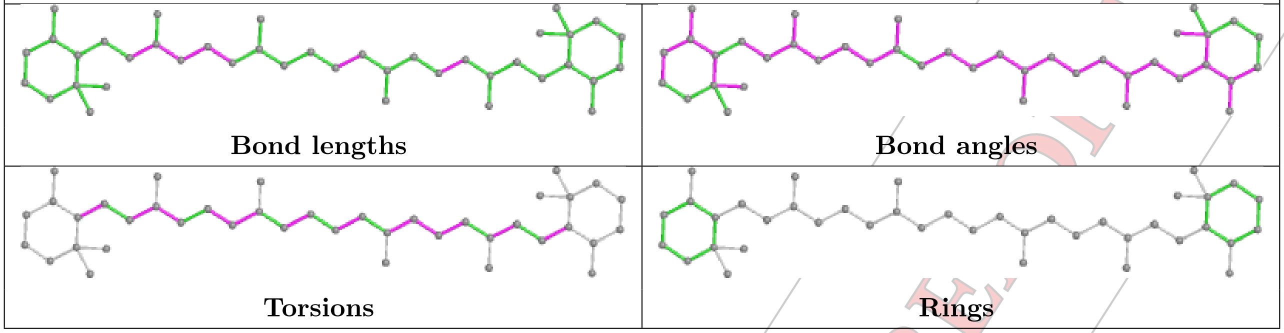

Ligand BCR bA 4013

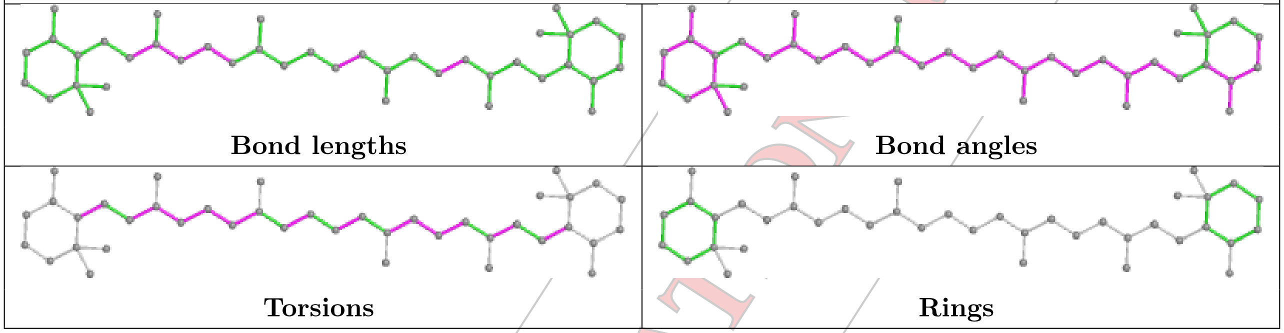

Ligand BCR bA 4014

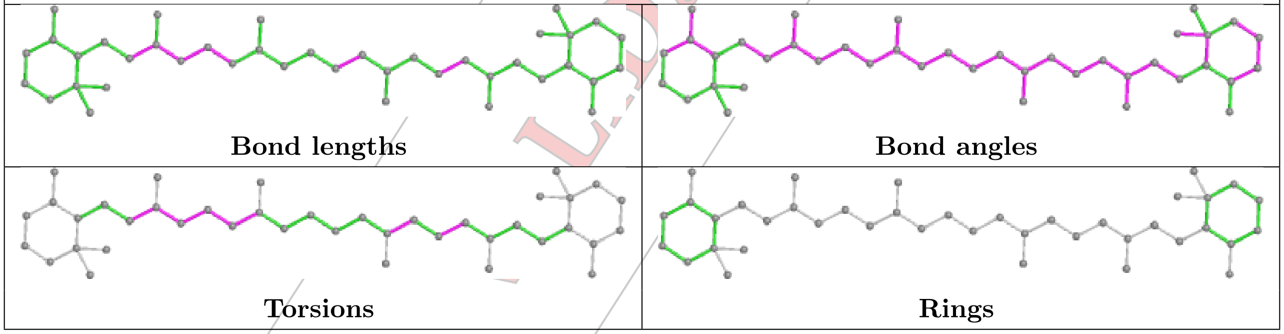

Ligand BCR bA 4016

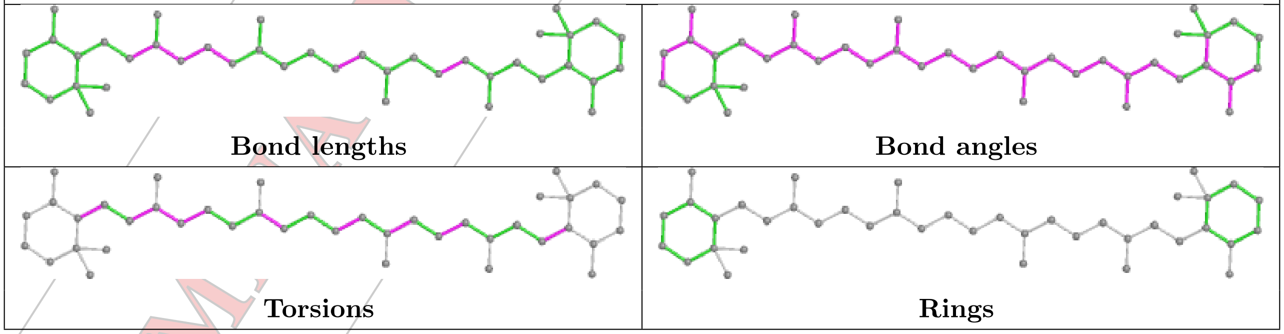

PRELIMINARY REPORT

Ligand BCR bA 4017

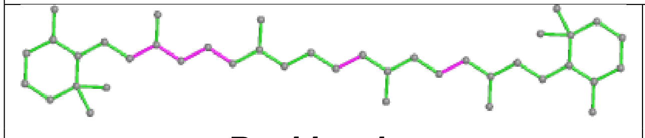
Bond lengths

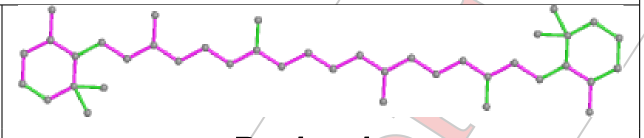
Bond angles

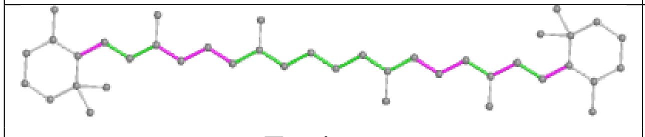
Torsions

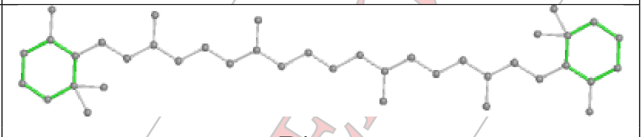
Rings

Ligand LMG bA 5002

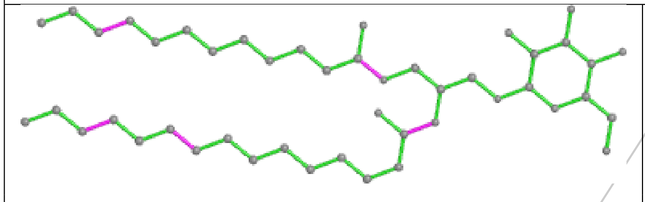
Bond lengths

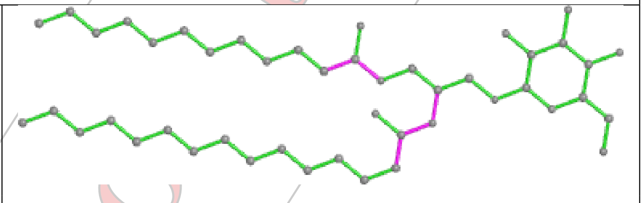
Bond angles

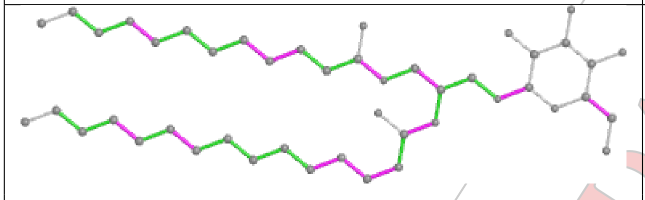
Torsions

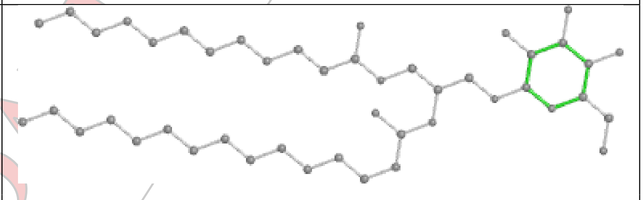
Rings

PRELIMINARY VALIDATION REPORT

Ligand LHG bA 5004

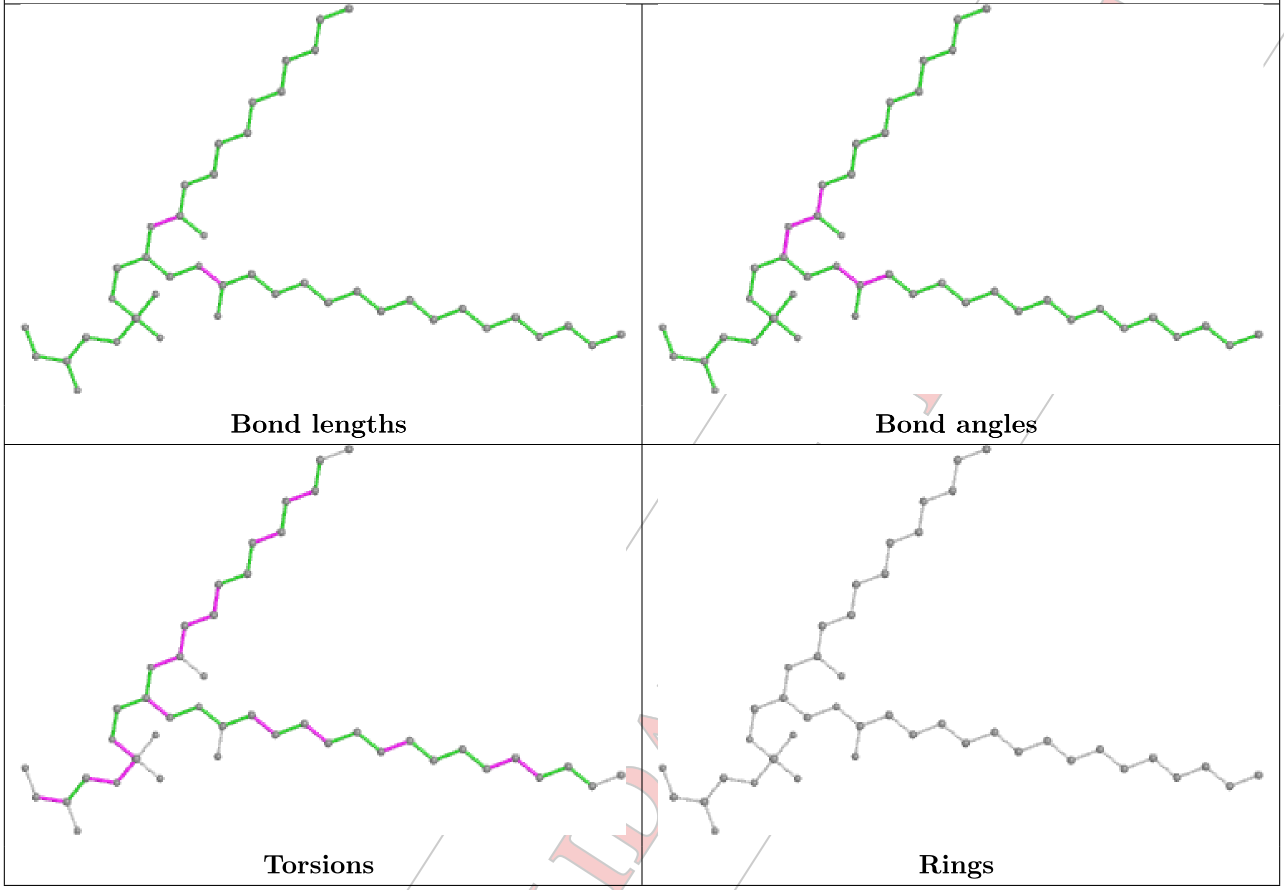

Ligand BCR iA 4018

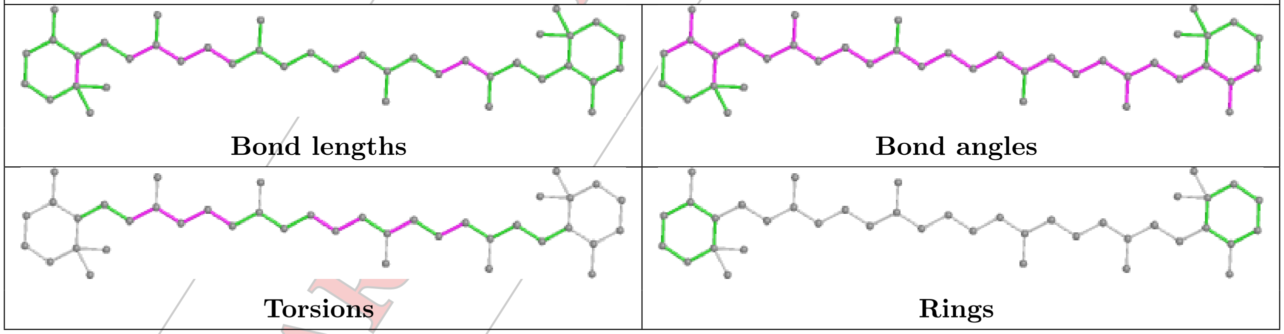

Ligand BCR iA 4020

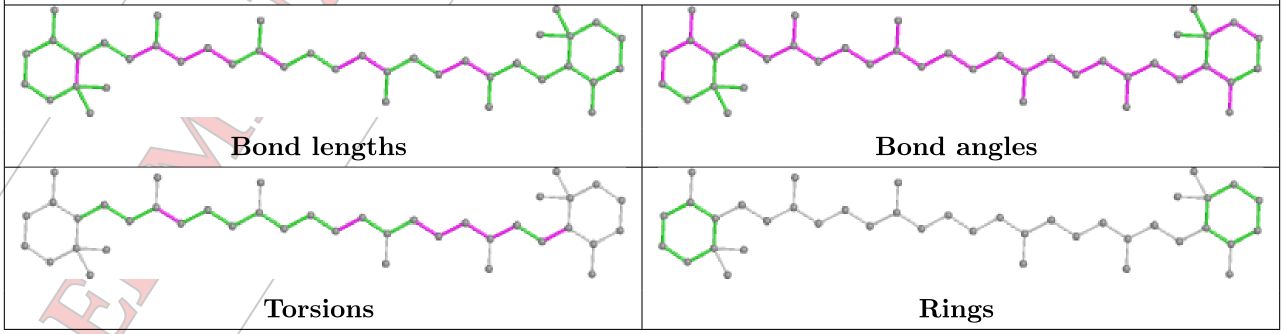

Ligand LMG iA 5006

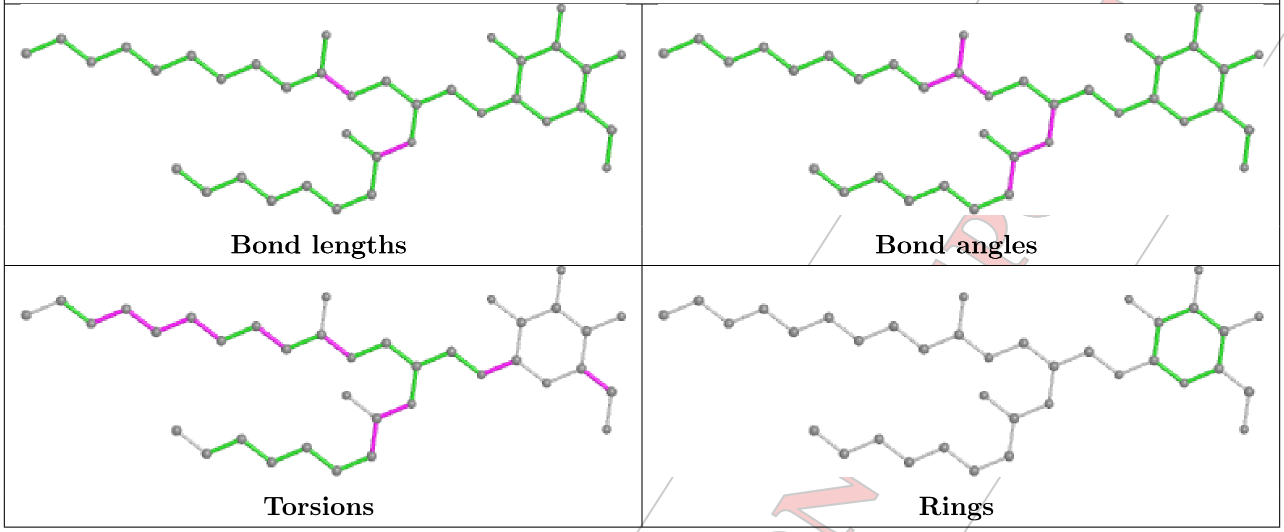

Ligand CLA kA 1401

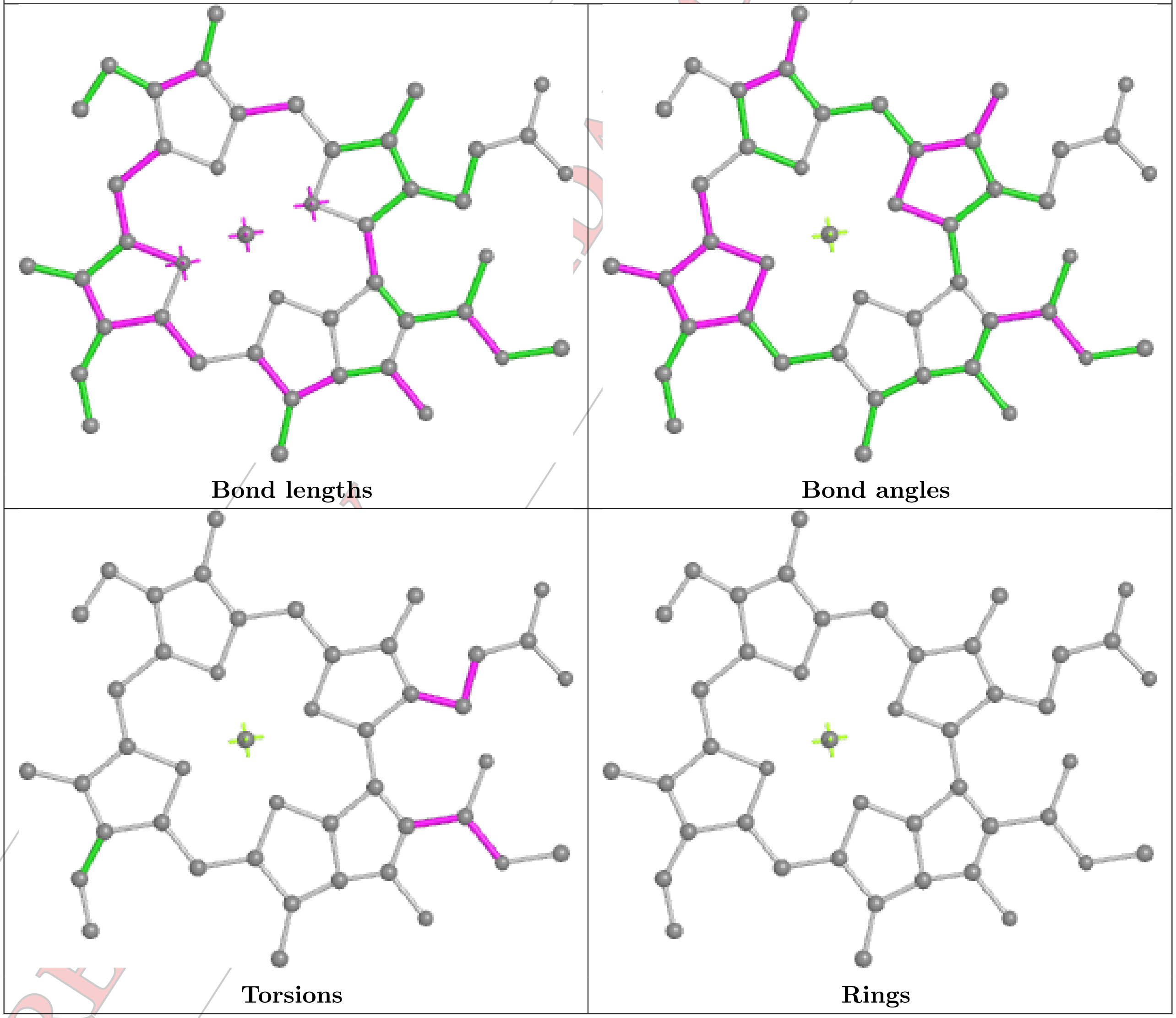

Ligand CLA lA 1501

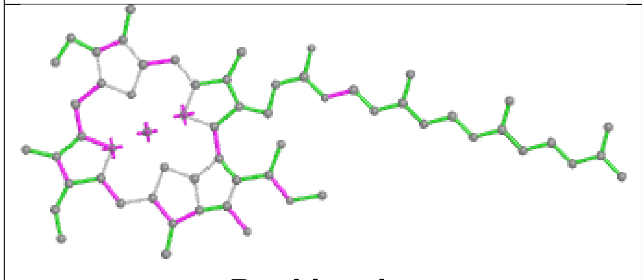
Bond lengths

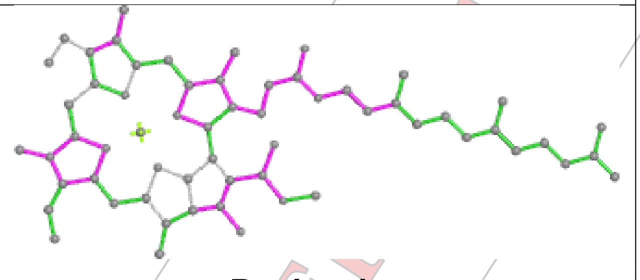
Bond angles

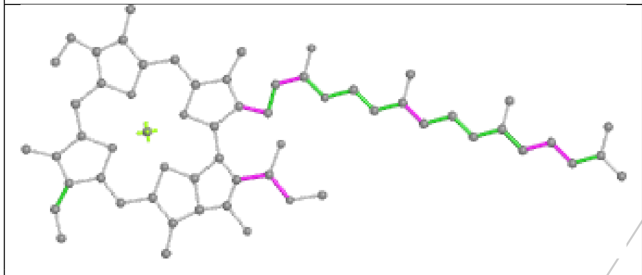
Torsions

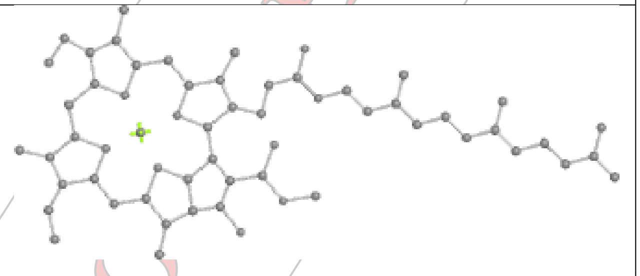
Rings

Ligand CLA lA 1502

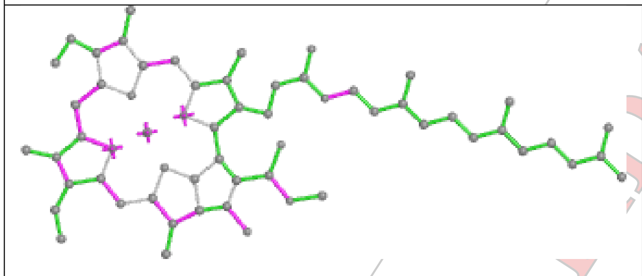
Bond lengths

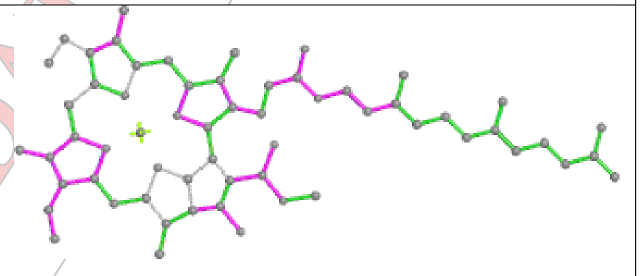
Bond angles

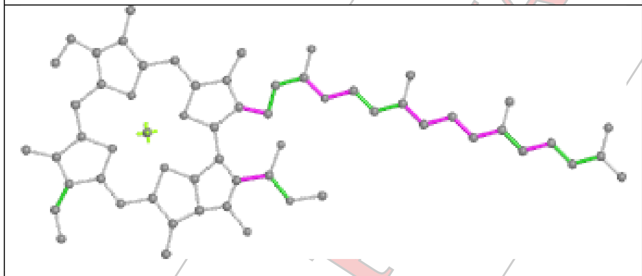
Torsions

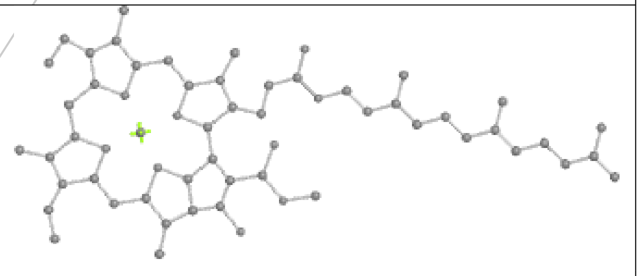
Rings

PRELIMINARY

Ligand CLA lA 1503

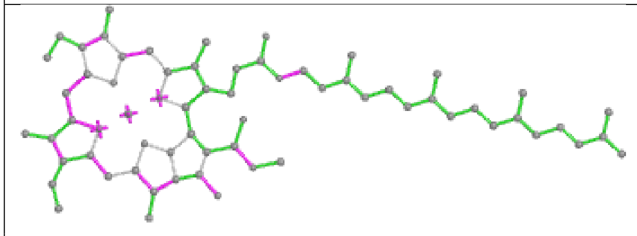
Bond lengths

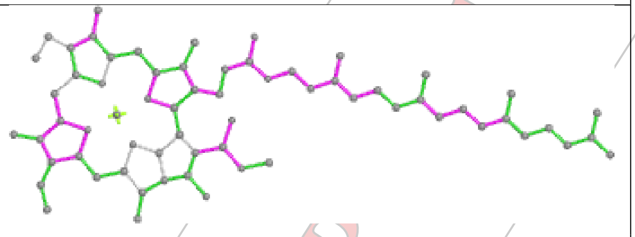
Bond angles

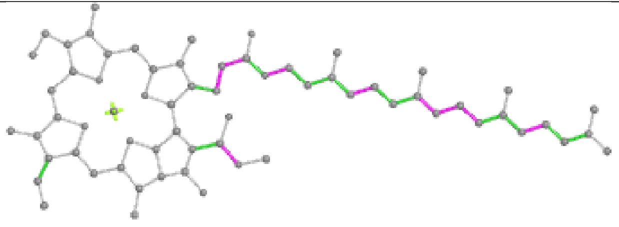
Torsions

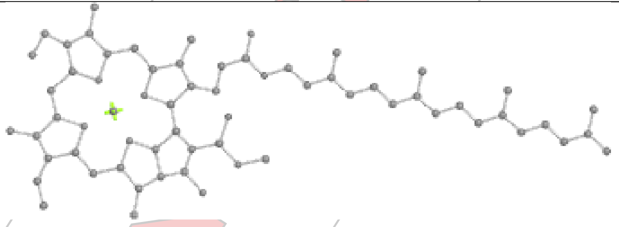
Rings

Ligand BCR lA 4019

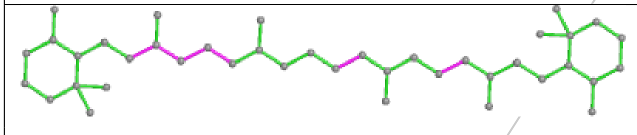
Bond lengths

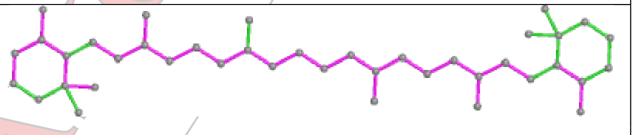
Bond angles

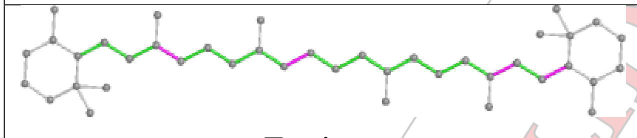
Torsions

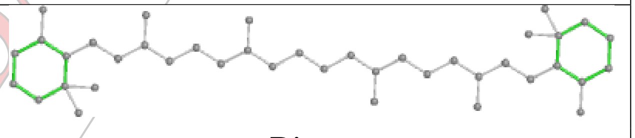
Rings

Ligand BCR lA 4022

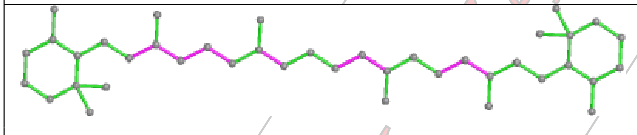
Bond lengths

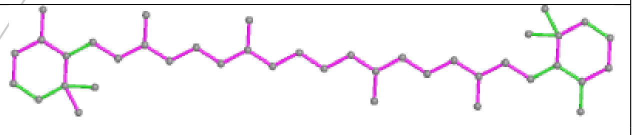
Bond angles

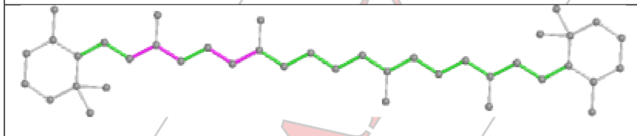
Torsions

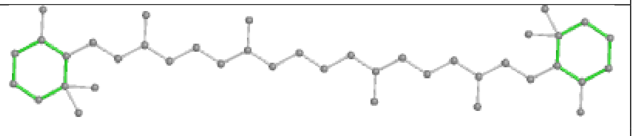
Rings

PRELIMINARY EVALUATION

Ligand LMG lA 5007

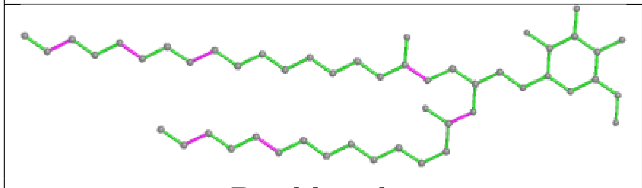
Bond lengths

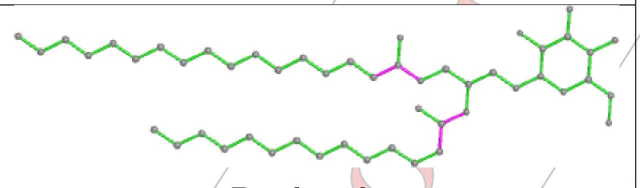
Bond angles

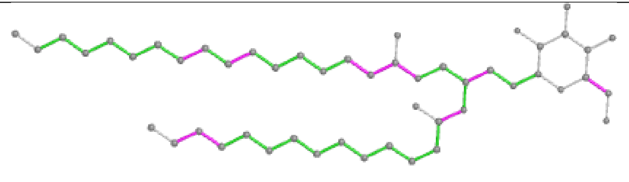
Torsions

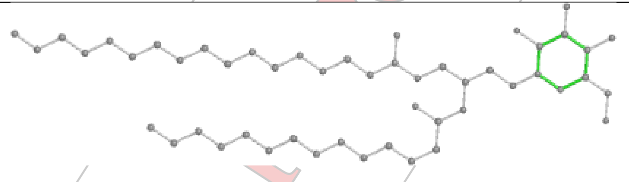
Rings

Ligand BCR mA 4021

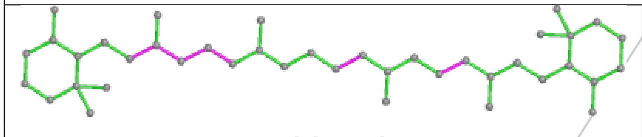
Bond lengths

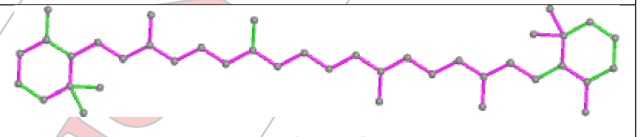
Bond angles

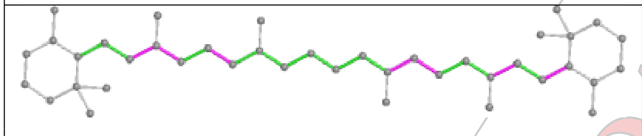
Torsions

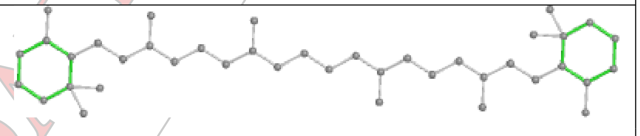
Rings

PRELIMINARY VALIDATION

Ligand CLA xA 1701

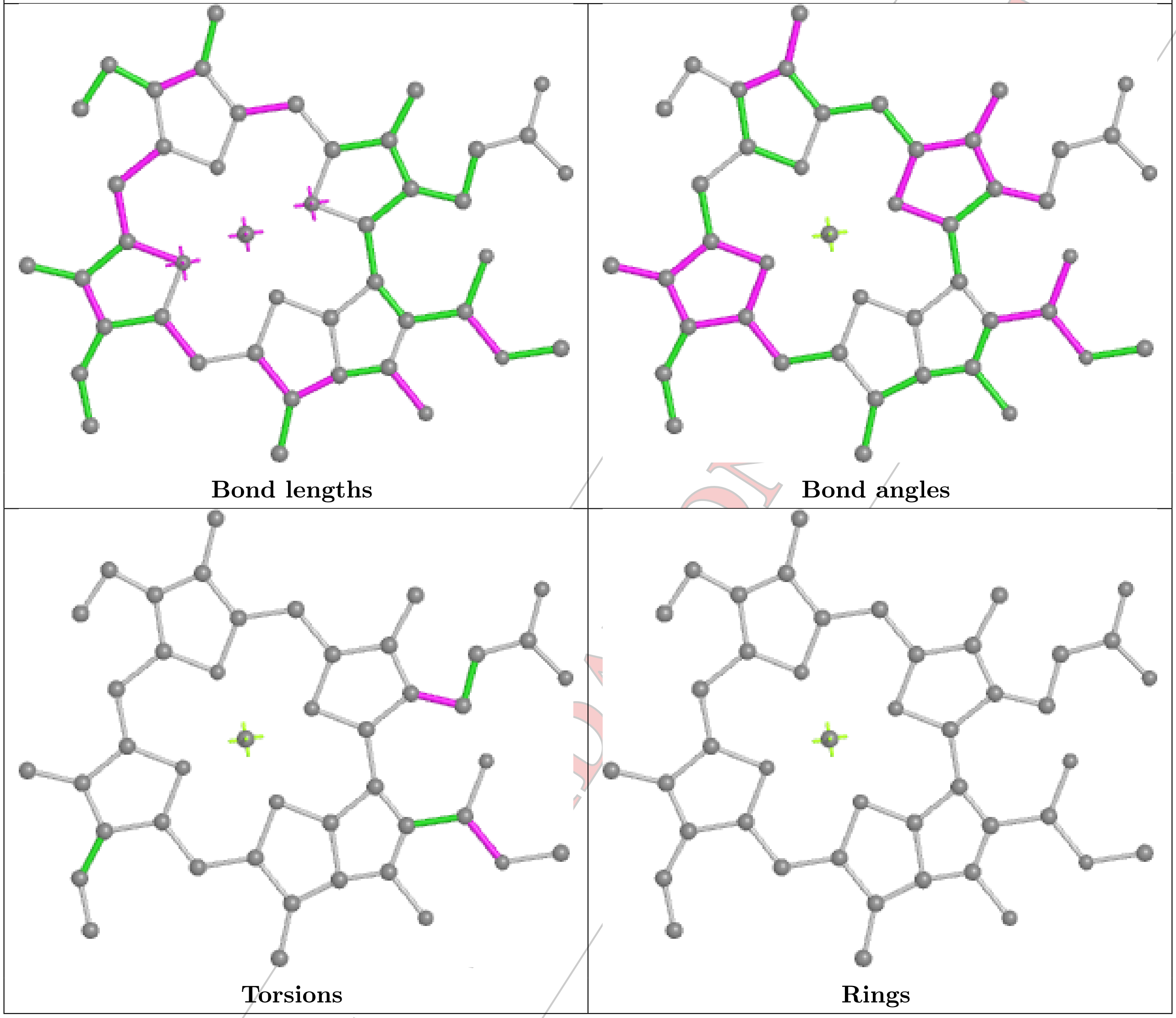

PRELIMINARY

Ligand F6C GA 1120

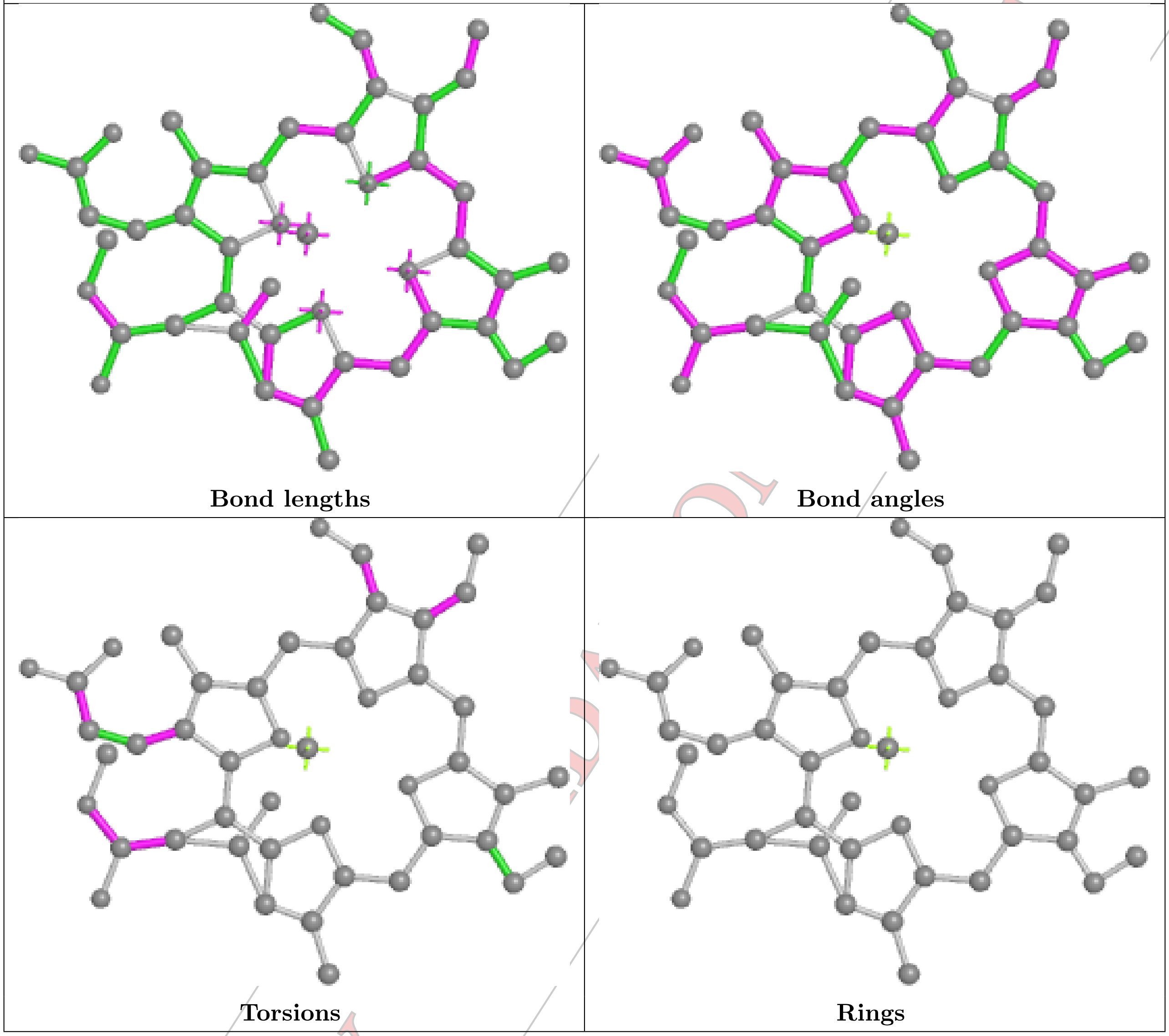

PRELIMINARY

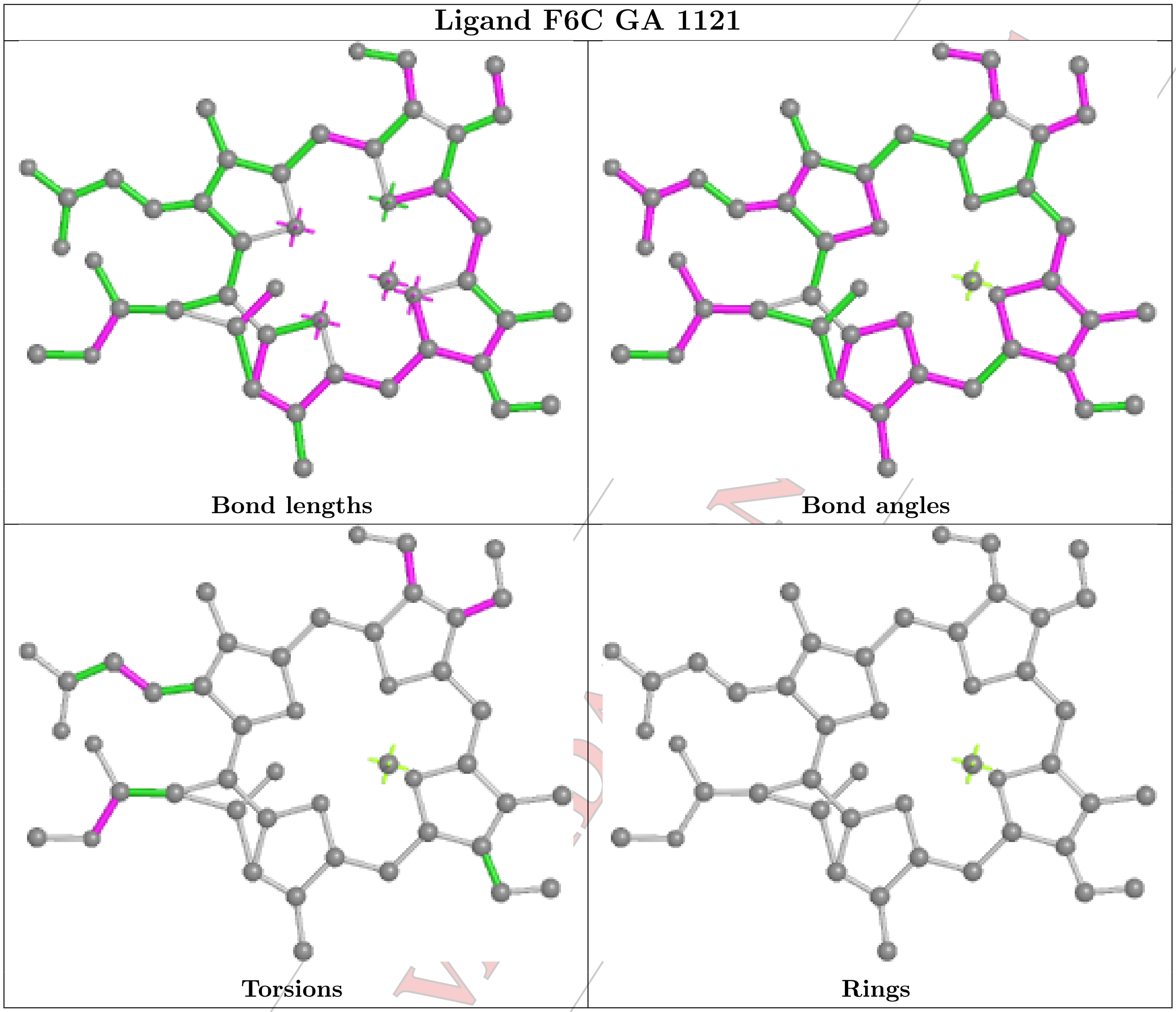

PRELIMINARY

Ligand F6C AA 1120

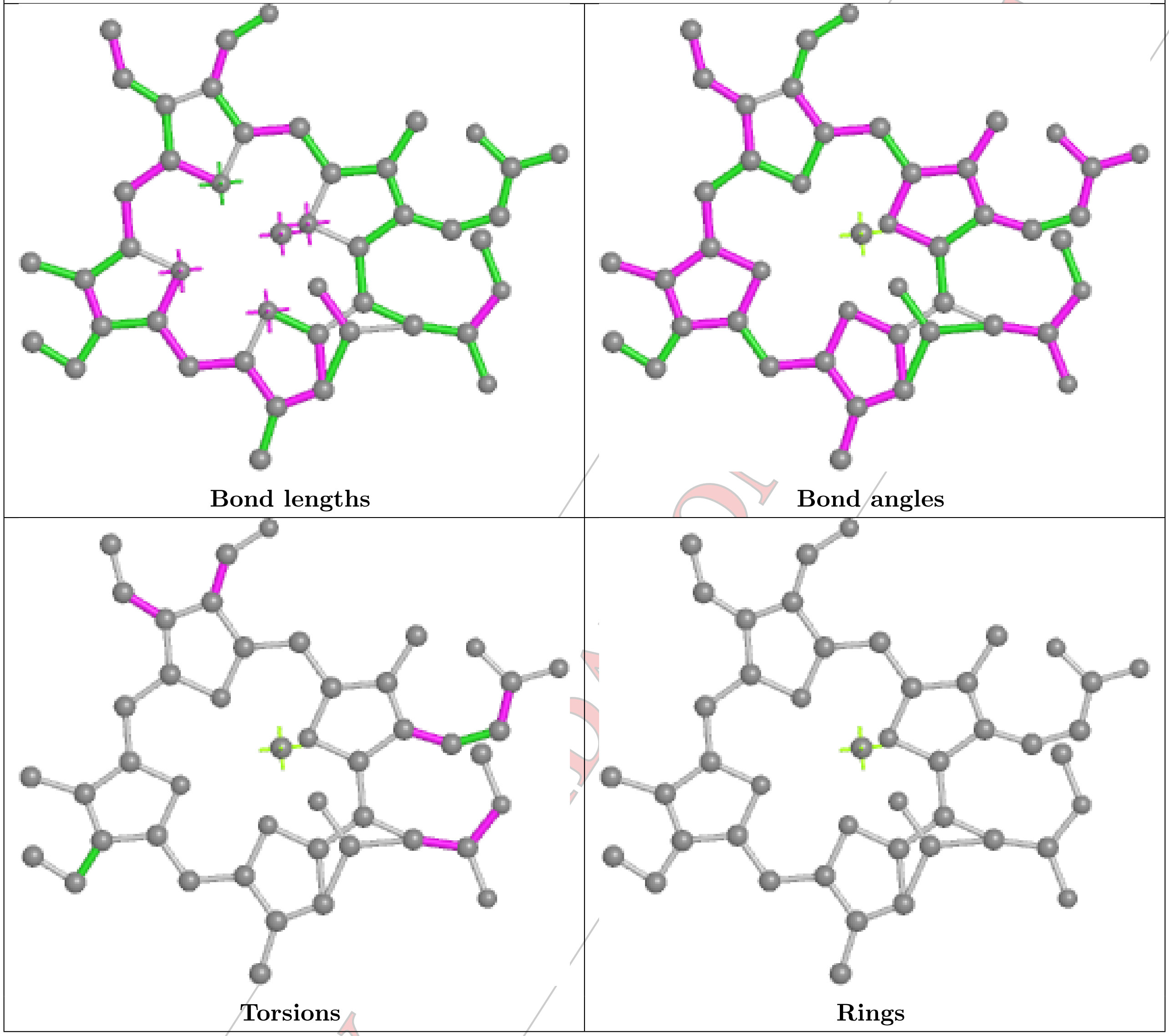

PRELIMINARY

Ligand F6C aA 1121

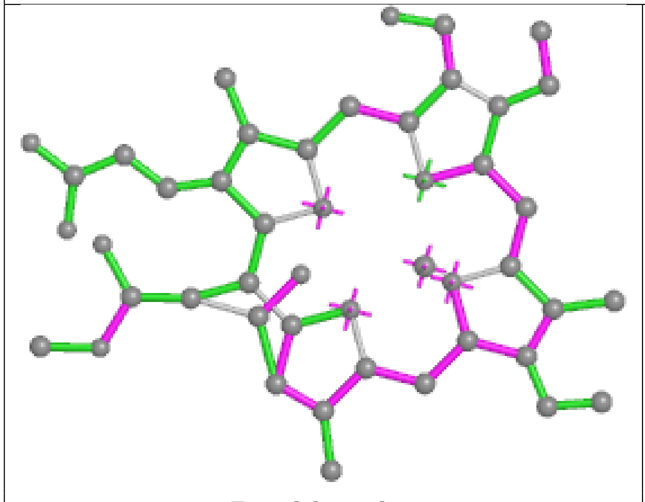

Bond lengths

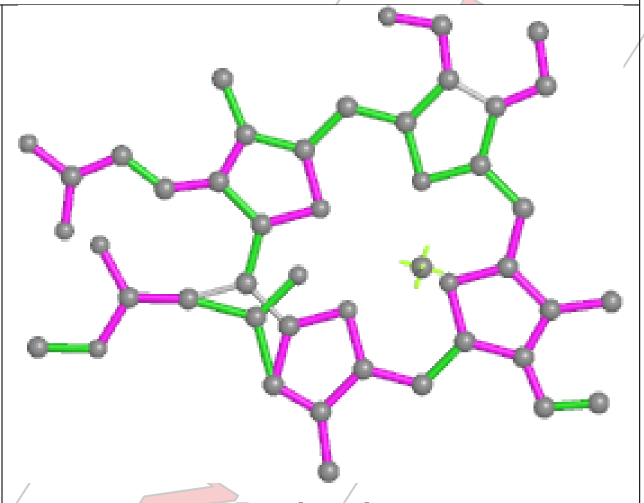

Bond angles

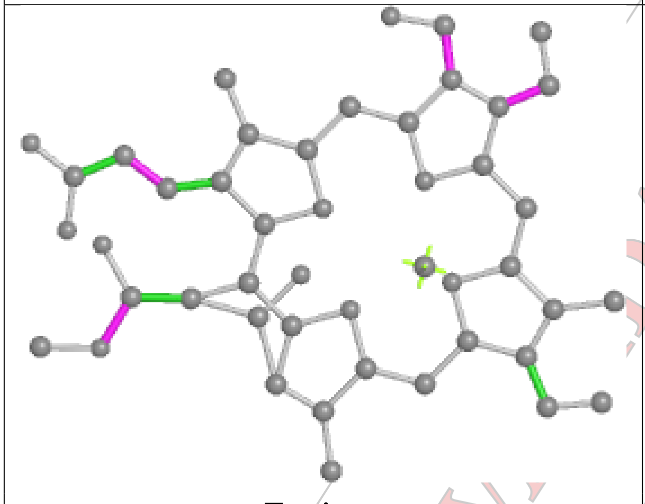

Torsions

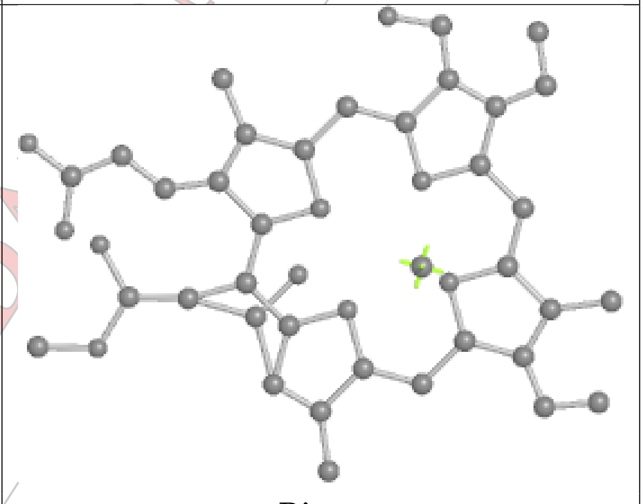

Rings

PRELIMINARY

Ligand F6C AA 1121

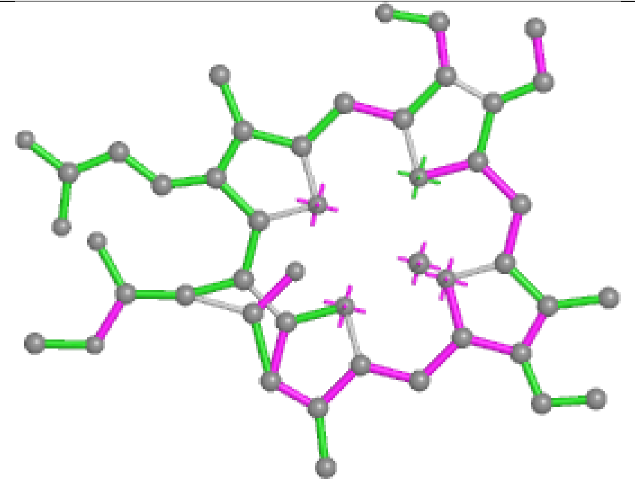
Bond lengths

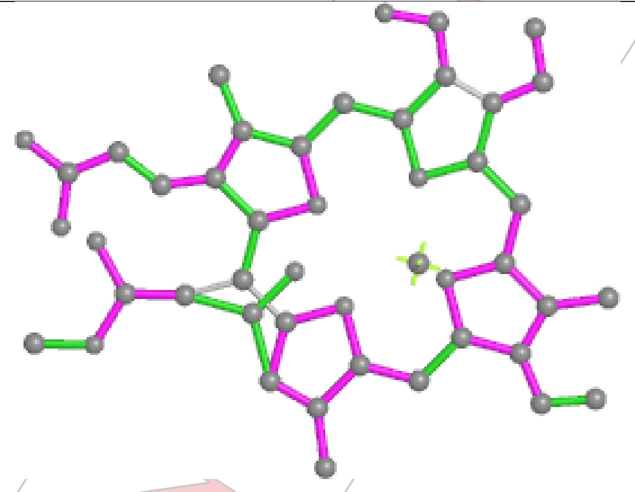
Bond angles

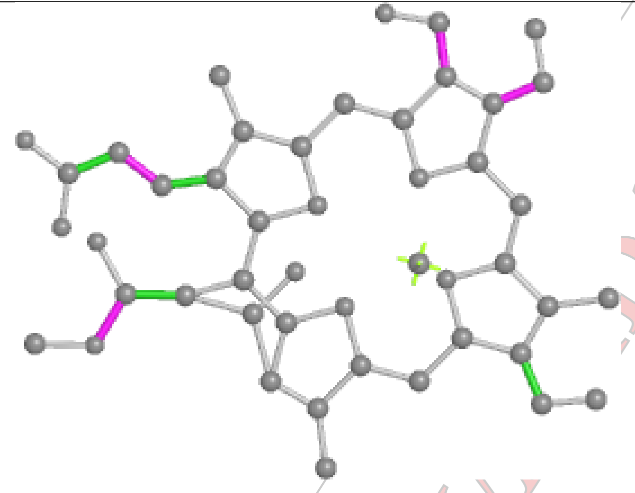
Torsions

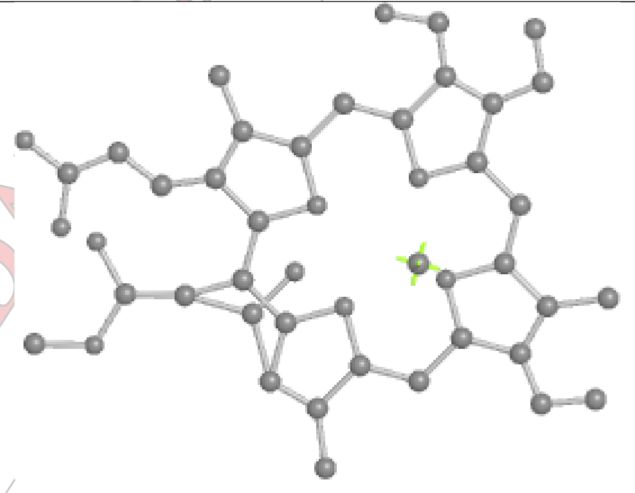
Rings

PRELIMINARY VERSION

Ligand F6C bA 1230

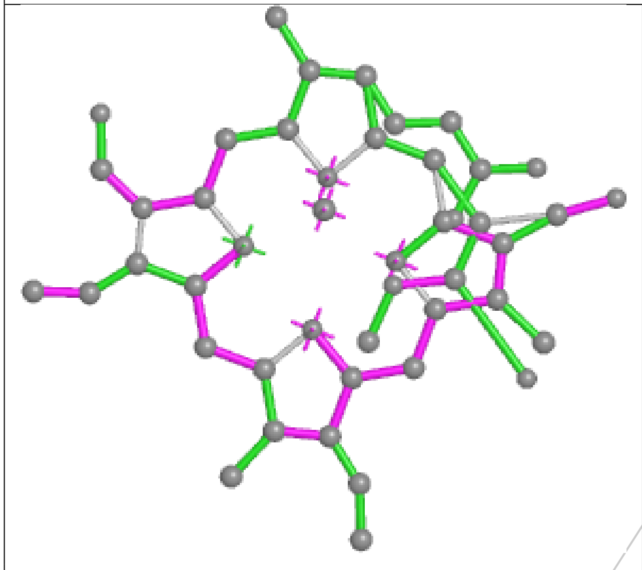

Bond lengths

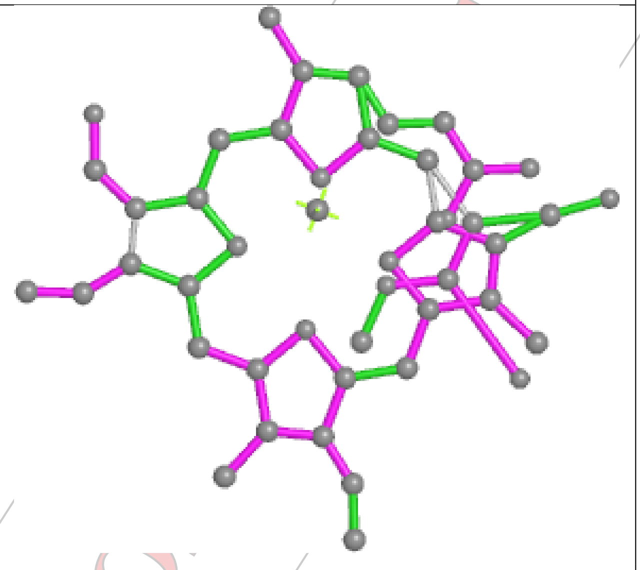

Bond angles

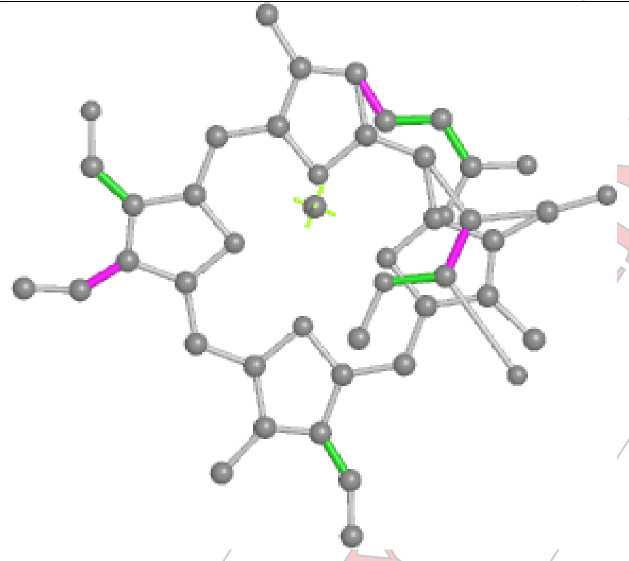

Torsions

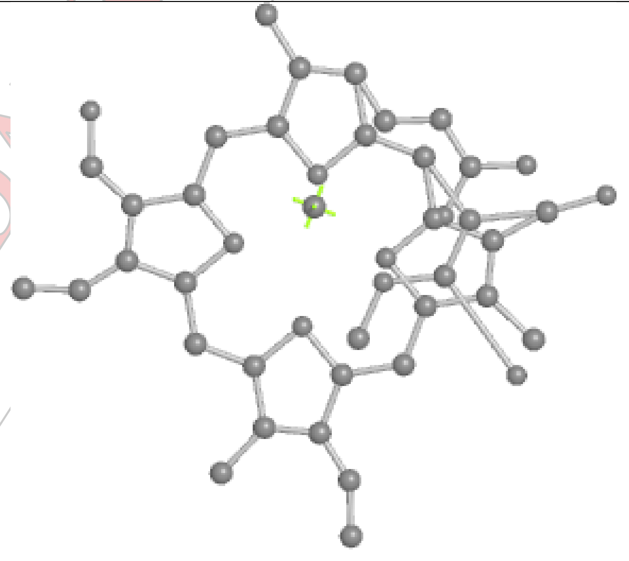

Rings

PRELIMINARY

Ligand F6C bA 1237	
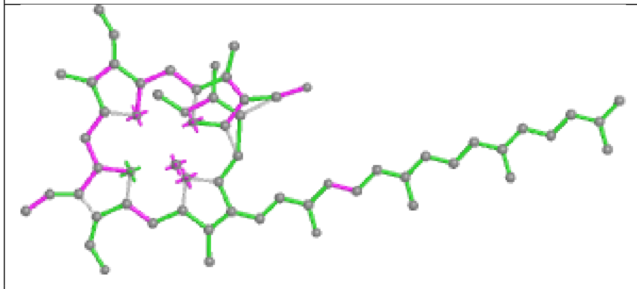 Bond lengths	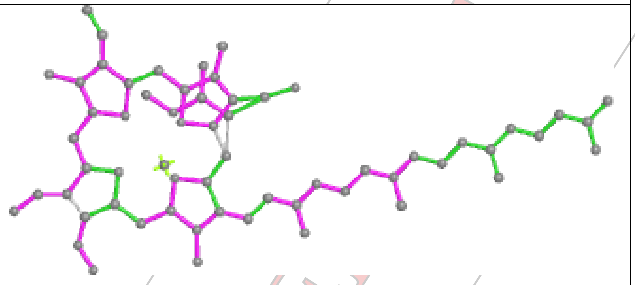 Bond angles
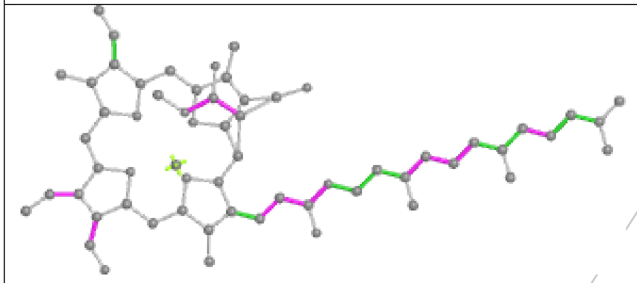 Torsions	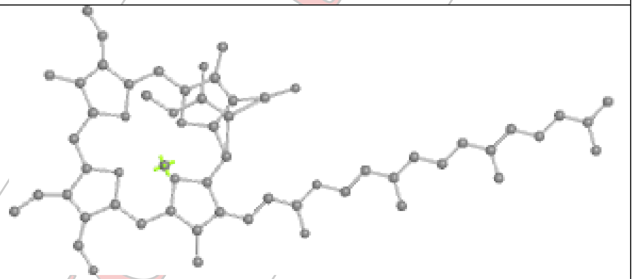 Rings

PRELIMINARY VALIDATION

Ligand F6C HA 1230

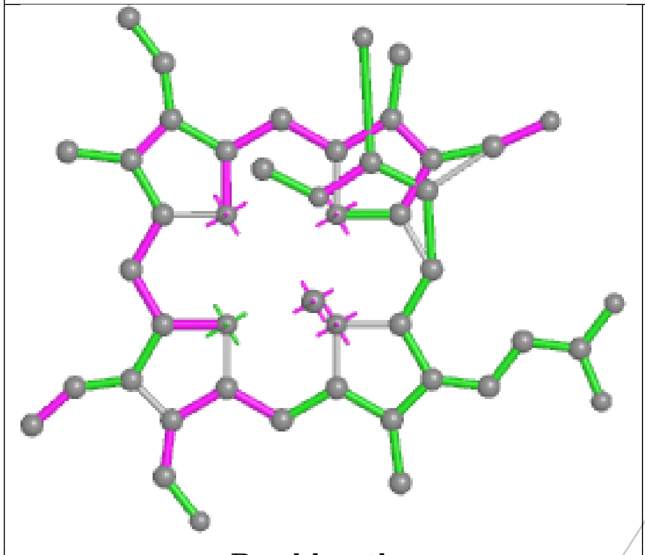
Bond lengths

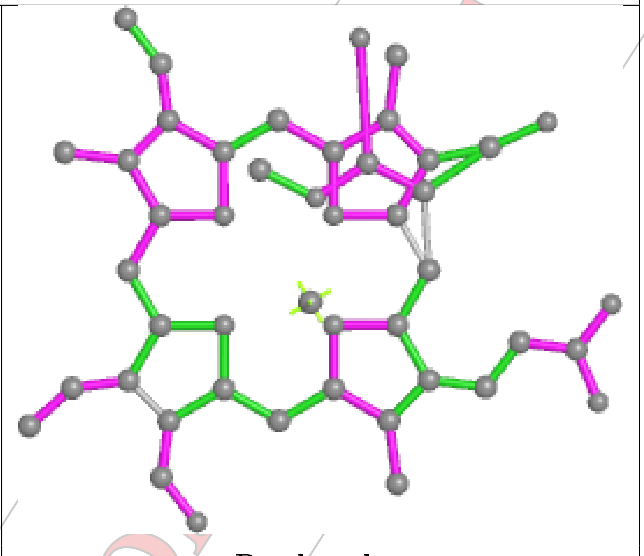
Bond angles

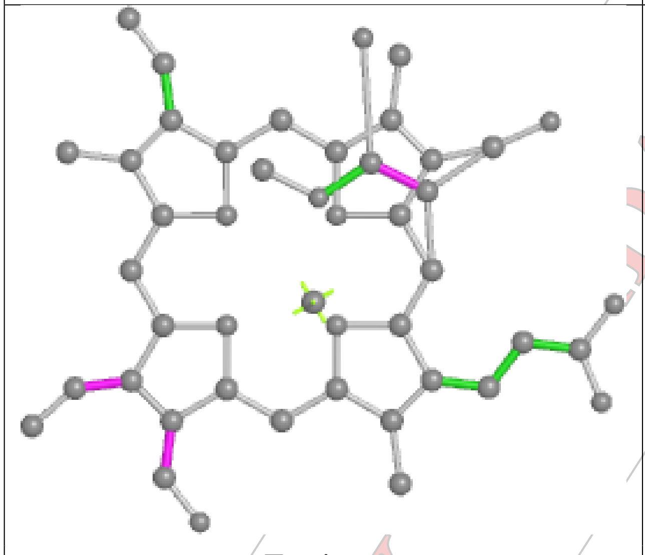
Torsions

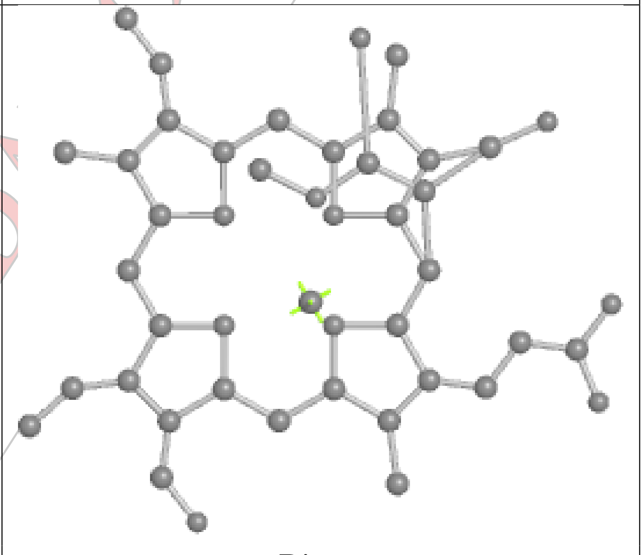
Rings

PRELIMINARY

Ligand F6C BA 1230

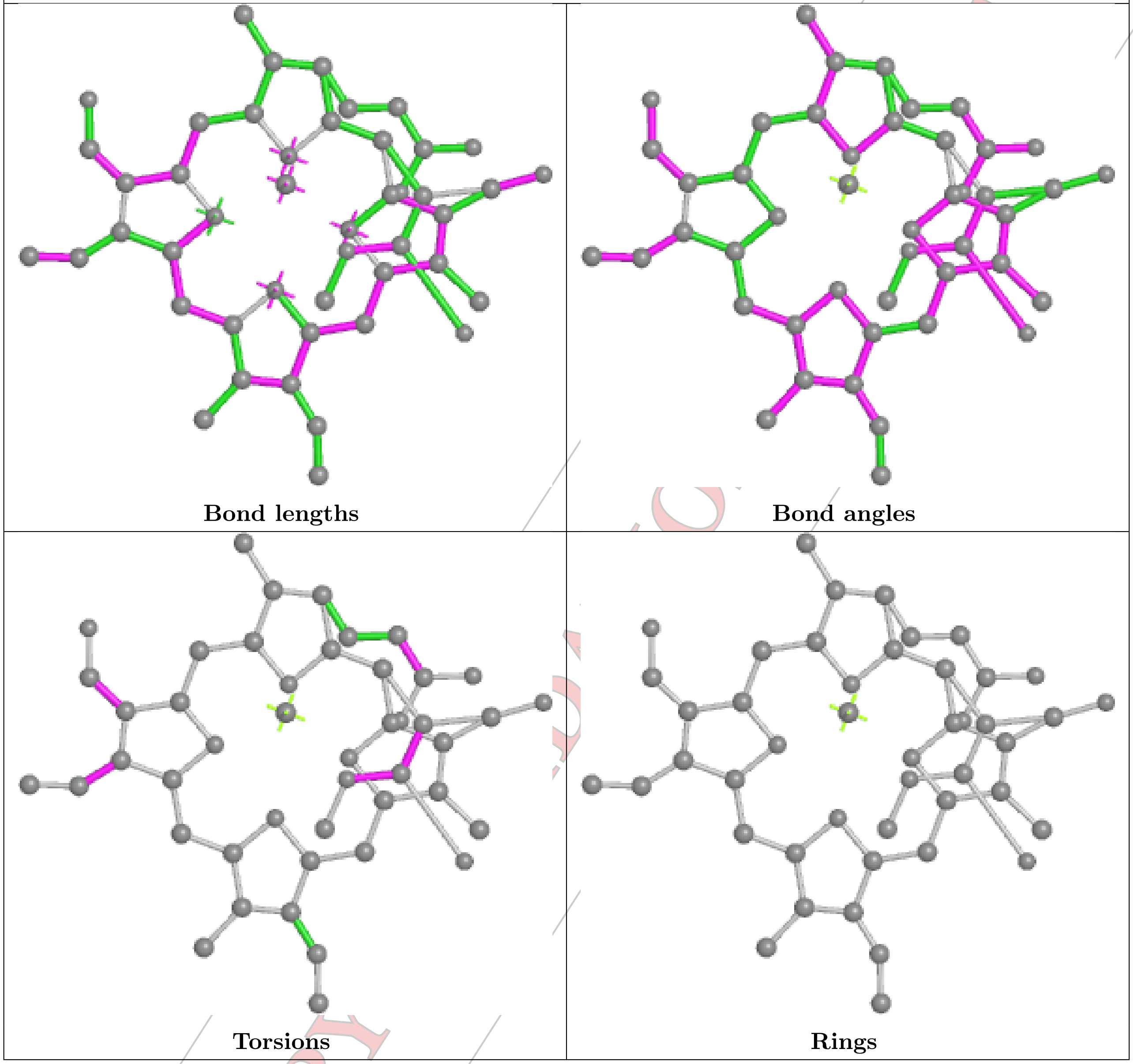

PRELIMINARY

Ligand F6C BA 1237	
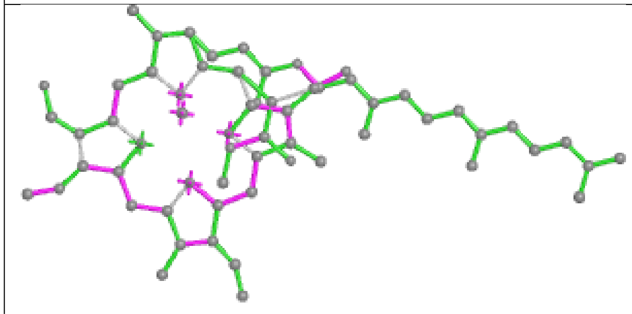 Bond lengths	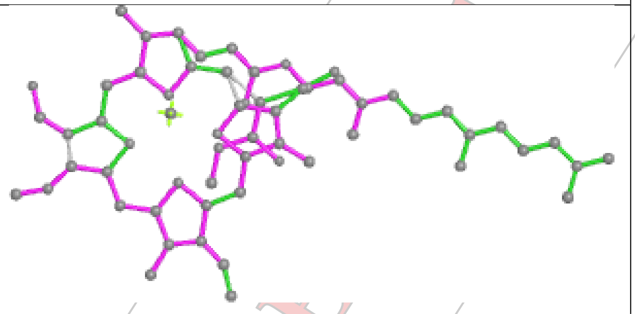 Bond angles
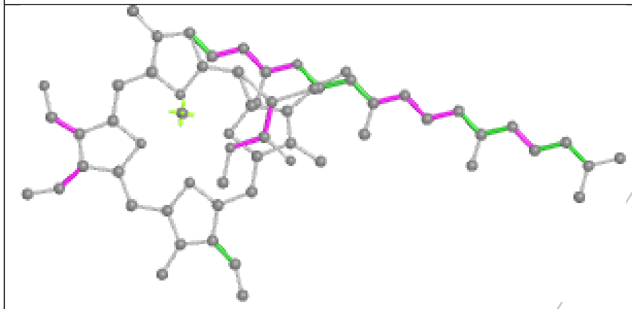 Torsions	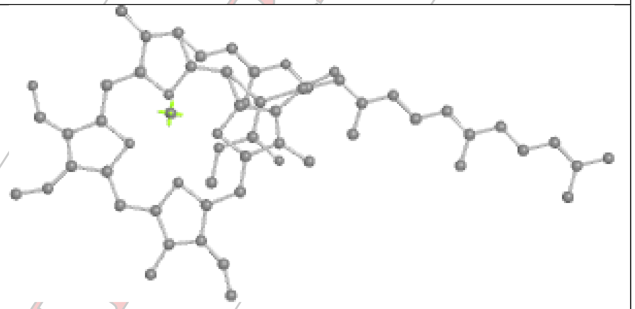 Rings

PRELIMINARY VALIDATION

Ligand F6C aA 1120

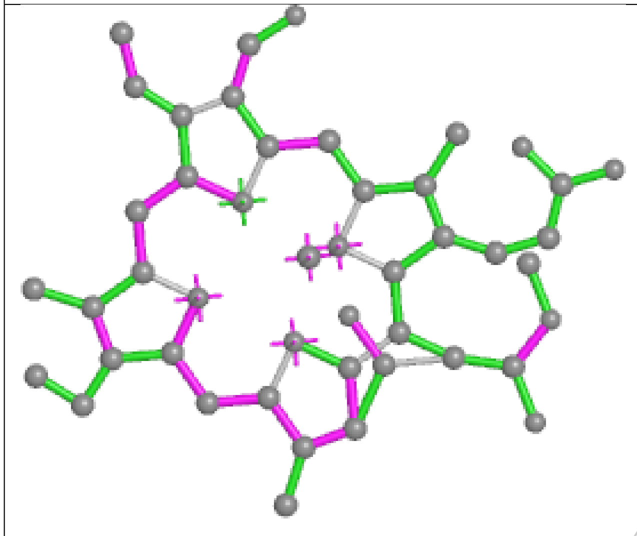

Bond lengths

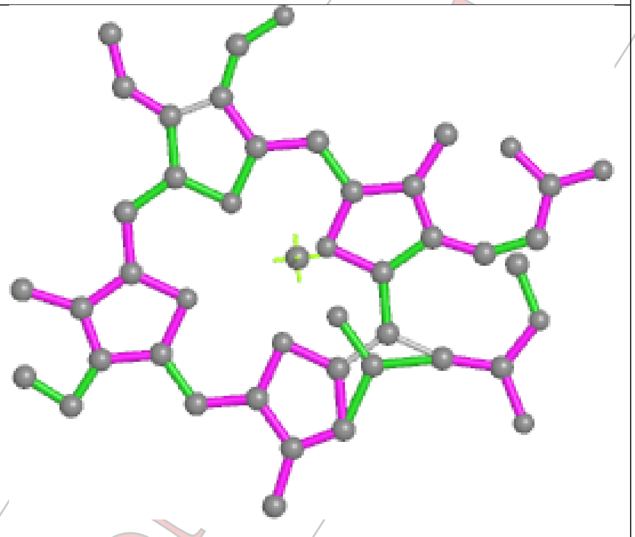

Bond angles

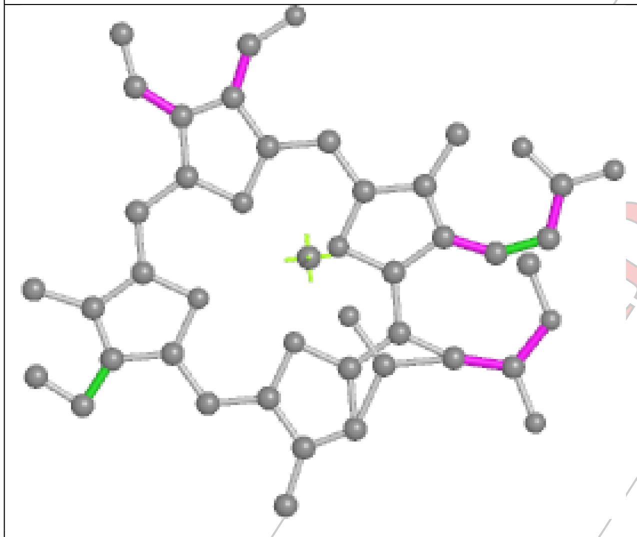

Torsions

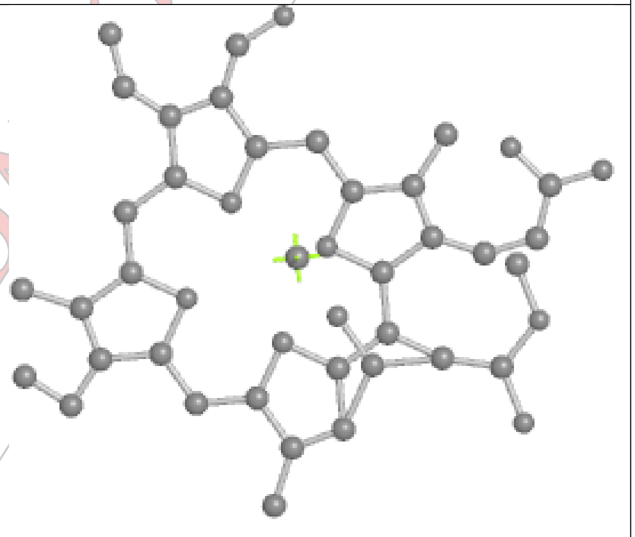

Rings

PRELIMINARY

Ligand F6C HA 1237

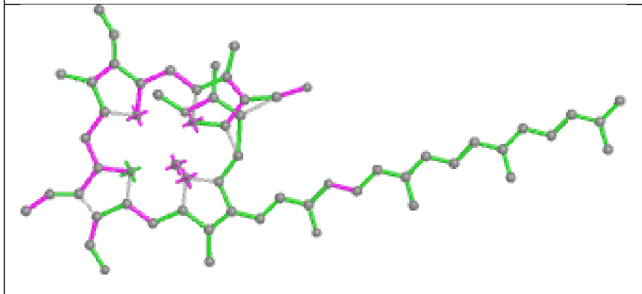

Bond lengths

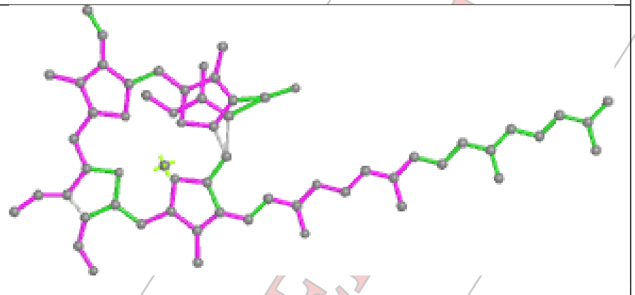

Bond angles

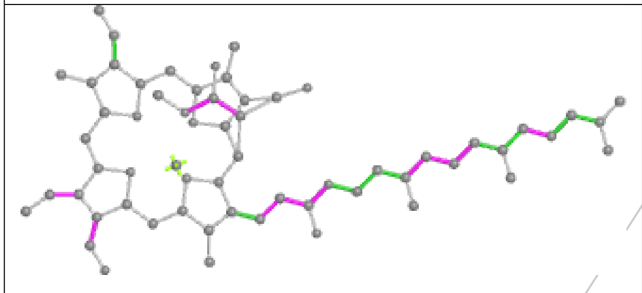

Torsions

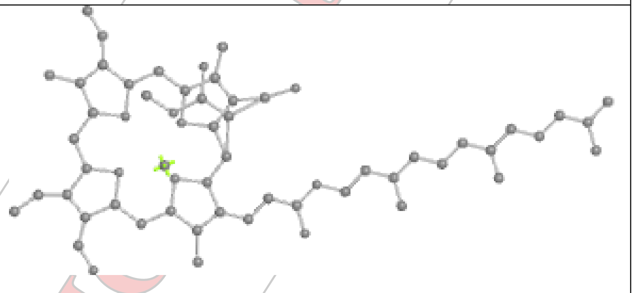

Rings

5.7 Other polymers (i)

There are no such residues in this entry.

5.8 Polymer linkage issues (i)

There are no chain breaks in this entry.

PRELIMINARY VALIDATION
